# Towards the Prediction of Antimicrobial Efficacy for Hydrogen Bonded, Self‐Associating Amphiphiles

**DOI:** 10.1002/cmdc.202000533

**Published:** 2020-10-16

**Authors:** Nyasha Allen, Lisa J. White, Jessica E. Boles, George T. Williams, Dominique F. Chu, Rebecca J. Ellaby, Helena J. Shepherd, Kendrick K. L. Ng, Laura R. Blackholly, Ben Wilson, Daniel P. Mulvihill, Jennifer R. Hiscock

**Affiliations:** ^1^ School of Biosciences University of Kent Canterbury Kent CT2 7NH UK; ^2^ School of Physical Sciences University of Kent Canterbury Kent CT2 7NH UK; ^3^ School of Computing University of Kent Canterbury Kent CT2 7NH UK

**Keywords:** Antimicrobial, Amphiphiles, Supramolecular Chemistry, Hydrogen bonds, Structure-activity relationships

## Abstract

Herein we report 50 structurally related supramolecular self‐associating amphiphilic (SSA) salts and related compounds. These SSAs are shown to act as antimicrobial agents, active against model Gram‐positive (methicillin‐resistant *Staphylococcus aureus*) and/or Gram‐negative (*Escherichia coli*) bacteria of clinical interest. Through a combination of solution‐state, gas‐phase, solid‐state and in silico measurements, we determine 14 different physicochemical parameters for each of these 50 structurally related compounds. These parameter sets are then used to identify molecular structure‐physicochemical property‐antimicrobial activity relationships for our model Gram‐negative and Gram‐positive bacteria, while simultaneously providing insight towards the elucidation of SSA mode of antimicrobial action.

## Graphical abstract

**The antimicrobial efficacy** of 50 supramolecular self‐associating salts (SSAs) and related compounds was established against MRSA and *E. coli*. In addition, 14 different physicochemical properties were determined experimentally for each compound within this library. From these data we identified relationships between compound structure, physiochemical property, and antimicrobial activity.

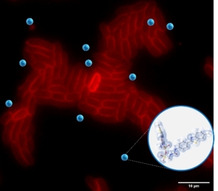

## Introduction

Since the discovery of the first antibiotic, humanity has relied heavily upon antimicrobial agents to combat and/or prevent communicable bacterial diseases or infections. Unfortunately, bacterial strains have now been identified which are resistant to all antimicrobial agents currently marketed.^[1]^ This includes the antibiotic of last resort – colistin^[2]^ – and commonly used antiseptics such as octenidine.^[3]^

A recent report commissioned by the UK government has predicted that the number of global annual deaths directly attributed to antimicrobial resistance (AMR) is set to rise from 0.7 million (2014) to 10 million (2050), overtaking those directly attributed to cancer. Although AMR is a global issue of high importance, the effects will be most felt in developing countries located in Africa and Asia.^[4]^ To curb the rise of AMR, great efforts have been made to improve antimicrobial/antibiotic stewardship.^[5]^ This includes the removal of antibiotics from animal feedstuffs^[6]^ and the intelligent prescription of antibiotics by both clinicians^[7]^ and veterinarians,^[8]^ combined with antibiotic susceptibility testing pre‐treatment.^[9]^ However, the discovery of novel antimicrobial agents with potential for development as antibiotics remains unmet, mainly because of the cost involved in drug discovery in combination with poor market returns.[^1^, ^10^]

In response to this need new strategies towards antimicrobial agents have been developed, including those inspired by supramolecular chemistry. Discoveries in this area include an example from M. Zhang et al., who have utilised the pillar[5]arene scaffold to produce synthetic channels capable of selectively inserting into bacterial membranes and demonstrating efficacy against Gram‐positive *Staphylococcus epidermidis*.^[11]^ Furthermore, El‐Sheshtawy et al., have demonstrated the ability of cucurbit[7]uril to extend both the shelf‐life and antimicrobial activity of fluoroquinolone antibiotics, through the formation of inclusion complexes.^[12]^ In addition, a neutral pyridyl urea scaffold developed by Gunnlaugsson et al., has been shown to self‐associate and form supramolecular gels, which demonstrate antimicrobial activity against both Gram‐positive Methicillin‐Resistant *Staphylococcus aureus* (MRSA) and Gram‐negative *Escherichia coli* (*E. coli*).^[13]^

**Figure 1:**
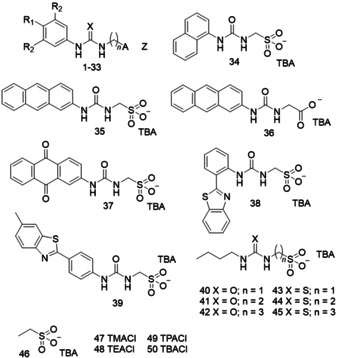
Chemical structures of 1–50. For the substituents of individual compounds 1–33 please refer to Table 1. TMA=tetramethylammonium, TEA=tetraethylammonium, TPA=tetrapropylammonium, TBA=tetrabutylammonium.

To date our work on this topic has focused on the development of a novel class of Supramolecular Self‐associating Amphiphile (SSA) such as those illustrated in Figure 1 and Table 1.[^14^, ^15^, ^16^, ^17^, ^18^] Members from this class of compound have also been identified as antimicrobial agents against both Gram‐positive and Gram‐negative bacteria.[^19^, ^20^, ^21^] In this work, we now expand our initial SSA library and combine low‐level computational modelling and physicochemical self‐associative property determination towards the elucidation of SSA structure‐physicochemical property‐antimicrobial activity relationships against Gram‐positive (MRSA) and/or Gram‐negative (*E. coli*) bacteria of clinical interest.^[22]^ This has resulted in the production of predictive models to drive the intelligent design of ever more effective, next generation SSA technologies.

**Table 1:** Substituents for the general chemical structure shown in Figure 1, giving rise to compounds **1**–**33**.

SSA	R_1_	R_2_	X	*n*	A	Z
**1**	CF_3_	H	O	1	SO_3_^−^	TBA
**2**	CF_3_	H	O	2	SO_3_^−^	TBA
**3**	CF_3_	H	O	3	SO_3_^−^	TBA
**4**	CF_3_	H	S	1	SO_3_^−^	TBA
**5**	CF_3_	H	S	2	SO_3_^−^	TBA
**6**	CF_3_	H	S	3	SO_3_^−^	TBA
**7**	CF_3_	H	O	1	SO_3_^−^	Na^+^
**8**	CF_3_	H	O	1	SO_3_^−^	PyrH^+^
**9**	CF_3_	H	O	1	SO_3_^−^	TMA
**10**	CF_3_	H	O	1	SO_3_^−^	TEA
**11**	CF_3_	H	O	1	SO_3_^−^	TPA
**12**	CF_3_	H	O	1	SO_3_^−^	TPeA
**13**	CF_3_	H	O	1	SO_3_^−^	THA
**14**	CF_3_	H	S	1	SO_3_^−^	Na^+^
**15**	CF_3_	H	S	1	SO_3_^−^	PyrH^+^
**16**	CF_3_	H	S	1	SO_3_^−^	TMA
**17**	CF_3_	H	S	1	SO_3_^−^	TEA
**18**	CF_3_	H	S	1	SO_3_^−^	TPA
**19**	NH_2_	H	O	1	SO_3_^−^	TBA
**20**	OMe	H	O	1	SO_3_^−^	TBA
**21**	H	H	O	1	SO_3_^−^	TBA
**22**	NO_2_	H	O	1	SO_3_^−^	TBA
**23**	H	CF_3_	O	1	SO_3_^−^	TBA
**24**	OMe	H	S	1	SO_3_^−^	TBA
**25**	H	H	S	1	SO_3_^−^	TBA
**26**	NO_2_	H	S	1	SO_3_^−^	TBA
**27**	H	CF_3_	S	1	SO_3_^−^	TBA
**28**	CF_3_	H	O	1	CO_2_^t^Bu	N/A
**29**	CF_3_	H	O	1	COOH	N/A
**30**	CF_3_	H	O	1	COO^−^	TBA
**31**	CF_3_	H	S	1	CO_2_^t^Bu	N/A
**32**	CF_3_	H	S	1	COOH	N/A
**33**	CF_3_	H	S	1	COO^−^	TBA

PyrH+=pyridinium, TPeA=tetrapentylammonium, THA=tetrahexylammonium.

## Results and Discussion

### Synthesis

The library of SSAs and related compounds **1**–**50** were designed through stepwise alteration of **1**, to enable the elucidation of SSA structure‐physicochemical property‐antimicrobial activity relationships. The syntheses of **1**–**13**, **19**–**39** and **46** have previously been reported,[^14^, ^16^, ^17^, ^18^, ^19^] while **47**–**50** were obtained from commercial sources. Compound **14** was obtained via cation exchange from **15**; however, it was found to be unstable and so was not used in any further studies. Compound **15** was obtained as a white solid in a 65 % yield through the reaction of 4‐(trifluoromethyl) phenyl isothiocyanate with aminomethanesulfonic acid in pyridine. Compounds **16**–**18** were obtained through the addition of one equivalent of the appropriate quaternary ammonium hydroxide to **15** in H_2_O and isolated as white solids in yields of 98 %, 99 %, and 88 % respectively. Compounds **40**–**45** were synthesised through the reaction of the appropriate tetrabutylammonium (TBA) amino sulfonate salt with the appropriate isocyanate or isothiocyanate in pyridine or chloroform and obtained in yields of 63 %, 73 %, 56 %, 32 %, 64 %, and 58 % respectively, as either viscous oils (**40**–**44**) or a cream solid (**45**).

### Antimicrobial activity

Most amphiphilic antimicrobials in use are cationic in nature and include a wide variety of natural and synthetic scaffolds such as peptides (polymyxin)^[23]^ and antiseptics (octenidine and chlorohexidine).^[24]^ Here the targeting of these agents towards bacteria over mammalian cells is largely driven by electrostatic interactions between the antimicrobial agent and the negatively charged bacterial surface.^[25]^ Conversely, the more active portion of the SSA construct is often the anionic component. The selectivity of the SSA anion towards bacterial membranes is thought to be due to preferential host:guest complexation events between the SSA and those phospholipid head groups present in higher proportions at the surface of bacteria, compared to eukaryotic cells. Evidence supporting this hypothesis was provided through the completion of an NMR nanodisc study, in which selective adhesion of **39** to model bacterial over eukaryotic cell membranes was observed.^[26]^

Expanding our proof‐of‐principle antimicrobial efficacy studies, MIC_50_ (Minimum Inhibitory Concentration required to decrease microbial growth by 50 %) values were determined for compounds **1**–**50** which passed initial antimicrobial screening, were an agent was found to decrease microbial growth by>10 % after 15 hours when supplied to the microbial culture at 3.3 mM.^[27]^ The results of these microbial studies are summarised in Table 2.

**Table 2:** Overview of MIC_50_ values (mM) determined for **1**–**50** against clinically relevant Gram‐positive MRSA USA300 and Gram‐negative *E. coli* DH10B at an initial calibrated cell concentration equal to the 0.5 McFarland standard, after 900 mins. Fail=compound failed initial antimicrobial screening. Ratio=MIC_50_ MRSA: MIC_50_ *E. coli*. Ampicillin was used as a control to test the validity of the antimicrobial MIC_50_ determination methods.

SSA	MRSA	*E. coli*	ratio	SSA	MRSA	*E. coli*	ratio
**1** ^[21]^	0.46	3.85^[a]^	1 : 8.4	**26**	[b]	[b]	N/A
**2**	0.98	3.93^[a]^	1 : 4.0	**27**	1.10^[d]^	Fail	N/A
**3**	[b]	1.48^[a]^	N/A	**28**	[b]	[b]	N/A
**4**	3.03	[b]	N/A	**29**	Fail	Fail	N/A
**5**	0.25	[b]	N/A	**30**	1.14	1.25	1 : 1.1
**6**	1.08	1.85^[a]^	1 : 1.7	**31**	[b]	[b]	N/A
**7**	Fail	Fail	N/A	**32**	0.83	Fail	N/A
**8**	0.35	Fail	N/A	**33**	0.77	Fail	N/A
**9**	2.17	10.8^[a]^	1 : 5.0	**34**	2.45	4.30^[a]^	1 : 1.8
**10**	2.85	Fail	N/A	**35** ^[19]^	0.46	[b]	N/A
**11**	0.42	5.96^[a]^	1 : 14	**36** ^[19]^	0.61	Fail	N/A
**12**	[b]	[b]	N/A	**37** ^[19]^	0.71	Fail	N/A
**13**	[b]	[b]	N/A	**38** ^[d][21]^	0.99	3.57^[a]^	1 : 3.6
**14**	[c]	[c]	N/A	**39** ^[d][21]^	0.93	5.02^[a]^	1 : 5.4
**15**	Fail	Fail	N/A	**40**	4.41	Fail	N/A
**16**	0.27	Fail	N/A	**41**	2.85	5.67^[a]^	1 : 2.0
**17**	0.92	Fail	N/A	**42**	5.78	[b]	N/A
**18**	5.10	Fail	N/A	**43**	3.07	6.03^[a]^	1.96
**19**	3.00	Fail	N/A	**44**	2.78	Fail	N/A
**20**	1.53	Fail	N/A	**45**	8.99	6.91^[a]^	1:0.8
**21**	0.98	[b]	N/A	**46**	3.12	6.26^[a]^	1 : 2.0
**22**	2.59	[b]	N/A	**47**	Fail	Fail	N/A
**23**	1.65^[d]^	Fail	N/A	**48**	Fail	Fail	N/A
**24**	1.96	8.65^[a]^	1 : 4.4	**49**	Fail	Fail	N/A
**25**	2.24	7.37^[a]^	1 : 3.3	**50**	3.18	6.36^[a]^	1 : 2.0
Ampicillin					0.0003	0.003	1 : 10

[a] Endpoint of experiment predicted due to compound solubility. [b] MIC_50_ value greater than compound solubility. [c] SSA unstable in solution. [d] Clouding of media observed upon addition of SSA solution to bacterial culture, MIC_50_ data should be treated with caution.

The molecular structures of compounds **1**–**50** have been designed using a stepwise approach to the alteration of SSA **1**. For example, **2** and **3** enable us to gain an understanding of the effects of increasing the distance between the urea and anionic group. Compounds **4**–**6** then allow observation of the effect of altering the lipophobicity and hydrogen bond donating and accepting properties of the urea as opposed to the thiourea group. In a similar fashion changing the SSA R groups (**19**–**27**) and, substitution of the phenyl ring system for a butyl chain (**40**–**45**) allows us to understand the effects of substituting electron withdrawing/donating (hydrogen bond activating/deactivating moieties) or hydrophobic/hydrophilic units to the SSA structure. To investigate the effects of the counter cation on SSA properties compounds **7**–**18** and **47**–**50** were obtained while, **28**–**33** were design to enable investigation of the effects of the SSA anion. Finally, **34**–**39** facilitate the exploration of multiple aromatic ring system addition to the core SSA structure while, **38**, **39** and **46** allow us to understand the effects of covalently linking the thio/urea array to the SSA anion.

**Figure 2:**
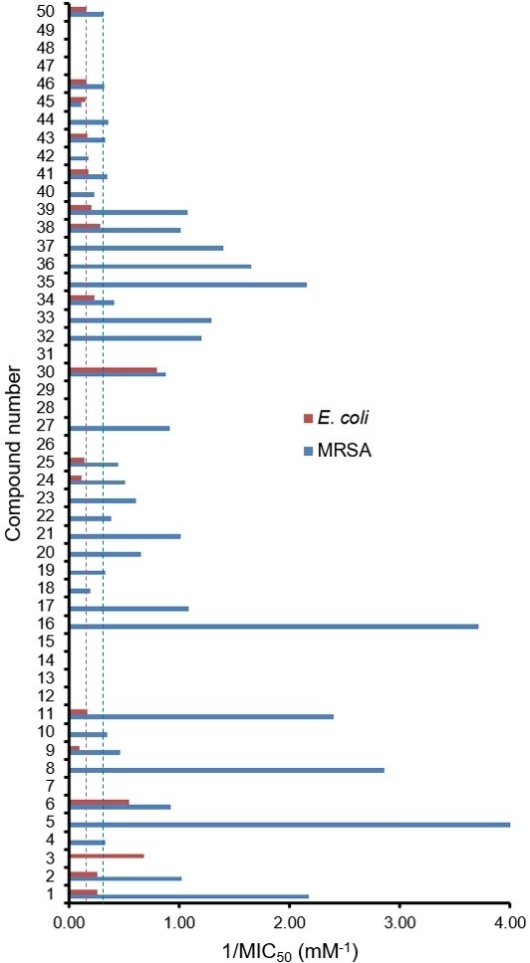
Antimicrobial efficacy of **1**–**50** against model bacteria. Dashed lines indicate the activity of control compound **50** (TBACl) against those same model organisms.

The majority of SSAs were synthesised as the TBA salt however, simple TBA salts are known to exhibit some antimicrobial effects^[28]^ and therefore, the antimicrobial efficacy of SSAs **1**–**46** was compared against that of TBACl (**50**). These data are presented in Figure 2 and show that the presence of the SSA counter anion results in increased antimicrobial activity against MRSA (**1**, **2**, **4**–**6**, **8**–**11**, **16**, **17**, **19**–**25**, **27**, **30**, **32**–**39**, **41**, **43**, **44** and **46**) and *E. coli* (**1**–**3**, **6**, **11**, **30**, **34**, **38** and **39**). Of these SSAs, **5** or **16** (MIC_50_=0.25 mM and 0.27 mM respectively) and **30** (MIC_50_=1.25 mM) were identified as the most effective antimicrobial agents against MRSA and *E. coli*, respectively. Additionally, **30** was identified to be the most effective broad spectrum antimicrobial SSA, demonstrating comparable activity against both model Gram‐negative and Gram‐positive bacteria.

As mentioned previously, the structure of the SSA anion has been designed to selectively interact with specific phospholipid head groups. Further evidence supporting the tailorable selectivity of SSA technologies towards different cell membranes can also be found through comparison of the antimicrobial activity data shown in Table 2 and Figure 2. For example, these data show **8** to be an effective antimicrobial against MRSA but not *E. coli*. However, the reverse is true for **3**. Given this evidence, it is also plausible to hypothesise that, through structural variation of the SSA construct, membrane selectivity may be further fine‐tuned to produce SSA candidates that preferentially interact with different types of bacterial cell membranes. It is known that the phospholipid composition of bacterial membranes differ between microbial species.[^29^, ^30^] Further evidence to support this hypothesis was obtained when ranking the five most effective antimicrobial SSAs against MRSA (**5**≈**16**>**8**>**11**>**1**=**35**) and *E. coli* (**30**>**3**>**6**>**38**>**1**). Here the order of SSA efficacy was found to differ significantly for the model Gram‐positive, which contains a high proportion of phosphatidylglycerol – PG (57 %) but no phosphatidylethanolamine (PE) and Gram‐negative bacteria, which contains a high proportion of PE (85 %) compared to PG (15 %).^[30]^

**Figure 3:**
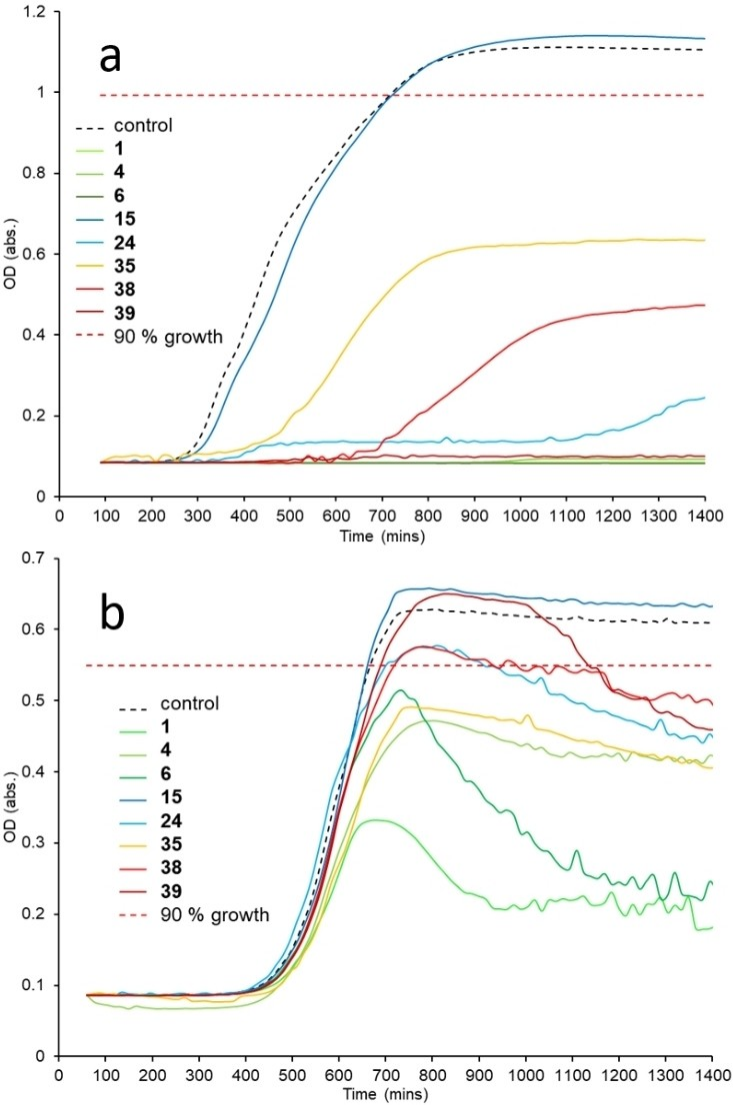
Example screening data obtained for SSAs at 3.3 mM against a) MRSA USA300 and b) *E. coli* DH10B at an initial calibrated cell concentration equal to the 0.5 McFarland standard. Control data demonstrates normal bacterial growth in the absence of **1**–**50**.

It is also known that the phospholipid membrane composition of bacteria alters depending on factors such as stage of life cycle^[31]^ and growth phase, ergo this ranking may change depending on these factors.^[32]^ The results of initial compound antimicrobial screening assays, as highlighted in Figure 3, provides evidence for this. Here, Figure 3a shows that the addition of an SSA to MRSA immediately affects log phase growth where that compound is found to exhibit antimicrobial activity. However, in analogous antimicrobial efficacy experiments conducted against *E. coli* (Figure 3b), the antimicrobial effect of an SSA is only observed after≈600 mins, as the microbial culture enters the stationary phase. Therefore, although MRSA is susceptible to SSAs during log‐phase, the susceptibility of *E. coli* towards SSAs was found to increase as the bacteria enters stationary phase.

### Microscopy studies

**Figure 4:**
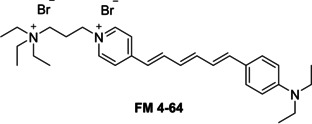
Non‐selective, styryl lipid membrane binding dye, FM 4‐64.

We have previously shown that SSA **39**, when supplied to the cell culture as a 5 % EtOH solution, is presented at the cell surface as a spherical aggregate.^[21]^ This SSA was then shown to coat the surface of MRSA and *E. coli*, before being internalised. Using FM 4‐64,^[33]^ a fluorescent non‐selective lipid binding molecule (Figure 4), competitive membrane association assays were carried out with SSA **39**, to further probe the coordination of SSAs to these bacterial membranes.

**Figure 5:**
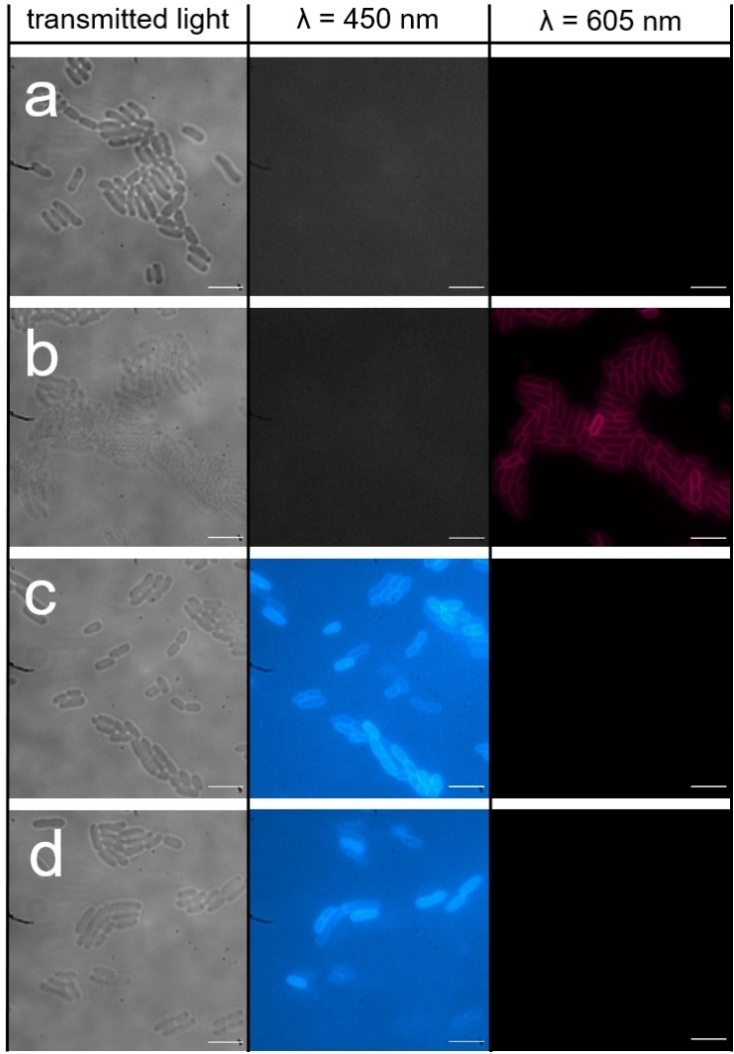
Fluorescence images of *E. coli*: a) in the absence of any compound; b) incubated with membrane binding compound FM 4‐64 (shown in red) for 30 mins; c) incubated with SSA **39** (shown in blue) for 30 mins; d) incubated initially with FM 4‐64 (1 min) followed by the addition of **39** (for 30 mins). Scale bar: 10 μm, fluorescence images produced using a 450 nm or a 605 nm filter.

**Figure 6:**
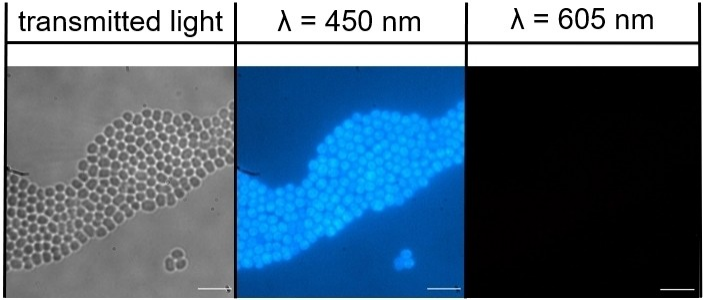
Microscopy images of MRSA in the presence of **39** and FM 4‐64 in combination at T=30 min. This sample was incubated initially with FM 4‐64 for 1 min before the addition of **39**. Scale bars=10 μm, fluorescence images produced using a 450 nm filter or a 605 nm filter.

**Figure 7:**
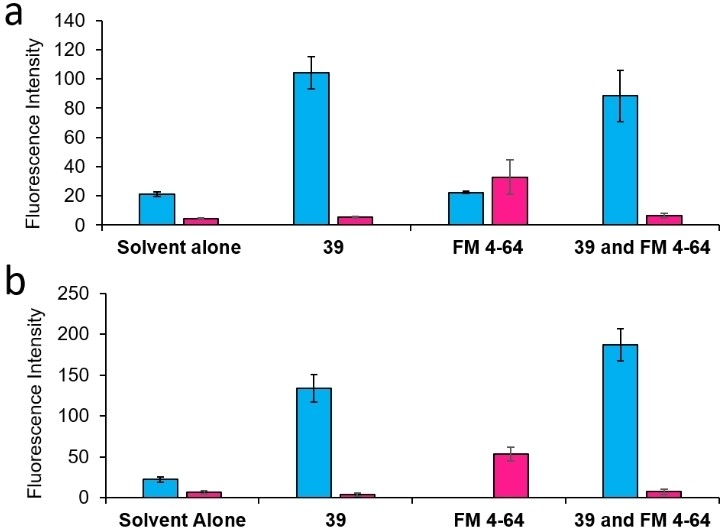
Fluorescence intensity of a) MRSA USA300 cells and b) *E. coli* DH10B cells measured at both 450 nm (blue) and 605 nm (pink) in the absence of FM 4‐64 or **39** (solvent alone) or the presence of **39**, FM 4‐64 or a combination of **39** and FM 4‐64. Results obtained at time T=30 mins. Where both FM 4‐64 and **39** are present, **39** was added 1 min after the addition of FM 4‐64 (T=0 mins). Error=one standard deviation. All quantitative analysis was carried out using FIJI (ImageJ) software.^[35]^

These competitive fluorescence microscopy studies were undertaken with both Gram‐negative (*E. coli*) and Gram‐positive (MRSA) bacteria. Within the scope of these studies, the effects of both SSA **39** and FM 4‐64 addition (when added to the cell culture alone or in combination) were studied. As shown in Figure 5, these two molecules fluoresce at different wavelengths, allowing both molecules to be visualised independently of one another during the experiment. Within the scope of these fluorescence microscopy experiments, a 450 nm filter was used to visualise **39** and a 605 nm filter was used to visualise FM 4‐64 (Figures 5 and 6). In the absence of **39**, FM 4‐64 can clearly be observed binding to the microbial membrane (Figure 5b) however, when **39** is added to a bacterial sample already stained with FM 4‐64 (Figure 5d and Figure 6), there is a decrease in the fluorescence of FM 4‐64 to a background level. This shows **39** to have outcompeted the FM 4‐64, previously bound to the microbial membrane. In order to confirm these observations, comparison of the proportions of FM 4‐64 and **39** associated/interacting with the bacteria were performed through quantitative microscopy analysis.^[34]^ The results of this comparison are summarised in Figure 7. Here we compare the average fluorescence intensity observed under each filter (450 nm and 605 nm) for>30 cells under the experimental conditions described in Figures 5 and 6 and the Supporting Information.

**Figure 8:**
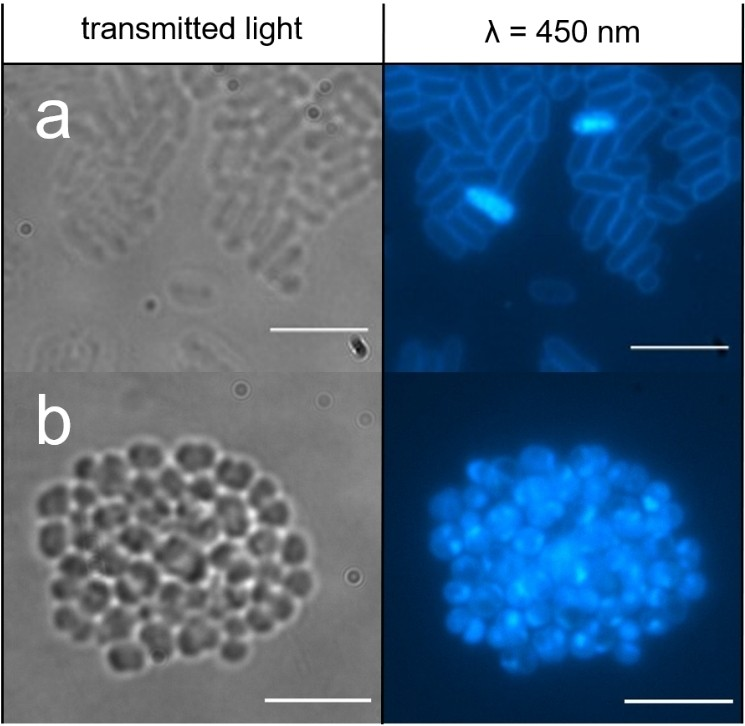
a) The morphology of *E. coli* cells in the presence of **39** at T=4 hrs. b) The morphology of MRSA and *E. coli* cells in the presence of **39** at T=4 hrs. Scale bars=10 μm, fluorescence images produced using a 450 nm filter.

After 4 hours in the presence of **39** this SSA is observed to have internalized within both MRSA and *E. coli*. Interestingly, although the morphology of the *E. coli* cells (Figure 8a) remains similar to the untreated cells seen in Figure 5a, there is a pronounced effect on the morphology of the MRSA cells (Figure 8b). Here there is an apparent loss of membrane integrity, evidenced by the loss of individual cell definition and regular cell shape. This difference in cell morphology over time offers a potential explanation for the five‐fold enhancement in the efficacy of **39** against MRSA over *E. coli* (Figure 2 and Table 2).

### Physicochemical property overview

We have previously hypothesised that SSA antimicrobial activity is linked to SSA physicochemical properties, resulting from molecular structure. It is envisioned that the effective elucidation of molecular structure‐physicochemical property‐antimicrobial activity relationships will allow the informed design of ever‐more effective next generation SSAs, whilst also providing further insights into SSA mode of antimicrobial action. The physicochemical data collected for **1**–**50**, was used in the production of the 14 parameter datasets listed in Table 3. These data include measurements from solution state, solid state, gas phase, and in silico studies. Details of which are supplied within the Supporting Information and summarised where relevant in Tables 4 and 5.

**Table 3:** List of parameters (P) used in the search of SSA molecular structure‐physicochemical property‐antimicrobial activity relationships. The data for **P2** was transferred into binary values for this portion of the study: no=0; yes=1.

P	Parameter description	Unit
**P1**	Molar mass	G mol^−1^
**P2**	SSA dimer in the gas phase (e. g. Table 3)	N/A
**P3**	Dimerisation constant – k_dim_ (e. g. Table 3)	M^−1^
**P4**	Self‐associated aggregate size (e. g. Table 3)	nm
**P5**	Zeta potential (e. g. Table 3)	mV
**P6**	CMC (e. g. Table 3)	mM
**P7**	Surface tension at CMC (e. g. Table 3)	mN m^−1^
**P8**	% of SSA to become NMR silent (e. g. Table 4)	%
**P9**	*E*_min_ of SSA anionic component (e. g. Figure 10)	kJ mol^−1^
**P10**	*E*_max_ of SSA anionic component (e. g. Figure 10)	kJ mol^−1^
**P11**	Log*P* of SSA anionic R group	N/A
**P12**	*E*_min_ of SSA cationic component	kJ mol^−1^
**P13**	*E*_max_ of SSA cationic component	kJ mol^−1^
**P14**	Log*P* of SSA cationic R group	N/A

Solid state characterisation of SSA self‐association.

**Table 4:** Overview of relevant self‐associative data generated for **1**–**6**, **8**–**11**, **14**–**18**, **23**, **27** and **40**–**50** from: gas phase high resolution ESI – ve mass spectrometry studies (presence of SSA anion dimer: Y=yes); solution state ^1^H NMR dilution studies conducted in a [D_6_]DMSO/0.5 % H_2_O mixture at 25 °C to enable the calculation of dimerisation constants (k_*dim*_);^[36]^ solution state characterization of those self‐associated aggregates formed in an EtOH/H_2_O 1 : 19 solution through DLS size, zeta potential stability and tensiometry CMC and surface tension at CMC measurements. A full data table for **1**–**50** can be found in the Supporting Information.

SSA	Gas phase dimer	k_*dim*_ [M^−1^]	Size [nm]	Zeta potential [mV]	CMC [mM]	Surface tension [mN m^−1^]
**1** ^[14]^	Y	2.7	164	−76	10.4	37.45
**2** ^[14]^	Y	0.1	459	−78	10.7	38.49
**3** ^[14]^	Y	3.3	122	−94	8.9	36.78
**4** ^[14]^	Y	[c]	295	−92	24.1	34.35
**5** ^[14]^	Y	0.2	142	−34	6.1	42.24
**6** ^[14]^	Y	2.6	122	−38	5.6	33.59
**8** ^[14]^	Y	[a]	220	−28	198.4	36.16
**9** ^[14]^	Y	6.7^[b]^	164	−24	210.0	41.78
**10** ^[14]^	Y	3.2	190	−26	103.1	33.75
**11** ^[14]^	Y	3.3	190	−48	34.6	36.09
**14**	Y	N/A	N/A	N/A	N/A	N/A
**15**	Y	[c]	235	−21	29.0	30.25
**16**	Y	[c]	[g]	−19	82.3	31.62
**17**	Y	[c]	160	[g]	85.7	33.00
**18**	Y	[c]	192	[g]	17.3	37.73
**23**	Y	63.7	182	−57	3.7	28.65
**27**	Y	[c]	[f]	[f]	[f]	[f]
**40**	Y	<0.1	208	−38	69.7	42.85
**41**	Y	[d]	174	−28	44.9	42.10
**42**	Y	[e]	248	−29	60.5	48.92
**43**	Y	[c]	220	−37	38.4	39.55
**44**	Y	[d]	121	−30	59.6	41.38
**45**	Y	[e]	153	−35	66.5	40.79
**46**	N/A	N/A	N/A	N/A	N/A	N/A
**47**	N/A	N/A	N/A	N/A	N/A	N/A
**48**	N/A	N/A	N/A	N/A	N/A	N/A
**49**	N/A	N/A	N/A	N/A	N/A	N/A
**50**	N/A	N/A	[g]	[g]	198.0	37.10

[a] Multiple association event prevents data fitting. [b] Data should be treated with caution as multiple self‐association events suspected. [c] Slow exchange event prevented data fitting. [d] Overall change in chemical shift<0.01 ppm indicating no complex formation. [e] NH resonances could not be observed. [f] Could not be calculated due to compound solubility. [g] Could not be determined due to lack of reproducibility.

**Table 5:** Overview of results from quantitative ^1^H NMR studies. Values given represent the % of compound to become NMR silent in [D_6_]DMSO/1 % CH_2_Cl_2_ (112 mM) and D_2_O/5 % EtOH (5.56 mM) at 25 °C, due to the formation of large self‐associated aggregates which exhibit solid‐like properties.^§^

SSA	[D_6_]DMSO (1 % CH_2_Cl_2_)	D_2_O (5 % EtOH)	SSA	[D_6_]DMSO (1 % CH_2_Cl_2_)	D_2_O (5 % EtOH)
**1** ^[14]^	0	51	**36** ^[19]^	15^[b]^	92
**4** ^[14]^	0	50	**37** ^[19]^	0	34
**15**	0	46	**39** ^[16]^	0	10
**16**	0	48	**40**	0	56
**17**	0	55	**41**	0	41
**18**	0	55	**42**	0	43
**23**	0	76	**43**	0	47
**27**	0	[a]	**44**	0	48
**30** ^[14]^	0	68	**45**	0	59
**33** ^[14]^	0	59	**50**	0	47
**35** ^[16]^	0	77

[a] Could not be calculated due to compound solubility. [b] Solubility prevented observation at 112 mM, experiments were instead performed at 56 mM.

**Figure 9:**
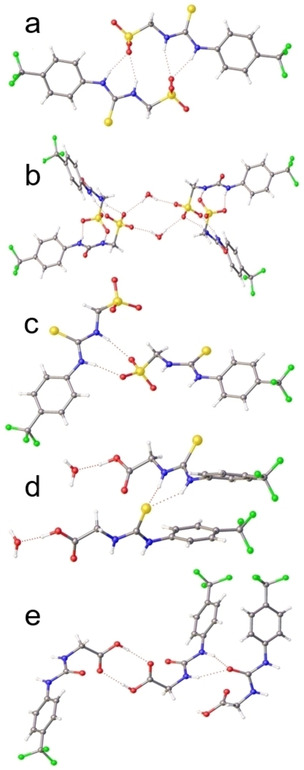
Single crystal X‐ray structure (obtained via slow evaporation of a MeOH/H_2_O solution of the appropriate SSA) of: a) **18**, illustrating dimerisation through urea‐anion complexation. TPA counter cations have been omitted for clarity. Internal angle of dimerisation=179.9 °; b) **11**, illustrating dimerisation through urea‐anion complexation and further association through bridging water molecules. Internal angle of dimerisation=84.7 °. TPA counter cations have been omitted for clarity; c) **17**, illustrating dimerisation through urea‐anion complexation. TEA counter cations have been omitted for clarity; d) **32**, illustrating complexation through thiourea‐thiourea hydrogen bonding. Red=oxygen, blue=nitrogen, green=fluorine, yellow=sulfur, grey=carbon, white=hydrogen; e) **29**, illustrating complexation through urea‐urea stacking and carboxylic acid‐carboxylic acid hydrogen bonding. In all cases red dashed lines=hydrogen bonds.

The anionic portion of an SSA salt contains an uneven number of hydrogen bond donating and hydrogen bond accepting functionalities. This means the anionic unit can access multiple self‐associative binding modes simultaneously. However, we have previously shown the binding modes for this class of amphiphilic salt to be cation‐dependent within the solid state.^[18]^ Here, the self‐associated binding modes of SSAs are observed using single crystal X‐ray diffraction techniques.^≠^ To date we have observed three different self‐associative binding modes for the anionic component of an SSA which are as follows: 1) thio/urea‐anion hydrogen bonded dimers (observed for **1**–**4**, **6**, **11**, **18**–**23**, **26**–**27**, **34**, **37**–**39**); 2) thio/urea‐anion hydrogen bonded tapes (observed for **5**, **8**, **17**, **30**); 3) and thio/urea‐thio/urea hydrogen bonded stacks (observed for **7**, **9**, **29**, **32**).[^14^, ^15^, ^16^, ^17^, ^18^, ^19^] These different hydrogen bonding modes are exemplified by the novel structures shown in Figure 9. Within Figure 9 each hydrogen bond is shown as a red dashed line however, for further crystallographic images or structural information please refer to the Supporting Information. Here, **11** and **18** form thio/urea‐anion hydrogen bonded dimers (Figures 9a and b), **17** was found to form a thiourea‐sulfonate hydrogen bonded tape (Figure 9c), while **29** and **32** were found to form thio/urea‐thio/urea hydrogen bonded stacks (Figures 9d and e).

### Gas phase characterisation of SSA self‐association

Consistent with solid state analysis, gas phase studies also demonstrate the presence of SSA anion dimeric species for the majority of **1**–**45** (Supporting Information and Table 4). However, where the anionic portion of the SSA is protected, or where intramolecular hydrogen bonding events weaken the formation of any potential self‐associative complexes (**28**, **31** and **38**), dimerisation is not observed.

### Solution state characterisation of SSA self‐association

Characterising SSA self‐association events in the solution state is complex and requires a combination of complementary techniques. To aid these efforts, each SSA was studied in two different solvent systems, enabling us to unpick individual association events from the more complex extended aggregate formation. In [D_6_]DMSO, unless specifically stated, the results of quantitative ^1^H NMR^§^, ^1^H NMR self‐association constant determination and ^1^H NMR DOSY experiments (Tables 4 and 5) support the formation of hydrogen bonded dimers whereas, in a 5 % aqueous ethanol solution SSAs are found to form larger self‐associated aggregates (Supporting Information and Tables 4–5). Although there are distinct differences in the self‐associative events taking place within the two different solvent systems, we have previously shown a correlation between the strength of SSA dimerisation (obtained for SSAs within a [D_6_]DMSO/0.5 % H_2_O solution at 25 °C^§§^) and Critical Micelle Concentration (CMC) (established for SSAs in a H_2_O/EtOH 19 : 1 solution).[^14^, ^16^] Additional analysis performed to further characterise the self‐associated SSA aggregates formed within a 19 : 1 H_2_O/EtOH solution includes the use of microscopy^[16]^ and dynamic light scattering (DLS), to establish aggregate size and/or shape, and zeta potential measurements to establish aggregate stability (Table 4 and Supporting Information).

Supporting our previous results,[^14^, ^16^, ^19^] the hydrodynamic diameter of the self‐associated aggregates formed from **15**–**18**, **23** and **40**–**45** at 5.56 mM in a 19 : 1 H_2_O/EtOH solution at 25 °C, obtained from DLS intensity distribution maxima were found to range between 150–250 nm. Complimentary zeta potential measurements performed under the same experimental conditions showed aggregates produced via the self‐association of thiourea‐based SSAs **15**–**18** to form less stable structures than those of the analogous urea‐based SSAs **8**–**11**. The presence of more strongly coordinating counter cations (**15**–**18**) was also shown to result in less stable aggregates relative to the analogous TBA SSA (**4**). Increasing the acidity of the hydrogen bond donor groups through the addition of two (**23**) rather than a single (**1**) CF_3_ functionality was also shown to destabilise the aggregates formed. We hypothesise that this may be due to steric effects caused by the addition of a second bulky CF_3_ moiety to the phenyl ring system. The addition of a second CF_3_ functionality was also found to decrease the CMC from 10.39 mM to 3.39 mM, and the surface tension at CMC from 37.49 mN m^−1^ to 28.65 mN m^−1^, for **1** and **23** respectively. This is attributed to the comparative hydrophobicity of the two phenyl ring systems and increased strength of hydrogen bonded self‐association (k_*dim*_=2.7 M^−1^ and 63.7 M^−1^ for **1** and **23** respectively).

The presence of the more strongly coordinating tetraalkylammonium ions in **16** and **17**, and the replacement of aromatic ring system(s) with butyl chains, was also found to cause a comparative increase in CMC and surface tension measurements (**40**–**45**) when compared to **1**. It is presumed that this is due to the weakening of the self‐associated hydrogen bonded complex through competitive interactions (**16** and **17**), a decrease in cation hydrophobicity (**16** and **17**), or the deactivation of the thio/urea hydrogen bond donor groups (**40**–**45**).

### *In silico* characterisation of SSA self‐association

**Figure 10:**
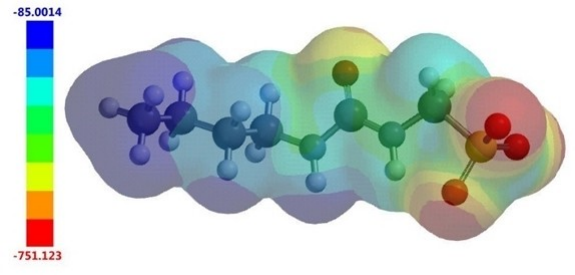
Example electrostatic surface potential map calculated for **41** using Spartan'16, optimised geometries and semi‐empirical PM6 modelling methods. *E*_max_ (blue) and *E*_min_ (red) values depicted in the figure legend are given in kJ mol^−1^ and represent the principle hydrogen bond donating (the most positively charged point on the surface of an SSA) and accepting (the most negatively charged point on the surface of an SSA) groups respectively.

Expanding upon work by Hunter^[37]^ and Stuart,^[38]^ our previous studies indicate that parameter sets derived from electrostatic potential maps (Figure 10) (**P9**, **P10**, **P12** and **P13**) and calculated Log*P* values (**P11** and **P14**) for an SSA anion could be used to predict experimentally derived physicochemical properties for SSAs such as dimerisation constants (**P3** – Table 4) and CMC values (**P6** – Table 4).[^14^, ^16^] In line with these findings, we hypothesise that these parameters (**P9**‐**P14**) may also be used to describe self‐associative non‐covalent complex formation and membrane permeation, and thus inform antimicrobial activity. To explore this possibility, analogous parameters for **1**–**50** (**P9**‐**P14**) have been generated^§§§^ and are detailed within the Supporting Information.

### Molecular structure‐physicochemical property‐antimicrobial activity relationships

**Figure 11:**
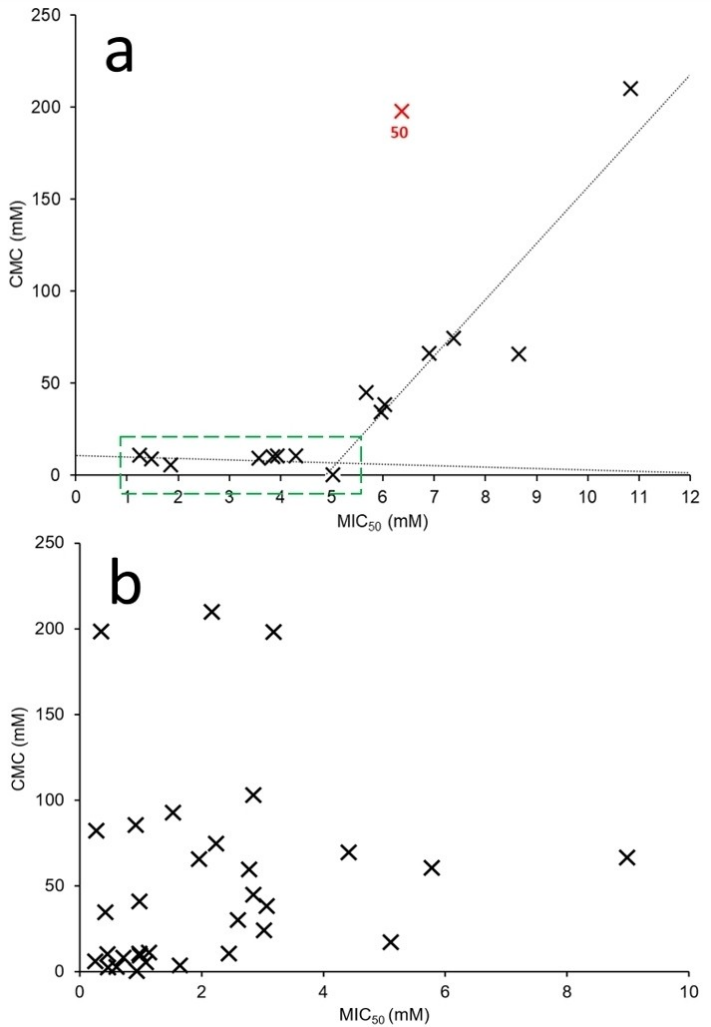
a) Correlations identified for the activity of **1**–**3**, **6**, **9**, **11**, **24**, **25**, **30**, **34**, **38**, **39**, **41**, **43**, **45** and **50** against ***E. coli*** with CMC. Points outlined in green indicate the point at which decreasing CMC (<1 mM) no longer relates to decreasing MIC_50_. Red values indicate potential outliers where differing properties may affect MIC_50_ values. Here potential outliers were identified as those points which deviated the most from the correlation. b) Comparative scatter plot showing a lack of correlation between the efficacy of **1**, **2**, **4**–**6**, **8**–**11**, **16**–**18**, **20**–**25**, **30**, **34**–**45** and **50** against **MRSA** and CMC.

With antimicrobial efficacy for **1**–**50** (Table 2) established against model Gram‐positive and/or Gram‐negative organisms, SSA structure‐physicochemical property‐antimicrobial activity relationships were established for MRSA and *E. coli*, using the physicochemical parameters (**P1**‐**P14**) listed in Table 3. When investigating MIC_50_ values generated for **1**–**50** against *E. coli*, a decreasing CMC was found to correlate with decreasing MIC_50_ value to≈11 mM and≈5 mM respectively (Figure 11a). Therefore, we define the SSA critical CMC value required for the greatest antimicrobial activity against *E. coli* to be achieved to be<11 mM. This suggests that for *E. coli*, SSAs may need to self‐assemble to be active. However, most of the MIC_50_ values measured for SSAs **1**–**45** are lower than the corresponding CMC value determined for the same SSA. This may be explained by observations that show CMC values measured in the absence of other species can be lowered by the presence of biological targets.^[39]^ The only compound which is shown to deviate substantially from the correlation depicted in Figure 11a is **50**. However, **50** is the TBACl control, added to this series of molecules to allow us to establish the antimicrobial activity of the cationic component common to most SSAs. It is therefore expected that this salt will not adopt the same mechanism of action as SSAs **1**–**45**. Interestingly, no analogous correlation could be identified when comparing CMC values to MIC_50_ values generated for MRSA (Figure 11b). This supports the observations made when comparing those data presented in Table 2 and Figures 2–3, which suggest a different mode of action and therefore a dependence on different individual physicochemical SSA properties when considering the efficacy of an SSA towards either MRSA or *E. coli*.

**Figure 12:**
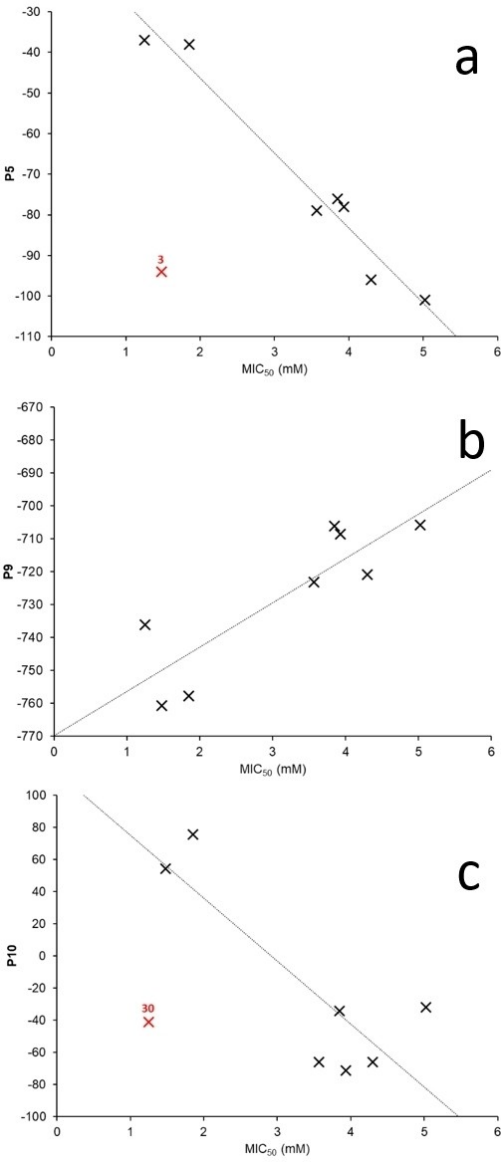
Correlations identified for the activity of **1**–**3**, **6**, **30**, **34**, **38** and **39** against *E. coli* with: a) **P5** (zeta potential); b) **P9** (*E*_min_ of SSA anionic component); and c) **P10** (*E*_max_ of SSA anionic component). Red values indicate potential outliers where differing properties may affect MIC_50_ values. Here potential outliers were identified as those points which deviated the most and, to a greater extent than any remaining points from a correlation.

For those SSAs which demonstrated both an antimicrobial activity against *E. coli* and a CMC<11 mM (**1**–**3**, **6**, **30**, **34**, **38**, **39**), the MIC_50_ values for these SSAs were further correlated against the remaining parameters **P1**–**P14** (excluding **P6** – CMC). The most favorable results from these correlation studies are detailed in Figure 12. Here we see correlations with: zeta potential – **P5** (Figure 12a) which is directly related to SSA aggregate stability; Log*P* of the SSA cationic group – **P9** (Figure 12b), which we have shown to be influential in SSA aggregate stability; and *E*_max_ of SSA anionic component – **P10** (Figure 12c), which is a computer model generated parameter we have previously shown to correlate directly to the strength of SSA self‐association^[16]^ and CMC.^[14]^ Interestingly, the SSAs which exhibit the greatest activity against *E. coli* (**30** and **3**) deviate the most from the correlations depicted in Figures 12a and 12c respectively. We therefore hypothesise that this defines the correlation shown in Figure 12b as the most accurate model to aid in the predictive design of next generation SSAs, with enhanced antimicrobial efficacy towards *E. coli* using physicochemical parameters **P1**–**P14**.

### Exhaustive search methods

As no correlation could be determined for any single parameter (**P1**–**P14**) and the MIC_50_ values determined for **1**–**50** against MRSA, we extended our search to establish SSA structure‐physicochemical property‐antimicrobial activity relationships to include multiple parameters. Here correlations were identified by an exhaustive search of models and possible combinations of parameters. Using the R data analysis software,^[40]^ we completed two types of model searches. Firstly, we searched all possible linear models involving up to four of the parameters listed in Table 3 (Eq. 1). Secondly, we searched all possible combinations of multiplicative models involving two (Eq. 2) and three parameters (Eq. 3).

(1) $$\mathrm{MI}C_{50}=a\cdot \mathrm{PW}+b\;\cdot \;\mathrm{PX}+c\;\cdot \;\mathrm{PY}+d\;\cdot \;\mathrm{PZ}$$

(2) $$\mathrm{MI}C_{50}=a\cdot \mathrm{PX}\cdot \mathrm{PY}$$

(3) $$\mathrm{MI}C_{50}=a\cdot \mathrm{PX}\cdot \mathrm{PY}\cdot \mathrm{PZ}$$

The result of the automated searches for model Eq. 1 which yielded the highest R^2^ value (see Supporting Information), were found to incorporate parameters **P8** ^§^ (% of SSA to become NMR silent), **P10** (*E*_max_ of SSA anionic component), and **P11** (Log*P* of SSA anionic R group). Of the compounds included, **4**, **18**, **42**, **45**, and **50** were shown to deviate the most from the trend identified. As previously discussed, **50** is a control compound, so deviation from this correlation is expected. Both compounds **42** and **45** possess a similar chemical structure, so this may provide supporting evidence that these butyl appended SSAs, which contain a propyl spacer between the thio/urea and anionic units, may adopt a different mode of antimicrobial action. However, there is no clear reason as to why **4** and **18** should also deviate from that correlation identified, meaning that this model may not be the most reliable of those identified.

**Figure 13:**
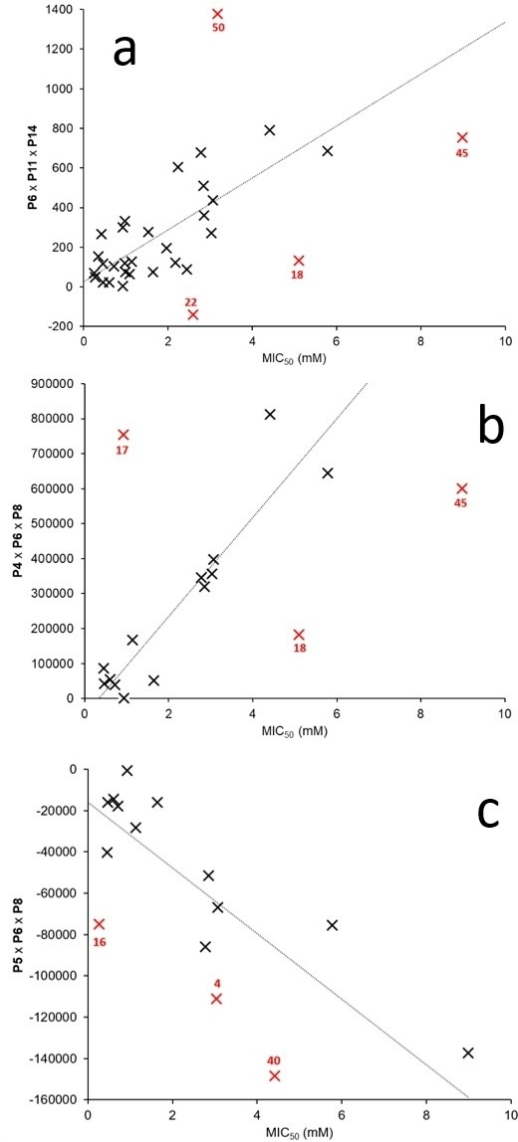
Correlations identified for the activity of **1**–**50** against MRSA with parameters **P1‐P14** demonstrating the highest R^2^ values, generated using Eq. 2 and 3. Red values indicate potential outliers where differing properties may affect MIC_50_ values. **P4**=Self‐associated aggregate size; **P5**=zeta potential; **P6**=CMC; **P8**=% of SSA to become NMR silent; **P11**=Log*P* of SSA anionic R group; **P14**=Log*P* of SSA cationic R group. Here potential outliers were identified as those points which deviated the most from a correlation.

From all fits to Eq. 2 and 3, the parameter combinations which exhibited the three highest R^2^ values (Figure 13) were those with three parameters and, most importantly, all incorporate **P6** (CMC). Although no direct correlation could be identified when directly comparing CMC to MIC_50_ values generated for MRSA (Figure 11b), these complex correlations identified using the exhaustive search methods reinforce the importance of this parameter towards increasing the efficacy of SSAs against MRSA. These results also suggest that SSAs may need to be self‐assembled in order to exhibit antimicrobial efficacy. The correlation identified in Figure 13a includes **1**, **2**, **4**–**6**, **8**–**11**, **16**–**18**, **20**–**25**, **30**, **34**–**45** and **50** and represents the most inclusive relationship identified using the data available. Here compounds **18**, **22**, **45** and **50** are found to deviate the most from the calculated correlation. Interestingly **18**, **45** and **50** were also identified as outliers when fitting these data to model Eq. 1, while **22** was excluded from this previous correlation. This is further supportive evidence that these compounds may elicit an antimicrobial effect through a different mode of action.

Figure 13b shows a correlation between **P4** (self‐associated aggregate size), **P6** (CMC) and **P8** (% of SSA to become NMR silent) and includes MIC_50_ values generated against MRSA for **1**, **4**, **17**, **18**, **23**, **30** and **35**–**45**. Again, **18** and **45** are found to deviate the most from the correlation identified. However, in this instance, **17** is also observed to deviate from this correlation. The final correlation shown in Figure 13c incorporates **P5** (zeta potential), **P6** (CMC) and **P8** (% of SSA to become NMR silent) and **1**, **4**, **16**, **23** and **35**–**45**. In this example, three SSAs (**4**, **16** and **40**) were identified as potential outliers from this correlation. Although **16** is the only SSA analyzed in this way which does not contain a TBA cation, **4** and **40** demonstrate little structural similarity. In summary, although there are six different parameters used to produce the three models detailed in Figure 13, all of the parameters are directly related to the effective formation and/or stability of self‐associated spherical aggregates within an aqueous solution as demonstrated within our previous work.^[14]^ However, our initial work to investigate SSA membrane association events also indicated that phospholipid selective interactions are likely to play a role in SSA antimicrobial activity. This is a factor that is not taken into account within the scope of these studies.^[26]^

## Conclusions

In previous work we developed first generation predictive models to derive SSA CMC and dimerisation values using in silico parameters, generated through low‐level computational modelling.[^14^, ^16^] We have now extended this initial work, establishing the antimicrobial activity of compounds **1**–**50** against model Gram‐positive (MRSA) and Gram‐negative (*E. coli*) bacteria. By expanding our previous library of compounds, we have been able to build structure‐activity models from 14 physiochemical parameters for the first time. This has also enabled us to gain a greater level of antimicrobial mechanistic insight. In addition, the use of fluorescence microscopy to perform competitive membrane binding assays has offered further evidence that SSAs are able to bind to the bacterial membrane. These experiments also offered an explanation as to why the MIC_50_ value for compound **39** is five times greater for Gram‐negative *E. coli* than for Gram‐positive MRSA.

In initial mode of action and physicochemical‐antimicrobial activity analysis we demonstrated the potential of predictive models to develop this class of antimicrobial agents, aided through the exploitation of accessible, low‐level computational modelling. Specifically, a correlation was identified between the CMC>11 mM in EtOH/H_2_O 1: 19 of **1**–**50** and MIC_50_ against *E. coli*. Below this CMC value parameters relating to the stability of aggregate formation were found to predominate. Thus, we propose that SSAs may need to be self‐assembled into larger structures to optimise antimicrobial activity and, through the enhancement of complementary phospholipid/self‐associative hydrogen bonding modes, we will be able to enhance the antimicrobial activity and specificity of future SSA generations. High throughput automated correlation analysis of the MIC_50_ values generated against MRSA indicates that physicochemical parameters associated with the formation and stability of SSA aggregates are also correlated to the antimicrobial activity against this strain of bacteria. However, these relationships appear at present to be more complex than those associated with *E. coli*.

Finally, it is hoped that future utilisation of the next‐generation antimicrobial predictive technologies discussed herein will help to guide the design of novel supramolecular cell surface agents in the fight against AMR, reducing the restrictions placed on antimicrobial drug development, through the lowering of associated costs. Future work in this area is already underway, focusing on investigating the cytotoxicity of SSAs toward human cells as well as the elucidation of pharmacokinetic and pharmacodynamic properties of lead compounds. The results of these studies will feed into the work discuss herein to produce ever more effective, next‐generation SSAs that we hope to become pharmacologically relevant.

## Notes

≠ A suitable crystal of each amphiphile was selected and mounted on a Rigaku Oxford Diffraction Supernova diffractometer. Data were collected using Cu Kα radiation at 100 K. Structures were solved with the ShelXT^[41]^ or ShelXS structure solution programs via Direct Methods and refined with ShelXL^[42]^ on Least Squares minimisation. Olex2^[43]^ was used as an interface to all ShelX programs. CCDC deposition numbers for those structures shown in Figure 9=1935640–1935643 and 1935562.

^§^ Quantitative ^1^H NMR studies conducted with SSAs in both [D_6_]DMSO/CH_2_Cl_2_ 99 : 1 (≈112 mM) and D_2_O/EtOH 19 : 1 (≈5.56 mM) indicate the presence of any large self‐associated aggregates in solution. Since the proportion of SSA employed in the construction of any large aggregates adopts solid‐like characteristics, meaning that it becomes NMR silent in the solution state, although it is also important to note that exchange dynamics in and out of the assembly will also play a role in whether the NMR signals can be seen. Comparative integration with an internal standard (CH_2_Cl_2_ and EtOH respectively) allows quantification of the percentage of SSA apparently ‘lost’ from the NMR experiment. The results of experiments conducted for a representative group of SSAs are summarised in Table 4.

^§§^ Proton NMR dilution studies in [D_6_]DMSO/0.5 % H_2_O at 25 °C (Table 3) were performed in order to quantify the comparative strength of SSA hydrogen bonded dimerisation, through fitting to self‐associative isotherms using BindFit v0.5.^[36]^

^§§§^ Additional *E*_max_ and *E*_min_ values were calculated for both the cationic and anionic components of unpublished molecules **14**–**18**, **23**, **27**, **40**–**50** from electrostatic surface potential maps produced using Spartan'16 and optimised geometries and semi‐empirical PM6 modelling methods. Additional Log*P* values for those same compounds calculated for the phenyl or alkyl substituent attached to the terminal end of the thio/urea functional group of the anionic component for the respective SSA and the cationic component separately using Spartan'16 and optimised geometries at the PM6 level.

## Conflict of interest

The authors declare no conflict of interest.

## Supporting information

As a service to our authors and readers, this journal provides supporting information supplied by the authors. Such materials are peer reviewed and may be re‐organized for online delivery, but are not copy‐edited or typeset. Technical support issues arising from supporting information (other than missing files) should be addressed to the authors.

Supplementary
