# Supplementary material for: Towards the Prediction of Antimicrobial Efficacy for Hydrogen Bonded, Self‐Associating Amphiphiles
Source: ChemMedChem. 2020 Oct 16;15(22):2193–205. doi: 10.1002/cmdc.202000533 (PMC7756459; doi:10.1002/cmdc.202000533)
Supplement: Supplementary file 1 — Supplementary [file CMDC-15-2193-s001.pdf]

ChemMedChem

Supporting Information

Towards the Prediction of Antimicrobial Efficacy for Hydrogen Bonded, Self-Associating Amphiphiles

Nyasha Allen, Lisa J. White[+], Jessica E. Boles[+], George T. Williams, Dominique F. Chu, Rebecca J. Ellaby, Helena J. Shepherd, Kendrick K. L. Ng, Laura R. Blackholly, Ben Wilson, Daniel P. Mulvihill,* and Jennifer R. Hiscock*

Contents

Tables of Data ... 3

Experimental ... 7

Biological experiments [1] ... 8

- **Bacteria experimental** ... 8

Chemical structures ... 10

Chemical Synthesis ... 11

NMR ... 19

- **Characterisation NMR** ... 19
- **^{1}H DOSY NMR experiments** ... 32
- **Quantitative ^{1}H NMR experiments** ... 34
- **^{1}H NMR self-association studies** ... 47

Dynamic Light Scattering data ... 52

- **Overview** ... 67

Surface Tension and Stability Data ... 68

- **Zeta Potential** ... 68
- **Surface Tension Measurements and Critical Micelle Concentration (CMC) Determination** ... 72
- **Overview** ... 78

Single crystal X-ray structures ... 79

In-silico modelling ... 84

- **Overview** ... 88

Mass Spectrum Data ... 89

- **Overview** ... 101

Screening Growth Curves ... 102

- **USA 300 Methicillin-resistant *Staphylococcus aureus* (MRSA)** ... 102
- ***Escherichia coli* (*E. coli*)** ... 107

MIC_{50} Growth Curves ... 112

- **USA 300 Methicillin-resistant *Staphylococcus aureus* (MRSA)** ... 112
- ***Escherichia coli* (*E. coli*)** ... 131

Origin Graphs ... 142

USA 300 Methicillin-resistant *Staphylococcus aureus* (MRSA) 142

***Escherichia coli* (*E. coli*)** 160

Additional physicochemical – antimicrobial activity relationships identified by an exhaustive search of models 170

Quantitative Microscopy Analysis 171

MRSA 171

E. coli 312

Summary Table 462

References 463

Tables of Data

Table S1 - Overview of self-associative binding mode observed for **1**-**9**, **11**, **17**-**23**, **26**-**30**, **32**, **34** and **37**-**39** through single crystal X-ray diffraction data analysis.

Compound	Hydrogen bonding mode	Angle of dimerization (°)	Compound	Hydrogen bonding mode	Angle of dimerization (°)
**1**[4]	dimer	22.6	**21**[4]	dimer	160.7
**2**$_{3}$	dimer	180.0	**22**[4]	dimer	21.2, 19.9
**3**[3]	dimer	170.9, 175.7	**23**[4]	dimer	53.5
**4**[4]	dimer	29.8	**26**[3]	dimer	24.6
**5**[3]	tape	N/A	**27**[4]	dimer	32.6
**6**[3]	dimer	180.0	**28**[3]	solvent	N/A
**7**[4]	stack	N/A	**29**	stack	N/A
**8**[4]	tape	N/A	**30**[3]	tape[a]	N/A
**9**[3]	stack	N/A	**32**	stack	N/A
**11**	dimer	84.7	**34**[5]	dimer	180.0
**17**	tape	N/A	**37**[6]	dimer	26.1
**18**	dimer	179.9	**38**[5]	dimer	180.0
**19**[3]	dimer	180.0	**39**[5]	dimer	55.1
**20**[4]	dimer	73.6			

a - Original SSA underwent reaction during crystallisation process.

Table S2 – Overview of gaseous and solution state studies observed for compounds **1**-**50**.

Compound	Gas phase dimer	k_{dim} (M^{-1})	Size (nm)	Zeta potential (mV)	CMC (mM)	Surface tension (mN m^{-1})
**1**[3]	Y	2.7	164	-76	10.39	37.45
**2**[3]	Y	0.1	459	-78	10.70	38.49
**3**[3]	Y	3.3	122	-94	8.85	36.78
**4**[3]	Y	*c*	295	-92	24.14	34.35
**5**[3]	Y	0.2	142	-34	6.12	42.24
**6**[3]	Y	2.6	122	-38	5.61	33.59
**7**[3]	Y	*a*	531	-55	96.35	36.65
**8**[3]	Y	*a*	220	-28	198.42	36.16
**9**[3]	Y	6.7[b]	164	-24	209.98	41.78
**10**[3]	Y	3.2	190	-26	103.13	33.75
**11**[3]	Y	3.3	190	-48	34.57	36.09
**12**[3]	Y	2.1	*g*	*g*	*g*	*g*
**13**[3]	Y	2.5	*g*	*g*	*g*	*g*
**14**	Y	N/A	N/A	N/A	N/A	N/A
**15**	Y	*c*	235	-21	28.95	30.25
**16**	Y	*c*	*h*	-19	82.27	31.62
**17**	Y	*c*	160	*h*	85.65	33.00
**18**	Y	*c*	192	*h*	17.25	37.73
**19**[3]	Y	1.8	342	-30	*g*	*g*
**20**[3]	Y	0.6	190	-98	92.67	46.14
**21**[5]	Y	0.3	220	-19	40.89	47.90
**22**[3]	Y	4.5	91	*h*	30.29	44.94
**23**	Y	63.7	182	-57	3.69	28.65

**24**[3]	Y	*c*	220	-30	65.83	45.05
**25**[3]	Y	*c*	190	-66	74.59	42.85
**26**[3]	Y	*c*	106	*h*	*g*	*g*
**27**	Y	c	*g*	*g*	*g*	*g*
**28**[3]	N	*d*	*g*	*g*	*g*	*g*
**29**[3]	Y	<0.1	396	*h*	*g*	*g*
**30**[3]	Y	41.4	220	-37	11.21	36.33
**31**[3]	N	6.0	*g*	*g*	*g*	*g*
**32**[3]	Y	5.4	459	-23	*g*	*g*
**33**[3]	Y	104.7	164	-4	*g*	*g*
**34**[3]	Y	<0.1	164	-96	10.67	46.67
**35**[5]	Y	1.5	220	-82	2.52	43.15
**36**[6]	Y	*i*	217	-57	2.75	52.65
**37**[6]	Y	16.8	142	-64	8.17	52.75
**38**[5]	N	0.6	295	-79	9.54	48.71
**39**[5]	Y	2.7	122	-101	0.50	46.50
**40**	Y	<0.1	208	-38	69.73	42.85
**41**	Y	*e*	174	-28	44.91	42.10
**42**	Y	*f*	248	-29	60.49	48.92
**43**	Y	*c*	220	-37	38.44	39.55
**44**	Y	*e*	121	-30	59.64	41.38
**45**	Y	*f*	153	-35	66.46	40.79
**46**	N/A	N/A	N/A	N/A	N/A	N/A
**47**	N/A	N/A	N/A	N/A	N/A	N/A
**48**	N/A	N/A	N/A	N/A	N/A	N/A
**49**	N/A	N/A	N/A	N/A	N/A	N/A
**50**	N/A	N/A	*h*	*h*	198.01	37.10

a – Multiple association event prevents data fitting.
b – Data should be treated with caution as multiple self-association events suspected.
c – Slow exchange event prevented data fitting.
d – Data could not be fitted.
e – Overall chance in chemical shift < 0.01 ppm indicating no complex formation.
f – NH resonances could not be observed.
g – Could not be calculated due to compound solubility.
h – Could not be determined due to lack of reproducibility.
i – Extended aggregate formation prevented binding constant determination.

Table S3 – Overview of the results from quantitative ^{1}H NMR studies. Values given in % represent the observed quantity of compound lost from solution for compounds **1**, **4**, **15-18**, **23**, **27**, **30**, **33**, **35-37**, **39-45** and **50**.

Compound	DMSO-d_6 %	D_2O %	Compound	DMSO-d_6 %	D_2O %
**1**[3]	0	51	**36**[6]	15	92
**4**[3]	0	50	**37**[6]	0	34
**15**	0	46	**39**[5]	0	10
**16**	0	48	**40**	0	56
**17**	0	55	**41**	0	41
**18**	0	55	**42**	0	43
**23**	0	76	**43**	0	47
**27**	0	*a*	**44**	0	48
**30**[3]	0	68	**45**	0	59
**33**[3]	0	59	**50**	0	47
**35**[5]	0	77			

a - could not be calculated due to compound solubility.

Table S4 – Overview of the calculated E_{max}, E_{min} and Log*P* values using semi-empirical PM6 modelling methods of the anionic and cationic components of compounds **1**-**50**. [7]

Compound	Anion			Cation		
	E_{max} (kJ mol^{-1})	E_{max} (kJ mol^{-1})	Log*P*	E_{max} (kJ mol^{-1})	E_{max} (kJ mol^{-1})	Log*P*
**1**[3]	-706.19	-34.28	2.16	203.79	483.66	5.23
**2**[3]	-708.55	-71.20	2.16	203.79	483.66	5.23
**3**[3]	-760.64	54.43	2.16	203.79	483.66	5.23
**4**[3]	-687.15	-17.83	2.16	203.79	483.66	5.23
**5**[3]	-680.84	-61.68	2.16	203.79	483.66	5.23
**6**[3]	-757.75	75.64	2.16	203.79	483.66	5.23
**7**[3]	-706.19	-34.28	2.16	N/A	N/A	N/A
**8**[3]	-706.19	-34.28	2.16	409.80	626.12	0.36
**9**[3]	-706.19	-34.28	2.16	417.86	532.76	0.27
**10**[3]	-706.19	-34.28	2.16	321.70	493.83	1.62
**11**[3]	-706.19	-34.28	2.16	252.18	484.17	3.57
**12**[3]	-706.19	-34.28	2.16	163.20	481.51	6.90
**13**[3]	-706.19	-34.28	2.16	132.00	480.09	8.57
**14**	-687.15	-17.83	2.16	N/A	N/A	N/A
**15**	-687.15	-17.83	2.16	409.80	626.12	0.36
**16**	-687.15	-17.83	2.16	417.86	532.76	0.27
**17**	-687.15	-17.83	2.16	321.70	493.83	1.62
**18**	-687.15	-17.83	2.16	252.18	484.17	3.57
**19**[3]	-727.63	-12.26	-0.17	203.79	483.66	5.23
**20**[3]	-730.37	-48.92	0.57	203.79	483.66	5.23
**21**[5]	-728.74	-69.10	1.55	203.79	483.66	5.23
**22**[3]	-692.64	-16.50	-0.89	203.79	483.66	5.23
**23**	-696.63	-16.22	3.88	203.79	483.66	5.23
**24**[3]	-716.01	-45.05	0.57	203.79	483.66	5.23
**25**[3]	-710.29	-52.82	1.55	203.79	483.66	5.23
**26**[3]	-673.04	2.42	-0.89	203.79	483.66	5.23
**27**	-683.48	-6.17	3.88	203.79	483.66	5.23
**28**	-248.94	232.18	2.16	N/A	N/A	N/A
**29**[3]	-232.38	249.96	2.16	N/A	N/A	N/A
**30**[3]	-736.14	-41.02	2.16	203.79	483.66	5.23
**31**[3]	-217.03	244.14	2.16	N/A	N/A	N/A
**32**[3]	-202.84	260.70	2.16	N/A	N/A	N/A
**33**[3]	-716.37	-28.66	2.16	203.79	483.66	5.23
**34**[5]	-720.79	-65.85	1.60	203.79	483.66	5.23
**35**[5]	-714.30	-60.12	1.65	203.79	483.66	5.23
**36**[6]	-737.00	-63.00	1.65	203.79	483.66	5.23
**37**[6]	-699.00	-29.00	2.42	203.79	483.66	5.23
**38**[5]	-723.07	-66.07	1.50	203.79	483.66	5.23
**39**[5]	-705.73	-31.97	1.67	203.79	483.66	5.23
**40**	-751.12	-85.00	2.17	203.79	483.66	5.23
**41**	-750.95	-88.98	2.17	203.79	483.66	5.23
**42**	-783.10	-16.26	2.17	203.79	483.66	5.23
**43**	-730.15	-68.32	2.17	203.79	483.66	5.23
**44**	-728.71	-88.98	2.17	203.79	483.66	5.23
**45**	-774.74	19.89	2.17	203.79	483.66	5.23
**46**	N/A	N/A	N/A	203.79	483.66	5.23
**47**	N/A	N/A	N/A	417.86	532.76	0.27
**48**	N/A	N/A	N/A	321.70	493.83	1.62
**49**	N/A	N/A	N/A	252.18	484.17	3.57
**50**	-811.67	-202.83	1.33	203.79	483.66	5.23

Table S5 - Overview of MIC_{50} values (mM) determined for **1**-**50** clinically relevant Gram-positive MRSA and Gram-negative *E. Coli*.

Compound	MRSA	*E. coli*	Compound	MRSA	*E. coli*
**1**	0.46	3.85[a]	**26**	*b*	*b*
**2**	0.98	3.93[a]	**27**	1.10[d]	*F*
**3**	*b*	1.48[a]	**28**	*b*	*b*
**4**	3.03	*b*	**29**	*F*	*F*
**5**	0.25	*b*	**30**	1.14	1.25
**6**	1.08	1.85[a]	**31**	*b*	*b*
**7**	*F*	*F*	**32**	0.83	*F*
**8**	0.35	*F*	**33**	0.77	*F*
**9**	2.17	10.84[a]	**34**	2.45	4.30[a]
**10**	2.85	*F*	**35**	0.46[6]	*b*
**11**	0.42	5.96[a]	**36**	0.61[6]	*F*
**12**	*b*	*b*	**37**	0.71[6]	*F*
**13**	*b*	*b*	**38**[d]	0.99	3.57[a]
**14**	*c*	*c*	**39**[d]	0.93	5.02[a]
**15**	*F*	*F*	**40**	4.41	*F*
**16**	0.27	*F*	**41**	2.85	5.67[a]
**17**	0.92	*F*	**42**	5.78	*b*
**18**	5.10	*F*	**43**	3.07	6.03[a]
**19**	3.00	*F*	**44**	2.78	*F*
**20**	1.53	*F*	**45**	8.99	6.91[a]
**21**	0.98	*b*	**46**	3.12	6.26[a]
**22**	2.59	*b*	**47**	*F*	*F*
**23**	1.65[d]	*F*	**48**	*F*	*F*
**24**	1.96	8.65[a]	**49**	*F*	*F*
**25**	2.24	7.37[a]	**50**	3.18	6.36[a]
			**Ampicillin**		

a – End point of experiment predicted due to compound solubility.
b – MIC_{50} determination not possible due to compound solubility.
c – SSA unstable in solution.
d – Clouding of media observed upon addition of SSA solution to bacterial culture, MIC50 data should be treated with caution.
F – Compound failed initial screening.

Experimental

General remarks: A positive pressure of nitrogen and oven dried glassware were used for all reactions. All solvents and starting materials were purchased from known chemical suppliers or available stores and used without any further purification unless specifically stipulated. The NMR spectra were obtained using a Burker AV2 400 MHz or AVNEO 400 MHz spectrometer. The data was processed using ACD Labs, MestReNova or Topspin software. NMR Chemical shift values are reported in parts per million (ppm) and calibrated to the centre of the residual solvent peak set (s = singlet, br = broad, d = doublet, t = triplet, q = quartet, m = multiplet). Tensiometry measurements were undertaken using the Biolin Scientific Theta Attension optical tensiometer. The data was processed using Biolin OneAttension software. A Hamilton (309) syringe was used for the measurements. The melting point for each compound was measured using Stuart SMP10 melting point apparatus. High resolution mass spectrometry was performed using a Bruker microTOF-Q mass spectrometer and spectra recorded and processed using Bruker's Compass Data Analysis software. Infrared spectra were obtained using a Shimadzu IR-Affinity-1 model Infrared spectrometer. The data are analysed in wavenumbers (cm^{-1}) using IRsolution software. DLS and Zeta Potential studies were carried out using Anton Paar Litesizer™ 500 and processed using KalliopeTM

Professional or using a Malvern Zetasizer Nano ZS. Cellular growth curve measurements obtained using Thermo Scientific Multiscan Go 1510-0318C plate reader and recorded using the SkanIt Software 4.0 and a Clariostar plater reader using MARS data analysis software.

Mass Spectrometry: Approximately 1 mg of each compound was dissolved in 1 mL of methanol. This solution was further diluted 100-fold before undergoing analysis where 10 µL of each sample was then injected directly into a flow of 10 mM ammonium acetate in 95 % water (flow rate = 0.02 mL/min).

Self-association constant calculation: Self-association constants were determined using Bindfit v0.5 (http://app.supramolecular.org/bindfit/). All the data can be accessed online using the hyperlinks provided.

Tensiometry Studies: All the samples were prepared in an EtOH: H2O (1:19) solution. All samples underwent an annealing process in which the various solutions were heated to approximately 40 °C before being allowed to cool to room temperature, allowing each sample to reach a thermodynamic minimum. All samples were prepared through serial dilution of the most concentrated sample. Three surface tension measurements were obtained for each sample at a given concentration, using the pendant drop method. The average values were then used to calculate the critical micelle concentration (CMC).

DLS Studies: All vials used for preparing the samples were clean dry. All solvents used were filtered to remove any particulates that may interfere with the results obtained. Samples of differing concentrations were obtained through serial dilution of a concentrated solution. All samples underwent an annealing process, in which they were heated to 40 °C before being allowed to cool to 25 °C. A series of 9 or 10 runs were recorded at 25 °C.

Zeta Potential Studies: All vials used for preparing the samples were clean dry. All solvents used were filtered to remove any particulates that may interfere with the results obtained. All samples underwent an annealing process in which the various solutions were heated to approximately 40 °C before cooling to room temperature, allowing each sample to reach a thermodynamic minimum. The final zeta potential value given is an average of the number of experiments conducted at 25 °C.

Single Crystal X-ray Studies: A suitable crystal of each amphiphile was selected and mounted on a Rigaku Oxford Diffraction Supernova diffractometer. Data were collected using Cu Kα radiation at 100 K or 293 K as necessary due to crystal instability at lower temperatures. Structures were solved with the ShelXT or ShelXS structure solution programs via Direct Methods and refined with ShelXL by Least Squares minimisation. Olex2 was used as an interface to all ShelX programs (CCDC 1866274-1866275).

Biological experiments [1]

Bacteria experimental

Preparation of Luria Broth media (LB): Yeast extract (5 g), tryptone (10 g) and sodium chloride (10 g) were dissolved in dH_2O (1 L) then divided into bottles and autoclaved.

Preparation of Luria Broth (LB) agar plates: Agar (6 g) was added to LB (400 mL) and autoclaved. Once cool, the LB agar was poured into sterile petri dishes under sterile conditions and allowed to set. LB plates were stored at 4 °C until use.

Preparation of McFarland standard: Barium chloride (1%, 50 µL) was added to sulfuric acid (1 %, 9.95 mL) and mixed. The optical density was recorded at 600 nm.

Preparation of antimicrobial compounds for screening: Compounds were dissolved in 5 % ethanol to make up 20 mM solutions on the day of experiment.

Preparation of antimicrobial compounds for MIC_{50} calculations: Compounds were dissolved into 5 % ethanol to make up solutions on the day of experiment. Eight concentrations ranging were made up using 5 % ethanol.

Preparation of bacterial plates: Sterile LB agar plates were streaked using the desired bacteria (either *Escherichia coli* or USA300 *Methicillin-Resistant Staphylococcus aureus)* then incubated at 25°C overnight.

Preparation of Inoculum: An initial culture was made up by inoculating LB media (5 mL) with at least 4 single colonies of the desired bacteria under sterile conditions and incubating at 37 °C overnight. The following day, a subculture was made up using LB (5 mL) and the initial culture (50 µL), then incubated at 37°C until the culture had reached an optical density (OD) of 0.4 at 600 nm. Optical Density was adjusted using sterile dH2O to equal 0.5 McFarland standard (10^7 – 10^8 cfu/mL), then a 1:10 dilution was carried out using sterile dH2O (900 µL) and the McFarland adjusted suspension (100 µL). A final dilution (1:100) was carried using the 1:10 suspension (150 µL) and LB (14.85 mL) before use to achieve a final cell concentration of 10^5 cfu/mL.

Preparation of 96 well microplate for screening: 20 mM solutions of each compound to be tested were made up using 5 % ethanol. The 1:100 cell suspension (150 µL) was pipetted into the wells. Compound solutions (30 µL) were added into 6 wells on the plate so that 14 compounds could be screened on each plate. The final screening concentration for each compound was 3.3 mM in the well. These were incubated for 20 hours in a plate reader, with optical density readings being taken at 600 nm every 15 minutes. Optical density readings were plotted against time to produce growth curves. Compounds that inhibited growth by 10 % or more were taken forward for MIC_{50} calculations.

Preparation of 96 well microplate for MIC_{50}: The 1:100 cell suspension (150 µL) was dispensed into individual wells under sterile conditions. Compounds (30 µL) were added to the wells to equal a total volume of 180 µL in the wells. Six repeats of each concentration for each compound were created each plate. The plates were sealed using parafilm, then incubated at 37°C in a microplate reader for 18-25 hours. Optical density readings were taken at 600 nm every 15 min. Data was used to generate growth curves.

Calculation of MIC_{50}: Growth curves were plotted using OD_{600} optical density readings in Microsoft ® Excel ® 2013. OD_{600} optical density readings 900 minutes from the start of each of these growth cultures for each concentration of drug were plotted in Origin ® 2015. The resultant curve was normalized and fitted using the Boltzmann fit to define the MIC_{50} values for each drug.

Competitive binding assay (*E. coli* and MRSA)

Cultures: An initial culture was created by inoculating LB media (5 mL) with at least 4 single colonies of desired bacteria under sterile conditions and then incubating at 37 °C overnight. The following day, the initial culture (100 µL) was used to inoculate fresh LB media (10 mL) and incubated at 37 °C to form a secondary culture. This was grown to mid log phase (~ OD 0.4), then each culture was separated into four 1.5 mL Eppendorfs under sterile conditions (500 µL in each). In the Eppendorf with no compound or FM 4-64 present, only the cell culture, Fresh LB (100 µL) and 5% ethanol (100 µL, v/v) was added (total volume 700 µL). In the Eppendorf with only FM 4-64 present, the cell culture (500 µL), fresh LB (100 µL) 5% ethanol (100 µL) and FM 4-64 (0.7 µL from a stock solution of 5 $ugmL^{-1}$) were added. For the Eppendorf with only compound **39** present, the cell culture (500 µL), fresh LB (100 µL) and compound **39** (100 µl, 5

mM) dissolved in 5% ethanol. For the Eppendorf with both compound **39** and FM 4-64 present, the culture (500 µL), fresh LB (100 µL) were added, then FM 4-64 was added before adding compound **39** (100 µL) roughly a minute after. These Eppendorfs were incubated for 30 minutes at 25 °C, then microscopy samples (6-8 µL) were taken.

Microscopy: Cells were mounted onto the agarose LB pads under coverslips as described previously.[8] Samples were visualised using an Olympus IX71 microscope with UAPON 150x 1.45 NA TIRF lens mounted on a PIFOC z-axis focus drive (Physik Instrumente, Karlsruhe, Germany), and illuminated using LED light sources (Cairn Research Ltd, Faversham, UK) with appropriate filters (Chroma, Bellows Falls, VT). Samples were visualised using a Zyla 4.2 CMOS camera (Andor), and the system was controlled with Metamorph software (Molecular Devices). Images were analysed using Metamorph and Fiji software.[9]

Chemical structures

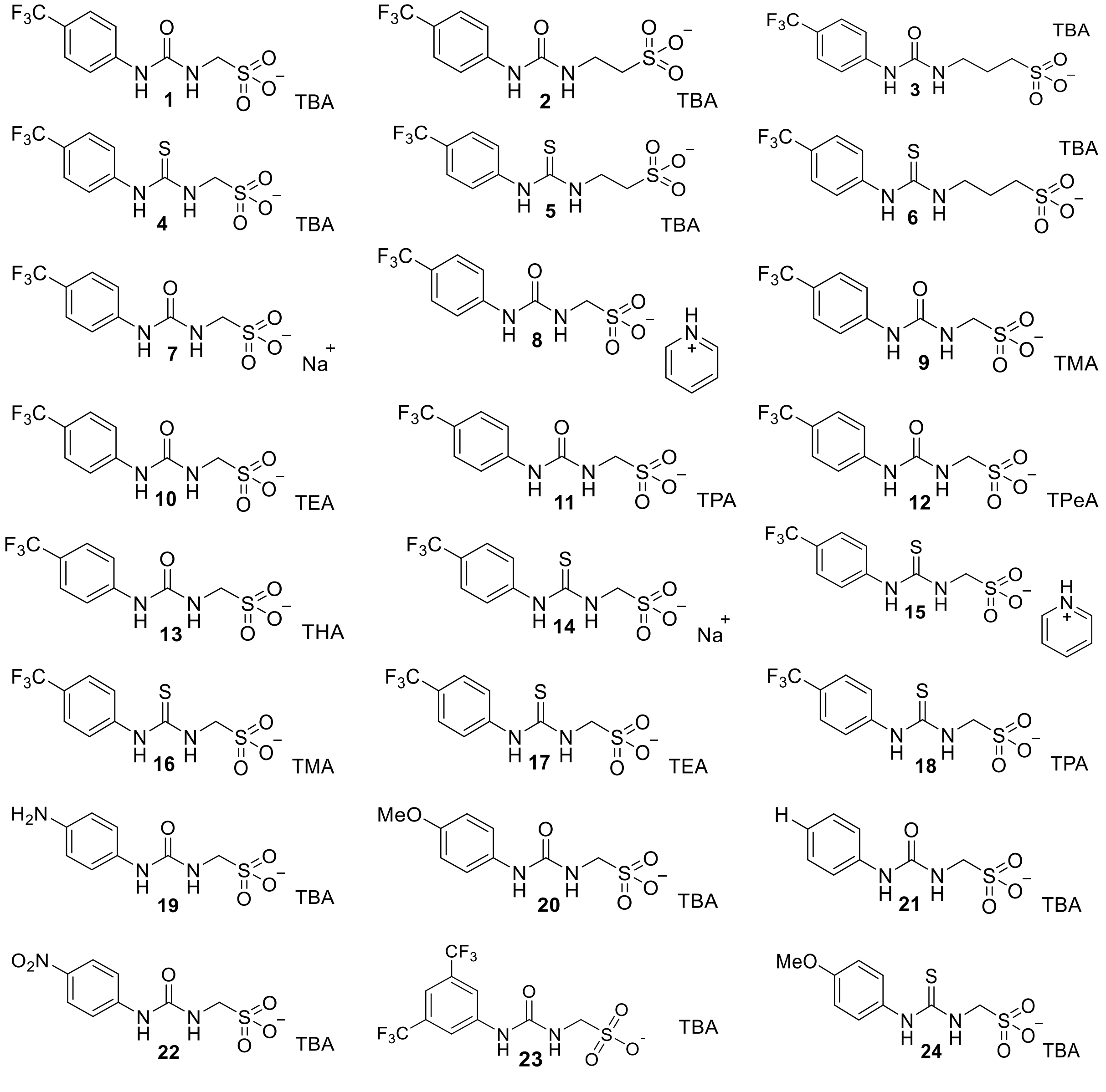

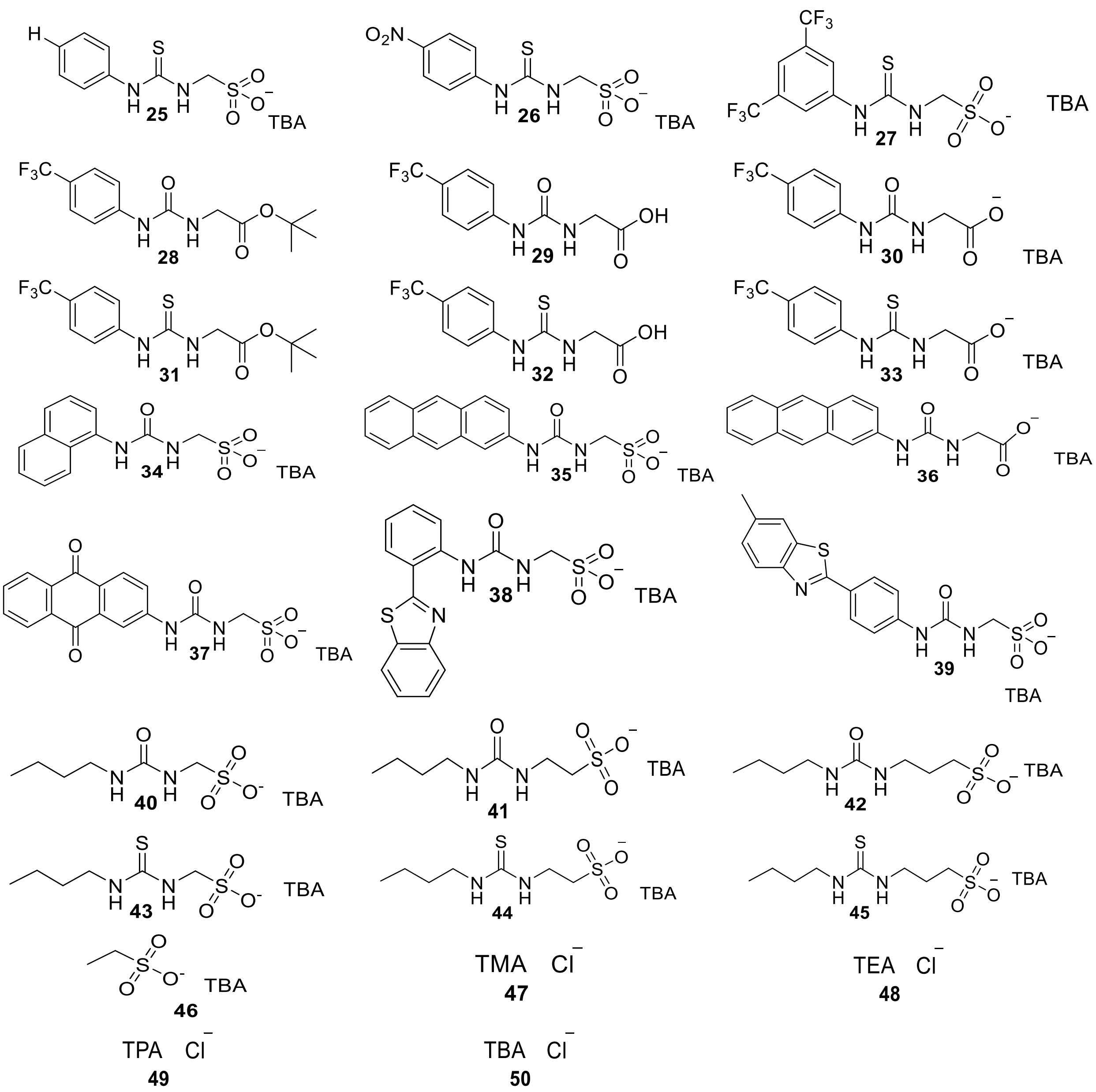

Figure S1 - TMA = Tetramethylammonium, TEA = tetraethylammonium, TPA = Tetrapropylammonium, TBA = Tetrabutylammonium, TPeA = Tetrapentylammonium, THA = Tetrahexylammonium.

Chemical Synthesis

Compound 1: This compound was synthesised in line with our previously published methods. Proton NMR were found to match our previously published values.[2] ^{1}H NMR (400 MHz, 298.15 K, DMSO-d_6): δ: 9.26 (s, 1H), 7.56 (d, *J* = 8.72 Hz, 2H), 7.49 (d, *J* = 8.72 Hz, 2H), 6.98 (t, *J* = 5.96 Hz, 1H), 3.93 (d, *J* = 5.96 Hz, 2H), 3.18 - 3.14 (m, 8H), 1.60 - 1.52 (m, 8H), 1.35 - 1.25 (m, 8H), 0.92 (t, *J* = 7.36 Hz, 12H).

Compound 2: This compound was synthesised in line with our previously published methods. Proton NMR were found to match our previously published values.[3] ^{1}H NMR (400 MHz, 298.15 K, DMSO-d_6): δ: 9.35 (s, 1H), 7.60 (d, *J* = 8.71 Hz, 2H), 7.53 (d, *J* = 8.81 Hz, 2H), 6.53 (t, *J* = 5.33 Hz, 1H), 3.38 (t, *J* = 6.04 Hz, 2H), 3.15 (m, 8H), 2.56 (t, *J* = 5.92 Hz, 2H), 1.55 (m, 8H), 1.40 - 1.20 (m, 8H), 0.92 (t, *J* = 7.33 Hz, 12H).

Compound 3: This compound was synthesised in line with our previously published methods. Proton NMR were found to match our previously published values.[3] ^{1}H NMR (400 MHz, 298.15 K, DMSO-d_6): δ: 8.93 (s, 1H), 7.52 (dd, *J* = 21.49, 8.84 Hz, 4H), 6.41 (t, *J* = 5.75 Hz, 1H), 3.11 (m, 10H), 2.44 - 2.37 (m, 2H), 1.75 - 1.61 (m, 2H), 1.52 (m, 8H), 1.36 - 1.12 (m, 8H), 0.89 (t, *J* = 7.34 Hz, 12H).

Compound 4: This compound was synthesised in line with our previously published methods. Proton NMR were found to match our previously published values.[4] ^{1}H NMR (400 MHz, 333.15 K, DMSO-d_6): δ: 10.28 (s, 1H), 8.19 (br s, 1H), 7.87 (br s, 2H), 7.60 (d, *J* = 8.24 Hz, 2H), 4.34 (br s, 2H), 3.20 - 3.16 (m, 8H), 1.63 - 1.55 (m, 8H), 1.33 (m, 8H), 0.94 (t, *J* = 7.33 Hz, 12H).

Compound 5: This compound was synthesised in line with our previously published methods. Proton NMR were found to match our previously published values.[3] ^{1}H NMR (400 MHz, 298.15 K, DMSO-d_6): δ: 10.23 (s, 1H), 8.23 (s, 1H), 7.68 (dd, *J* = 49.51 Hz, 8.62 Hz, 4H), 3.76 (d, *J* = 5.33 Hz, 2H), 3.16 (m, 8H), 2.69 (t, *J* = 12.32 Hz, 2H), 1.66 - 1.42 (m, 8H), 1.42 - 1.12 (m, 8H), 0.93 (t, *J* = 7.34 Hz, 12H).

Compound 6: This compound was synthesised in line with our previously published methods. Proton NMR were found to match our previously published values.[3] ^{1}H NMR (400 MHz, 333.15 K, DMSO-d_6): δ: 9.95 (s, 1H), 8.25 (s, 1H), 7.77 (d, *J* = 8.10 Hz, 2H), 7.62 (d, *J* = 8.66 Hz, 2H), 3.56 (d, *J* = 5.29 Hz, 2H), 3.21 - 3.10 (m, 8H), 1.94 - 1.75 (m, 2H), 1.56 (m, 8H), 1.39 - 1.20 (m, 8H), 0.93 (t, *J* = 7.33 Hz, 12H).

Compound 7: This compound was synthesised in line with our previously published methods. Proton NMR were found to match our previously published values.[4] ^{1}H NMR (400 MHz, 298.15 K, DMSO-d_6): δ: 3.93 (d, *J* = 5.95 Hz, 2H), 6.87 (t, *J* = 4.58 Hz, 1H), 7.51 (d, *J* = 9.16 Hz 2H), 7.56 (d, *J* = 8.70 Hz, 2H), 9.22 (s, 1H).

Compound 8: This compound was synthesised in line with our previously published methods. Proton NMR were found to match our previously published values. [4] ^{1}H NMR (400 MHz, 298.15 K, DMSO-d_6): δ: 9.17 (s, 1H), 8.87 (d, *J* = 4.58 Hz, 2H), 8.45 (t, *J* = 6.41 Hz, 1H), 7.95 (t, *J* = 7.33 Hz, 2H), 7.56 (s, 4H), 6.60 (s, 1H), 3.88 (d, *J* = 5.48 Hz, 2H).

Compound 9: This compound was synthesised in line with our previously published methods. Proton NMR were found to match our previously published values.[3] ^{1}H NMR (400 MHz, 298.15 K, DMSO-d_6): δ: 9.18 (s, 1H), 7.55 (s, 4H), 6.68 (s, 1H), 3.89 (d, *J* = 5.89 Hz, 2H), 3.09 (s, 12H).

Compound 10: This compound was synthesised in line with our previously published methods. Proton NMR were found to match our previously published values.[3] ^{1}H NMR (400 MHz, 298.15 K, DMSO-d_6): δ: 9.33 (s, 1H), 7.78 - 7.18 (m, 5H), 4.01 (s, 2H), 3.18 (d, *J* = 3.71 Hz, 8H), 1.13 (s, 12H).

Compound 11: This compound was synthesised in line with our previously published methods. Proton NMR were found to match our previously published values.[3] ^{1}H NMR (400 MHz, 298.15 K, DMSO-d_6): δ: 9.19 (s, 1H), 7.56 (s, 4H), 6.69 (s, 1H), 3.89 (d, *J* = 5.87 Hz, 2H), 3.23 - 2.95 (m, 8H), 1.61 (q, *J* = 14.96 Hz, 7.31 Hz, 8H), 0.89 (t, *J* = 7.27 Hz, 12H).

Compound 12: This compound was synthesised in line with our previously published methods. Proton NMR were found to match our previously published values.[3] ^{1}H NMR (400 MHz, 298.15 K, DMSO-d_6): δ 9.20 (s, 1H), 7.48 (dd, *J* = 23.57 Hz, 8.5 Hz, 4H), 6.89 (s, 1H), 3.88 (d, *J* = 5.65 Hz, 2H), 3.31 (d, *J* = 1.11 Hz, 4H), 3.21 - 3.01 (m, 8H), 2.45 (d, *J* = 1.23 Hz, 2H), 1.52 (s, 1H), 1.39 - 1.02 (m, 17H), 0.83 (t, *J* = 6.82 Hz, 12H).

Compound 13: This compound was synthesised in line with our previously published methods. Proton NMR were found to match our previously published values.[3] ^{1}H NMR (400 MHz, 298.15 K, DMSO-d_6): δ: 9.42 (s, 1H), 7.51 (dd, *J* = 44.67 Hz, 8.27 Hz, 4H), 7.23 (s, 1H), 3.96 (d, *J* = 5.54 Hz, 2H), 3.15 (d, *J* = 12.42 Hz, 8H), 1.55 (s, 8H), 1.27 (s, 24H), 0.86 (s, 12H).

Compound 14: Aminomethane sulfonic acid (0.22 g, 2.00 mM) was added to a stirring solution of 4-(Trifluoromethyl) phenyl isothiocyanate (0.41 g, 2.00 mM) in anhydrous pyridine (20 mL) under an inert atmosphere. The mixture was heated to 60 °C overnight. The pyridinium salt was then removed by filtration as a white solid with a yield of 78 % (0.62 g, 1.57 mM). The pyridinium salt (0.59 g, 1.50 mM) was dissolved in a solution of sodium hydroxide (0.059 g, 1.50 mM) to give the pure product as a white solid with a yield of 100 % (0.50 g, 1.50 mM); This compounds presence was verified by HRMS for the sulfonate-thiourea ion ($C_9H_8F_3N_2O_3S_2^-$) (ESI$^-$): m/z: act: 312.9934 [M]$^-$ cal: 312.9934 [M]$^-$. Although compound **14** was obtained, it was found to be unstable so could not be investigated further.

Compound 15: Aminomethane sulfonic acid (0.22 g, 2.00 mM) was added to a stirring solution of 4-(Trifluoromethyl) phenyl isothiocyanate (0.41 g, 2.00 mM) in anhydrous pyridine (10 mL) under an inert atmosphere. The mixture was heated to 60 °C overnight. The pyridinium salt was then removed by filtration to give the product as a white solid with a yield of 65 % (0.51 g, 1.31 mM); Melting Point: 178 °C; ^{1}H NMR (400 MHz, 333.15 K, DMSO-d_6): δ: 10.19 (s, 1H), 8.90 (d, *J* = 5.35 Hz, 2H), 8.57 (t, *J* = 7.83 Hz, 1H), 8.19 (s, 1H), 8.05 (t, *J* = 6.72 Hz, 2H), 7.83 (s, 1H), 7.60 (d, *J* = 8.44 Hz, 2H), 4.36 (s, 2H); ^{13}C{^{1}H} NMR (100 MHz, 298.15 K, DMSO-d_6): δ: 180.7 (CS), 144.1 (ArC), 127.7 (ArCH), 125.9 (d, *J* = 3.53 Hz, ArC), 124.9 (q, *J* = 270.0 Hz, CF_3), 123.9 (ArCH), 123.6 (ArCH), 122.6 (ArCH), 122.0 (ArCH), 60.4 (CH_2); IR (film): ν (cm^{-1}) = 3125 (NH stretch), 1697, 1155, 1037, 840; HRMS for the sulfonate-thiourea ion ($C_9H_8F_3N_2O_3S_2^-$) (ESI$^-$): m/z: act: 312.9933 [M]$^-$ cal: 312.9934 [M]$^-$.

Compound 16: Aminomethane sulfonic acid (0.44 g, 4.00 mM) was added to a stirring solution of 4-(Trifluoromethyl) phenyl isothiocyanate (0.82 g, 4.00 mM) in anhydrous pyridine (20 mL) under an inert atmosphere. The mixture was heated to 60 °C overnight. The pyridinium salt was then removed by filtration as a white solid with a yield of 78 % (1.23 g, 3.14 mM). The pyridinium salt (0.78 g, 2.00 mM) was dissolved in a solution of tetramethylammonium (TMA) hydroxide pentahydrate (0.19 g) and H_2O (0.90 g) to give the pure product as a white solid with a yield of 98 % (0.77 g, 1.96 mM); Melting Point: 192 °C; ^{1}H NMR (400 MHz, 333.15 K, DMSO-d_6): δ: 10.29 (s, 1H), 8.20 (s, 1H), 7.84 (d, *J* = 7.42 Hz, 2H), 7.61 (d, *J* = 8.29 2H), 4.26 (s, 2H), 3.13 (s, 12H); ^{13}C{^{1}H} NMR (100 MHz, 298.15 K, DMSO-d_6): δ: 180.1 (CS), 144.1 (ArC), 127.3 (ArCH), 125.9 (d, *J* = 3.74 Hz, ArCH), 124.9 (q, *J* = 269.94 Hz, CF_3), 123.8 (q, *J* = 269.94 Hz, ArC), 122.6 (ArCH), 122.1 (ArCH), 60.4 (CH_2), 54.7 (CH_3); IR (film): ν (cm^{-1}) = 3094 (NH stretch), 1689, 1159, 1031, 842; HRMS for the sulfonate-thiourea ion ($C_9H_8F_3N_2O_3S_2^-$) (ESI$^-$): m/z: act: 312.9914 [M]$^-$ cal: 312.9934 [M]$^-$.

Compound 17: This compound was produced with an analogous method to that described with the synthesis of compound **16**. The pyridinium salt (0.78 g, 2.00 mM) was dissolved in a solution of tetraethylammonium (TEA) hydroxide in H_2O (0.53 g) to give the pure product as a white solid with a yield of 99 % (0.87 g, 1.97 mM); Melting Point: 158 °C; ^{1}H NMR (400 MHz, 298.15 K, DMSO-d_6): δ: 10.33 (s, 1H), 7.82 (d, *J* = 7.60 Hz, 2H), 7.60 (d, *J* = 8.44 Hz, 2H), 4.25 (s, 2H), 3.23 (q, *J* = 7.24 Hz 8H), 1.20 (t, 7.16 Hz, 12H); ^{13}C{^{1}H} NMR (100 MHz, 298.15 K, DMSO-d_6): δ: 180.6 (CS), 144.3 (ArC), 125.9 (ArC), 124.9 (d, *J* = 269.05 Hz, CF_3), 123.7 (ArCH), 123.5 (ArCH), 122.6 (ArCH), 122.1 (ArCH), 60.6 (CH_2), 51.9 (CH_2), 7.5 (CH_3); IR (film): ν (cm^{-1}) = 3116 (NH stretch), 1614, 1157, 1039, 841; HRMS for the sulfonate-thiourea ion ($C_9H_8F_3N_2O_3S_2^-$) (ESI$^-$): m/z: act: 312.9907 [M]$^-$ cal: 312.9934 [M]$^-$.

Compound 18: This compound was produced with an analogous method to that described with the synthesis of compound **16**. The pyridinium salt (0.78 g, 2.00 mM) was dissolved in a solution of Tetrapropylammonium (TPA) hydroxide in H_2O (0.73 g) to give the pure product as a white solid with a yield of 88 % (0.98 g, 1.97 mM); Melting Point: 139 °C; ^{1}H NMR (400 MHz, 298.15 K, DMSO-d_6): δ: 8.61 (s, 1H), 7.83 (d, *J* = 5.44 Hz, 2H), 7.59 (d, *J* = 8.24 Hz, 2H), 4.28 (s, 2H), 3.14 (t, *J* = 8.08 Hz, 8H), 1.65 (m, 8H), 0.92 (t, *J* = 7.04 Hz, 12H); ^{13}C{^{1}H} NMR (100 MHz, 298.15 K, DMSO-d_6): δ: 180.6 (CS), 144.2 (ArC), 125.9 (ArCH), 124.9 (d, *J* = 269.52 Hz, CF_3), 123.7 (ArC), 123.5 (ArCH), 122.6 (ArCH), 122.1 (ArCH), 60.6 (CH_2), 59.7 (t, *J* = 2.54 Hz, CH_2), 15.3 (CH_2), 11.0 (CH_3); IR (film): ν (cm^{-1}) = 3199 (NH stretch), 1616, 1165, 1037, 851; HRMS for the sulfonate-thiourea ion ($C_9H_8F_3N_2O_3S_2^-$) (ESI$^-$): m/z: act: 312.9911 [M]$^-$ cal: 312.9934 [M]$^-$.

Compound 19: This compound was synthesised in line with our previously published methods. Proton NMR were found to match our previously published values.[3] ^{1}H NMR (400 MHz, 298.15 K, DMSO-d_6): δ: 8.42 (s, 1H), 7.04 (d, *J* = 8.64 Hz, 2H), 6.51 (d, *J* = 8.64 Hz, 2H), 6.41 (t, *J* = 5.92 Hz, 1H), 3.88 (d, *J* = 5.93 Hz, 2H), 3.28 - 3.01 (m, 8H), 1.73 - 1.40 (m, 8H), 1.29 (m, 8H), 0.92 (t, *J* = 7.33 Hz, 12H).

Compound 20: This compound was synthesised in line with our previously published methods. Proton NMR were found to match our previously published values.[4] ^{1}H NMR (400 MHz, 298.15 K, DMSO-d_6): δ: 8.62 (s, 1H), 8.70 (d, *J* = 7.27 Hz, 2H), 6.79 (d, *J* = 9.16 Hz, 2H), 6.50 (t, *J* = 5.95 Hz, 1H), 3.87 (d, *J* = 5.95 Hz, 2H), 3.68 (s, 3H), 3.18 - 3.13 (m, 8H), 1.60 - 1.52 (m, 8H), 1.30 (m, 8H), 0.93 (t, *J* = 7.33 Hz, 12H).

Compound 21: This compound was synthesised in line with our previously published methods. Proton NMR were found to match our previously published values.[4] ^{1}H NMR (400 MHz, 298.15 K, DMSO-d_6): δ: 8.76 (s, 1H), 7.36 (d, *J* = 8.28 Hz, 2H), 7.21 (t, *J* = 7.32 Hz, 2H), 6.87 (t, *J* = 7.32 Hz, 1H), 6.47 (t, *J* = 5.52 Hz, 1H), 3.86 (d, *J* = 5.92 Hz, 2H), 3.18 - 3.14 (m, 8H), 1.60 - 1.52 (m, 8H), 1.32 (m, 8H), 0.93 (t, *J* = 7.32 Hz, 12H).

Compound 22: This compound was synthesised in line with our previously published methods. Proton NMR were found to match our previously published values.[2] ^{1}H NMR (400 MHz, 298.15 K, DMSO-d_6): δ: 9.63 (s, 1H), 8.04 (d, *J* = 9.16 Hz, 2H), 7.58 (d, *J* = 9.16 Hz, 2H), 7.30 (t, *J* = 5.96 Hz, 1H), 3.96 (d, *J* = 5.96 Hz, 2H), 3.18 - 3.14 (m, 8H), 1.60 - 1.52 (m, 8H), 1.35 - 1.25 (m, 8H), 0.92 (t, *J* = 7.36 Hz, 12H).

Compound 23: This compound was synthesised in line with our previously published methods. Proton NMR were found to match our previously published values.[4] ^{1}H NMR (400 MHz, 298.15 K, DMSO-d_6): δ: 9.71 (s, 1H), 7.89 (s, 2H), 7.74 (s, 1H), 7.24 (s, 1H), 4.02 (d, *J* = 5.95 Hz, 2H), 3.18 - 3.14 (m, 8H), 1.60 - 1.52 (m, 8H), 1.30 (m, 8H), 0.92 (t, *J* = 7.79 Hz, 12H).

Compound 24: This compound was synthesised in line with our previously published methods. Proton NMR were found to match our previously published values.[3] ^{1}H NMR (400 MHz, 333.15 K, DMSO-d_6): δ: 9.79 (s, 1H), 7.56 (s, 1H), 7.36 (d, *J* = 7.94 Hz, 2H), 6.87 (d, *J* = 7.89 Hz, 2H), 4.23 (s, 8H), 3.75 (s, 1H), 3.34 - 3.03 (m, 1H), 1.76 - 1.46 (m, 8H), 1.47 - 1.20 (m, 8H), 0.95 (t, *J* = 7.25 Hz, 12H).

Compound 25: This compound was synthesised in line with our previously published methods. Proton NMR were found to match our previously published values.[3] ^{1}H NMR (400 MHz, 333.15 K, DMSO-d_6): δ: 9.93 (s, 1H), 7.77 (s, 1H), 7.54 (d, *J* = 7.84 Hz, 2H), 7.29 (t, *J* = 7.29 Hz, 2H), 7.07 (t, *J* = 7.46 Hz, 1H), 4.24 (s, 2H), 3.30 - 2.95 (m, 8H), 1.60 (m, 8H), 1.48 - 1.14 (m, 8H), 0.95 (t, *J* = 7.27 Hz, 12H).

Compound 26: This compound was synthesised in line with our previously published methods. Proton NMR were found to match our previously published values.[3] ^{1}H NMR (400 MHz, 333.15 K, DMSO-d_6): δ: 10.50 (bs, 1H), 8.33 (bs, 1H), 8.14 (d, *J* = 7.68 Hz, 2H), 7.96 (s, 2H), 4.33 (s, 2H), 3.25 - 3.14 (m, 1H), 1.61 (d, *J* = 6.69 Hz, 8H), 1.34 (m, 8H), 0.94 (t, *J* = 6.54 Hz, 12H).

Compound 27: This compound was synthesised in line with our previously published methods. Proton NMR were found to match our previously published values.[4] ^{1}H NMR (400 MHz, 333.15 K, DMSO-d_6): δ: 10.51 (s, 1H), 8.48 (s, 1H), 8.35 (s, 2H), 7.64 (s, 1H), 4.40 (br s, 2H), 3.20 - 3.16 (m, 8H), 1.63 -1.55 (m, 8H), 1.33 (m, 8H), 0.94 (t, *J* = 7.33 Hz, 12H).

Compound 28: This compound was synthesised in line with our previously published methods. Proton NMR were found to match our previously published values.[3] ^{1}H NMR (400 MHz, 298.15 K, DMSO-d_6): δ: 9.23 (s, 1H), 7.73 - 7.41 (m, 4H), 6.53 (t, *J* = 5.90 Hz, 1H), 3.77 (d, *J* = 5.91 Hz, 2H), 1.42 (s, 9H).

Compound 29: This compound was synthesised in line with our previously published methods. Proton NMR were found to match our previously published values.[3] ^{1}H NMR (400 MHz, 298.15 K, DMSO-d_6): δ: 9.22 (s, 1H), 7.58 (s, 4H), 6.51 (t, *J* = 5.64 Hz, 1H), 3.79 (d, *J* = 5.71 Hz, 2H).

Compound 30: This compound was synthesised in line with our previously published methods. Proton NMR were found to match our previously published values.[3] ^{1}H NMR (400 MHz, 298.15 K, DMSO-d_6): δ: 9.51 (s, 1H), 7.73 (d, *J* = 8.74 Hz, 2H), 7.42 (d, *J* = 8.74 Hz, 2H), 6.49 (s, 1H), 3.38 (d, *J* = 4.00 Hz, 8H), 3.20 - 3.07 (m, 14H), 1.67 - 1.44 (m, 8H), 1.40 - 1.18 (m, 8H), 0.92 (t, *J* = 7.34 Hz, 12H).

Compound 31: This compound was synthesised in line with our previously published methods. Proton NMR were found to match our previously published values.[3] ^{1}H NMR (400 MHz, 298.15 K, DMSO-d_6): δ: 10.27 (bs, 1H), 8.23 (t, *J* = 5.53 Hz, 1H), 7.71 (dd, *J* = 26.91 Hz, 8.68 Hz, 4H), 4.18 (d, *J* = 5.50 Hz, 2H), 1.43 (s, 9H).

Compound 32: This compound was synthesised in line with our previously published methods. Proton NMR were found to match our previously published values.[3] ^{1}H NMR (400 MHz, 298.15 K, DMSO-d_6): δ: 12.78 (s, 1H), 10.26 (s, 1H), 8.20 (t, *J* = 5.38 Hz, 1H), 7.73 (dd, *J* = 38.27 Hz, 8.60 Hz, 4H), 4.23 (d, *J* = 5.32 Hz, 2H).

Compound 33: This compound was synthesised in line with our previously published methods. Proton NMR were found to match our previously published values.[3] ^{1}H NMR (400 MHz, 298.15 K, DMSO-d_6): δ: 12.30 (s, 1H), 8.77 (s, 1H), 8.38 (d, *J* = 8.09 Hz, 2H), 7.55 (d, *J* = 8.28 Hz, 2H), 3.73 (s, 2H), 3.22 - 3.01 (m, 8H), 1.66 - 1.43 (m, 8H), 1.43 - 1.15 (m, 8H), 0.92 (t, *J* = 7.33 Hz, 12H).

Compound 34: This compound was synthesised in line with our previously published methods. Proton NMR were found to match our previously published values.[5] ^{1}H NMR (400 MHz, 298.15 K, DMSO-d_6): δ: 8.80 (s, 1H), 8.17 (d, *J* = 7.99 Hz, 1H), 8.09 (d, *J* = 5.44 Hz, 1H), 7.88 (d, *J* = 8.18 Hz, 1H), 7.60 – 7.46 (m, 3H), 7.41 (t, *J* = 7.89 Hz, 1H), 7.11 (t, *J* = 5.68 Hz, 1H), 3.94 (d, *J* = 5.72 Hz, 2H), 3.21 – 3.05 (m, 8H), 1.64 – 1.40 (m, 8H), 1.41 – 1.21 (m, 8H), 0.92 (t, *J* = 7.31 Hz, 12H).

Compound 35: This compound was synthesised in line with our previously published methods. Proton NMR were found to match our previously published values.[5] ^{1}H NMR (400 MHz, 298.15 K, DMSO-d_6): δ: 9.11 (br s, 1H), 8.41 (s, 1H), 8.30 (s, 1H), 8.21 (s, 1H), 7.97 (dd, *J* = 12.97, 8.00 Hz, 3H), 7.50 – 7.29 (m, 3H),

6.80 (t, *J* = 5.67 Hz, 1H), 3.98 (t, *J* = 8.12 Hz, 2H), 3.23 – 3.00 (m, 8H), 1.64 – 1.43 (m, 8H), 1.38 – 1.20 (m, 8H), 0.91 (t, *J* = 7.32 Hz, 12H).

Compound 36: This compound was synthesised in line with our previously published methods. Proton NMR were found to match our previously published values.[6] ^{1}H NMR (400 MHz, 298.15 K, DMSO-d_6): δ: 9.09 (s, 1H), 8.44 (s, 1H), 8.34 (s, 1H), 8.22 (s, 1H), 8.01 – 7.97 (m, 3H), 7.47 – 7.39 (m, 3H), 6.53 (t, *J* = 5.88 Hz, 1H), 3.83 (d, *J* = 5.88 Hz, 2H), 1.44 (s, 9H).

Compound 37: This compound was synthesised in line with our previously published methods. Proton NMR were found to match our previously published values.[6] ^{1}H NMR (400 MHz, 298.15 K, DMSO-d_6): δ: 9.68 (s, 1H), 8.25 (d, *J* = 2.12 Hz, 1H), 7.97 – 7.86 (m, 3H), 7.76 – 7.73 (m, 2H), 7.59 (d, *J* = 8.48 Hz, 1H), 7.51 (t, 1H), 4.03 (d, *J* = 5.68 Hz, 2H), 3.17 – 3.13 (m, 8H), 1.59 – 1.51 (m, 8H), 1.34 – 1.25 (m, 8H), 0.92 (t, *J* = 7.28 Hz, 12H).

Compound 38: This compound was synthesised in line with our previously published methods. Proton NMR were found to match our previously published values.[5] ^{1}H NMR (400 MHz, 298.15 K, DMSO-d_6): δ: 10.67 (s, 1H) 8.45 (d, *J* = 7.77 Hz, 1H), 8.30 (d, *J* = 7.79 Hz, 1H), 8.15 (d, *J* = 7.92 Hz, 1H), 7.85 (d, *J* = 7.72 Hz, 2H), 7.58 (t, *J* = 7.58 Hz, 1H), 7.47 (tt, *J* = 11.69, 7.49 Hz, 2H), 7.10 (t, *J* = 7.52 Hz, 1H), 3.96 (s, 2H), 3.23 – 2.99 (m, 8H), 1.62 – 1.44 (m, 8H), 1.41 – 1.19 (m, 8H), 0.92 (t, *J* = 7.32 Hz, 12H).

Compound 39: This compound was synthesised in line with our previously published methods. Proton NMR were found to match our previously published values.[5] ^{1}H NMR (400 MHz, 298.15 K, DMSO-d_6): δ: 9.25 (s, 1H), 7.95 – 7.75 (m, 4H), 7.58 (d, *J* = 8.74 Hz, 2H), 7.37 – 7.20 (m, 1H), 6.98 (t, *J* = 5.88 Hz, 1H), 3.96 (d, *J* = 5.88 Hz, 2H), 3.20 – 3.01 (m, 9H), 2.43 (s, 3H), 1.63 – 1.43 (m, 9H), 1.40 – 1.17 (m, 9H), 0.91 (t, *J* = 7.32 Hz, 12H).

Compound 40: Aminomethane sulfonic acid (0.16 g, 1.50 mM) was dissolved in tetrabutylammonium in methanol (1.50 mL, 1.50 mM) with excess methanol to aid dissolving before being taken to complete dryness. Tetrabutylammonium aminomethane sulfonate (1.50 mM) was dissolved in chloroform (12.5 mL) and left overnight in a sealed vial with 1-isocyanatobutane (0.15 g, 1.50 mM). The resulting solution was taken to dryness, re-dissolved in ethyl acetate (15 mL) and extracted with water (15 mL). The pure product was obtained by flash chromatography 100 % ethyl acetate followed by 100 % methanol. The methanol fraction was taken to dryness to give the pure product as an opaque viscous oil with a yield of 63 % (0.28 g, 0.62 mM); Melting Point: 131 °C; ^{1}H NMR (400 MHz, 298.15 K, DMSO-d_6): δ: 6.16 (s, 1H), 6.05 (t, *J* = 5.52 Hz, 1H), 3.75 (d, *J* = 5.96 Hz, 2H), 3.16 (t, *J* = 8.24 Hz, 8H), 2.96 (q, *J* = 5.96 Hz, 2H), 1.56 (m, 8H), 1.30 (m, 12H), 0.93 (t, *J* = 6.88 Hz, 12H), 0.86 (t, *J* = 6.84 Hz, 3H); ^{13}C{^{1}H} NMR (100 MHz, 298.15 K, DMSO-d_6): δ: 157.9 (CO), 58.0 (CH_2), 57.2 (CH_2), 49.1 (CH_2), 32.6 (CH_2), 23.6(CH_2), 20.1 (CH_2), 19.7 (CH_2), 14.2 (CH_3), 14.0 (CH_3); IR (film): ν (cm^{-1}) = 3317 (NH stretch), 1658, 1170, 1035, 883; HRMS for the sulfonate-urea ion ($C_6H_{13}N_2O_4S^-$) (ESI$^-$): m/z: act: 209.0602 $[M]^-$ cal: 209.0590 $[M]^-$.

Compound 41: Tetrabutylammonium in methanol (1.50 mL, 1.50 mM) was added to 2-aminoethane-1-sulfonic acid (0.19 g, 1.50 mM), excess methanol was added to aid solubility before being taken to complete dryness. Tetrabutylammonium 2-aminoethane-1-sulfonate (1.50 mM) was dissolved in pyridine (2.0 mL) and left overnight in a sealed vial with 1-isocyanatobutane (0.15 g, 1.50 mM). The resulting solution was taken to dryness, re-dissolved in ethyl acetate (15 mL) and extracted with water (15 mL). The pure product was obtained by flash chromatography 100 % ethyl acetate followed by 100 % methanol. The methanol fraction was taken to dryness to give the pure product as an opaque viscous oil with a yield of 73 % (0.31 g, 0.67 mM); Melting Point: 135 °C; ^{1}H NMR (400 MHz, 298.15 K, DMSO-d_6): δ: 6.20 (t, *J* =

5.52 Hz, 1H), 5.87 (t, *J* = 5.04 Hz, 1H), 3.23 (q, *J* = 5.96 Hz, 2H), 3.16 (t, *J* = 8.68 Hz, 8H), 2.92 (q, *J* = 5.92 Hz, 2H), 2.48 (t, *J* = 6.40 Hz, 2H) , 1.55 (m, 8H), 1.29 (m, 12H), 0.93 (t, *J* = 7.80 Hz, 12H), 0.85 (t, *J* = 7.32 Hz, 3H); $^{13}C\{^1H\}$ NMR (100 MHz, 298.15 K, DMSO-d_6): δ: 158.4 (CO), 58.0 (CH_2), 51.9 (CH_2), 39.5 (CH_2), 36.7 (CH_2), 32.6 (CH_2), 23.6 (CH_2), 20.1 (CH_2), 19.7 (CH_2), 14.3 (CH_3), 14.0 (CH_3); IR (film): ν (cm^{-1}) = 3335 (NH stretch), 1664, 1209, 1031, 883; HRMS for the sulfonate-urea ion ($C_7H_{15}N_2O_3S^-$) (ESI^-): m/z: act: 223.0758 $[M]^-$ cal: 223.0740 $[M]^-$.

Compound 42: Tetrabutylammonium in methanol (1.50 mL, 1.50 mM) was added to 3-aminopropane-1-sulfonic acid (0.21 g, 1.50 mM), excess methanol was added to aid solubility before being taken to complete dryness. Tetrabutylammonium 3-aminopropane-1-sulfonate (1.50 mM) was dissolved in pyridine (2.0 mL) and left overnight in a sealed vial with 1-isocyanatobutane (0.15 g, 1.50 mM). The resulting solution was taken to dryness, re-dissolved in ethyl acetate (15 mL) and extracted with water (15 mL). The pure product was obtained by flash chromatography 100 % ethyl acetate followed by 100 % methanol. The methanol fraction was taken to dryness to give the pure product as a cream oil with a yield of 56 % (0.27 g, 0.56 mM); Melting Point: 145 °C; ^1H NMR (400 MHz, 298.15 K, DMSO-d_6): δ: 5.79 (s, 1H), 5.78 (s, 1H), 3.17 (t, *J* = 8.16 Hz, 8H), 2.97 (m, 4H), 2.37 (t, *J* = 8.0 Hz, 2H), 1.59 (m, 10H), 1.27 (m, 12H), 0.93 (t, *J* = 8.05, 12H), 0.86 (t, *J* = 7.04 Hz, 3H); $^{13}C\{^1H\}$ NMR (100 MHz, 298.15 K, DMSO-d_6): δ: 158.6 (CO), 58.0 (CH_2), 49.6 (CH_2), 39.4 (CH_2), 39.0 (CH_2), 32.7 (CH_2), 26.9 (CH_2), 23.6 (CH_2), 20.1 (CH_2), 19.7 (CH_2) 14.3 (CH_3), 14.2 (CH_3): IR (film): ν (cm^{-1}) = 3346 (NH stretch), 1664, 1180, 1033, 883; HRMS for the sulfonate-urea ion ($C_8H_{17}N_2O_4S^-$) (ESI^-): m/z: act: 237.0920 $[M]^-$ cal: 237.0890 $[M]^-$.

Compound 43: Aminomethane sulfonic acid (0.16 g, 1.50 mM) was dissolved in tetrabutylammonium in methanol (1.50 mL) with excess methanol to aid dissolving before being taken to complete dryness. Tetrabutylammonium aminomethane sulfonate (1.50 mM) was dissolved in chloroform (12.5 mL) and left overnight in a sealed vial with 1-isothiocyanatobutane (0.18 mL, 1.50 mM). The resulting solution was taken to dryness, re-dissolved in ethyl acetate (15 mL) and extracted with water (15 mL). The pure product was obtained by flash chromatography 100 % ethyl acetate followed by 100 % methanol. The methanol fraction was taken to dryness to give the pure product as an opaque viscous oil with a yield of 32 % (0.21 g, 0.45 mM); Melting Point: 129 °C; ^1H NMR (400 MHz, 333.15 K, DMSO-d_6): δ: 7.98 (s, 1H), 7.33 (s, 1H), 4.10 (d, *J* = 4.48 Hz, 2H), 3.40 (q, *J* = 6.28 Hz, 2H), 3.19 (t, *J* = 8.36 Hz, 8H), 1.61 (m, 8H), 1.47 (m, 2H), 1.33 (m, 10H), 0.93 (t, *J* = 7.28 Hz, 12H) , 0.88 (t, *J* = 7.24, 3H); $^{13}C\{^1H\}$ NMR (100 MHz, 298.15 K, DMSO-d_6): δ: 183.0 (CS), 61.1 (CH_2), 58.0 (CH_2), 44.0 (CH_2), 31.3 (CH_2), 23.6 (CH_2), 20.1 (CH_2), 19.7 (CH_2), 14.2 (CH_3), 14.0 (CH_3); IR (film): ν (cm^{-1}) = 3263 (NH stretch), 1556, 1211, 1166, 881; HRMS for the sulfonate-urea ion ($C_6H_{13}N_2O_4S_2$) (ESI^-): m/z: act: 225.0373 $[M]^-$ cal: 225.0360 $[M]^-$.

Compound 44: Tetrabutylammonium in methanol (1.50 mL, 1.50 mM) was added to 2-aminoethane-1-sulfonic acid (0.19 g, 1.50 mM), excess methanol was added to aid solubility before being taken to complete dryness. Tetrabutylammonium 2-aminoethane-1-sulfonate (1.50 mM) was dissolved in pyridine (2.0 mL) and left overnight in a sealed vial with 1-isothiocyanatobutane (0.18 mL, 1.50 mM). The resulting solution was taken to dryness, re-dissolved in ethyl acetate (15 mL) and extracted with water (15 mL). The pure product was obtained by flash chromatography 100 % ethyl acetate followed by 100 % methanol. The methanol fraction was taken to dryness to give the pure product as an opaque viscous oil with a yield of 64 % (0.31 g, 0.64 mM); Melting Point: 132 °C; ^1H NMR (400 MHz, 298.15 K, DMSO-d_6): δ: 7.50 (s, 1H), 7.41 (s, 1H), 3.64 (q, *J* = 5.52 Hz, 2H), 3.31 (q, *J* = 5.96 Hz, 2H), 3.19 (t, *J* = 8.24 Hz, 8H), 2.62 (t, *J* = 5.92 Hz, 2H), 1.61 (m, 8H), 1.33 (m, 12H), 0.96 (t, *J* = 7.32 Hz, 12H), 0.89 (t, *J* = 7.32, 3H); $^{13}C\{^1H\}$ NMR (100 MHz, 298.15 K, DMSO-d_6): δ: 182.5 (CS), 58.0 (CH_2), 50.5 (CH_2), 43.8 (CH_2), 41.1 (CH_2), 31.3 (CH_2), 23.5 (CH_2), 20.1 (CH_2), 19.7 (CH_2), 14.2 (CH_3), 14.0 (CH_3); IR (film): ν (cm^{-1}) = 3248 (NH stretch), 1556, 1209, 1028, 873; HRMS for the sulfonate-urea ion ($C_7H_{15}N_2O_3S_2^-$) (ESI^-): m/z: act: 239.0530 $[M]^-$ cal: 239.0510 $[M]^-$.

Compound 45: Tetrabutylammonium in methanol (1.50 mL, 1.50 mM) was added to 3-Aminopropane-1-sulfonic acid (0.21 g, 1.50 mM), excess methanol was added to aid solubility before being taken to complete dryness. Tetrabutylammonium 3-aminopropane-1-sulfonate (1.50 mM) was dissolved in pyridine (2.0 mL) and left overnight in a sealed vial with 1-isothiocyanatobutane (0.18 mL, 1.50 mM). The resulting solution was taken to dryness, re-dissolved in ethyl acetate (15 mL) and extracted with water (15 mL). The pure product was obtained by flash chromatography 100 % ethyl acetate followed by 100 % methanol. The methanol fraction was taken to dryness to give the pure product as a cream solid with a yield of 58 % (0.29 g, 0.59 mM); Melting Point: 137 °C; ^{1}H NMR (400 MHz, 298.15 K, DMSO-d_6): δ: 7.35 (s, 1H), 7.31 (s, 1H), 3.44 (q, *J* = 5.96 Hz, 2H), 3.36 (q, *J* = 6.40 Hz, 2H), 3.19 (t, *J* = 8.72 Hz, 8H), 2.46 (t, *J* = 6.88 Hz, 2H), 1.79 (m, 2H), 1.61 (m, 8H), 1.49 (m, 2H), 1.39 (m, 10H), 0.95 (t, *J* = 7.76, 12H), 0.89 (t, *J* = 7.80 Hz, 3H); $^{13}C\{^{1}H\}$ NMR (100 MHz, 298.15 K, DMSO-d_6): δ: 182.85 (CO), 58.0 (CH_2), 49.5 (CH_2), 43.7 (CH_2), 43.3 (CH_2), 31.4 (CH_2), 25.7 (CH_2), 23.6 (CH_2), 20.1 (CH_2), 19.7 (CH_2) 14.2 (CH_3), 14.0 (CH_3); IR (film): ν (cm^{-1}) = 3290 (NH stretch), 1685, 1207, 1035,881; HRMS for the sulfonate-urea ion ($C_8H_{17}N_2O_3S_2^-$) (ESI^-): m/z: act: 253.0690 $[M]^-$ cal: 253.0670 $[M]^-$.

Compound 46: This compound was synthesised in line with our previously published methods. Proton NMR were found to match our previously published values.[3]

NMR

Characterisation NMR

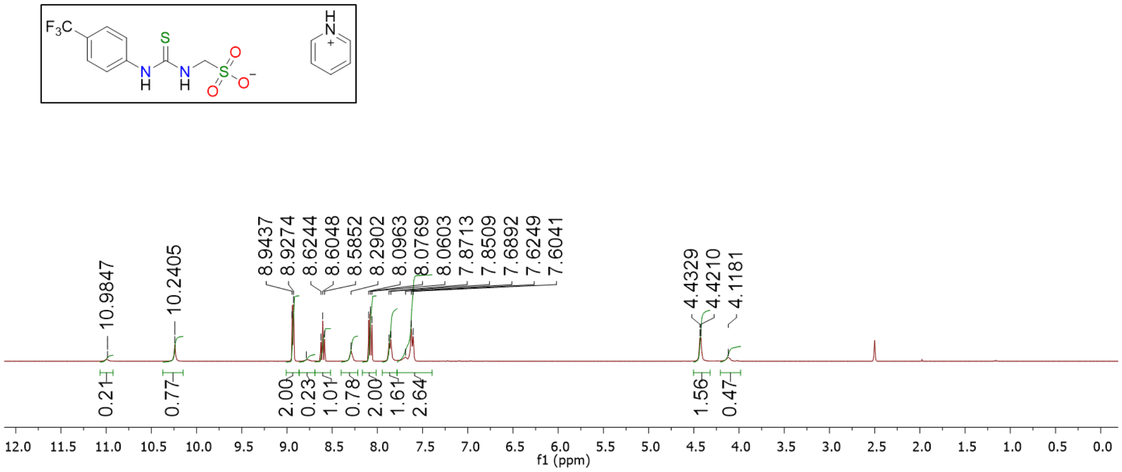

Figure S2 - ^{1}H NMR of compound **15** in DMSO-d_6 conducted at 298.15 K.

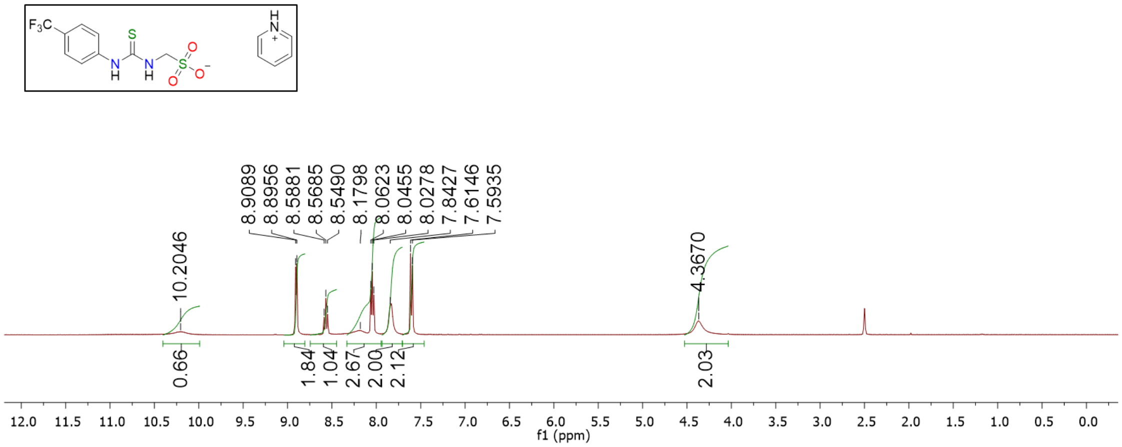

Figure S3 - ^{1}H NMR of compound **15** in DMSO-d_6 conducted at 333.15 K.

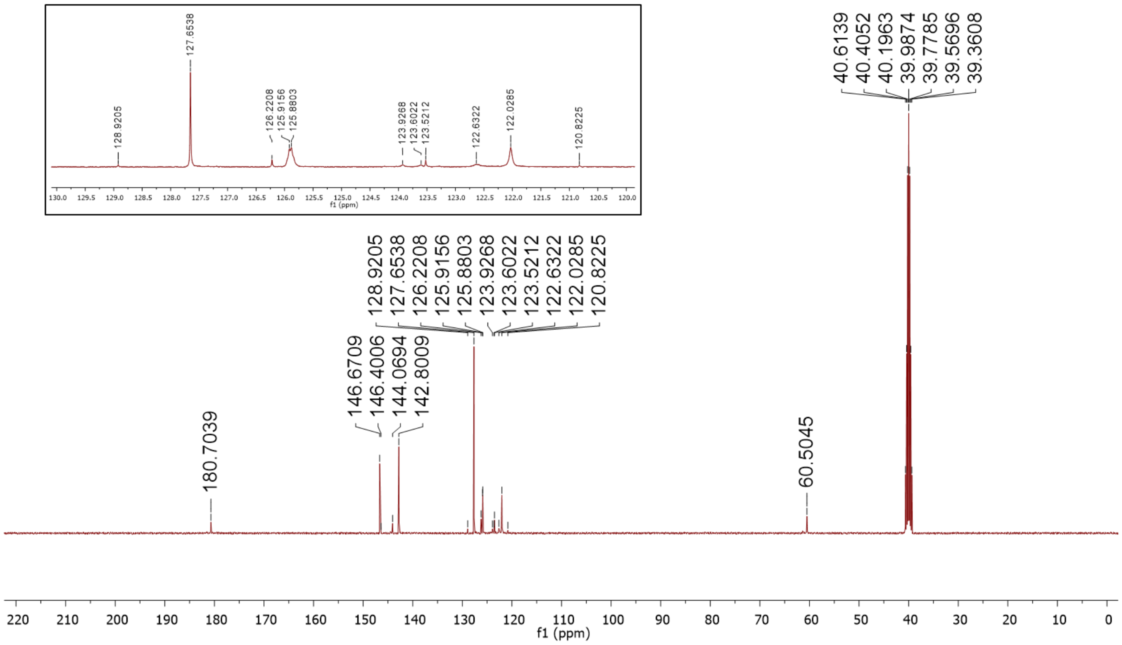

Figure S4 - $^{13}C\{^{1}H\}$ NMR of compound **15** in DMSO-d_6 conducted at 298.15 K.

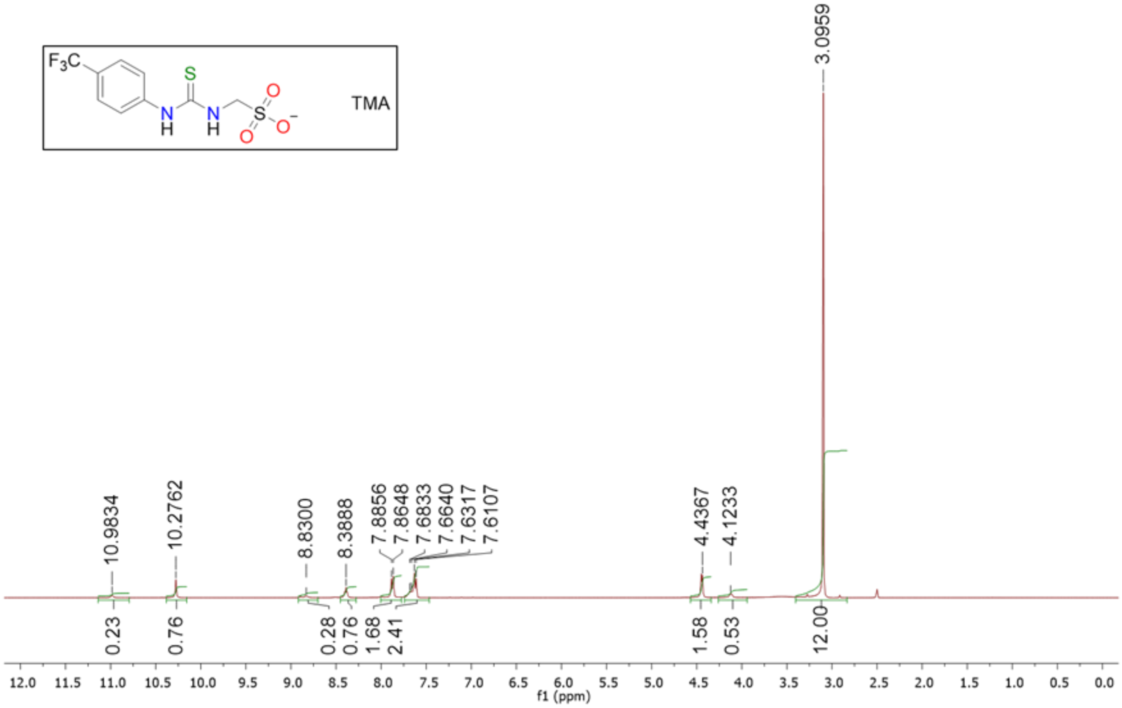

Figure S5 - ^{1}H NMR of compound **16** in DMSO-d_6 conducted at 298.15 K.

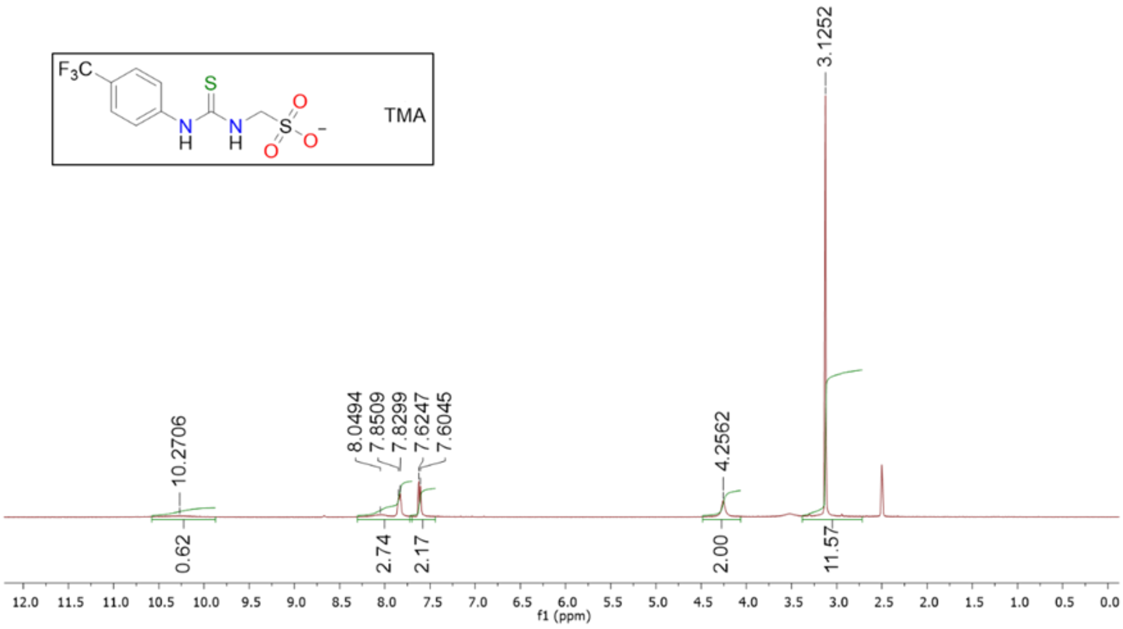

Figure S6 - ^{1}H NMR of compound **16** in DMSO-d_6 conducted at 333.15 K.

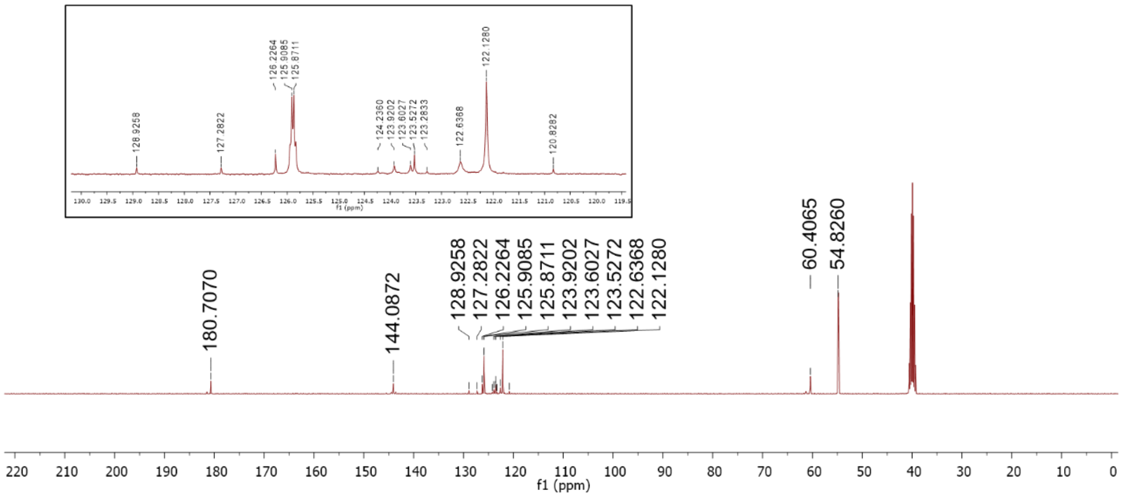

Figure S7 - ^{13}C{^{1}H} NMR of compound **16** in DMSO-d_6 conducted at 298.15 K.

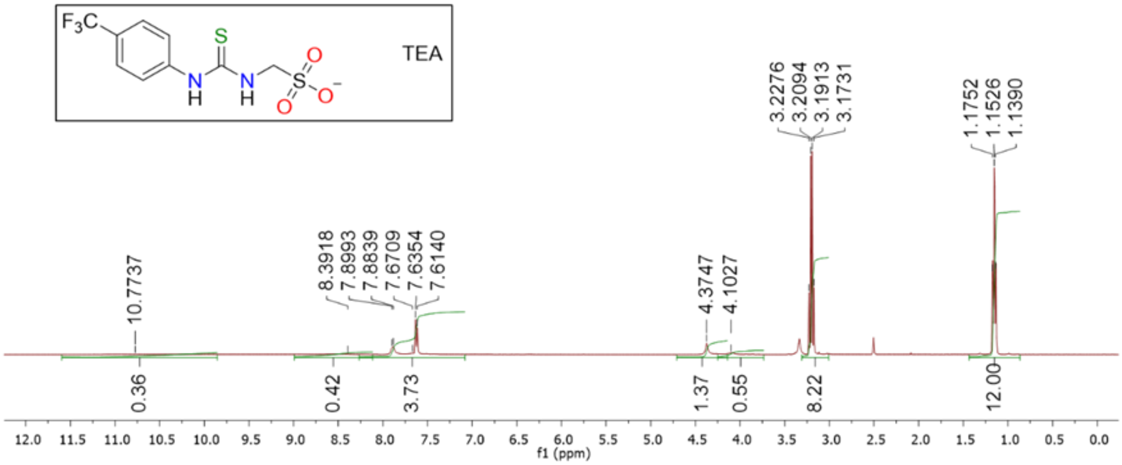

Figure S8 - ^{1}H NMR of compound **17** in DMSO-d_6 conducted at 298.15 K.

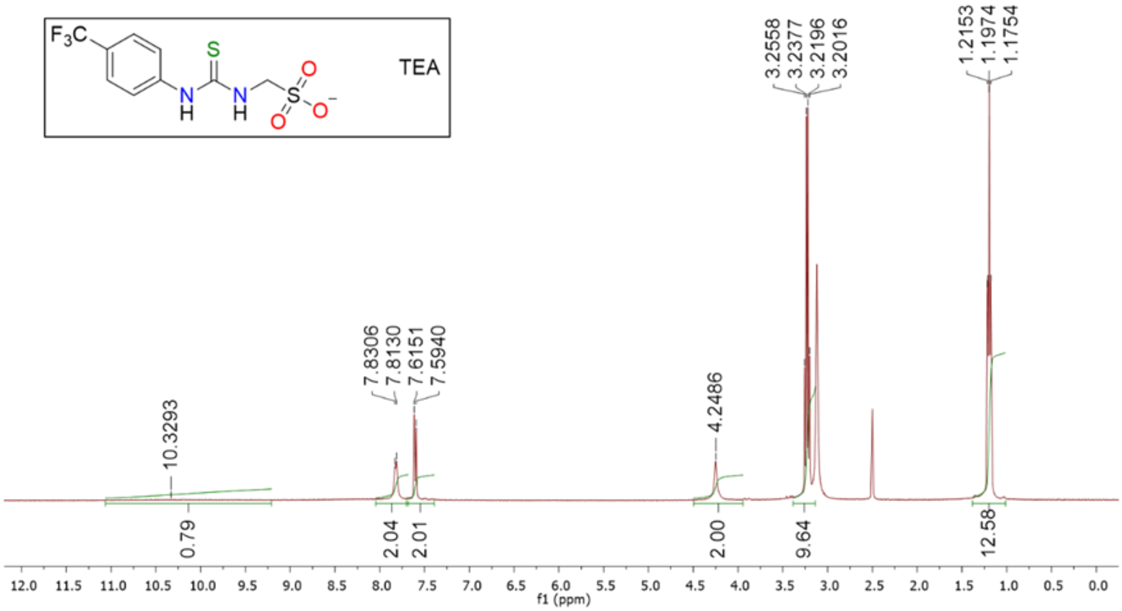

Figure S9 - ^{1}H NMR of compound **17** in DMSO-d_6 conducted at 333.15 K.

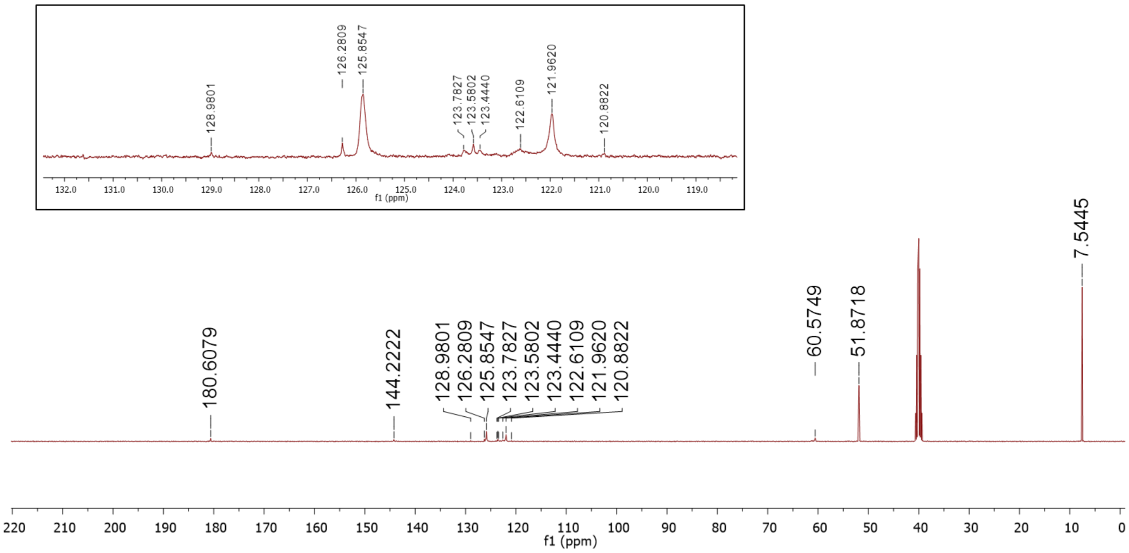

Figure S10 - $^{13}C\{^{1}H\}$ NMR of compound **17** in DMSO-d_6 conducted at 298.15 K.

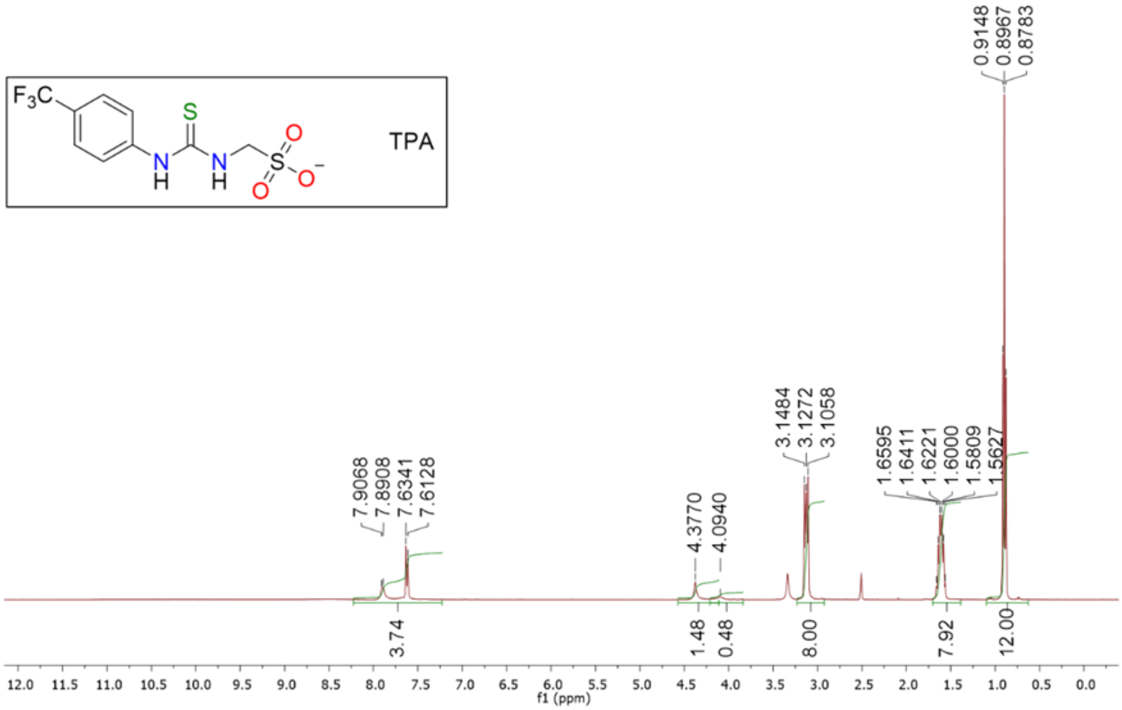

Figure S11 - ^{1}H NMR of compound **18** in DMSO-d_6 conducted at 298.15 K.

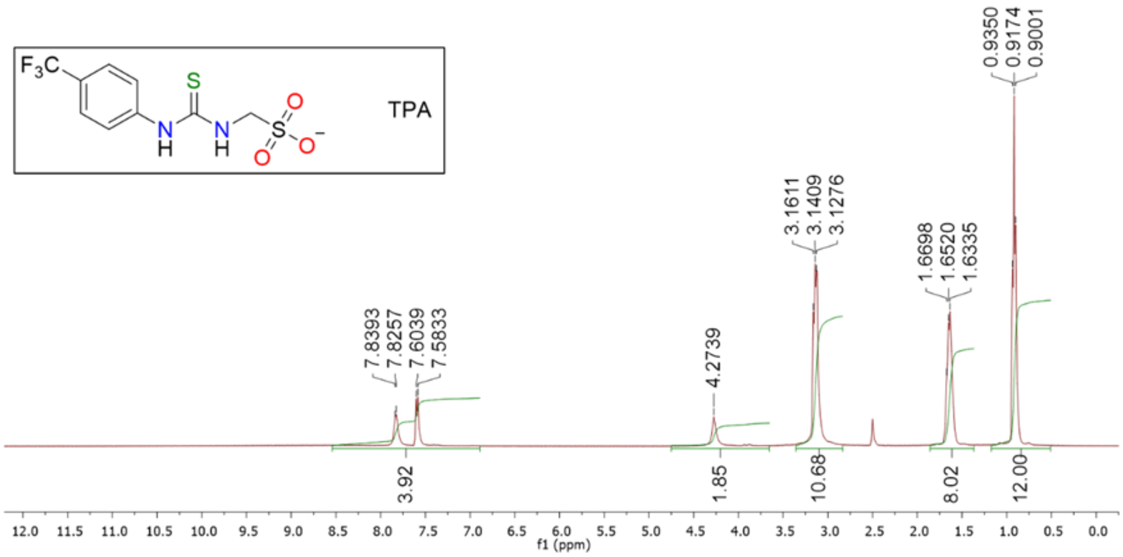

Figure S12 - ^{1}H NMR of compound **18** in DMSO-d_6 conducted at 333.15 K.

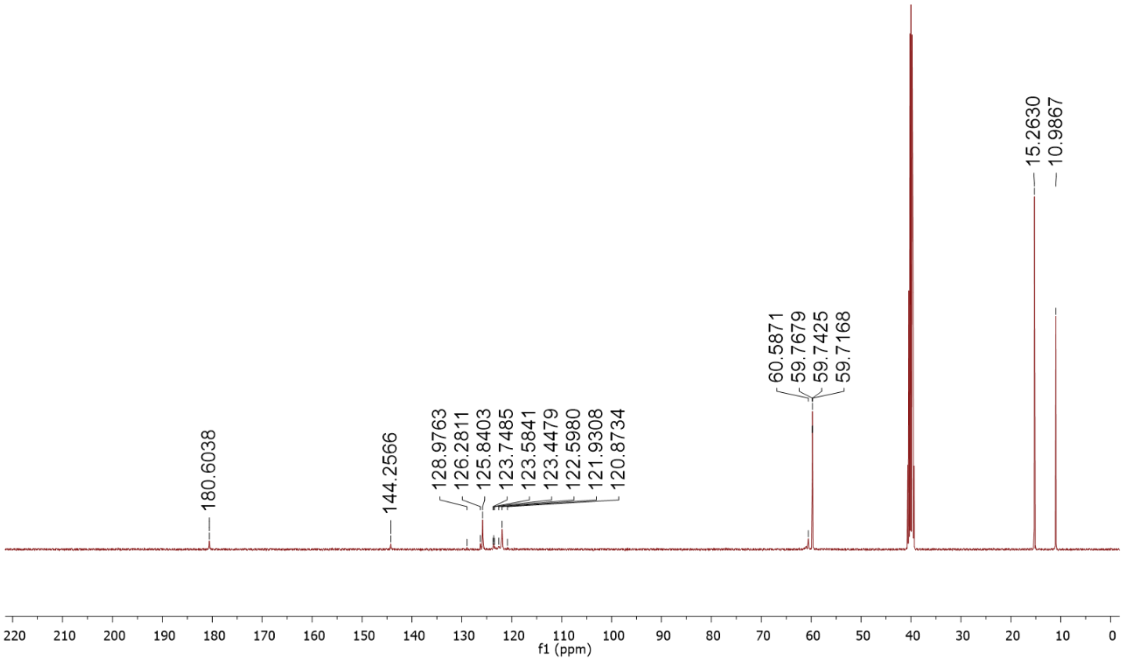

Figure S13 - $^{13}C\{^{1}H\}$ NMR of compound **18** in DMSO-d_6 conducted at 298.15 K.

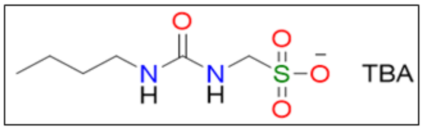

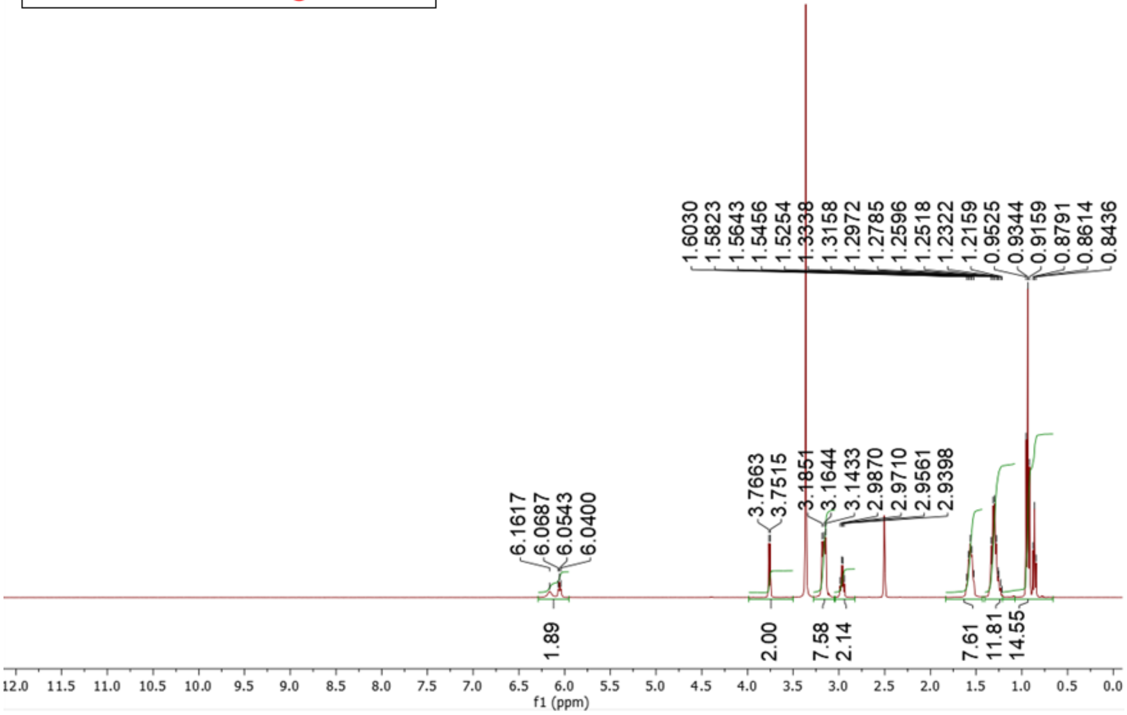

Figure S14 - ^{1}H NMR of compound **40** in DMSO-d_6 conducted at 298.15 K.

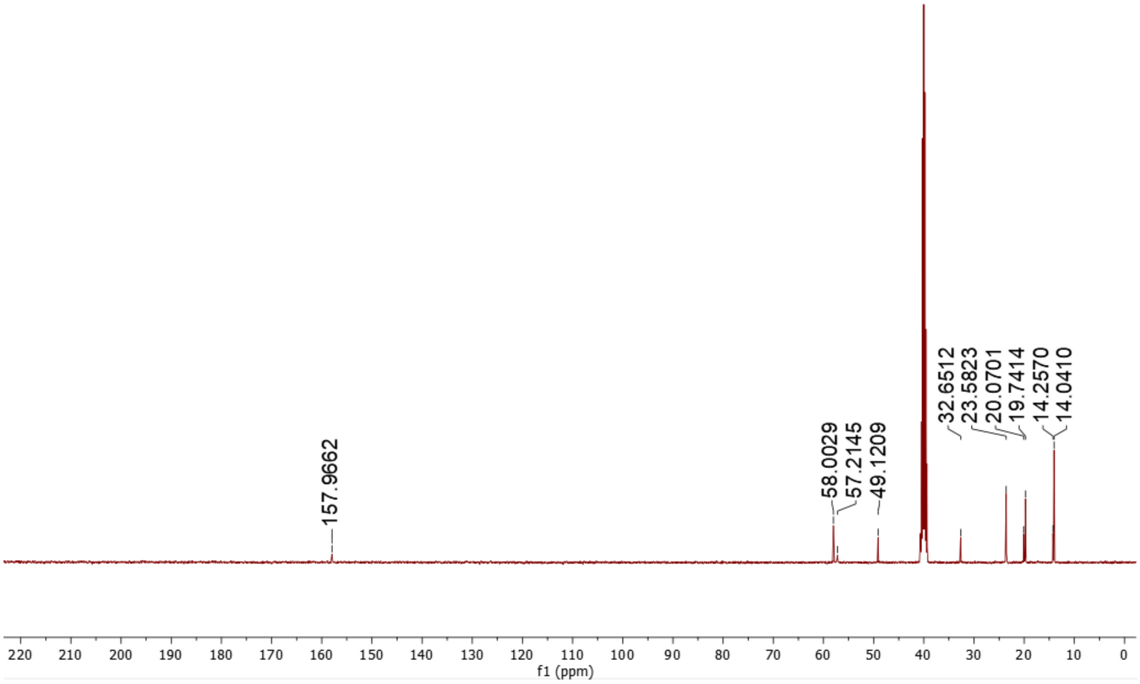

Figure S15 - $^{13}C\{^{1}H\}$ NMR of compound **40** in DMSO-d_6 conducted at 298.15 K.

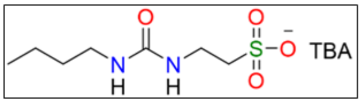

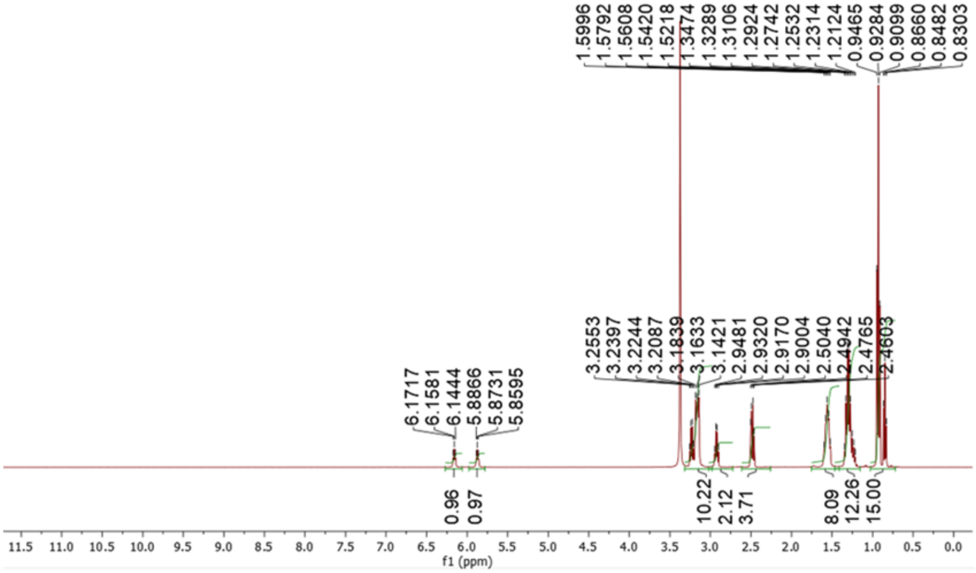

Figure S16 - ^{1}H NMR of compound **41** in DMSO-d_6 conducted at 298.15 K.

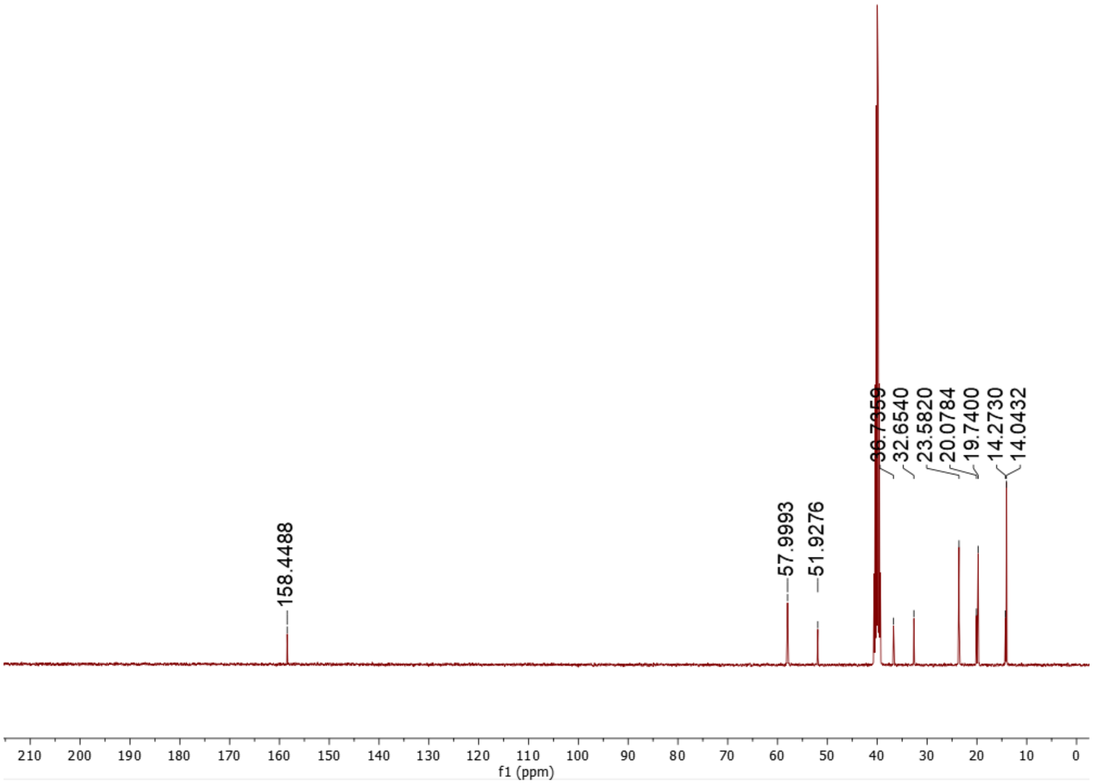

Figure S17 - ^{13}C{^{1}H} NMR of compound **41** in DMSO-d_6 conducted at 298.15 K.

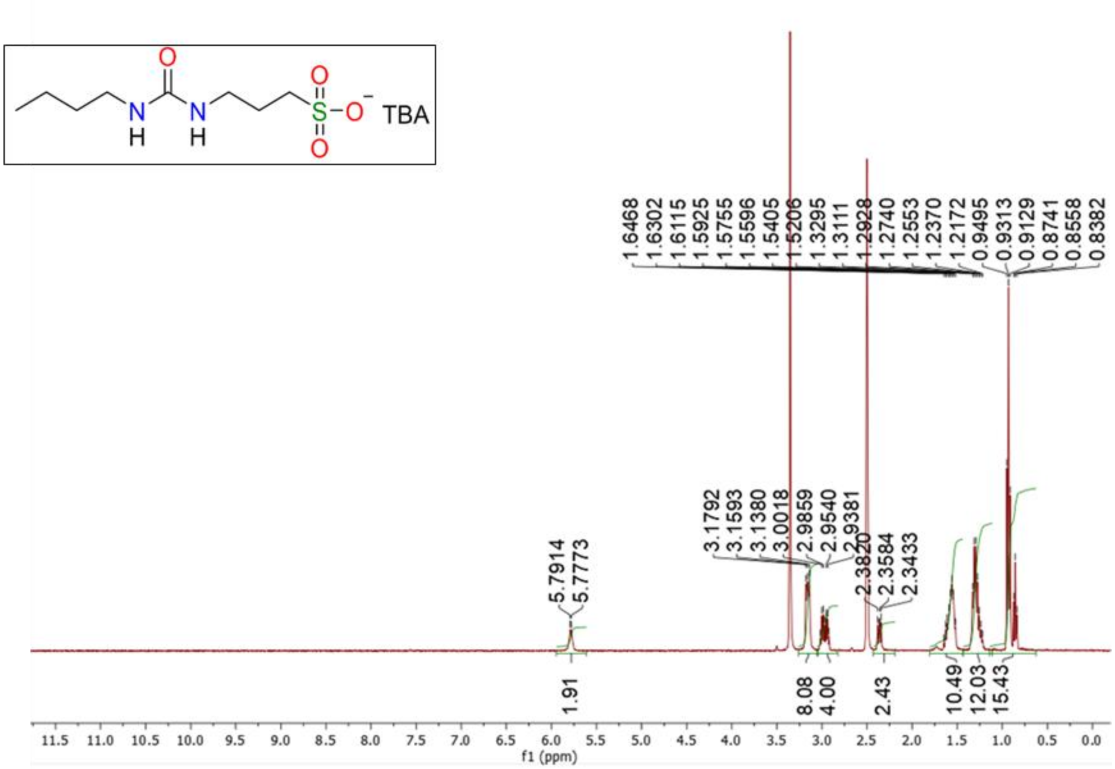

Figure S18 - ^{1}H NMR of compound **42** in DMSO-d_6 conducted at 298.15 K.

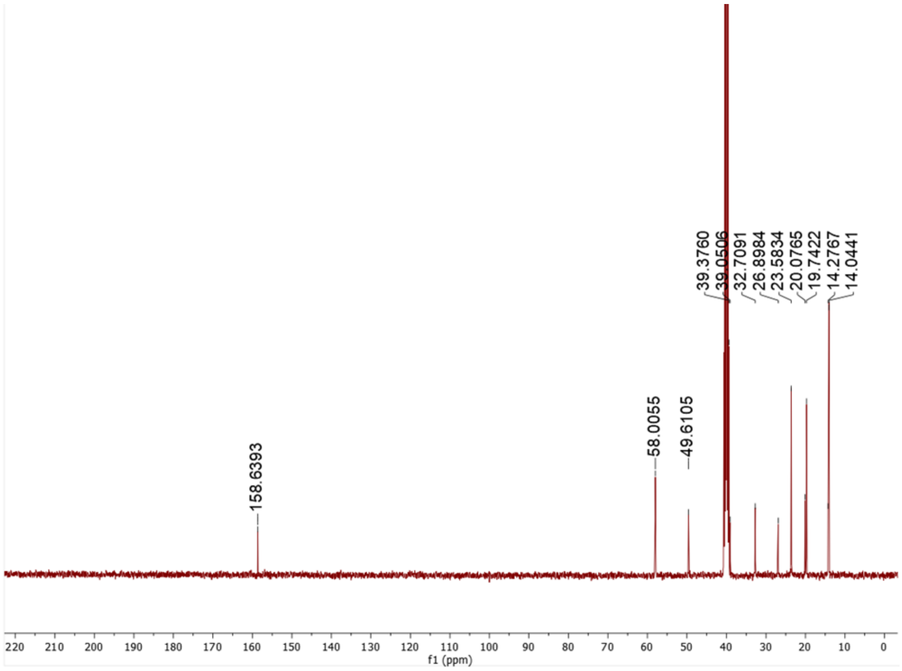

Figure S19 - $^{13}C\{^{1}H\}$ NMR of compound **42** in DMSO-d_6 conducted at 298.15 K.

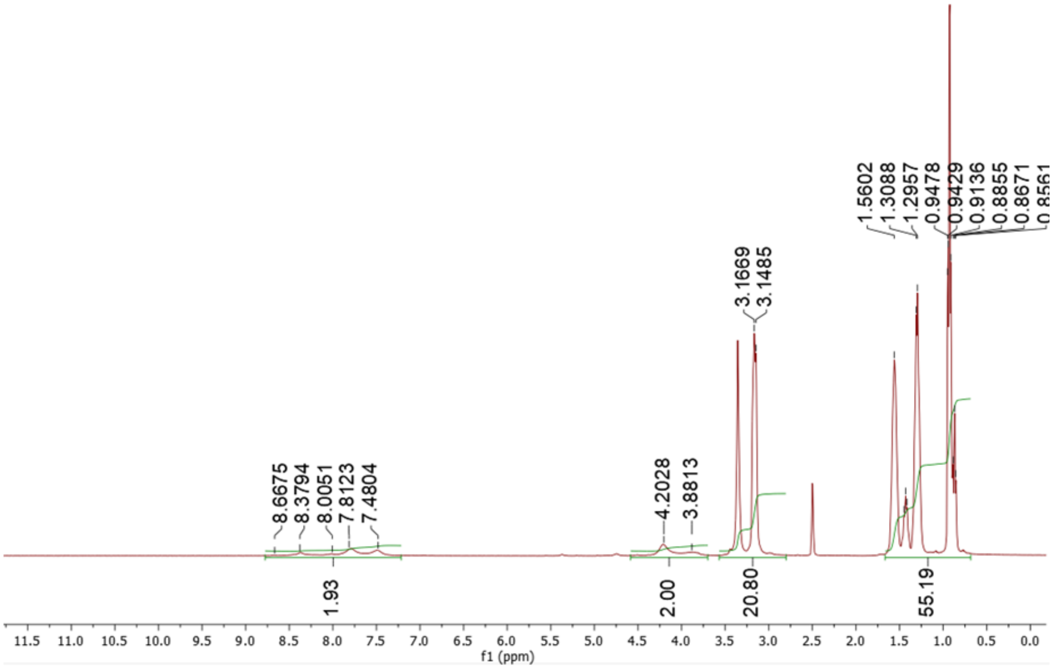

Figure S20 - ^{1}H NMR of compound **43** in DMSO-d_6 conducted at 298.15 K.

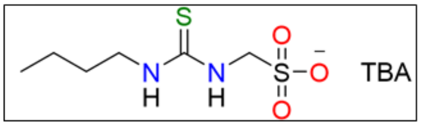

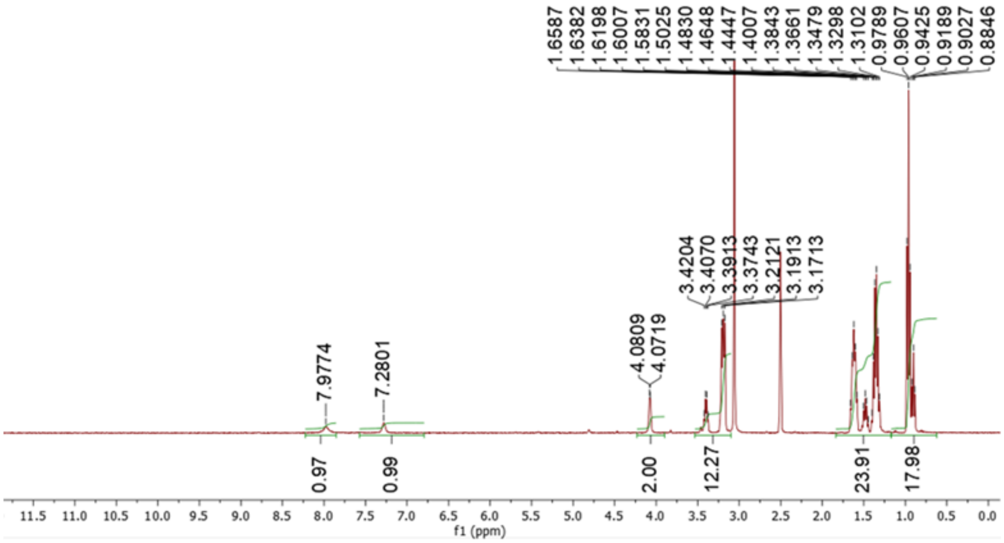

Figure S21 - ^{1}H NMR of compound **43** in DMSO-d_6 conducted at 333.15 K.

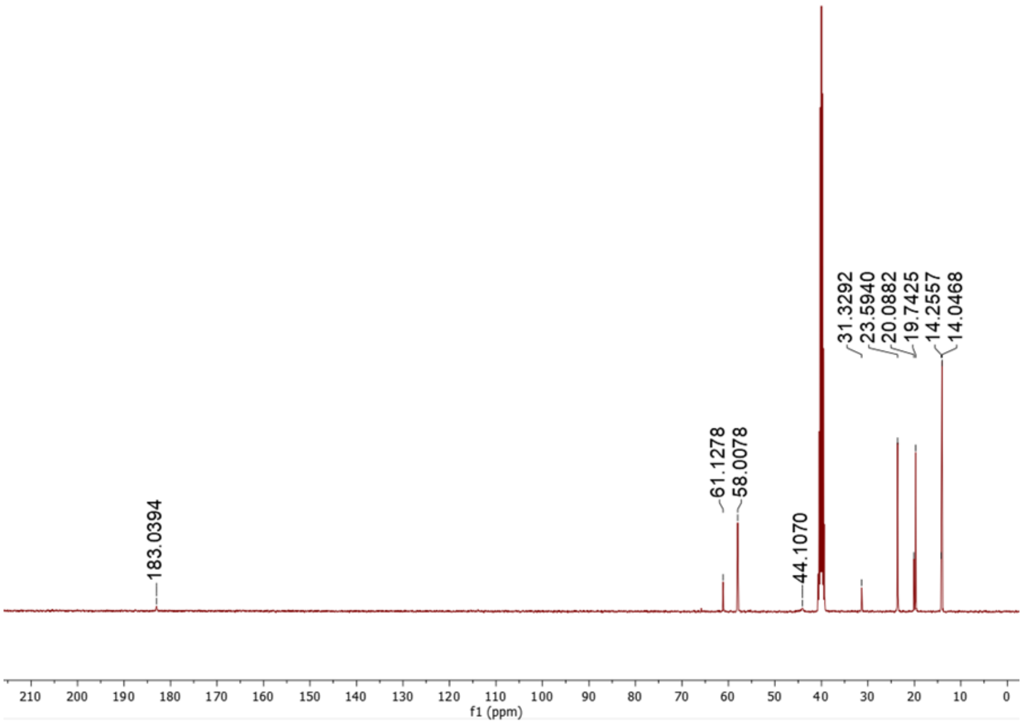

Figure S22 - $^{13}C\{^{1}H\}$ NMR of compound **43** in DMSO-d_6 conducted at 298.15 K.

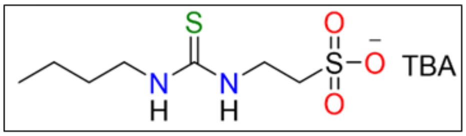

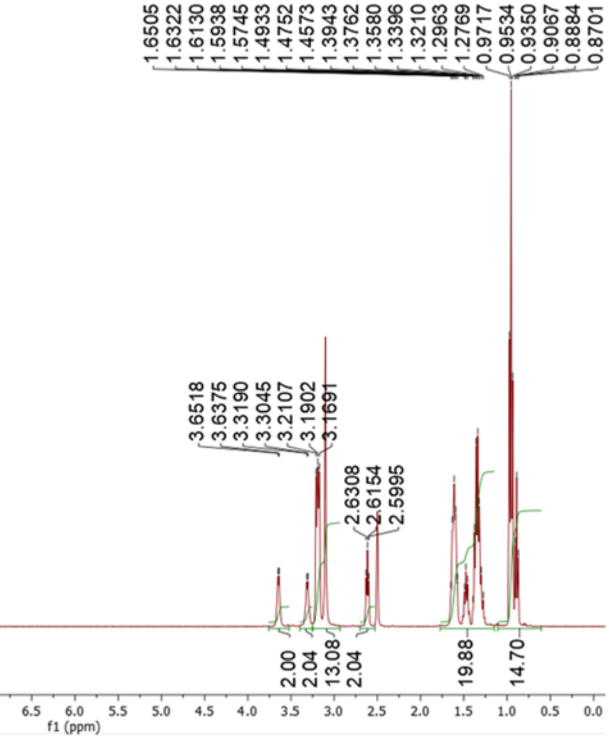

Figure S23 - ^{1}H NMR of compound **44** in DMSO-d_6 conducted at 298.15 K.

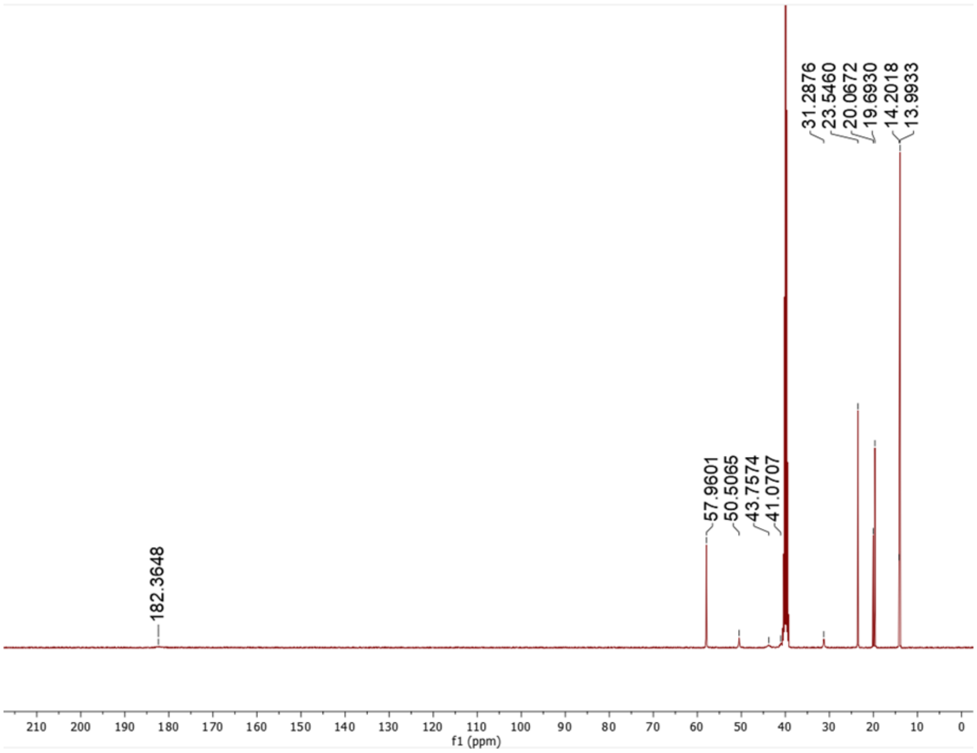

Figure S24 - $^{13}C\{^{1}H\}$ NMR of compound **44** in DMSO-d_6 conducted at 298.15 K.

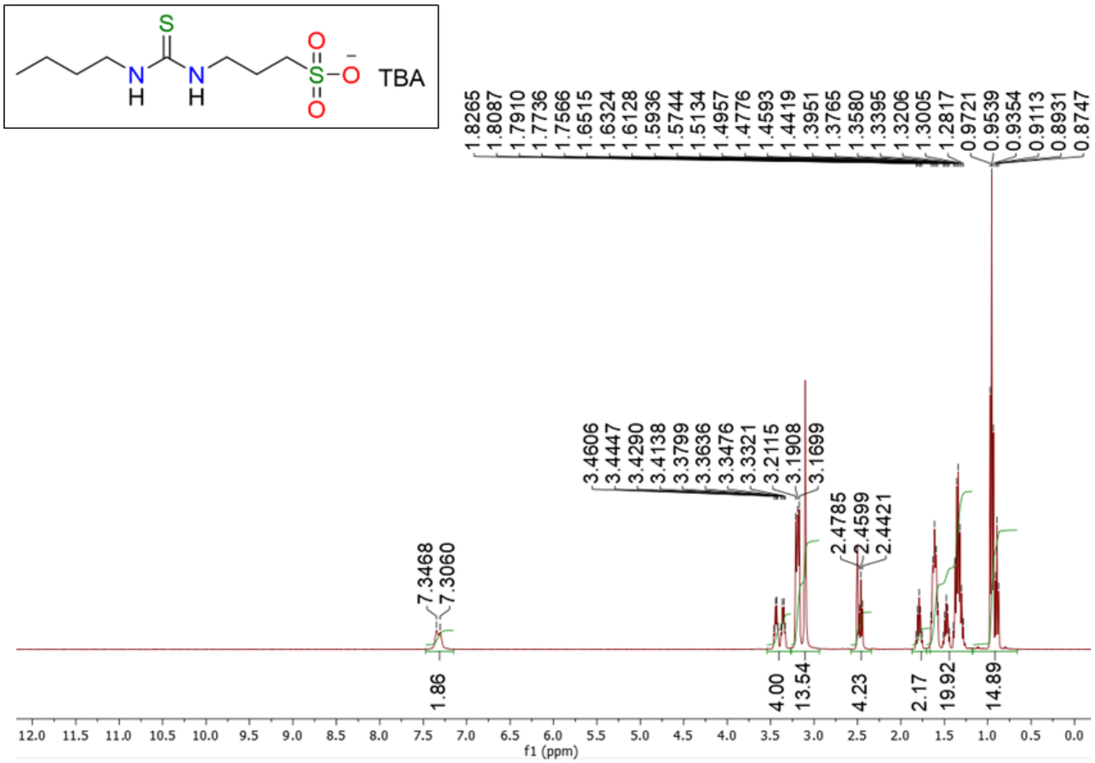

Figure S25 - ^{1}H NMR of compound **45** in DMSO-d_6 conducted at 298.15 K.

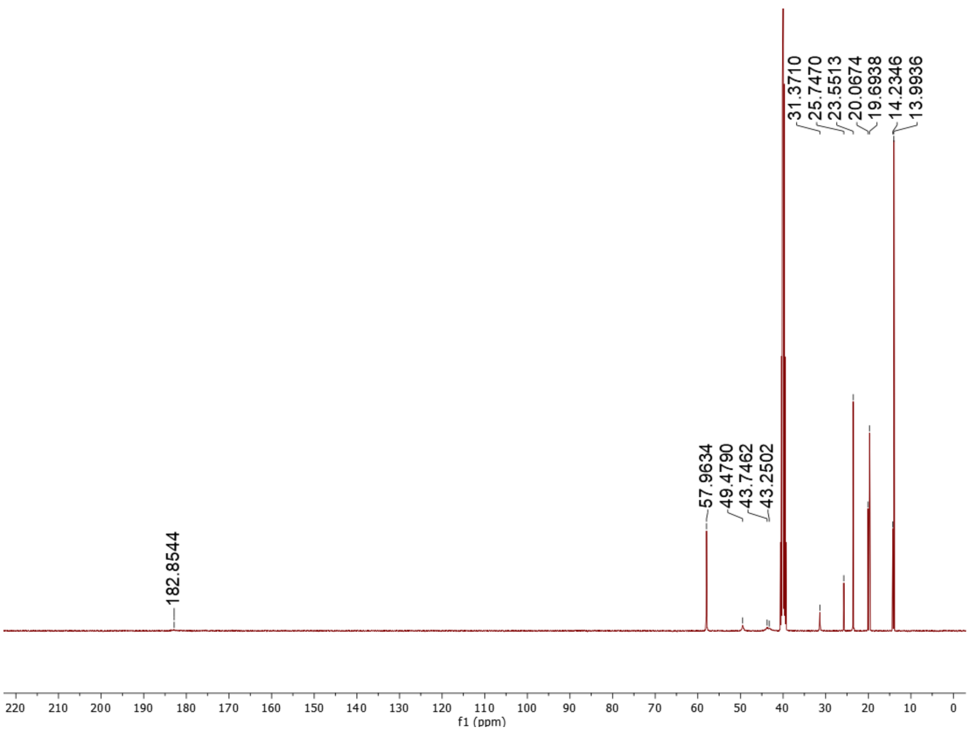

Figure S26 - $^{13}C\{^1H\}$ NMR of compound **45** in DMSO-d_6 conducted at 298.15 K.

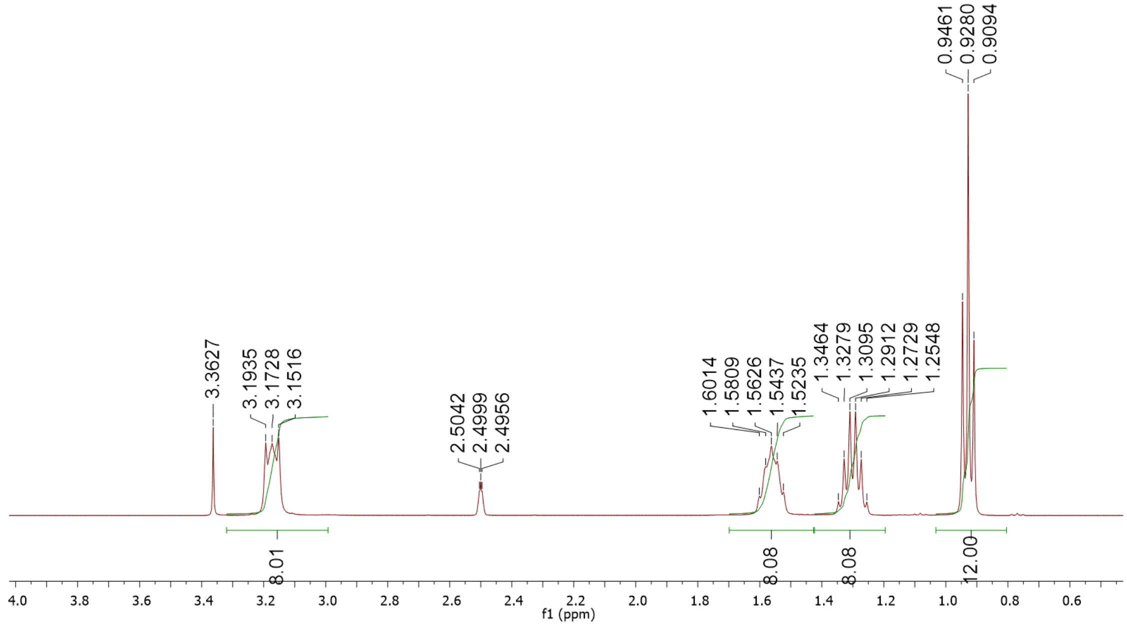

Figure S27 - ^1H NMR of compound **50** in DMSO-d_6 conducted at 298.15 K.

^{1}H DOSY NMR experiments

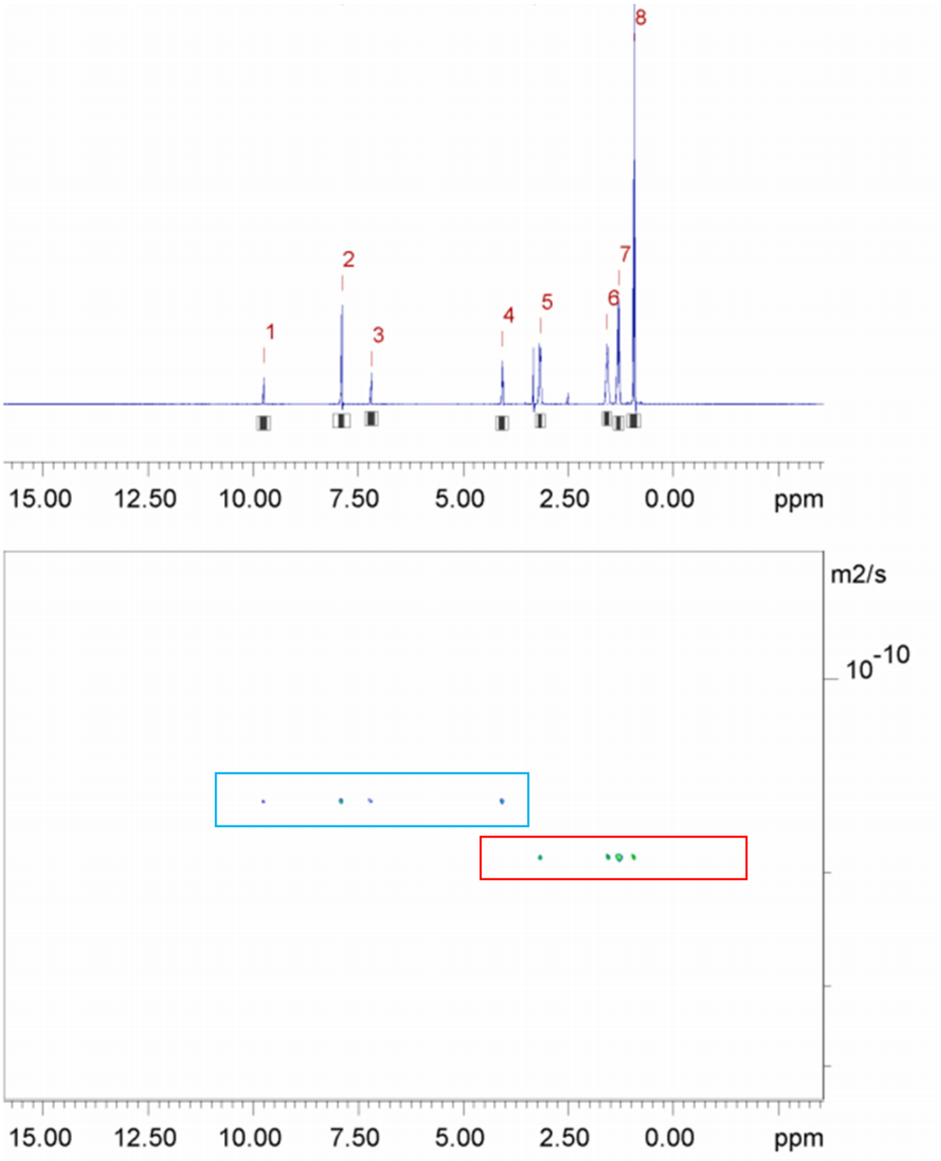

Peak name	F2 [ppm]	Io	error	D [m2/s]	error
1	9.748	1.29e+09	1.519e+05	1.54e-10	3.972e-14
2	7.892	4.52e+09	1.785e+05	1.54e-10	1.329e-14
3	7.177	1.58e+09	1.542e+05	1.55e-10	3.289e-14
4	4.067	2.69e+09	1.479e+05	1.54e-10	1.850e-14
5	3.155	8.22e+09	1.401e+05	1.90e-10	6.960e-15
6	1.574	9.29e+09	1.324e+05	1.90e-10	5.815e-15
7	1.300	1.18e+10	1.449e+05	1.90e-10	4.996e-15
8	0.936	1.95e+10	1.610e+05	1.90e-10	3.368e-15

Figure S28 - ^{1}H DOSY NMR spectrum of compounds **23** (55.56 mM) in DMSO-d_6 at 298 K and a table reporting the diffusion constants calculated for each peak used to determine the hydrodynamic diameter of the anionic components of **23** (d_H = 1.42 nm). Peaks 1-4 correspond to the anionic component of **23** while peaks 5-8 correspond to the cationic component of **23**.

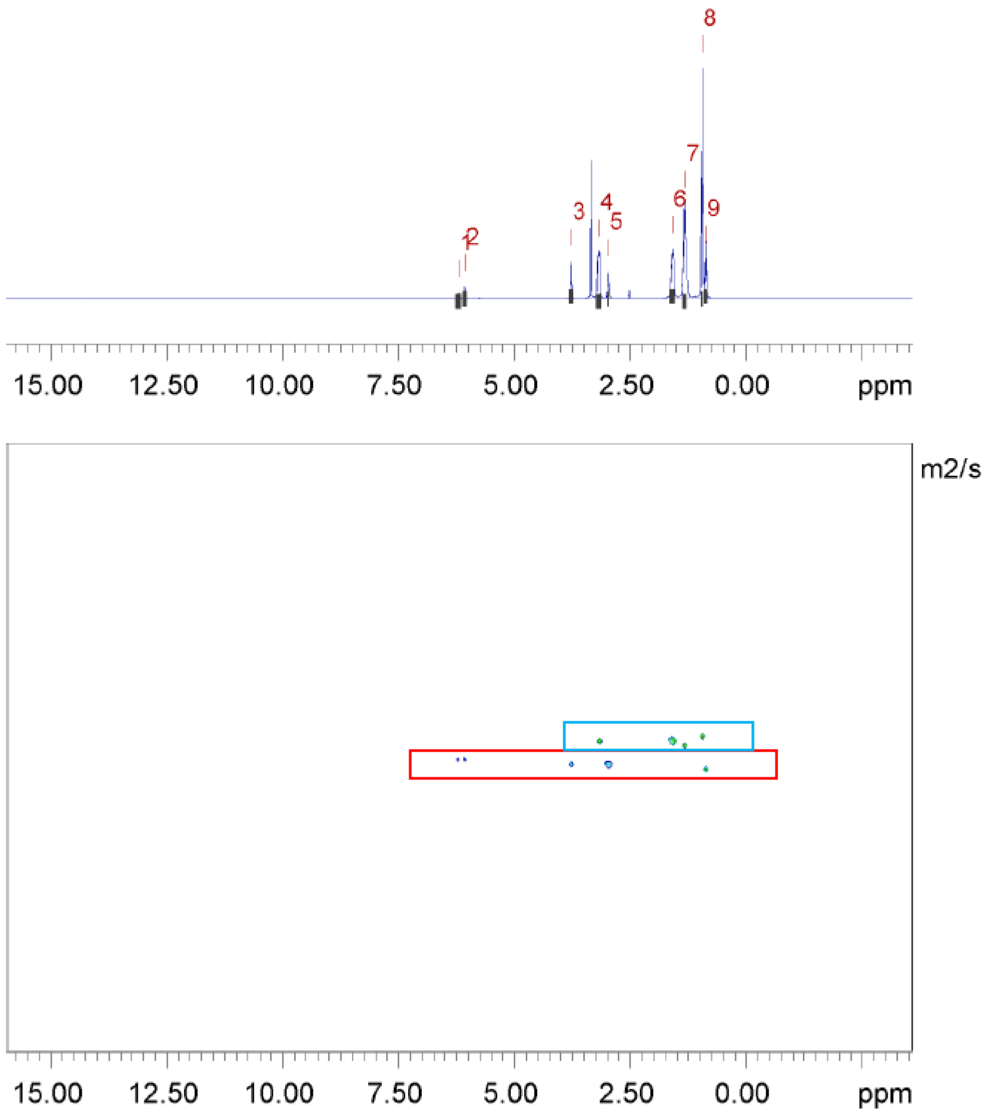

Peak name	F2 [ppm]	Io	error	D [m2/s]	error
1	6.201	1.72e+09	1.944e+05	2.09e-10	5.196e-14
2	6.067	2.26e+09	1.628e+05	2.09e-10	3.322e-14
3	3.768	4.88e+09	1.694e+05	2.10e-10	1.600e-14
4	3.174	1.50e+10	1.916e+05	1.96e-10	5.526e-15
5	2.972	3.67e+09	1.065e+05	2.12e-10	1.353e-14
6	1.584	1.67e+10	1.859e+05	1.95e-10	4.826e-15
7	1.321	2.43e+10	1.472e+05	2.00e-10	2.668e-15
8	0.946	2.70e+10	1.146e+05	1.94e-10	1.826e-15
9	0.869	8.48e+09	1.477e+05	2.13e-10	8.168e-15

Figure S29 - ^{1}H DOSY NMR spectrum of compounds **40** (55.56 mM) in DMSO-d_6 at 298 K and a table reporting the diffusion constants calculated for each peak used to determine the hydrodynamic diameter of the anionic components of **40** (d_H = 1.04 nm). Peaks 1-3, 5 and 9 correspond to the anionic component of **40** while peaks 4, 6-8 correspond to the cationic component of **40**.

Quantitative ^{1}H NMR experiments

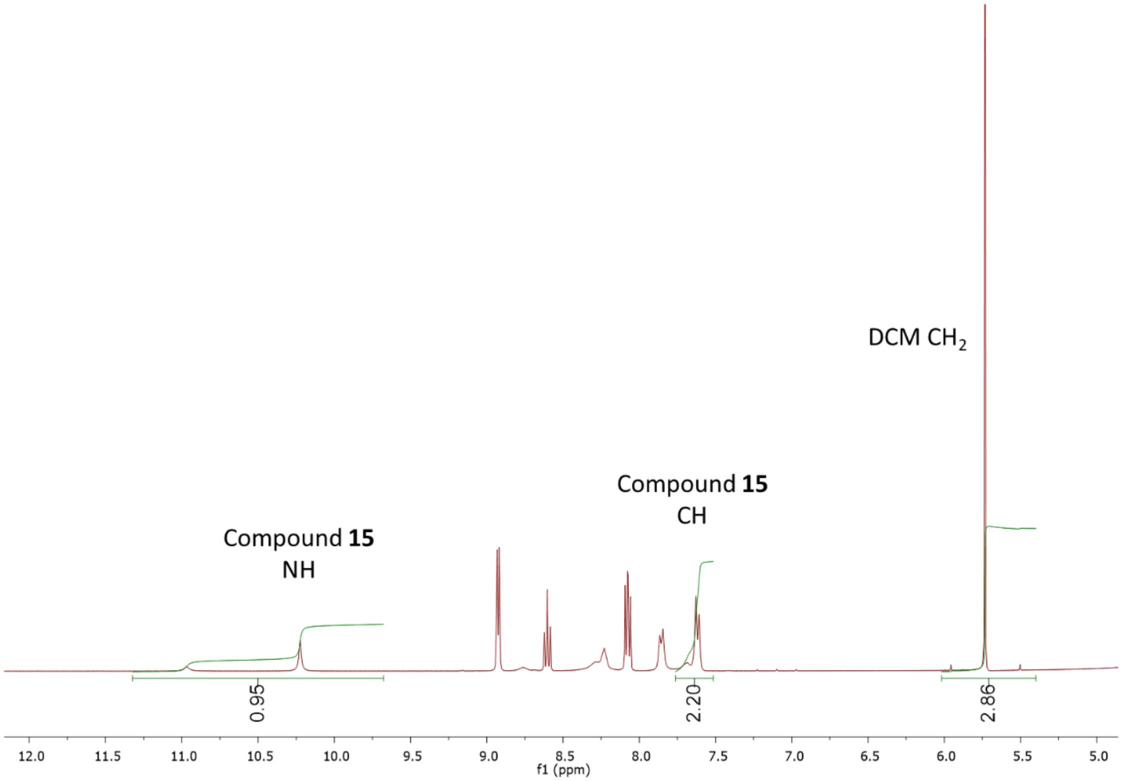

Figure S30 - ^{1}H NMR spectrum with a delay (d_1 = 60 s) of compound **15** (111.8 mM) in DMSO-d_6/ 1.0 % DCM. No apparent loss of compound observed upon comparative signal integration.

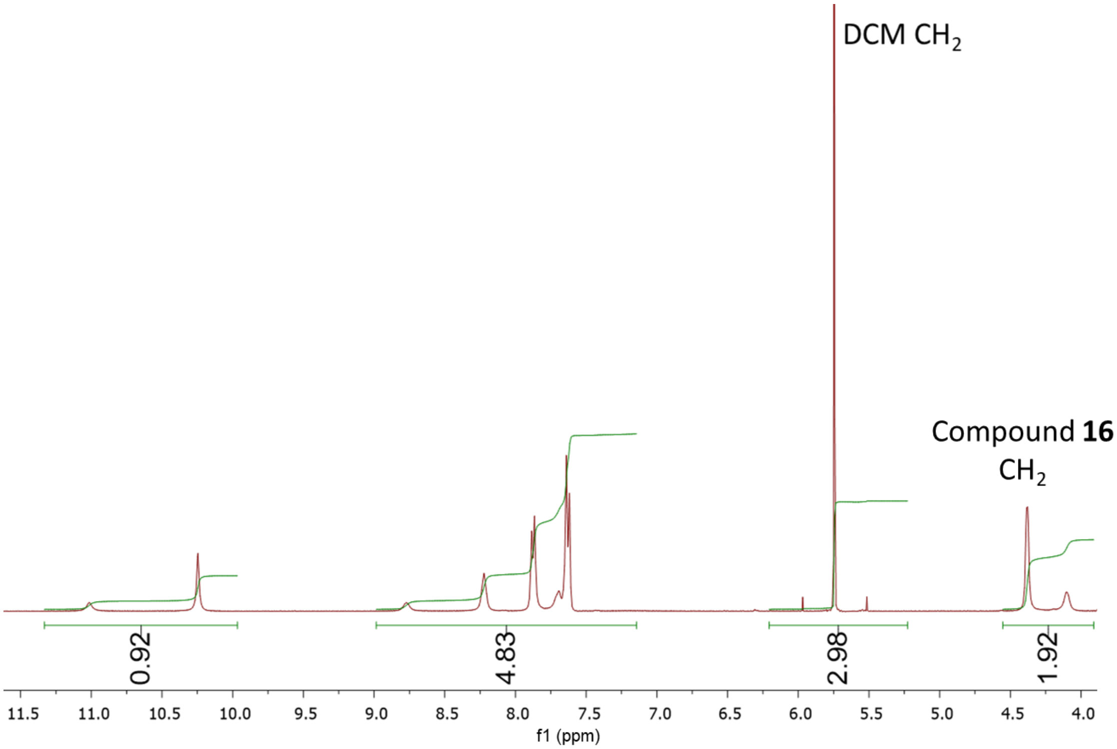

Figure S31 - ^{1}H NMR spectrum with a delay (d_1 = 60 s) of compound **16** (107.4 mM) in DMSO-d_6/ 1.0 % DCM. No apparent loss of compound observed upon comparative signal integration.

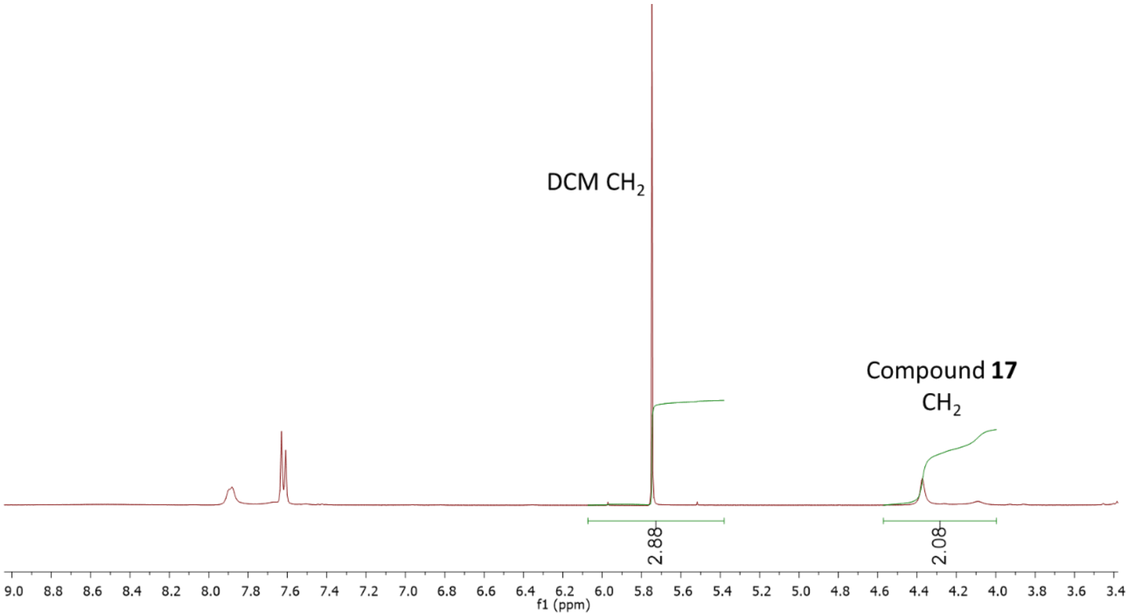

Figure S32 - ^{1}H NMR spectrum with a delay (d_1 = 60 s) of compound **16** (111.1 mM) in DMSO-d_6/ 1.0 % DCM. No apparent loss of compound observed upon comparative signal integration.

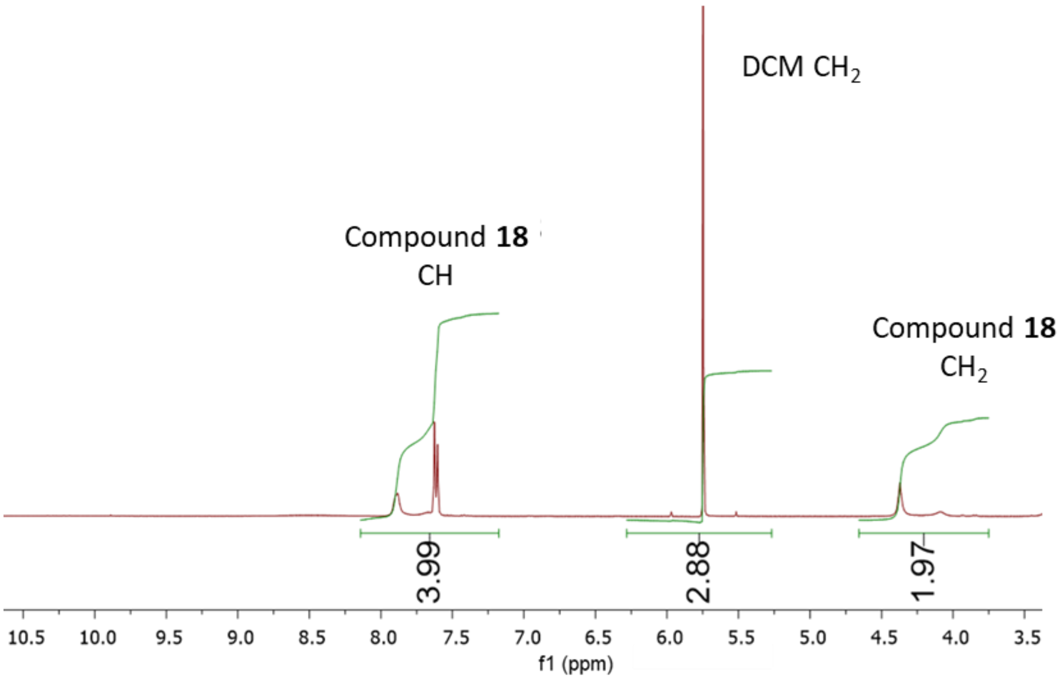

Figure S33 - ^{1}H NMR spectrum with a delay (d_1 = 60 s) of compound **18** (111.1 mM) in DMSO-d_6/ 1.0 % DCM. No apparent loss of compound observed upon comparative signal integration.

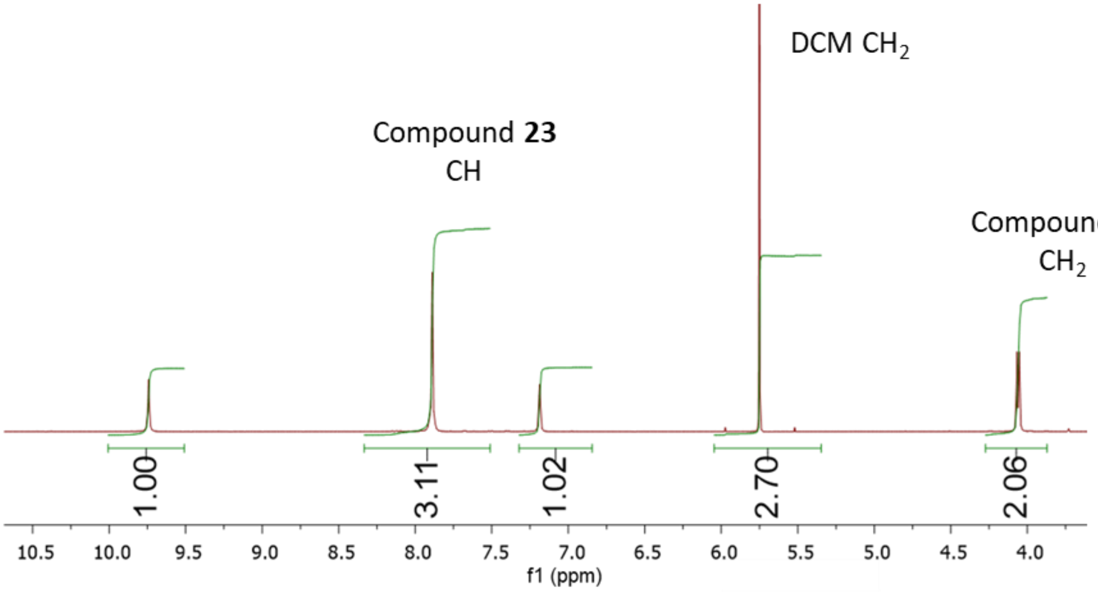

Figure S34 - ^{1}H NMR spectrum with a delay (d_1 = 60 s) of compound **23** (118.5 mM) in DMSO-d_6/ 1.0 % DCM. No apparent loss of compound observed upon comparative signal integration.

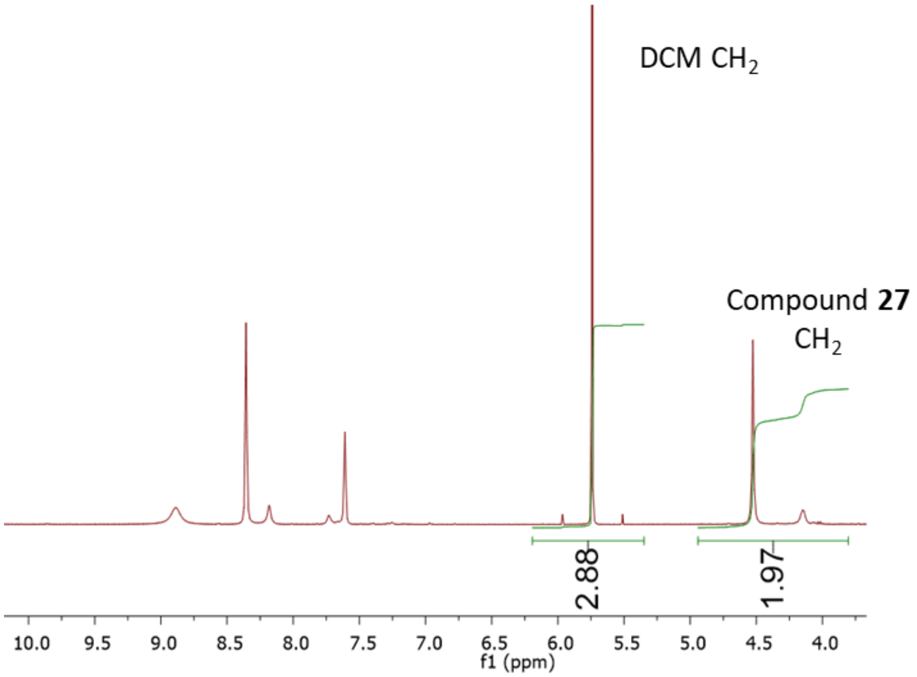

Figure S35 - ^{1}H NMR spectrum with a delay (d_1 = 60 s) of compound **27** (111.1 mM) in DMSO-d_6/ 1.0 % DCM. No apparent loss of compound observed upon comparative signal integration.

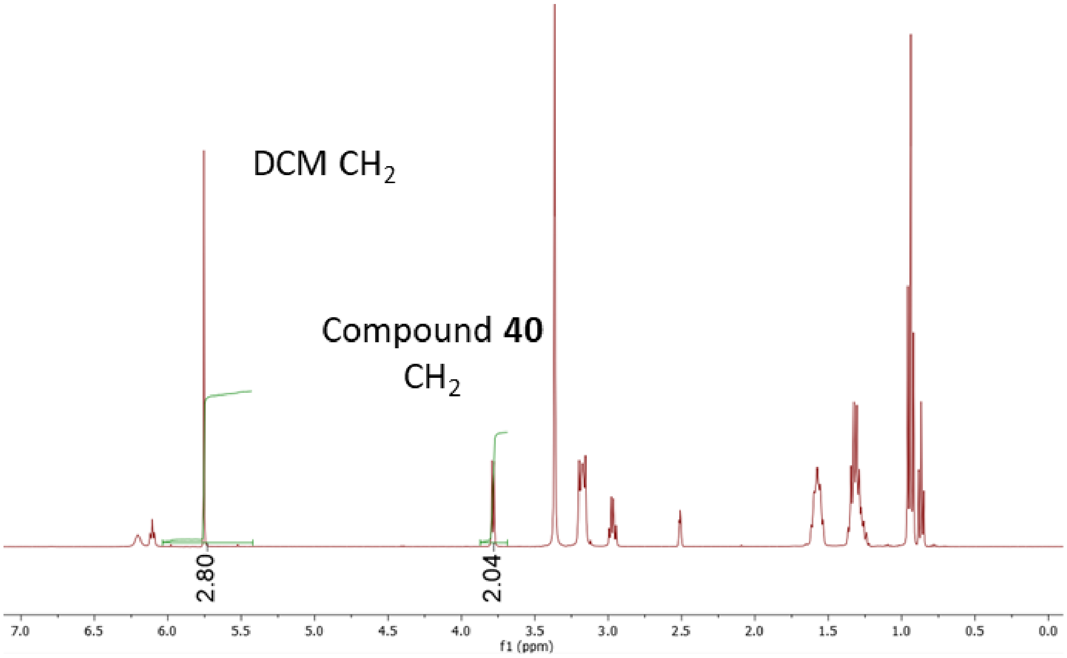

Figure S36 - ^{1}H NMR spectrum with a delay (d_1 = 60 s) of compound **40** (114.3 mM) in DMSO-d_6/ 1.0 % DCM. No apparent loss of compound observed upon comparative signal integration.

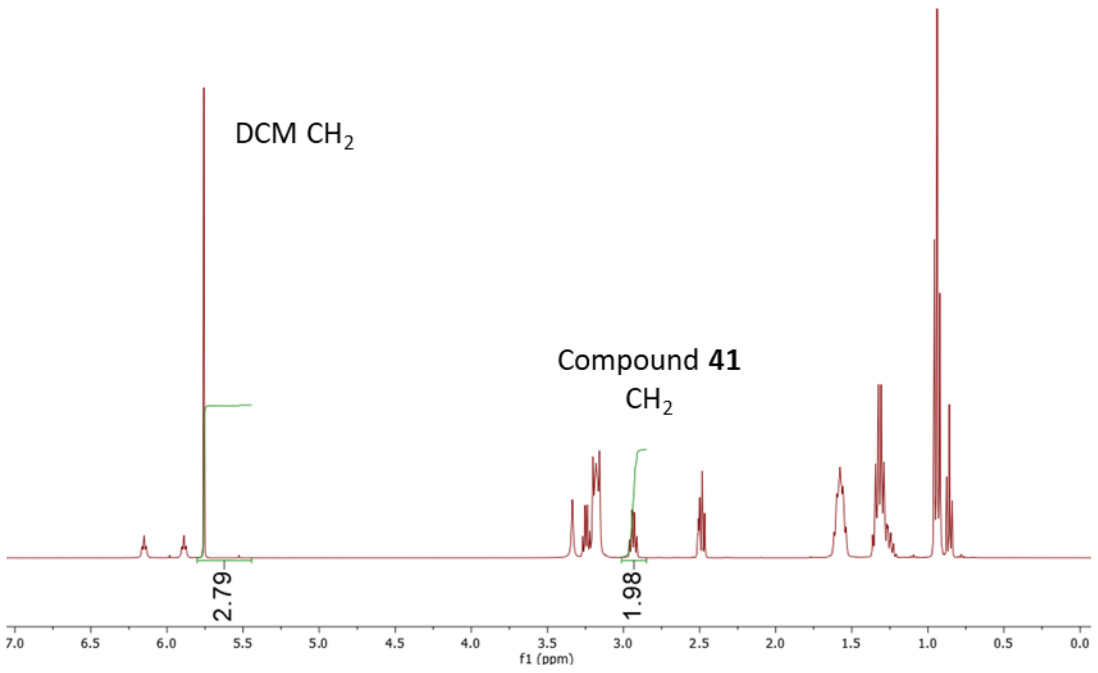

Figure S37 - ^{1}H NMR spectrum with a delay (d_1 = 60 s) of compound **41** (114.7 mM) in DMSO-d_6/ 1.0 % DCM. No apparent loss of compound observed upon comparative signal integration.

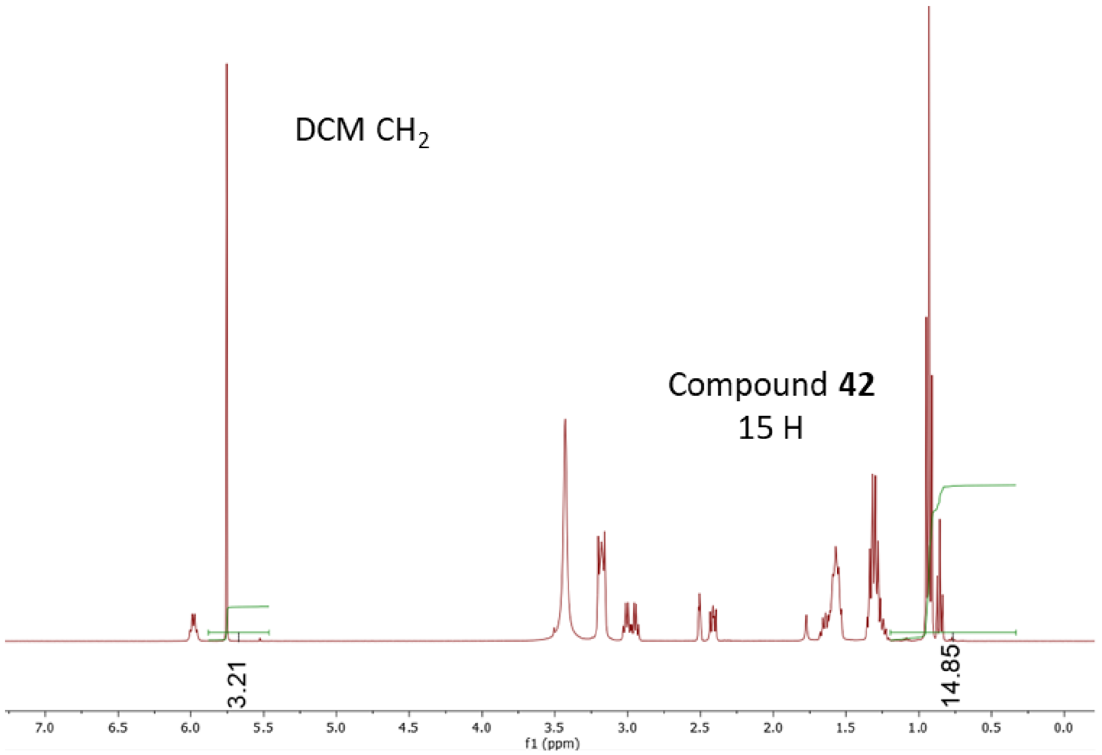

Figure S38 - ^{1}H NMR spectrum with a delay (d_1 = 60 s) of compound **42** (99.7 mM) in DMSO-d_6/ 1.0 % DCM. No apparent loss of compound observed upon comparative signal integration.

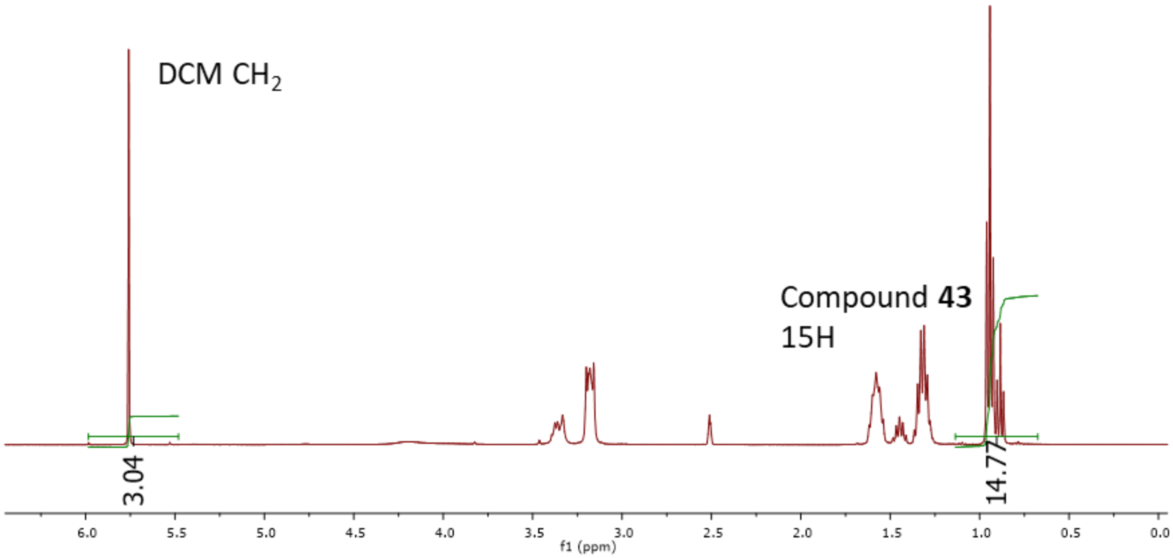

Figure S39 - ^{1}H NMR spectrum with a delay (d_1 = 60 s) of compound **43** (105.3 mM) in DMSO-d_6/ 1.0 % DCM. No apparent loss of compound observed upon comparative signal integration.

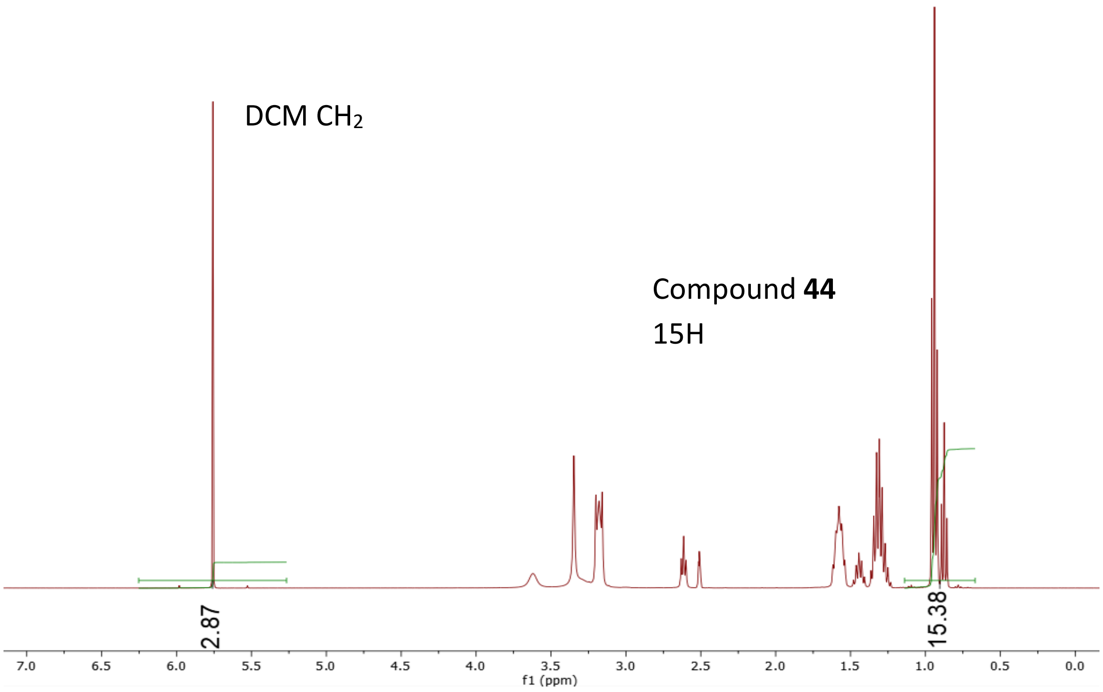

Figure S40 - ^{1}H NMR spectrum with a delay (d_1 = 60 s) of compound **44** (111.5 mM) in DMSO-d_6/ 1.0 % DCM. No apparent loss of compound observed upon comparative signal integration.

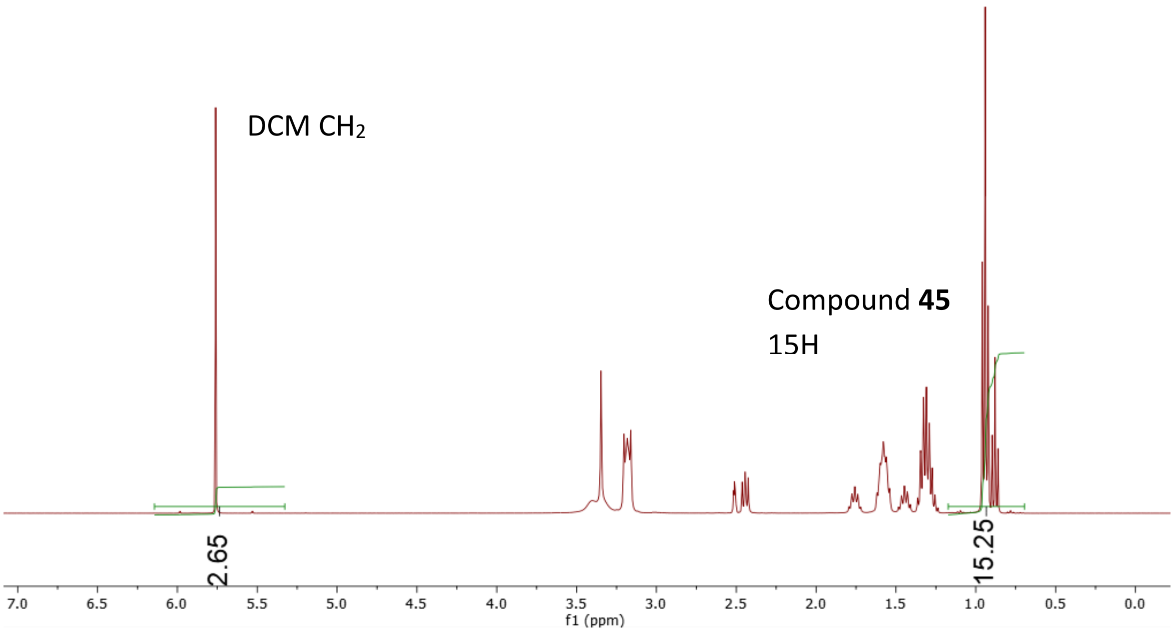

Figure S41 - ^1H NMR spectrum with a delay (d_1 = 60 s) of compound **45** (120.8 mM) in DMSO-d_6/ 1.0 % DCM. No apparent loss of compound observed upon comparative signal integration.

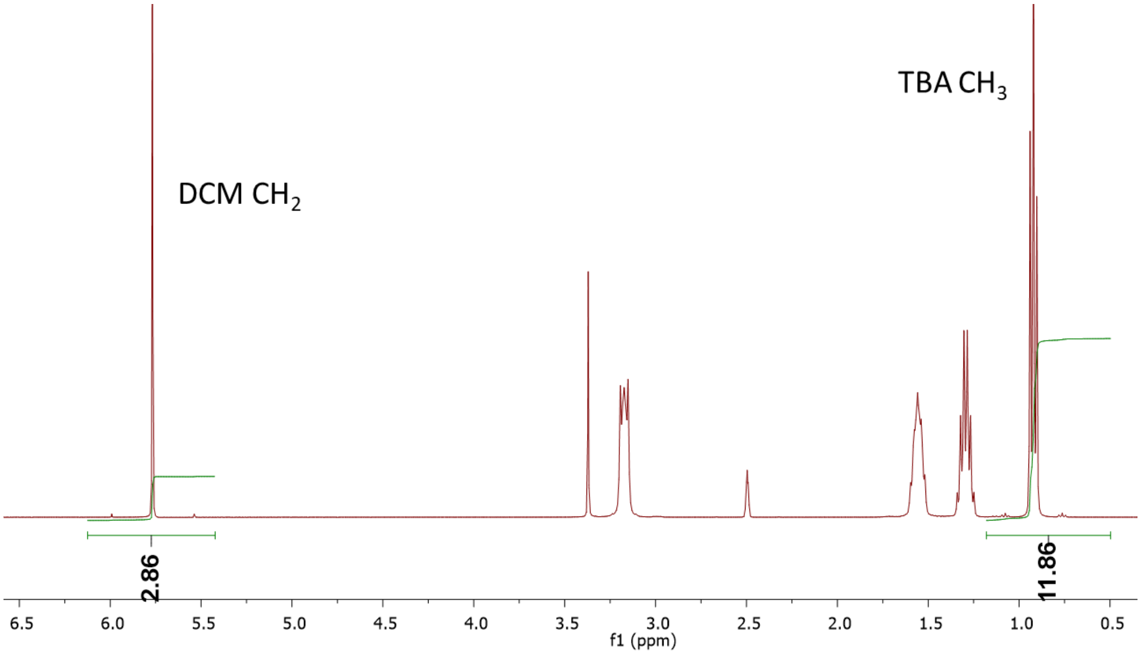

Figure S42 - ^1H NMR spectrum with a delay (d_1 = 60 s) of compound **50** (111.9 mM) in DMSO-d_6/ 1.0 % DCM. No apparent loss of compound observed upon comparative signal integration.

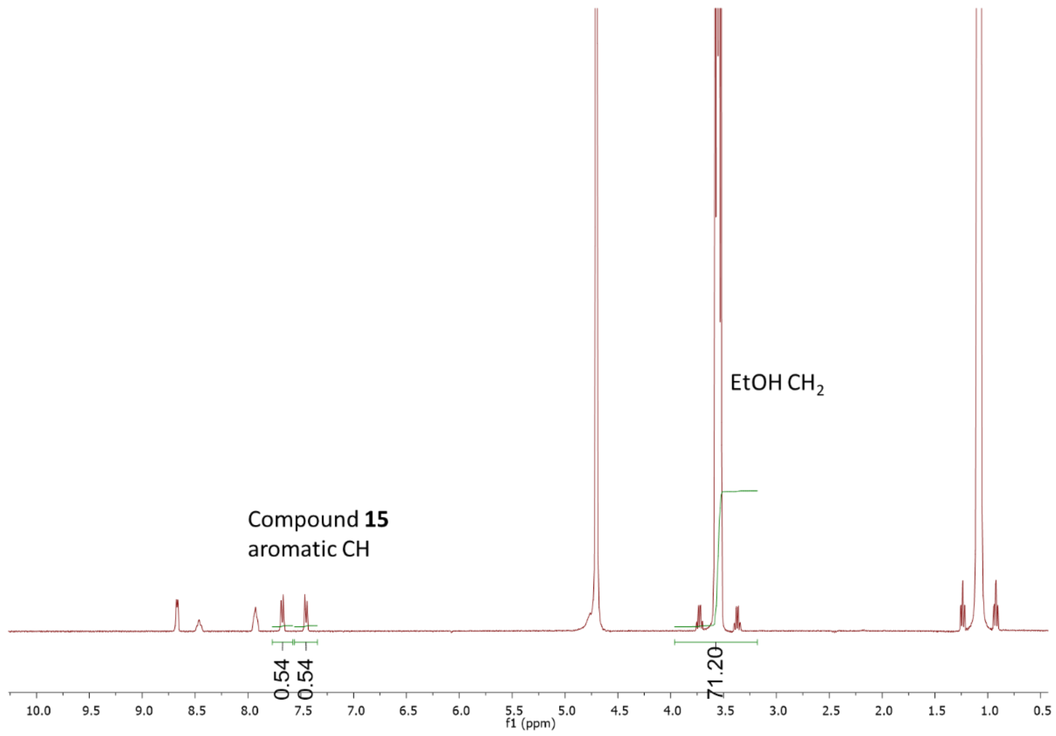

Figure S43 - ^{1}H NMR spectrum with a delay (d_1 = 60 s) of compound **15** (6.01 mM) in D_2O/ 5.0 % EtOH. An apparent 46 % loss of compound was observed upon comparative signal integration.

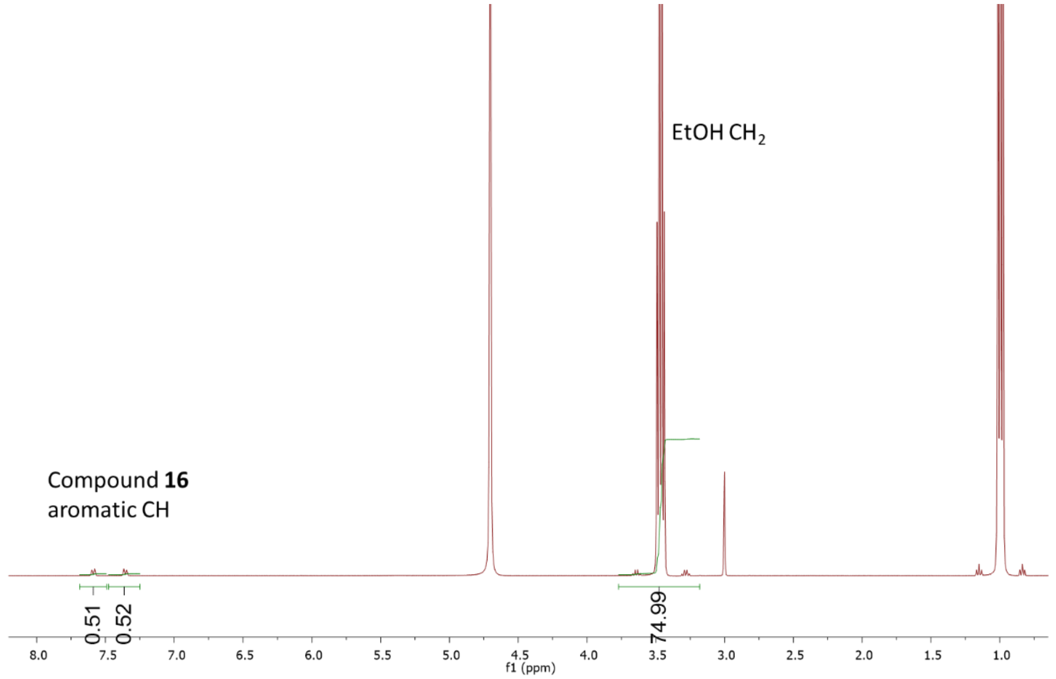

Figure S44 - ^{1}H NMR spectrum with a delay (d_1 = 60 s) of compound **16** (5.73 mM) in D_2O/ 5.0 % EtOH. An apparent 48 % loss of compound was observed upon comparative signal integration.

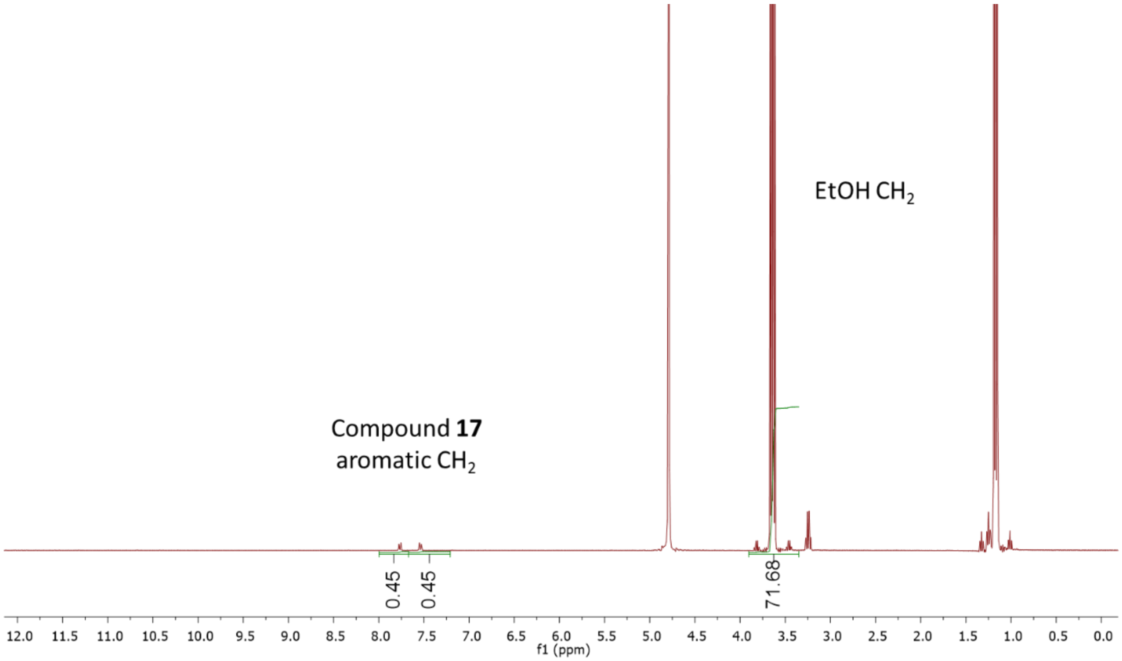

Figure S45 - ^{1}H NMR spectrum with a delay (d_1 = 60 s) of compound **17** (5.99 mM) in D_2O/ 5.0 % EtOH. An apparent 55 % loss of compound was observed upon comparative signal integration.

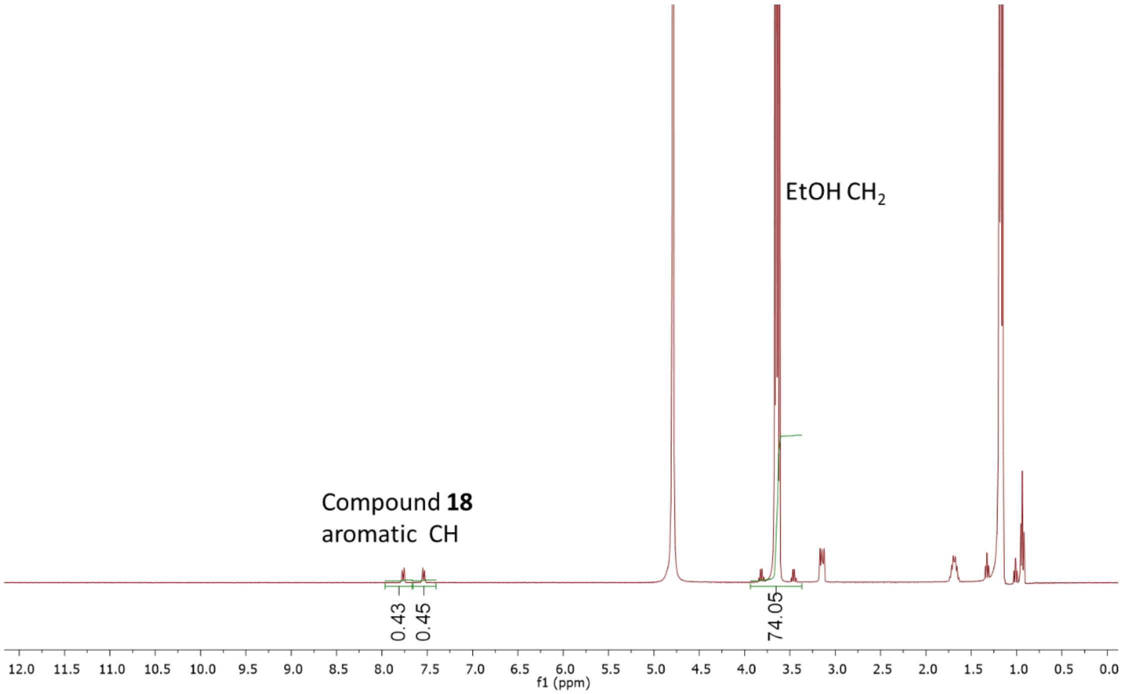

Figure S46 - ^{1}H NMR spectrum with a delay (d_1 = 60 s) of compound **18** (5.81 mM) in D_2O/ 5.0 % EtOH. An apparent 55 % loss of compound was observed upon comparative signal integration.

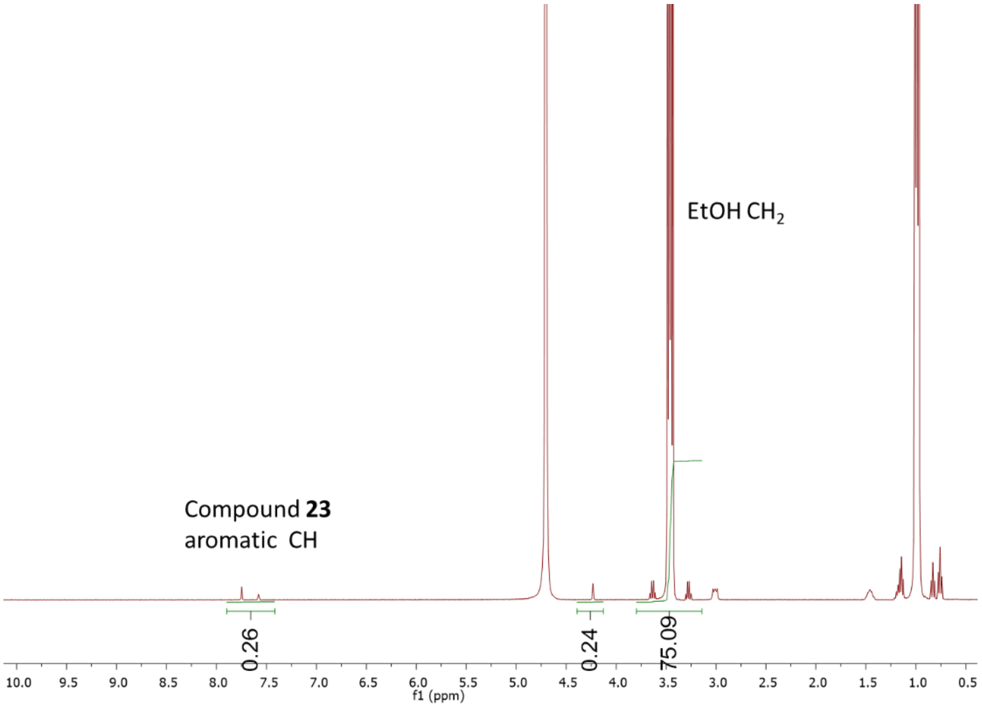

Figure S47 - ^{1}H NMR spectrum with a delay (d_1 = 60 s) of compound **23** (5.73 mM) in D_2O/ 5.0 % EtOH. An apparent 76 % loss of compound was observed upon comparative signal integration.

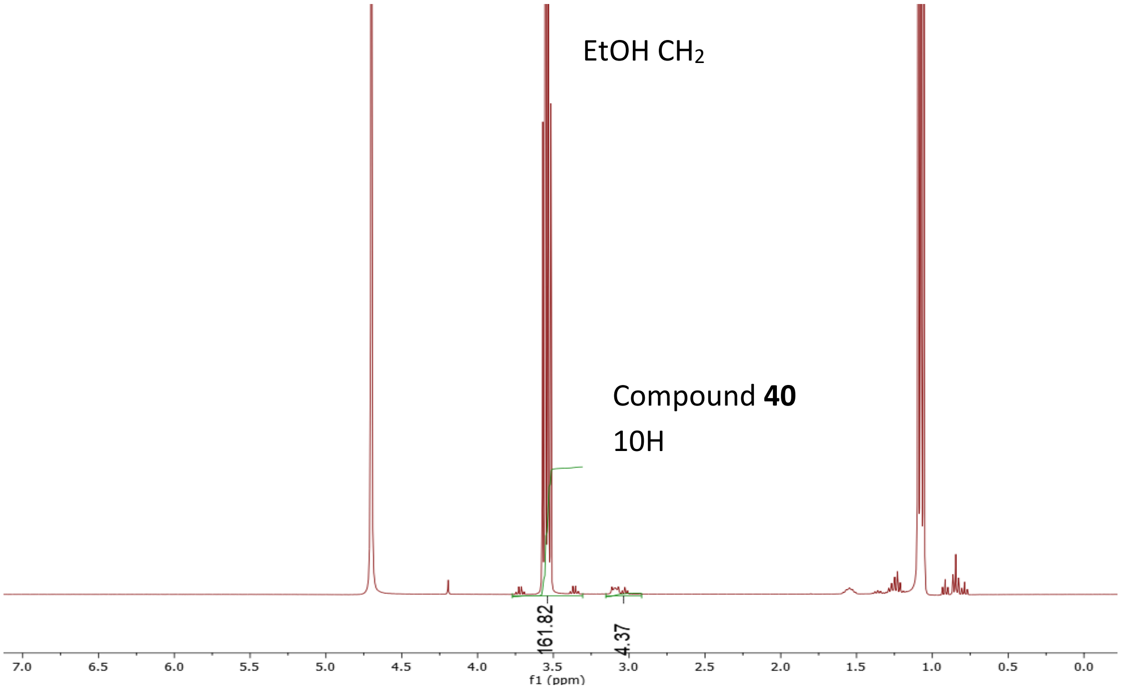

Figure S48 - ^{1}H NMR spectrum with a delay (d_1 = 60 s) of compound **40** (5.31 mM) in D_2O/ 5.0 % EtOH. An apparent 56 % loss of compound was observed upon comparative signal integration.

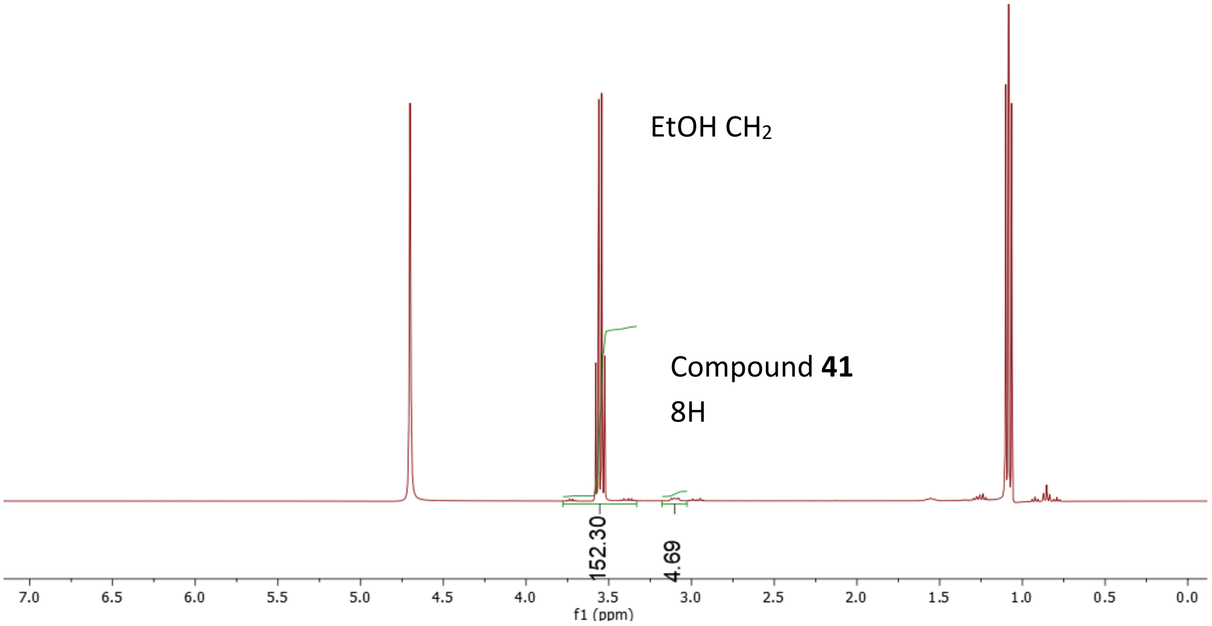

Figure S49 - ^{1}H NMR spectrum with a delay (d_1 = 60 s) of compound **41** (5.65 mM) in D_2O/ 5.0 % EtOH. An apparent 41 % loss of compound was observed upon comparative signal integration.

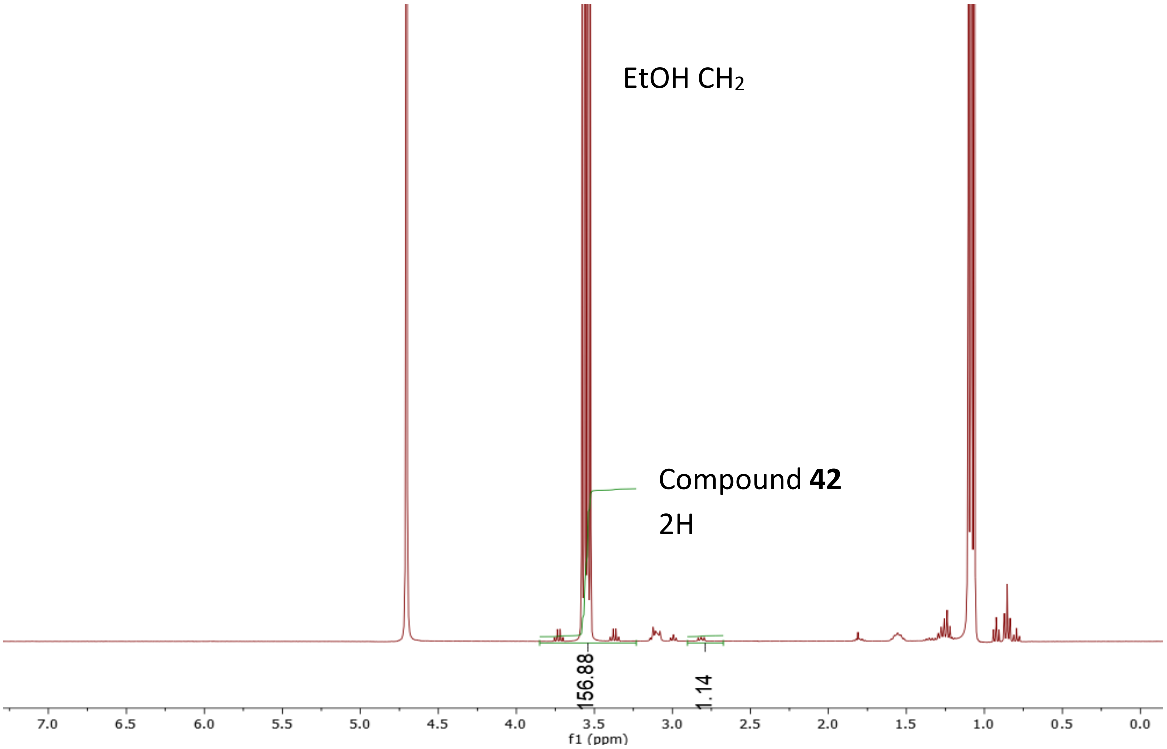

Figure S50 - ^{1}H NMR spectrum with a delay (d_1 = 60 s) of compound **42** (5.48 mM) in D_2O/ 5.0 % EtOH. An apparent 43 % loss of compound was observed upon comparative signal integration.

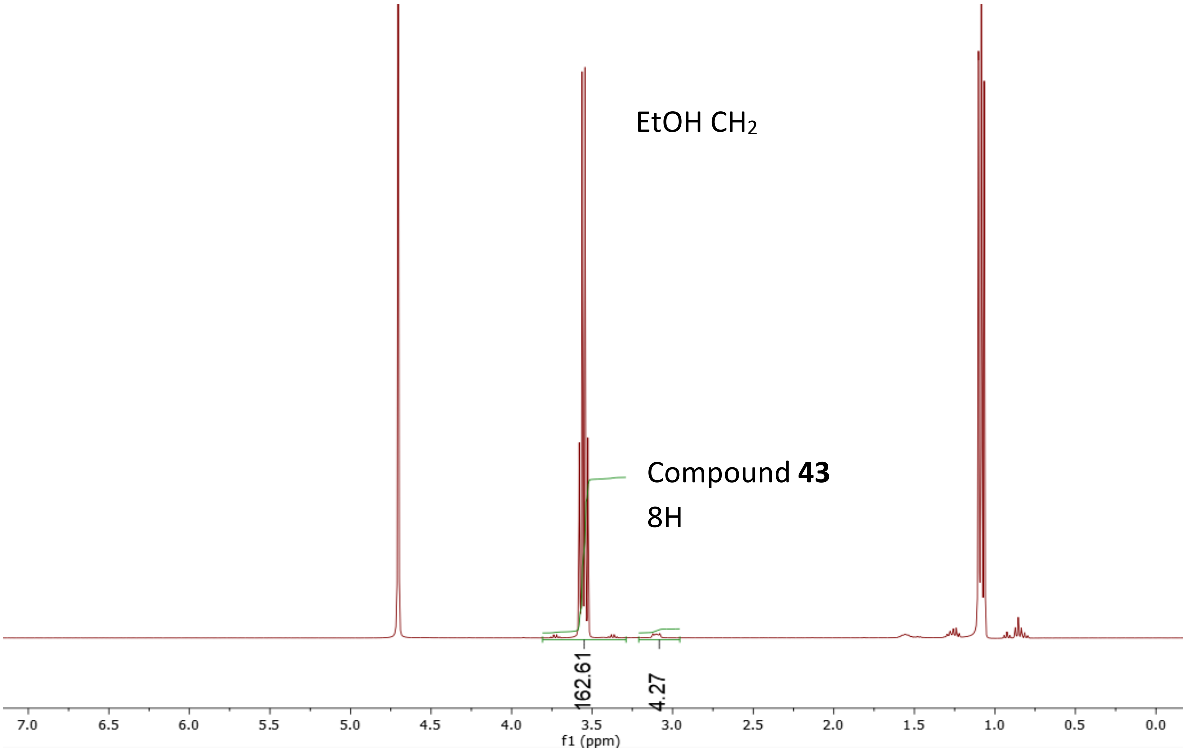

Figure S51 - ^{1}H NMR spectrum with a delay (d_1 = 60 s) of compound **43** (5.29 mM) in D_2O/ 5.0 % EtOH. An apparent 47 % loss of compound was observed upon comparative signal integration.

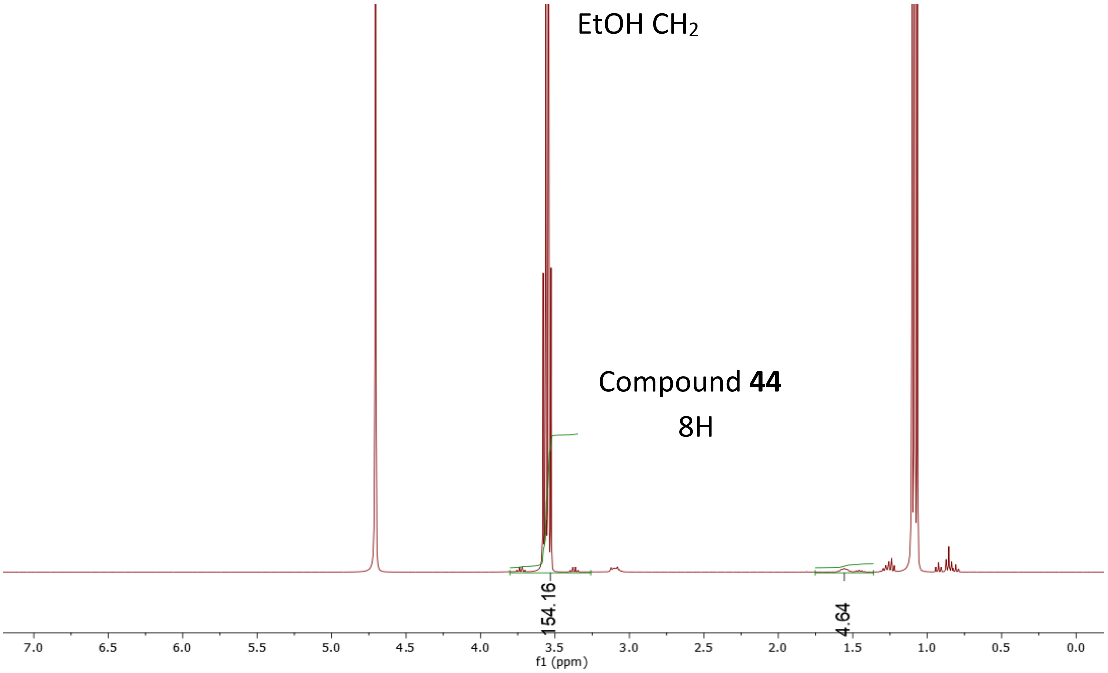

Figure S52 - ^{1}H NMR spectrum with a delay (d_1 = 60 s) of compound **44** (5.58 mM) in D_2O/ 5.0 % EtOH. An apparent 48 % loss of compound was observed upon comparative signal integration.

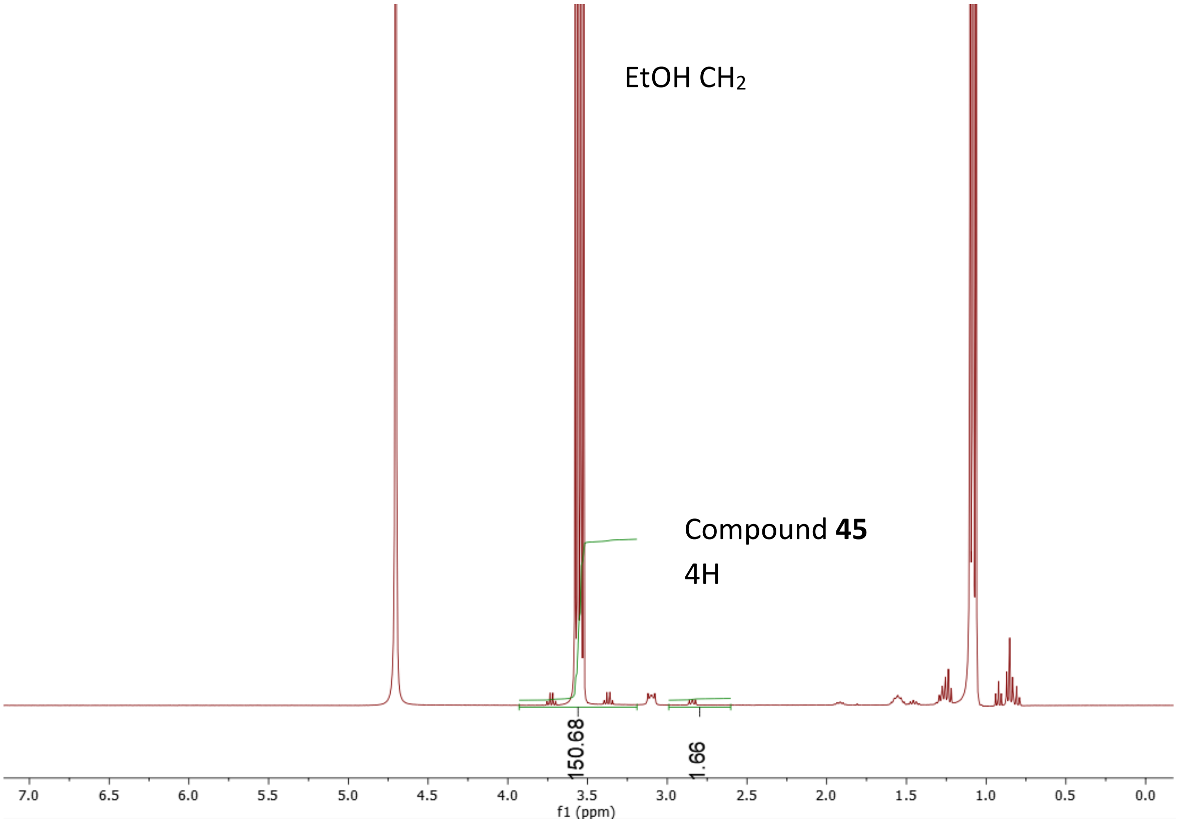

Figure S53 - ^{1}H NMR spectrum with a delay (d_1 = 60 s) of compound **45** (5.71 mM) in D_2O/ 5.0 % EtOH. An apparent 59 % loss of compound was observed upon comparative signal integration.

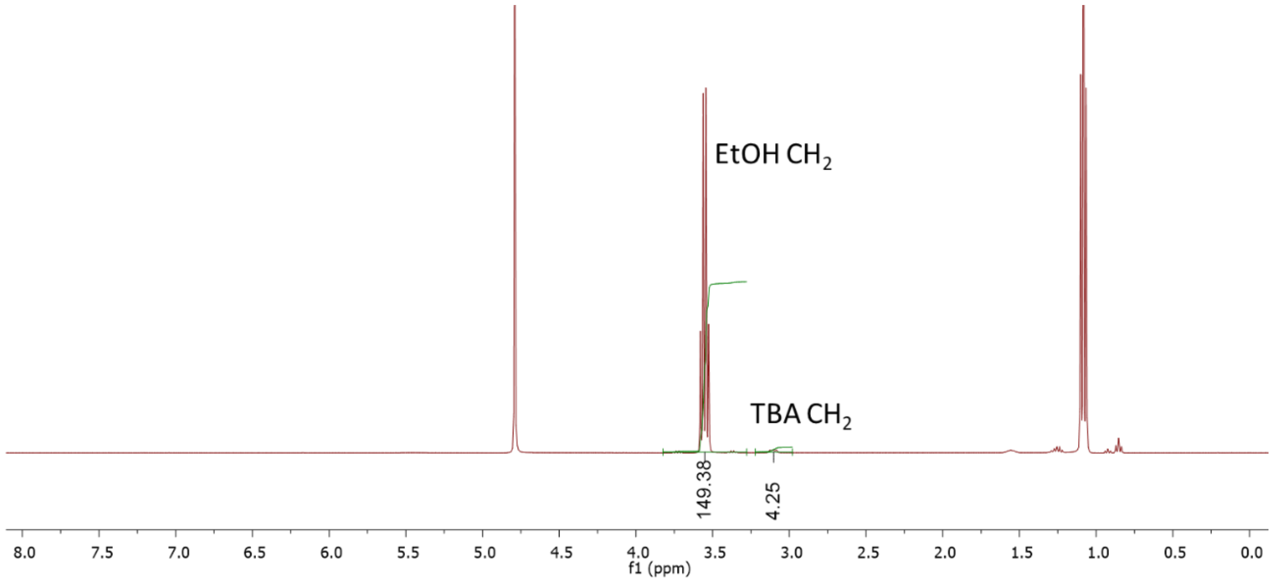

Figure S54 - ^{1}H NMR spectrum with a delay (d_1 = 60 s) of compound **50** (5.76 mM) in D_2O/ 5.0 % EtOH. An apparent 47 % loss of compound was observed upon comparative signal integration.

^{1}H NMR self-association studies

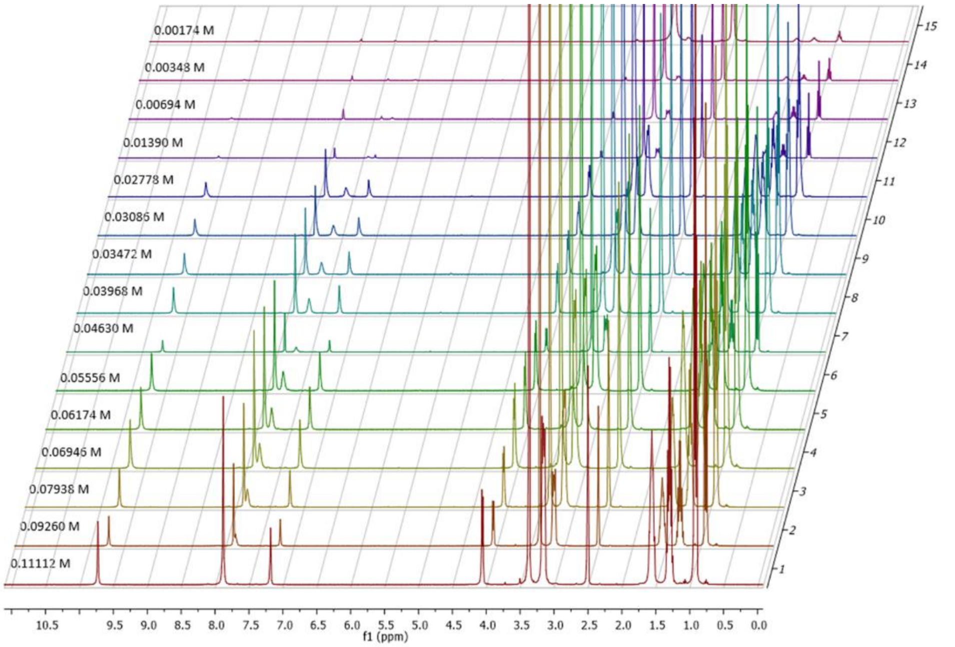

Figure S55 - ^{1}H NMR stack plot of compound **23** in DMSO-d_6 0.5 % H_2O solution. Samples were prepared in series with an aliquot of the most concentrated solution undergoing serial dilution.

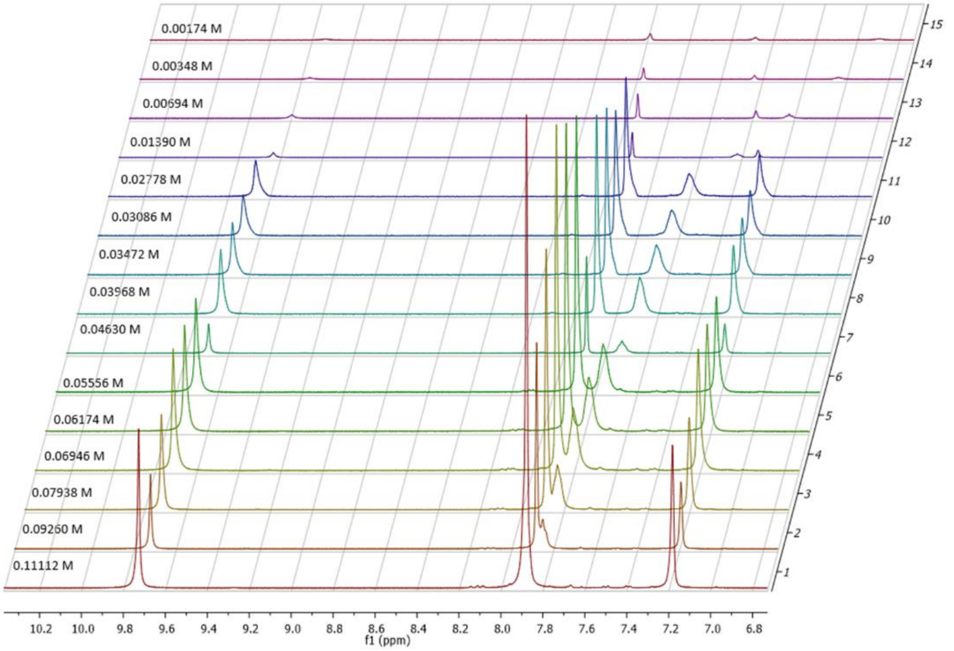

Figure S56 - Enlarged ^{1}H NMR stack plot of compound **23** in DMSO-d_6 0.5 % H_2O solution. Samples were prepared in series with an aliquot of the most concentrated solution undergoing serial dilution.

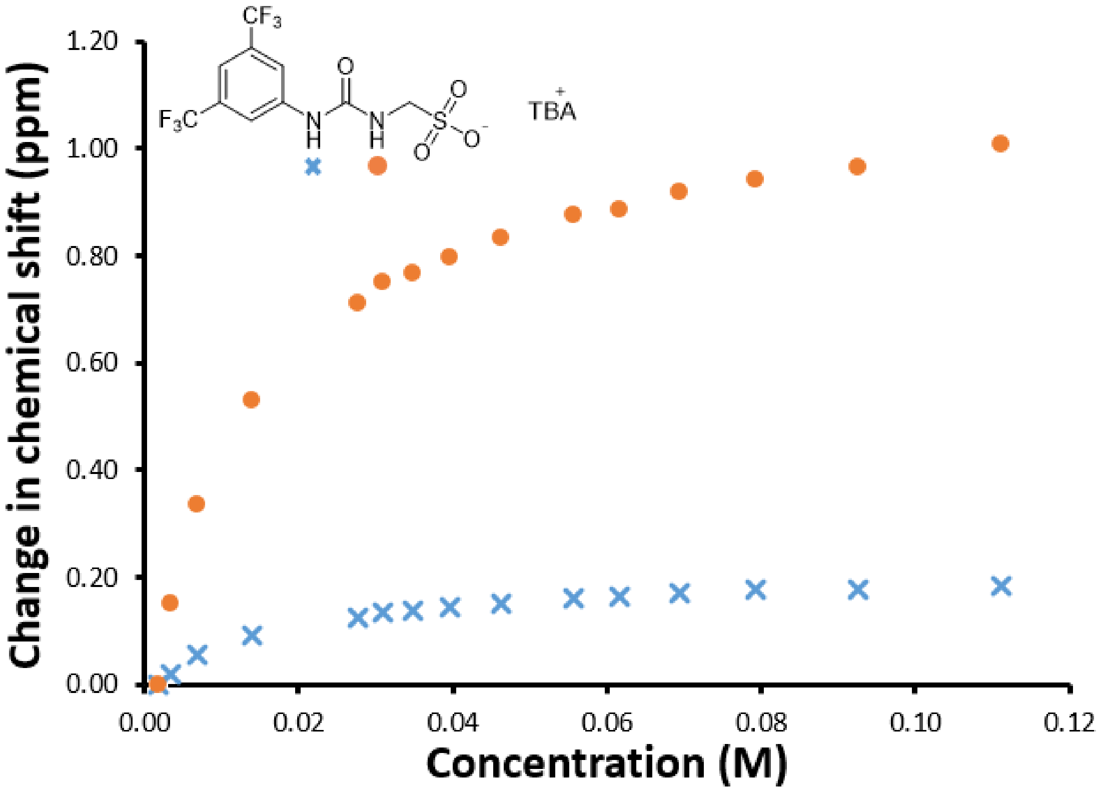

Figure S57 - Graph illustrating the ^{1}H NMR down-field change in chemical shift of urea NH resonances with increasing concentration of compound **23** in DMSO-d_6 0.5 % H_2O (298.15 K).

Self-association constant calculation

Compound **23** - Dilution study in DMSO-d_6 5 % H_2O. Values calculated from data gathered from both NH 1 and 2.

Equal K/Dimerization model

K_e =127.48 M^{-1} ± 0.2090 % K_{dim} = 63.74 M^{-1} ± 0.5105 %

http://app.supramolecular.org/bindfit/view/9a81e451-257f-40af-9fdd-de118b1cc132

CoEK model

K_e = 127.28 M^{-1} ± 1.0985 % K_{dim} = 63.64 M^{-1} ± 0.5492 %   ρ = 0.95 ± 3.0510 %

http://app.supramolecular.org/bindfit/view/ef2981ee-8b18-47ce-a8e9-00986a9ebd67

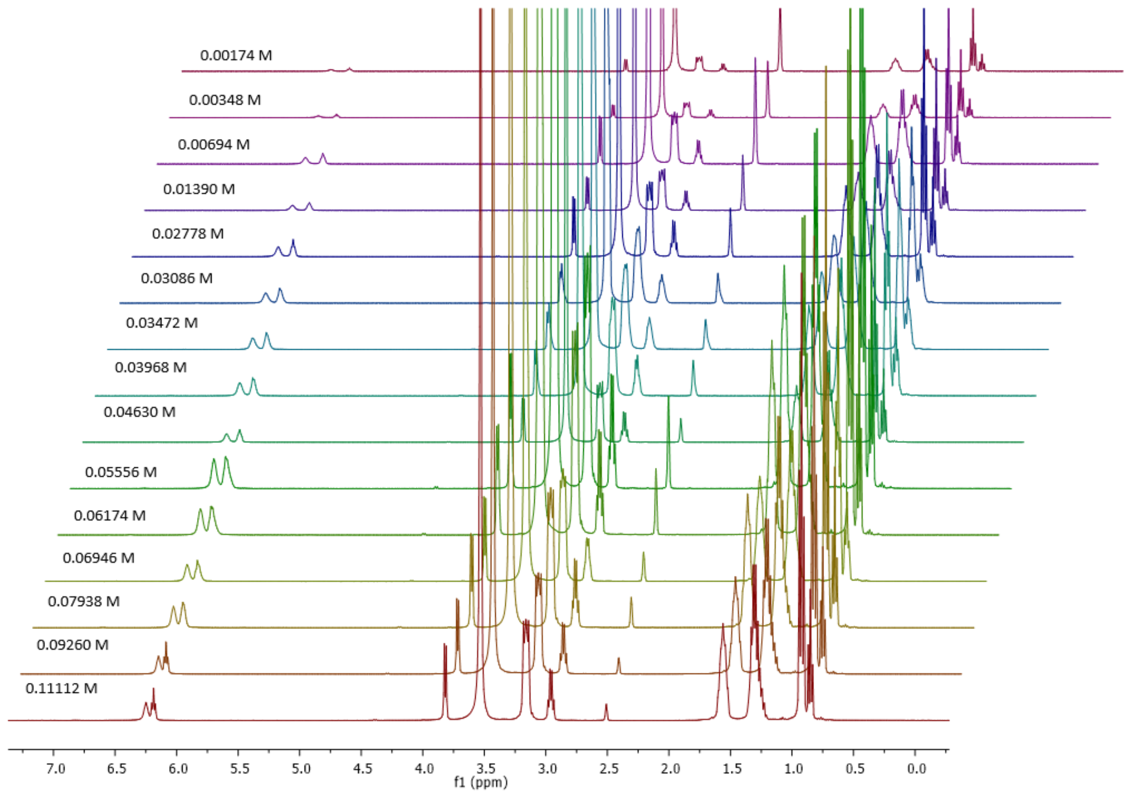

Figure S58 - ^{1}H NMR stack plot of compound **40** in DMSO-*d*$_6$ 0.5 % H_2O solution. Samples were prepared in series with an aliquot of the most concentrated solution undergoing serial dilution.

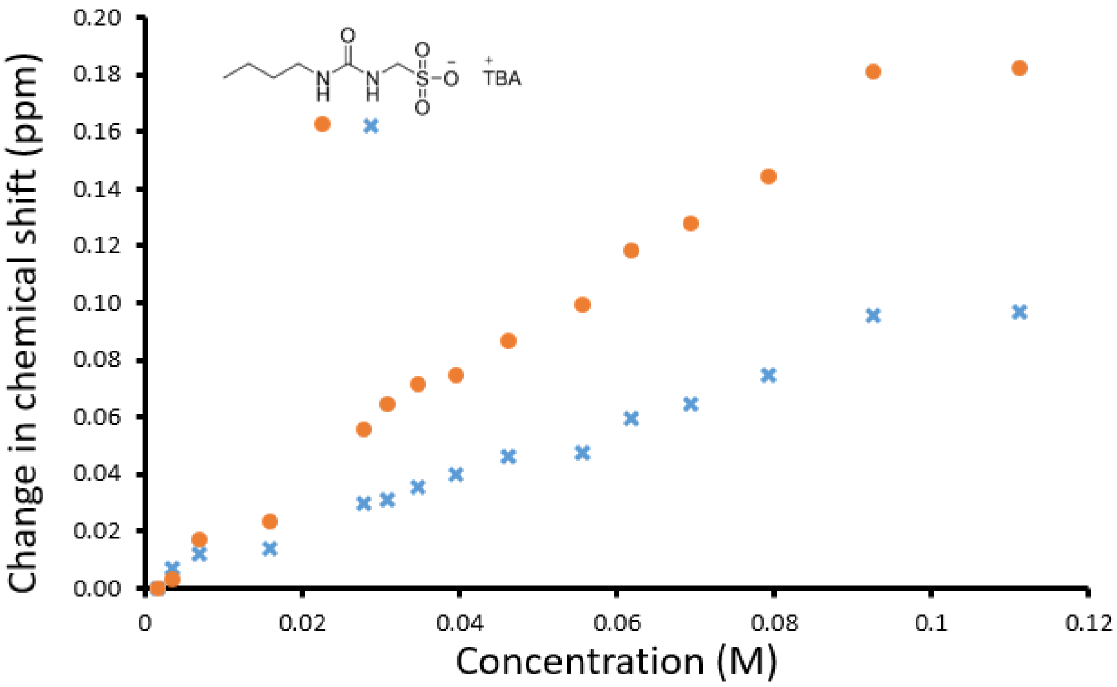

Figure S59 - Graph illustrating the ^{1}H NMR down-field change in chemical shift of urea NH resonances with increasing concentration of compound **40** in DMSO-d_6 0.5 % H_2O (298.15 K).

Self-association constant calculation

Compound **40** - Dilution study in DMSO-d_6 5 % H_2O. Values calculated from data gathered from both NH 1 and 2.

Equal K/Dimerization model

K_e = 8.54e-4 M^{-1} ± 3.1686 % K_{dim} = 4.27e-4 M^{-1} ± 1.5843 %

http://app.supramolecular.org/bindfit/view/9e73b9e9-e046-4d72-868e-c1ec1faa515e

CoEK model

Compound **40** could not be fitted to the CoEK model.

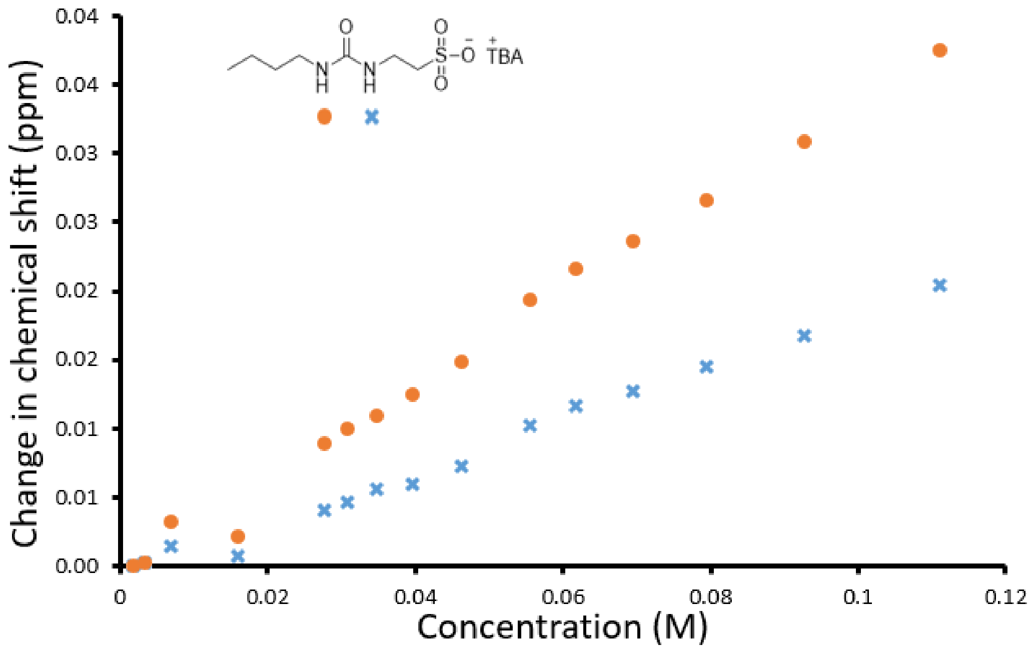

Figure S60 - Graph illustrating the ^{1}H NMR down-field change in chemical shift of urea NH resonances with increasing concentration of compound **41** in DMSO-d_6 0.5 % H_2O (298.15 K).

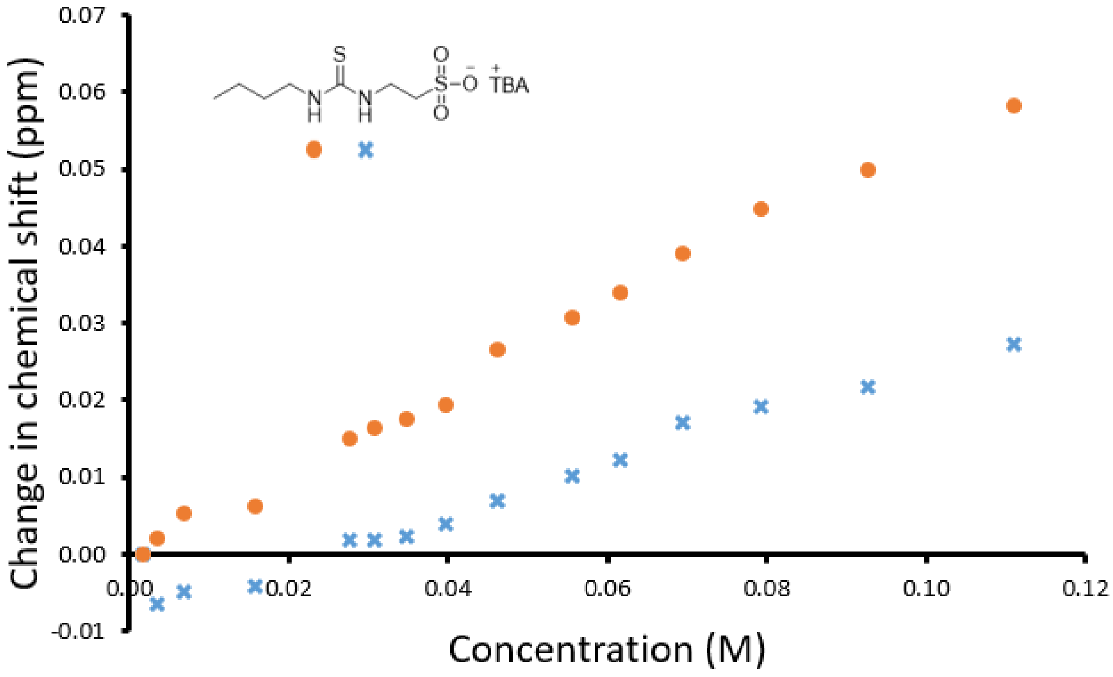

Figure S61 - Graph illustrating the ^{1}H NMR down-field change in chemical shift of urea NH resonances with increasing concentration of compound **44** in DMSO-d_6 0.5 % H_2O (298.15 K).

Dynamic Light Scattering data

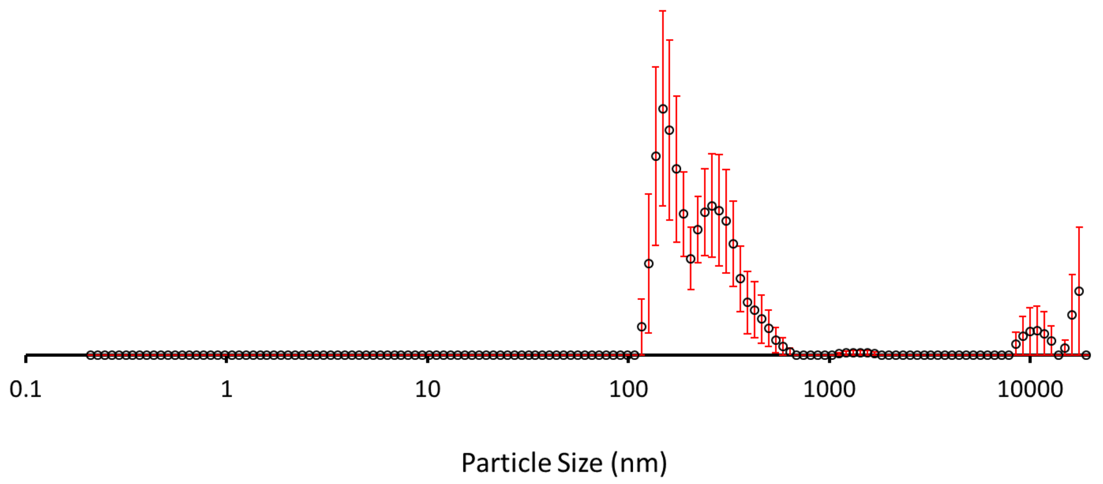

Figure S62 - The average intensity particle size distribution calculated using 9 DLS runs for compound **15** (5.56 mM) in an EtOH:H_2O (1:19) solution at 298 K.

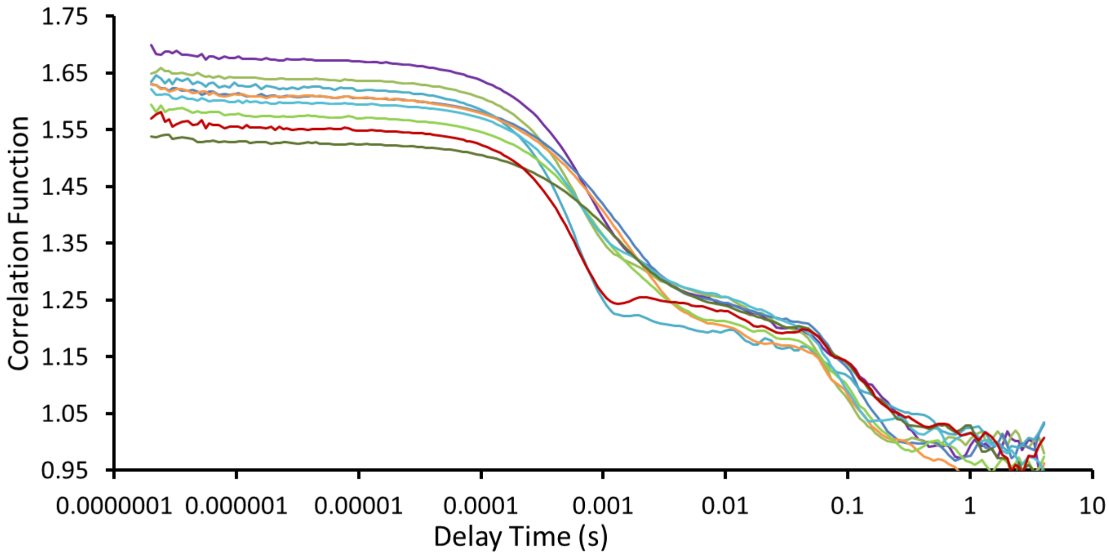

Figure S63 - Correlation function data for 9 DLS runs of compound **15** (5.56 mM) in an EtOH:H_2O (1:19) solution at 298 K.

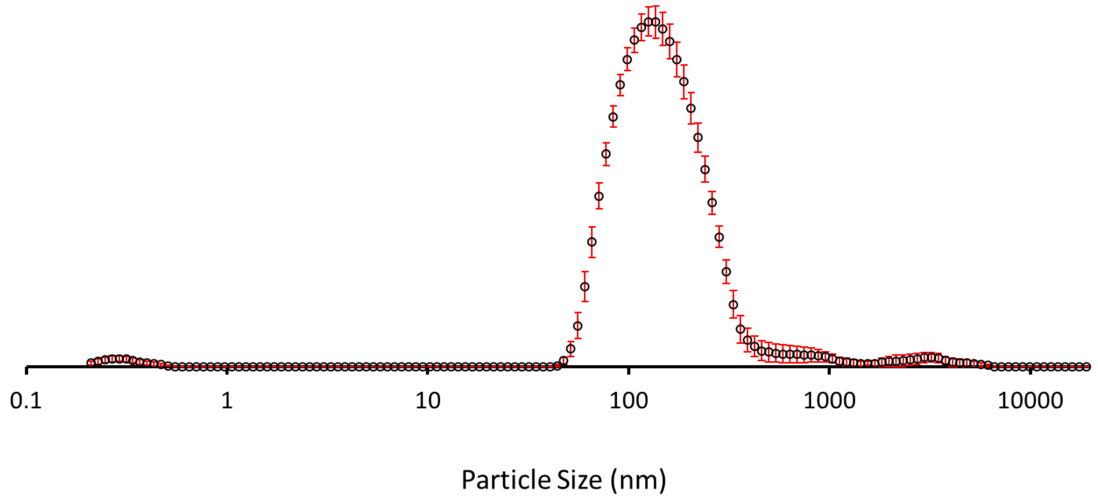

Figure S64 - The average intensity particle size distribution calculated using 9 DLS runs for compound **17** (5.56 mM) in an EtOH:H_2O (1:19) solution at 298 K.

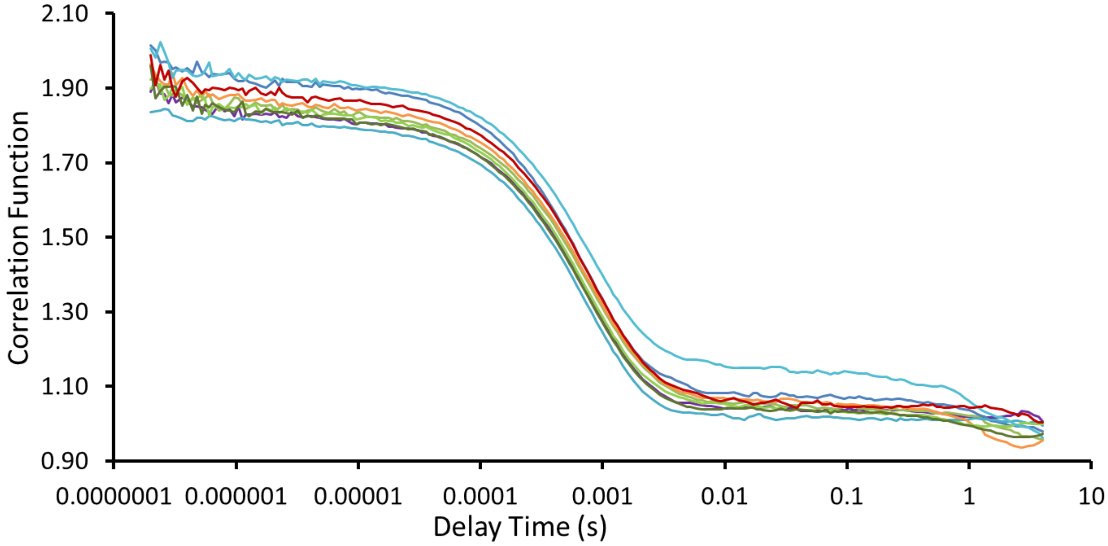

Figure S65 - Correlation function data for 9 DLS runs of compound **17** (5.56 mM) in an EtOH:H_2O (1:19) solution at 298 K.

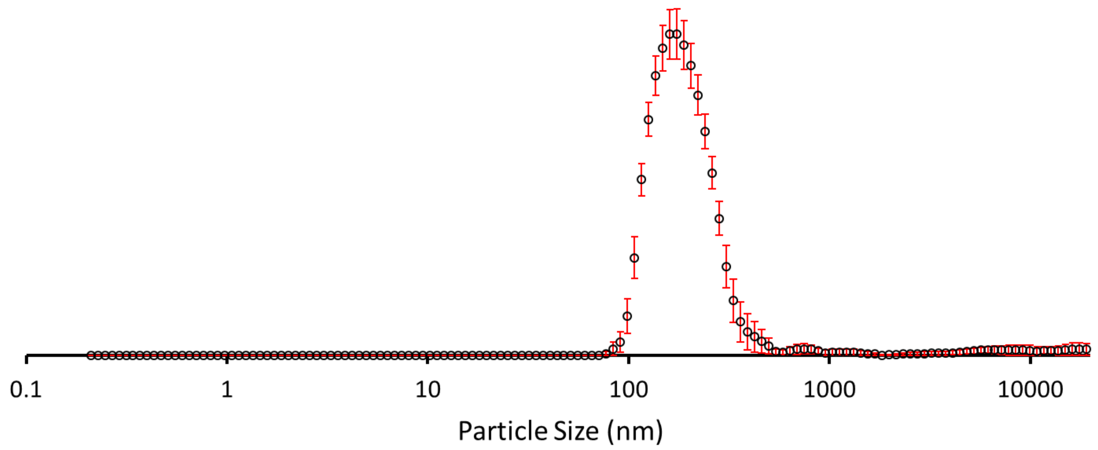

Figure S66 - The average intensity particle size distribution calculated using 9 DLS runs for compound **18** (5.56 mM) in an EtOH:H_2O (1:19) solution at 298 K.

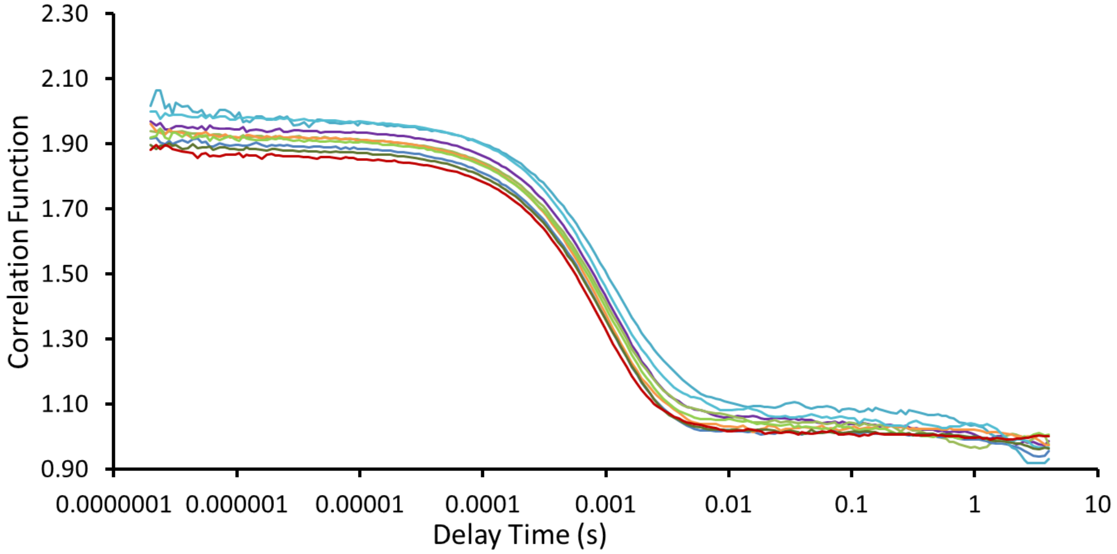

Figure S67 - Correlation function data for 9 DLS runs of compound **18** (5.56 mM) in an EtOH:H_2O (1:19) solution at 298 K.

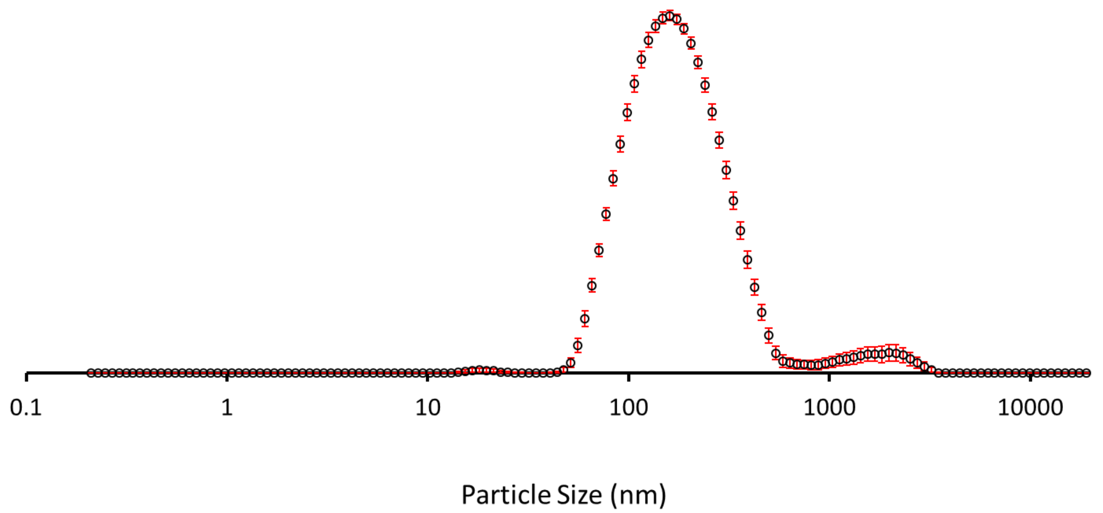

Figure S68 - The average intensity particle size distribution calculated using 9 DLS runs for compound **23** (5.56 mM) in an EtOH:H_2O (1:19) solution at 298 K.

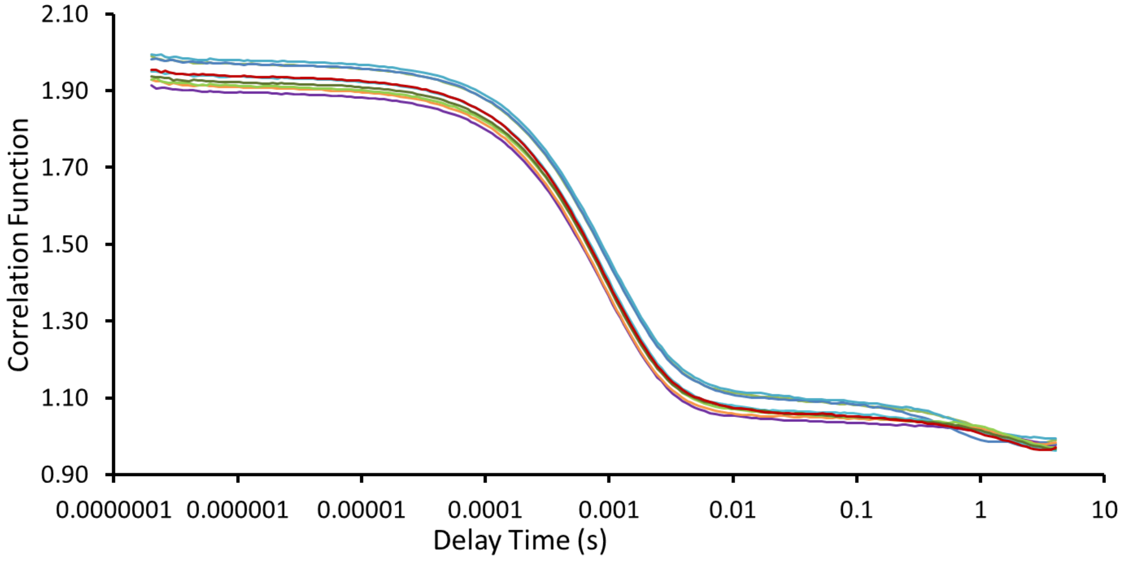

Figure S69 - Correlation function data for 9 DLS runs of compound **23** (5.56 mM) in an EtOH:H_2O (1:19) solution at 298 K.

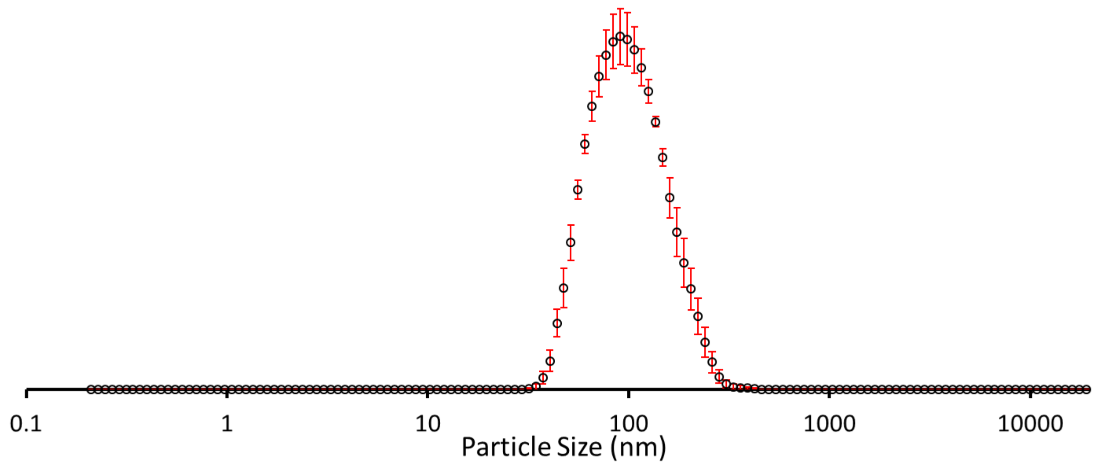

Figure S70 - The average intensity particle size distribution calculated using 9 DLS runs for compound **23** (0.56 mM) in an EtOH:H_2O (1:19) solution at 298 K.

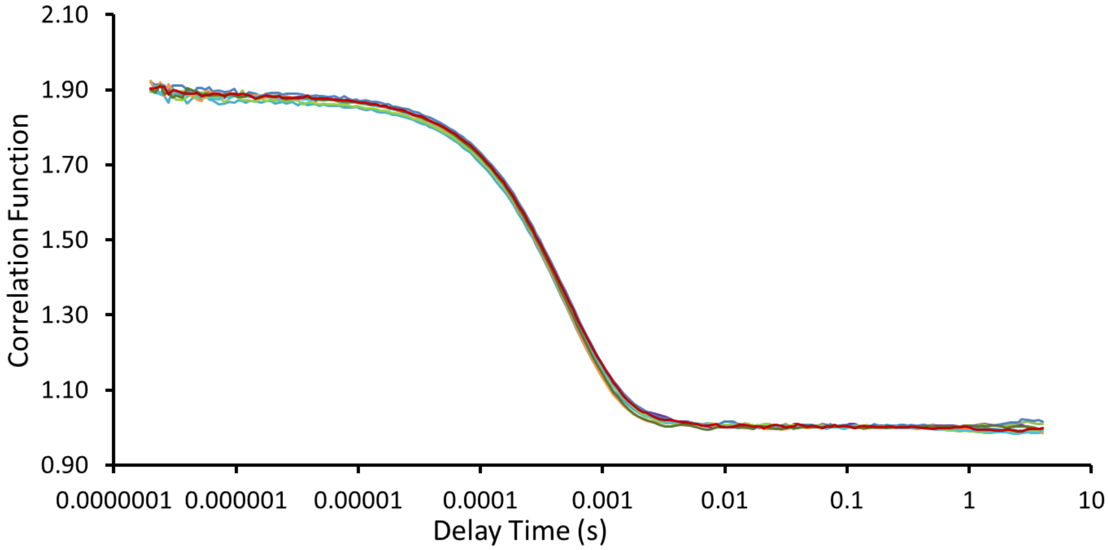

Figure S71 - Correlation function data for 9 DLS runs of compound **23** (0.56 mM) in an EtOH:H_2O (1:19) solution at 298 K.

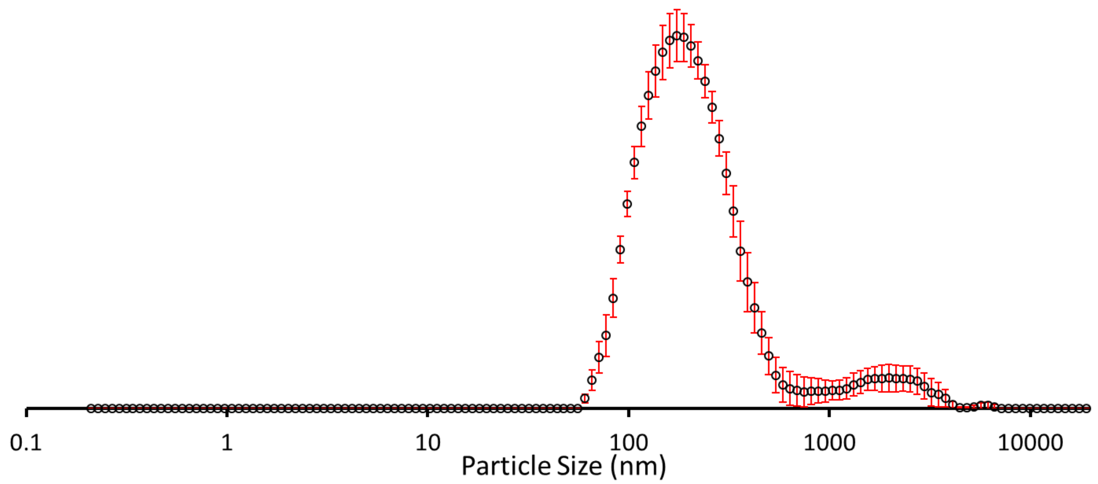

Figure S72 - The average intensity particle size distribution calculated using 9 DLS runs for compound **40** (5.56 mM) in an EtOH:H_2O (1:19) solution at 298 K.

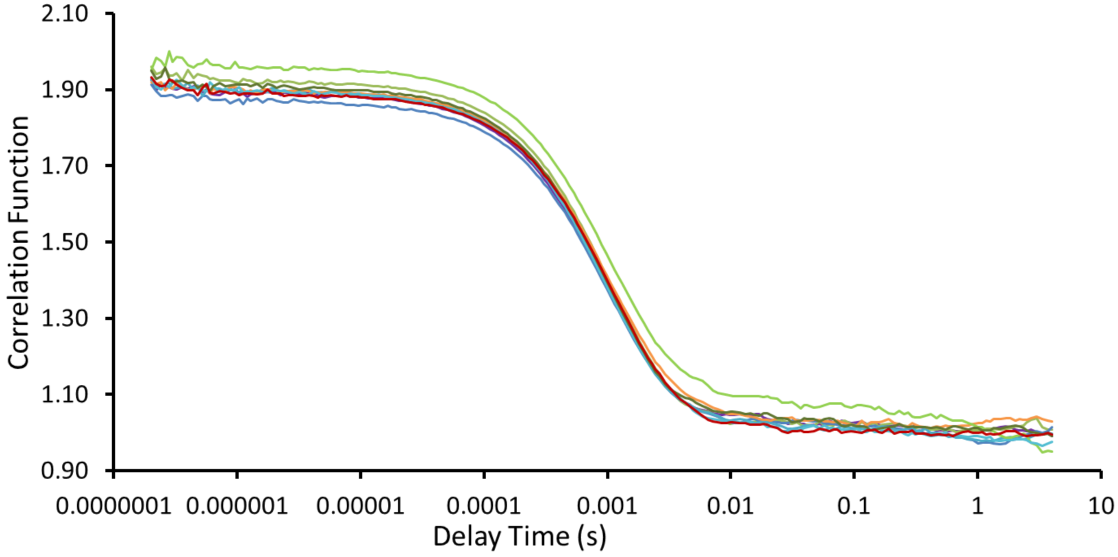

Figure S73 - Correlation function data for 9 DLS runs of compound **40** (5.56 mM) in an EtOH:H_2O (1:19) solution at 298 K.

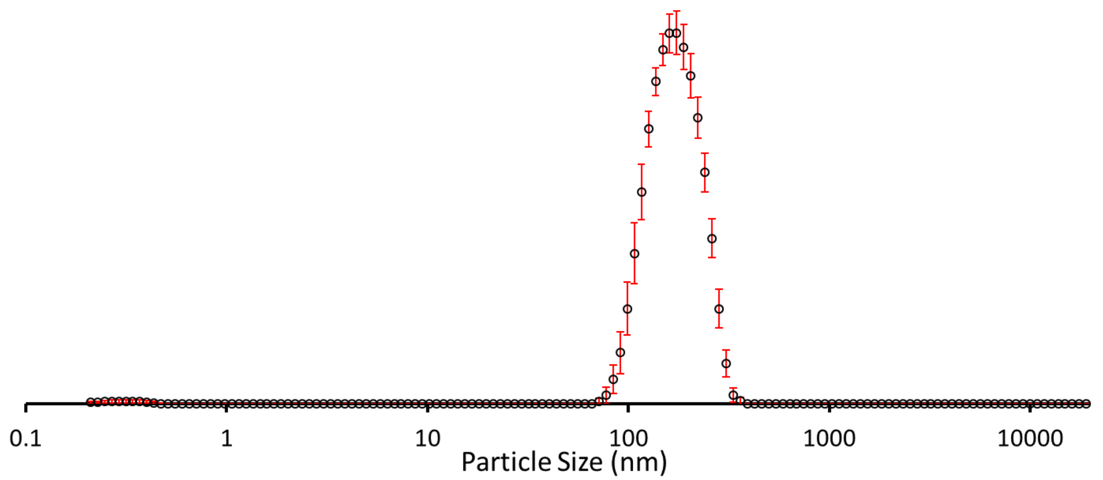

Figure S74 - The average intensity particle size distribution calculated using 9 DLS runs for compound **40** (0.56 mM) in an EtOH:H_2O (1:19) solution at 298 K.

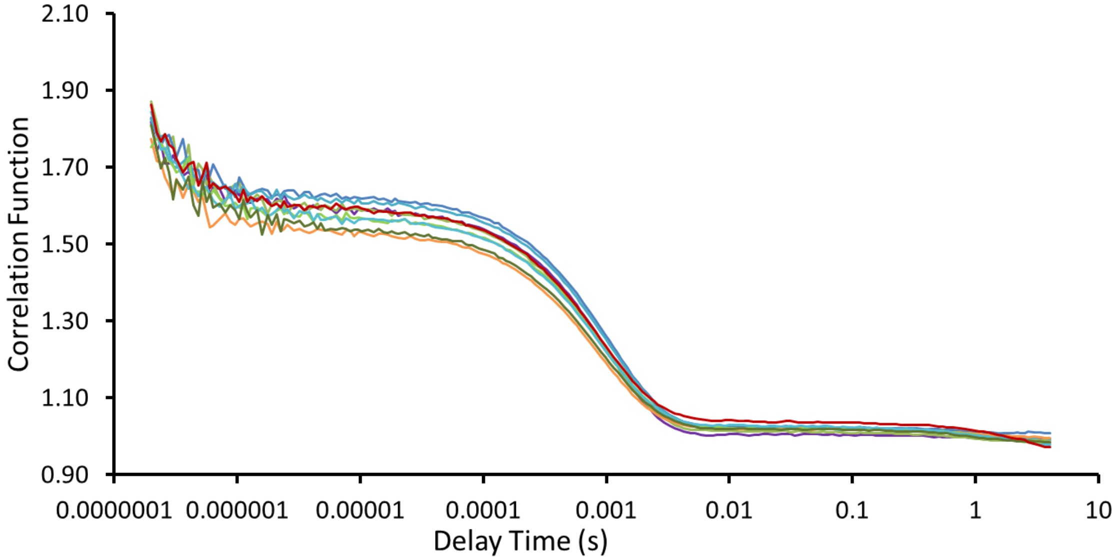

Figure S75 - Correlation function data for 9 DLS runs of compound **40** (0.56 mM) in an EtOH:H_2O (1:19) solution at 298 K.

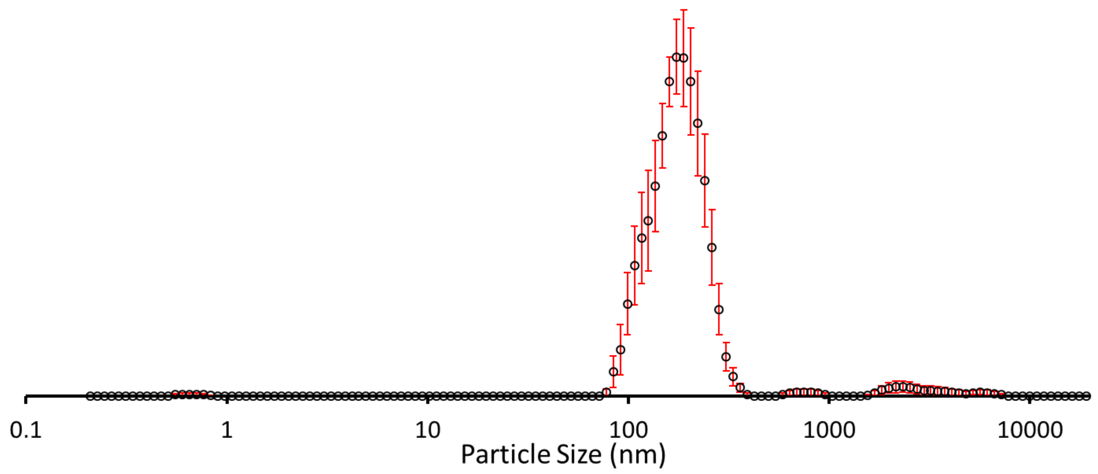

Figure S76 - The average intensity particle size distribution calculated using 9 DLS runs for compound **41** (5.56 mM) in an EtOH:H_2O (1:19) solution at 298 K.

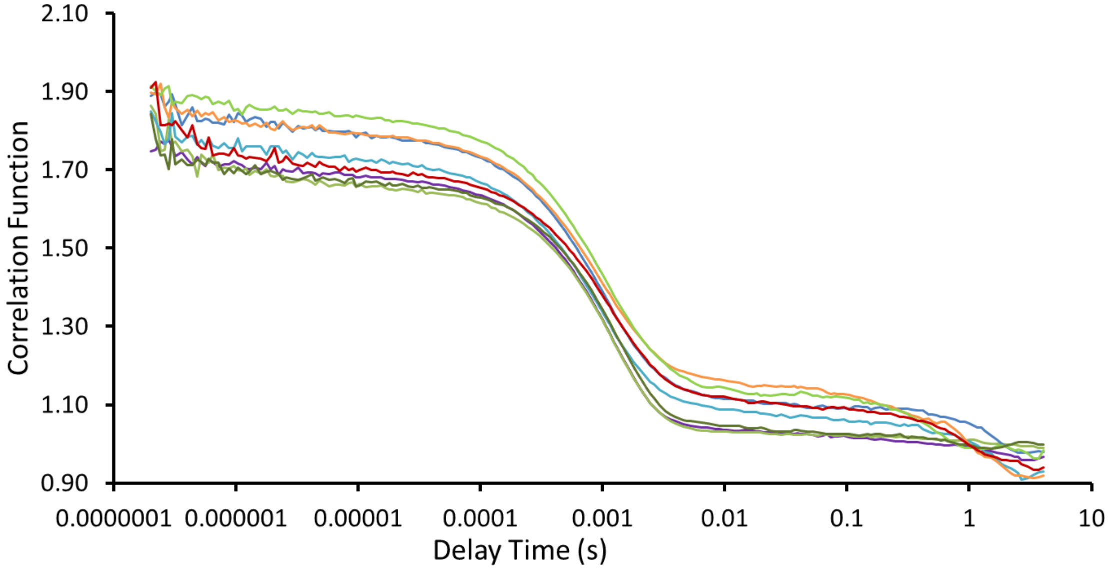

Figure S77 - Correlation function data for 9 DLS runs of compound **41** (5.56 mM) in an EtOH:H_2O (1:19) solution at 298 K.

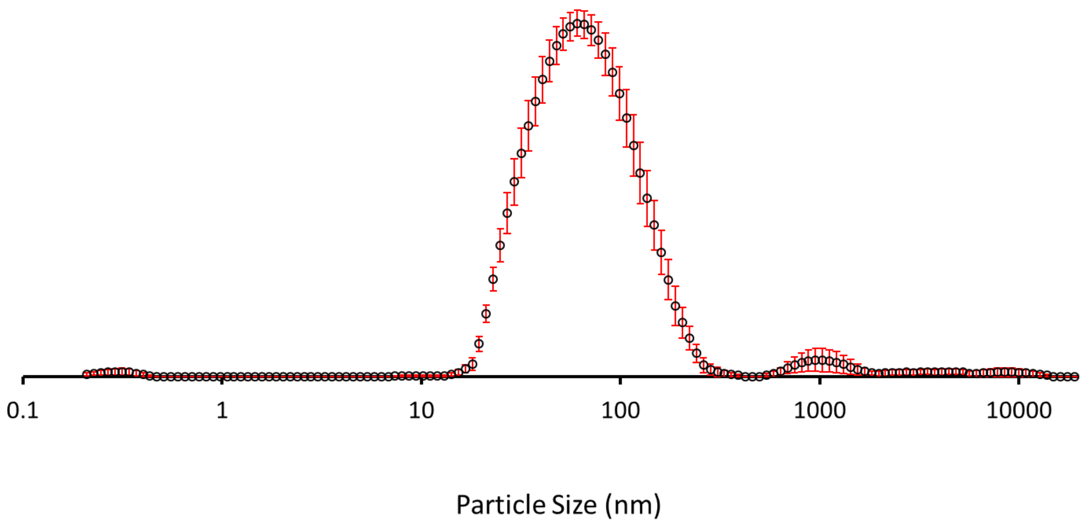

Figure S78 - The average intensity particle size distribution calculated using 9 DLS runs for compound **41** (0.56 mM) in an EtOH:H_2O (1:19) solution at 298 K.

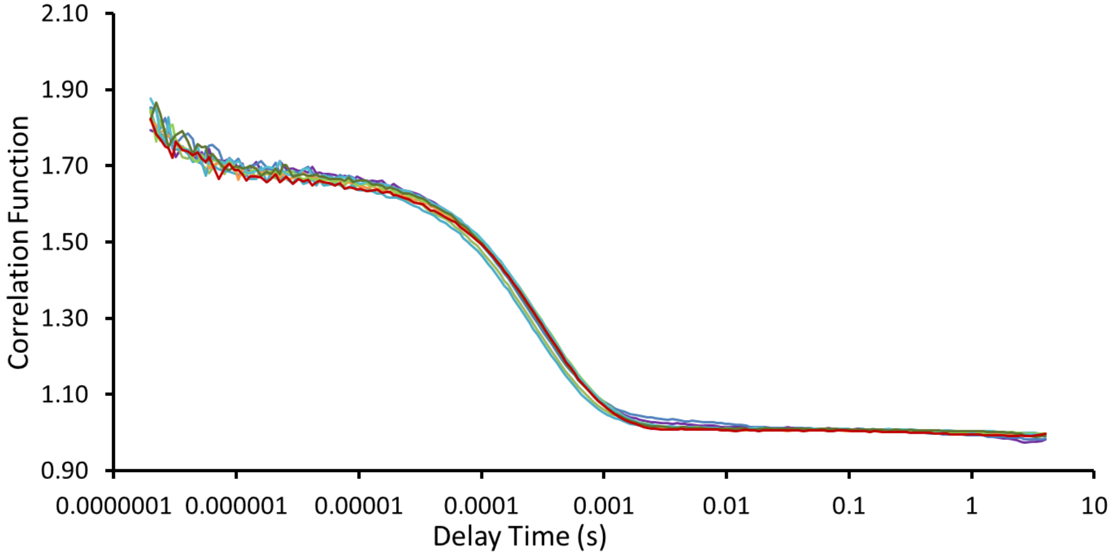

Figure S79 - Correlation function data for 9 DLS runs of compound **41** (0.56 mM) in an EtOH:H_2O (1:19) solution at 298 K.

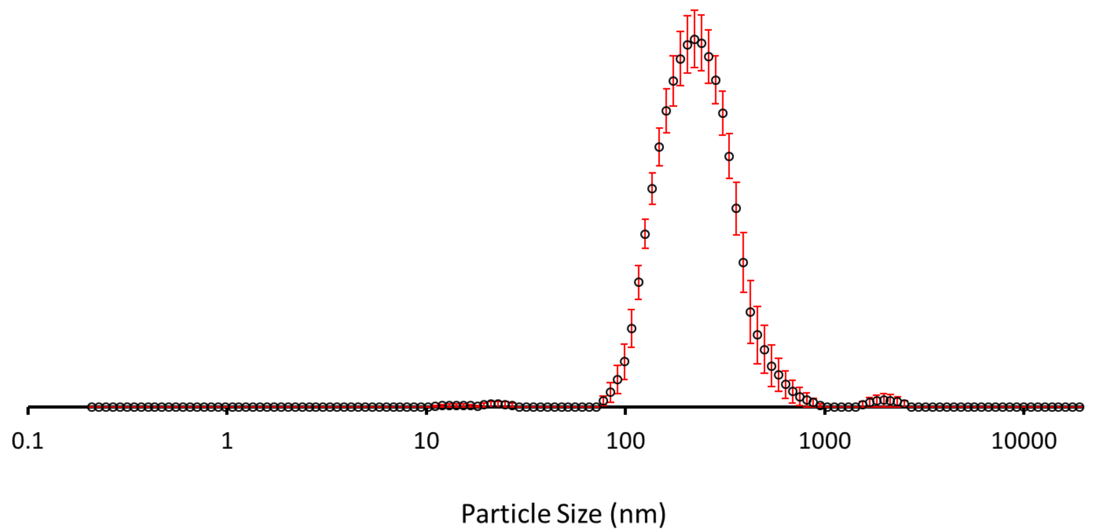

Figure S80 - The average intensity particle size distribution calculated using 9 DLS runs for compound **42** (5.56 mM) in an EtOH:H_2O (1:19) solution at 298 K.

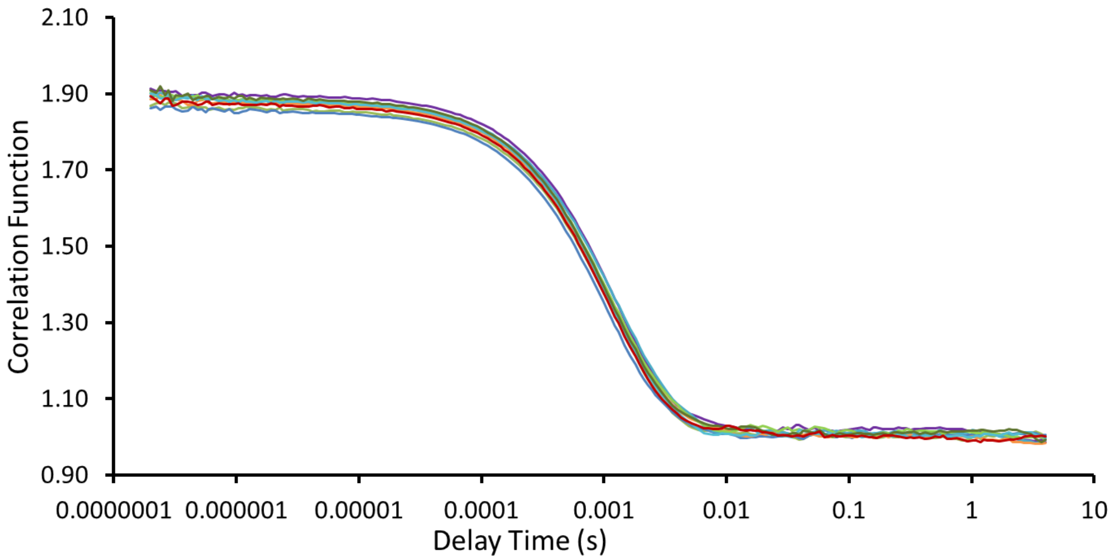

Figure S81 - Correlation function data for 9 DLS runs of compound **42** (5.56 mM) in an EtOH:H_2O (1:19) solution at 298 K.

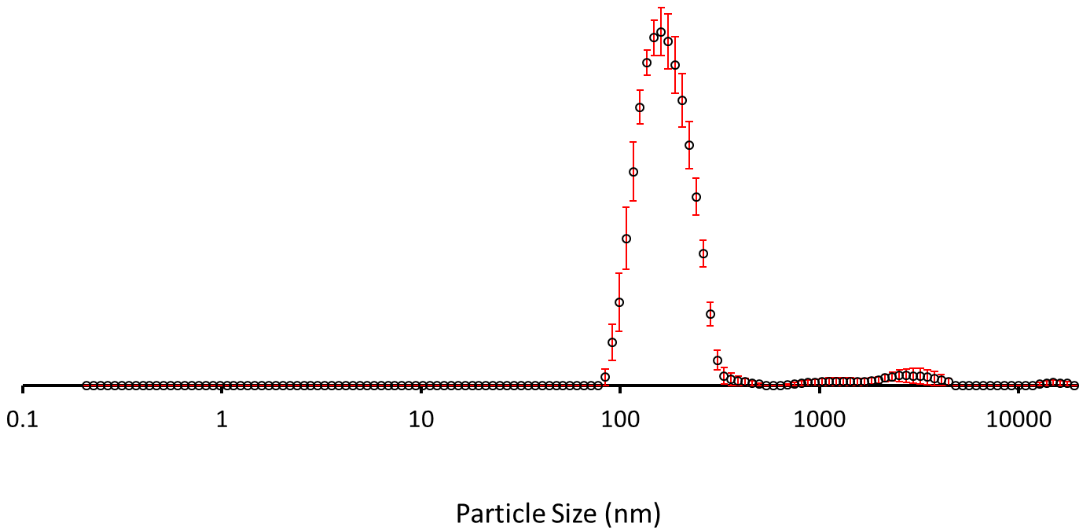

Figure S82 - The average intensity particle size distribution calculated using 9 DLS runs for compound **42** (0.56 mM) in an EtOH:H_2O (1:19) solution at 298 K.

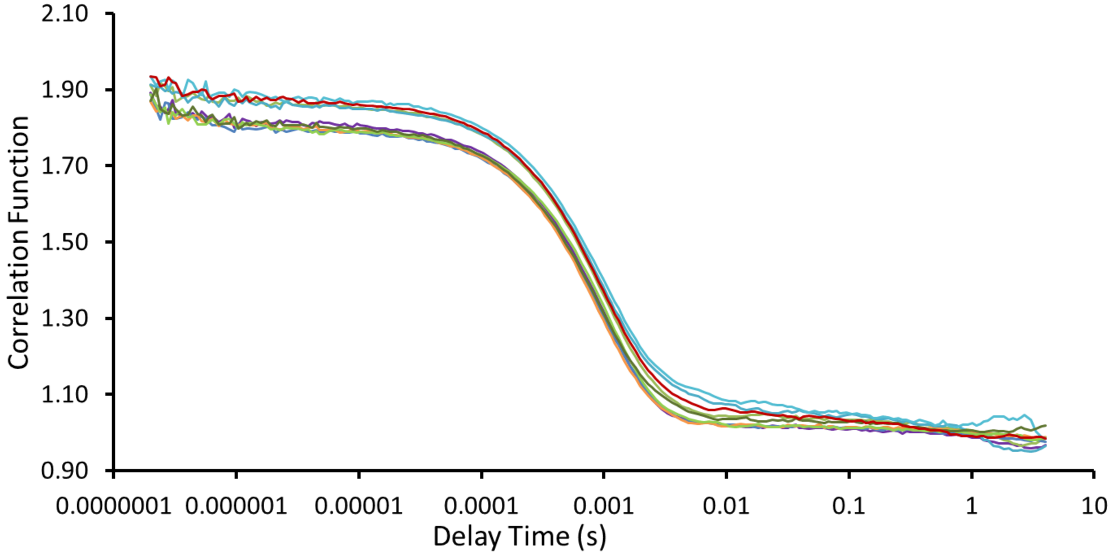

Figure S83 - Correlation function data for 9 DLS runs of compound **42** (0.56 mM) in an EtOH:H_2O (1:19) solution at 298 K.

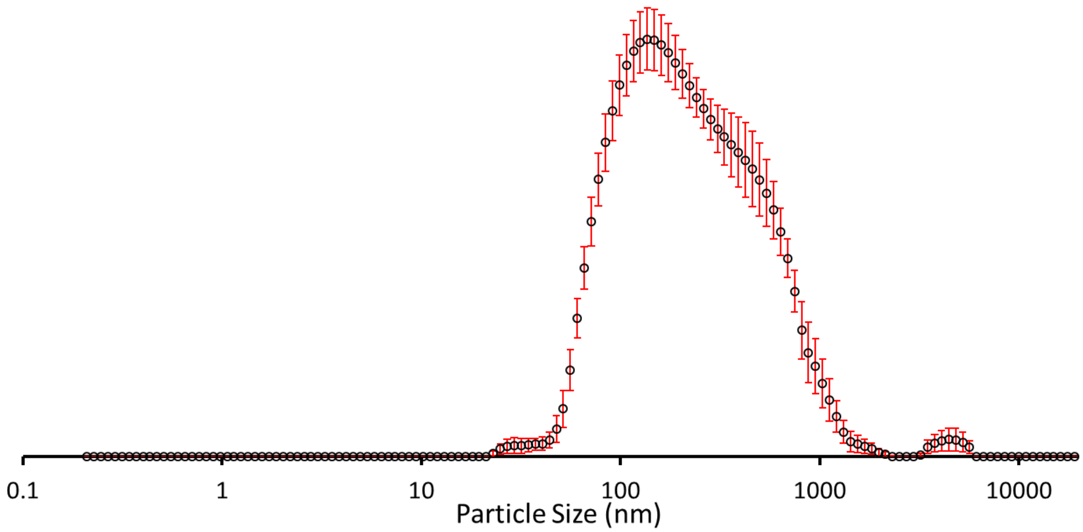

Figure S84 - The average intensity particle size distribution calculated using 9 DLS runs for compound **43** (5.56 mM) in an EtOH:H_2O (1:19) solution at 298 K.

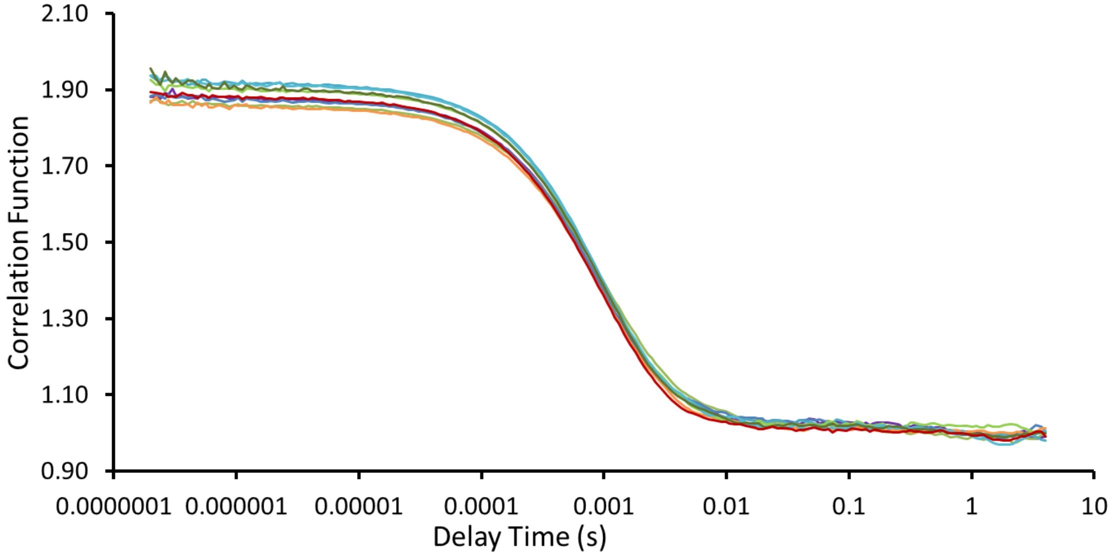

Figure S85 - Correlation function data for 9 DLS runs of compound **43** (5.56 mM) in an EtOH:H_2O (1:19) solution at 298 K.

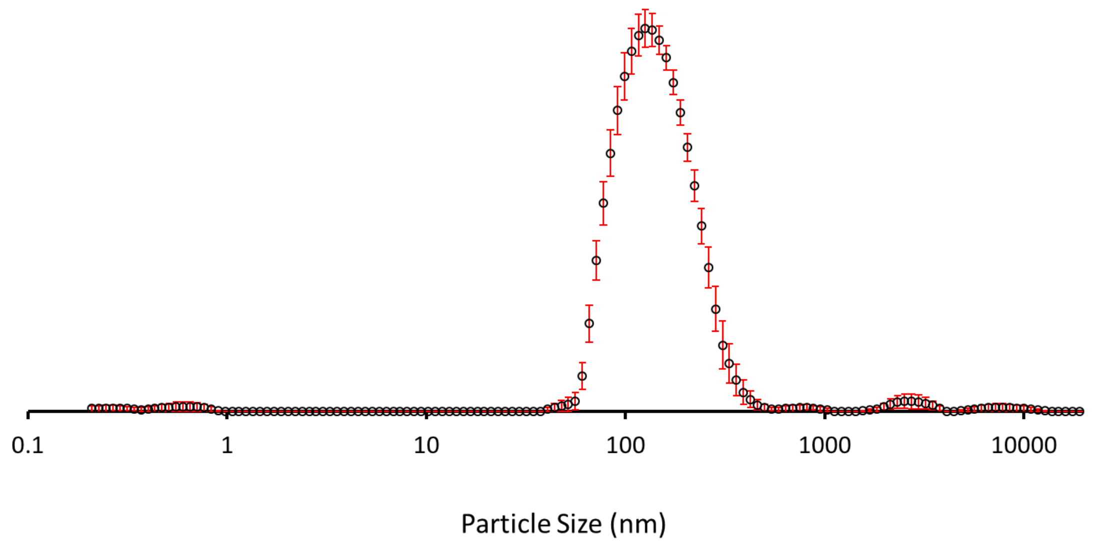

Figure S86 - The average intensity particle size distribution calculated using 9 DLS runs for compound **43** (0.56 mM) in an EtOH:H_2O (1:19) solution at 298 K.

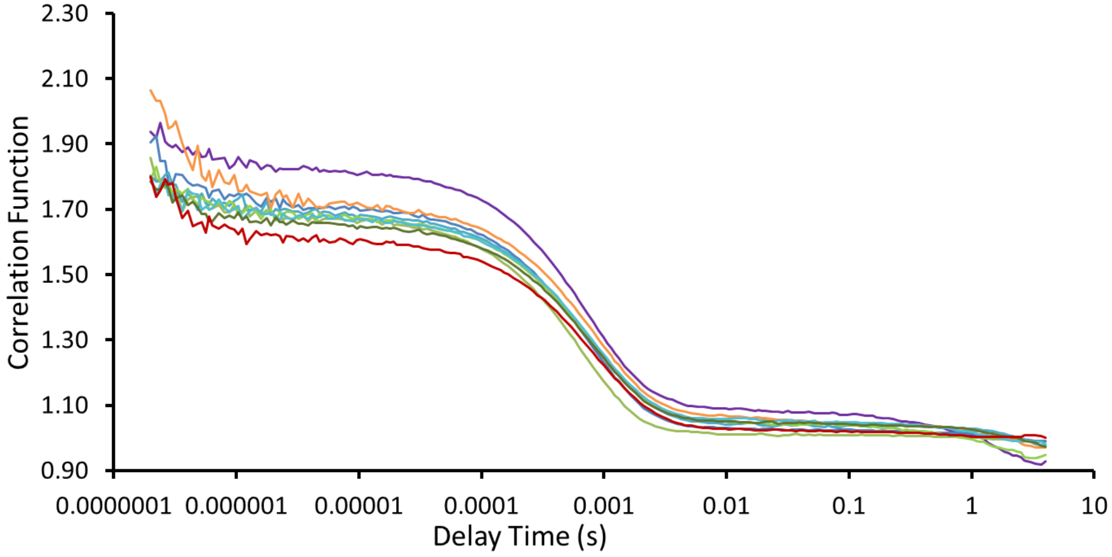

Figure S87 - Correlation function data for 9 DLS runs of compound **43** (0.56 mM) in an EtOH:H_2O (1:19) solution at 298 K.

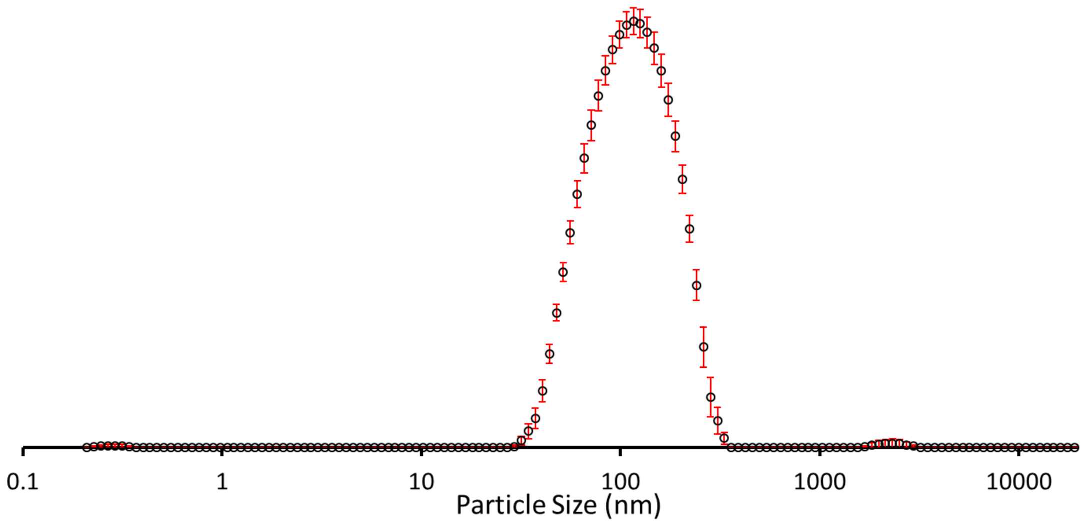

Figure S88 - The average intensity particle size distribution calculated using 9 DLS runs for compound **44** (5.56 mM) in an EtOH:H_2O (1:19) solution at 298 K.

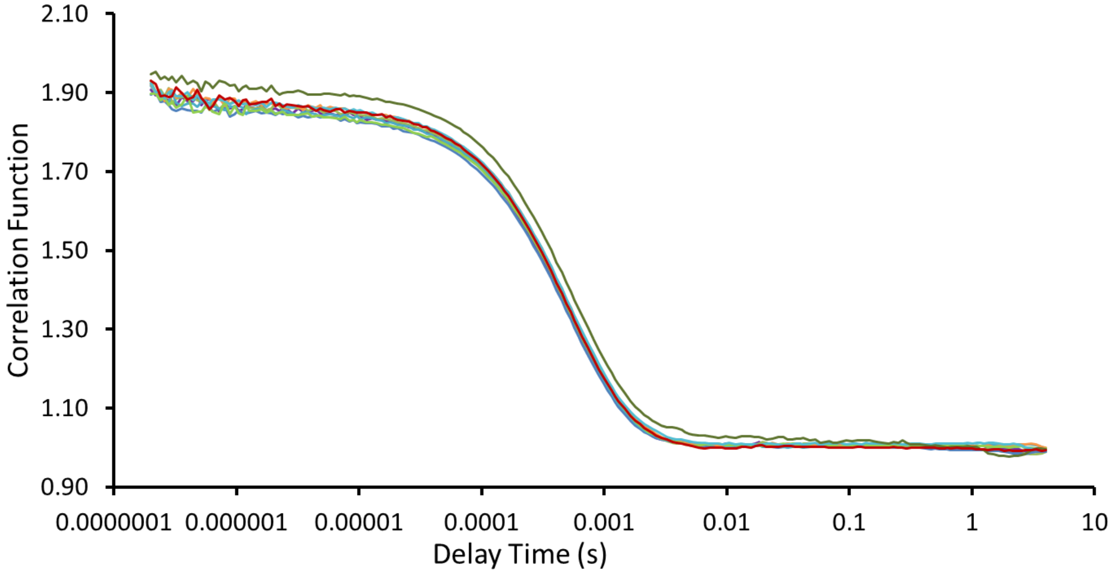

Figure S89 - Correlation function data for 9 DLS runs of compound **44** (5.56 mM) in an EtOH:H_2O (1:19) solution at 298 K.

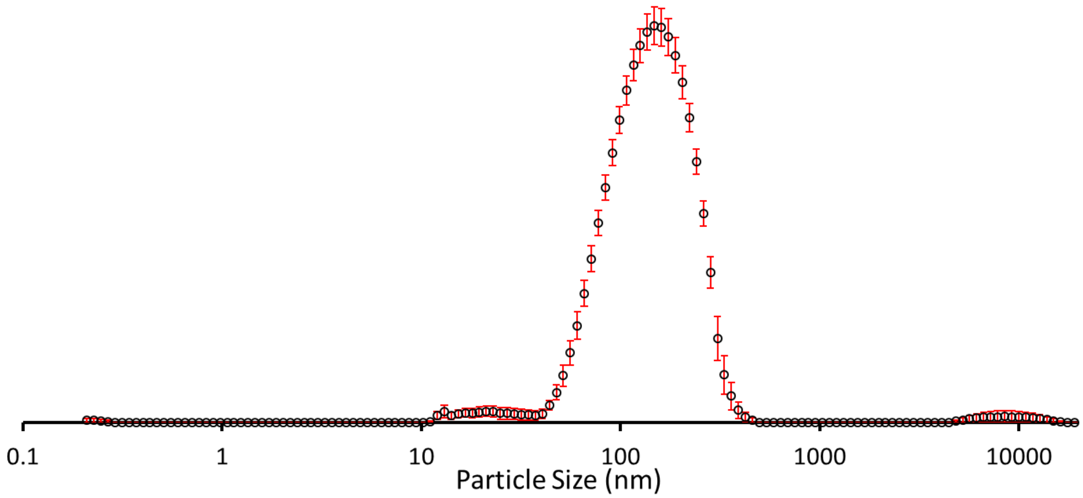

Figure S90 - The average intensity particle size distribution calculated using 9 DLS runs for compound **45** (5.56 mM) in an EtOH:H_2O (1:19) solution at 298 K.

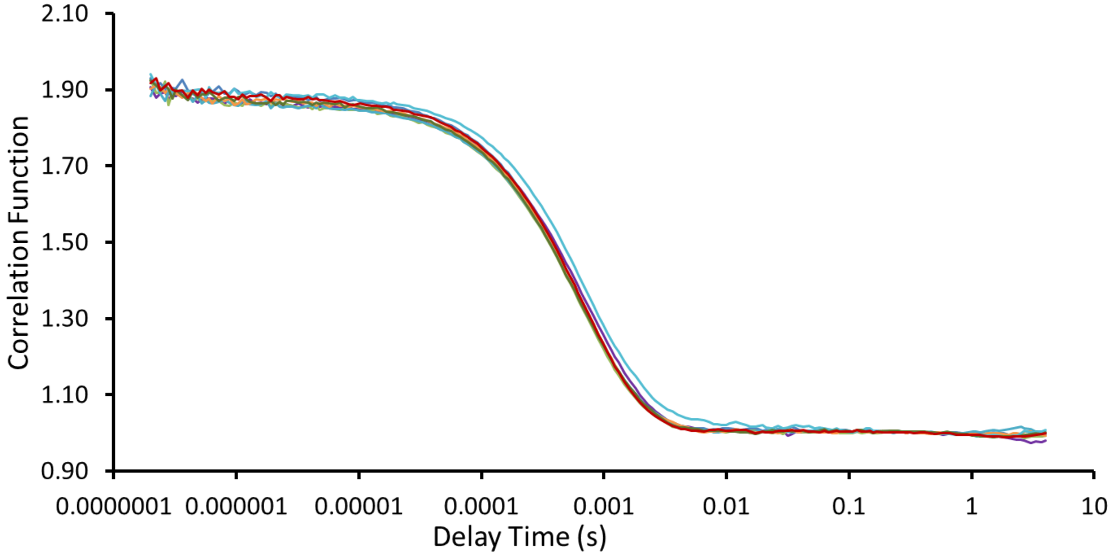

Figure S91 - Correlation function data for 9 DLS runs of compound **45** (5.56 mM) in an EtOH:H_2O (1:19) solution at 298 K.

Overview

Table S6 - Summary of average intensity particle size distribution data. Error = standard error of the mean.

H_2O: EtOH 19:1 Compound	Concentration (mM)	Peak maxima (nm)	Polydispersity (%)
**15**	5.56	235 (± 23.5219)	27 (± 4.4913)
	0.56	*a*	*a*
**16**	5.56	*a*	*a*
	0.56	*a*	*a*
**17**	5.56	160 (± 8.6276)	24 (± 1.1093)
	0.56	*a*	*a*
**18**	5.56	192 (± 6.0362)	21 (± 2.2897)
	0.56	*a*	*a*
**23**	5.56	182 (±2.8533)	24 (± 0.2718)
	0.56	106 (± 2.5418)	22 (± 0.8599)
**40**	5.56	208 (± 13.824)	26 (± 0.9197)
	0.56	169 (± 2.0725)	14 (± 2.0720)
**41**	5.56	174 (± 7.5417)	21 (± 2.3077)
	0.56	74 (± 3.5606)	24 (± 0.3690)
**42**	5.56	248 (± 0.0251)	24 (± 1.4597)
	0.56	173 (± 3.3980)	21 (± 1.2968)
**43**	5.56	220 (± 28.6332)	28 (± 0.5362)
	0.56	145 (± 7.6919)	23 (± 0.9277)
**44**	5.56	121 (± 1.9221)	24 (± 0.44824
	0.56	*a*	*a*
**45**	5.56	153 (± 2.1280)	24 (± 0.5609)
	0.56	*a*	*a*
**50**	5.56	*a*	*a*
	0.56	*a*	*a*

a - Reproducible correlation functions could not be obtained.

Surface Tension and Stability Data

Zeta Potential

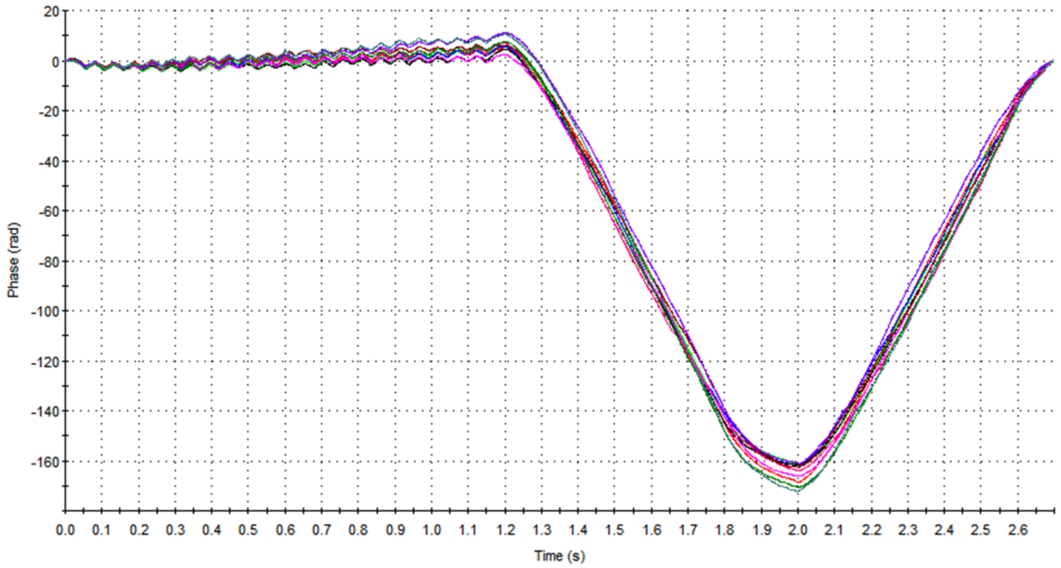

Figure S92 - Phase plot taken during the measurement of 10 Zeta Potential Transfer Standard (PTS) runs of compound **15** at a concentration of 5.56 mM in a solution EtOH: H_2O 1:19. Average measurement value **-**21.3 mV.

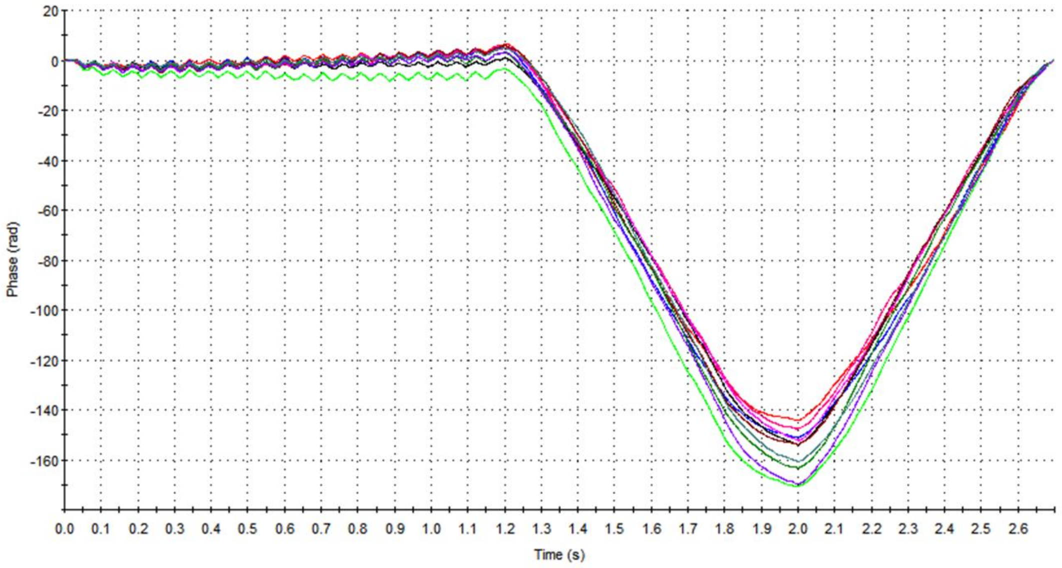

Figure S93 - Phase plot taken during the measurement of 10 Zeta Potential Transfer Standard (PTS) runs of compound **16** at a concentration of 5.56 mM in a solution EtOH:H_2O 1:19. Average measurement value **-**18.9 mV.

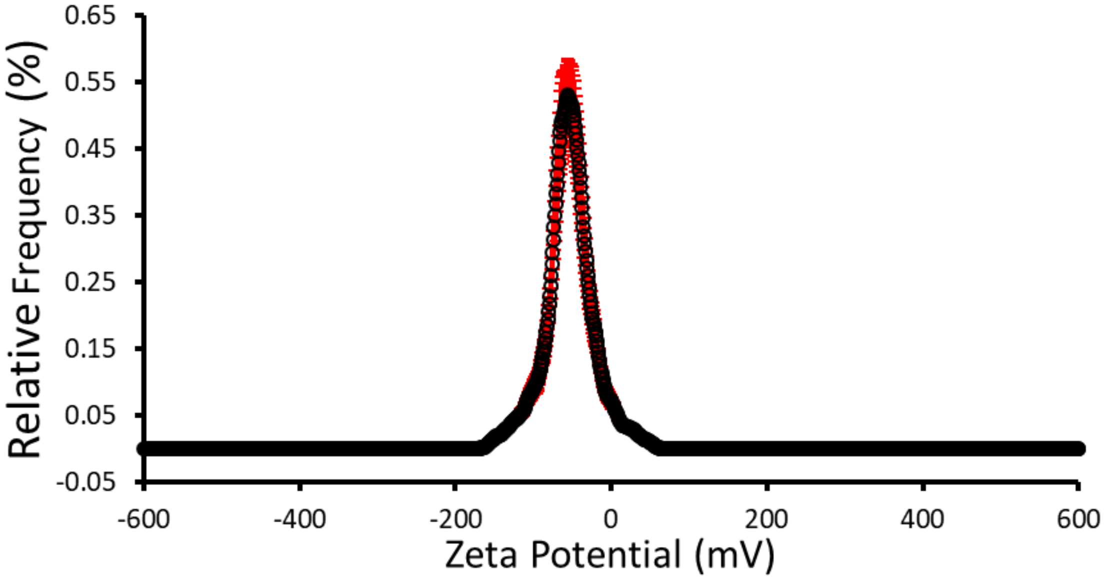

Figure S94 - The average zeta potential distribution calculated using 10 runs for compound **23** (5.56 mM) in an EtOH:H_2O (1:19) solution at 298 K. Average measurement value **-**57.2 mV.

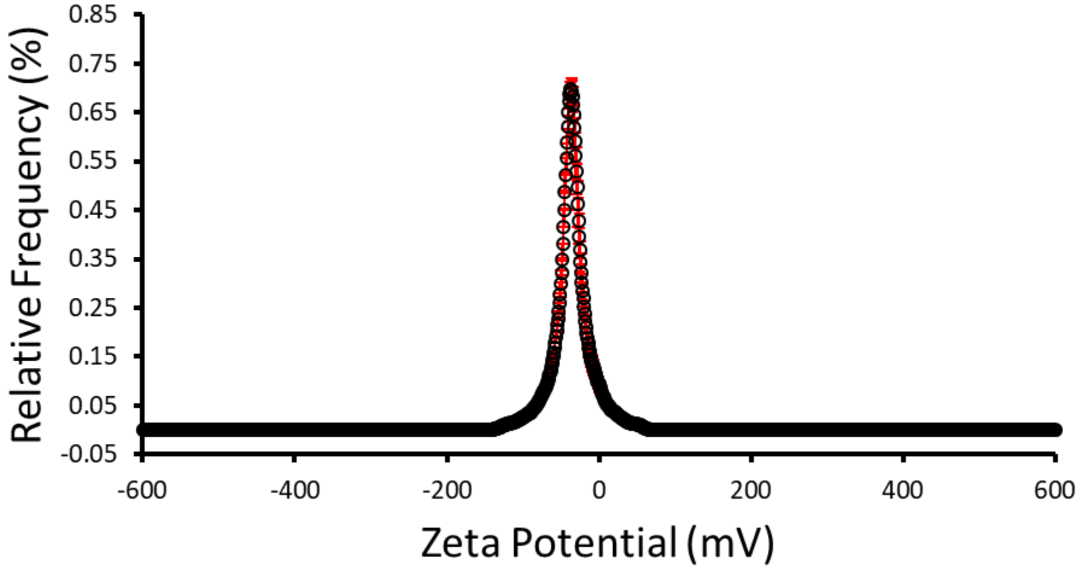

Figure S95 - The average zeta potential distribution calculated using 10 runs for compound **40** (5.56 mM) in an EtOH:H_2O (1:19) solution at 298 K. Average measurement value **-**38.3 mV.

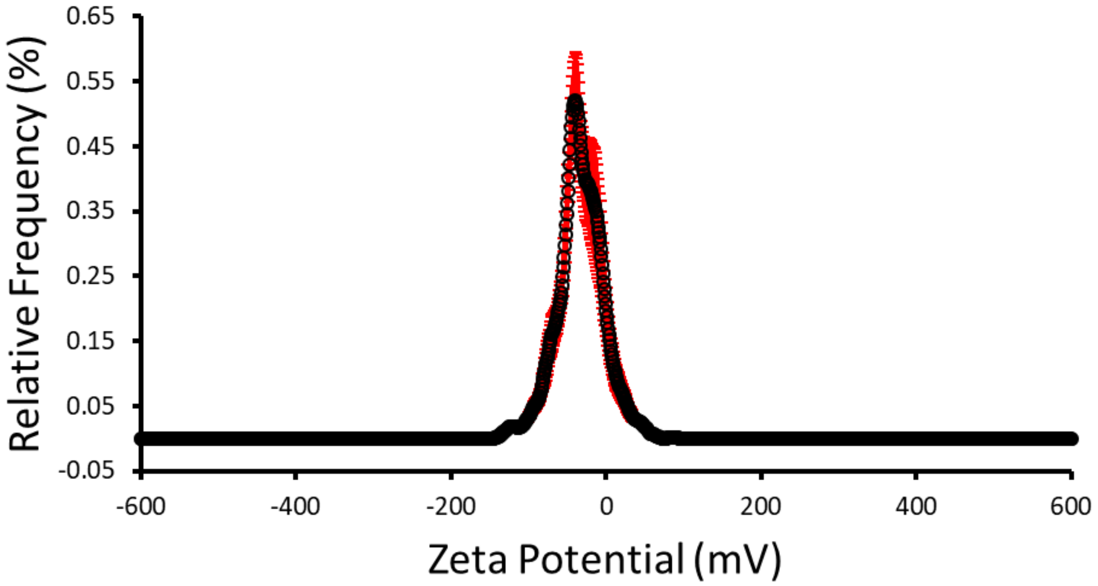

Figure S96 - The average zeta potential distribution calculated using 10 runs for compound **41** (5.56 mM) in an EtOH:H_2O (1:19) solution at 298 K. Average measurement value **-**27.6 mV.

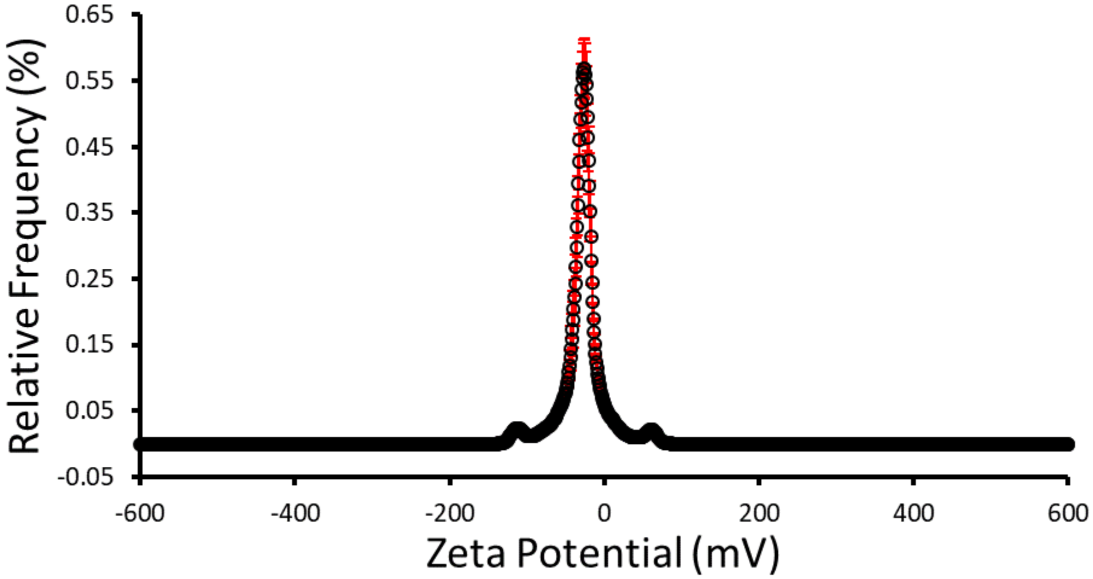

Figure S97 - The average zeta potential distribution calculated using 10 runs for compound **42** (5.56 mM) in an EtOH:H_2O (1:19) solution at 298 K. Average measurement value **-**29.4 mV.

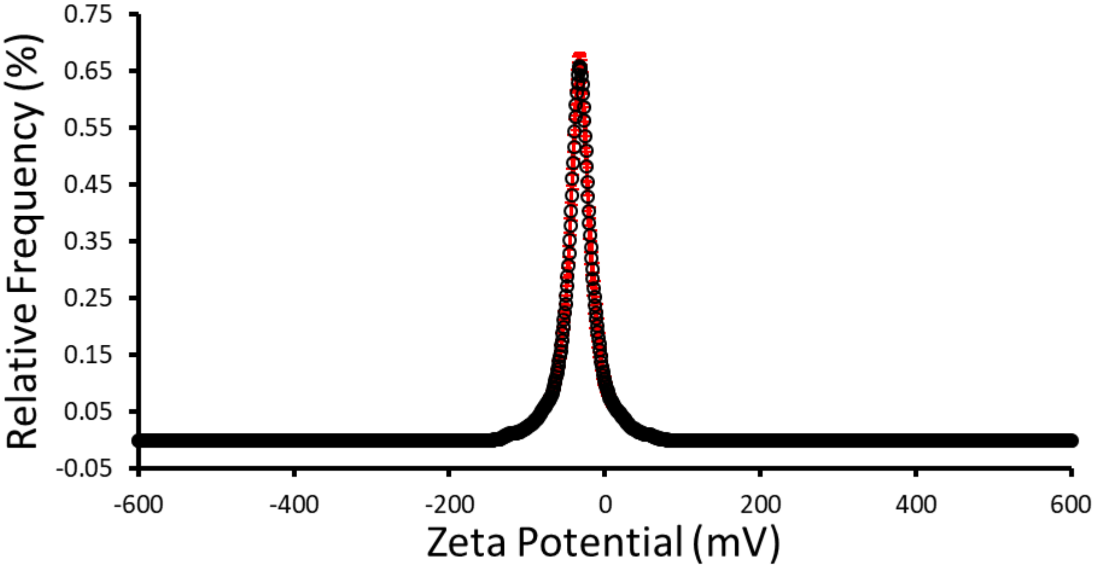

Figure S98 - The average zeta potential distribution calculated using 10 runs for compound **43** (5.56 mM) in an EtOH:H_2O (1:19) solution at 298 K. Average measurement value **-**37.4 mV.

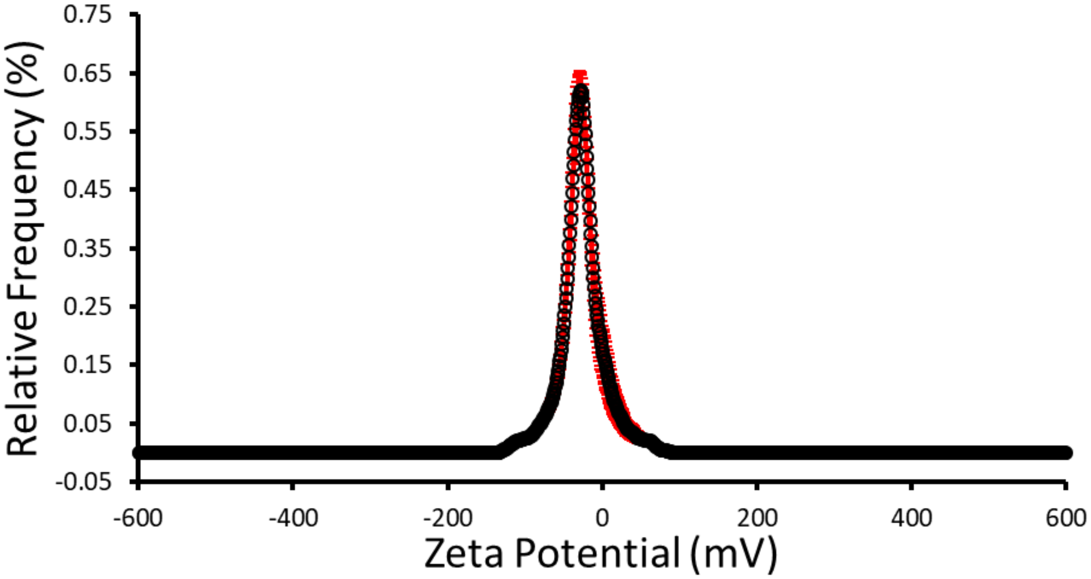

Figure S99 - The average zeta potential distribution calculated using 10 runs for compound **44** (5.56 mM) in an EtOH:H_2O (1:19) solution at 298 K. Average measurement value **-**30.8 mV.

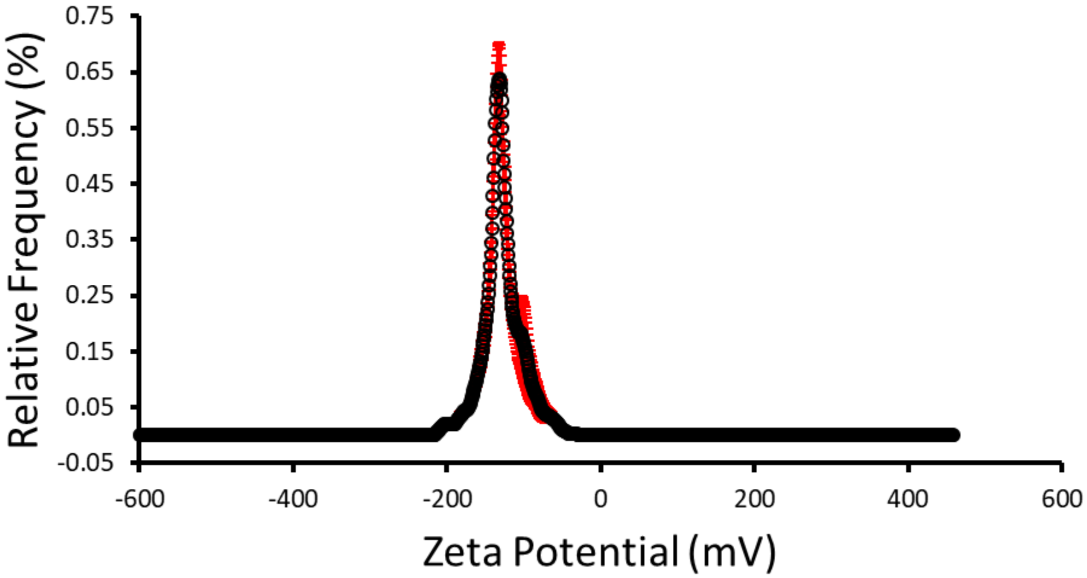

Figure S100 - The average zeta potential distribution calculated using 9 runs for compound **45** (5.56 mM) in an EtOH:H_2O (1:19) solution at 298 K. Average measurement value **-**35.3 mV.

Surface Tension Measurements and Critical Micelle Concentration (CMC) Determination

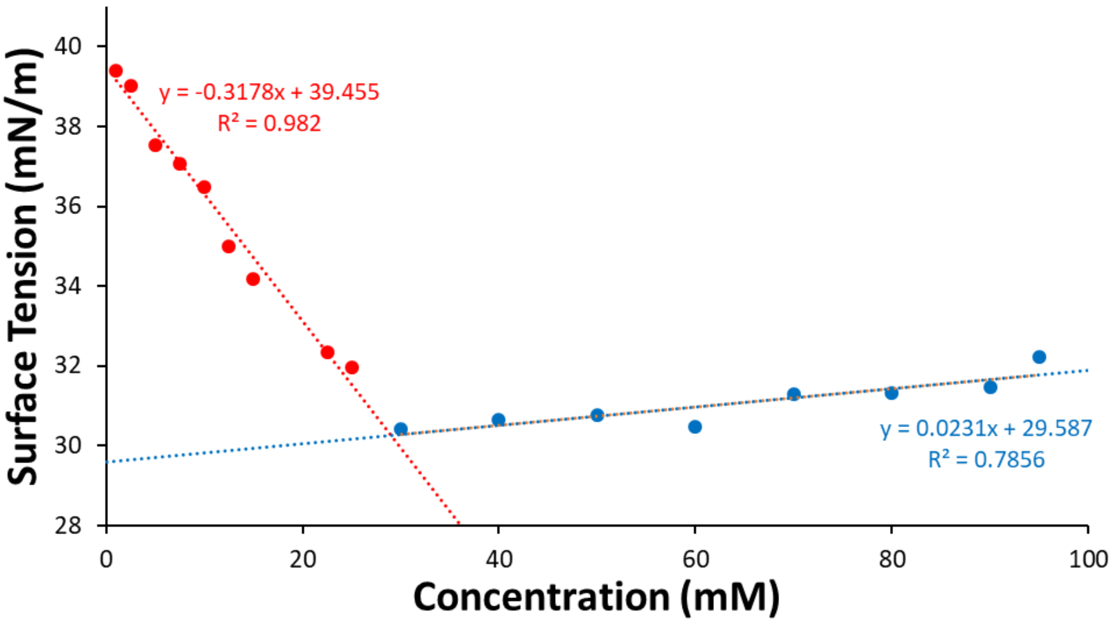

Figure S101 - Calculation of CMC (28.95 mM) for compound **15** in an EtOH: H2O 1:19 mixture using surface tension measurements.

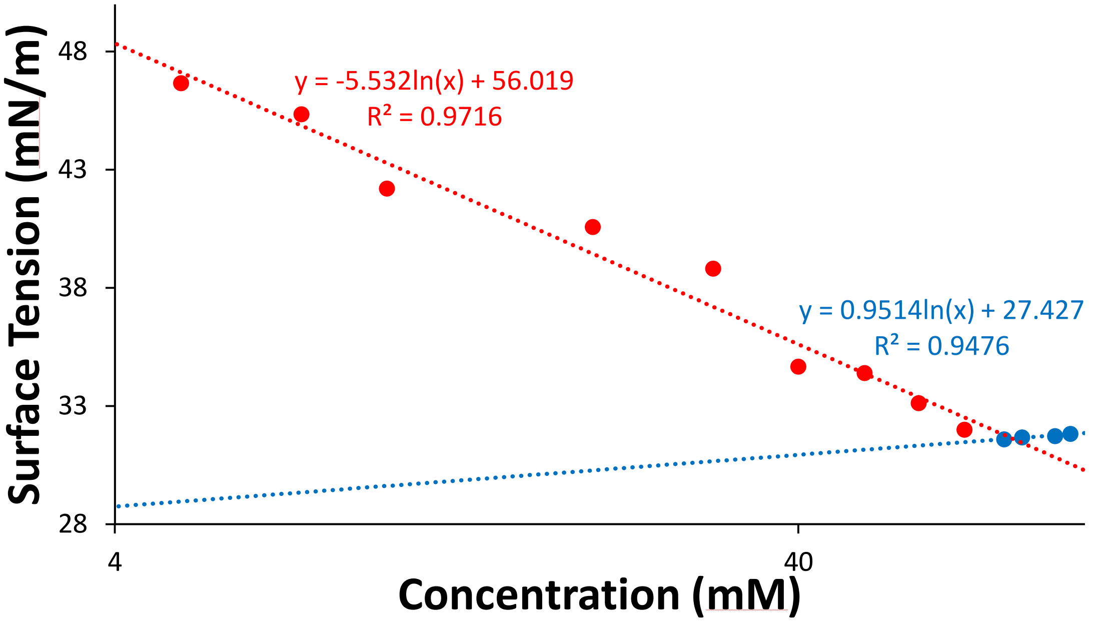

Figure S102 - Calculation of CMC (82.27 mM) for compound **16** in an EtOH: H2O 1:19 mixture using surface tension measurements.

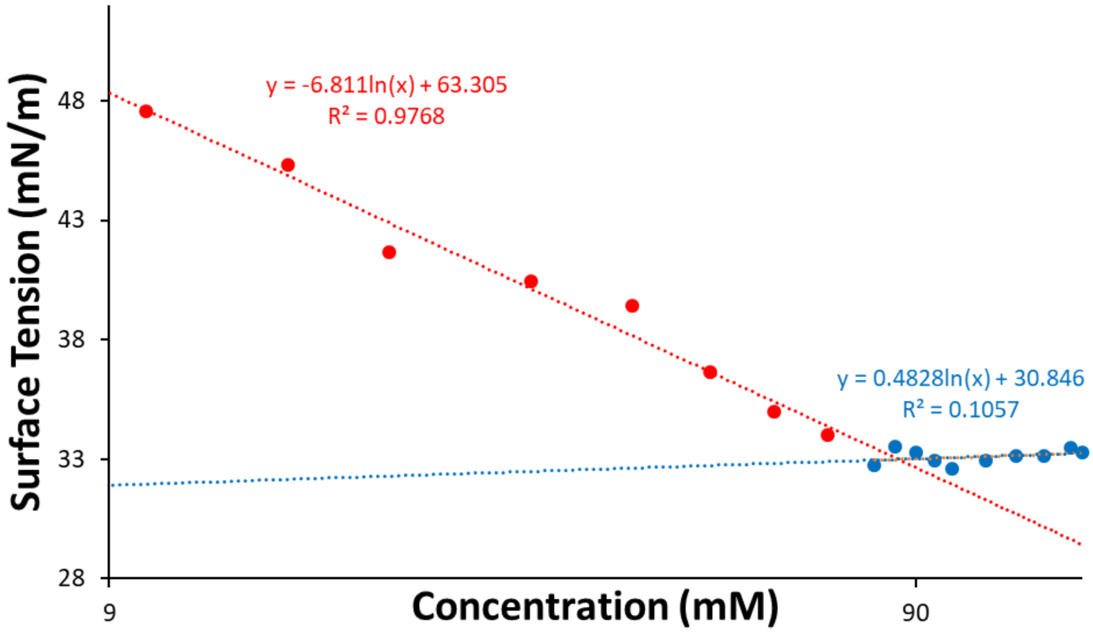

Figure S103 - Calculation of CMC (85.65 mM) for compound **17** in an EtOH: H2O 1:19 mixture using surface tension measurements.

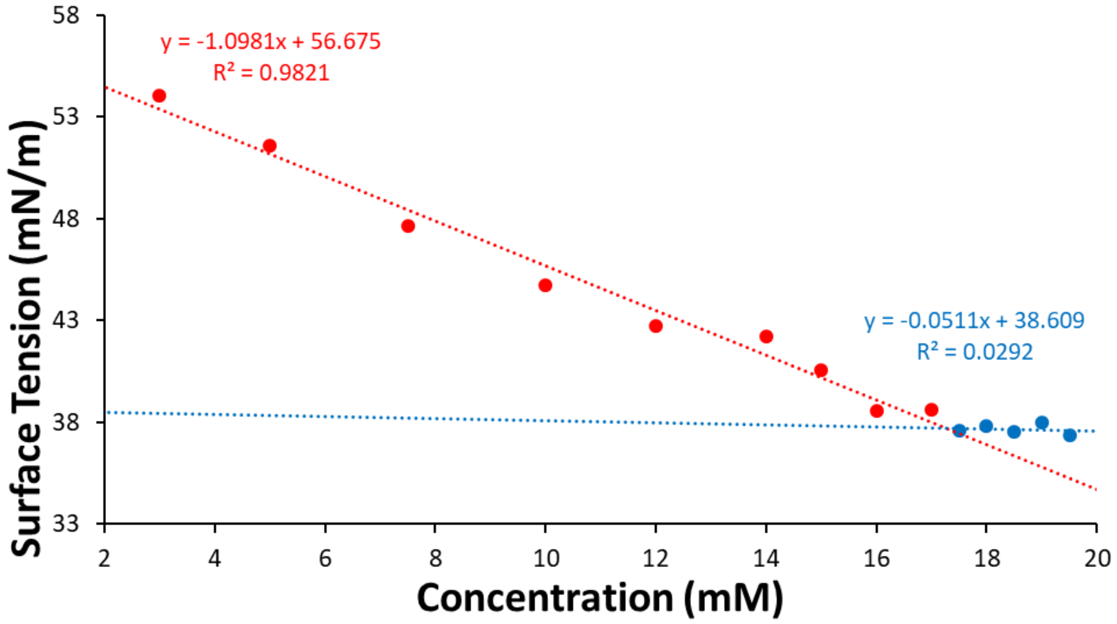

Figure S104 - Calculation of CMC (17.25 mM) for compound **18** in an EtOH: H2O 1:19 mixture using surface tension measurements.

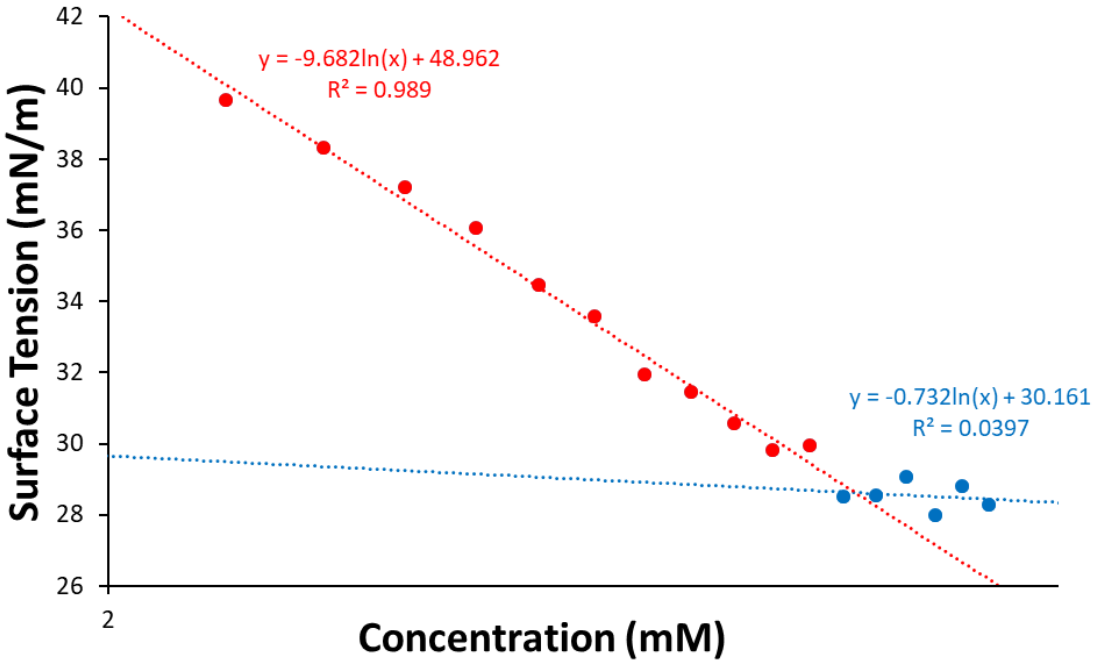

Figure S105 - Calculation of CMC (3.69 mM) for compound **23** in an EtOH: H2O 1:19 mixture using surface tension measurements.

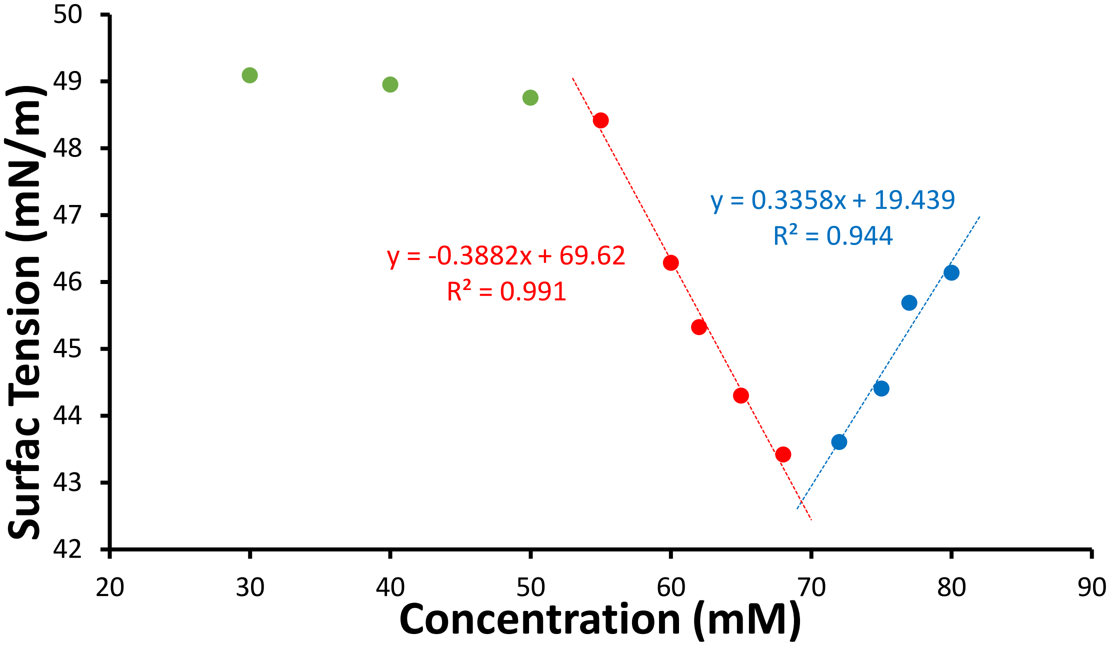

Figure S106 - Calculation of CMC (69.73 mM) for compound **40** in an EtOH: H2O 1:19 mixture using surface tension measurements.

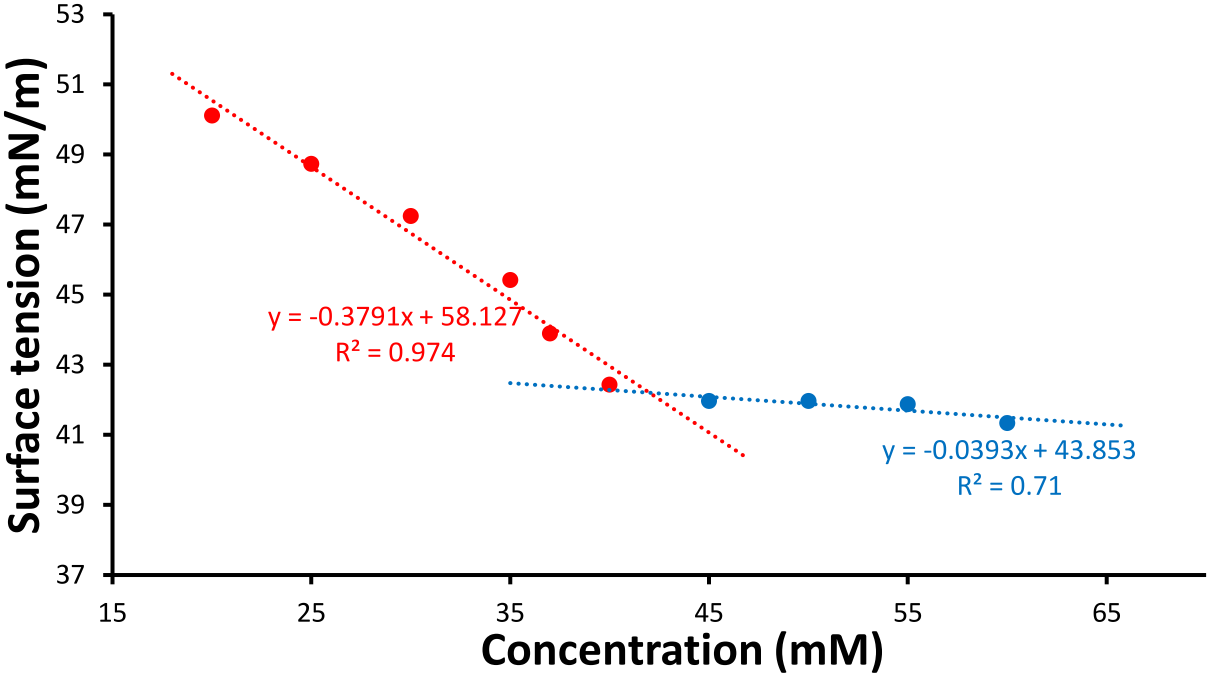

Figure S107 - Calculation of CMC (44.91 mM) for compound **41** in an EtOH: H2O 1:19 mixture using surface tension measurements.

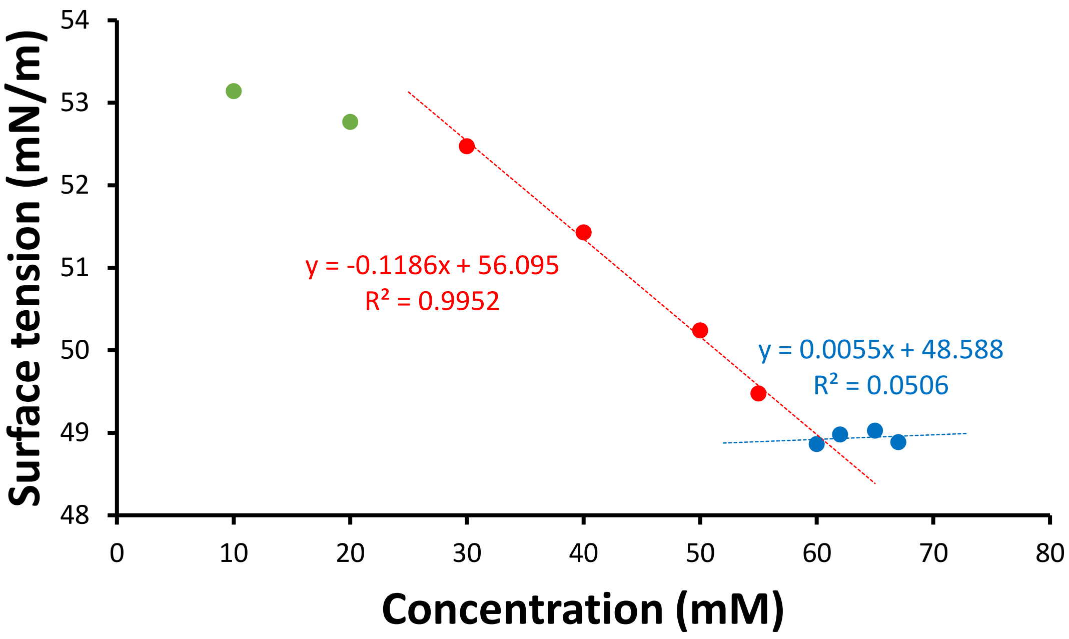

Figure S108 - Calculation of CMC (60.49 mM) for compound **42** in an EtOH: H2O 1:19 mixture using surface tension measurements.

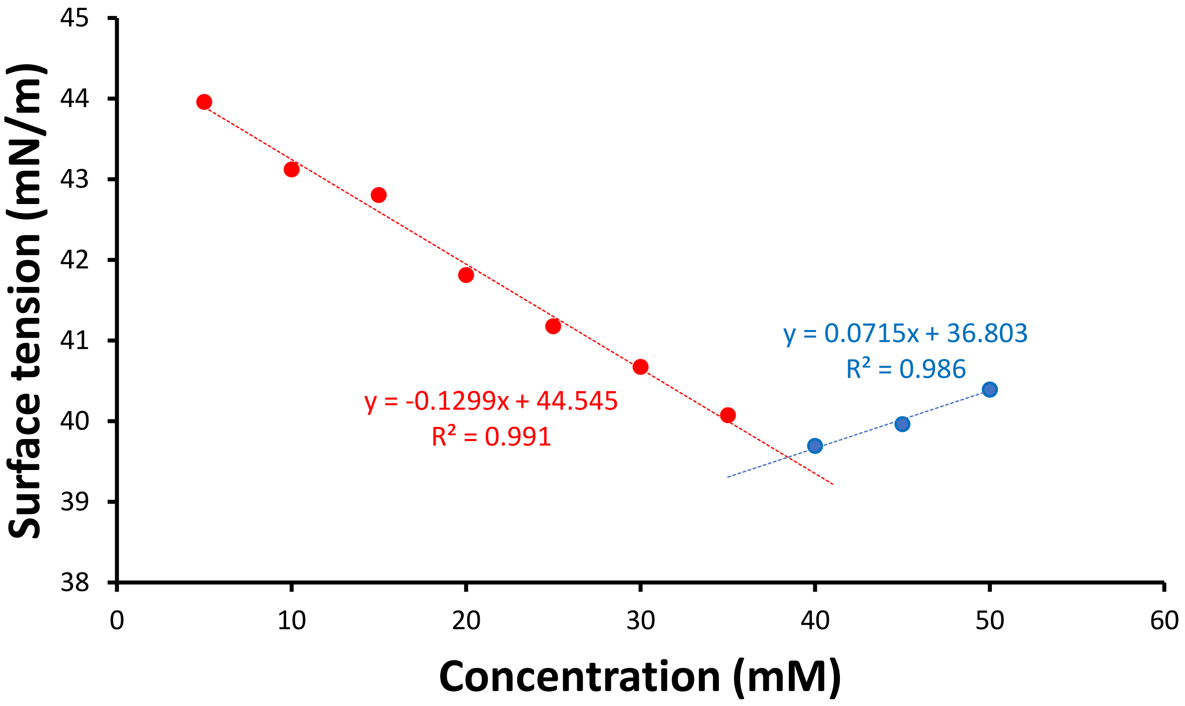

Figure S109 - Calculation of CMC (38.44 mM) for compound **43** in an EtOH: H2O 1:19 mixture using surface tension measurements.

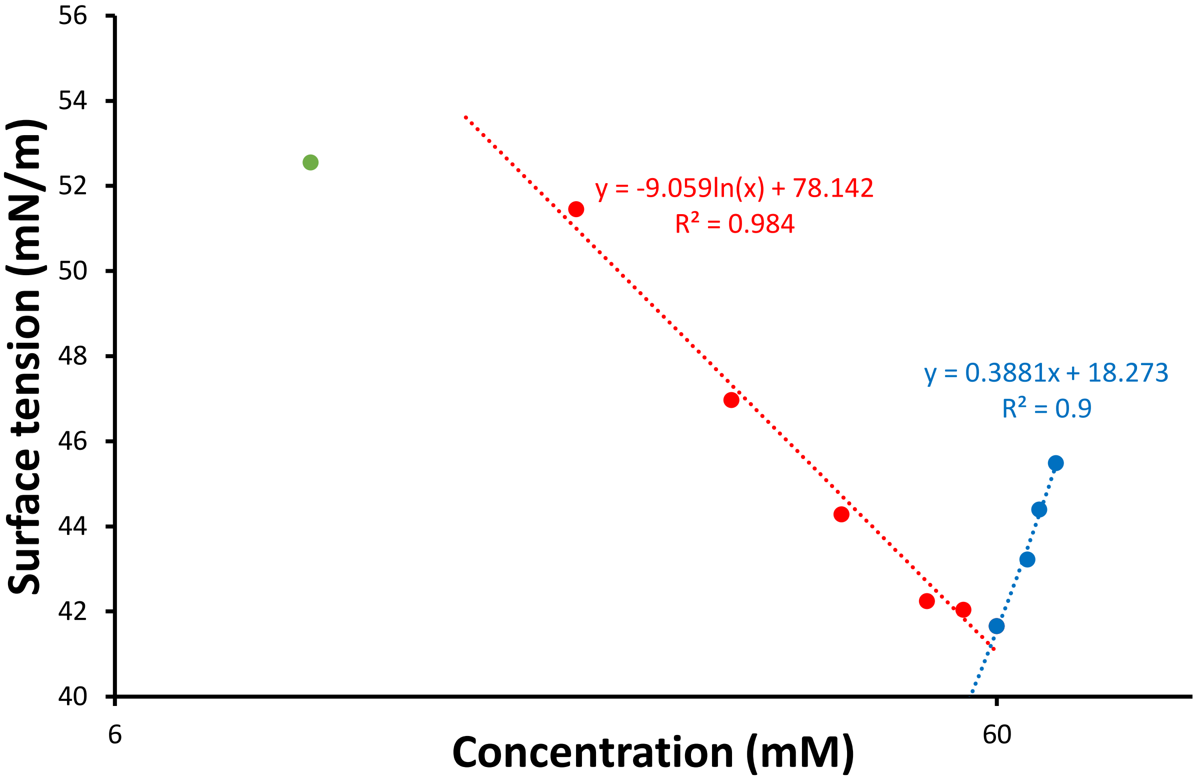

Figure S110 - Calculation of CMC (59.64 mM) for compound **44** in an EtOH: H2O 1:19 mixture using surface tension measurements.

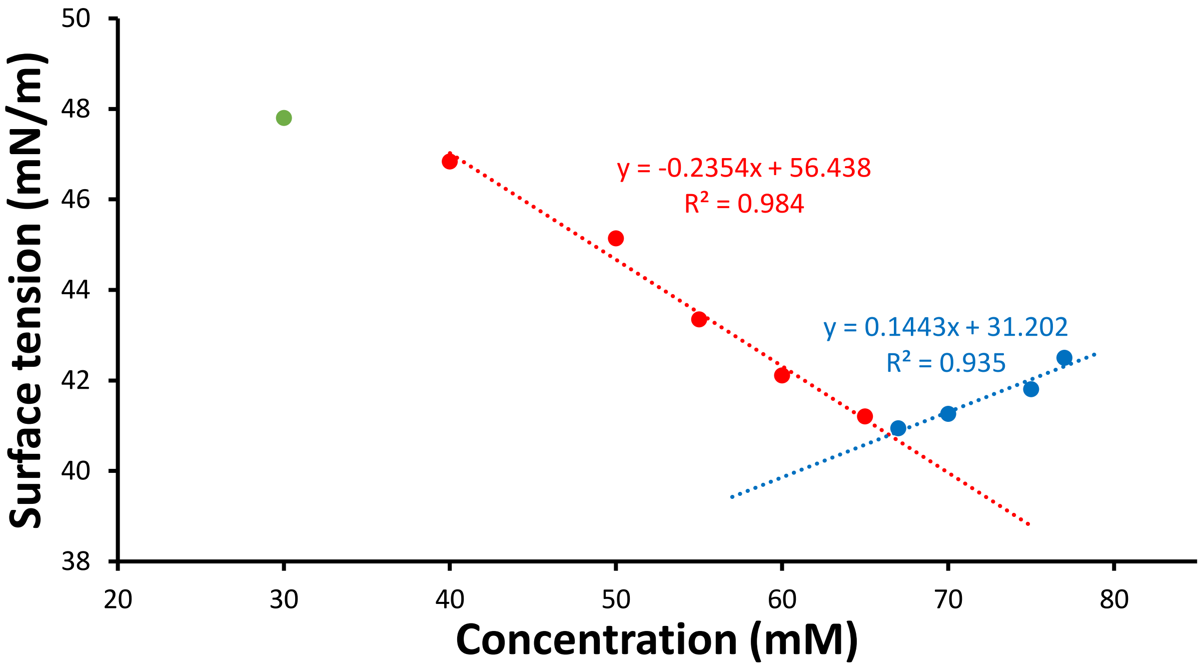

Figure S111 - Calculation of CMC (66.46 mM) for compound **45** in an EtOH: H2O 1:19 mixture using surface tension measurements.

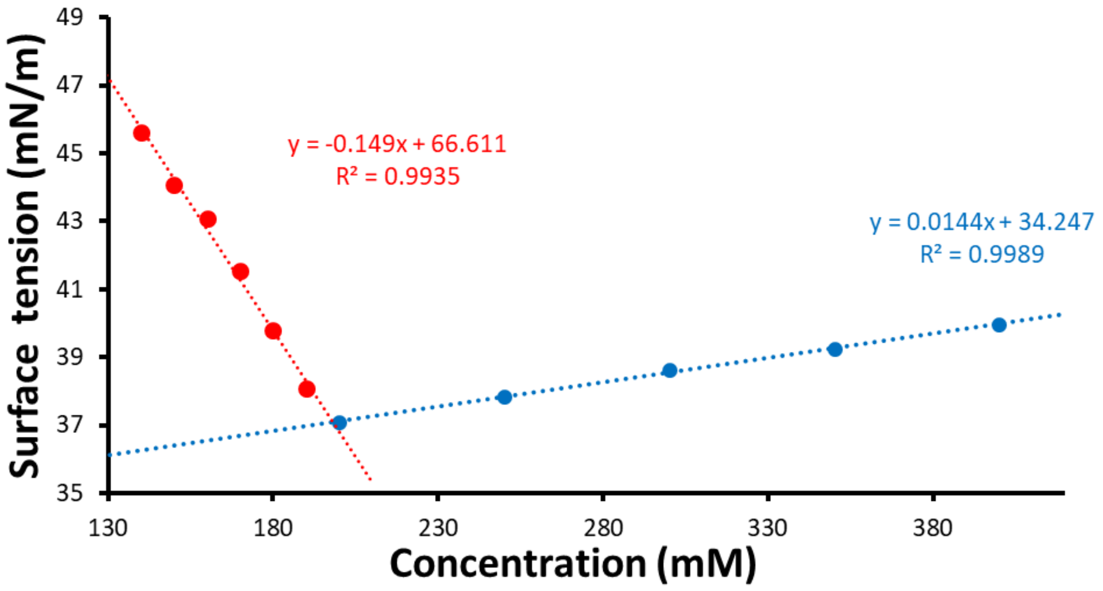

Figure S112 - Calculation of CMC (198.01 mM) for compound **50** in an EtOH: H2O 1:19 mixture using surface tension measurements.

Overview

Table S7 - Summary of zeta potential at 5.56 mM, CMC, and surface tension at CMC. Data obtained for compounds in an EtOH: H2O (1:19) solution.

Compound	Zeta potential (mV)	CMC (mM)	Surface tension at CMC (mN/m)
**15**	-21.30	28.95	30.25
**16**	-18.90	82.27	31.62
**17**	*a*	85.65	33.00
**18**	*a*	17.25	37.73
**23**	-57.17	3.69	28.65
**40**	-38.34	69.73	42.85
**41**	-27.58	44.91	42.10
**42**	-29.38	60.49	48.92
**43**	-37.38	38.44	39.55
**44**	-30.73	59.64	41.38
**45**	-35.32	66.46	40.79
**50**	*a*	198.01	37.10

a - Reproducible data sets could not be obtained.

Single crystal X-ray structures

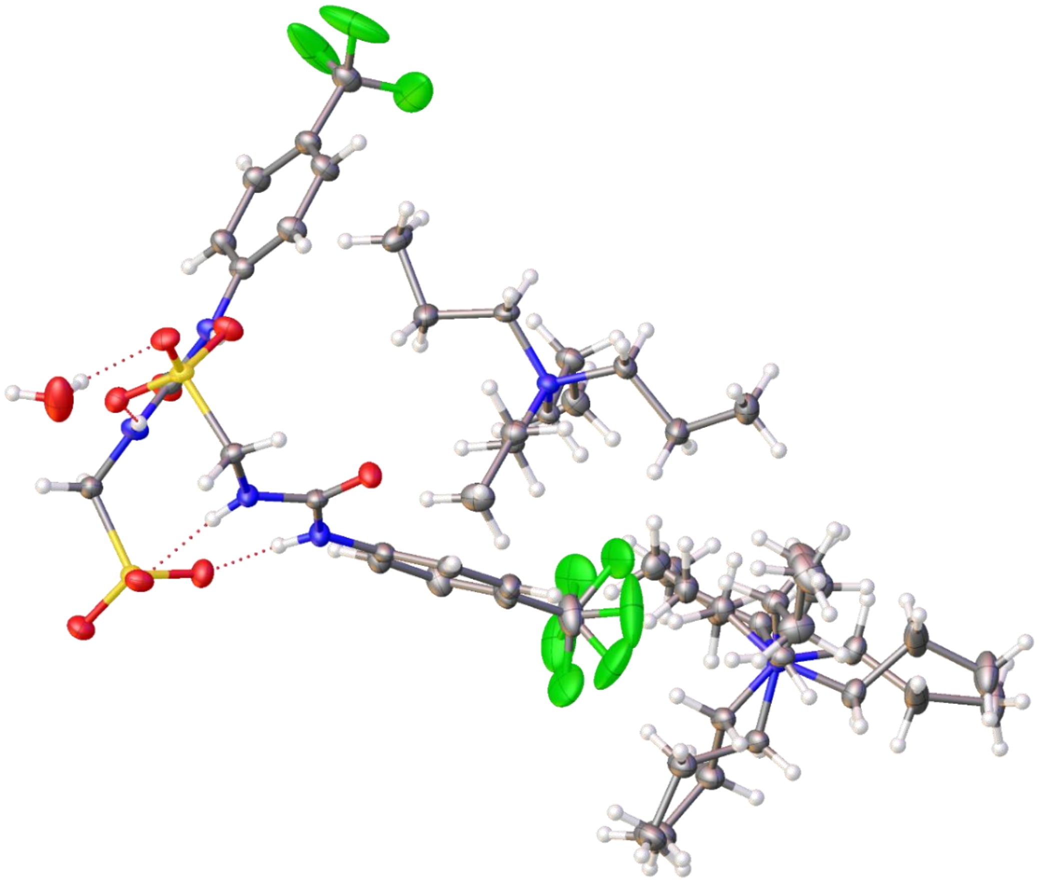

Figure S113 - Crystal data for compound **11**: red = oxygen; yellow = sulphur; blue = nitrogen; white = hydrogen; grey = carbon; green = fluorine. CCDC 1935642, $C_{42}H_{74}F_6N_6O_9S_2$ (M =985.19): monoclinic, space group P 21/c, a = 17.29752(19) Å, b = 12.22727(11) Å, c = 25.2681(3) Å, α = 90°, β = 109.1056(12)°, γ = 90°, V = 5049.86(10) Å^3, Z = 4, T = 100(1) K, CuK\α = 1.5418 Å, Dcalc = 1.296 g/cm^3, 34334 reflections measured (7.406 ≤ 2Θ ≤ 133.196), 8936 unique (R_{int} = 0.0276, R_{sigma} = 0.0231) which were used in all calculations. The final R_1 was 0.0395 (I > 2σ(I)) and wR_2 was 0.1020 (all data).

Table S8 - Hydrogen bond distances and angles observed for hydrogen bonded complex formation, calculated from single crystal X-ray structure.

Compound	Hydrogen bond donor	Hydrogen atom	Hydrogen bond acceptor	Hydrogen bond length (D•••A) (Å)	Hydrogen bond angle (D-H•••A) (°)
**11**	N1	H1	O7	2.8339 (18)	167.17 (10)
**11**	N2	H2	O8	2.8667 (19)	163.08 (10)
**11**	N3	H3	O3	2.9229 (19)	160.76 (10)
**11**	N4	H4	O4	2.9166 (19)	172.74 (10)
**11**	O9	H9C	O8	2.8930 (20)	167.37 (13)
**11**	O9	H9D	O6	2.848 (2)	158.03 (12)

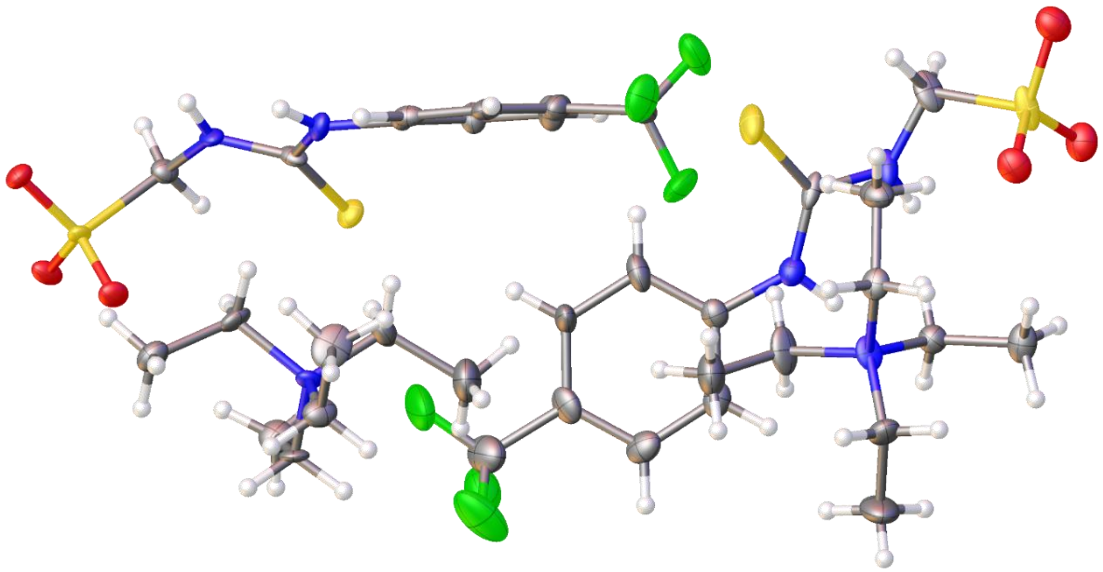

Figure S114 - Crystal data for compound **17**: red = oxygen; yellow = sulphur; blue = nitrogen; white = hydrogen; grey = carbon; green = fluorine. CCDC 1935641, $C_{17}H_{28}F_3N_3O_3S_2$ (M =443.54): orthorhombic, space group P 21 21 21, a = 8.5750(4) Å, b = 8.5822(7) Å, c = 58.926(2) Å, α = 90°, β = 90°, γ = 90°, V = 4336.5(4) $Å^3$, Z = 8, T = 100(1) K, CuK\α = 1.5418 Å, Dcalc = 1.359 g/cm^3, 10417 reflections measured (7.502 ≤ 2Θ ≤ 133.168), 6623 unique (R_{int} = 0.0532, R_{sigma} = 0.0782) which were used in all calculations. The final R_1 was 0.0632 (I > 2σ(I)) and wR_2 was 0.1478 (all data).

Table S9 - Hydrogen bond distances and angles observed for hydrogen bonded complex formation, calculated from single crystal X-ray structure.

**Compound**	**Hydrogen bond donor**	**Hydrogen atom**	**Hydrogen bond acceptor**	**Hydrogen bond length (D•••A) (Å)**	**Hydrogen bond angle (D-H•••A) (°)**
**17**	N1	H1	O2	2.906 (10)	167.3 (6)
**17**	N2	H2	O1	2.915 (10)	149.8 (6)
**17**	N3	H3	O5	2.869 (12)	169.1 (7)
**17**	N4	H4	O6	3.044 (11)	158.6 (6)

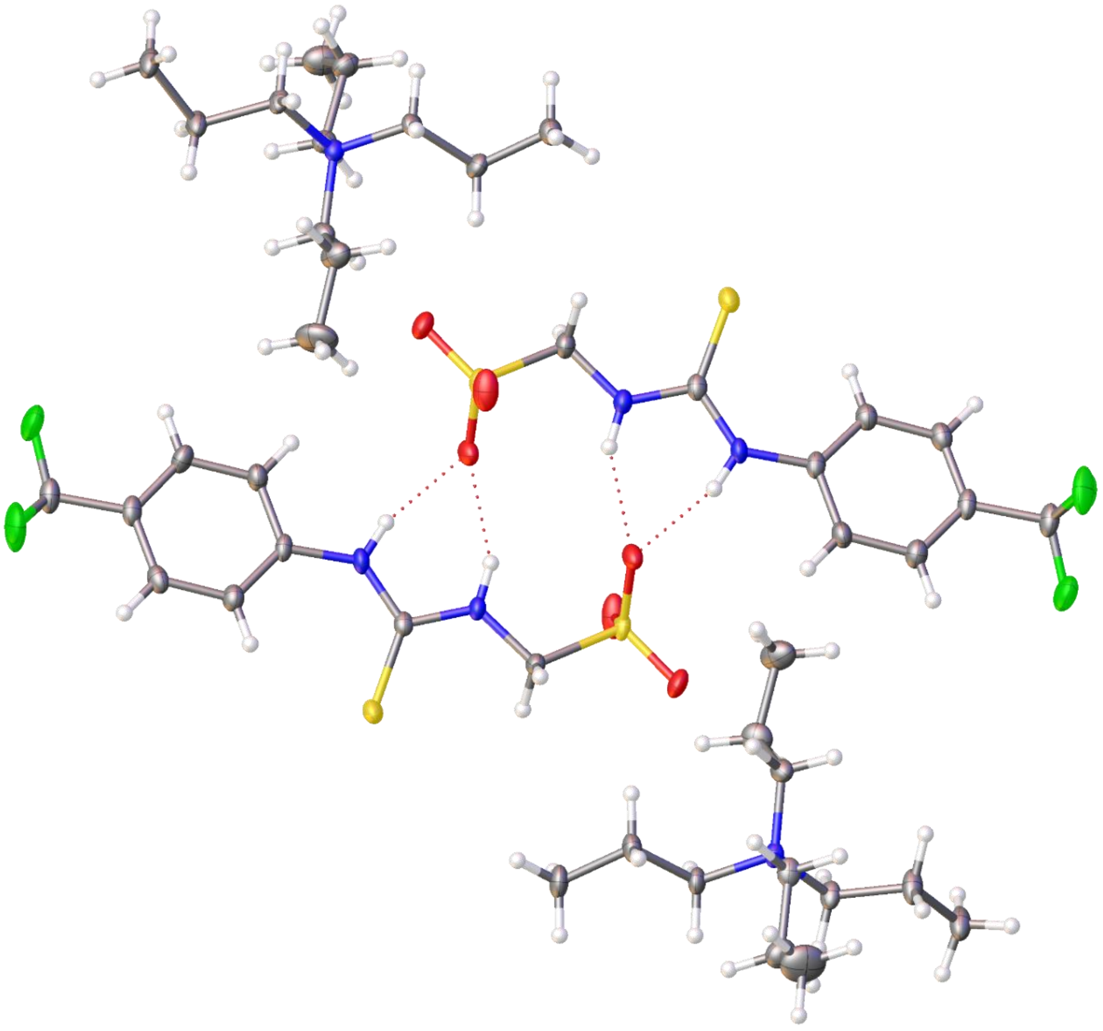

Figure S115 - Crystal data for compound **18**: red = oxygen; yellow = sulphur; blue = nitrogen; white = hydrogen; grey = carbon; green = fluorine. CCDC 1935643, $C_{21}H_{36}F_3N_3O_3S_2$ (M =499.65): orthorhombic, space group P c a 21, a = 21.9401(8) Å, b = 13.2290(6) Å, c = 17.5944(7) Å, α = 90°, β = 90°, γ = 90°, V = 5106.7(4) $Å^3$, Z = 8, T = 100(1) K, CuK\α = 1.5418 Å, Dcalc = 1.300 g/cm^3, 34801 reflections measured (7.740 ≤ 2Θ ≤ 145.334), 8952 unique (R_{int} = 0.0574, R_{sigma} = 0.0339) which were used in all calculations. The final R_1 was 0.0354 (I > 2σ(I)) and wR_2 was 0.0944 (all data).

Table S10 - Hydrogen bond distances and angles observed for hydrogen bonded complex formation, calculated from single crystal X-ray structure.

Compound	Hydrogen bond donor	Hydrogen atom	Hydrogen bond acceptor	Hydrogen bond length (D•••A) (Å)	Hydrogen bond angle (D-H•••A) (°)
**18**	N1	H1	O4	2.85058 (8)	163.9701 (2)
**18**	N2	H2	O4	2.94424 (11)	151.4911 (12)
**18**	N3	H3	O2	2.84431 (8)	164.4349 (2)
**18**	N4	H4	O2	2.95517 (11)	150.3394 (13)

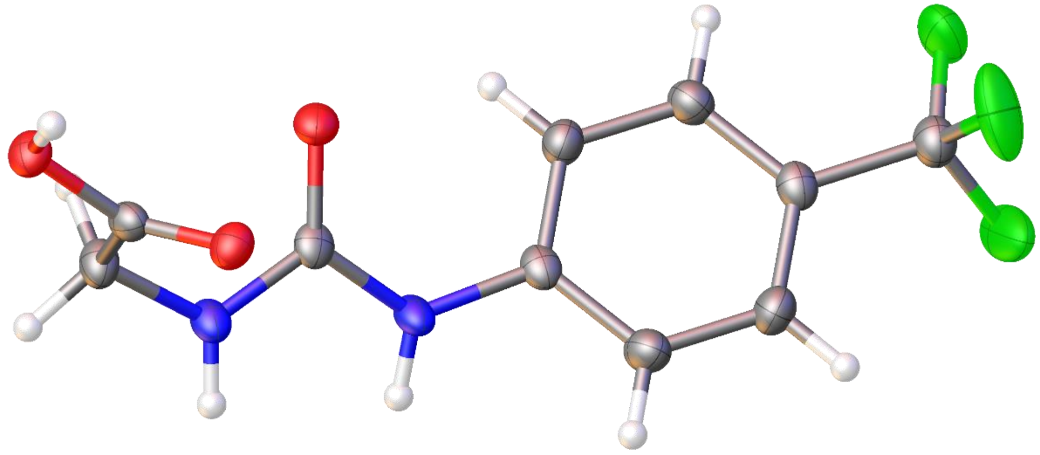

Figure S116 - Crystal data for compound **29**: red = oxygen; blue = nitrogen; white = hydrogen; grey = carbon; green = fluorine. CCDC 1935562, $C_{10}H_9 F_3N_2O_3$ (M =262.19): orthorhombic, space group P b c a, a = 10.3848(3) Å, b = 8.6429(2) Å, c = 23.9507(6) Å, α = 90°, β = 90°, γ = 90°, V = 2149.69(10) Å^3, Z = 8, T = 100(1) K, CuK\α = 1.5418 Å, Dcalc = 1.620 g/cm^3, 32184 reflections measured (7.382 ≤ 2Θ ≤ 133.198), 1897 unique (R_{int} = 0.0463, R_{sigma} = 0.0144) which were used in all calculations. The final R_1 was 0.0355 (I > 2σ(I)) and wR_2 was 0.0888 (all data).

Table S11 - Hydrogen bond distances and angles observed for hydrogen bonded complex formation, calculated from single crystal X-ray structure.

**Compound**	**Hydrogen bond donor**	**Hydrogen atom**	**Hydrogen bond acceptor**	**Hydrogen bond length (D•••A) (Å)**	**Hydrogen bond angle (D-H•••A) (°)**
**29**	N1	H1	O1	2.8961 (18)	154.92 (10)
**29**	N2	H2	O1	2.9329 (18)	152.03 (9)
**29**	O3	H3	O2	2.6711 (16)	172.72 (9)

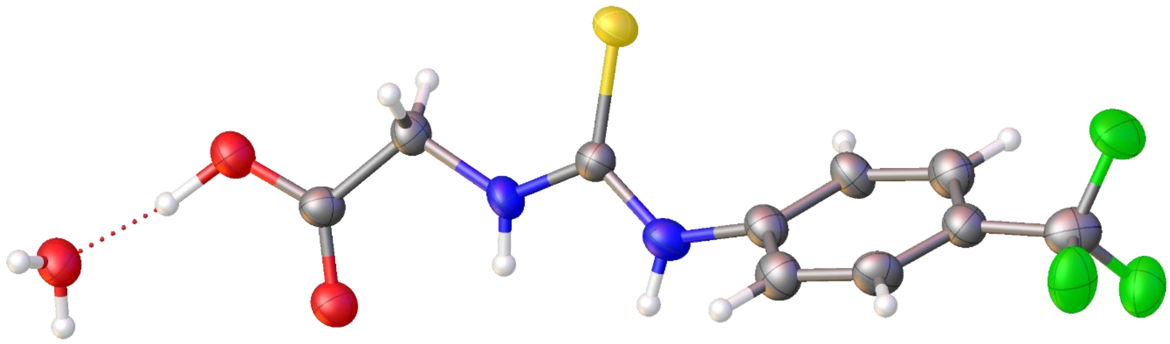

Figure S117 - Crystal data for compound **32**: red = oxygen; yellow = sulphur; blue = nitrogen; white = hydrogen; grey = carbon; green = fluorine. CCDC 1935640, $C_{10}H_{11}F_3N_2O_3S$ (M =296.27): monoclinic, space group P 21/c, a = 20.786(10) Å, b = 6.941(2) Å, c = 8.610(2) Å, α = 90°, β = 100.49(4)°, γ = 90°, V = 1221.5(8) $Å^3$, Z = 4, T = 100(1) K, CuK\α = 1.5418 Å, Dcalc = 1.611 g/cm^3, 2648 reflections measured (8.652 ≤ 2Θ ≤ 133.198), 1824 unique (R_{int} = 0.0845, R_{sigma} = 0.1182) which were used in all calculations. The final R_1 was 0.1125 (I > 2σ(I)) and wR_2 was 0.3216 (all data).

Table S12- Hydrogen bond distances and angles observed for hydrogen bonded complex formation, calculated from single crystal X-ray structure.

Compound	Hydrogen bond donor	Hydrogen atom	Hydrogen bond acceptor	Hydrogen bond length (D•••A) (Å)	Hydrogen bond angle (D-H•••A) (°)
**32**	O1	H1	O3	2.5640 (10)	171.859 (5)
**32**	O3	H3B	O2	2.8610 (8)	124.87 (3)
**32**	O3	H3B	O3	3.3993 (11)	133.91 (2)
**32**	O3	H3A	S1	3.3429 (16)	160.046 (9)
**32**	N1	H1A	S1	3.6511 (9)	130.7181 (8)
**32**	N2	H2	S1	3.2746 (8)	146.079 (16)

In silico modelling

Computational calculations to identify primary hydrogen bond donating and accepting sites were conducted in line with studies reported by Hunter using Spartan 16''.[10] Calculations were performed using semi-empirical PM6 methods, after energy minimisation calculations, to identify E_{max}, E_{min} and LogP values. PM6 was used over AM1 in line with research conducted by Stewart.[11]

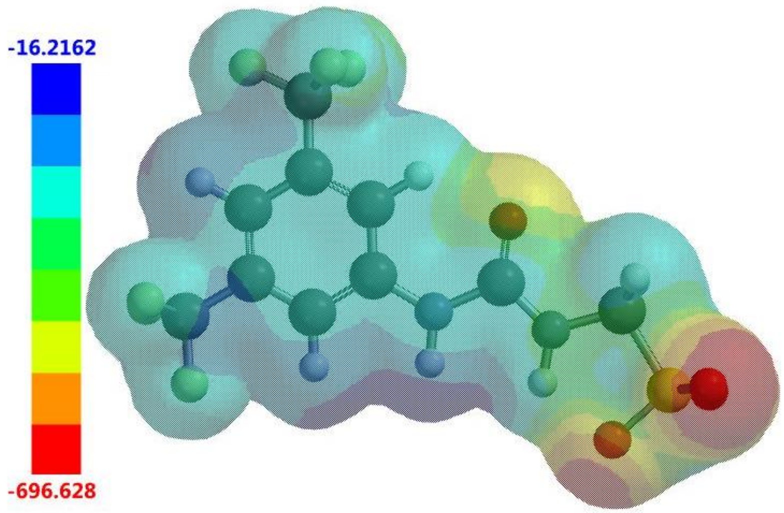

Figure S118 - Electrostatic potential map calculated for the anionic component of **23**. E_{max} and E_{min} values depicted in the figure legends are given in KJ/mol.

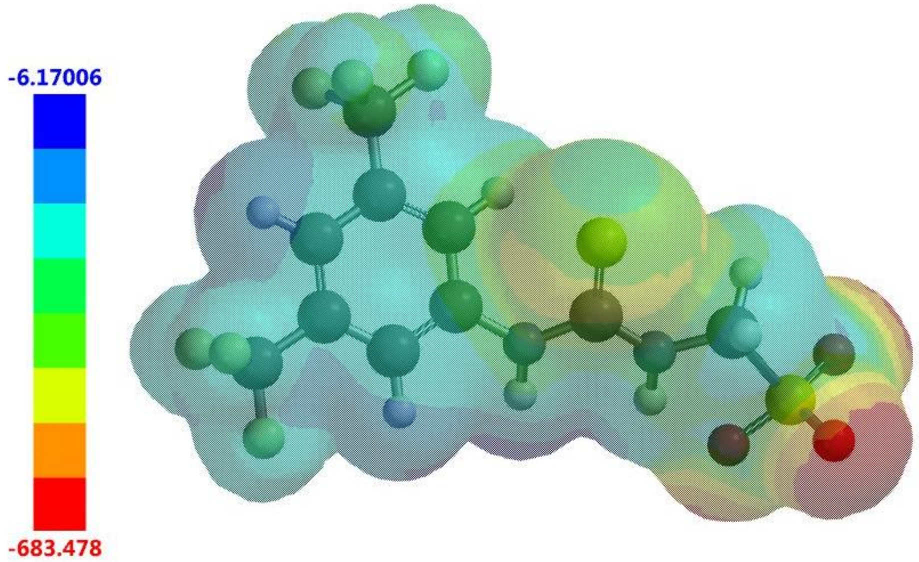

Figure S119 - Electrostatic potential map calculated for the anionic component of **27**. E_{max} and E_{min} values depicted in the figure legends are given in KJ/mol.

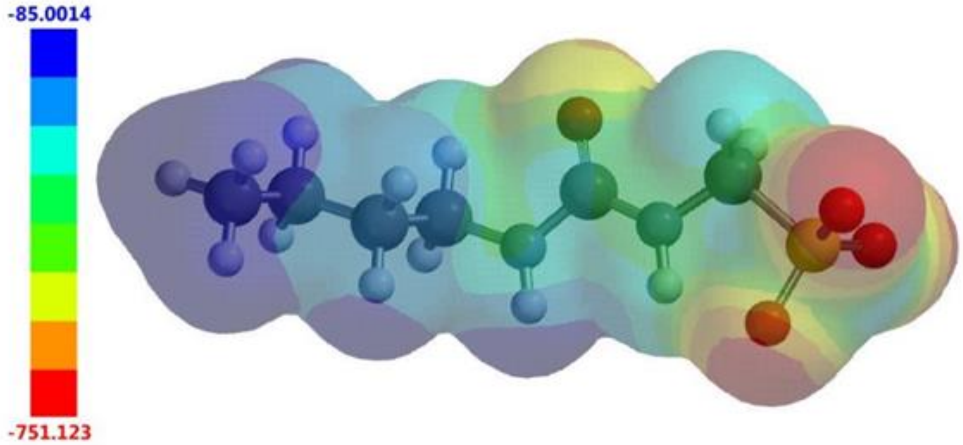

Figure S120 - Electrostatic potential map calculated for the anionic component of **40**. E_{max} and E_{min} values depicted in the figure legends are given in KJ/mol.

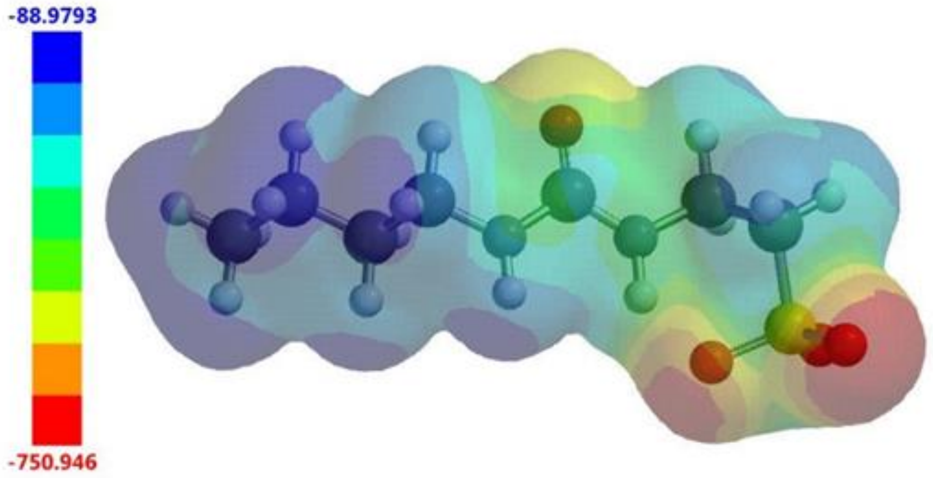

Figure S121 - Electrostatic potential map calculated for the anionic component of **41**. E_{max} and E_{min} values depicted in the figure legends are given in KJ/mol.

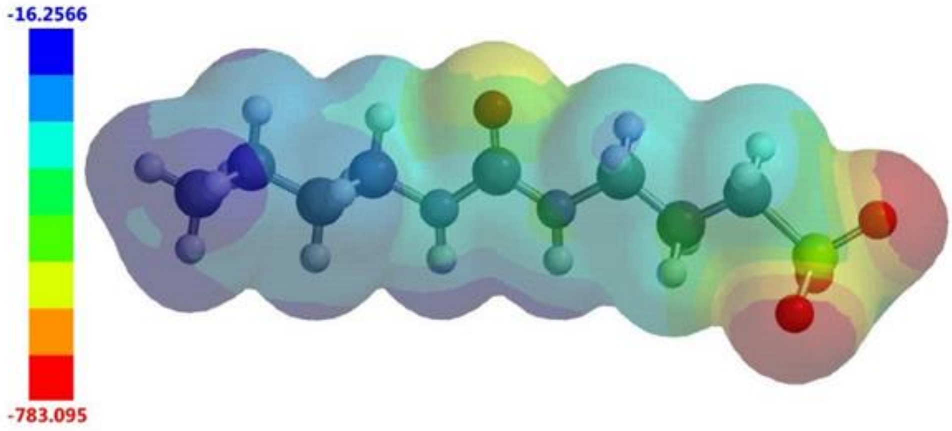

Figure S122 - Electrostatic potential map calculated for the anionic component of **42**. E_{max} and E_{min} values depicted in the figure legends are given in KJ/mol.

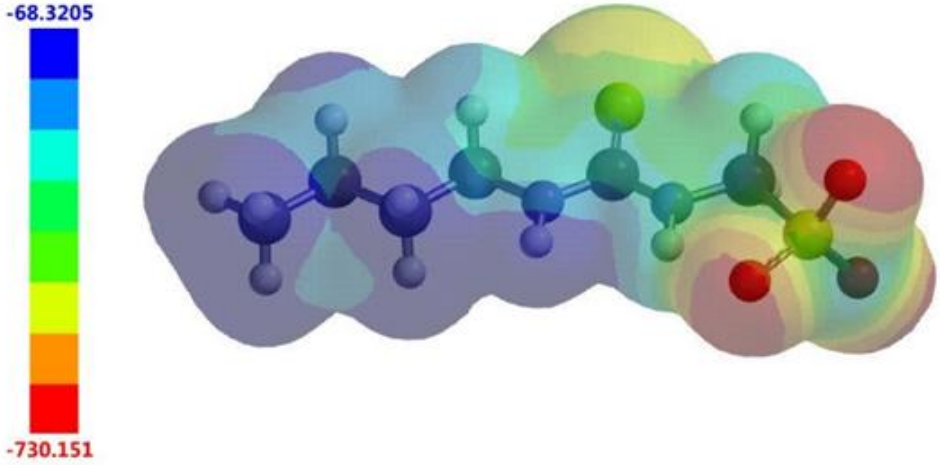

Figure S123 - Electrostatic potential map calculated for the anionic component of **43**. E_{max} and E_{min} values depicted in the figure legends are given in KJ/mol.

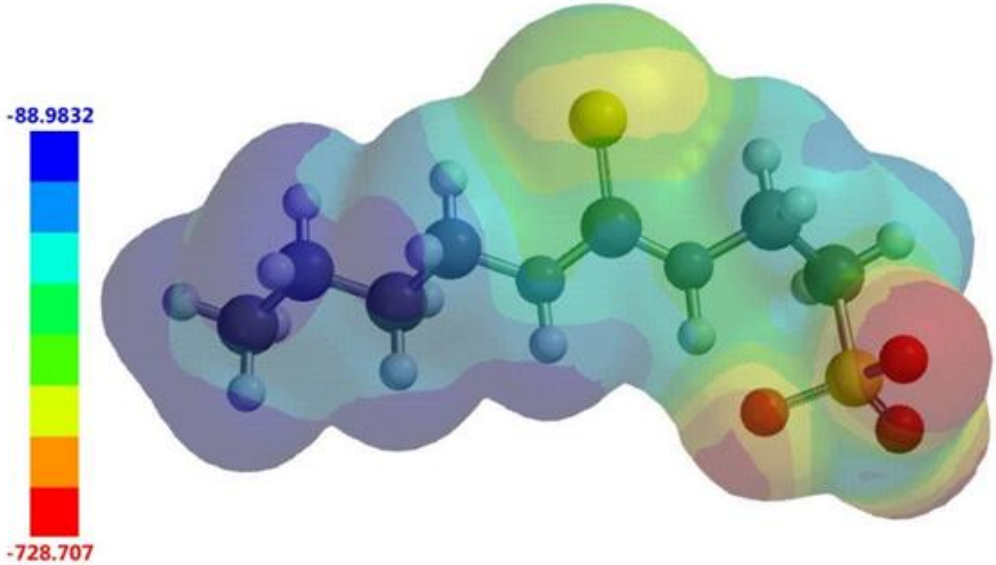

Figure S124 - Electrostatic potential map calculated for the anionic component of **44**. E_{max} and E_{min} values depicted in the figure legends are given in KJ/mol.

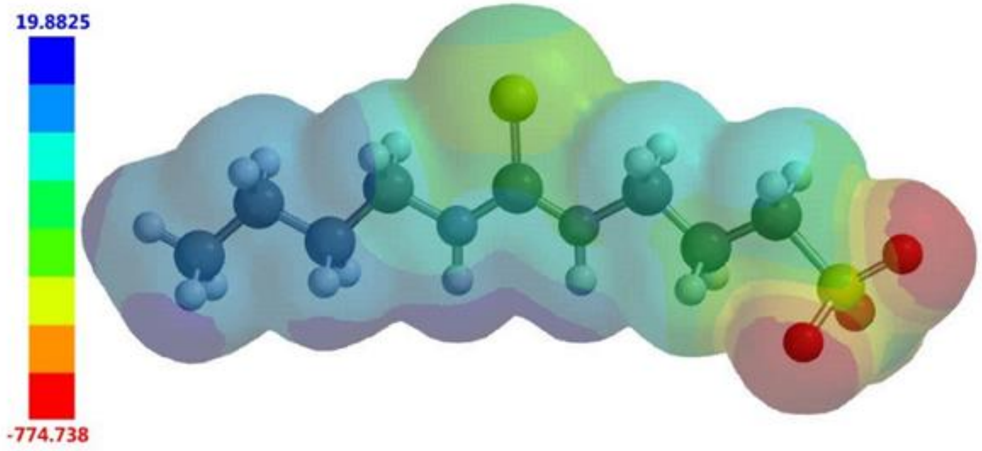

Figure S125 - Electrostatic potential map calculated for the anionic component of **45**. E_{max} and E_{min} values depicted in the figure legends are given in KJ/mol.

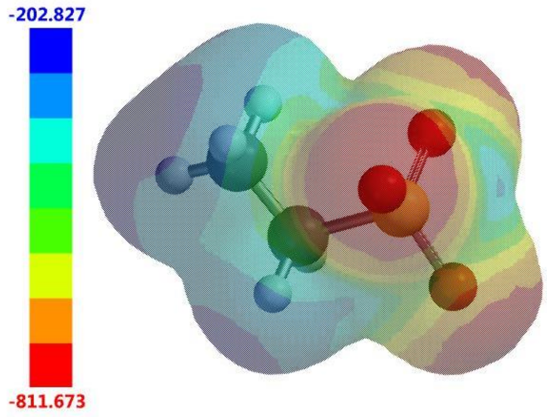

Figure S126 - Electrostatic potential map calculated for the anionic component of **46**. E_{max} and E_{min} values depicted in the figure legends are given in KJ/mol.

Overview

Table S13 - Summary of E_{max}, E_{min} and Log*P* values.

Compound	E_{max} (KJ/mol)	E_{min} (KJ/mol)	LogP
**23**	- 696.6280	- 16.2162	3.88
**27**	- 683.4780	- 6.1700	3.88
**40**	- 751.1230	- 85.0014	2.17
**41**	- 750.9460	- 88.9793	2.17
**42**	- 783.0950	- 16.2566	2.17
**43**	- 730.1510	- 68.3205	2.17
**44**	- 728.7070	- 88.9832	2.17
**45**	- 774.7380	+ 19.8825	2.17
**46**	- 811.6730	- 202.8270	N/A

Mass Spectrum Data

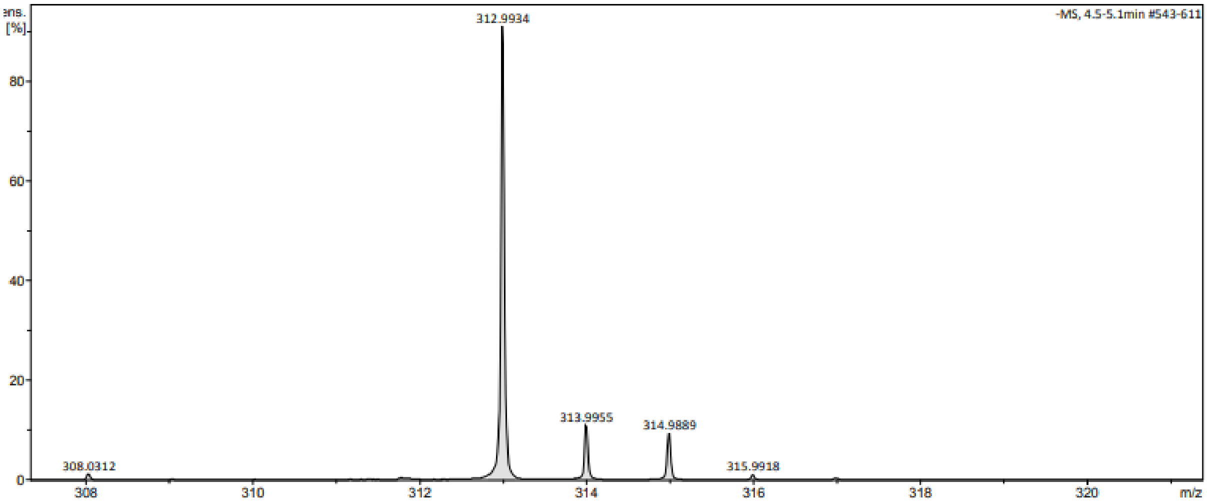

Figure S127 - A high-resolution mass spectrum (ESI$^-$) obtained for compound **14** in methanol.

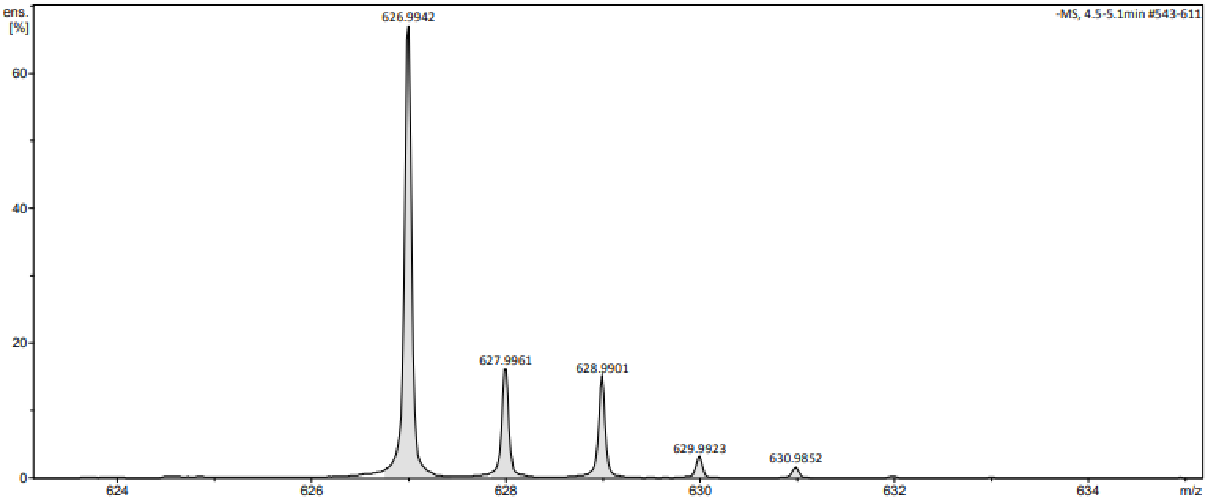

Figure S128 - A high-resolution mass spectrum (ESI$^-$) obtained for dimeric species of compound **14** in methanol, *m/z* $[M + M + H^+]^-$.

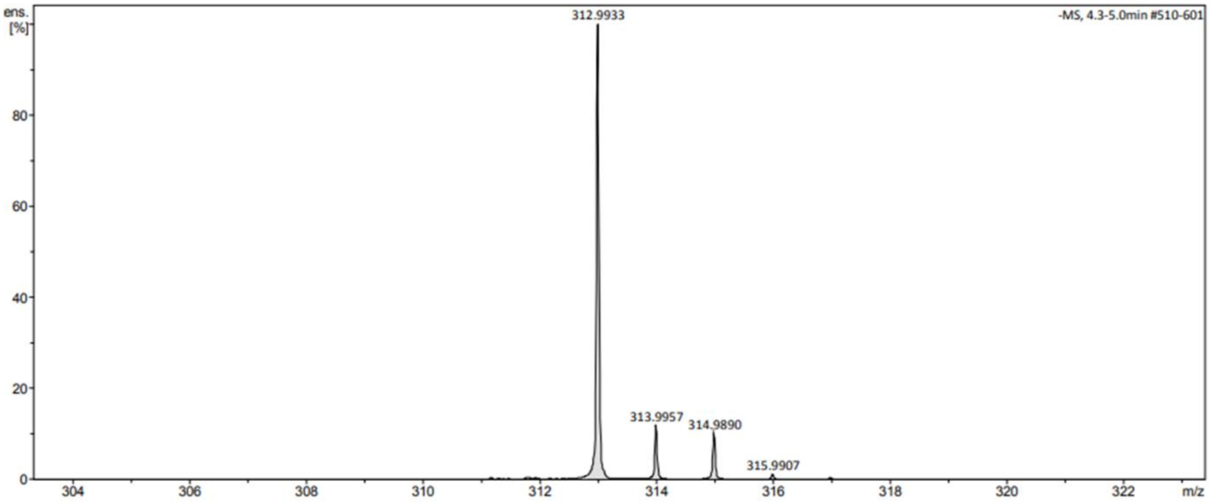

Figure S129 - A high-resolution mass spectrum (ESI⁻) obtained for compound **15** in methanol.

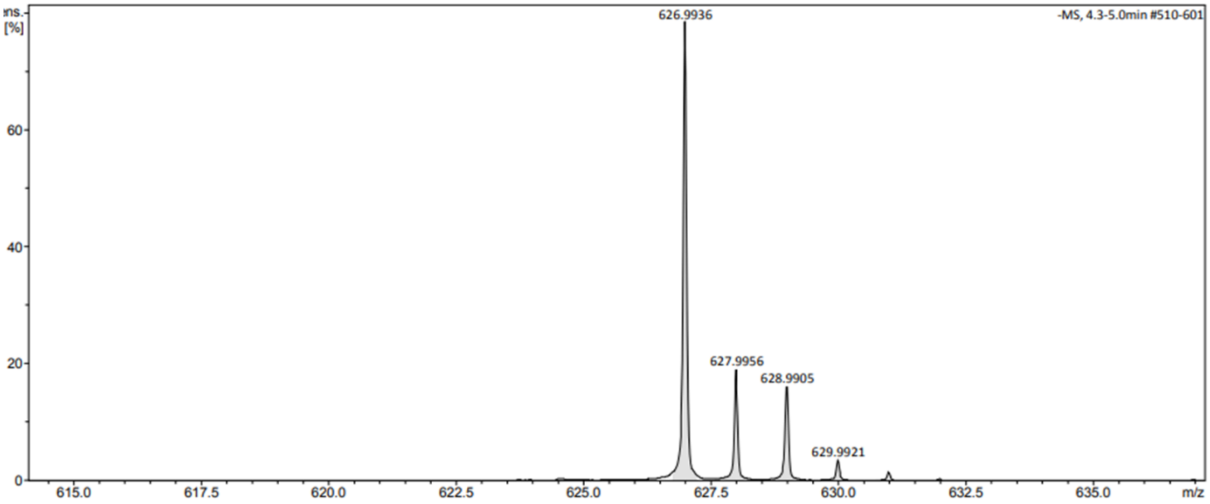

Figure S130 - A high-resolution mass spectrum (ESI⁻) obtained for dimeric species of compound **15** in methanol, *m/z* $[M + M + H^+]^-$.

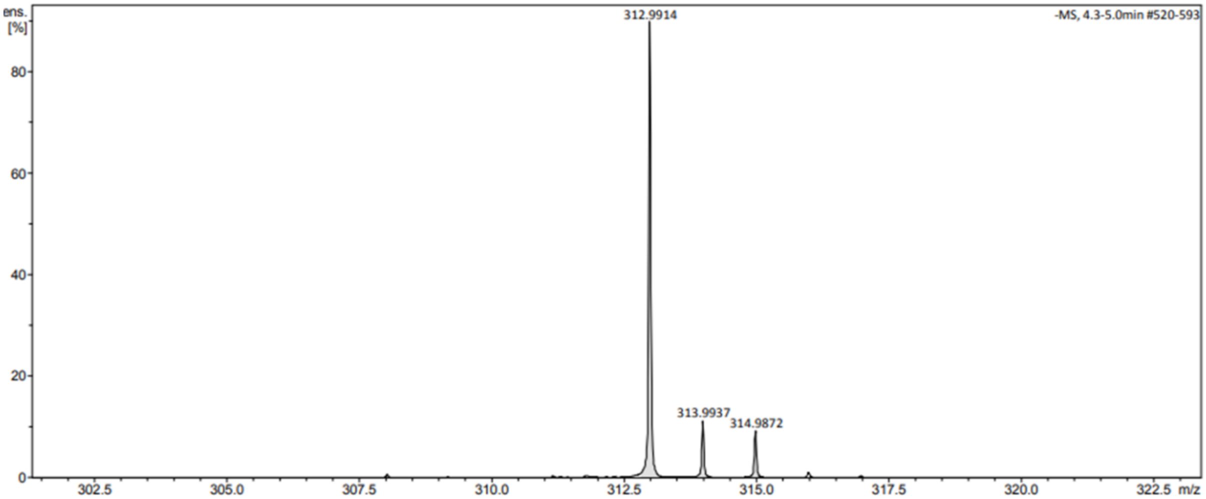

Figure S131 - A high-resolution mass spectrum (ESI⁻) obtained for compound **16** in methanol.

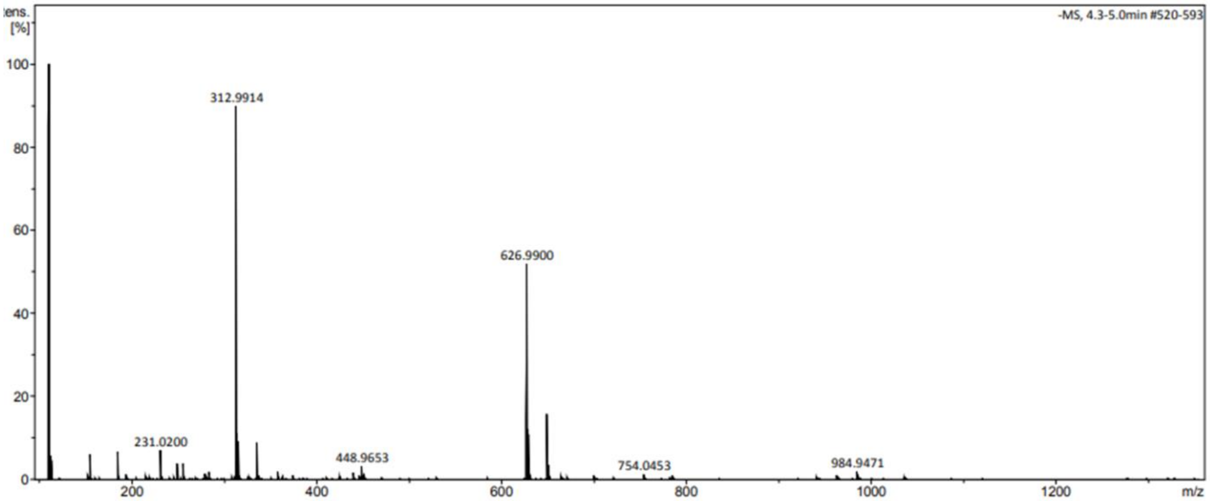

Figure S132 - A high-resolution mass spectrum (ESI⁻) obtained for dimeric species of compound **16** in methanol, *m/z* [M + M + H⁺]⁻.

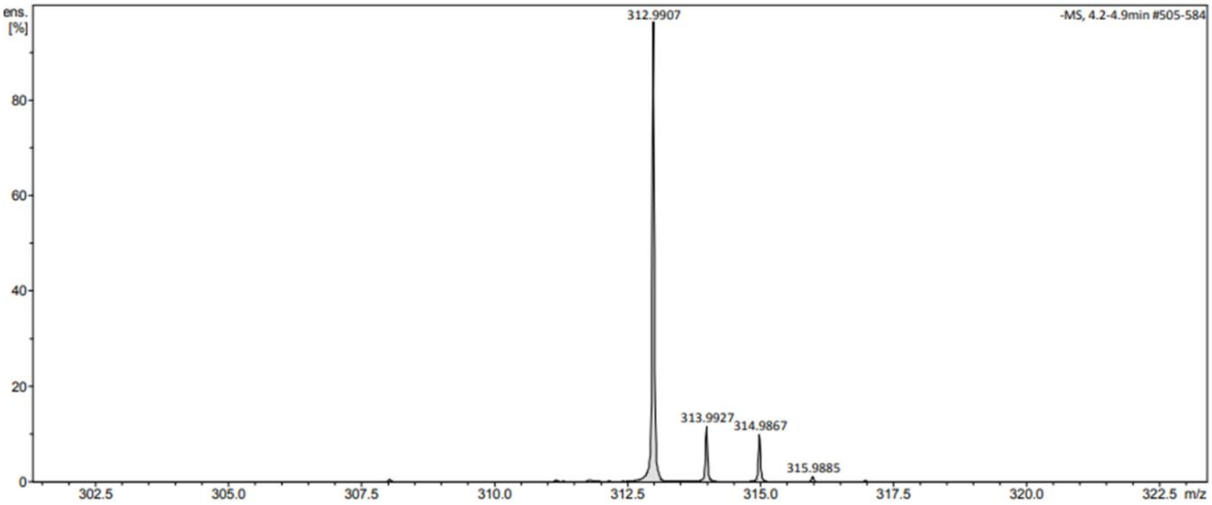

Figure S133 - A high-resolution mass spectrum (ESI$^-$) obtained for compound **17** in methanol.

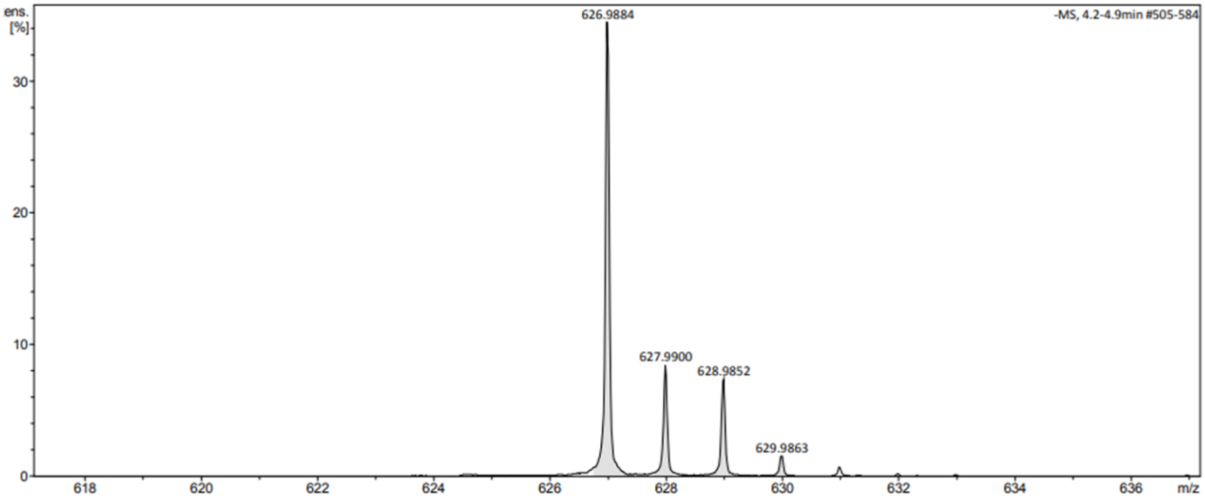

Figure S134 - A high-resolution mass spectrum (ESI$^-$) obtained for dimeric species of compound **17** in methanol, *m/z* $[M + M + H^+]^-$.

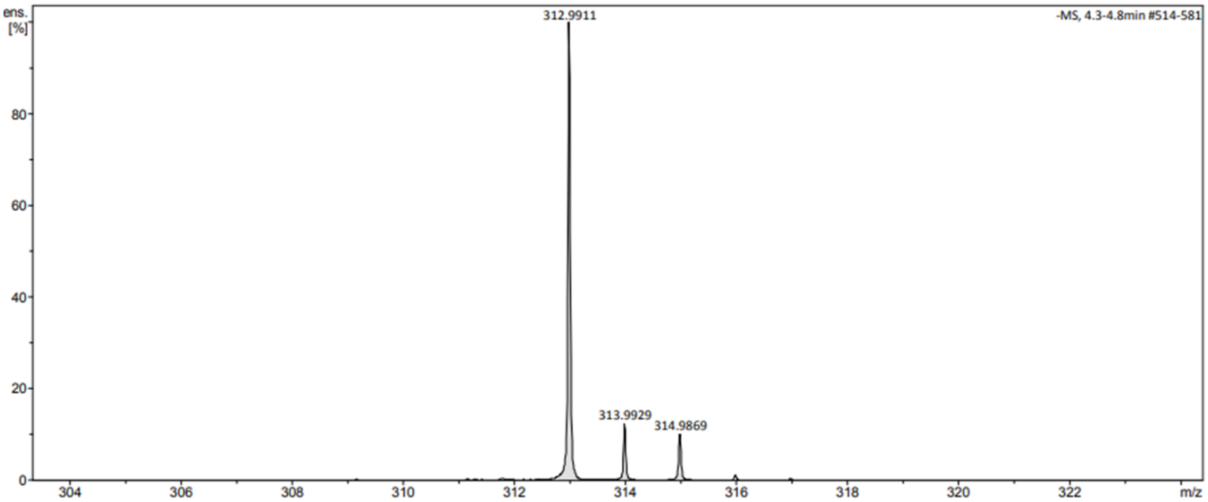

Figure S135 - A high-resolution mass spectrum (ESI$^-$) obtained for compound **18** in methanol.

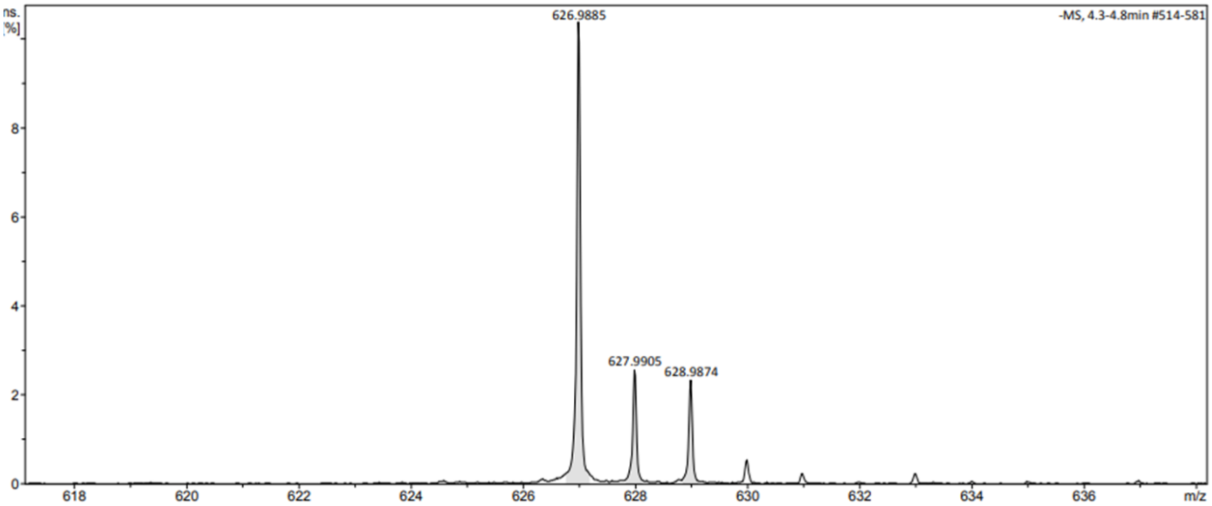

Figure S136 - A high-resolution mass spectrum (ESI$^-$) obtained for dimeric species of compound **18** in methanol, *m/z* [M + M + H$^+$]$^-$.

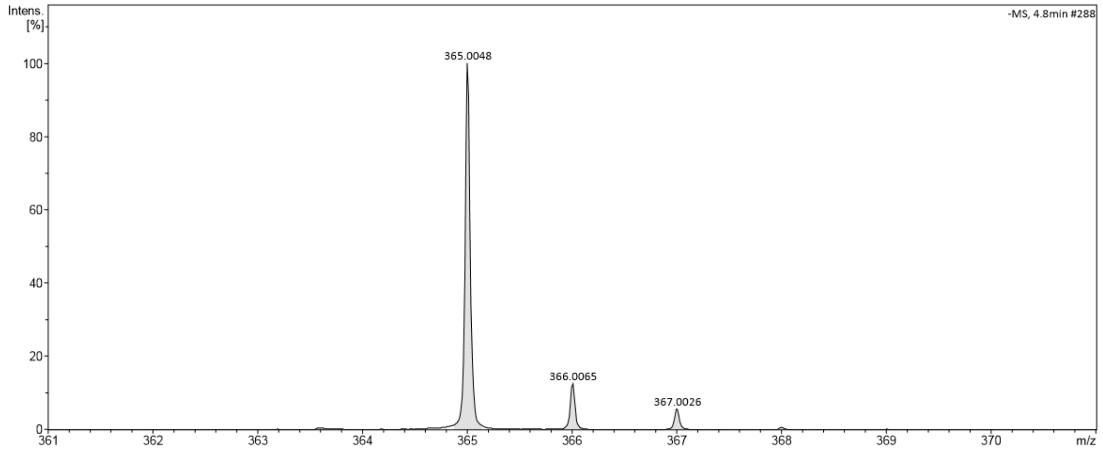

Figure S137 - A high-resolution mass spectrum (ESI$^-$) obtained for compound **23** in methanol.

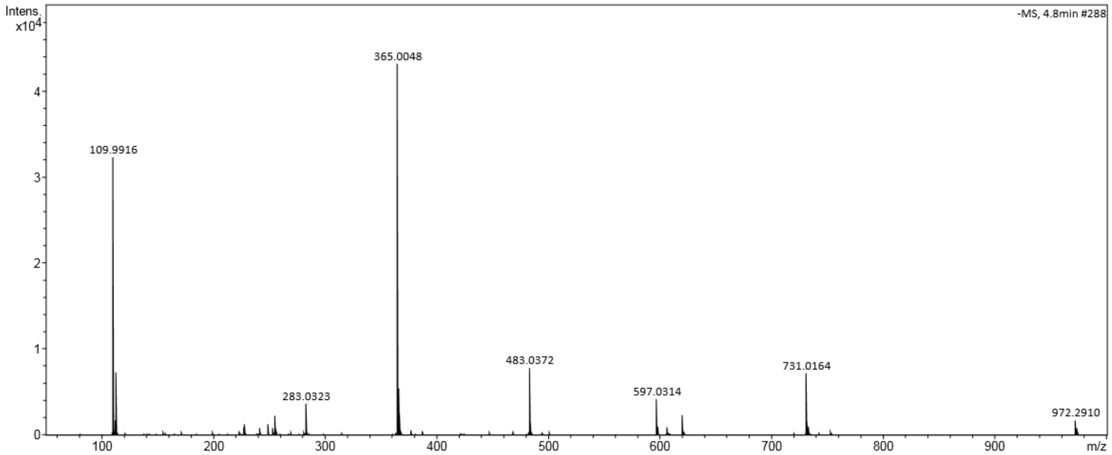

Figure S138 - A high-resolution mass spectrum (ESI$^-$) obtained for dimeric species of compound **23** in methanol, *m/z* [M + M + H$^+$]$^-$.

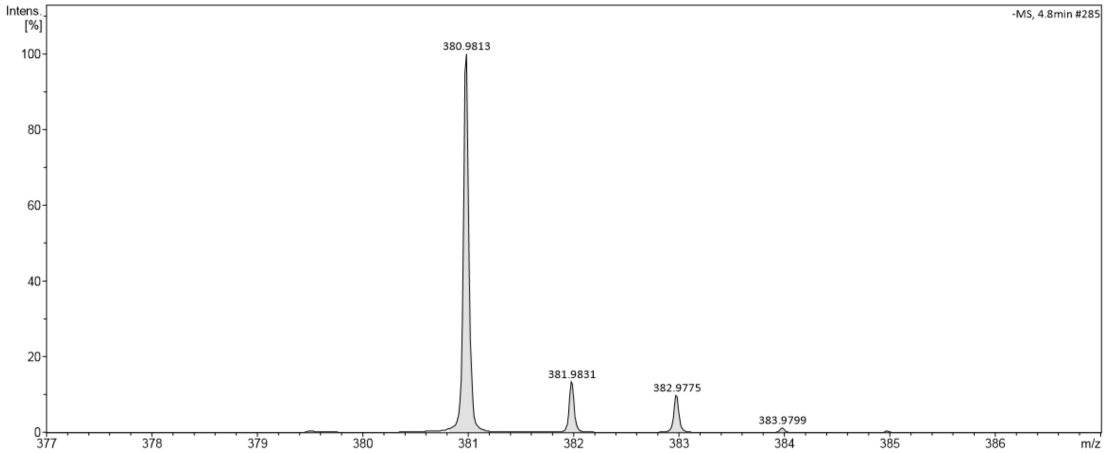

Figure S139 - A high-resolution mass spectrum (ESI$^-$) obtained for compound **27** in methanol.

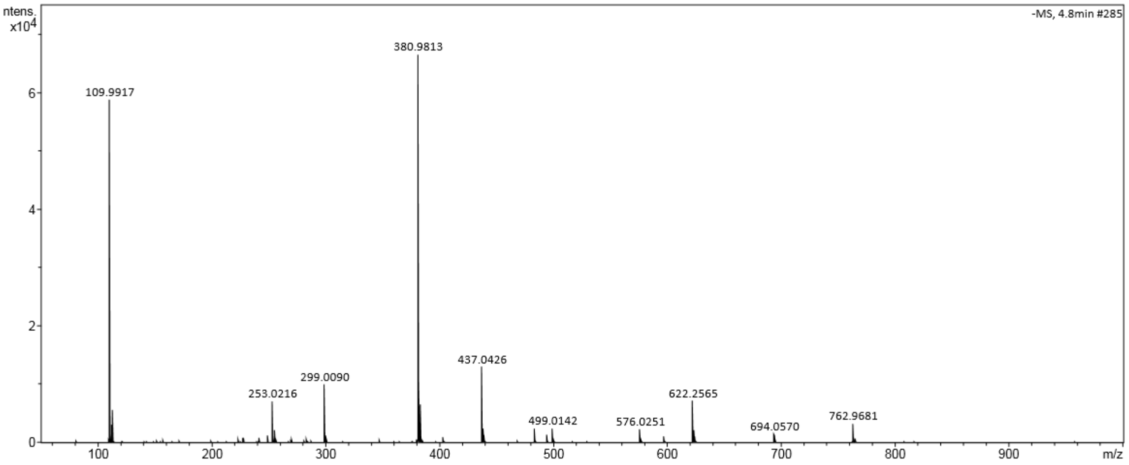

Figure S140 - A high-resolution mass spectrum (ESI$^-$) obtained for dimeric species of compound **27** in methanol, *m/z* [M + M + H$^+$]$^-$.

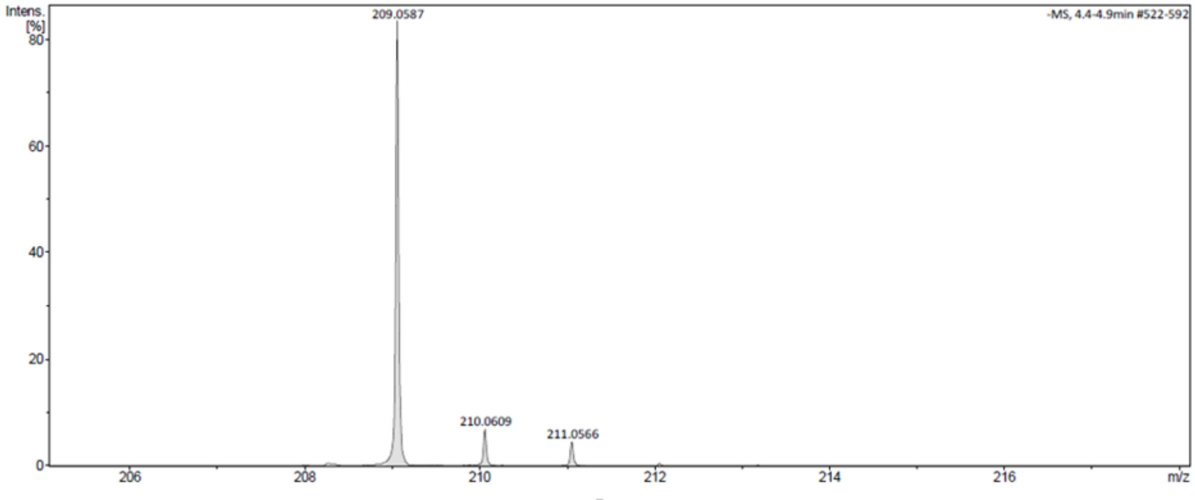

Figure S141 - A high-resolution mass spectrum (ESI$^-$) obtained for compound **40** in methanol.

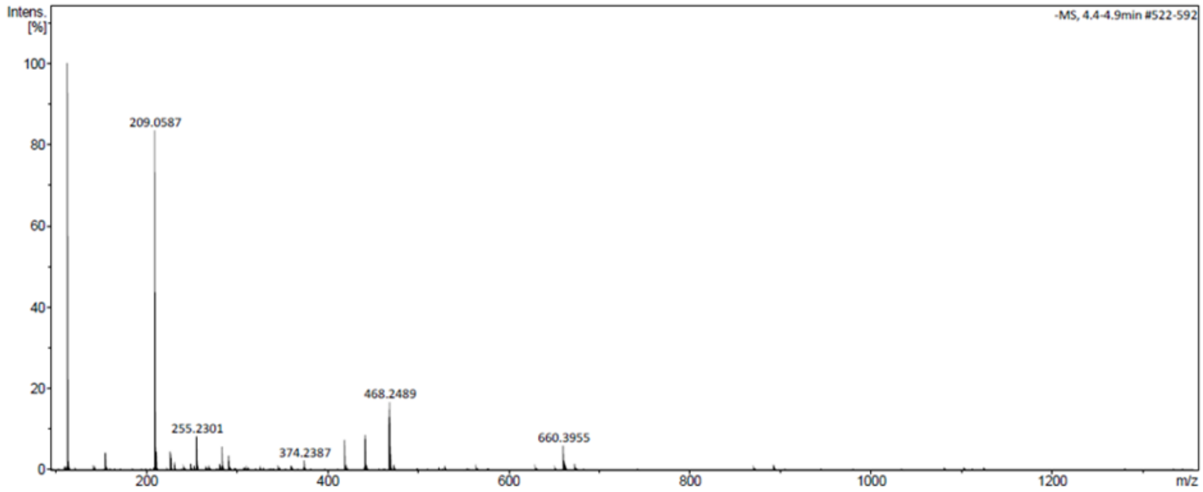

Figure S142 - A high-resolution mass spectrum (ESI$^-$) obtained for dimeric species of compound **40** in methanol, *m/z* [M + M + H$^+$]$^-$.

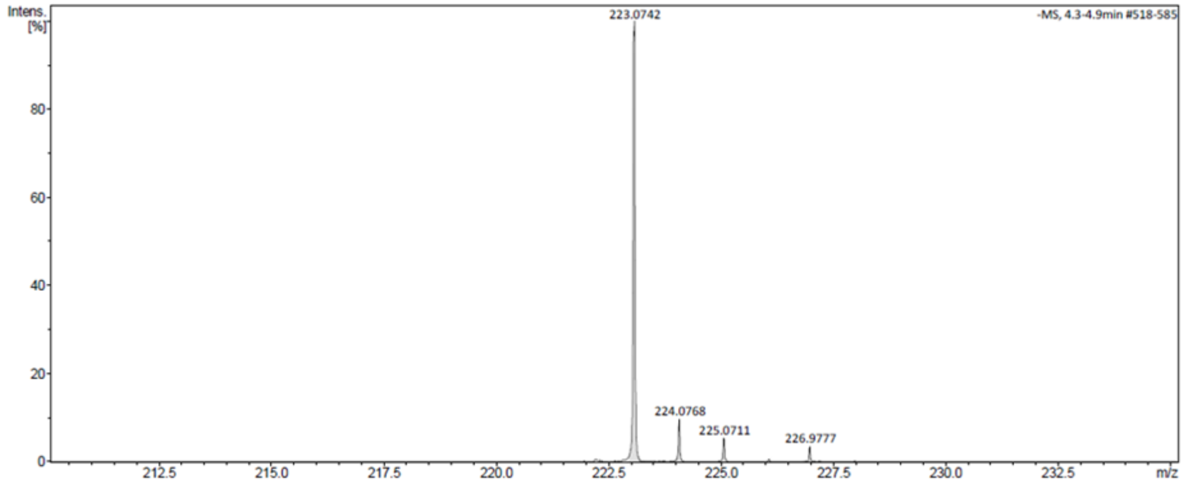

Figure S143 - A high-resolution mass spectrum (ESI$^-$) obtained for compound **41** in methanol.

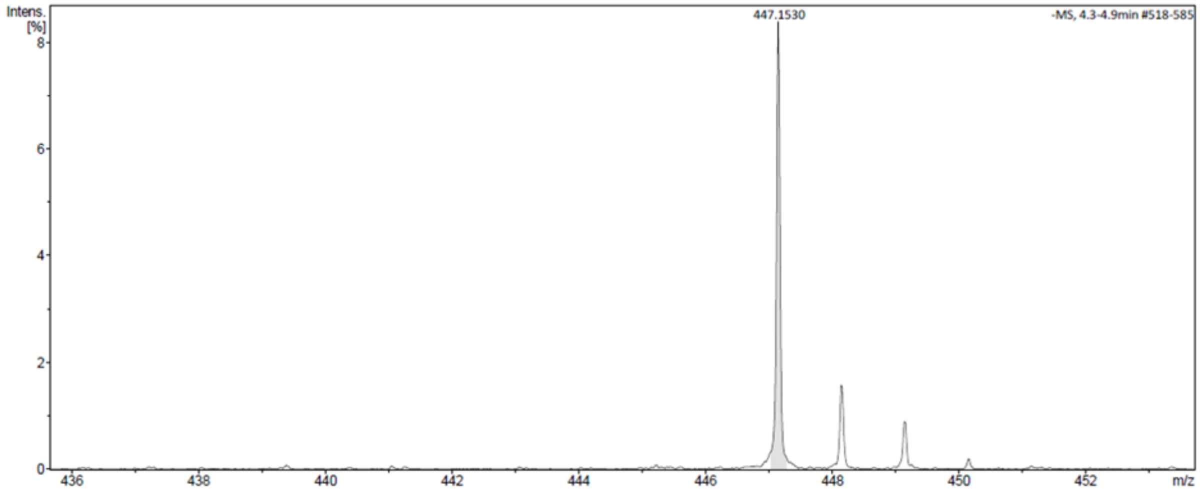

Figure S144 - A high-resolution mass spectrum (ESI$^-$) obtained for dimeric species of compound **41** in methanol, *m/z* [M + M + H$^+$]$^-$.

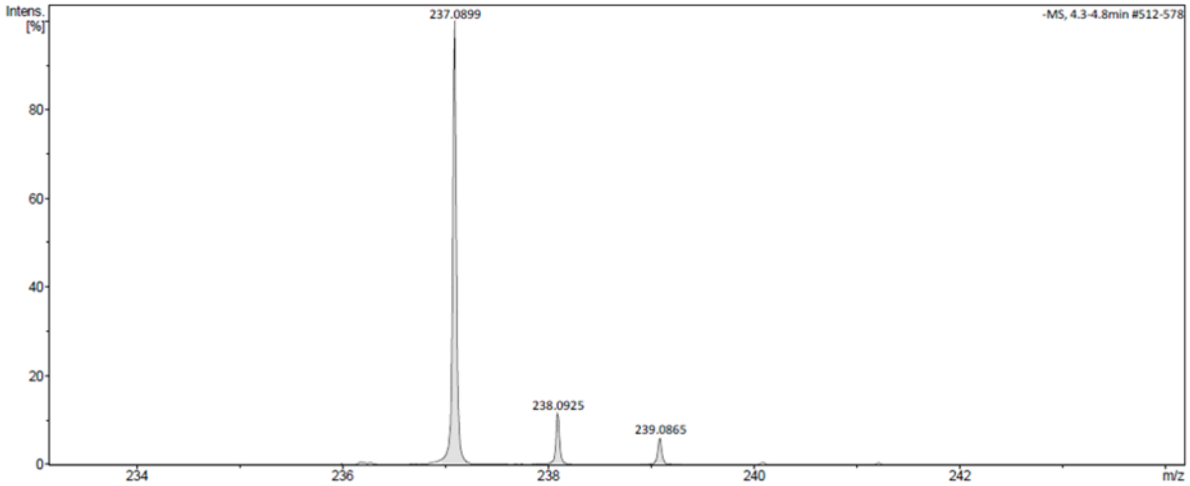

Figure S145 - A high-resolution mass spectrum (ESI$^-$) obtained for compound **42** in methanol.

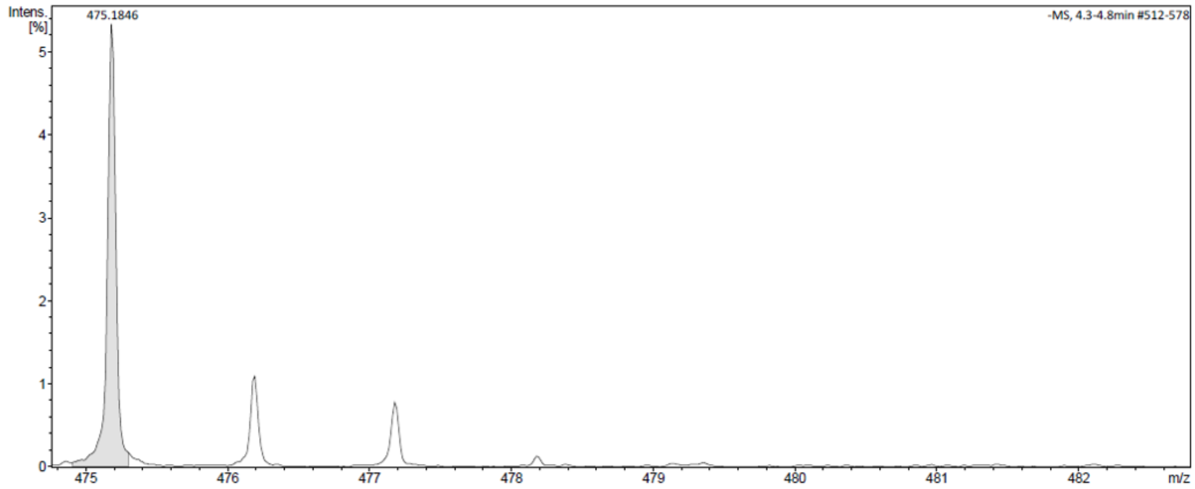

Figure S146 - A high-resolution mass spectrum (ESI-) obtained for dimeric species of compound **42** in methanol, *m/z* [M + M + H+]−.

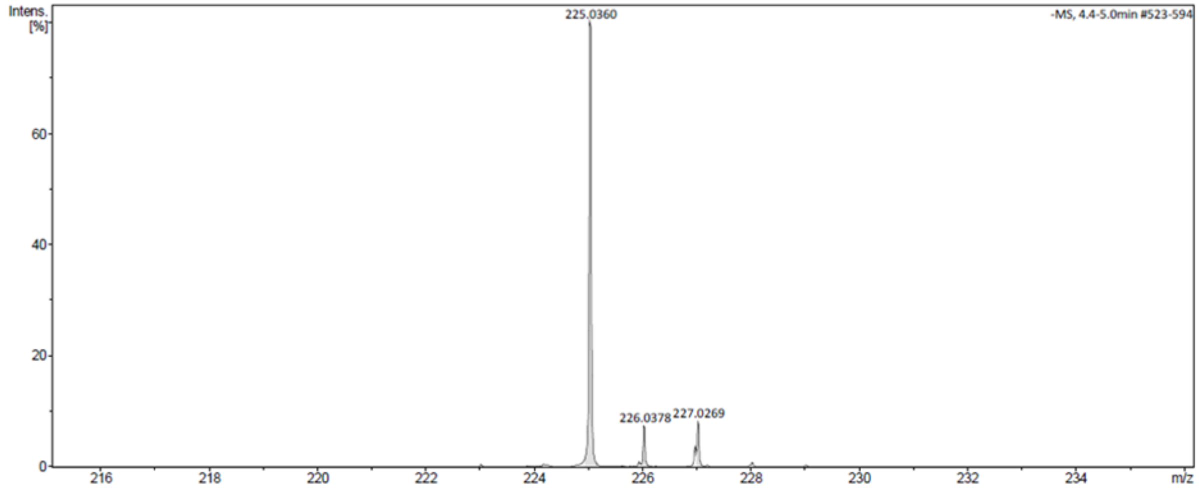

Figure S147 - A high-resolution mass spectrum (ESI-) obtained for compound **43** in methanol.

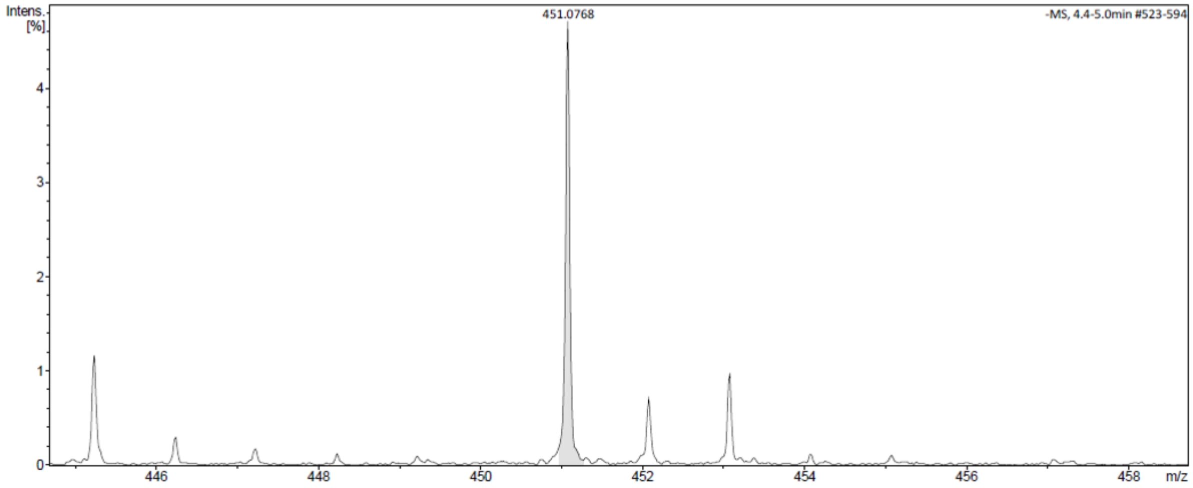

Figure S148 - A high-resolution mass spectrum (ESI$^-$) obtained for dimeric species of compound **43** in methanol, m/z [M + M + H$^+$]$^-$.

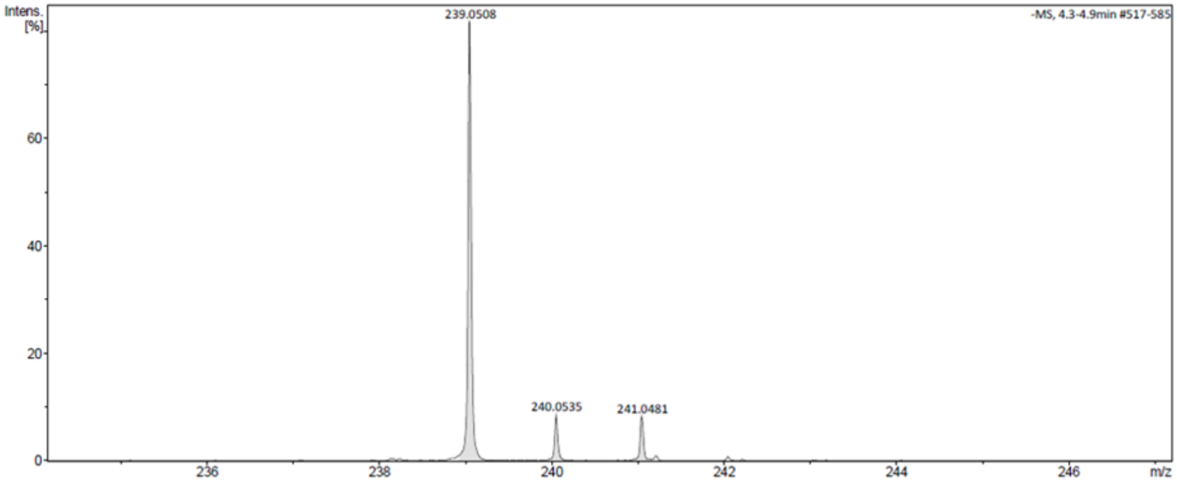

Figure S149 - A high-resolution mass spectrum (ESI$^-$) obtained for compound **44** in methanol.

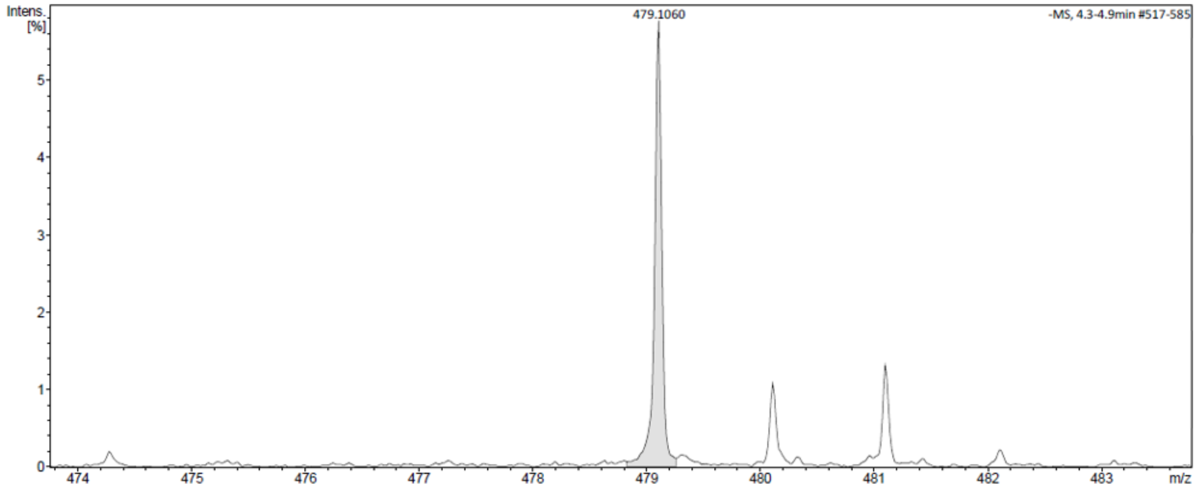

Figure S150 - A high-resolution mass spectrum (ESI$^-$) obtained for dimeric species of compound **44** in methanol, *m/z* [M + M + H$^+$]$^-$.

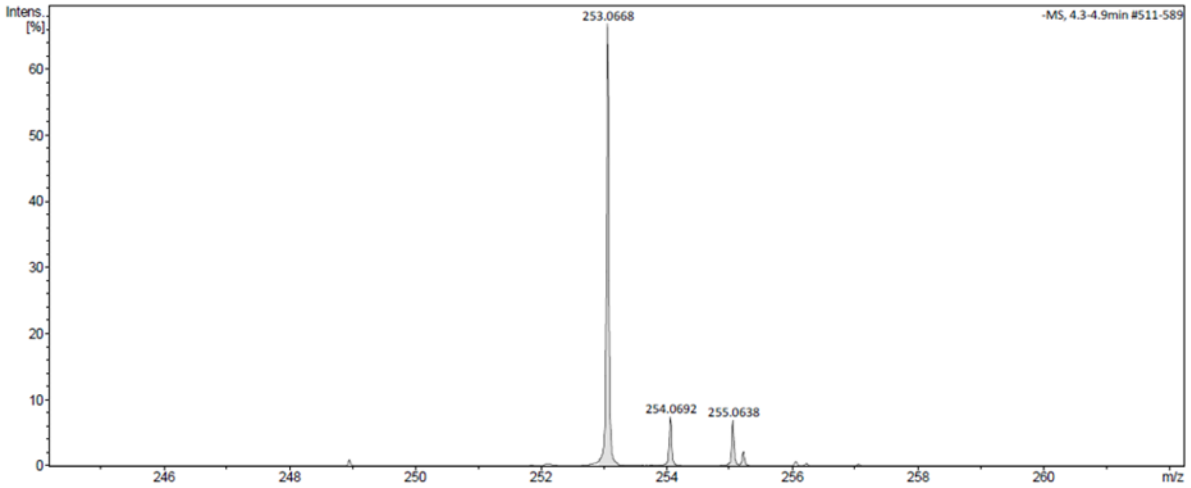

Figure S151 - A high-resolution mass spectrum (ESI$^-$) obtained for compound **45** in methanol.

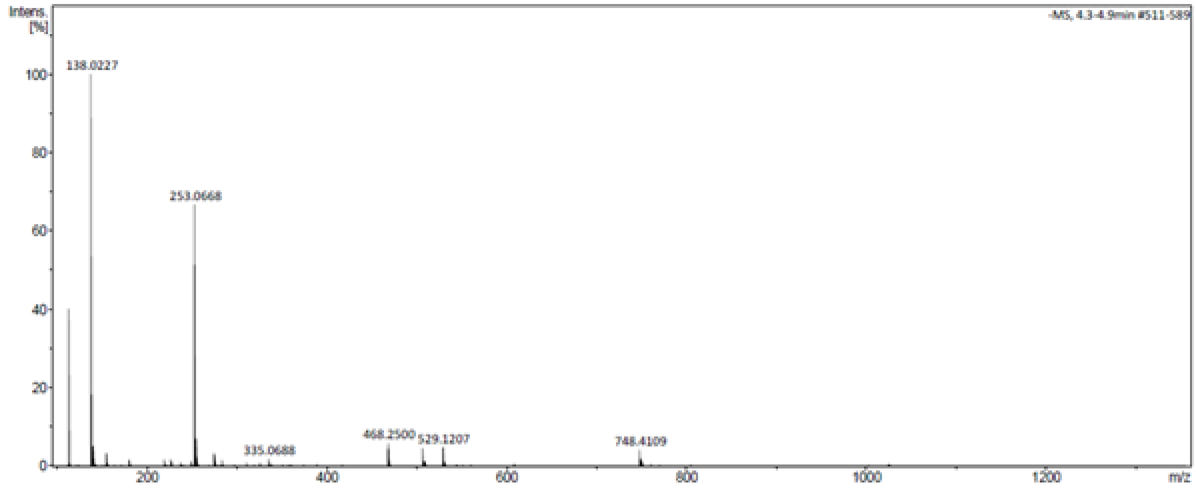

Figure S152- A high-resolution mass spectrum (ESI$^-$) obtained for dimeric species of compound **45** in methanol, *m/z* [M + M + Na$^+$]$^-$.

Overview

Table S14 – High resolution ESI$^-$ mass spectrometry theoretical and experimentally derived values.

Compound	m/z [M]$^-$		m/z [M + M + H]$^-$	
	Theoretical	Actual	Theoretical	Actual
**14**	312.9934	312.9934	625.9868	626.9942
**15**	312.9934	312.9933	625.9868	626.9936
**16**	312.9934	312.9914	625.9868	626.9900
**17**	312.9934	312.9907	625.9868	626.9884
**18**	312.9934	312.9911	625.9868	626.9885
**23**	365.0036	365.0048	730.0072	731.0164
**27**	380.9808	380.9813	761.9616	762.9681
**40**	209.0590	209.0587	418.1180	468.2489
**41**	223.0740	223.0742	446.1480	447.1530
**42**	237.0890	237.0899	474.1780	475.1846
**43**	225.0360	225.0360	450.0720	451.0768
**44**	239.0510	239.0508	478.1020	479.1060
**45**	253.0670	253.0668	506.1340	529.1207[a]

a – obtained as the [M + M + Na]$^-$ ion.

Screening Growth Curves

Compound screening was conducted using compounds at 3.3 mM

USA 300 Methicillin-resistant *Staphylococcus aureus* (MRSA)

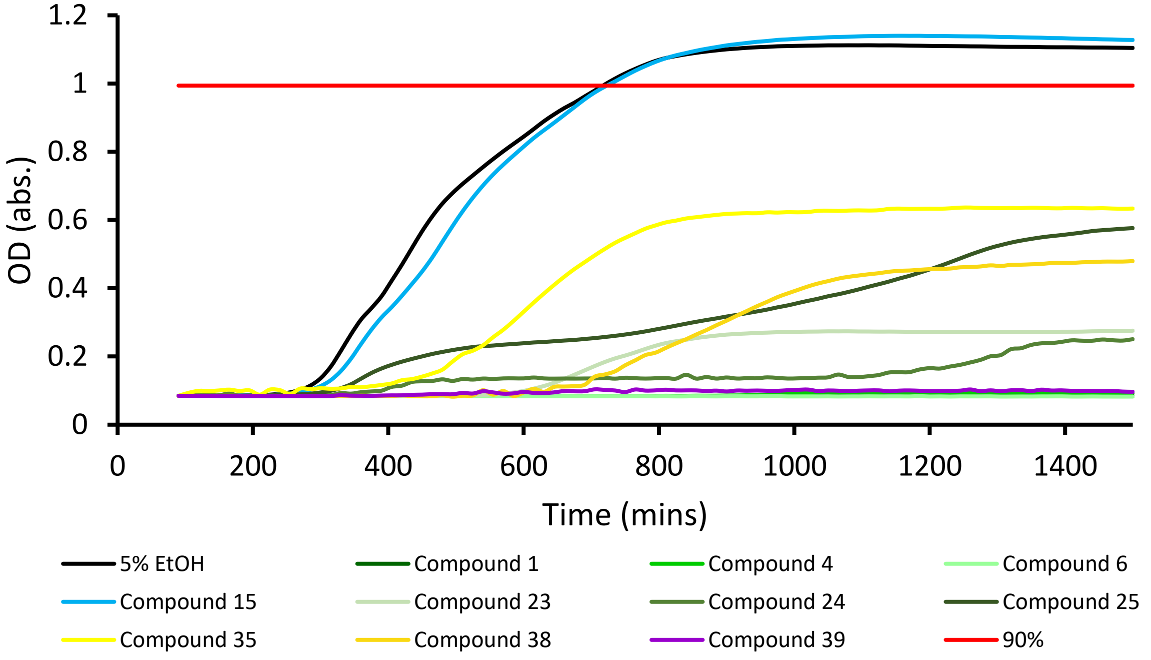

Figure S153 - MRSA growth curves created from an average of 6 optical density readings in the presence of compounds **1, 4**, **6**, **15**, **23**, **24**, **25**, **35**, **38** and **39**.

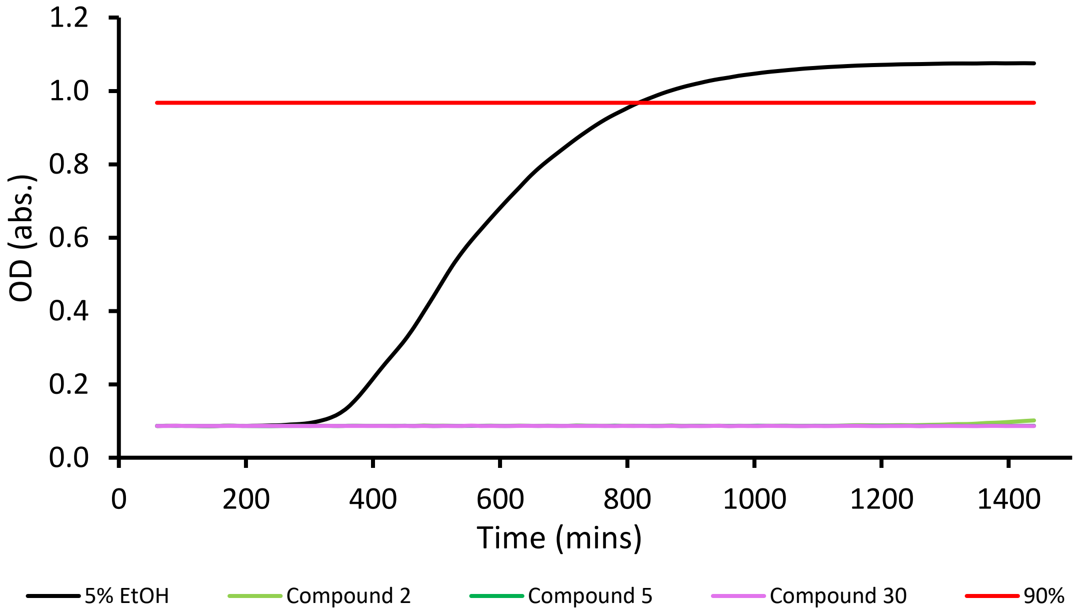

Figure S154 **-** MRSA growth curves created from an average of 6 optical density readings in the presence of compounds **2, 5,** and **30**.

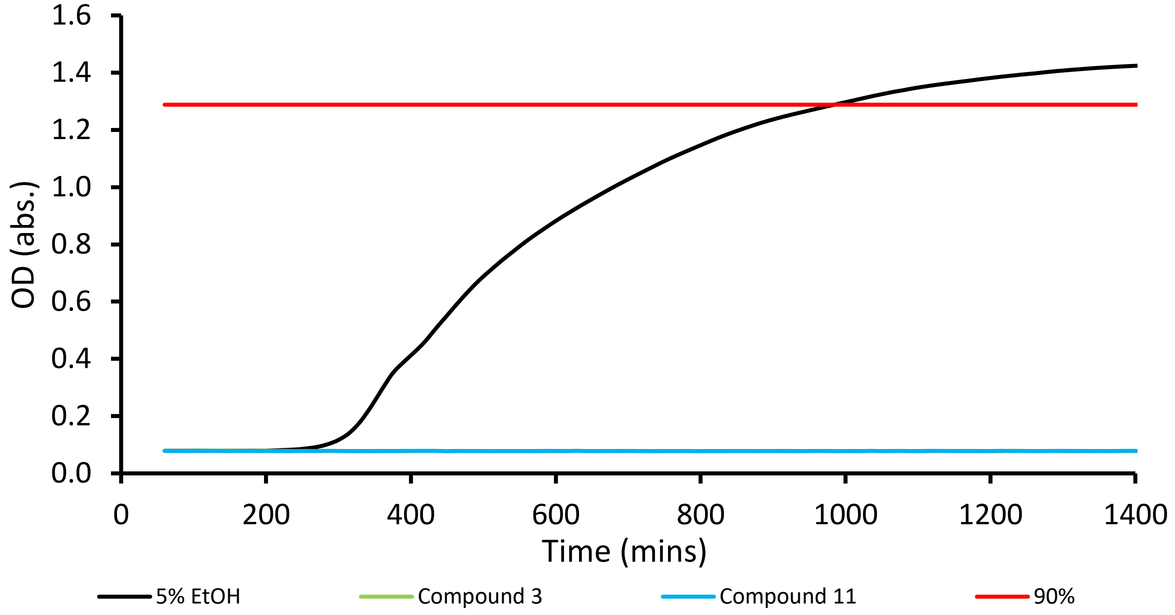

Figure S155 **-** MRSA growth curves created from an average of 6 optical density readings in the presence of compounds **3** and **11.**

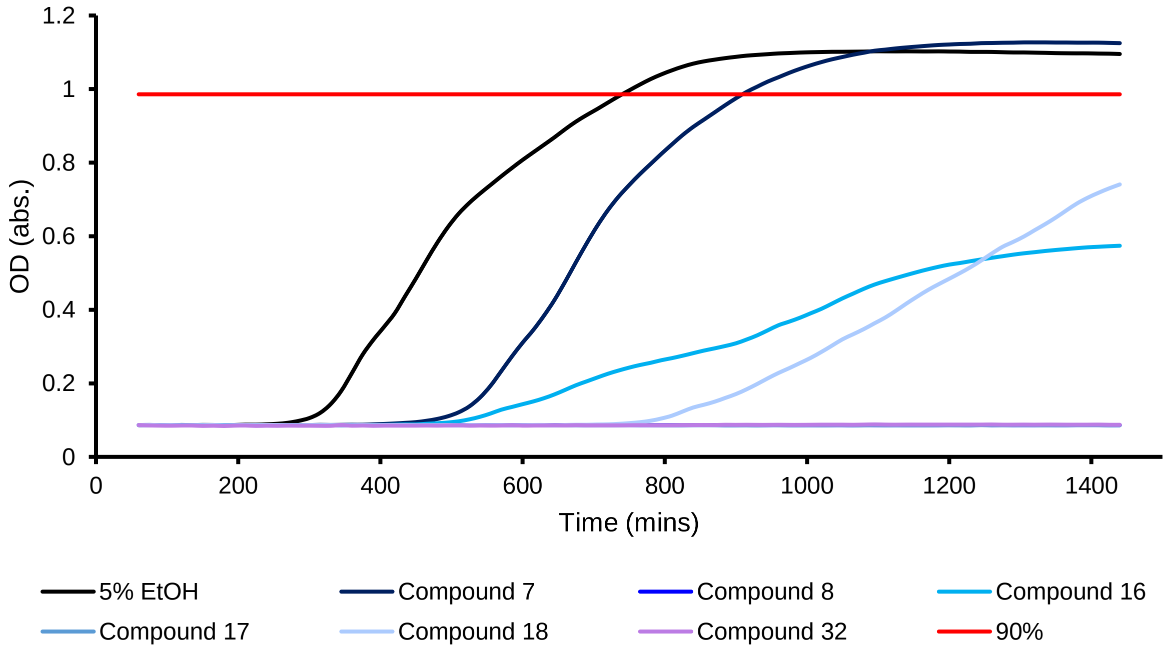

Figure S156 - MRSA growth curves created from an average of 6 optical density readings in the presence of compounds **7**, **8**, **16**, **17**, **18,** and **32**.

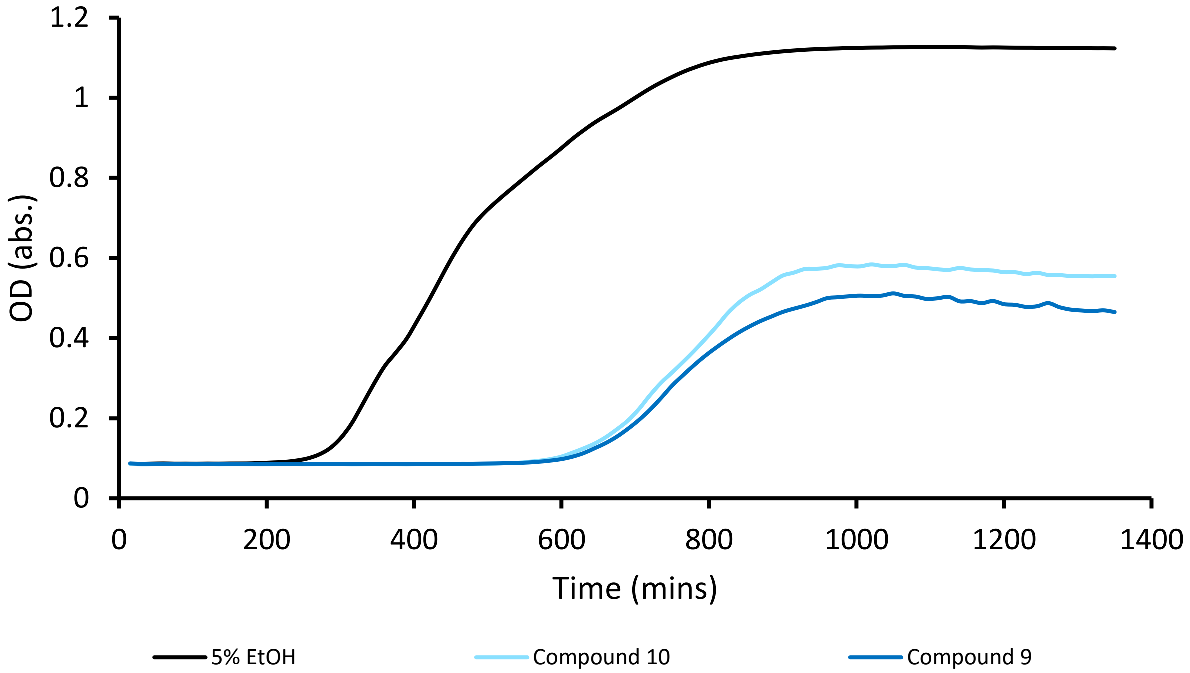

Figure S157 - MRSA growth curves created from an average of 6 optical density readings in the presence of compounds **9 and 10**.

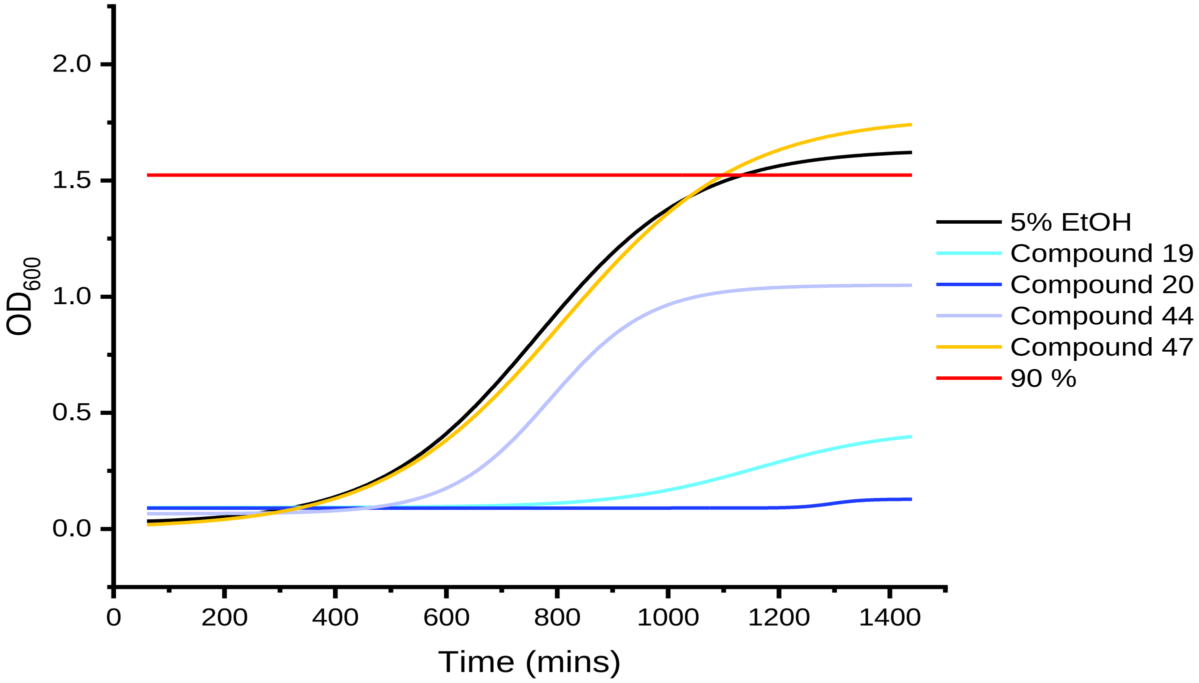

Figure S158 - MRSA growth curves created from an average of 6 optical density readings in the presence of compounds **19, 20, 44** and **47**.

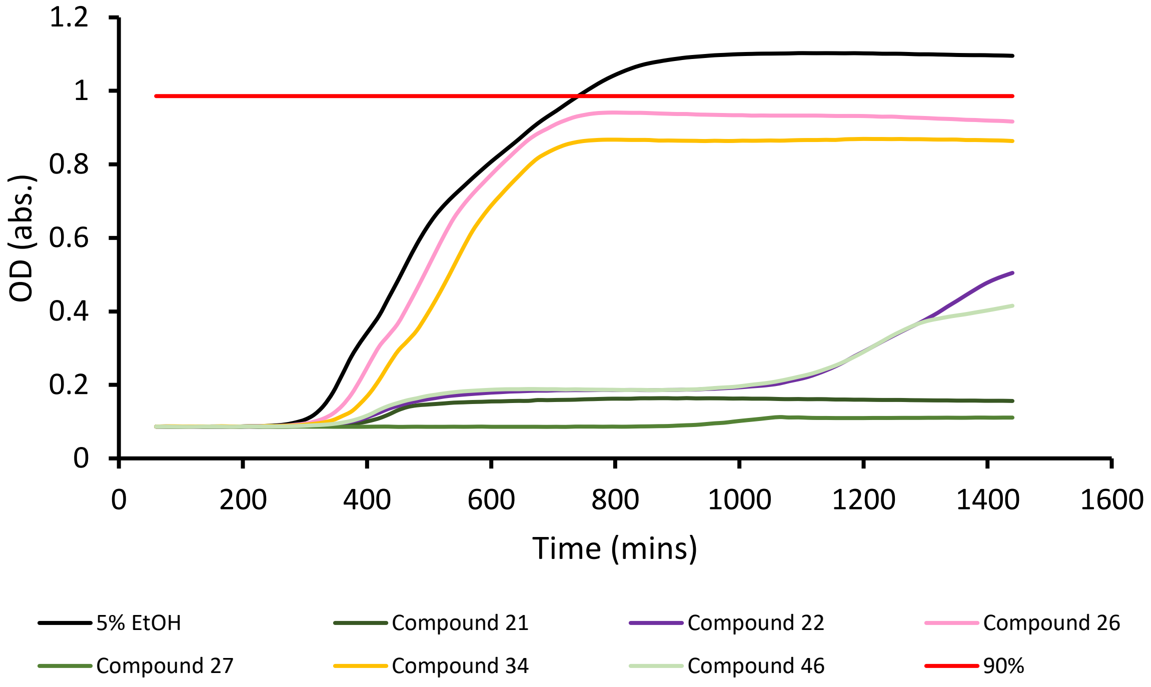

Figure S159 - MRSA growth curves created from an average of 6 optical density readings in the presence of compounds **21**, **22**, **26**, **27**, **34** and **46**.

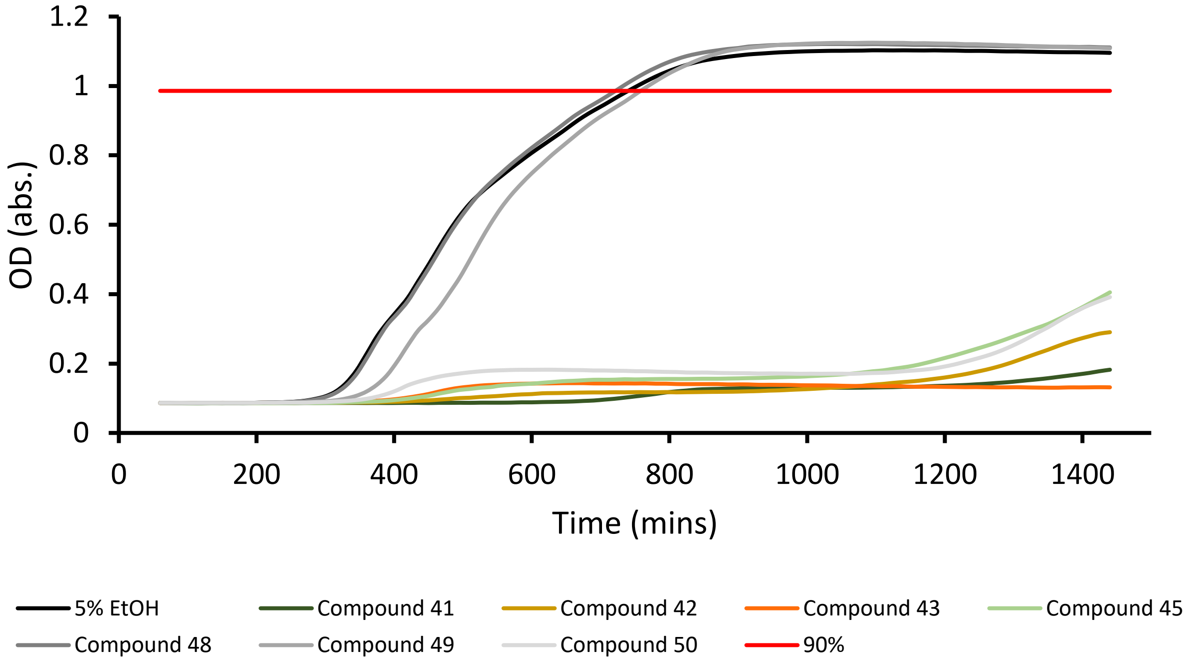

Figure S160 - MRSA growth curves created from an average of 6 optical density readings in the presence of compounds **41**, **42**, **43**, **45**, **48**, **49** and **50**.

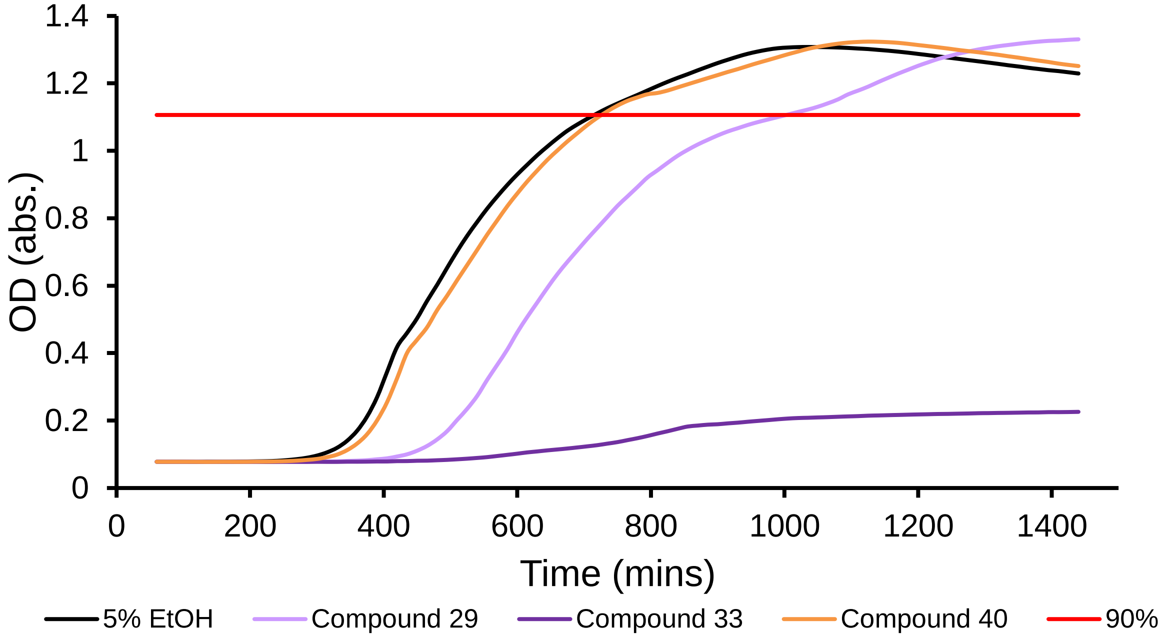

Figure S161 - MRSA growth curves created from an average of 6 optical density readings in the presence of compounds **29, 33** and **40**.

***Escherichia coli* (*E. coli*)**

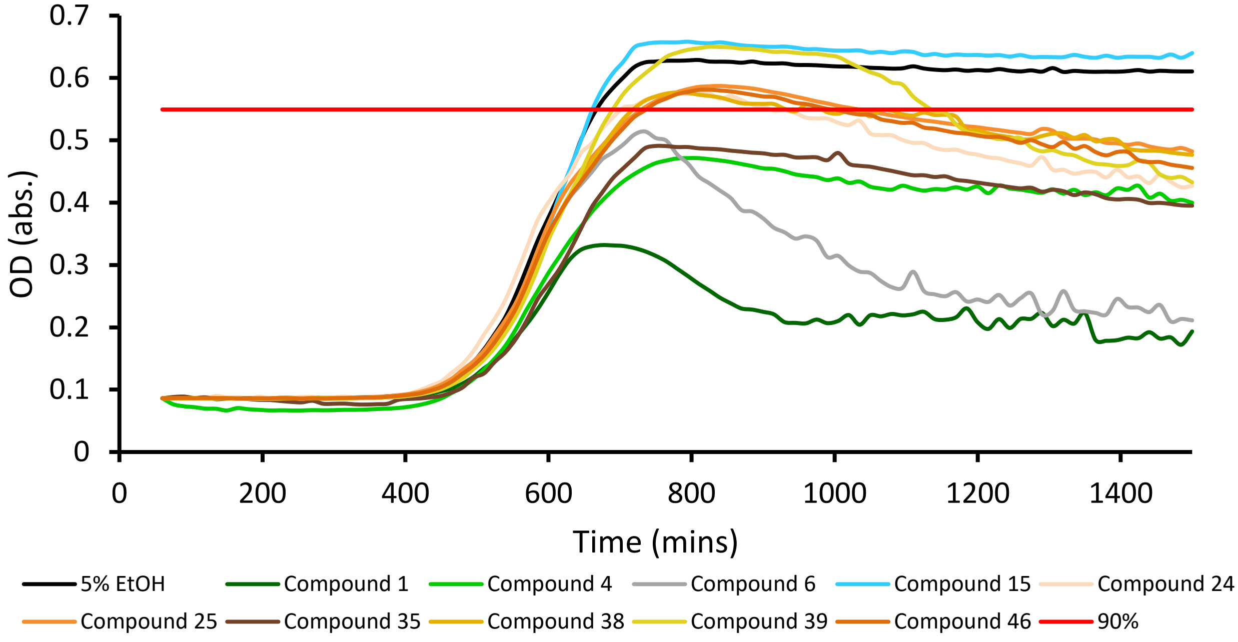

Figure S162 - *E. coli* DH10B growth curves created from an average of 6 optical density readings in the presence of compounds **1, 4**, **6**, **15**, **24**, **25**, **35**, **38, 39** and **46**.

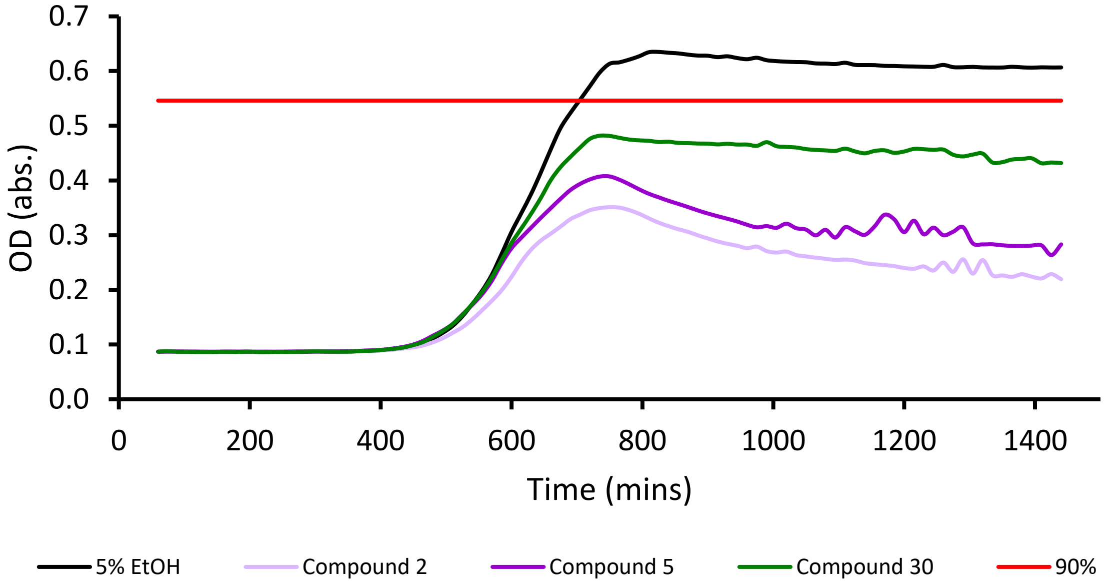

Figure S163 - *E. coli* DH10B growth curves created from an average of 6 optical density readings in the presence of compounds **2**, **5**, and **30.**

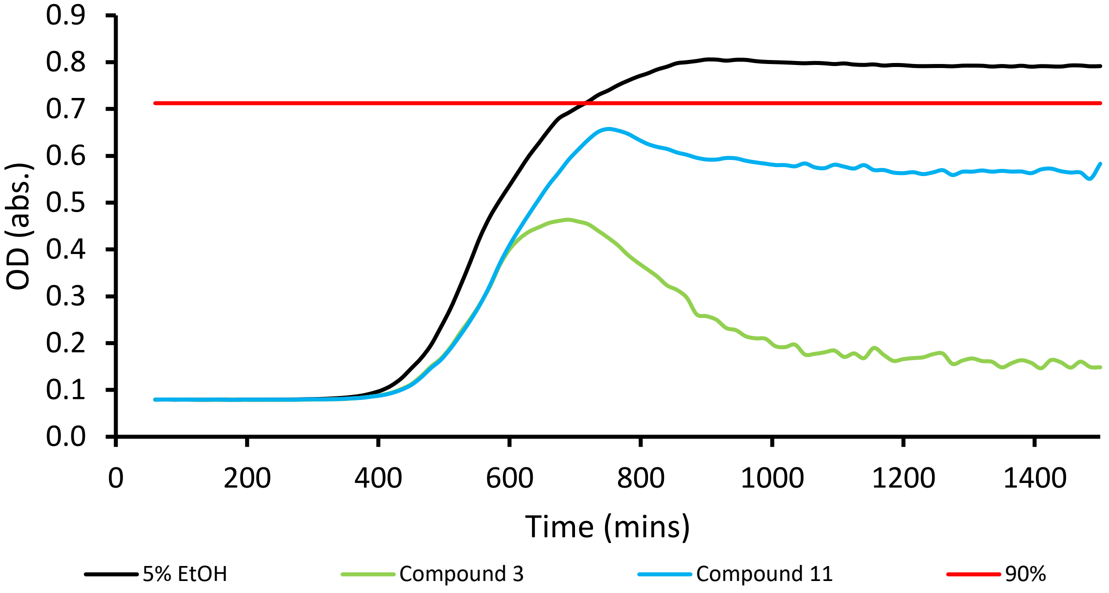

Figure S164 - *E. coli* DH10B growth curves created from an average of 6 optical density readings in the presence of compounds **3** and **11.**

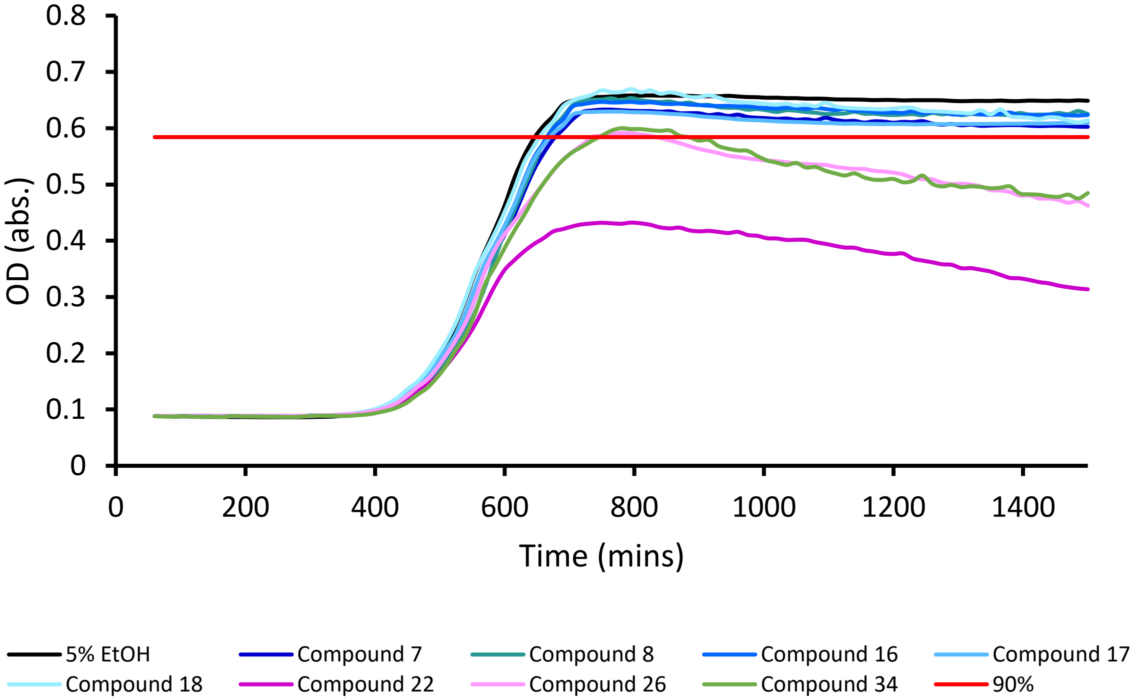

Figure S165 **-** *E. coli* DH10B growth curves created from an average of 6 optical density readings in the presence of compounds **7**, **8**, **16**, **17**, **18**, **22**, **26** and **34**.

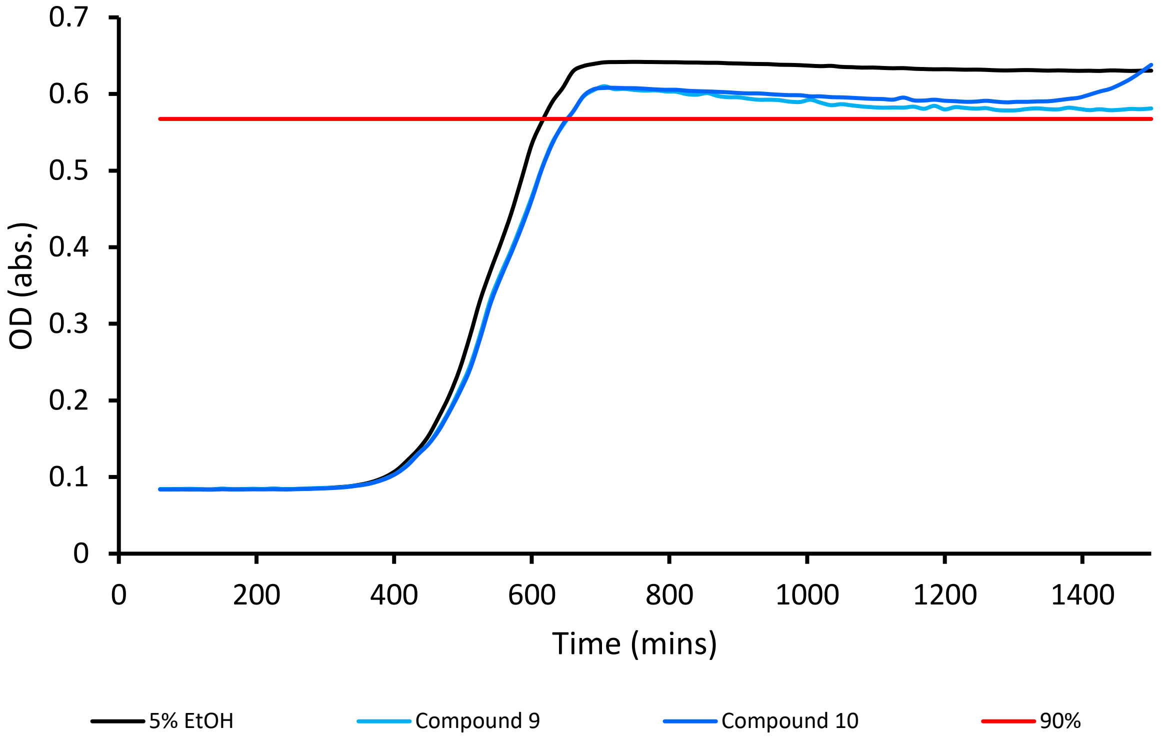

Figure S166 - *E. coli* DH10B growth curves created from an average of 6 optical density readings in the presence of compounds **9*** and **10.**

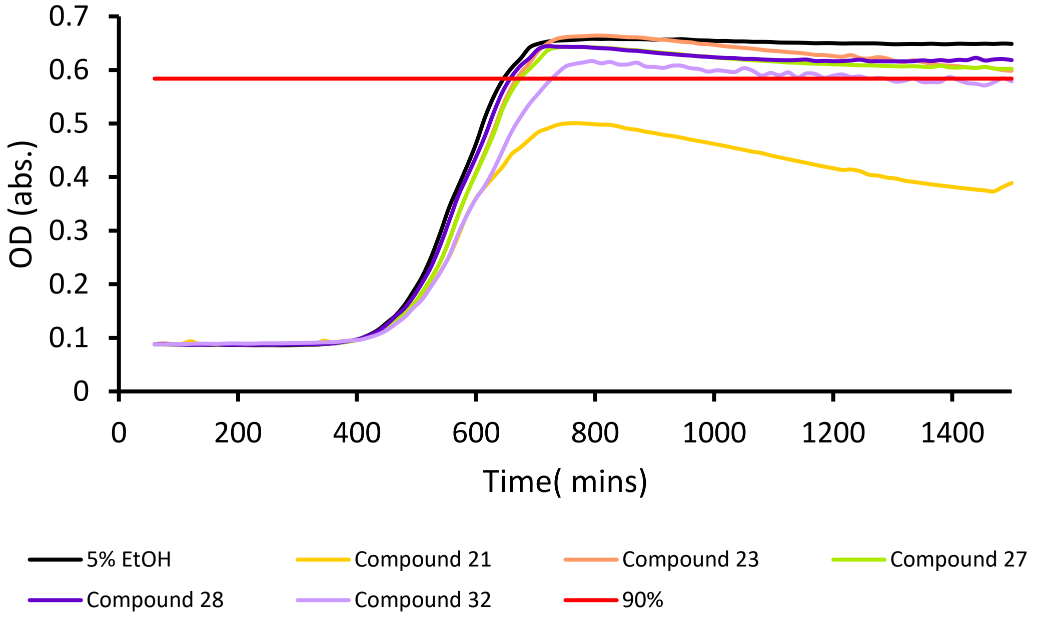

Figure S167 - *E. coli* DH10B growth curves created from an average of 6 optical density readings in the presence of compounds **21**, **23**, **27**, **28** and **32**.

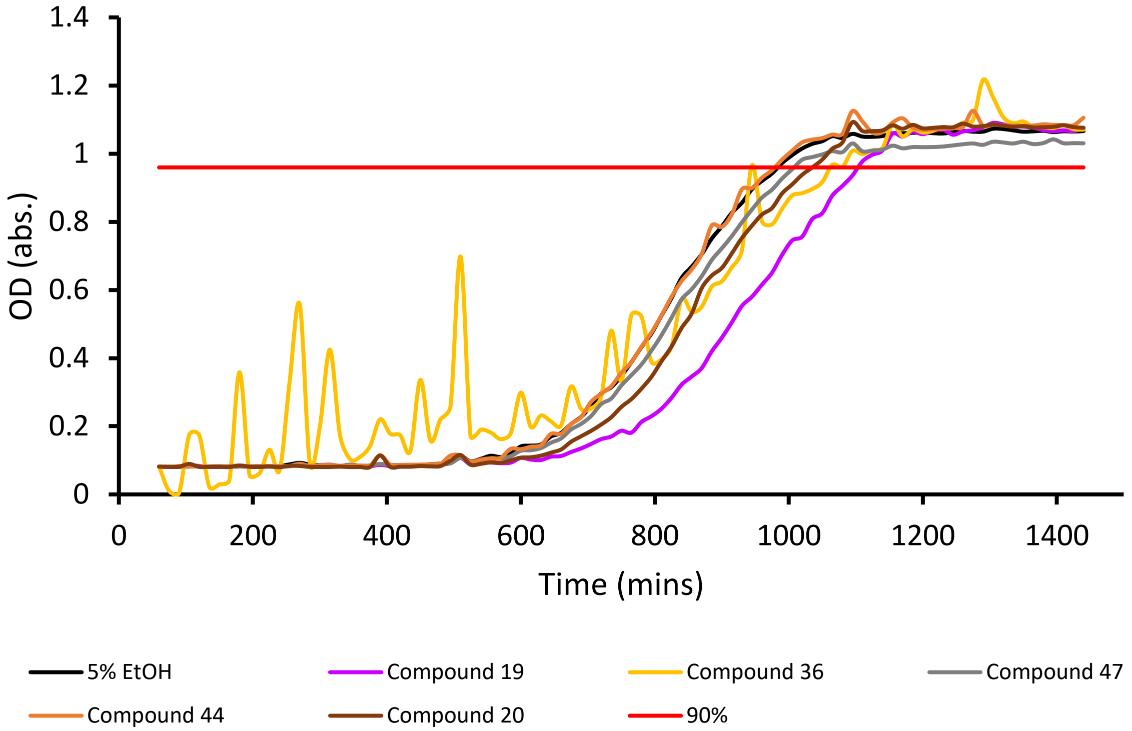

Figure S168 - *E. coli* DH10B growth curves created from an average of 6 optical density readings in the presence of compounds **19**, **36**, **47**, **44** and **20.**

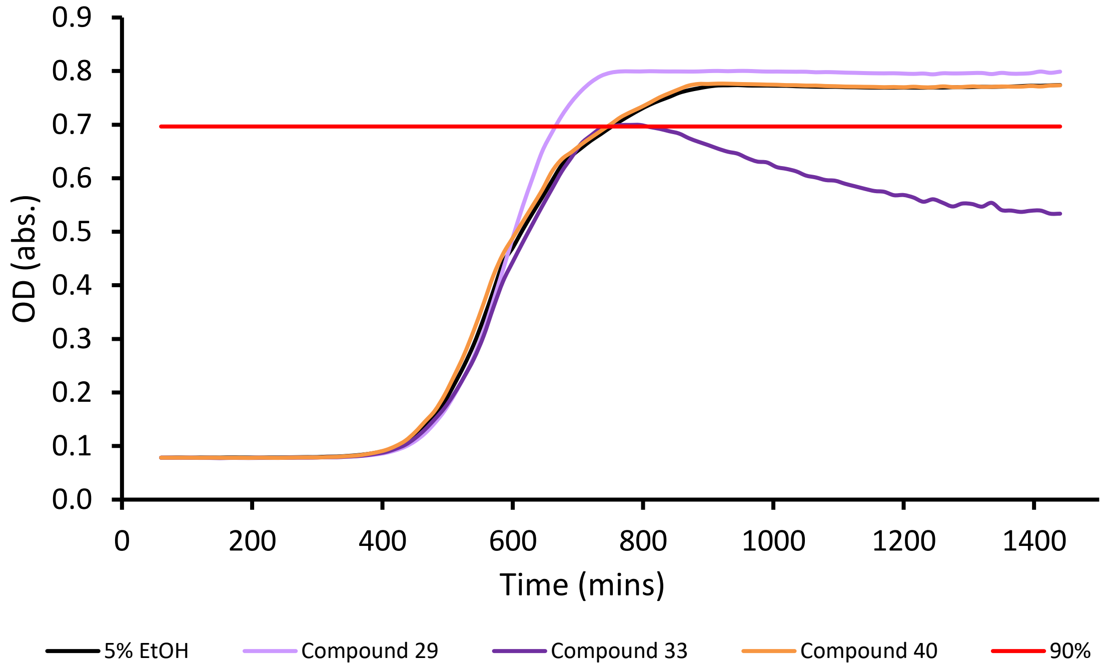

Figure S169 - *E. coli* DH10B growth curves created from an average of 6 optical density readings in the presence of compounds **29, 33** and **40.** MIC_{50} not calculated for compound **33** due to solubility of compound.

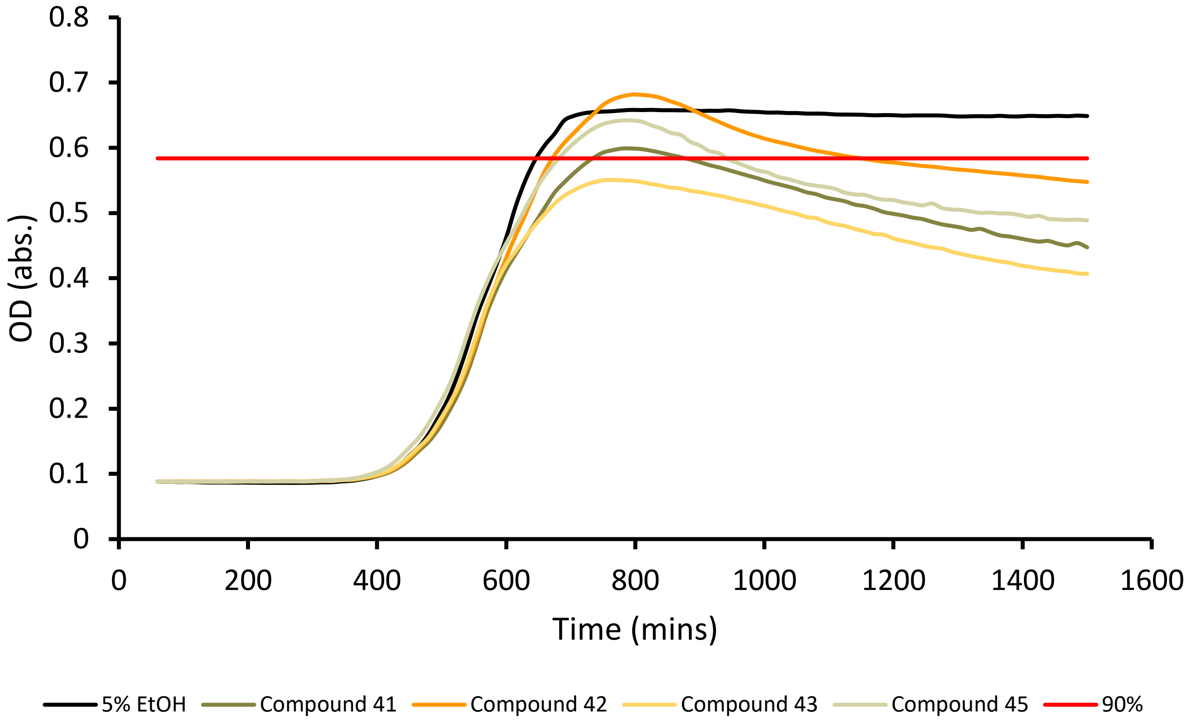

Figure S170 **-** *E. coli* DH10B growth curves created from an average of 6 optical density readings in the presence of compounds **41**, **42**, **43** and **45**.

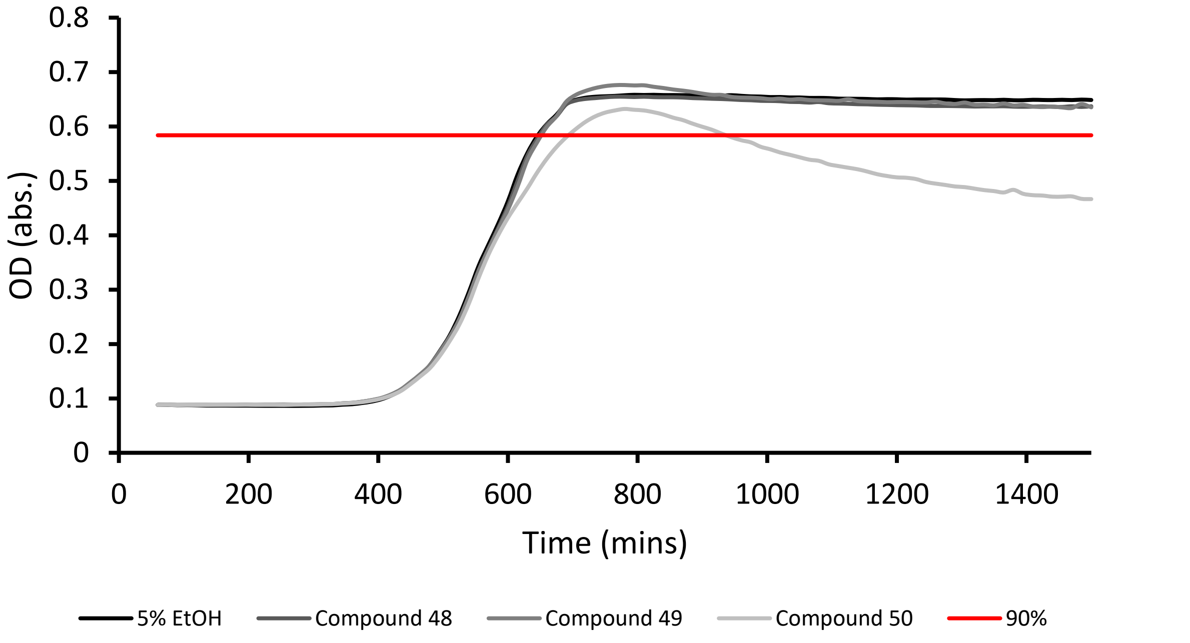

Figure S171 - *E. coli* DH10B growth curves created from an average of 6 optical density readings in the presence of compounds **48**, **49**, and **50**.

MIC$_{50}$ Growth Curves

USA 300 Methicillin-resistant *Staphylococcus aureus* (MRSA)

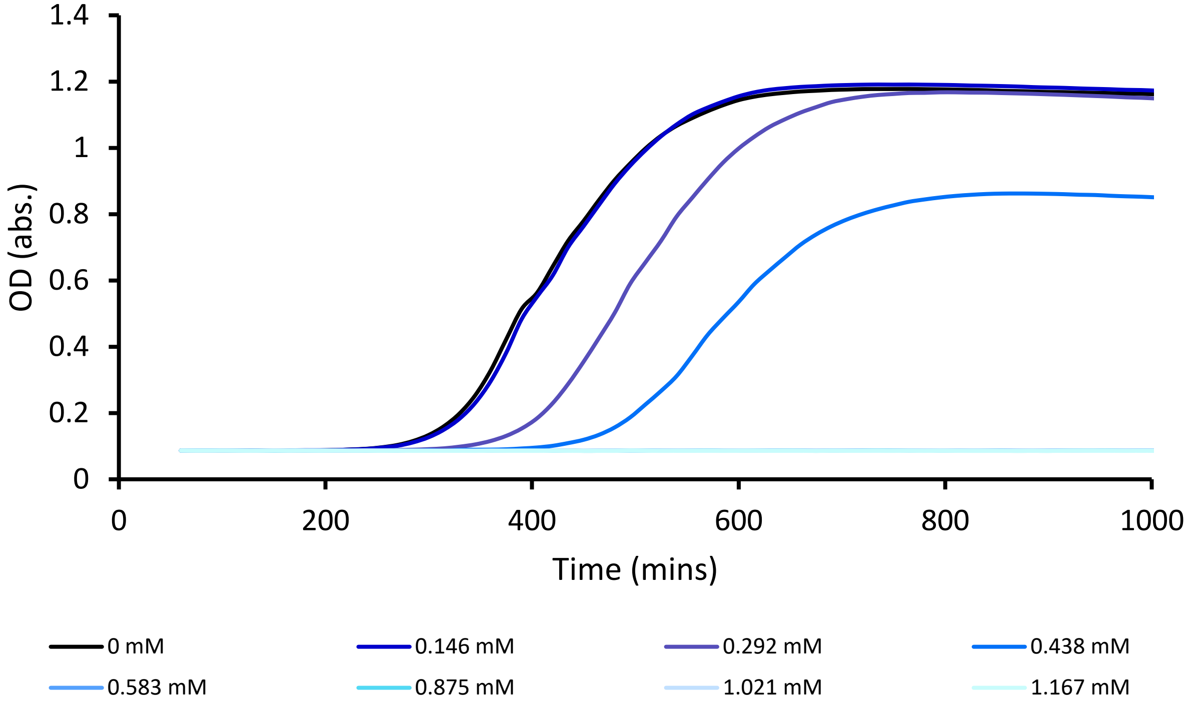

Figure S172 - MRSA growth curves created from an average of 6 optical density readings for each concentration in the presence of compound **1** at varying concentrations.

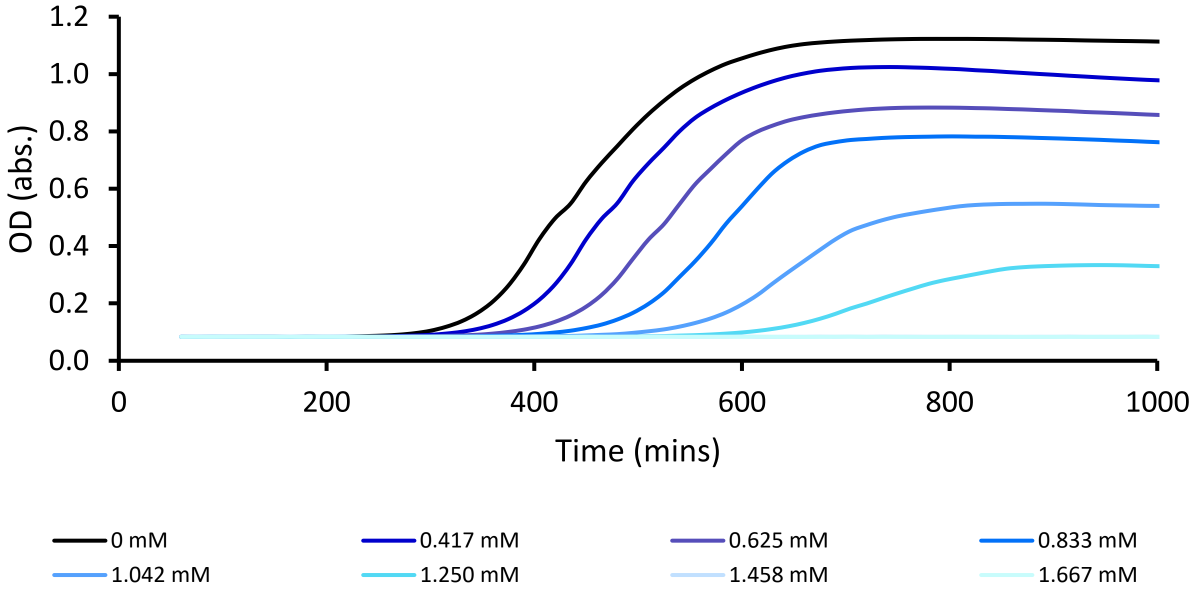

Figure S173 - MRSA growth curves created from an average of 6 optical density readings for each concentration in the presence of compound **2** at varying concentrations.

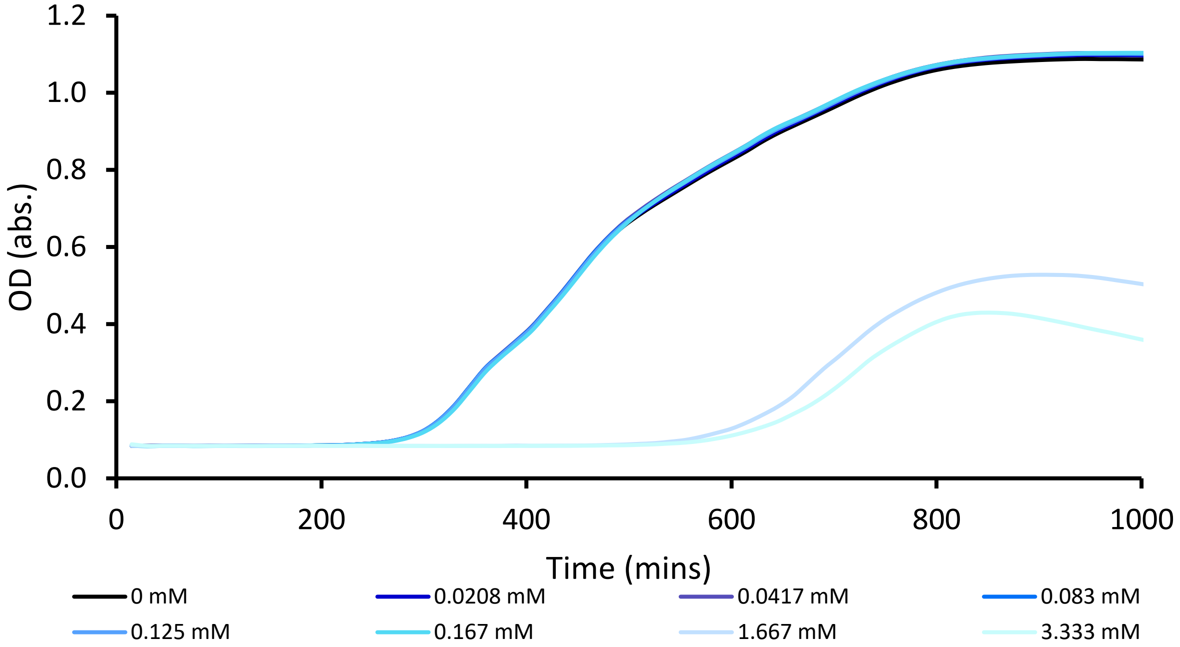

Figure S174 - MRSA growth curves created from an average of 6 optical density readings for each concentration in the presence of compound **3** at varying concentrations. Due to crystallization of compound, a MIC_{50} calculation was not possible.

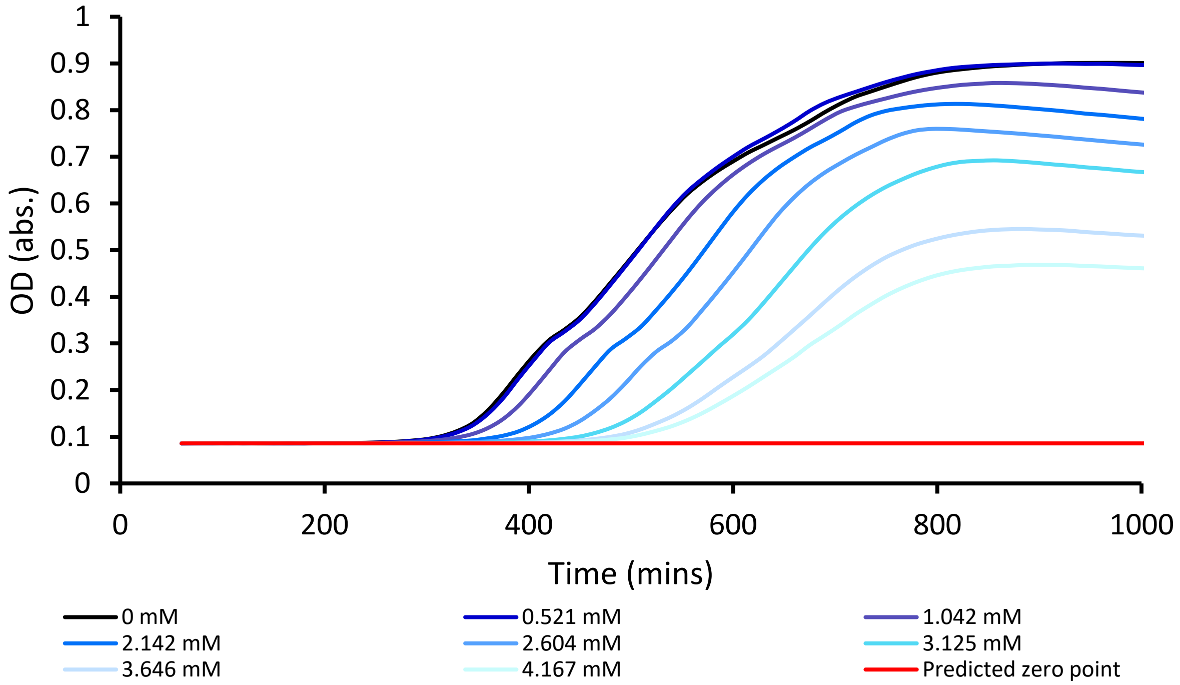

Figure S175 - MRSA growth curves created from an average of 6 optical density readings for each concentration in the presence of compound **4** at varying concentrations. Predicted zero point used in MIC_{50} calculation due to solubility of compound.

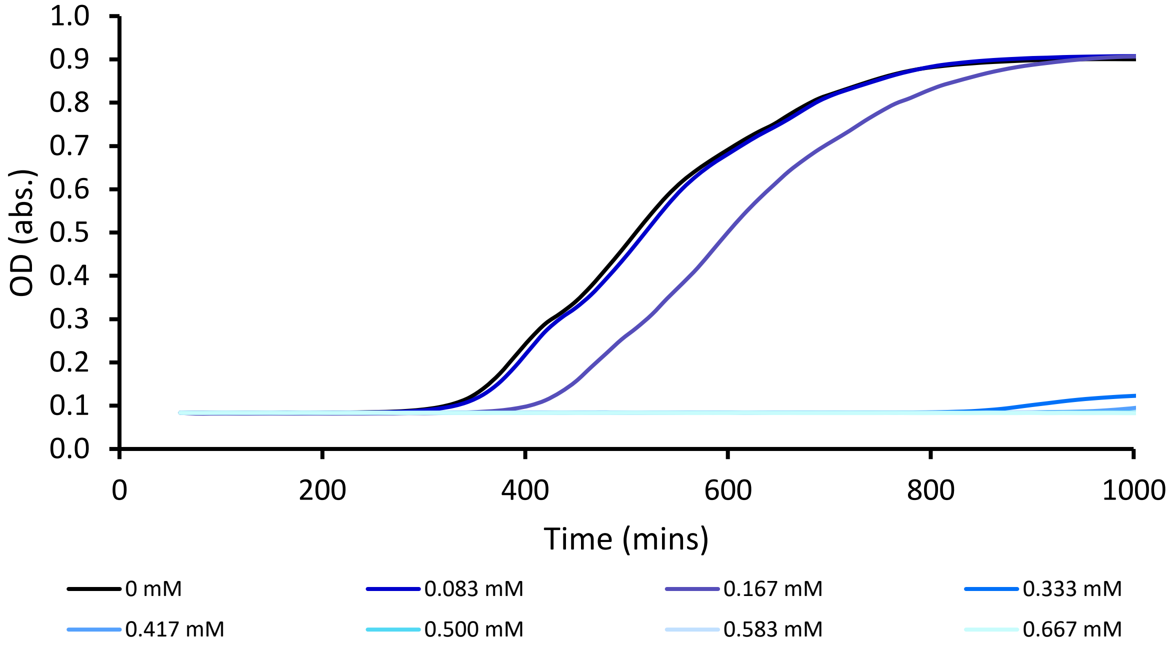

Figure S176 - MRSA growth curves created from an average of 6 optical density readings for each concentration in the presence of compound **5** at varying concentrations.

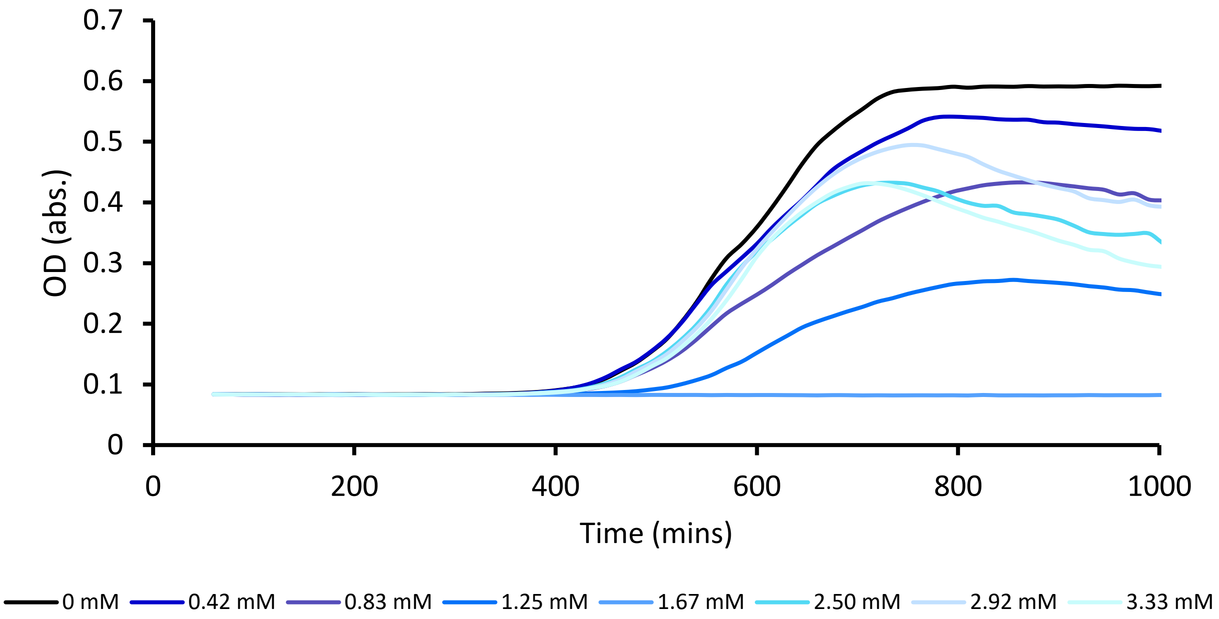

Figure S177 – MRSA growth curves created from an average of 6 optical density readings for each concentration in the presence of compound **6** at varying concentrations. MIC_{50} calculated using predicted end point. Due to crystallization of compound at higher concentrations, 2.50, 2.92 and 3.3 mM were omitted from the MIC_{50} calculation.

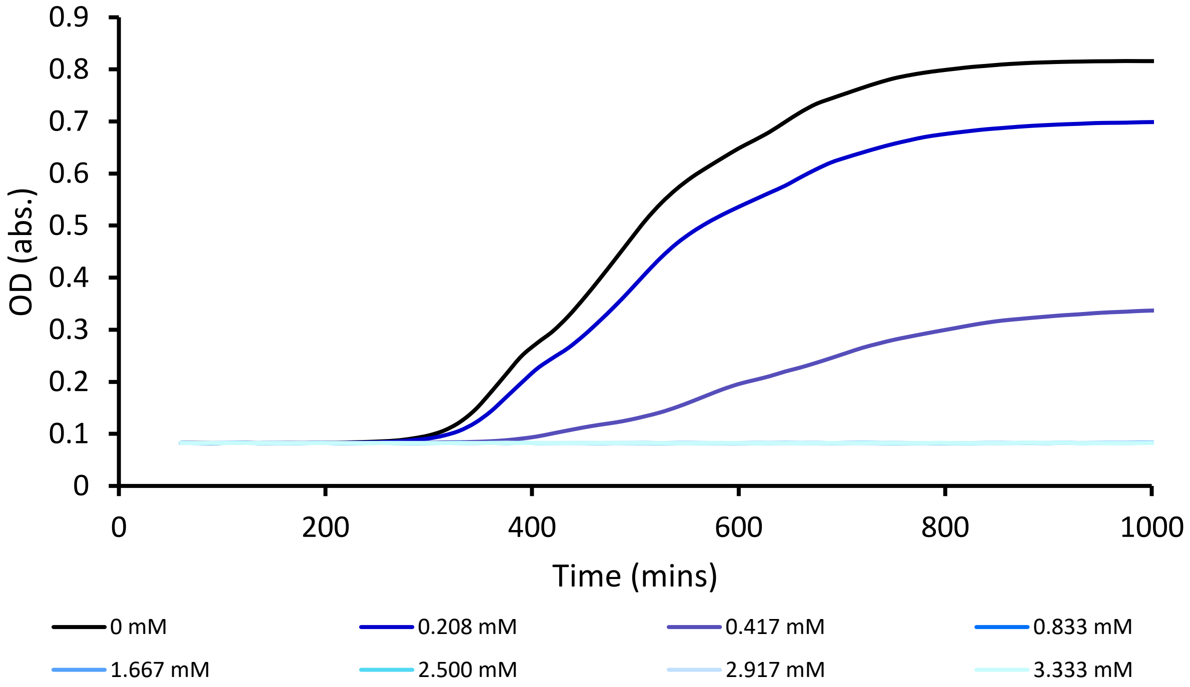

Figure S178 - MRSA growth curves created from an average of 6 optical density readings for each concentration in the presence of compound **8** at varying concentrations.

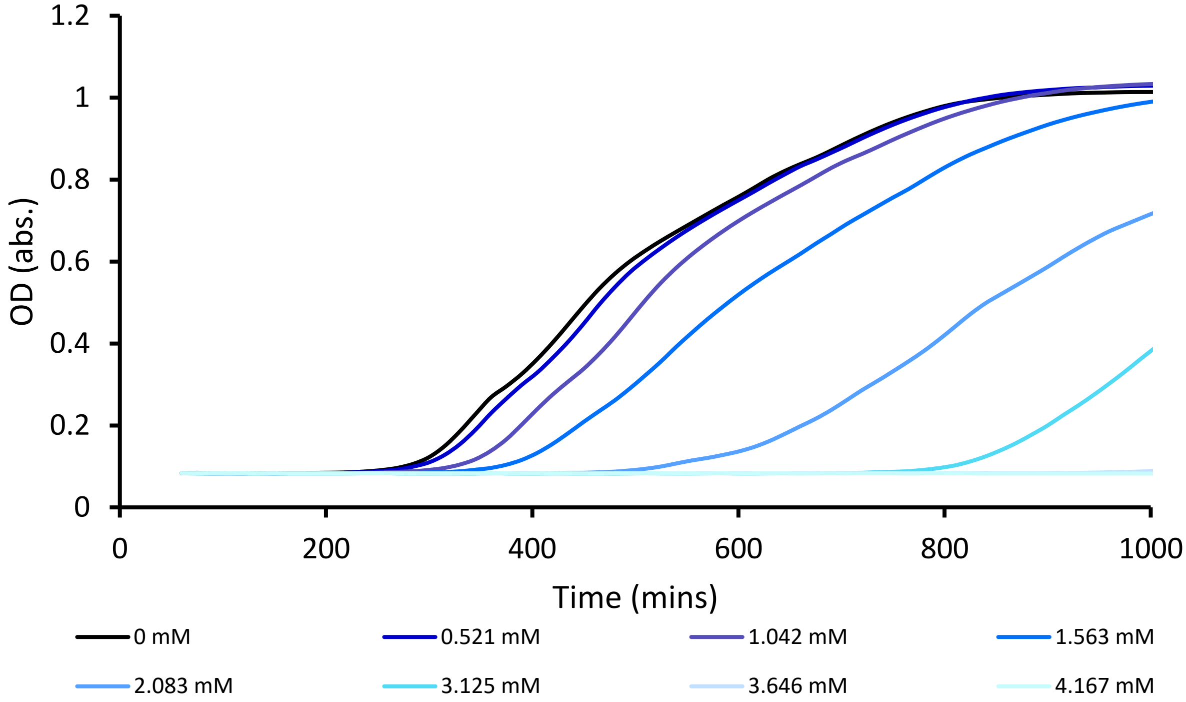

Figure S179 - MRSA growth curves created from an average of 6 optical density readings for each concentration in the presence of compound **9** at varying concentrations.

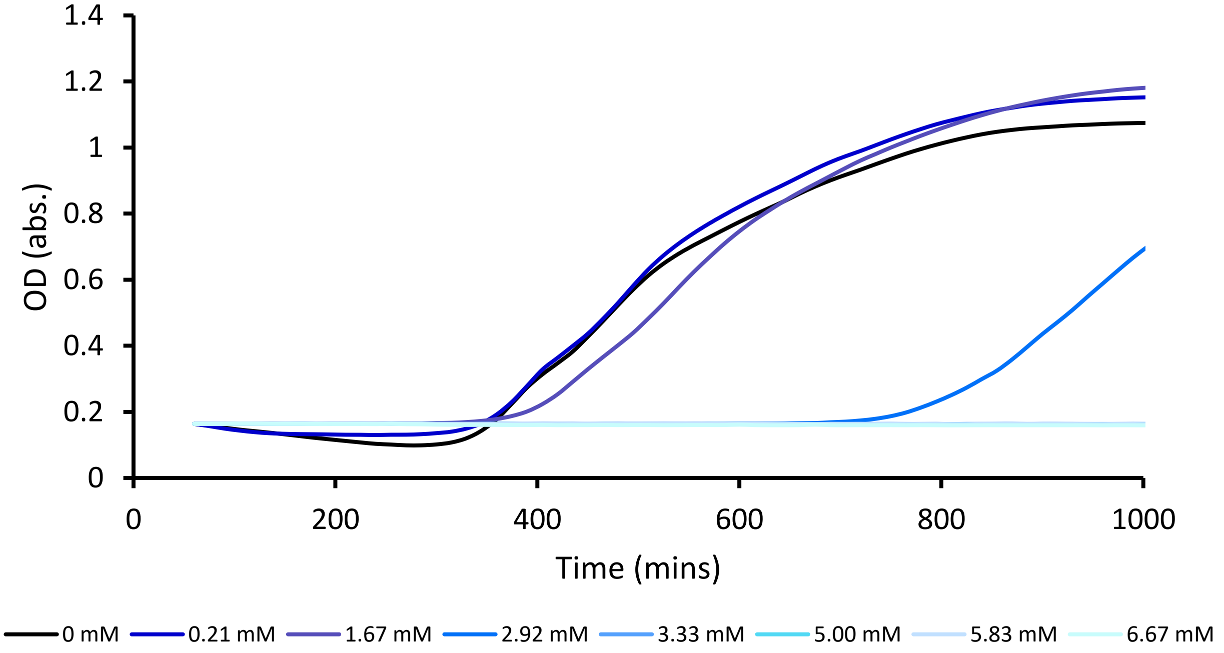

Figure S180 - MRSA growth curves created from an average of 6 optical density readings for each concentration in the presence of compound **10** at varying concentrations.

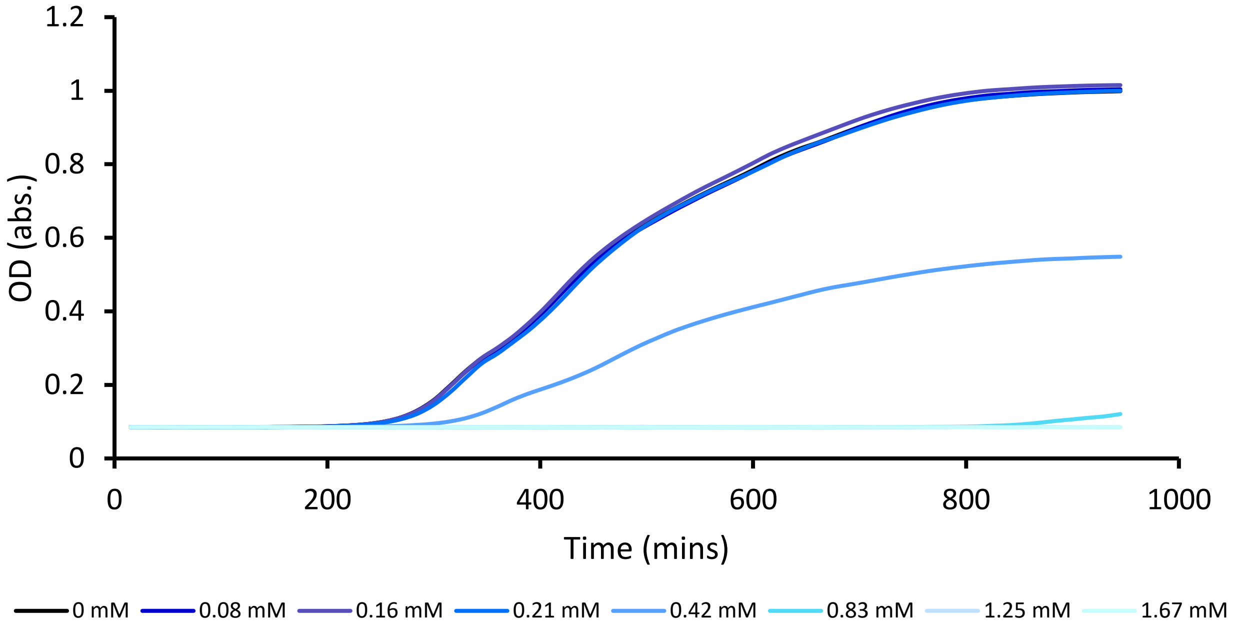

Figure S181 - MRSA growth curves created from an average of 6 optical density readings for each concentration in the presence of compound **11** at varying concentrations.

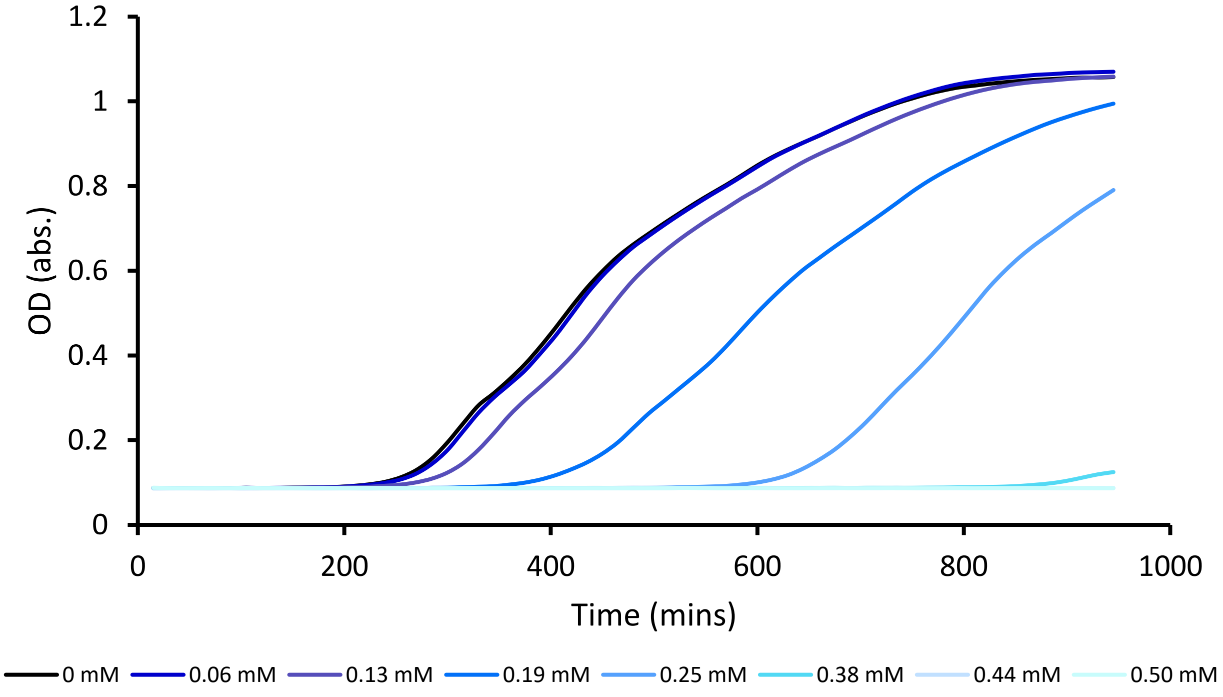

Figure S182 - MRSA growth curves created from an average of 6 optical density readings for each concentration in the presence of compound **16** at varying concentrations.

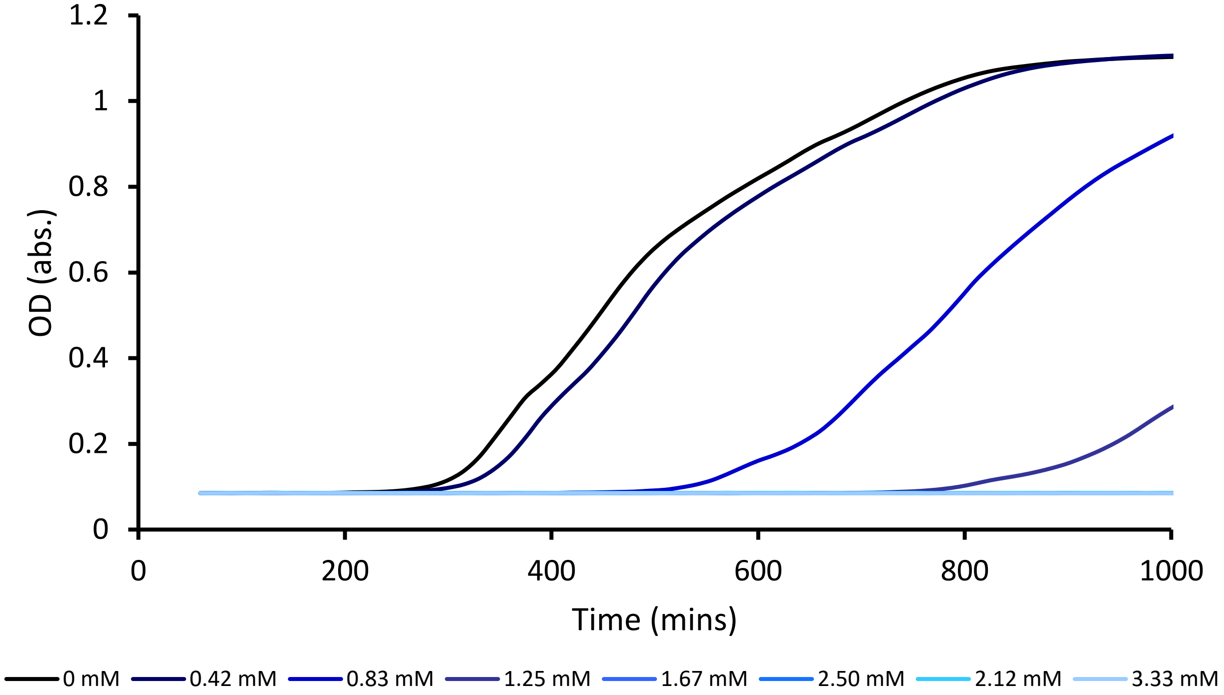

Figure S183 - MRSA growth curves created from an average of 6 optical density readings for each concentration in the presence of compound **17** at varying concentrations.

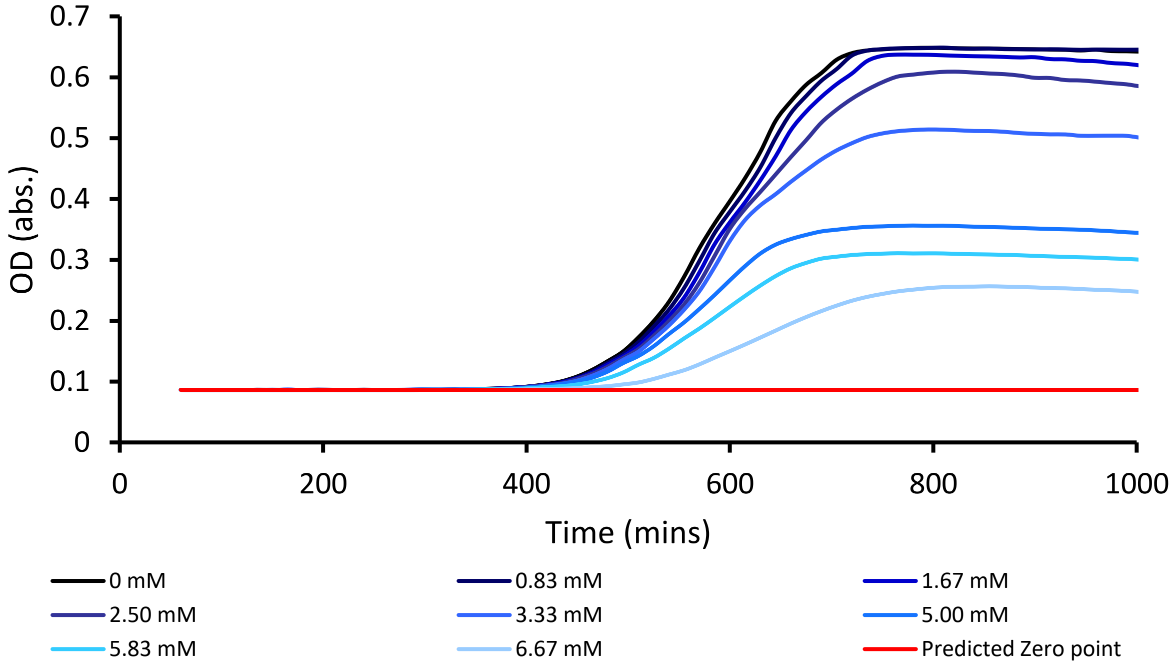

Figure S184 - MRSA growth curves created from an average of 6 optical density readings for each concentration in the presence of compound **18** at varying concentrations. Predicted zero point used in MIC_{50} calculation due to the solubility of compound.

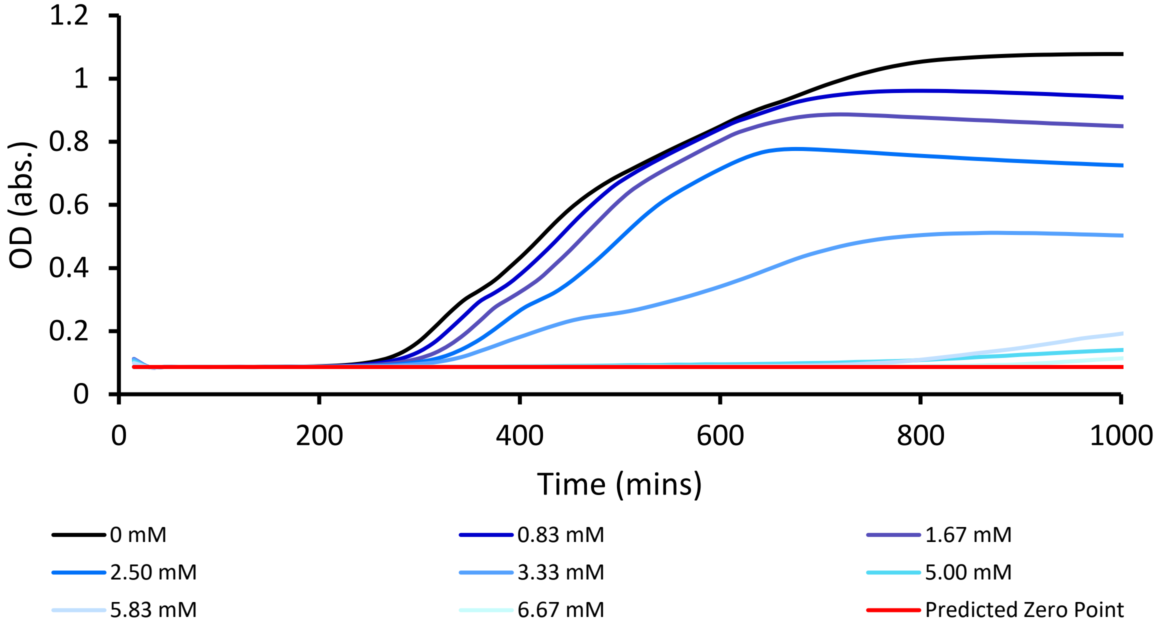

Figure S185 - MRSA growth curves created from an average of 6 optical density readings for each concentration in the presence of compound **19** at varying concentrations. Predicted zero point used in MIC_{50} calculation due to the solubility of compound.

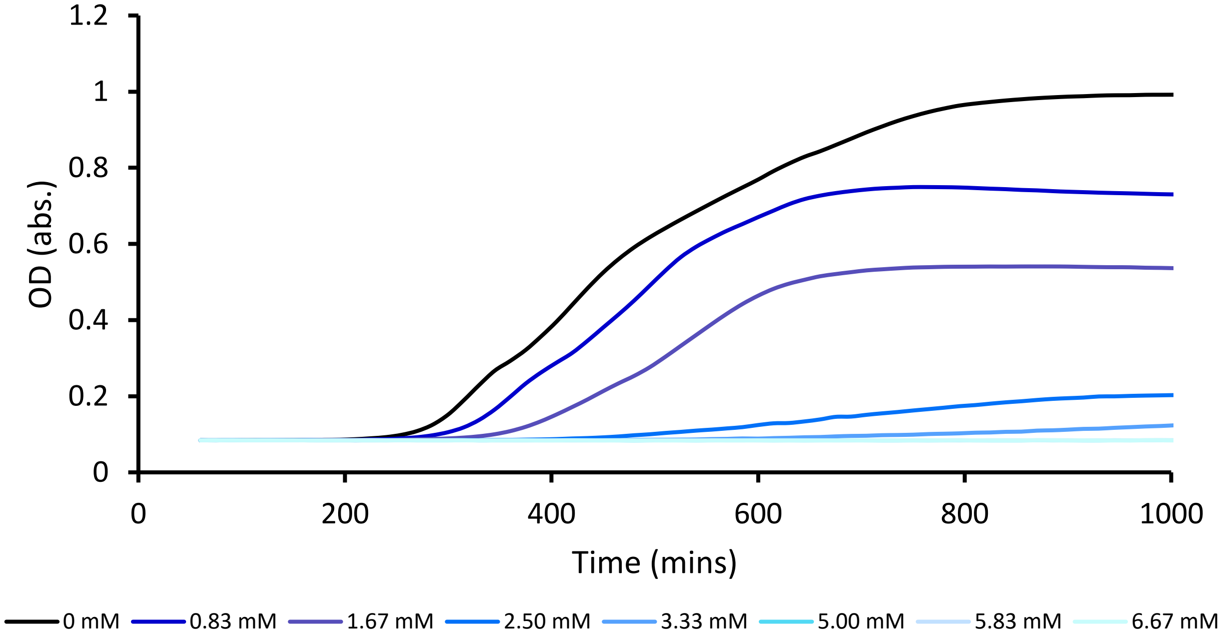

Figure S186 - MRSA growth curves created from an average of 6 optical density readings for each concentration in the presence of compound **20** at varying concentrations.

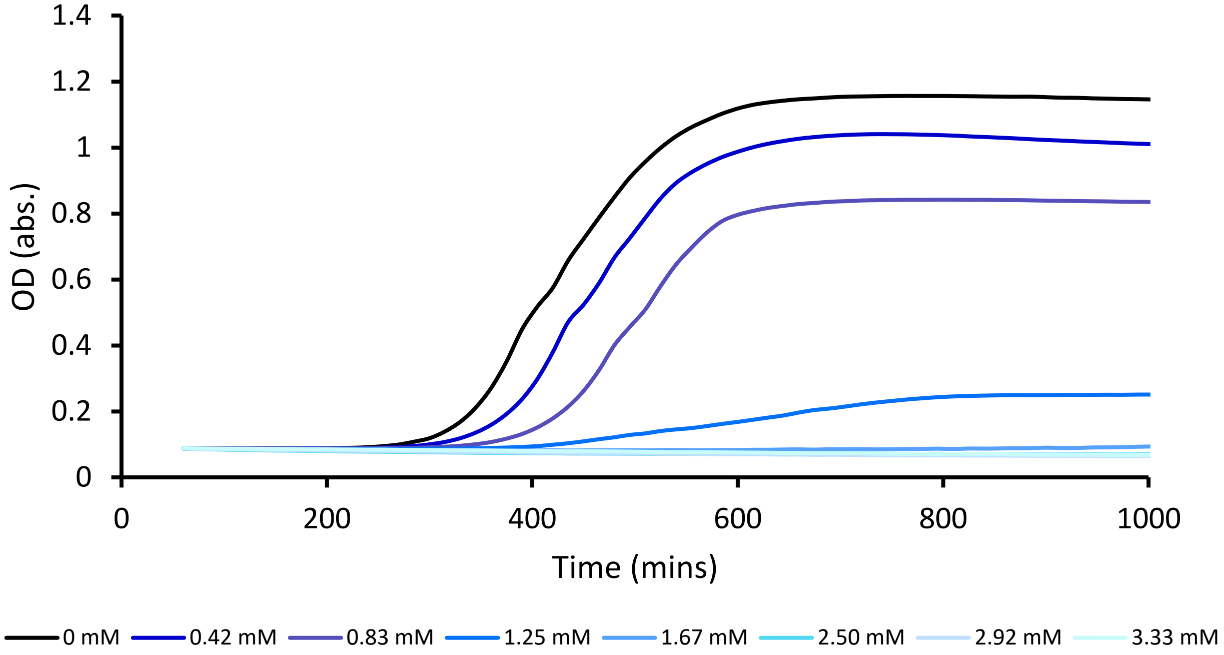

Figure S187 - MRSA growth curves created from an average of 6 optical density readings for each concentration in the presence of compound **21** at varying concentrations.

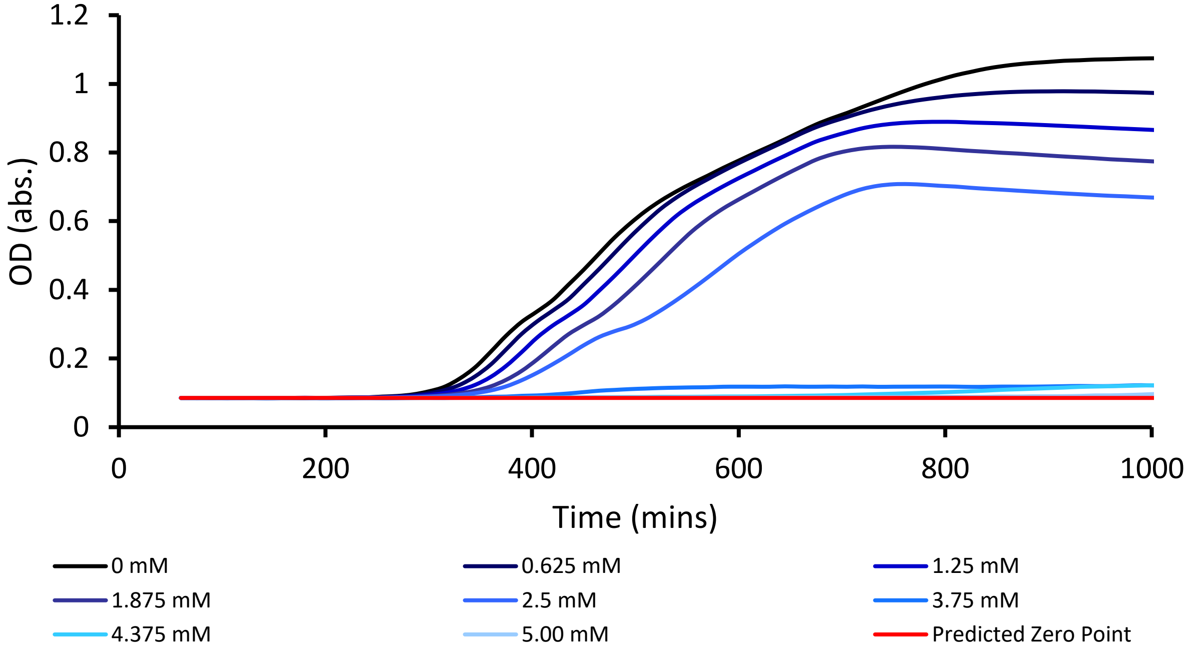

Figure S188 - MRSA growth curves created from an average of 6 optical density readings for each concentration in the presence of compound **22** at varying concentrations. Predicted zero point used in MIC_{50} calculation due to the solubility of compound.

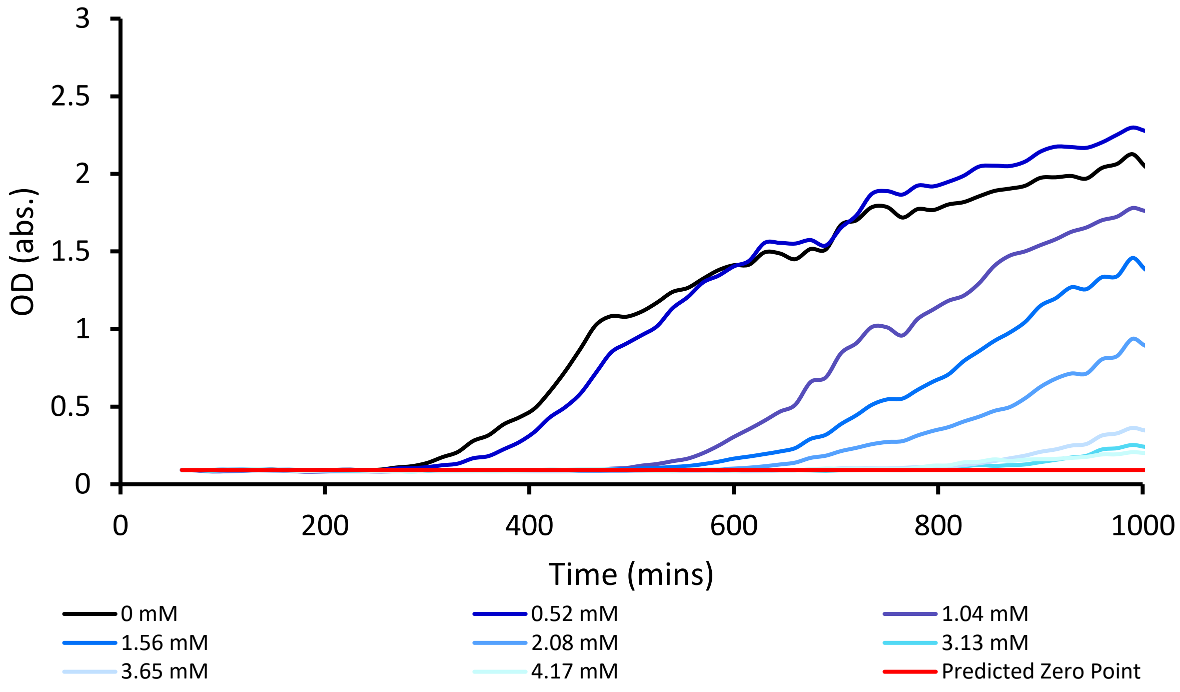

Figure S189 - MRSA growth curves created from an average of 6 optical density readings for each concentration in the presence of compound **23** at varying concentrations. Predicted zero point used in MIC_{50} calculation due to the solubility of compound.

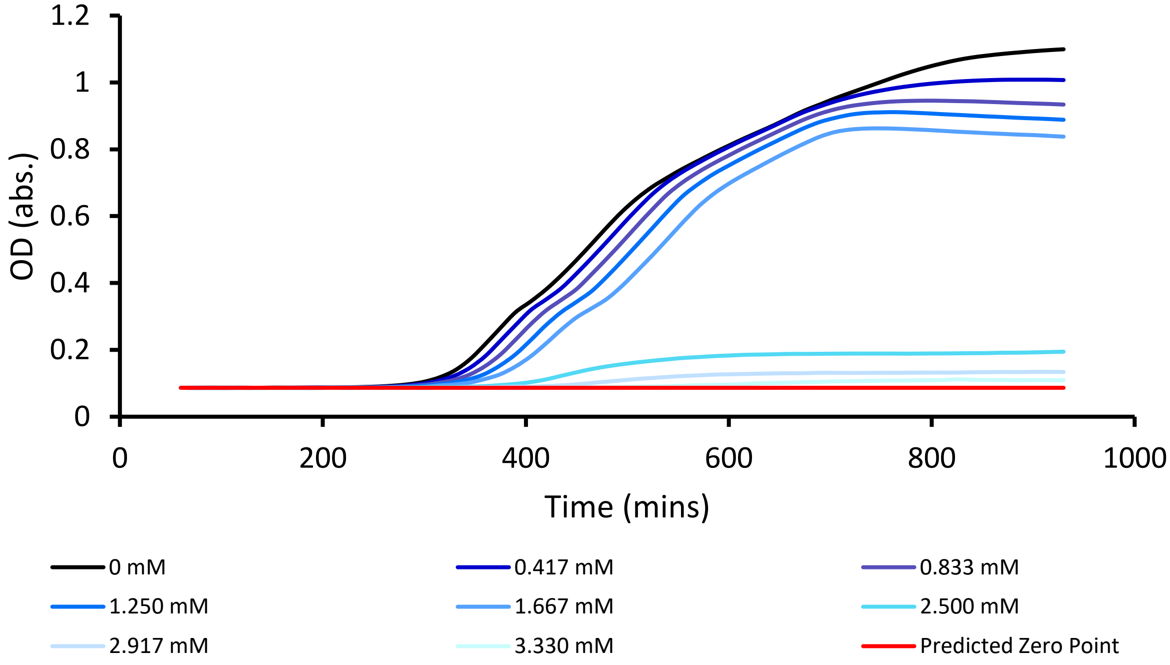

Figure S190 - MRSA growth curves created from an average of 6 optical density readings for each concentration in the presence of compound **24** at varying concentrations. Predicted zero point used in MIC_{50} calculation due to the solubility of compound.

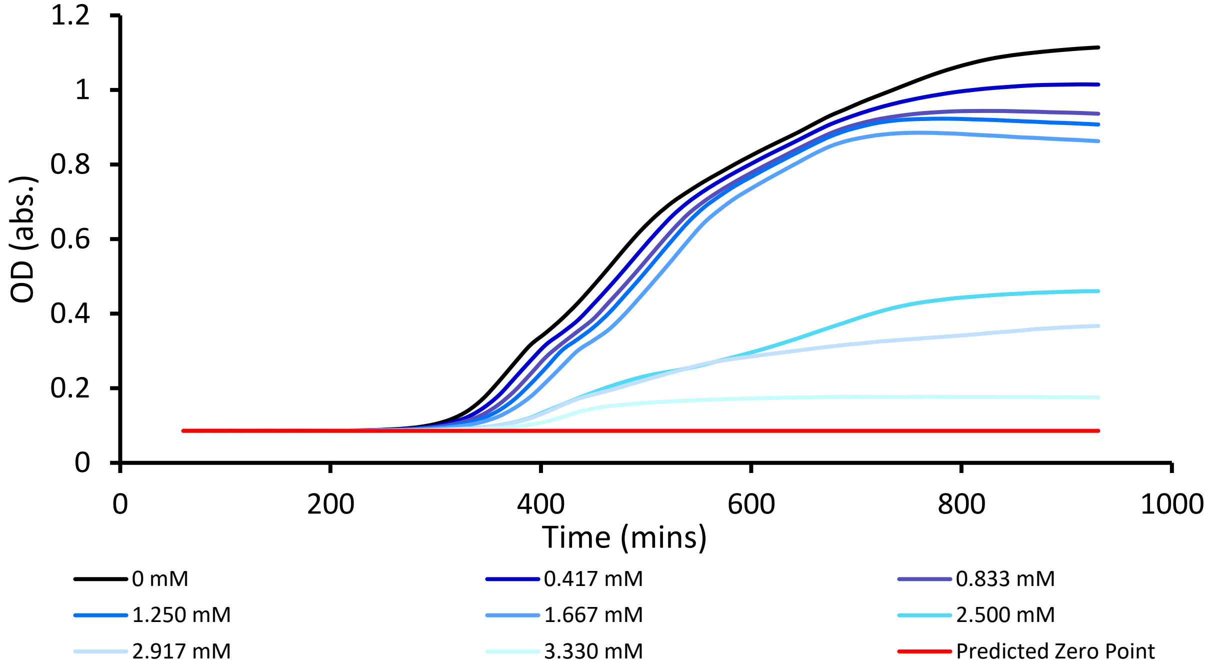

Figure S191 - MRSA growth curves created from an average of 6 optical density readings for each concentration in the presence of compound **25** at varying concentrations. Predicted zero point used in MIC_{50} calculation due to the solubility of compound.

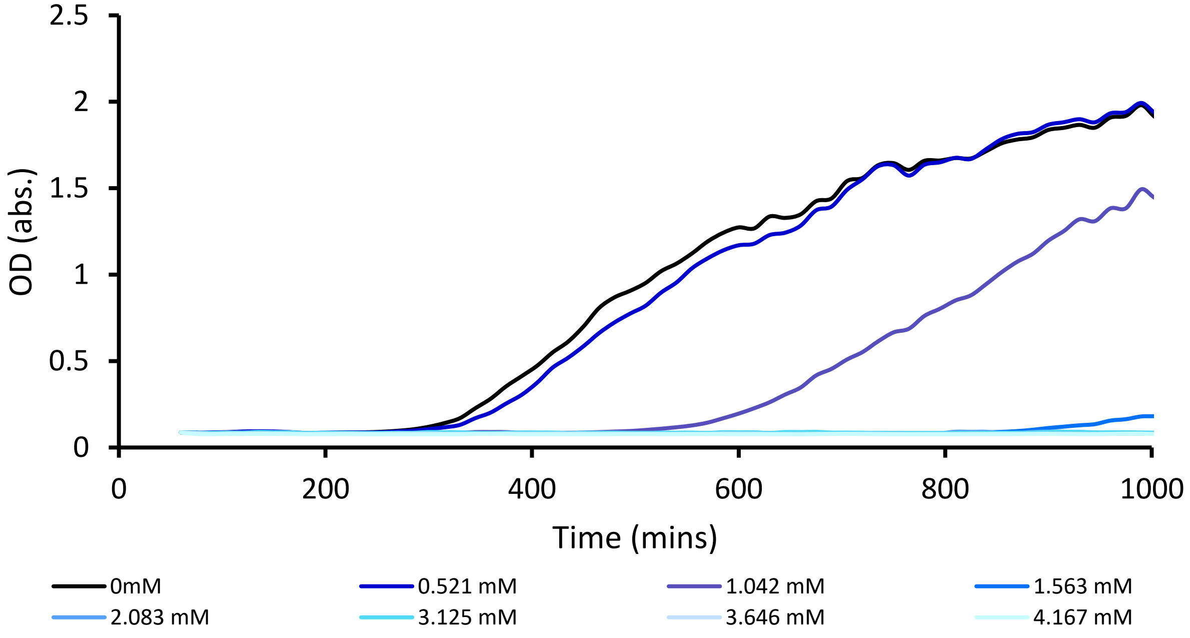

Figure S192 - MRSA growth curves created from an average of 6 optical density readings for each concentration in the presence of compound **27** at varying concentrations.

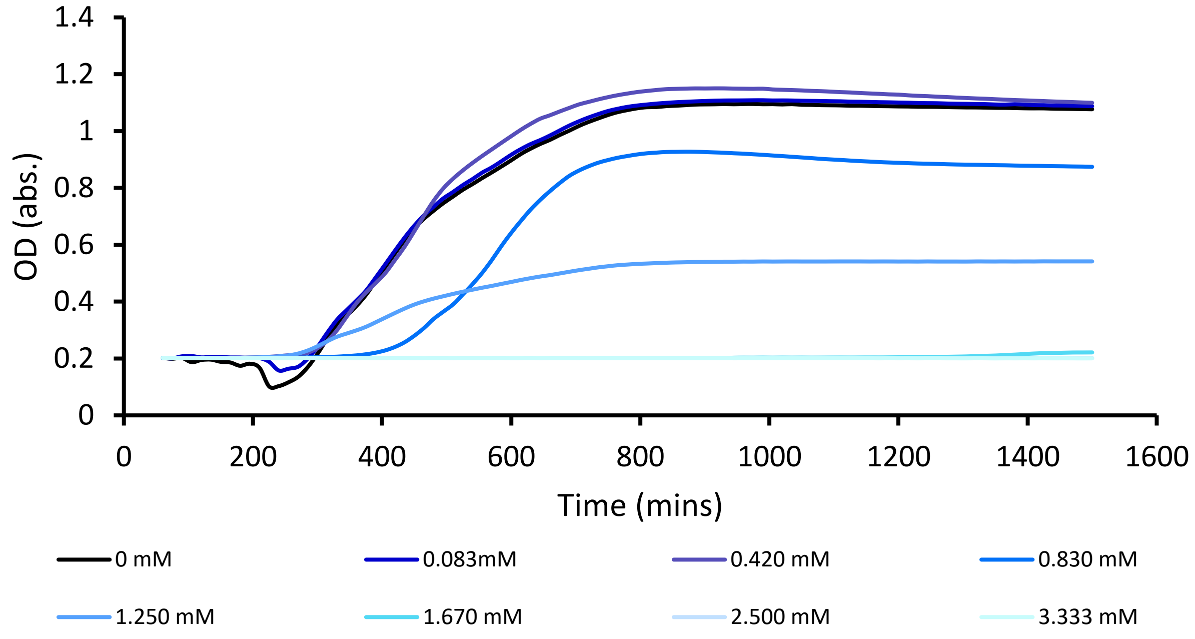

Figure S193 - MRSA growth curves created from an average of 6 optical density readings for each concentration in the presence of compound **30** at varying concentrations.

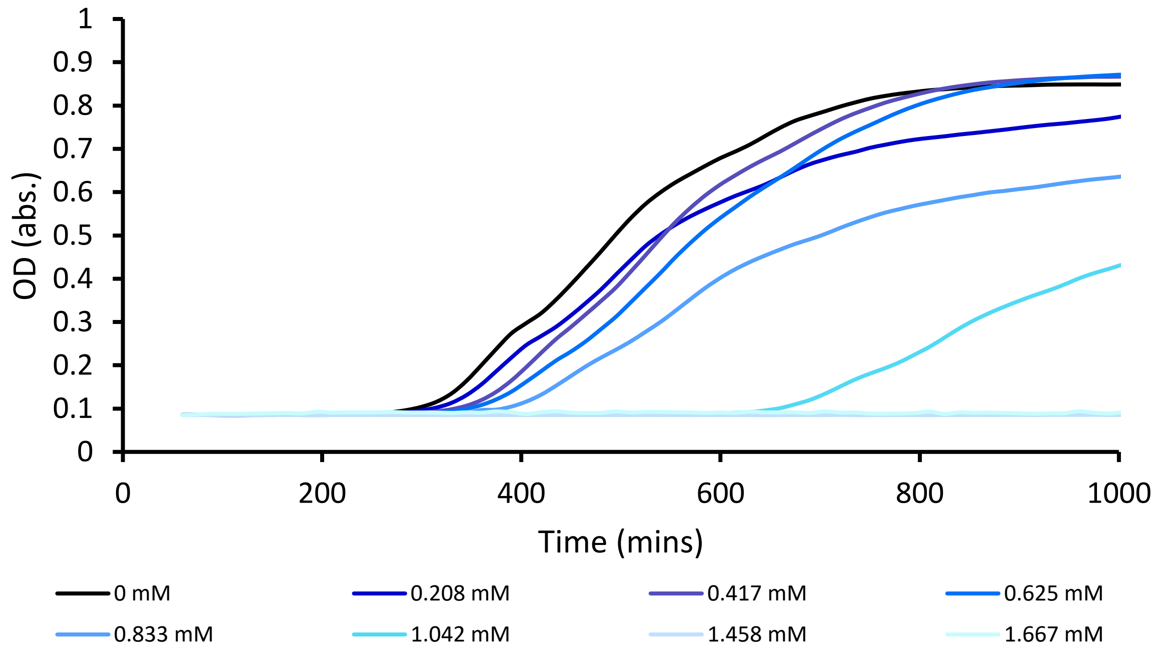

Figure S194 - MRSA growth curves created from an average of 6 optical density readings for each concentration in the presence of compound **31** at varying concentrations. Due to solubility of compound, these concentrations may not be accurate, an MIC_{50} was not calculated.

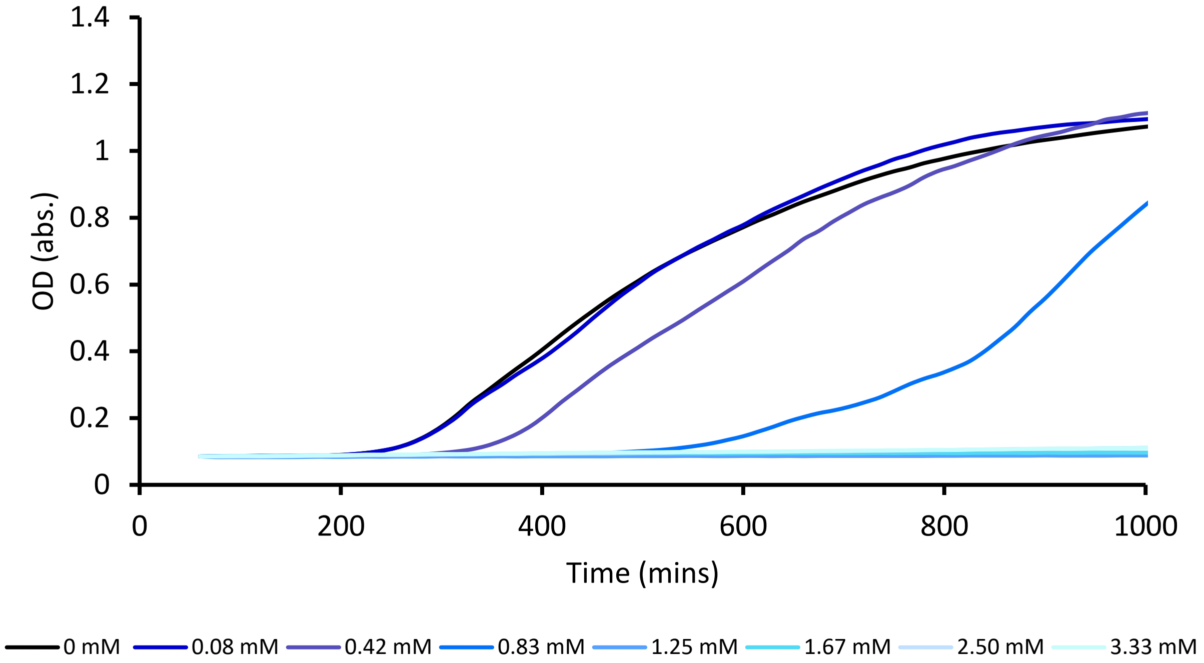

Figure S195 - MRSA growth curves created from an average of 6 optical density readings for each concentration in the presence of compound **32** at varying concentrations.

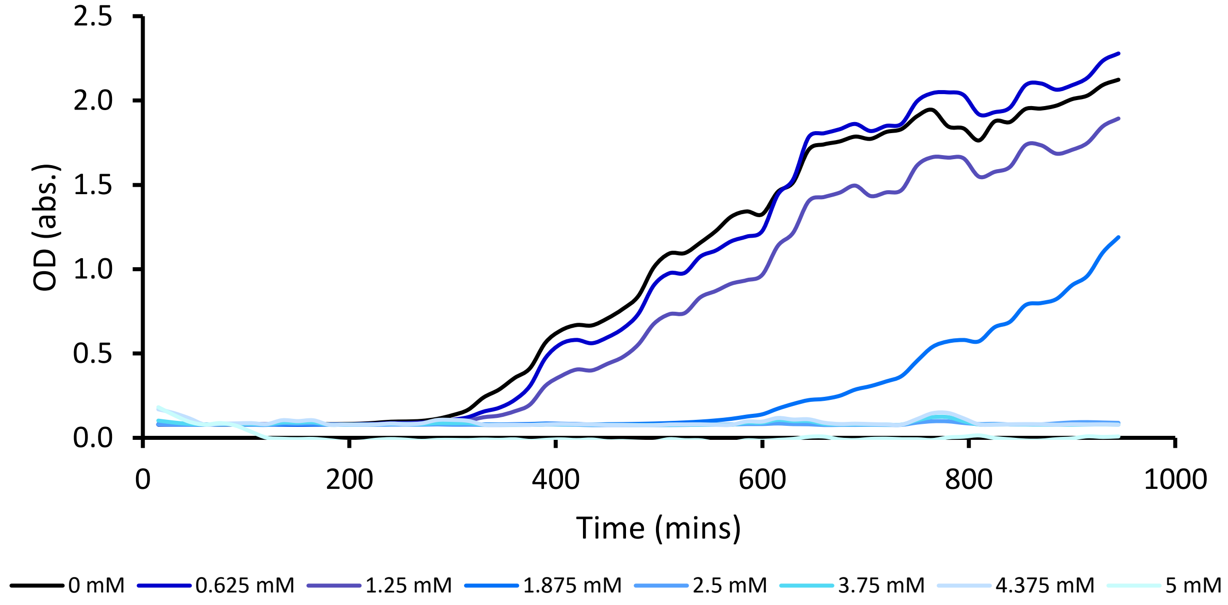

Figure S196 - MRSA growth curves created from an average of 6 optical density readings for each concentration in the presence of compound **33** at varying concentrations.

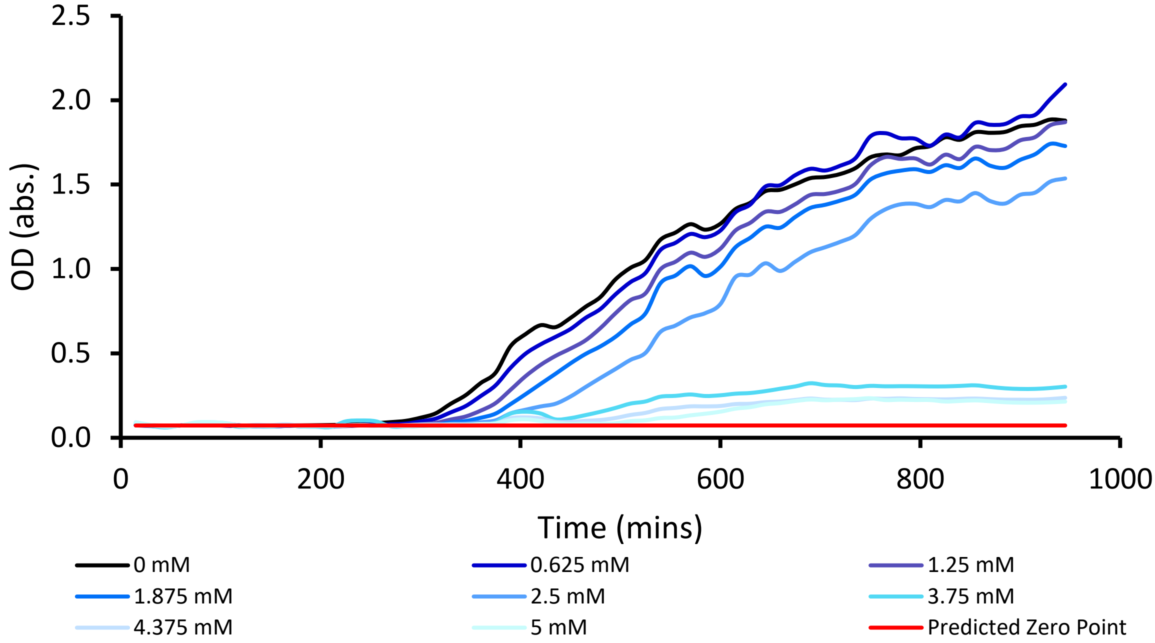

Figure S197 - MRSA growth curves created from an average of 6 optical density readings for each concentration in the presence of compound **34** at varying concentrations. Predicted zero point used in MIC_{50} calculation due to the solubility of compound.

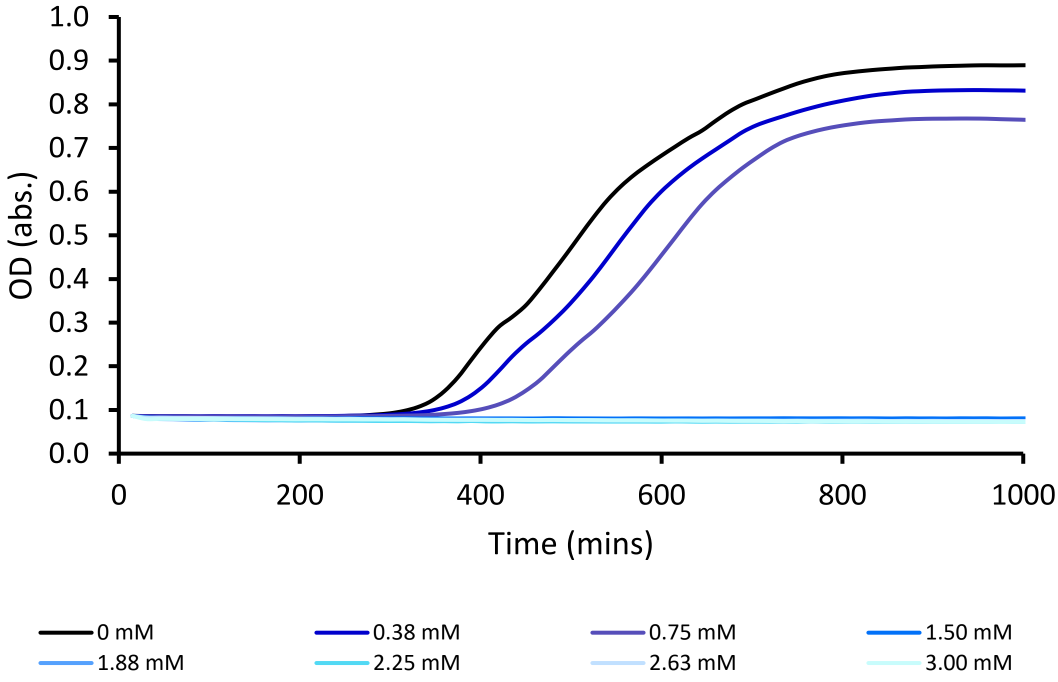

Figure S198 - MRSA growth curves created from an average of 6 optical density readings for each concentration in the presence of compound **38** at varying concentrations.

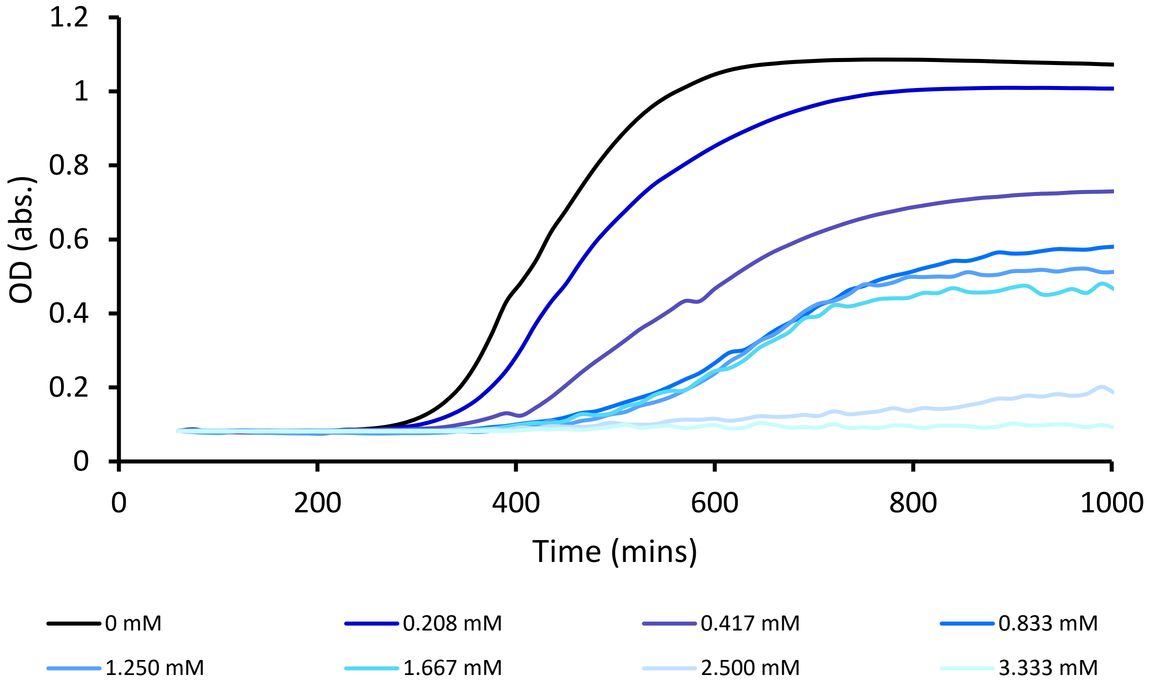

Figure S199 - MRSA growth curves created from an average of 6 optical density readings for each concentration in the presence of compound **39** at varying concentrations.

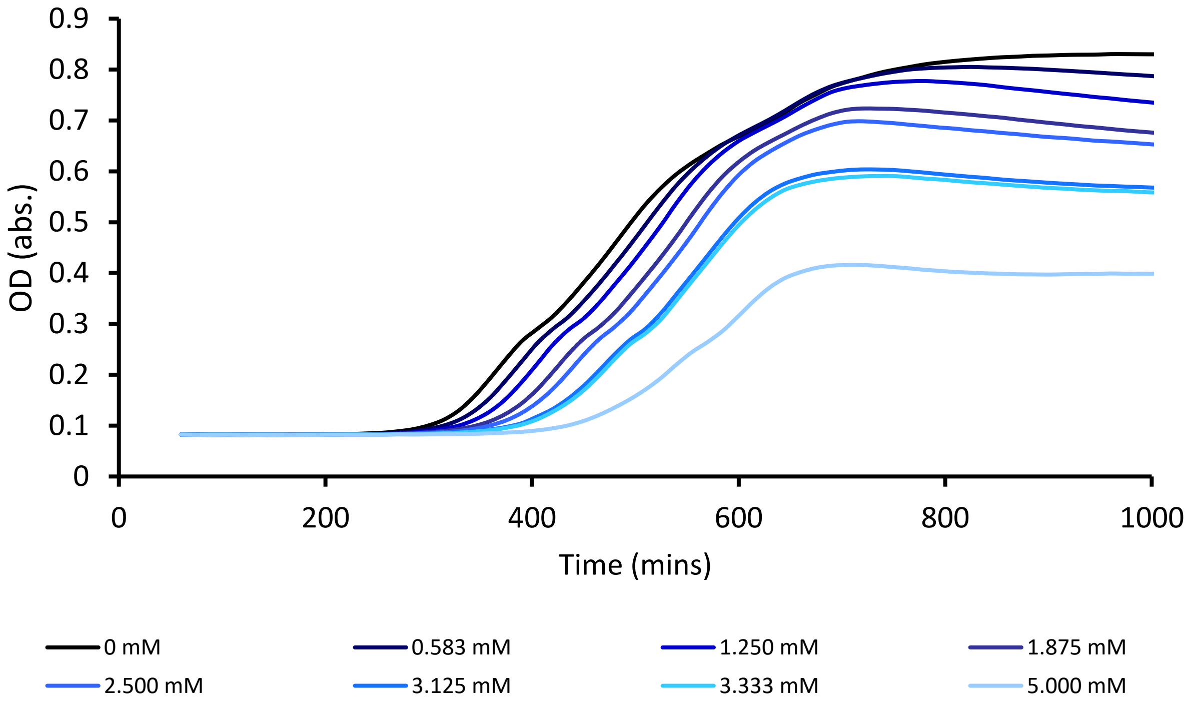

Figure S200 - MRSA growth curves created from an average of 6 optical density readings for each concentration in the presence of compound **40** at varying concentrations. Due to low compound solubility in 5 % EtOH, a MIC_{50} was not possible.

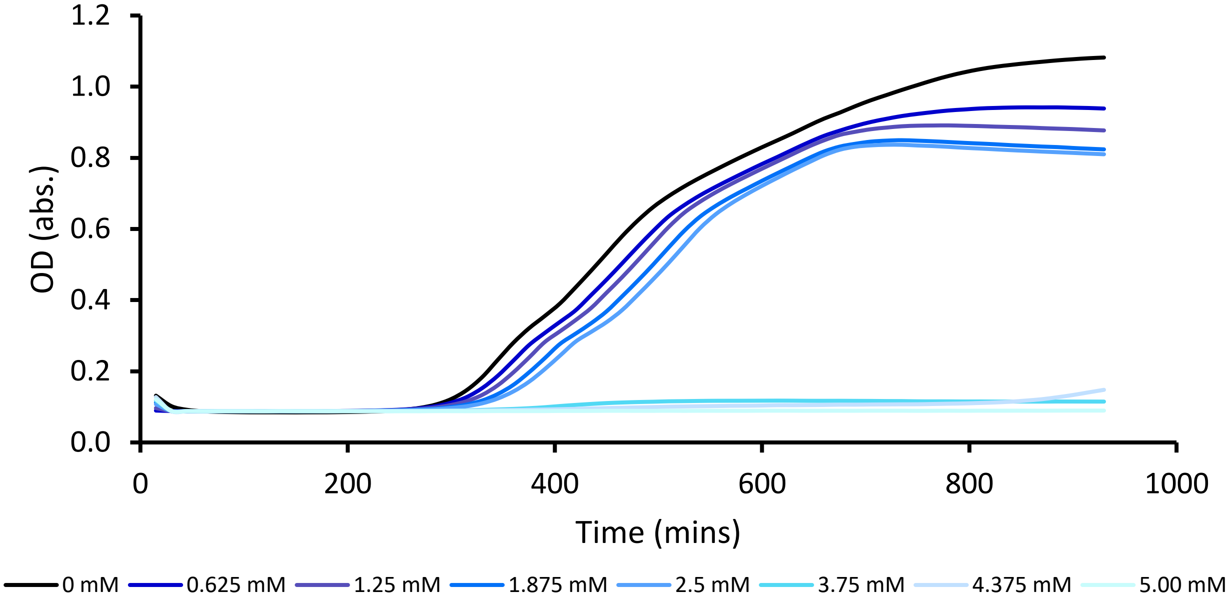

Figure S201 - MRSA growth curves created from an average of 6 optical density readings for each concentration in the presence of compound **41** at varying concentrations.

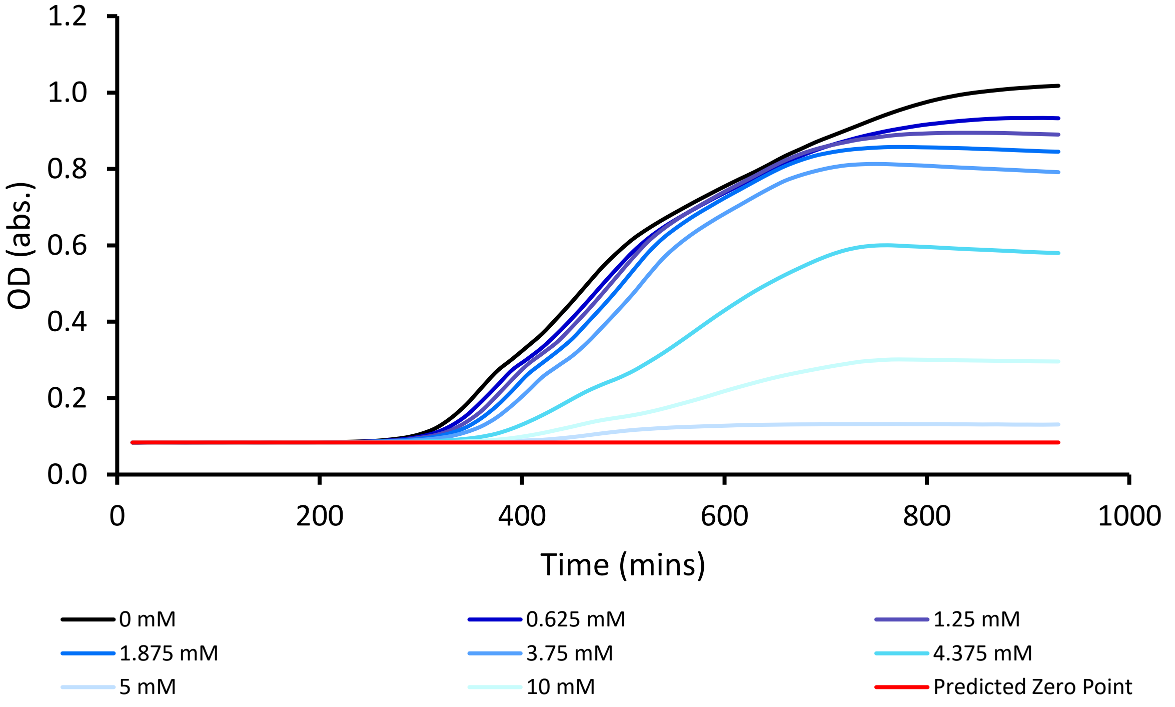

Figure S202 - MRSA growth curves created from an average of 6 optical density readings for each concentration in the presence of compound **42** at varying concentrations. Predicted zero point used in MIC_{50} calculation due to the solubility of compound.

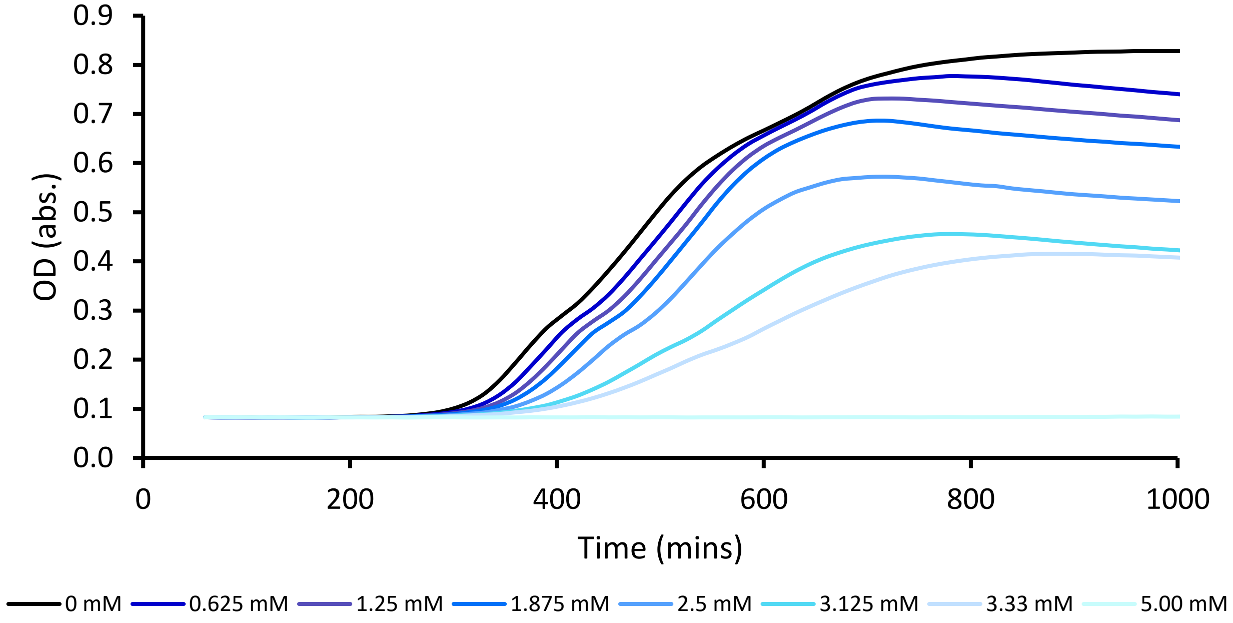

Figure S203 - MRSA growth curves created from an average of 6 optical density readings for each concentration in the presence of compound **43** at varying concentrations.

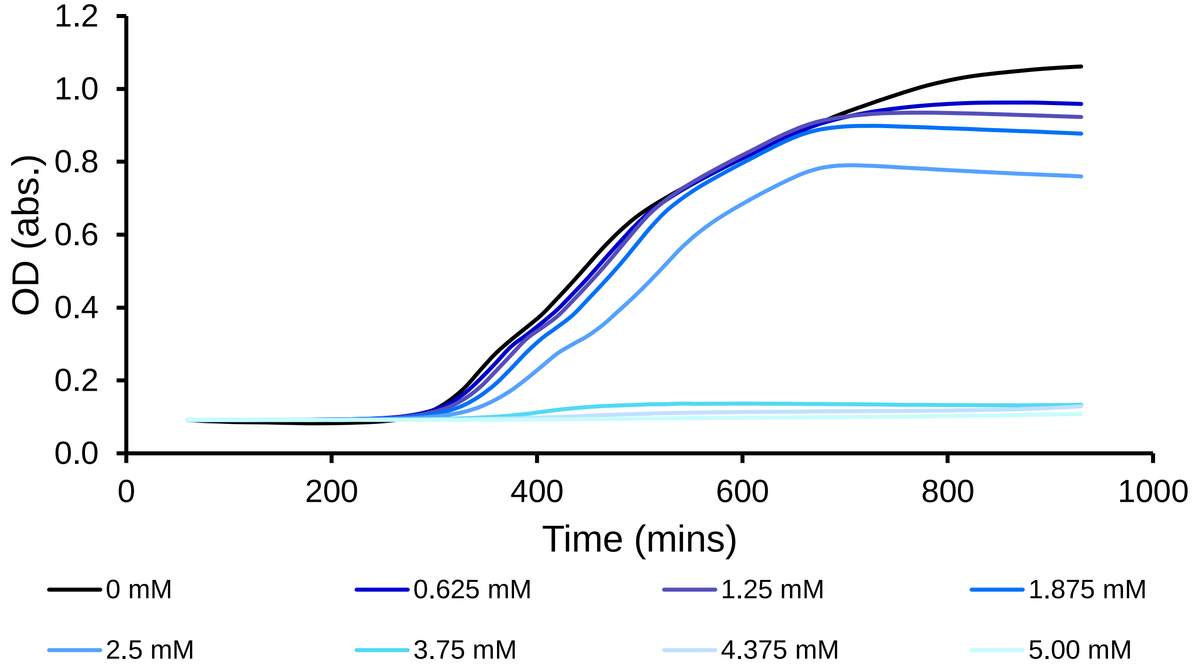

Figure S204 - MRSA growth curves created from an average of 6 optical density readings for each concentration in the presence of compound **44** at varying concentrations.

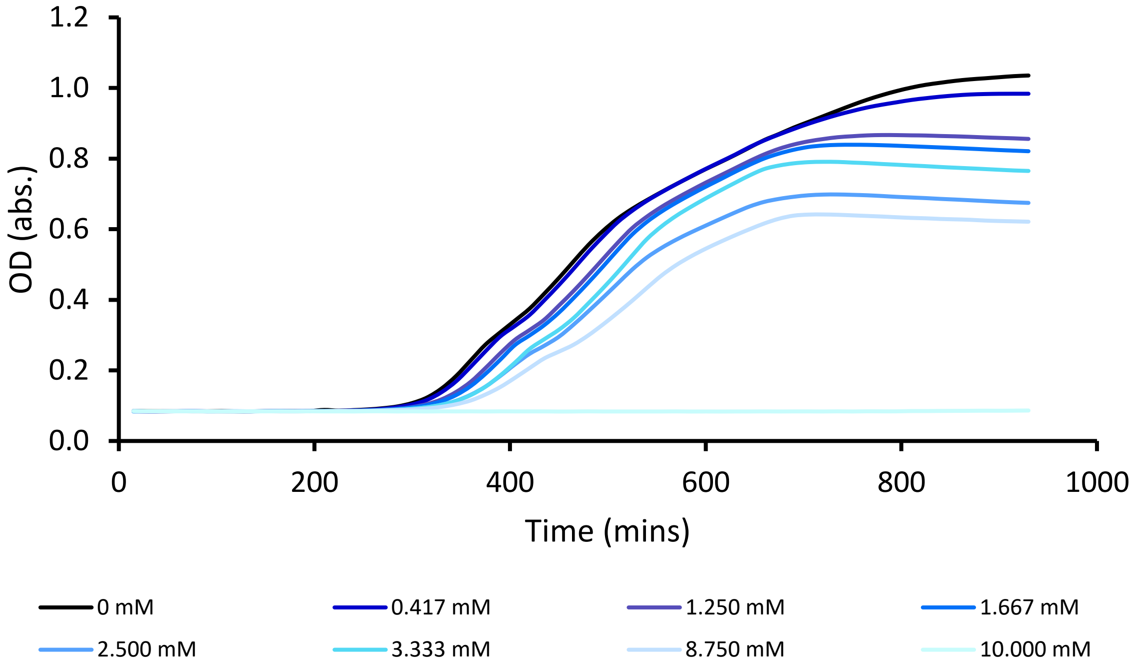

Figure S205 - MRSA growth curves created from an average of 6 optical density readings for each concentration in the presence of compound **45** at varying concentrations.

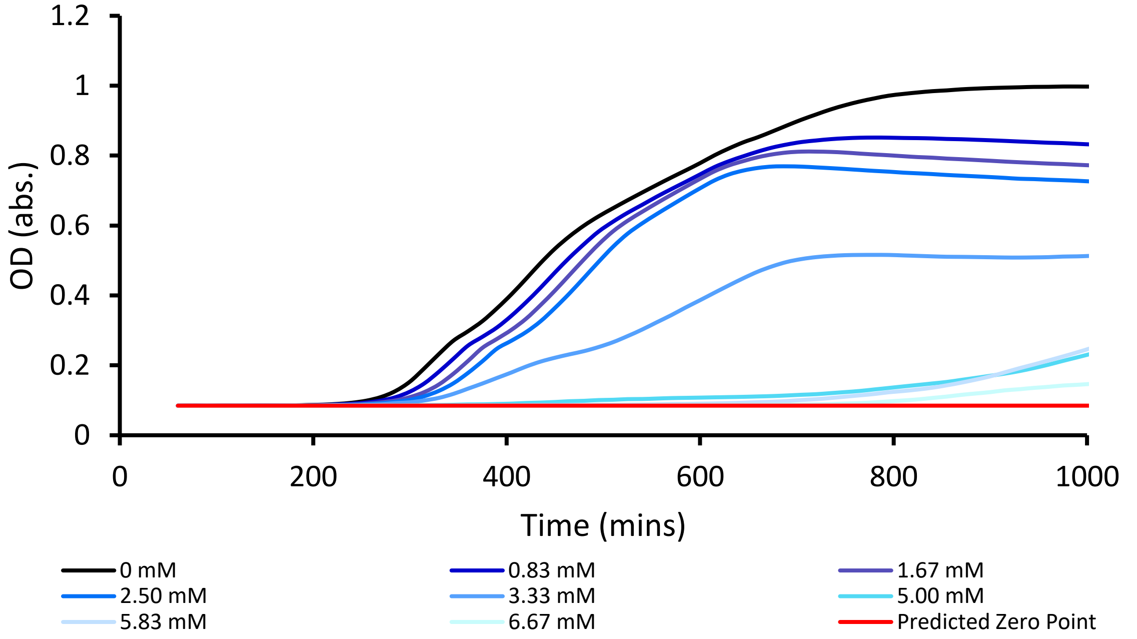

Figure S206 - MRSA growth curves created from an average of 6 optical density readings for each concentration in the presence of compound **46** at varying concentrations. Predicted zero point used in MIC_{50} calculation due to the solubility of compound.

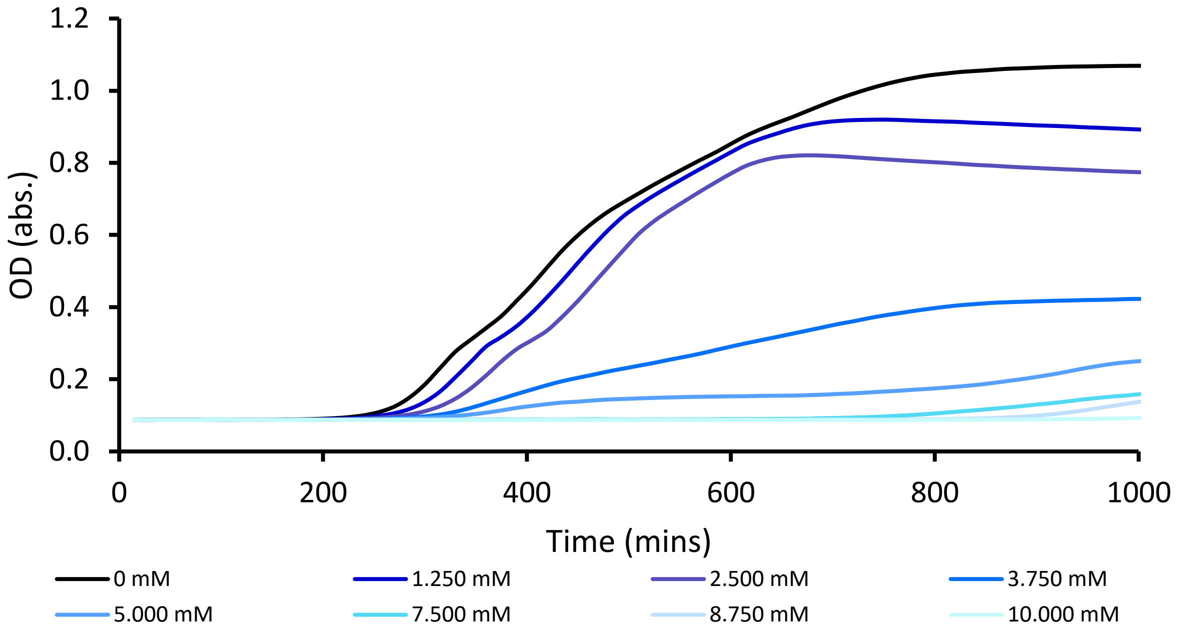

Figure S207 - MRSA growth curves created from an average of 6 optical density readings for each concentration in the presence of compound **50** at varying concentrations.

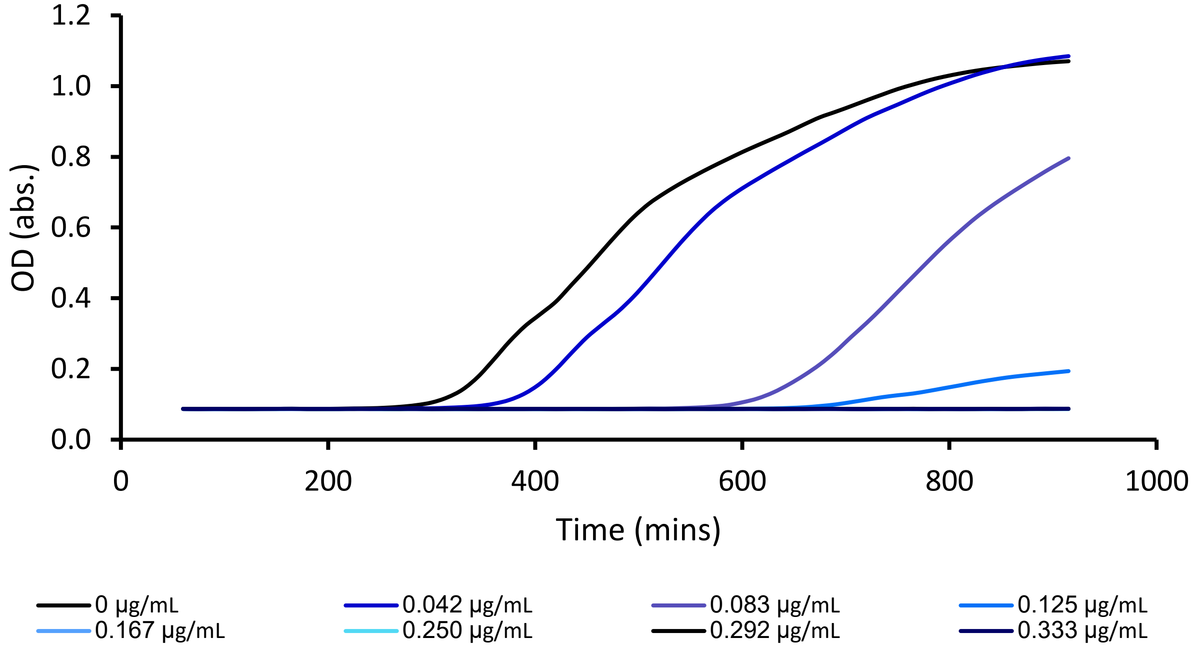

Figure S208 - MRSA growth curves created from an average of 6 optical density readings for each concentration in the presence of **ampicillin** at varying concentrations.

Escherichia coli (*E. coli*)

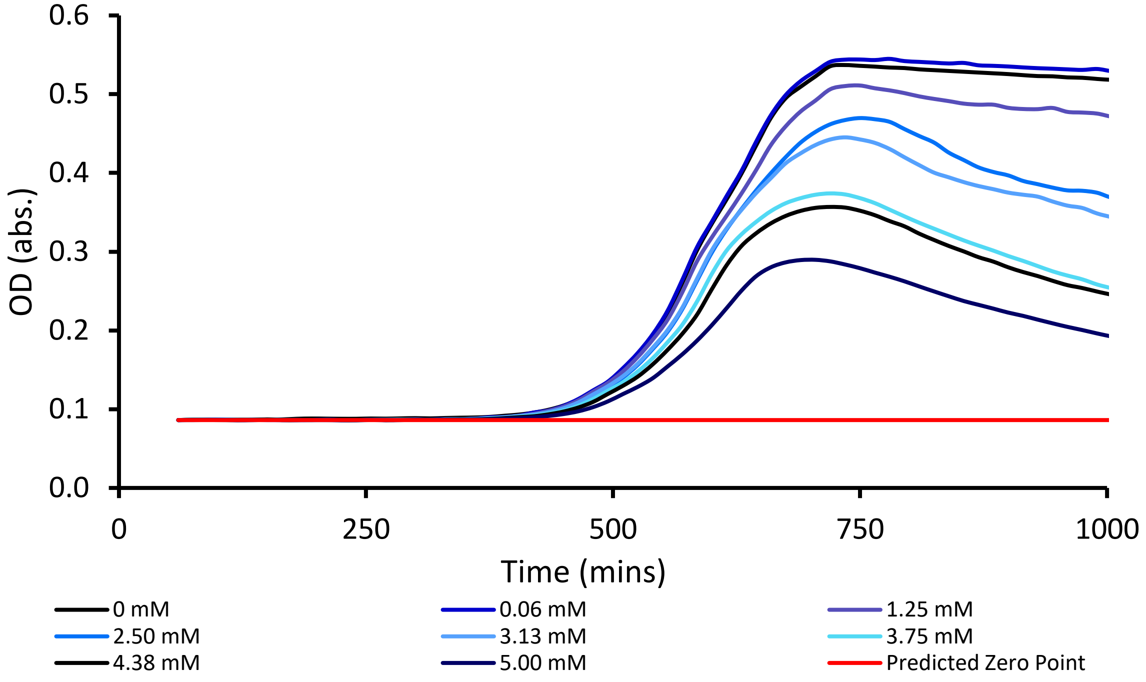

Figure S209 – *E. coli* DH10B growth curves created from an average of 6 optical density readings for each concentration in the presence of compound **1** at varying concentrations. MIC_{50} was calculated using predicted endpoint due to solubility.

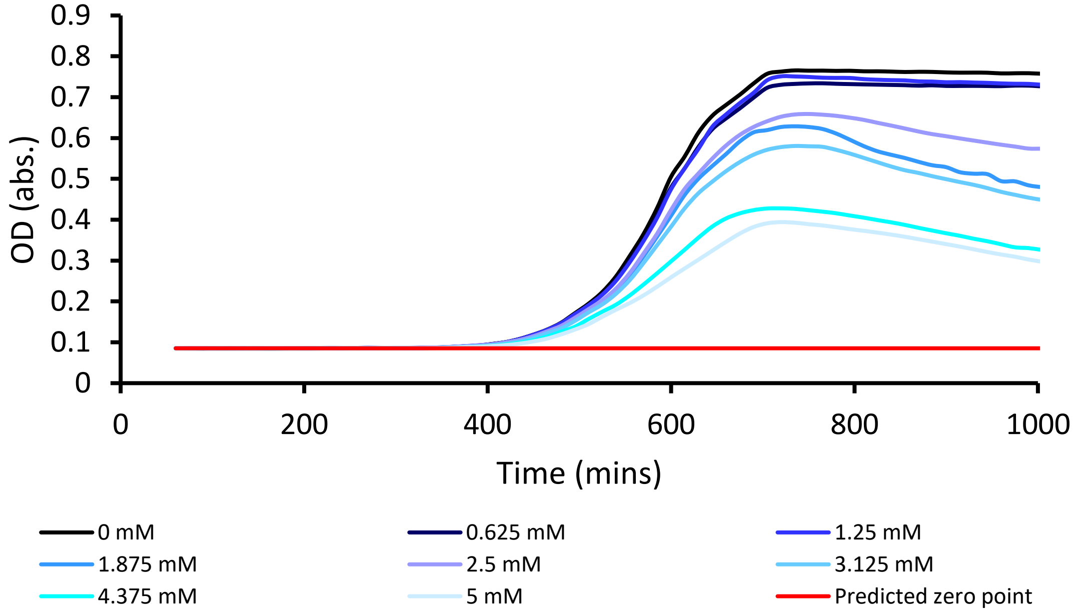

Figure S210 - *E. coli* DH10B growth curves created from an average of 6 optical density readings for each concentration in the presence of compound **2** at varying concentrations. MIC_{50} was calculated using predicted endpoint due to solubility.

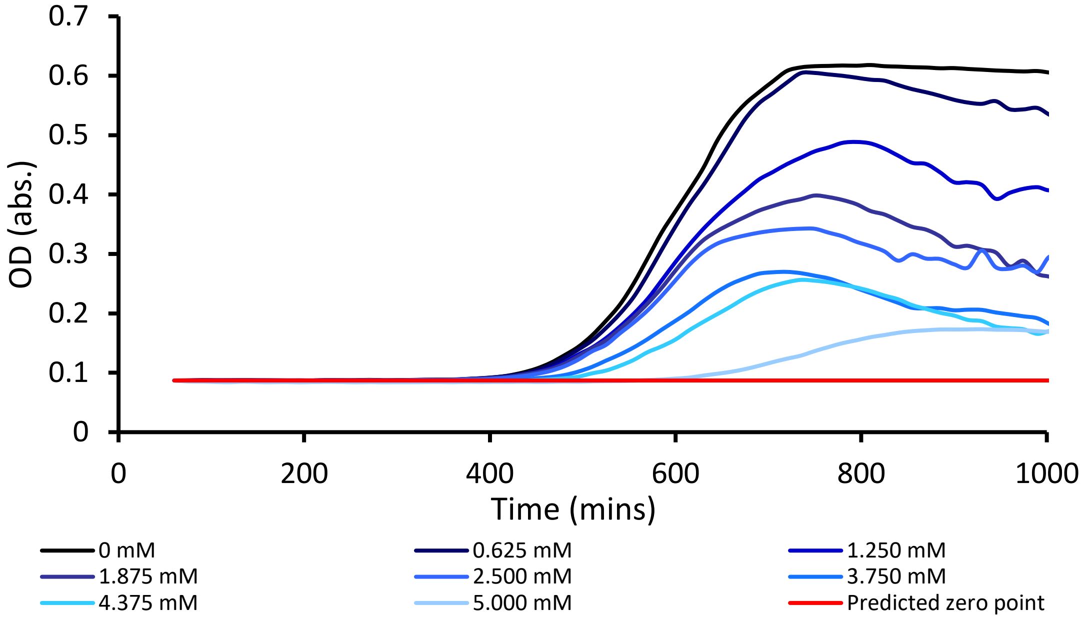

Figure S211 - *E. coli* DH10B growth curves created from an average of 6 optical density readings for each concentration in the presence of compound **3** at varying concentrations. MIC_{50} was calculated using predicted endpoint due to solubility.

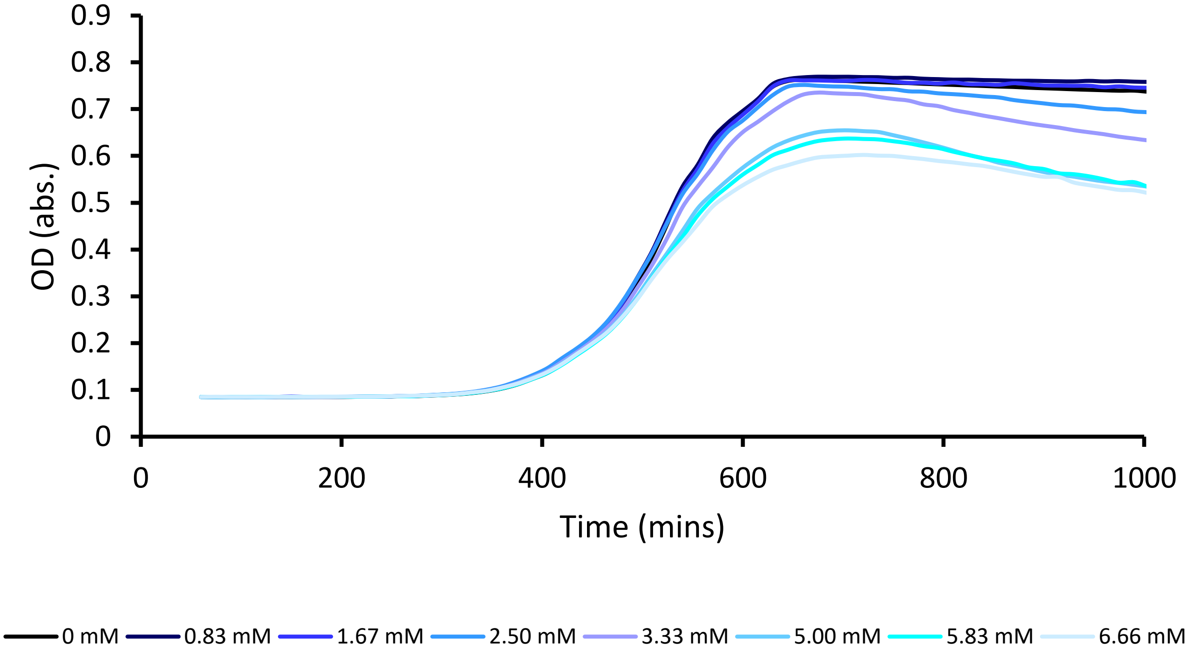

Figure **S**212 - *E. coli* DH10B growth curves created from an average of 6 optical density readings for each concentration in the presence of compound **4** at varying concentrations. Unable to calculate MIC_{50} due to solubility.

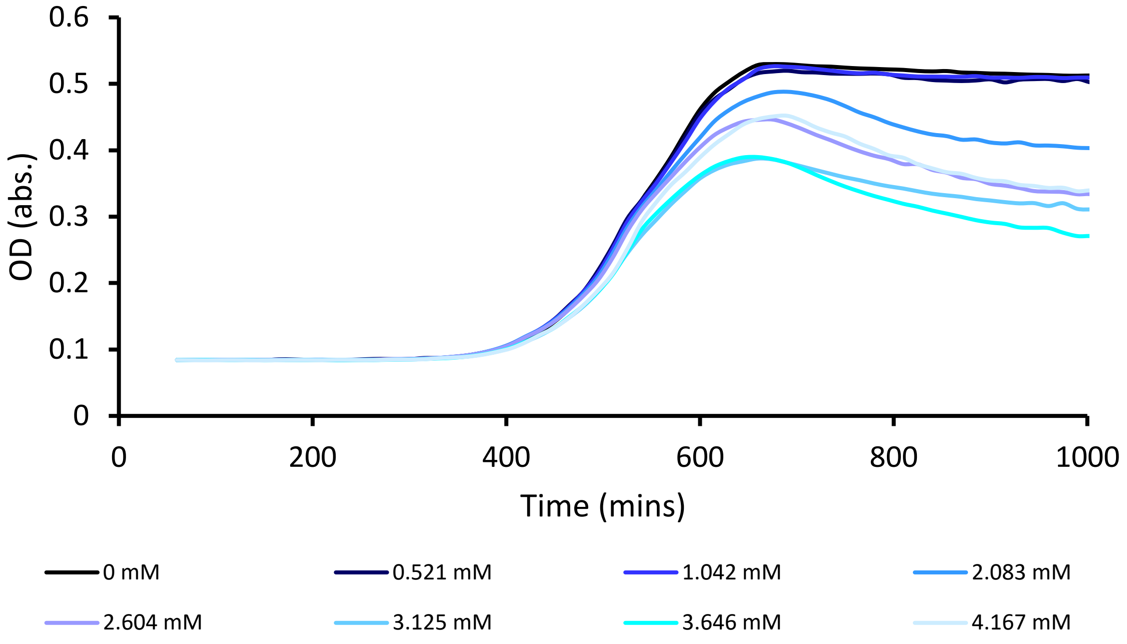

Figure S213 - *E. coli* DH10B growth curves created from an average of 6 optical density readings for each concentration in the presence of compound **5** at varying concentrations. Unable to calculate MIC_{50} due to solubility.

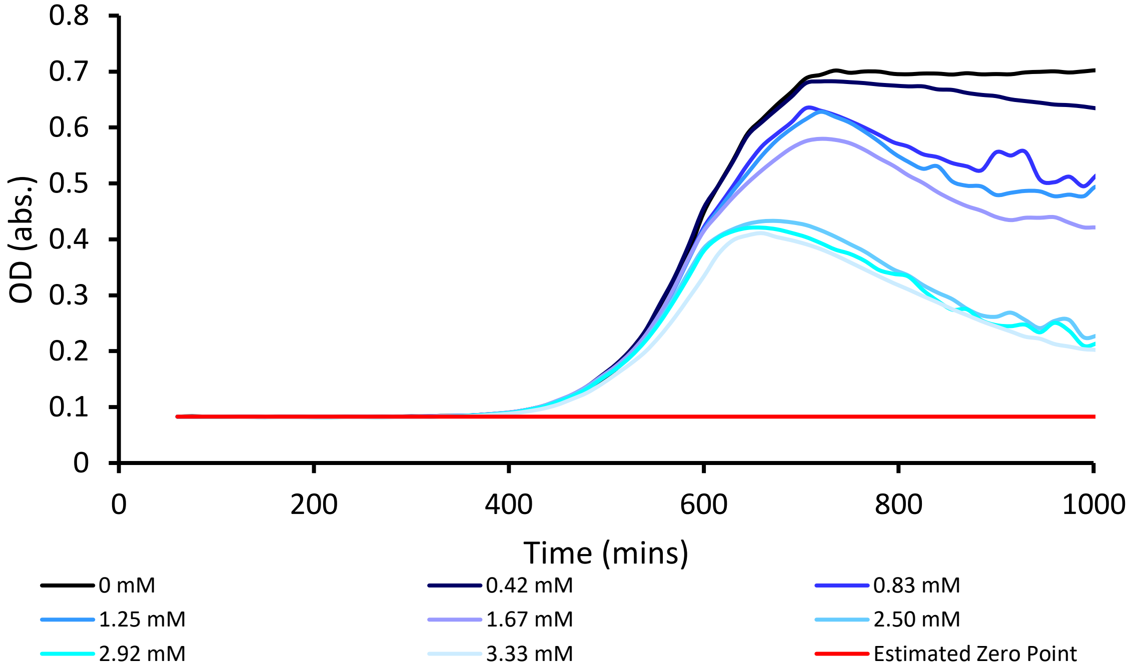

Figure S214 - *E. coli* DH10B growth curves created from an average of 6 optical density readings for each concentration in the presence of compound **6** at varying concentrations. MIC_{50} was calculated using predicted endpoint due to solubility.

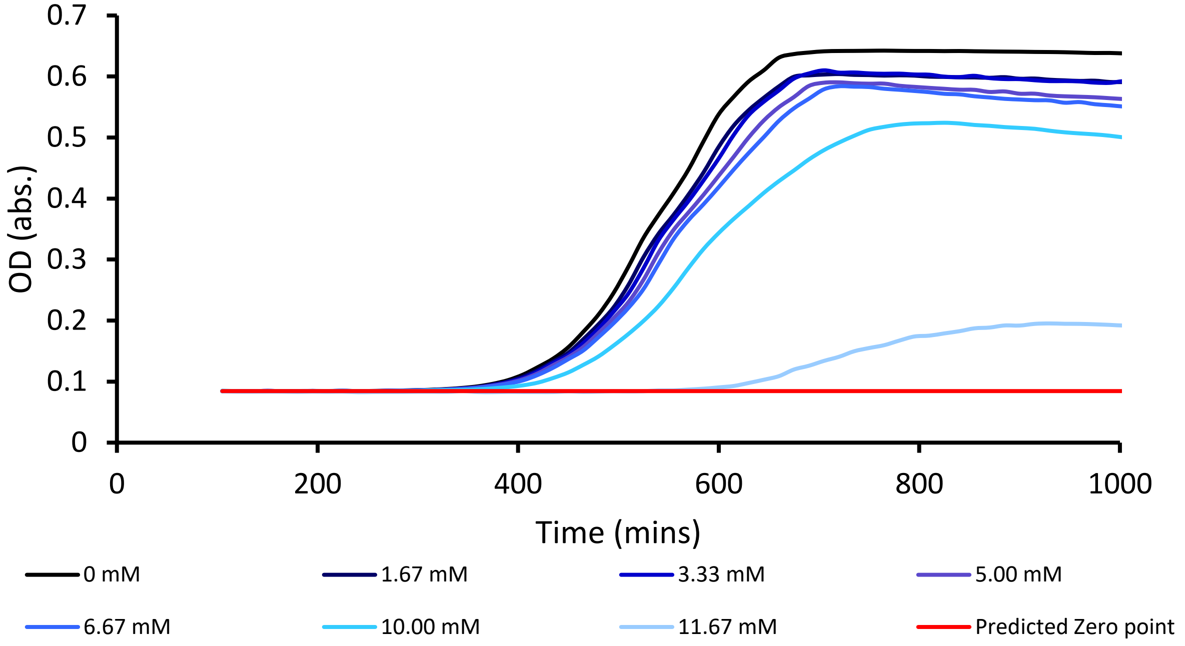

Figure S215 - *E. coli* DH10B growth curves created from an average of 6 optical density readings for each concentration in the presence of compound **9** at varying concentrations. MIC_{50} was calculated using predicted endpoint due to solubility.

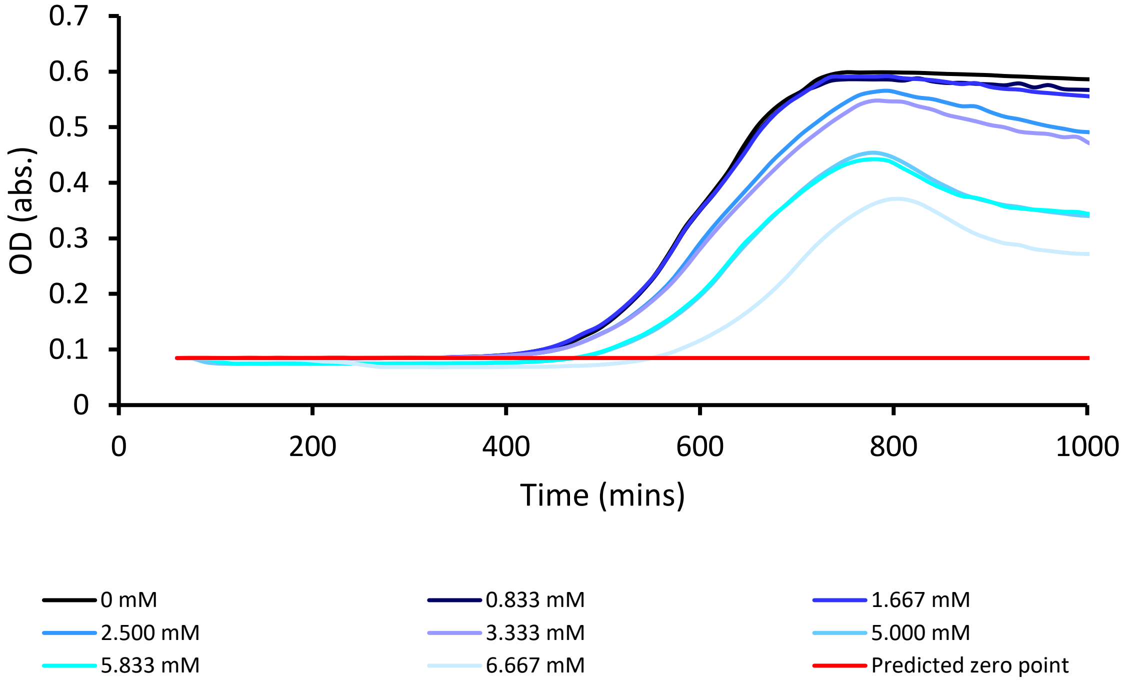

Figure S216 - *E. coli* DH10B growth curves created from an average of 6 optical density readings for each concentration in the presence of compound **11** at varying concentrations. MIC_{50} was calculated using predicted endpoint due to solubility.

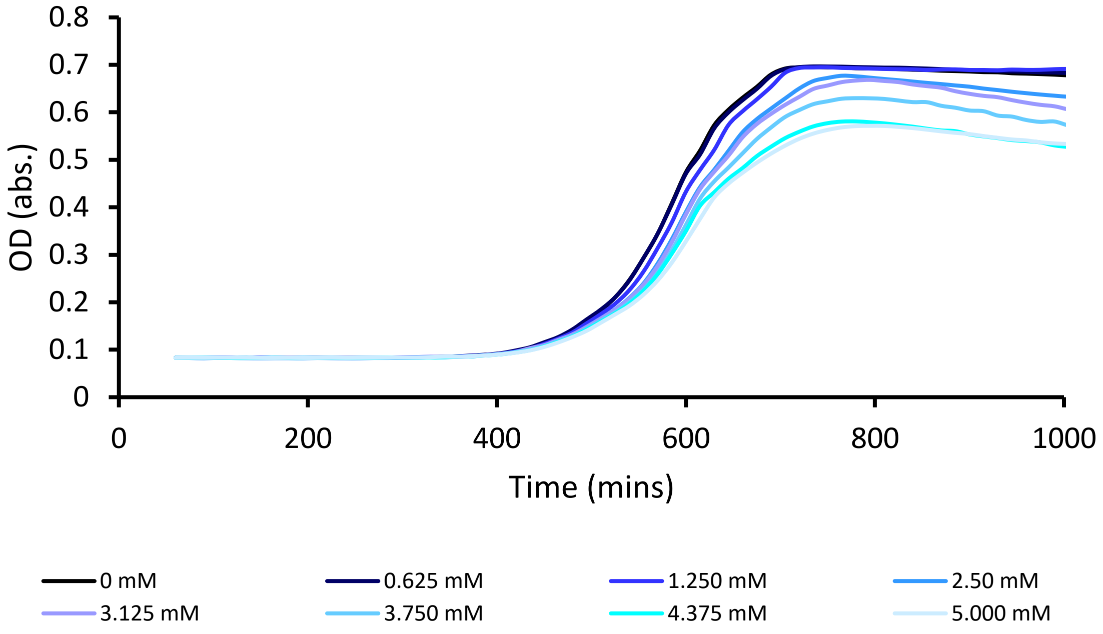

Figure S217 - *E. coli* DH10B growth curves created from an average of 6 optical density readings for each concentration in the presence of compound **21** at varying concentrations. Unable to calculate MIC_{50} due to solubility.

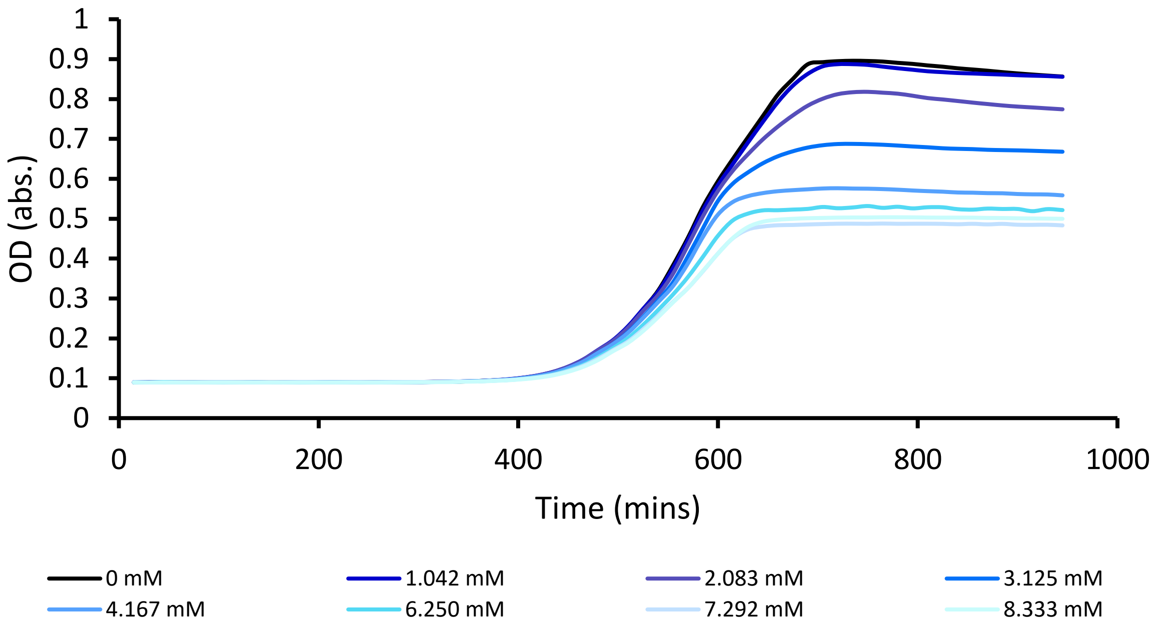

Figure S218 - *E. coli* DH10B growth curves created from an average of 6 optical density readings for each concentration in the presence of compound **22** at varying concentrations. Unable to calculate MIC_{50} due to solubility.

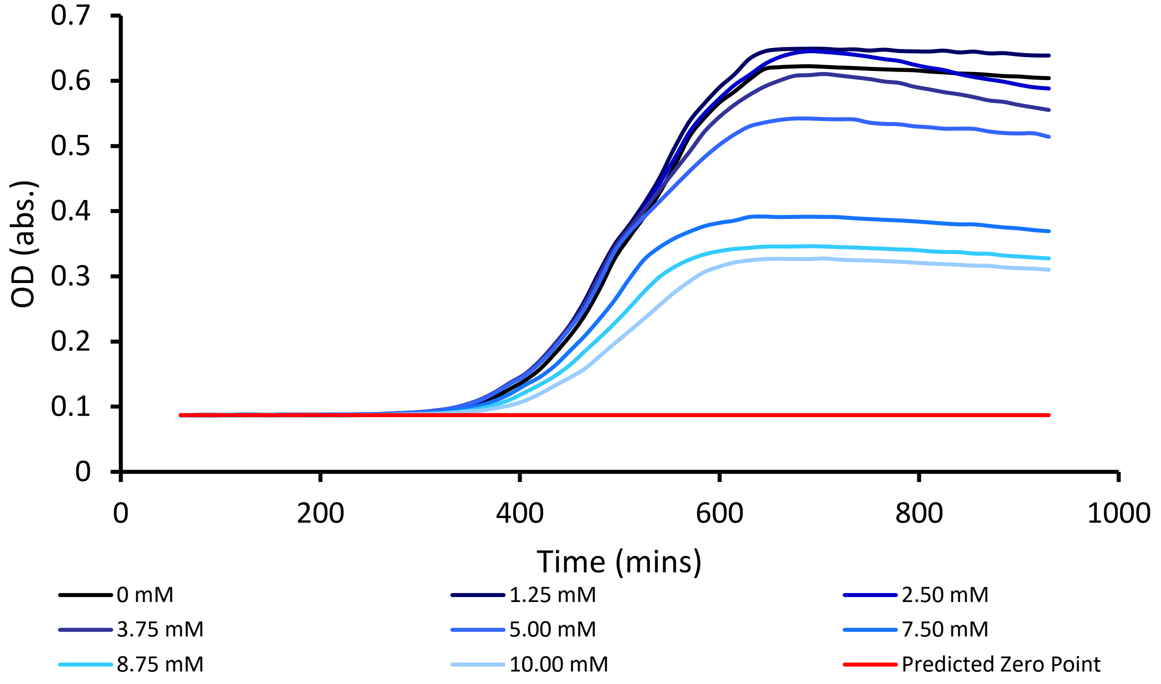

Figure S219 - *E. coli* DH10B growth curves created from an average of 6 optical density readings for each concentration in the presence of compound **24** at varying concentrations. Unable to calculate MIC_{50} due to solubility.

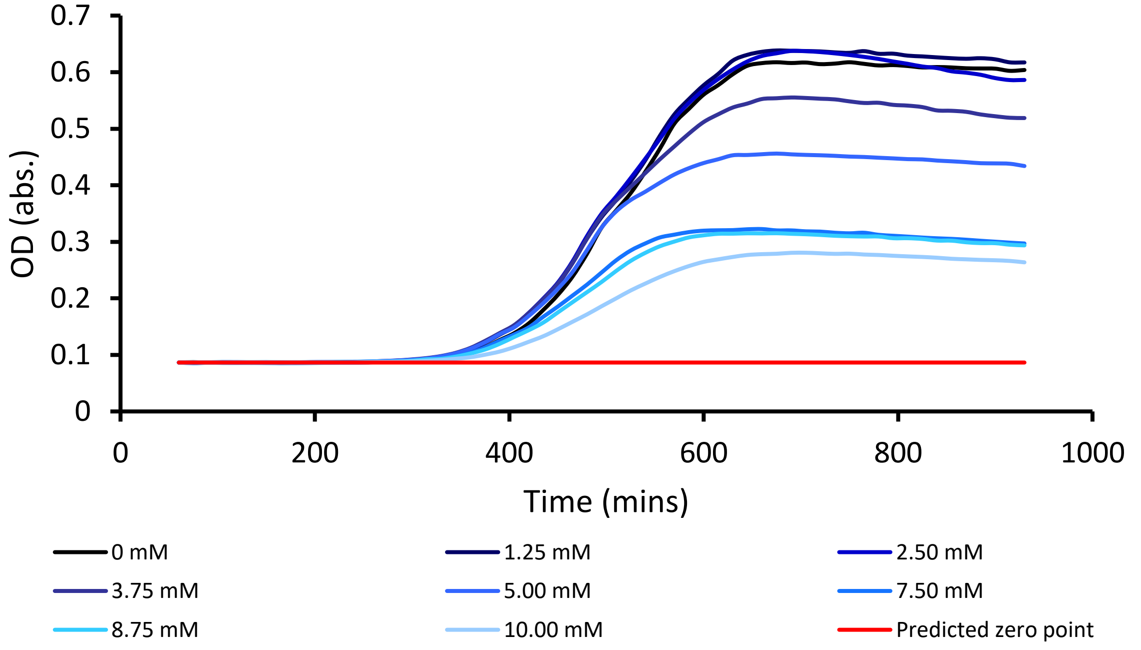

Figure S220 - *E. coli* DH10B growth curves created from an average of 6 optical density readings for each concentration in the presence of compound **25** at varying concentrations. MIC_{50} was calculated using predicted endpoint due to solubility.

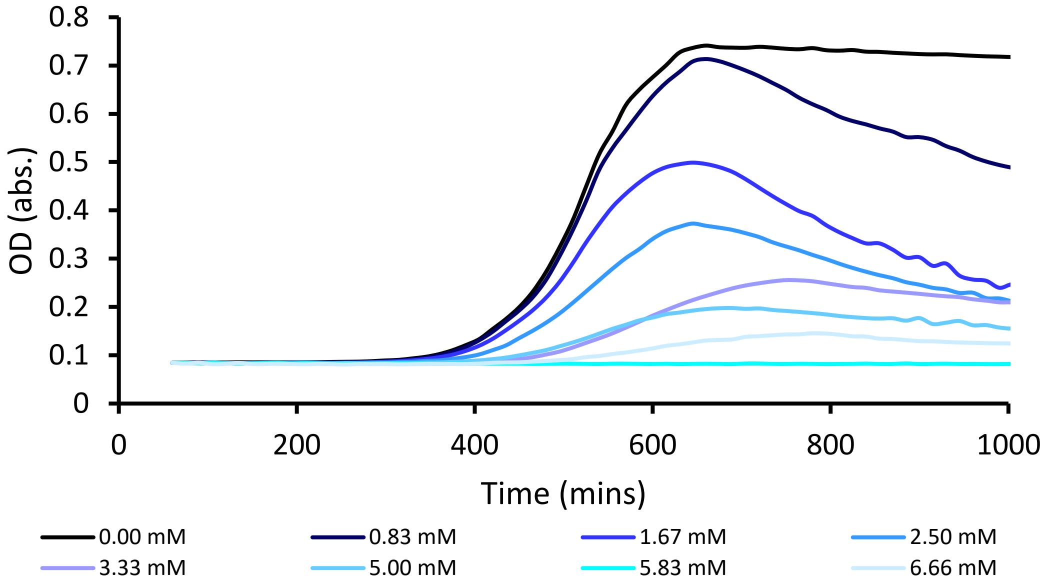

Figure S221 - *E. coli* DH10B growth curves created from an average of 6 optical density readings for each concentration in the presence of compound **30** at varying concentrations.

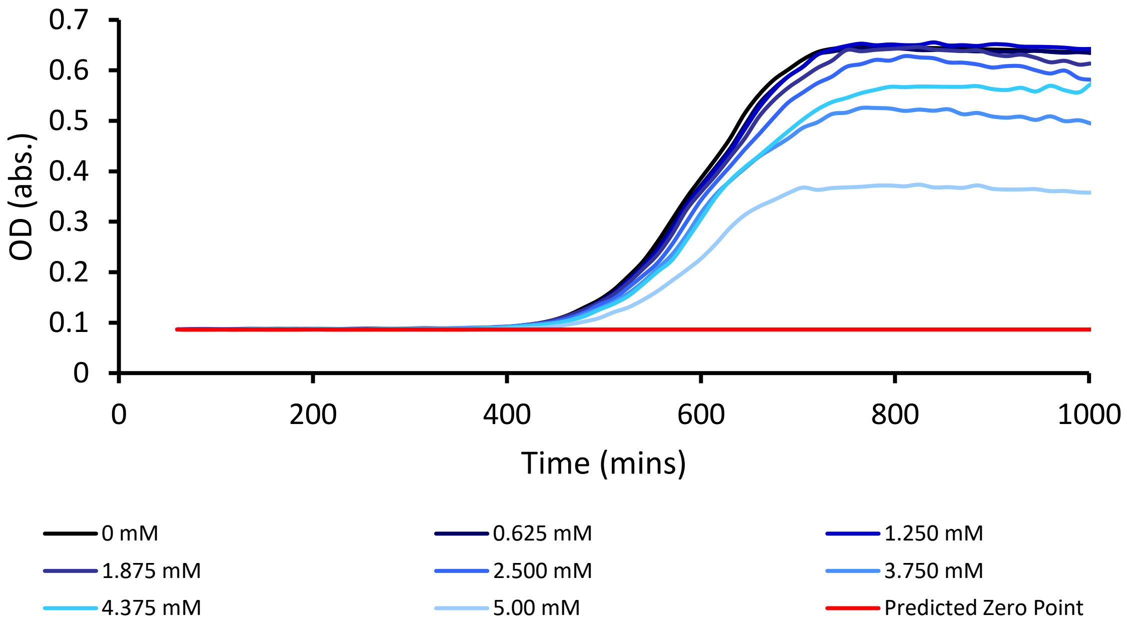

Figure S222 - *E. coli* DH10B growth curves created from an average of 6 optical density readings for each concentration in the presence of compound **34** at varying concentrations. MIC_{50} was calculated using predicted endpoint due to solubility.

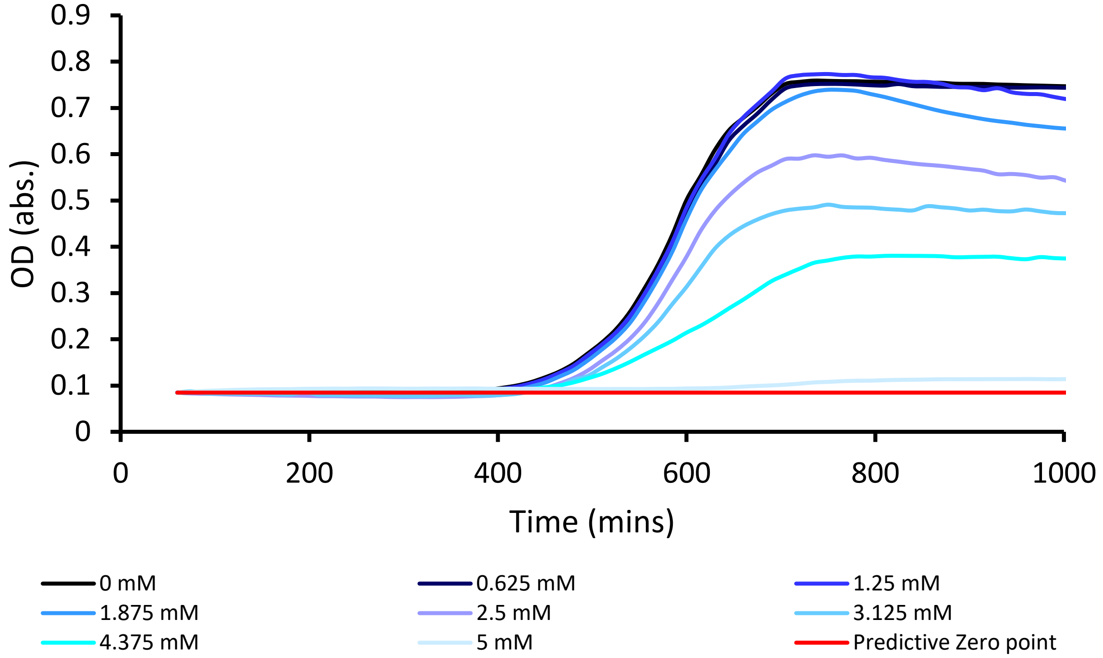

Figure S223 - *E. coli* DH10B growth curves created from an average of 6 optical density readings for each concentration in the presence of compound **38** at varying concentrations. MIC_{50} was calculated using predicted endpoint due to solubility.

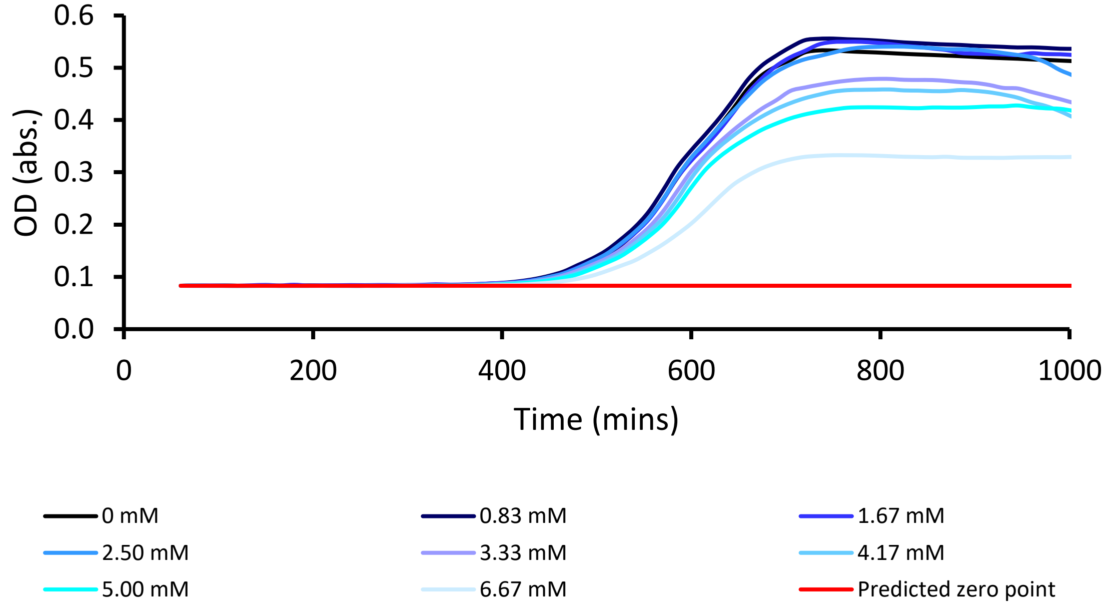

Figure S224 - *E. coli* DH10B growth curves created from an average of 6 optical density readings for each concentration in the presence of compound **39** at varying concentrations. MIC_{50} was calculated using predicted endpoint due to solubility.

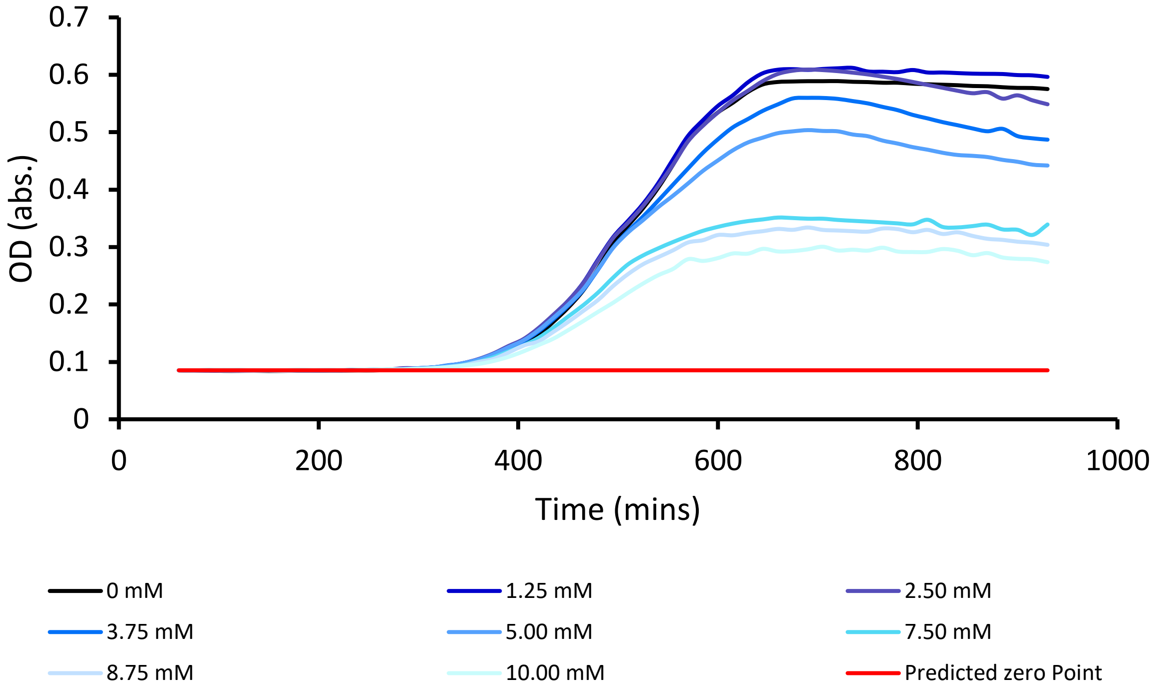

Figure S225 - *E. coli* DH10B growth curves created from an average of 6 optical density readings for each concentration in the presence of compound **41** at varying concentrations. MIC_{50} was calculated using predicted endpoint due to solubility.

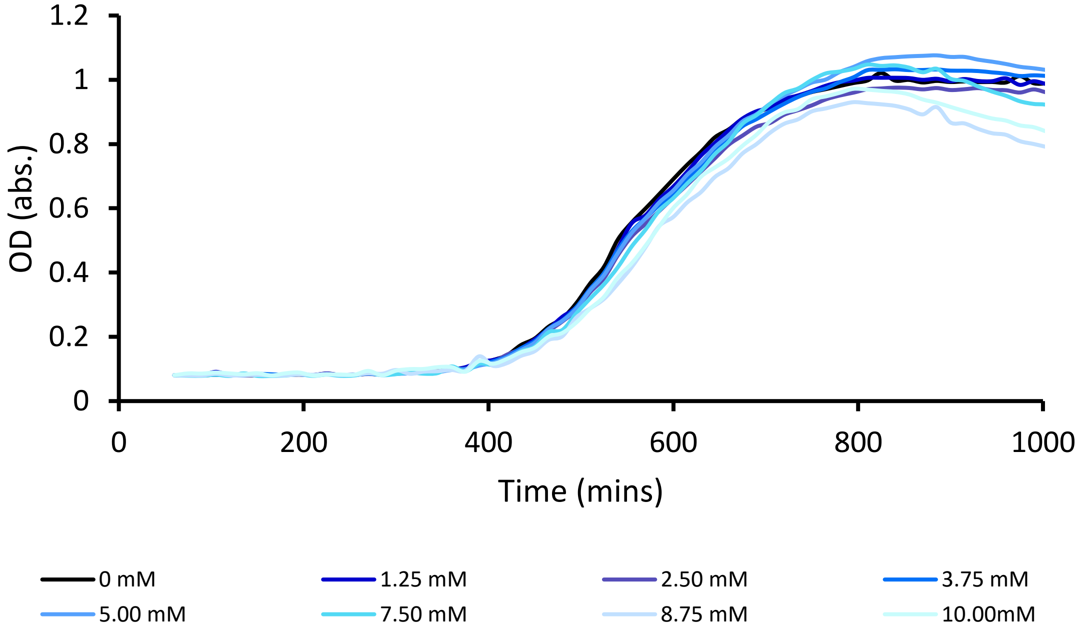

Figure S226 - *E. coli* DH10B growth curves created from an average of 6 optical density readings for each concentration in the presence of compound **42** at varying concentrations. Unable to calculate MIC_{50} due to solubility.

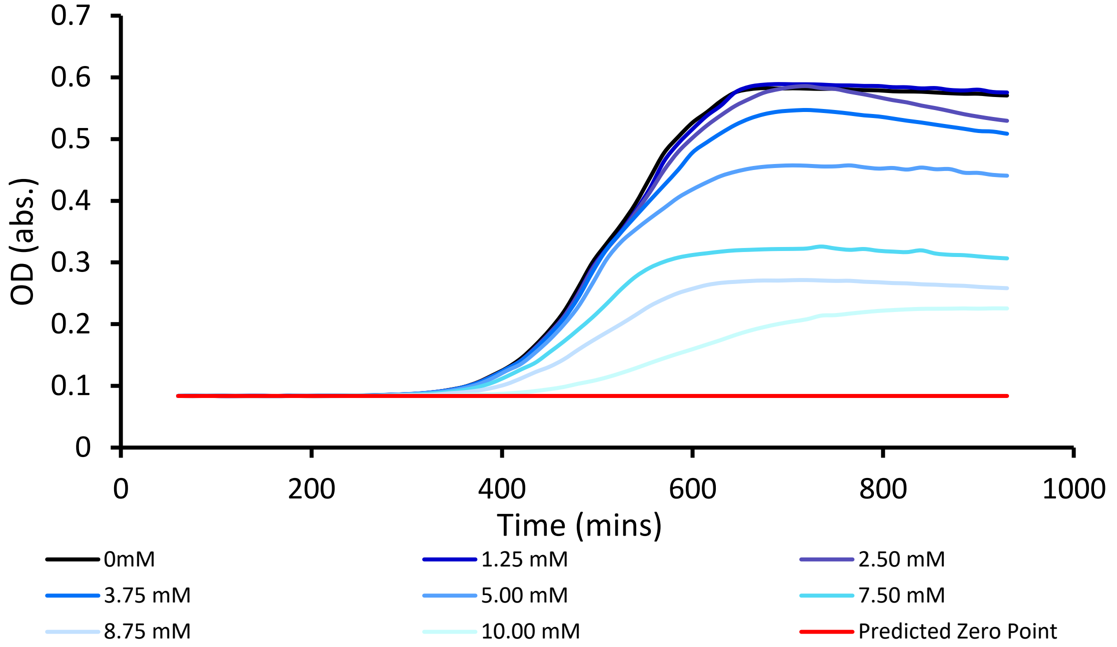

Figure S227 - *E. coli* DH10B growth curves created from an average of 6 optical density readings for each concentration in the presence of compound **43** at varying concentrations. MIC_{50} was calculated using predicted endpoint due to solubility.

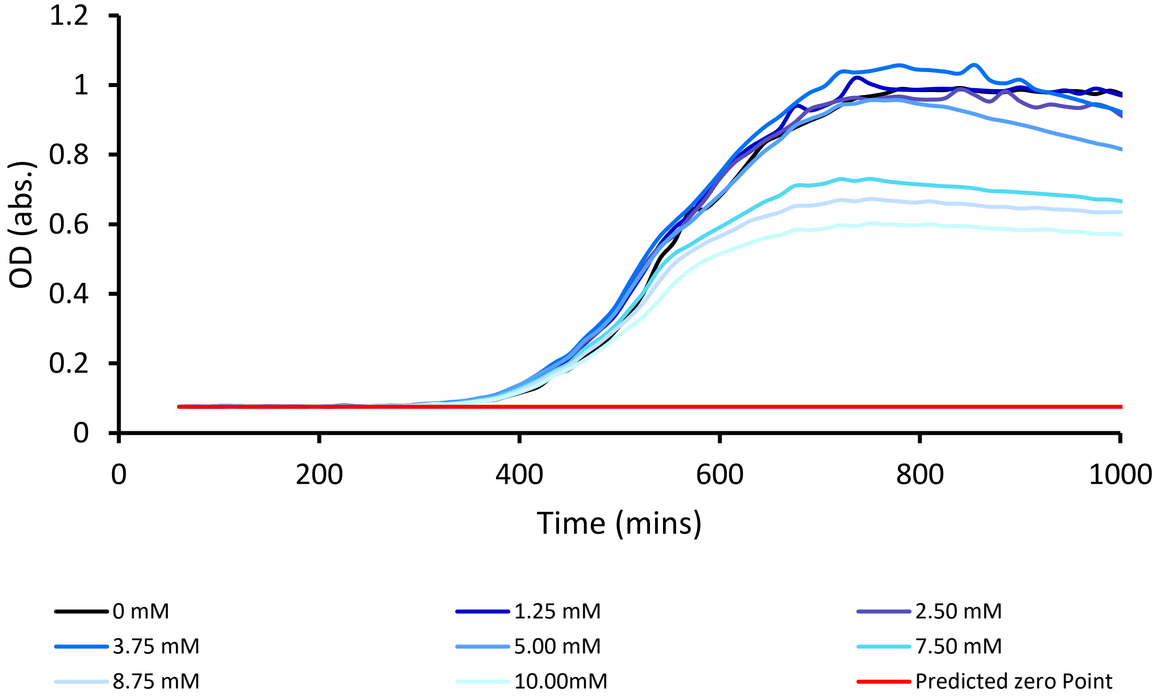

Figure S228 - *E. coli* DH10B growth curves created from an average of 6 optical density readings for each concentration in the presence of compound **45** at varying concentrations. MIC_{50} was calculated using predicted endpoint due to solubility.

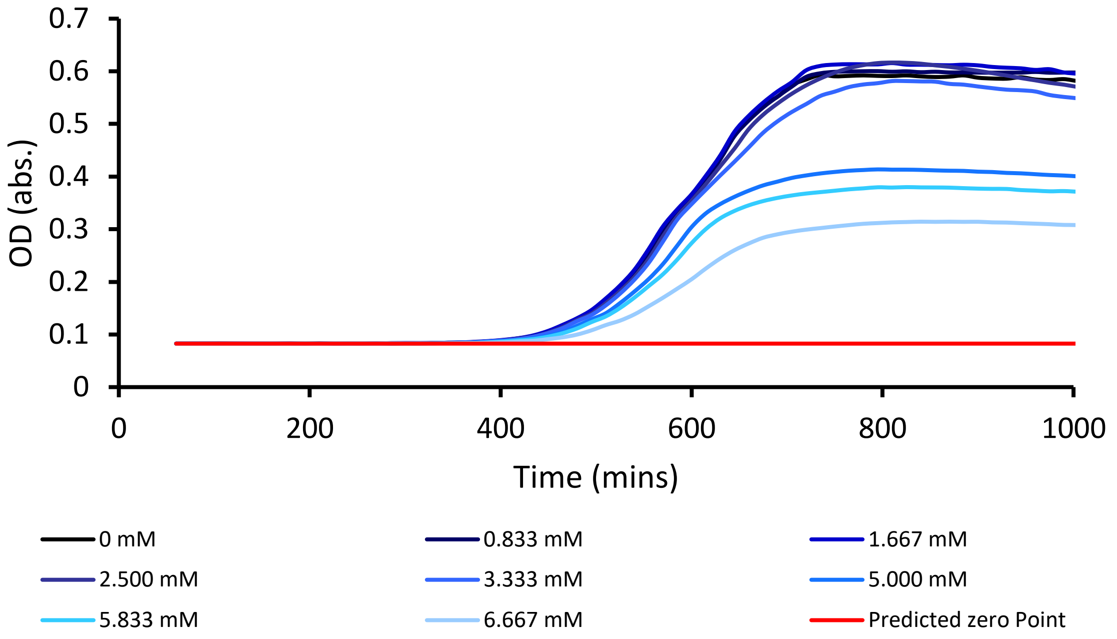

Figure S229 - *E. coli* DH10B growth curves created from an average of 6 optical density readings for each concentration in the presence of compound **46** at varying concentrations. MIC_{50} was calculated using predicted endpoint due to solubility.

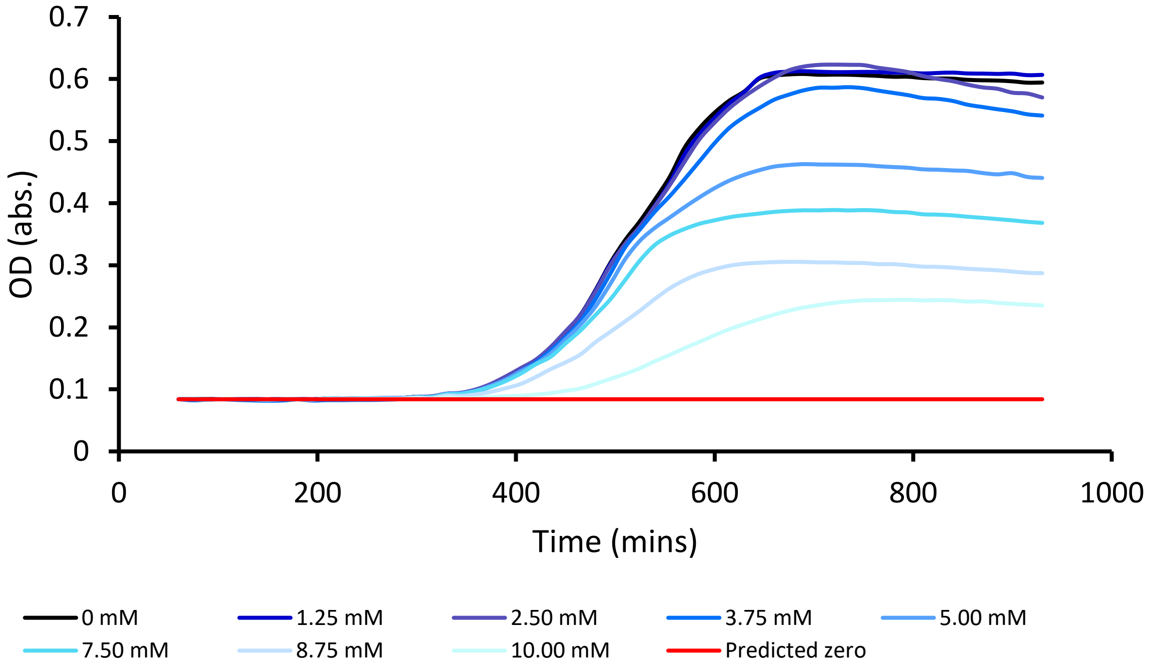

Figure S230 - *E. coli* DH10B growth curves created from an average of 6 optical density readings for each concentration in the presence of compound **50** at varying concentrations. MIC_{50} was calculated using predicted endpoint due to solubility.

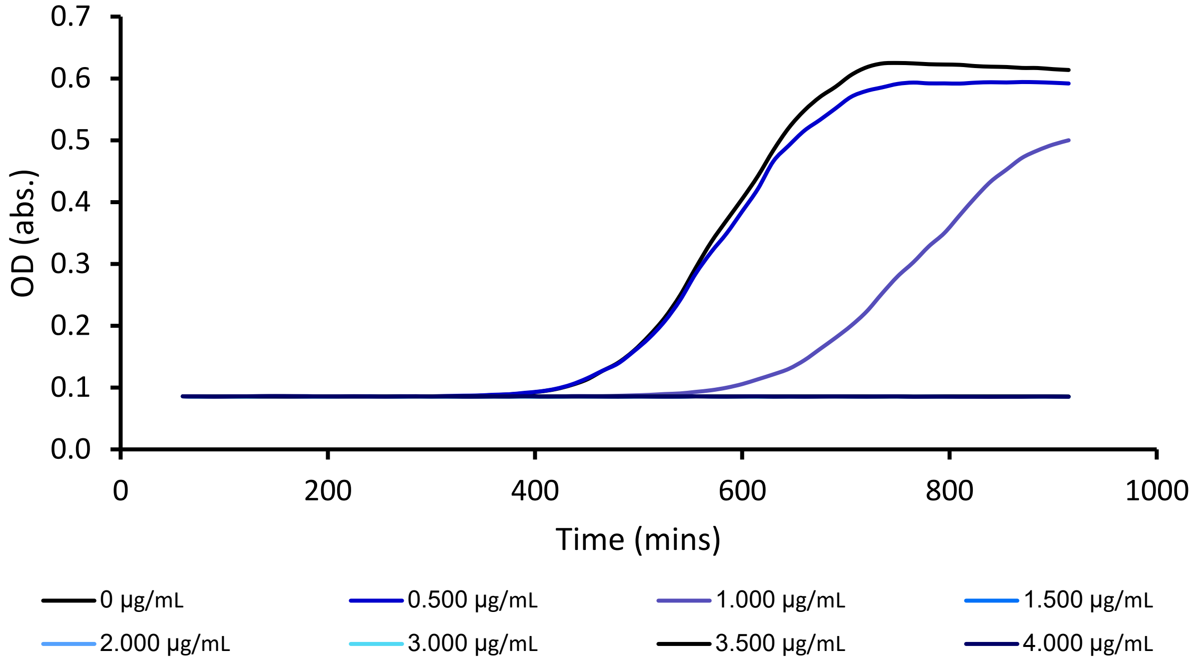

Figure S231 - *E. coli* DH10B growth curves created from an average of 6 optical density readings for each concentration in the presence of compound **ampicillin** at varying concentrations.

Origin Graphs

USA 300 Methicillin-resistant *Staphylococcus aureus* (MRSA)

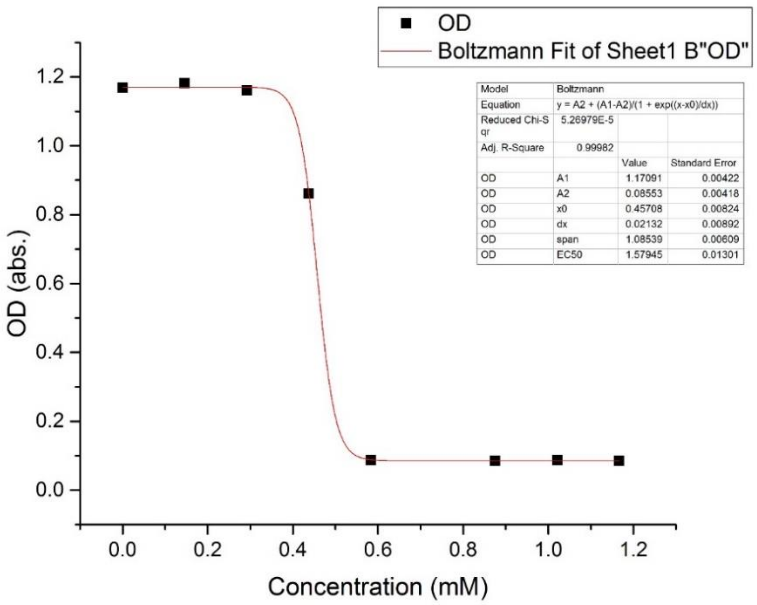

Figure S232 – Boltzmann fit used to calculate MIC_{50} using optical density values at 900 minutes for compound **1** at varying concentrations.

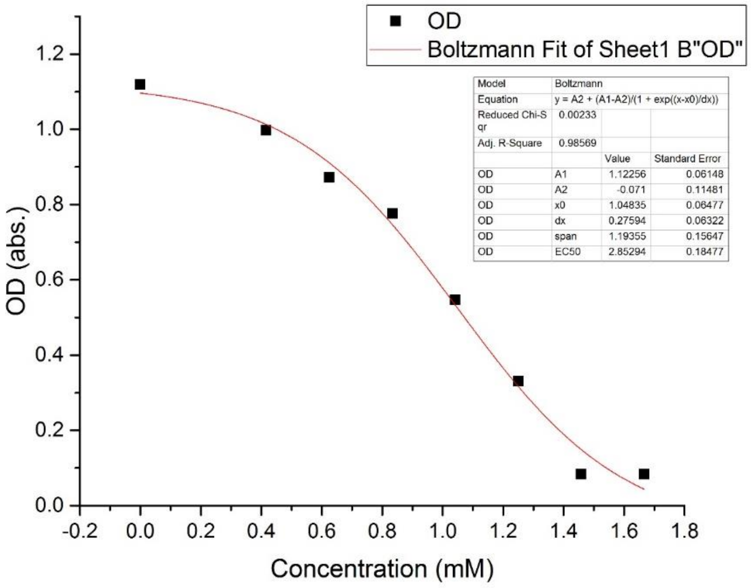

Figure S233 – Boltzmann fit used to calculate MIC_{50} using optical density values at 900 minutes for compound **2** at varying concentrations.

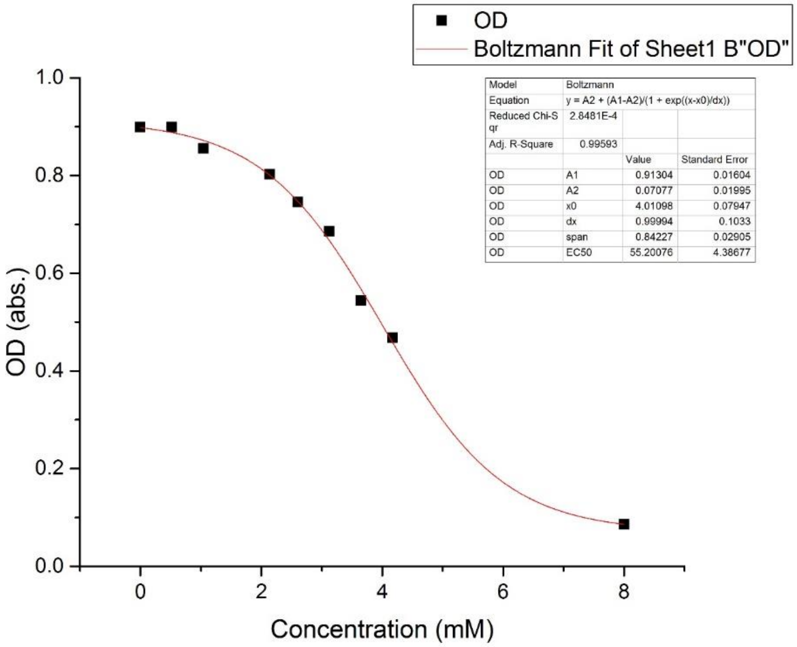

Figure S234 – Boltzmann fit used to calculate MIC_{50} using optical density values at 900 minutes for compound **4** at varying concentrations.

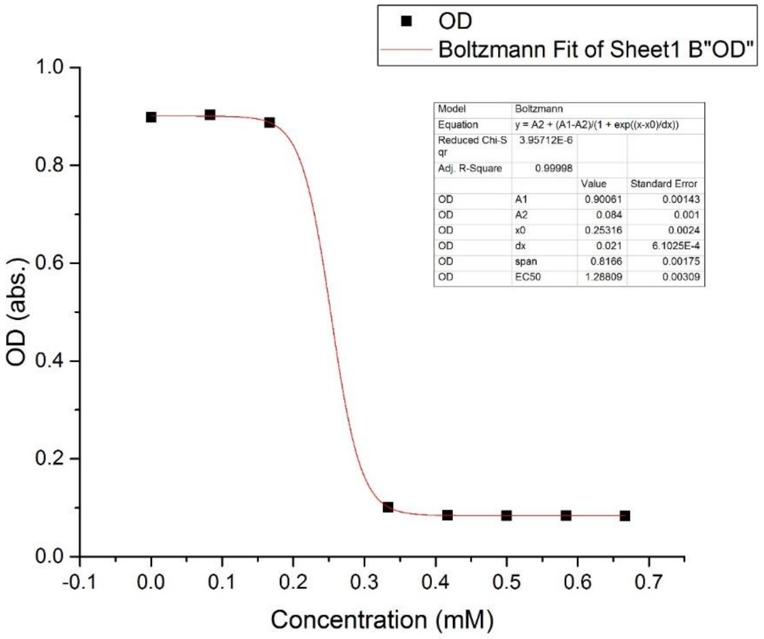

Figure S235 – Boltzmann fit used to calculate MIC_{50} using optical density values at 900 minutes for compound **5** at varying concentrations.

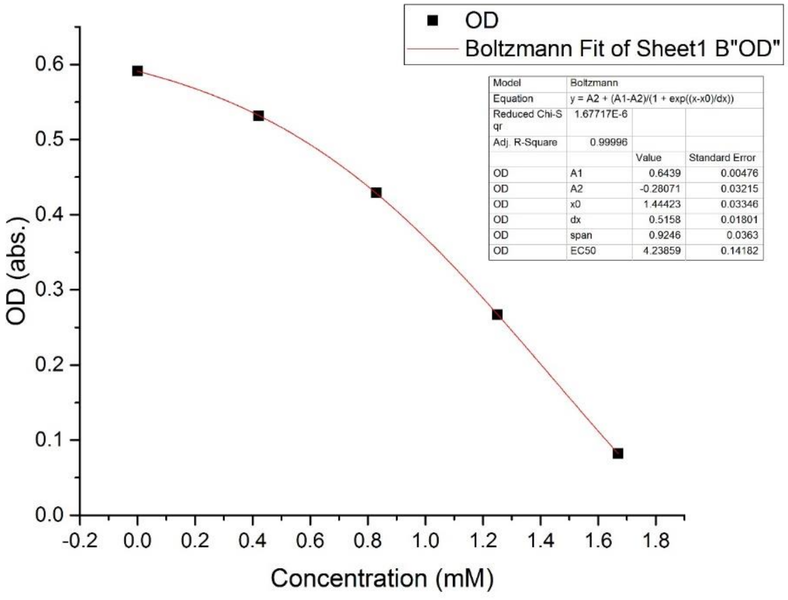

Figure S236 – Boltzmann fit used to calculate MIC_{50} using optical density values at 900 minutes for compound **6** at varying concentrations.

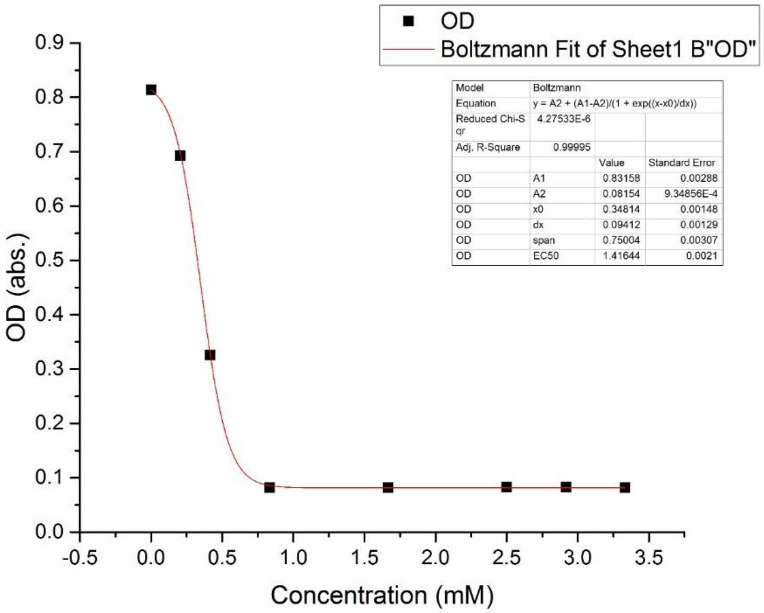

Figure S237 Boltzmann fit used to calculate MIC_{50} using optical density values at 900 minutes for compound **8** at varying concentrations.

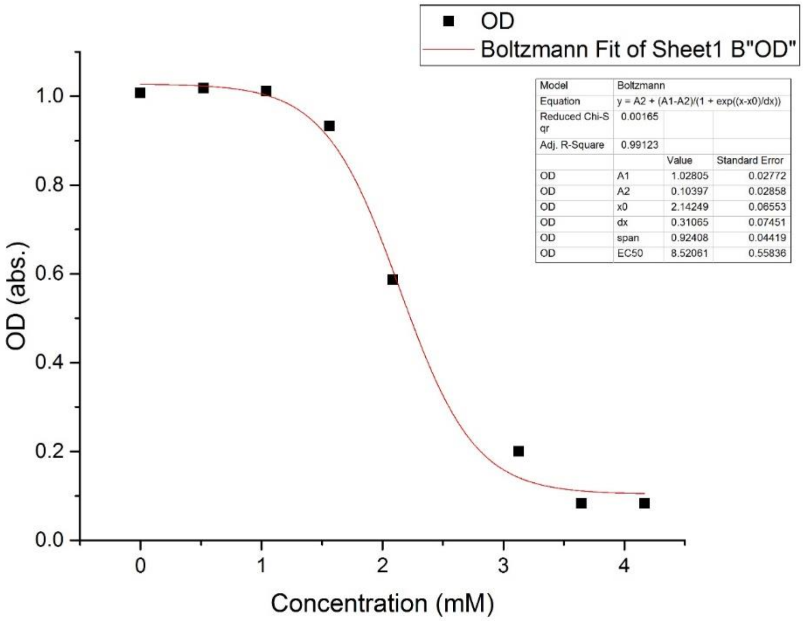

Figure S238 – Boltzmann fit used to calculate MIC_{50} using optical density values at 900 minutes for compound **9** at varying concentrations.

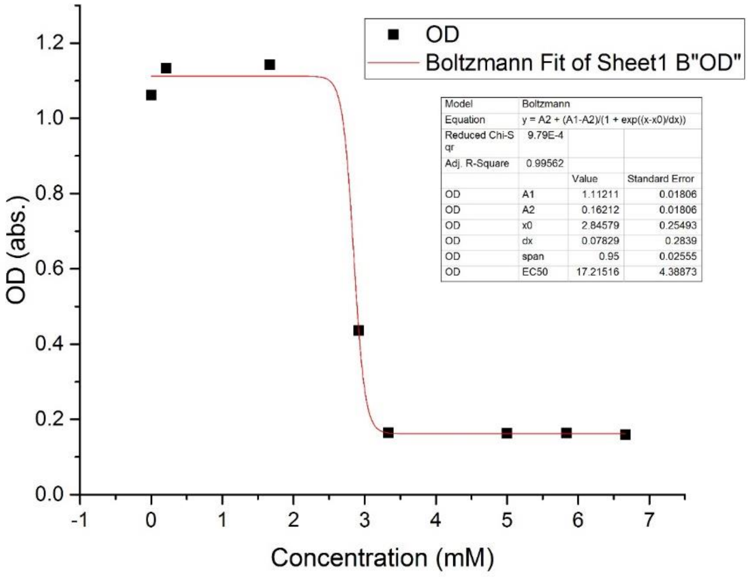

Figure S239 – Boltzmann fit used to calculate MIC_{50} using optical density values at 900 minutes for compound **10** at varying concentrations.

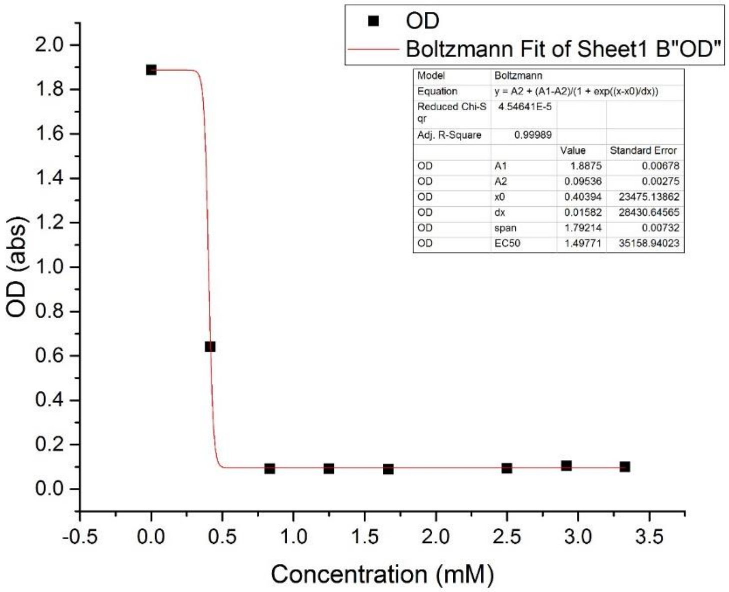

Figure S240 – Boltzmann fit used to calculate MIC_{50} using optical density values at 900 minutes for compound **11** at varying concentrations.

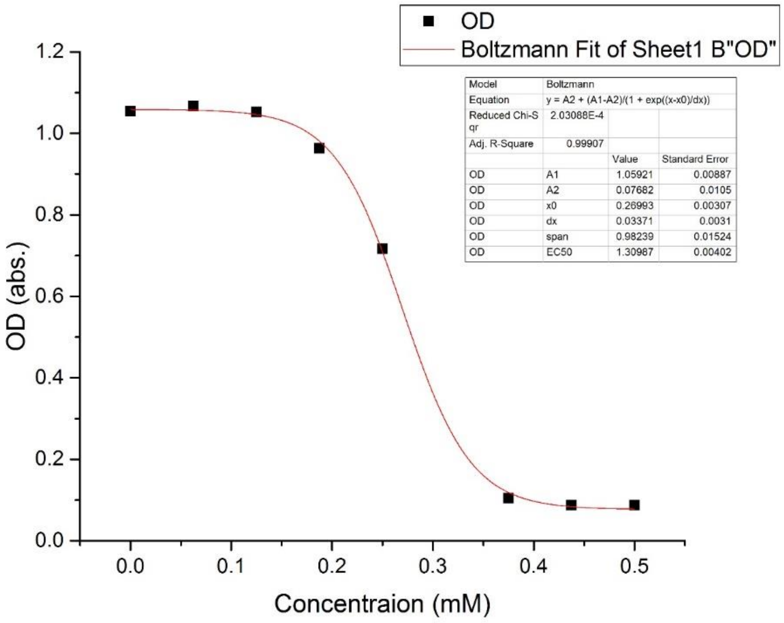

Figure S241 – Boltzmann fit used to calculate MIC_{50} using optical density values at 900 minutes for compound **16** at varying concentrations.

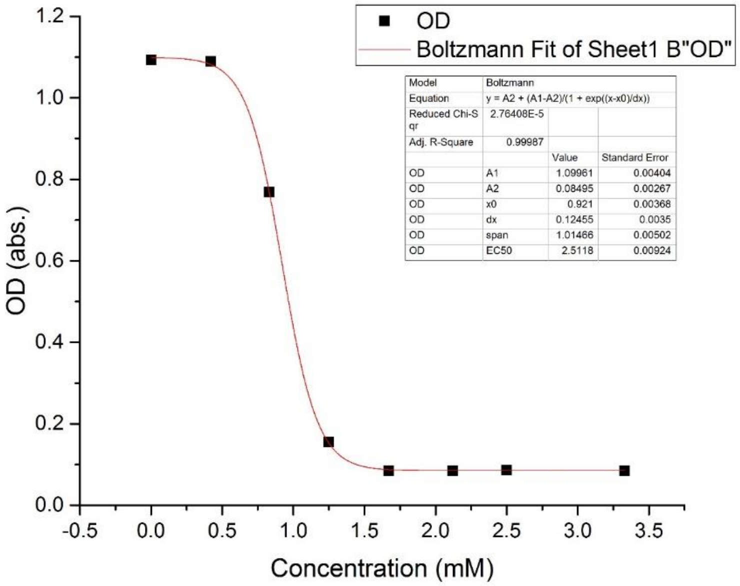

Figure S242 – Boltzmann fit used to calculate MIC_{50} using optical density values at 900 minutes for compound **17** at varying concentrations.

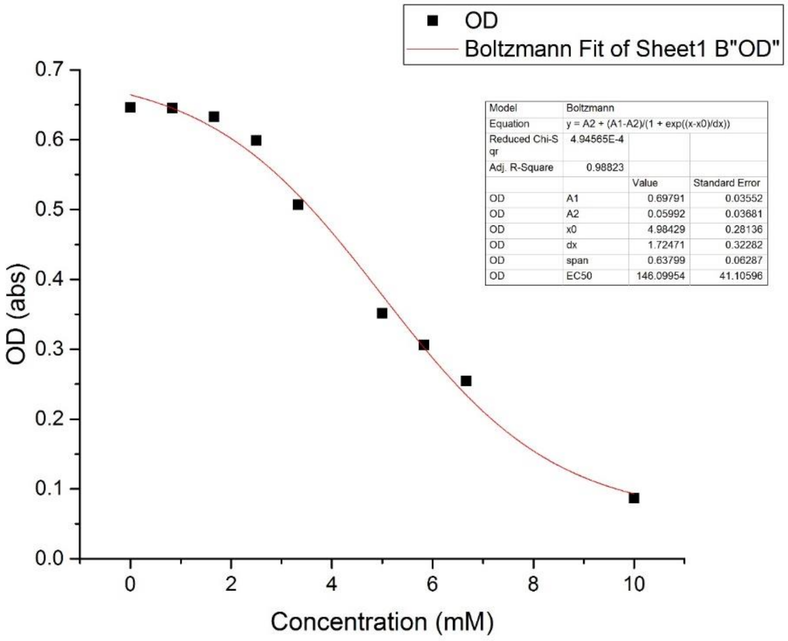

Figure S243 – Boltzmann fit used to calculate MIC_{50} using optical density values at 900 minutes for compound **18** at varying concentrations.

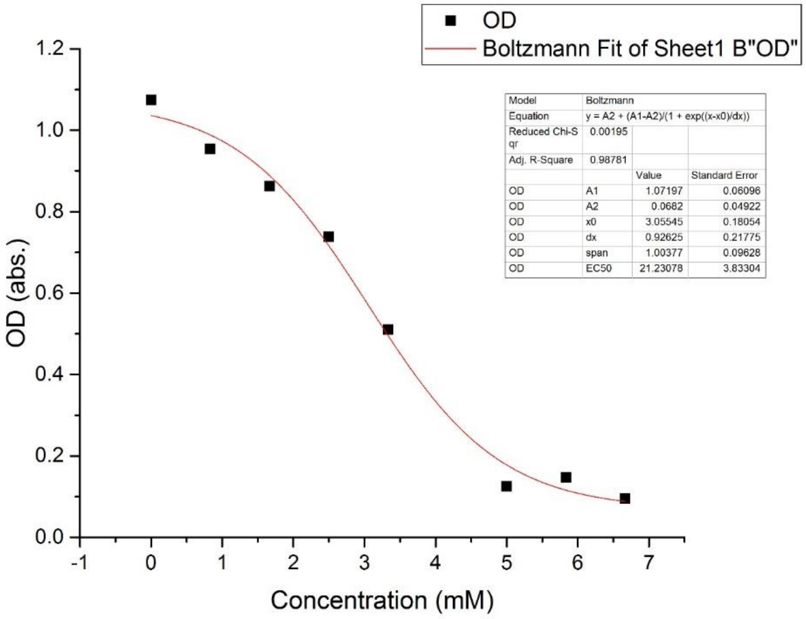

Figure S244 – Boltzmann fit used to calculate MIC_{50} using optical density values at 900 minutes for compound **19** at varying concentrations.

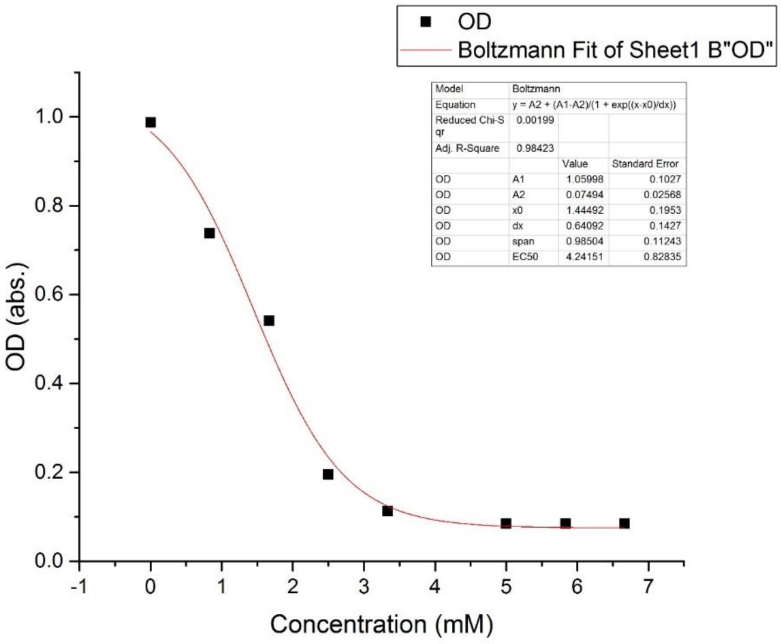

Figure S245 – Boltzmann fit used to calculate MIC_{50} using optical density values at 900 minutes for compound **20** at varying concentrations.

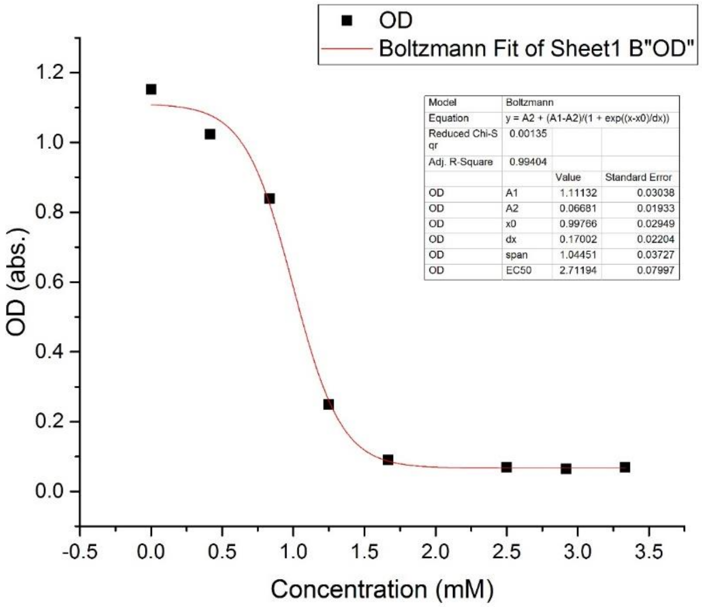

Figure S246 Boltzmann fit used to calculate MIC_{50} using optical density values at 900 minutes for compound **21** at varying concentrations.

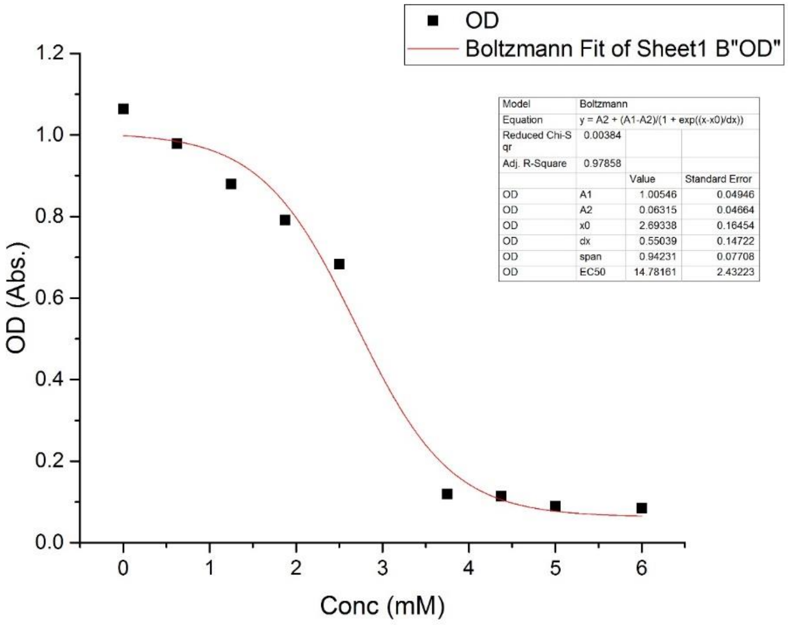

Figure S247 – Boltzmann fit used to calculate MIC_{50} using optical density values at 900 minutes for compound **22** at varying concentrations.

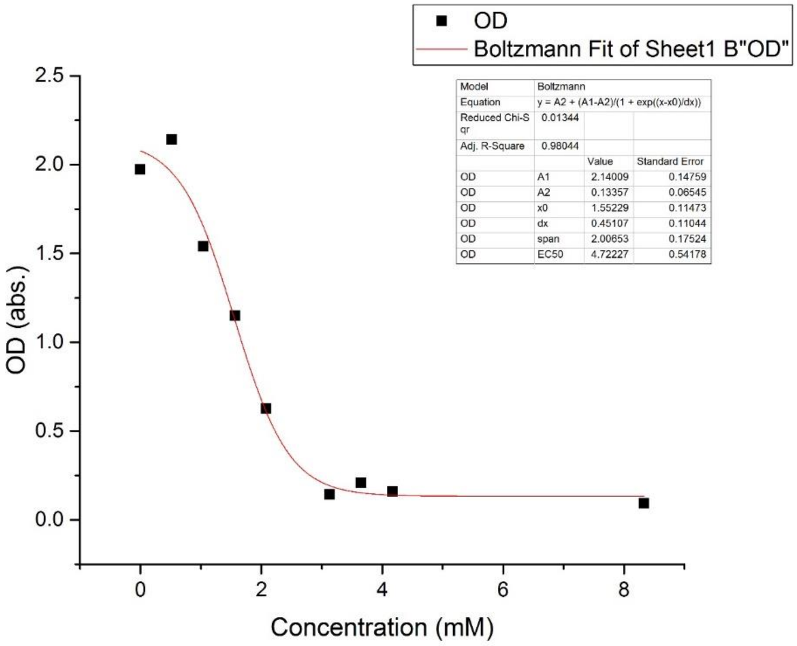

Figure S248 – Boltzmann fit used to calculate MIC_{50} using optical density values at 900 minutes for compound **23** at varying concentrations.

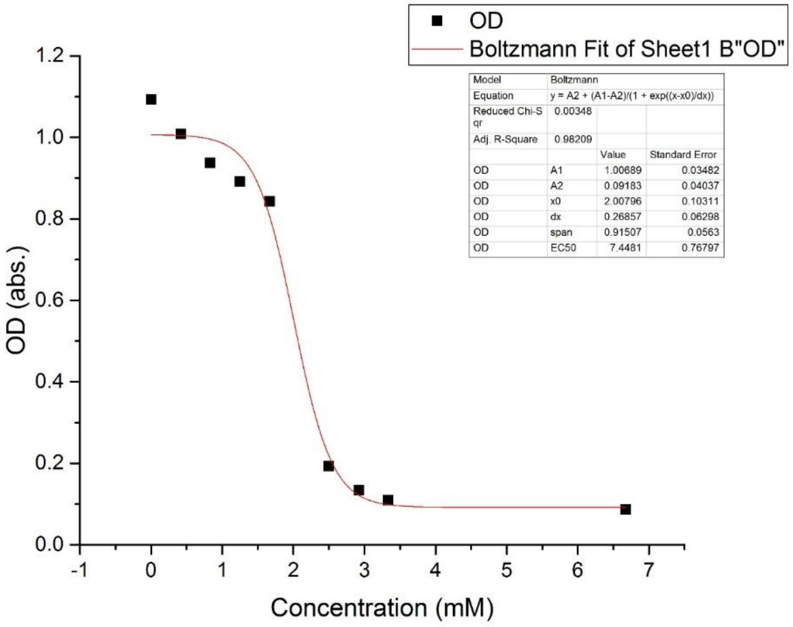

Figure S249 – Boltzmann fit used to calculate MIC_{50} using optical density values at 900 minutes for compound **24** at varying concentrations.

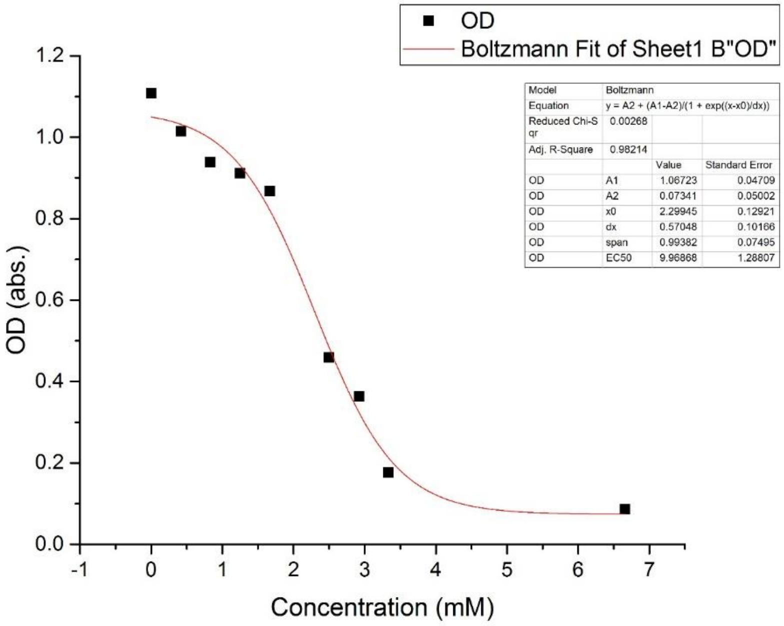

Figure S250 – Boltzmann fit used to calculate MIC_{50} using optical density values at 900 minutes for compound **25** at varying concentrations.

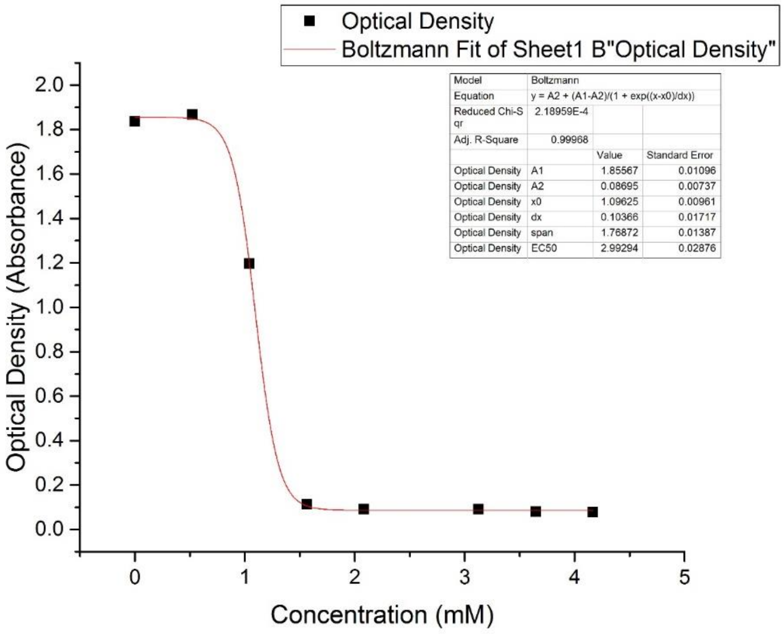

Figure S251 – Boltzmann fit used to calculate MIC_{50} using optical density values at 900 minutes for compound **27** at varying concentrations.

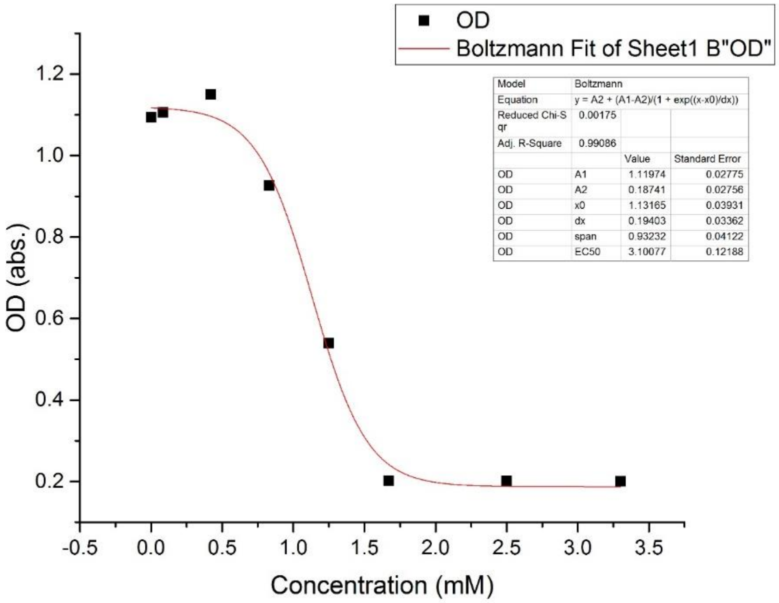

Figure S252 – Boltzmann fit used to calculate MIC_{50} using optical density values at 900 minutes for compound **30** at varying concentrations.

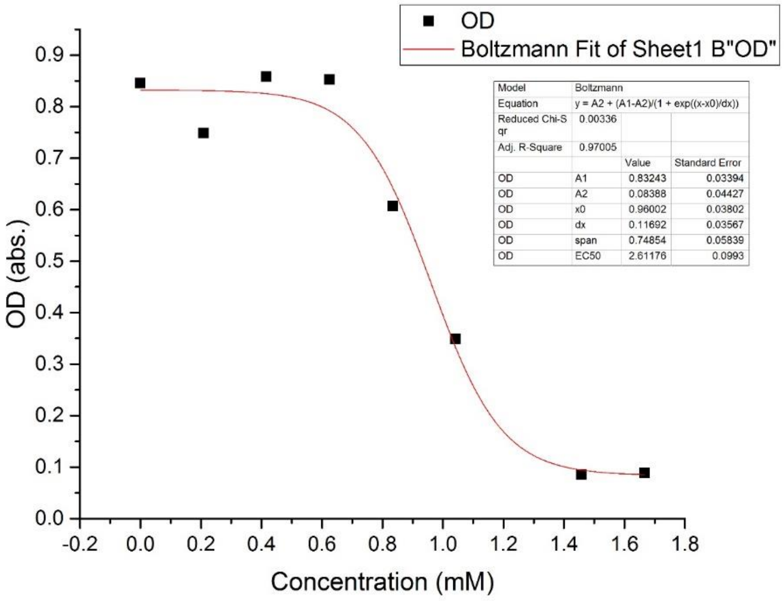

Figure S253 Boltzmann fit used to calculate MIC_{50} using optical density values at 900 minutes for compound **31** at varying concentrations.

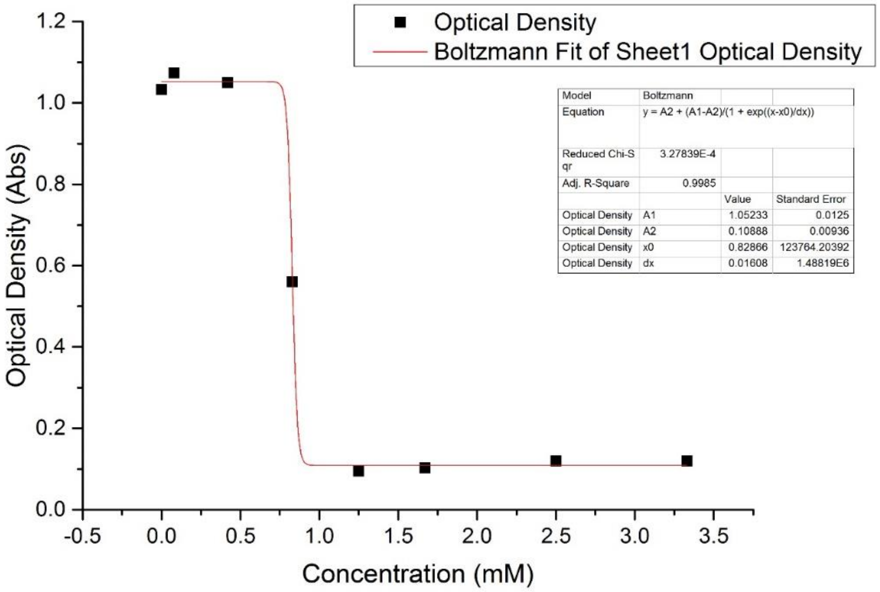

Figure S254 – Boltzmann fit used to calculate MIC_{50} using optical density values at 900 minutes for compound **32** at varying concentrations.

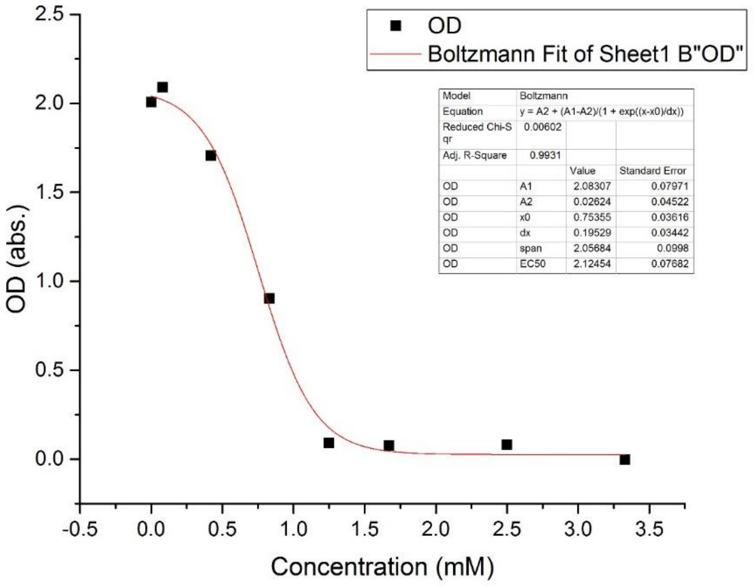

Figure S255 – Boltzmann fit used to calculate MIC_{50} using optical density values at 900 minutes for compound **33** at varying concentrations.

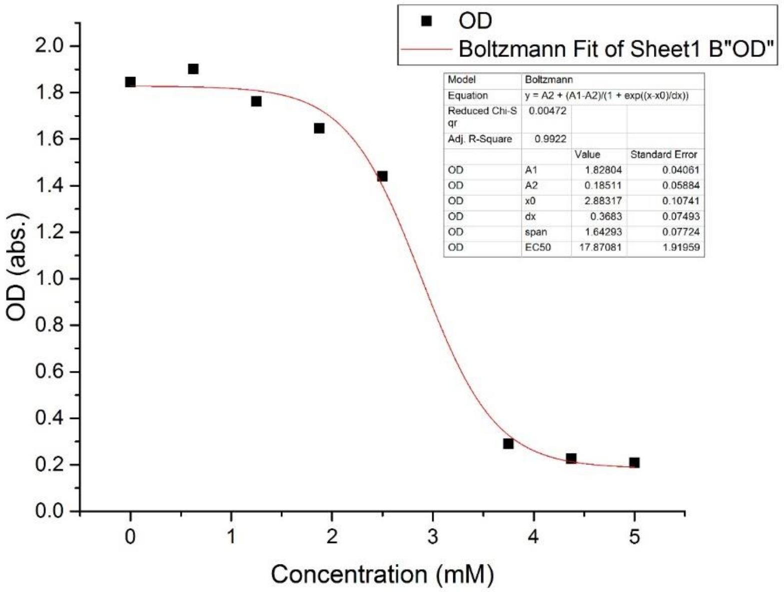

Figure S256 – Boltzmann fit used to calculate MIC_{50} using optical density values at 900 minutes for compound **34** at varying concentrations.

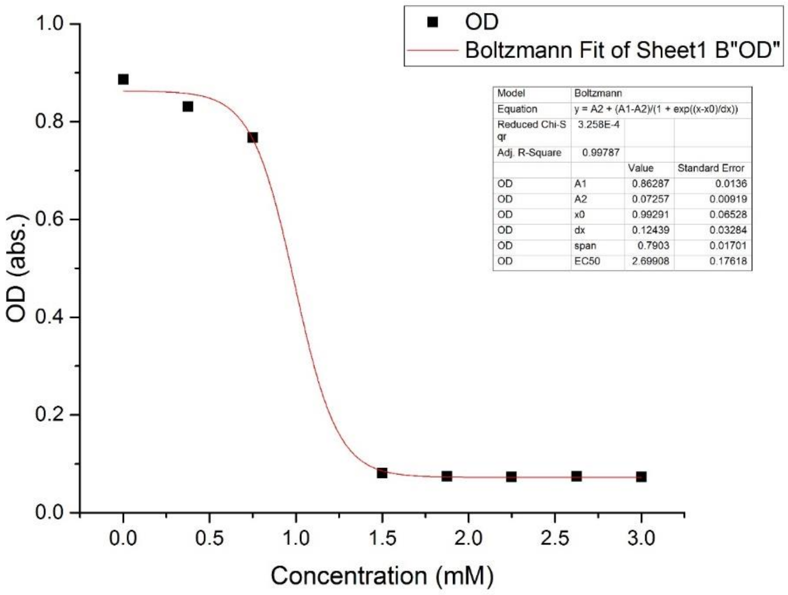

Figure S257 Boltzmann fit used to calculate MIC_{50} using optical density values at 900 minutes for compound **38** at varying concentrations.

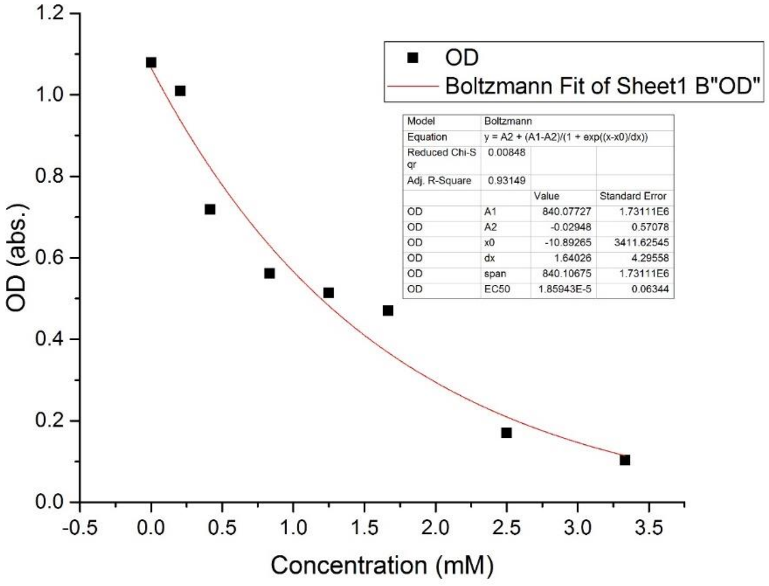

Figure S258 – Boltzmann fit used to calculate MIC_{50} using optical density values at 900 minutes for compound **39** at varying concentrations.

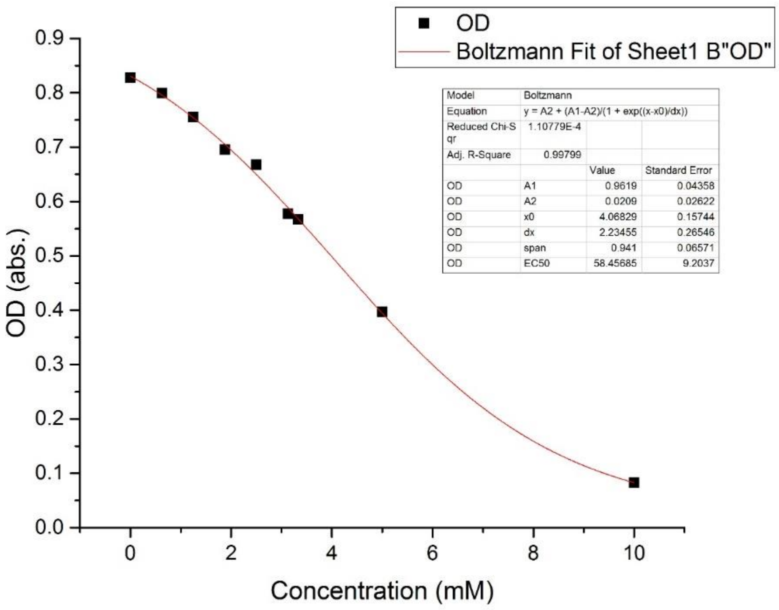

Figure S259 – Boltzmann fit used to calculate MIC_{50} using optical density values at 900 minutes for compound **40** at varying concentrations.

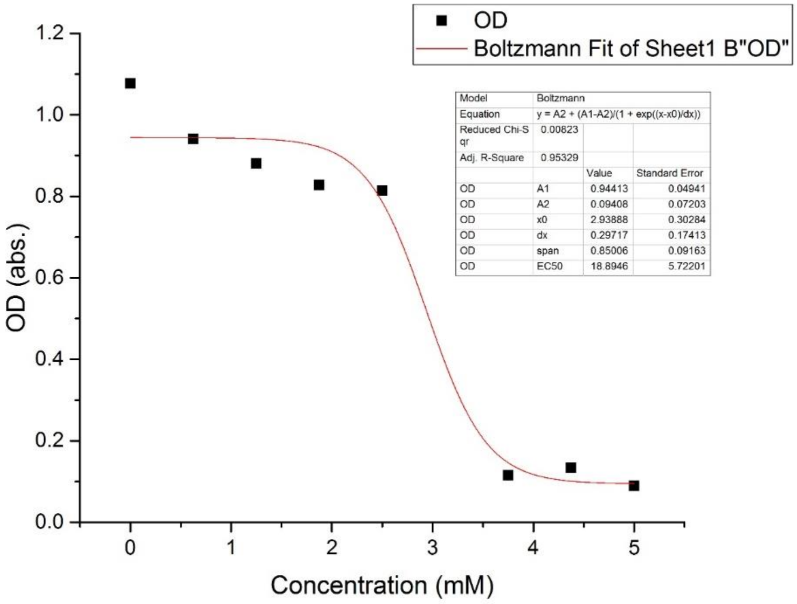

Figure S260 – Boltzmann fit used to calculate MIC_{50} using optical density values at 900 minutes for compound **41** at varying concentrations.

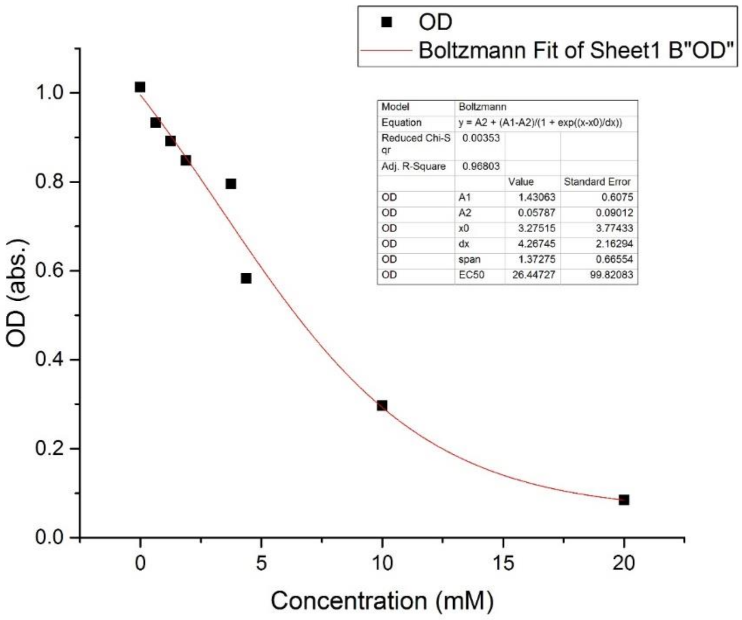

Figure S261 – Boltzmann fit used to calculate MIC_{50} using optical density values at 900 minutes for compound **42** at varying concentrations.

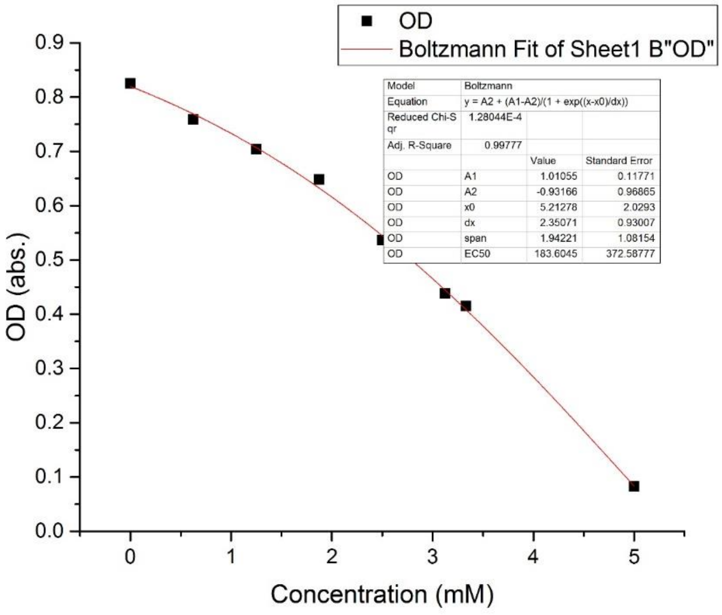

Figure S262 – Boltzmann fit used to calculate MIC_{50} using optical density values at 900 minutes for compound **43** at varying concentrations.

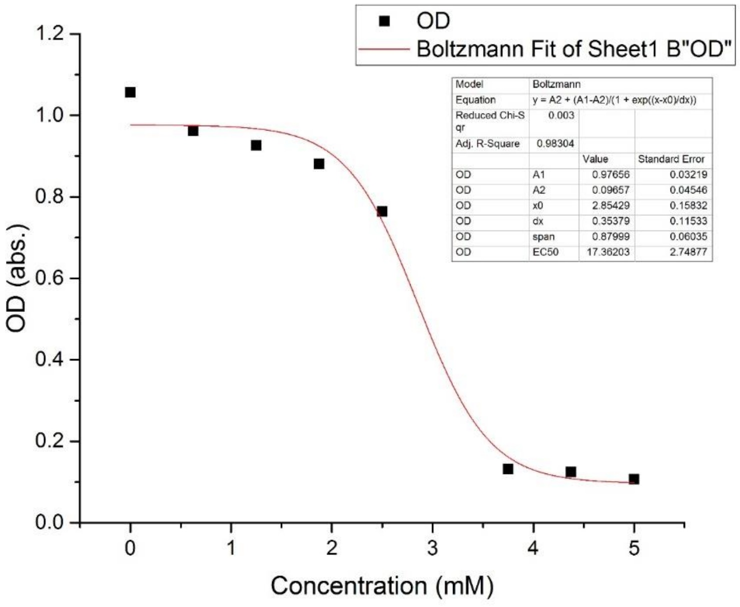

Figure S263 – Boltzmann fit used to calculate MIC_{50} using optical density values at 900 minutes for compound **44** at varying concentrations.

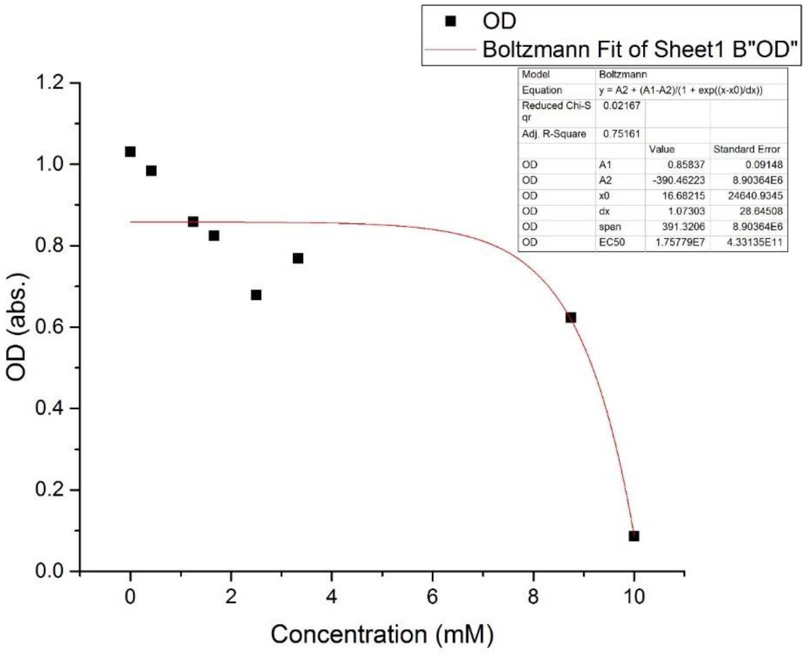

Figure S264 – Boltzmann fit used to calculate MIC_{50} using optical density values at 900 minutes for compound **45** at varying concentrations.

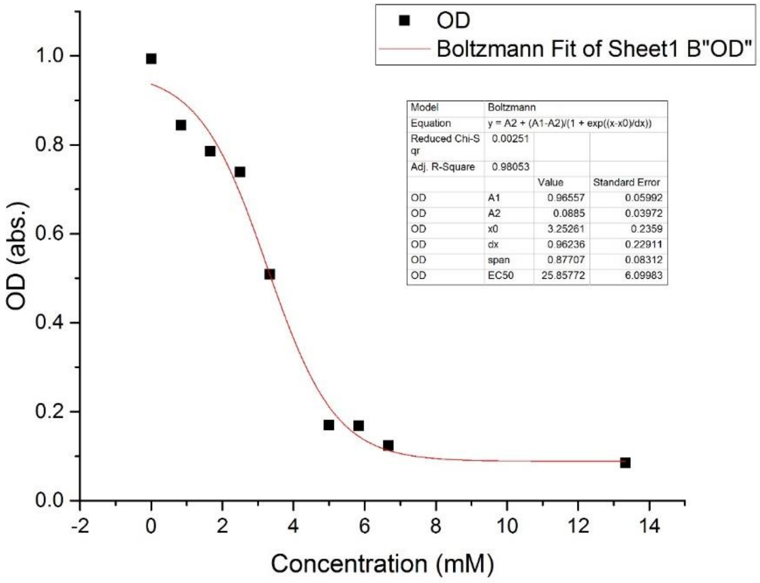

Figure S265 – Boltzmann fit used to calculate MIC_{50} using optical density values at 900 minutes for compound **46** at varying concentrations.

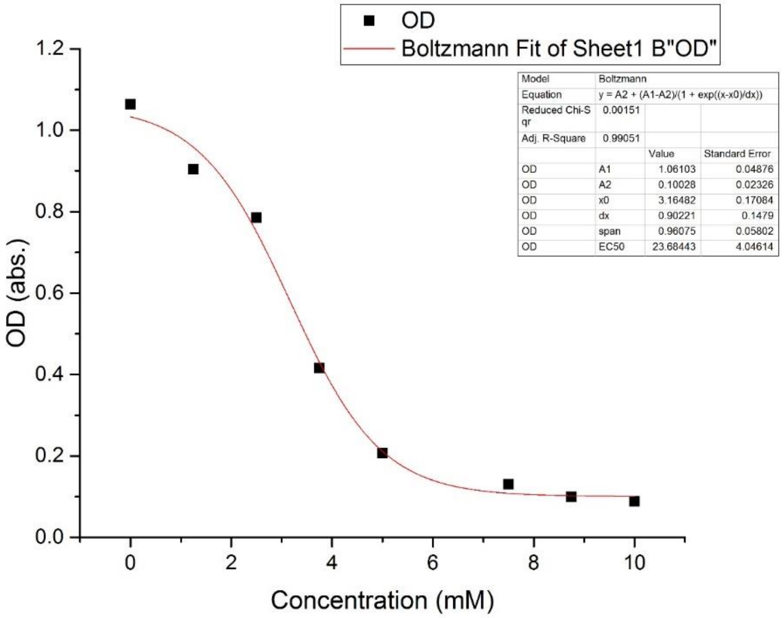

Figure S266 – Boltzmann fit used to calculate MIC_{50} using optical density values at 900 minutes for compound **50** at varying concentrations.

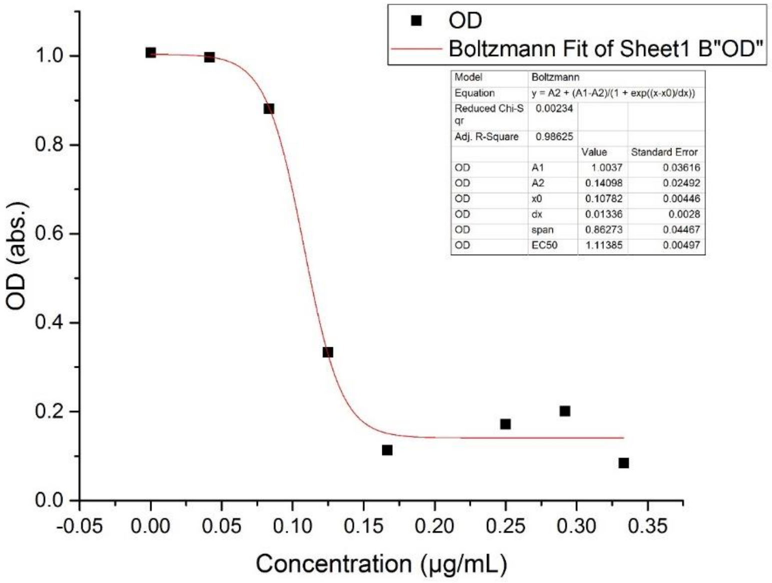

Figure S267 – Boltzmann fit used to calculate MIC_{50} using optical density values at 900 minutes for **ampicillin** at varying concentrations.

Escherichia coli (*E. coli*)

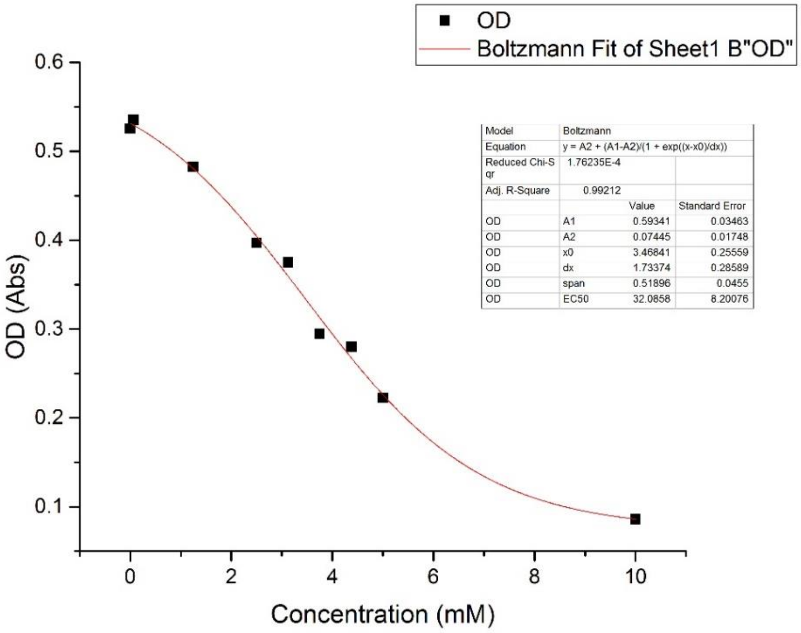

Figure S268 – Boltzmann fit used to calculate MIC_{50} using optical density values at 900 minutes for compound **1** at varying concentrations.

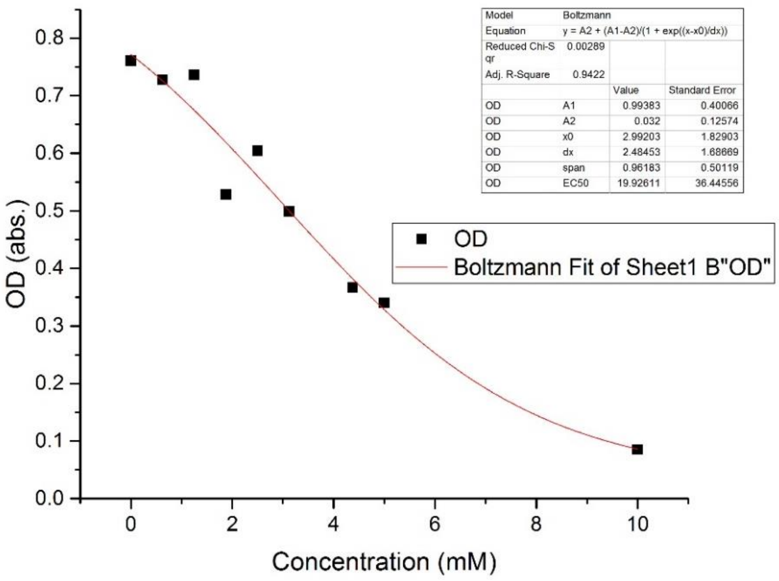

Figure S269 – Boltzmann fit used to calculate MIC_{50} using optical density values at 900 minutes for compound **2** at varying concentrations.

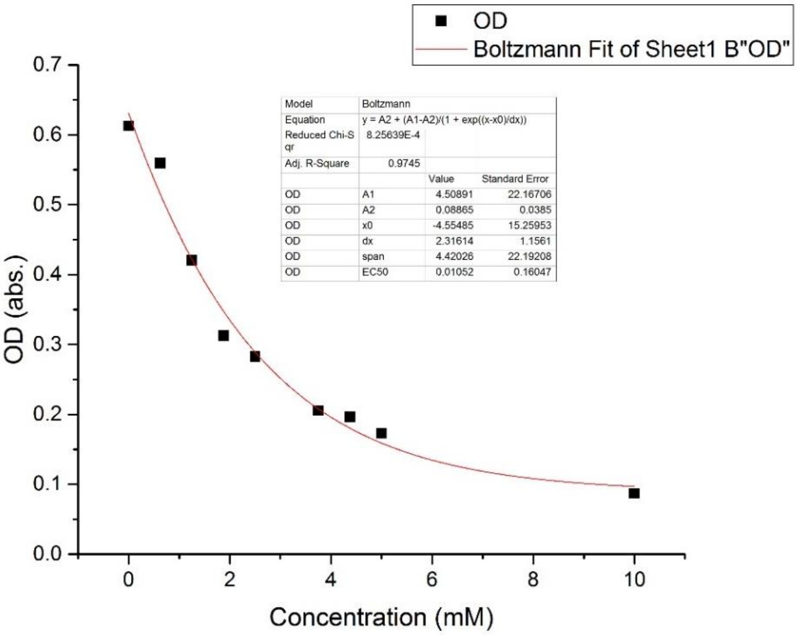

Figure S270 – Boltzmann fit used to calculate MIC_{50} using optical density values at 900 minutes for compound **3** at varying concentrations.

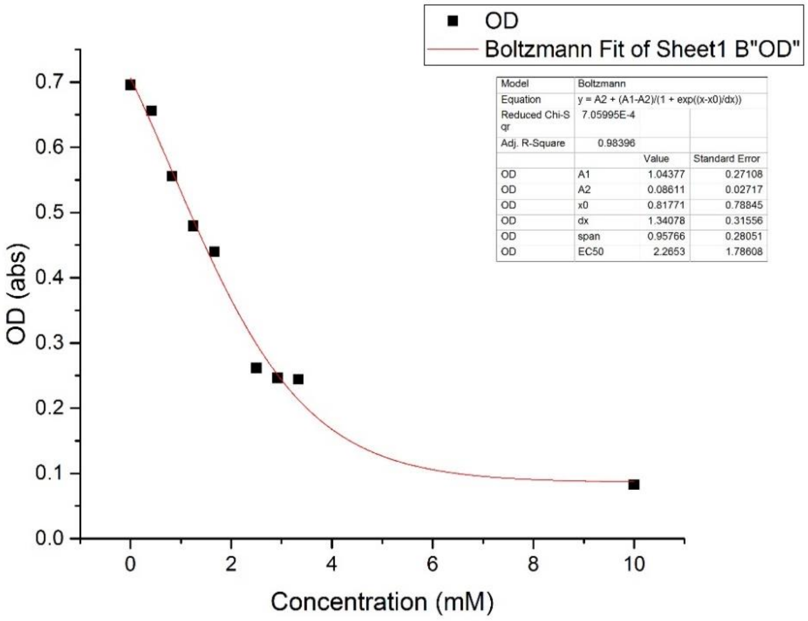

Figure S271 – Boltzmann fit used to calculate MIC_{50} using optical density values at 900 minutes for compound **6** at varying concentrations.

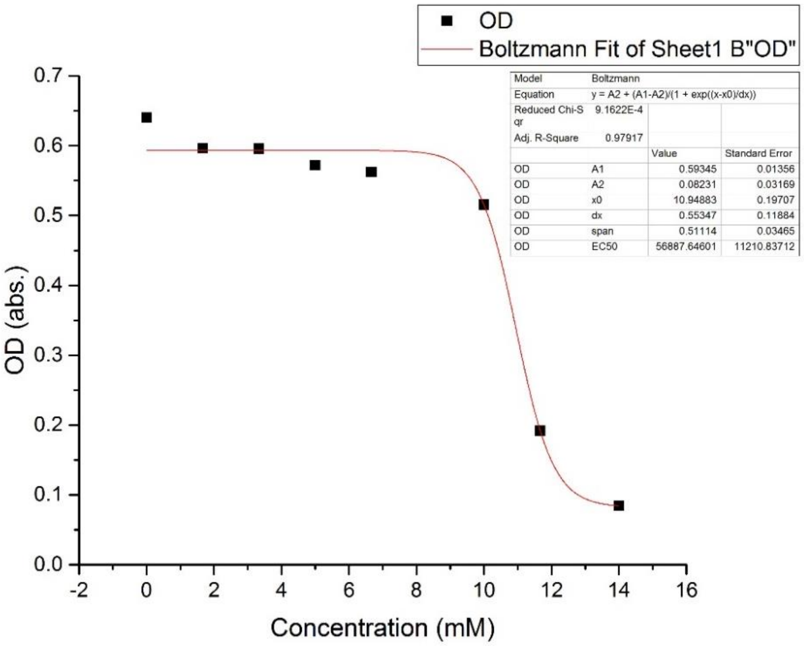

Figure S272 – Boltzmann fit used to calculate MIC_{50} using optical density values at 900 minutes for compound **9** at varying concentrations.

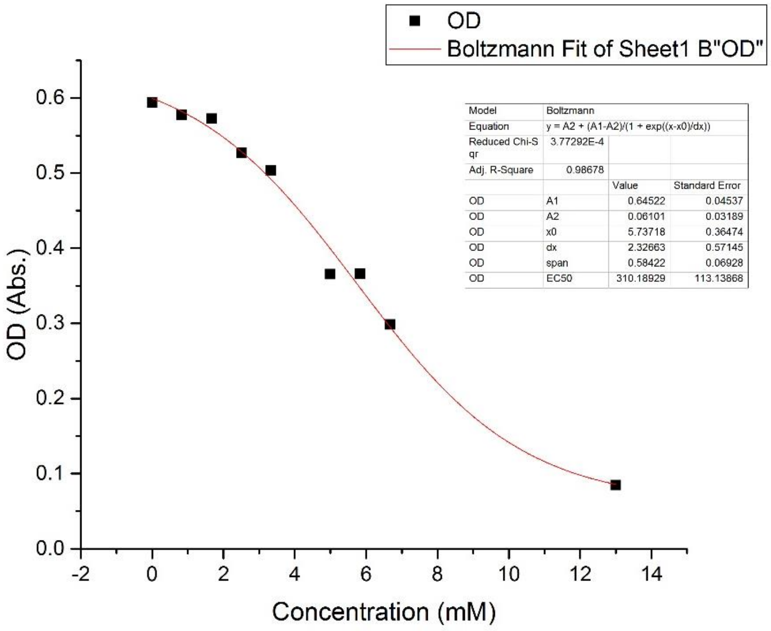

Figure S273 – Boltzmann fit used to calculate MIC_{50} using optical density values at 900 minutes for compound **11** at varying concentrations.

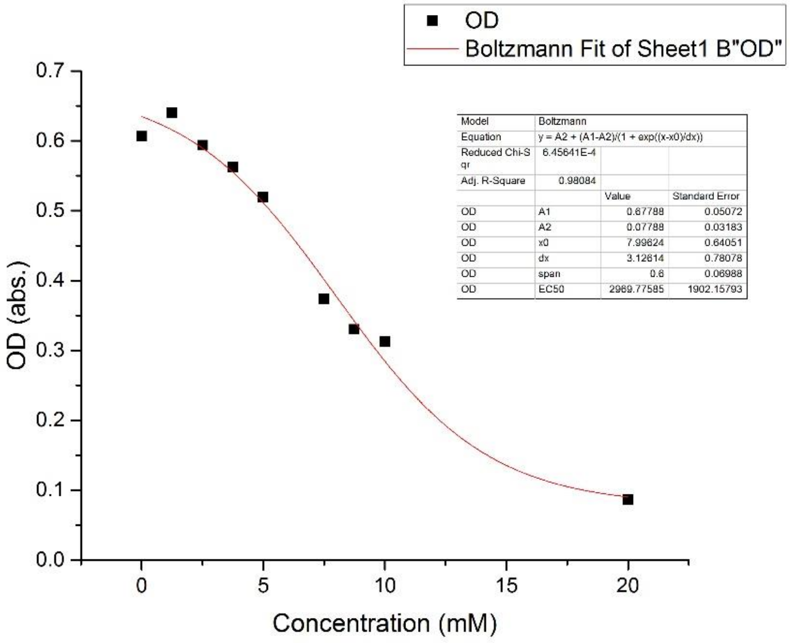

Figure S274 – Boltzmann fit used to calculate MIC_{50} using optical density values at 900 minutes for compound **24** at varying concentrations.

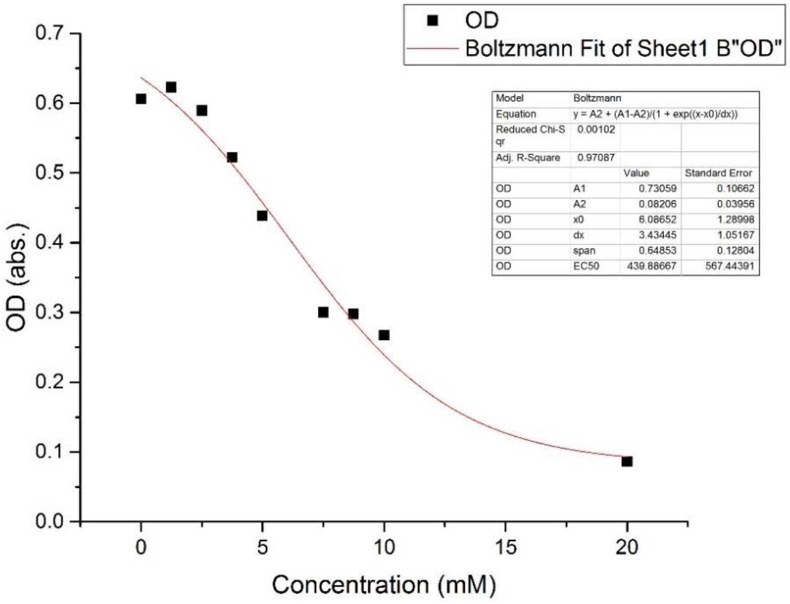

Figure S275 – Boltzmann fit used to calculate MIC_{50} using optical density values at 900 minutes for compound **25** at varying concentrations.

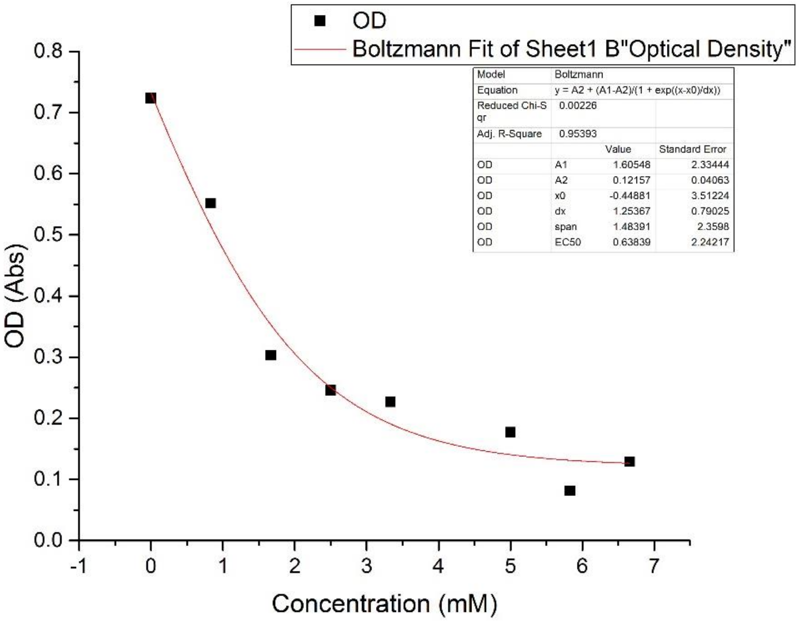

Figure S276 – Boltzmann fit used to calculate MIC_{50} using optical density values at 900 minutes for compound **30** at varying concentrations.

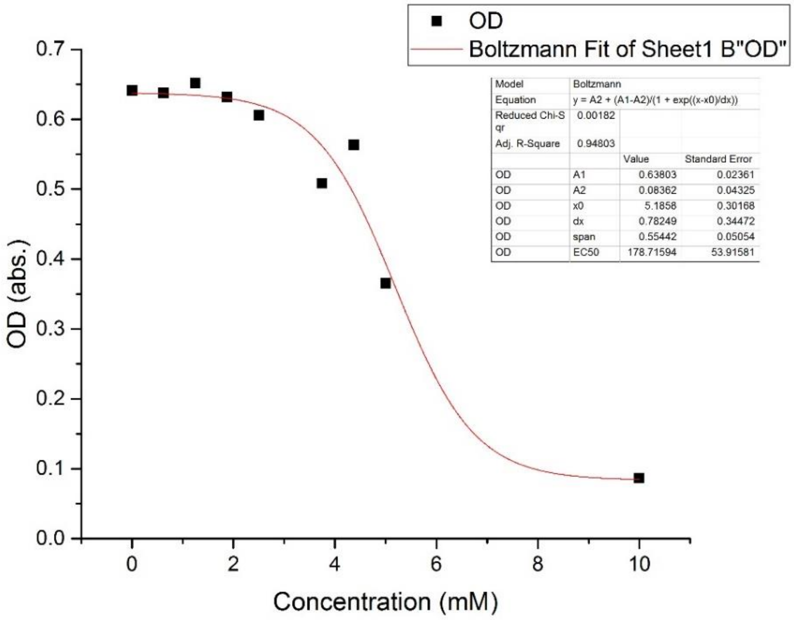

Figure S277 – Boltzmann fit used to calculate MIC_{50} using optical density values at 900 minutes for compound **34** at varying concentrations.

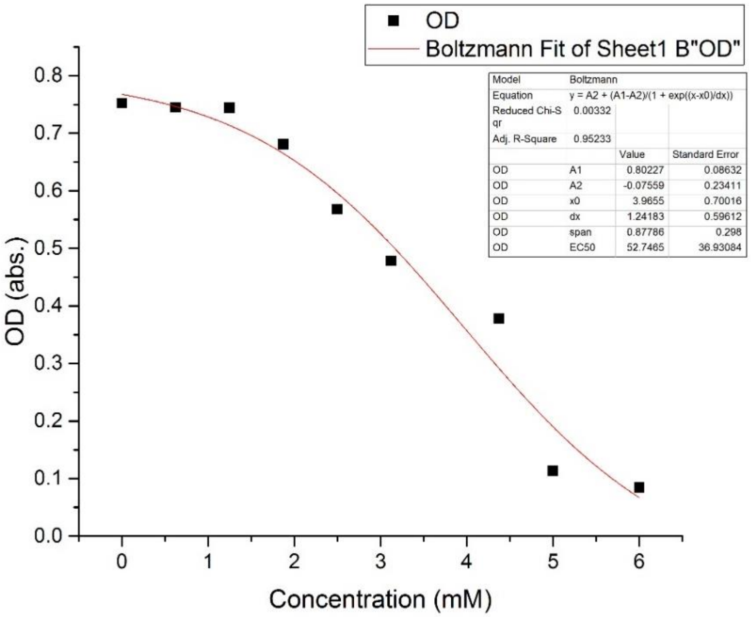

Figure S278 – Boltzmann fit used to calculate MIC_{50} using optical density values at 900 minutes for compound **38** at varying concentrations.

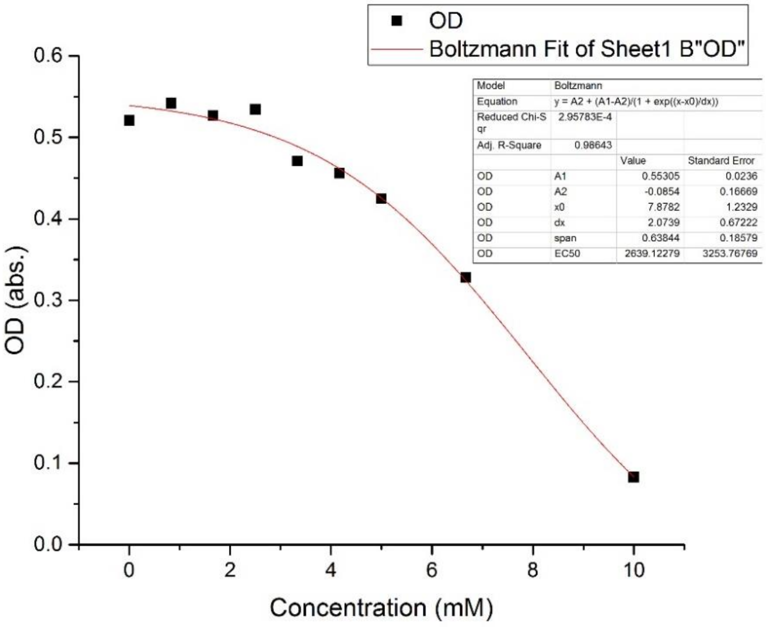

Figure S279 – Boltzmann fit used to calculate MIC_{50} using optical density values at 900 minutes for compound **39** at varying concentrations.

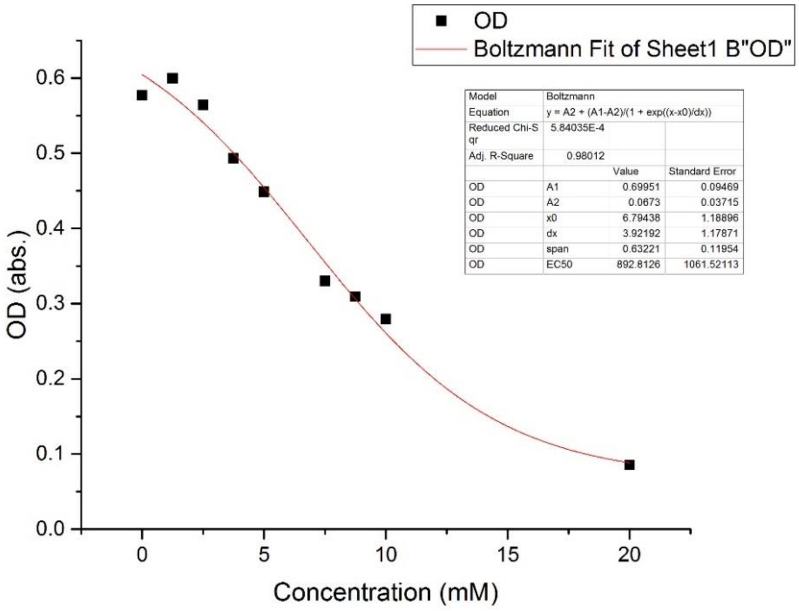

Figure S280 – Boltzmann fit used to calculate MIC_{50} using optical density values at 900 minutes for compound **41** at varying concentrations.

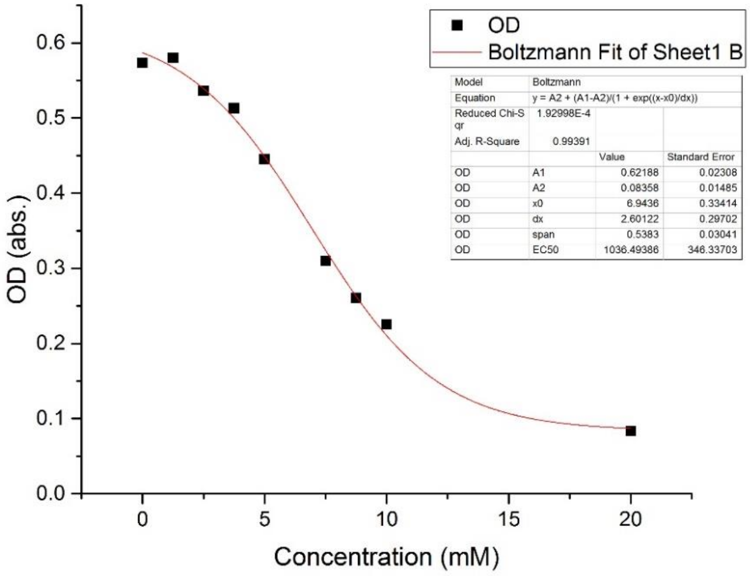

Figure S281 – Boltzmann fit used to calculate MIC_{50} using optical density values at 900 minutes for compound **43** at varying concentrations.

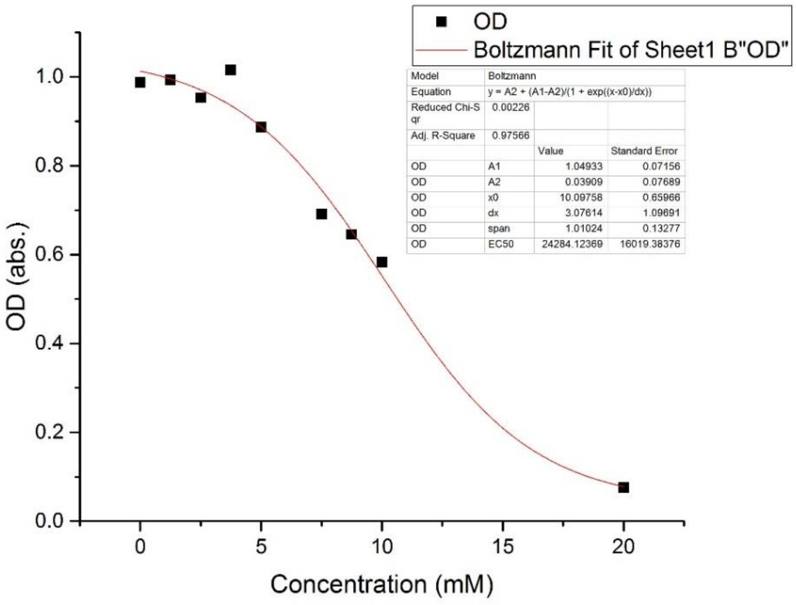

Figure S282 – Boltzmann fit used to calculate MIC_{50} using optical density values at 900 minutes for compound **45** at varying concentrations.

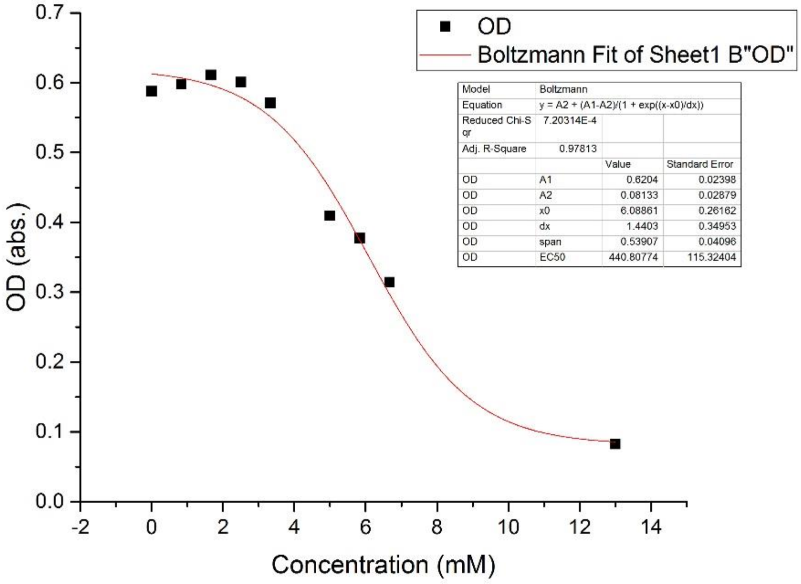

Figure S283 – Boltzmann fit used to calculate MIC_{50} using optical density values at 900 minutes for compound **46** at varying concentrations.

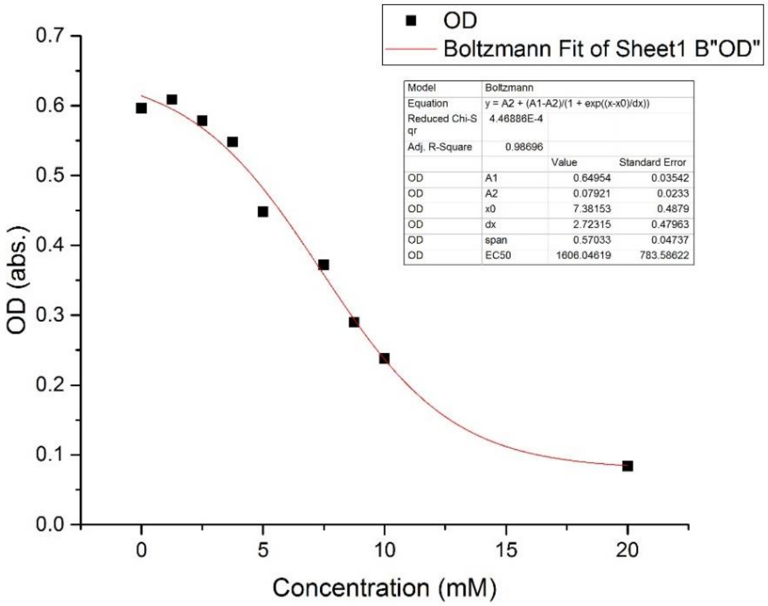

Figure S284 – Boltzmann fit used to calculate MIC_{50} using optical density values at 900 minutes for compound **50** at varying concentrations.

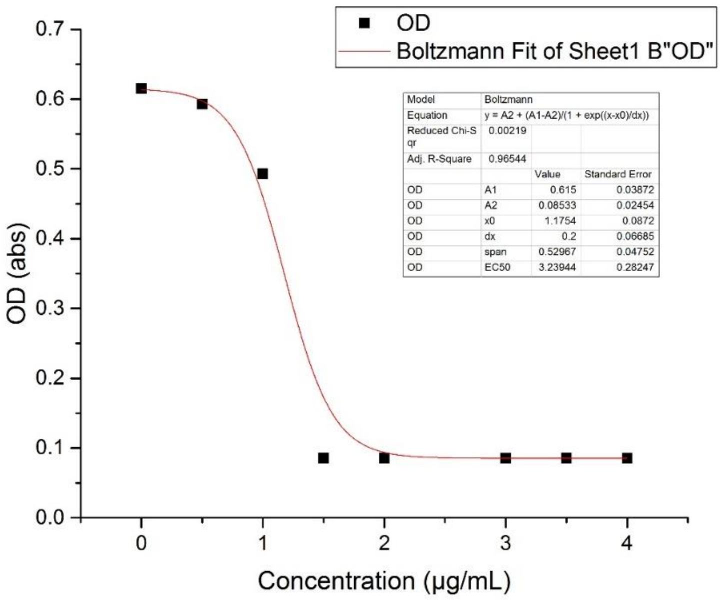

Figure S285 – Boltzmann fit used to calculate MIC_{50} using optical density values at 900 minutes for **ampicillin** at varying concentrations.

Additional physicochemical – antimicrobial activity relationships identified by an exhaustive search of models

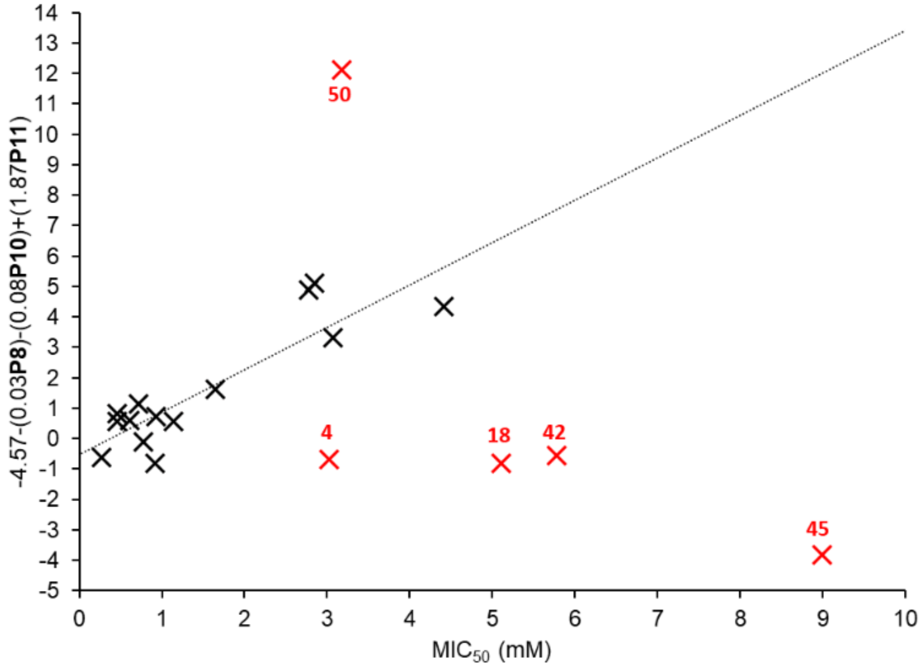

Figure S286 - Correlation identified for the activity of **1**-**50** against MRSA with parameters 1-14 demonstrating the highest R^2 values, generated using Eq. 1. Red values indicate potential outliers where differing properties may affect MIC_{50} values.

Quantitative Microscopy Analysis

MRSA

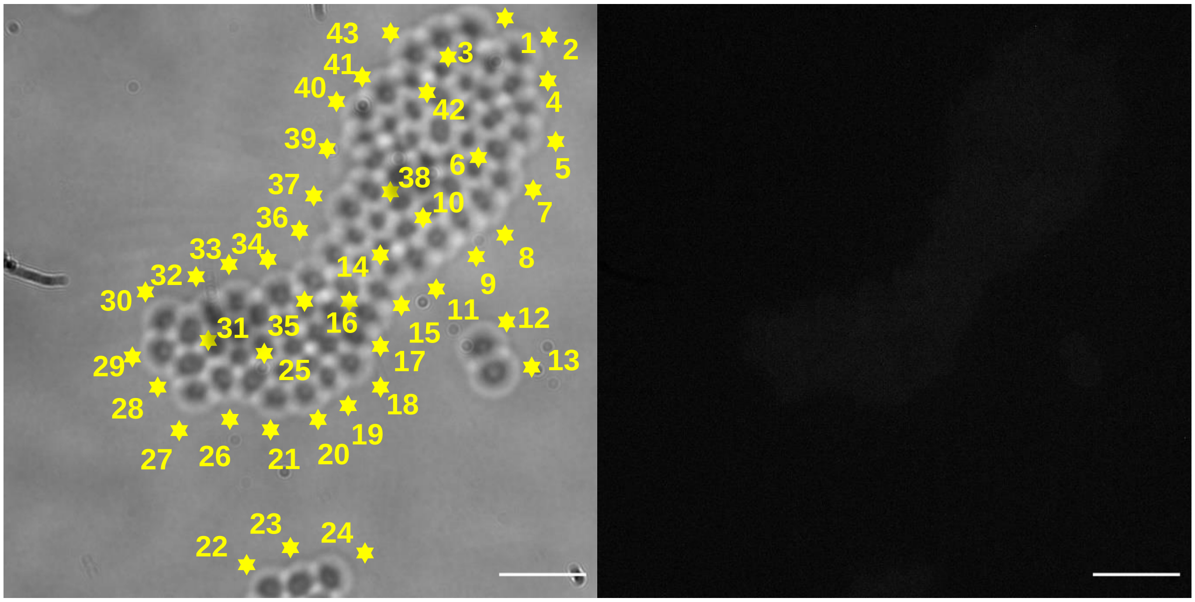

Figure S287 – MRSA transmitted image and fluorescence image at 450nm used in fluorescence intensity calculations in the absence of both compound **39** and FM4-64 at T = 30 minutes. Transmitted image used to locate cells on fluorescence image. Scale bar = 10µM.

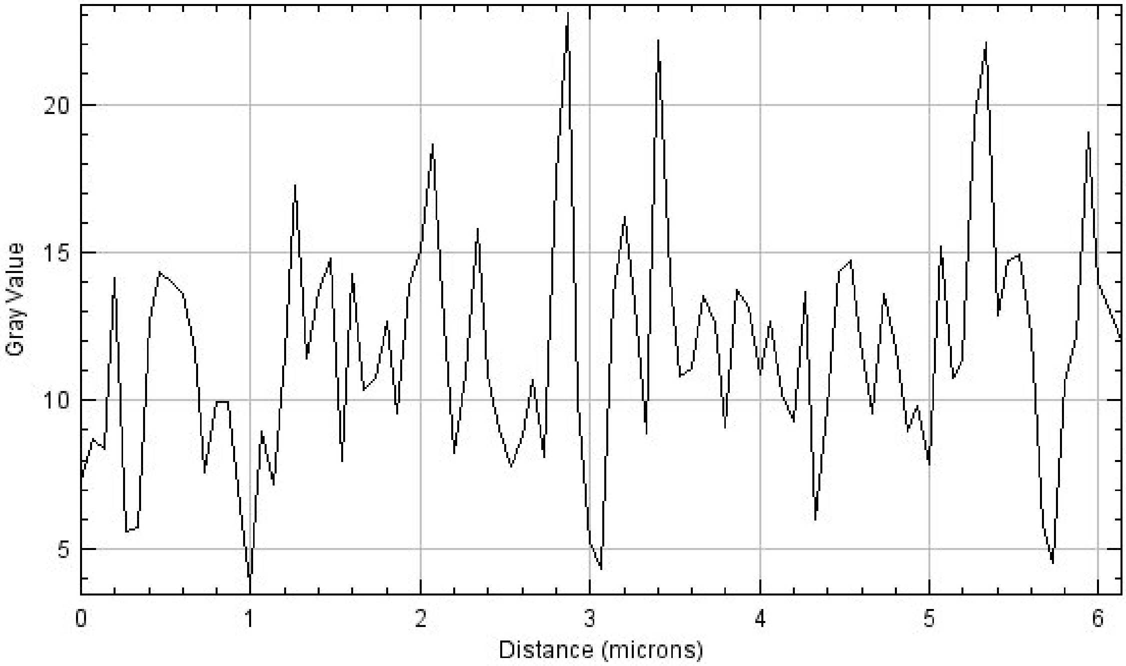

Figure S288 – Intensity profile of line scan generated for cell 1, Figure S287.

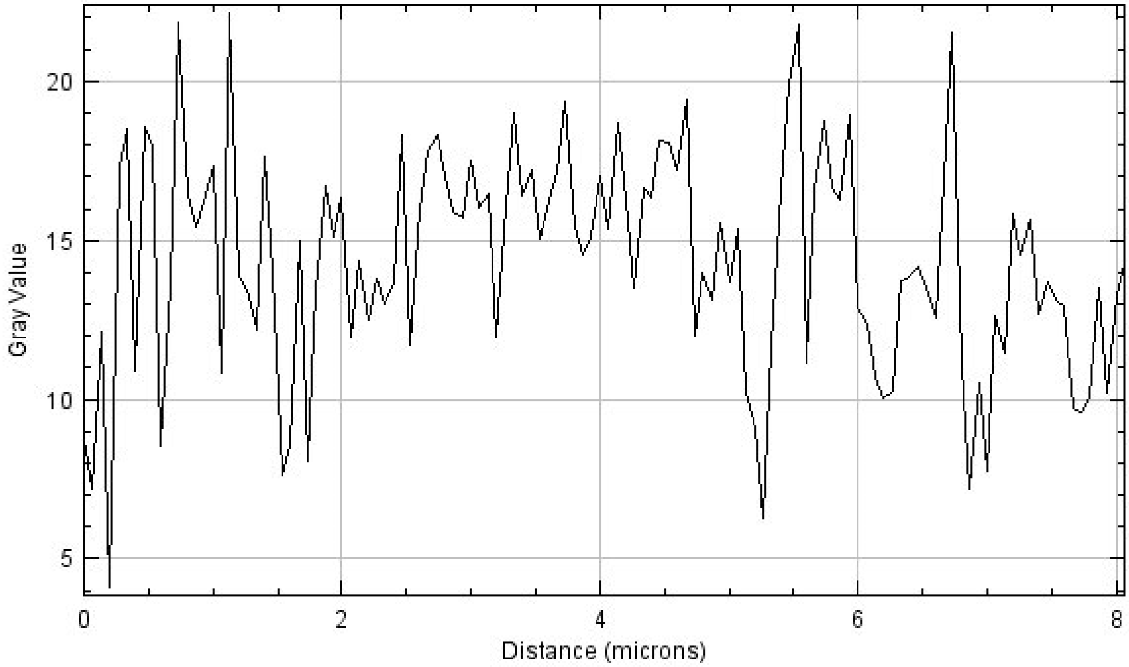

Figure S289 – Intensity profile of line scan generated for cell 2, Figure S287.

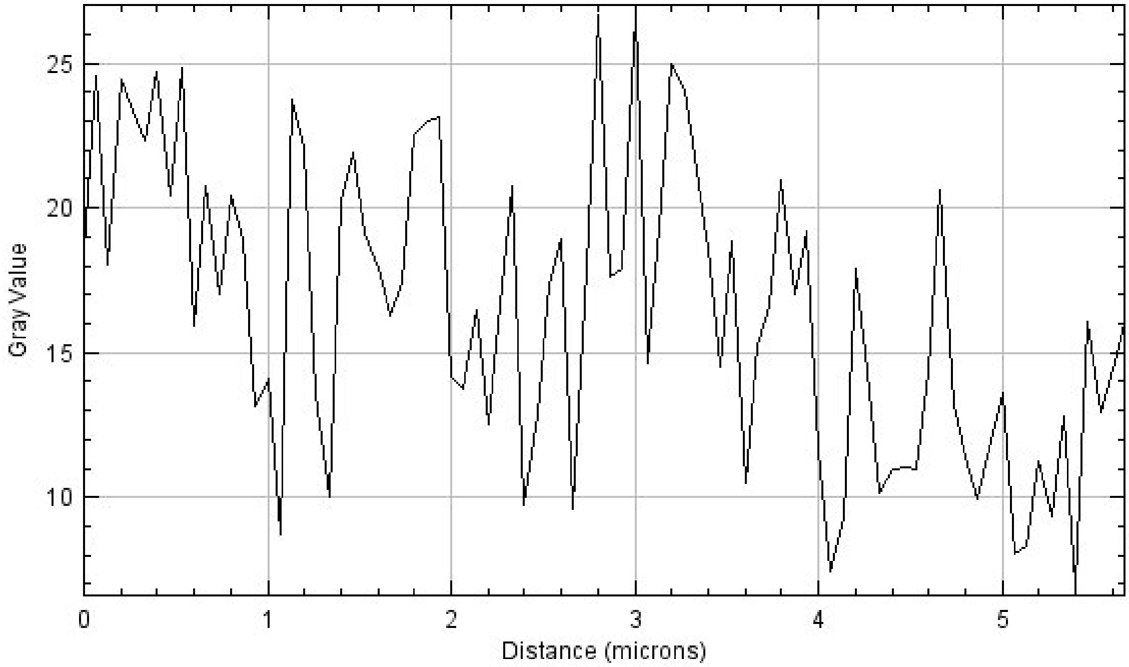

Figure S290 – Intensity profile of line scan generated for cell 3, Figure S287.

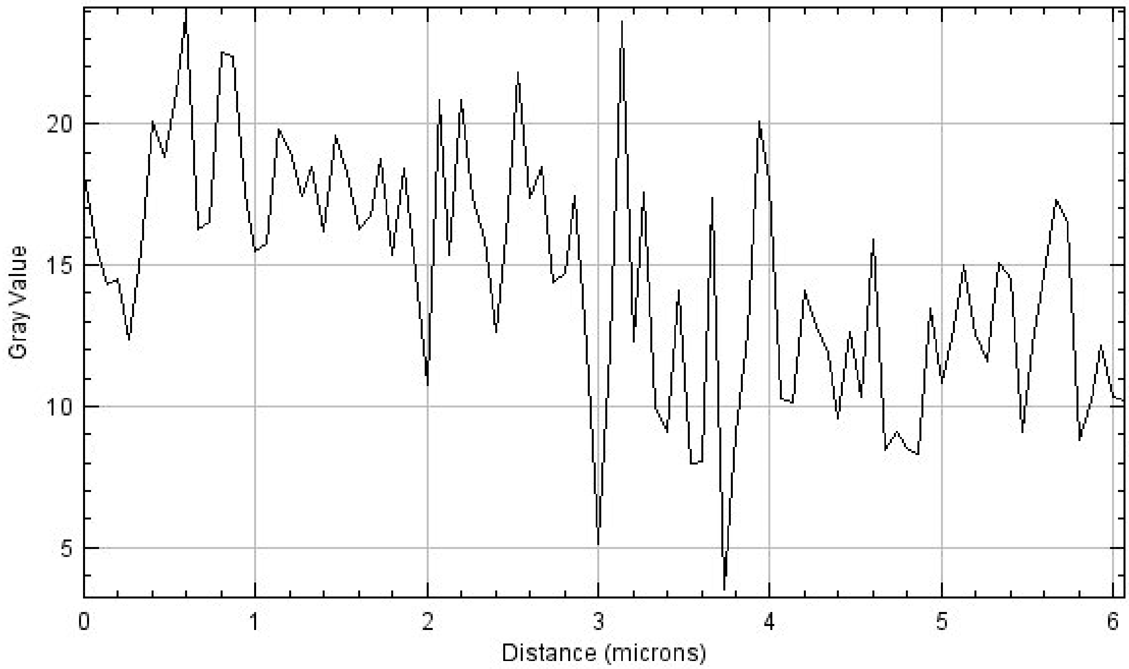

Figure S291 – Intensity profile of line scan generated for cell 4, Figure S287.

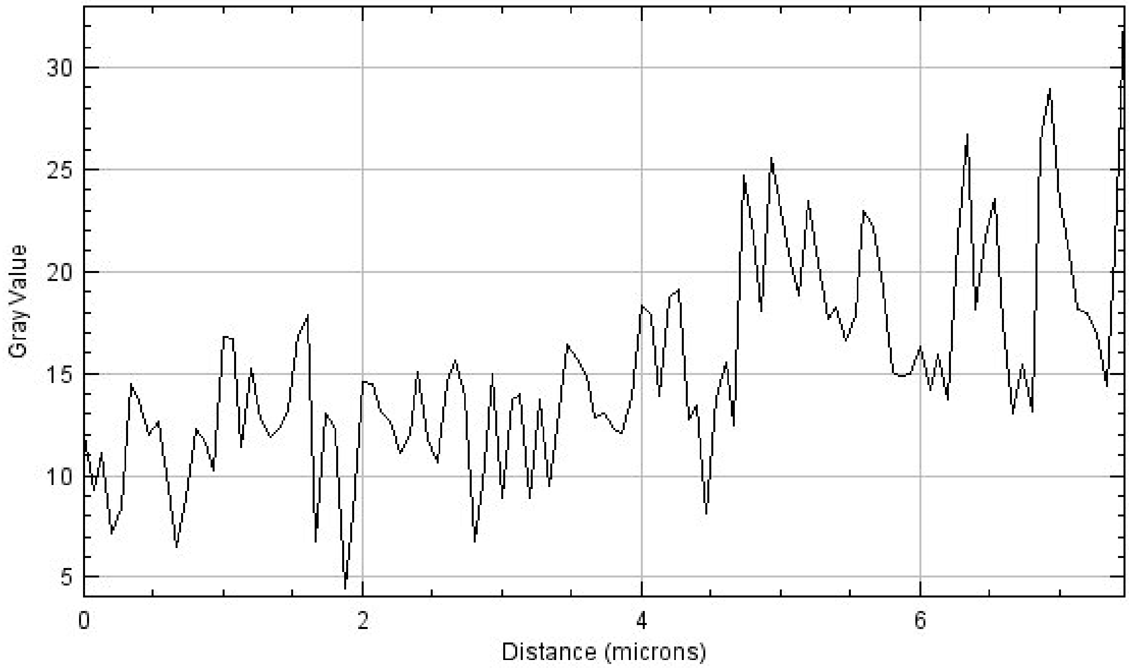

Figure S292 – Intensity profile of line scan generated for cell 5, Figure S287.

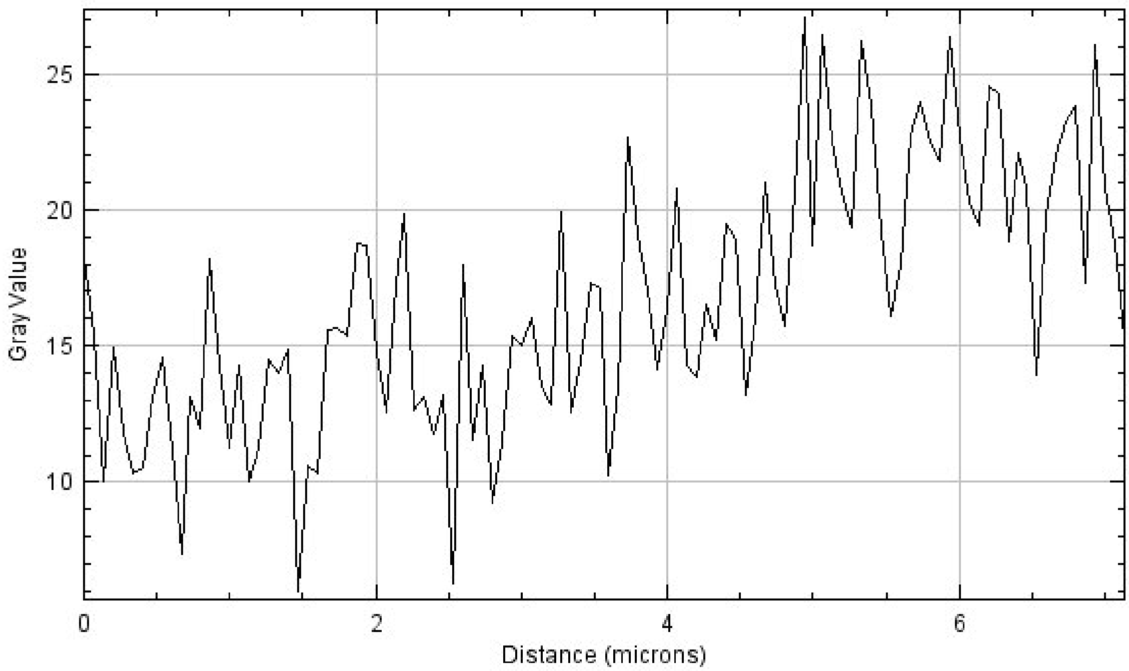

Figure S293 – Intensity profile of line scan generated for cell 6, Figure S287.

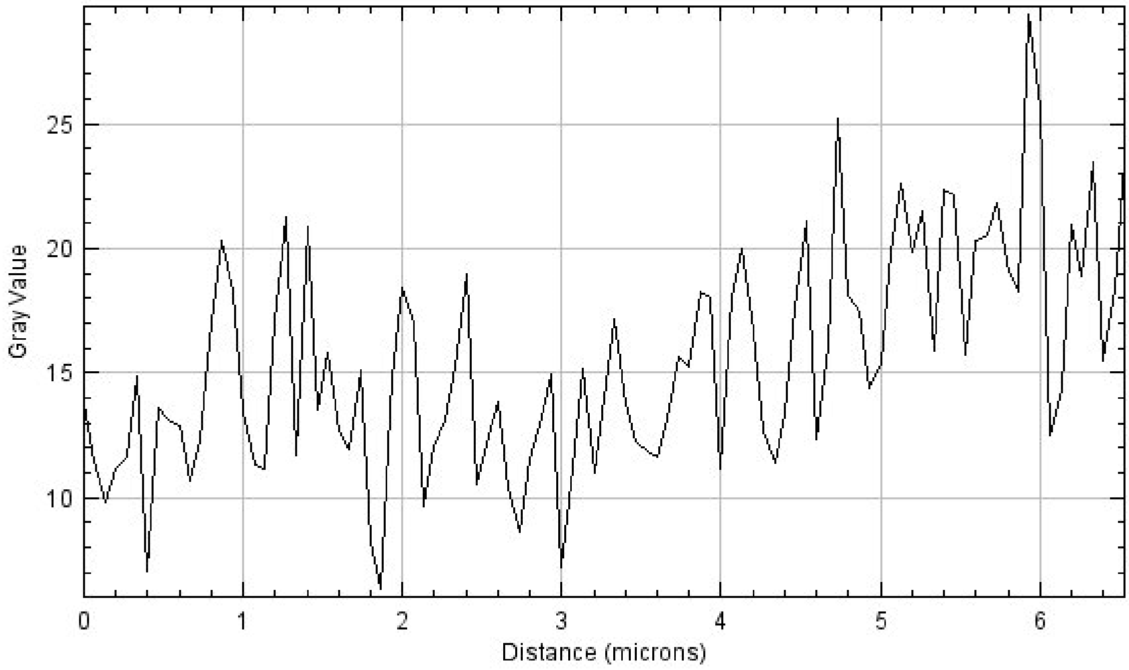

Figure S294 – Intensity profile of line scan generated for cell 7, Figure S287.

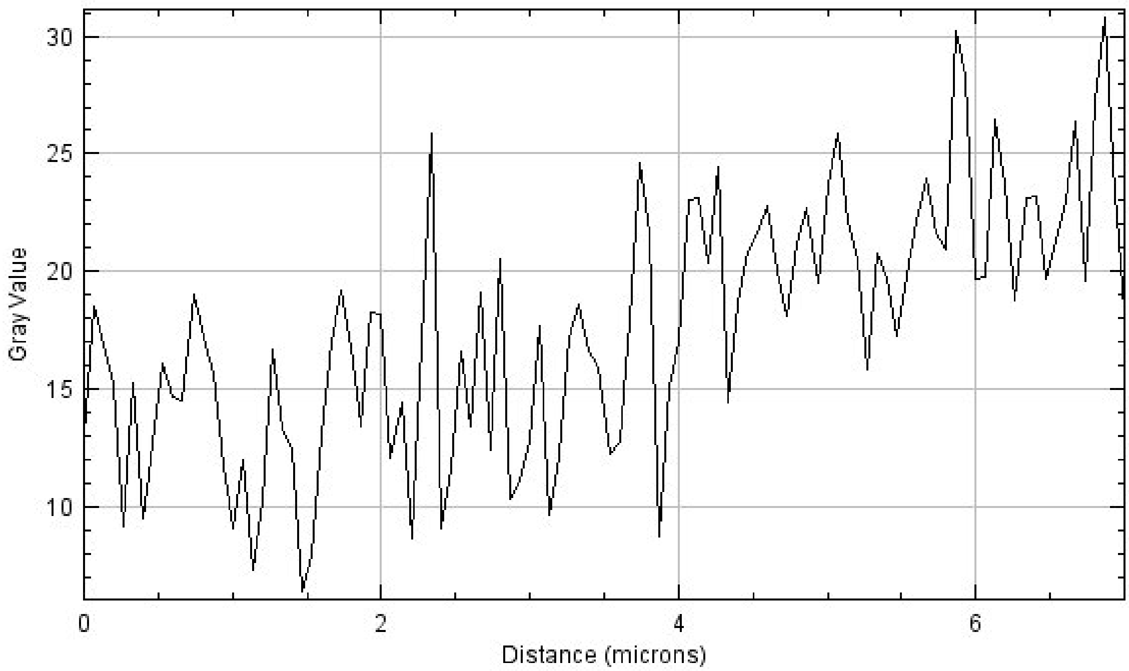

Figure S295 – Intensity profile of line scan generated for cell 8, Figure S287.

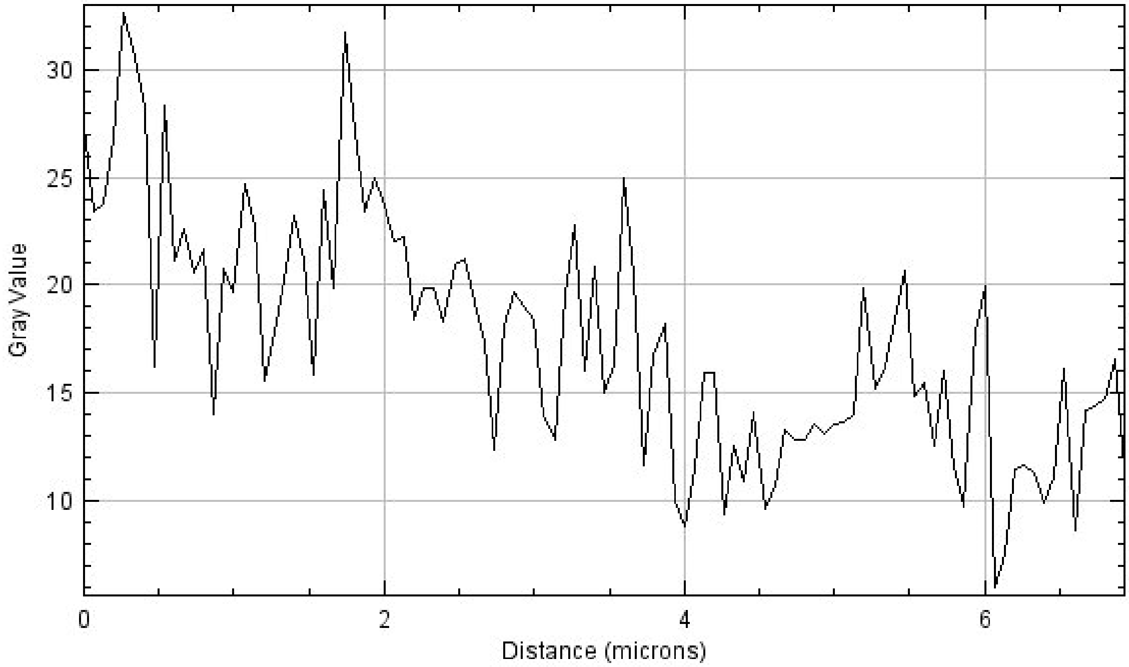

Figure S296 – Intensity profile of line scan generated for cell 9, Figure S287.

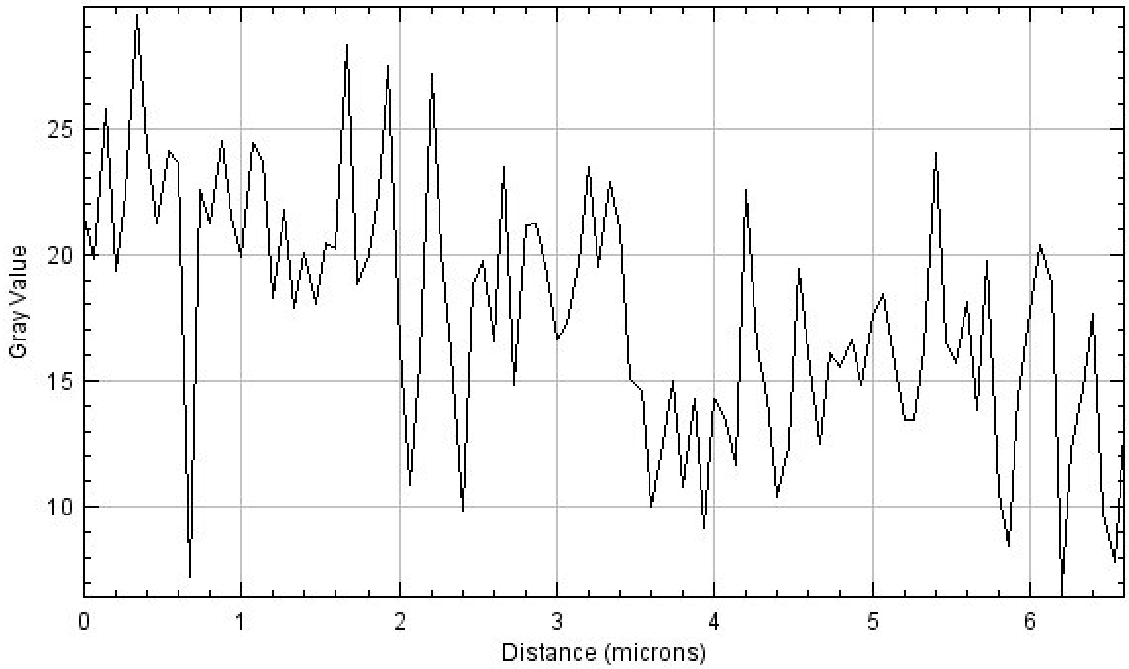

Figure S297 – Intensity profile of line scan generated for cell 10, Figure S287.

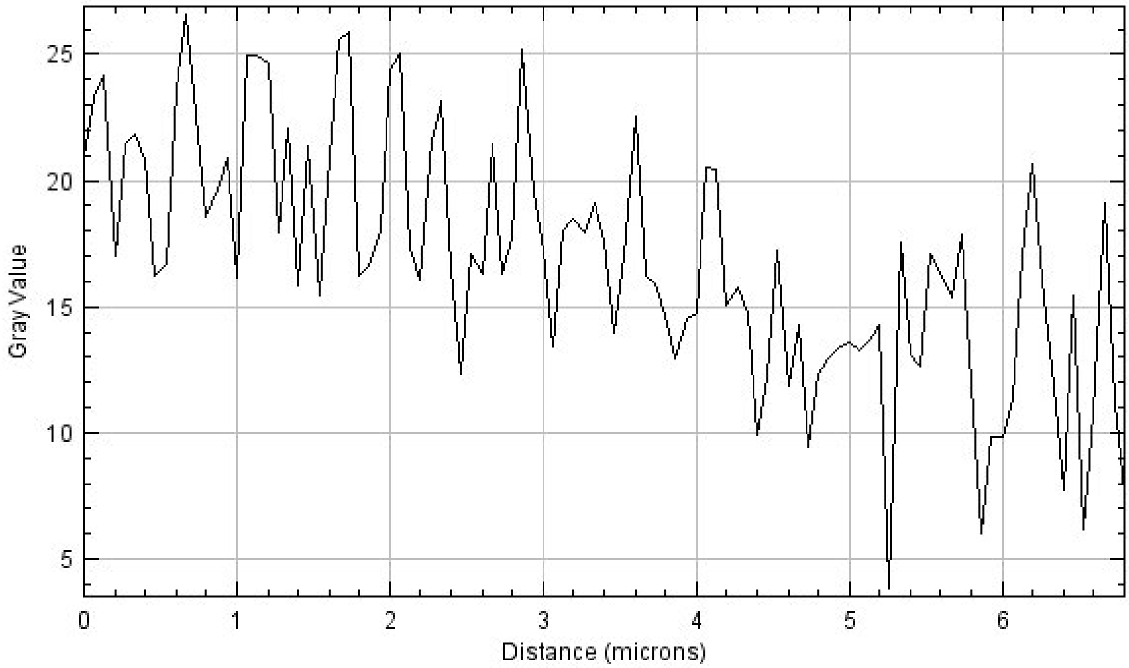

Figure S298 – Intensity profile of line scan generated for cell 11, Figure S287.

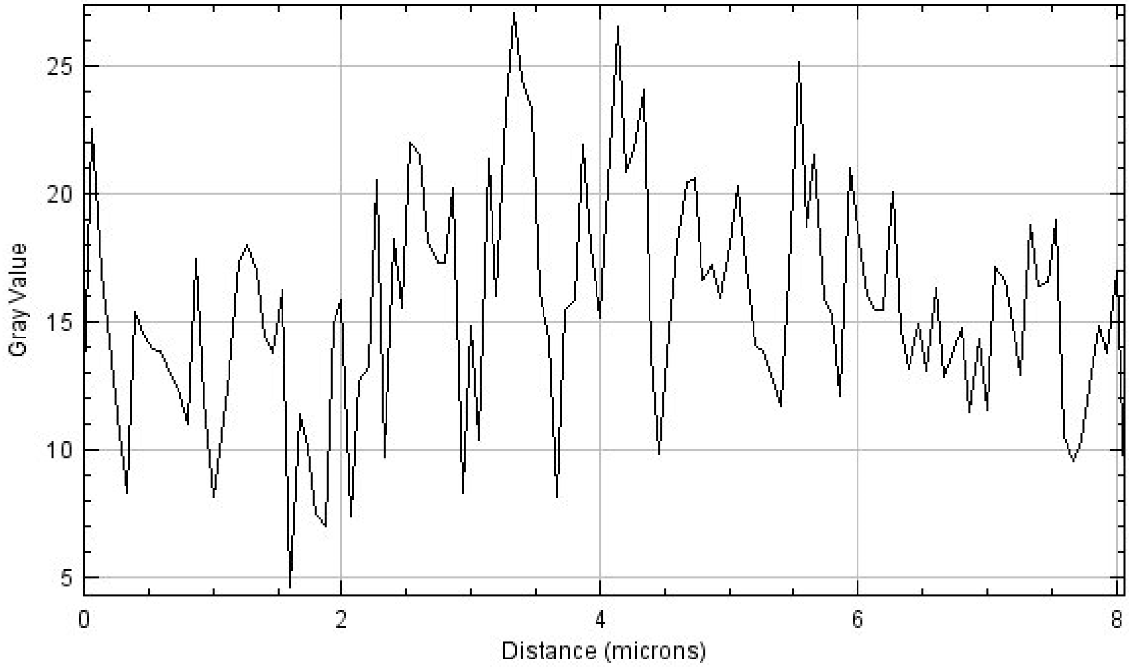

Figure S299 – Intensity profile of line scan generated for cell 12, Figure S287.

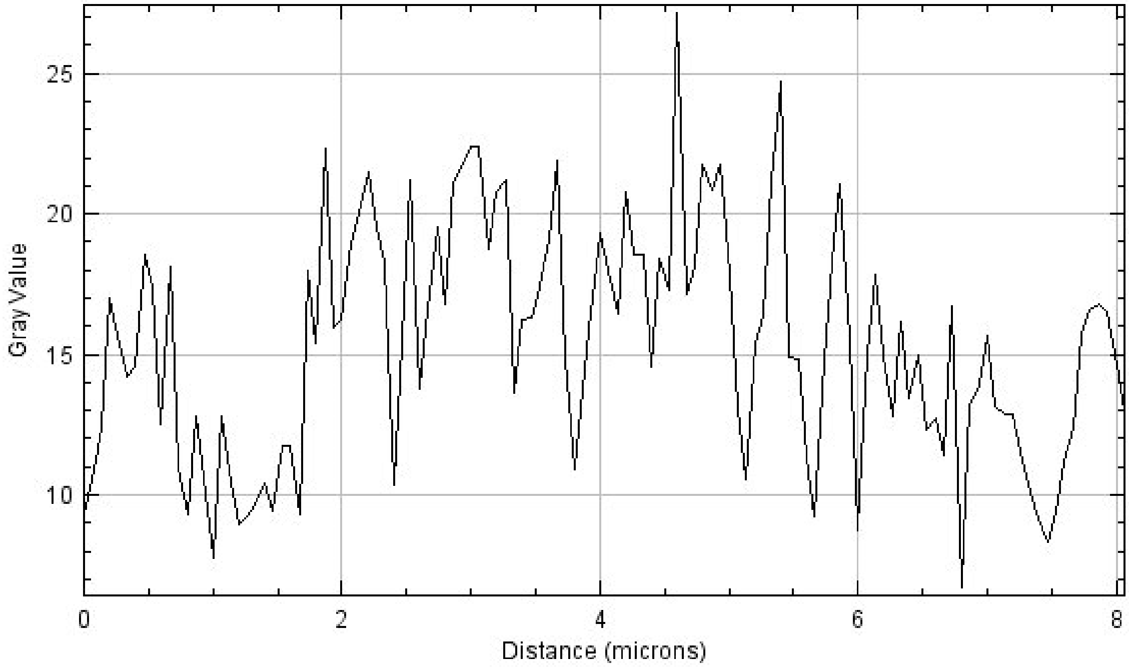

Figure S300 – Intensity profile of line scan generated for cell 13, Figure S287.

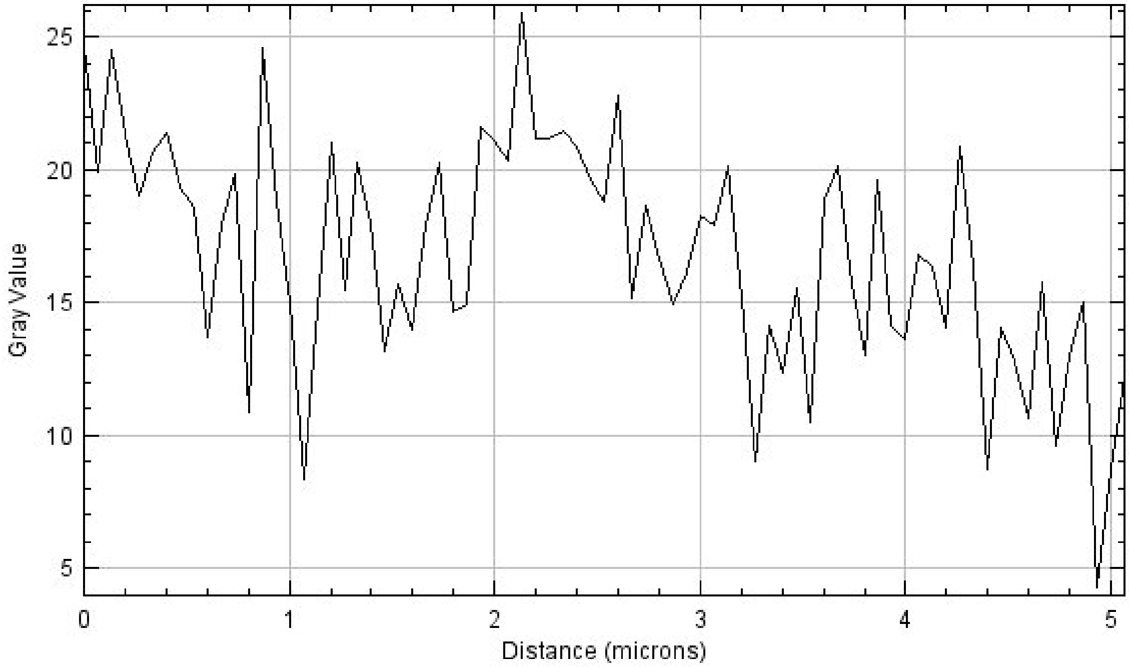

Figure S301 – Intensity profile of line scan generated for cell 14, Figure S287.

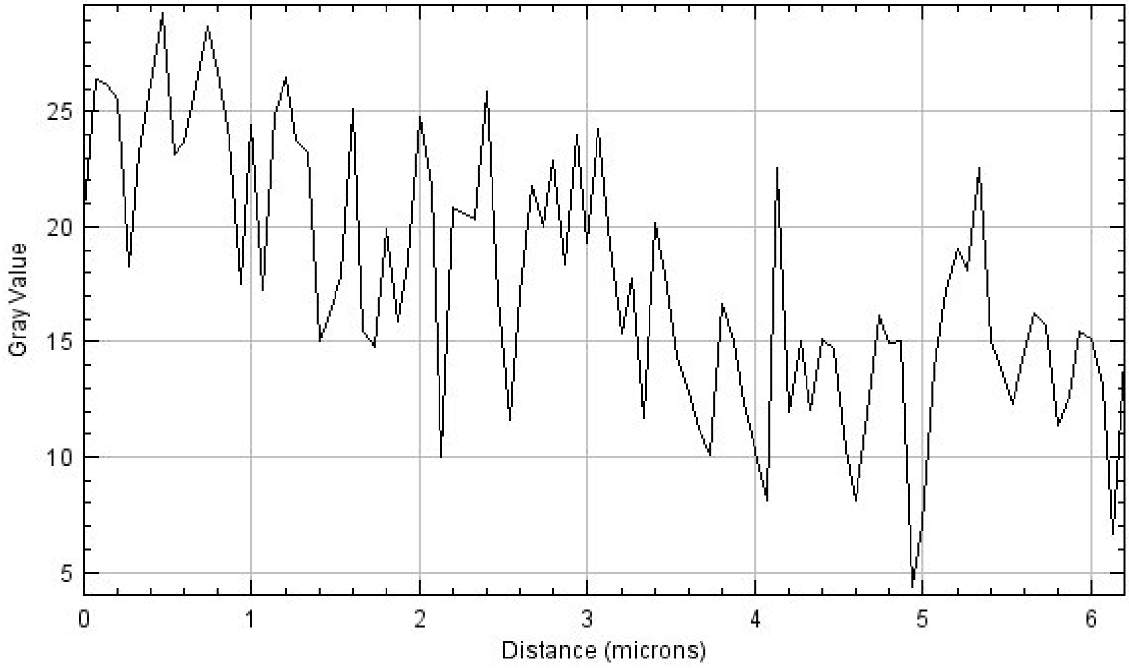

Figure S302 – Intensity profile of line scan generated for cell 15, Figure S287.

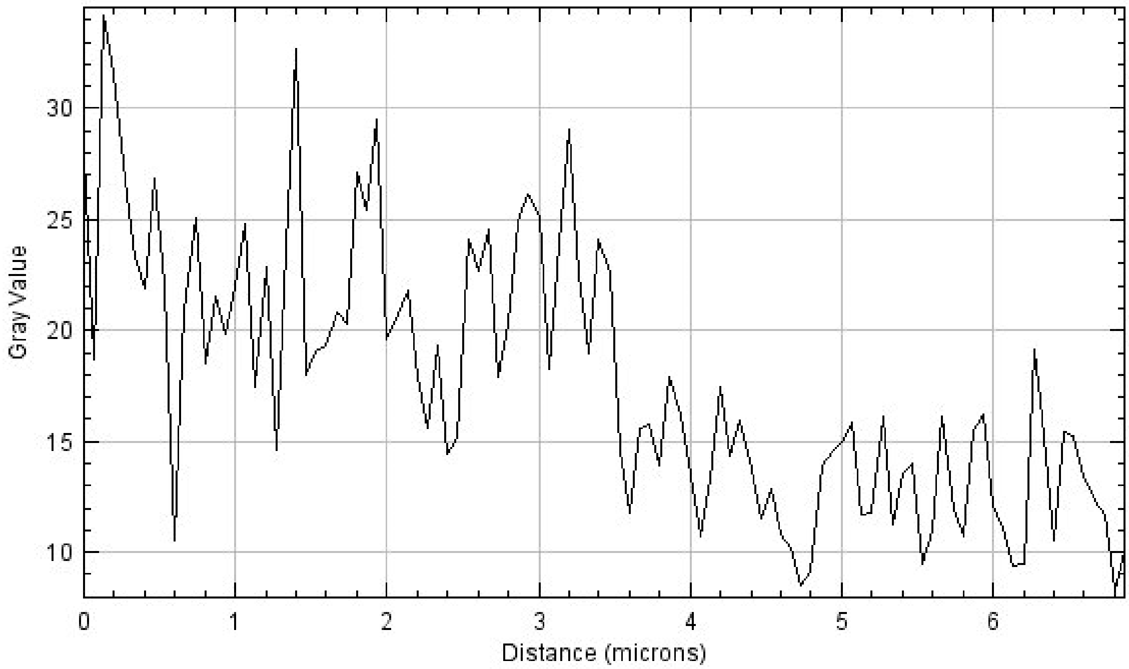

Figure S303 – Intensity profile of line scan generated for cell 16, Figure S287.

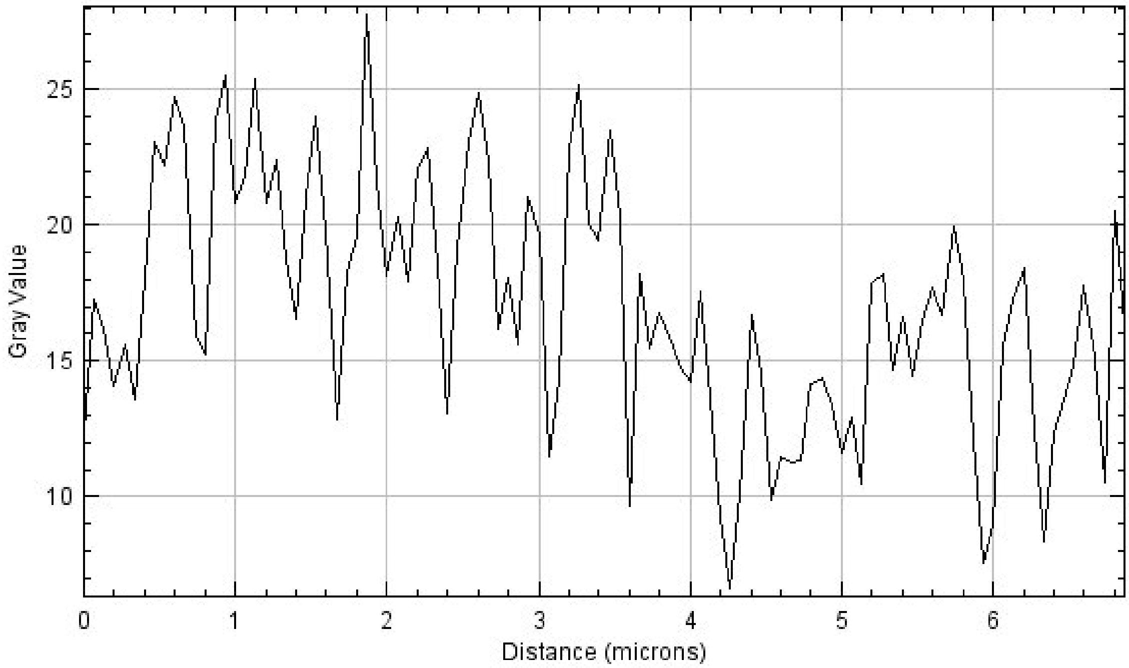

Figure S304 – Intensity profile of line scan generated for cell 17, Figure S287.

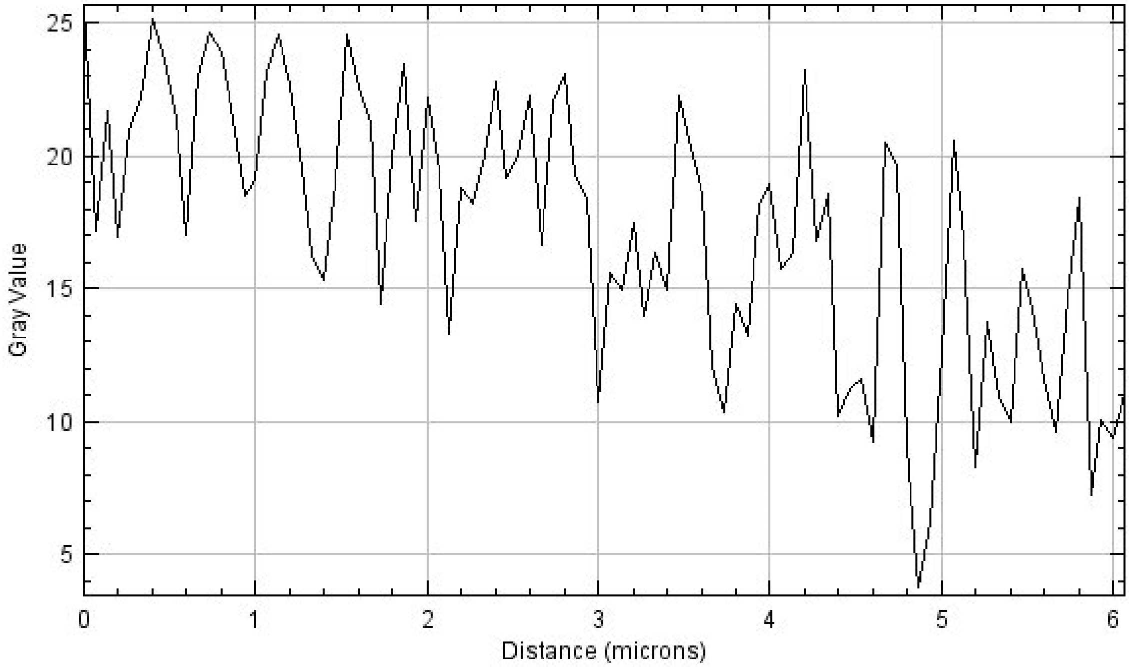

Figure S305 – Intensity profile of line scan generated for cell 18, Figure S287.

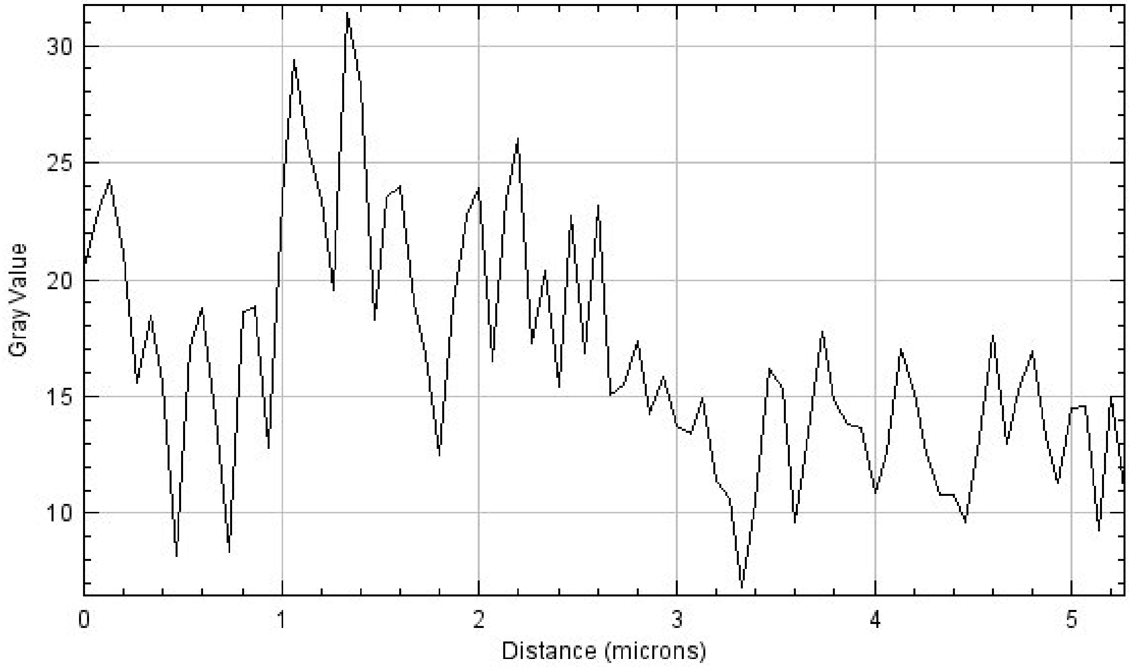

Figure S306 – Intensity profile of line scan generated for cell 19, Figure S287.

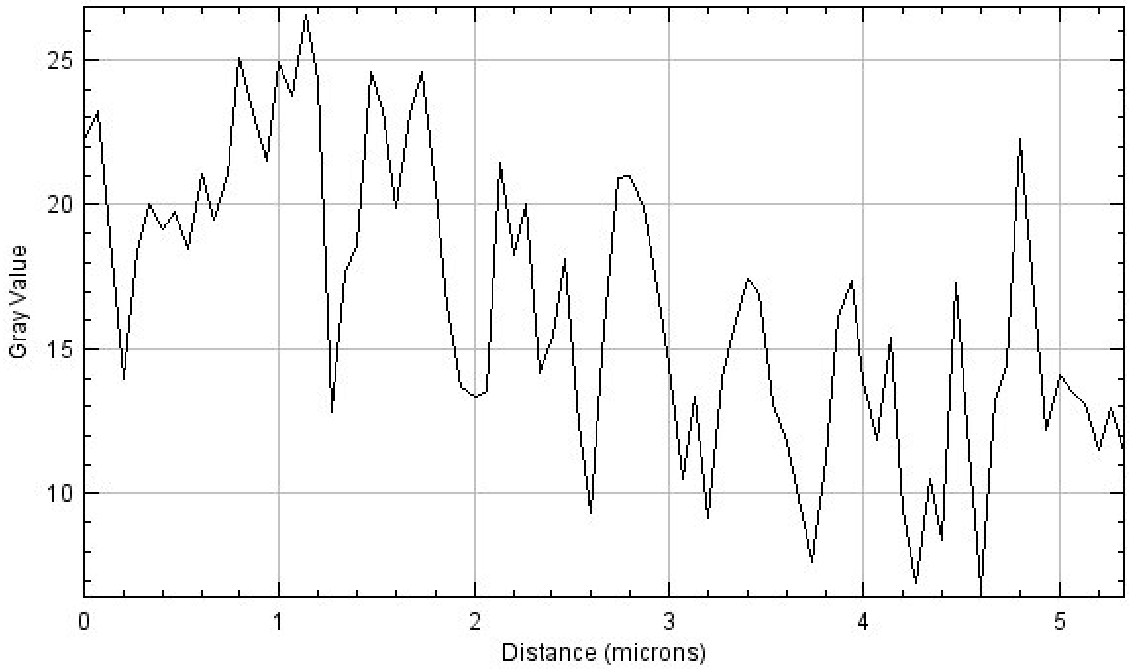

Figure S307 – Intensity profile of line scan generated for cell 20, Figure S287.

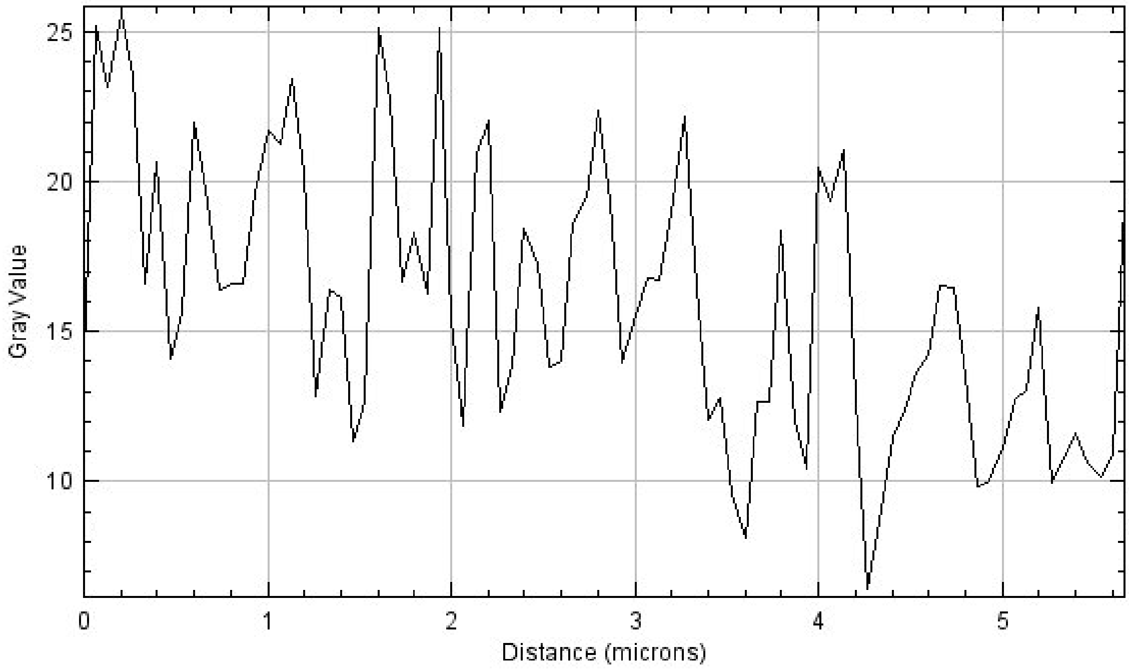

Figure S308 – Intensity profile of line scan generated for cell 21, Figure S287.

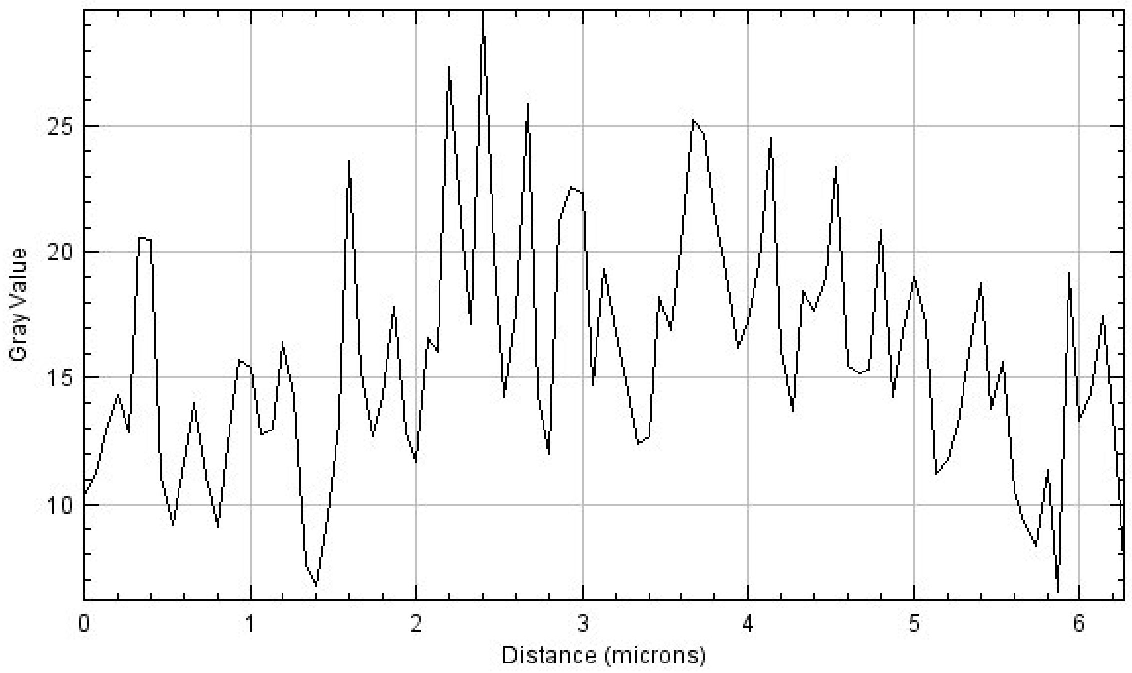

Figure S309 – Intensity profile of line scan generated for cell 22, Figure S287.

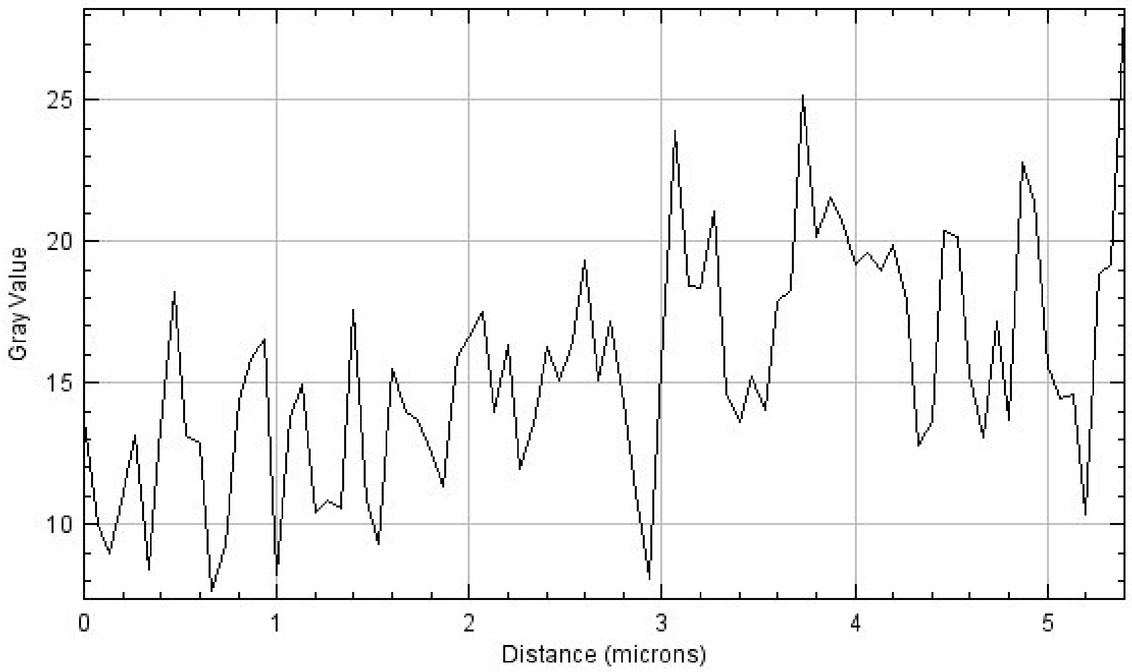

Figure S310 – Intensity profile of line scan generated for cell 23, Figure S287.

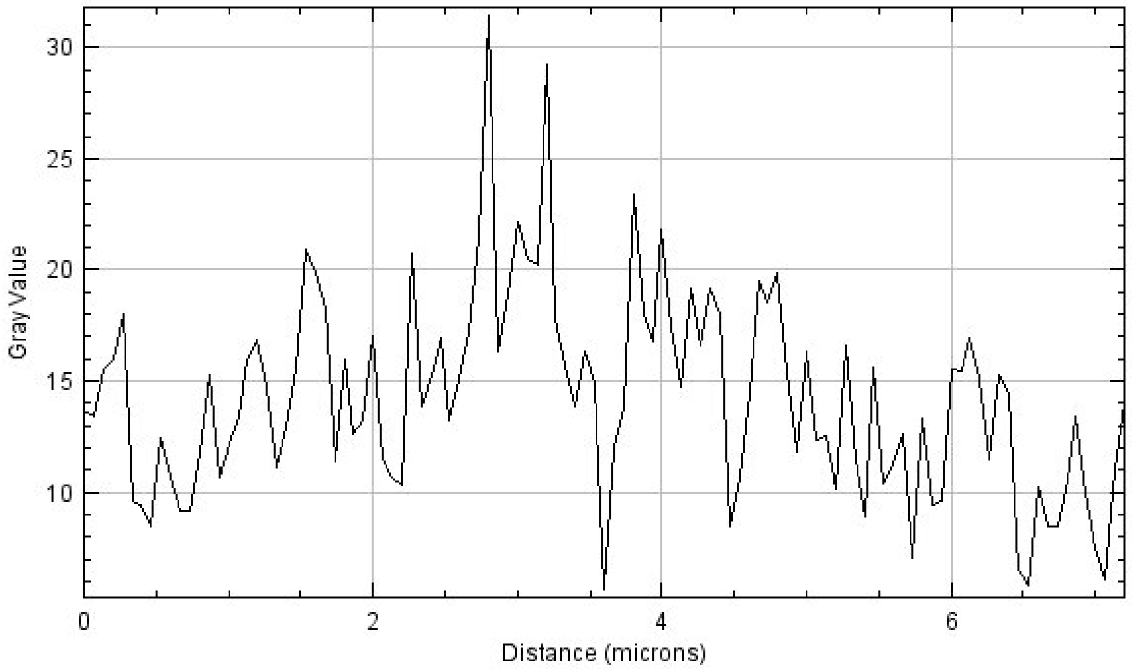

Figure S311 – Intensity profile of line scan generated for cell 24, Figure S287.

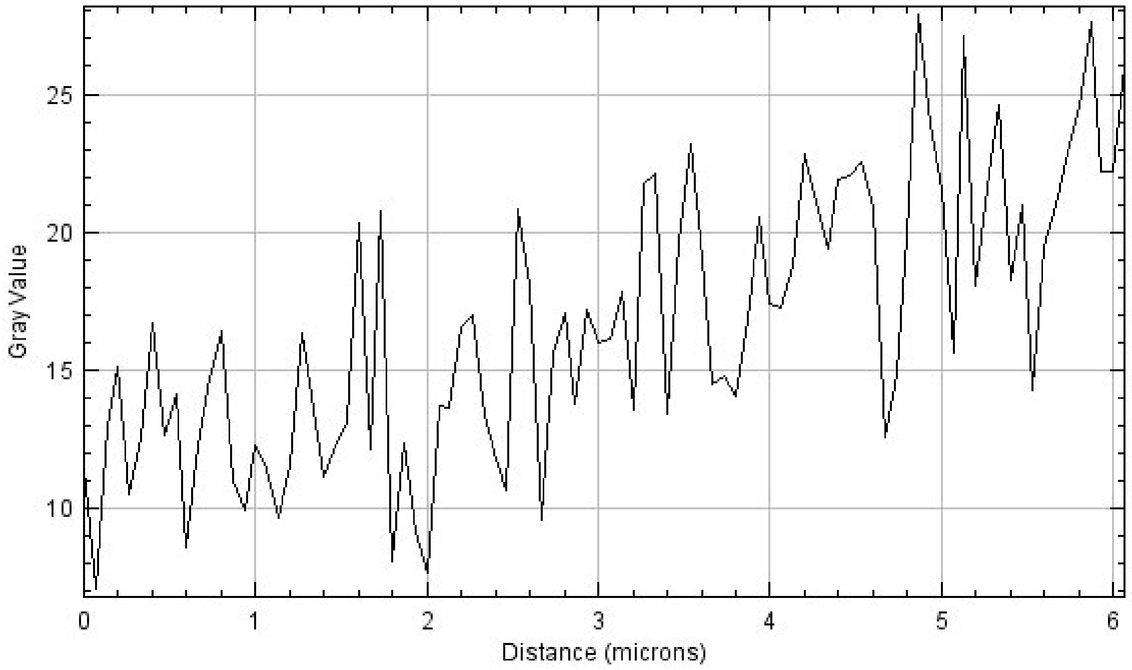

Figure S312 – Intensity profile of line scan generated for cell 25, Figure S287.

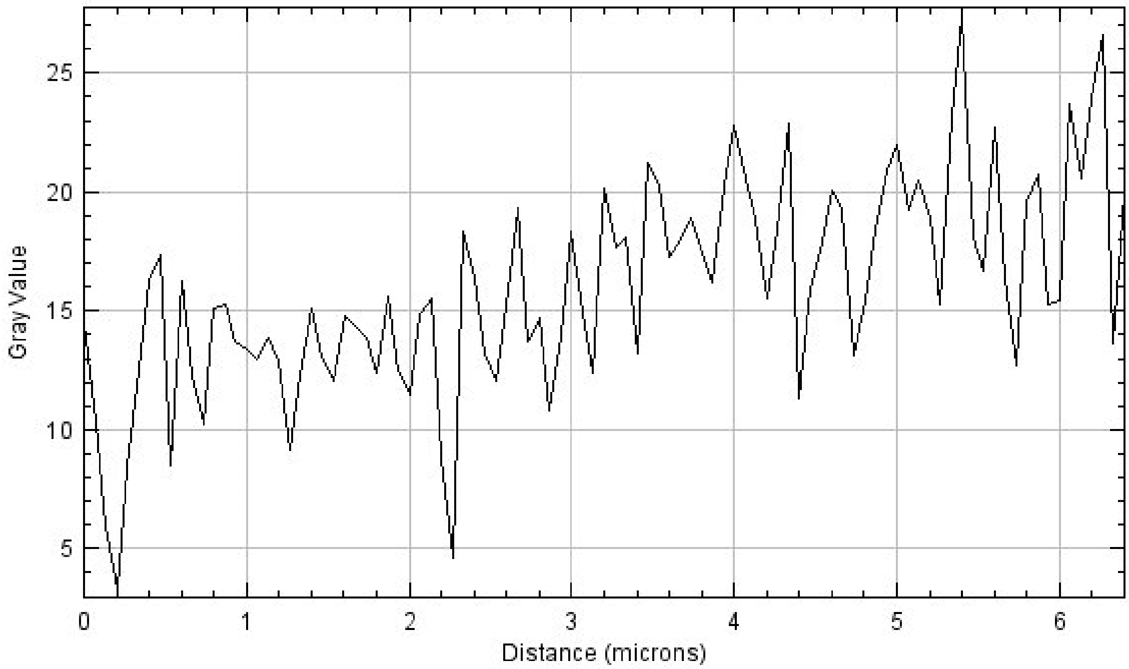

Figure S313 – Intensity profile of line scan generated for cell 26, Figure S287.

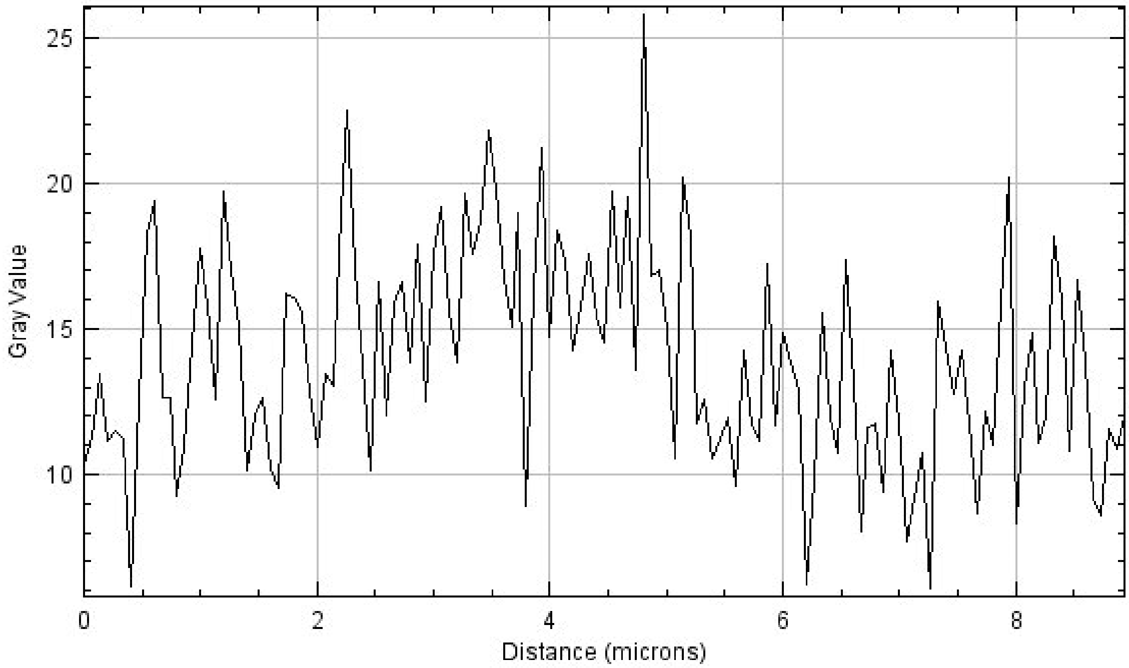

Figure S314 – Intensity profile of line scan generated for cell 27, Figure S287.

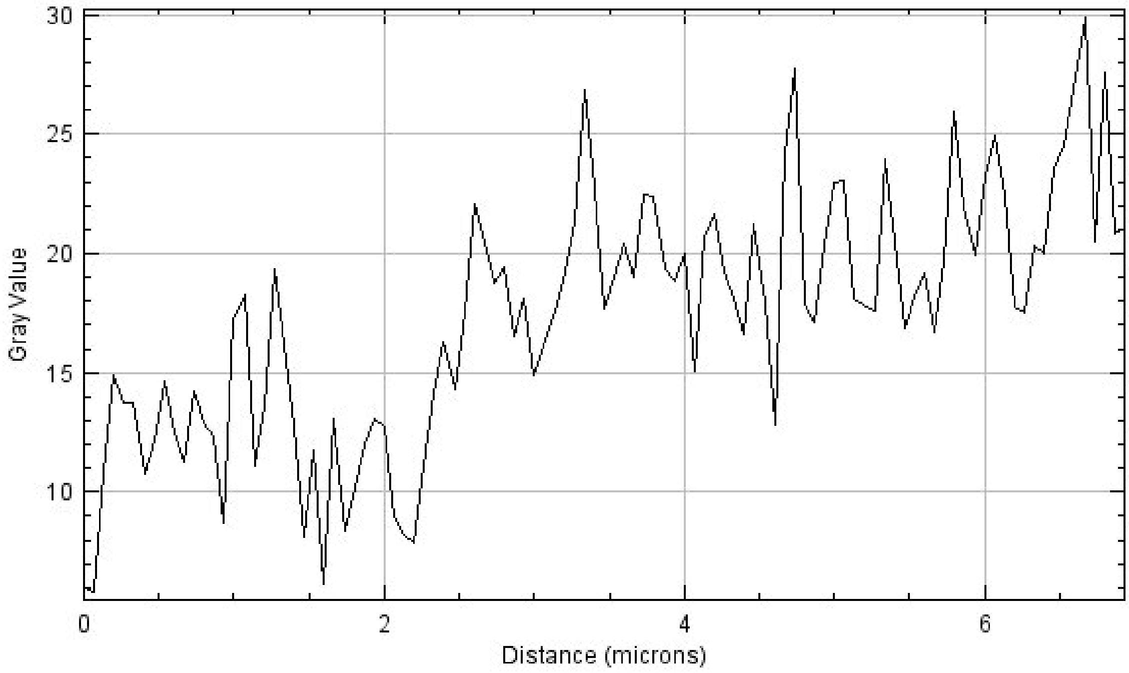

Figure S315 – Intensity profile of line scan generated for cell 28, Figure S287.

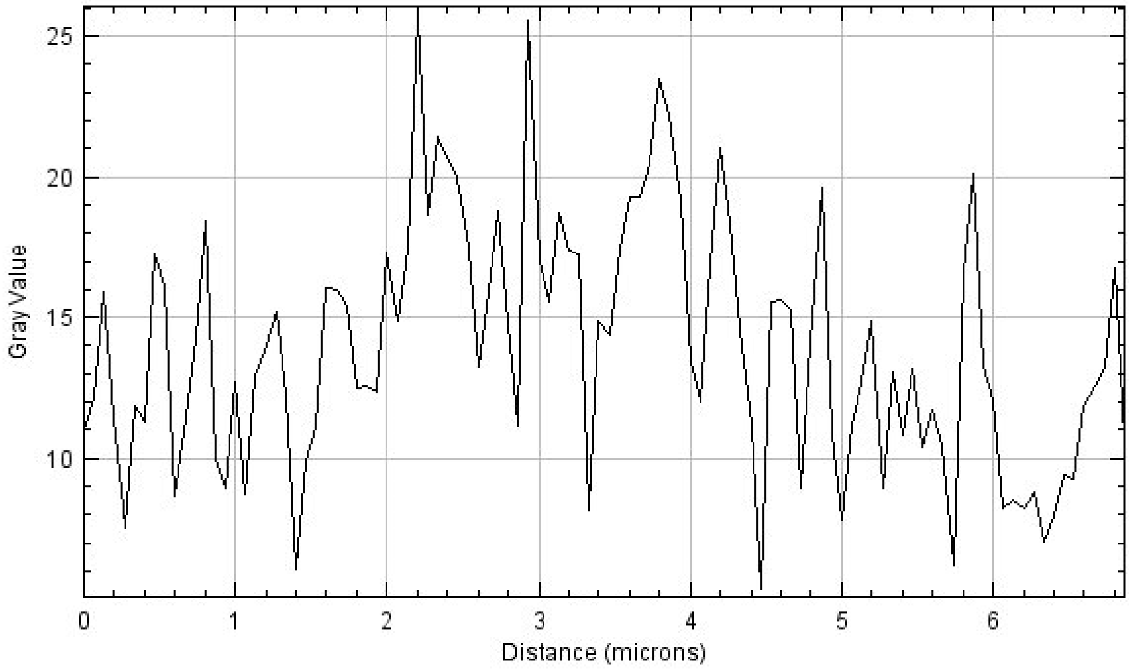

Figure S316 – Intensity profile of line scan generated for cell 29, Figure S287.

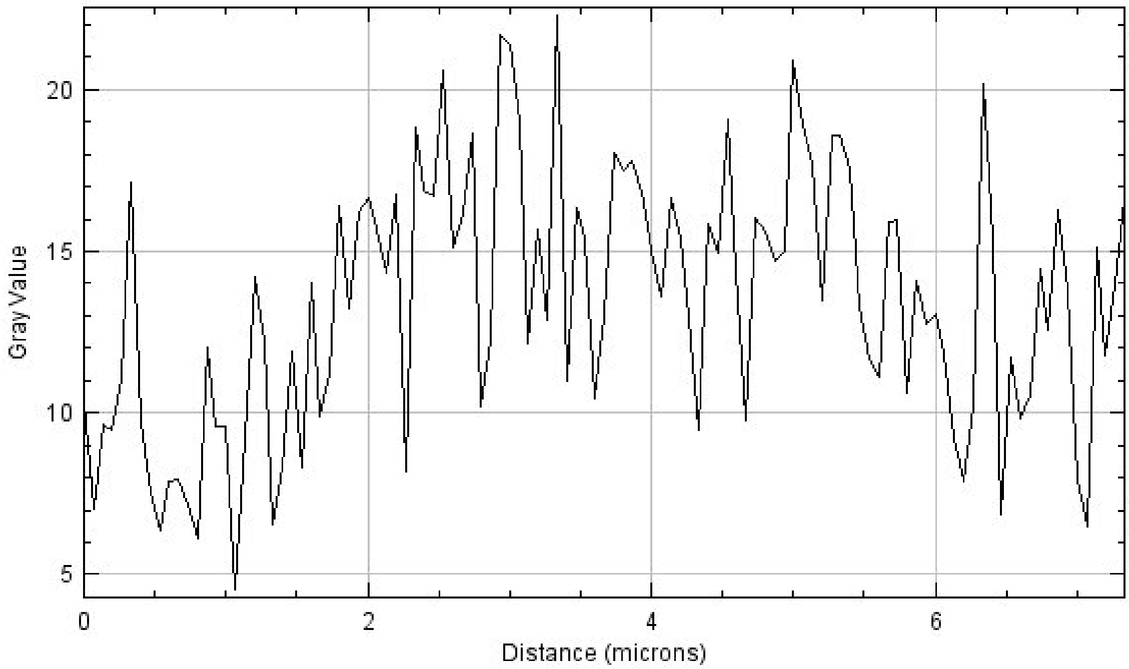

Figure S317 – Intensity profile of line scan generated for cell 30, Figure S287.

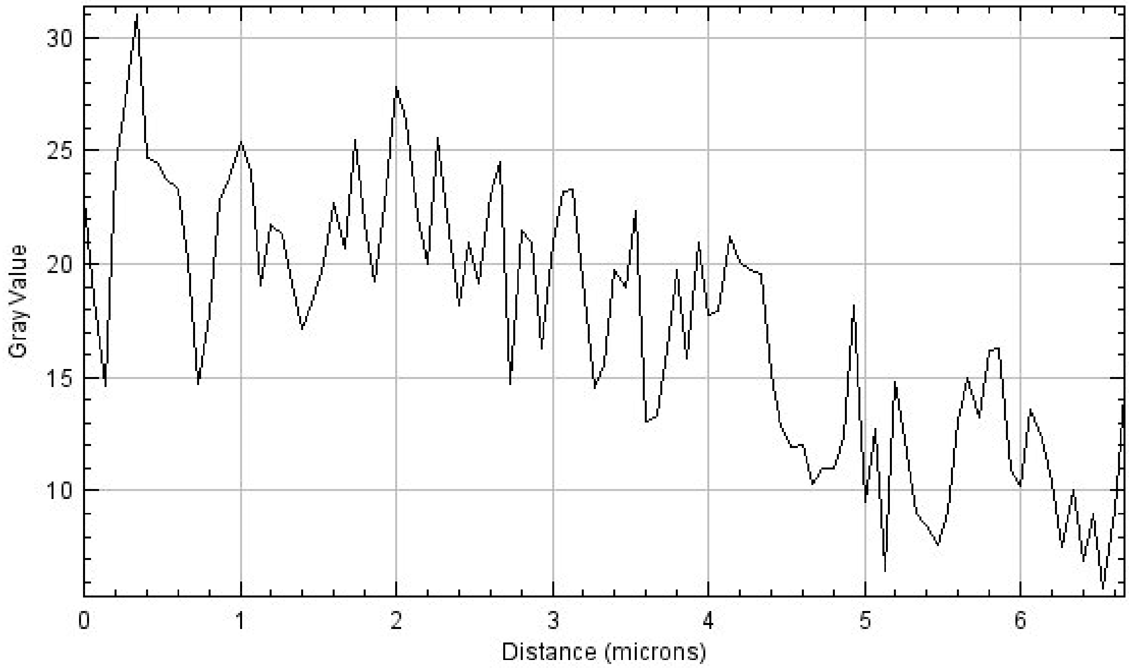

Figure S318 – Intensity profile of line scan generated for cell 31, Figure S287.

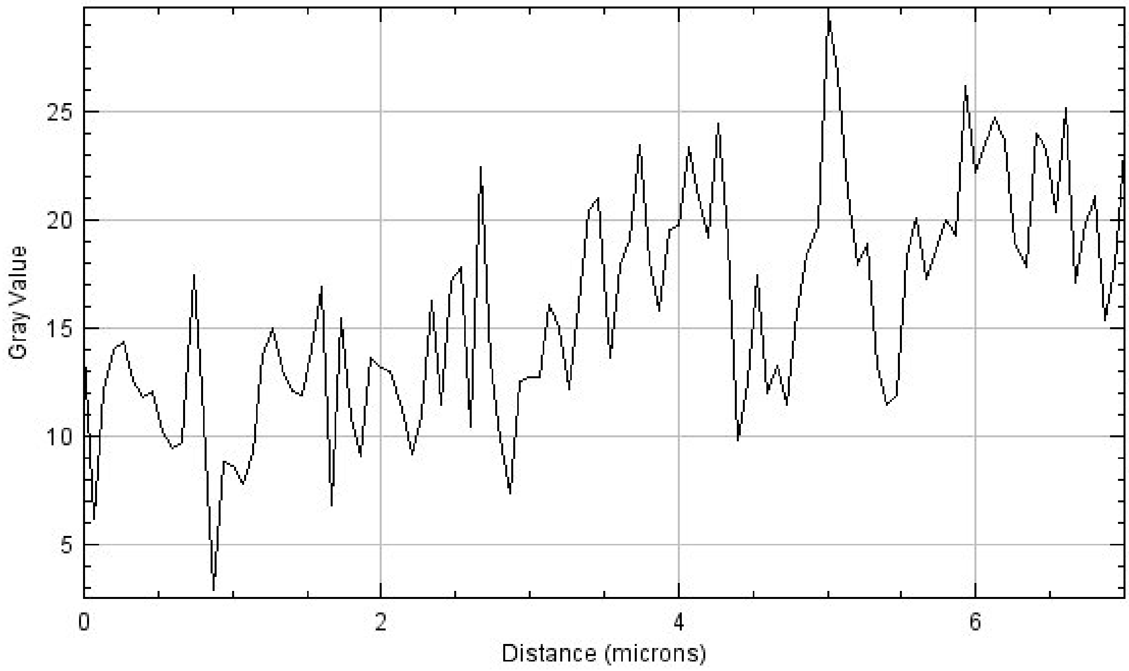

Figure S319 – Intensity profile of line scan generated for cell 32, Figure S287.

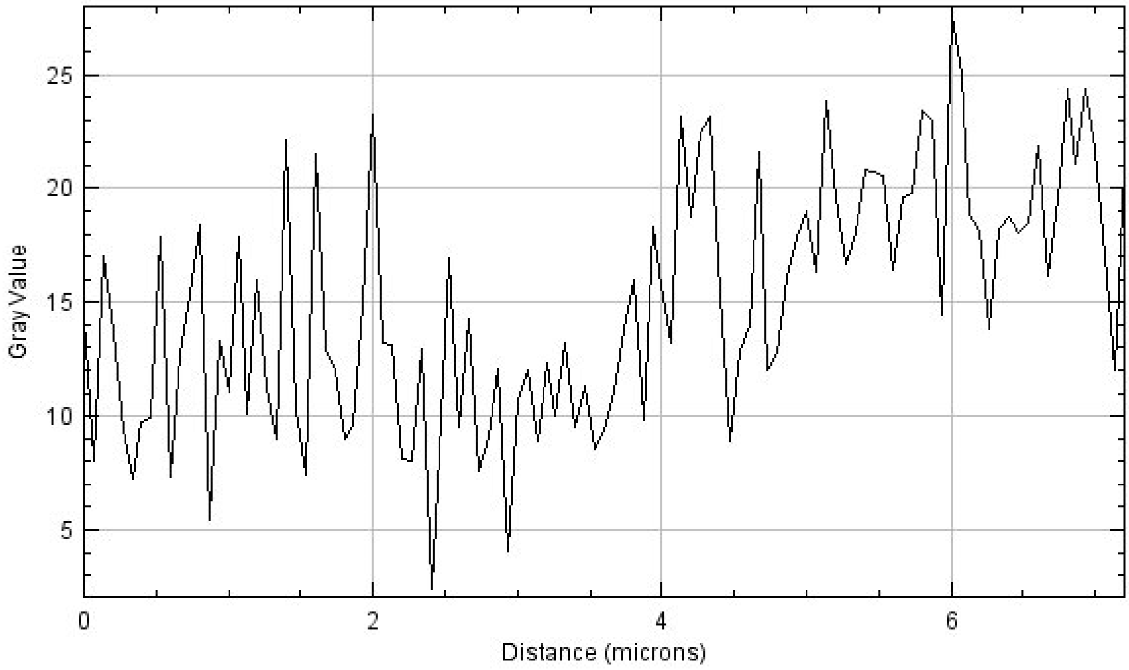

Figure S320 – Intensity profile of line scan generated for cell 33, Figure S287.

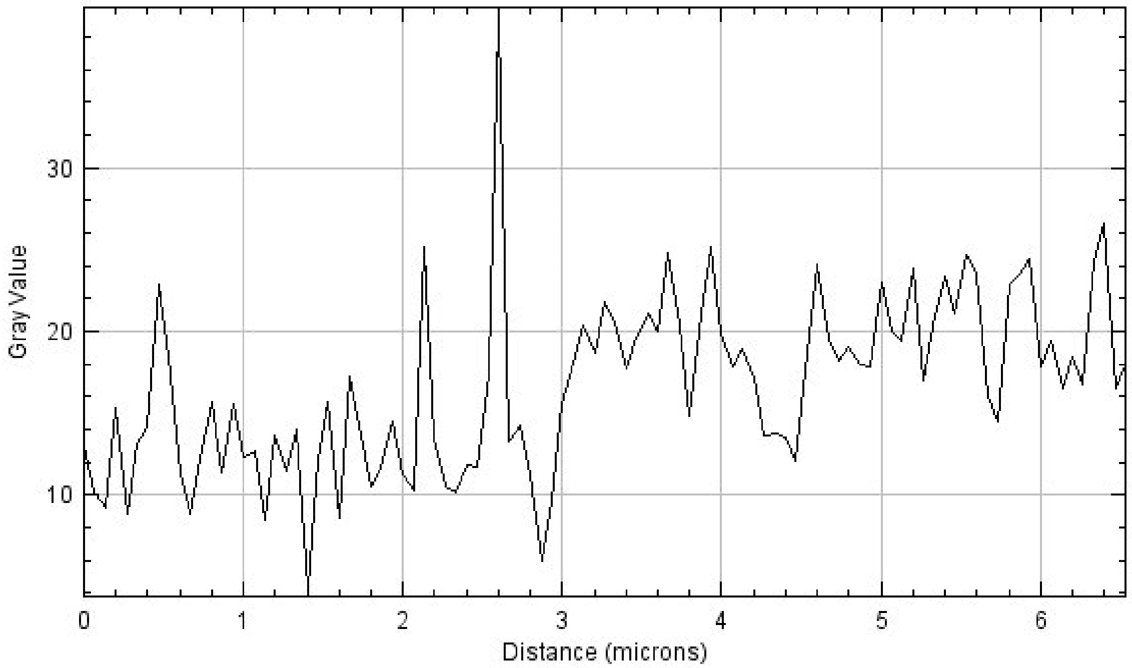

Figure S321 – Intensity profile of line scan generated for cell 34, Figure S287.

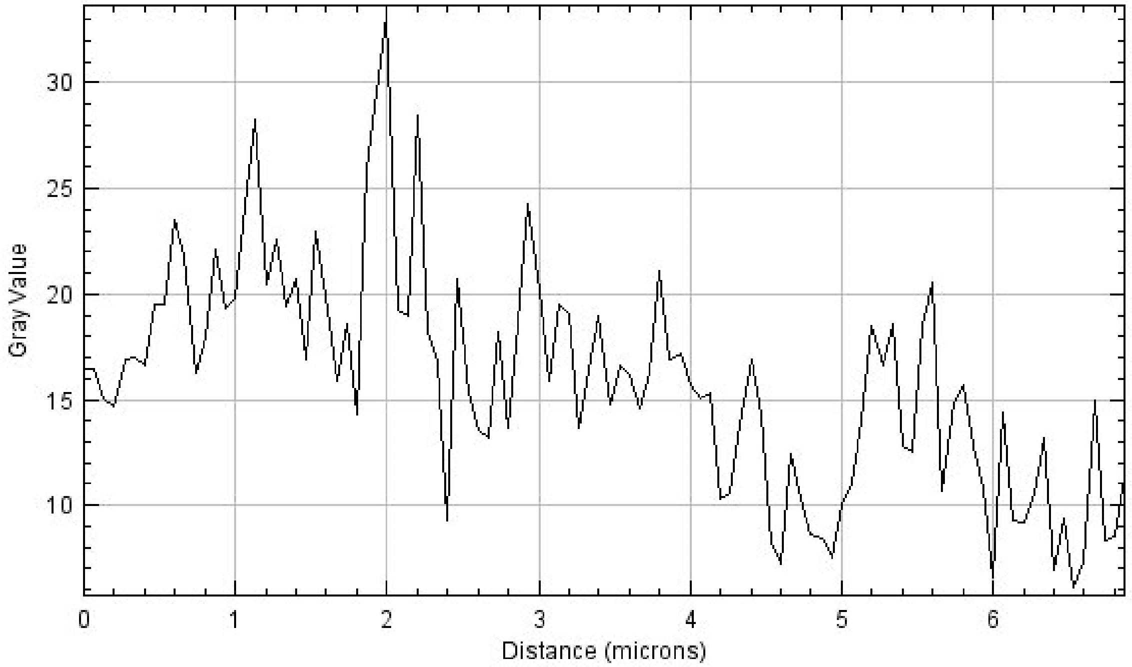

Figure S322 – Intensity profile of line scan generated for cell 35, Figure S287.

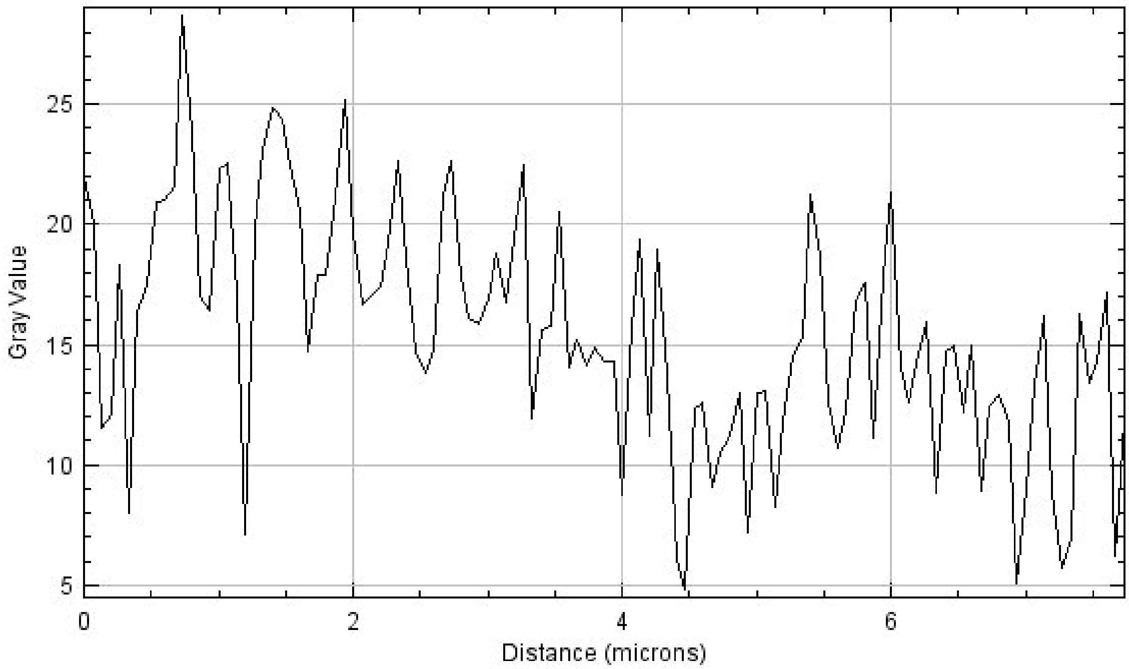

Figure S323 – Intensity profile of line scan generated for cell 36, Figure S287.

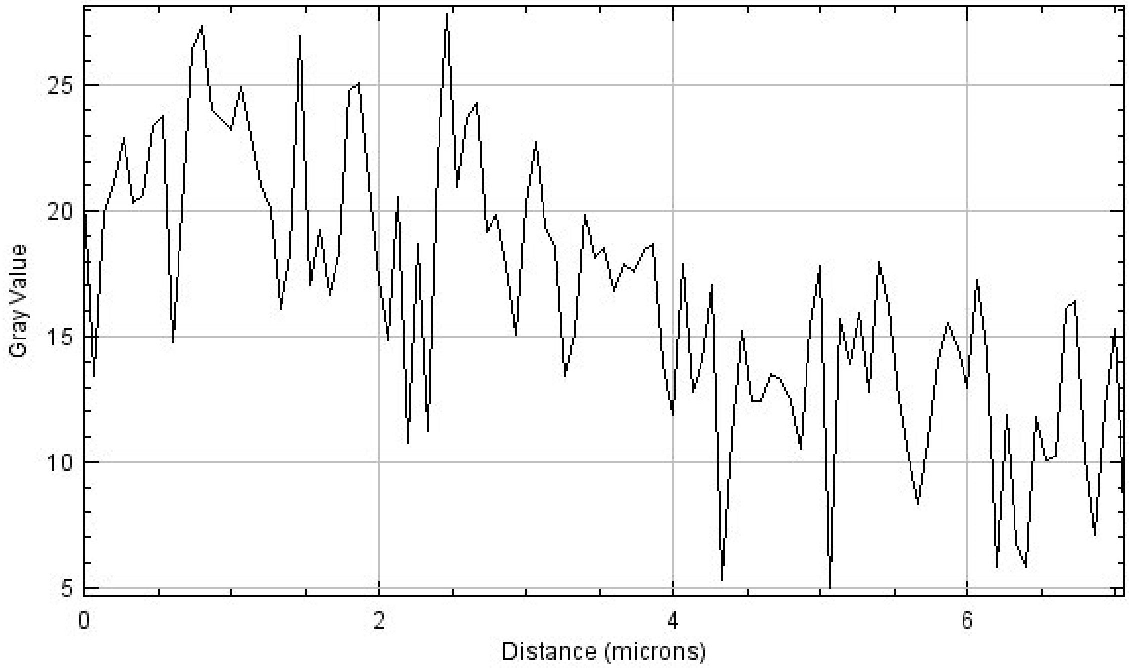

Figure S324 – Intensity profile of line scan generated for cell 37, Figure S287.

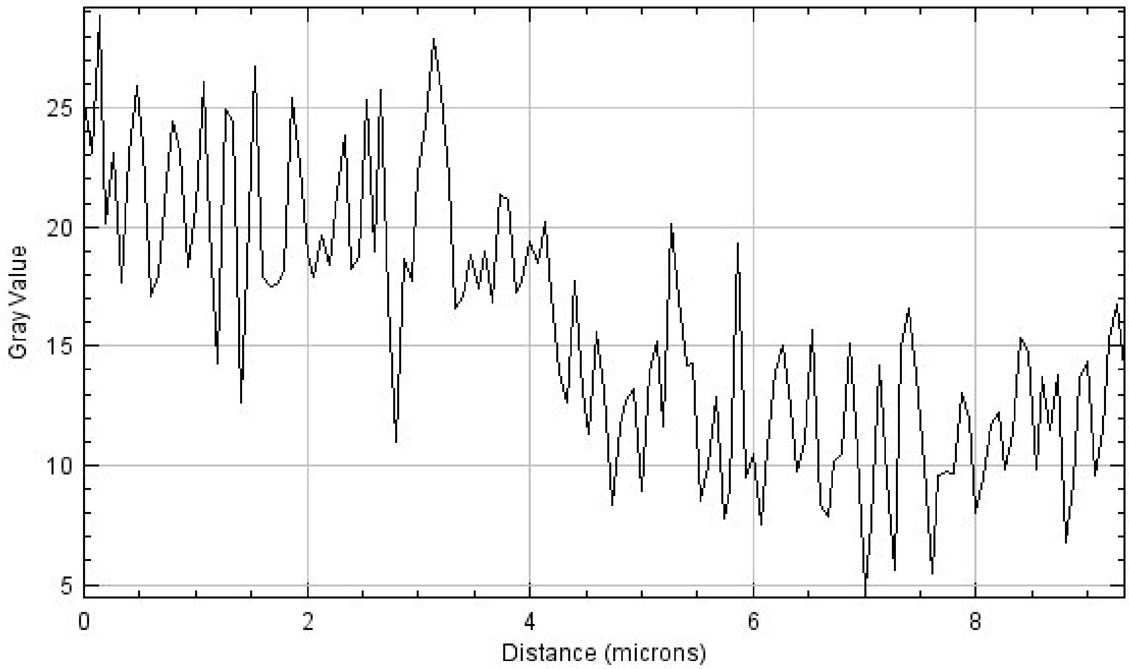

Figure S325 – Intensity profile of line scan generated for cell 38, Figure S287.

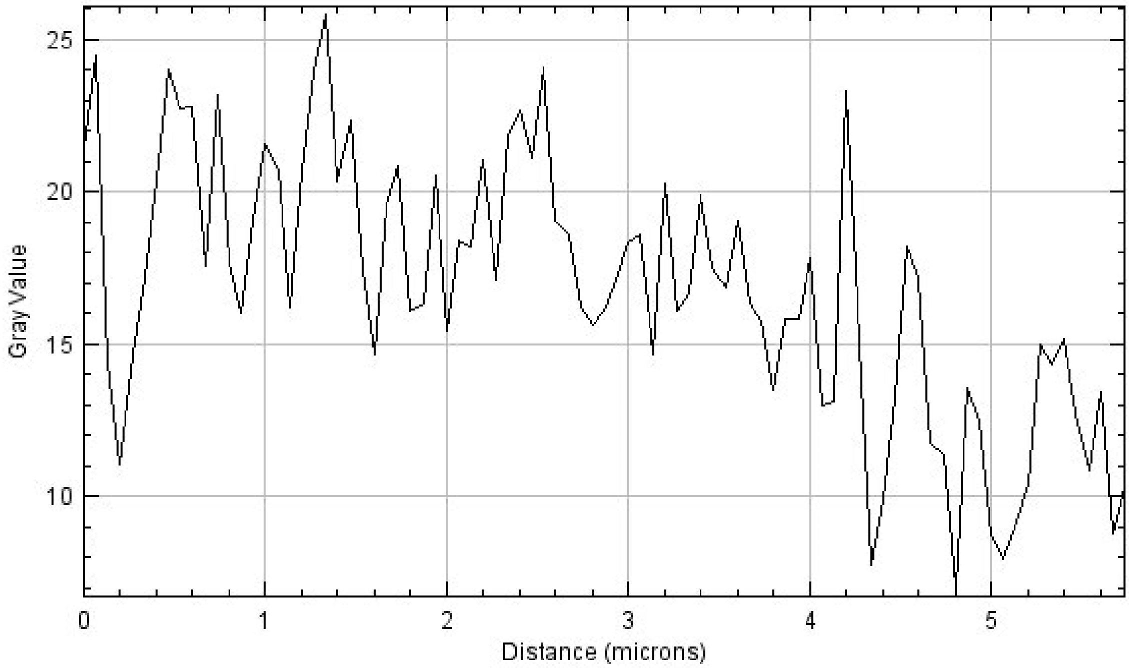

Figure S326 – Intensity profile of line scan generated for cell 39, Figure S287.

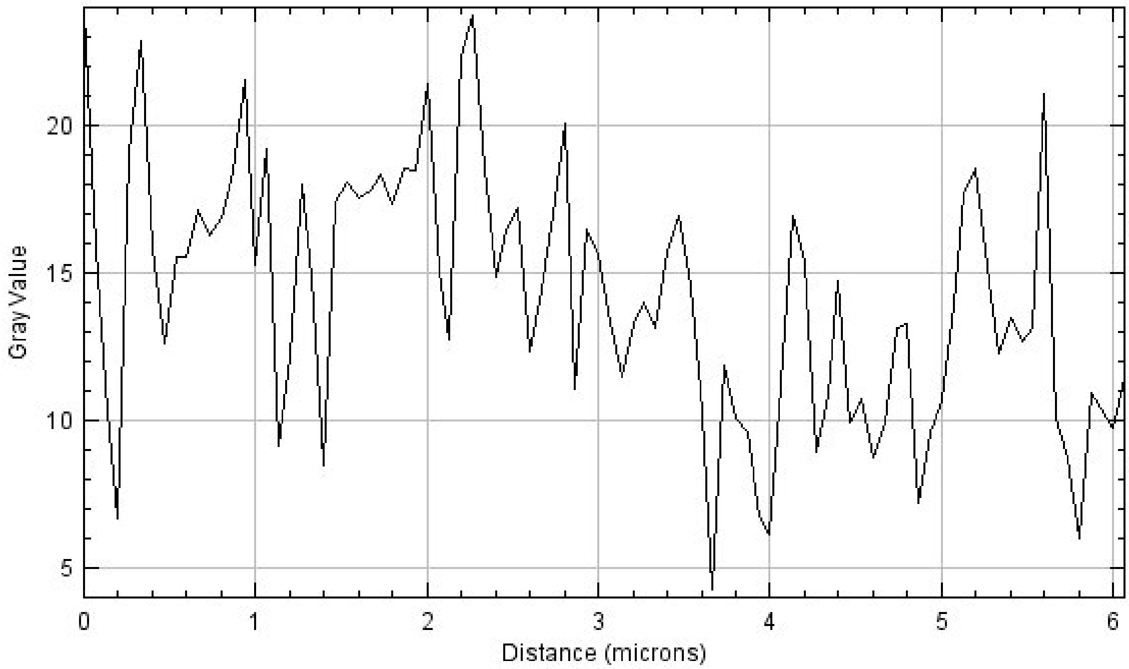

Figure S327 – Intensity profile of line scan generated for cell 40, Figure S287.

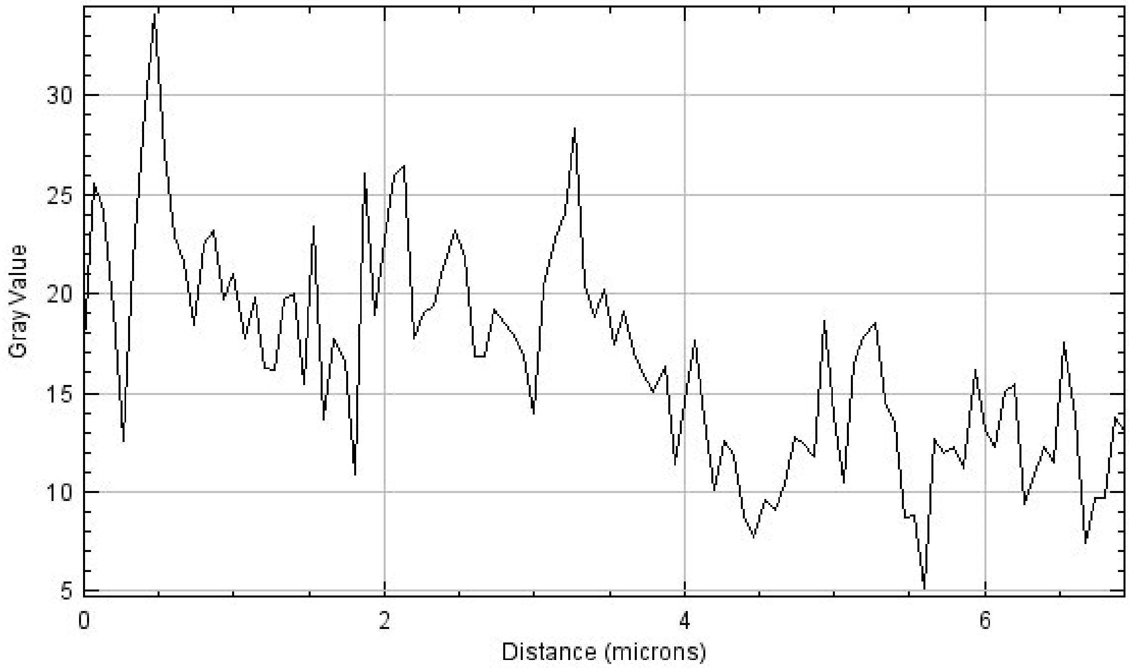

Figure S328 – Intensity profile of line scan generated for cell 41, Figure S287.

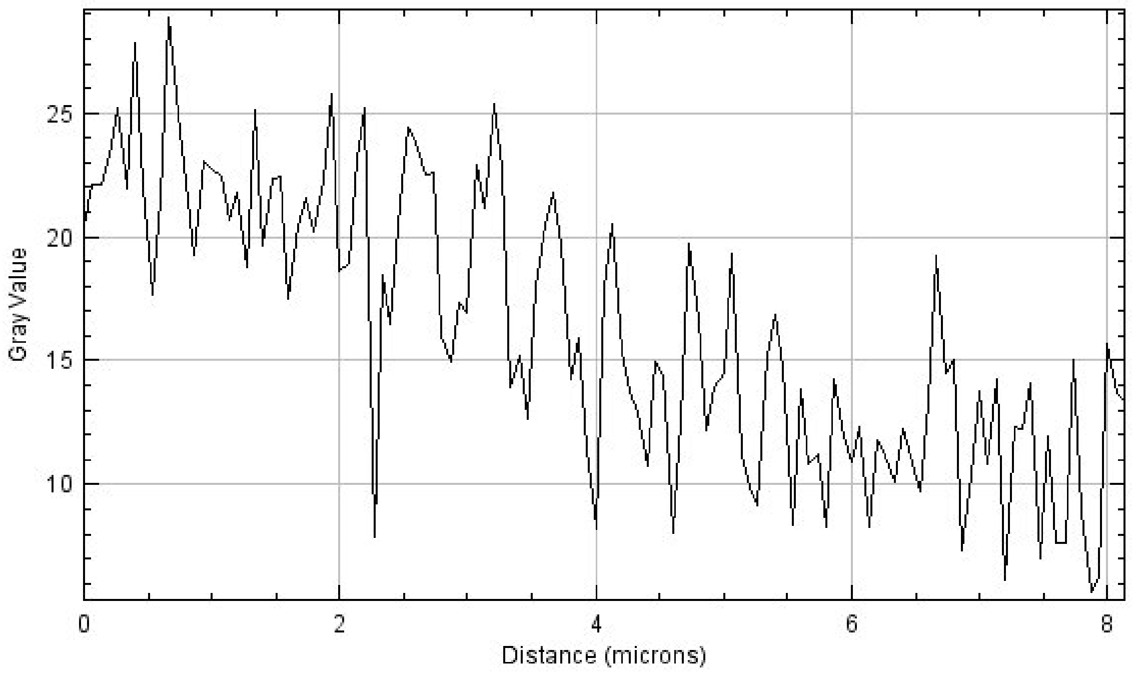

Figure S329 – Intensity profile of line scan generated for cell 42, Figure S287.

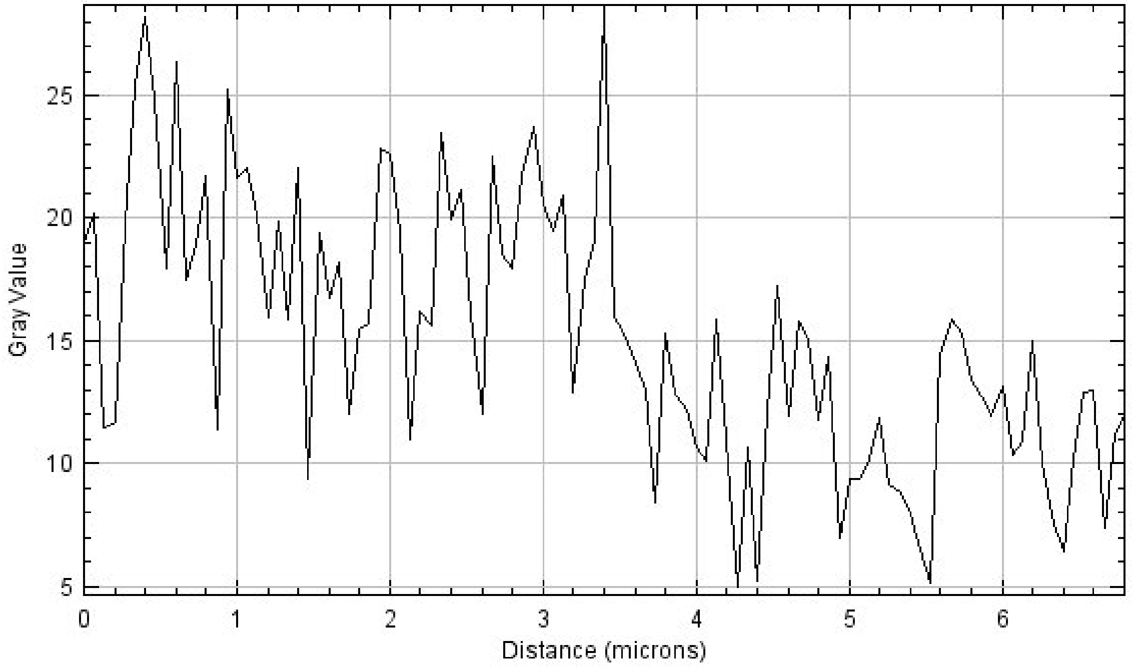

Figure S330 – Intensity profile of line scan generated for cell 43, Figure S287.

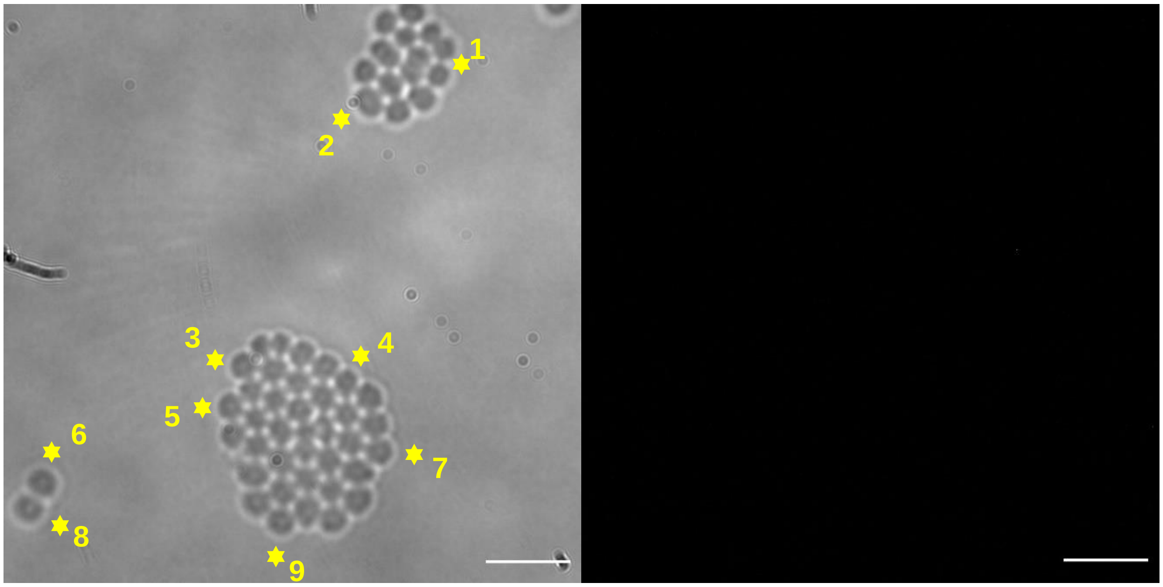

Figure S331 – MRSA transmitted image and fluorescence image at 605nm used in fluorescence intensity calculations in the absence of both compound **39** and **FM4-64** at T = 30 minutes. Transmitted image used to locate cells on fluorescence image. Scale bar = 10 µM.

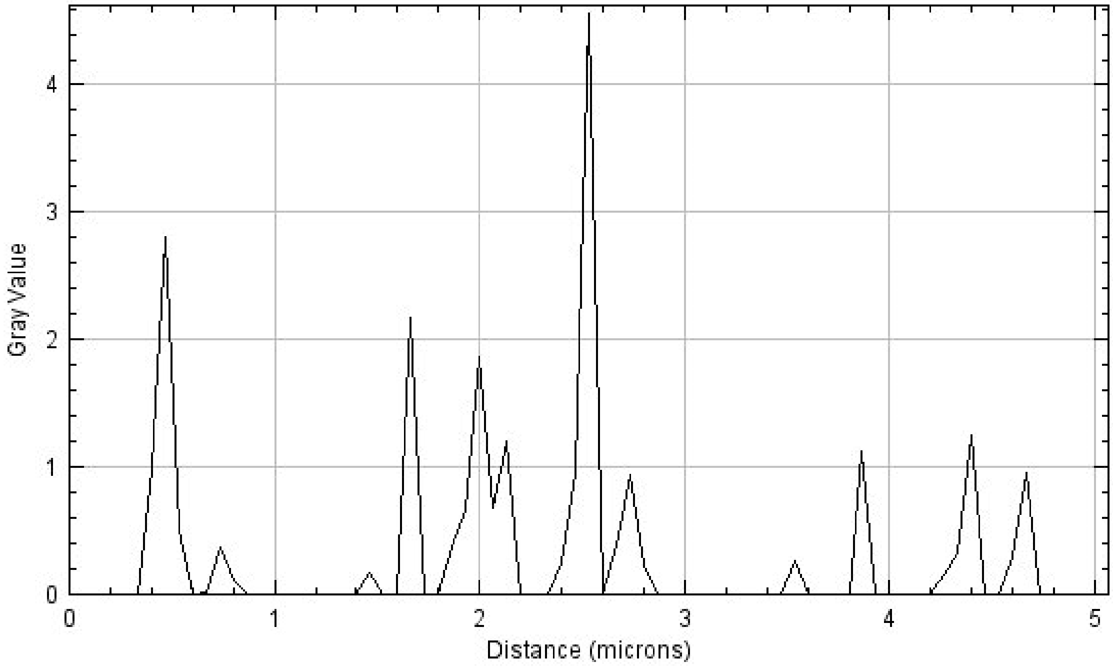

Figure S332 – Intensity profile of line scan generated for cell 1, Figure S331.

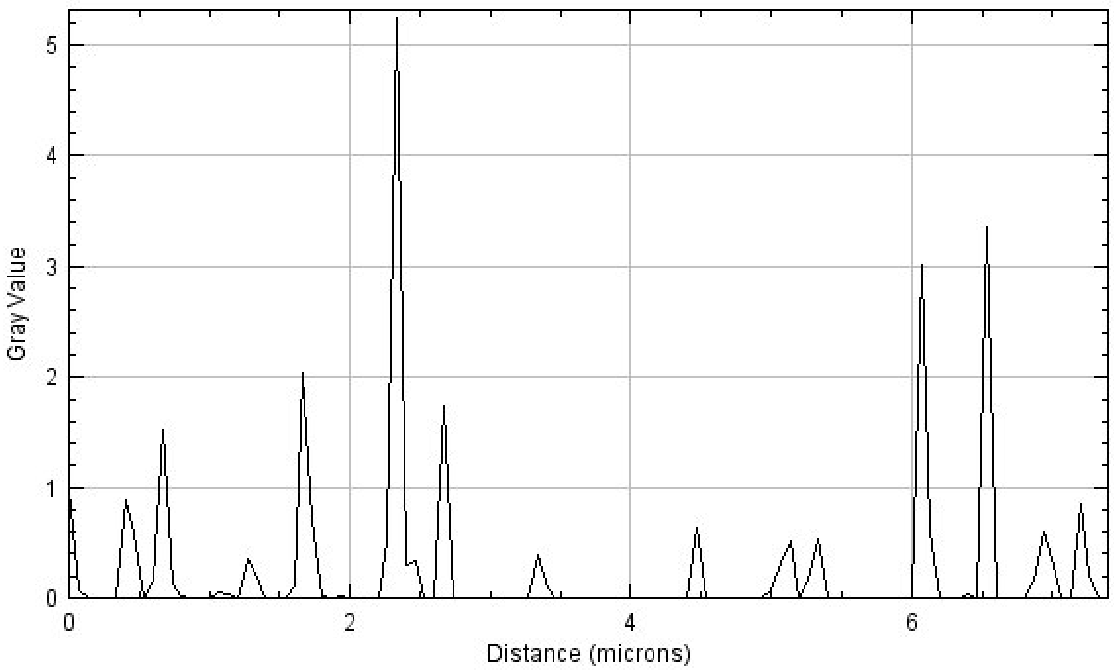

Figure S333 – Intensity profile of line scan generated for cell 2, Figure S331.

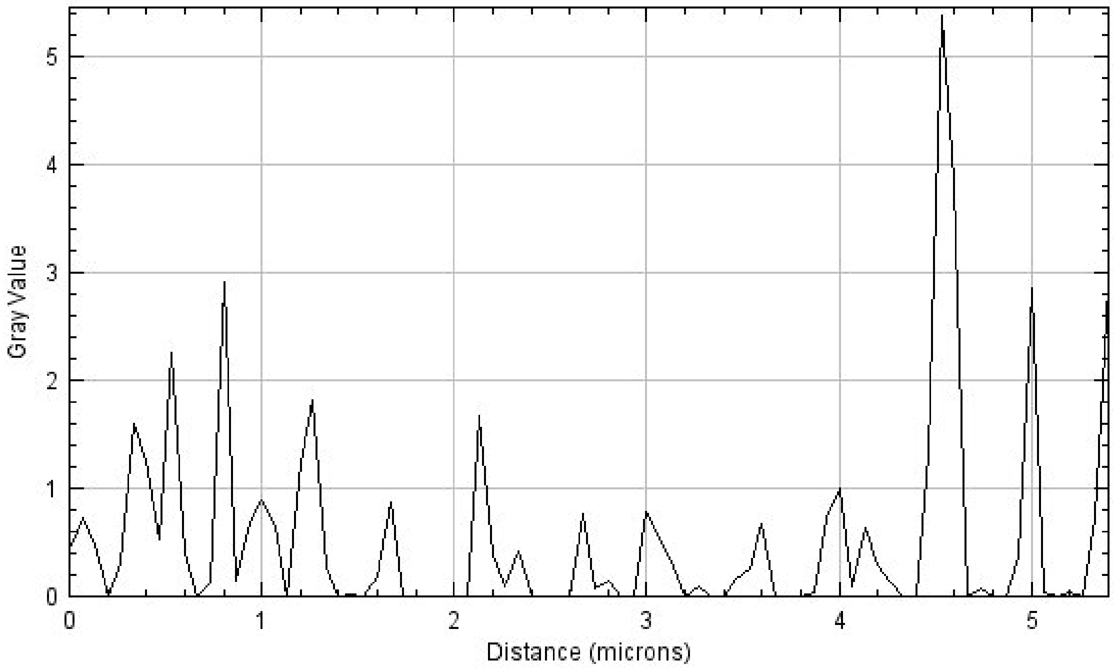

Figure S334 – Intensity profile of line scan generated for cell 3, Figure S331.

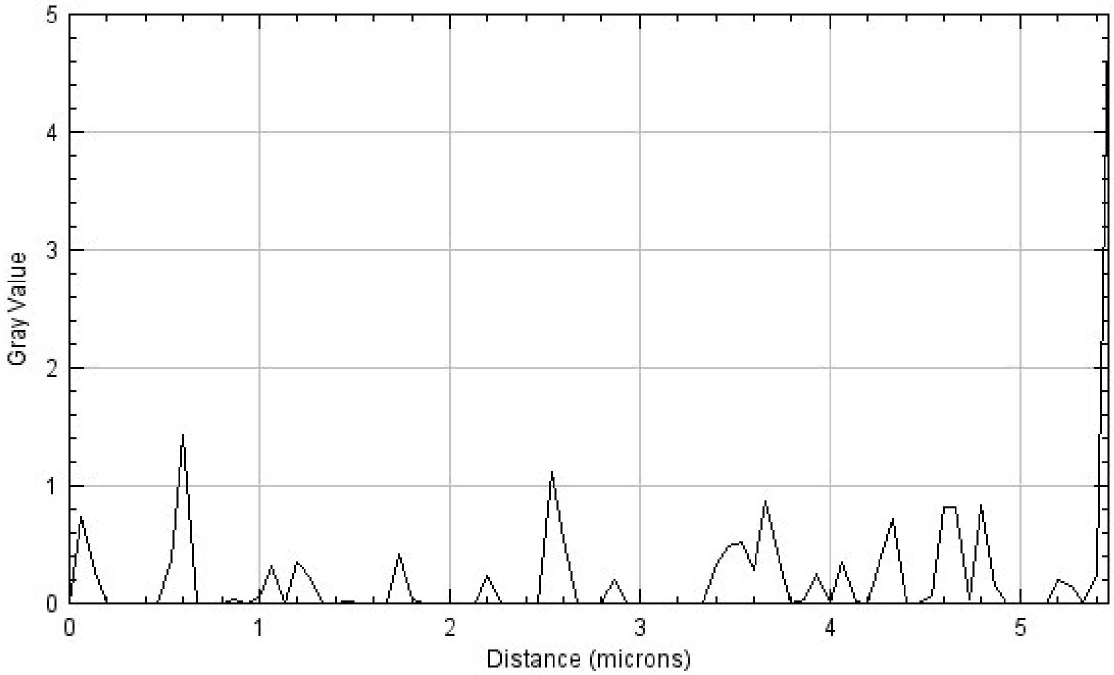

Figure S335 – Intensity profile of line scan generated for cell 4, Figure S331.

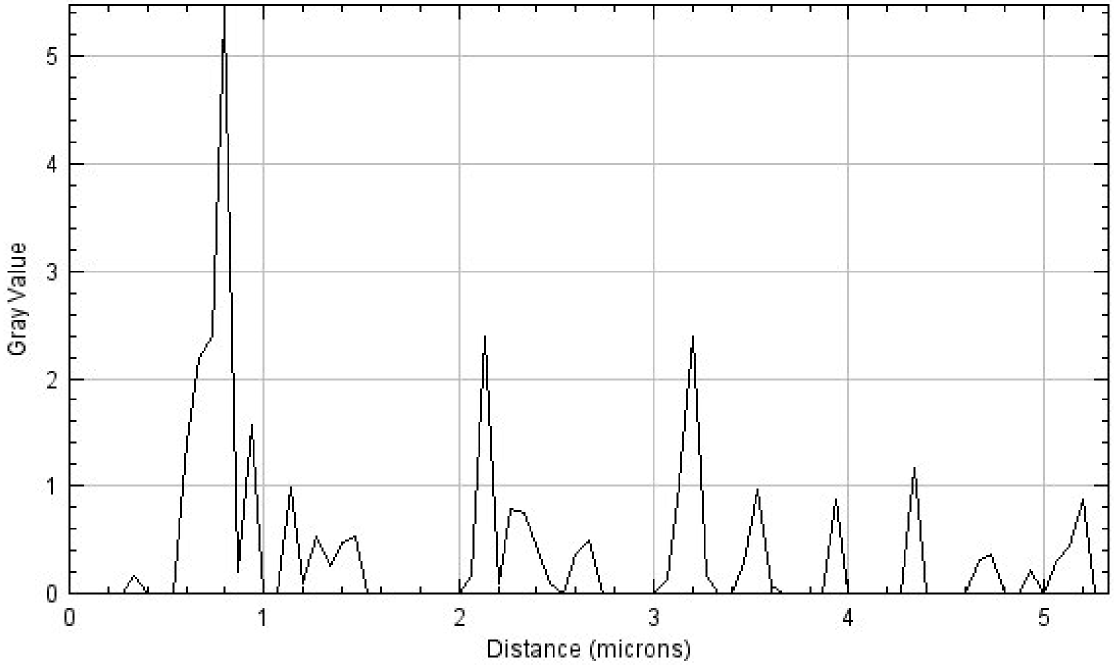

Figure S336 – Intensity profile of line scan generated for cell 5, Figure S331.

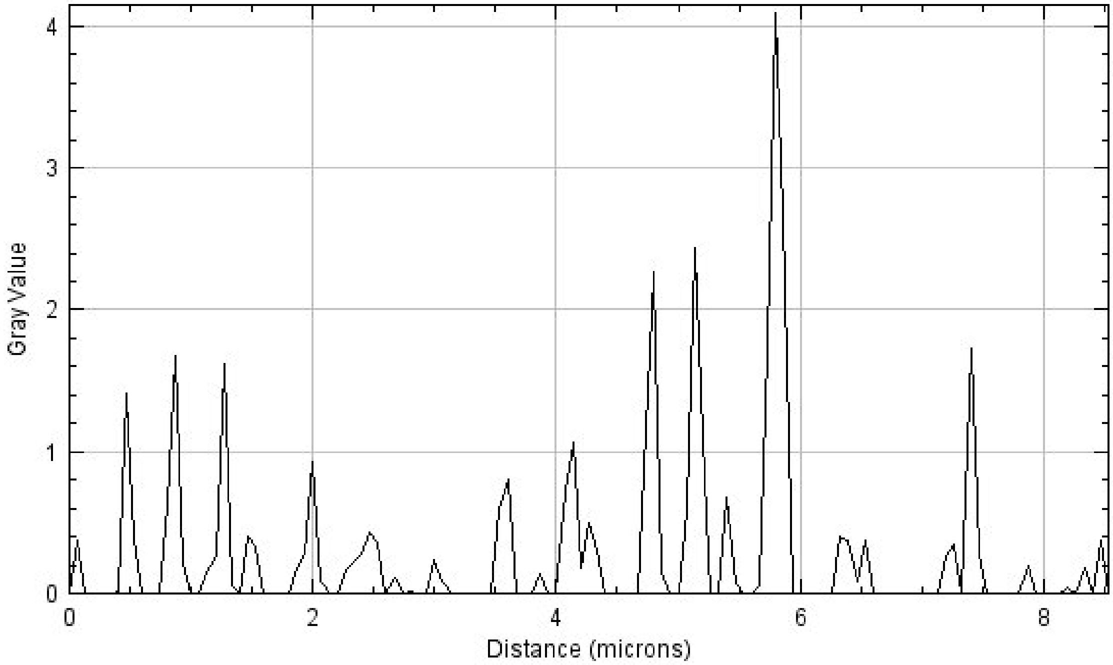

Figure S337 – Intensity profile of line scan generated for cell 6, Figure S331.

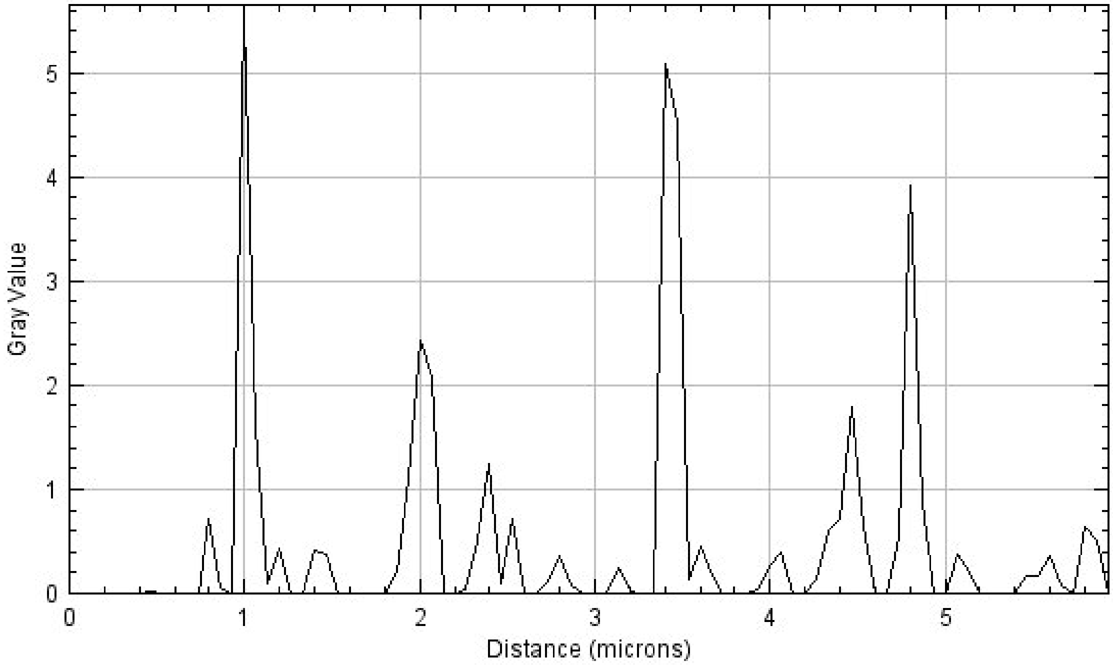

Figure S338 – Intensity profile of line scan generated for cell 7, Figure S331.

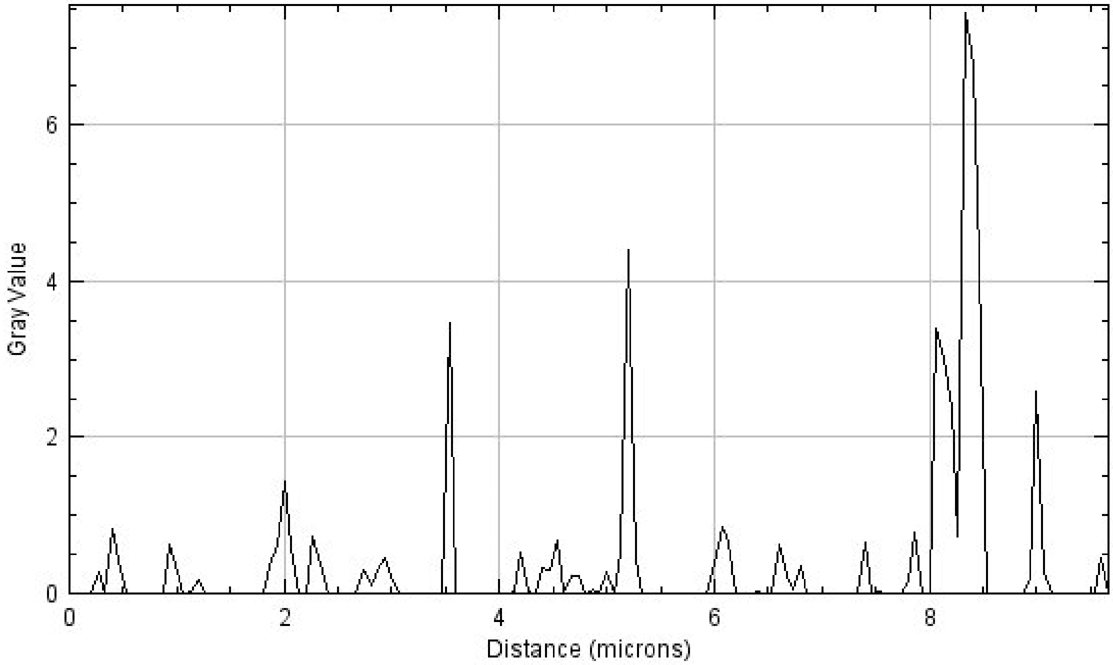

Figure S339 – Intensity profile of line scan generated for cell 8, Figure S331.

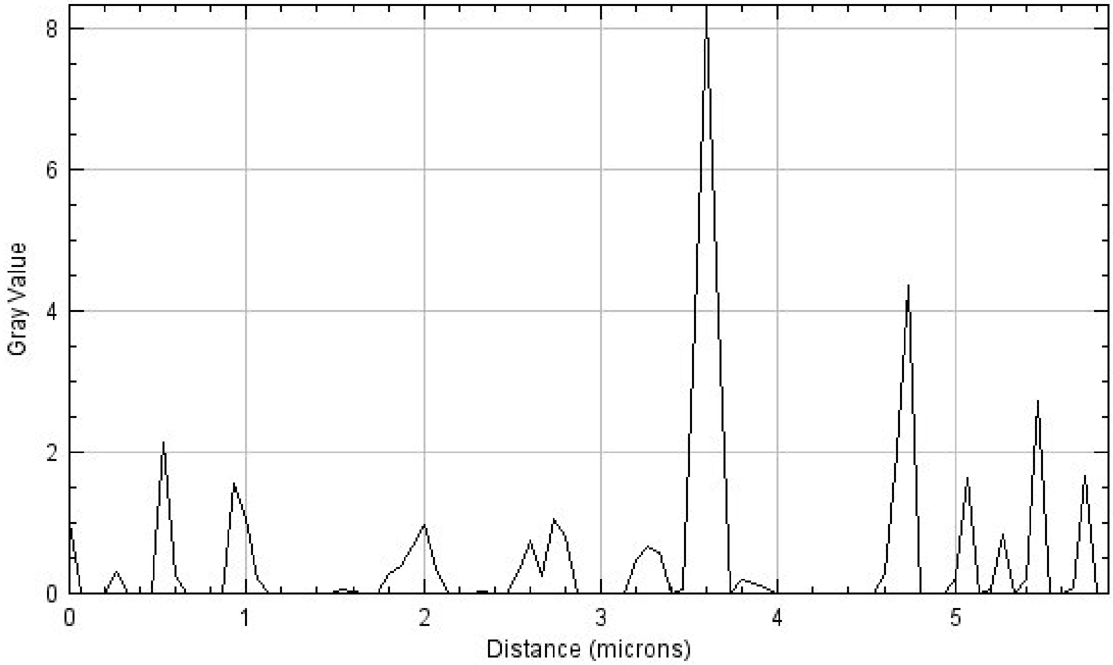

Figure S340 – Intensity profile of line scan generated for cell 9, Figure S331.

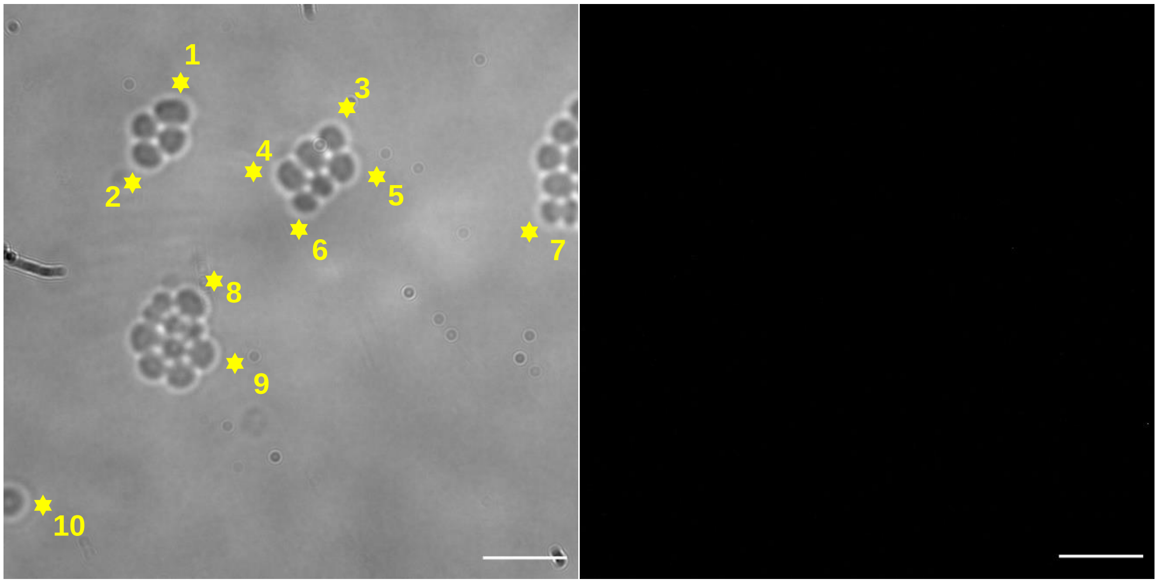

Figure S341 – MRSA transmitted image and fluorescence image at 605 nm used in fluorescence intensity calculations in the absence of both compound **39** and **FM4-64** at T = 30 minutes. Transmitted image used to locate cells on fluorescence image. Scale bar = 10 μM.

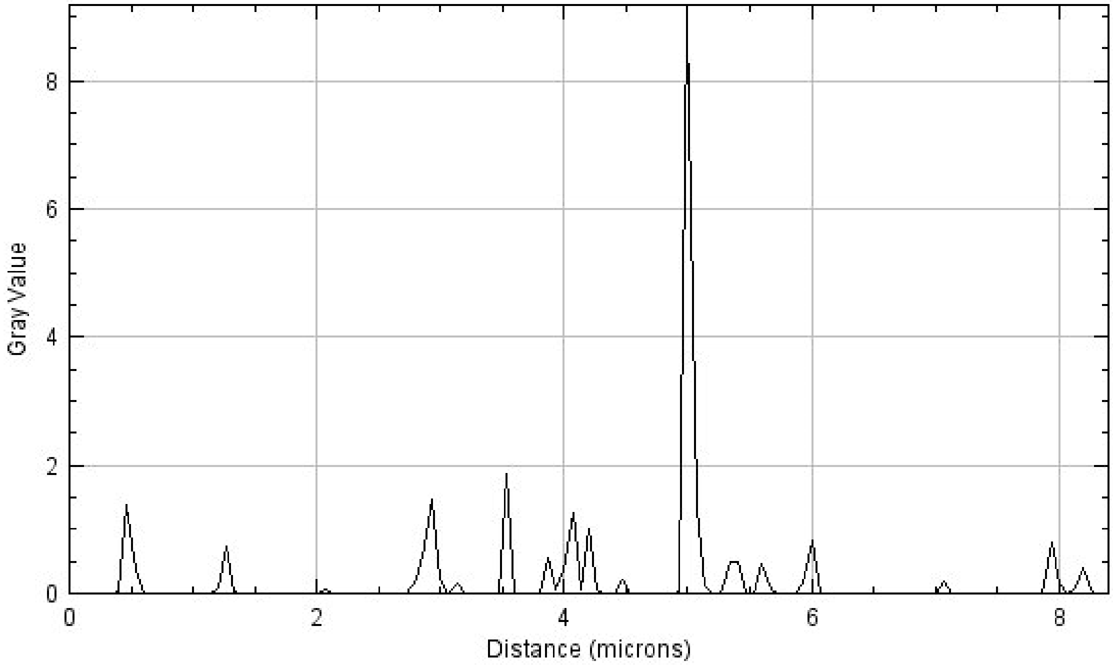

Figure S342 – Intensity profile of line scan generated for cell 1, Figure S341.

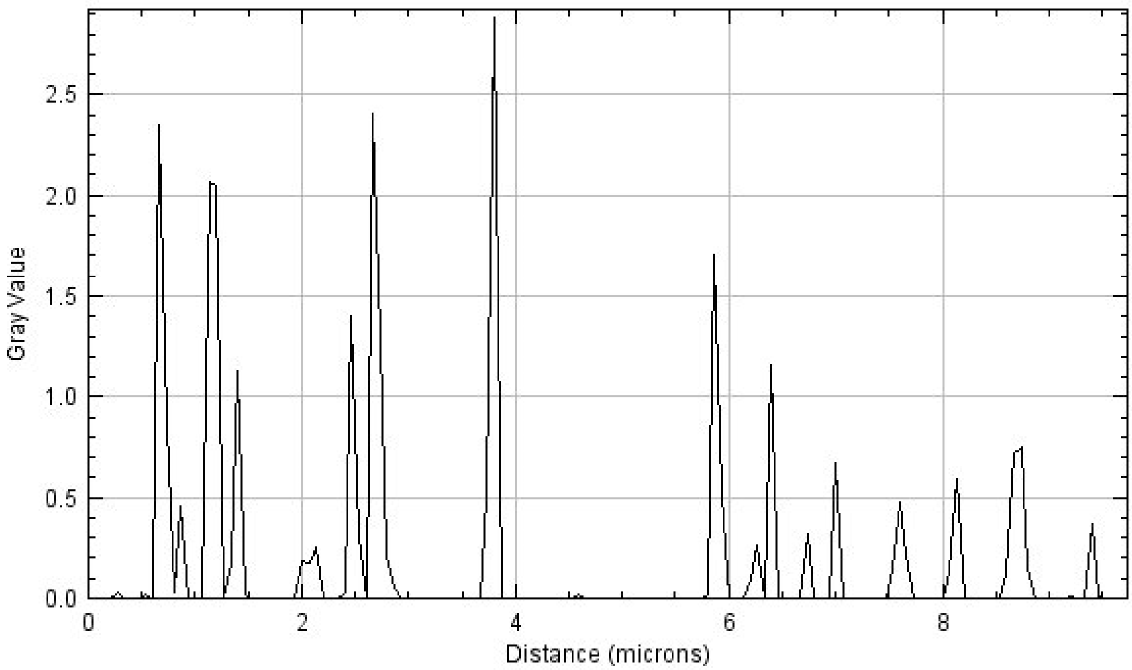

Figure S343 – Intensity profile of line scan generated for cell 2, Figure S341.

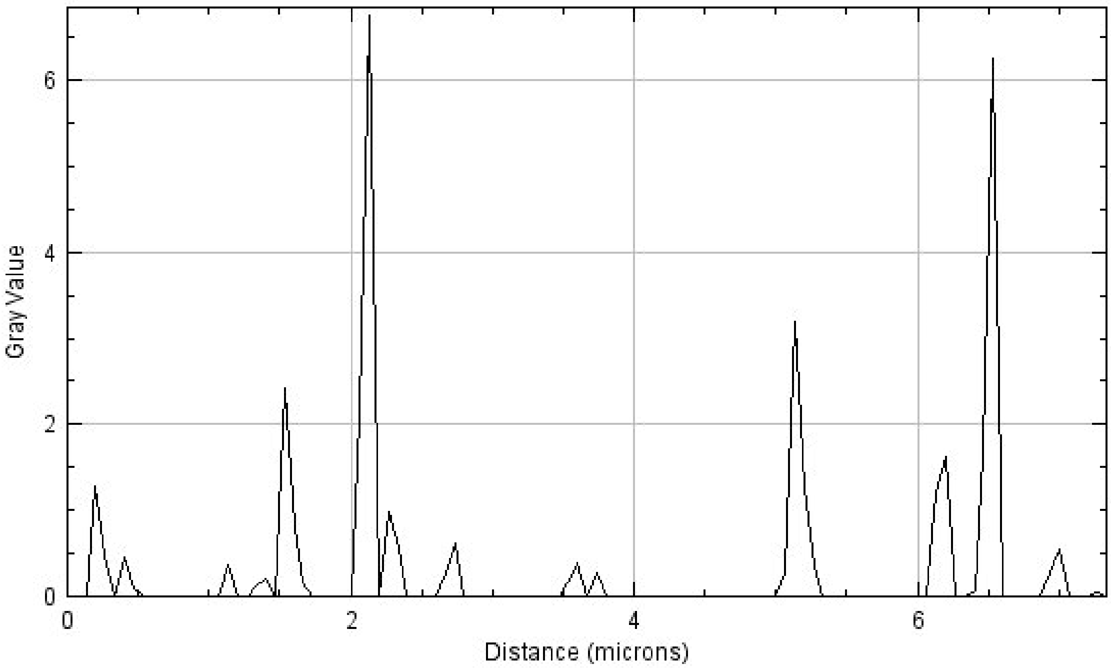

Figure S344 – Intensity profile of line scan generated for cell 3, Figure S341.

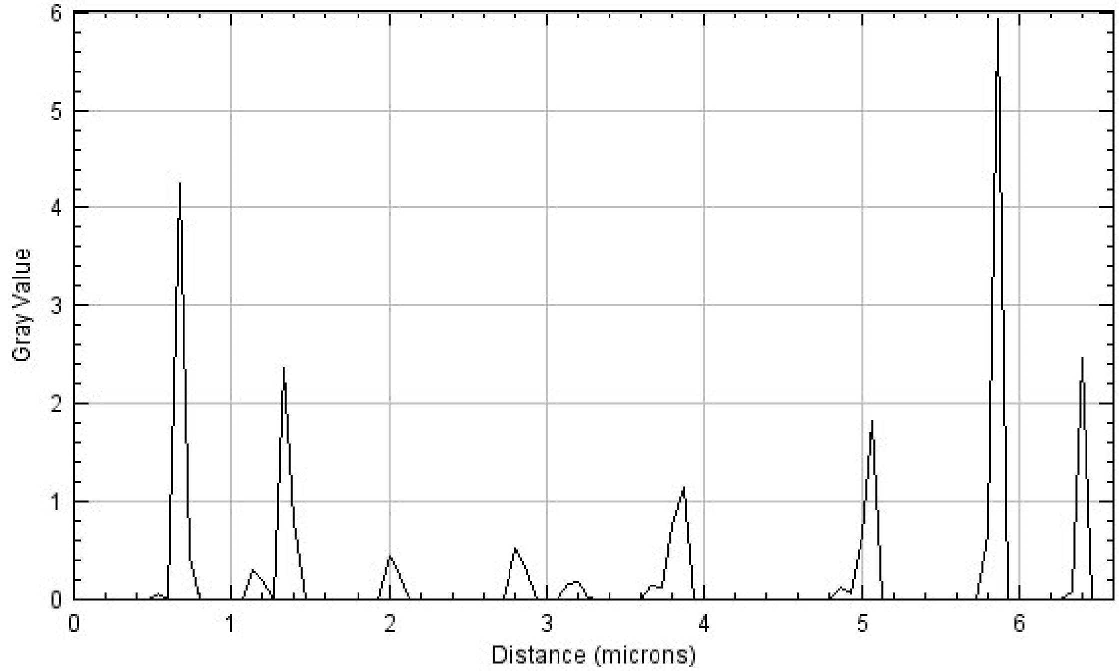

Figure S345 – Intensity profile of line scan generated for cell 4, Figure S341.

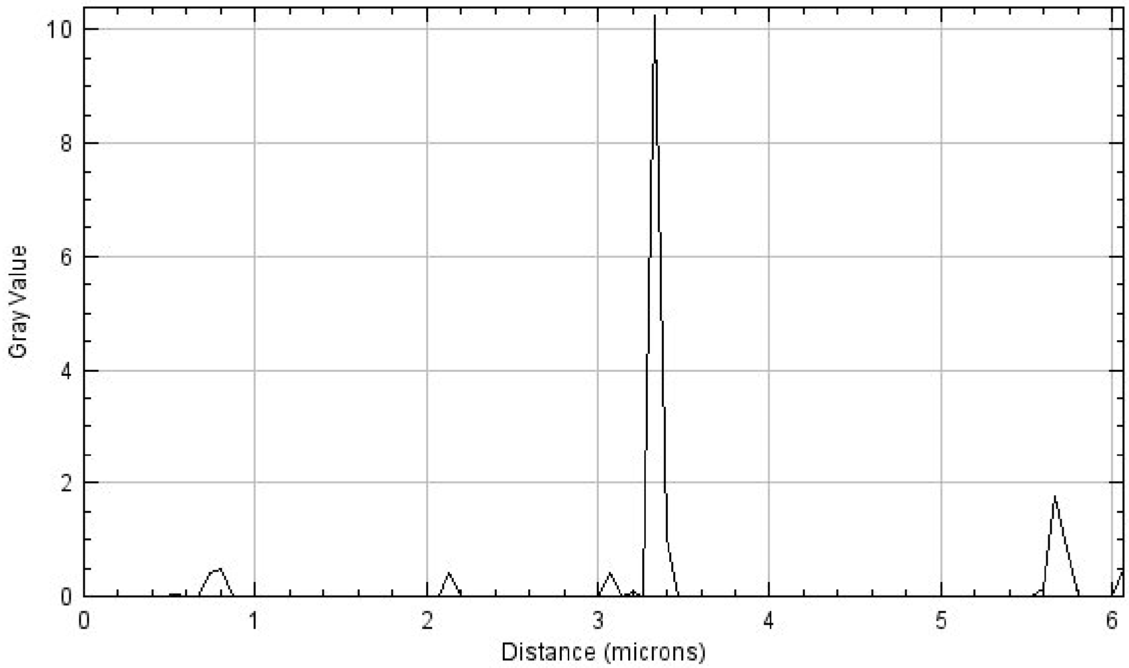

Figure S346 – Intensity profile of line scan generated for cell 5 , Figure S341.

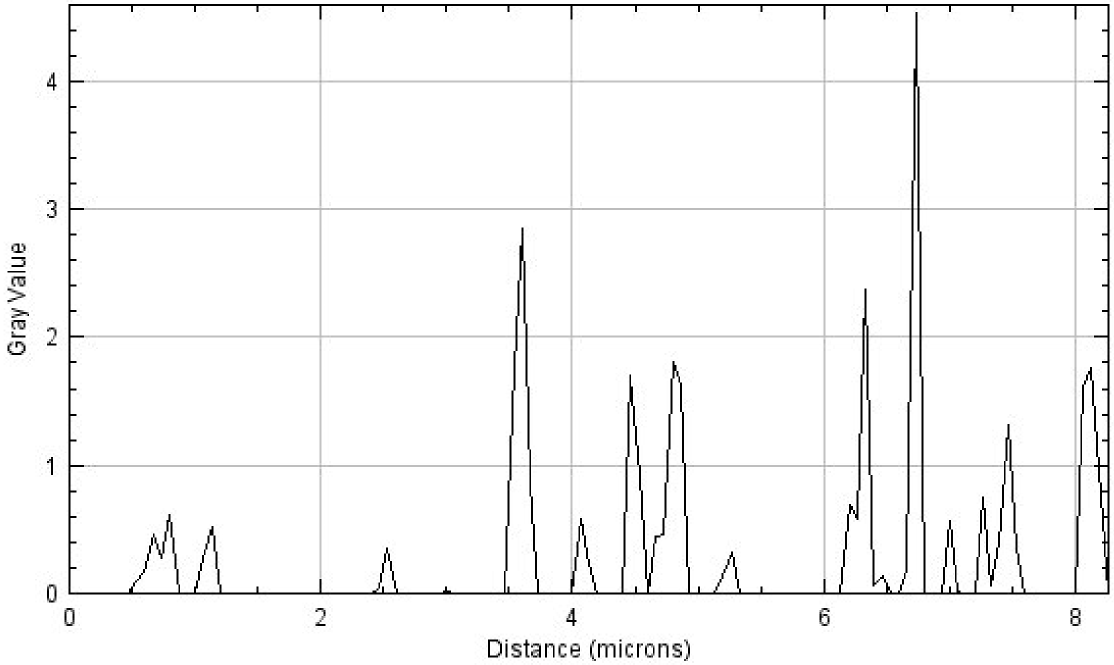

Figure S347 – Intensity profile of line scan generated for cell 6, Figure S341.

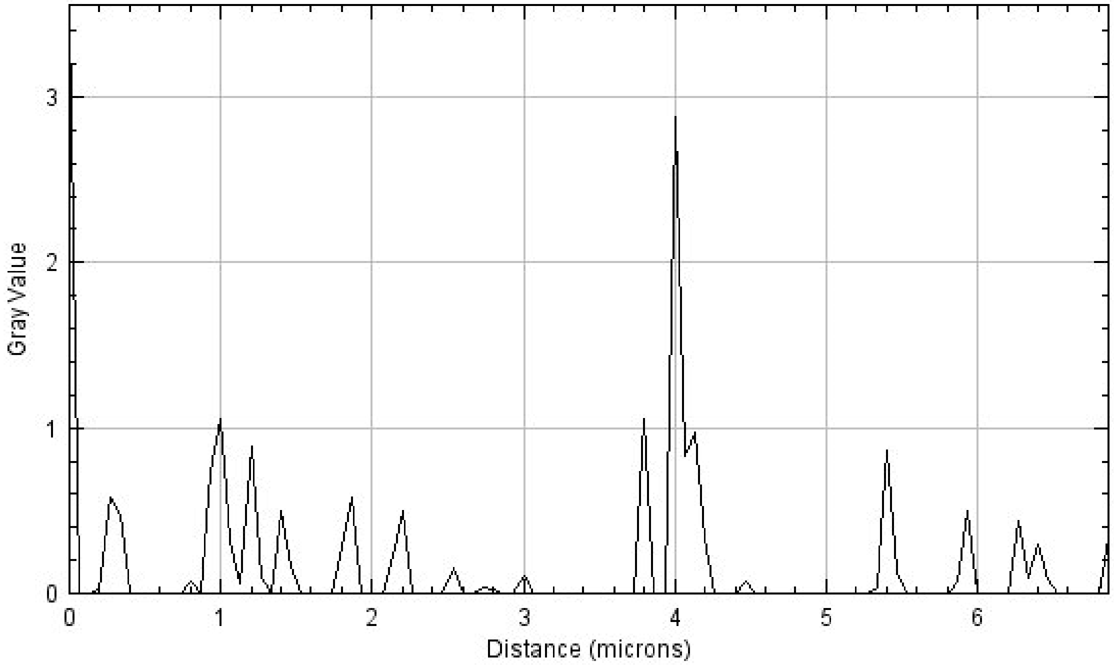

Figure S348 – Intensity profile of line scan generated for cell 7, Figure S341.

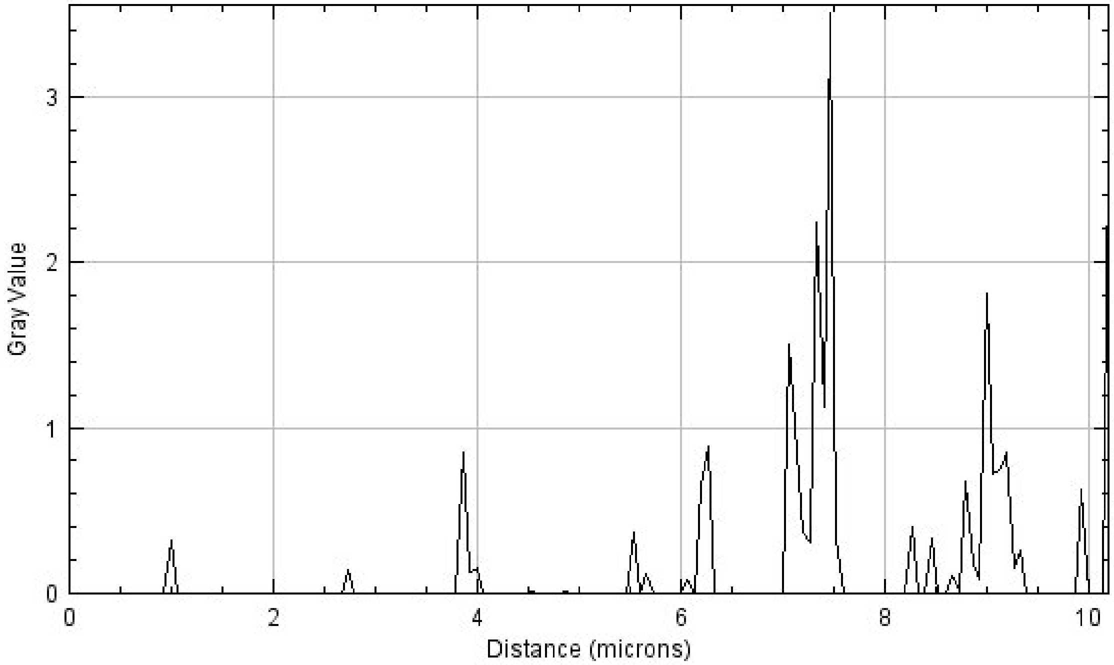

Figure S349 – Intensity profile of line scan generated for cell 8, Figure S341.

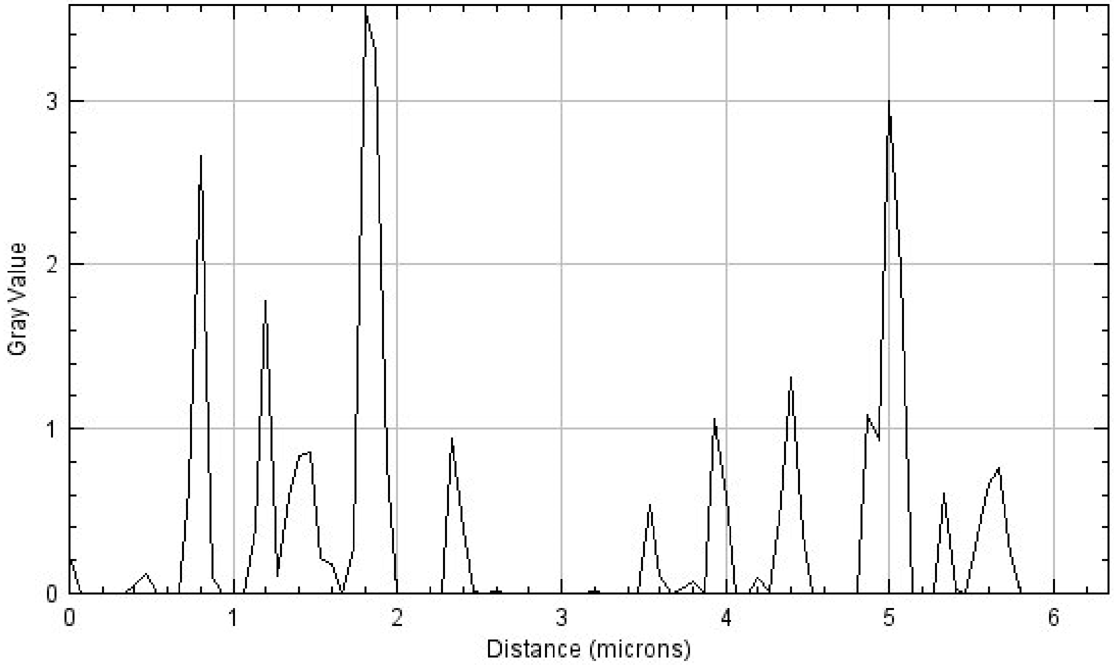

Figure S350 – Intensity profile of line scan generated for cell 9, Figure S341.

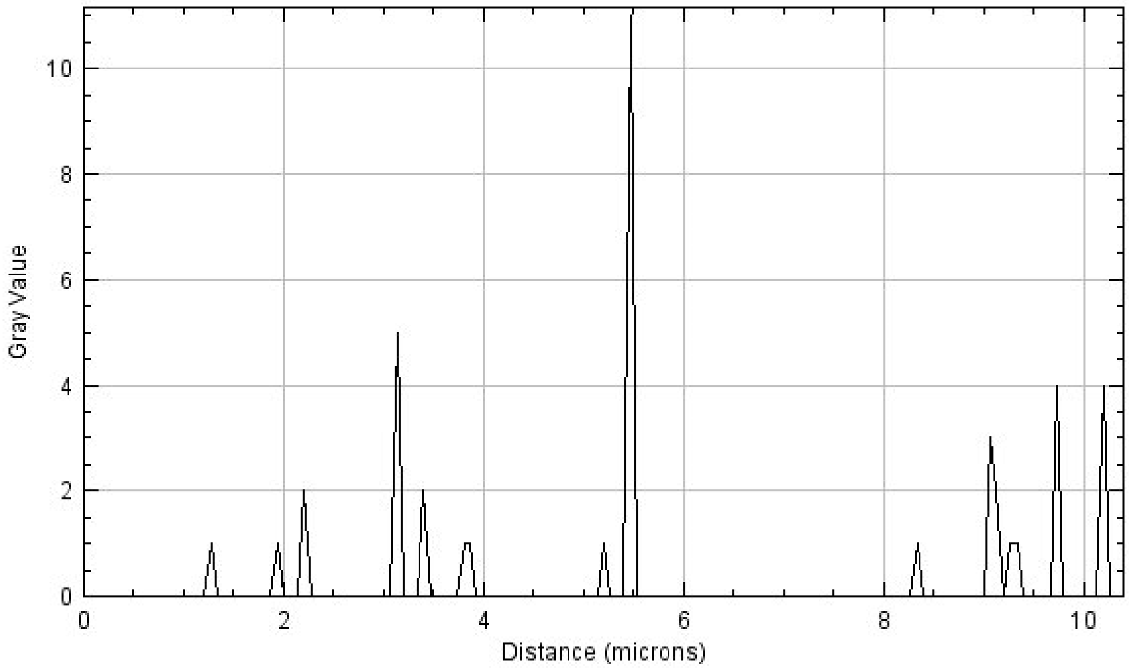

Figure S351 – Intensity profile of line scan generated for cell 10, Figure S341.

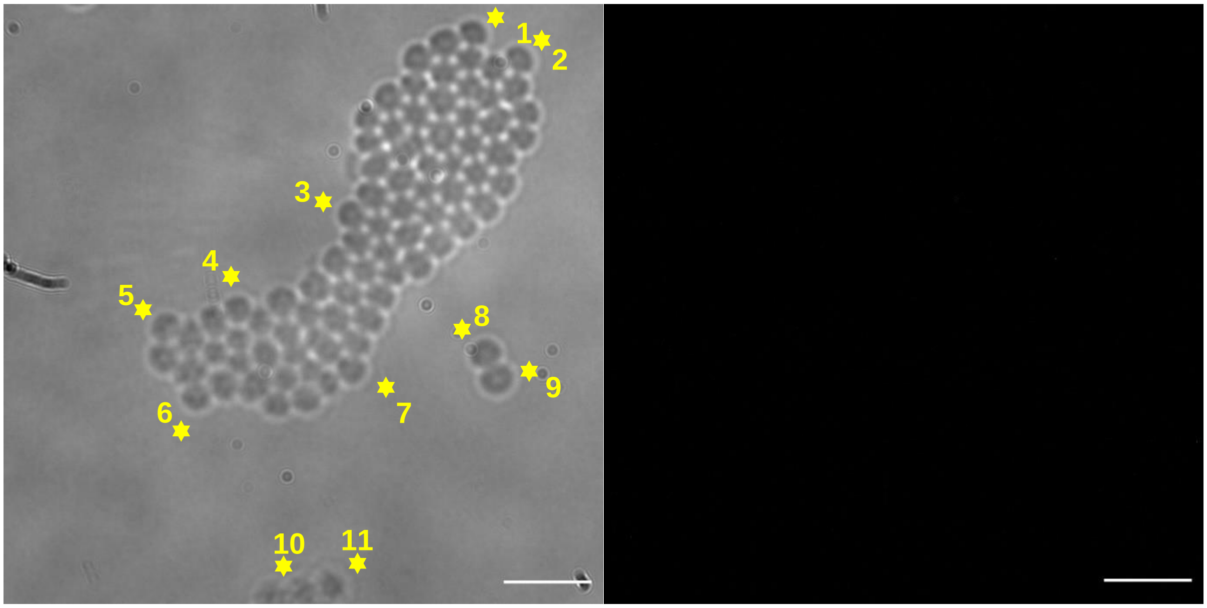

Figure S352 – MRSA transmitted image and fluorescence image at 605 nm used in fluorescence intensity calculations in the absence of both compound **39** and **FM4-64** at T = 30 minutes. Transmitted image used to locate cells on fluorescence image. Scale bar = 10 µM.

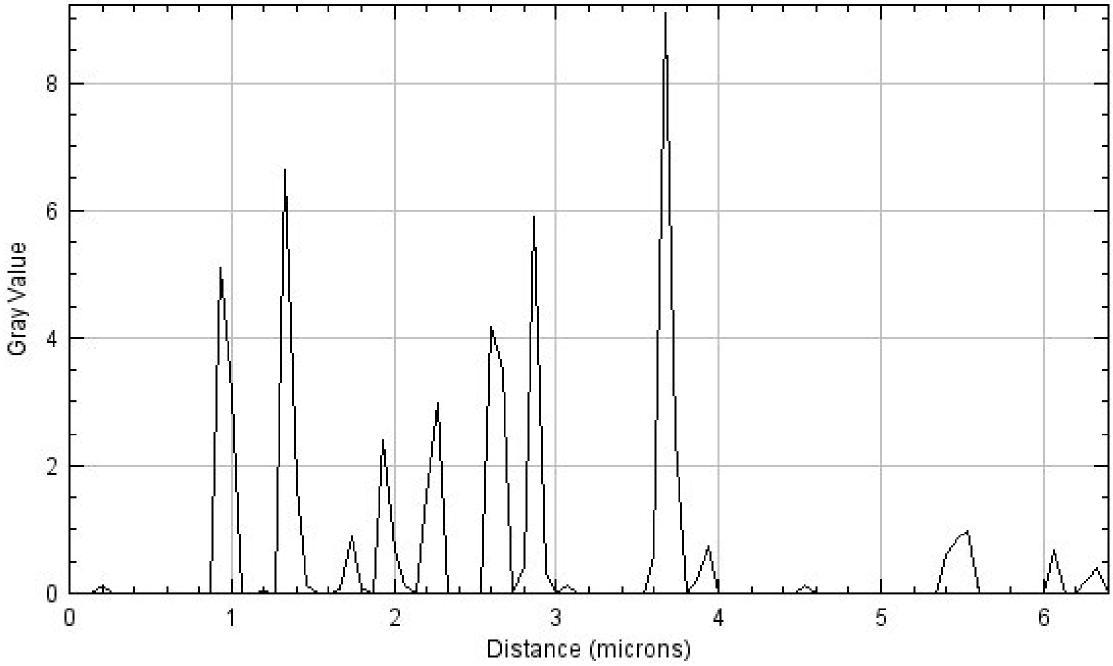

Figure S353 – Intensity profile of line scan generated for cell 1, Figure 352.

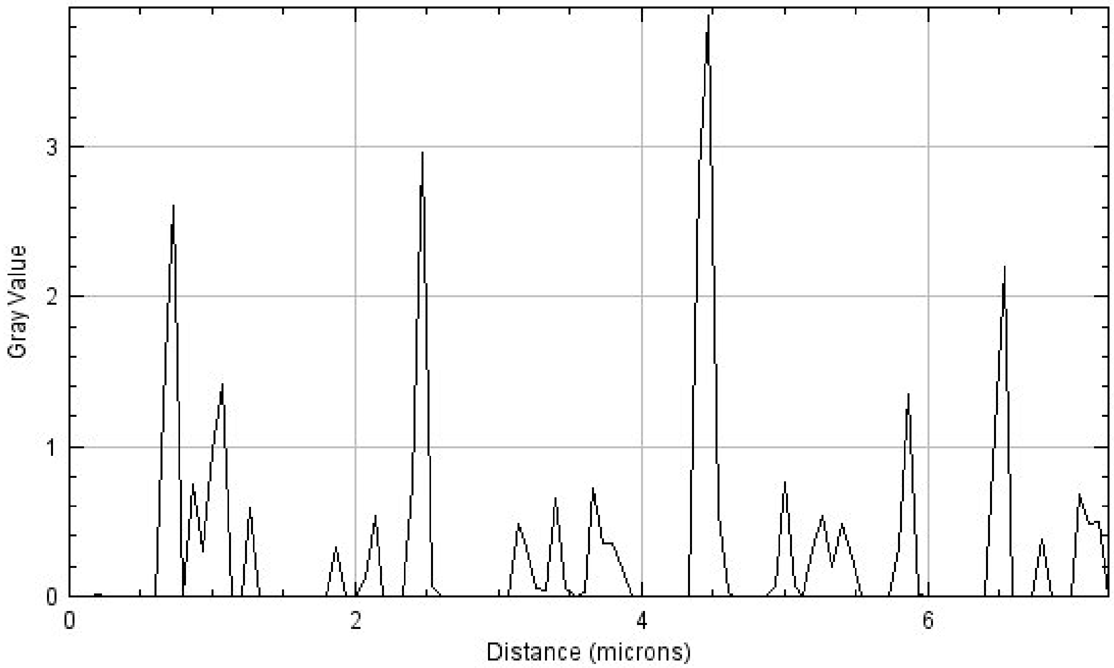

Figure S354 – Intensity profile of line scan generated for cell 2, Figure 352.

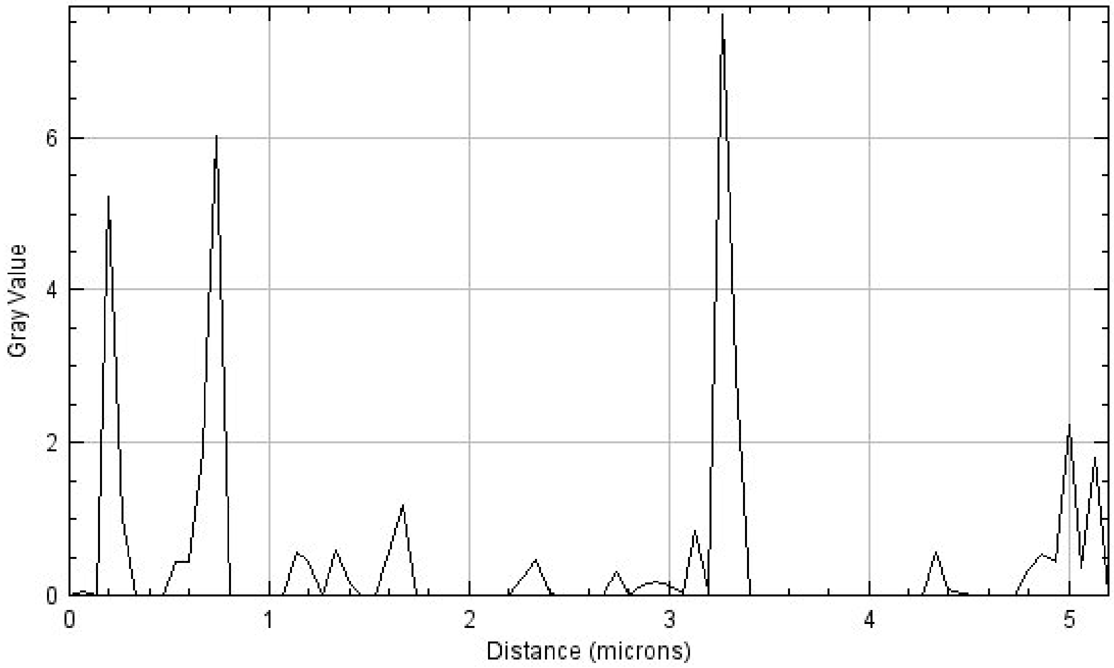

Figure S355 – Intensity profile of line scan generated for cell 3, Figure 352.

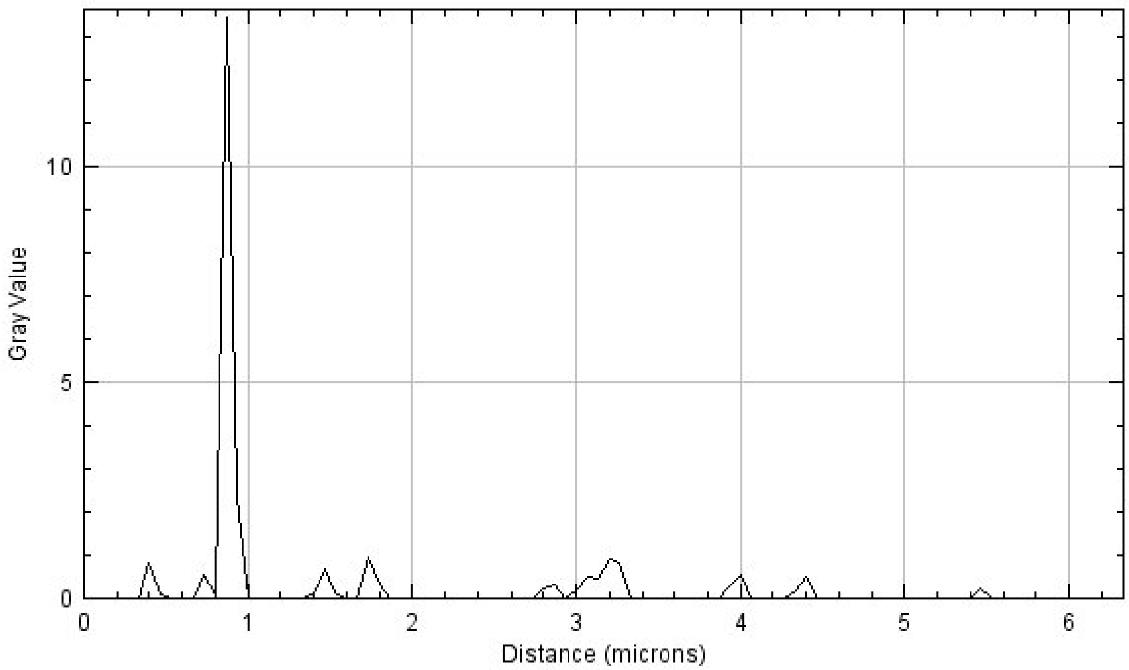

Figure S356 – Intensity profile of line scan generated for cell 4, Figure 352.

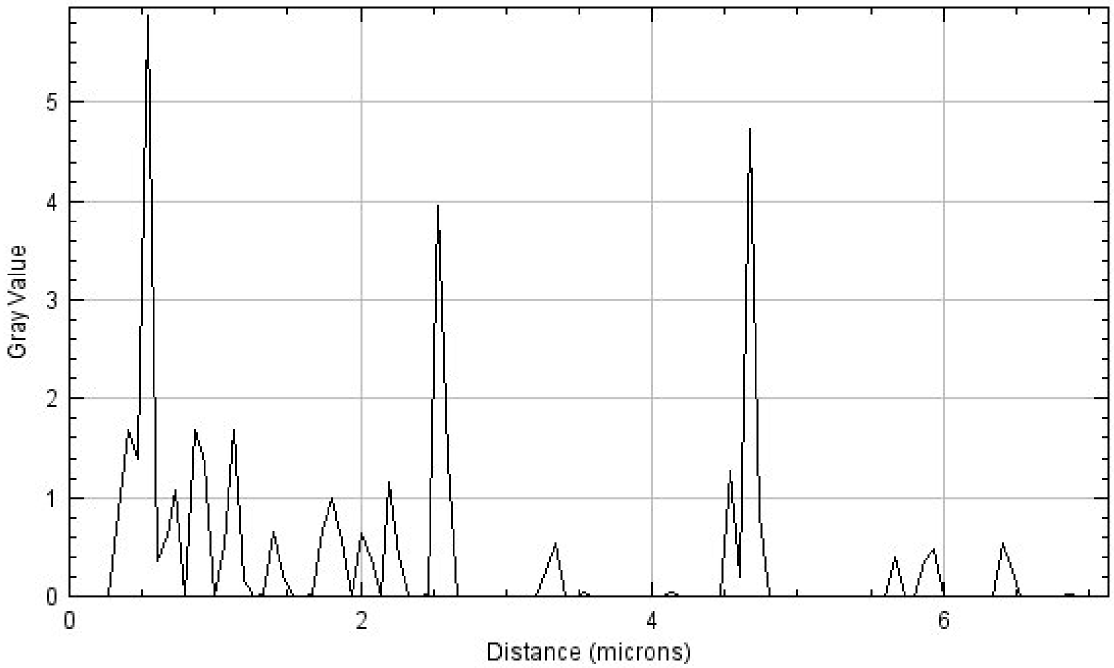

Figure S357 – Intensity profile of line scan generated for cell 5, Figure 352.

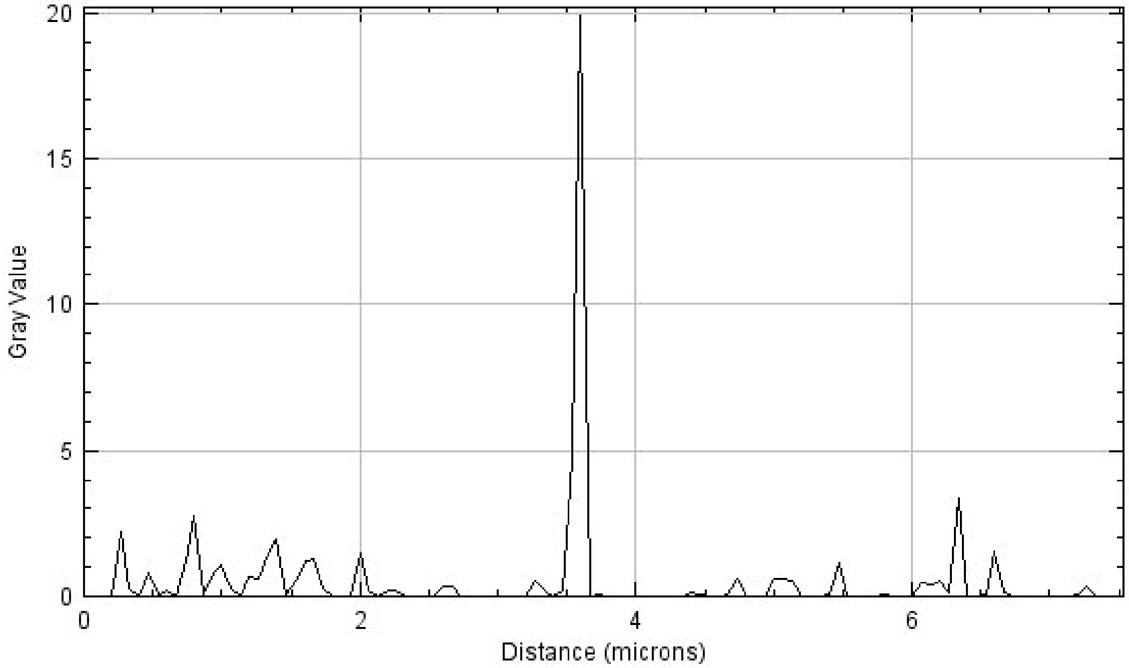

Figure S358 – Intensity profile of line scan generated for cell 6, Figure 352.

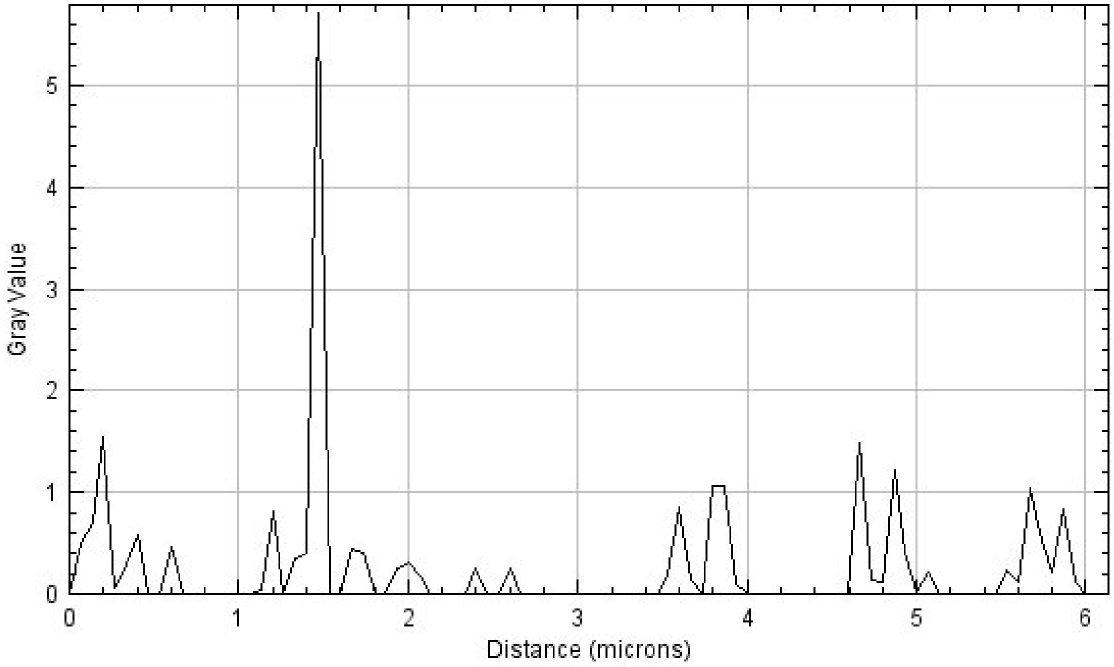

Figure S359 – Intensity profile of line scan generated for cell 7, Figure 352.

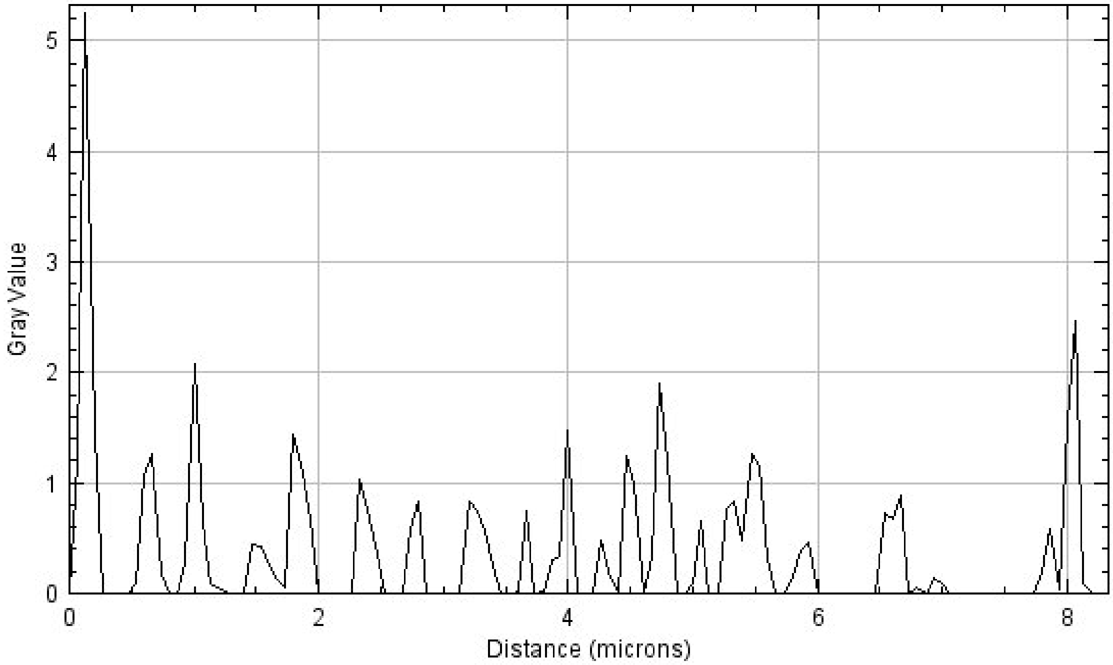

Figure S360 – Intensity profile of line scan generated for cell 8, Figure 352.

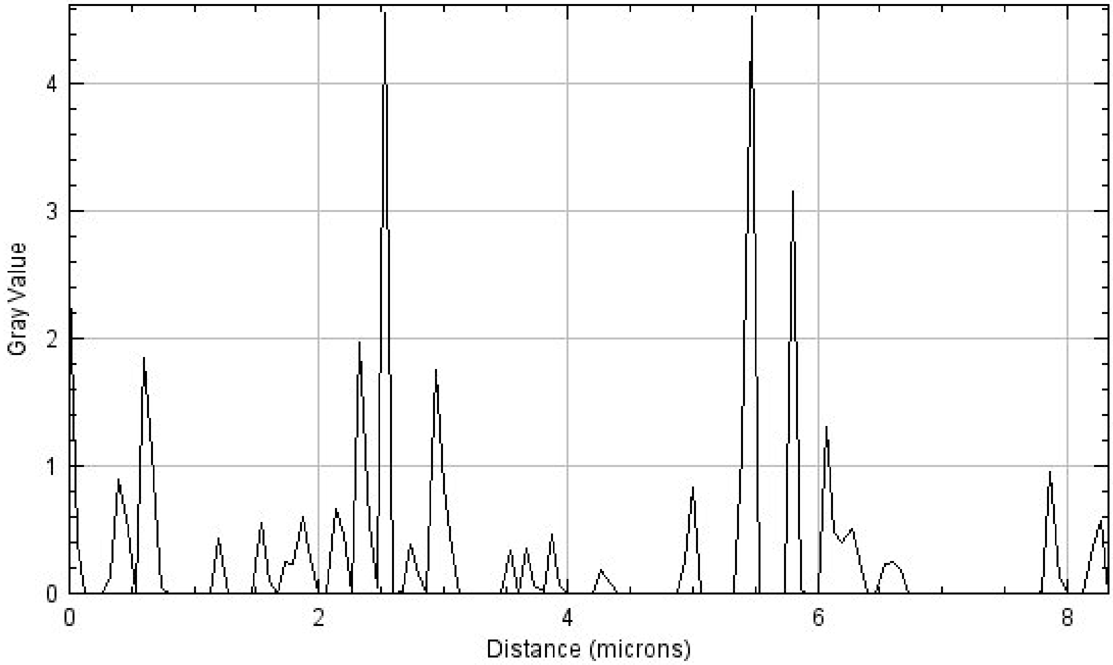

Figure S361 – Intensity profile of line scan generated for cell 9, Figure 352.

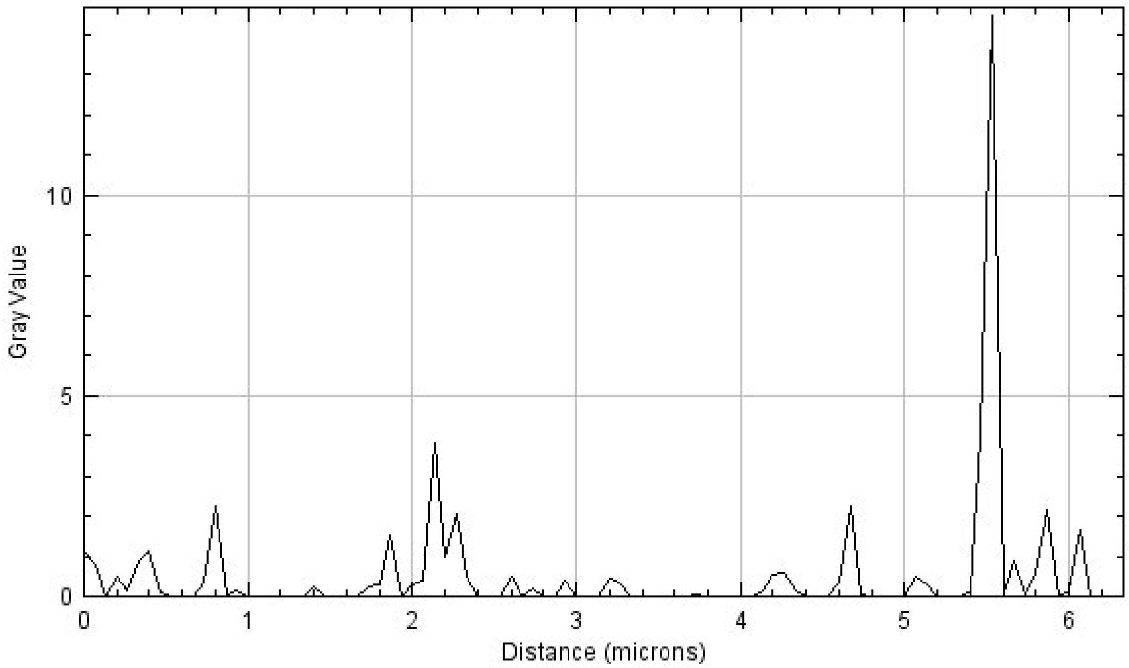

Figure S362 – Intensity profile of line scan generated for cell 10, Figure 352.

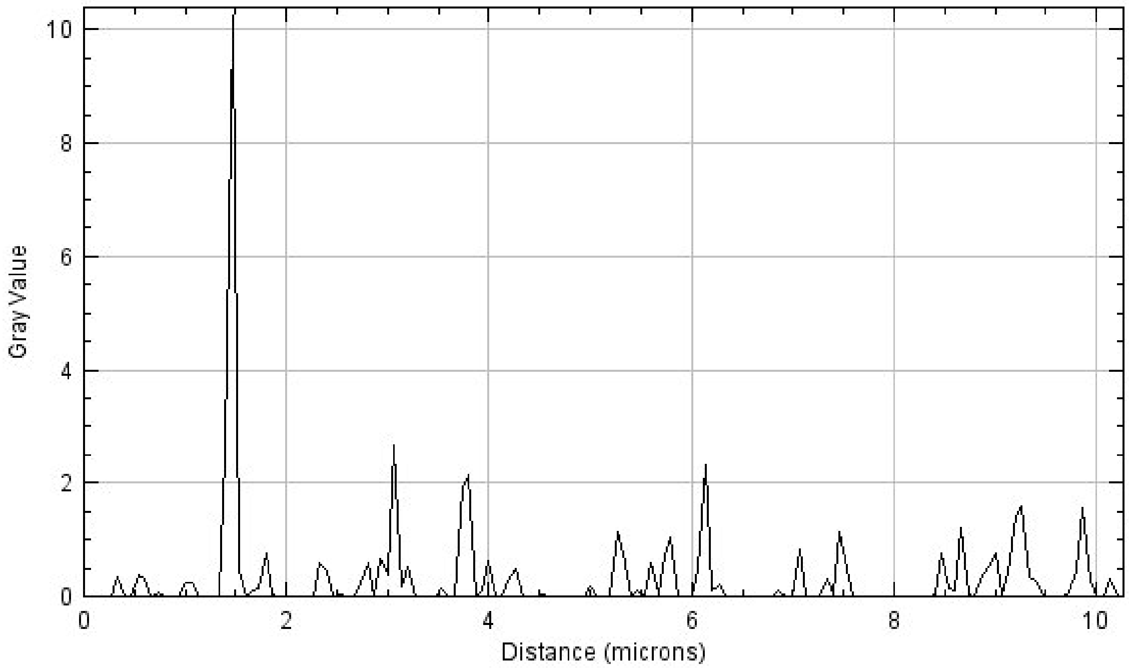

Figure S363 – Intensity profile of line scan generated for cell 11, Figure 352.

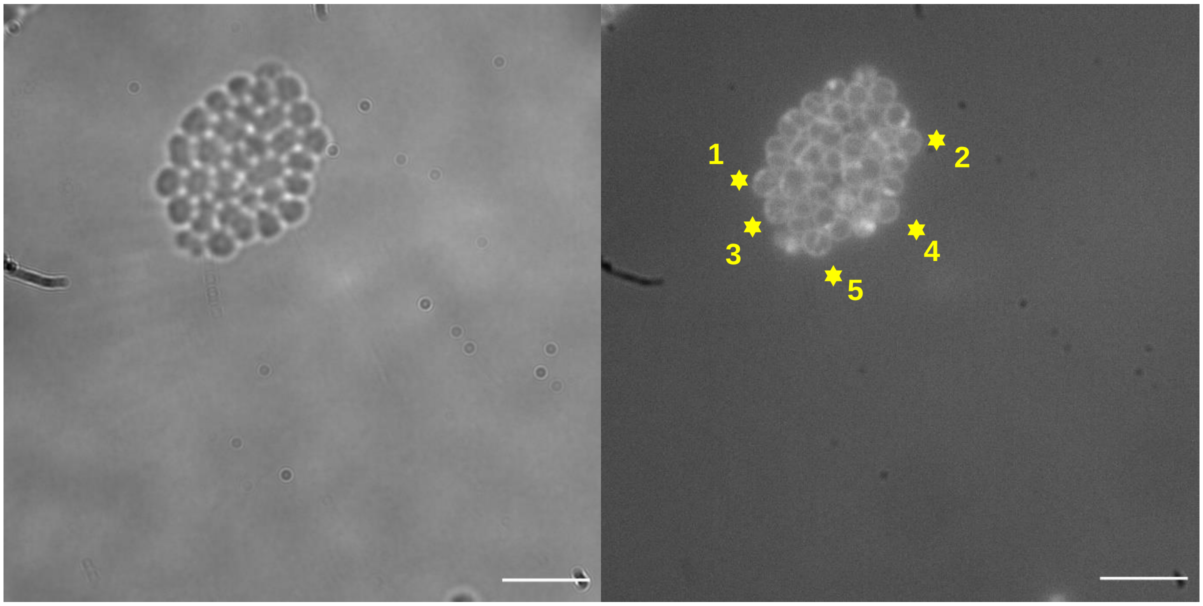

Figure S364 – MRSA transmitted image and fluorescence image at 450 nm used in fluorescence intensity calculations in the presence of compound **39** only at T = 30 minutes. Scale bar = 10μM

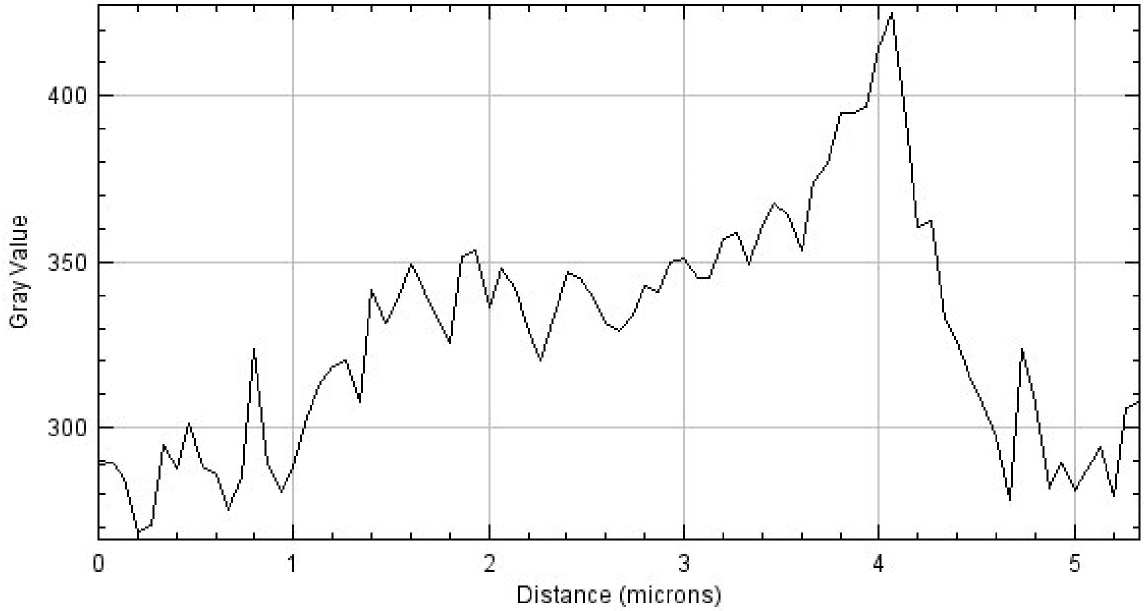

Figure S365 – Intensity profile of line scan generated for cell 1, Figure 352.

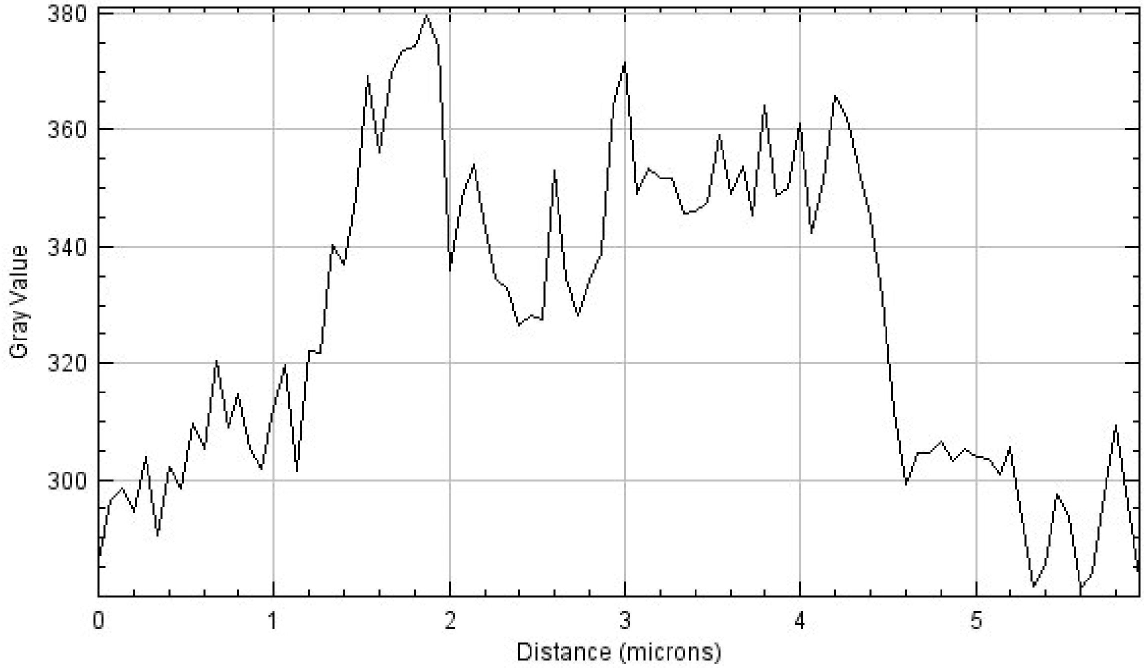

Figure S366 – Intensity profile of line scan generated for cell 2, Figure 352.

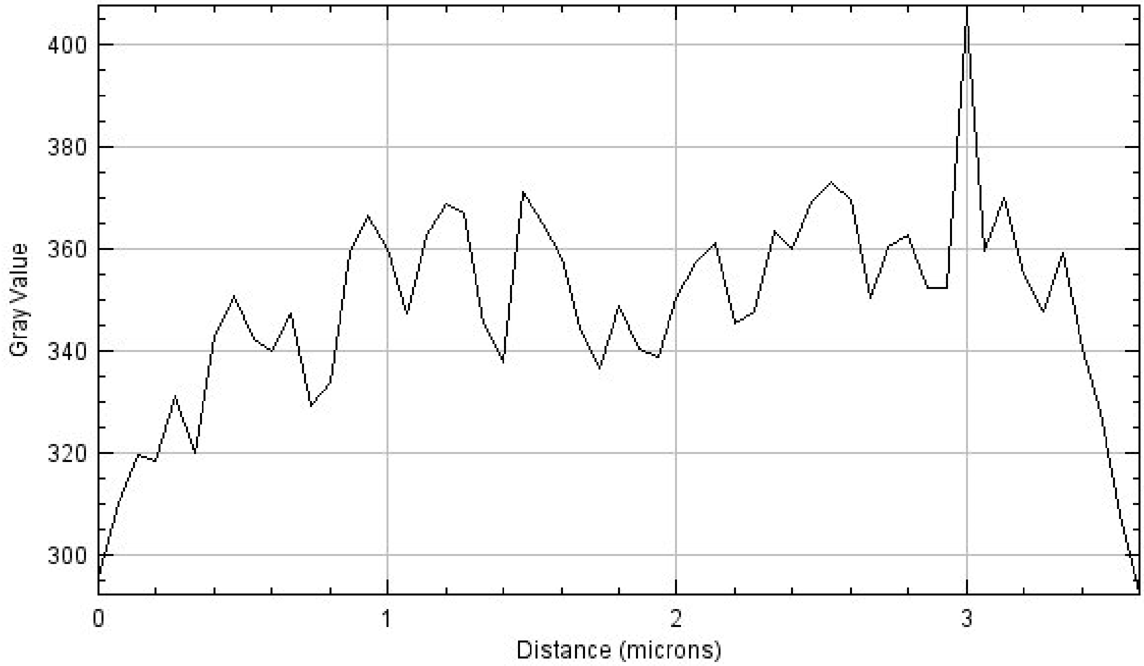

Figure S367 – Intensity profile of line scan generated for cell 3, Figure 352.

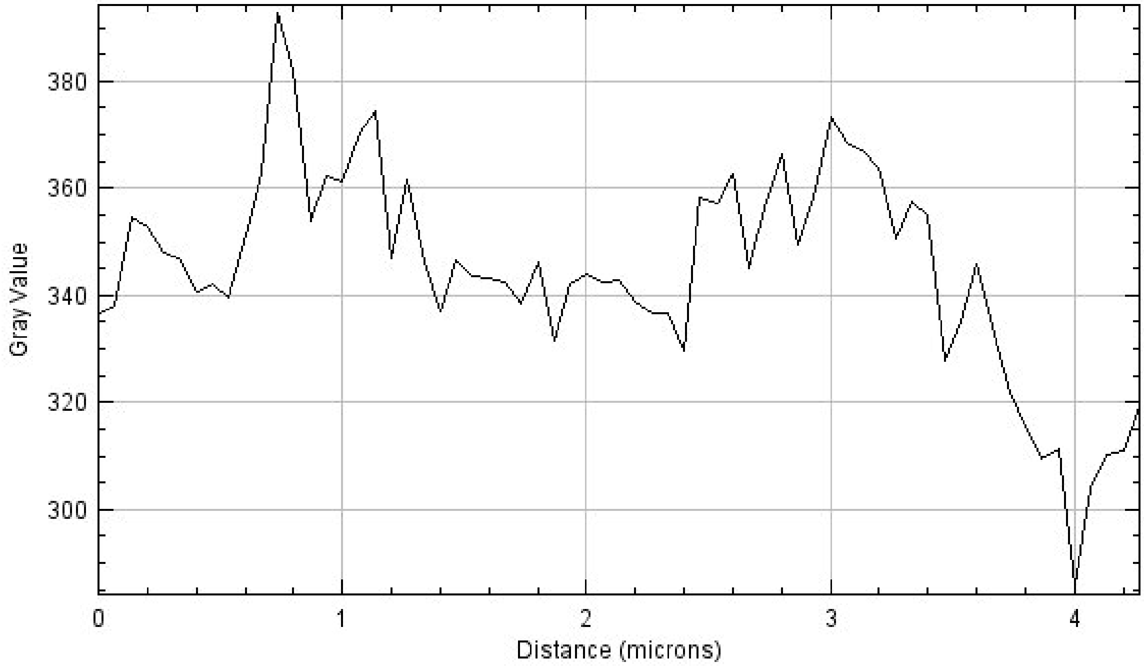

Figure S368 – Intensity profile of line scan generated for cell 4, Figure 352.

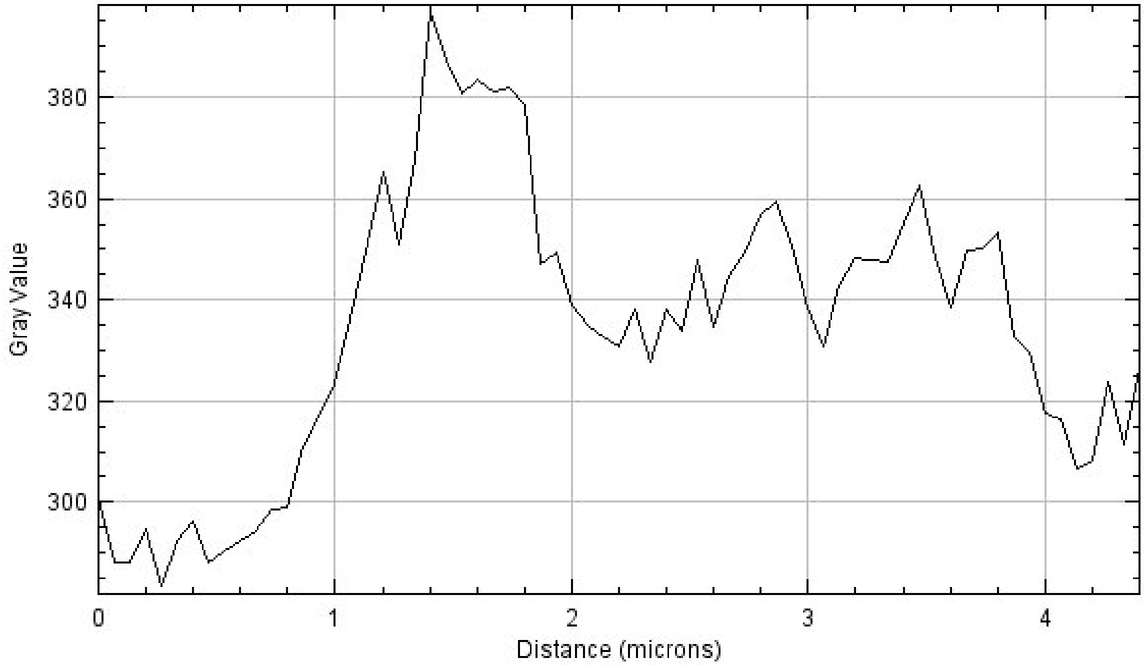

Figure S369 – Intensity profile of line scan generated for cell 5, Figure 352.

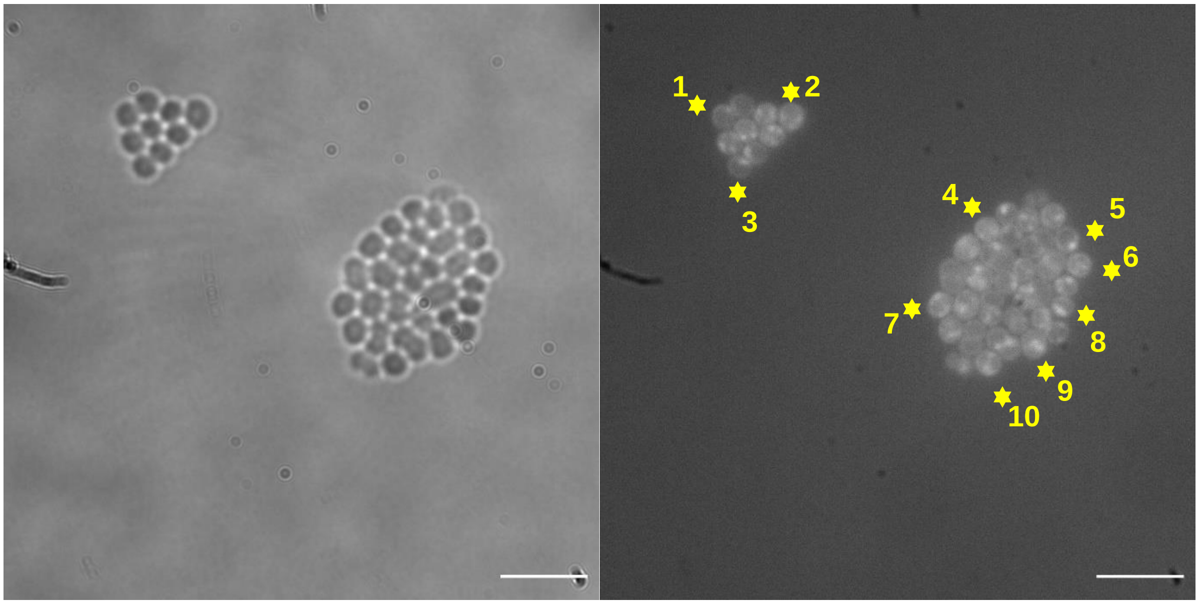

Figure S370 – MRSA transmitted image and fluorescence image at 450 nm used in fluorescence intensity calculations in the presence of compound **39** only at T = 30 minutes. Scale bar = 10 μM.

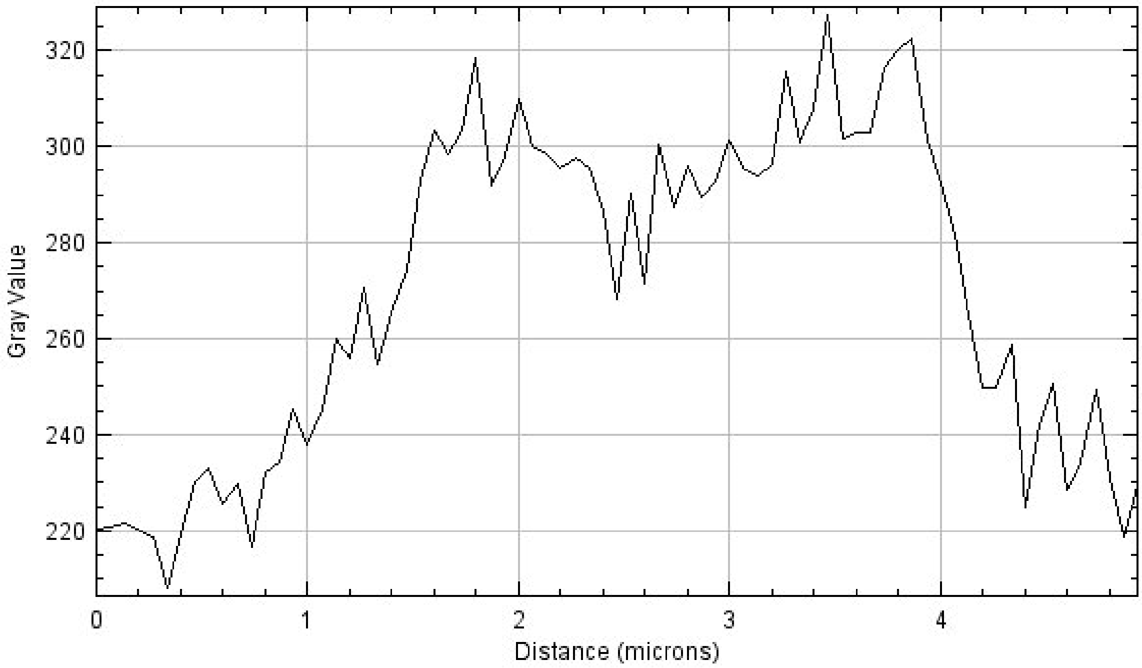

Figure S371 – Intensity profile of line scan generated for cell 1, Figure S370.

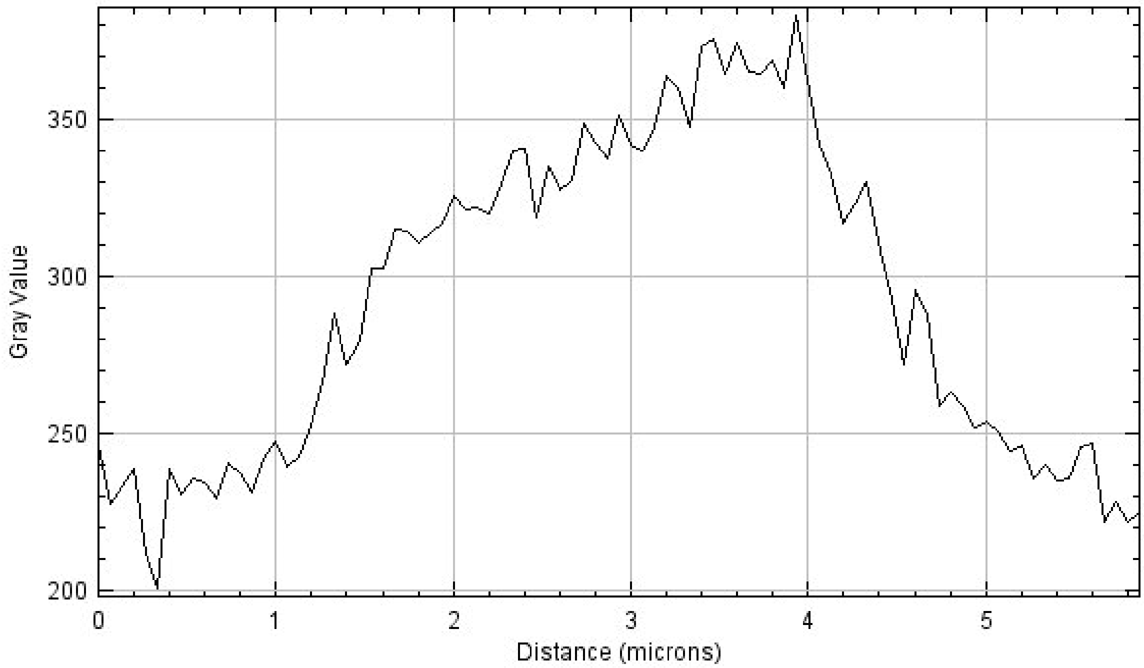

Figure S372 – Intensity profile of line scan generated for cell 2, Figure S370.

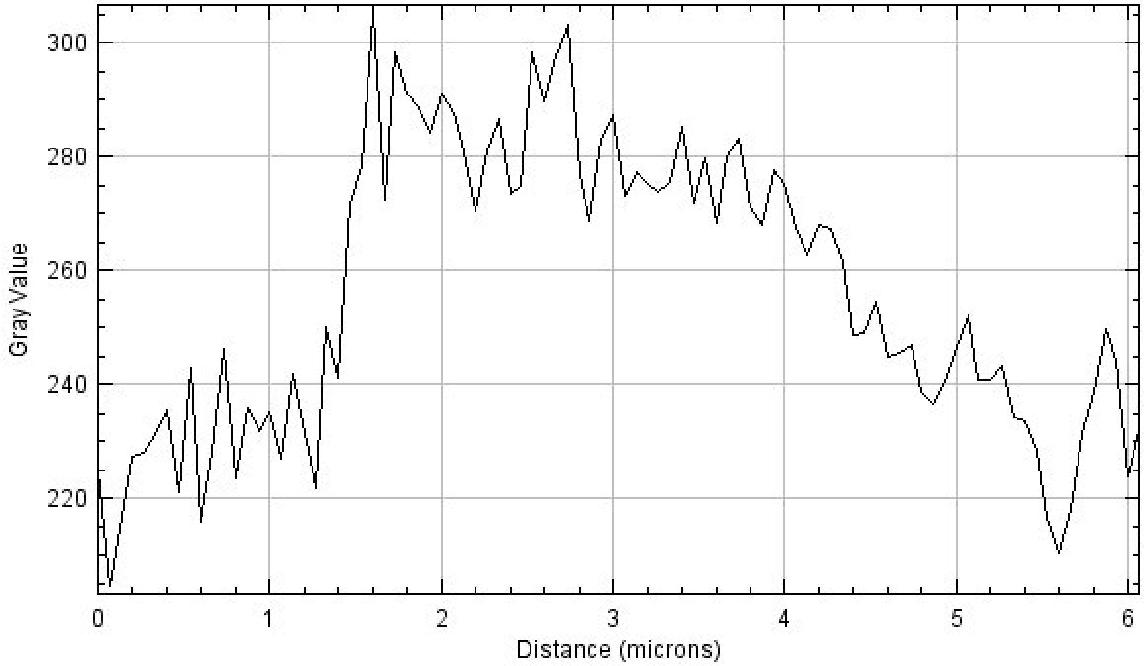

Figure S373 – Intensity profile of line scan generated for cell 3, Figure S370.

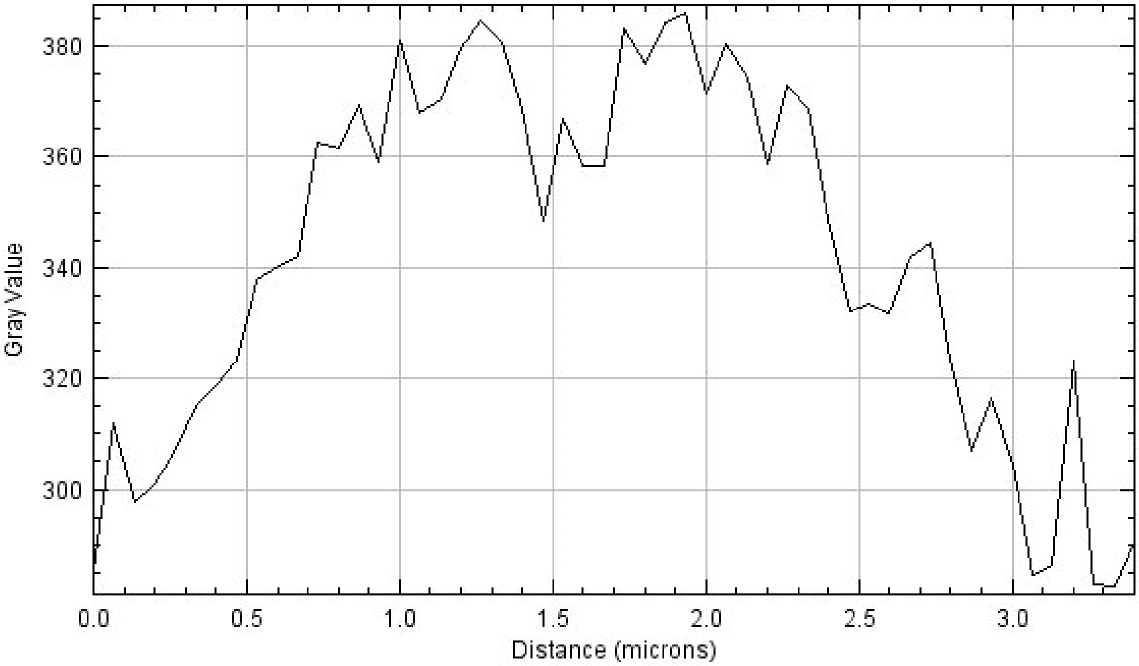

Figure S374 – Intensity profile of line scan generated for cell 4, Figure S370.

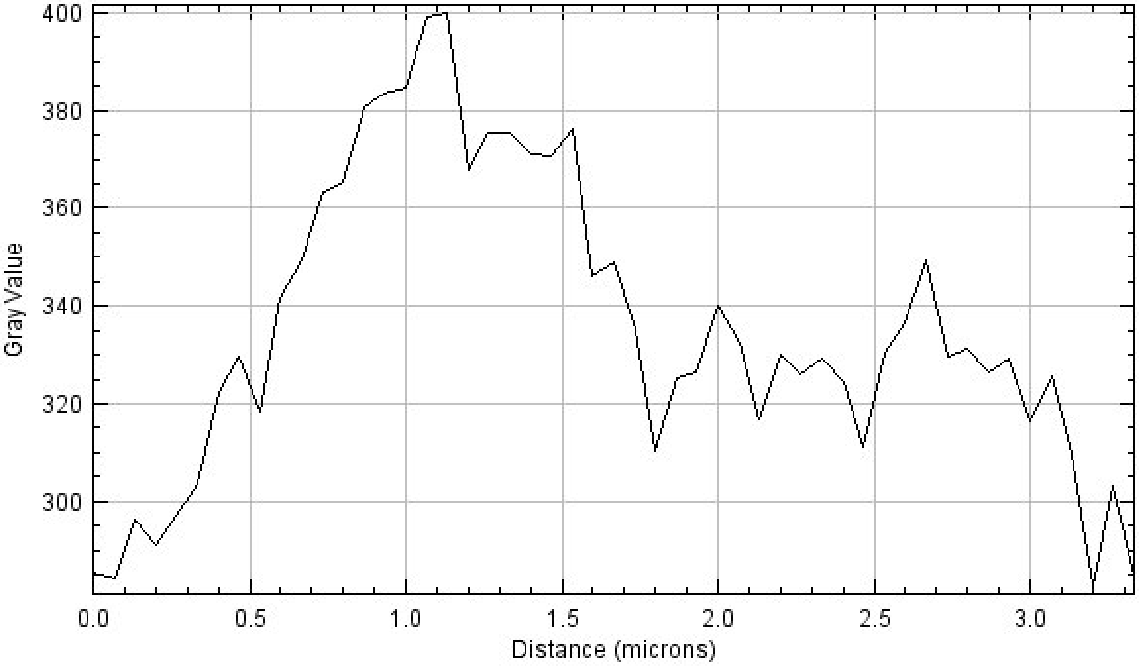

Figure S375 – Intensity profile of line scan generated for cell 5, Figure S370.

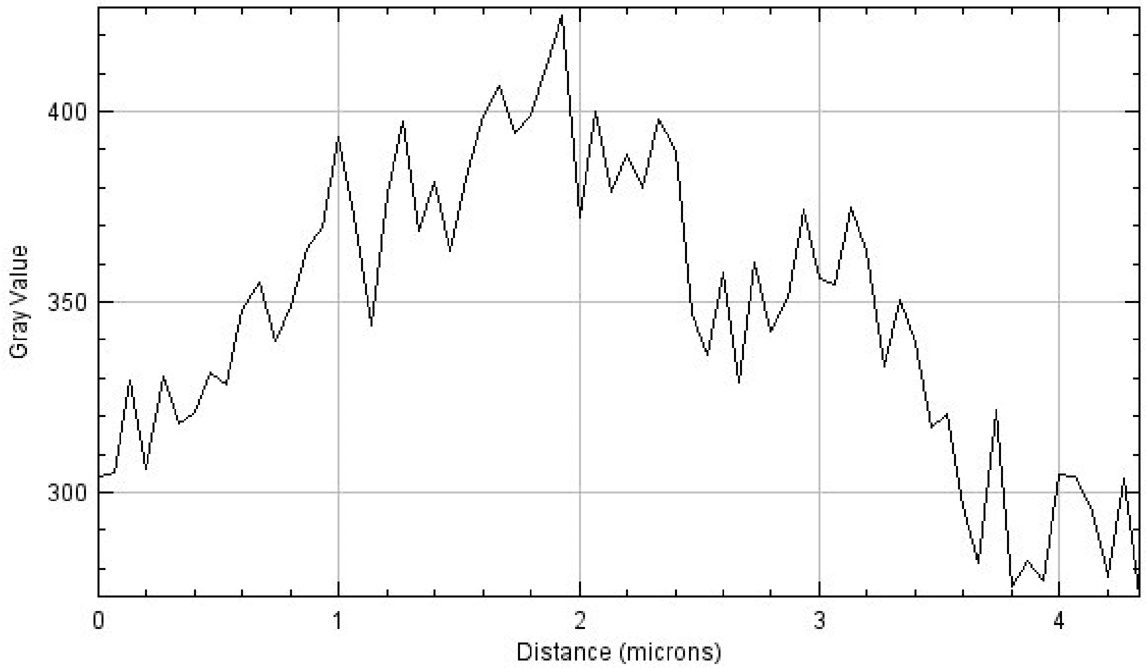

Figure S376 – Intensity profile of line scan generated for cell 6, Figure S370.

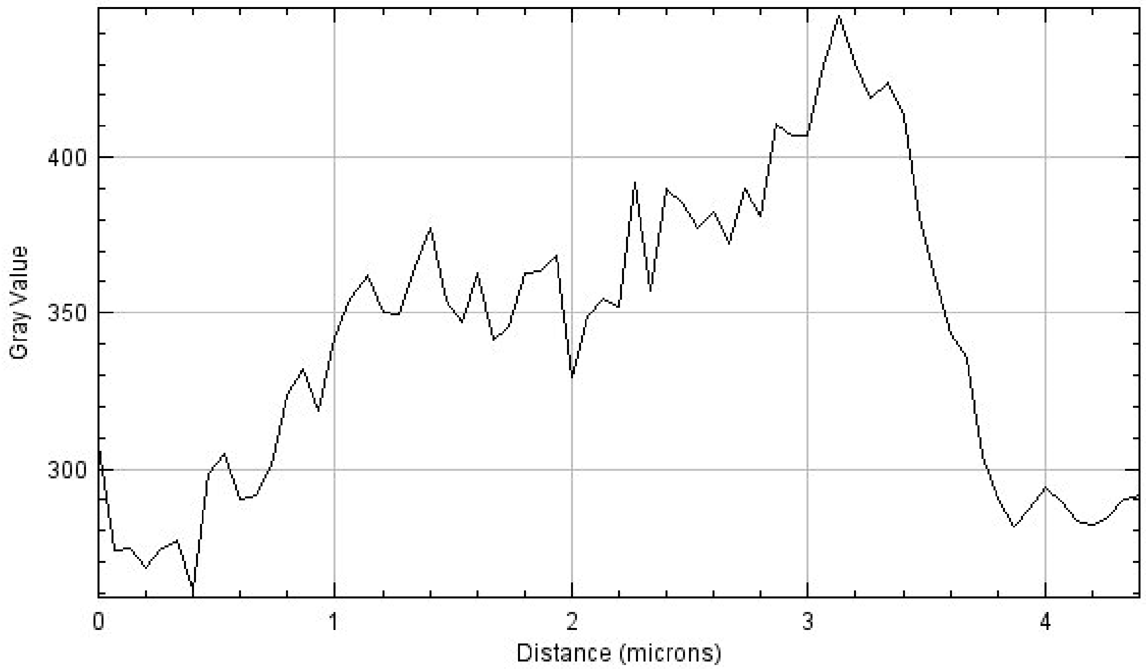

Figure S377 – Intensity profile of line scan generated for cell 7, Figure S370.

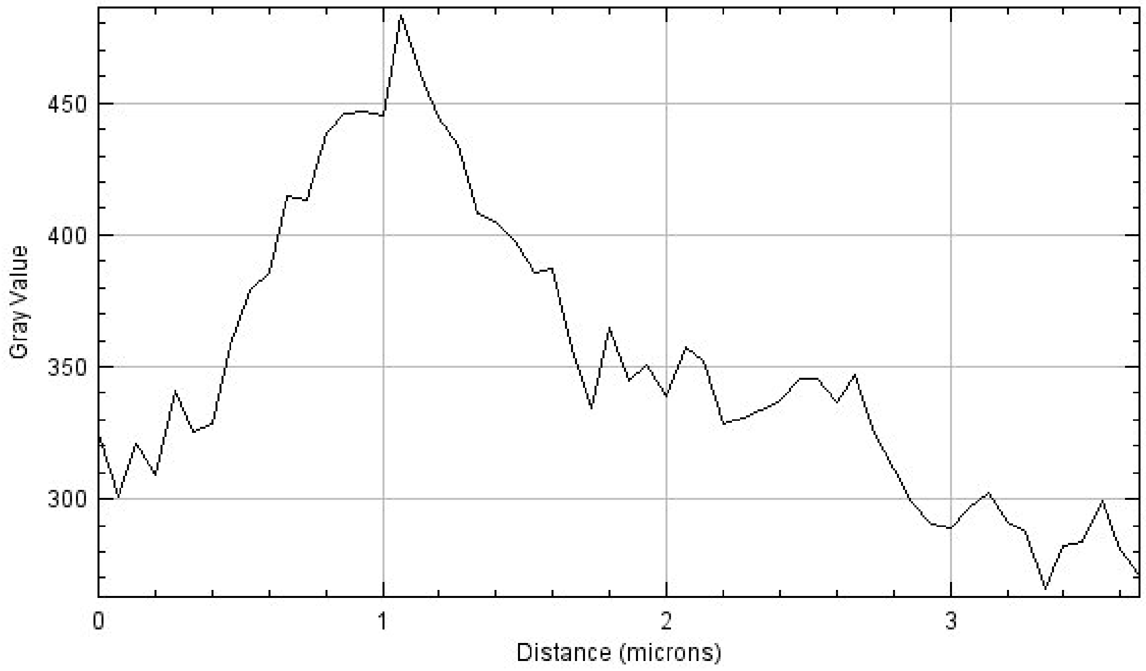

Figure S378 – Intensity profile of line scan generated for cell 8, Figure S370.

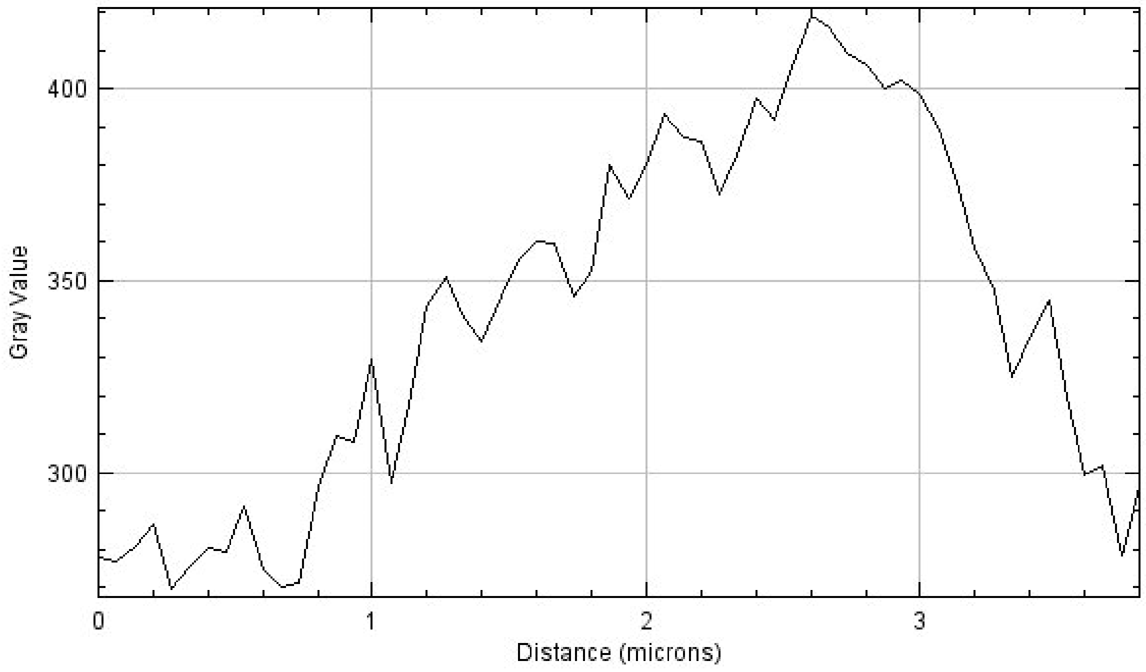

Figure S379 – Intensity profile of line scan generated for cell 9, Figure S370.

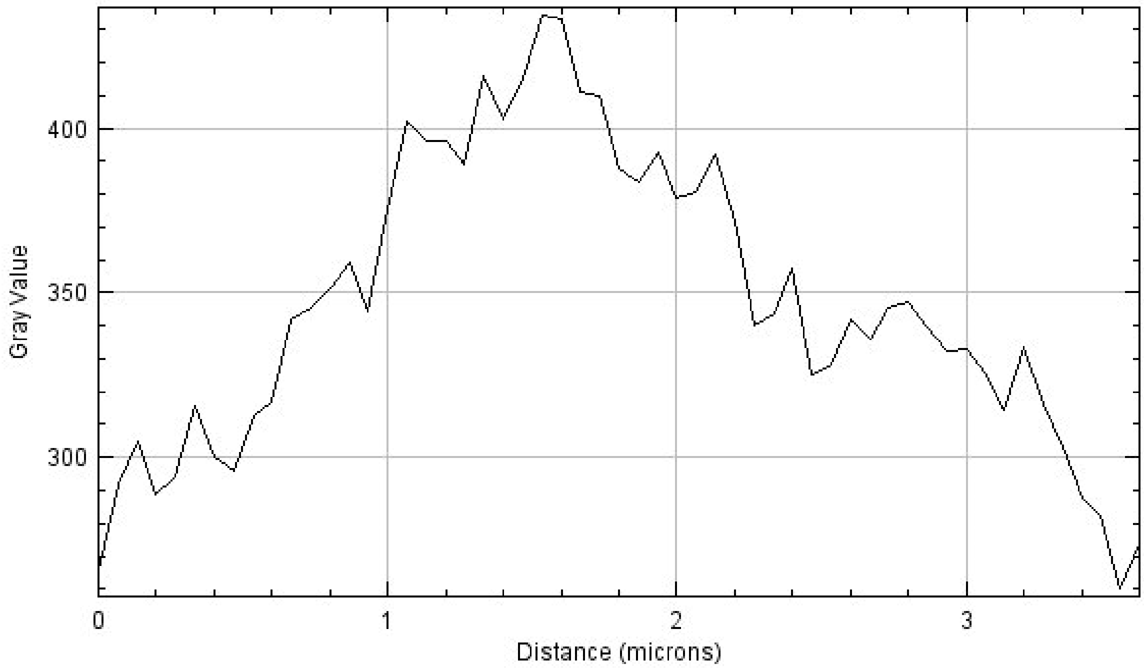

Figure S380 – Intensity profile of line scan generated for cell 10, Figure S370.

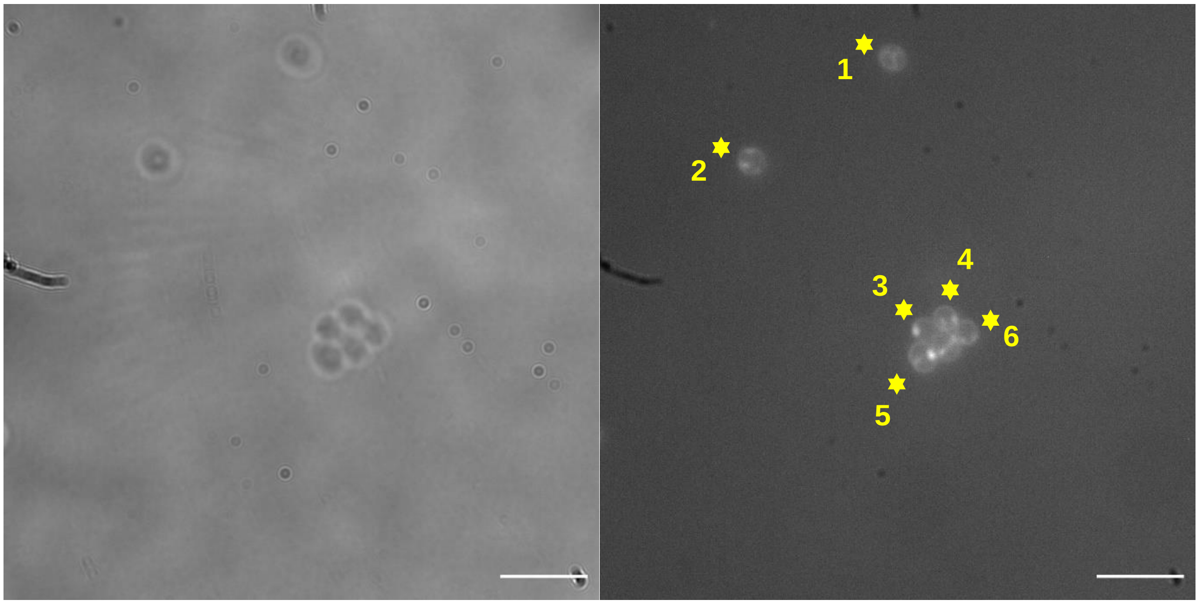

Figure S381 – MRSA transmitted image and fluorescence image at 450 nm used in fluorescence intensity calculations in the presence of compound **39** only at T = 30 minutes. Scale bar = 10 µM.

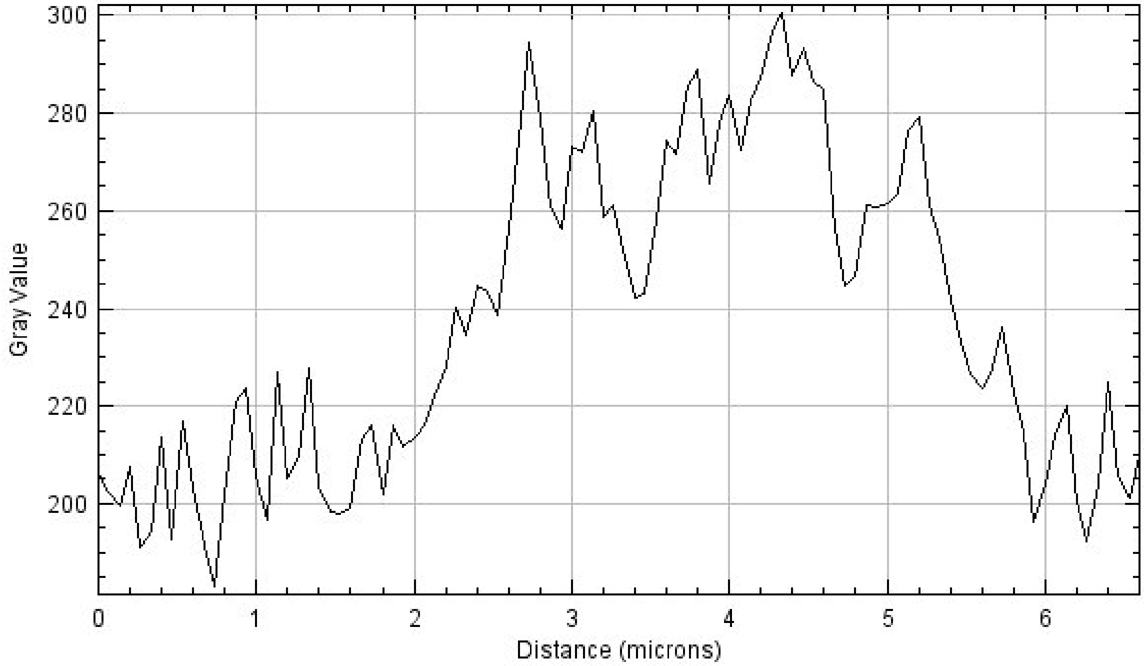

Figure S382 – Intensity profile of line scan generated for cell 1, Figure 381.

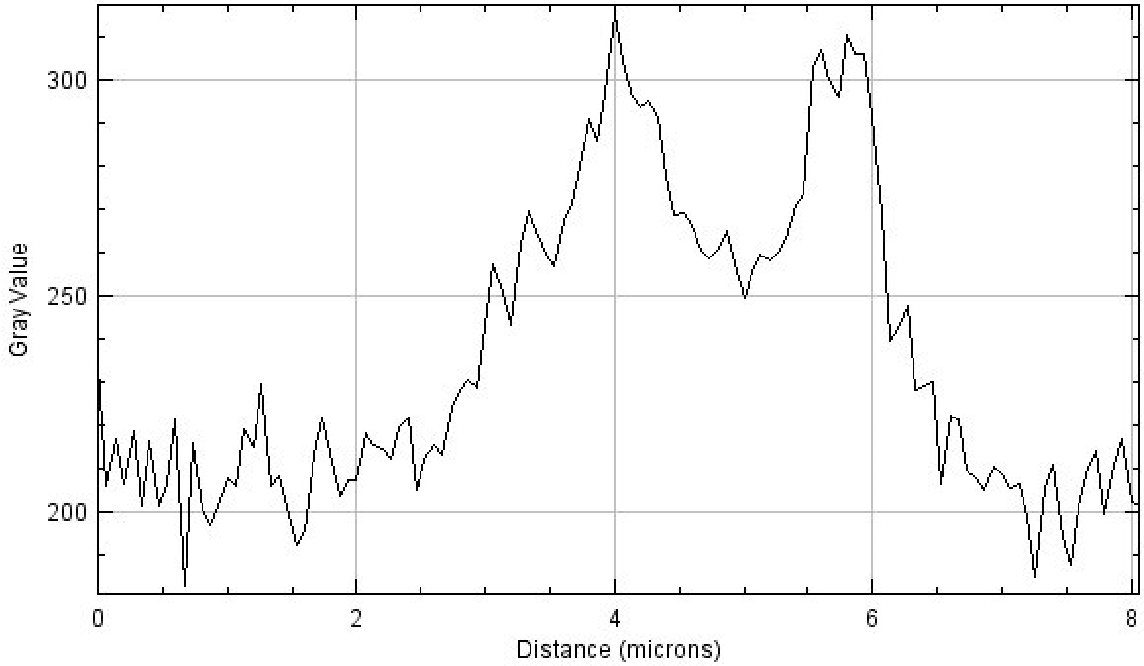

Figure S383 – Intensity profile of line scan generated for cell 2, Figure 381.

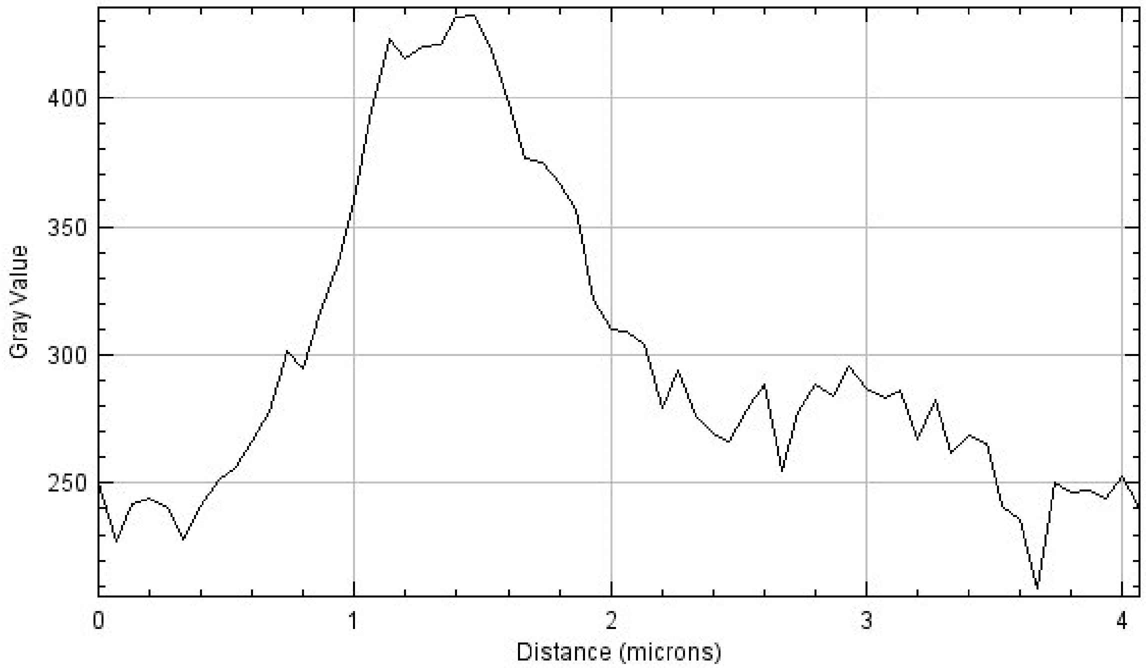

Figure S384 – Intensity profile of line scan generated for cell 3, Figure 381.

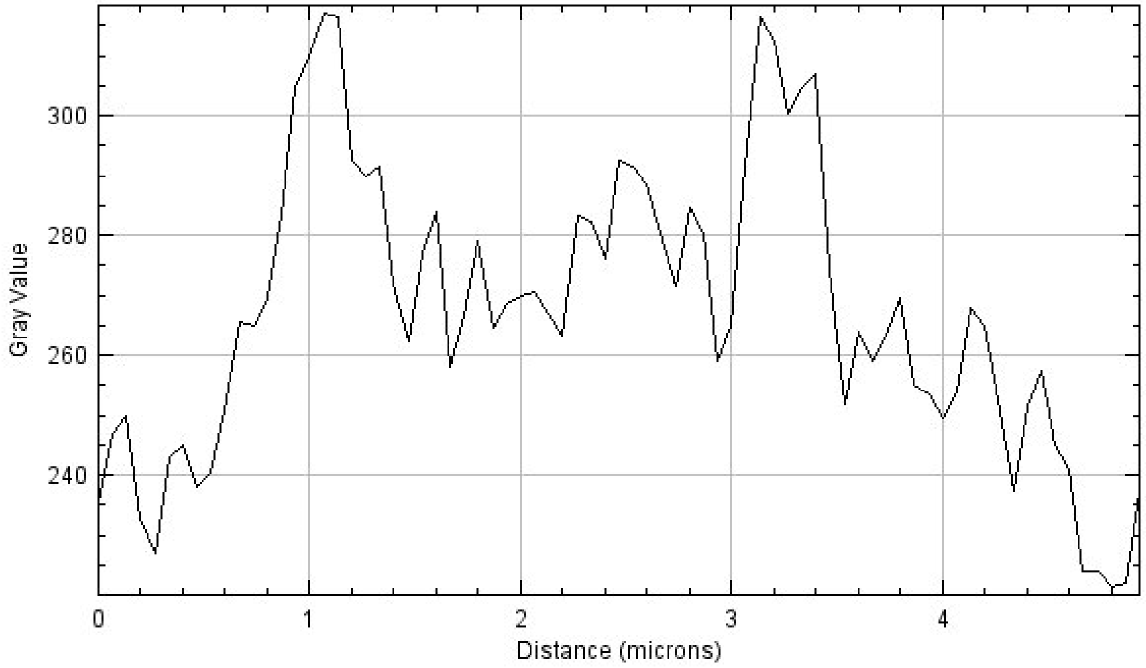

Figure S385 – Intensity profile of line scan generated for cell 4, Figure 381.

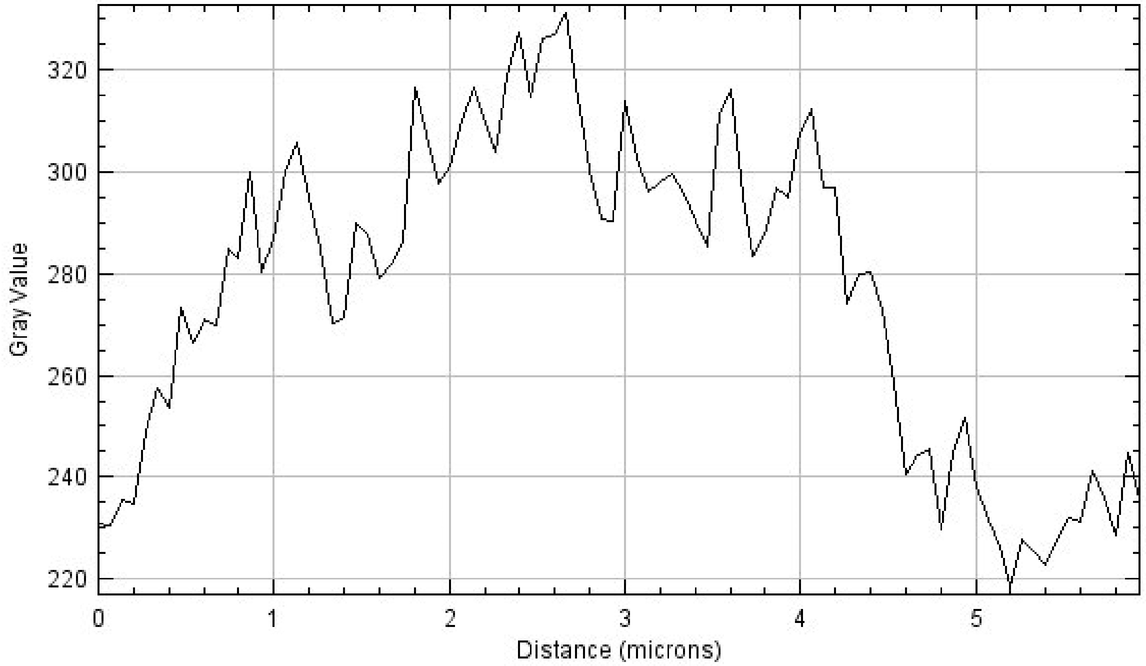

Figure S386 – Intensity profile of line scan generated for cell 5, Figure 381.

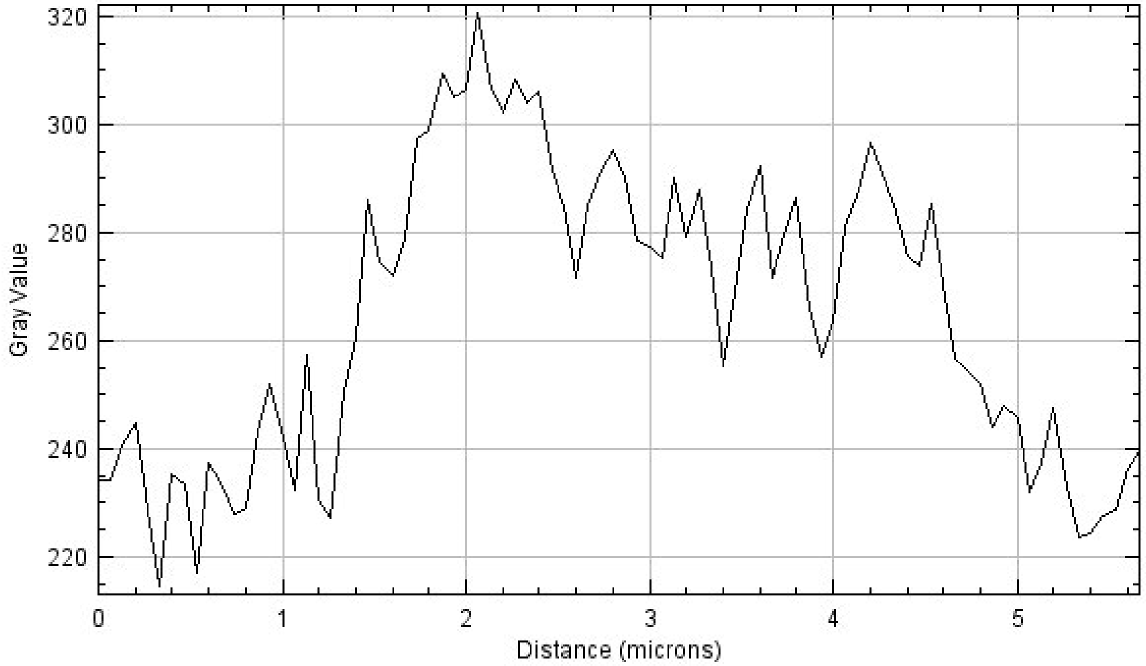

Figure S387 – Intensity profile of line scan generated for cell 6, Figure 381.

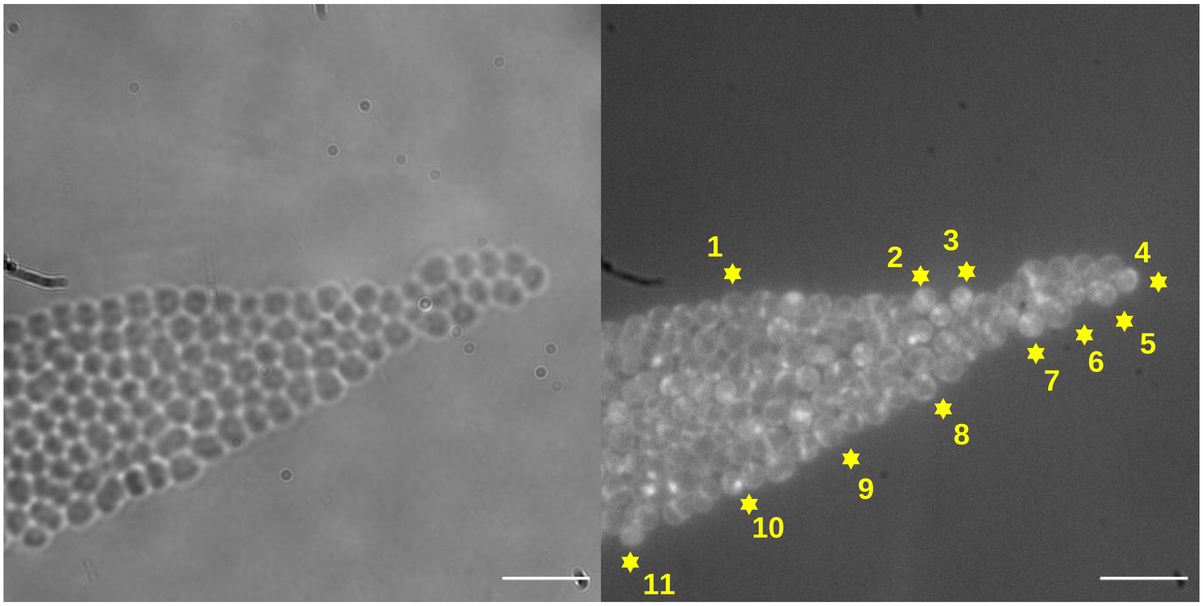

Figure S388 – MRSA transmitted image and fluorescence image at 450 nm used in fluorescence intensity calculations in the presence of compound **39** only at T = 30 minutes. Scale bar = 10 µM.

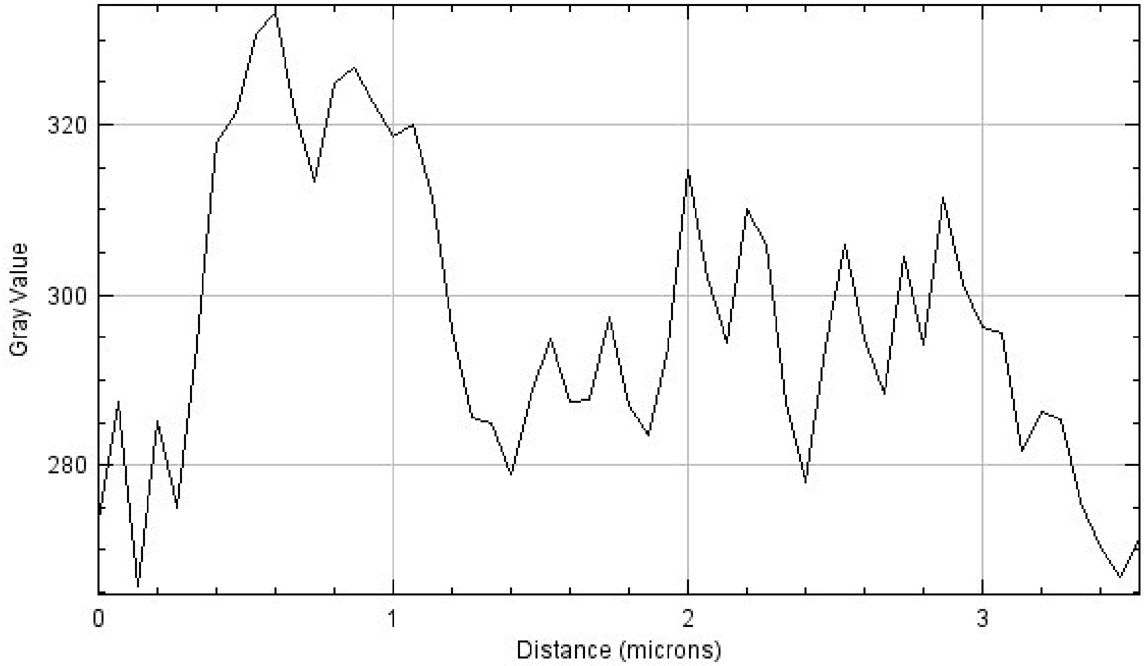

Figure S389 – Intensity profile of line scan generated for cell 1, Figure S388.

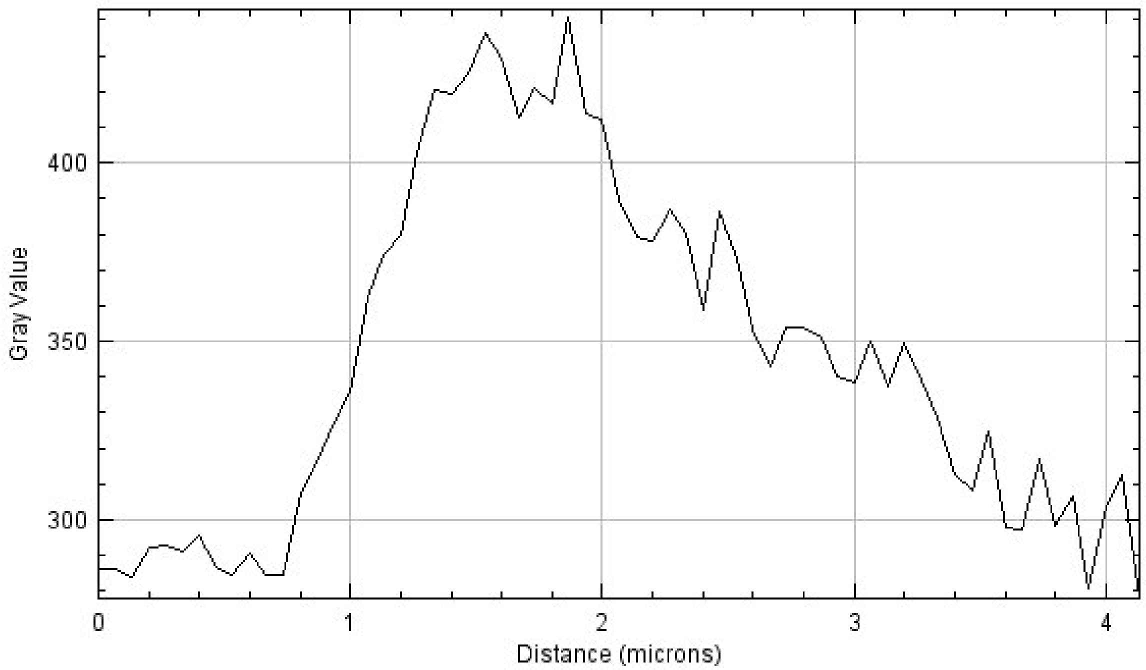

Figure S390 – Intensity profile of line scan generated for cell 2, Figure S388.

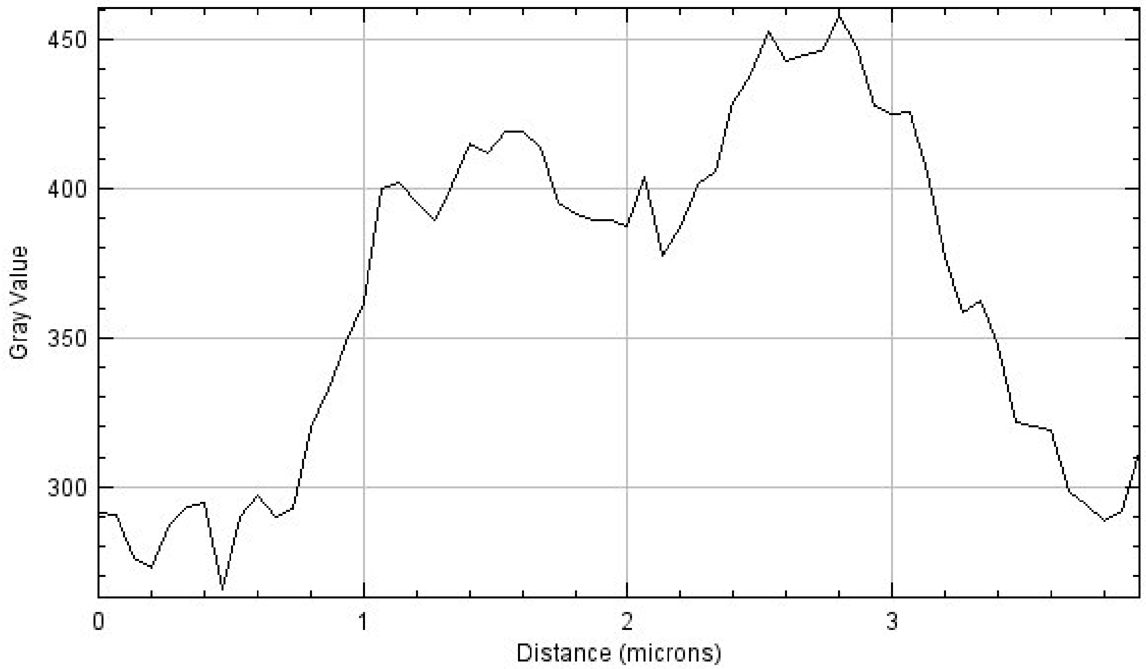

Figure S391 – Intensity profile of line scan generated for cell 3, Figure S388.

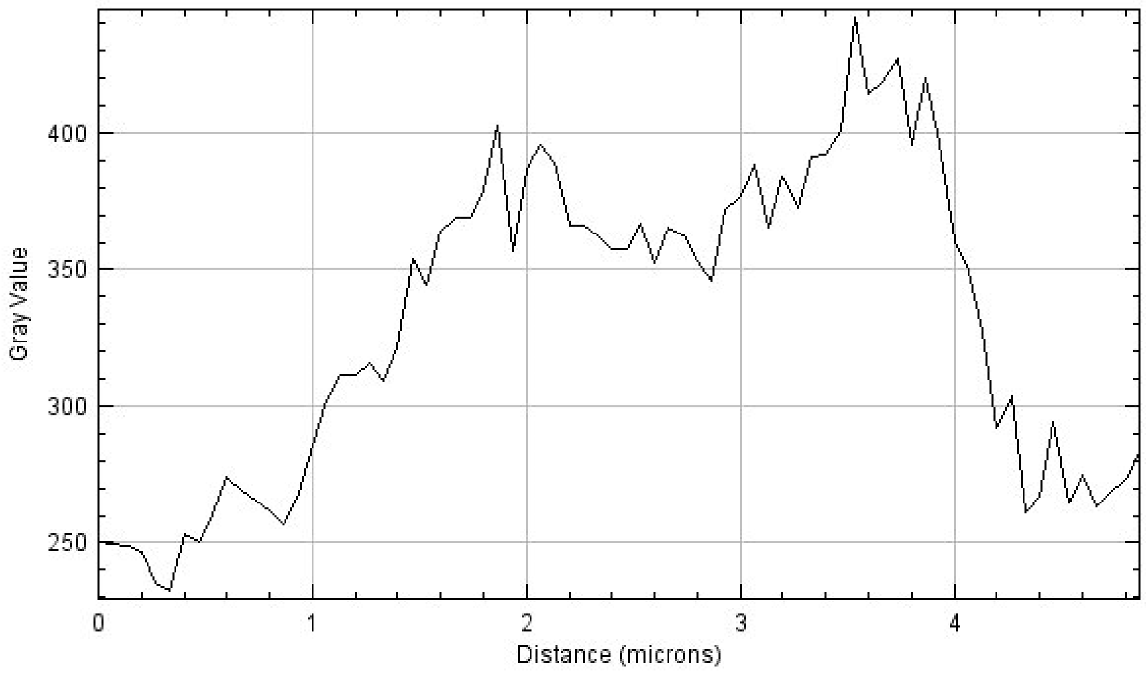

Figure S392 – Intensity profile of line scan generated for cell 4, Figure S388.

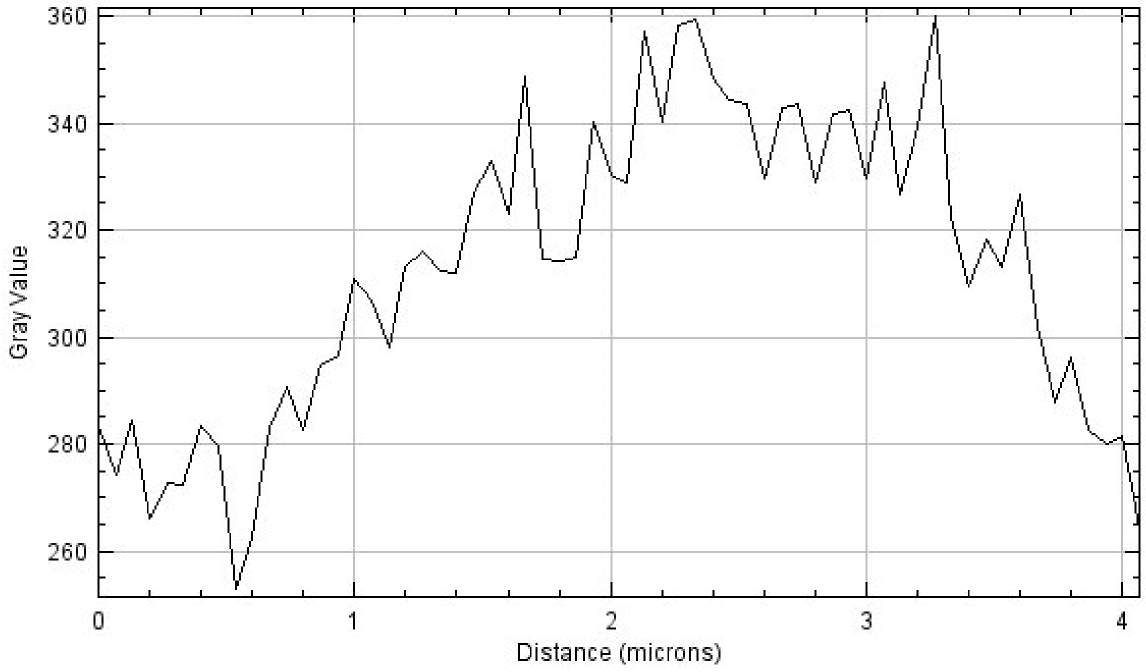

Figure S393 – Intensity profile of line scan generated for cell 5, Figure S388.

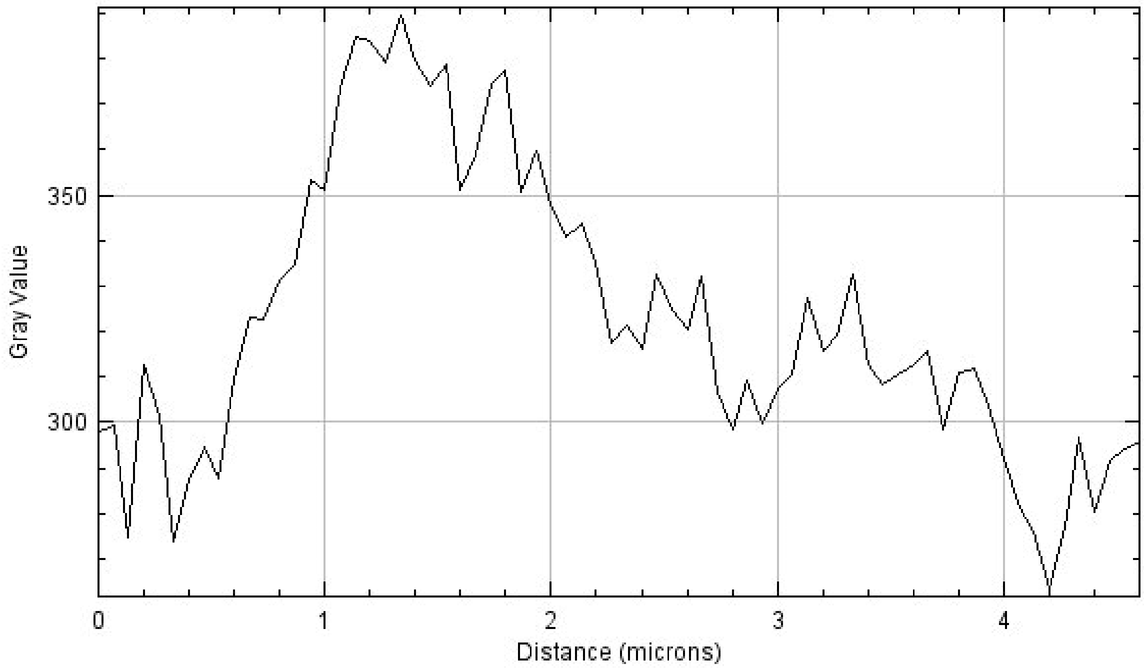

Figure S394 – Intensity profile of line scan generated for cell 6, Figure S388.

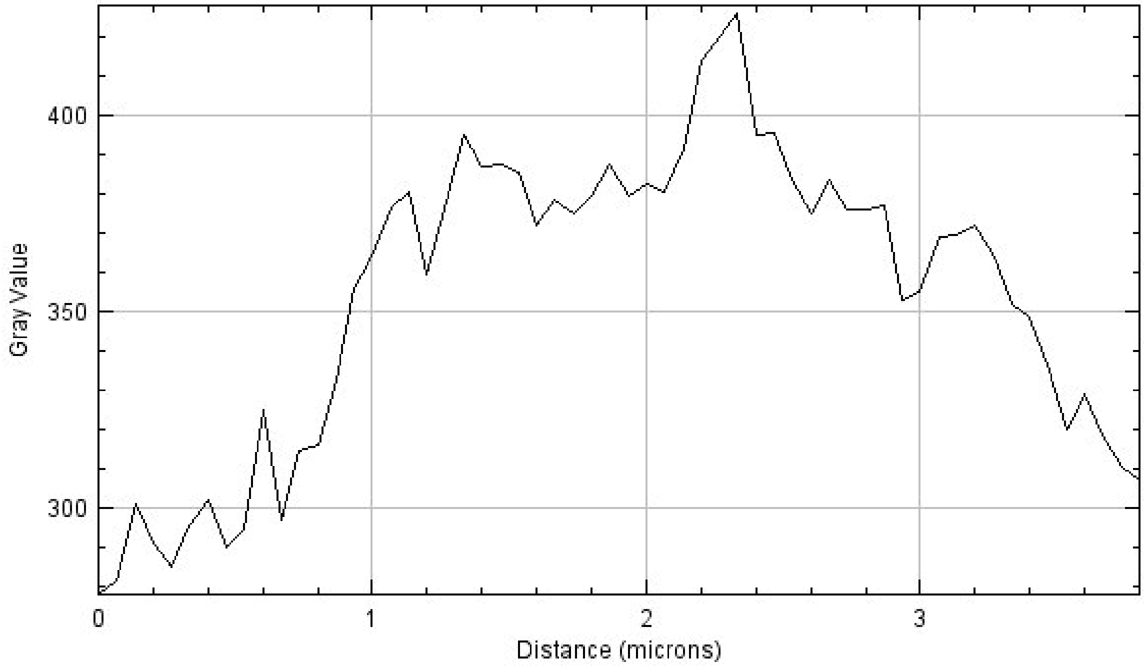

Figure S395 – Intensity profile of line scan generated for cell 7, Figure S388.

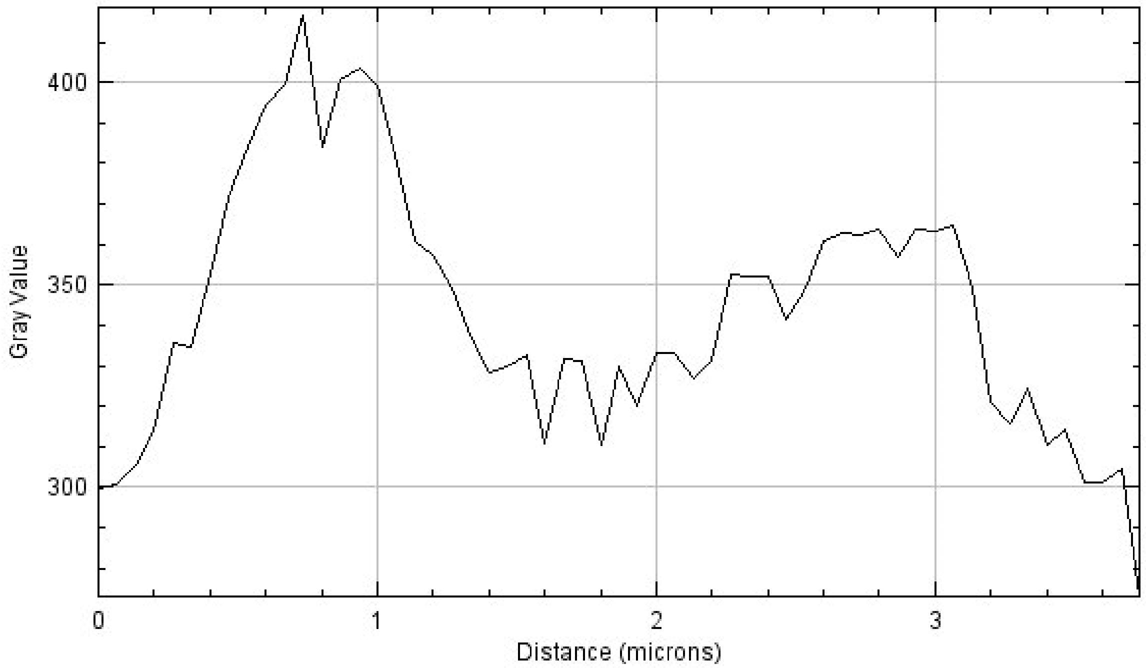

Figure S396 – Intensity profile of line scan generated for cell 8, Figure S388.

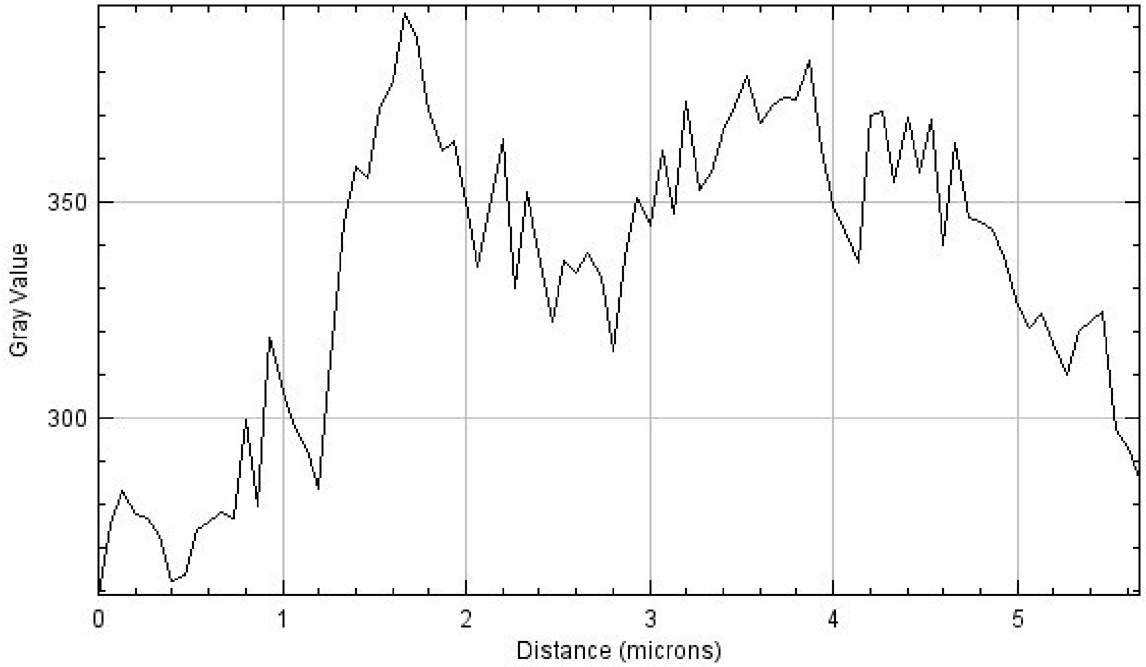

Figure S397 – Intensity profile of line scan generated for cell 9, Figure S388.

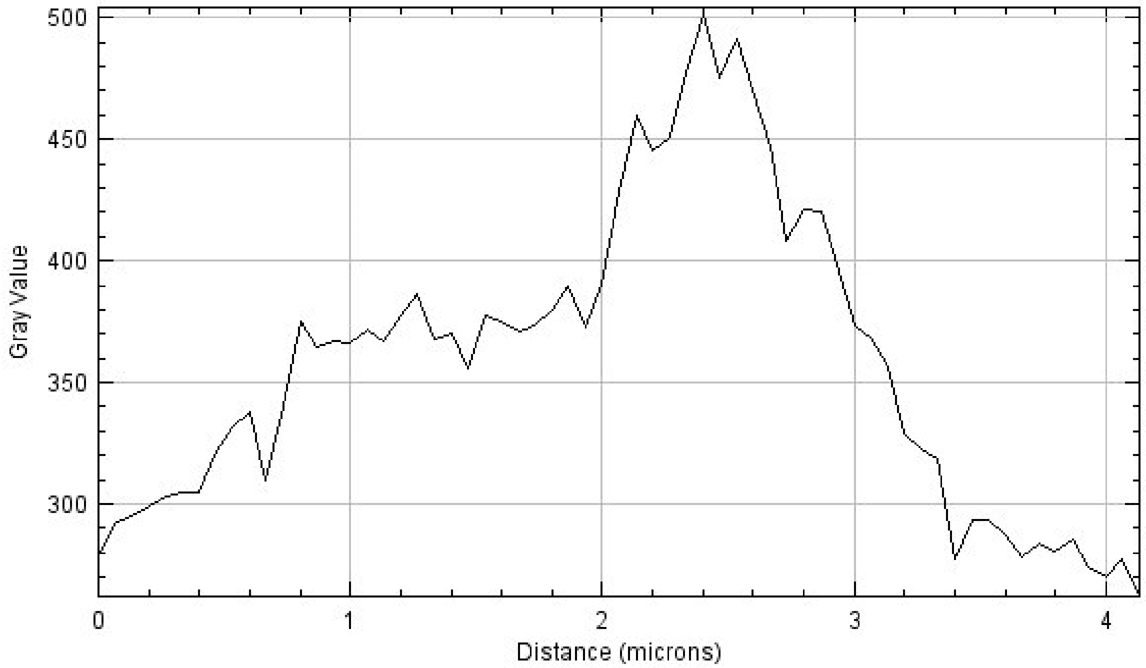

Figure S398 – Intensity profile of line scan generated for cell 10, Figure S388.

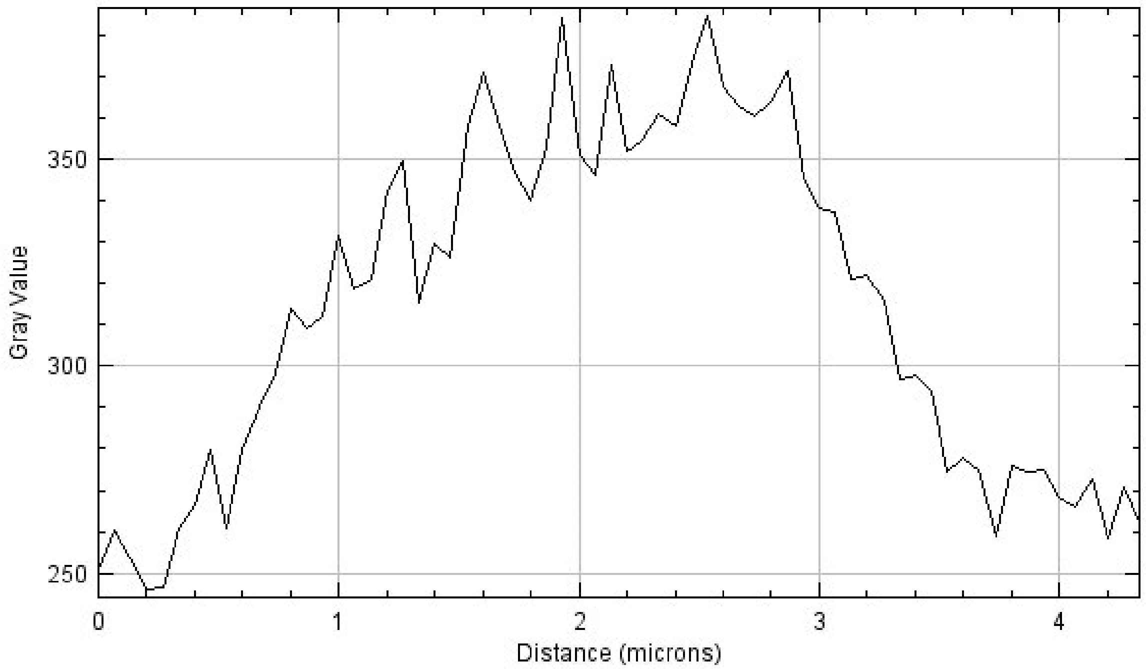

Figure S399 – Intensity profile of line scan generated for cell 11, Figure S388.

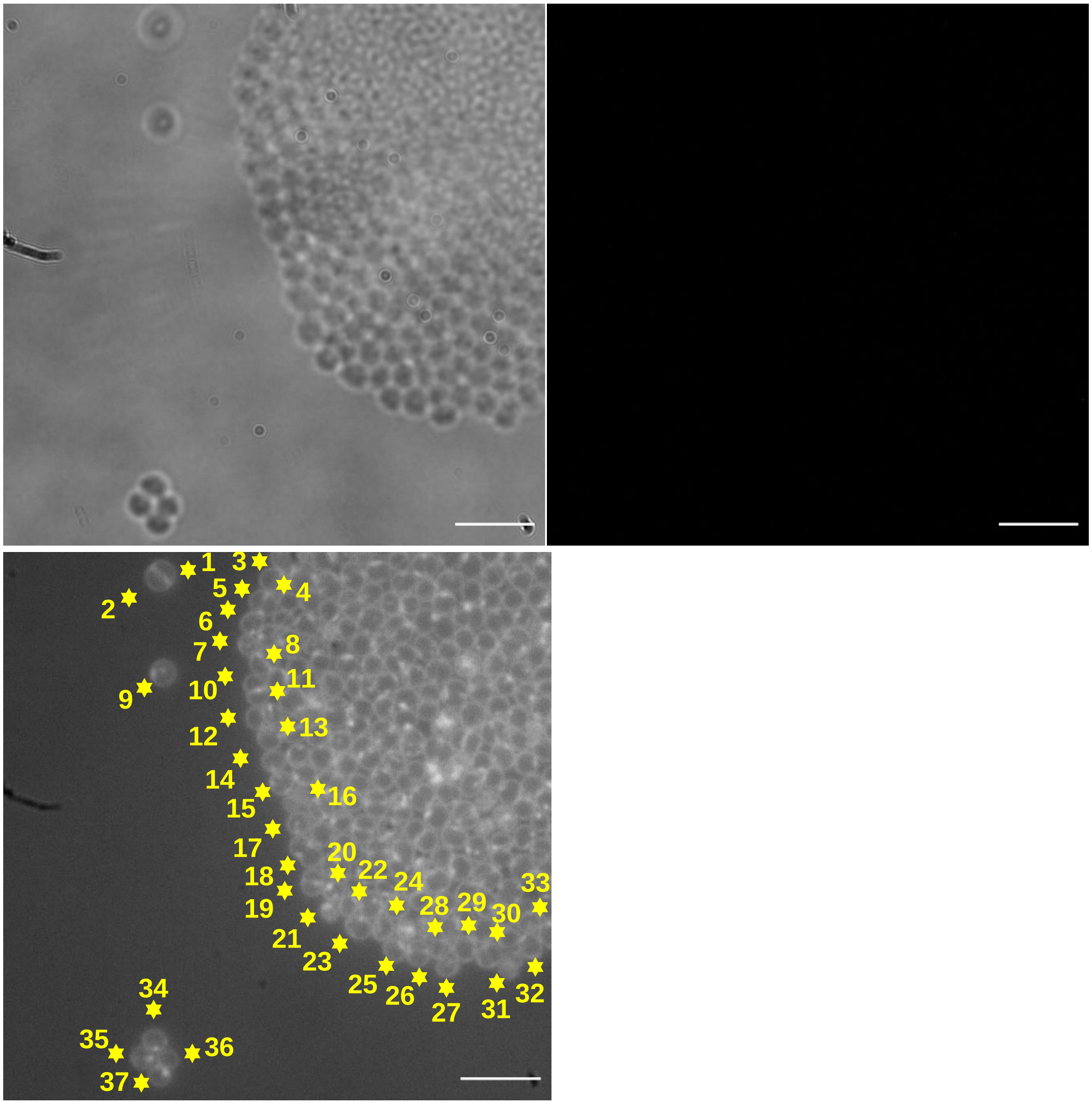

Figure S400 – MRSA transmitted image and fluorescence image at 605 nm used in fluorescence intensity calculations in the presence of compound **39** only at T = 30 minutes. Fluorescence image at 450 nm used to locate cells on fluorescence image at 605 nm. Scale bar = 10 μM.

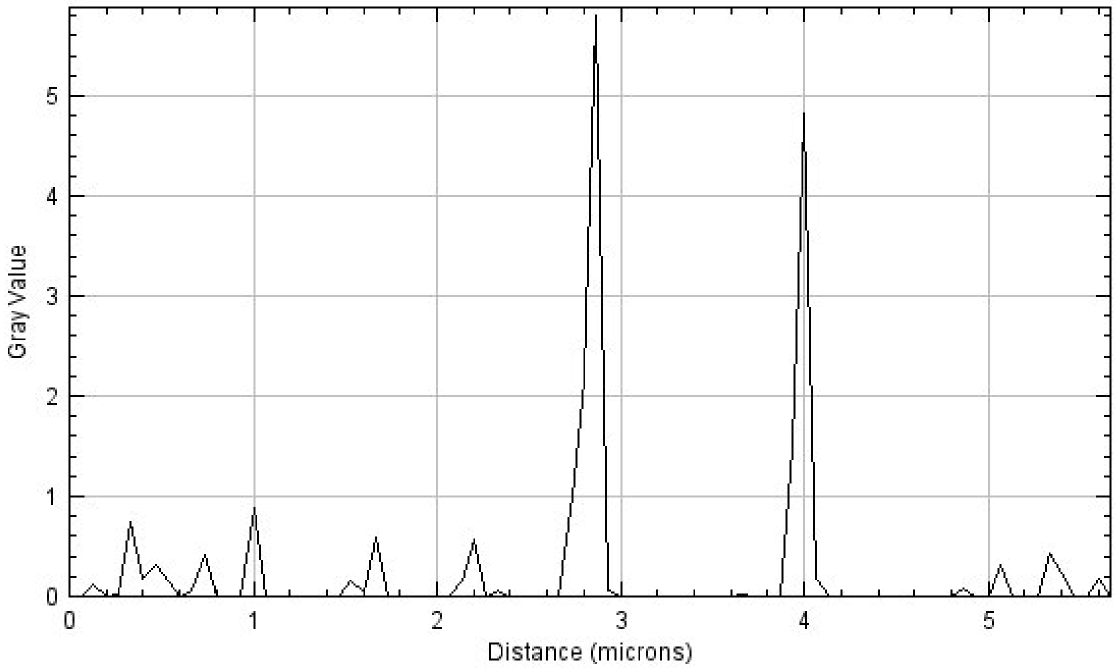

Figure S401 – Intensity profile of line scan generated for cell 1, Figure S400.

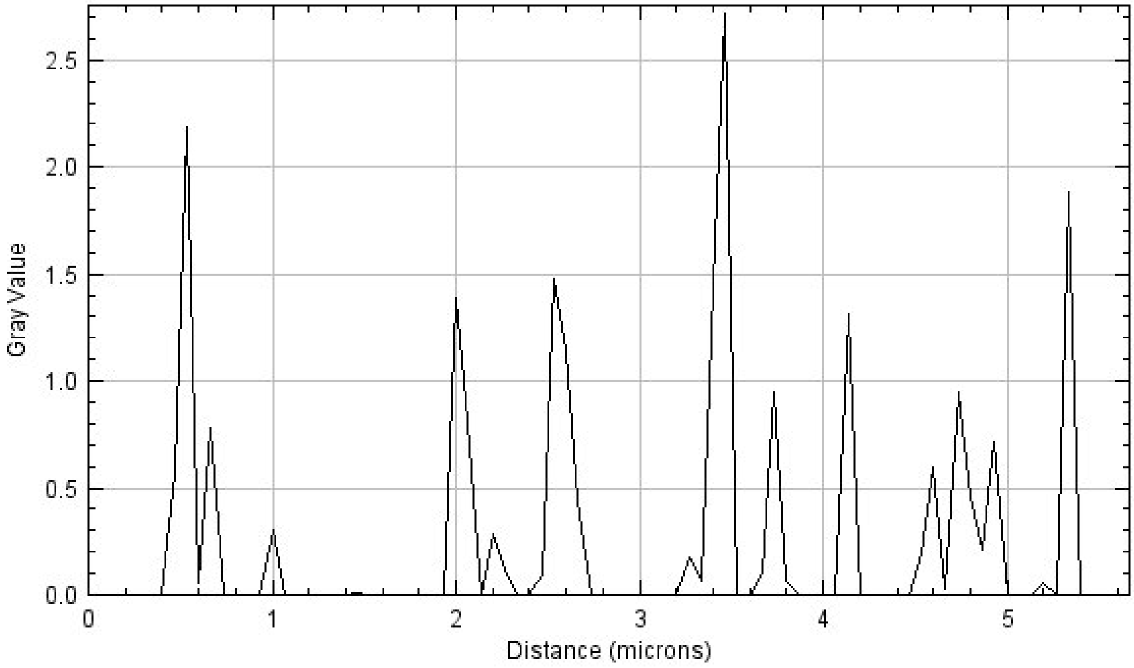

Figure S402 – Intensity profile of line scan generated for cell 2, Figure S400.

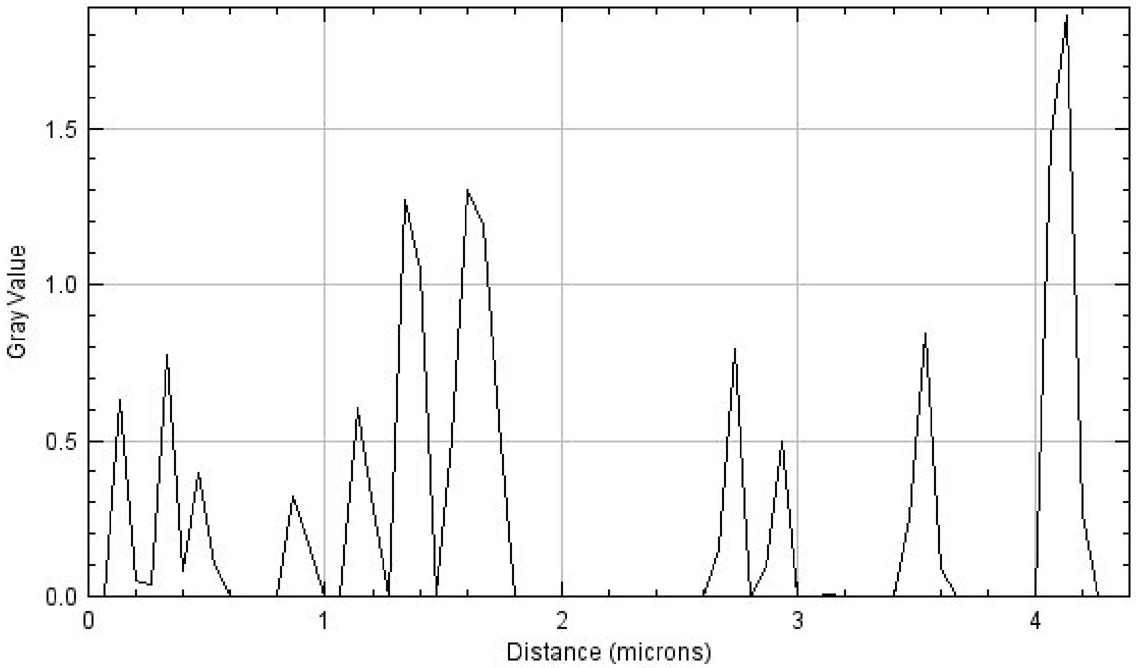

Figure S403 – Intensity profile of line scan generated for cell 3, Figure S400.

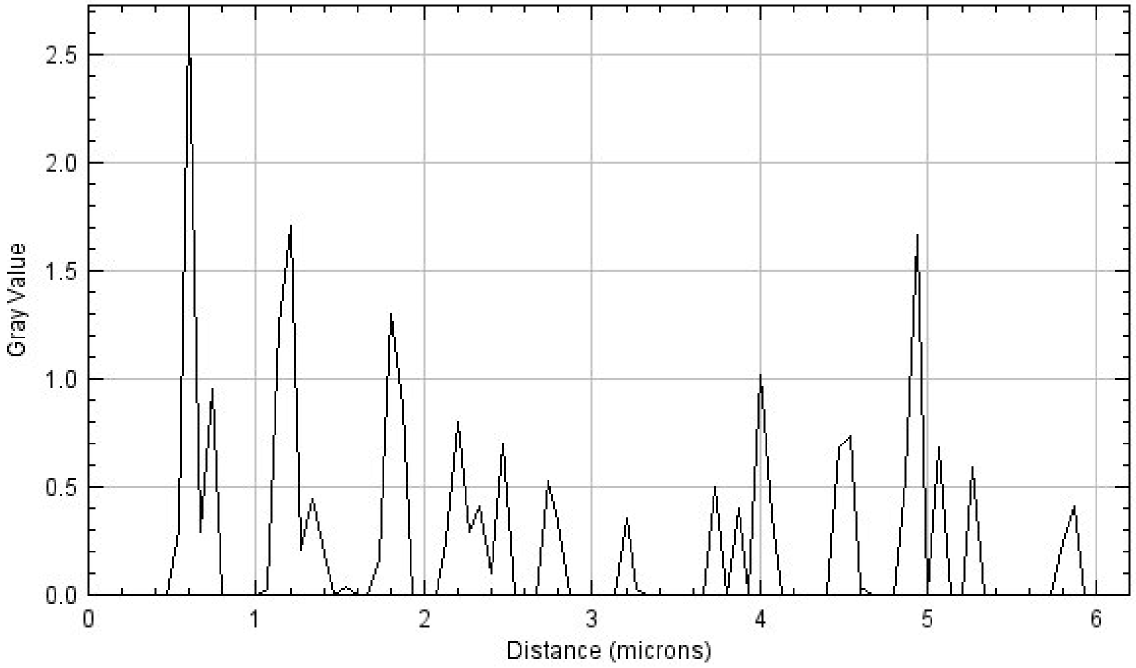

Figure S404 – Intensity profile of line scan generated for cell 4, Figure S400.

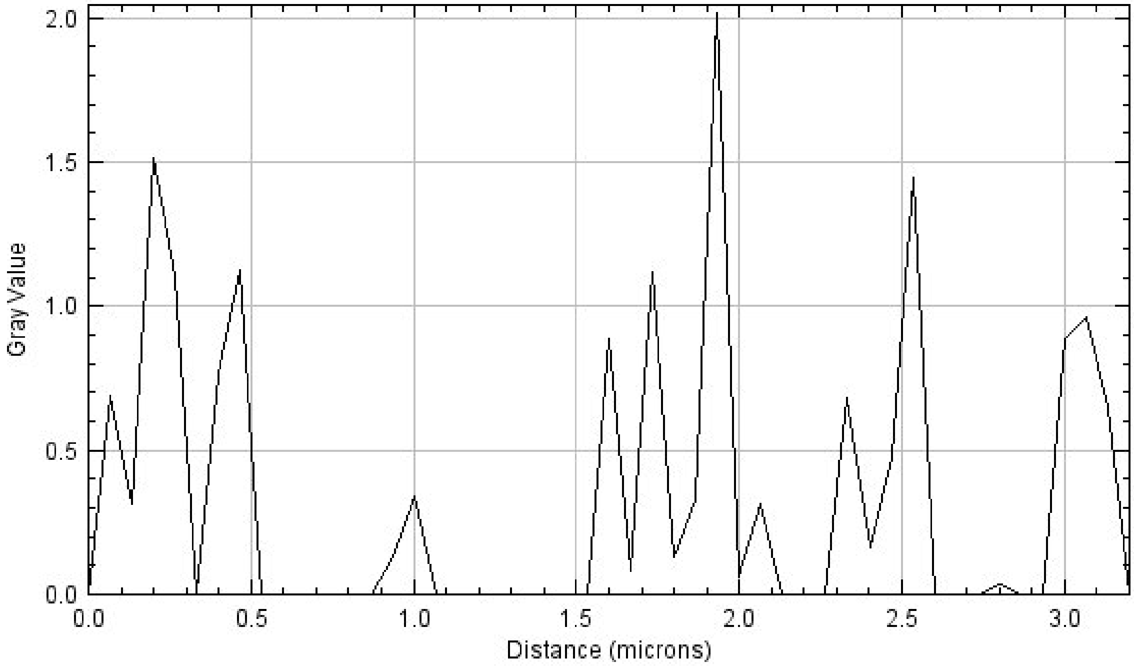

Figure S405 – Intensity profile of line scan generated for cell 5 , Figure S400.

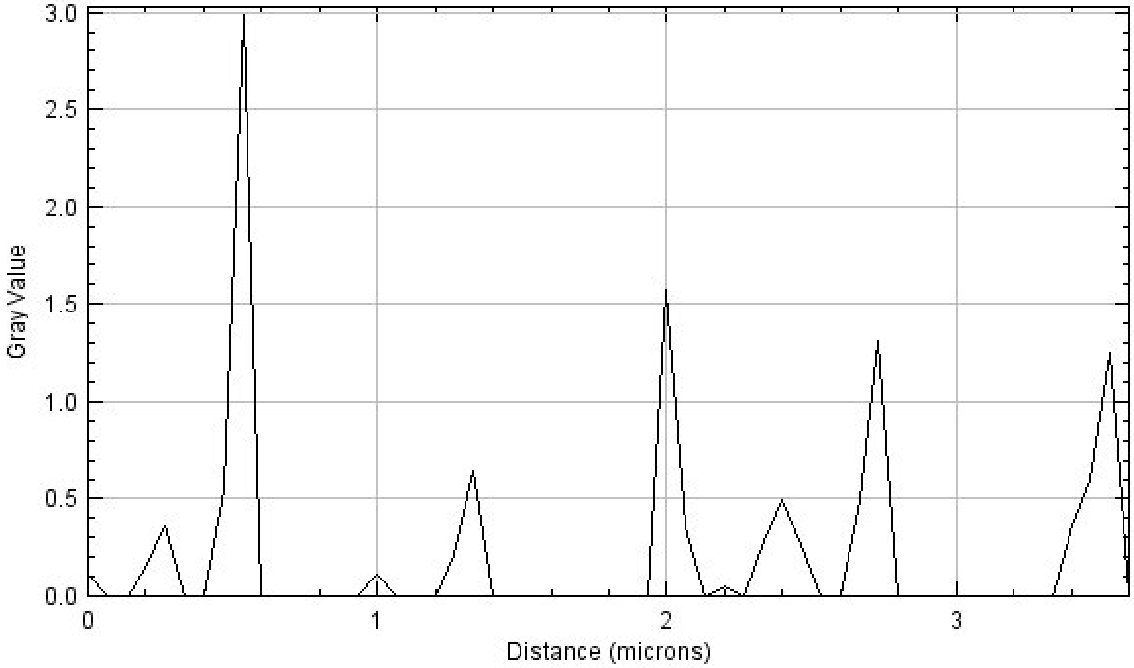

Figure S406 – Intensity profile of line scan generated for cell 6, Figure S400.

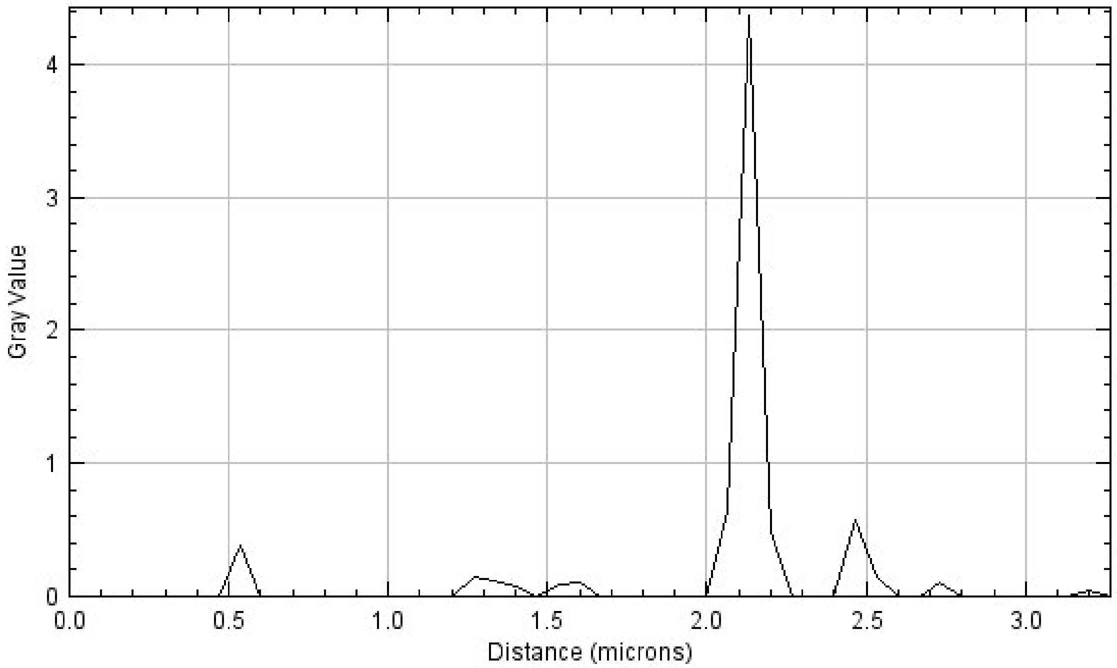

Figure S407 – Intensity profile of line scan generated for cell 7, Figure S400.

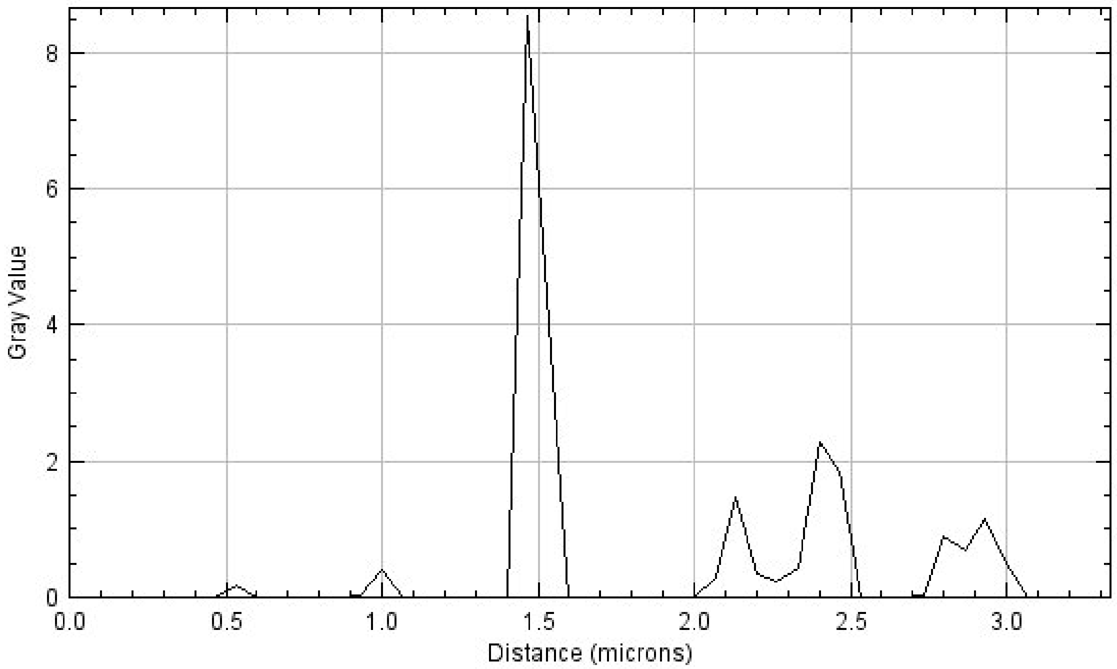

Figure S408 – Intensity profile of line scan generated for cell 8, Figure S400.

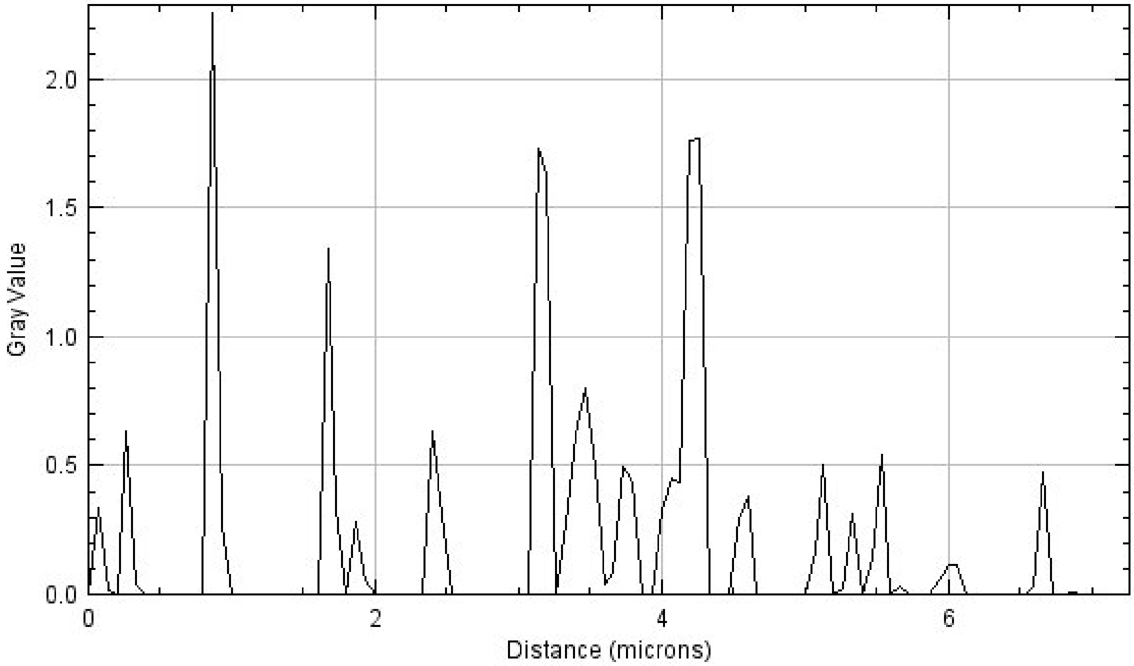

Figure S409 – Intensity profile of line scan generated for cell 9, Figure S400.

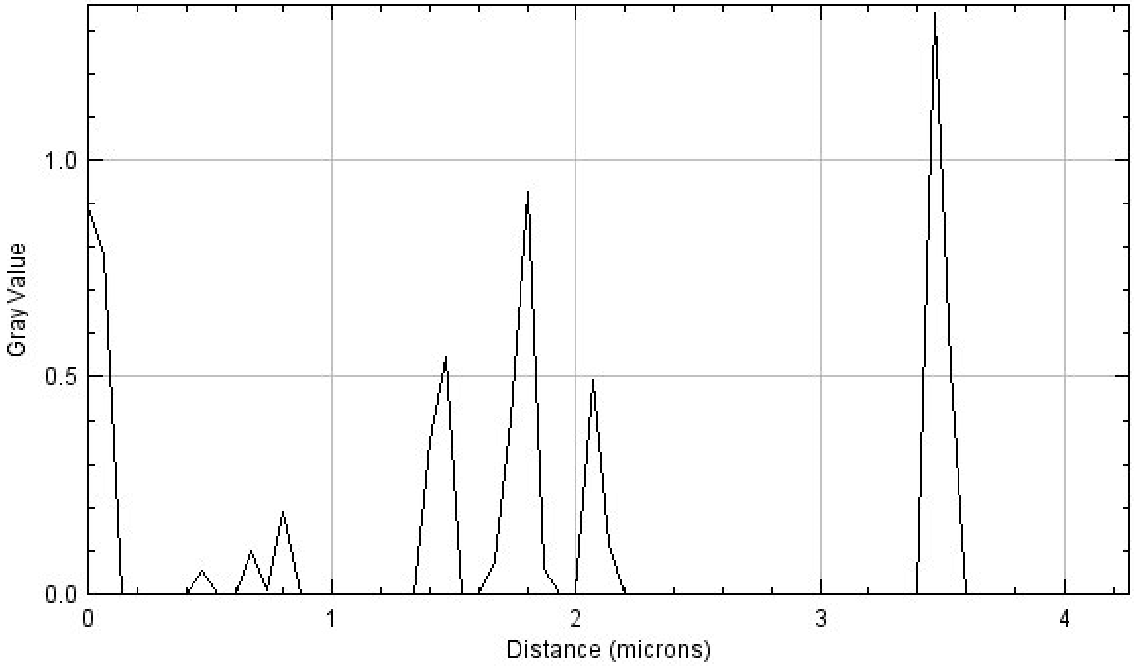

Figure S410 – Intensity profile of line scan generated for cell 10, Figure S400.

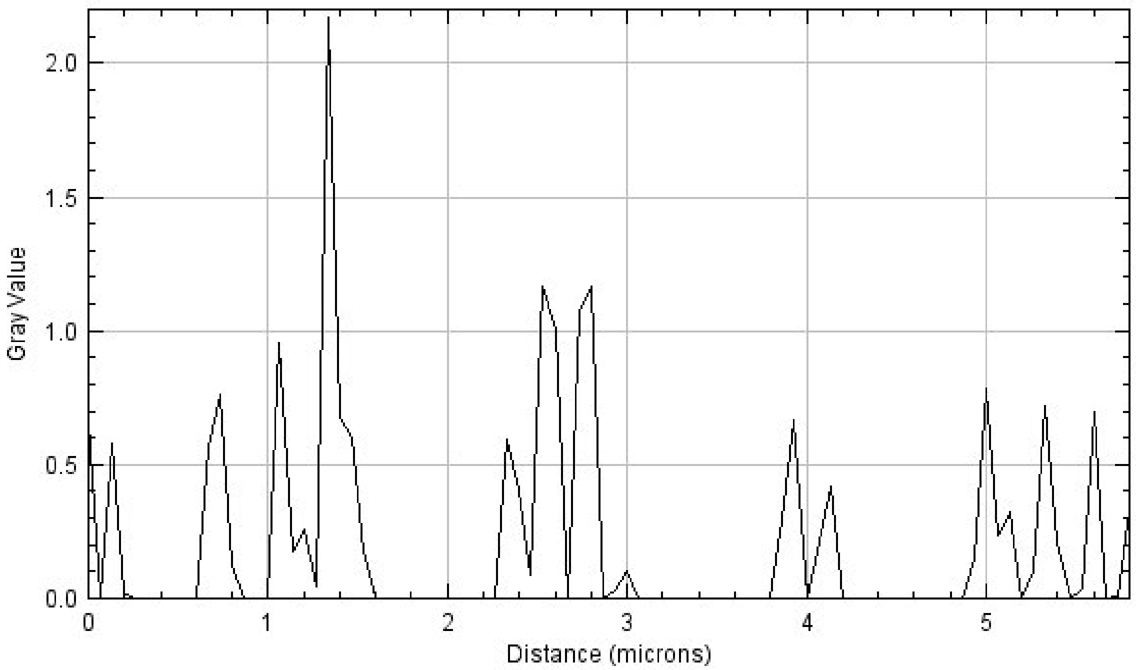

Figure S411 – Intensity profile of line scan generated for cell 11, Figure S400.

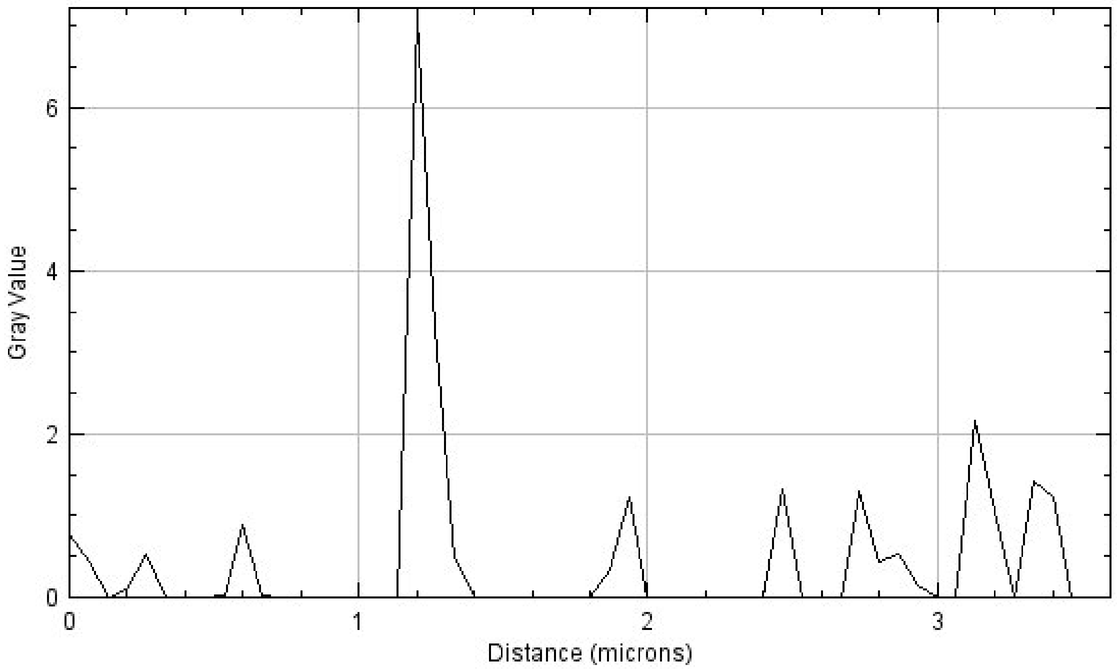

Figure S412 – Intensity profile of line scan generated for cell 12, Figure S400.

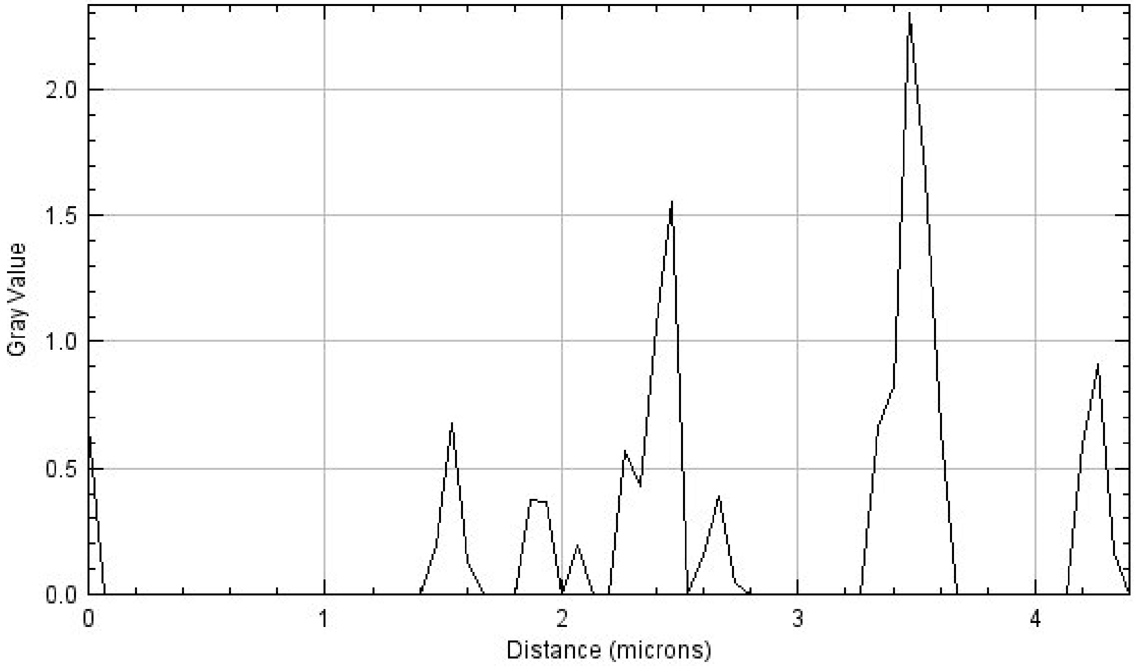

Figure S413 – Intensity profile of line scan generated for cell 13, Figure S400.

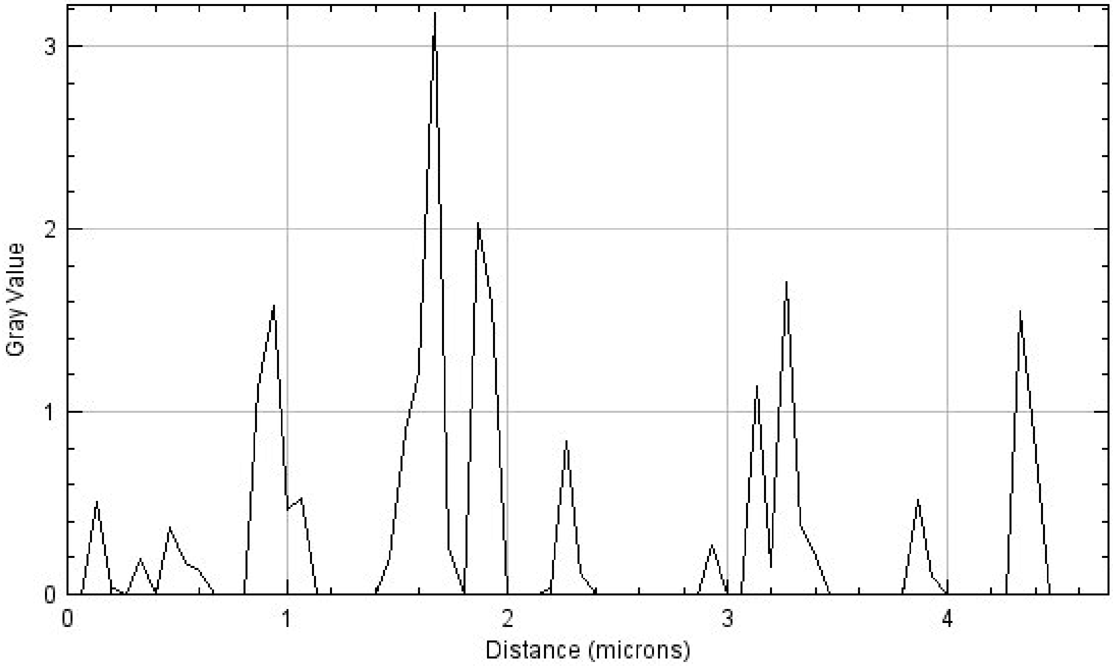

Figure S414 – Intensity profile of line scan generated for cell 14, Figure S400.

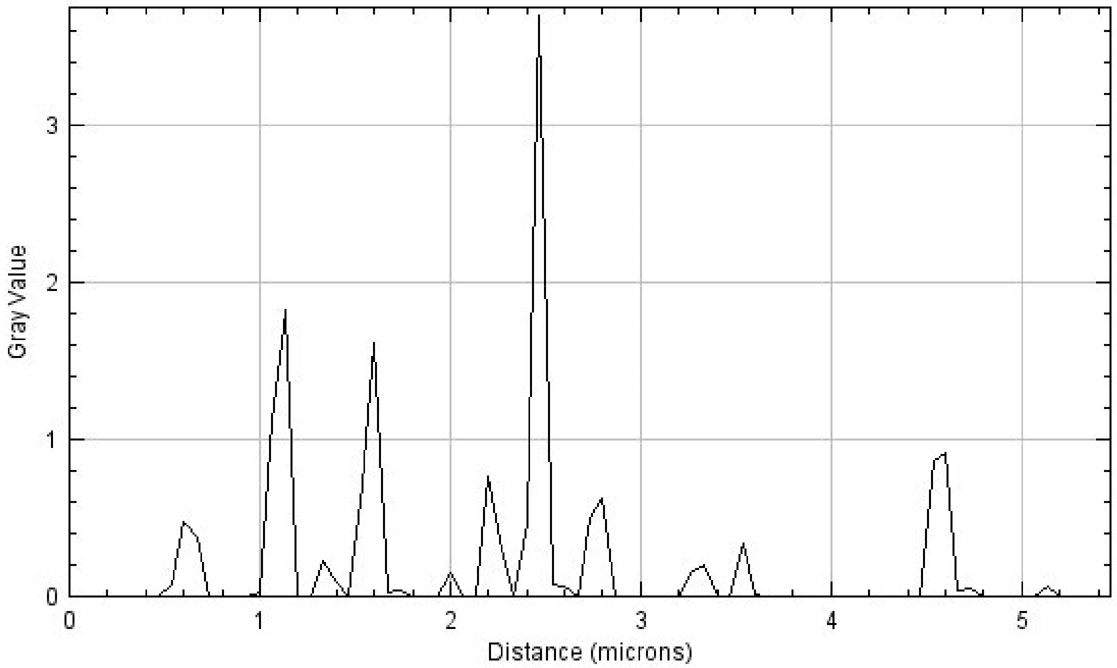

Figure S415 – Intensity profile of line scan generated for cell 15, Figure S400.

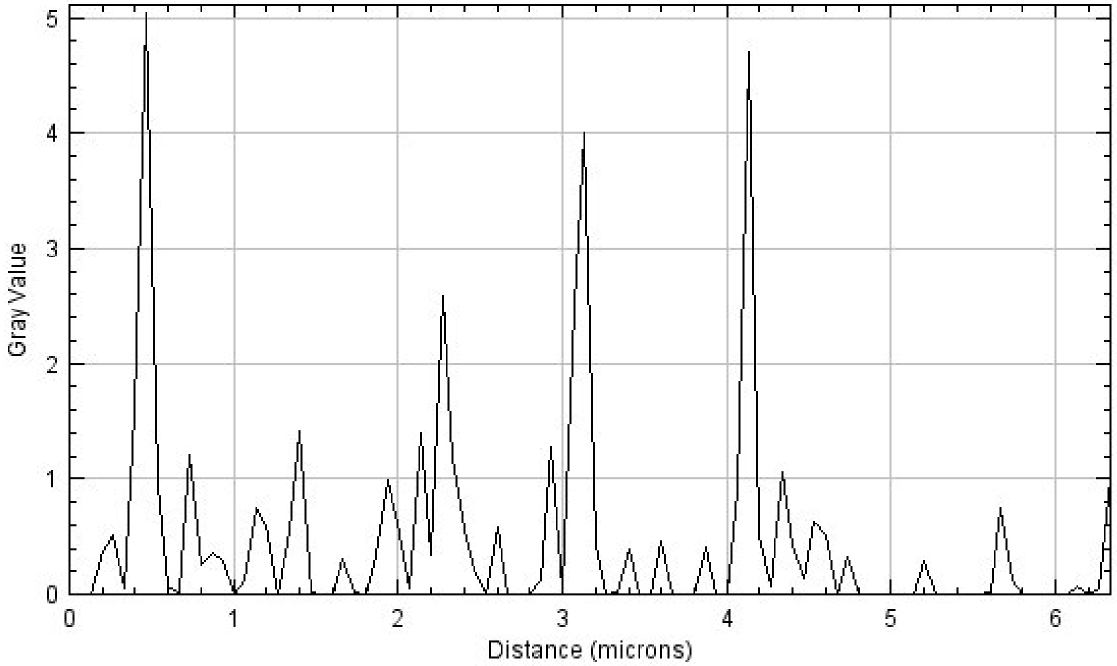

Figure S416 – Intensity profile of line scan generated for cell 16, Figure S400.

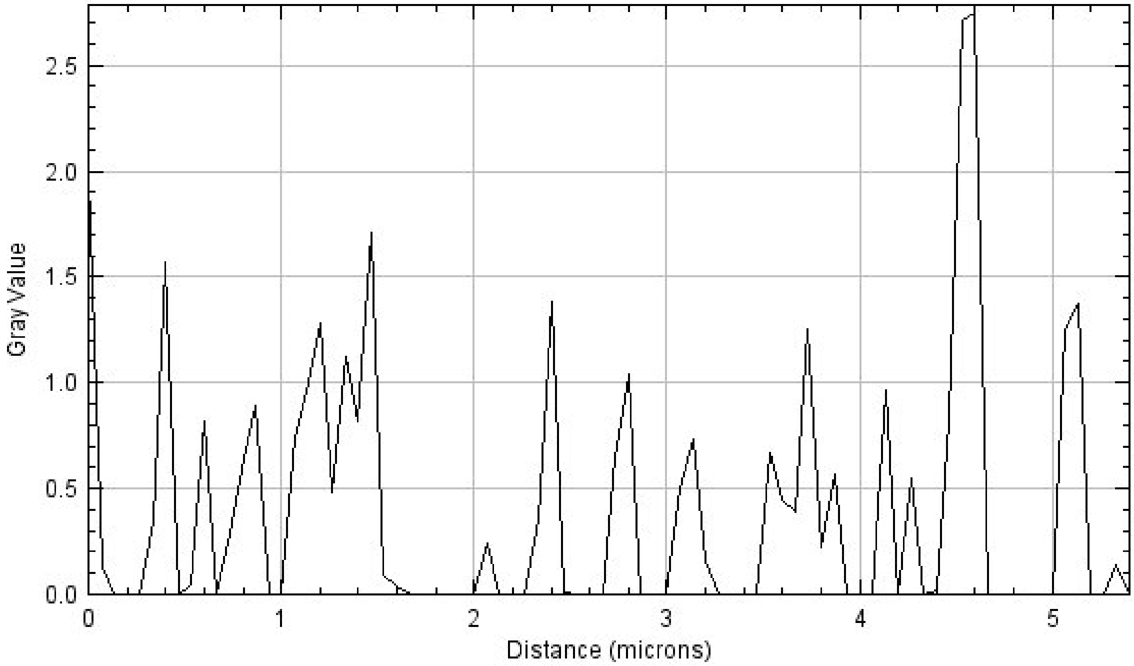

Figure S417 – Intensity profile of line scan generated for cell 17, Figure S400.

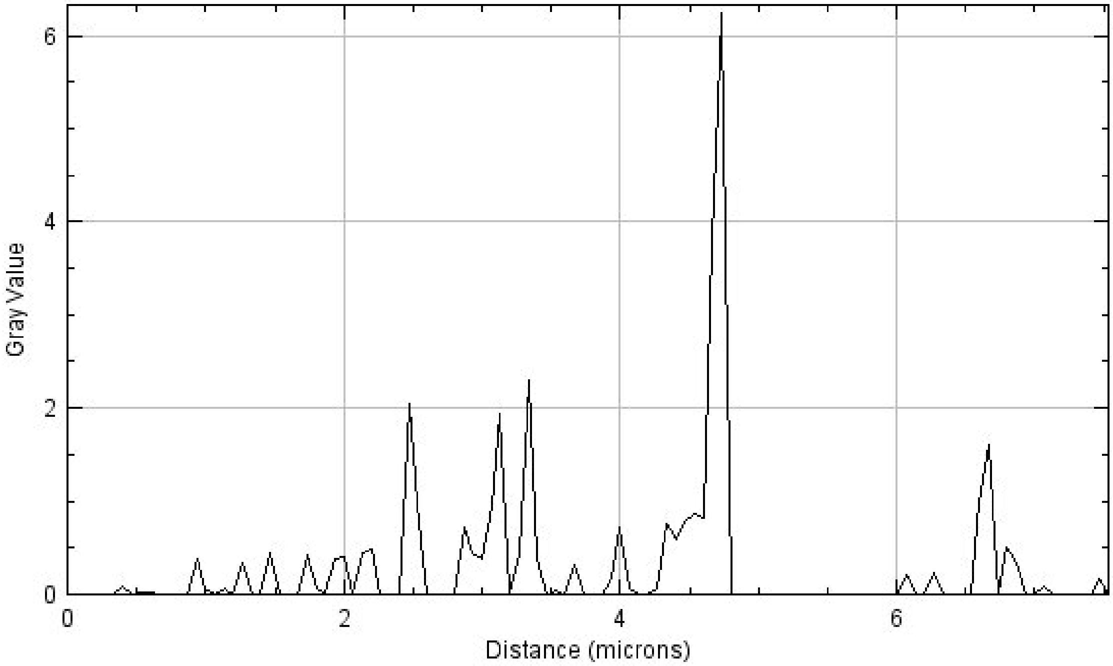

Figure S418 – Intensity profile of line scan generated for cell 18, Figure S400.

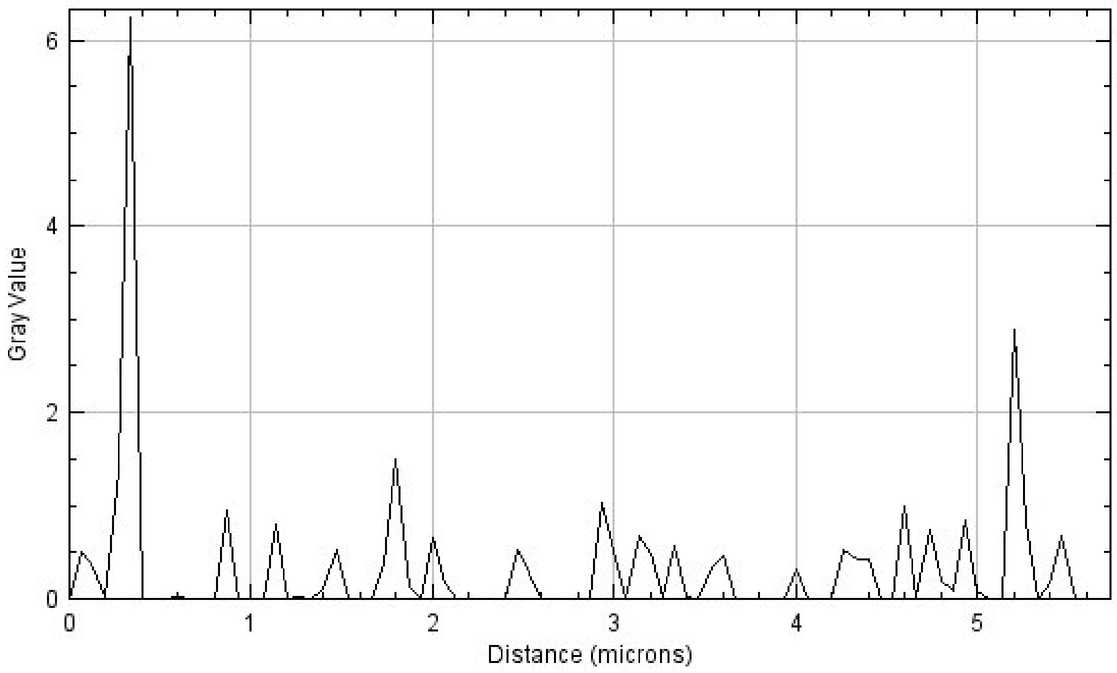

Figure S419 – Intensity profile of line scan generated for cell 19, Figure S400.

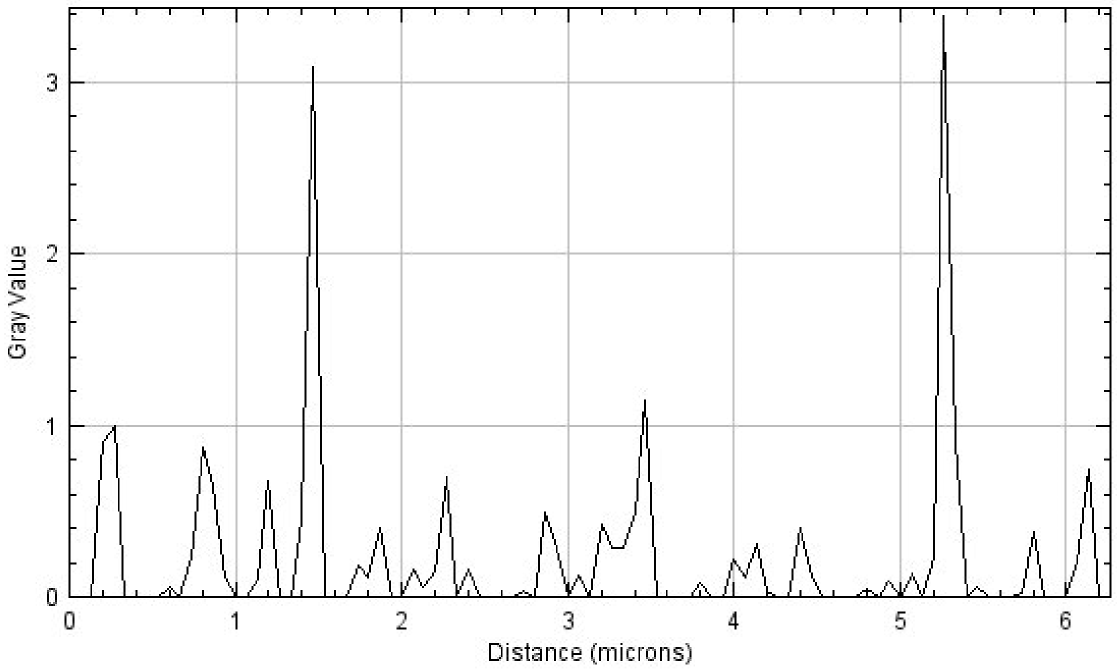

Figure S420 – Intensity profile of line scan generated for cell 20, Figure S400.

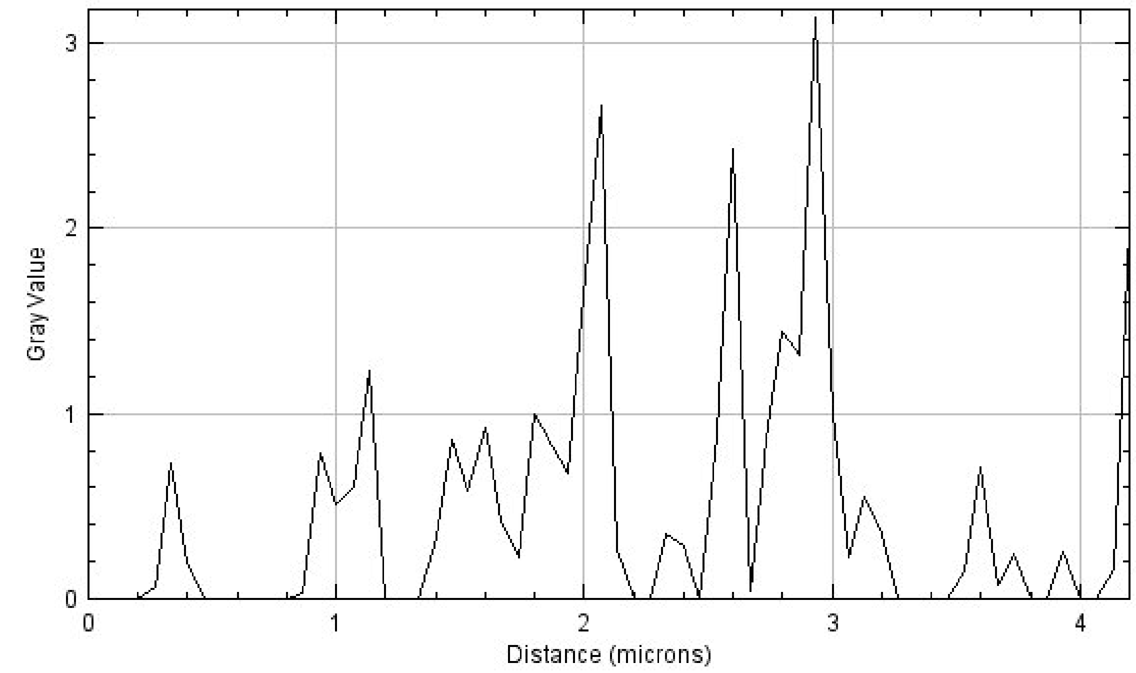

Figure S421 – Intensity profile of line scan generated for cell 21, Figure S400.

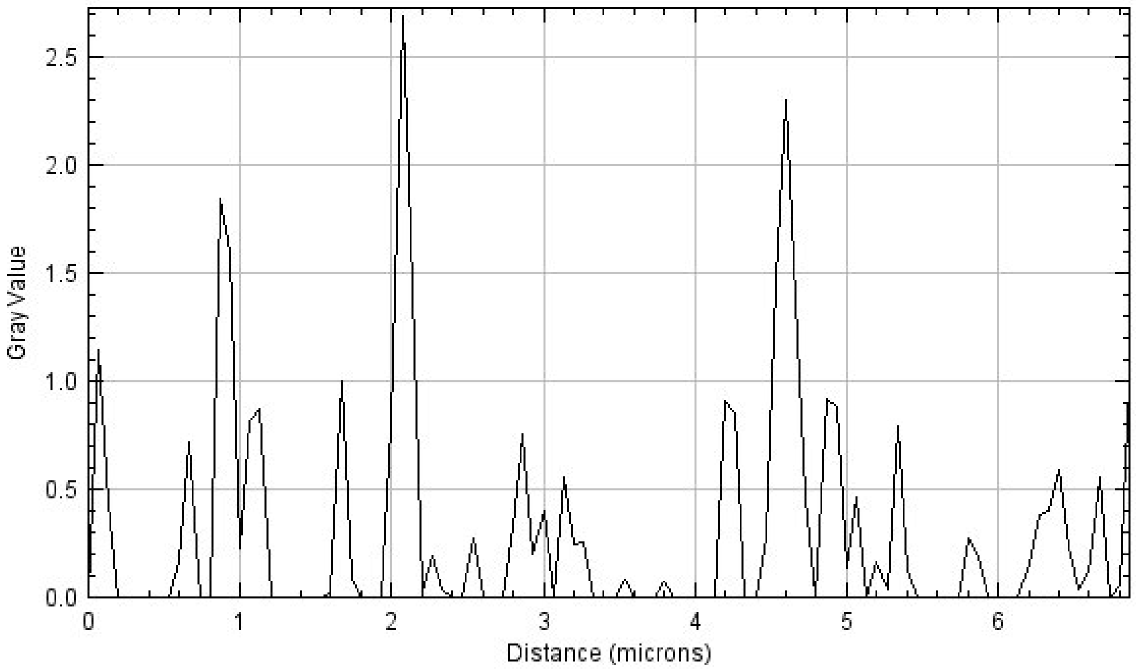

Figure S422 – Intensity profile of line scan generated for cell 22, Figure S400.

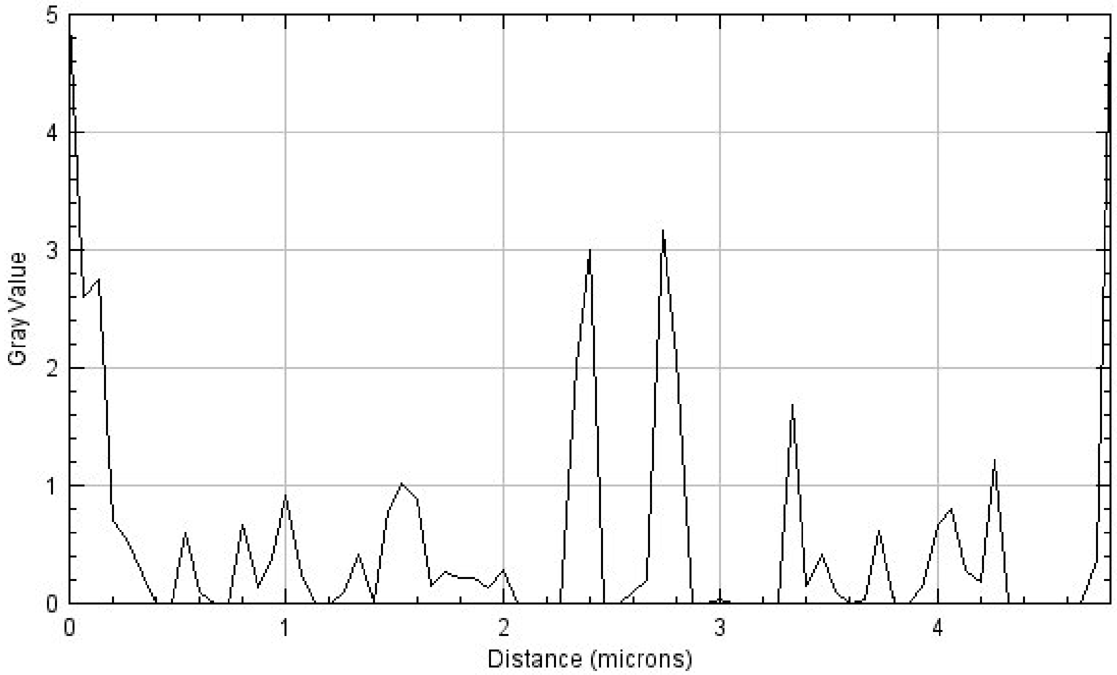

Figure S423 – Intensity profile of line scan generated for cell 23, Figure S400.

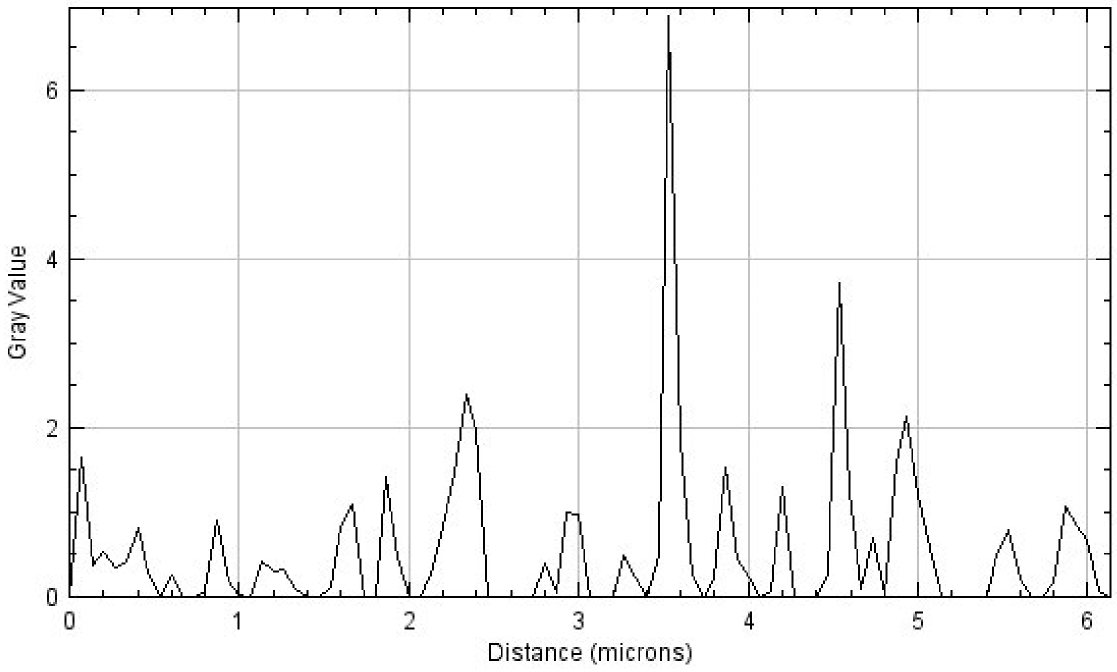

Figure S424 – Intensity profile of line scan generated for cell 24, Figure S400.

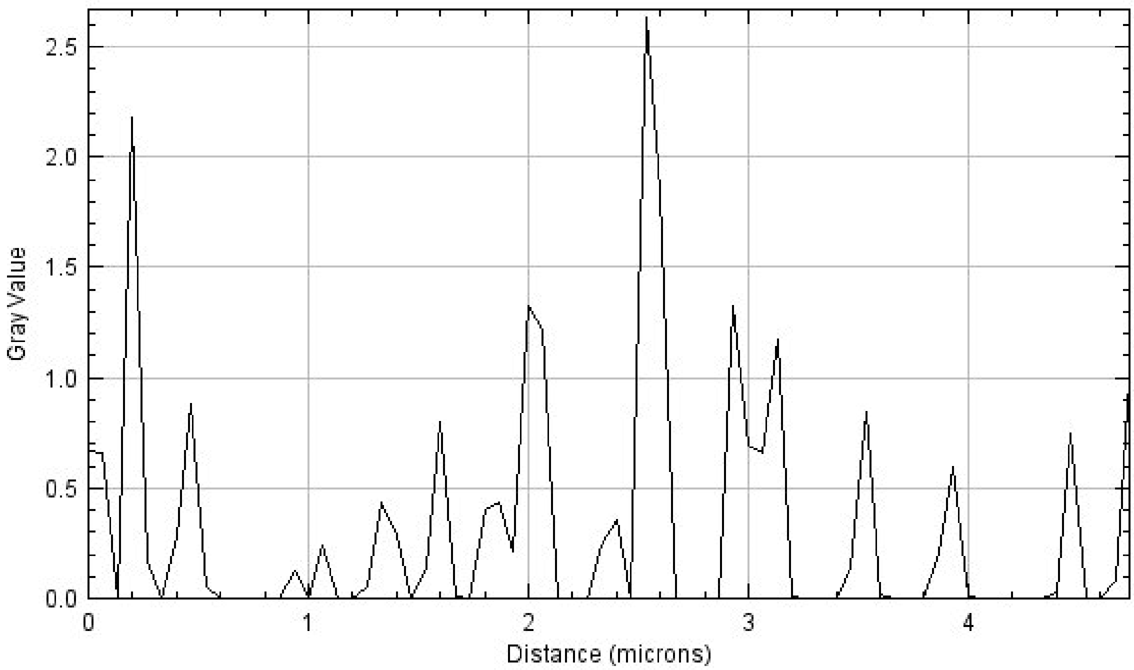

Figure S425 – Intensity profile of line scan generated for cell 25, Figure S400.

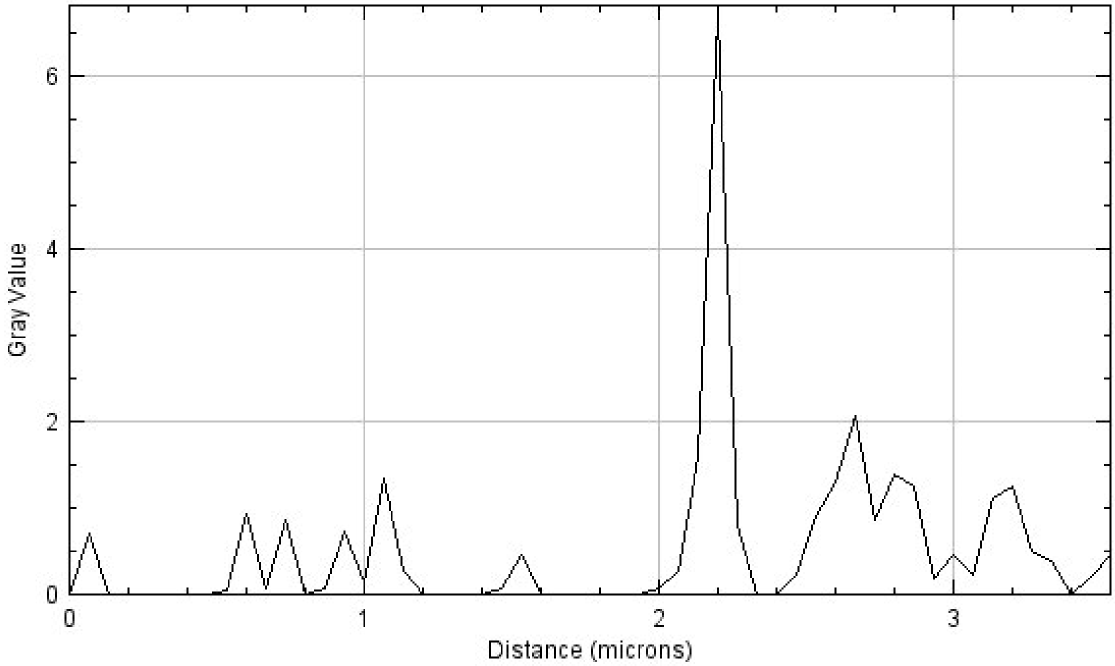

Figure S426 – Intensity profile of line scan generated for cell 26, Figure S400.

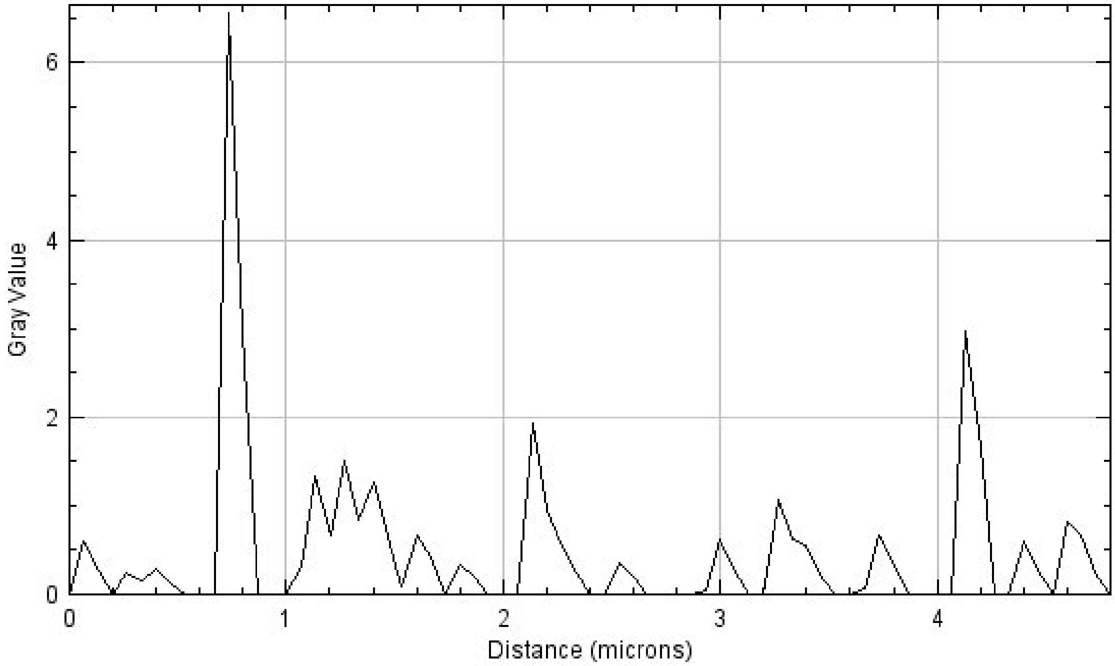

Figure S427 – Intensity profile of line scan generated for cell 27, Figure S400.

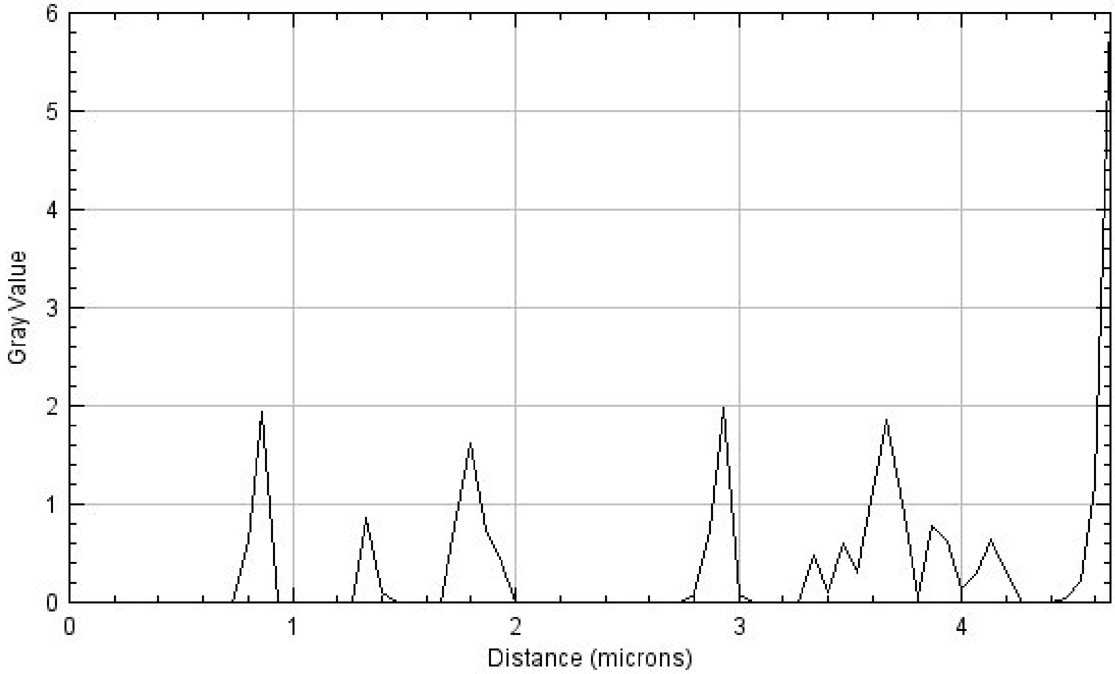

Figure S428 – Intensity profile of line scan generated for cell 28, Figure S400.

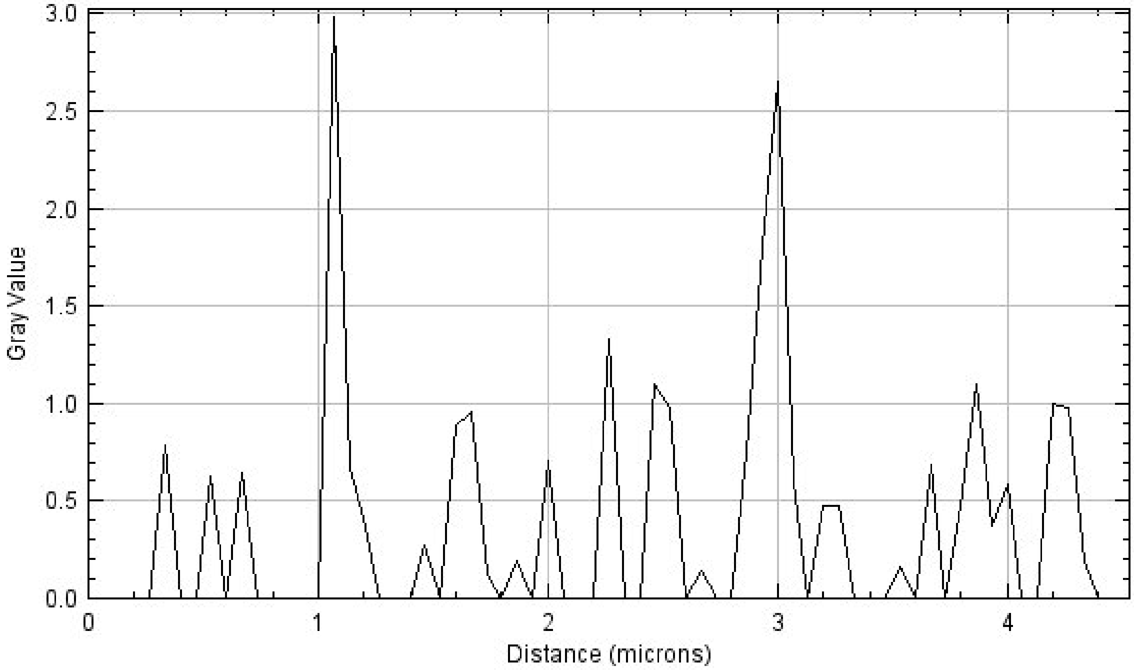

Figure S429 – Intensity profile of line scan generated for cell 29, Figure S400.

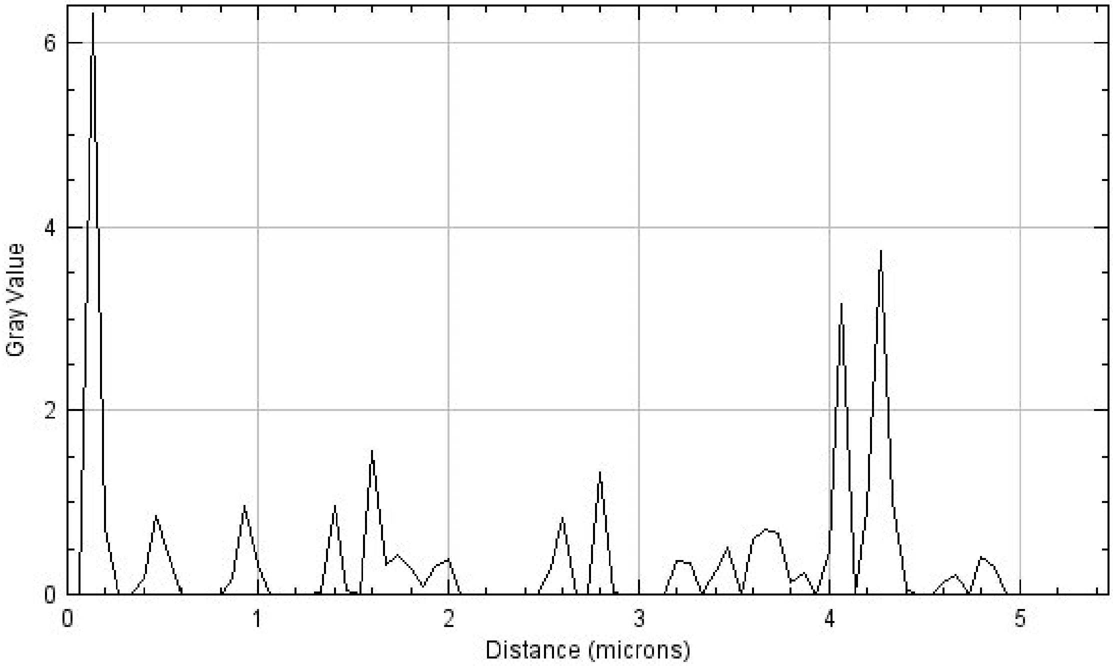

Figure S430 – Intensity profile of line scan generated for cell 30, Figure S400.

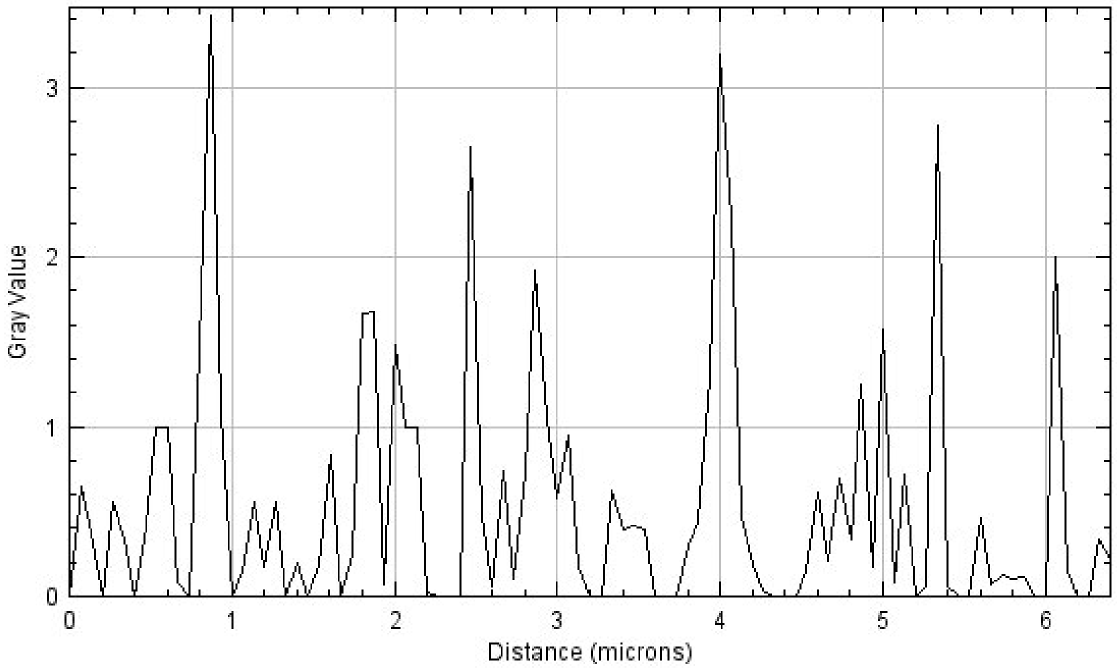

Figure S431 – Intensity profile of line scan generated for cell 31, Figure S400.

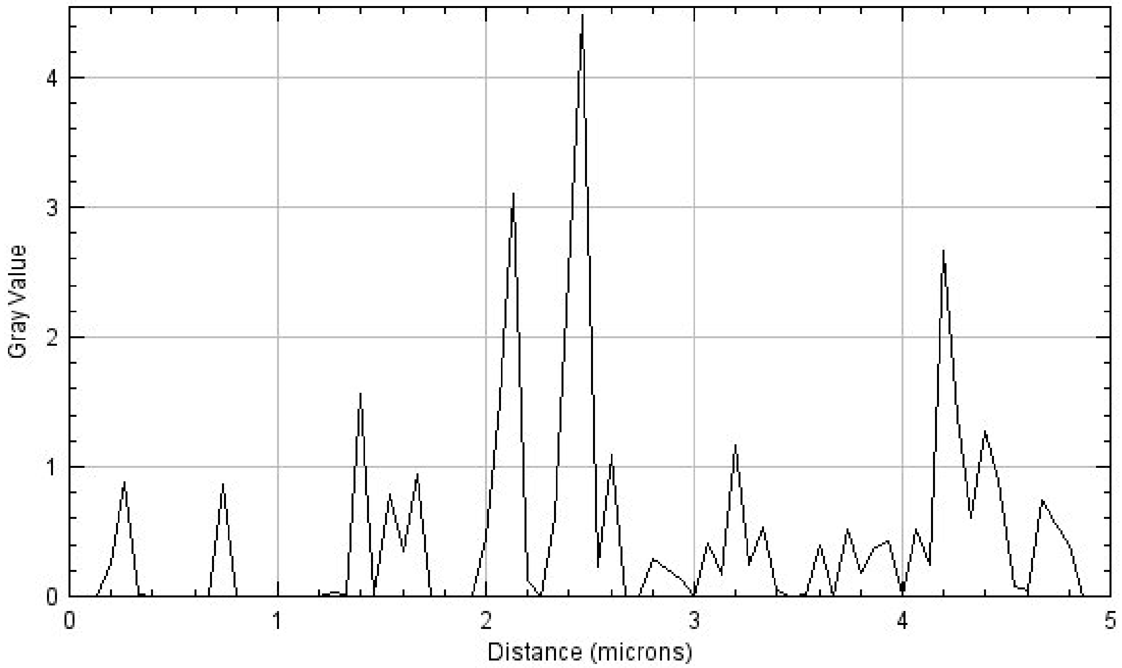

Figure S432 – Intensity profile of line scan generated for cell 32, Figure S400.

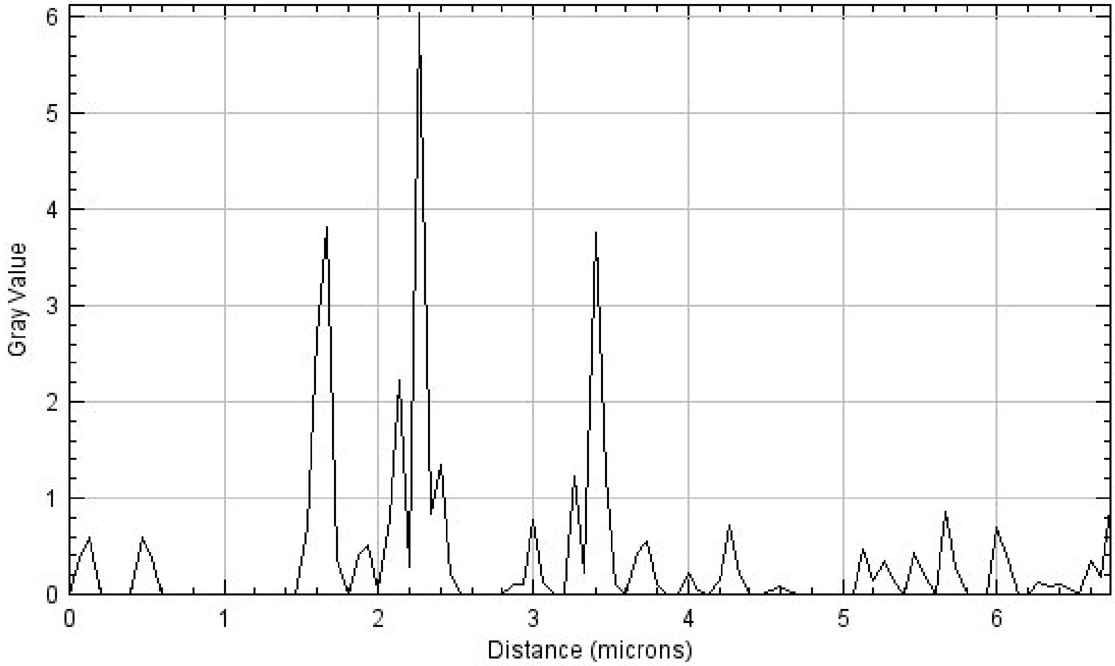

Figure S433 – Intensity profile of line scan generated for cell 33, Figure S400.

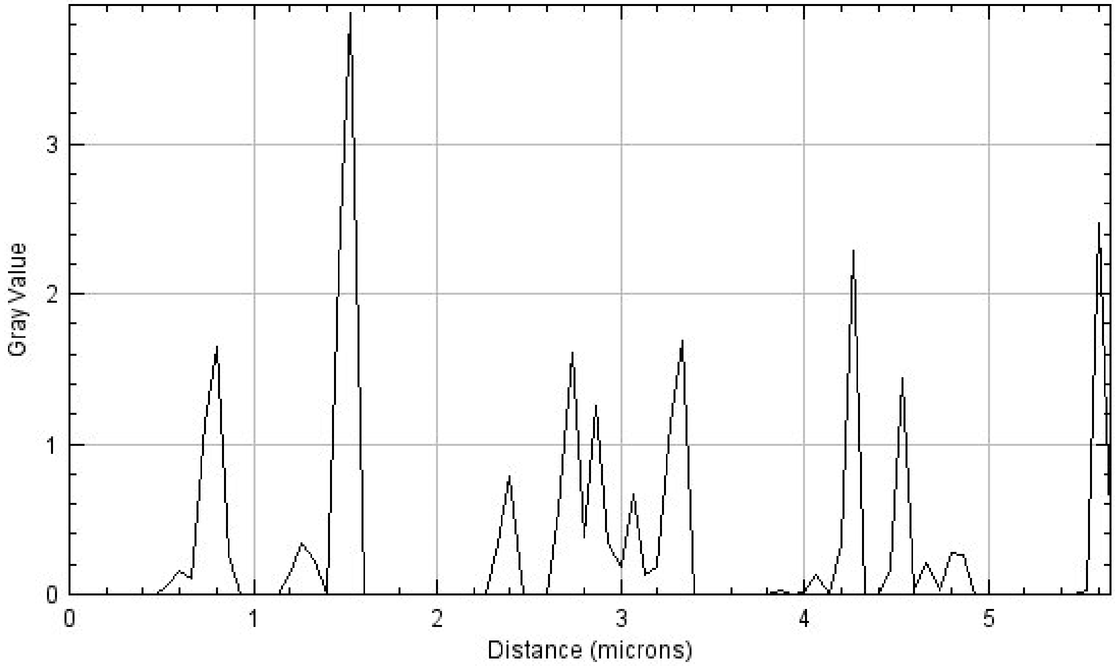

Figure S434 – Intensity profile of line scan generated for cell 34, Figure S400.

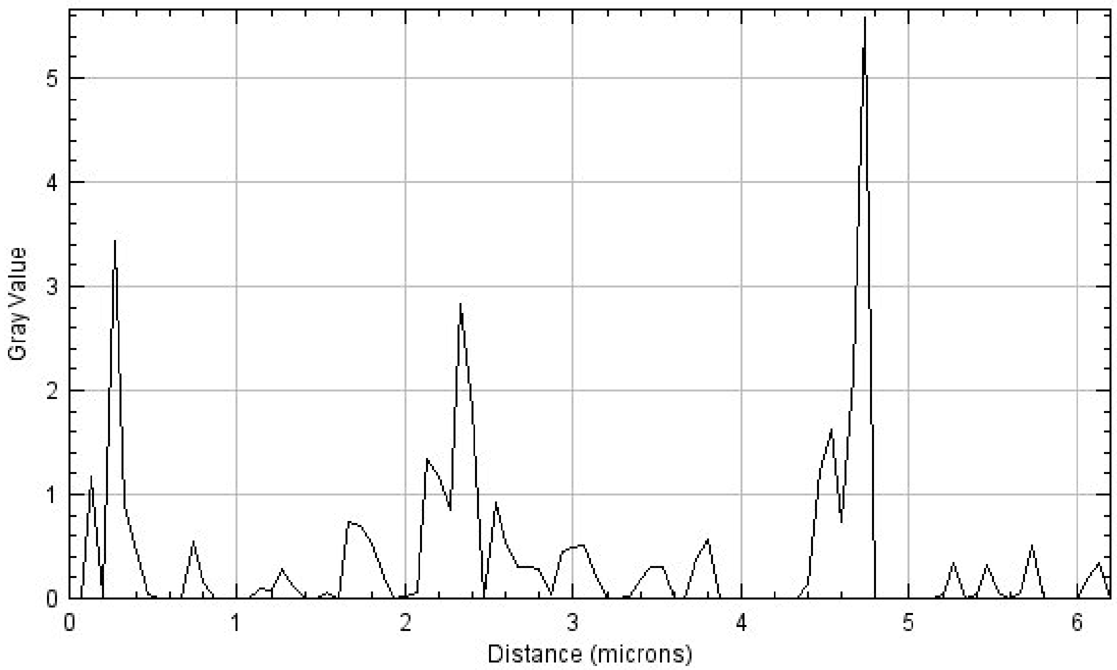

Figure S435 – Intensity profile of line scan generated for cell 35, Figure S400.

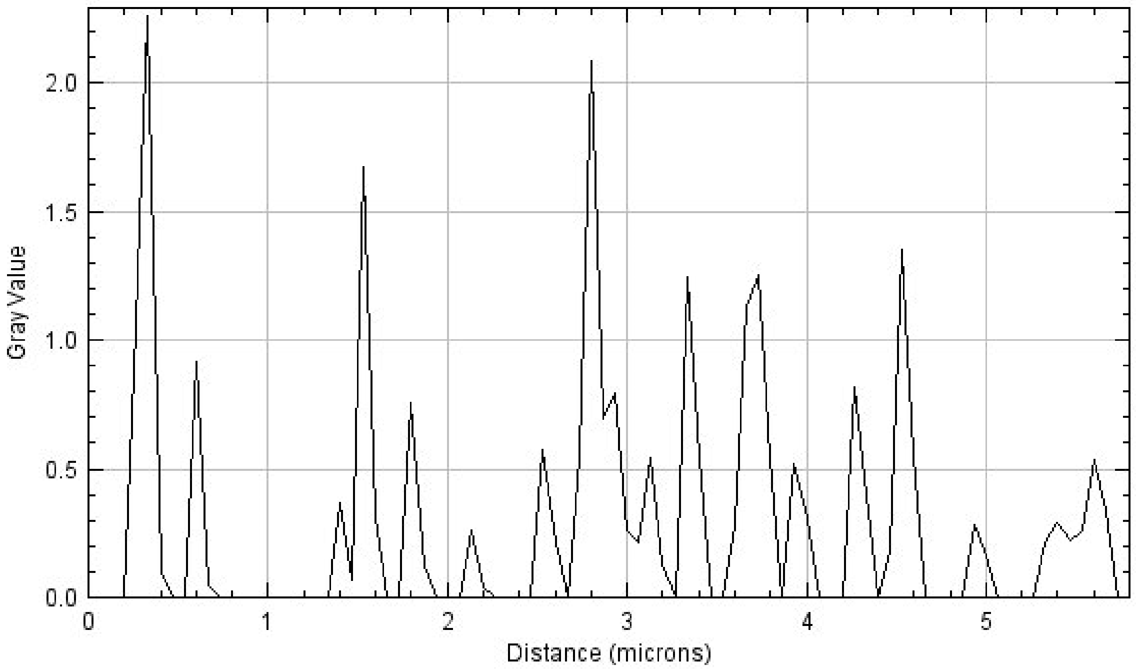

Figure S436 – Intensity profile of line scan generated for cell 36, Figure S400.

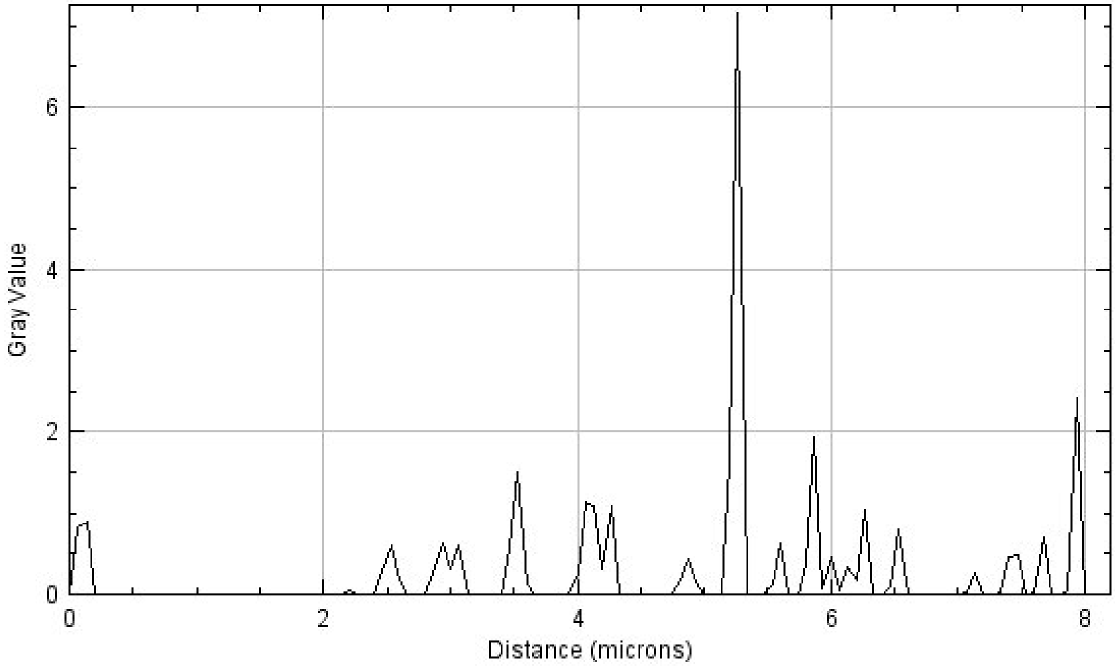

Figure S437 – Intensity profile of line scan generated for cell 37, Figure S400.

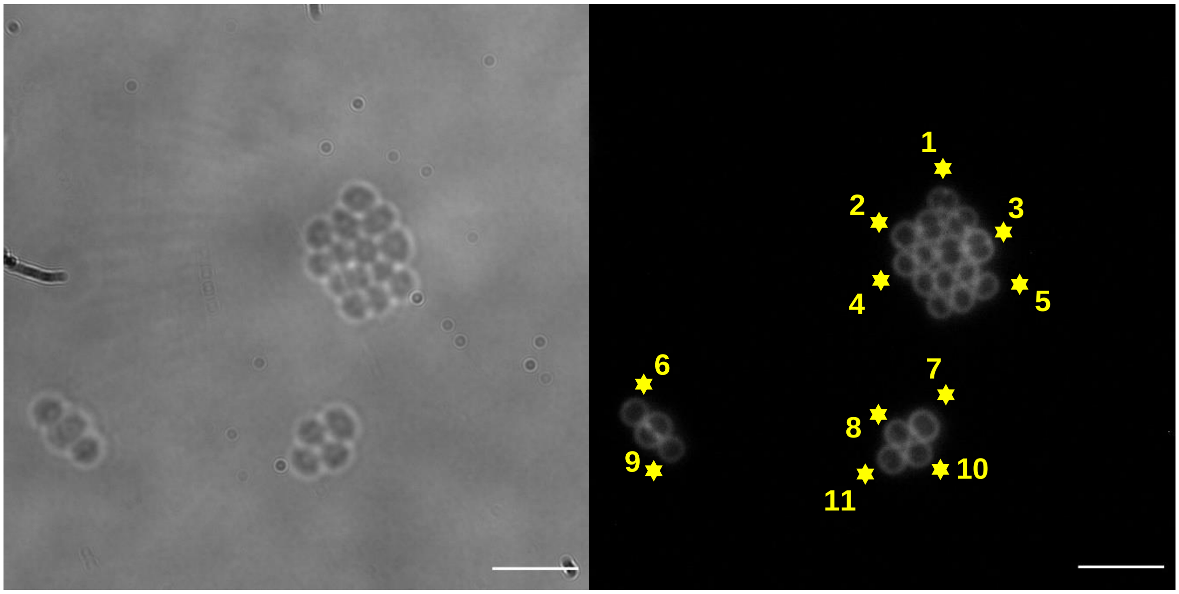

Figure S438 – MRSA transmitted image and fluorescence image at 605 nm used in fluorescence intensity calculations in the presence of **FM4-64** only at T = 30 minutes. Scale bar = 10 μM.

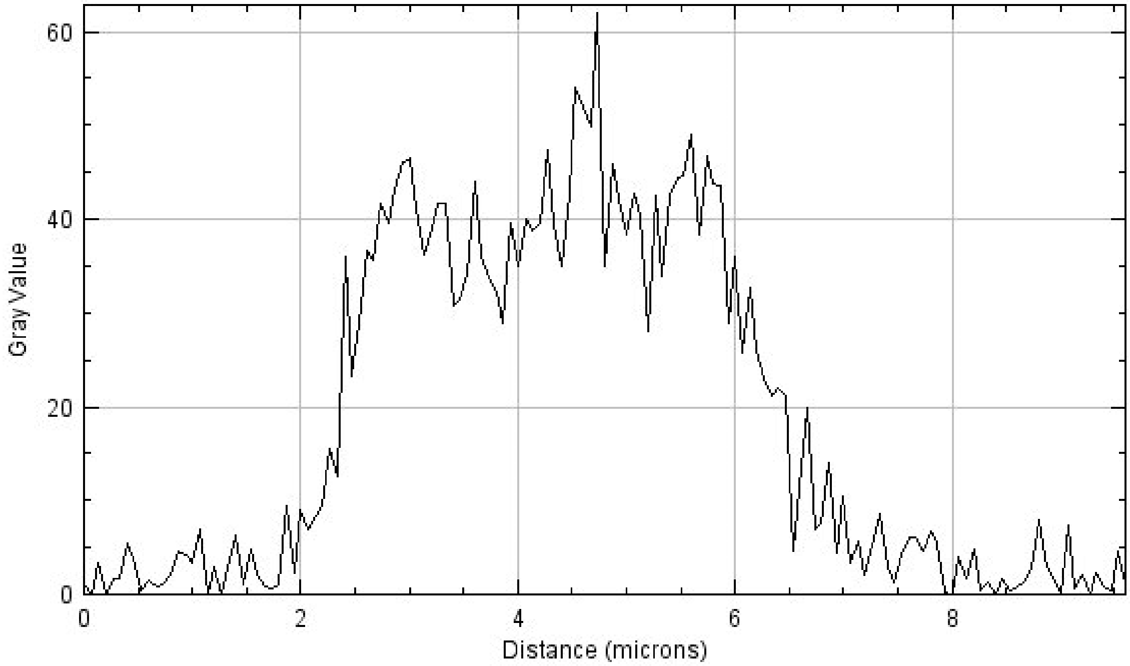

Figure S439 – Intensity profile of line scan generated for cell 1, Figure S438.

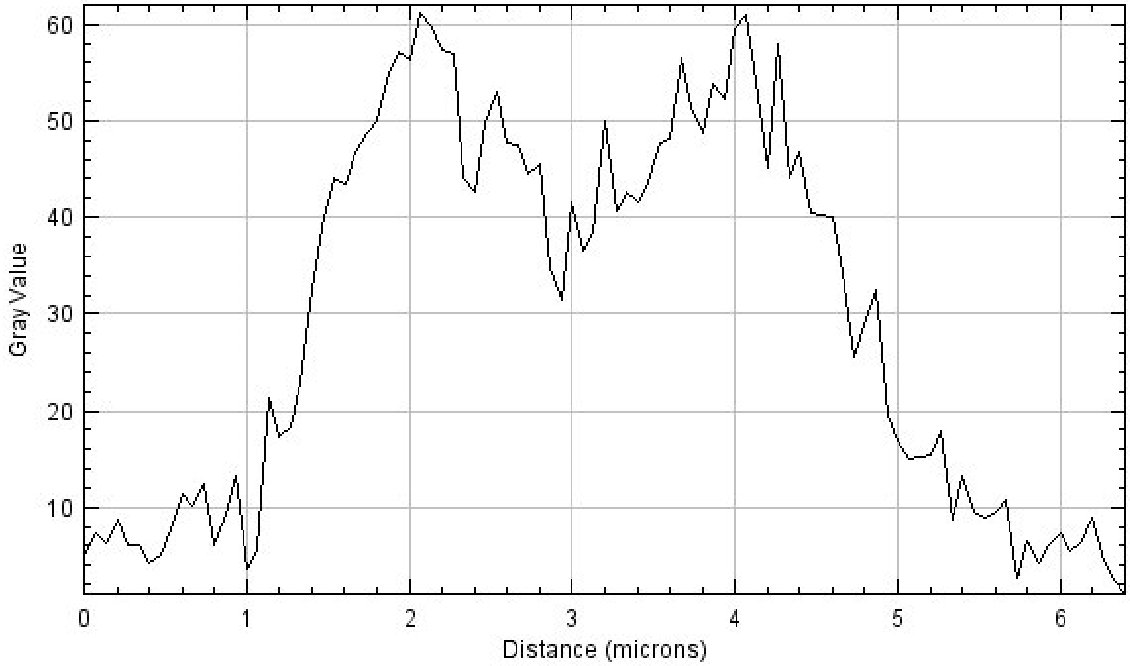

Figure S440 – Intensity profile of line scan generated for cell 2, Figure S438.

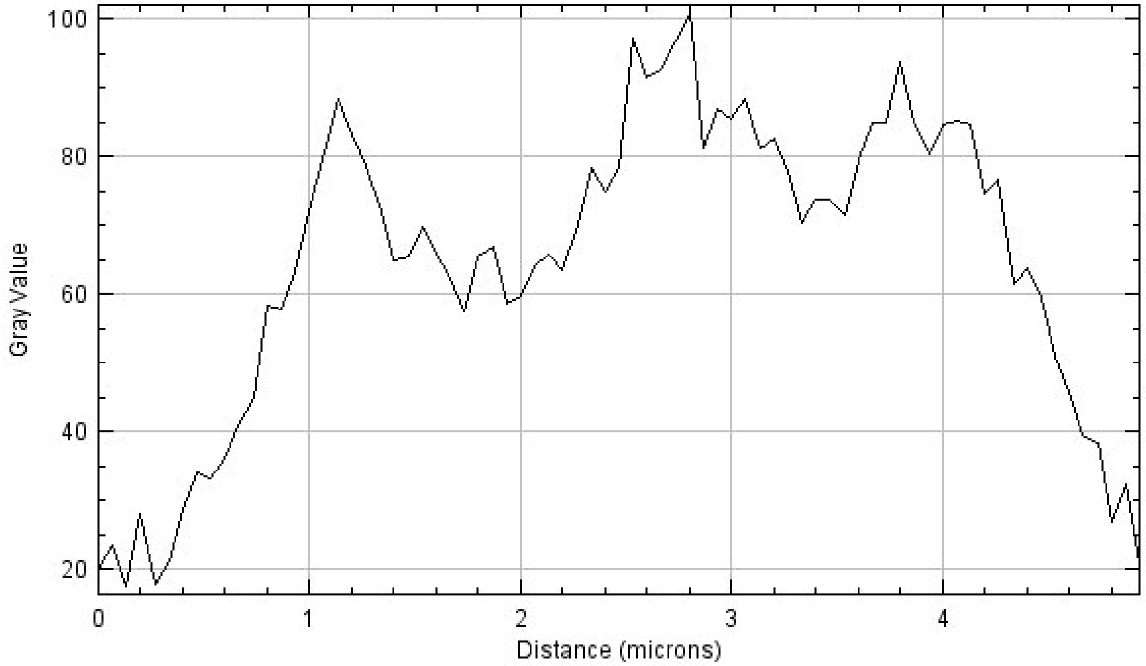

Figure S441 – Intensity profile of line scan generated for cell 3, Figure S438.

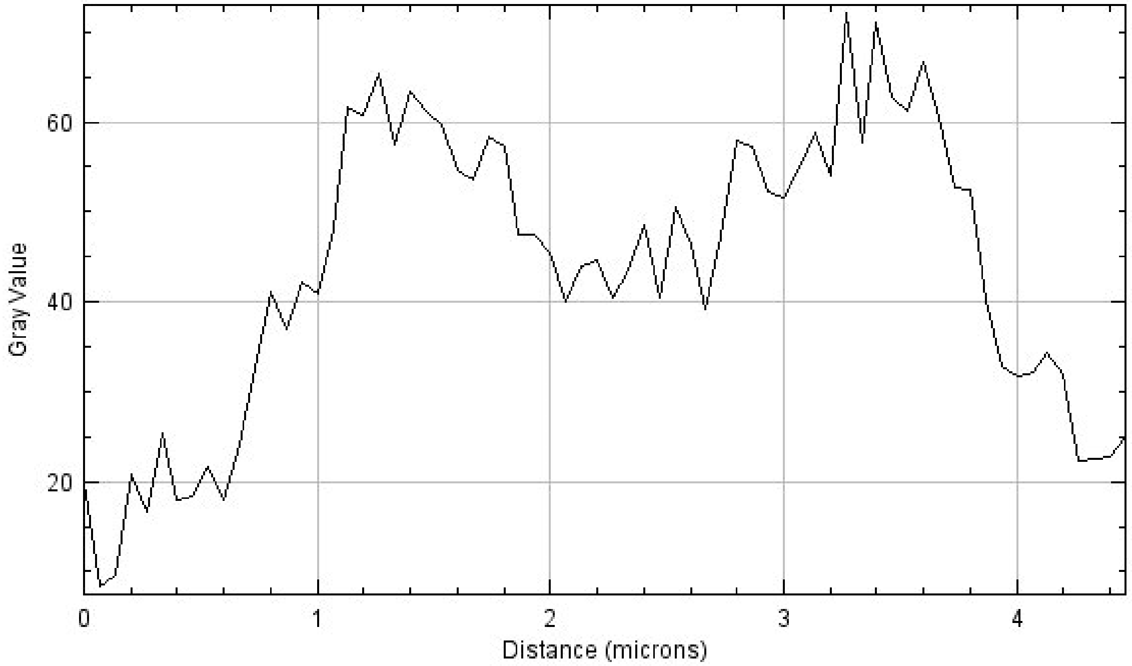

Figure S442 – Intensity profile of line scan generated for cell 4, Figure S438.

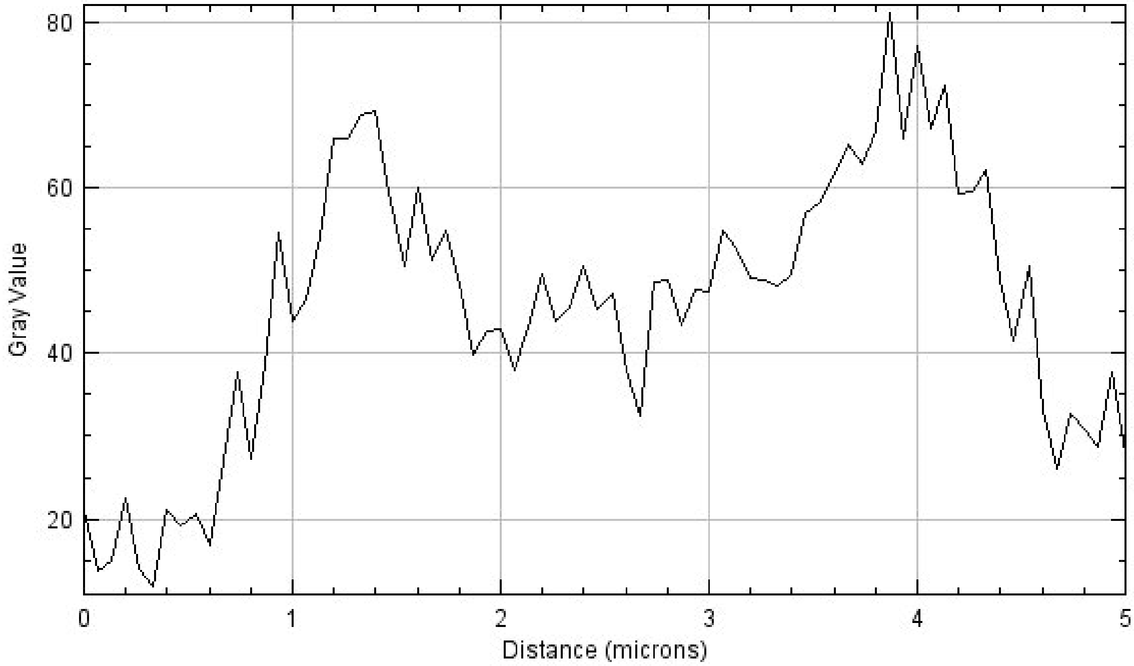

Figure S443 – Intensity profile of line scan generated for cell 5, Figure S438.

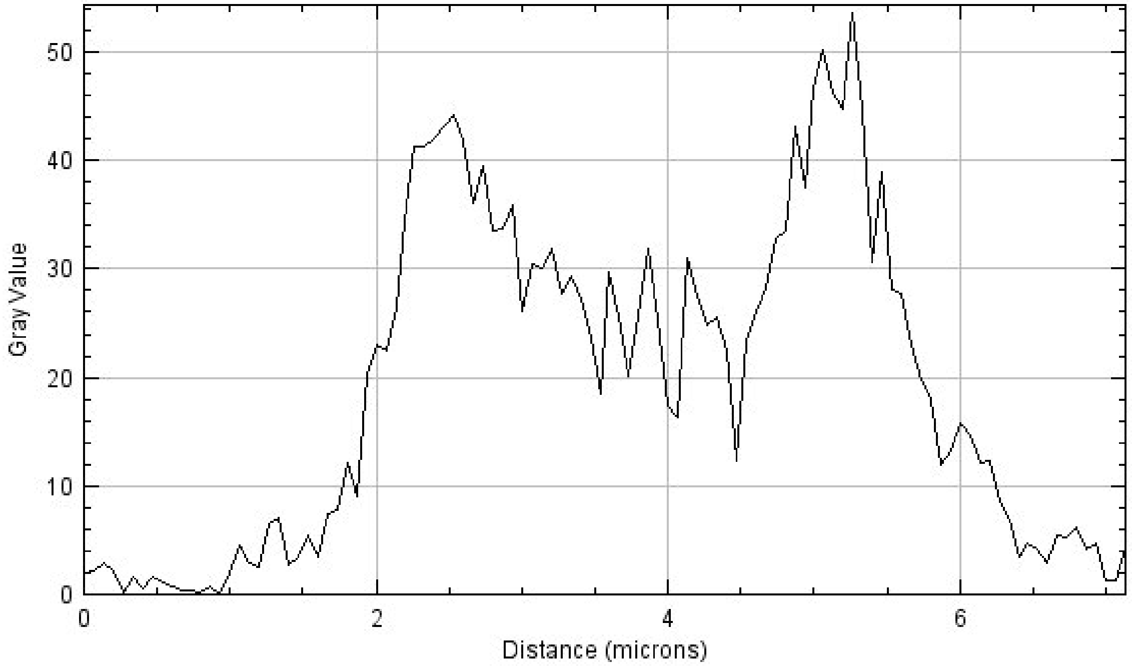

Figure S444 – Intensity profile of line scan generated for cell 6, Figure S438.

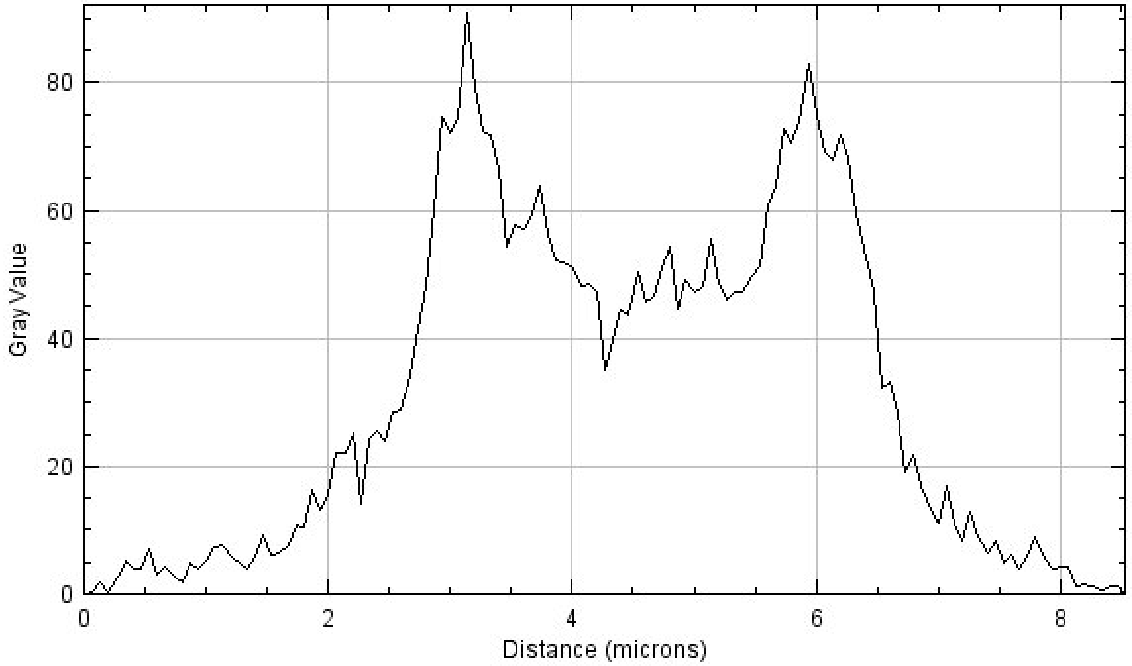

Figure S445 – Intensity profile of line scan generated for cell 7, Figure S438.

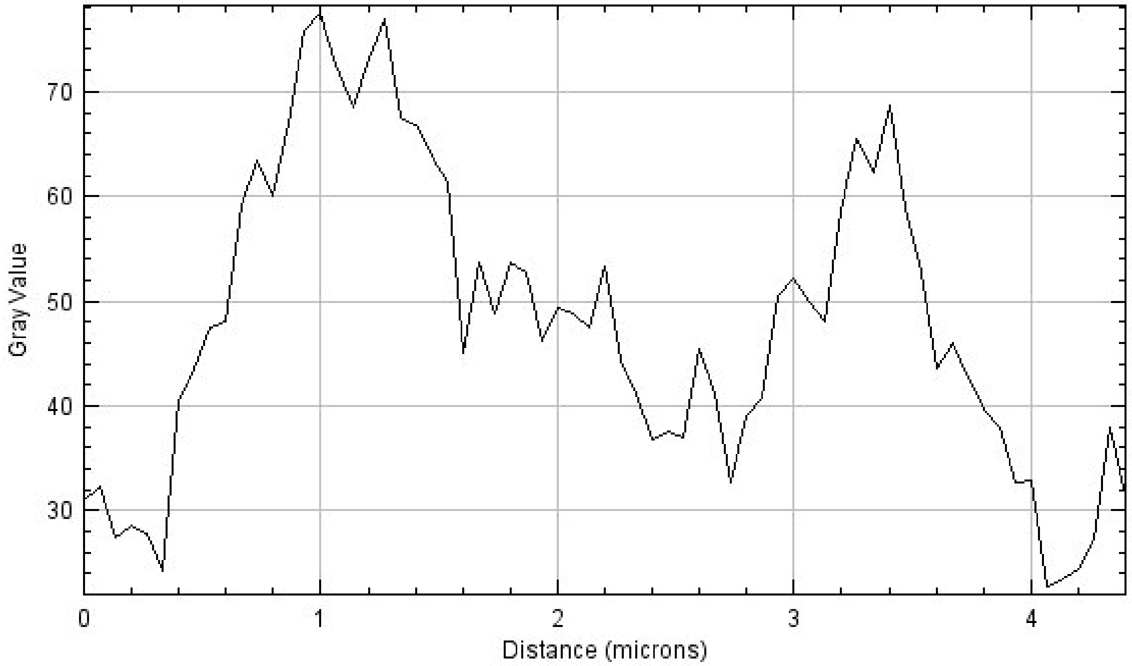

Figure S446 – Intensity profile of line scan generated for cell 8, Figure S438.

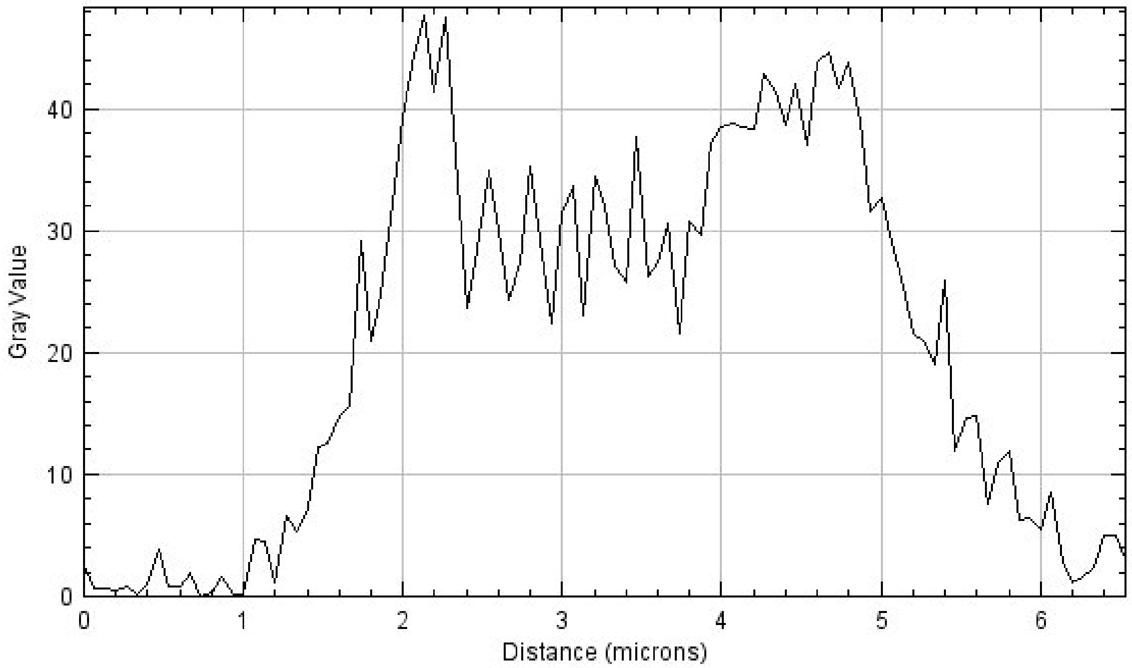

Figure S447 – Intensity profile of line scan generated for cell 9, Figure S438.

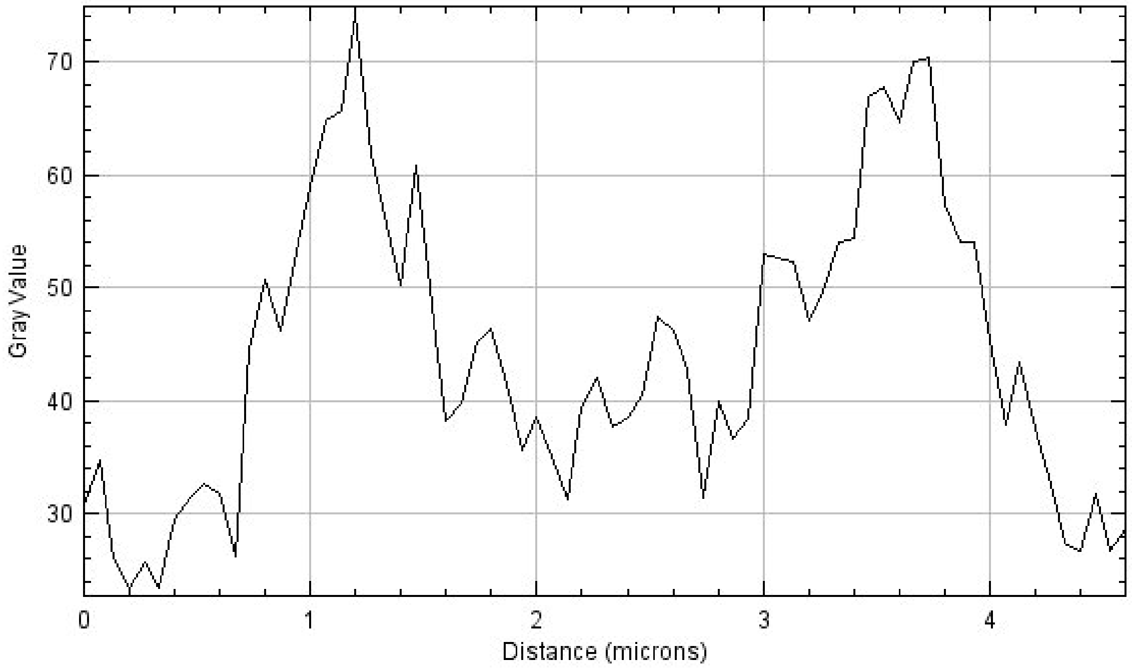

Figure S448 – Intensity profile of line scan generated for cell 10, Figure S438.

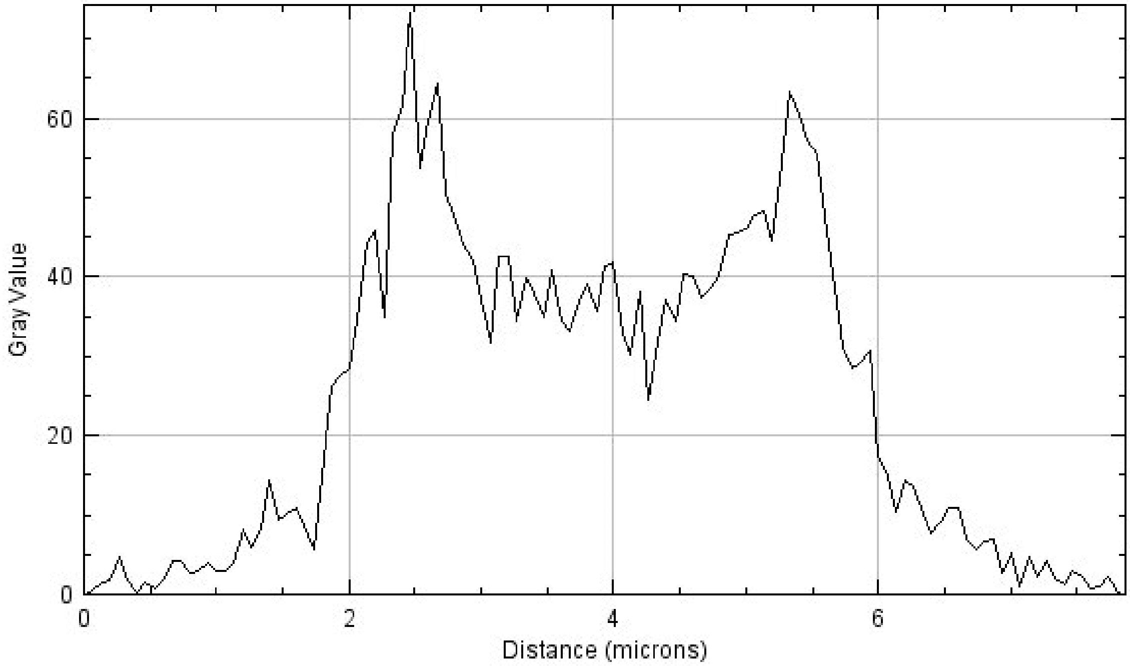

Figure S449 – Intensity profile of line scan generated for cell 11, Figure S438.

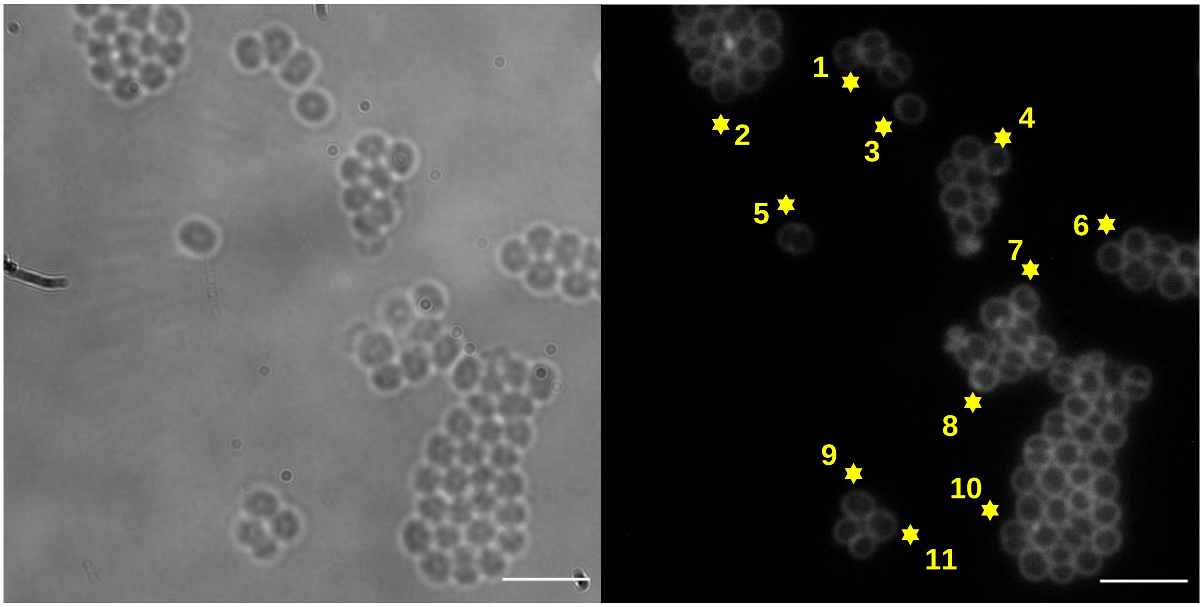

Figure S450 – MRSA transmitted image and fluorescence image at 605 nm used in fluorescence intensity calculations in the presence of **FM4-64** only at T = 30 minutes. Scale bar = 10 μM.

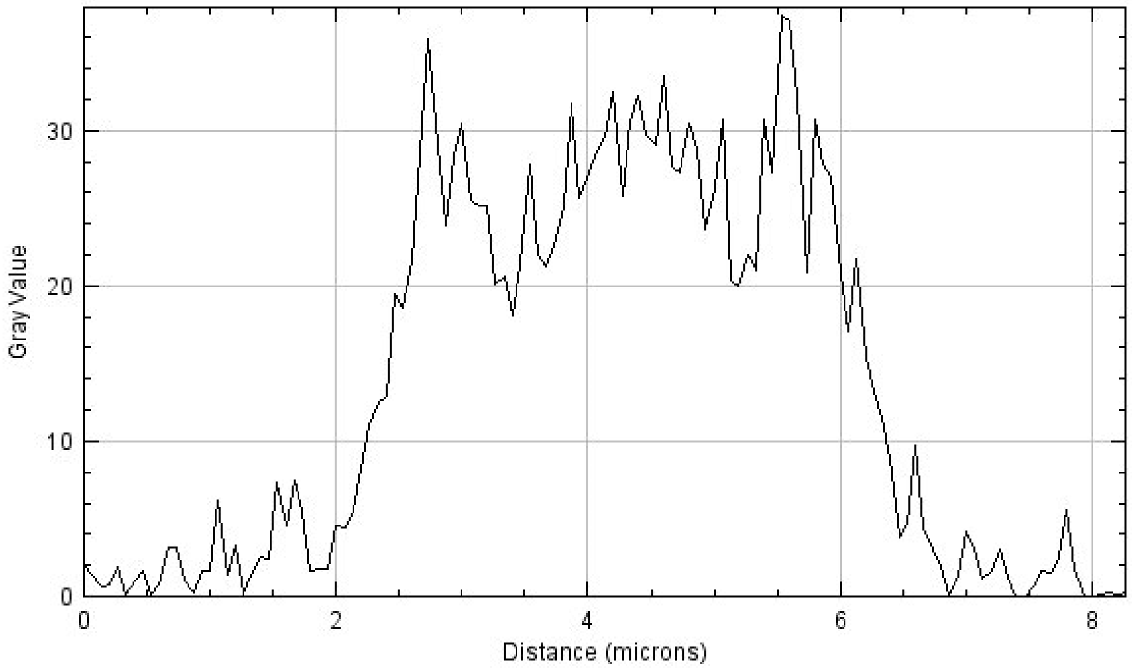

Figure S451 – Intensity profile of line scan generated for cell 1, Figure S450.

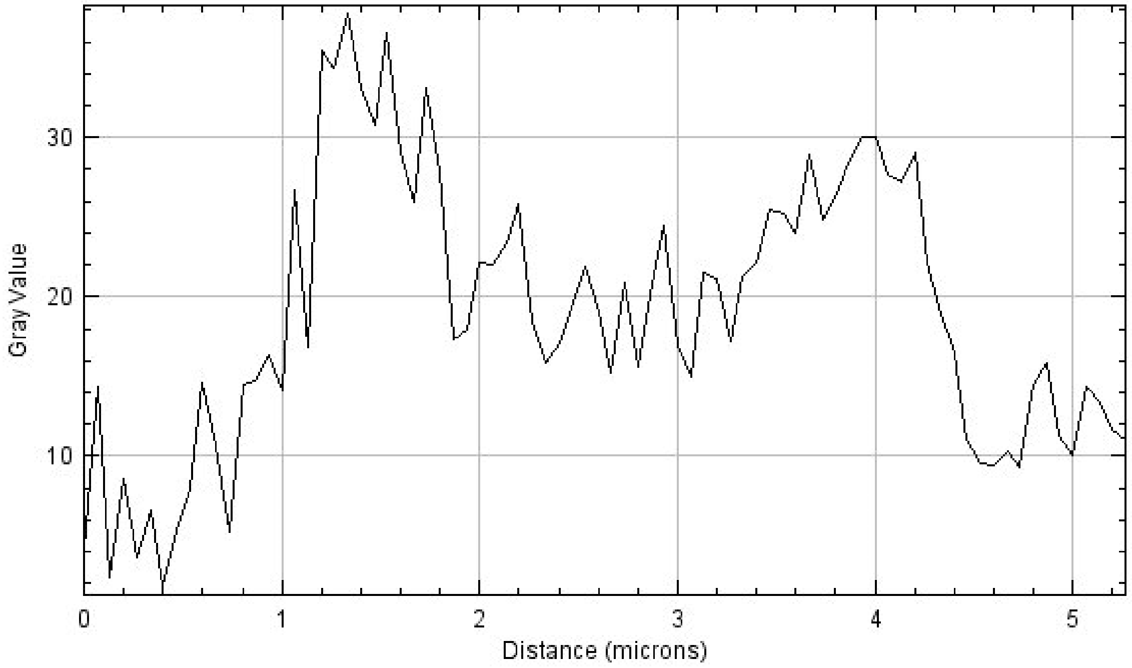

Figure S452 – Intensity profile of line scan generated for cell 2, Figure S450.

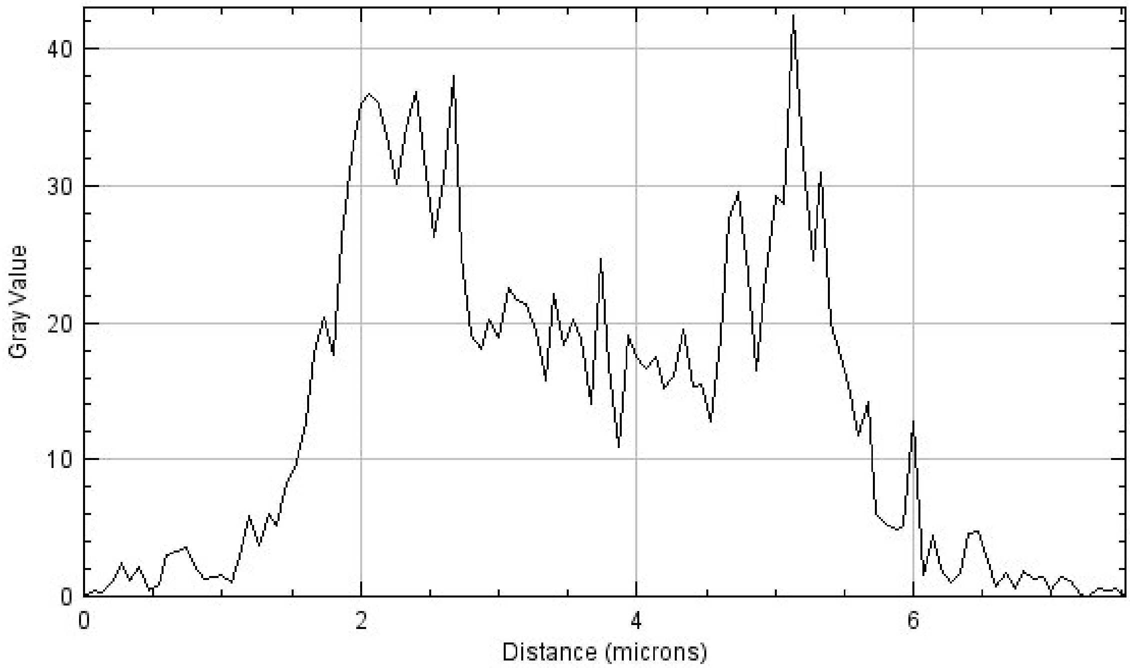

Figure S453 – Intensity profile of line scan generated for cell 3, Figure S450.

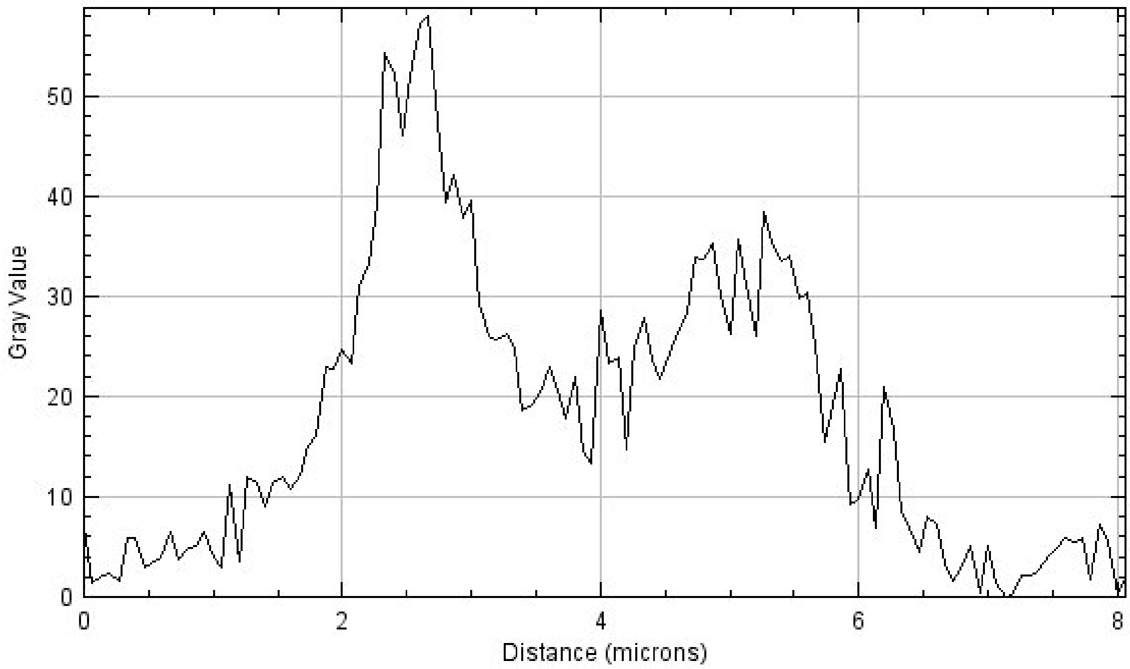

Figure S454 – Intensity profile of line scan generated for cell 4, Figure S450.

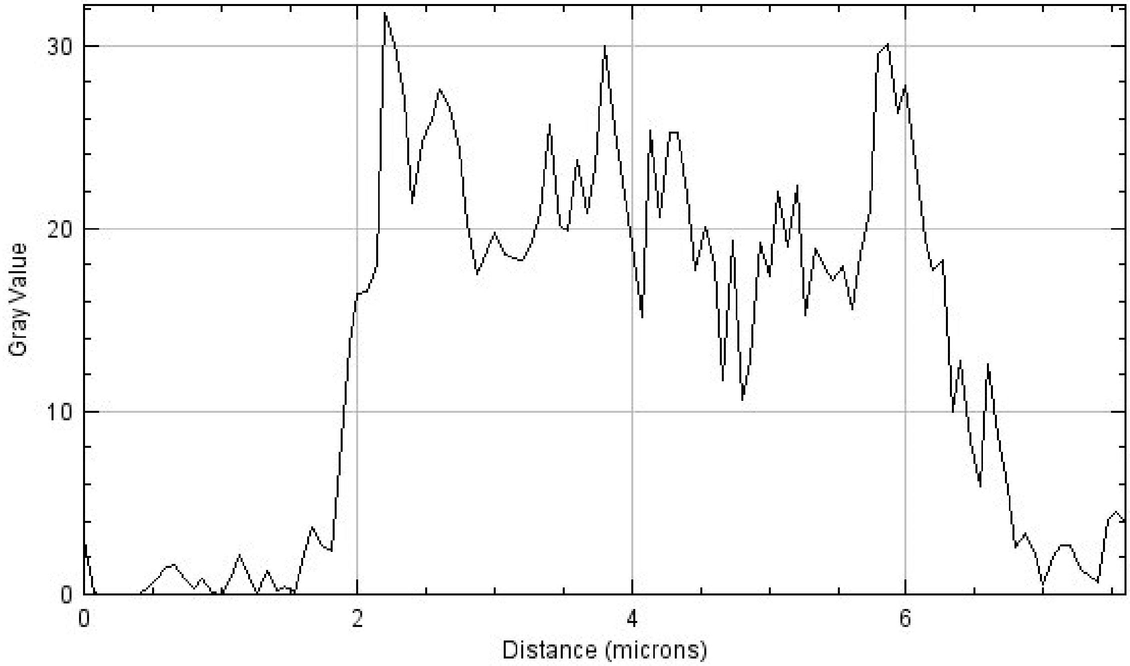

Figure S455 – Intensity profile of line scan generated for cell 5 , Figure S450.

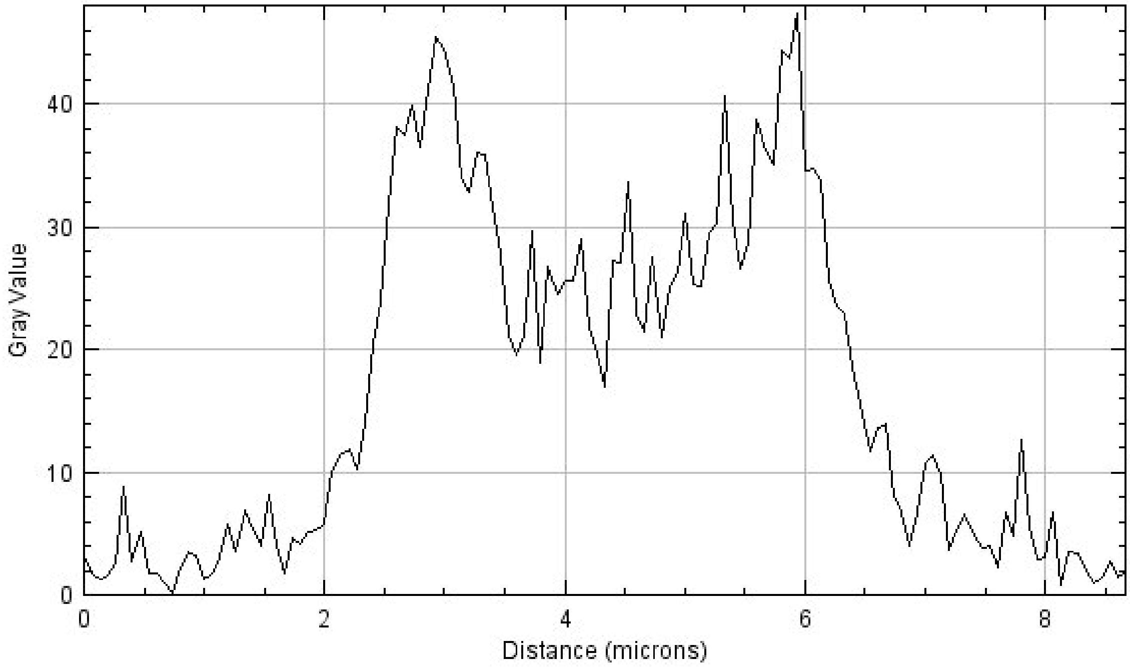

Figure S456 – Intensity profile of line scan generated for cell 6, Figure S450.

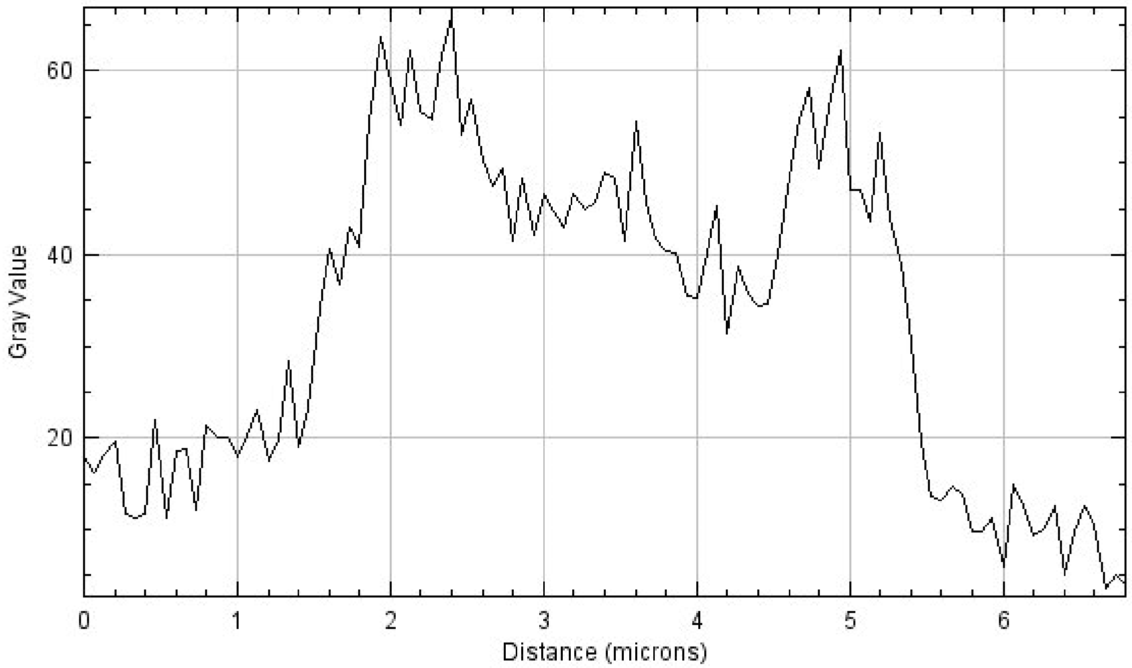

Figure S457 – Intensity profile of line scan generated for cell 7, Figure S450.

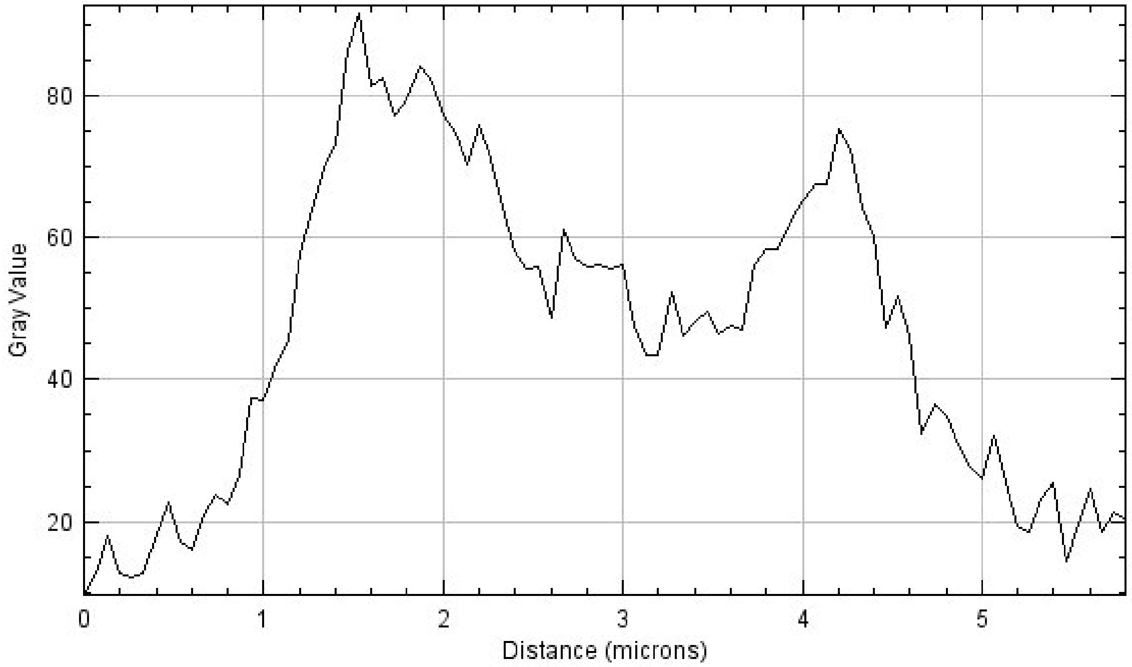

Figure S458 – Intensity profile of line scan generated for cell 8, Figure S450.

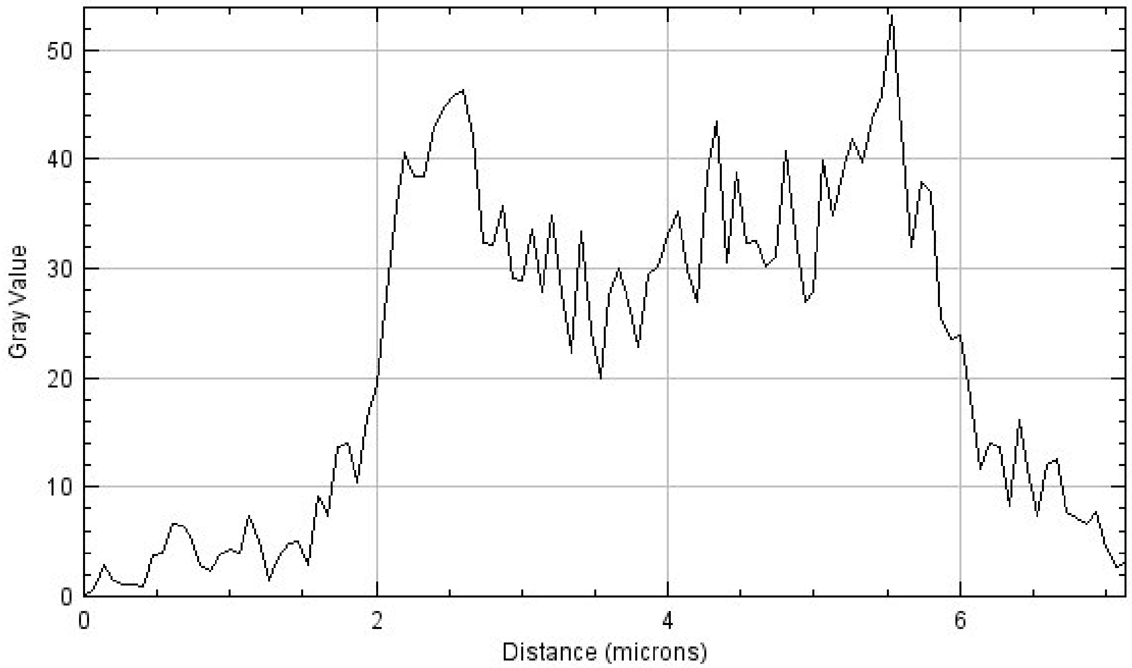

Figure S459 – Intensity profile of line scan generated for cell 9, Figure S450.

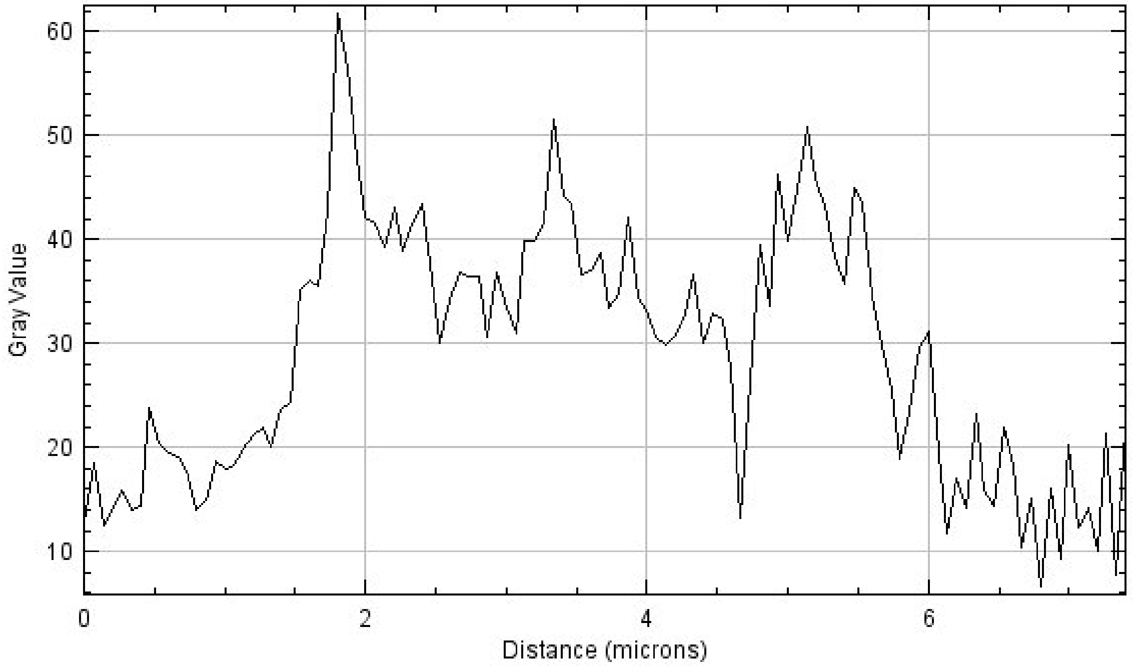

Figure S460 – Intensity profile of line scan generated for cell 10, Figure S450.

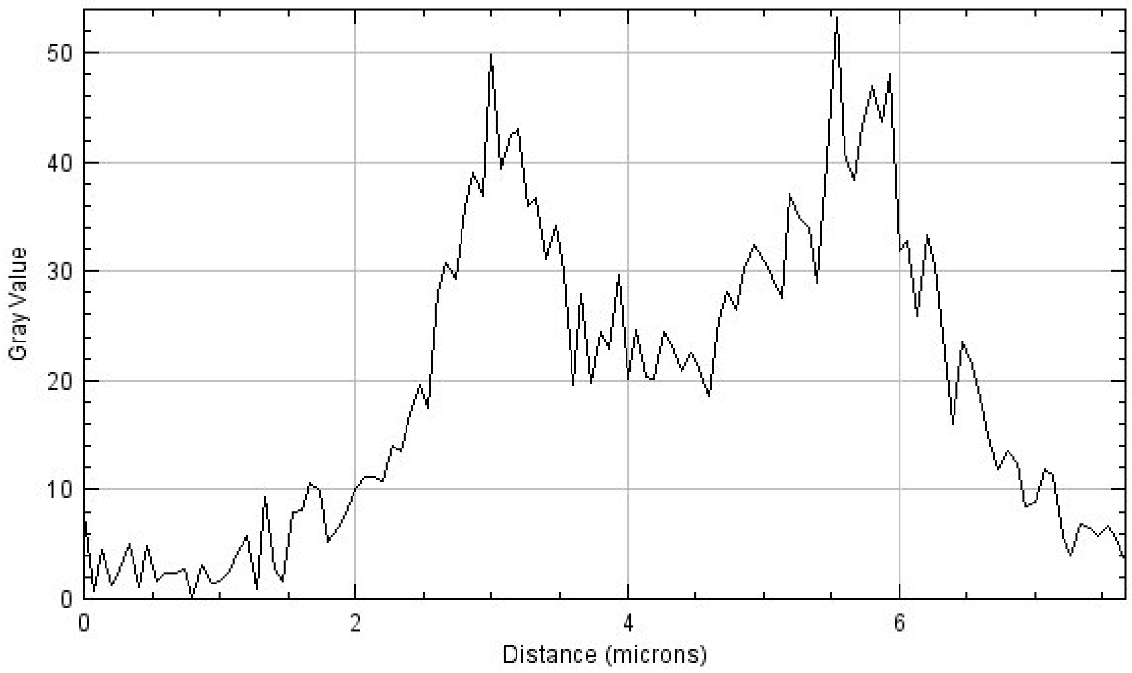

Figure S461 – Intensity profile of line scan generated for cell 11, Figure S450.

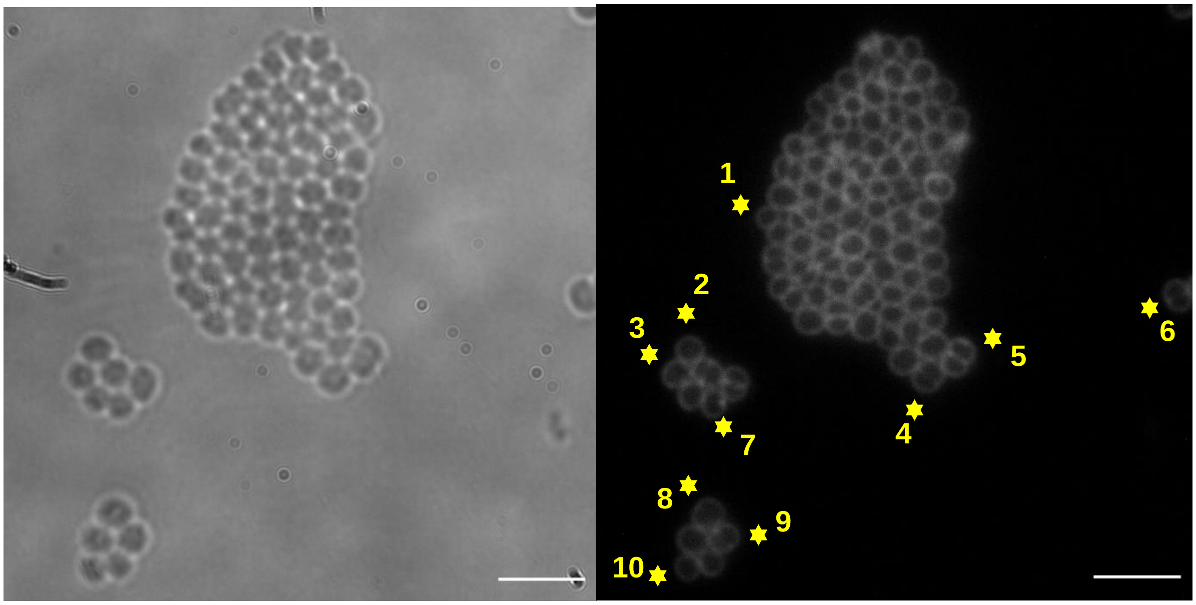

Figure S462 – MRSA transmitted image and fluorescence image at 605 nm used in fluorescence intensity calculations in the presence of **FM4-64** only at T = 30 minutes. Scale bar = 10 µM.

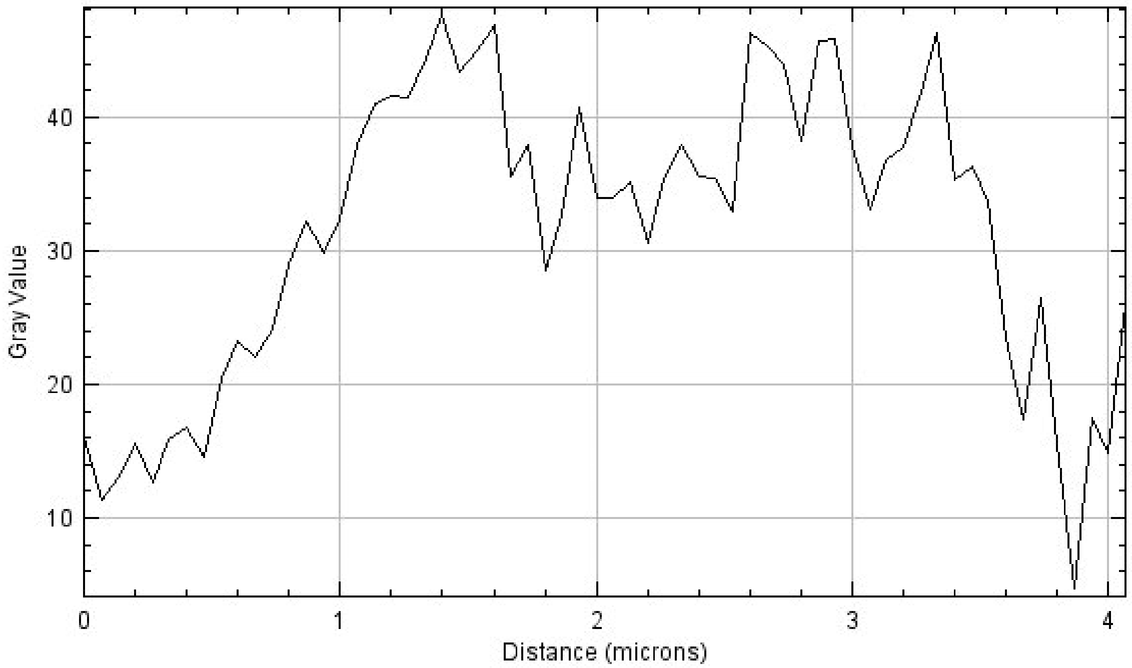

Figure S463 – Intensity profile of line scan generated for cell 1, Figure S462.

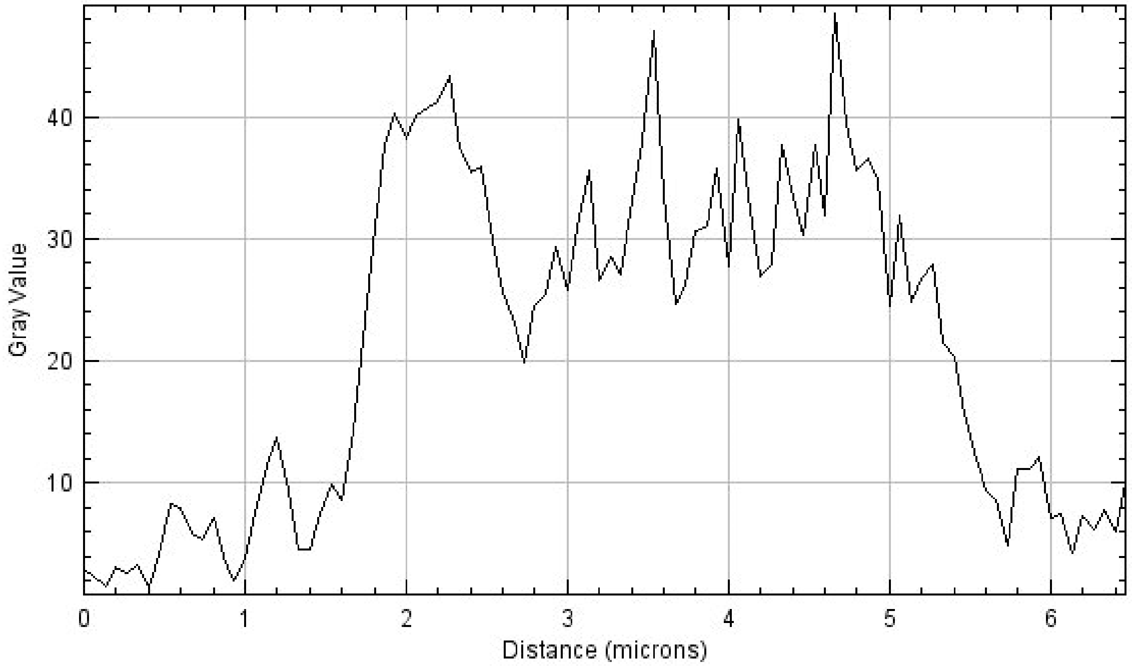

Figure S464 – Intensity profile of line scan generated for cell 2, Figure S462.

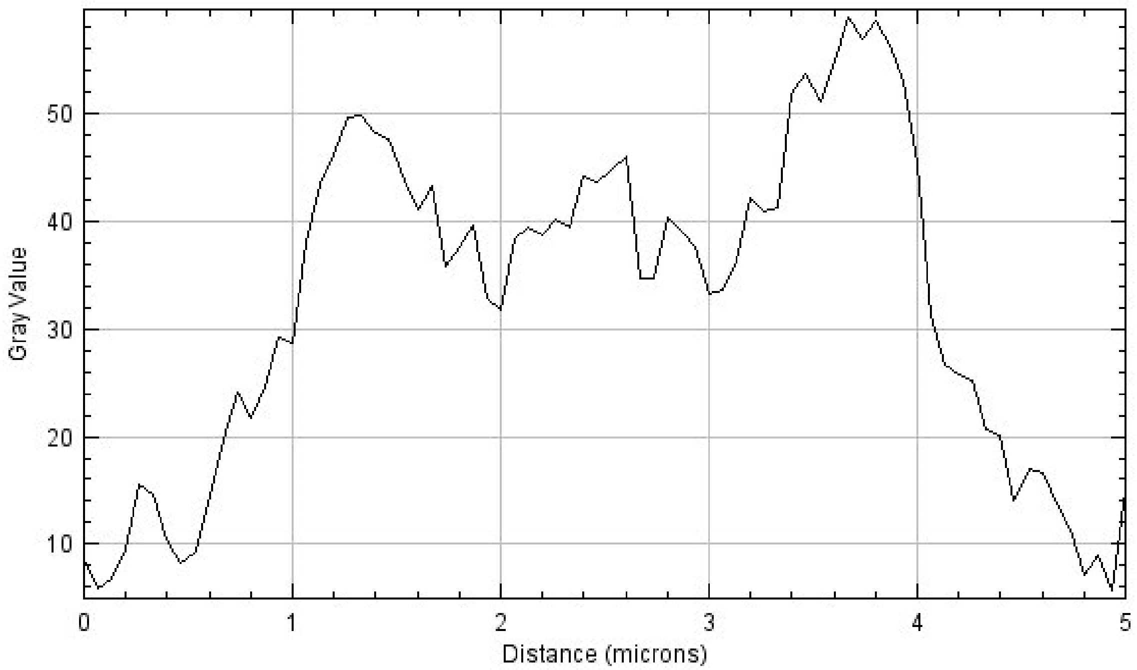

Figure S465 – Intensity profile of line scan generated for cell 3, Figure S462.

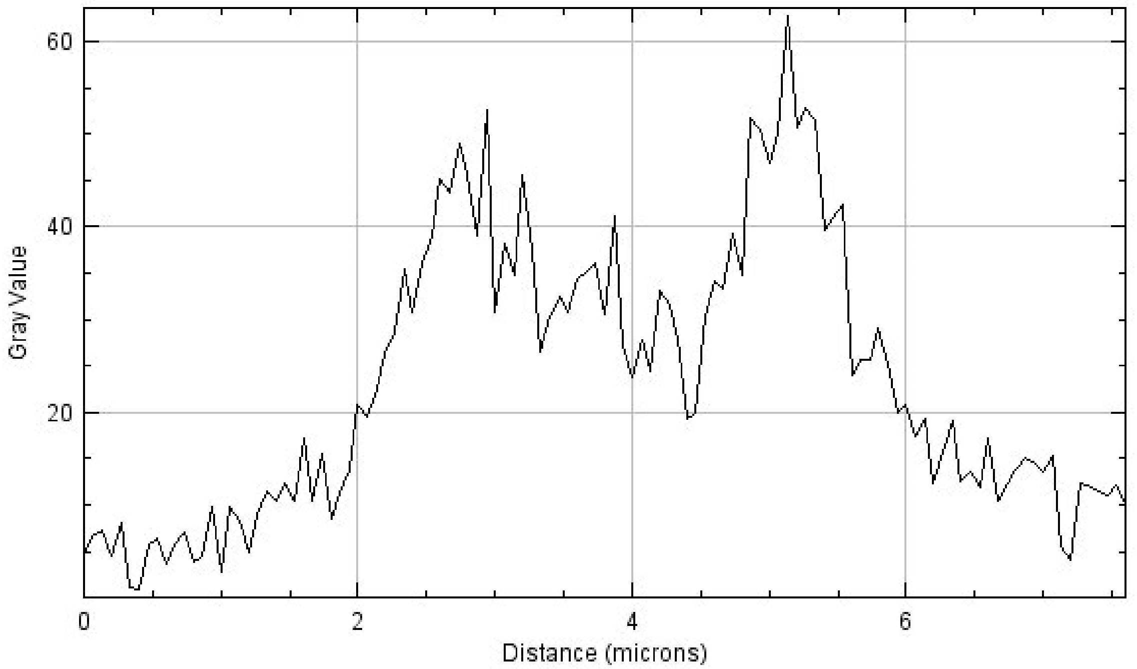

Figure S466 – Intensity profile of line scan generated for cell 4, Figure S462.

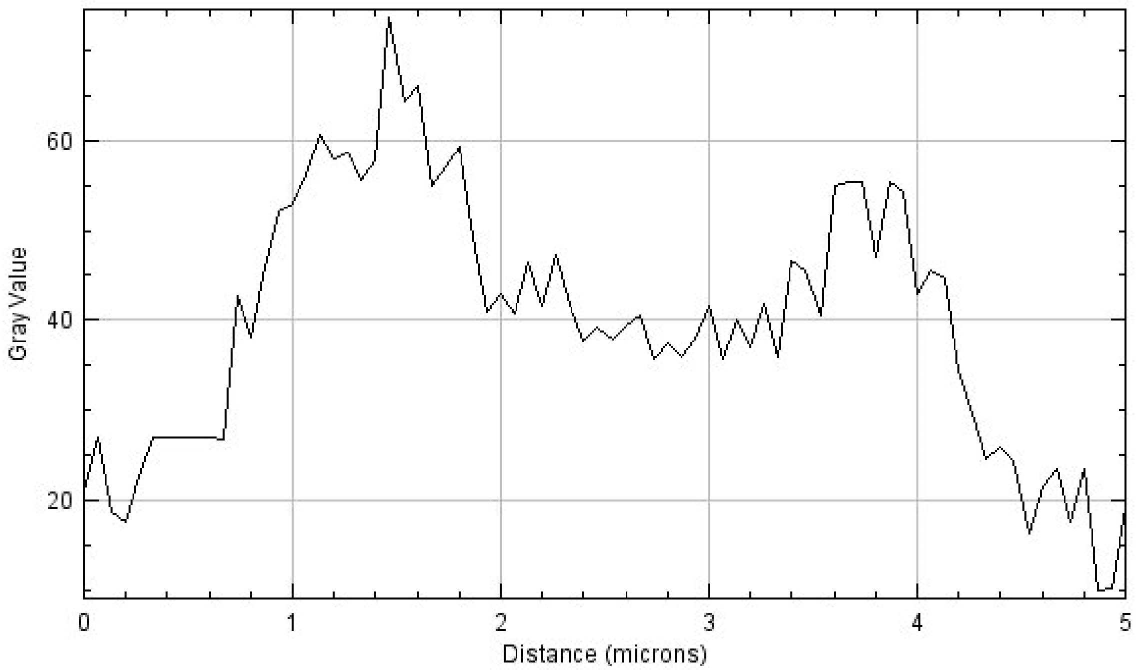

Figure S467 – Intensity profile of line scan generated for cell 5, Figure S462.

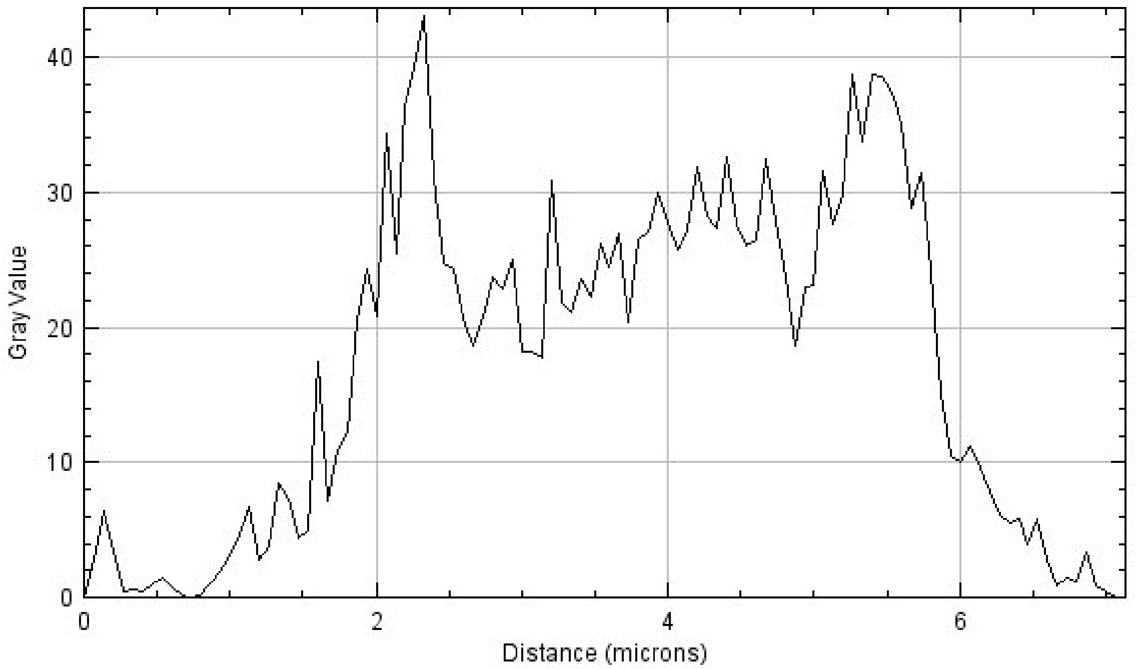

Figure S468 – Intensity profile of line scan generated for cell 6, Figure S462.

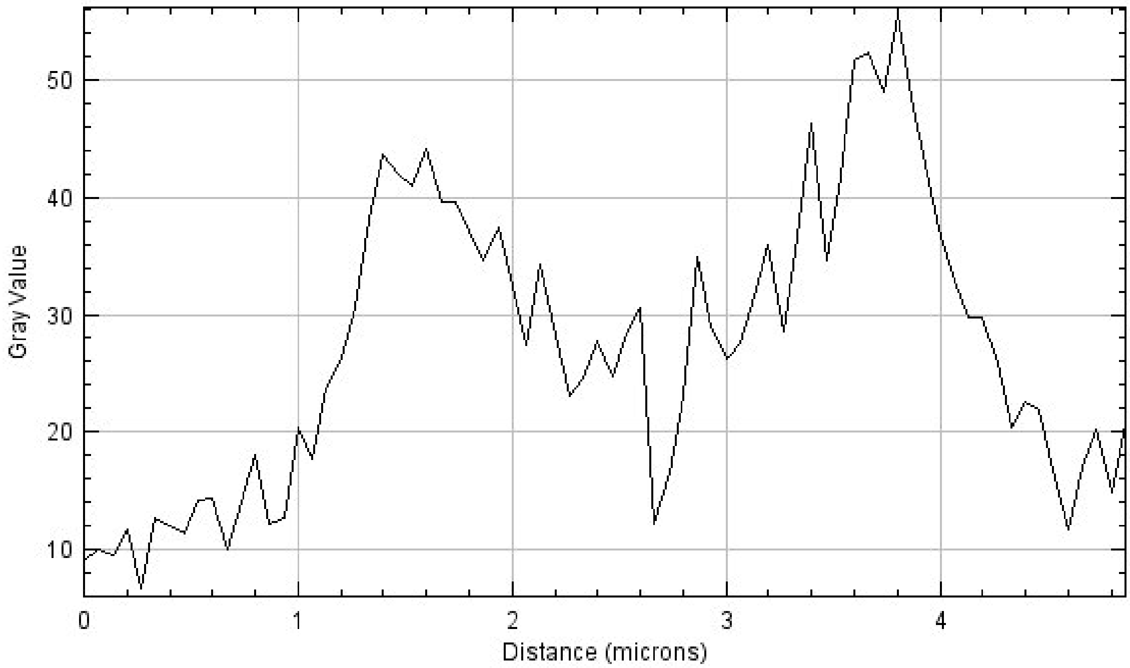

Figure S469 – Intensity profile of line scan generated for cell 7, Figure S462.

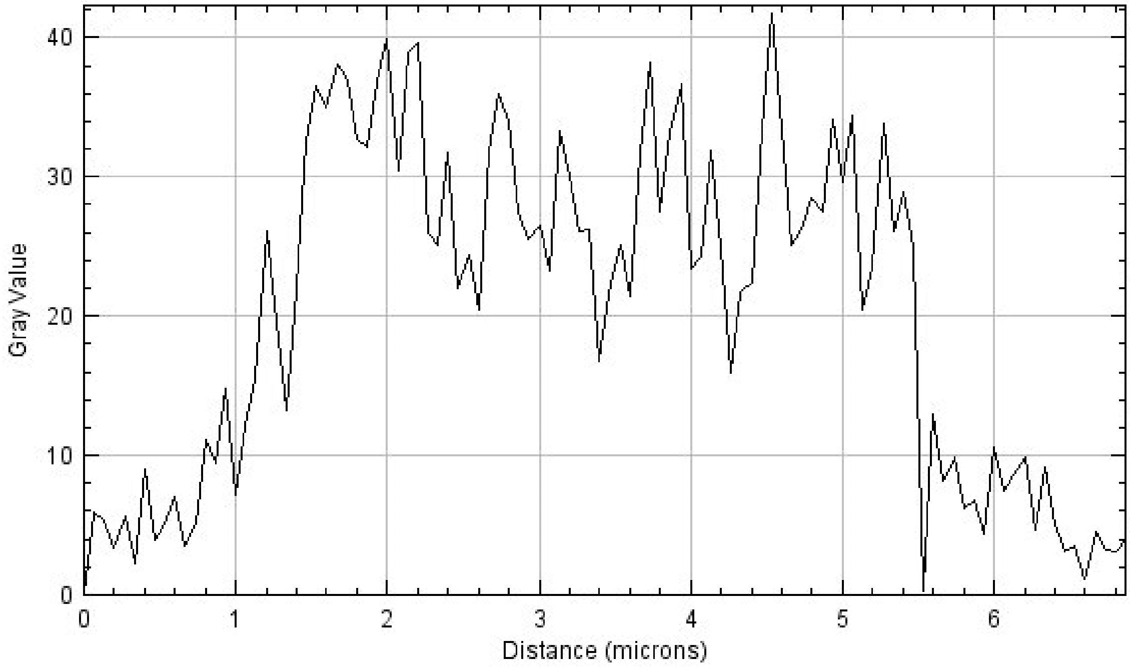

Figure S470 – Intensity profile of line scan generated for cell 8, Figure S462.

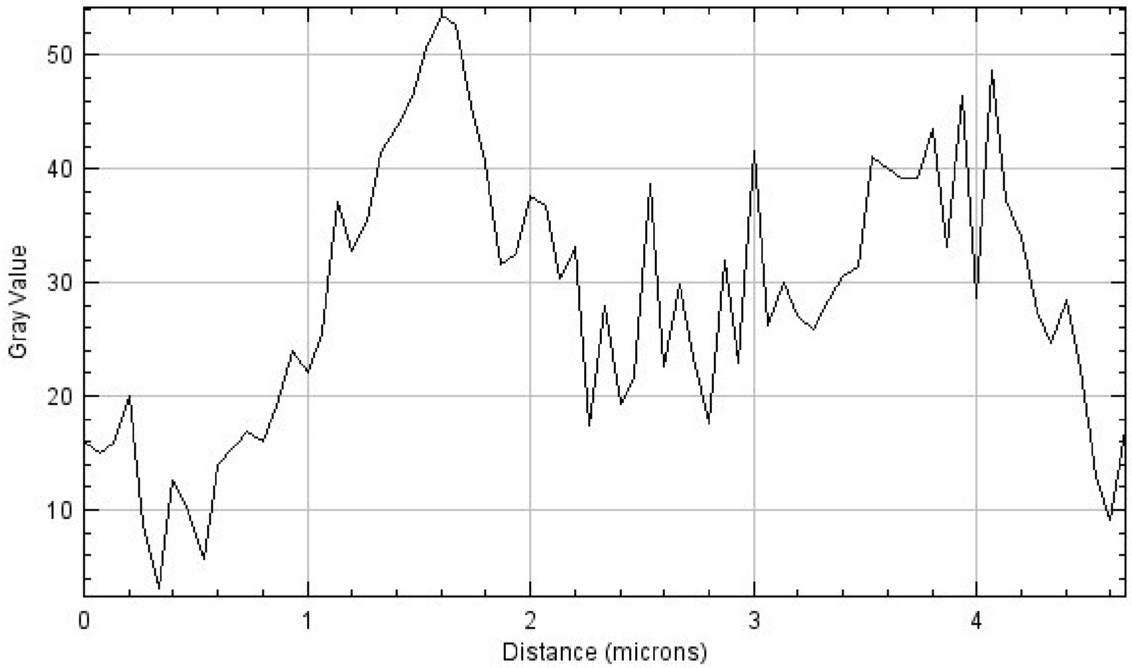

Figure S471 – Intensity profile of line scan generated for cell 9, Figure S462.

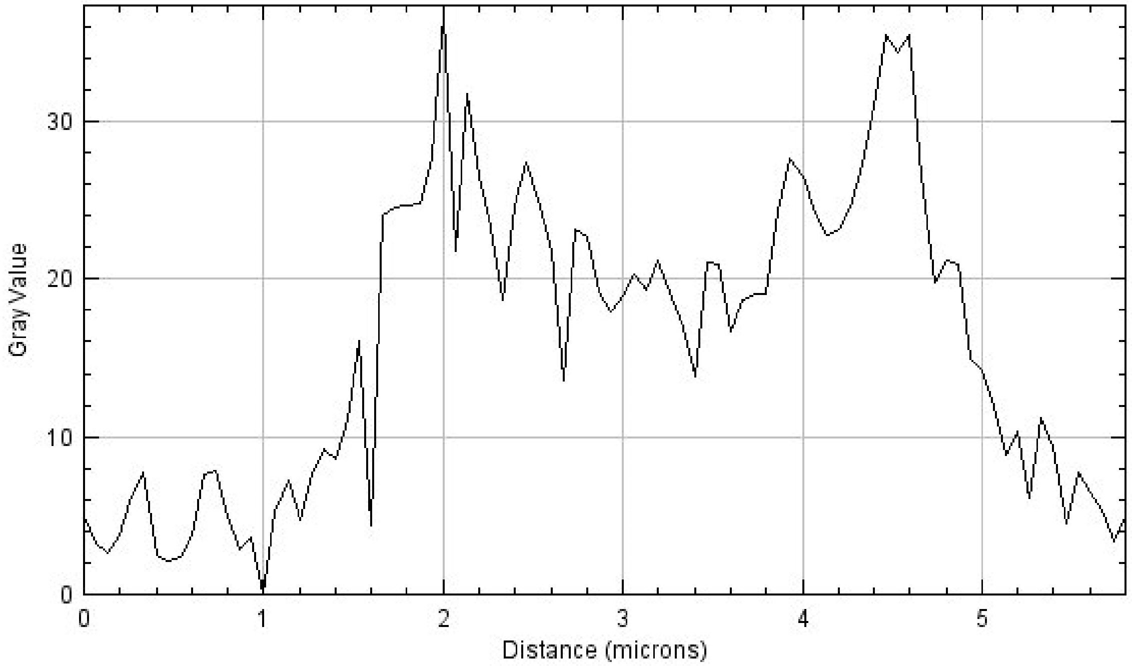

Figure S472 – Intensity profile of line scan generated for cell 10, Figure S462.

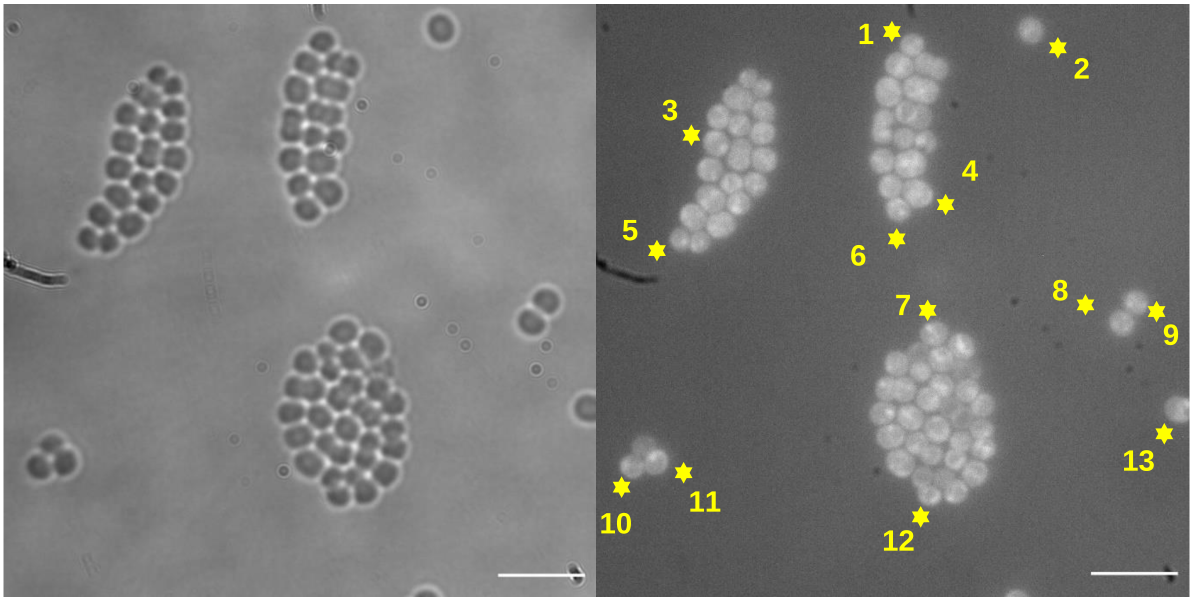

Figure S473 – MRSA transmitted image and fluorescence image at 450 nm used in fluorescence intensity calculations in the presence of both compound **39** and **FM4-64** at T = 30 minutes. Scale bar = 10µM

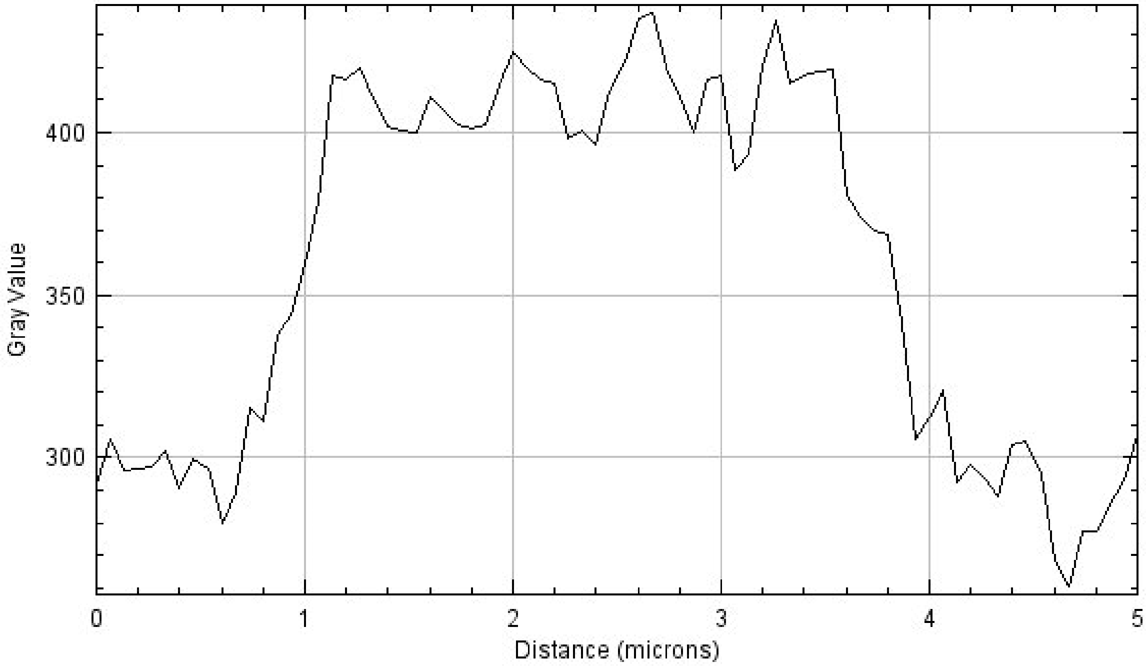

Figure S474 – Intensity profile of line scan generated for cell 1, Figure S473.

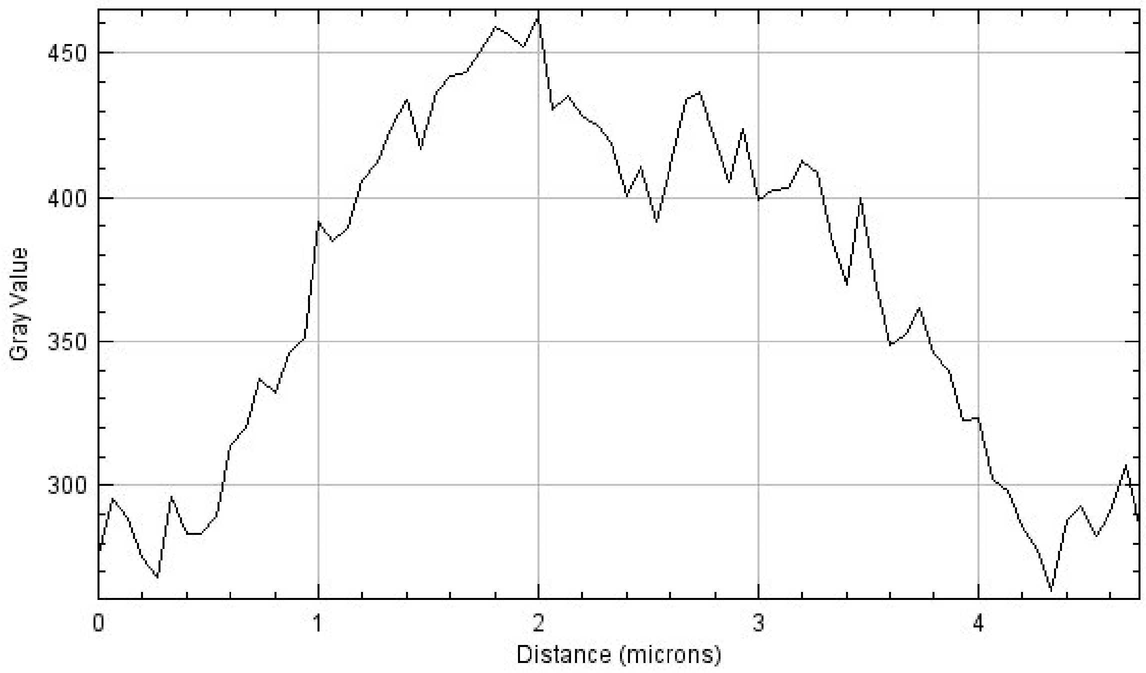

Figure S475 – Intensity profile of line scan generated for cell 2, Figure S473.

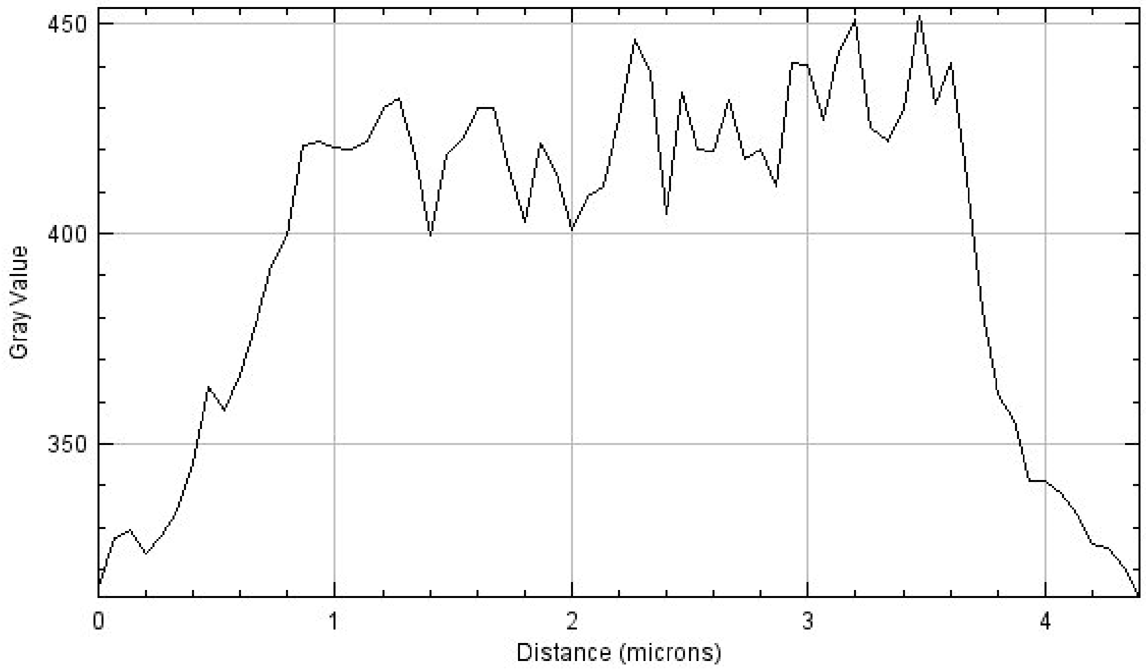

Figure S476 – Intensity profile of line scan generated for cell 3, Figure S473.

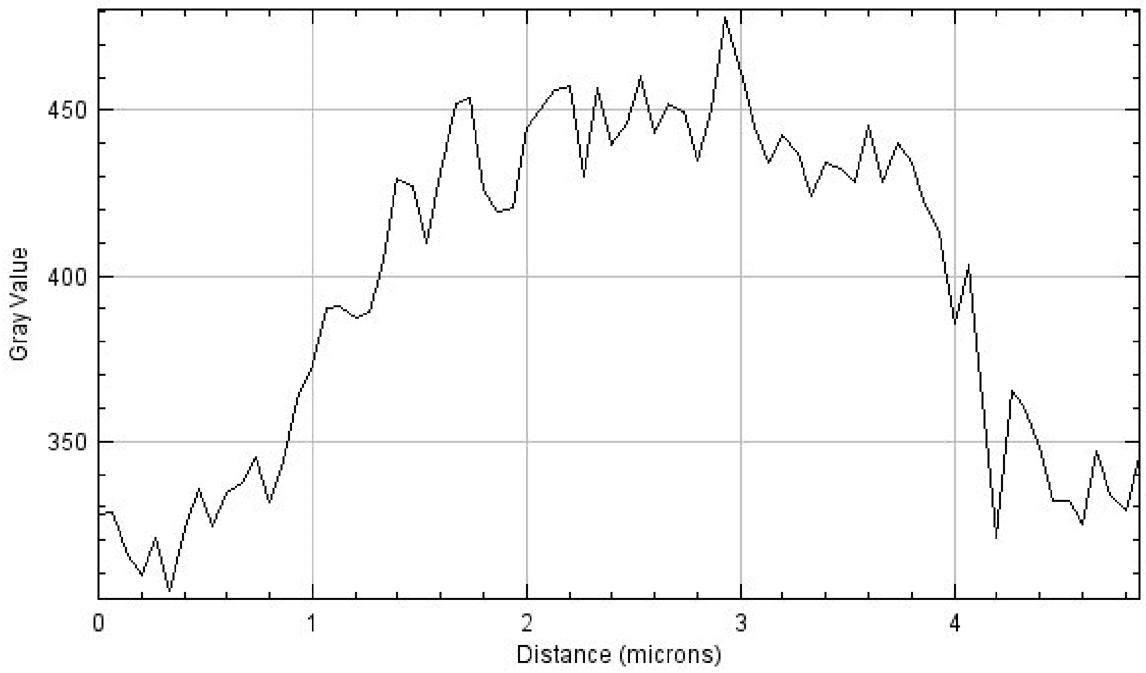

Figure S477 – Intensity profile of line scan generated for cell 4, Figure S473.

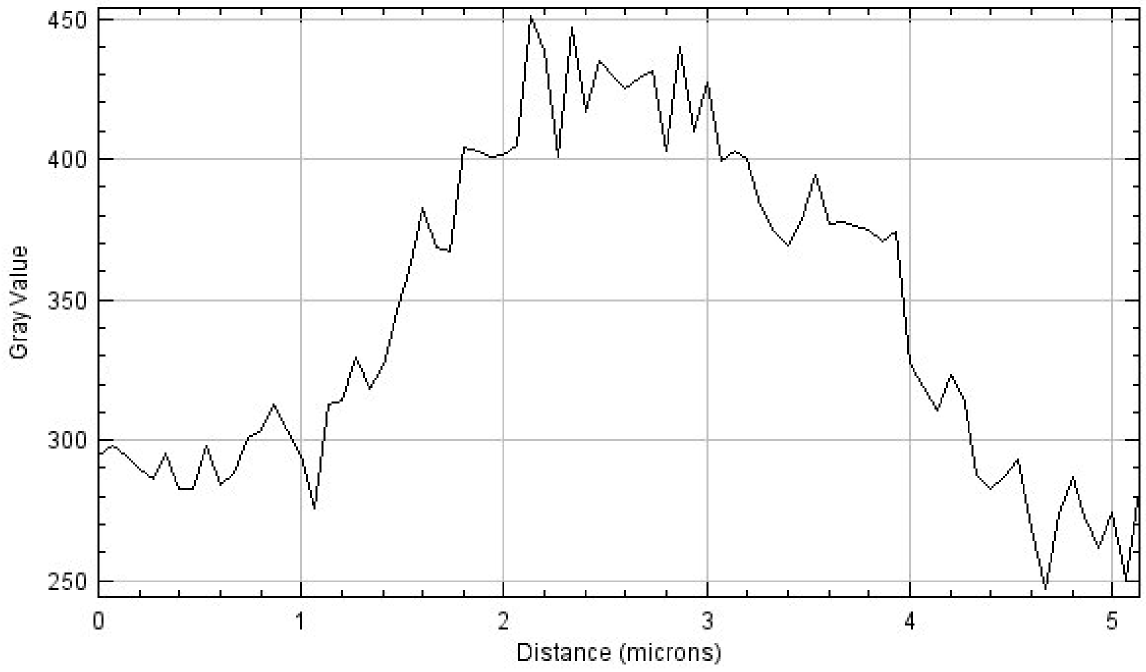

Figure S478 – Intensity profile of line scan generated for cell 5, Figure S473.

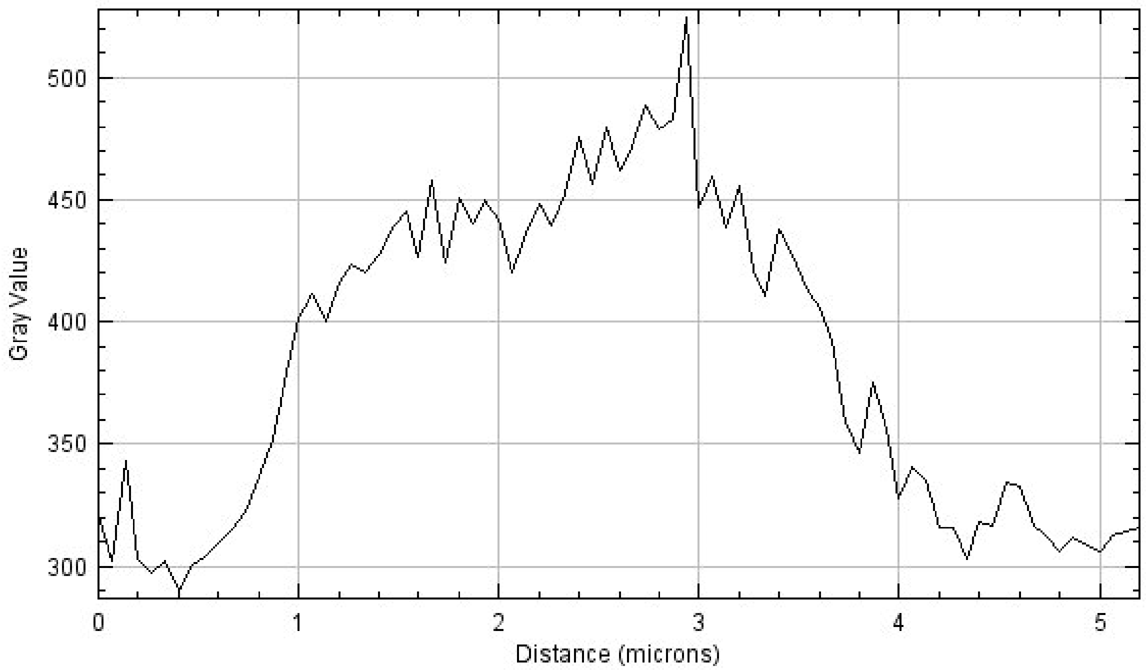

Figure S479 – Intensity profile of line scan generated for cell 6, Figure S473.

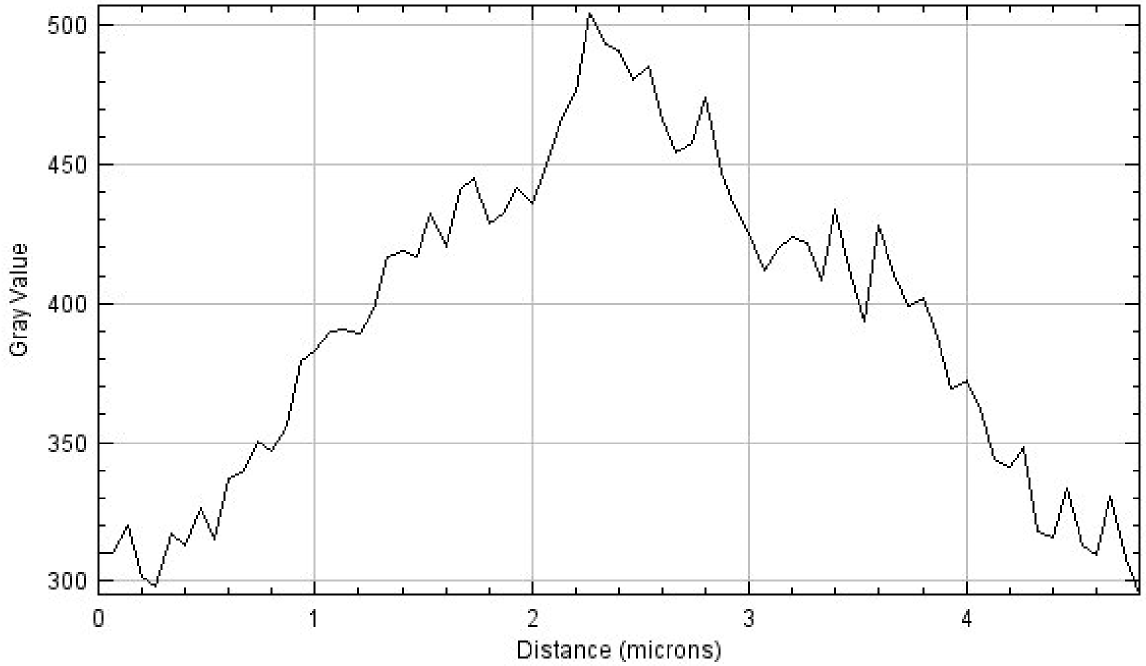

Figure S480 – Intensity profile of line scan generated for cell 7, Figure S473.

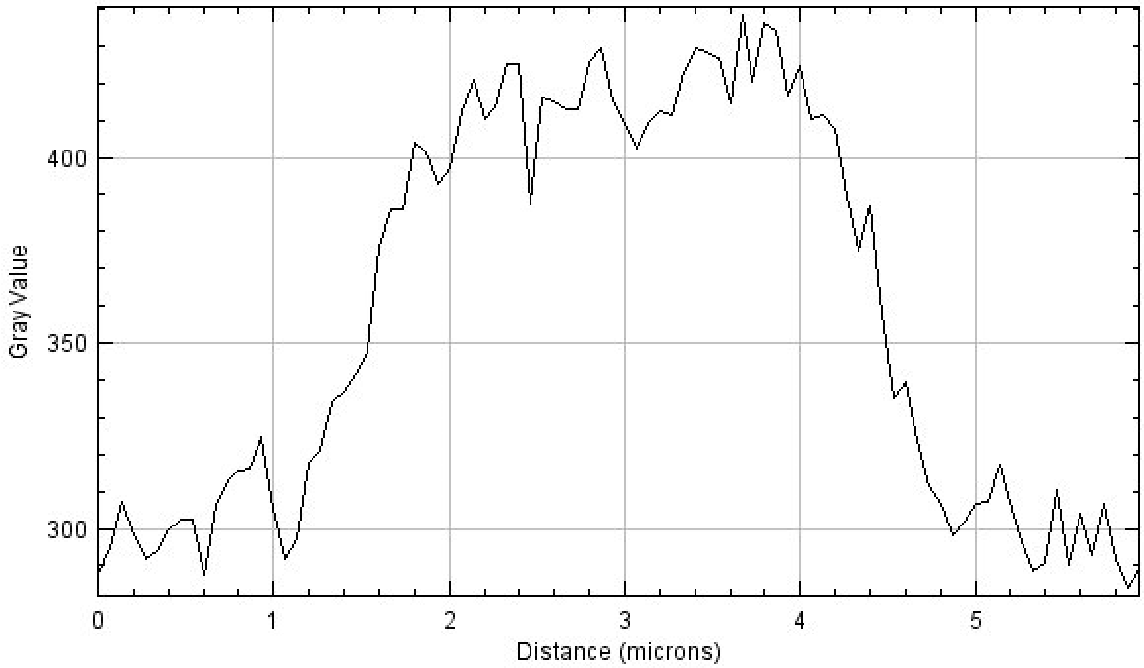

Figure S481 – Intensity profile of line scan generated for cell 8, Figure S473.

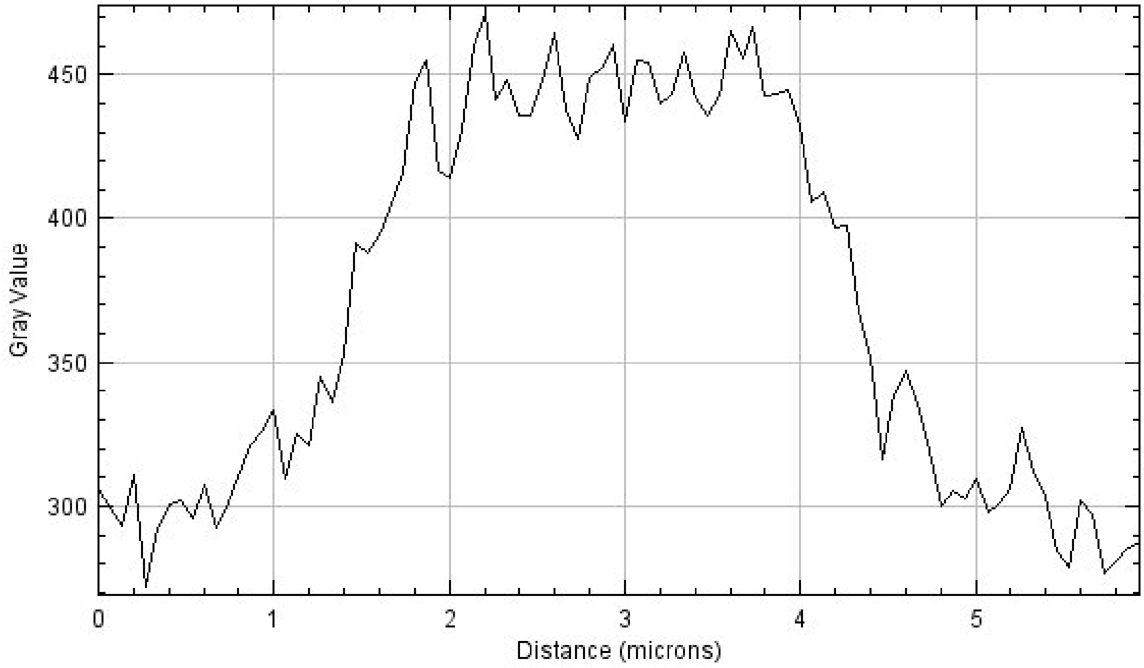

Figure S482 – Intensity profile of line scan generated for cell 9, Figure S473.

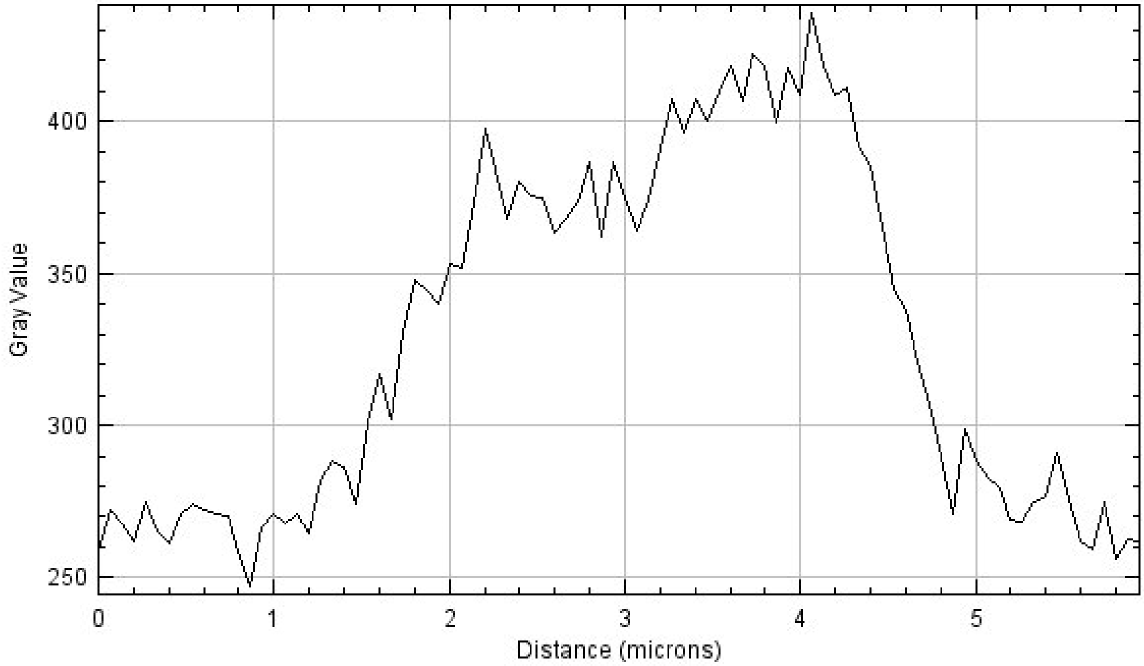

Figure S483 – Intensity profile of line scan generated for cell 1, Figure S473.0

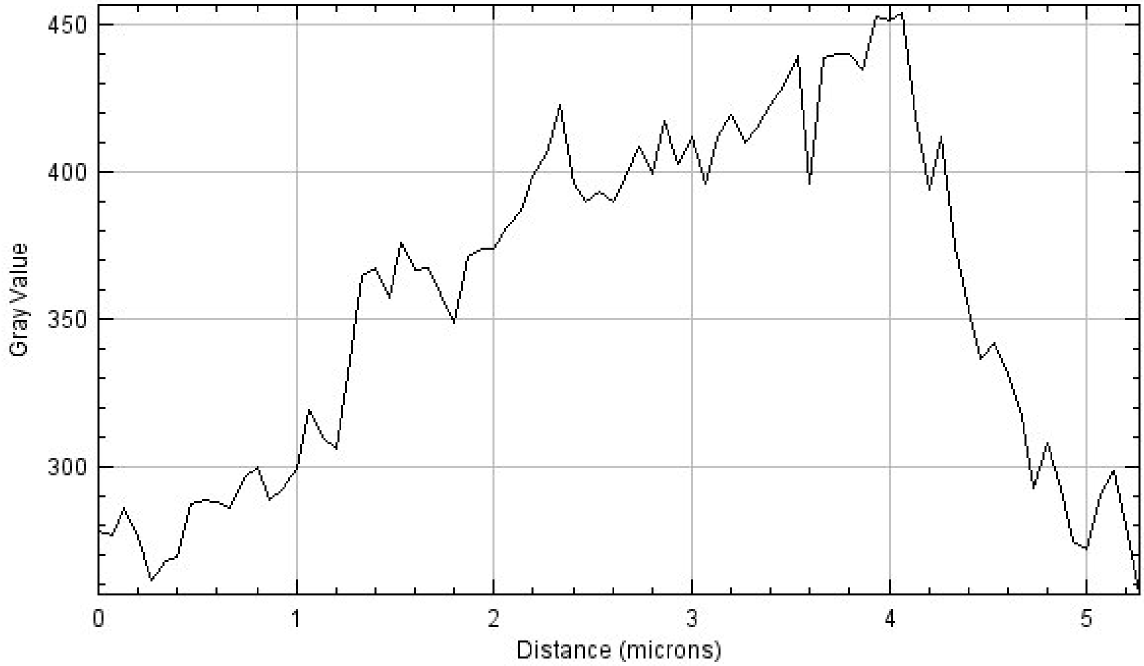

Intensity profile of line scan generated for cell 11, Figure S473.

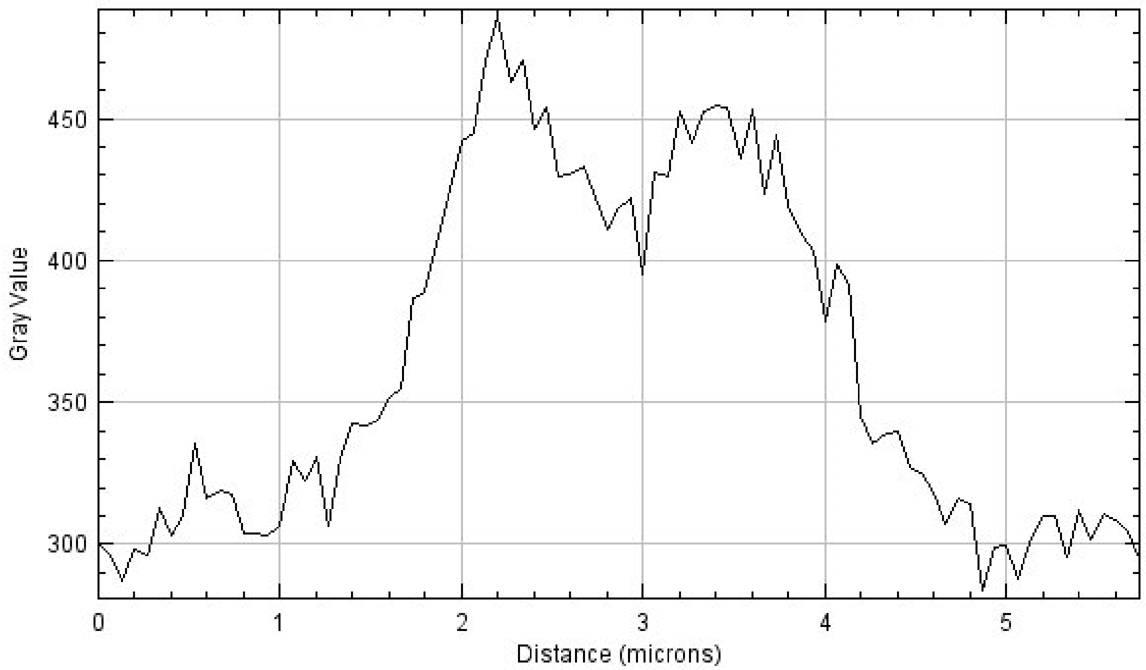

Figure S484 – Intensity profile of line scan generated for cell 12, Figure S473.

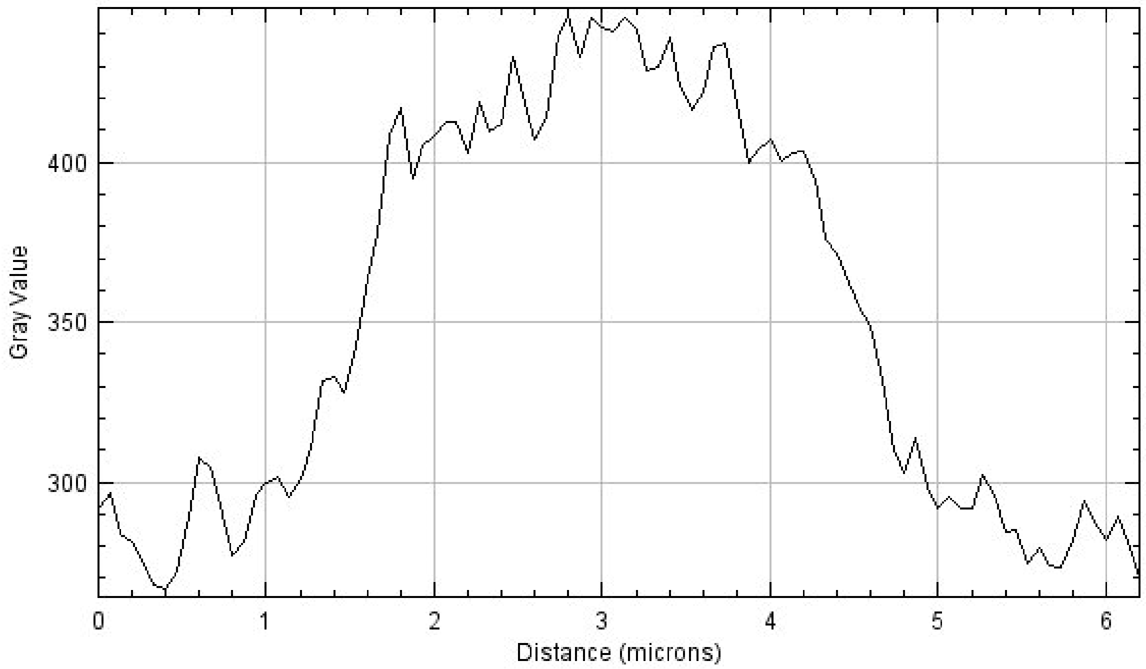

Figure S485 – Intensity profile of line scan generated for cell 13, Figure S473.

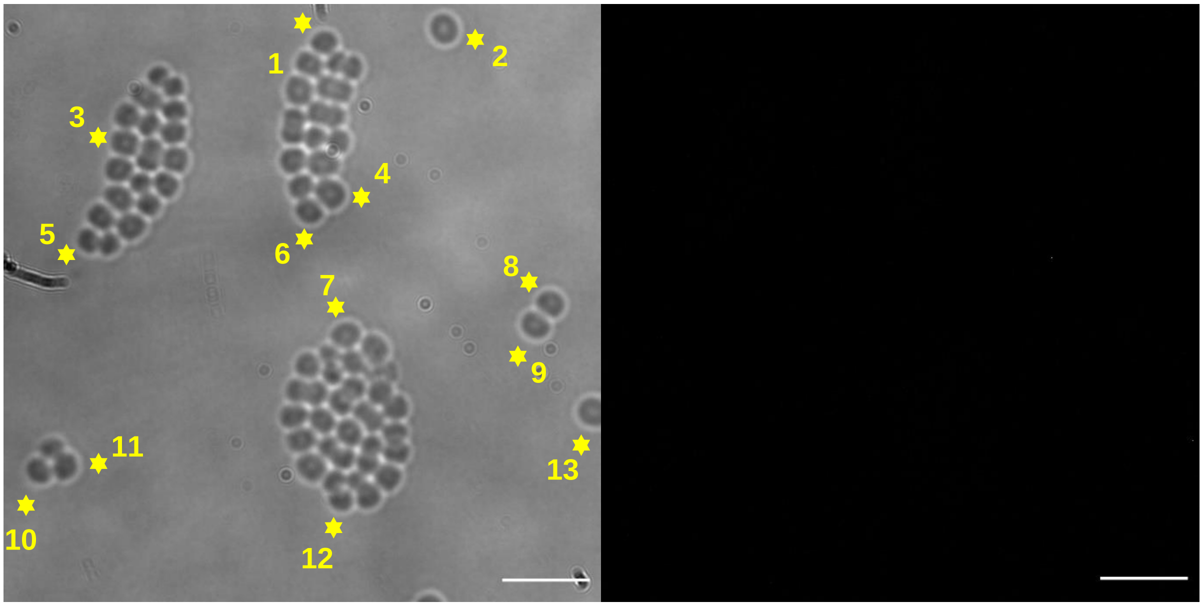

Figure S486 – MRSA transmitted image and fluorescence image at 605 nm used in fluorescence intensity calculations in the presence of both compound **39** and **FM4-64** at T = 30 minutes. Transmitted image used to locate cells on fluorescence image. Scale bar = 10 µM.

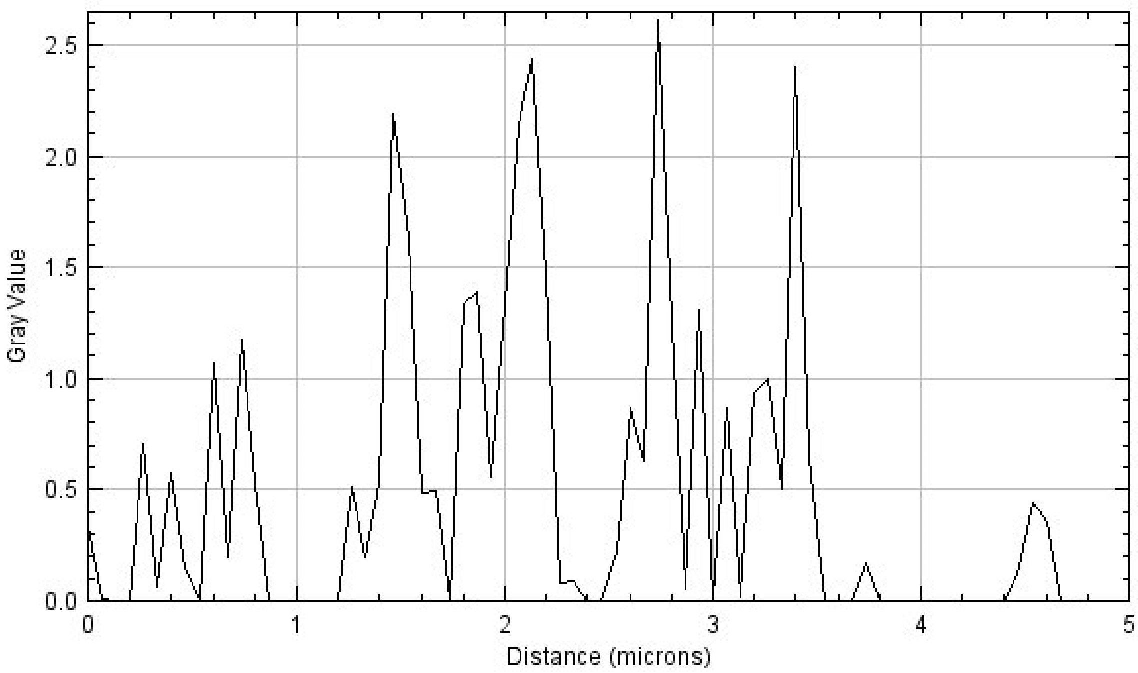

Figure S487 – Intensity profile of line scan generated for cell 1, Figure S486.

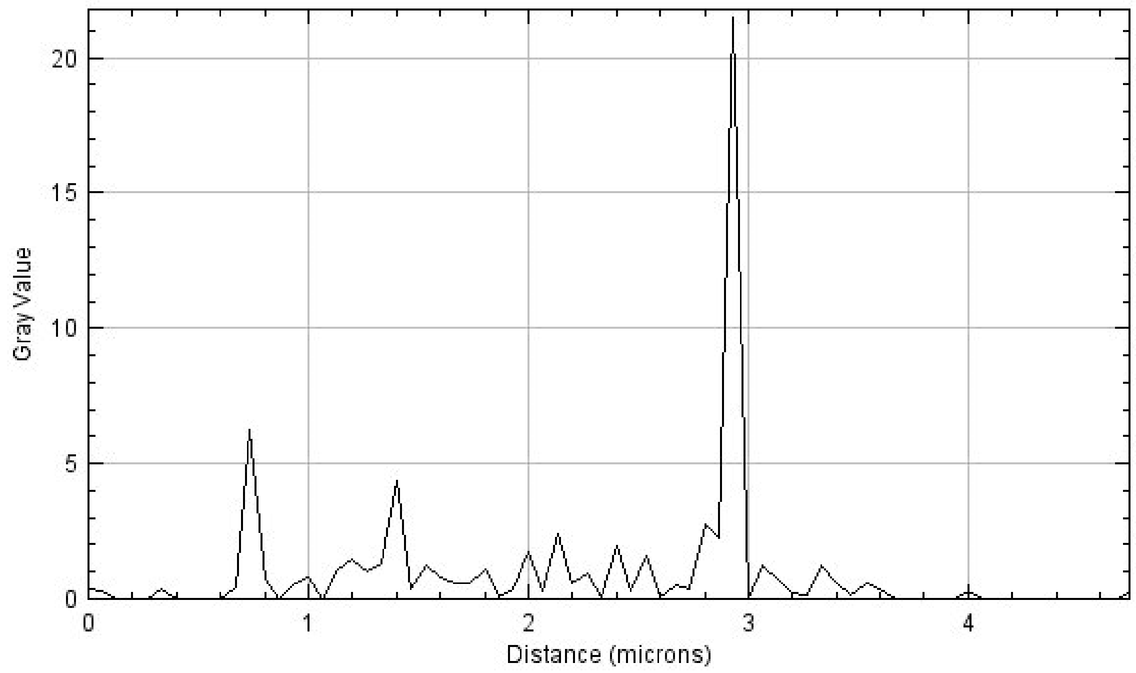

Figure S488 – Intensity profile of line scan generated for cell 2, Figure S486.

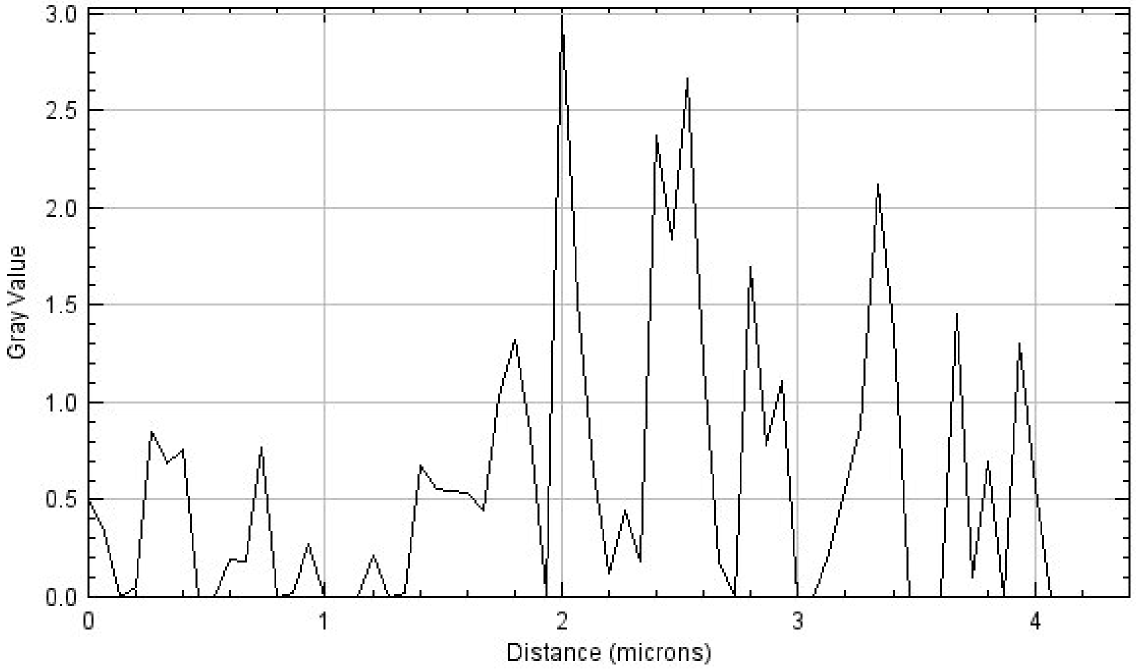

Figure S489 – Intensity profile of line scan generated for cell 3, Figure S486.

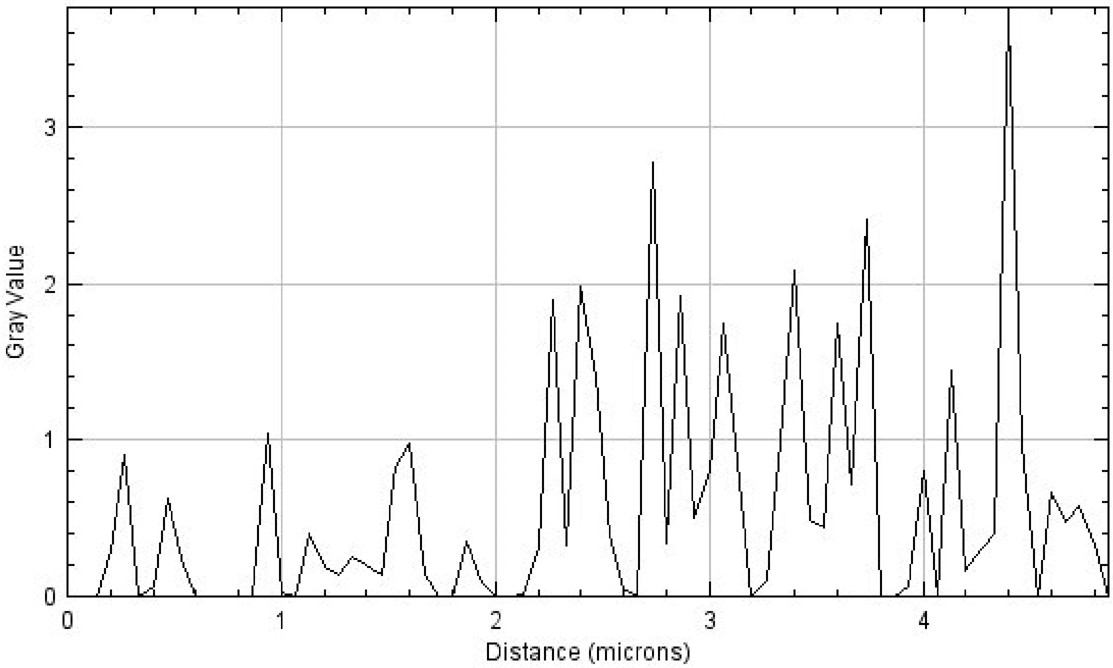

Figure S490 – Intensity profile of line scan generated for cell 4, Figure S486.

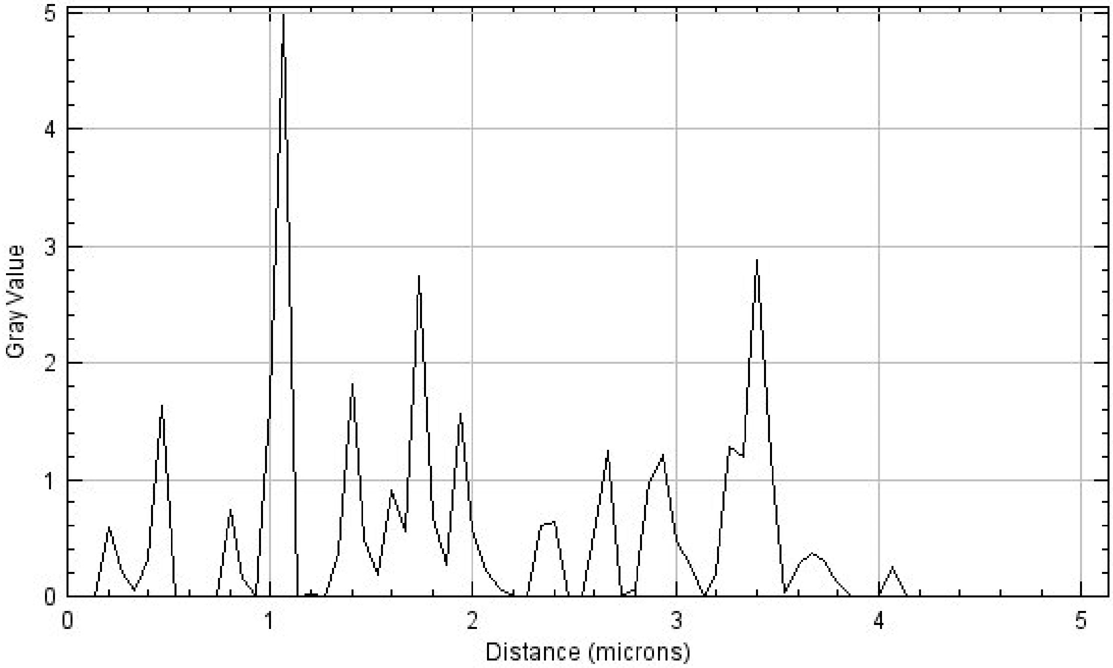

Figure S491 – Intensity profile of line scan generated for cell 5, Figure S486.

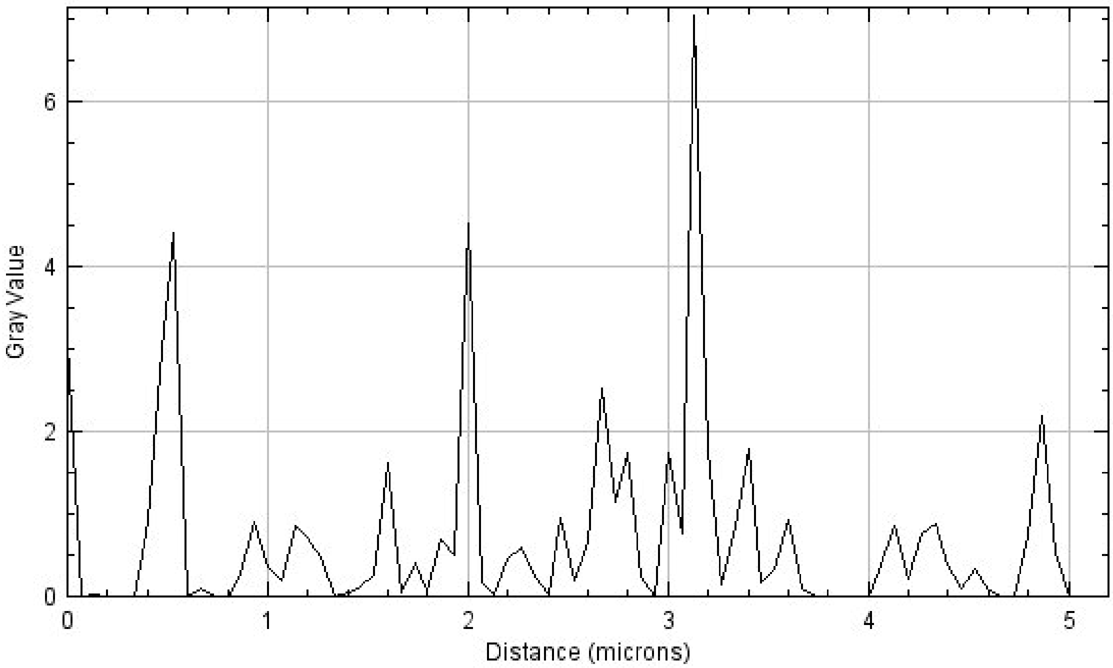

Figure S492 – Intensity profile of line scan generated for cell 6, Figure S486.

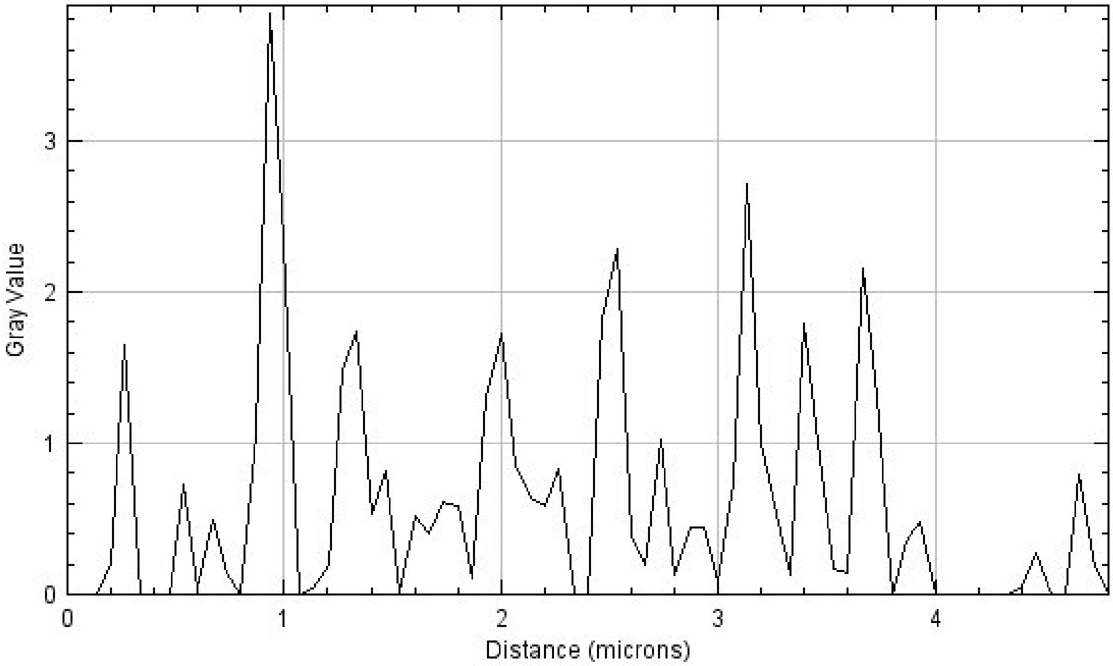

Figure S493 – Intensity profile of line scan generated for cell 7, Figure S486.

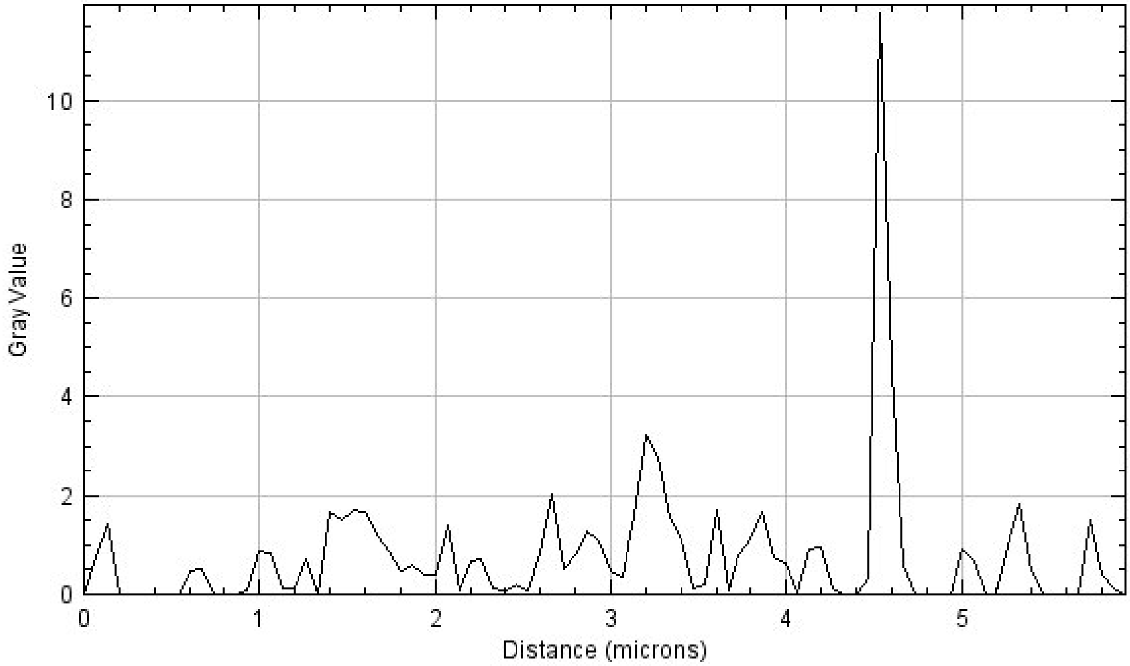

Figure S494 – Intensity profile of line scan generated for cell 8, Figure S486.

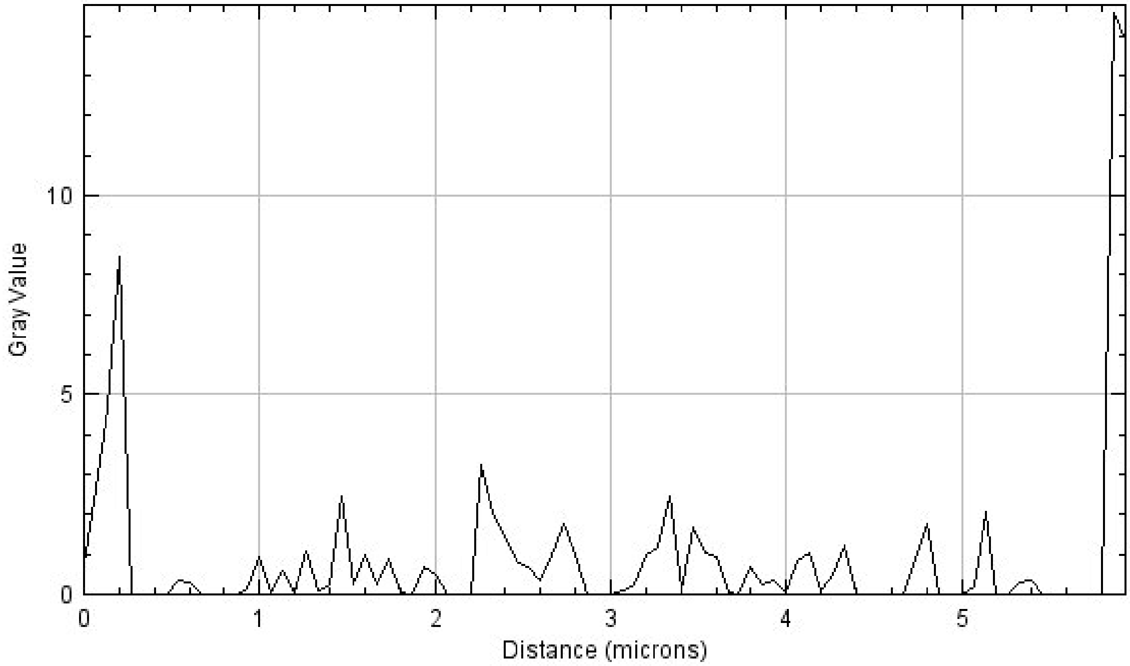

Figure S495 – Intensity profile of line scan generated for cell 9, Figure S486.

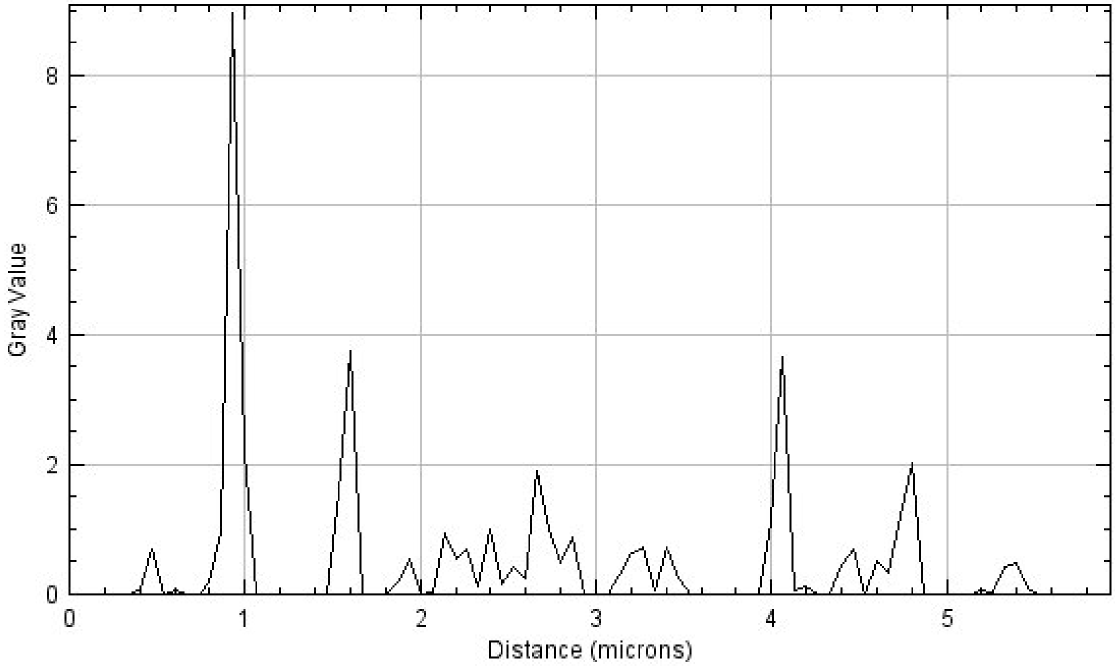

Figure S496 – Intensity profile of line scan generated for cell 10, Figure S486.

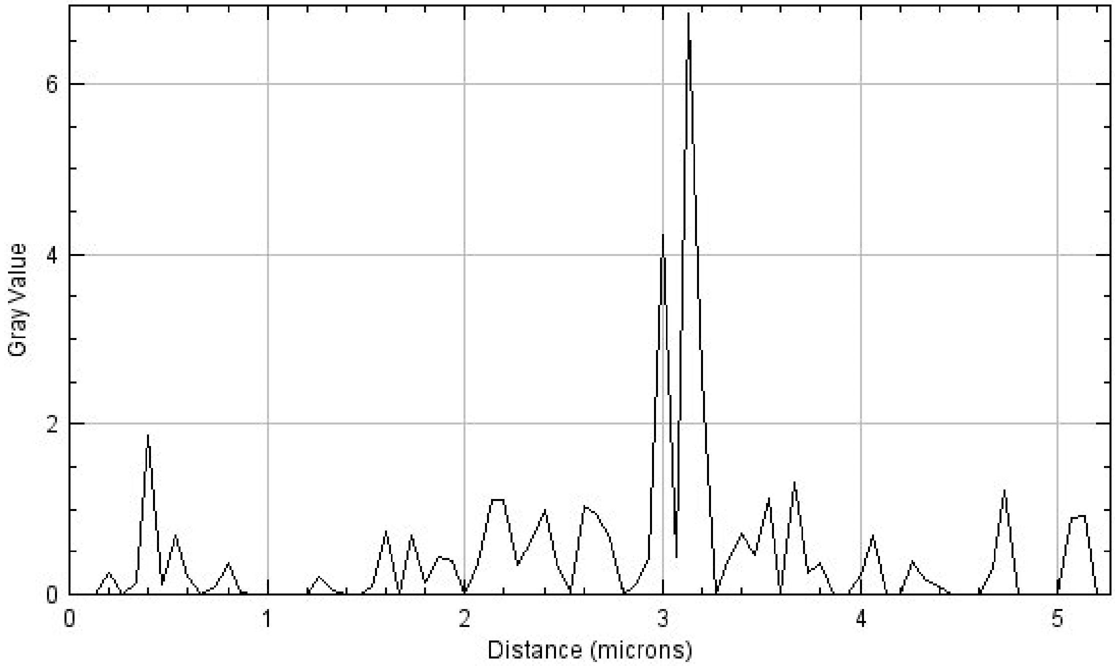

Figure S497 – Intensity profile of line scan generated for cell 11, Figure S486.

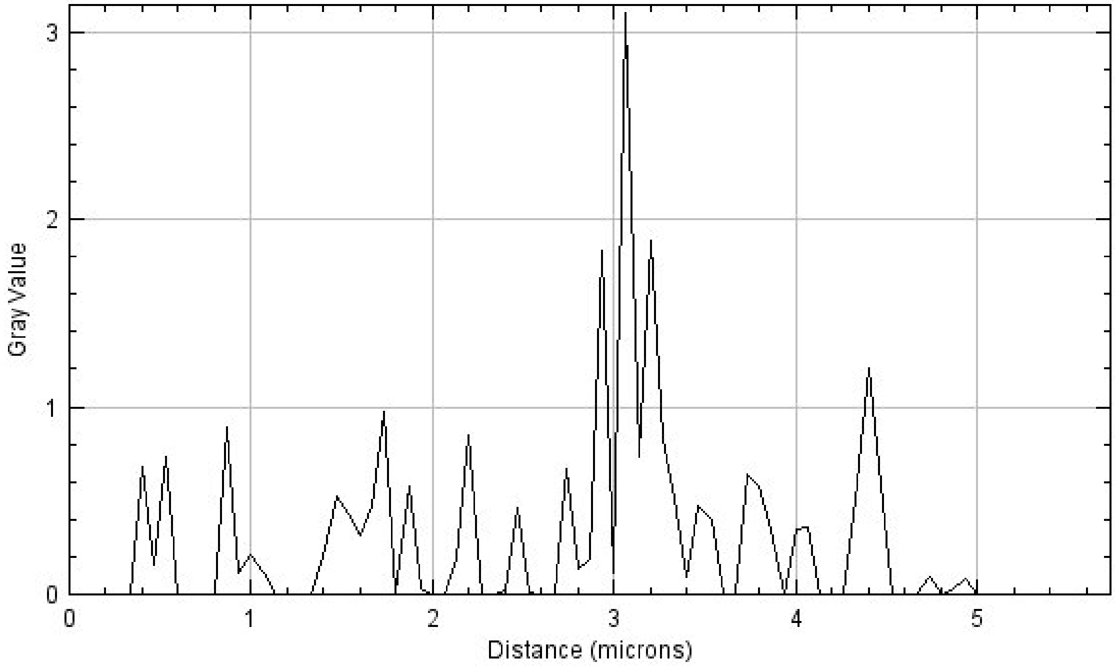

Figure S498 – Intensity profile of line scan generated for cell 12, Figure S486.

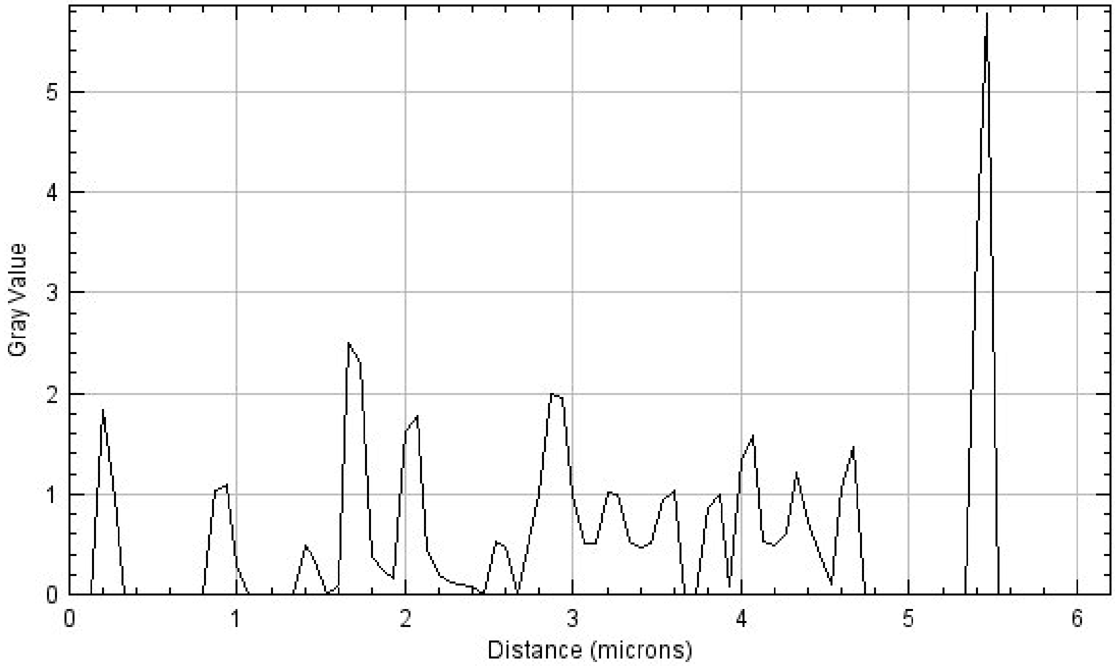

Figure S499 – Intensity profile of line scan generated for cell 13, Figure S486.

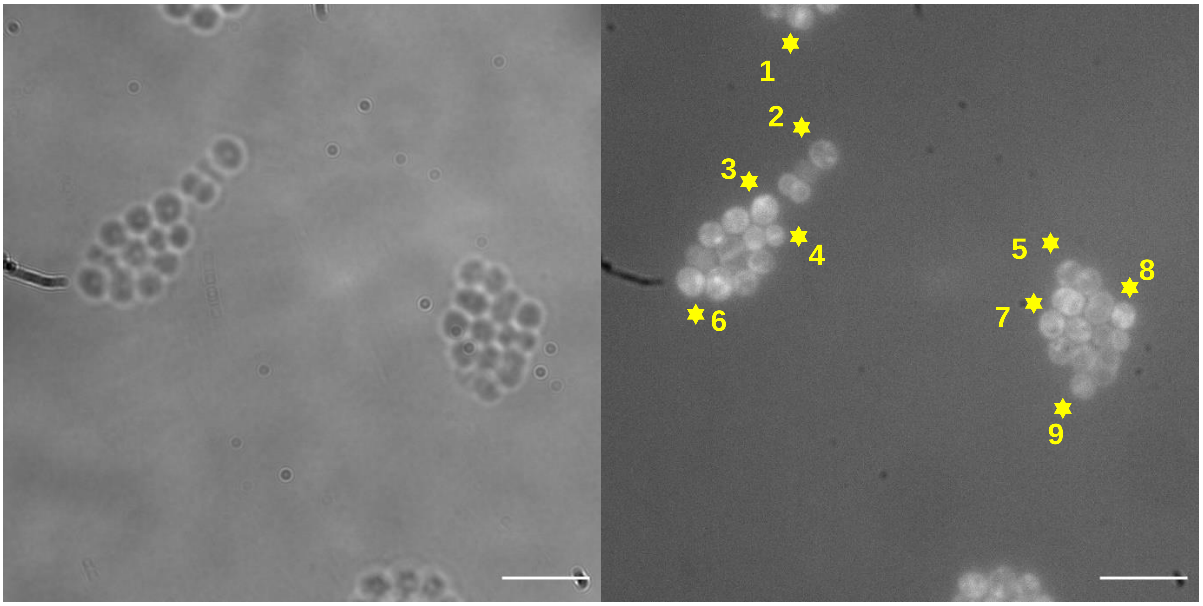

Figure S500 – MRSA transmitted image and fluorescence image at 450 nm used in fluorescence intensity calculations in the presence of both compound **39** and **FM4-64** at T = 30 minutes. Scale bar = 10 μM.

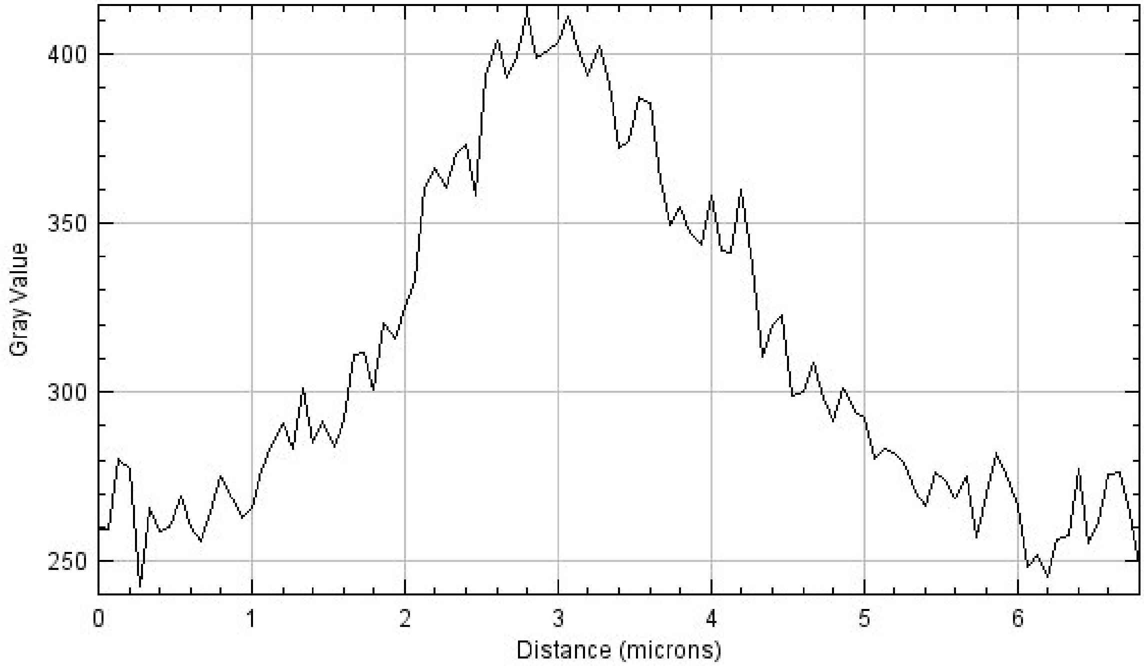

Figure S501 – Intensity profile of line scan generated for cell 1, Figure S500.

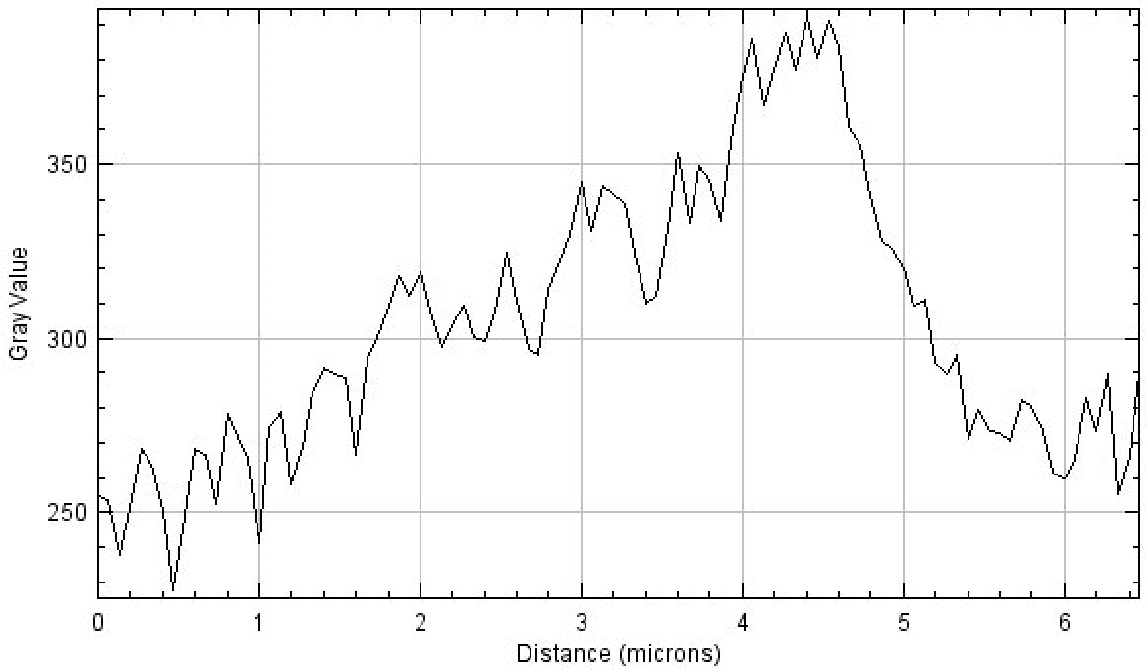

Figure S502 – Intensity profile of line scan generated for cell 2, Figure S500.

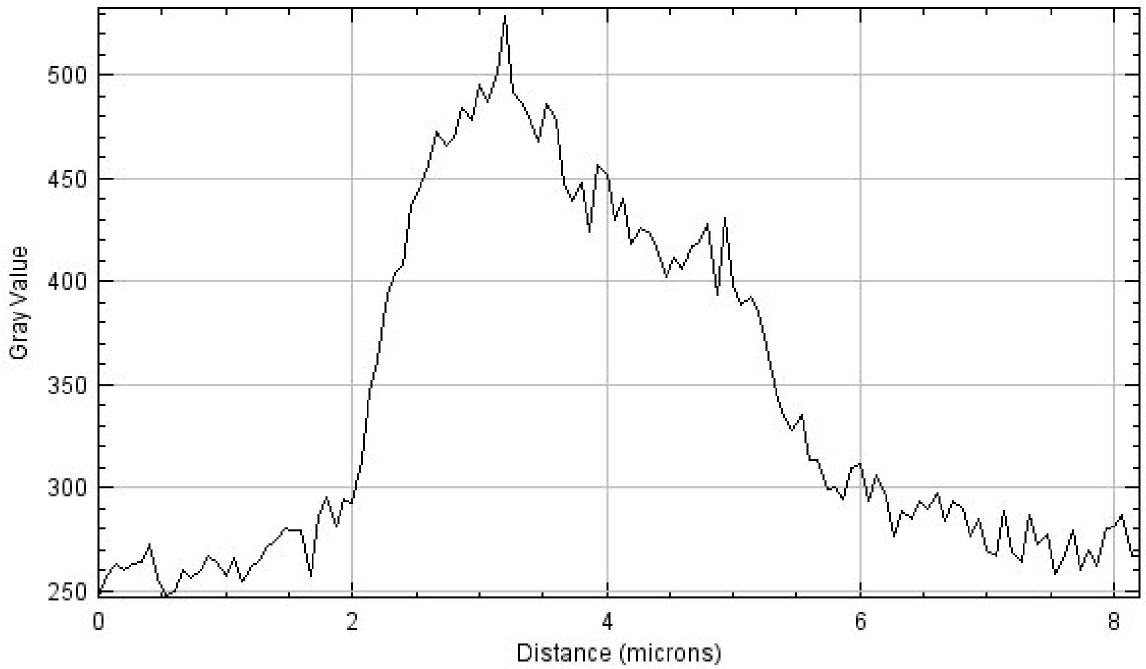

Figure S503 – Intensity profile of line scan generated for cell 3, Figure S500.

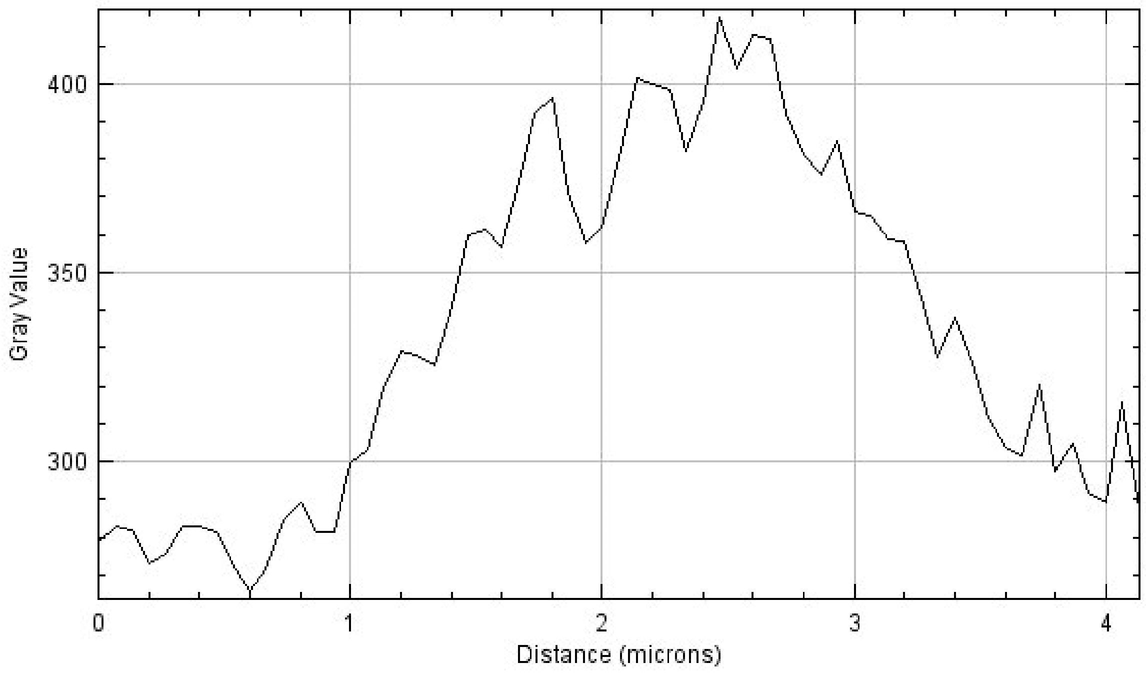

Figure S504 – Intensity profile of line scan generated for cell 4, Figure S500.

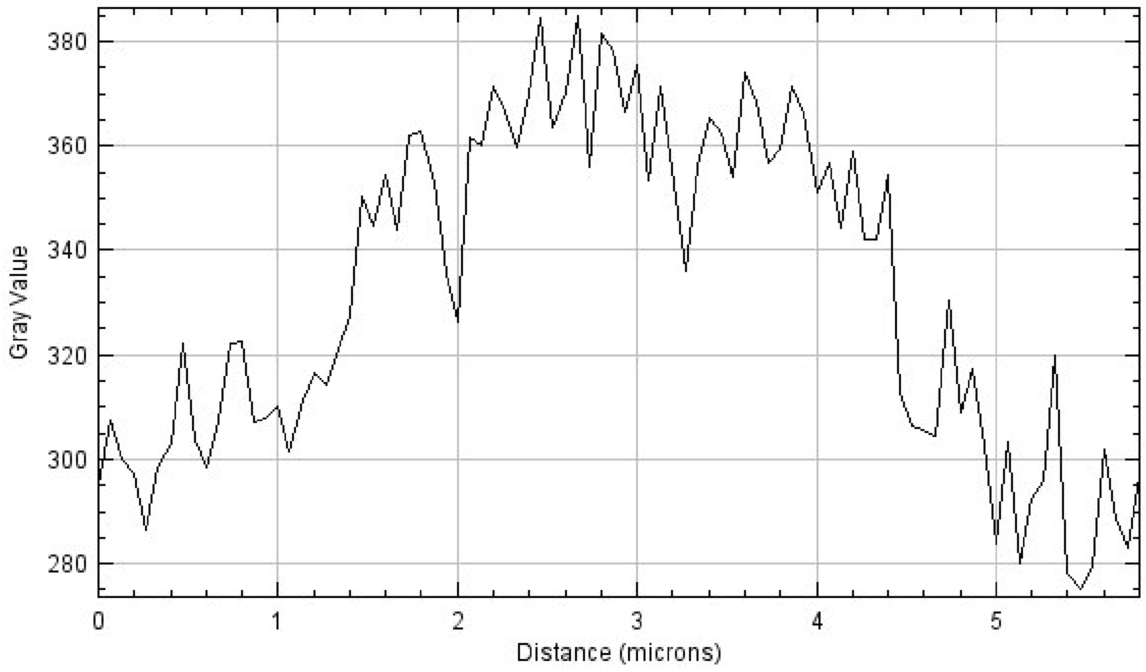

Figure S505 – Intensity profile of line scan generated for cell 5, Figure S500.

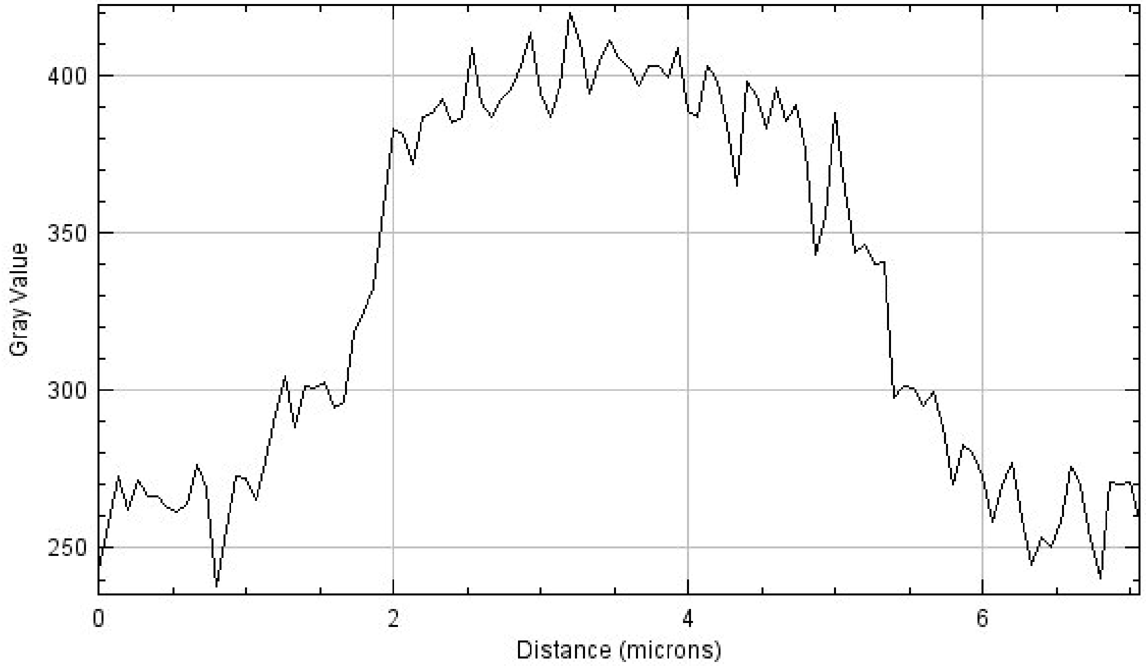

Figure S506 – Intensity profile of line scan generated for cell 6, Figure S500.

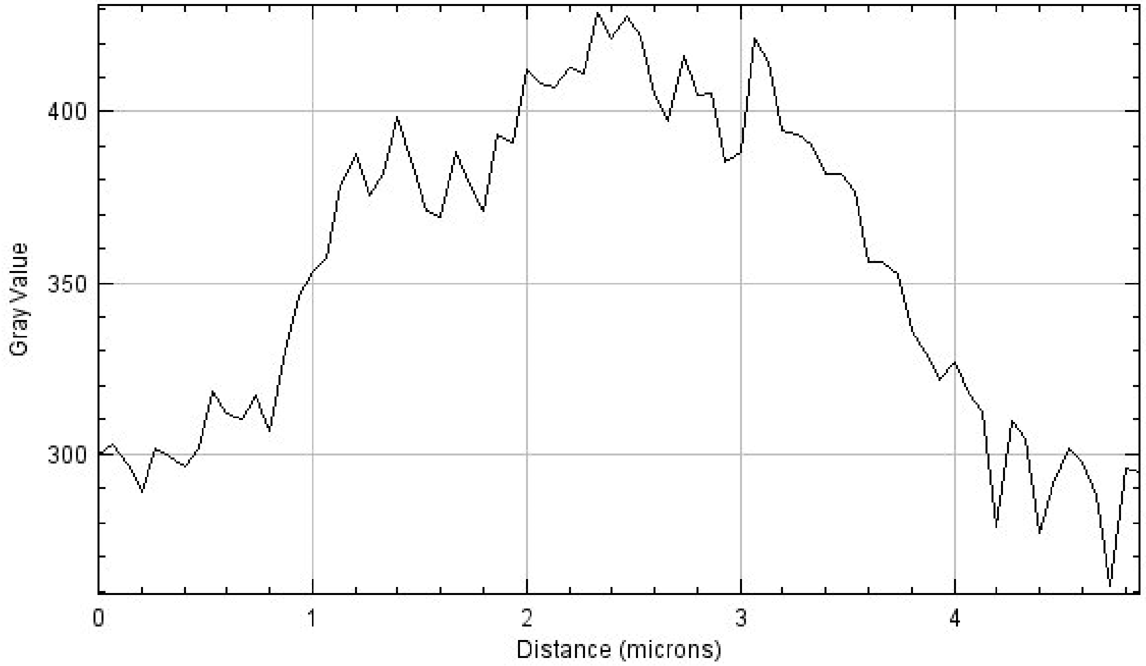

Figure S507 – Intensity profile of line scan generated for cell 7, Figure S500.

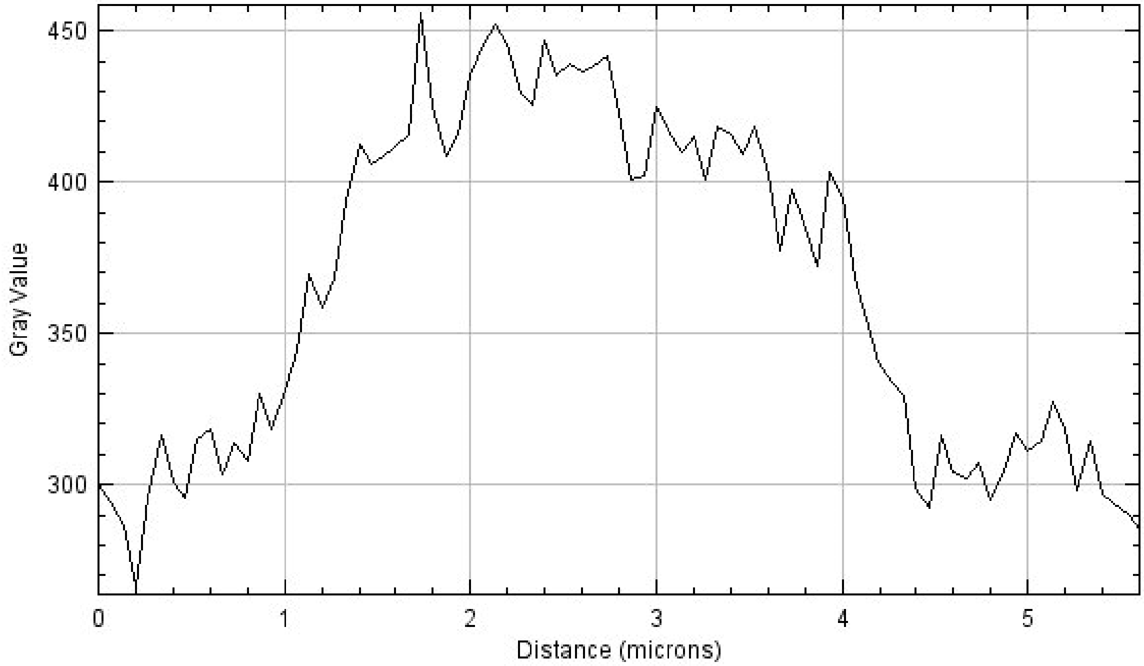

Figure S508 – Intensity profile of line scan generated for cell 8, Figure S500.

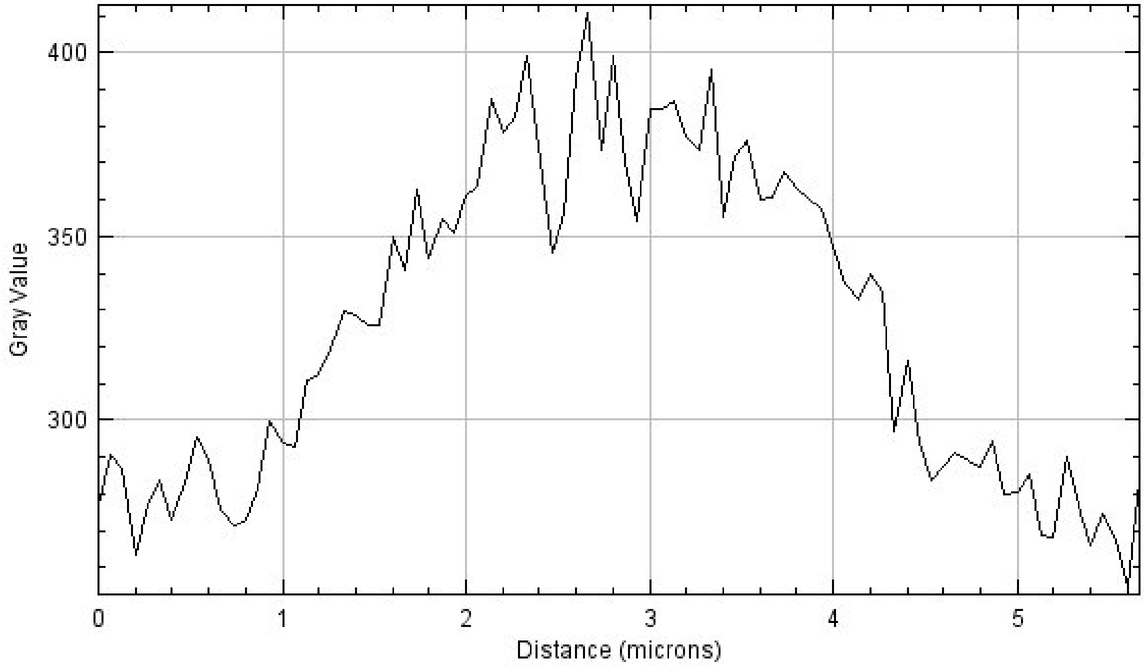

Figure S509 – Intensity profile of line scan generated for cell 9, Figure S500.

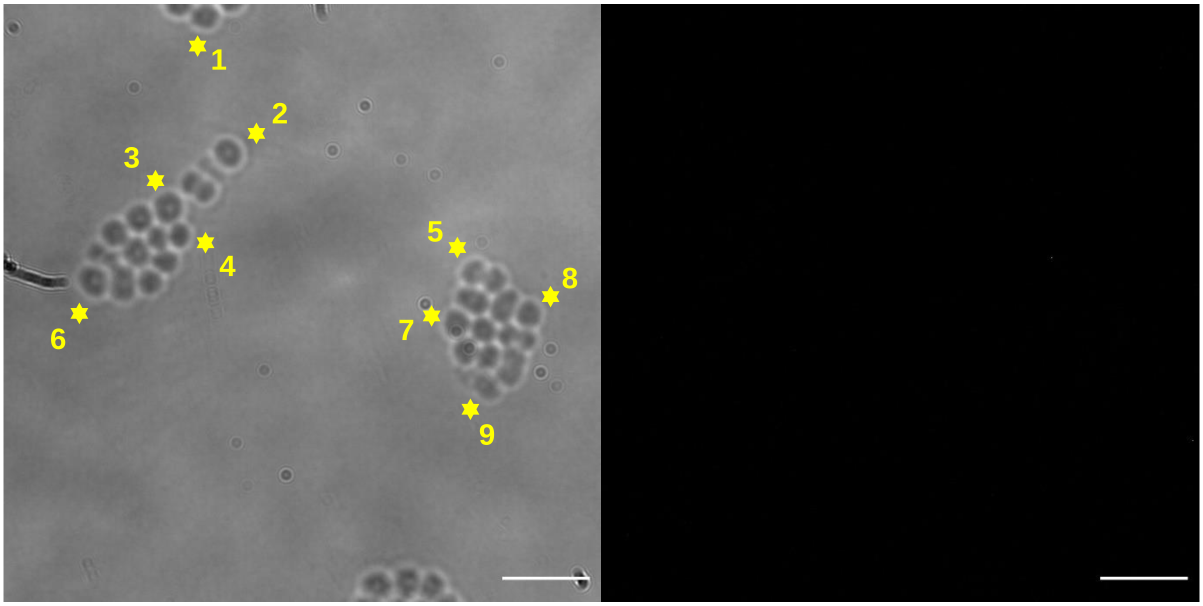

Figure S510 – MRSA transmitted image and fluorescence image at 605 nm used in fluorescence intensity calculations in the presence of both compound **39** and **FM4-64** at T = 30 minutes. Transmitted image used to locate cells on fluorescence image. Scale bar = 10μM

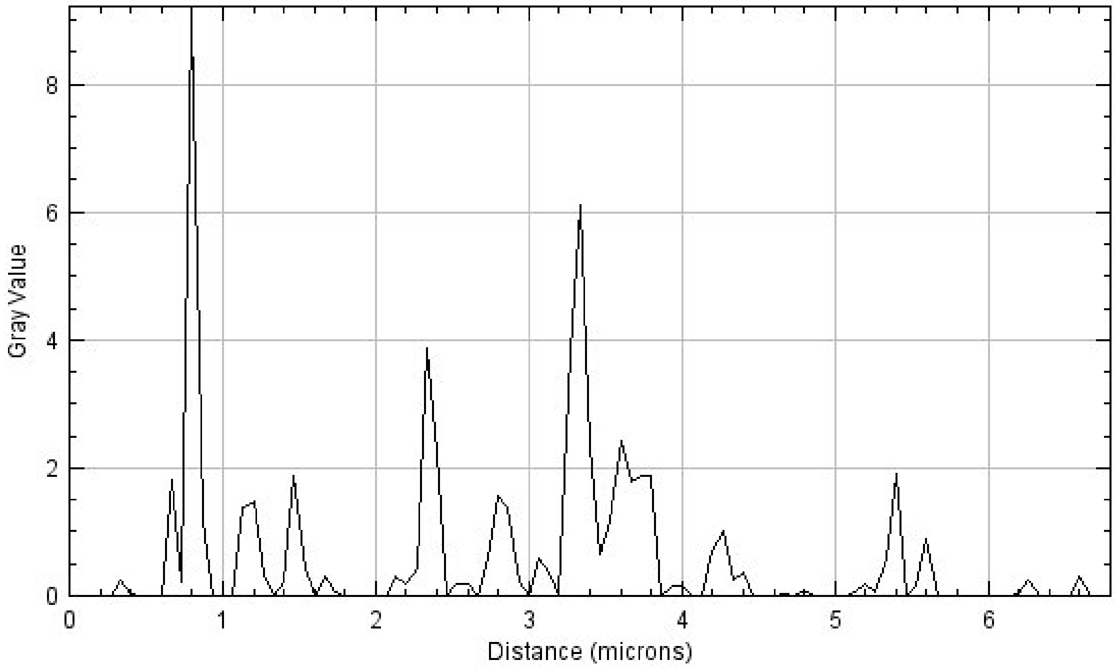

Figure S511 – Intensity profile of line scan generated for cell 1, Figure S510.

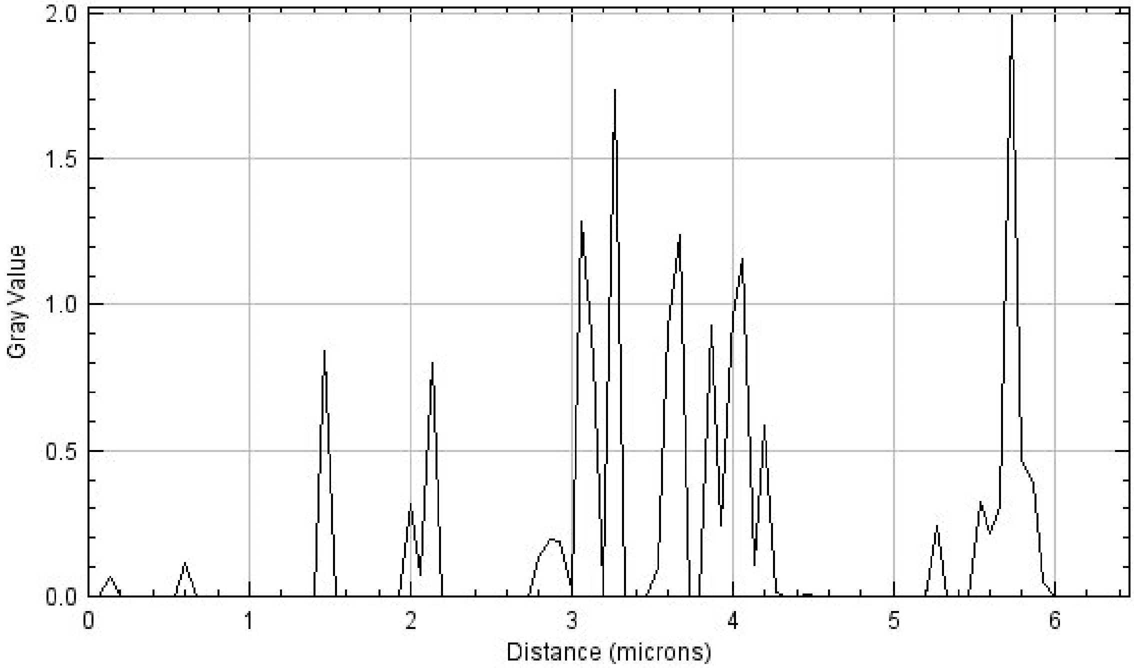

Figure S512 – Intensity profile of line scan generated for cell 2, Figure S510.

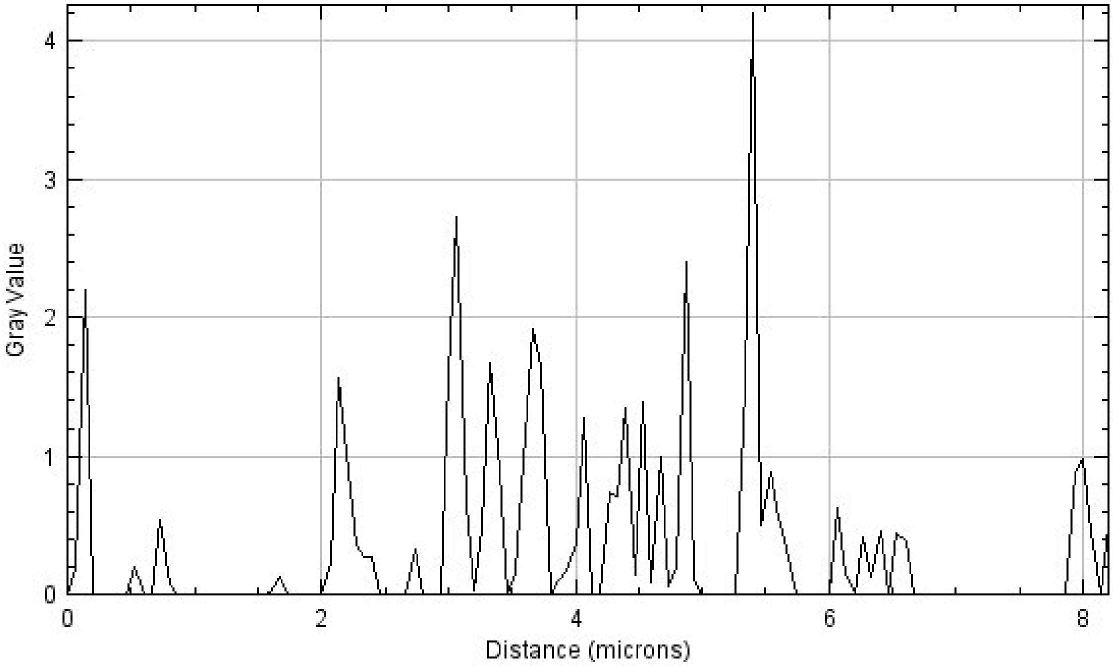

Figure S513 – Intensity profile of line scan generated for cell 3, Figure S510.

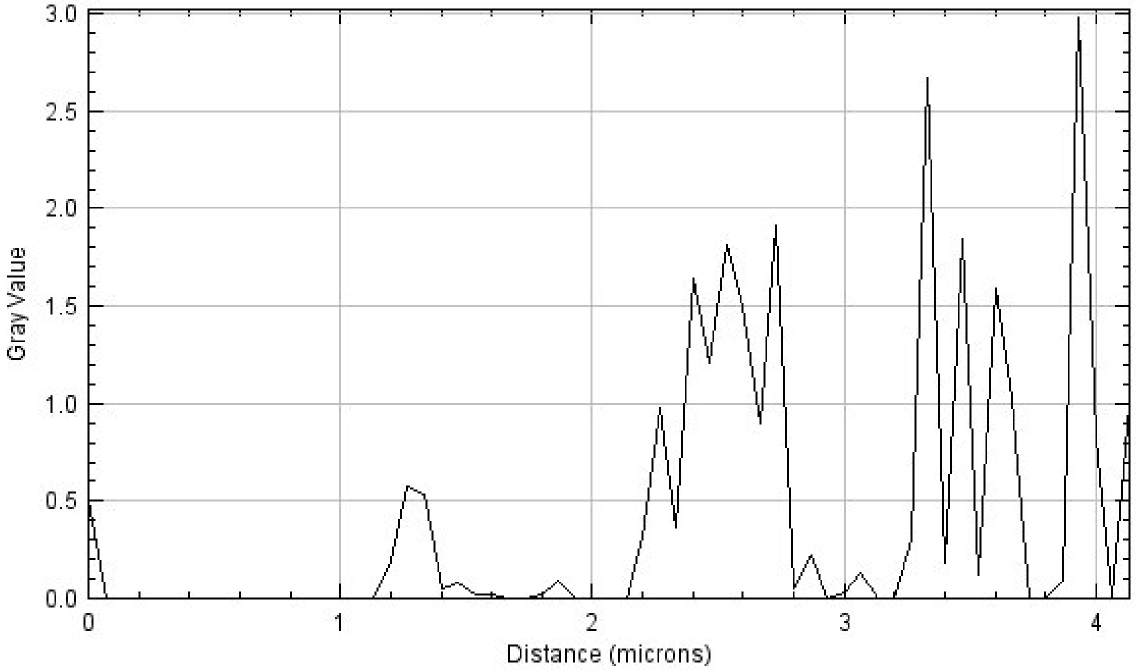

Figure S514 – Intensity profile of line scan generated for cell 4, Figure S510.

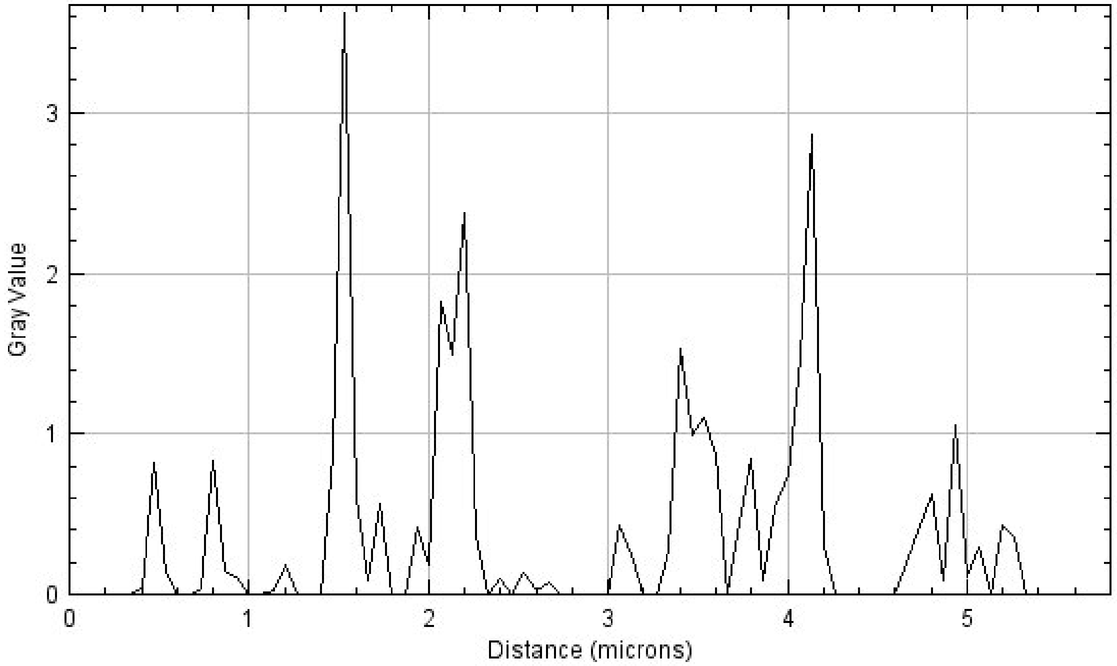

Figure S515 – Intensity profile of line scan generated for cell 5, Figure S510.

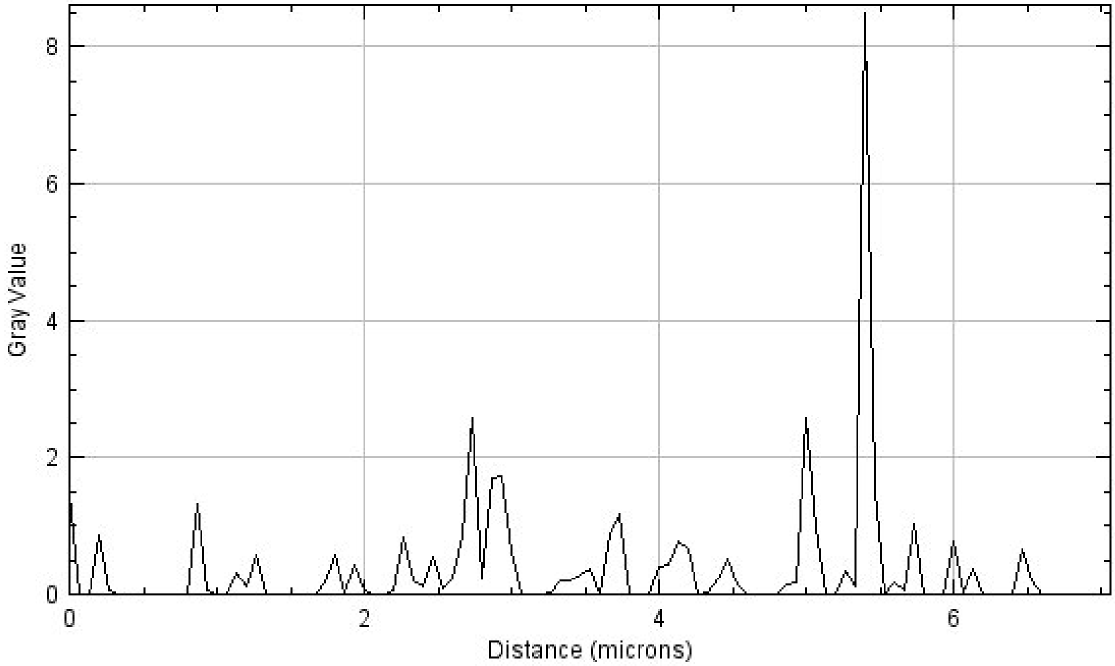

Figure S516 – Intensity profile of line scan generated for cell 6, Figure S510.

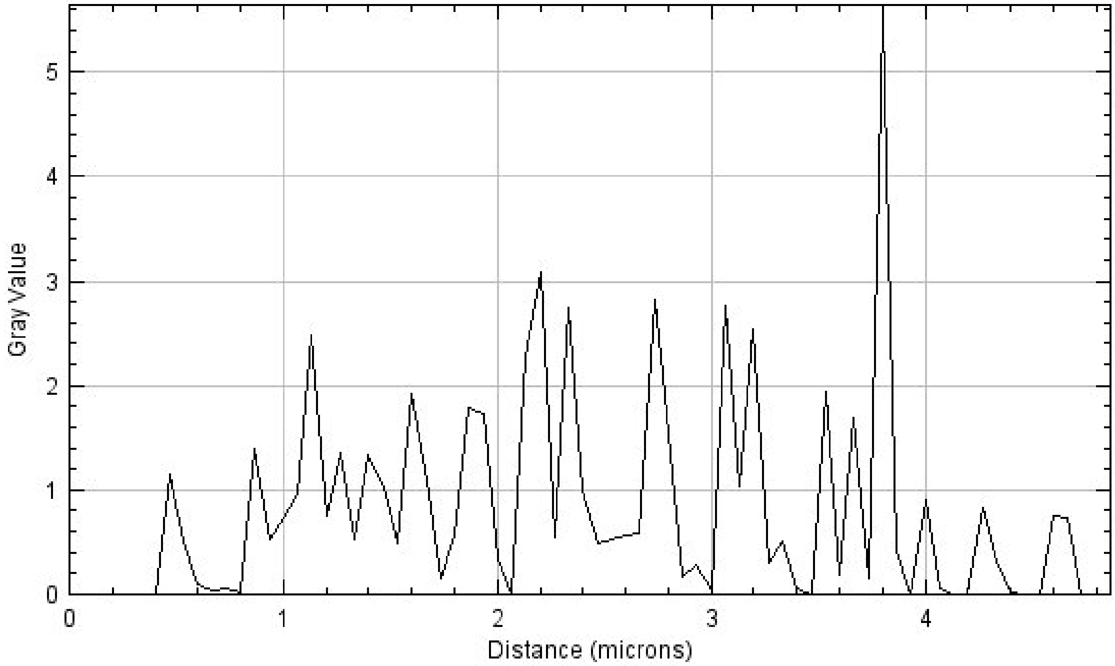

Figure S517 – Intensity profile of line scan generated for cell 7, Figure S510.

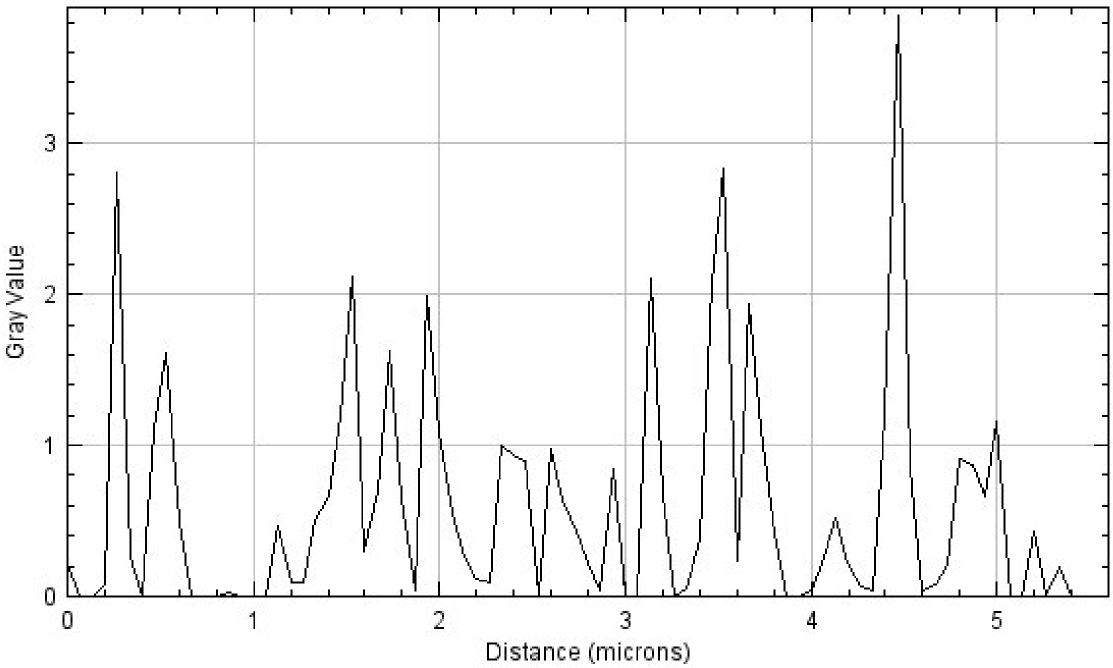

Figure S518 – Intensity profile of line scan generated for cell 8

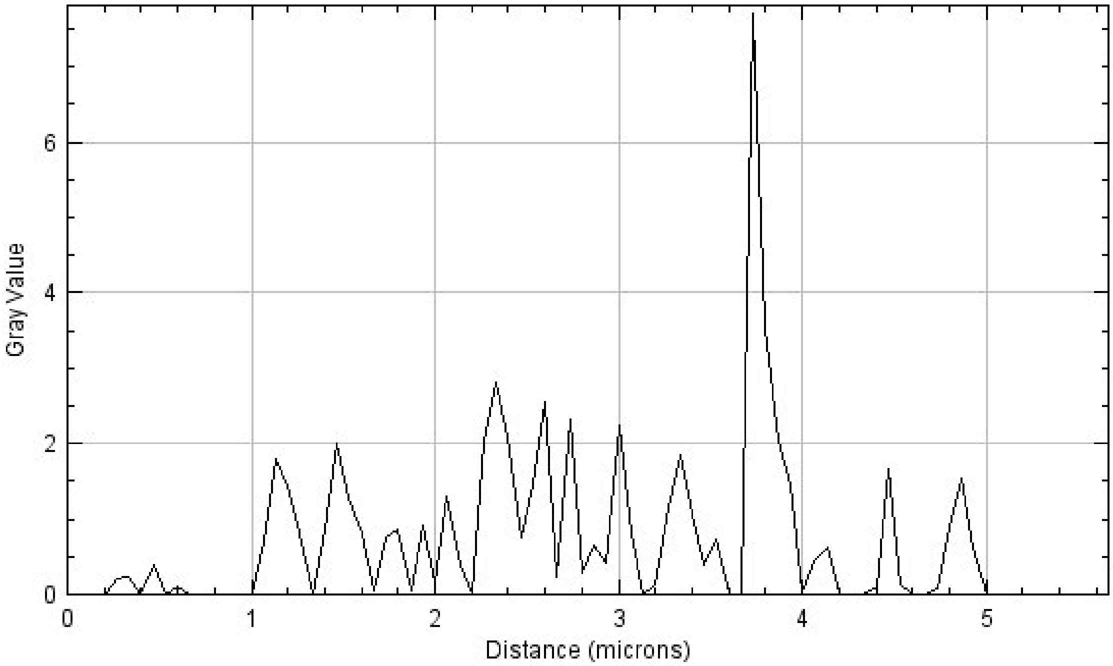

Figure S519 – Intensity profile of line scan generated for cell 9

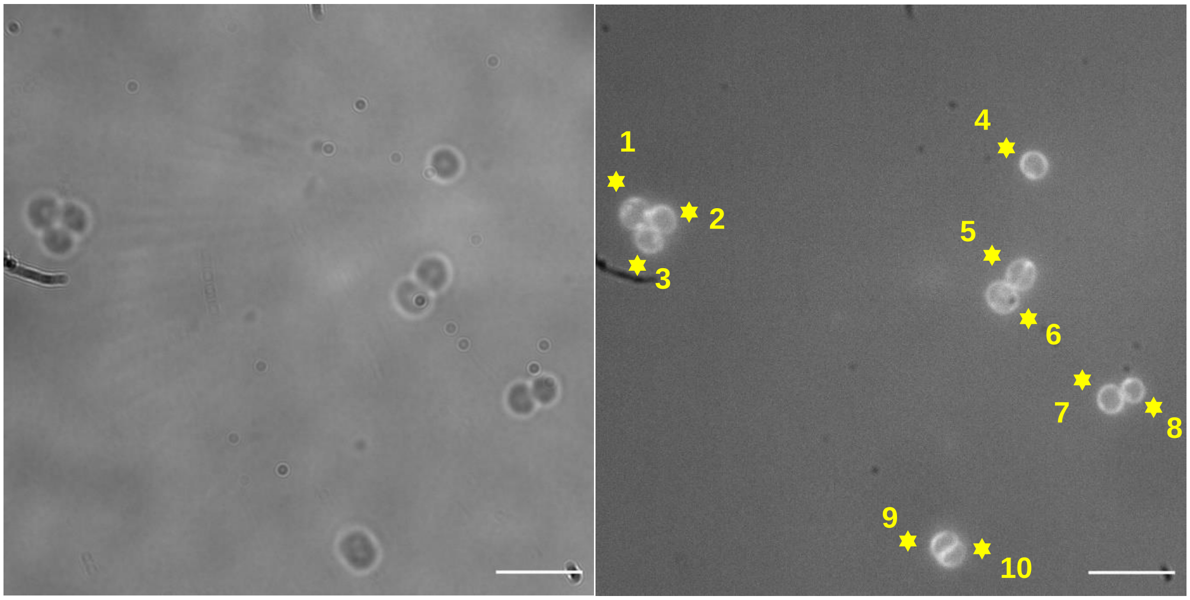

Figure S520 – MRSA transmitted image and fluorescence image at 450 nm used in fluorescence intensity calculations in the presence of both compound **39** and **FM4-64** at T = 30 minutes. Scale bar = 10 µM.

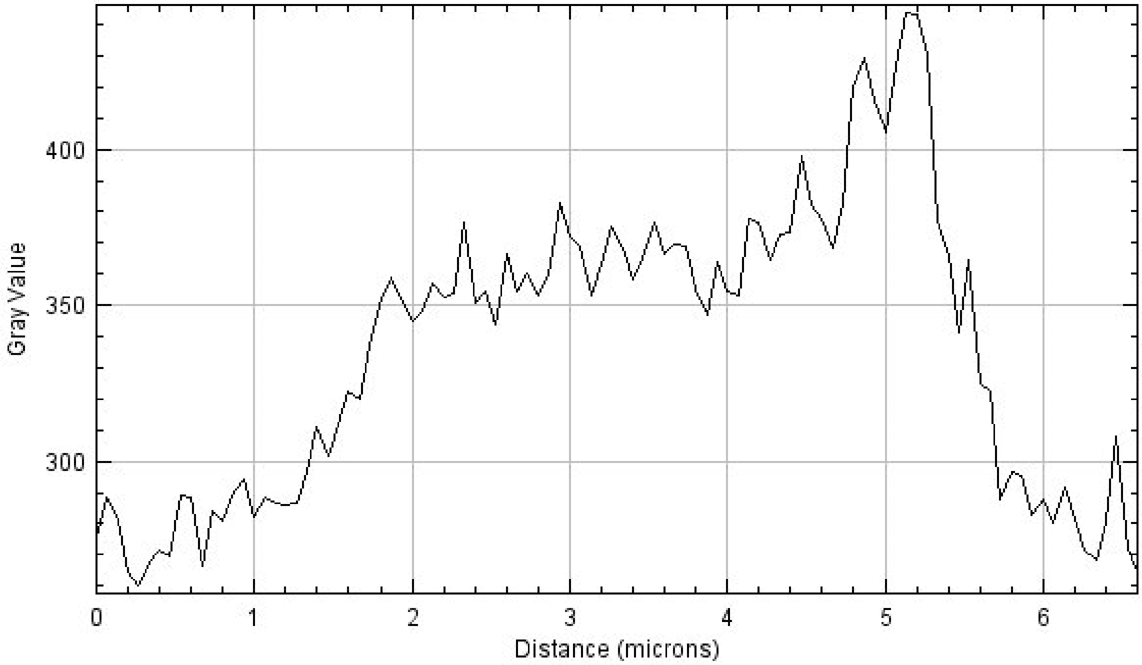

Figure S521 – Intensity profile of line scan generated for cell 1, Figure S520.

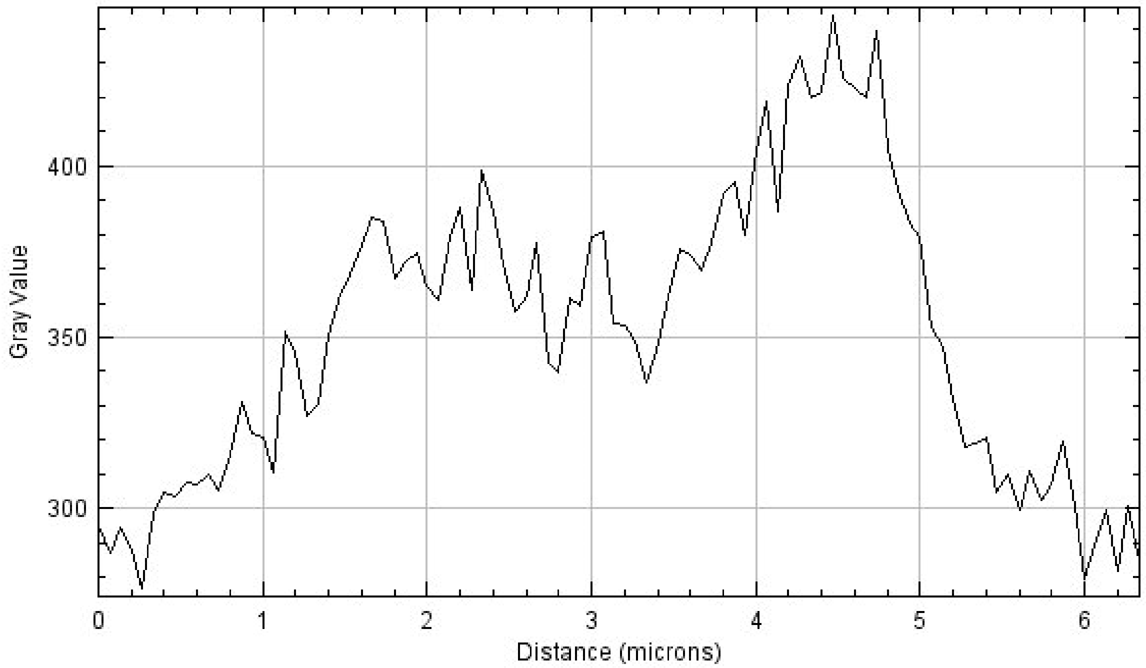

Figure S522 – Intensity profile of line scan generated for cell 2, Figure S520.

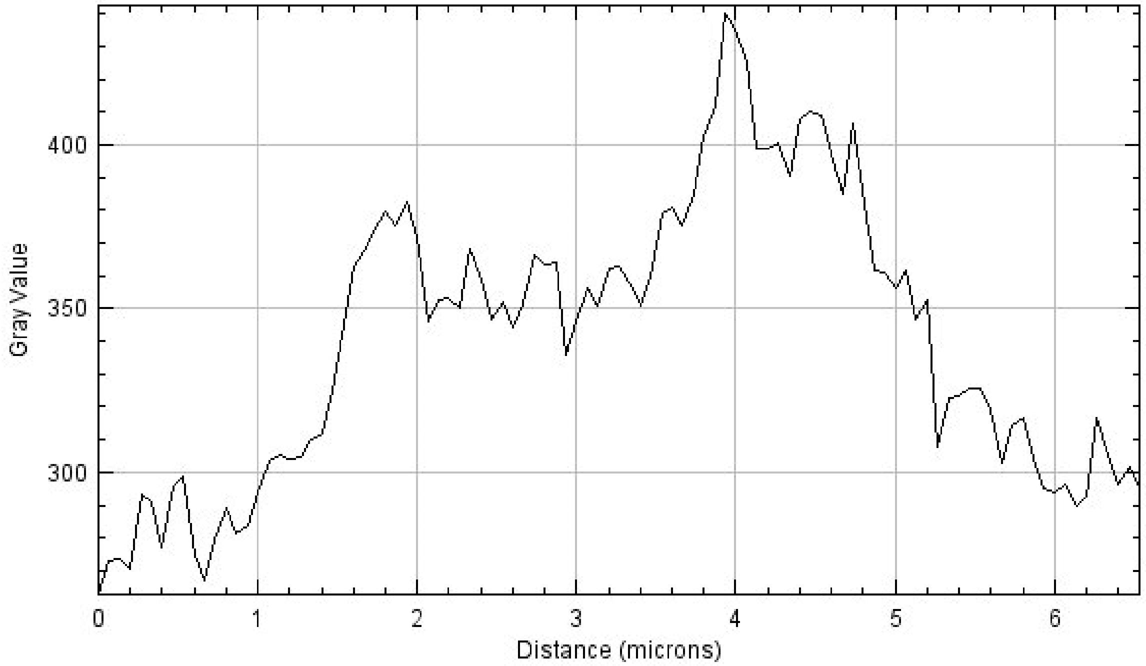

Figure S523 – Intensity profile of line scan generated for cell 3, Figure S520.

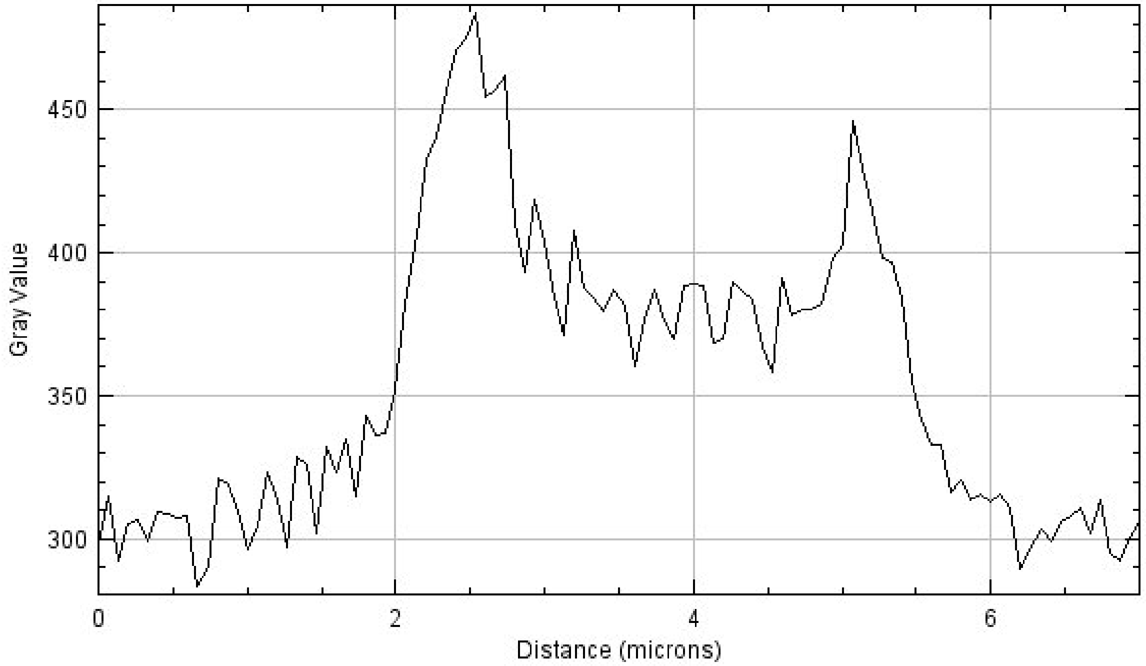

Figure S524 – Intensity profile of line scan generated for cell 4, Figure S520.

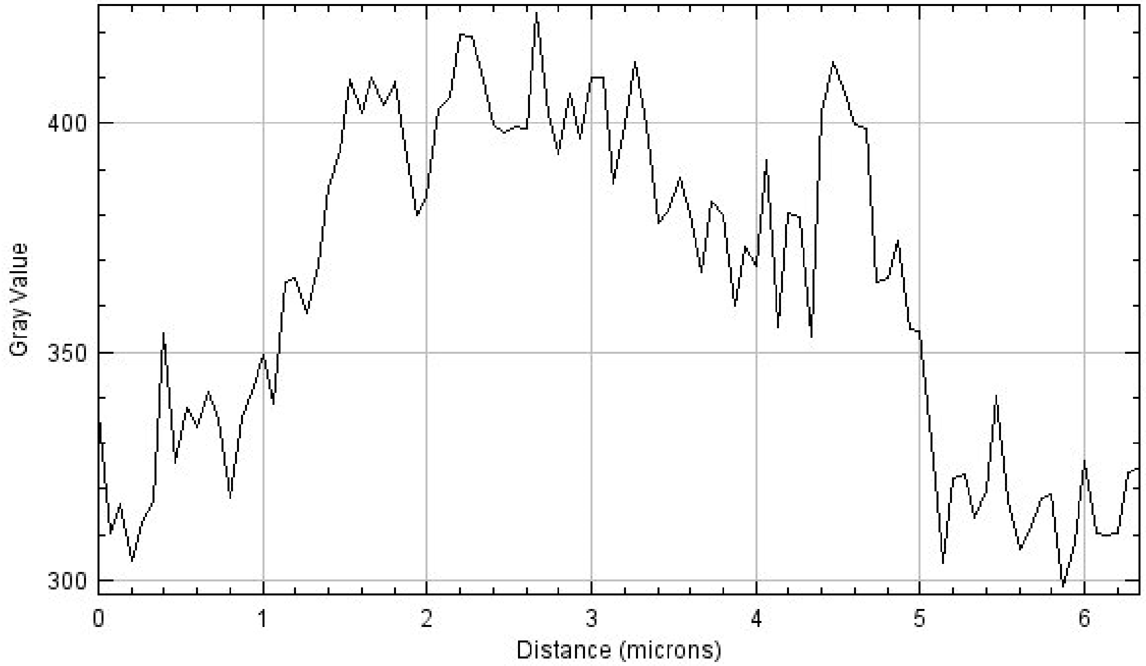

Figure S525 – Intensity profile of line scan generated for cell 5, Figure S520.

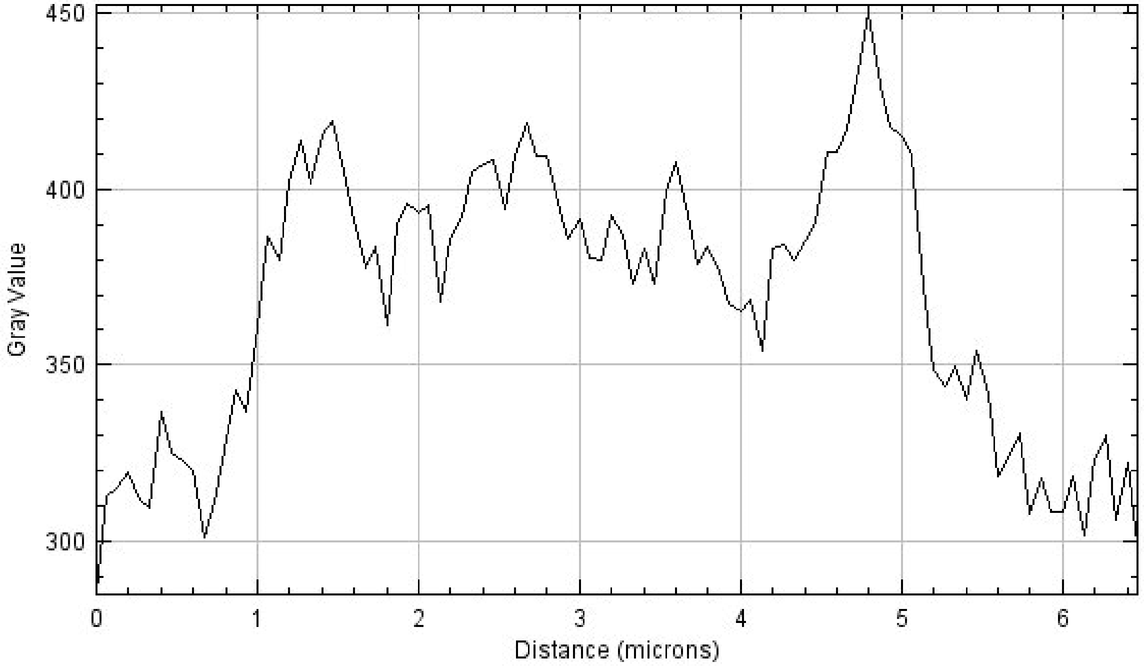

Figure S526 – Intensity profile of line scan generated for cell 6, Figure S520.

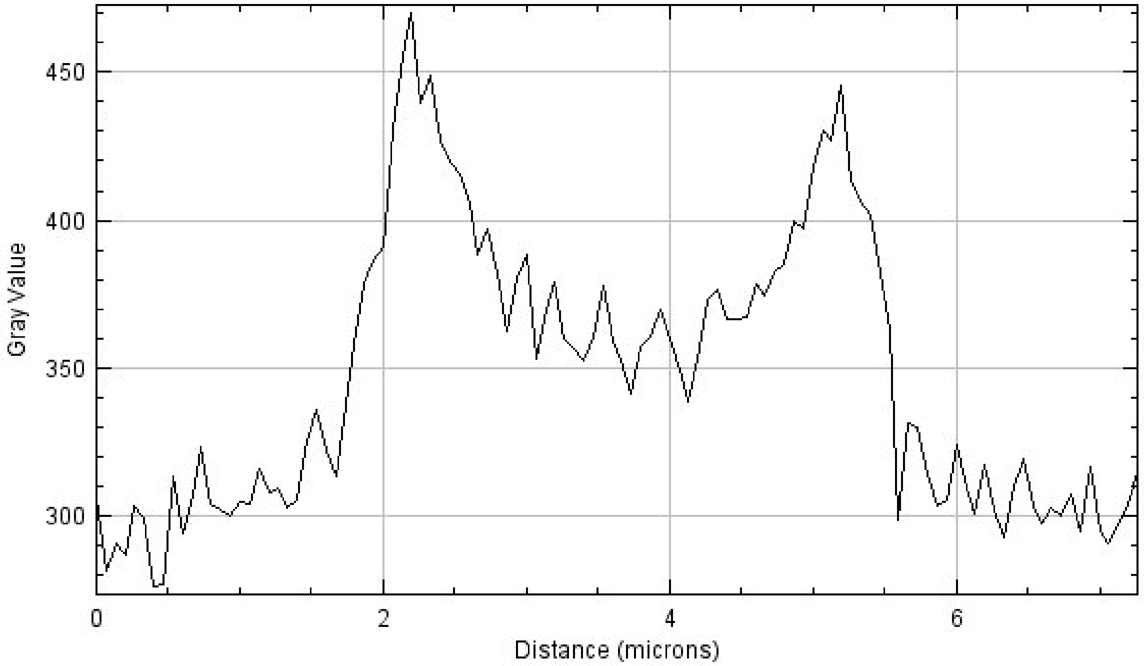

Figure S527 – Intensity profile of line scan generated for cell 7, Figure S520.

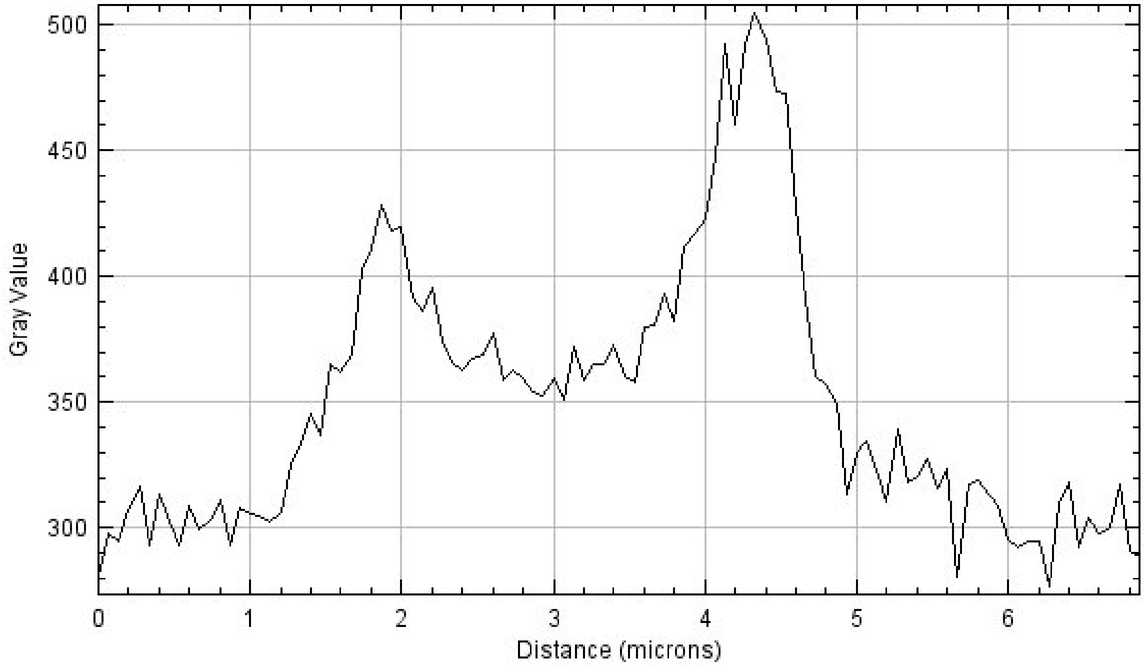

Figure S528 – Intensity profile of line scan generated for cell 8, Figure S520.

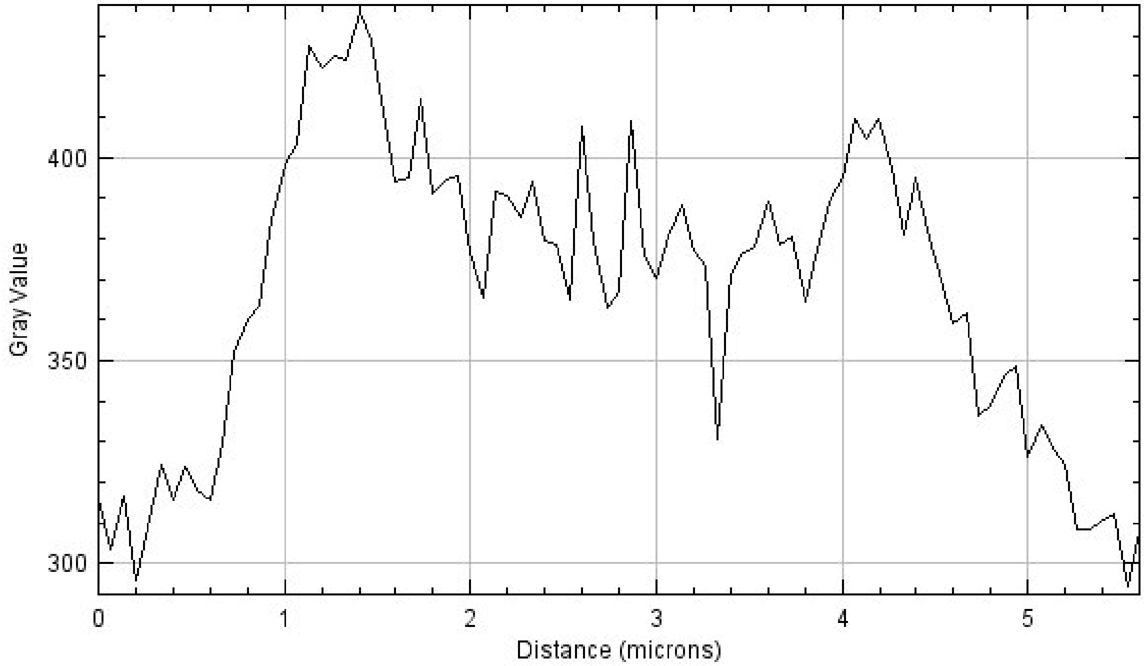

Figure S529 – Intensity profile of line scan generated for cell 9, Figure S520.

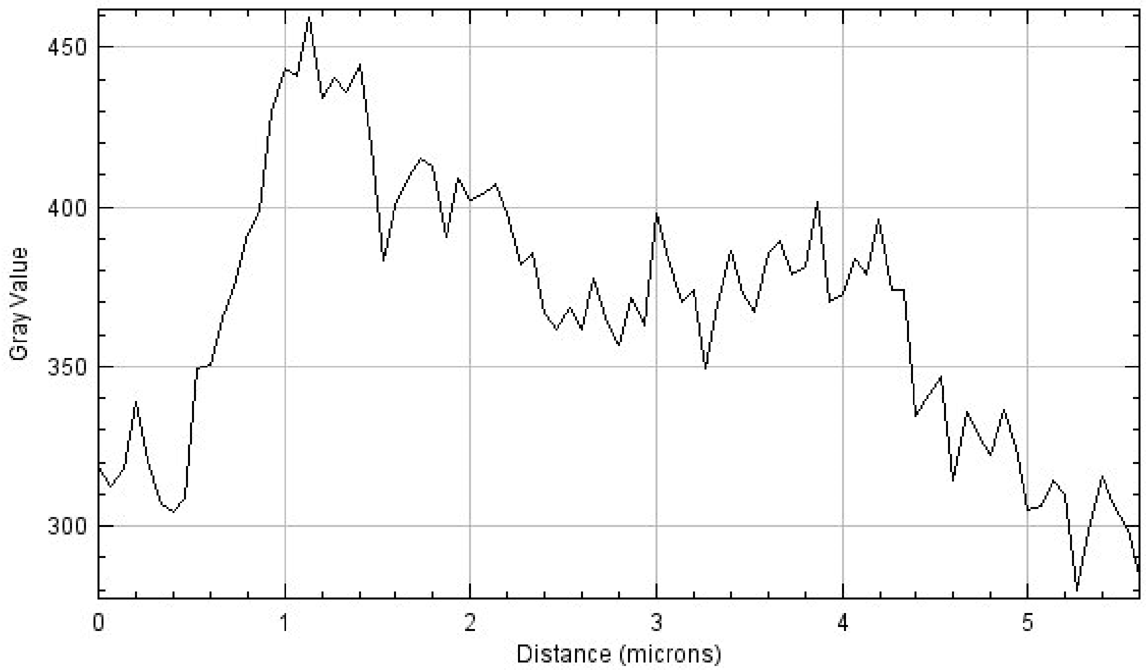

Figure S530 – Intensity profile of line scan generated for cell 10, Figure S520.

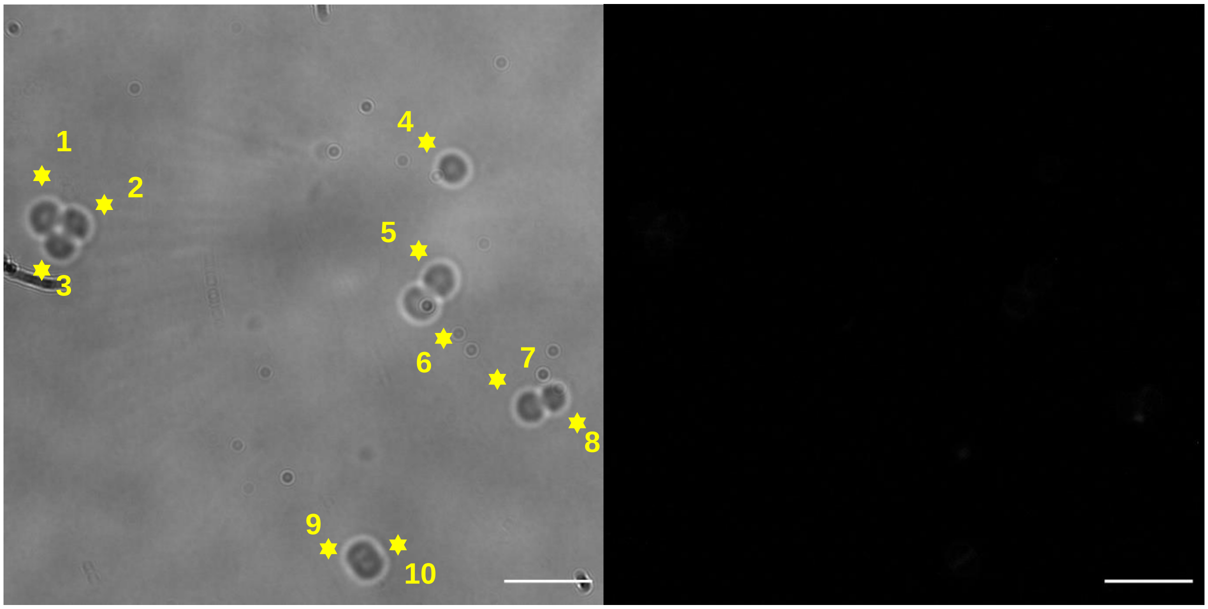

Figure S531 – MRSA transmitted image and fluorescence image at 605 nm used in fluorescence intensity calculations in the presence of both compound **39** and **FM4-64** at T = 30 minutes. Transmitted image used to locate cells on fluorescence image. Scale bar = 10 μM.

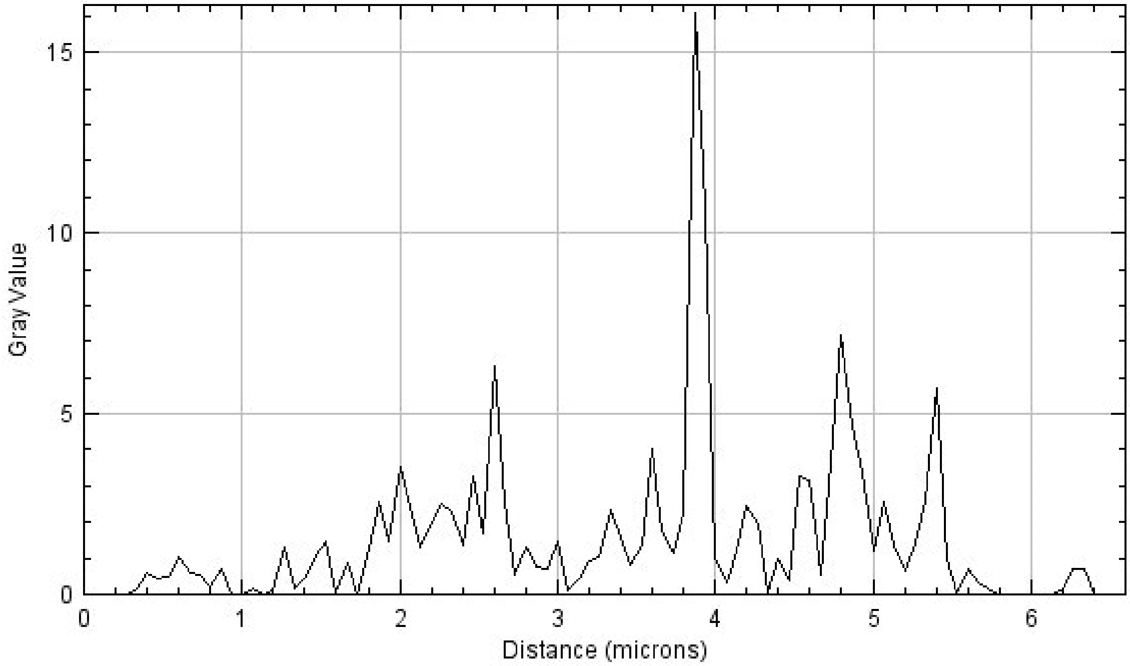

Figure S532 – Intensity profile of line scan generated for cell 1, Figure S531.

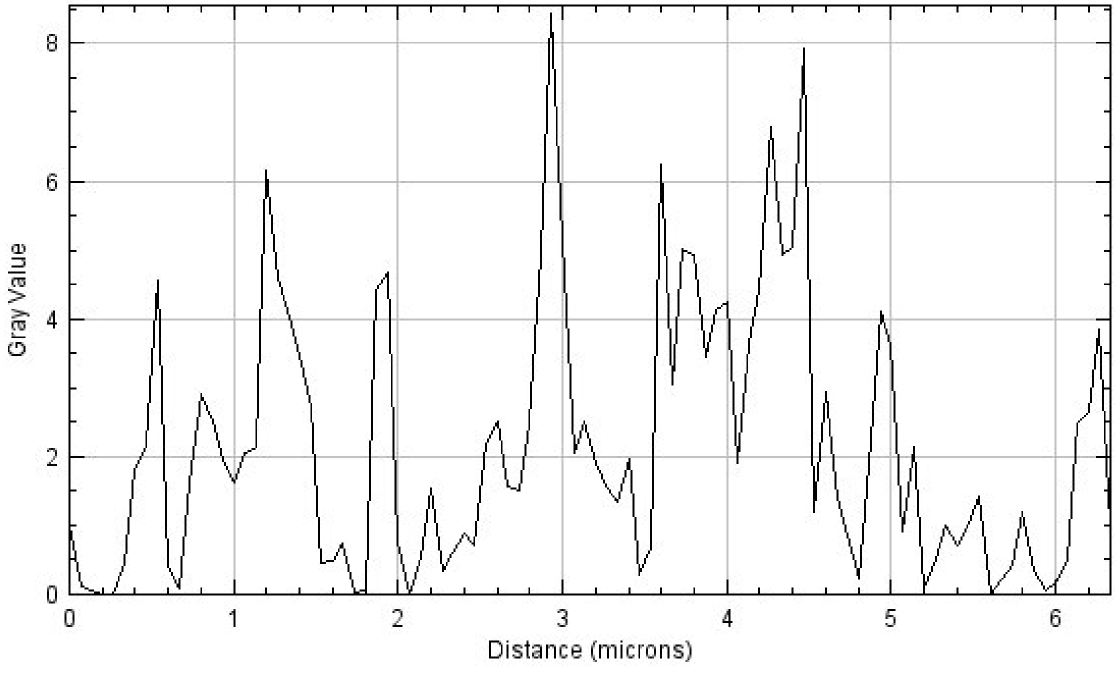

Figure S533 – Intensity profile of line scan generated for cell 2, Figure S531.

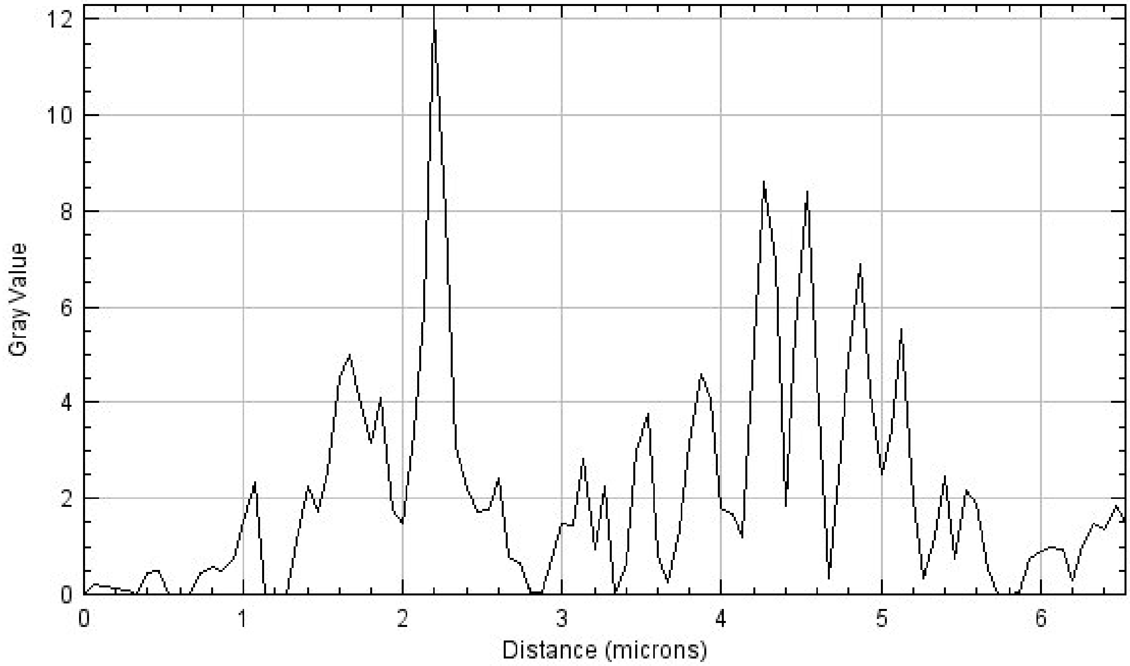

Figure S534 – Intensity profile of line scan generated for cell 3, Figure S531.

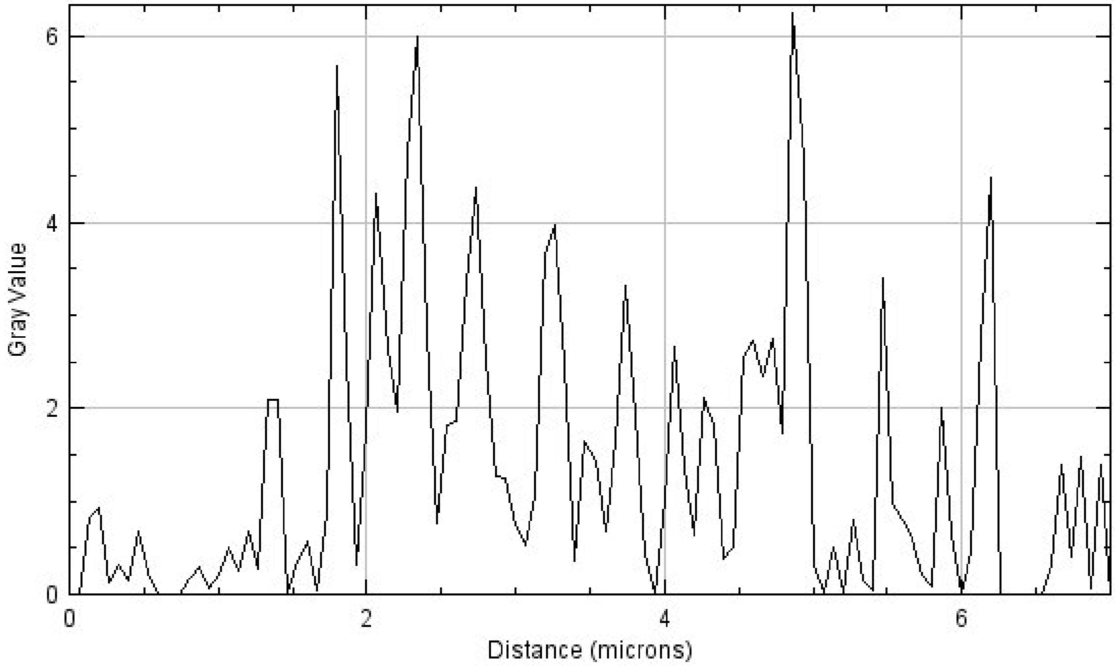

Figure S535 – Intensity profile of line scan generated for cell 4, Figure S531.

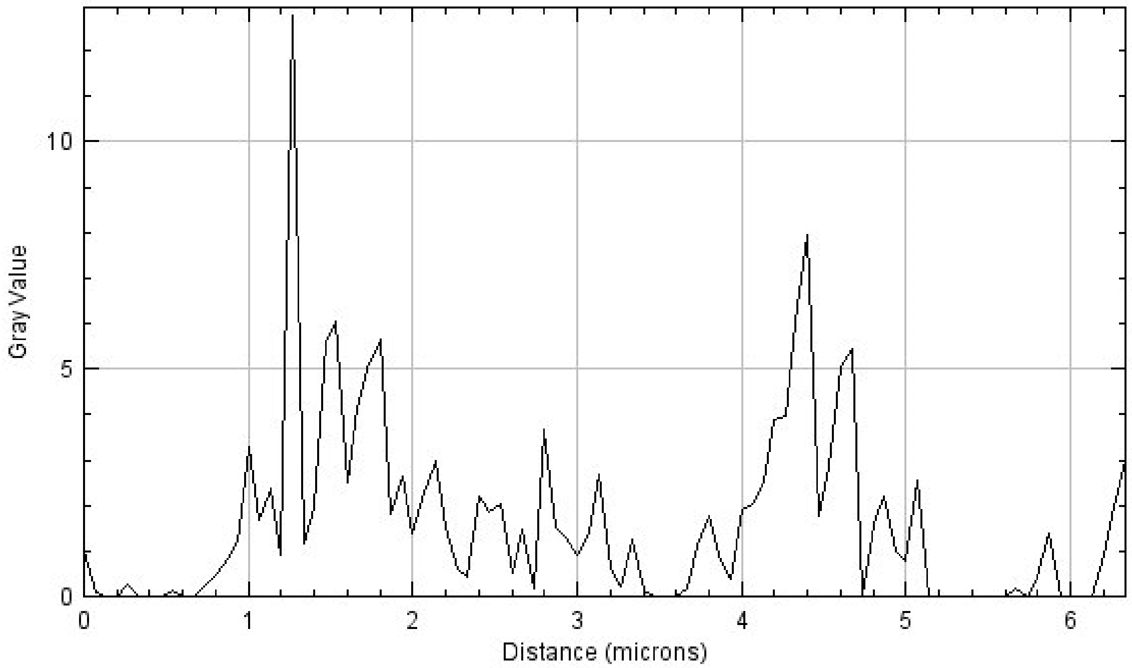

Figure S536 – Intensity profile of line scan generated for cell 5, Figure S531.

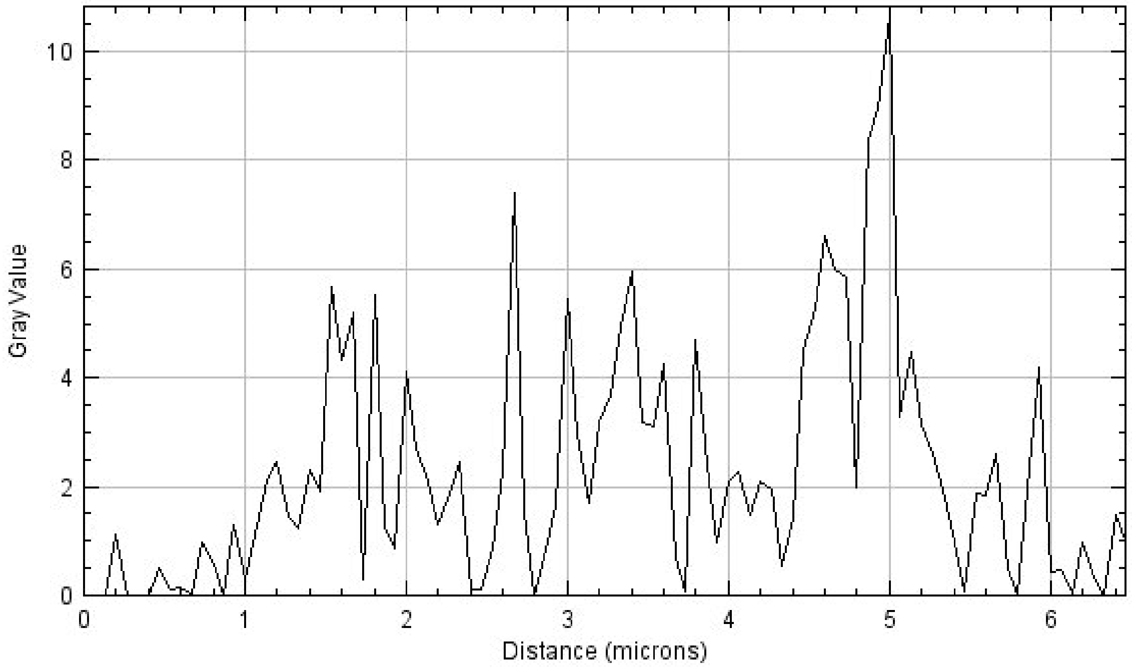

Figure S537 – Intensity profile of line scan generated for cell 6, Figure S531.

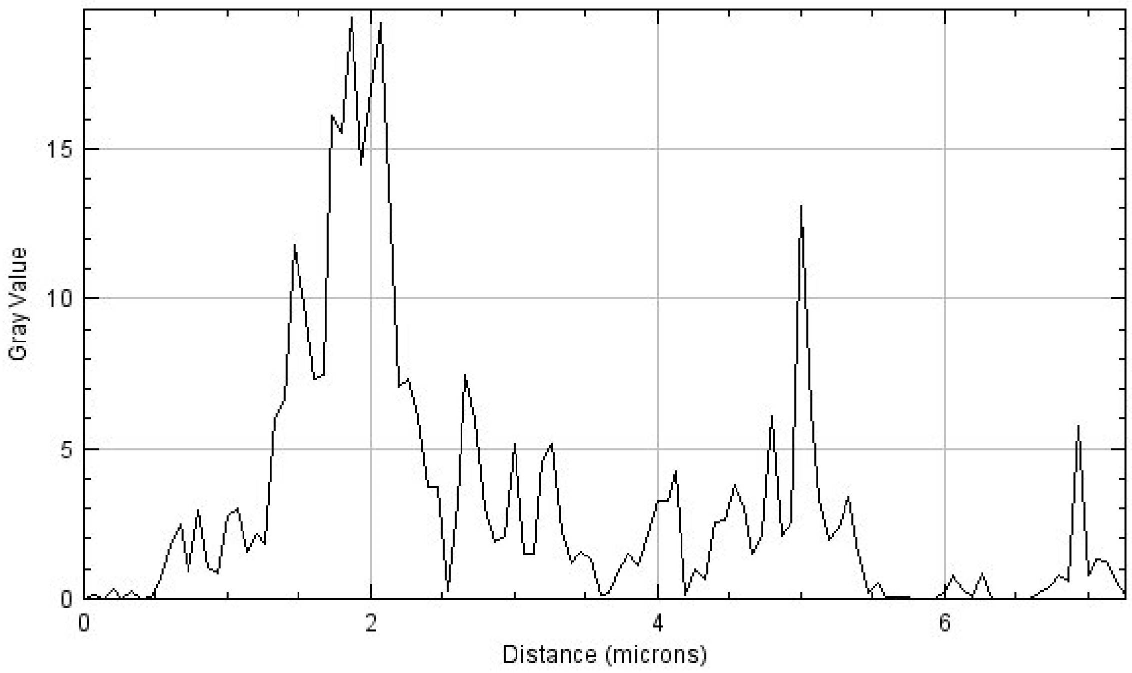

Figure S538 – Intensity profile of line scan generated for cell 7, Figure S531.

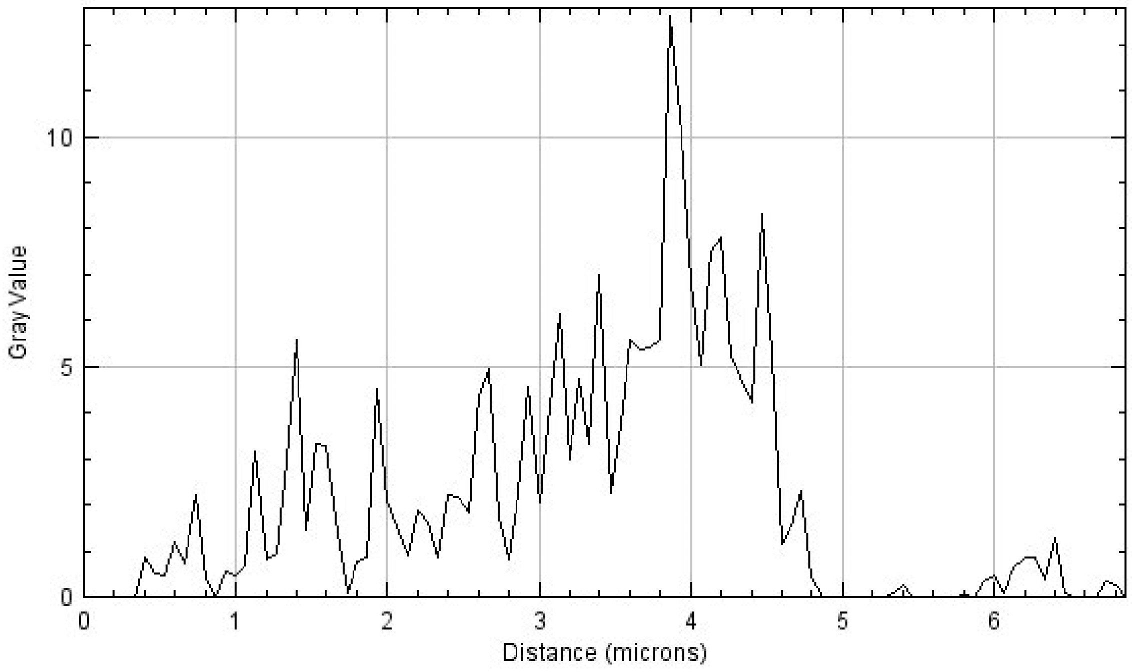

Figure S539 – Intensity profile of line scan generated for cell 8, Figure S531.

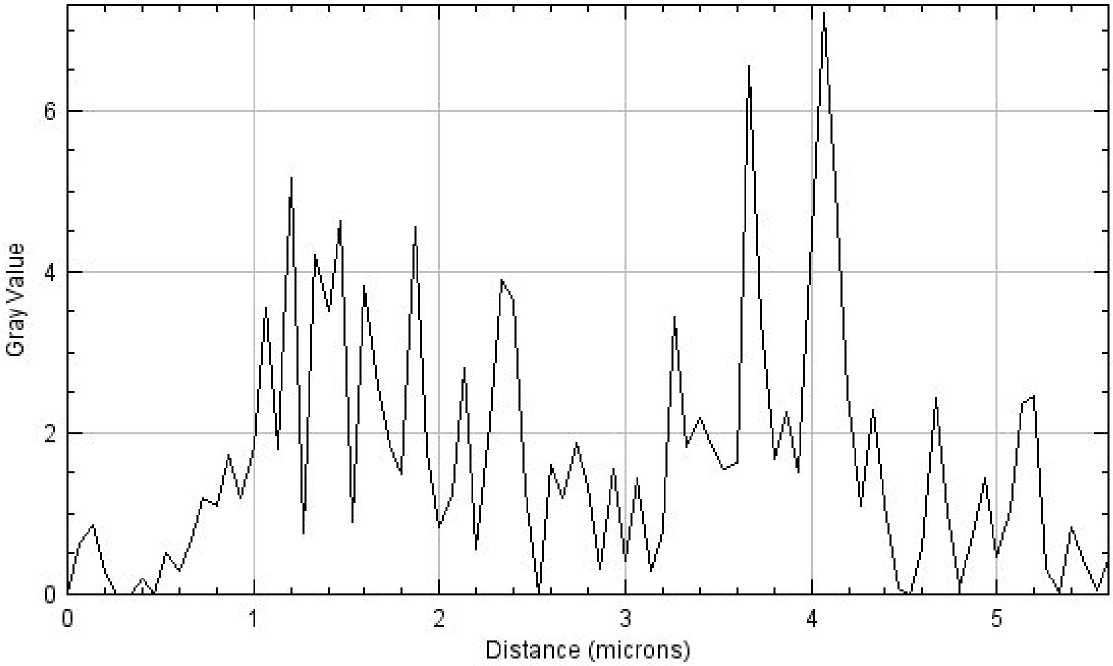

Figure S540 – Intensity profile of line scan generated for cell 9, Figure S531.

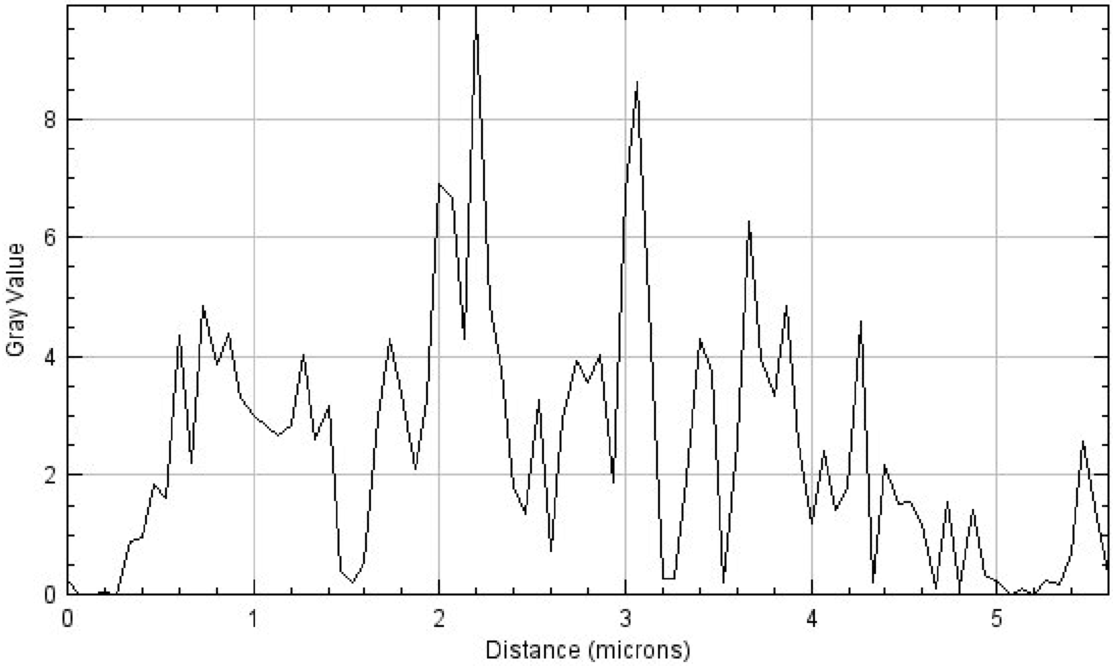

Figure S541 – Intensity profile of line scan generated for cell 10, Figure S531.

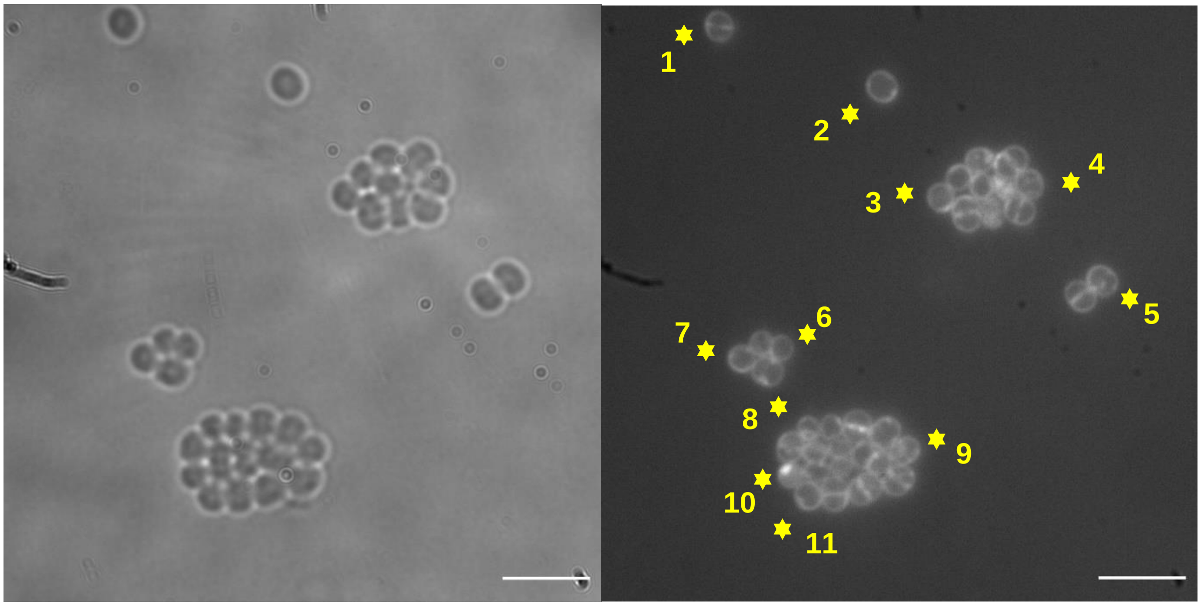

Figure S542 – MRSA transmitted image and fluorescence image at 450 nm used in fluorescence intensity calculations in the presence of both compound **39** and **FM4-64** at T = 30 minutes. Scale bar = 10 µM.

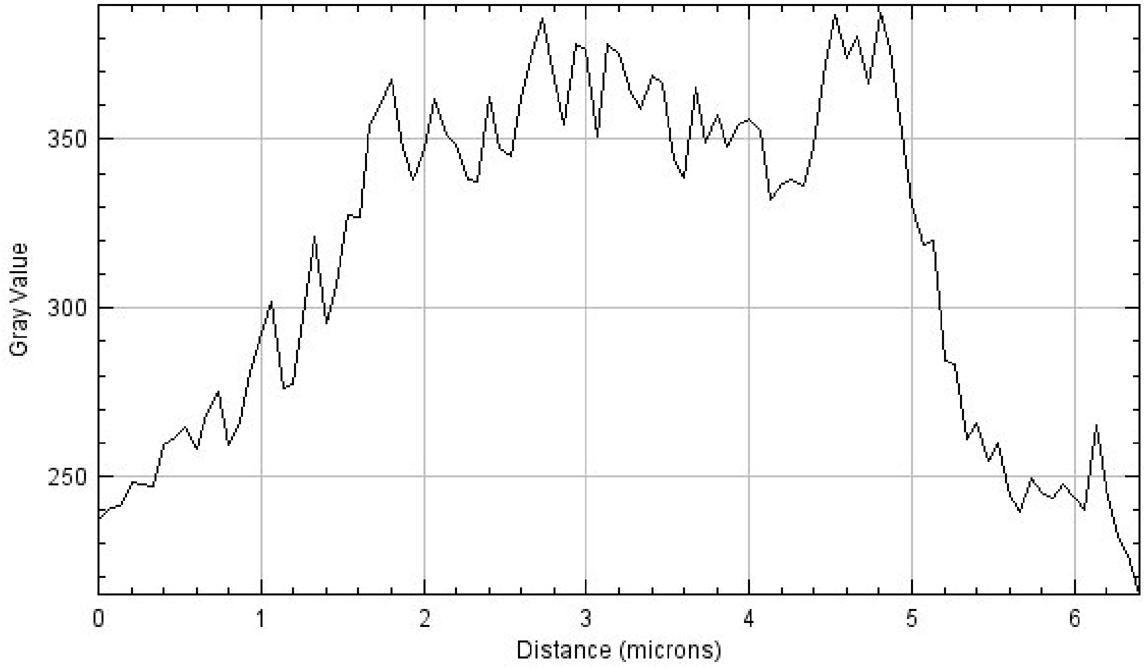

Figure S543 – Intensity profile of line scan generated for cell 1, Figure S542.

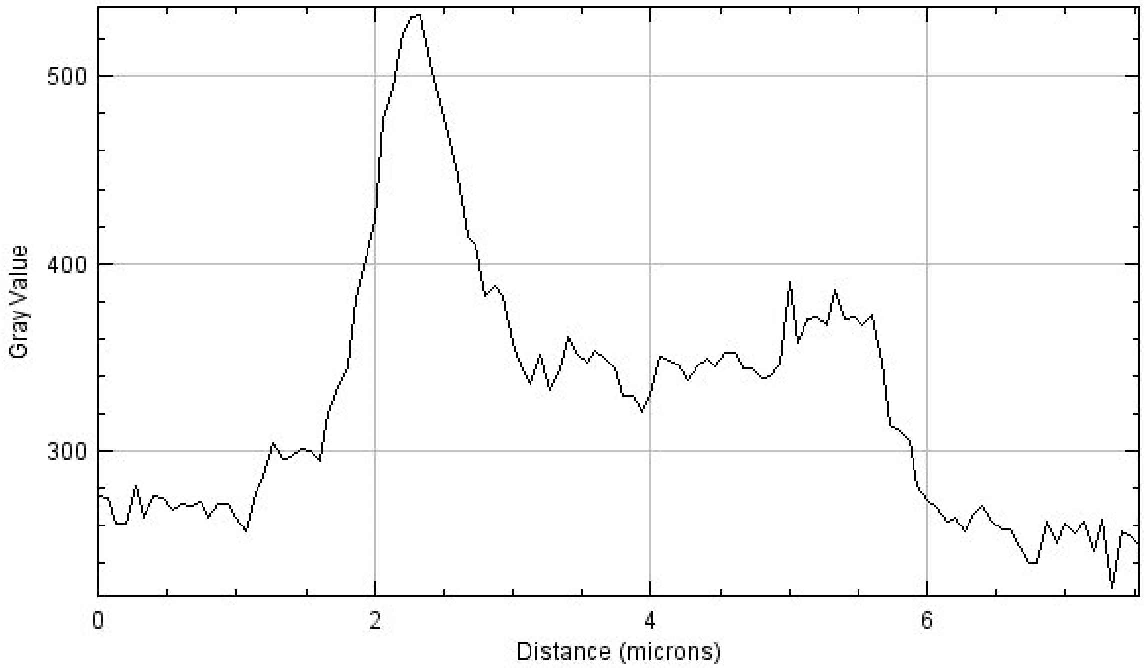

Figure S544 – Intensity profile of line scan generated for cell 2, Figure S542.

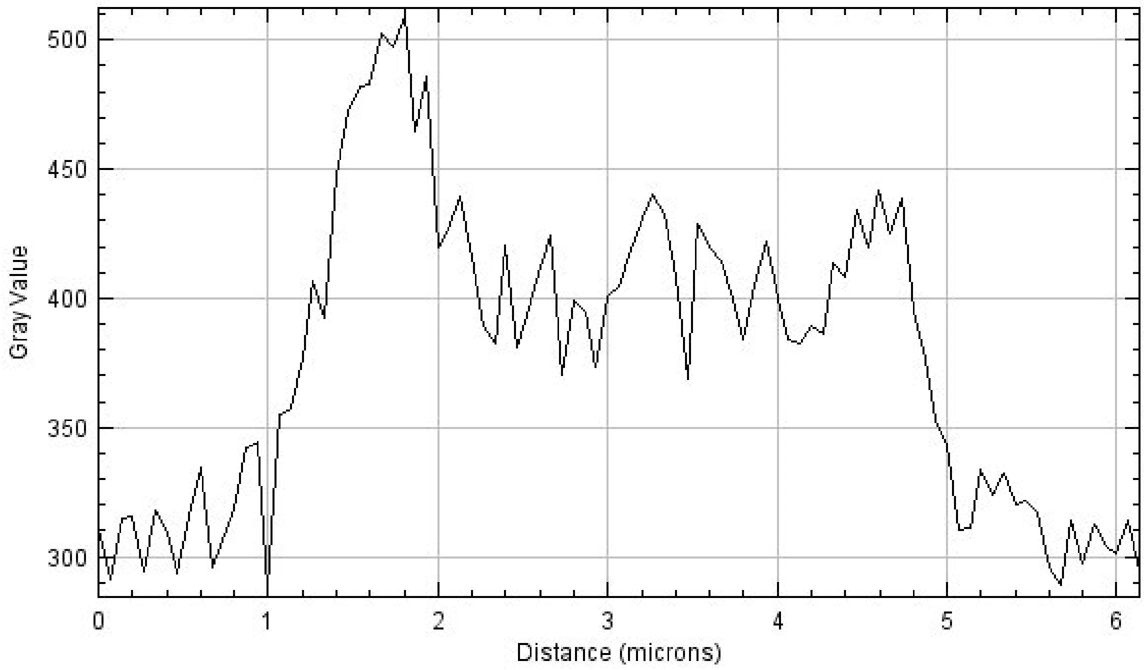

Figure S545 – Intensity profile of line scan generated for cell 3, Figure S542.

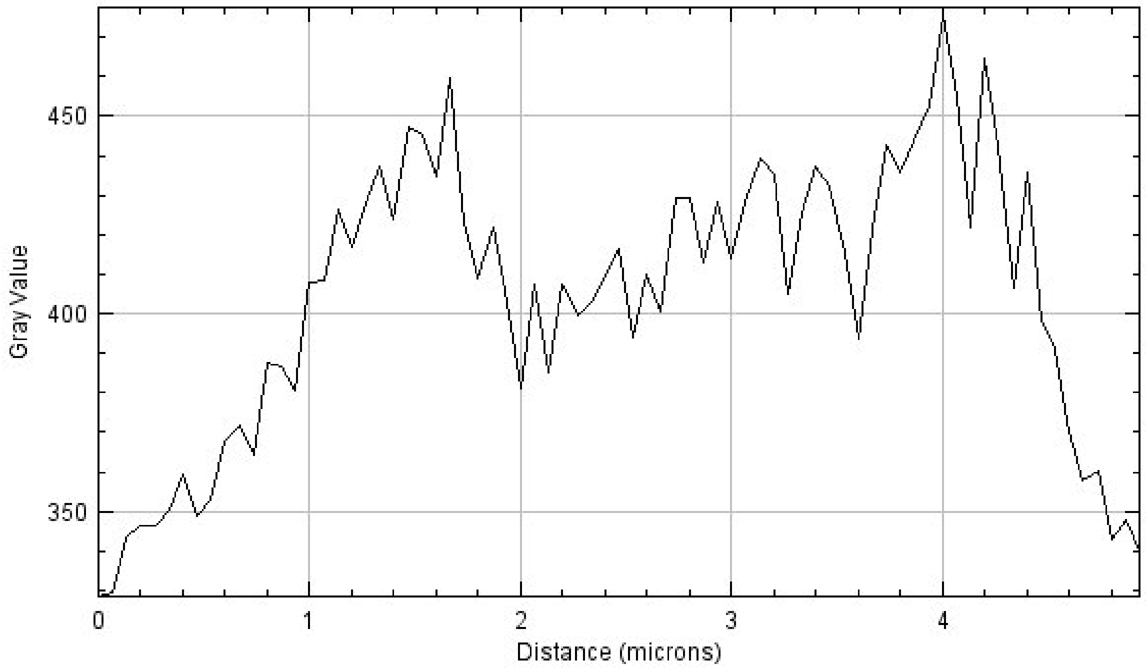

Figure S546 – Intensity profile of line scan generated for cell 4, Figure S542.

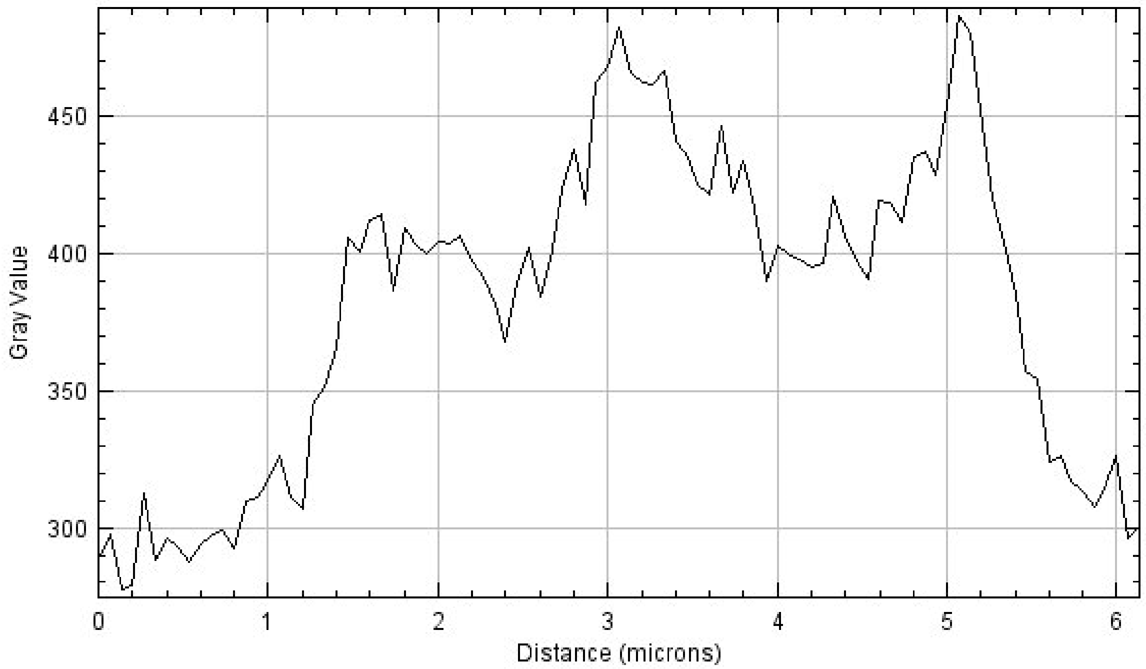

Figure S547 – Intensity profile of line scan generated for cell 5, Figure S542.

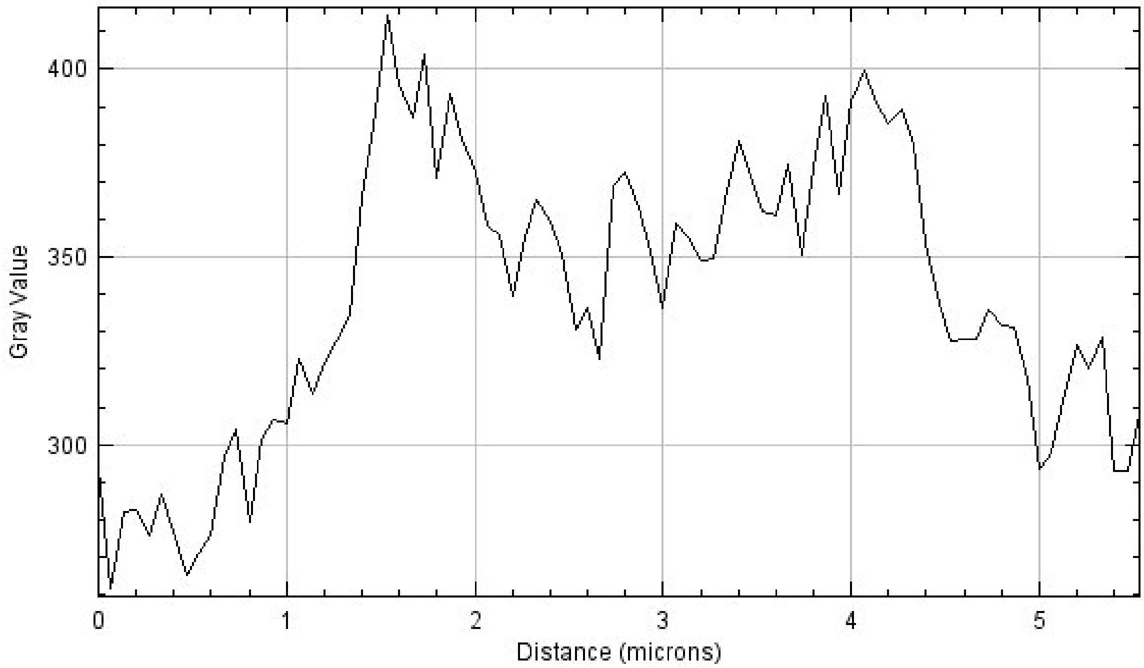

Figure S548 – Intensity profile of line scan generated for cell 6, Figure S542.

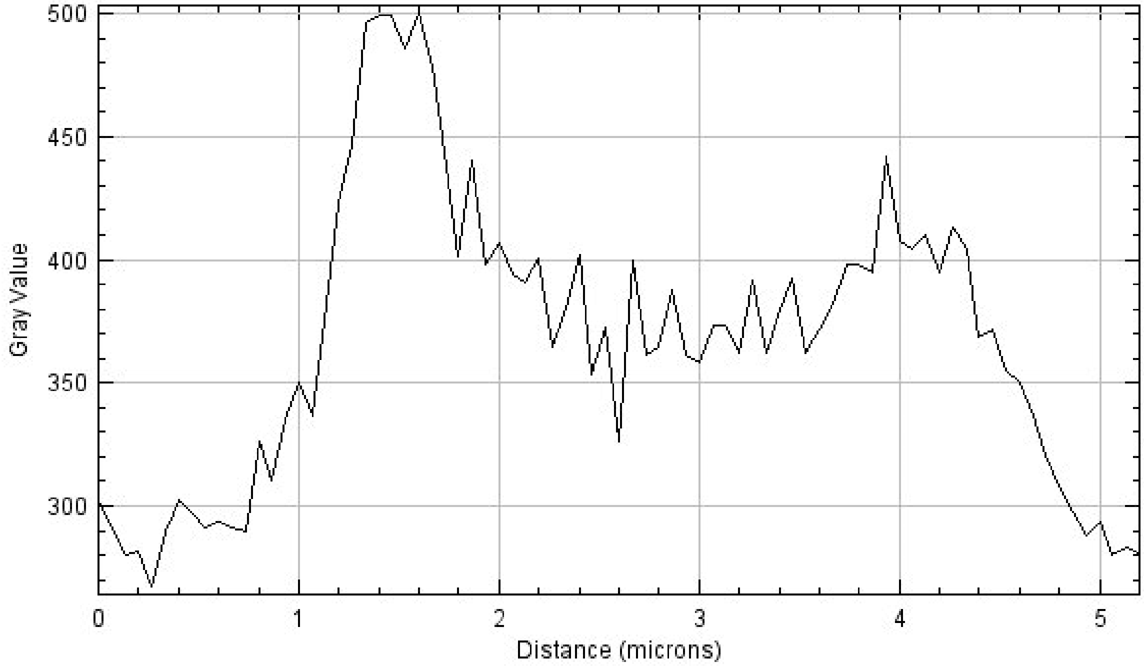

Figure S549 – Intensity profile of line scan generated for cell 7, Figure S542.

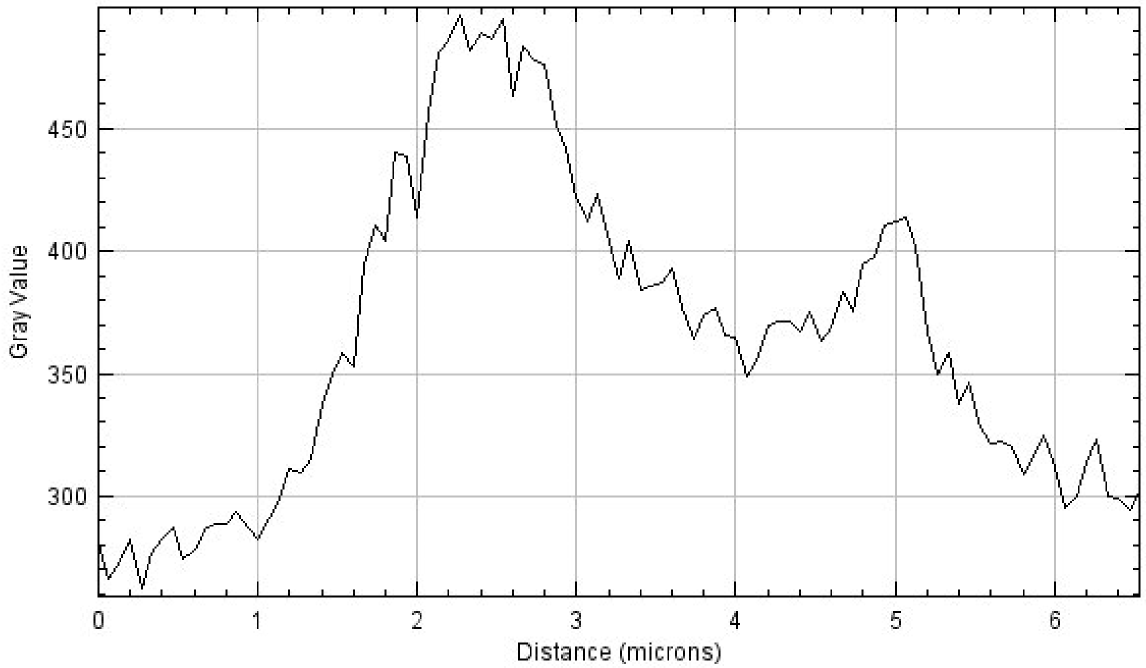

Figure S550 – Intensity profile of line scan generated for cell 8, Figure S542.

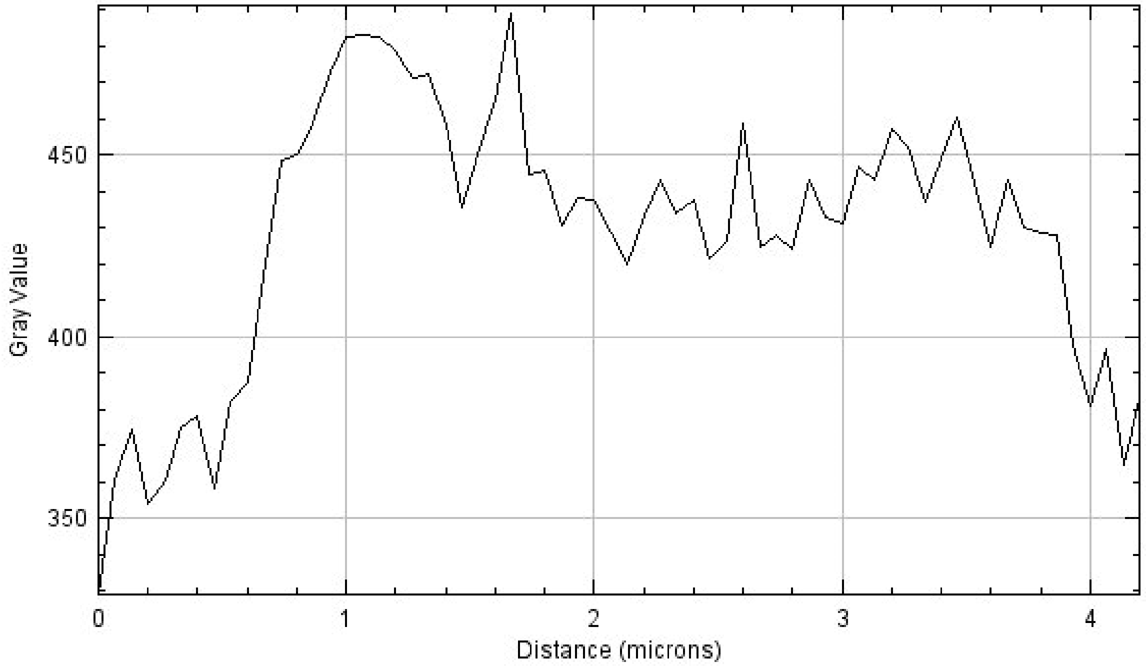

Figure S551 – Intensity profile of line scan generated for cell 9, Figure S542.

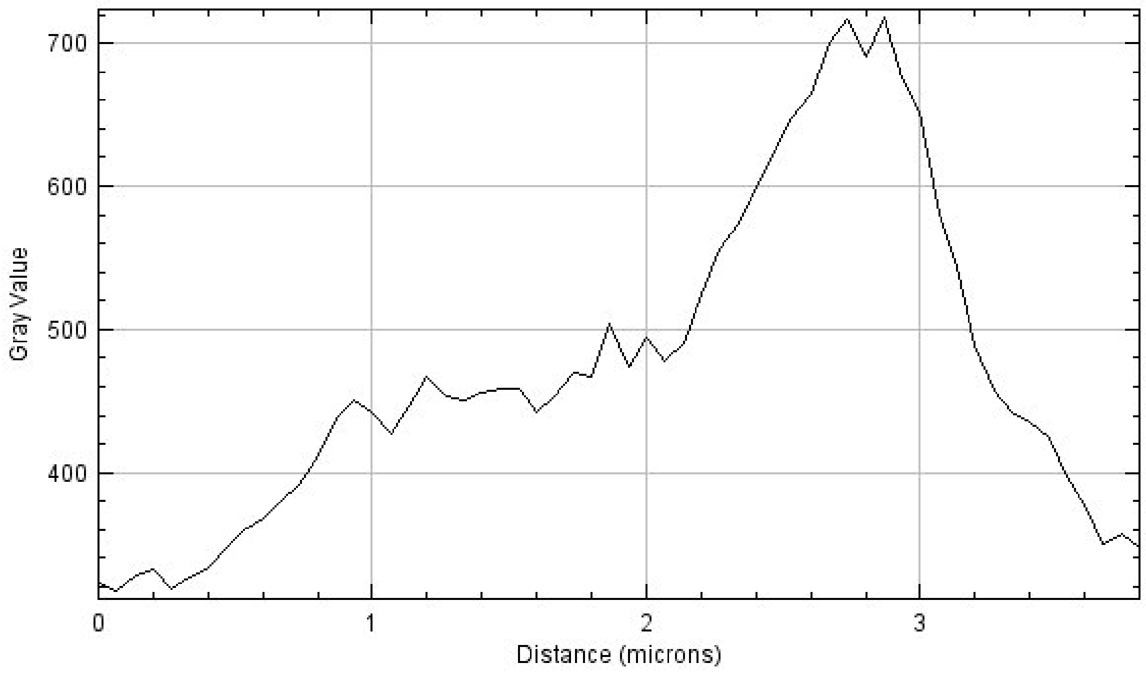

Figure S552 – Intensity profile of line scan generated for cell 10, Figure S542.

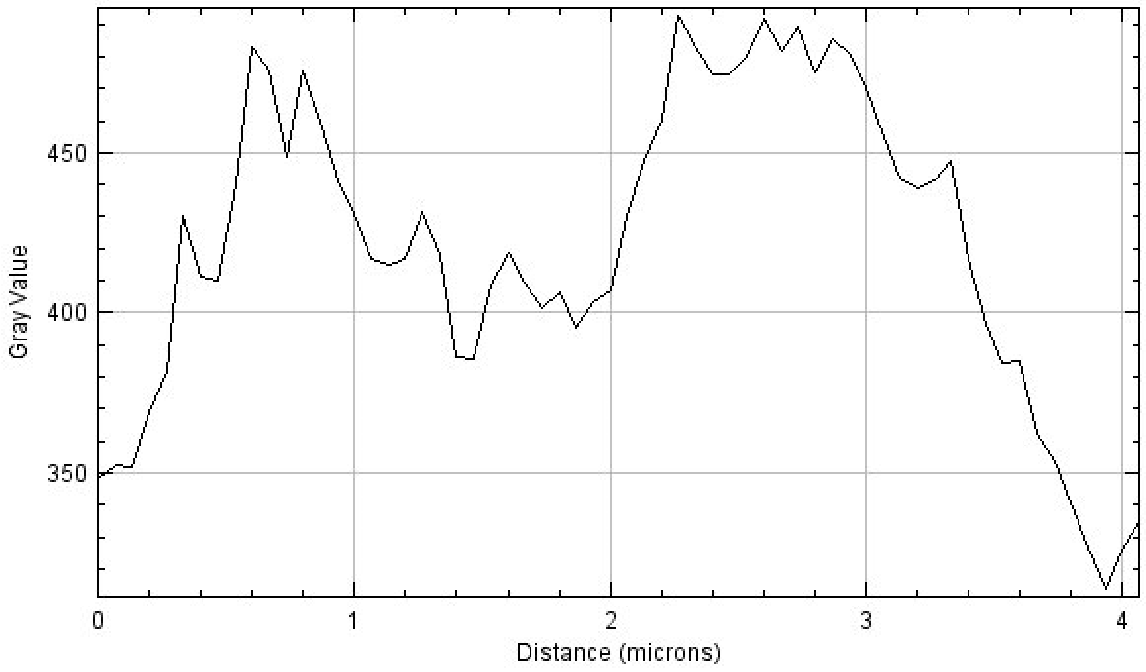

Figure S553 – Intensity profile of line scan generated for cell 11, Figure S542.

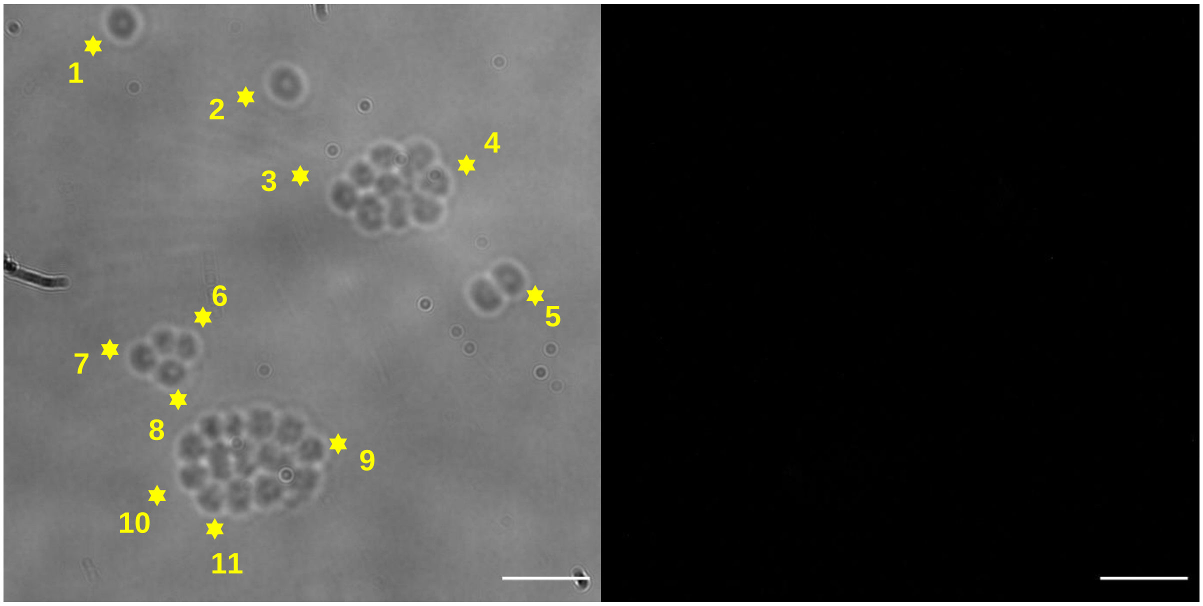

Figure S554 – MRSA transmitted image and fluorescence image at 605 nm used in fluorescence intensity calculations in the presence of both compound **39** and **FM4-64** at T = 30 minutes. Transmitted image used to locate cells on fluorescence image. Scale bar = 10 μM.

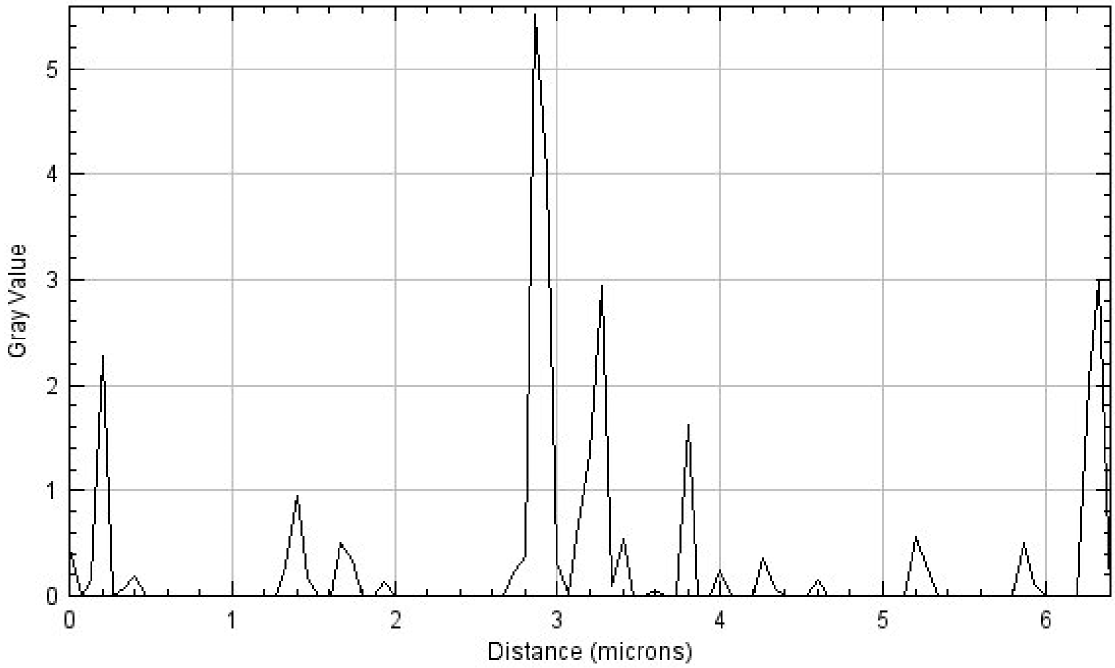

Figure S555 – Intensity profile of line scan generated for cell 1, Figure S554.

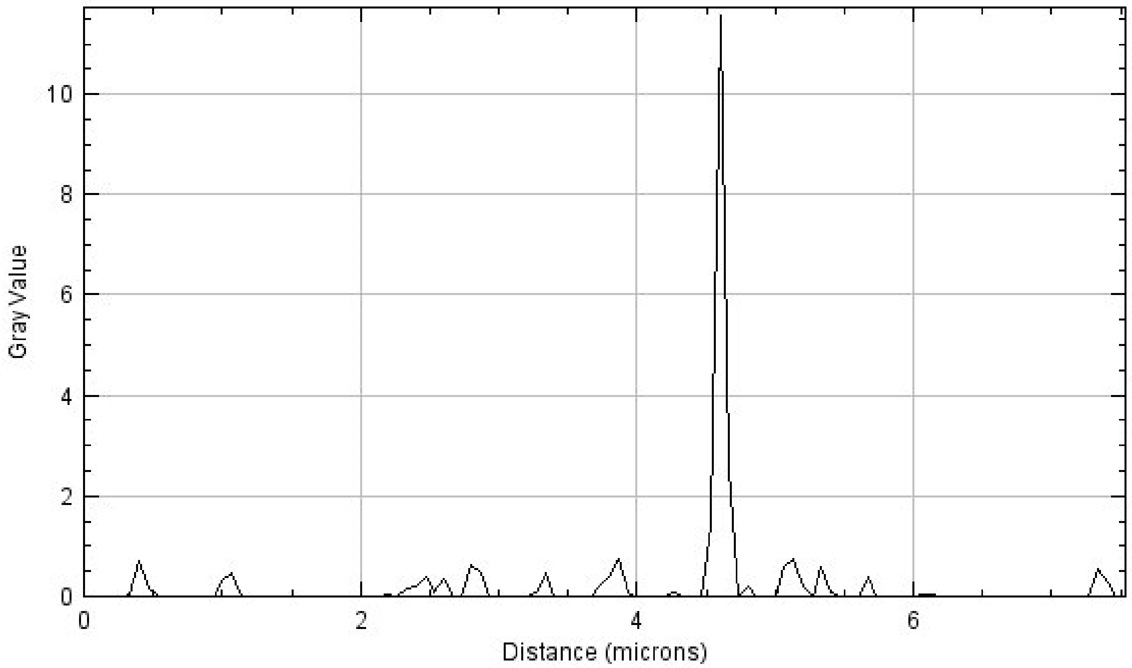

Figure S556 – Intensity profile of line scan generated for cell 2, Figure S554.

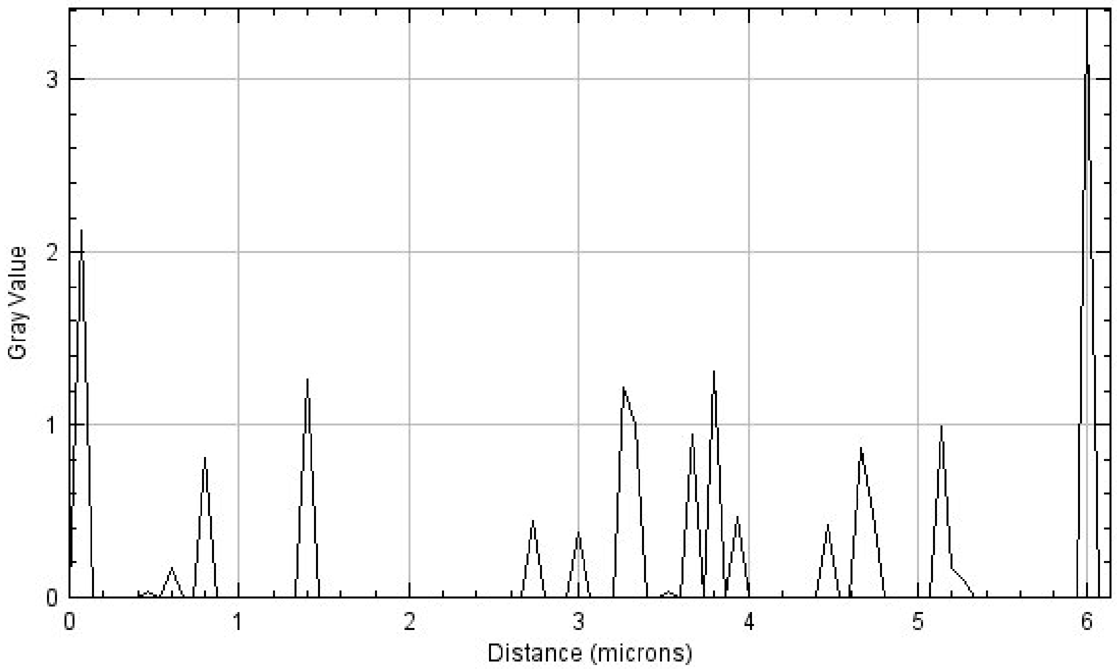

Figure S557 – Intensity profile of line scan generated for cell 3, Figure S554.

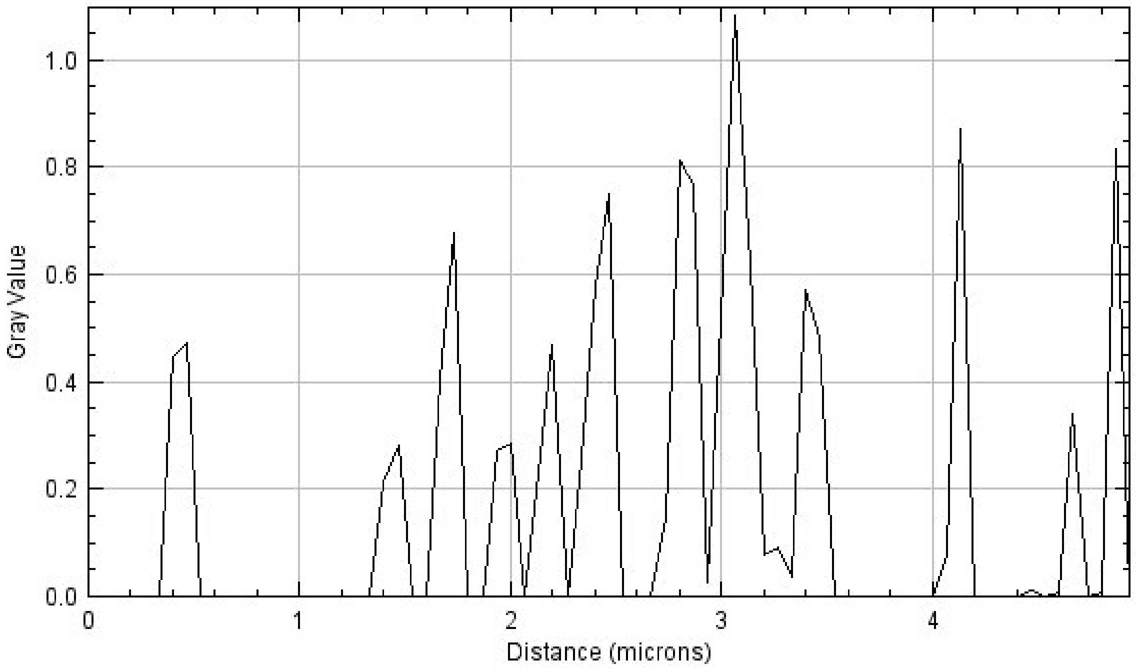

Figure S558 – Intensity profile of line scan generated for cell 4, Figure S554.

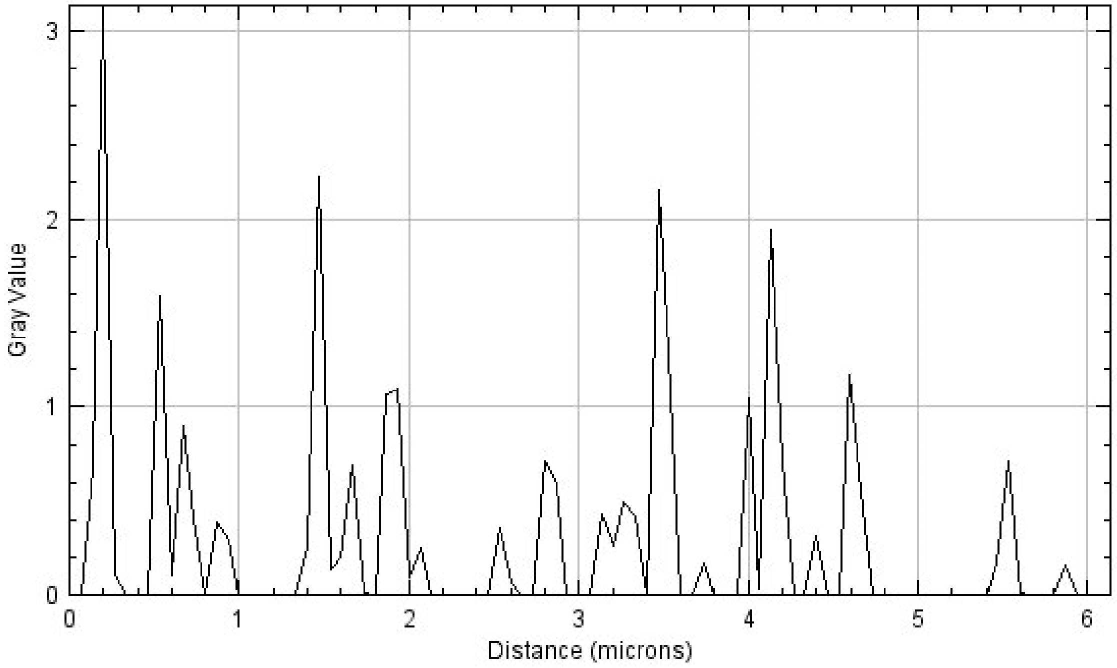

Figure S559 – Intensity profile of line scan generated for cell 5, Figure S554.

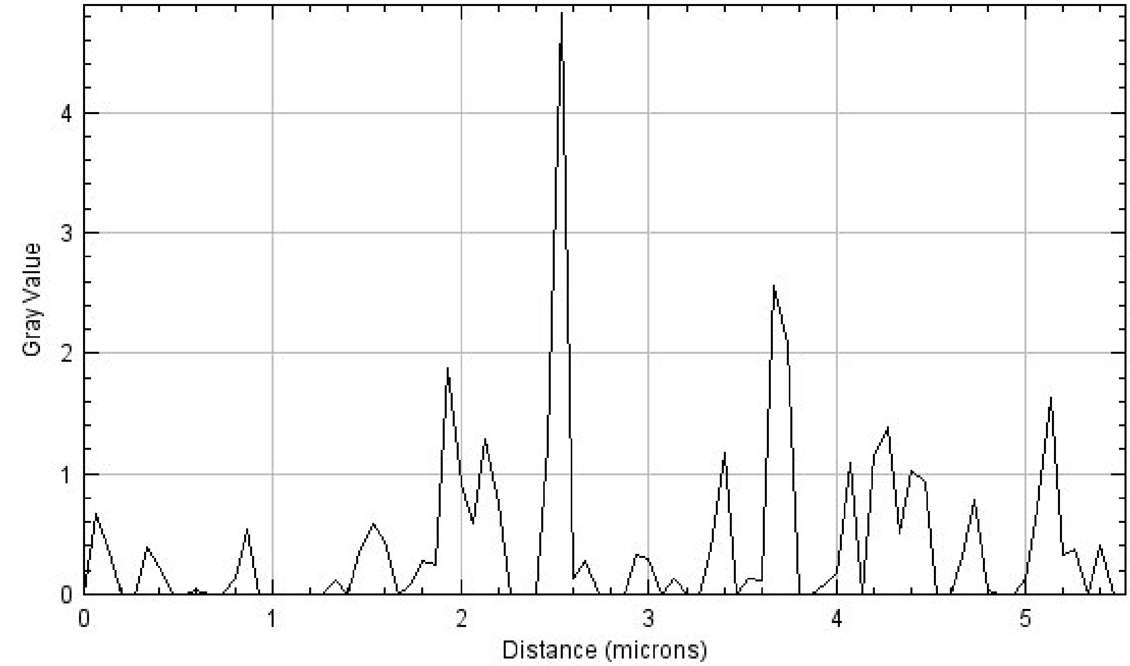

Figure S560 – Intensity profile of line scan generated for cell 6, Figure S554.

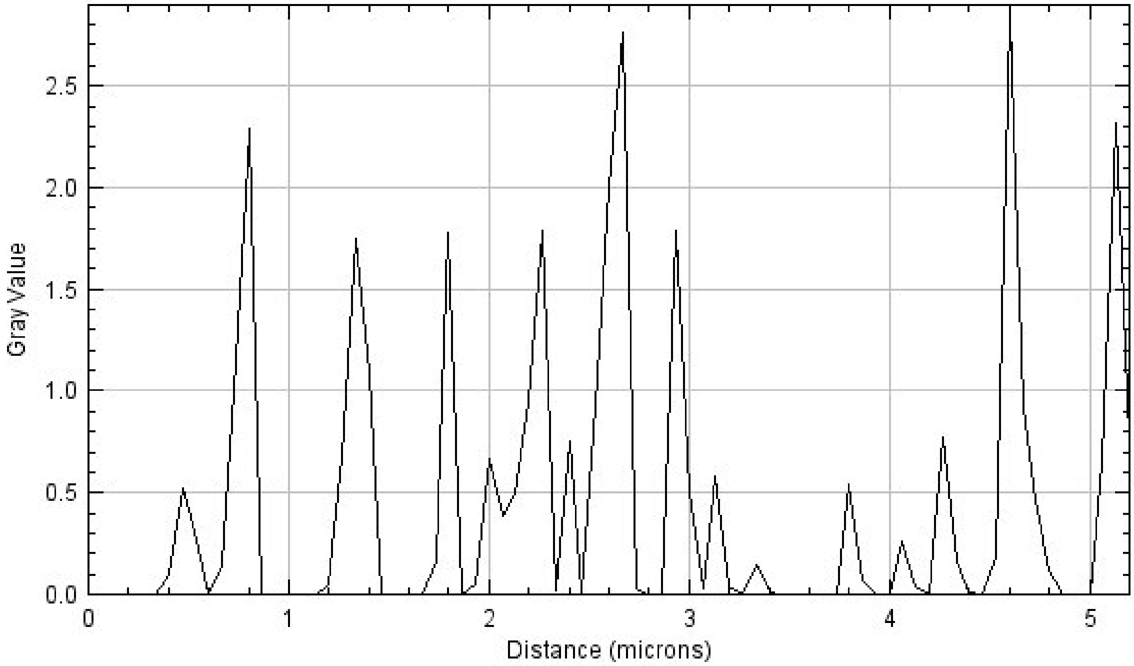

Figure S561 – Intensity profile of line scan generated for cell 7, Figure S554.

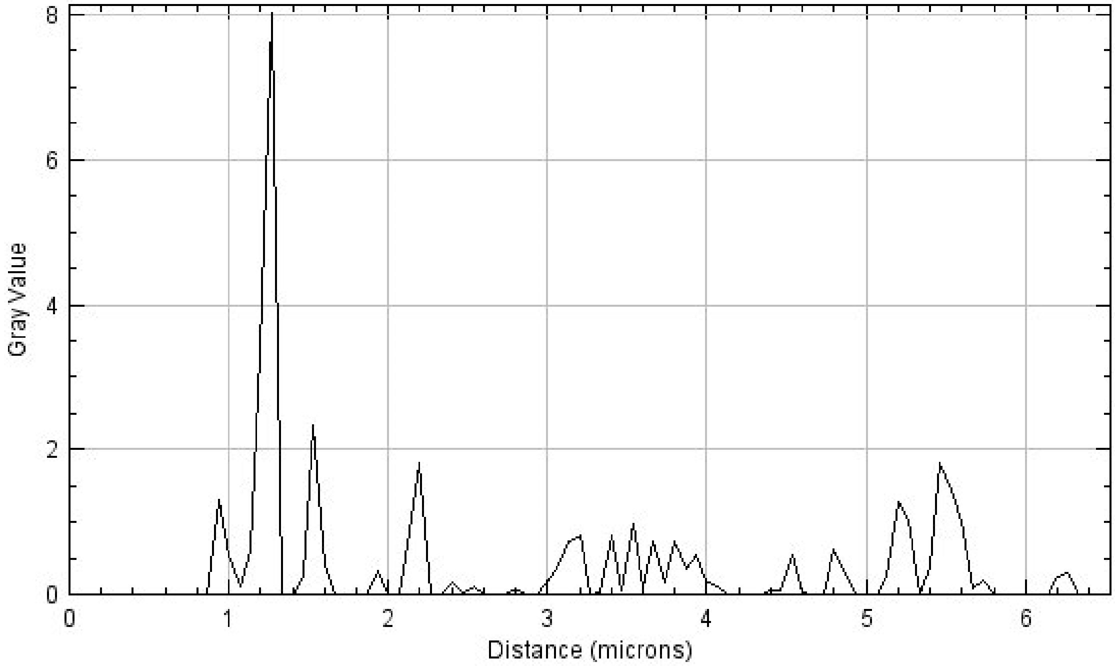

Figure S562 – Intensity profile of line scan generated for cell 8, Figure S554.

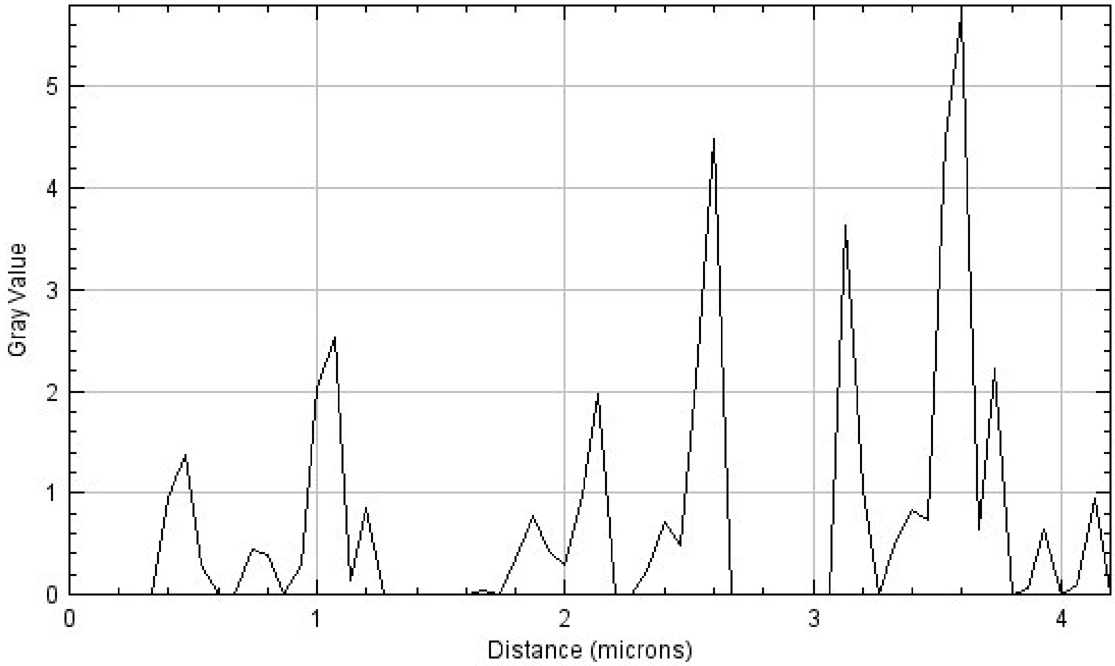

Figure S563 – Intensity profile of line scan generated for cell 9, Figure S554.

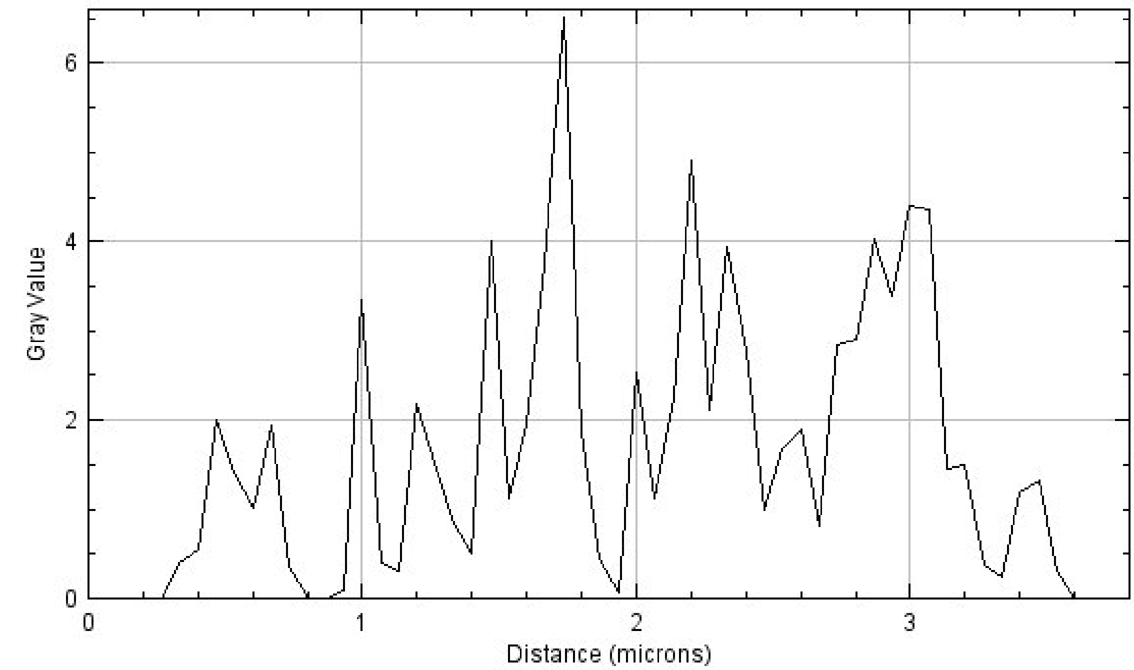

Figure S564 – Intensity profile of line scan generated for cell 10, Figure S554.

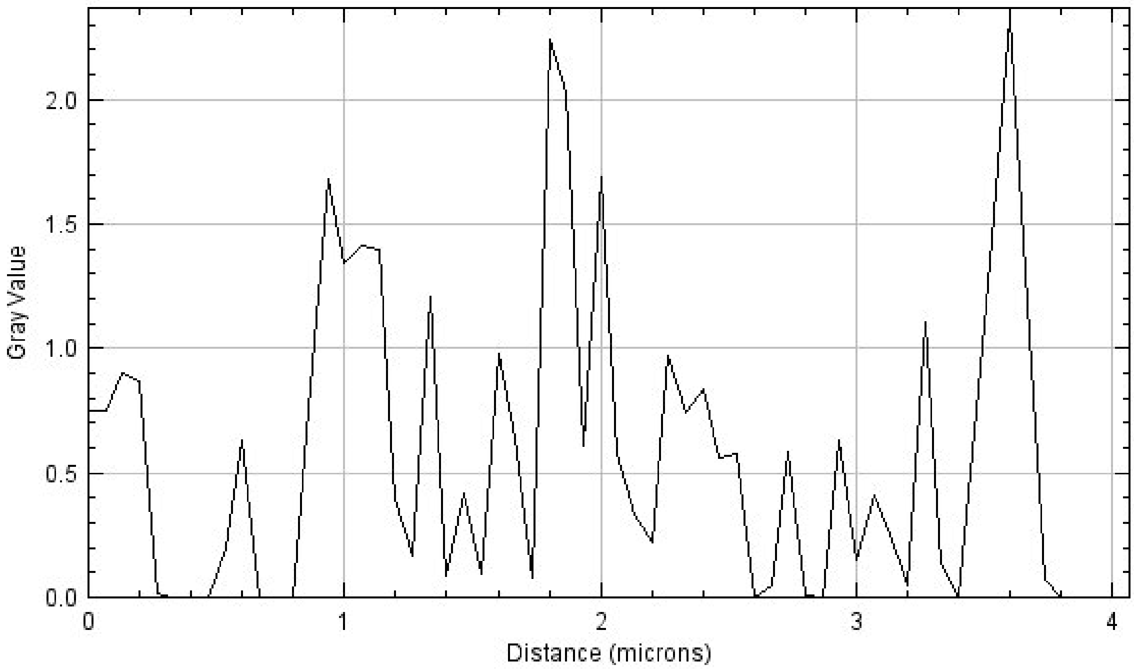

Figure S565 – Intensity profile of line scan generated for cell 11, Figure S554.

E. coli

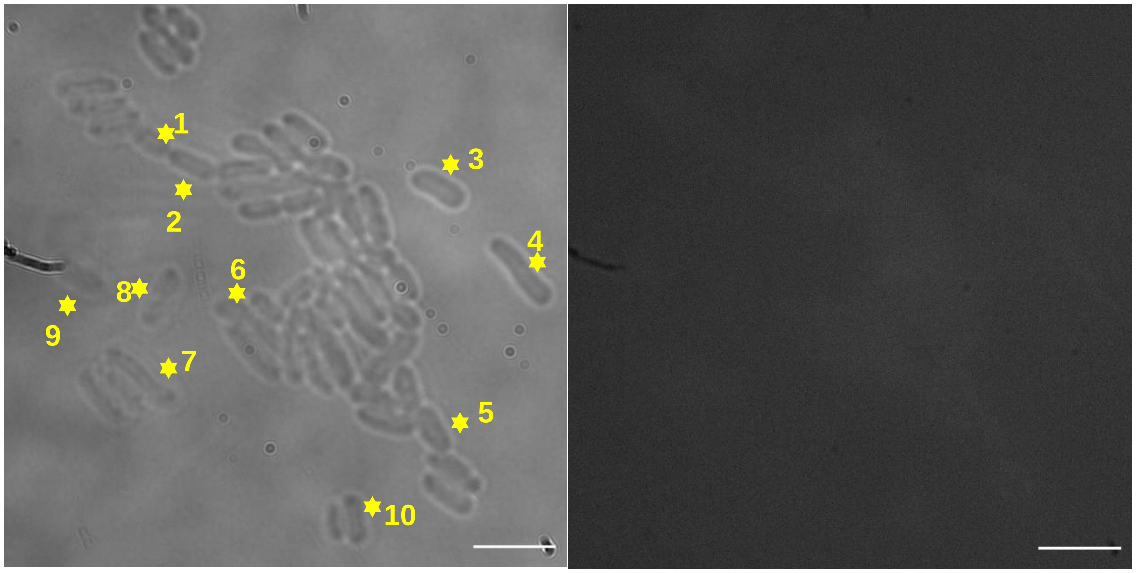

Figure S566 – *E. coli* transmitted and DAPI images used in fluorescence intensity calculations in the absence of both compound **39** and FM4-64 at T = 30 minutes. Transmitted image used to locate cells on DAPI. Scale bar = 10 μM.

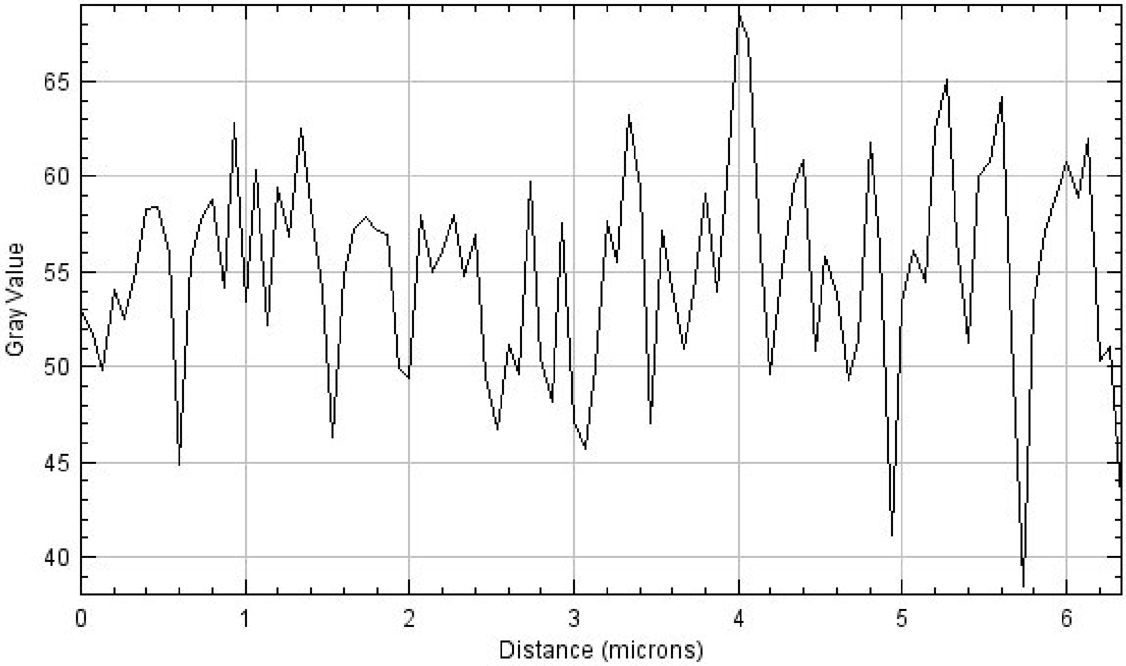

Figure S567 – Intensity profile of line scan generated for cell 1, Figure S566.

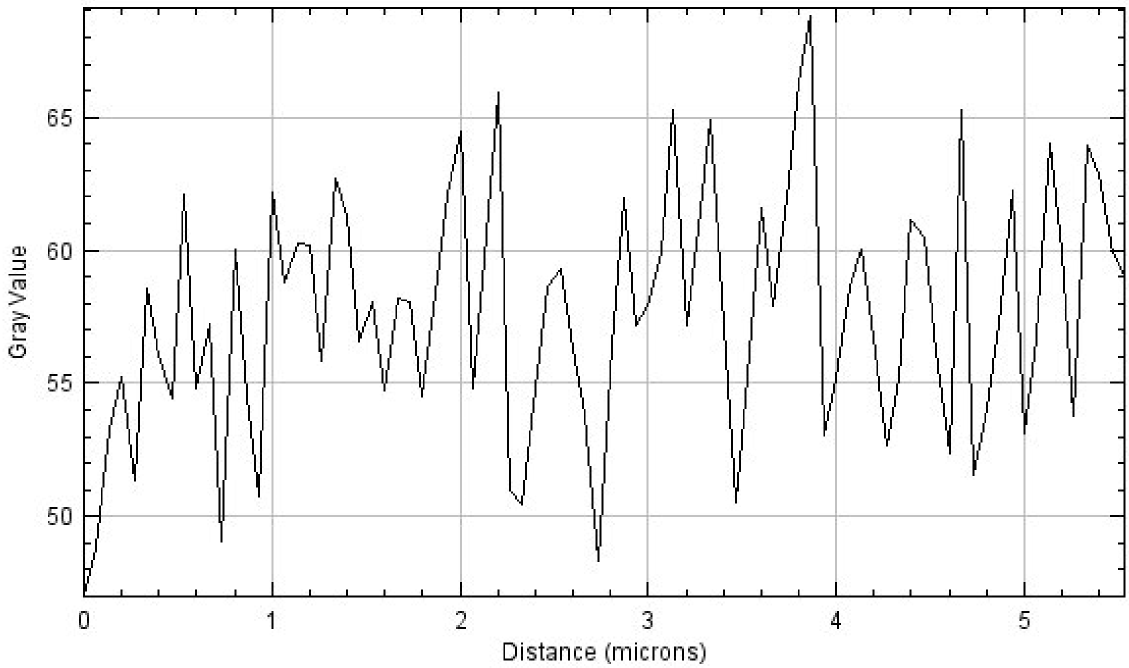

Figure S568 – Intensity profile of line scan generated for cell 2, Figure S566.

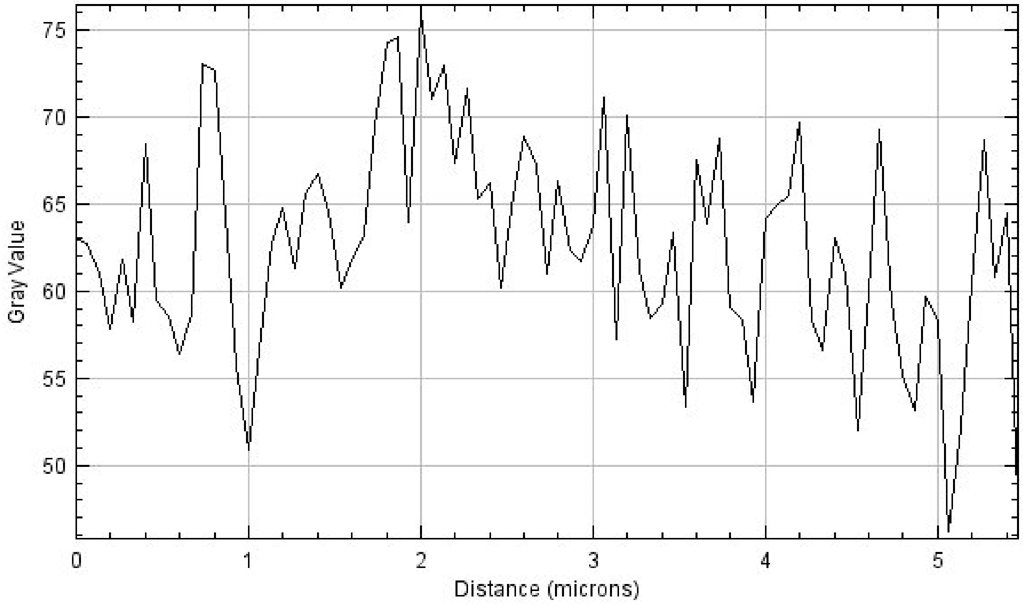

Figure S569 – Intensity profile of line scan generated for cell 3, Figure S566.

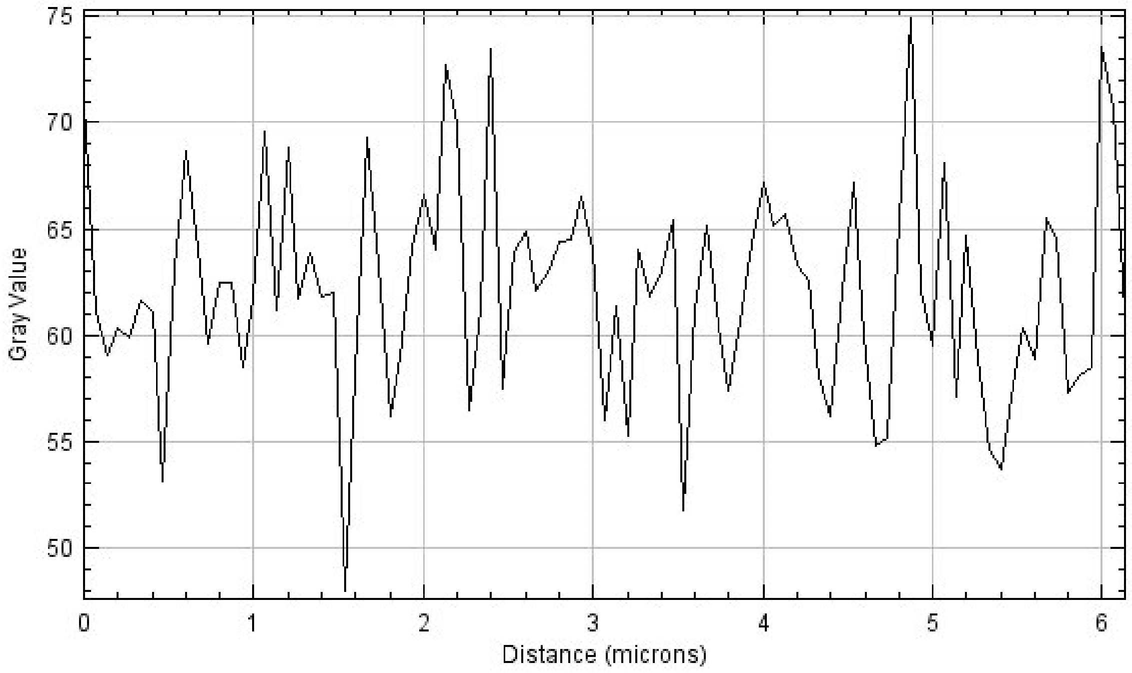

Figure S570 – Intensity profile of line scan generated for cell 4, Figure S566.

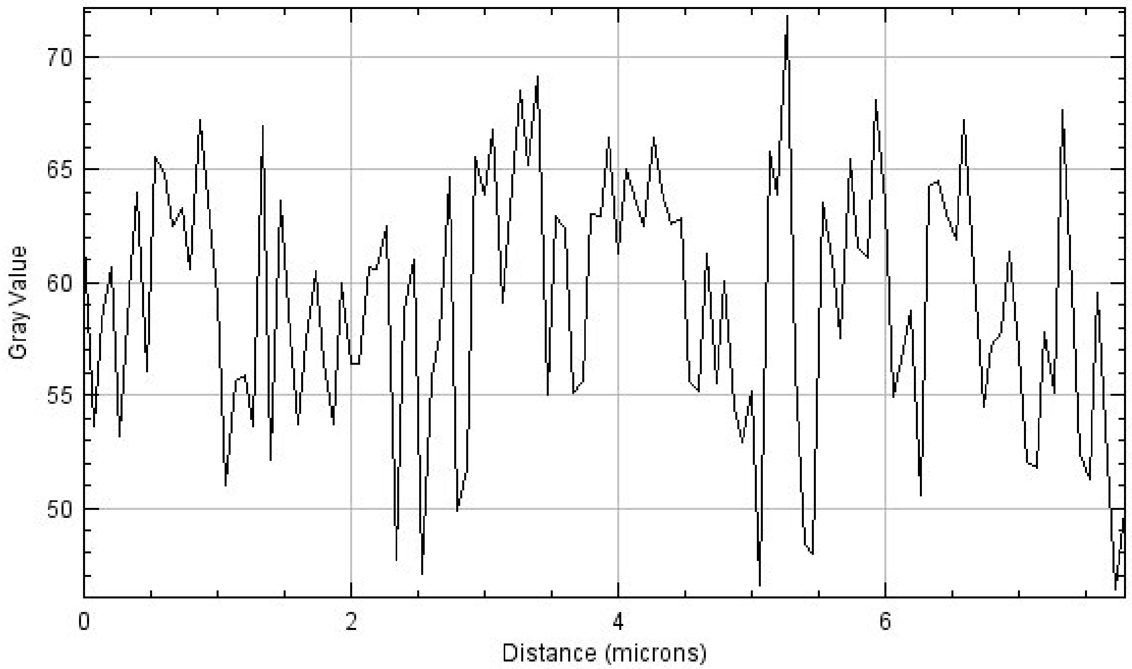

Figure S571 – Intensity profile of line scan generated for cell 5, Figure S566.

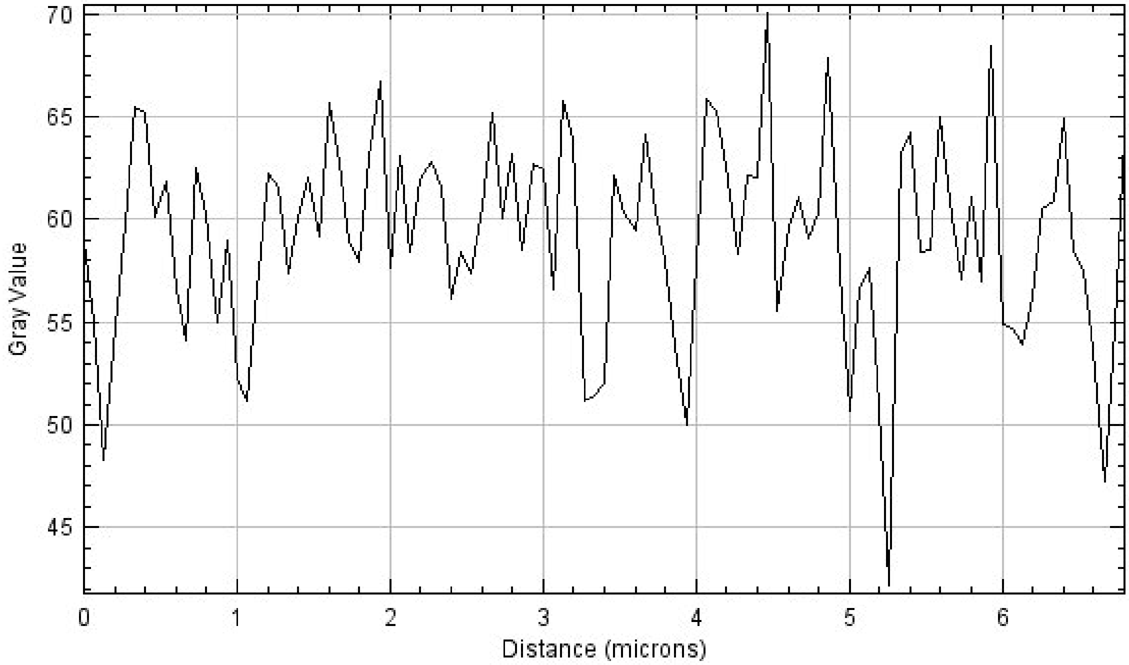

Figure S572 – Intensity profile of line scan generated for cell 6, Figure S566.

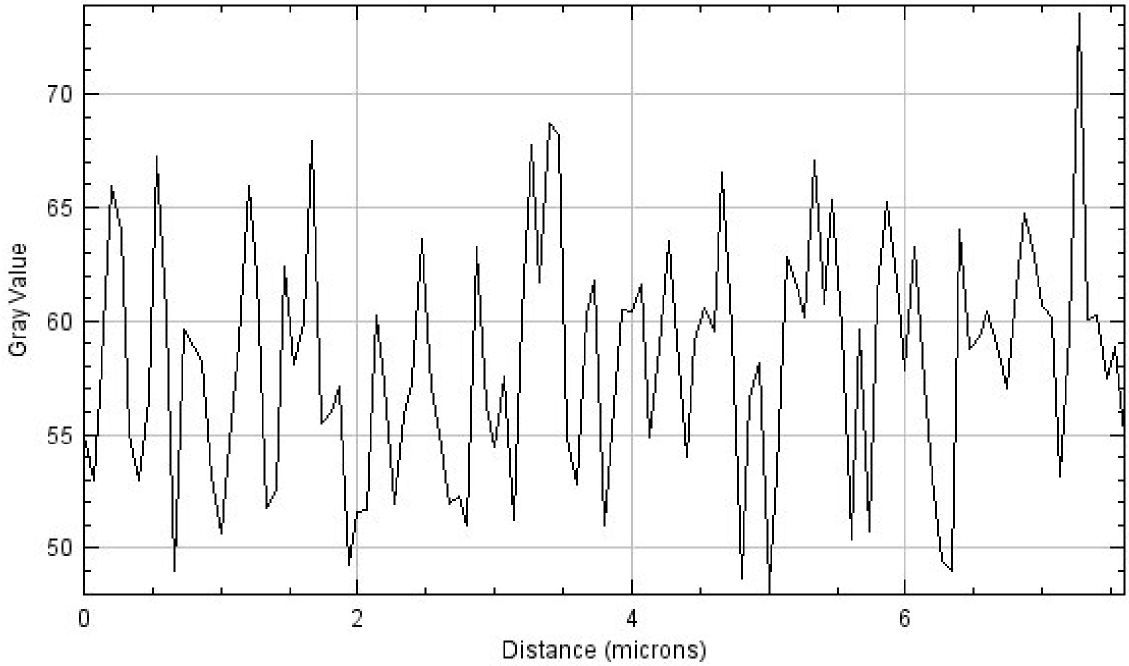

Figure S573 – Intensity profile of line scan generated for cell 7, Figure S566.

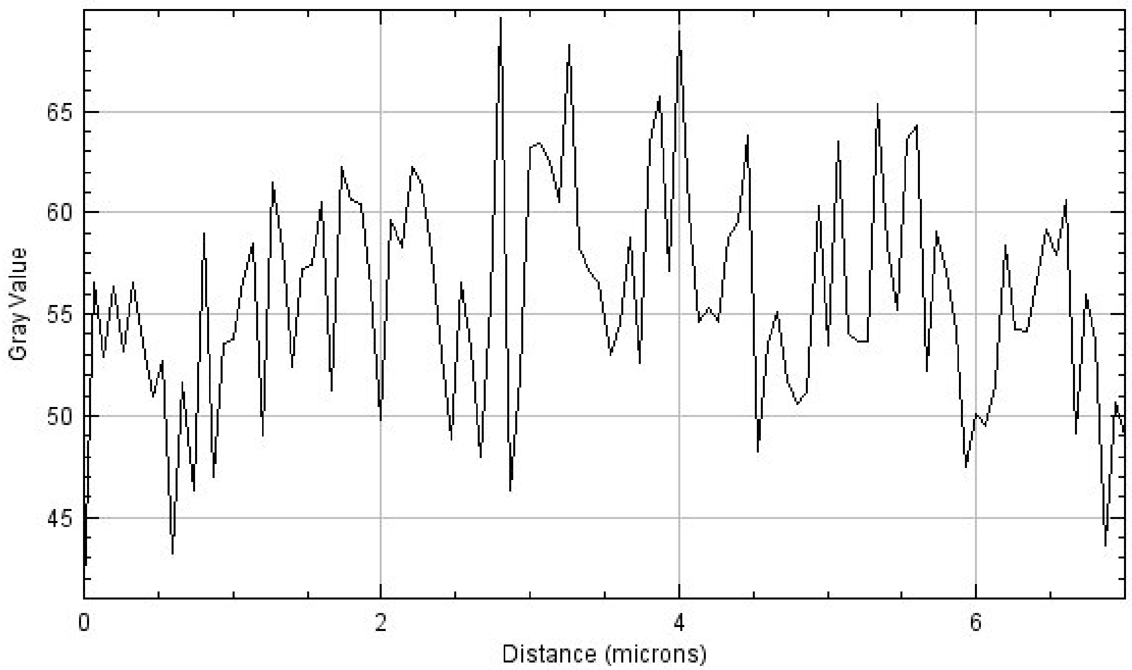

Figure S574 – Intensity profile of line scan generated for cell 8, Figure S566.

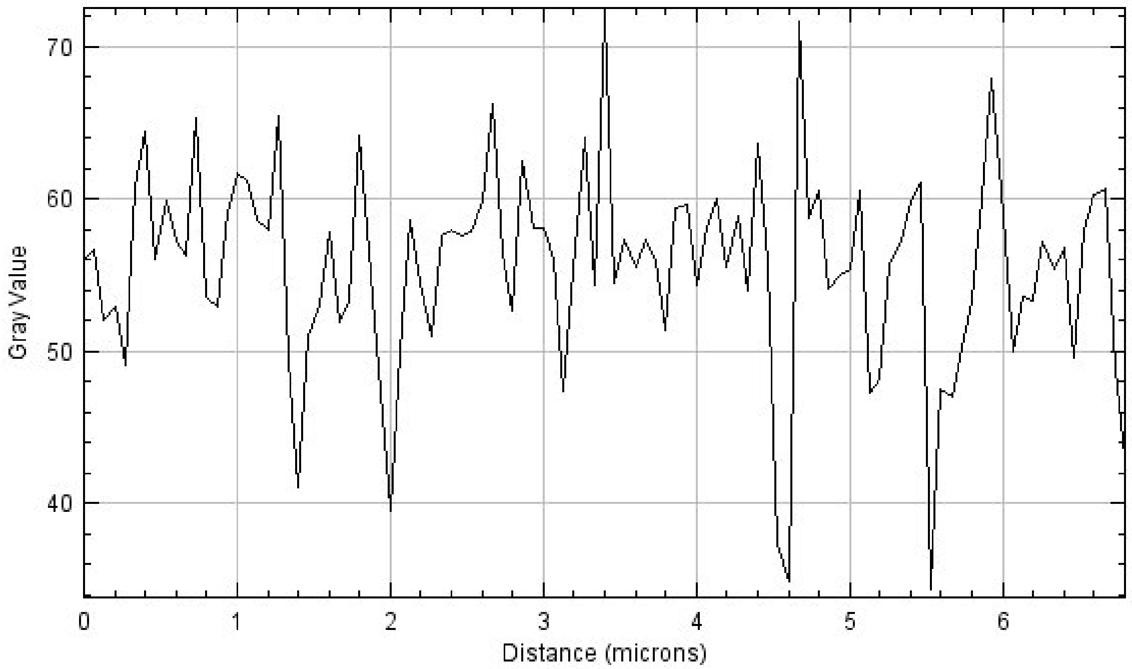

Figure S575 – Intensity profile of line scan generated for cell 9, Figure S566.

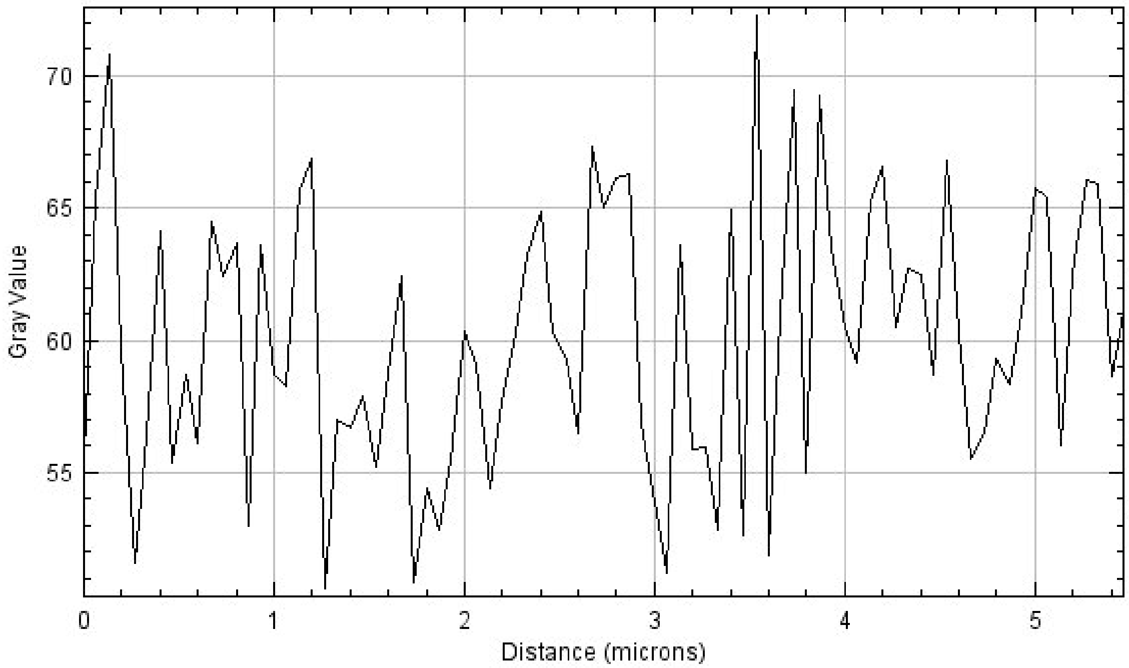

Figure S576 – Intensity profile of line scan generated for cell 10, Figure S566.

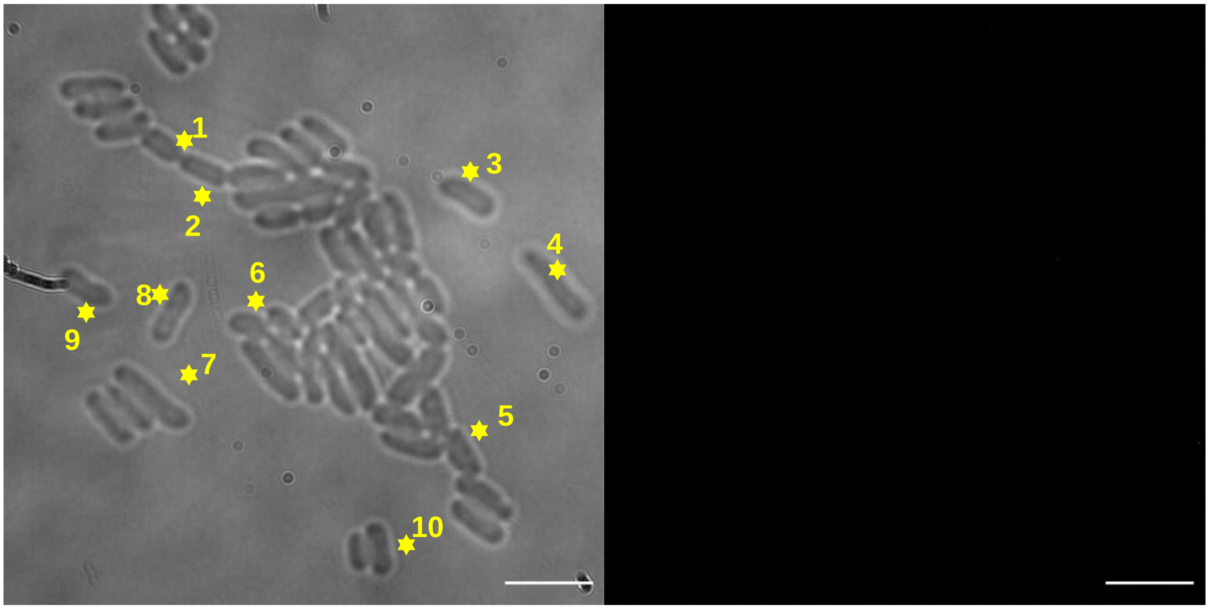

Figure S577 – *E. coli* transmitted and mCherry images used in fluorescence intensity calculations in the absence of both compound **39** and FM4-64 at T = 30 minutes. Transmitted image used to locate cells on mCherry image. Scale bar = 10 μM.

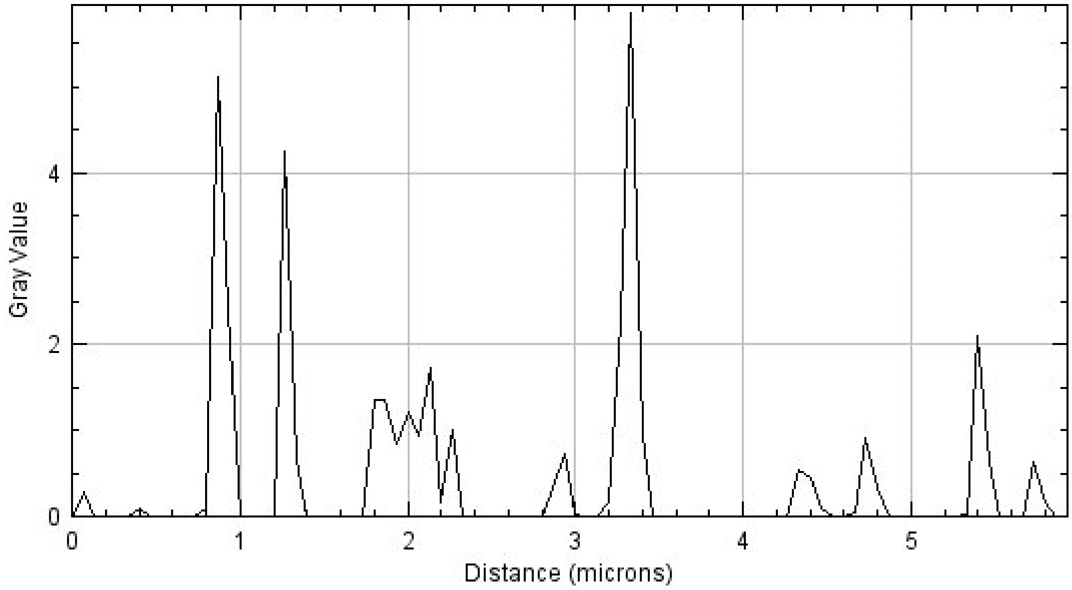

Figure S578 – Intensity profile of line scan generated for cell 1, Figure S577.

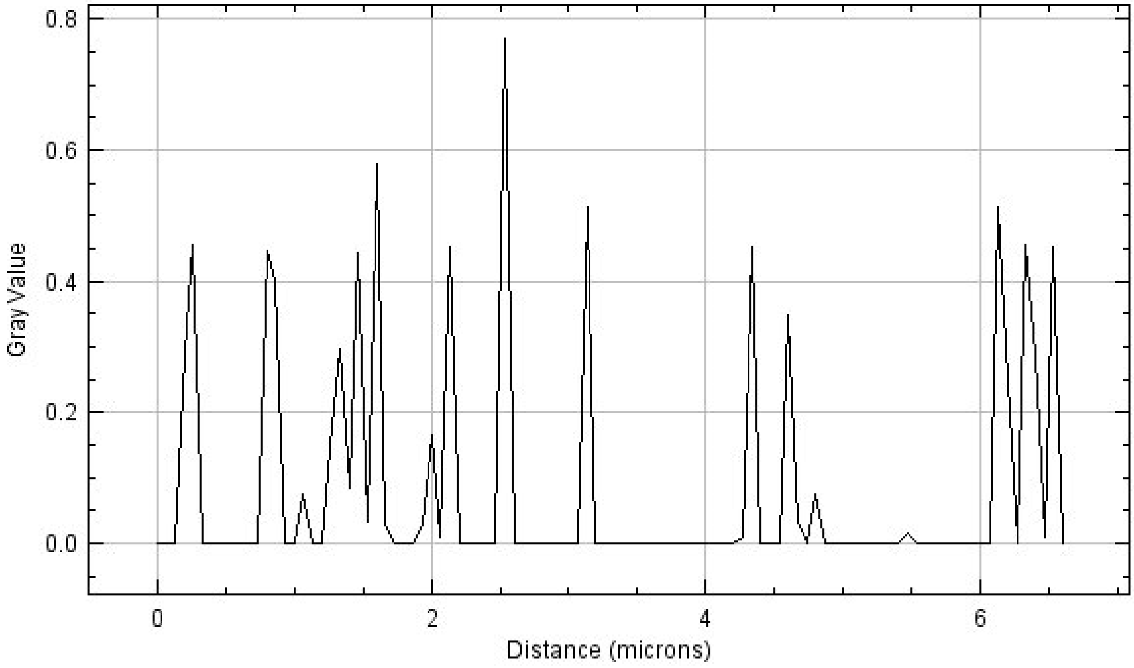

Figure S579 – Intensity profile of line scan generated for cell 2, Figure S577.

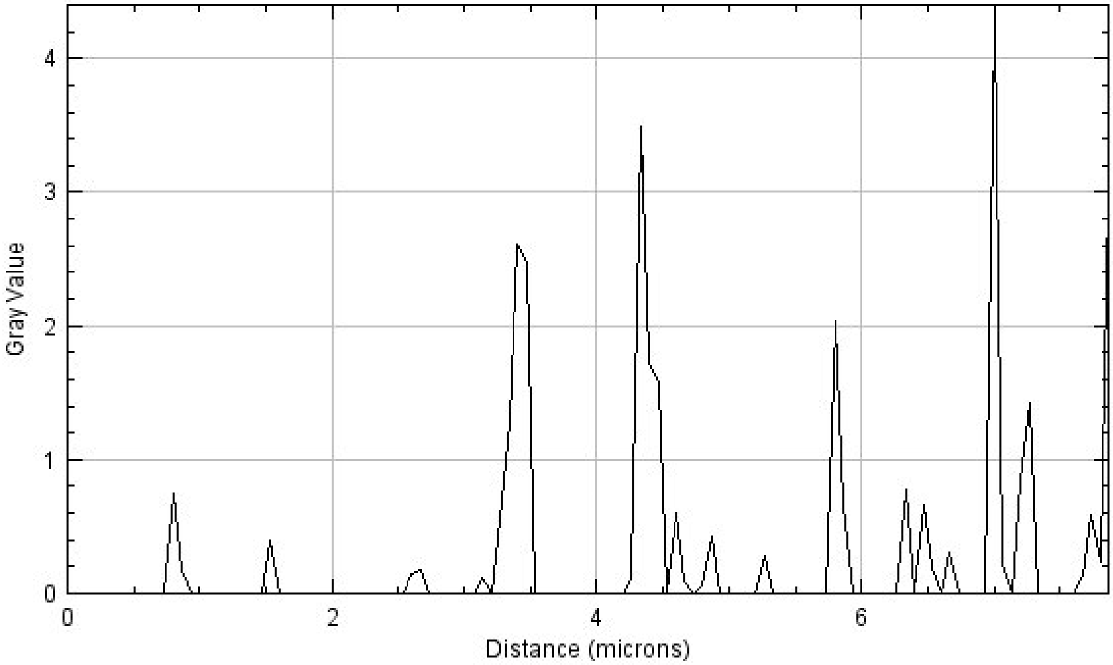

Figure S580 – Intensity profile of line scan generated for cell 3, Figure S577.

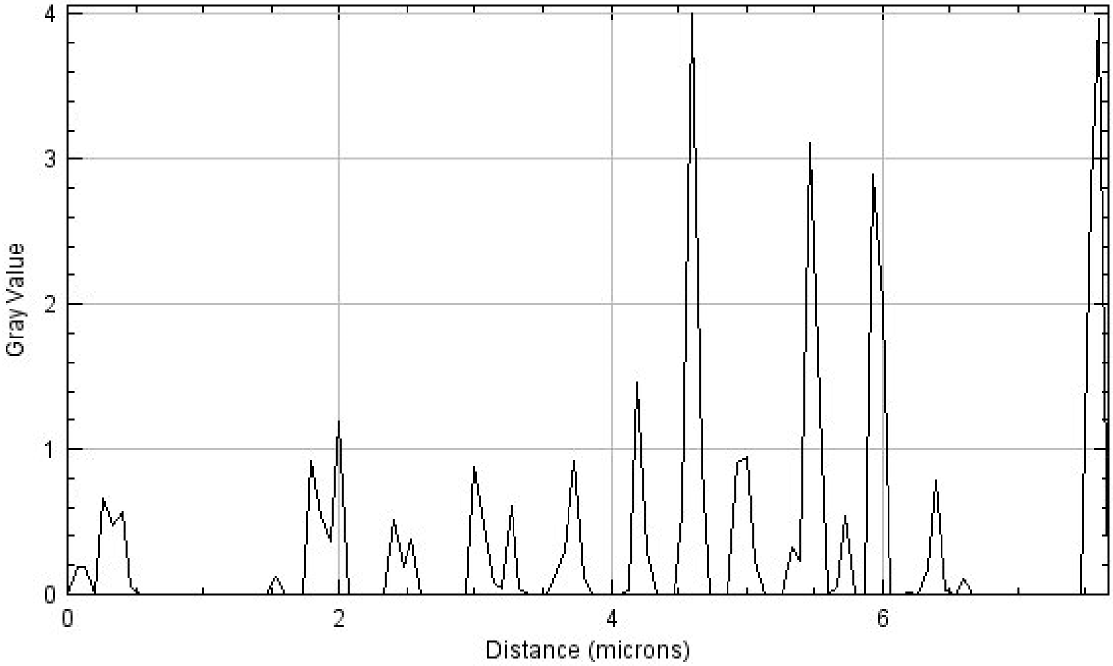

Figure S581 – Intensity profile of line scan generated for cell 4, Figure S577.

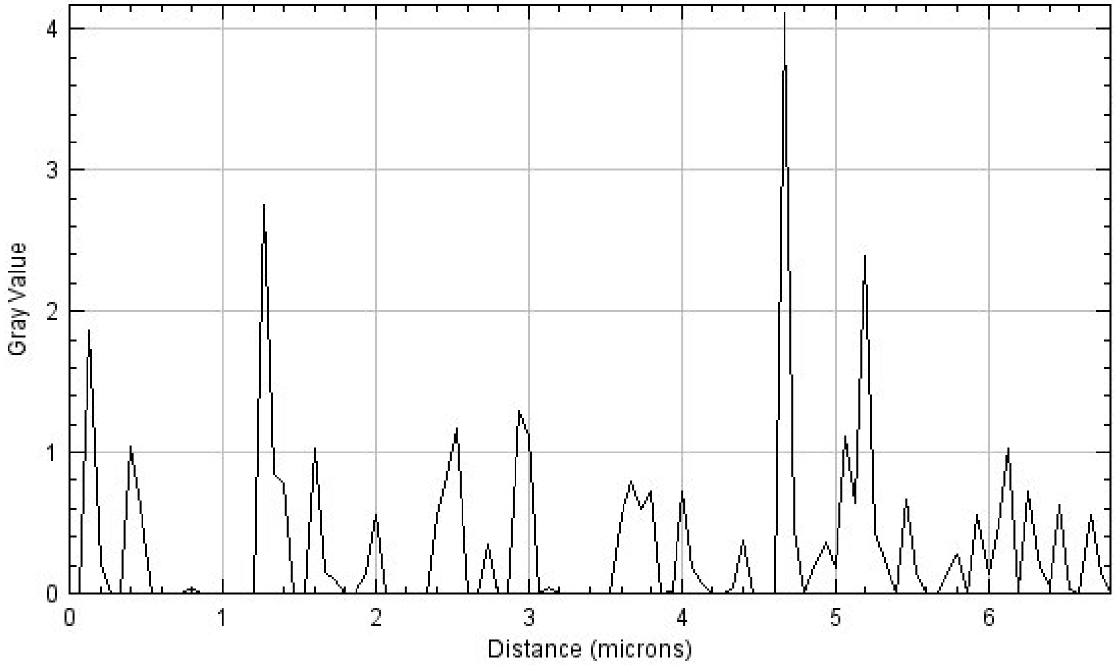

Figure S582 – Intensity profile of line scan generated for cell 5, Figure S577.

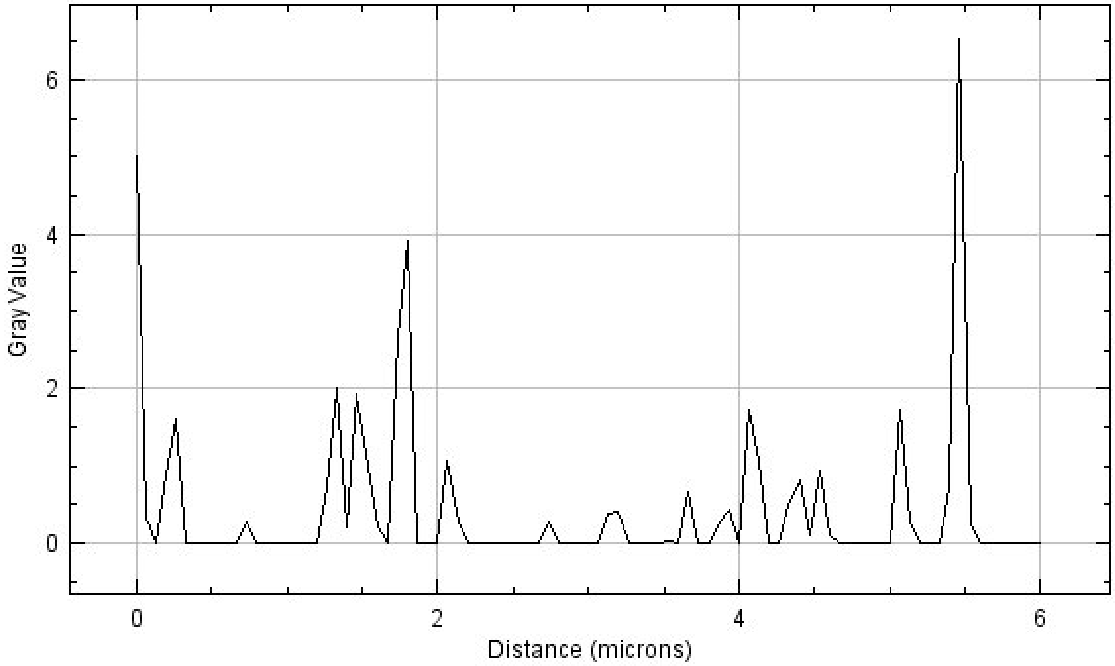

Figure S583 – Intensity profile of line scan generated for cell 6, Figure S577.

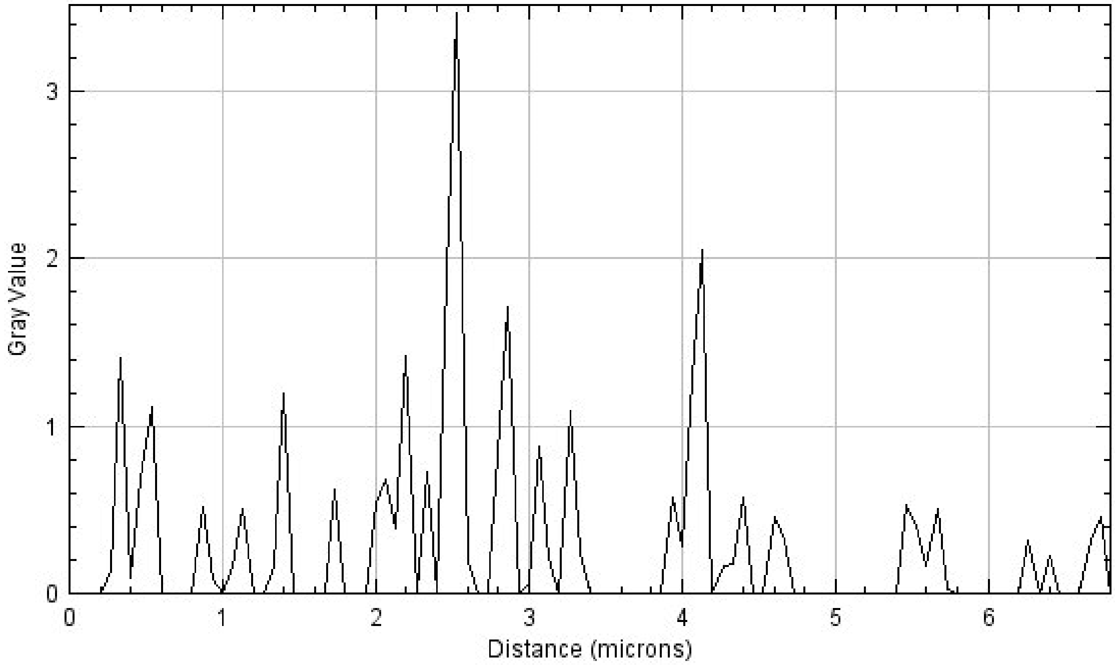

Figure S584 – Intensity profile of line scan generated for cell 7, Figure S577.

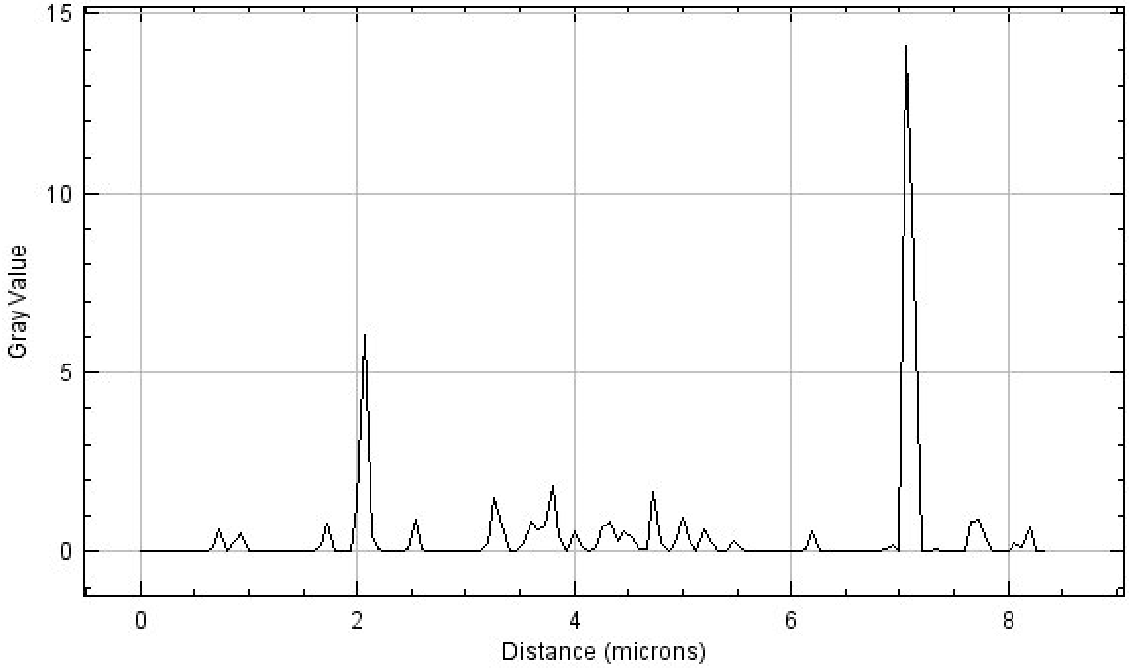

Figure S585 – Intensity profile of line scan generated for cell 8, Figure S577.

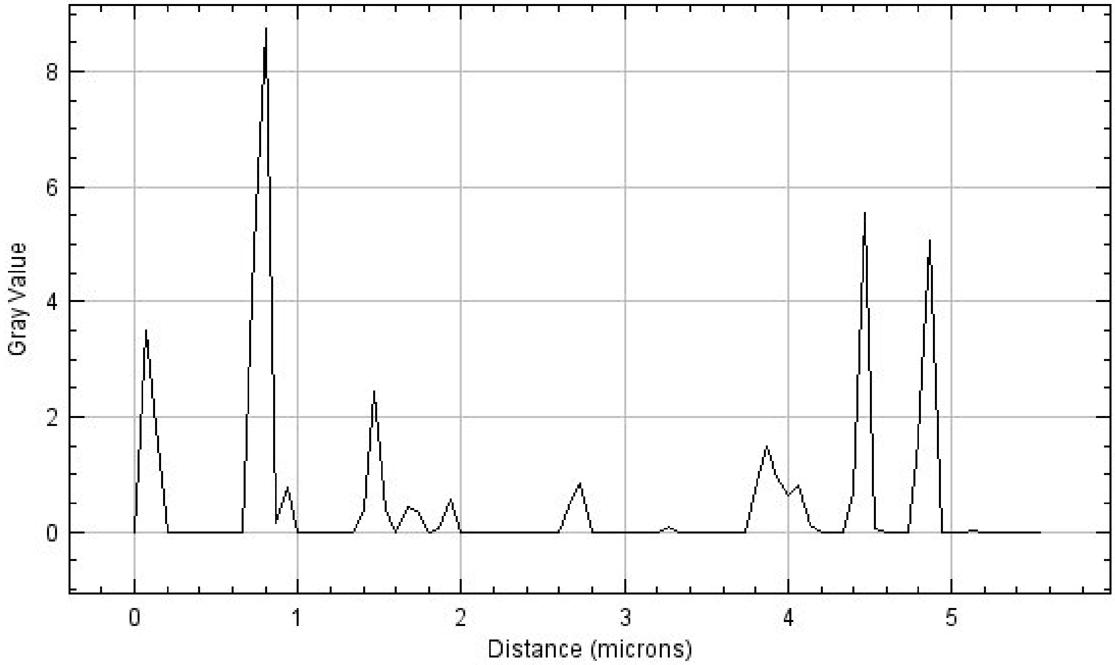

Figure S586 – Intensity profile of line scan generated for cell 9, Figure S577.

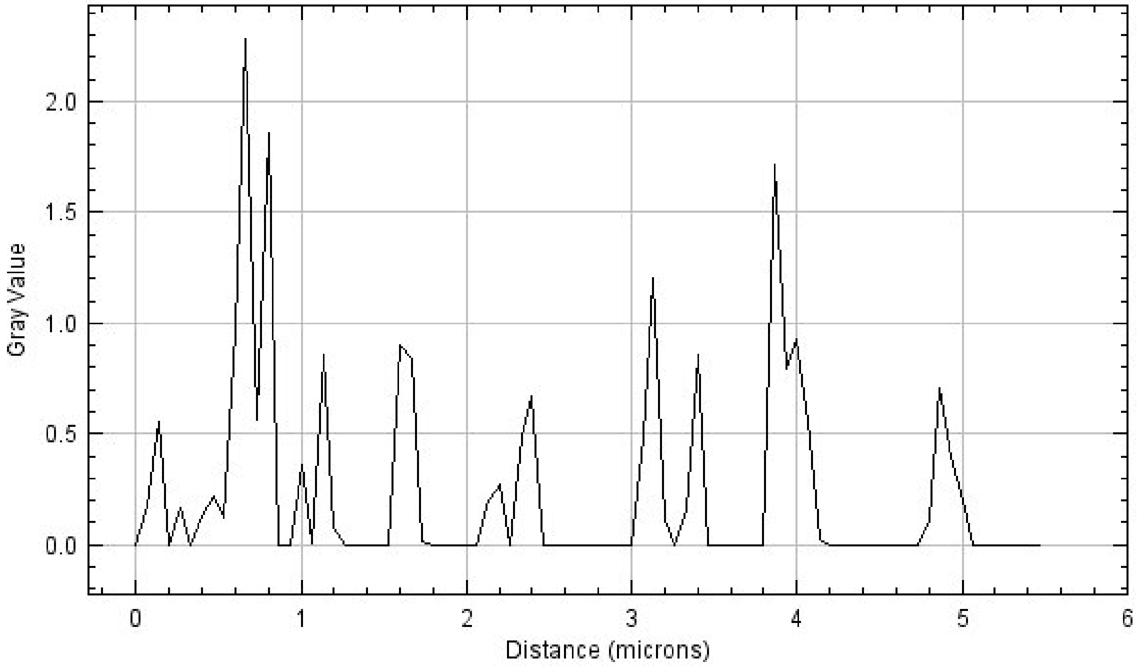

Figure S587 – Intensity profile of line scan generated for cell 10, Figure S577.

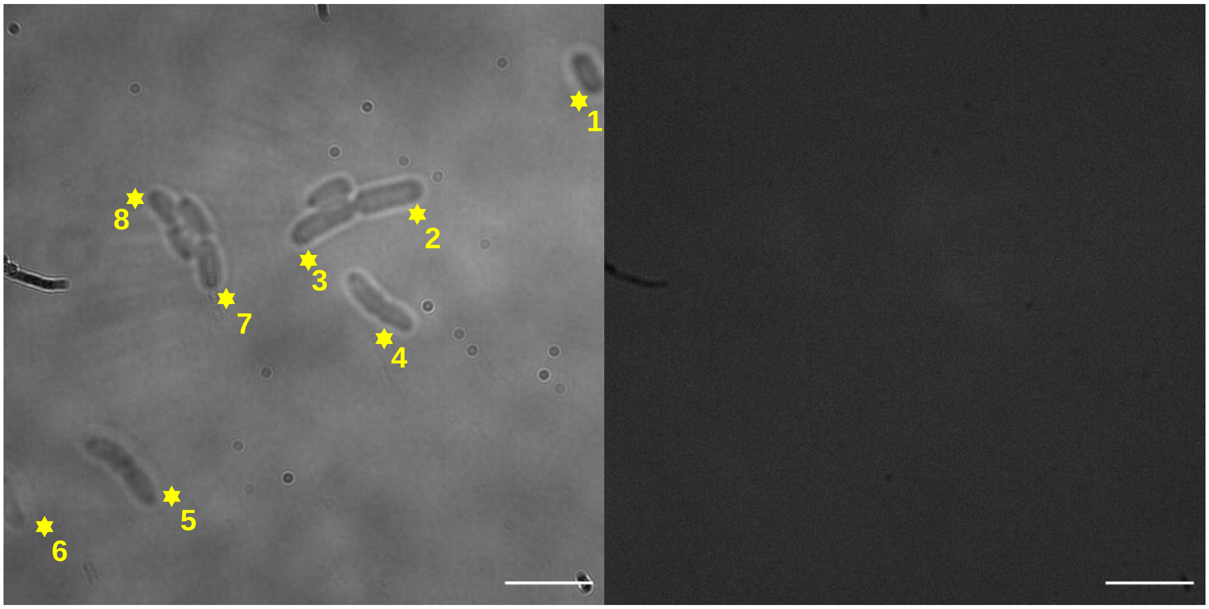

Figure S588 – *E. coli* transmitted and DAPI images used in fluorescence intensity calculations in the absence of both compound **39** and FM4-64 at T = 30 minutes. Transmitted image used to locate cells on DAPI image. Scale bar = 10 µM.

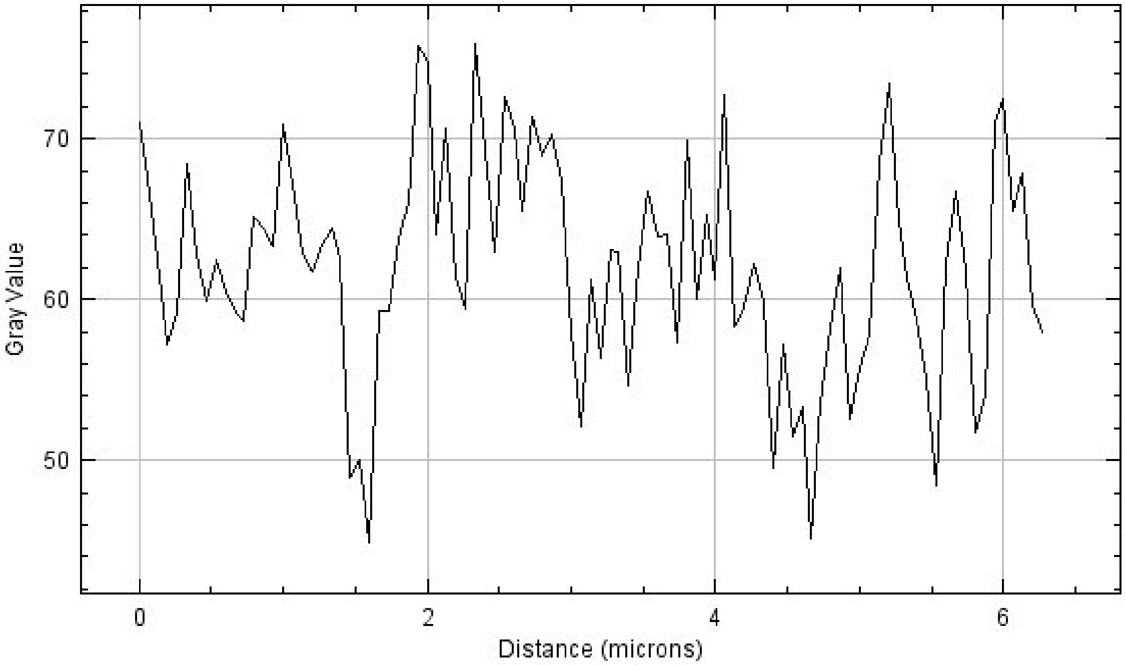

Figure S589 – Intensity profile of line scan generated for cell 1, Figure S588.

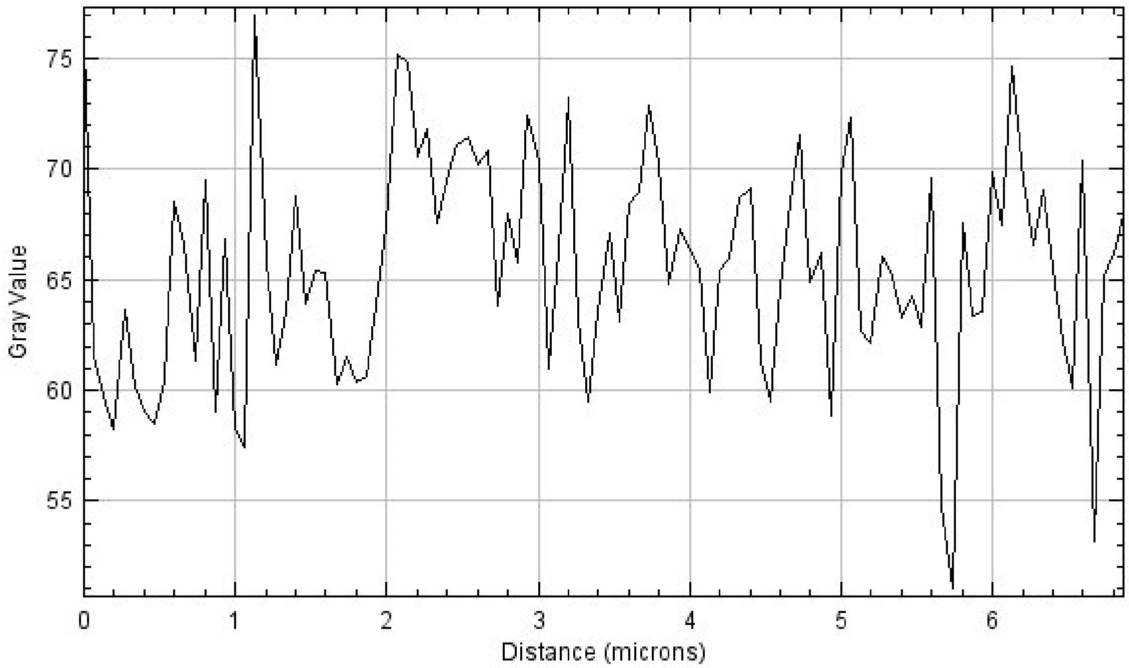

Figure S590 – Intensity profile of line scan generated for cell 2, Figure S588.

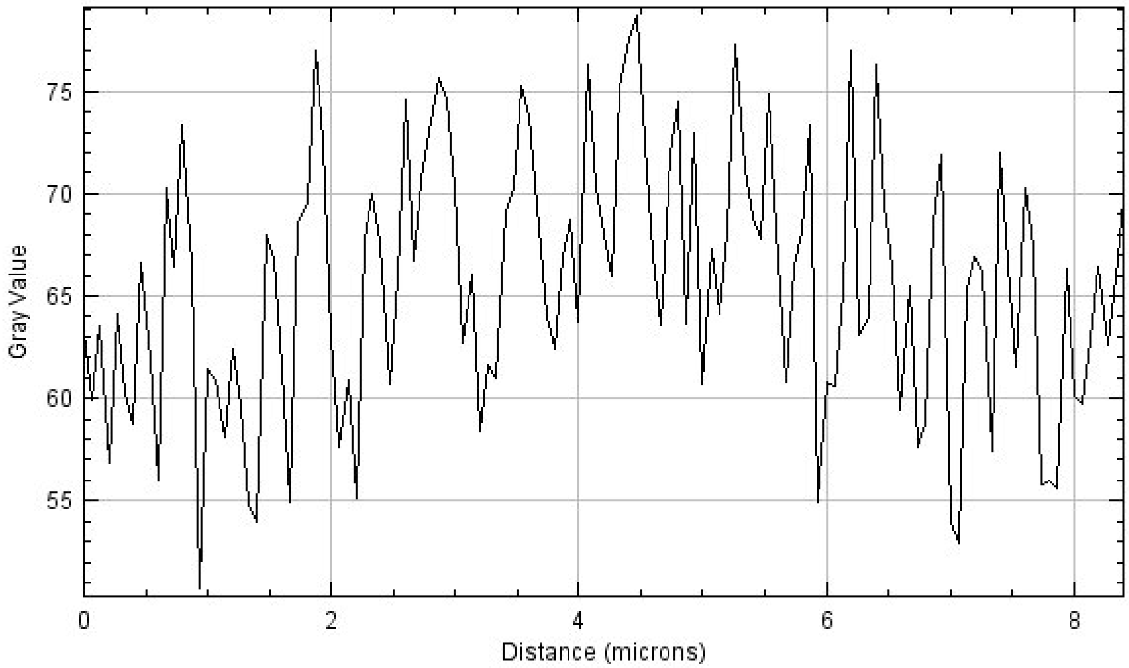

Figure S591 – Intensity profile of line scan generated for cell 3, Figure S588.

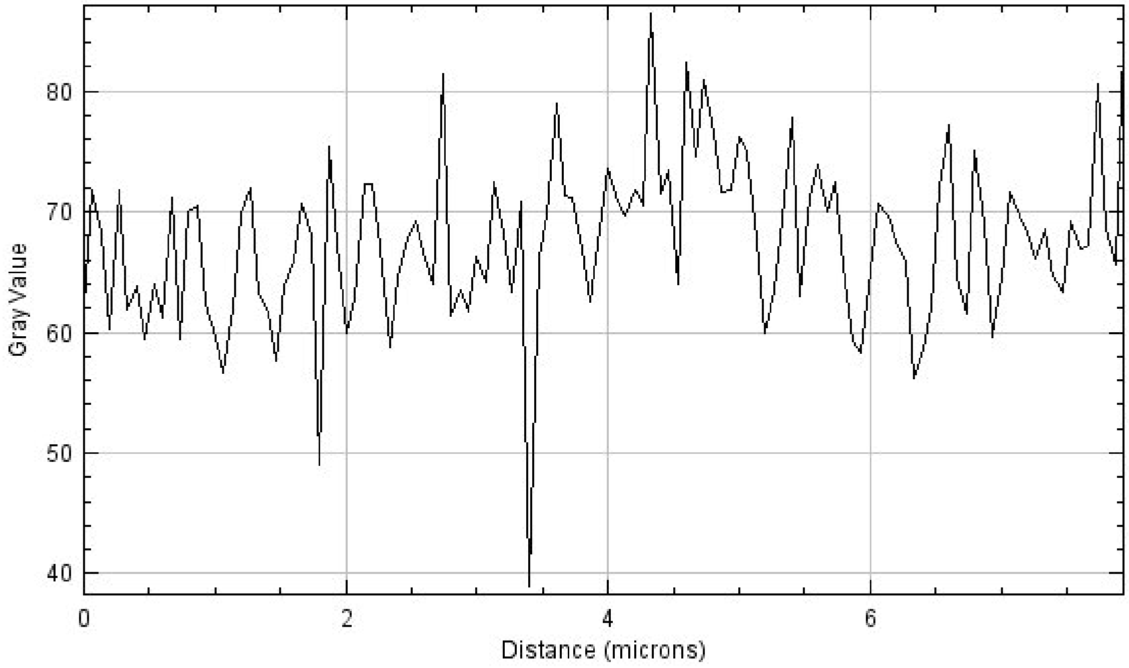

Figure S592 – Intensity profile of line scan generated for cell 4, Figure S588.

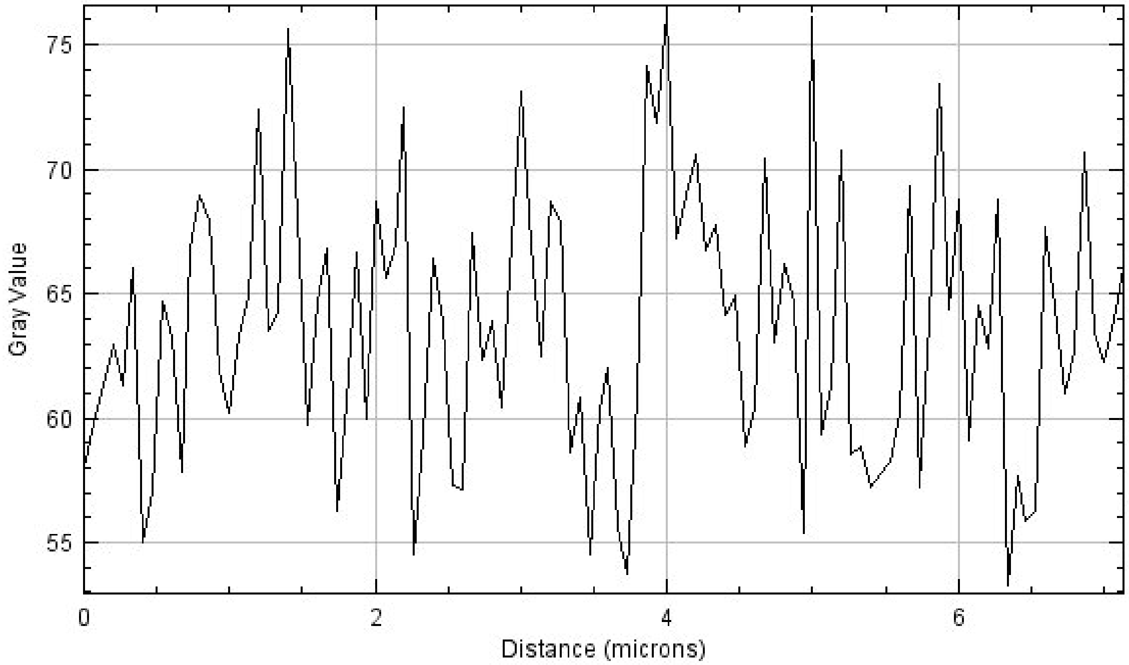

Figure S593 – Intensity profile of line scan generated for cell 5, Figure S588.

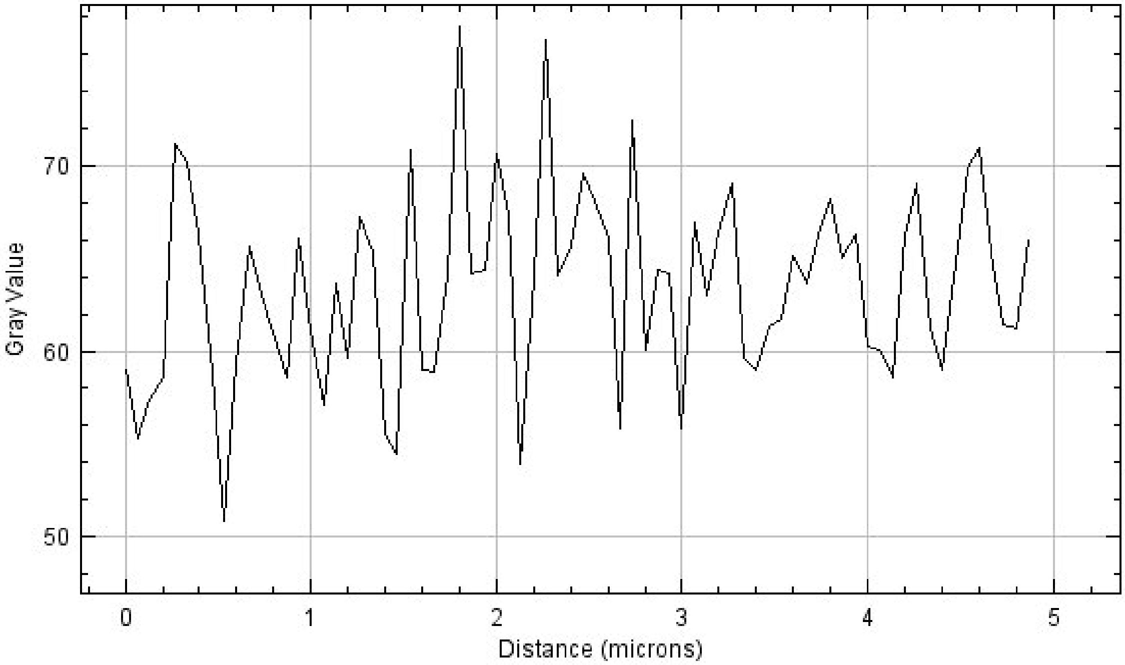

Figure S594 – Intensity profile of line scan generated for cell 6, Figure S588.

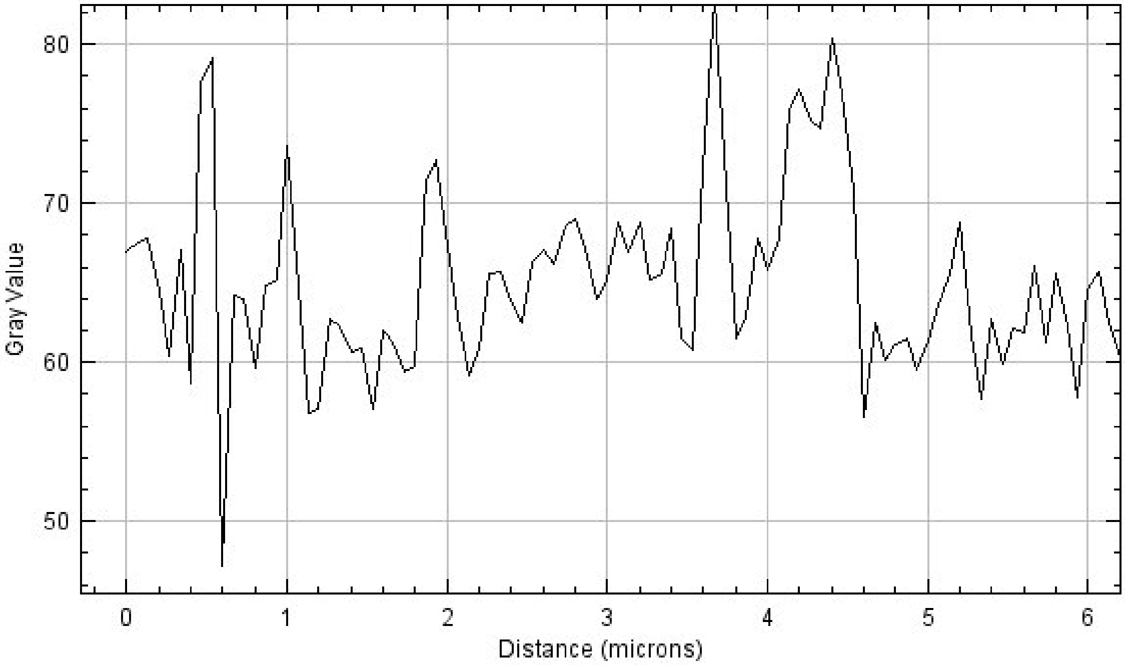

Figure S595 – Intensity profile of line scan generated for cell 7, Figure S588.

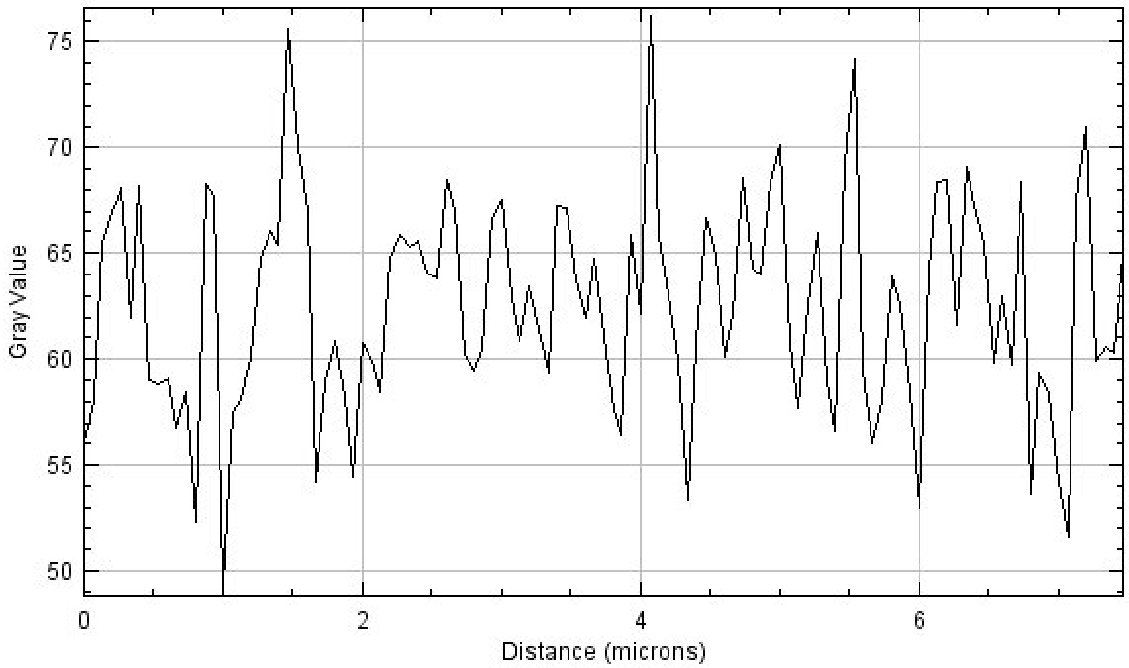

Figure S596 – Intensity profile of line scan generated for cell 8, Figure S588.

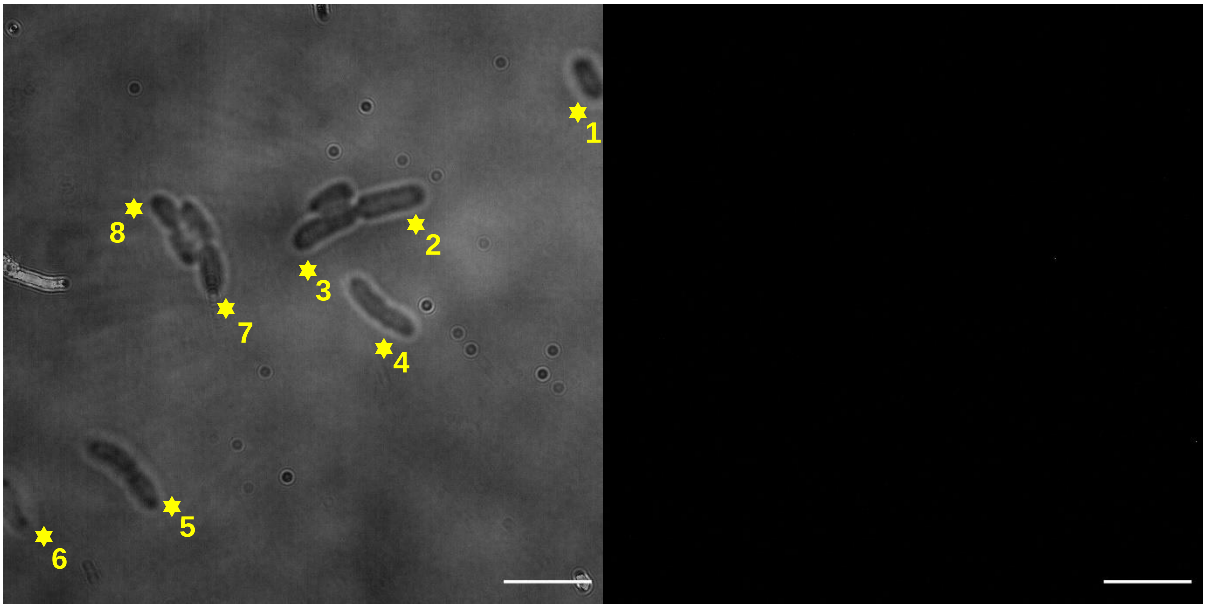

Figure S597 – *E. coli* transmitted and mCherry images used in fluorescence intensity calculations in the absence of both compound **39** and FM4-64 at T = 30 minutes. Transmitted image used to locate cells on mCherry image. Scale bar = 10 μM.

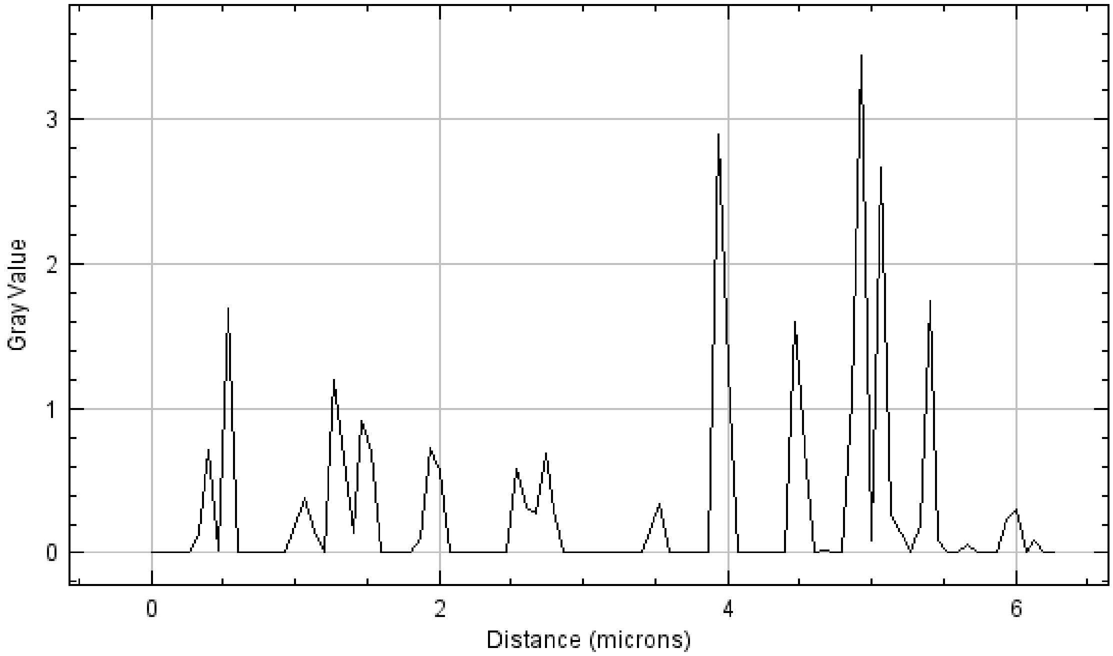

Figure S598 – Intensity profile of line scan generated for cell 1, Figure S597.

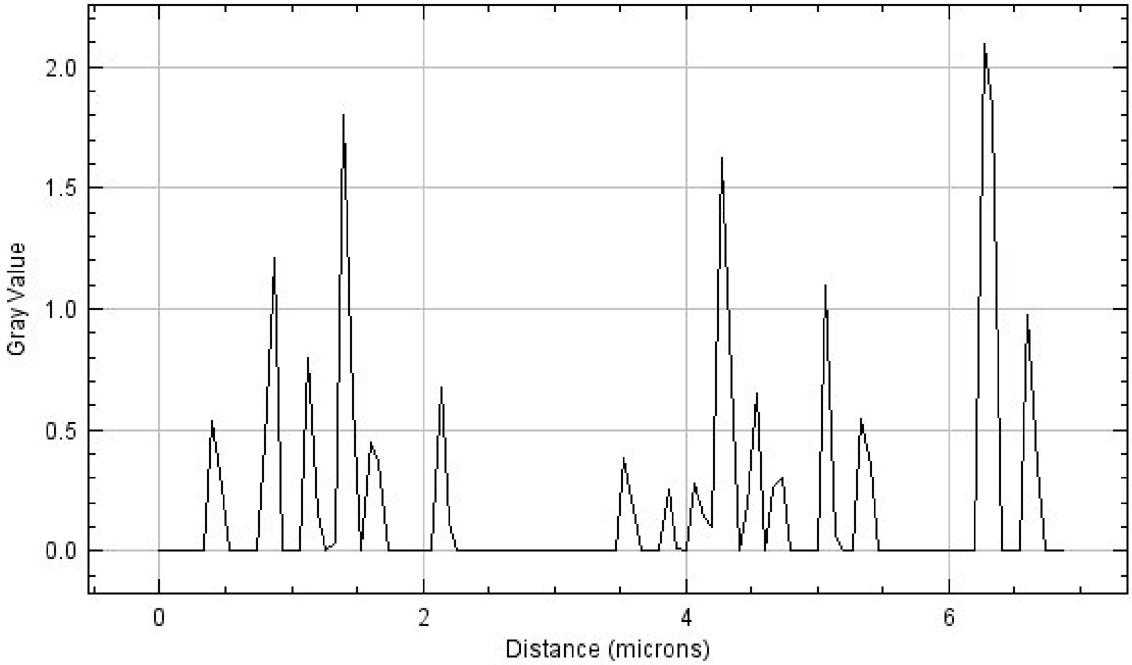

Figure S599 – Intensity profile of line scan generated for cell 2, Figure S597.

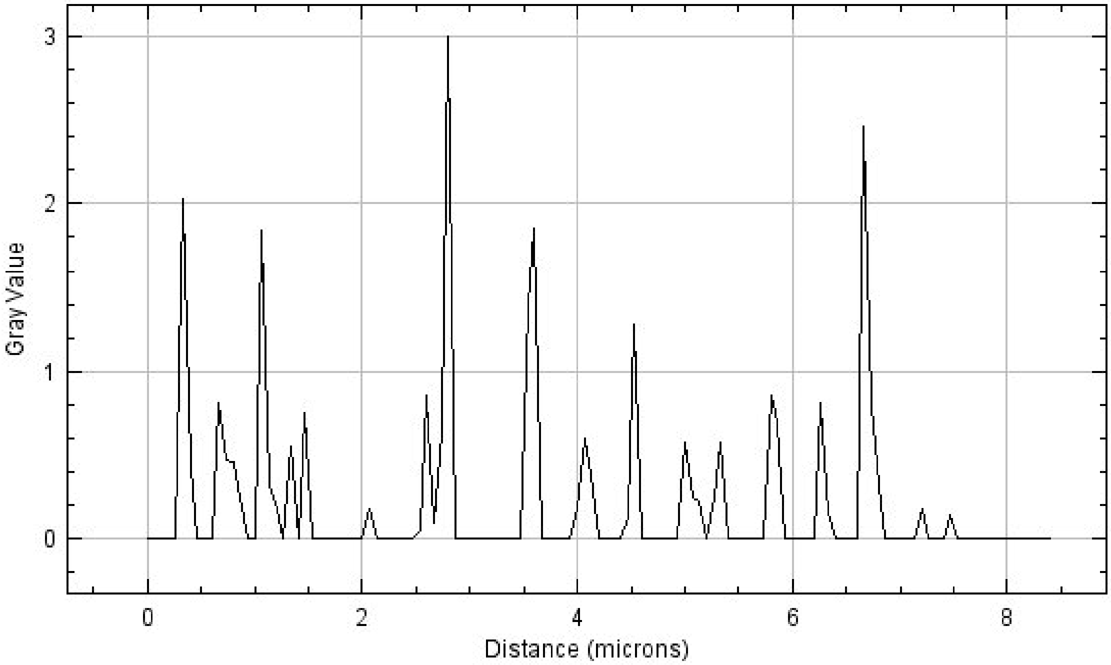

Figure S600 – Intensity profile of line scan generated for cell 3, Figure S597.

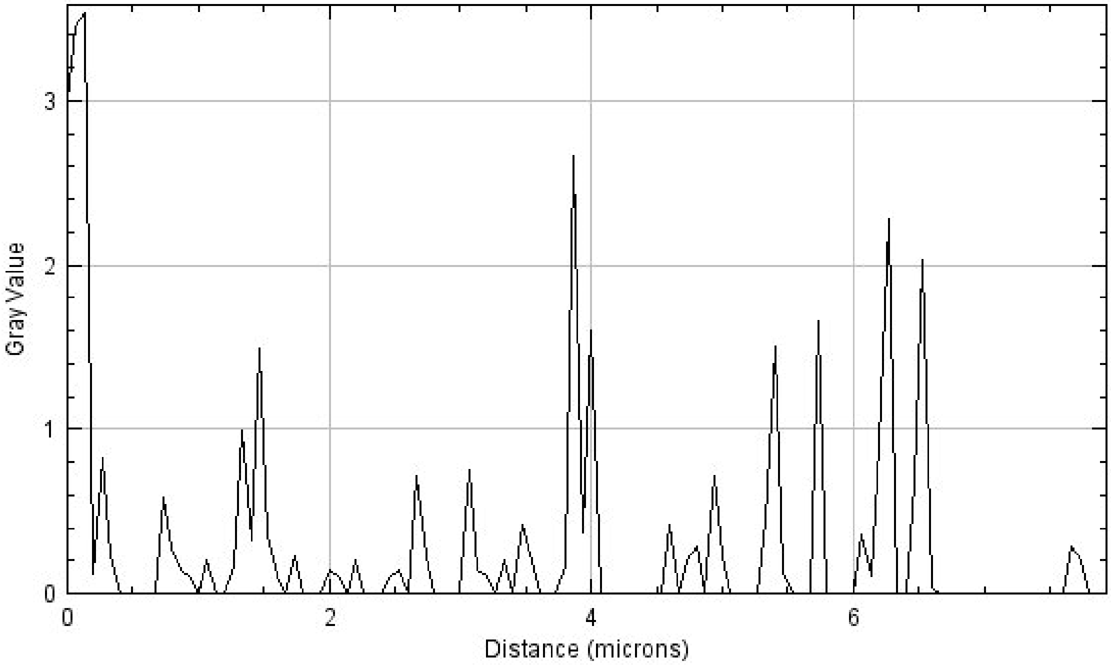

Figure S601 – Intensity profile of line scan generated for cell 4, Figure S597.

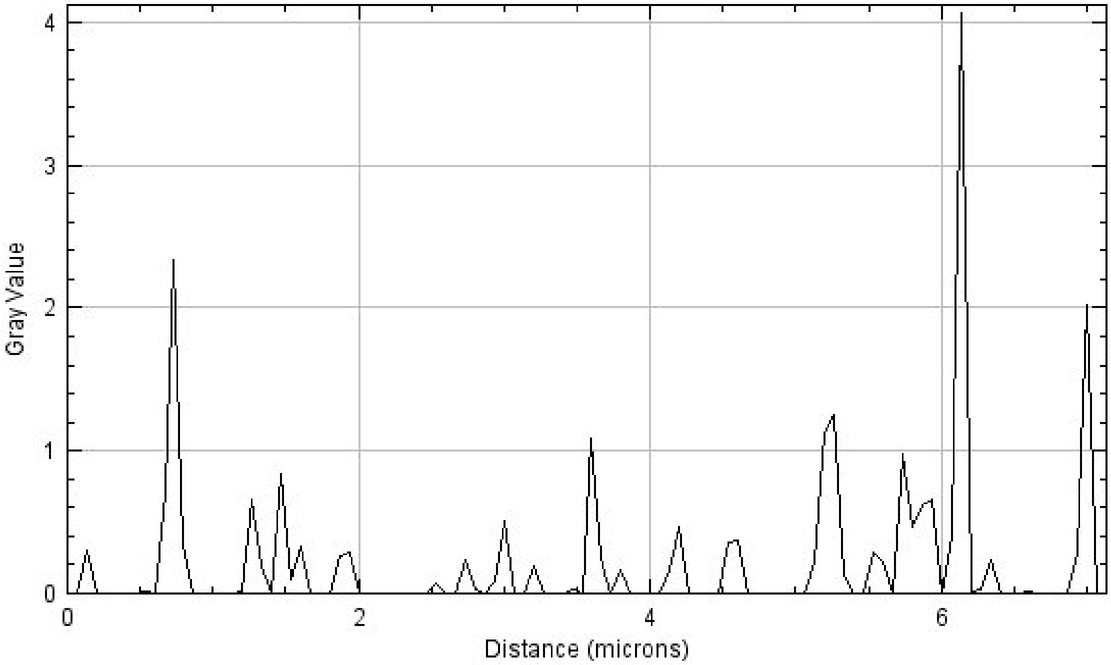

Figure S602 – Intensity profile of line scan generated for cell 5, Figure S597.

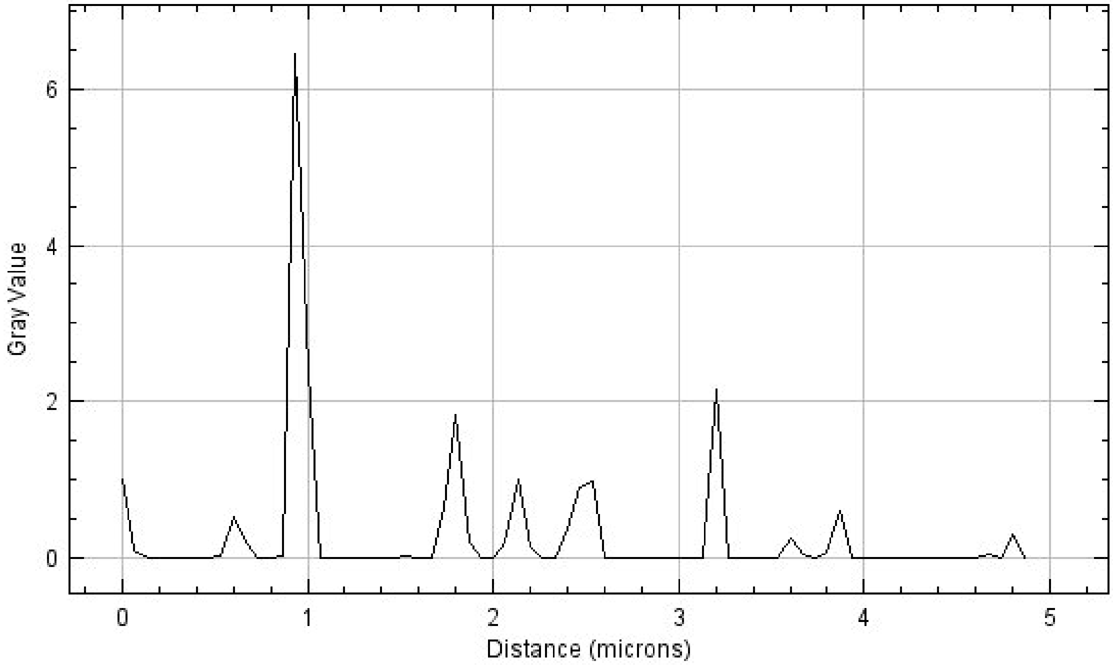

Figure S603 – Intensity profile of line scan generated for cell 6, Figure S597.

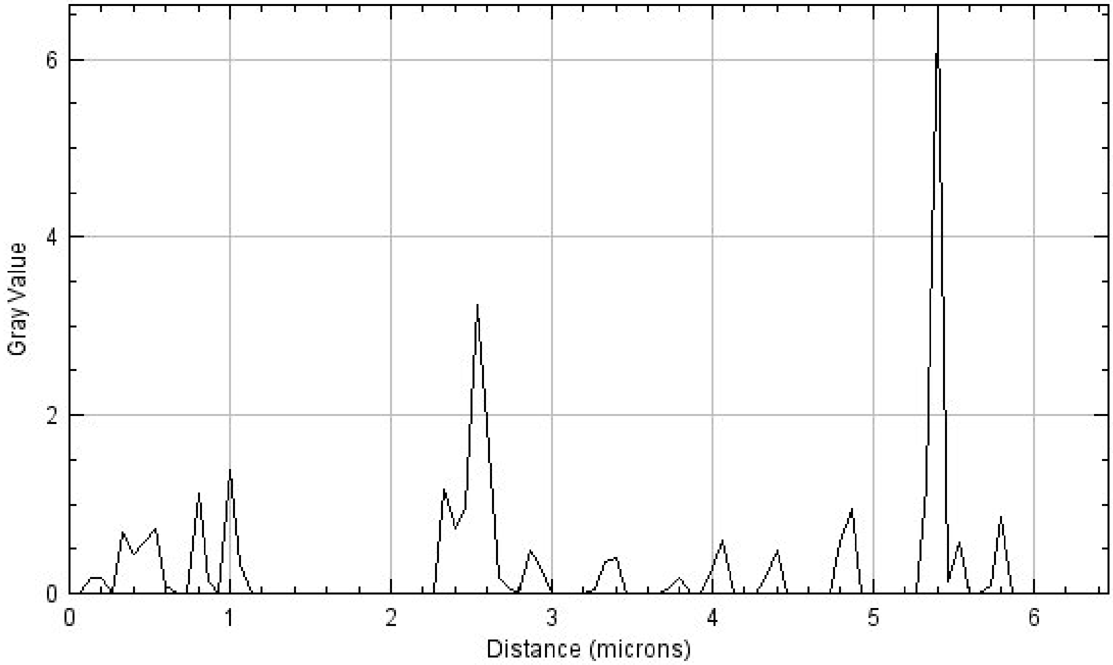

Figure S604 – Intensity profile of line scan generated for cell 7, Figure S597.

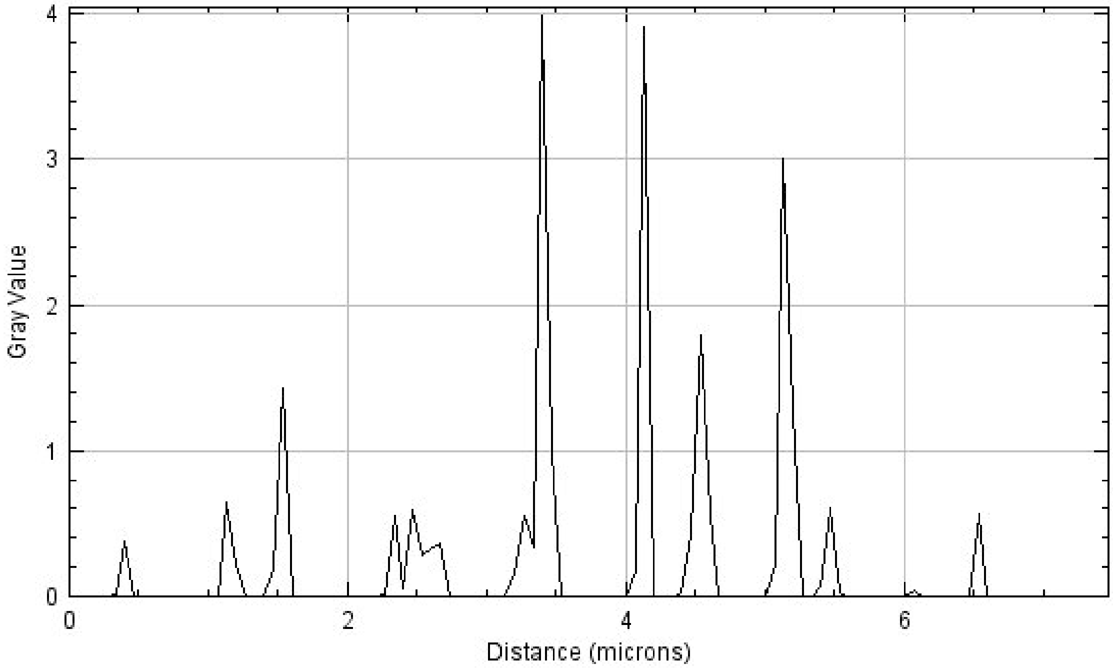

Figure S605 – Intensity profile of line scan generated for cell 8, Figure S597.

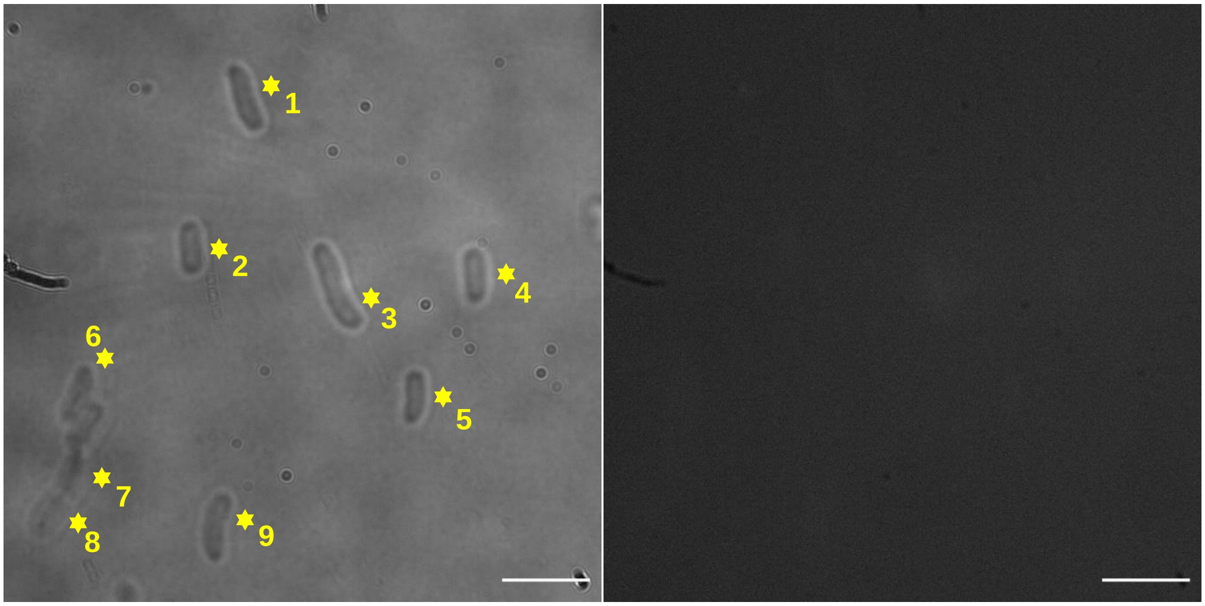

Figure S606 – *E. coli* transmitted and DAPI images used in fluorescence intensity calculations in the absence of both compound **39** and FM4-64 at T = 30 minutes. Transmitted image used to locate cells on DAPI image. Scale bar = 10 μM.

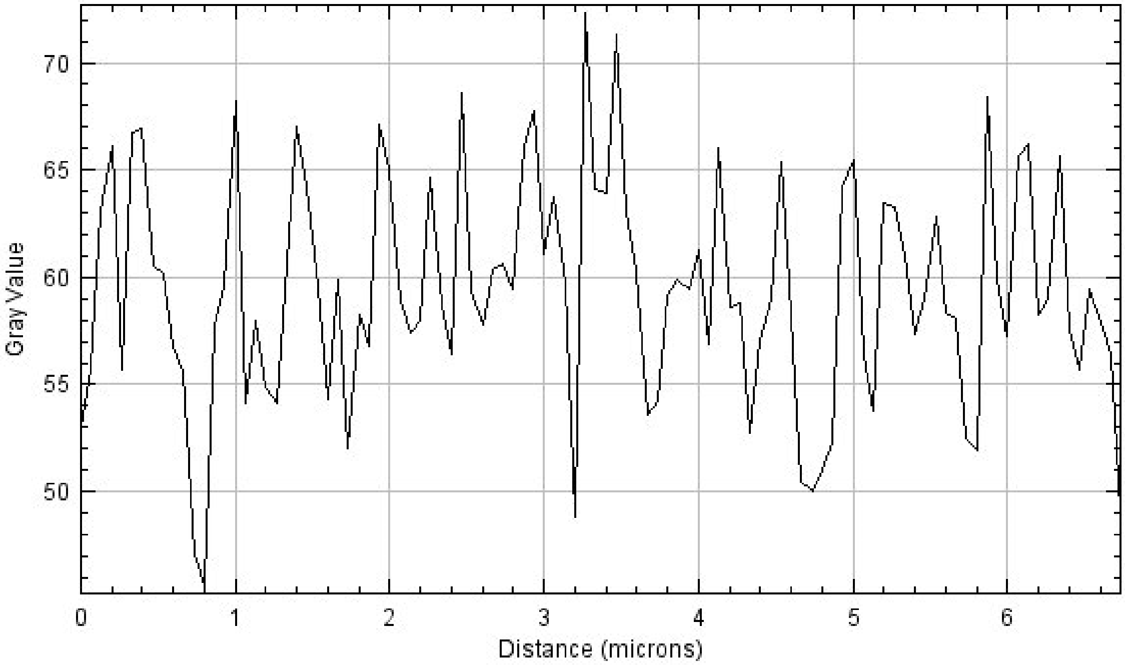

Figure S607 – Intensity profile of line scan generated for cell 1, Figure S606.

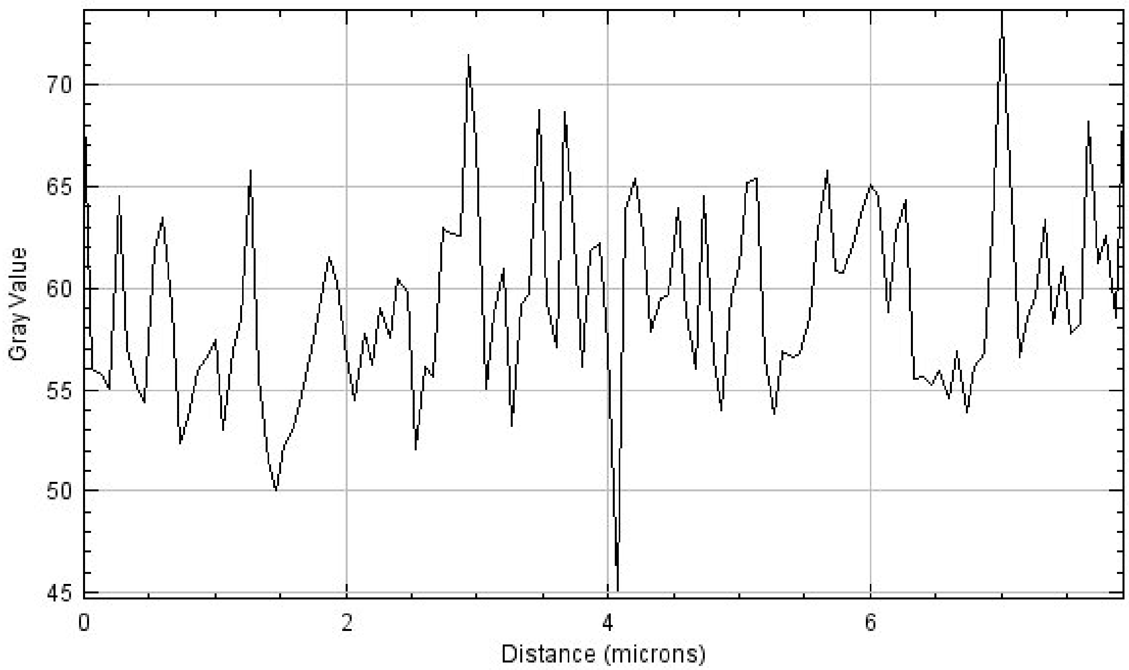

Figure S608 – Intensity profile of line scan generated for cell 2, Figure S606.

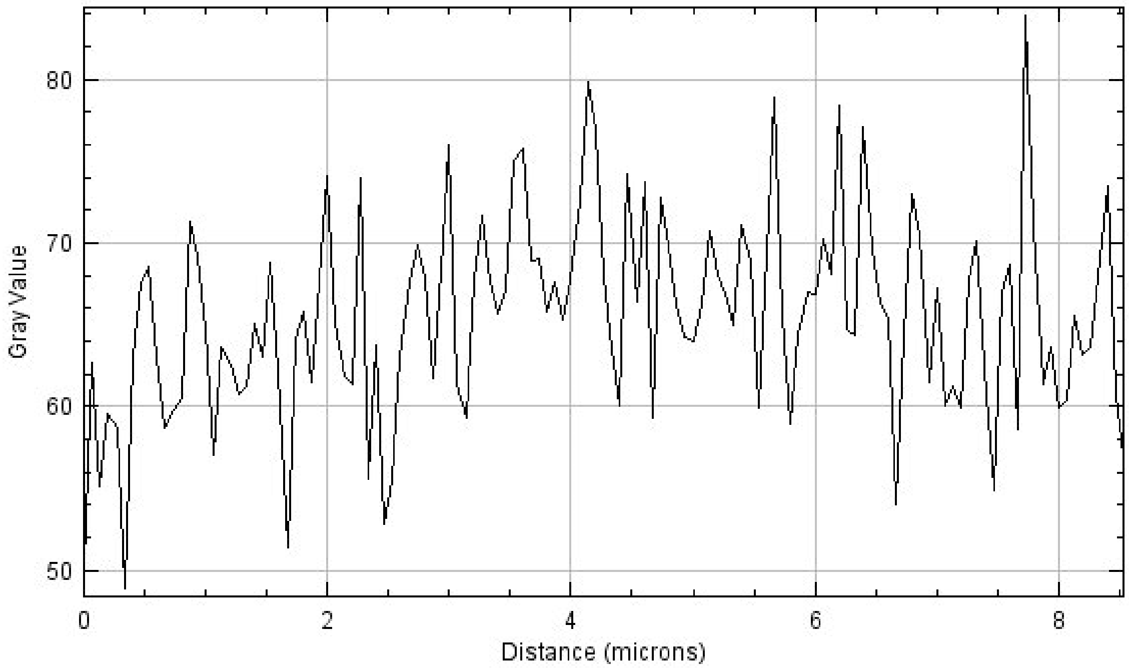

Figure S609 – Intensity profile of line scan generated for cell 3, Figure S606.

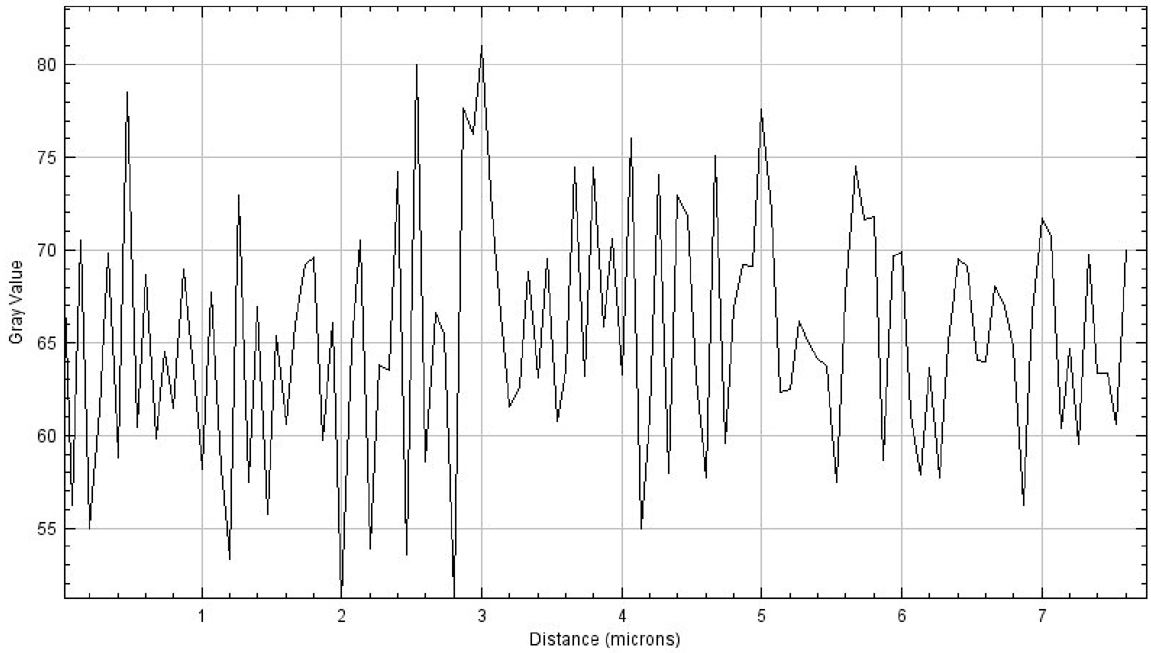

Figure S610 – Intensity profile of line scan generated for cell 4, Figure S606.

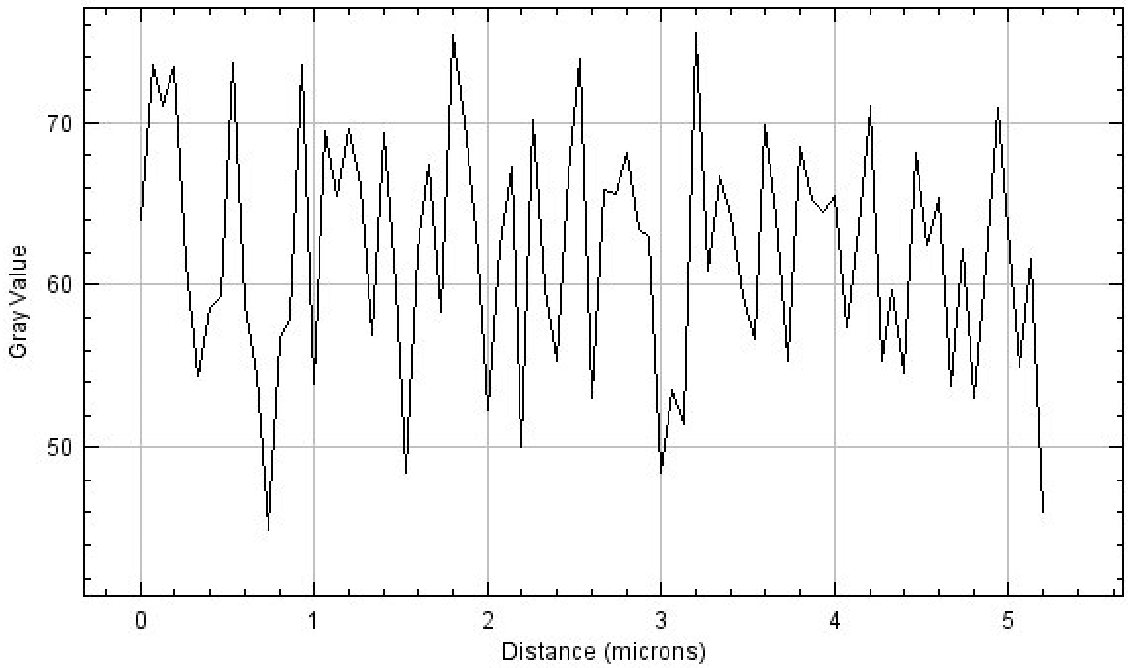

Figure S611 – Intensity profile of line scan generated for cell 5, Figure S606.

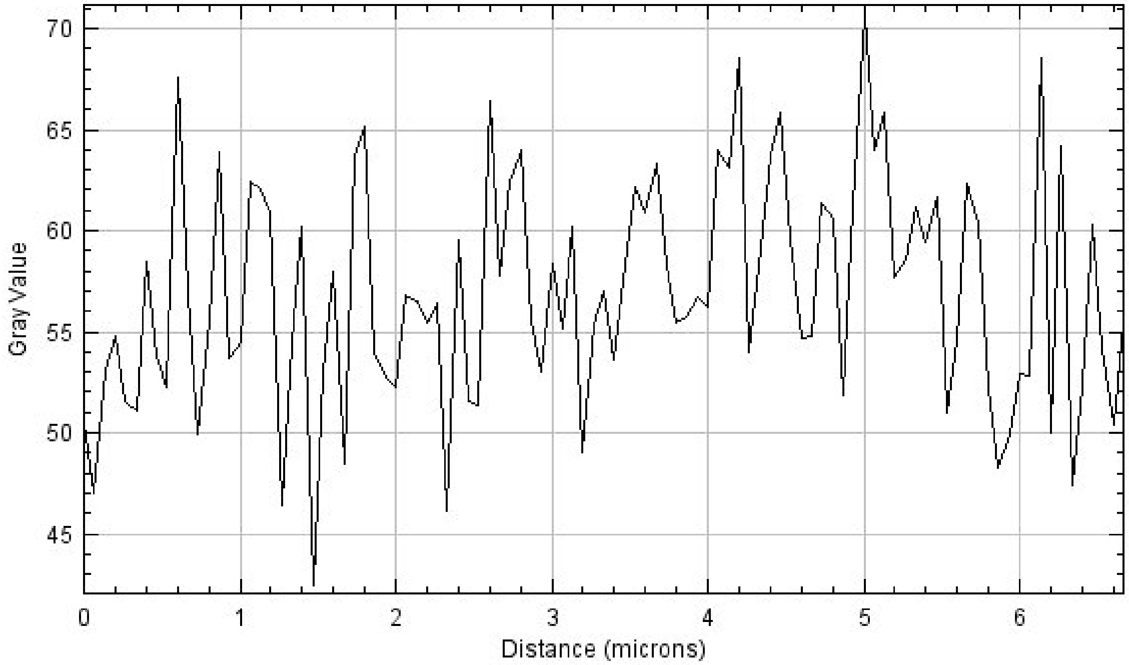

Figure S612 – Intensity profile of line scan generated for cell 6, Figure S606.

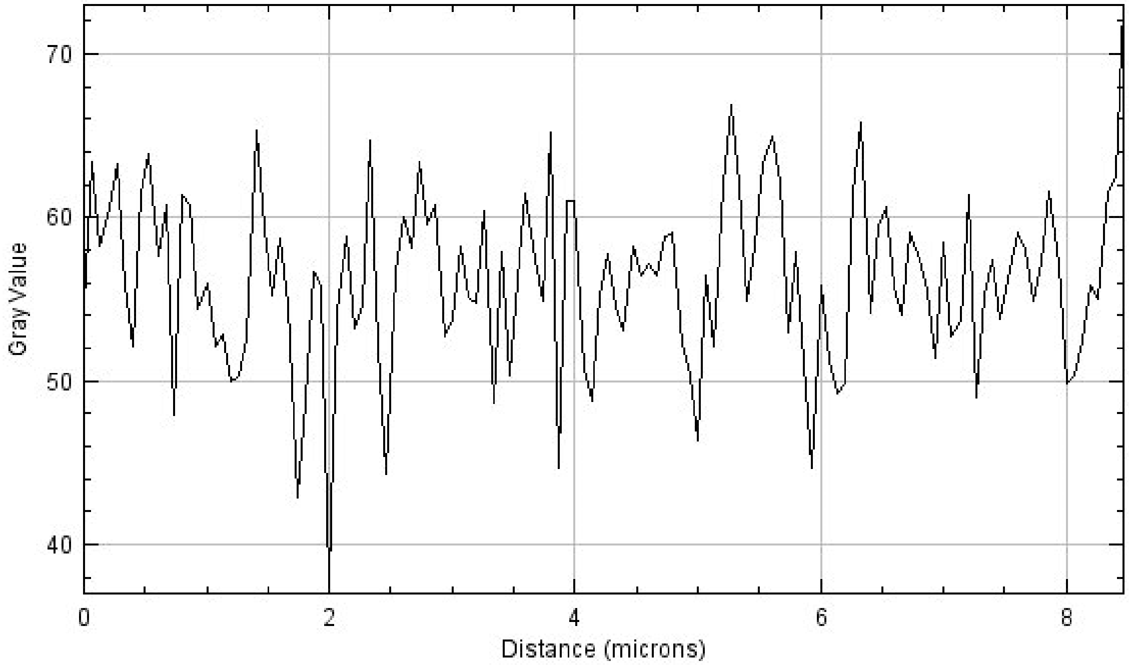

Figure S613 – Intensity profile of line scan generated for cell 7, Figure S606.

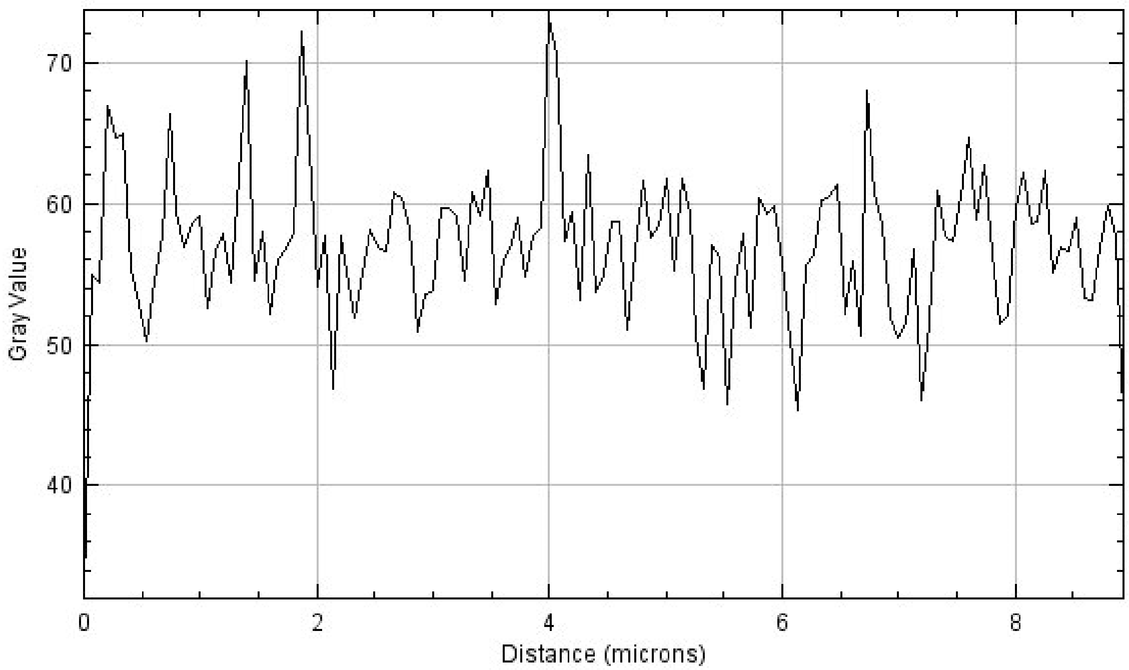

Figure S614 – Intensity profile of line scan generated for cell 8, Figure S606.

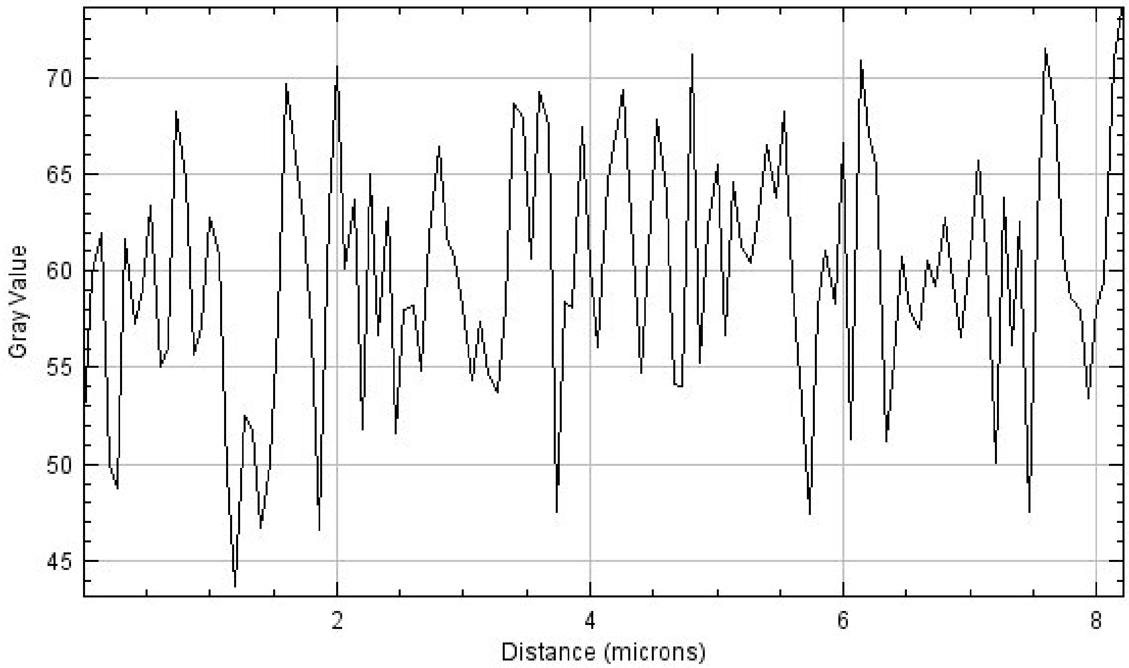

Figure S615 – Intensity profile of line scan generated for cell 9, Figure S606.

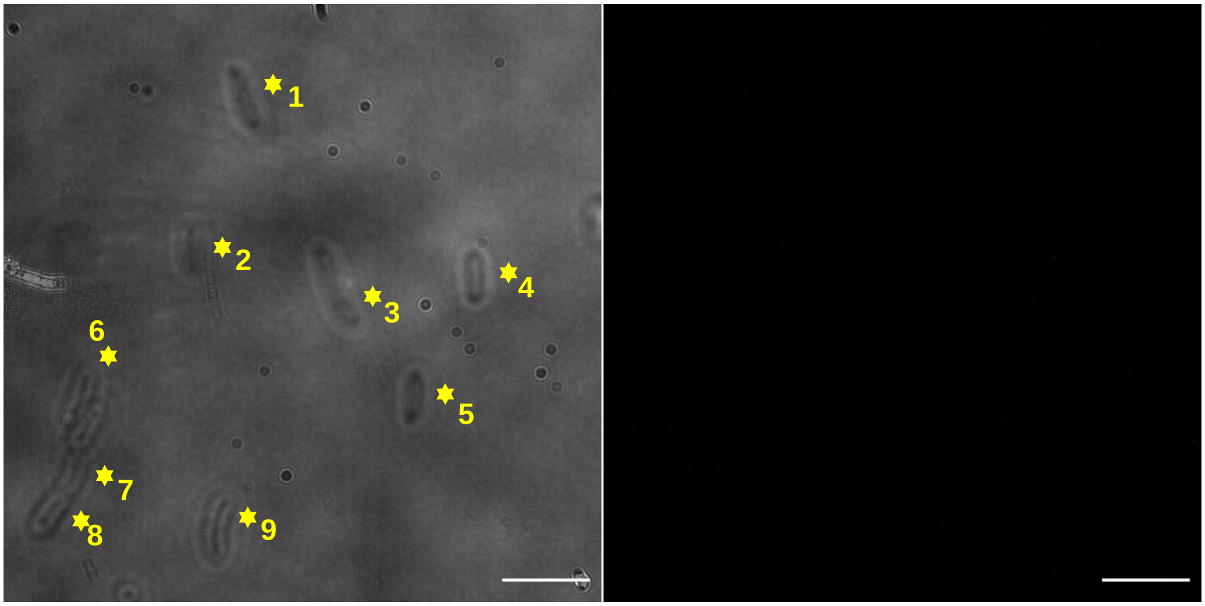

Figure S616 – *E. coli* transmitted and mCherry images used in fluorescence intensity calculations in the absence of both compound **39** and FM4-64 at T = 30 minutes. Transmitted image used to locate cells on mCherry image. Scale bar = 10 µM.

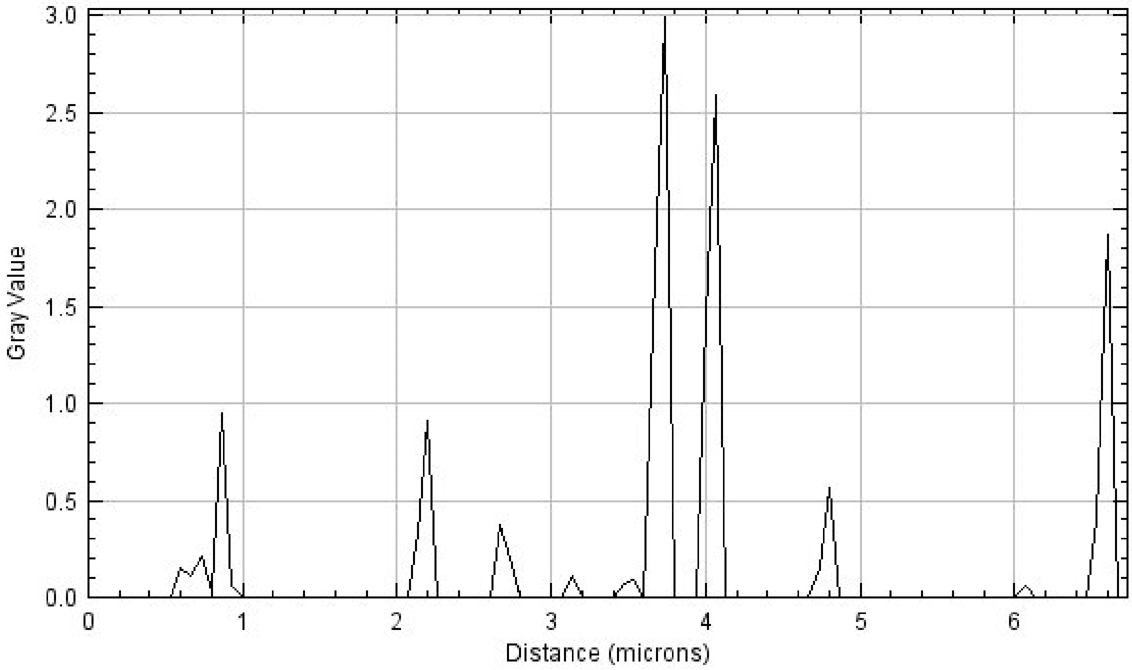

Figure S617 – Intensity profile of line scan generated for cell 1, Figure S616.

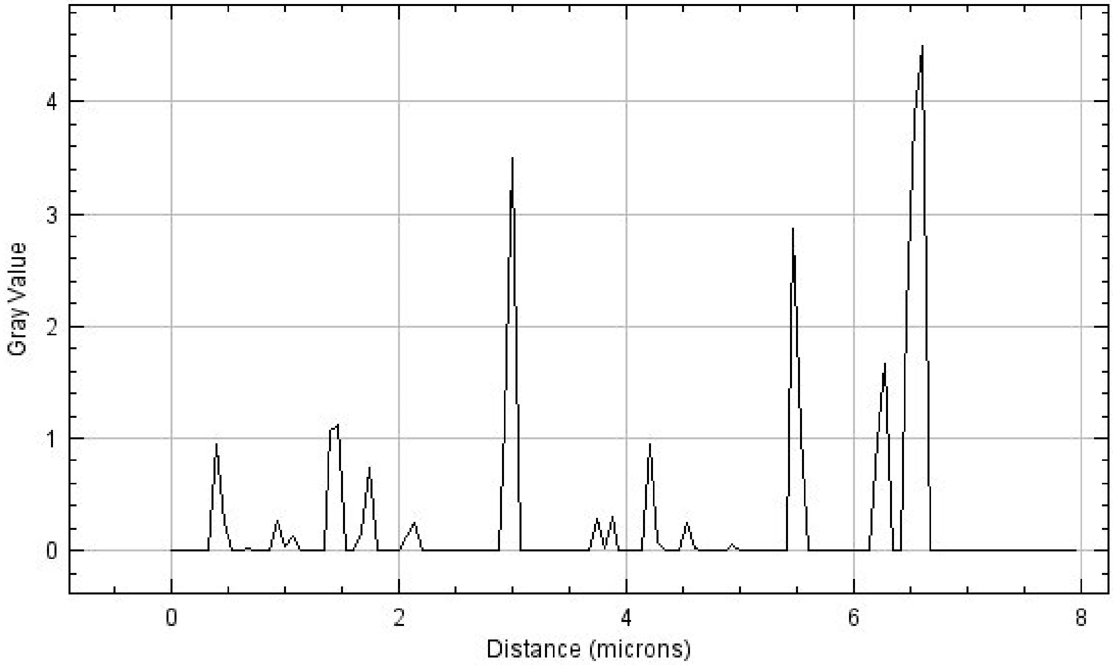

Figure S618 – Intensity profile of line scan generated for cell 2, Figure S616.

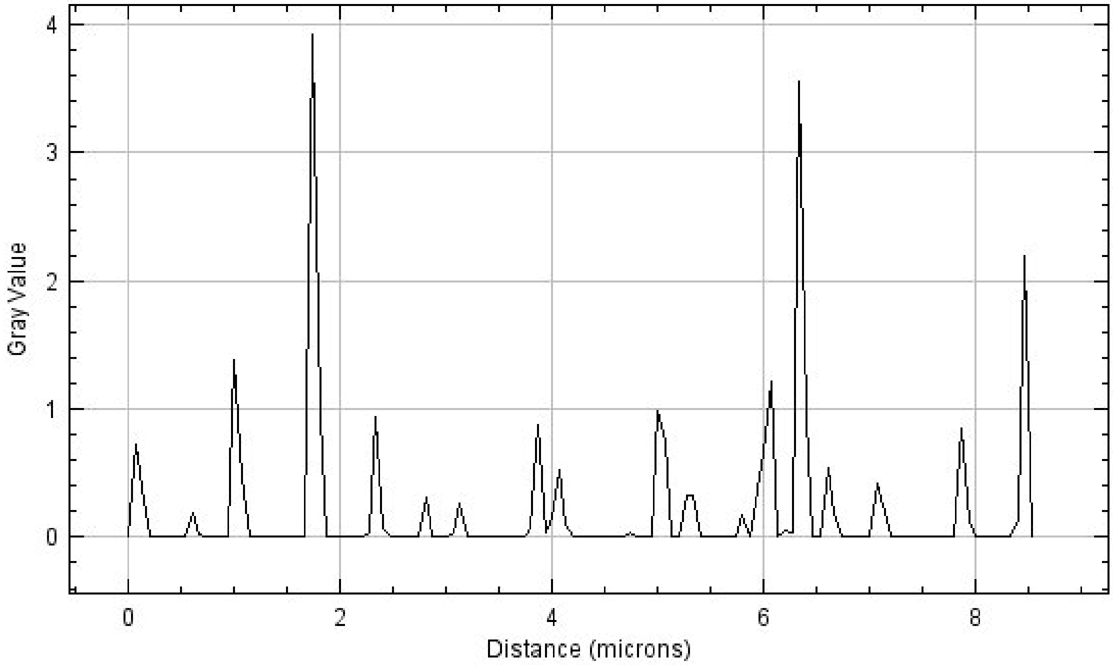

Figure S619 – Intensity profile of line scan generated for cell 3, Figure S616.

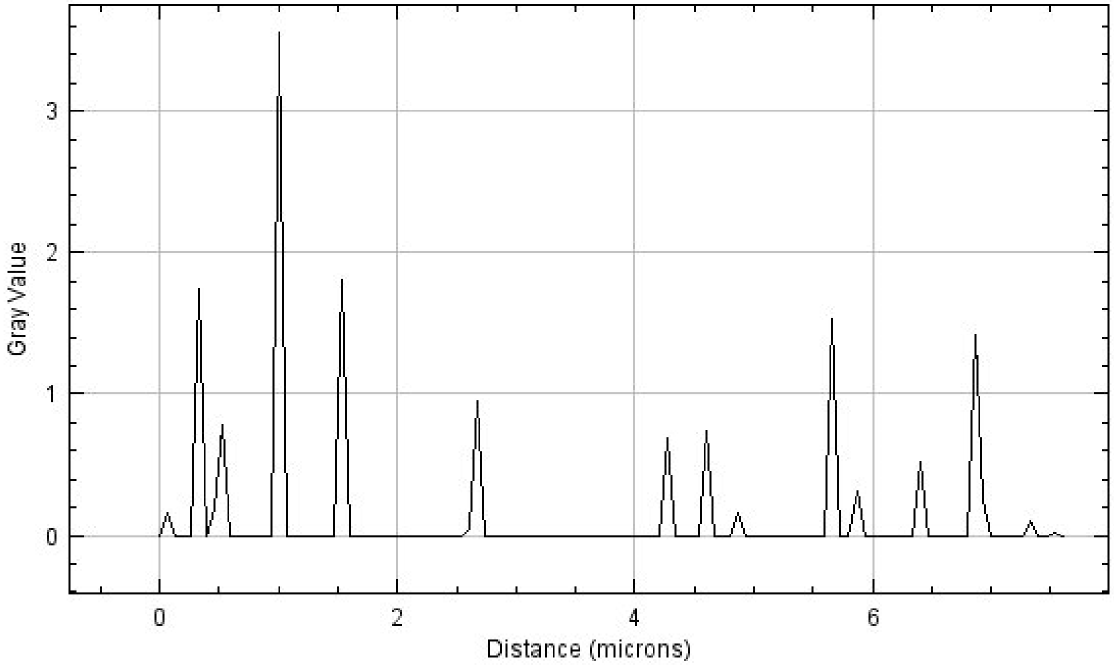

Figure S620 – Intensity profile of line scan generated for cell 4, Figure S616.

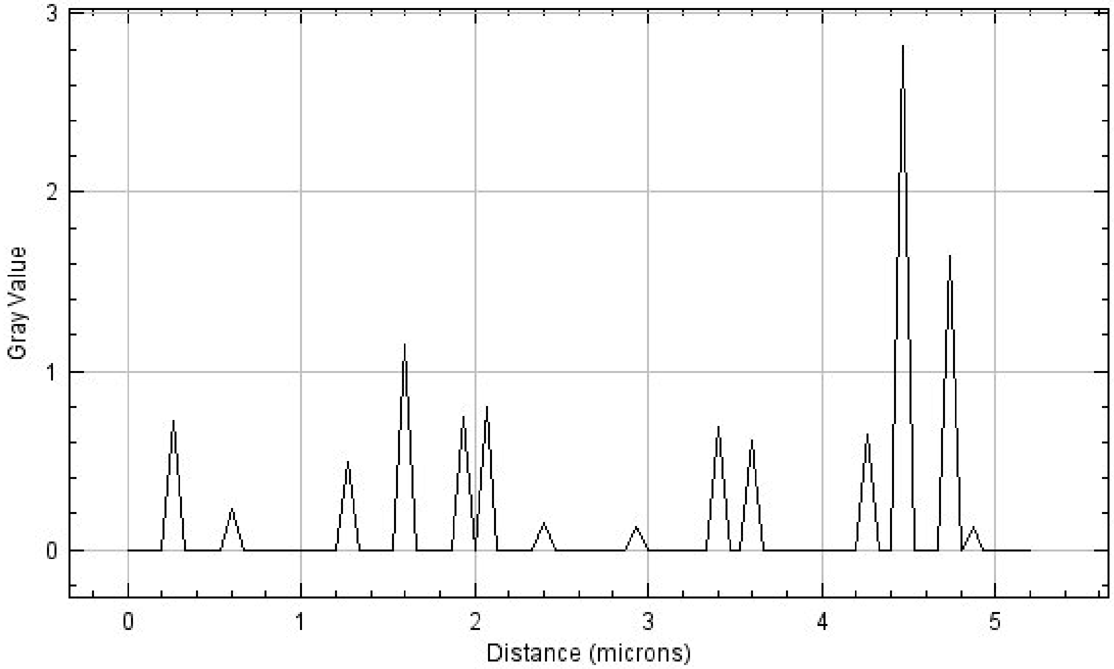

Figure S621 – Intensity profile of line scan generated for cell 5, Figure S616.

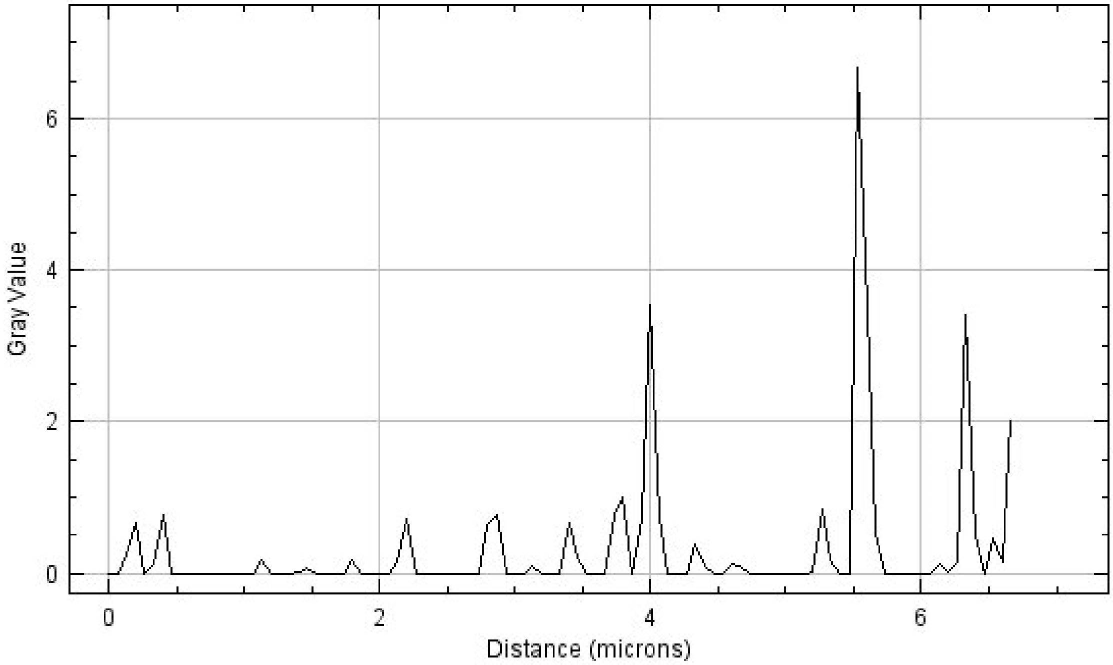

Figure S622 – Intensity profile of line scan generated for cell 6, Figure S616.

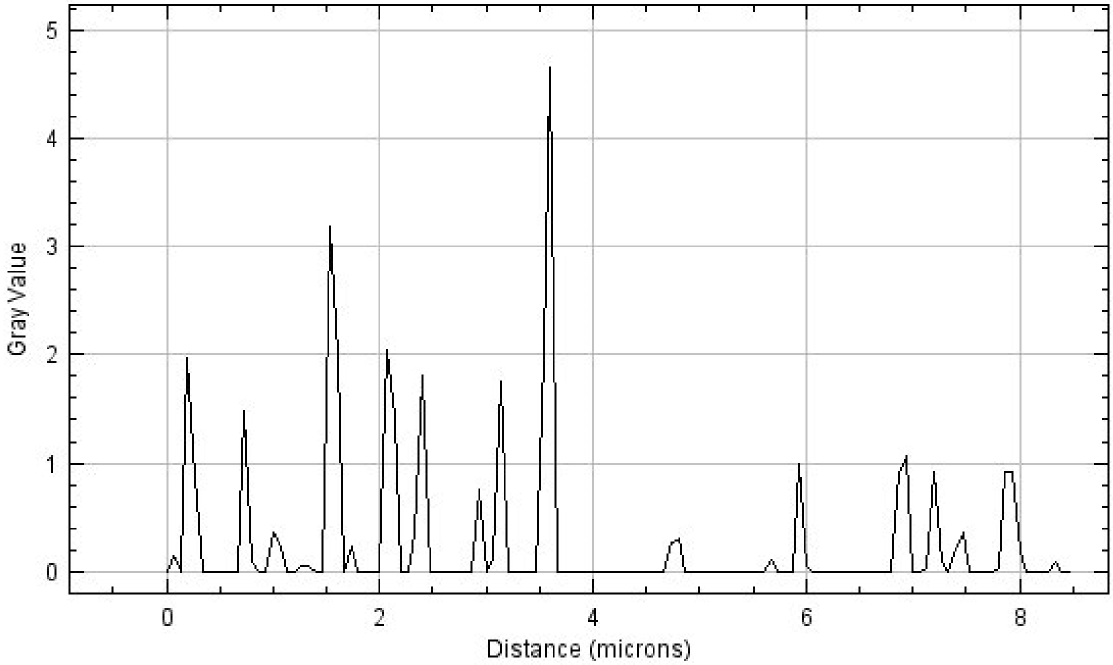

Figure S623 – Intensity profile of line scan generated for cell 7, Figure S616.

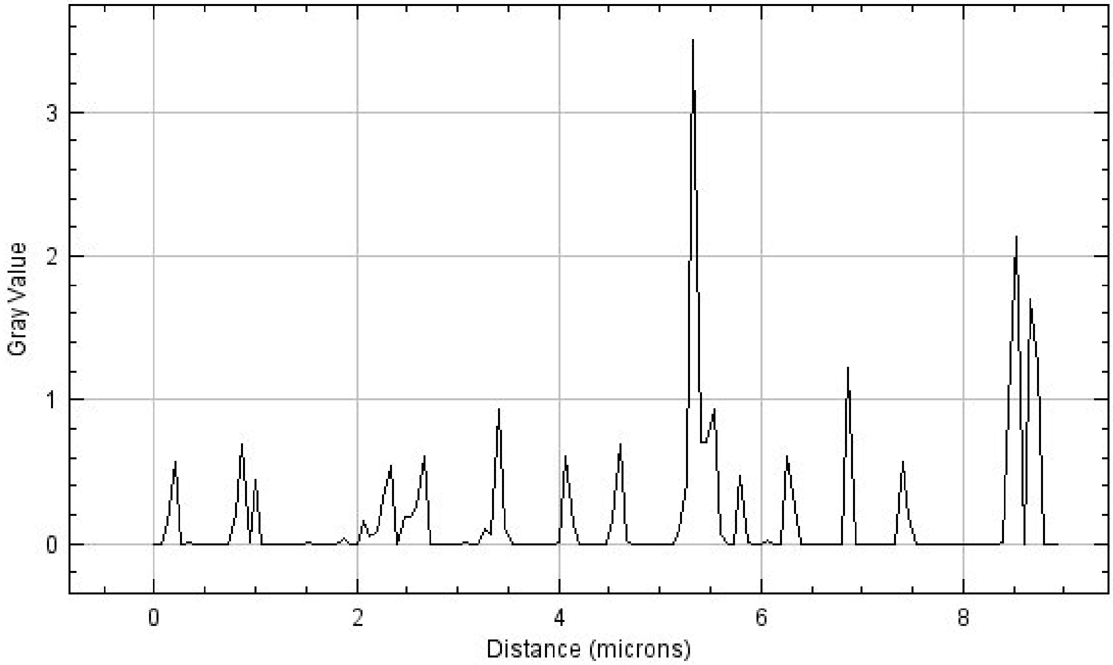

Figure S624 – Intensity profile of line scan generated for cell 8, Figure S616.

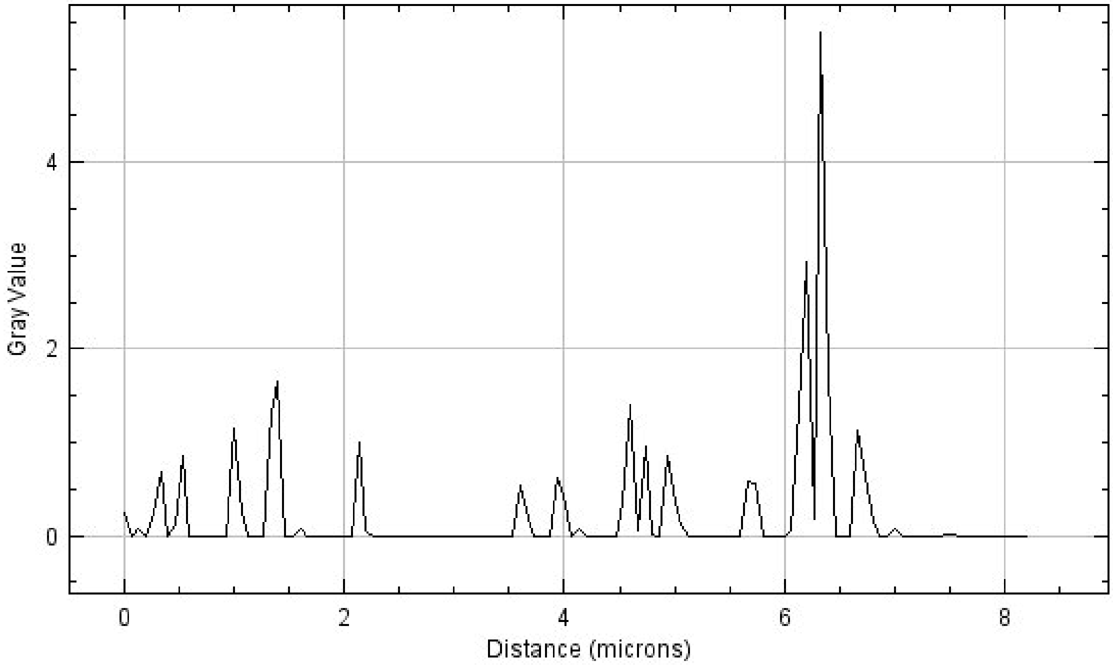

Figure S625 – Intensity profile of line scan generated for cell 9, Figure S616.

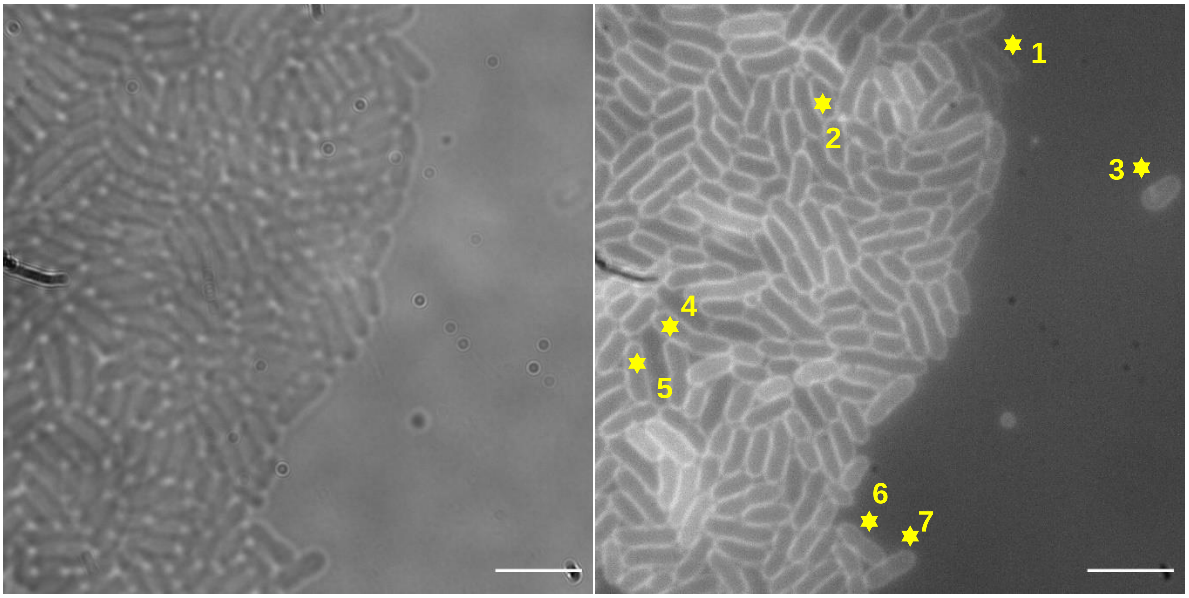

Figure S626 – *E. coli* transmitted and DAPI images used in fluorescence intensity calculations in the presence of compound **39** at T = 30 minutes. Scale bar = 10 μM.

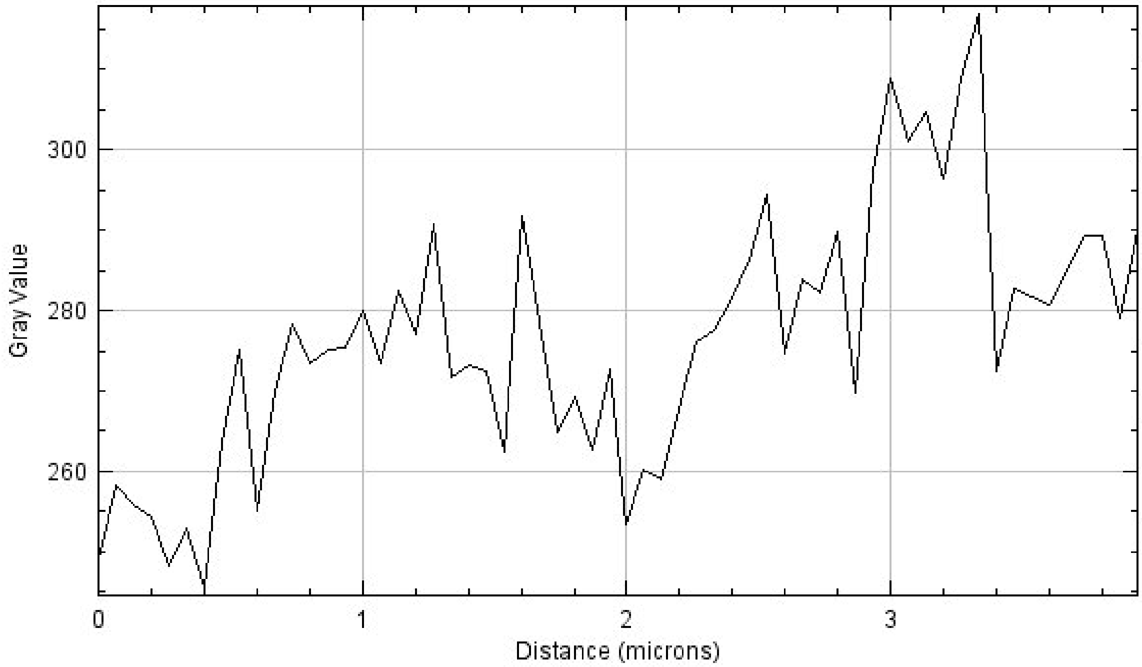

Figure S627 – Intensity profile of line scan generated for cell 1, Figure S626.

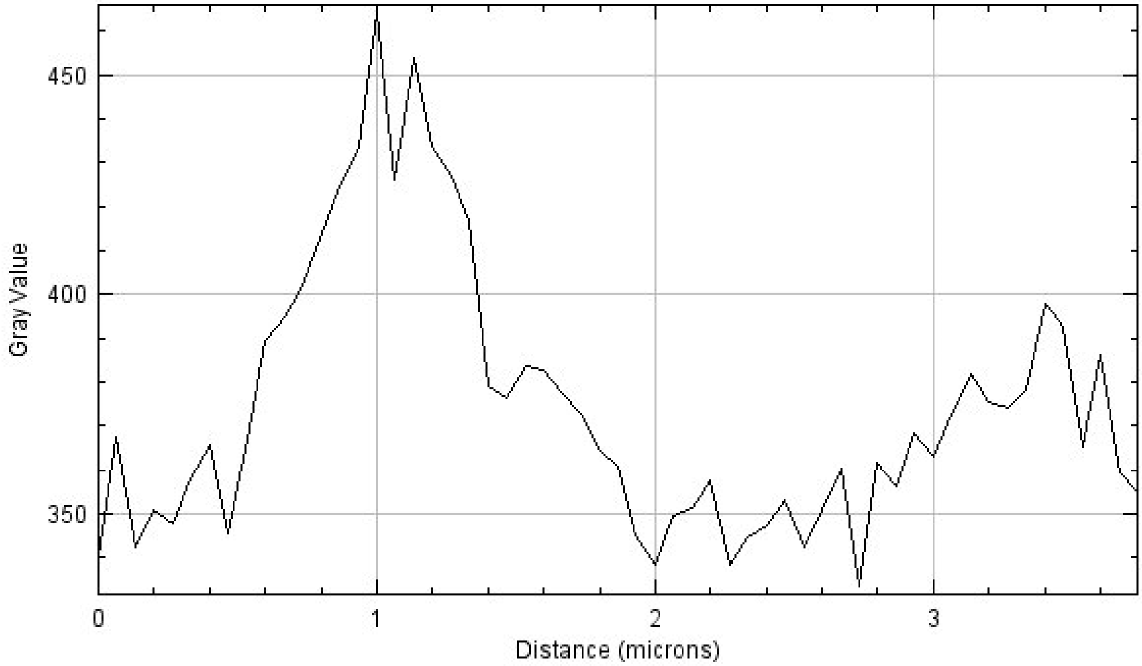

Figure S628 – Intensity profile of line scan generated for cell 2, Figure S626.

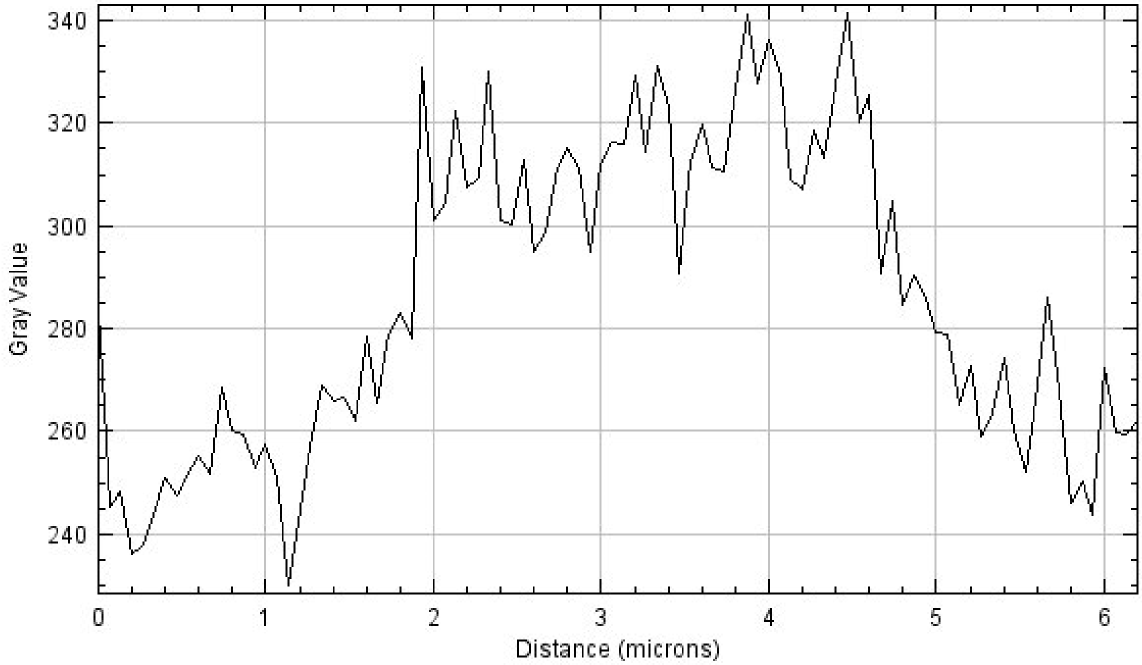

Figure S629 – Intensity profile of line scan generated for cell 3, Figure S626.

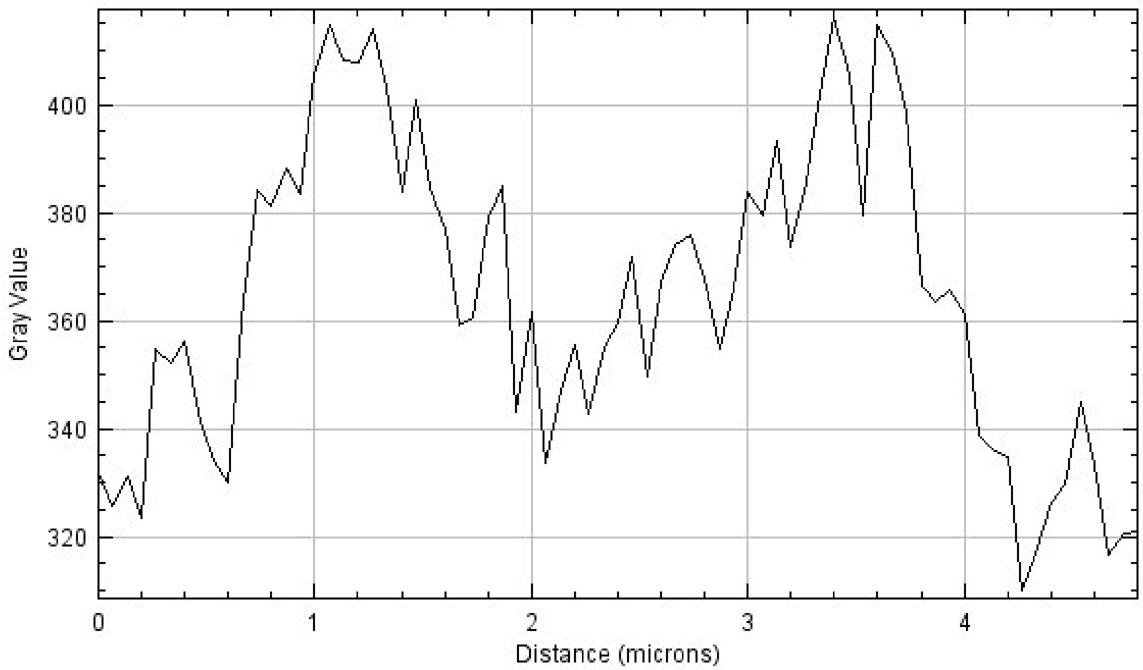

Figure S630 – Intensity profile of line scan generated for cell 4, Figure S626.

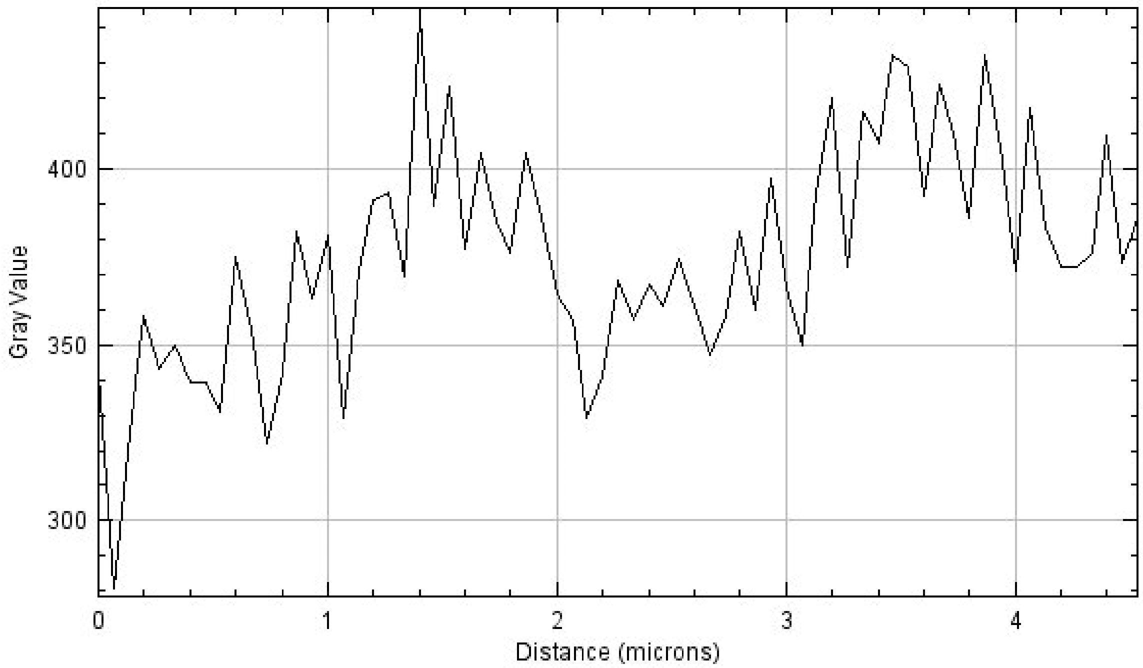

Figure S631 – Intensity profile of line scan generated for cell 5, Figure S626.

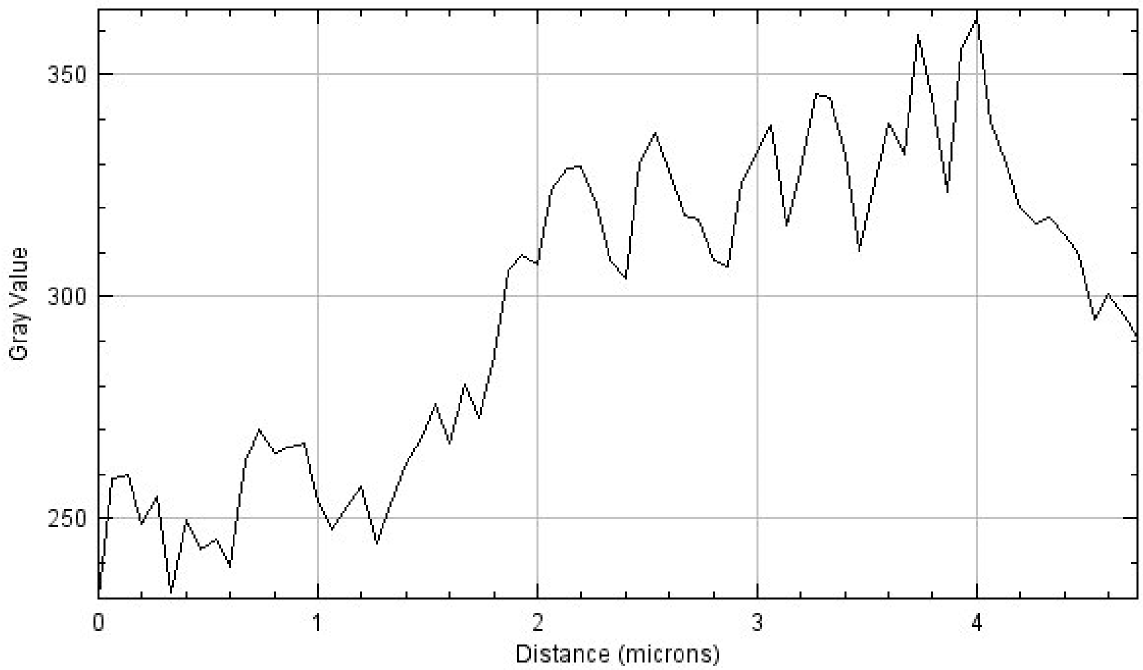

Figure S632 – Intensity profile of line scan generated for cell 6, Figure S626.

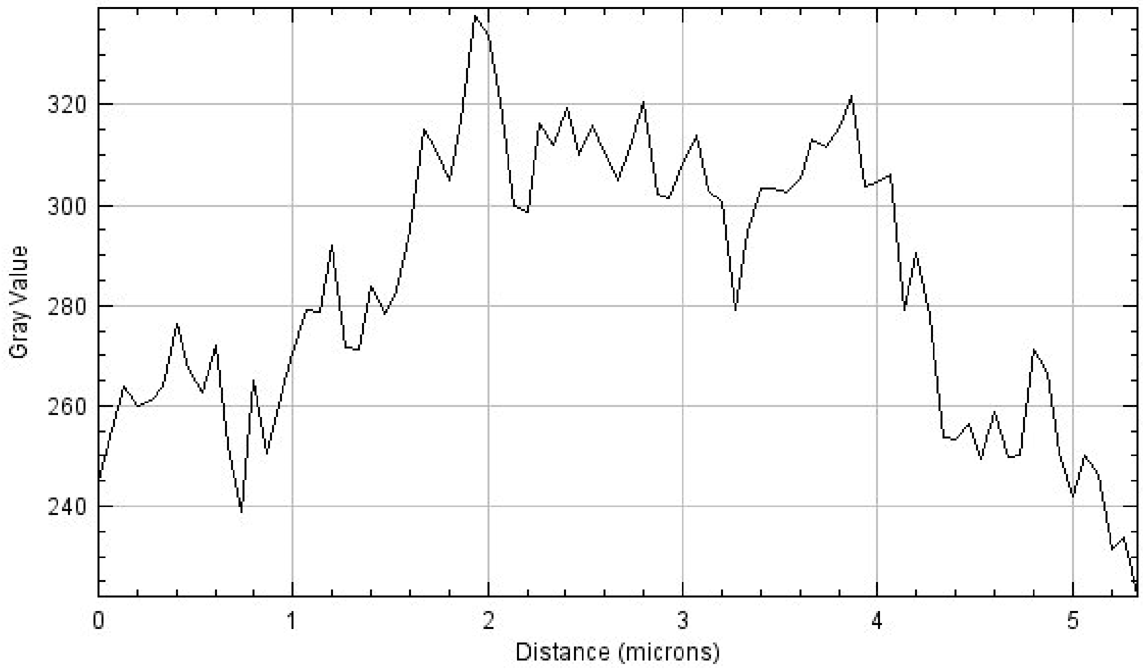

Figure S633 – Intensity profile of line scan generated for cell 7, Figure S626.

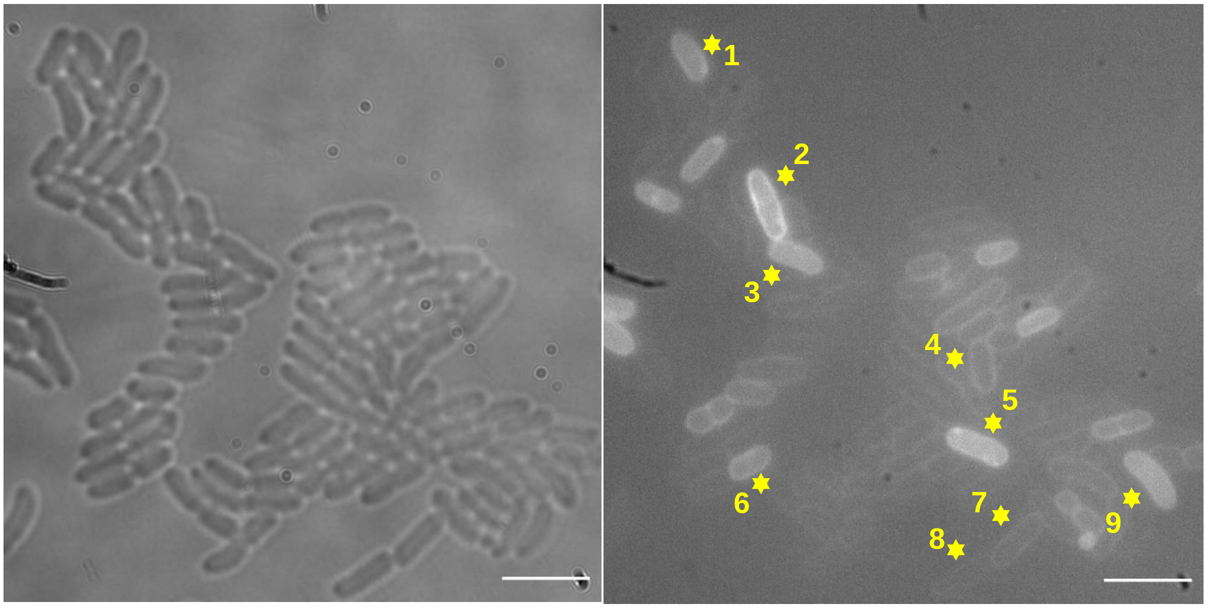

Figure S634 – *E. coli* transmitted and DAPI images used in fluorescence intensity calculations in the presence of compound **39** at T = 30 minutes. Scale bar = 10 µM.

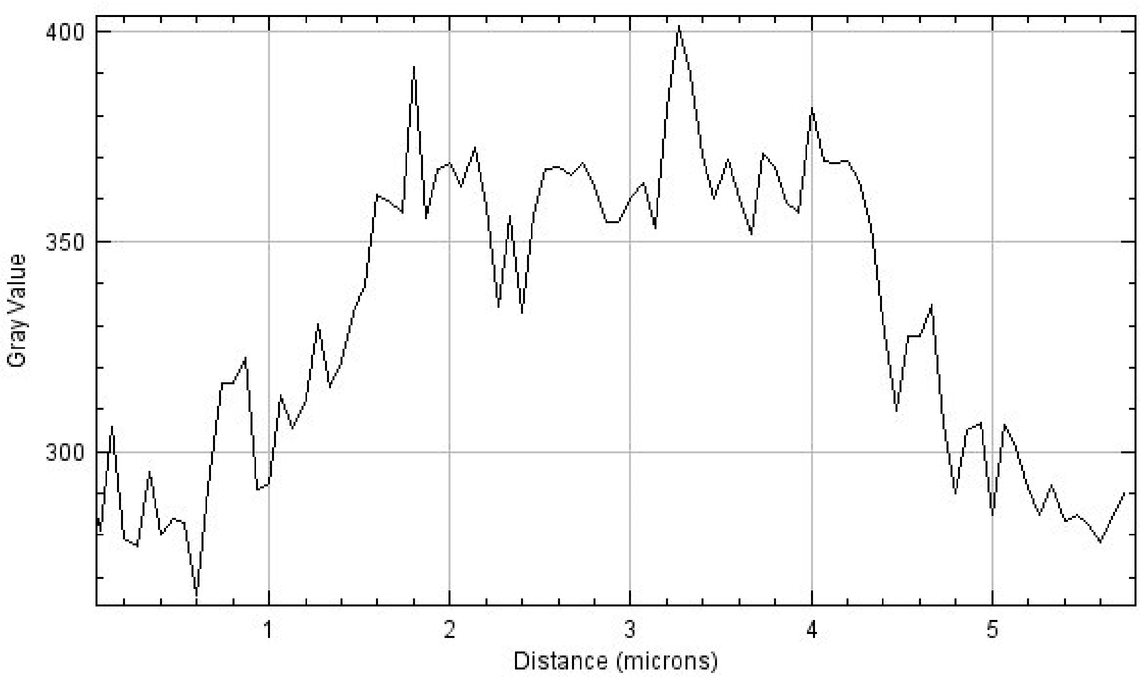

Figure S635 – Intensity profile of line scan generated for cell 1, Figure S634.

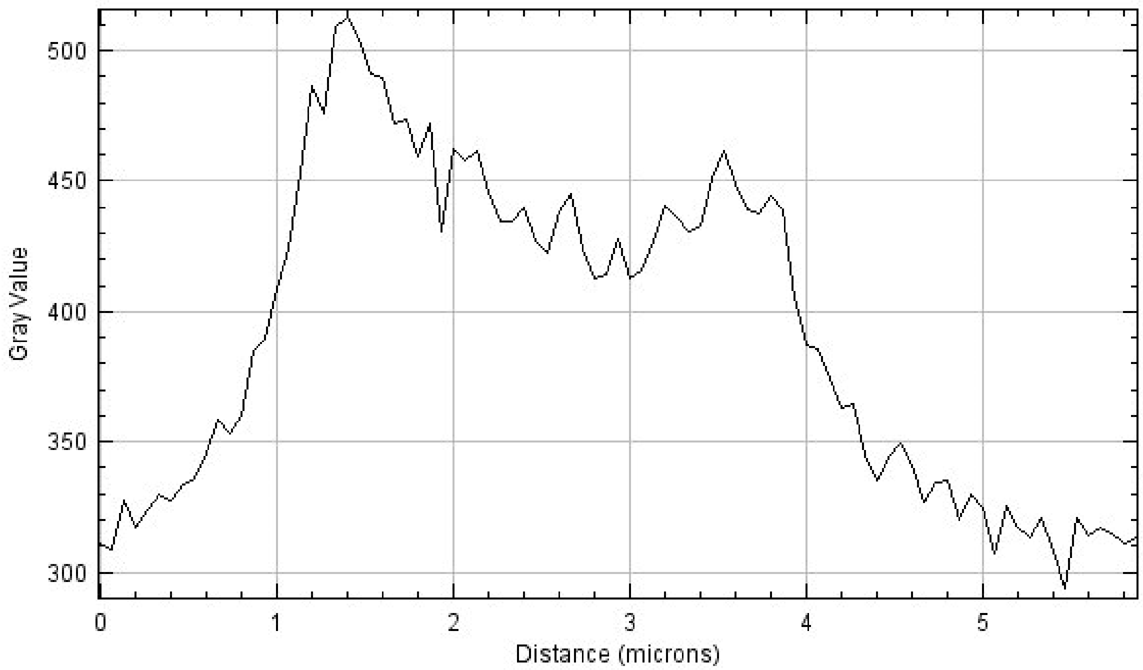

Figure S636 – Intensity profile of line scan generated for cell 2, Figure S634.

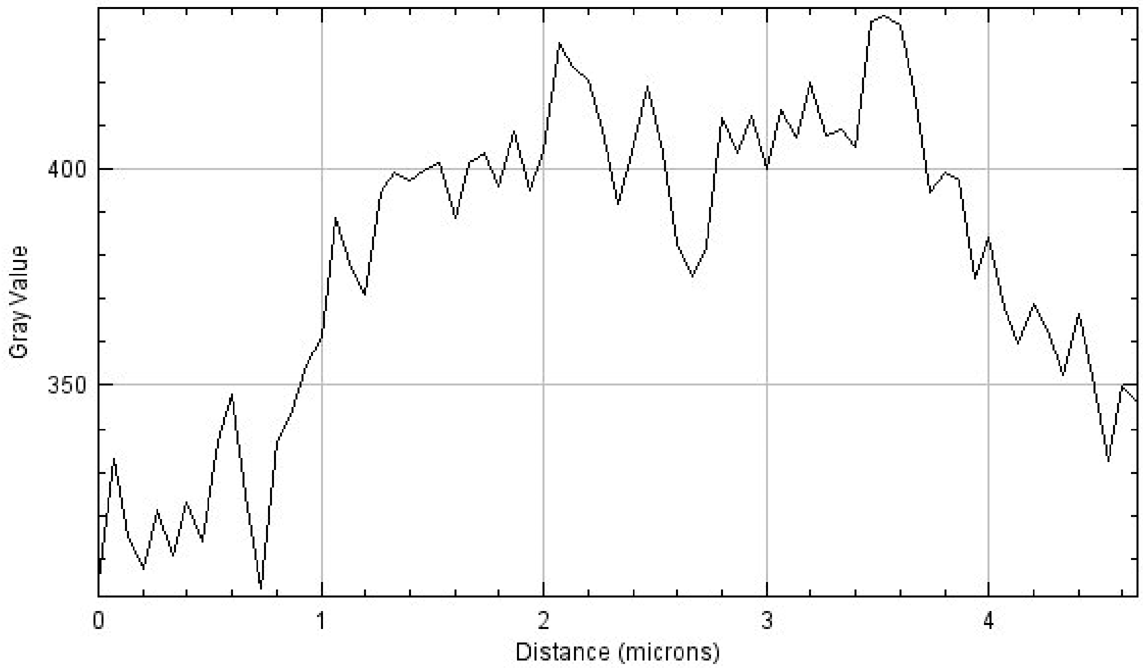

Figure S637 – Intensity profile of line scan generated for cell 3, Figure S634.

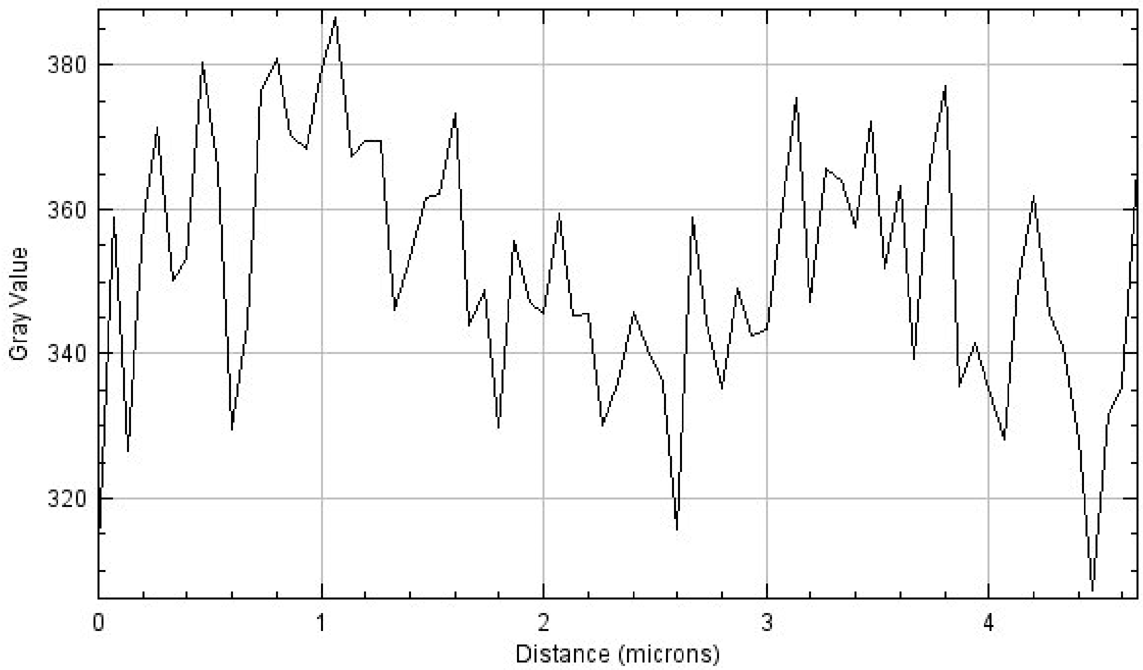

Figure S638 – Intensity profile of line scan generated for cell 4, Figure S634.

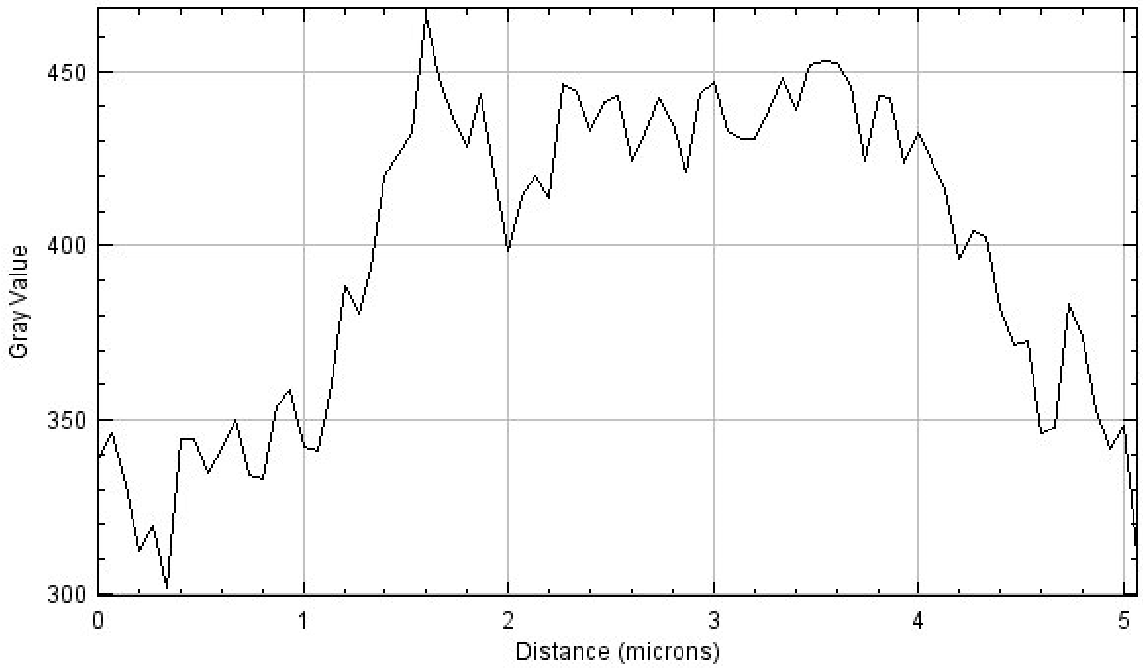

Figure S639 – Intensity profile of line scan generated for cell 5, Figure S634.

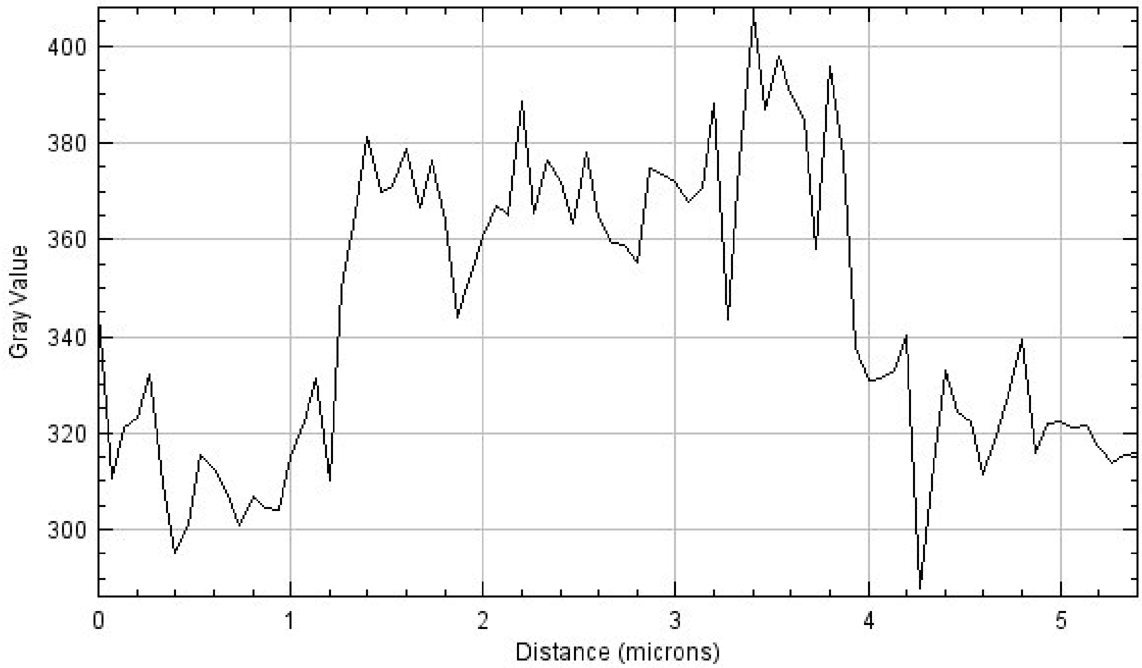

Figure S640 – Intensity profile of line scan generated for cell 6, Figure S634.

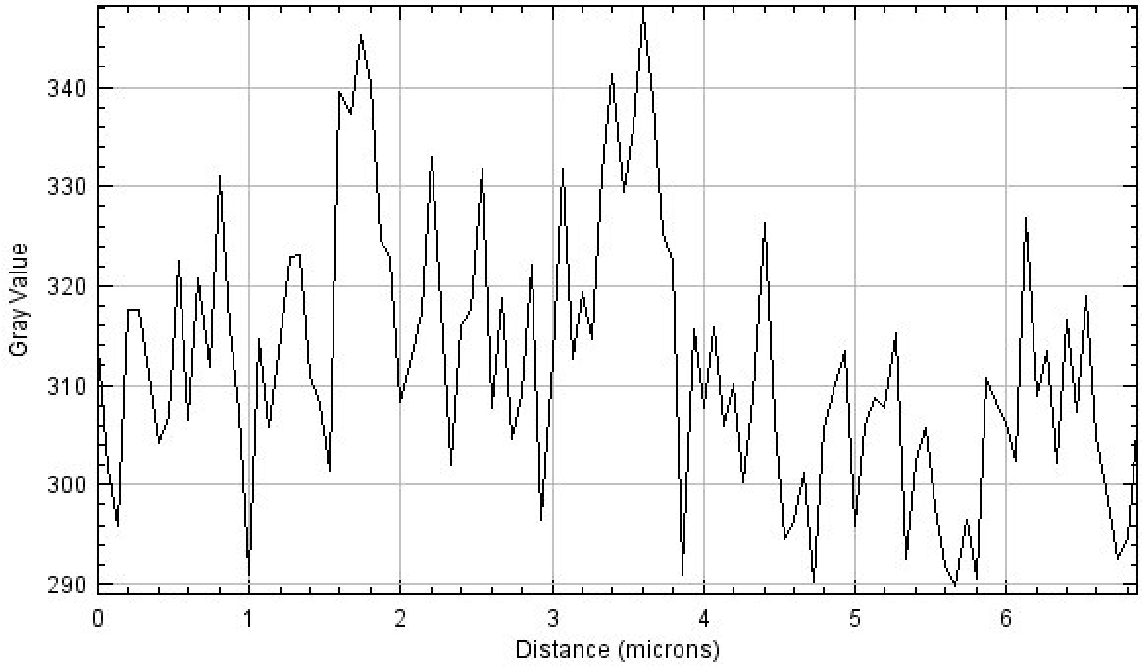

Figure S641 – Intensity profile of line scan generated for cell 7, Figure S634.

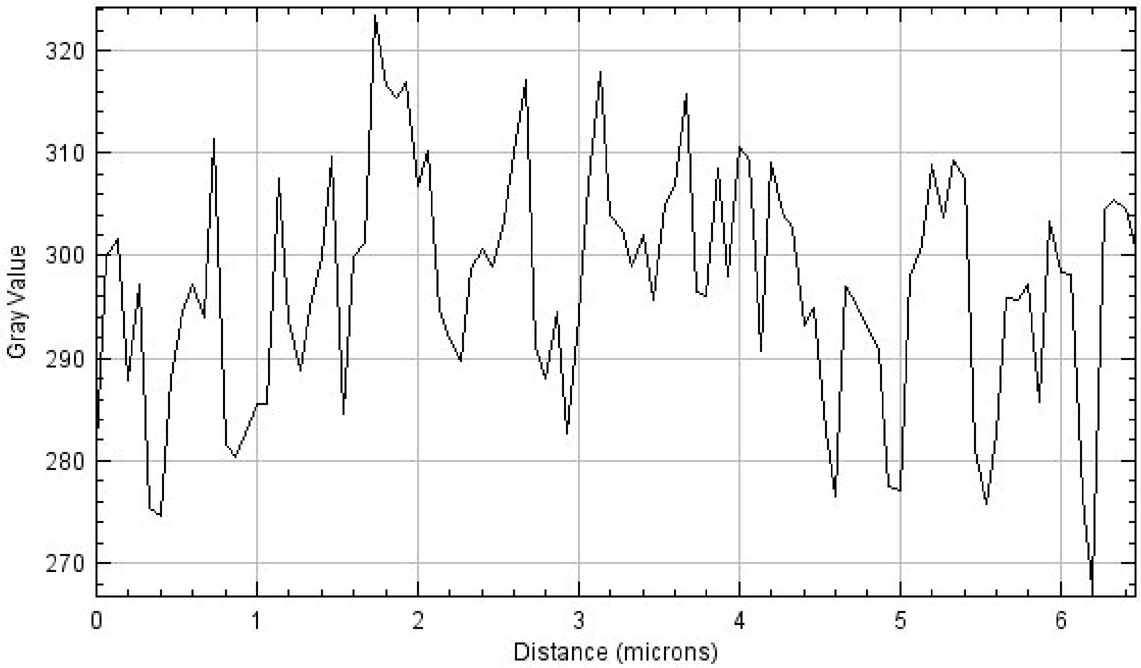

Figure S642 – Intensity profile of line scan generated for cell 8, Figure S634.

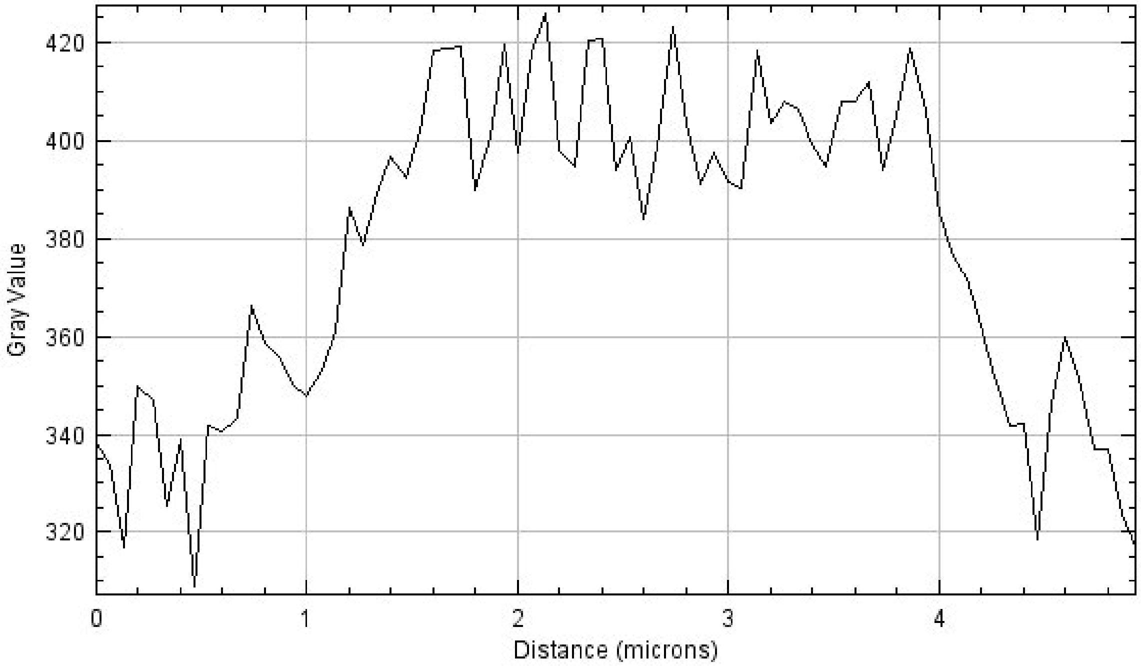

Figure S643 – Intensity profile of line scan generated for cell 9, Figure S634.

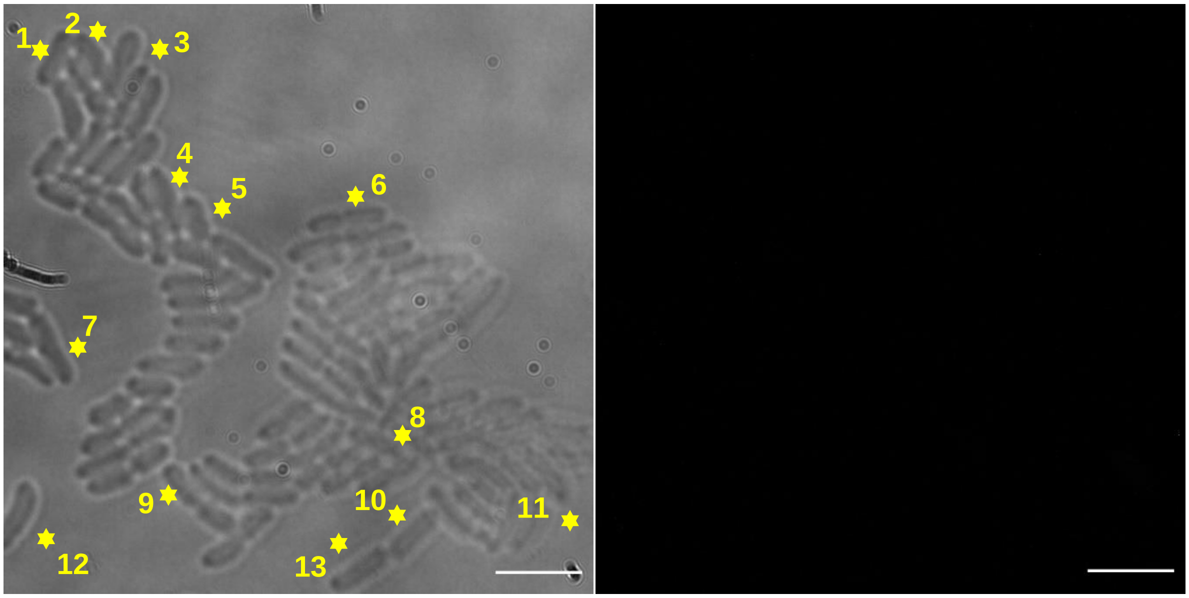

Figure S644 – *E. coli* transmitted and mCherry images used in fluorescence intensity calculations in the presence of compound **39** at T = 30 minutes. Scale bar = 10 µM.

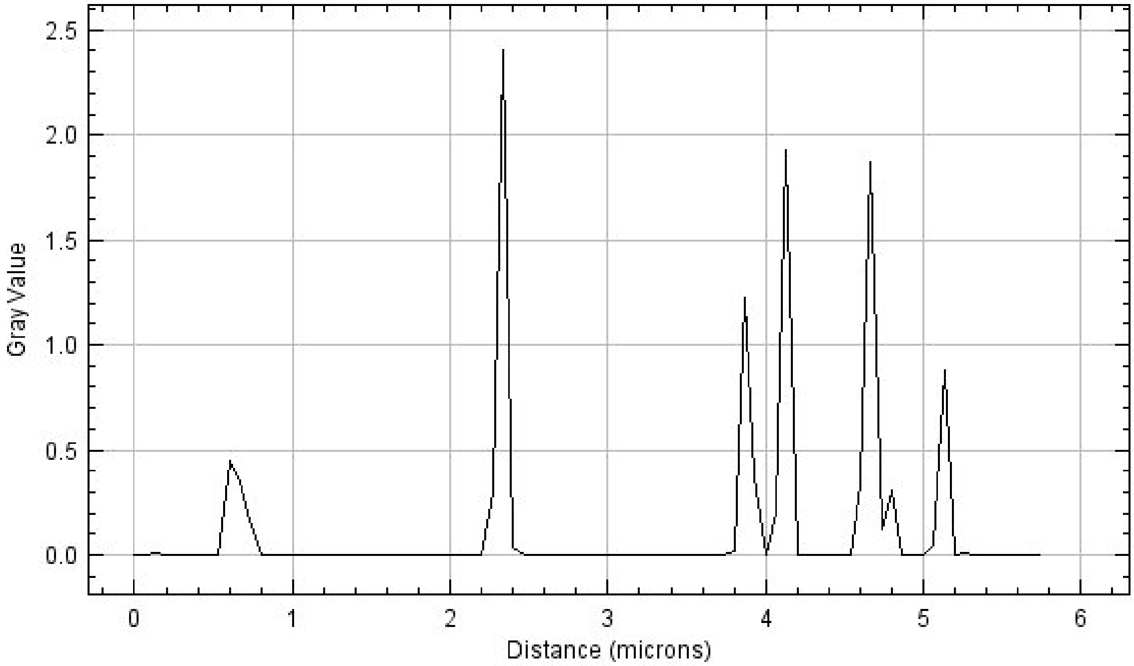

Figure S645 – Intensity profile of line scan generated for cell 1, Figure S644.

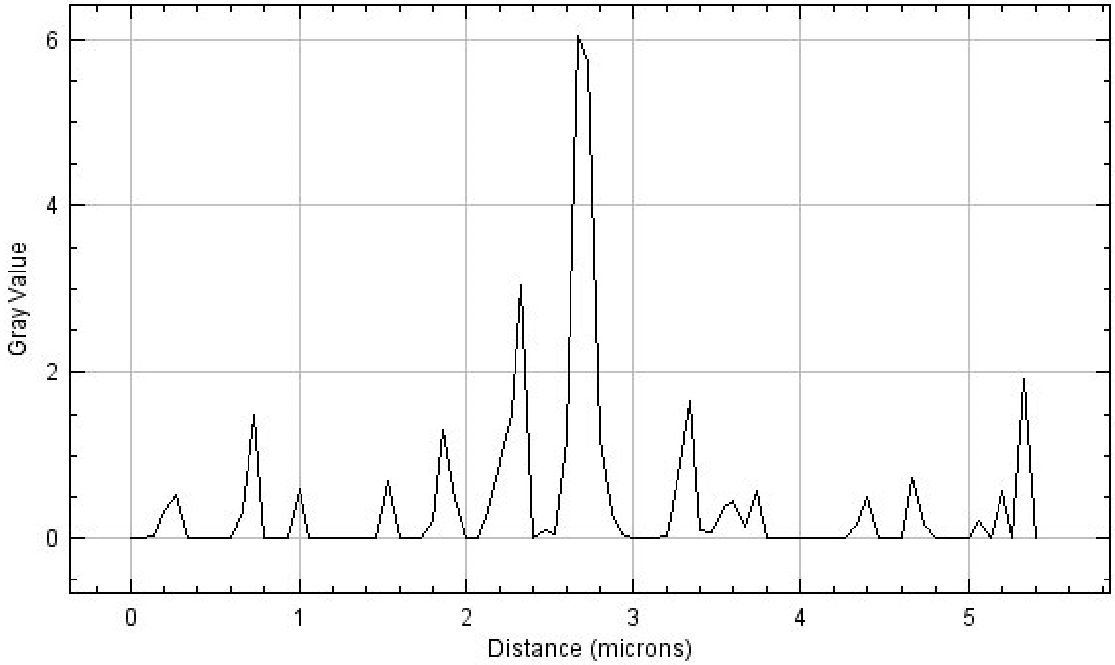

Figure S646 – Intensity profile of line scan generated for cell 2, Figure S644.

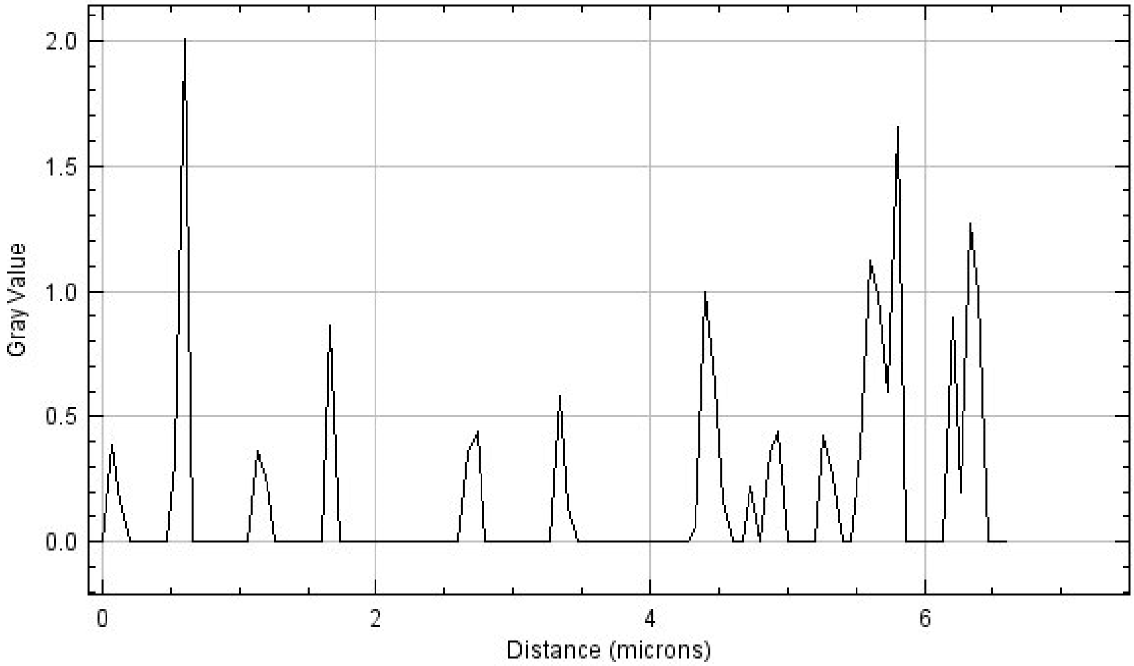

Figure S647 – Intensity profile of line scan generated for cell 3, Figure S644.

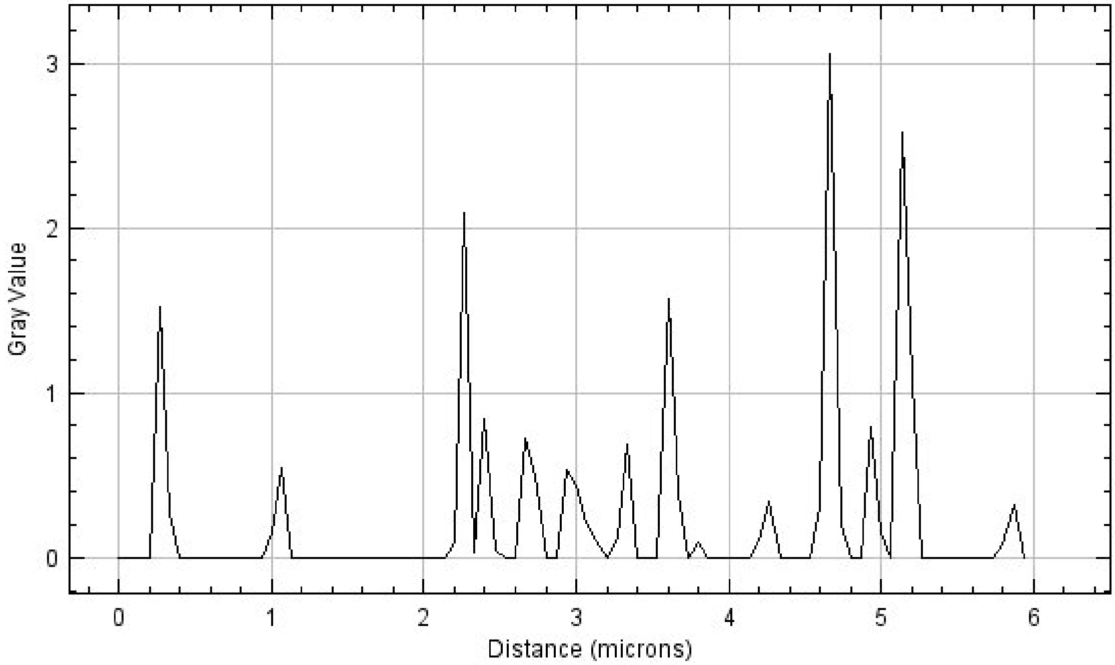

Figure S648 – Intensity profile of line scan generated for cell 4, Figure S644.

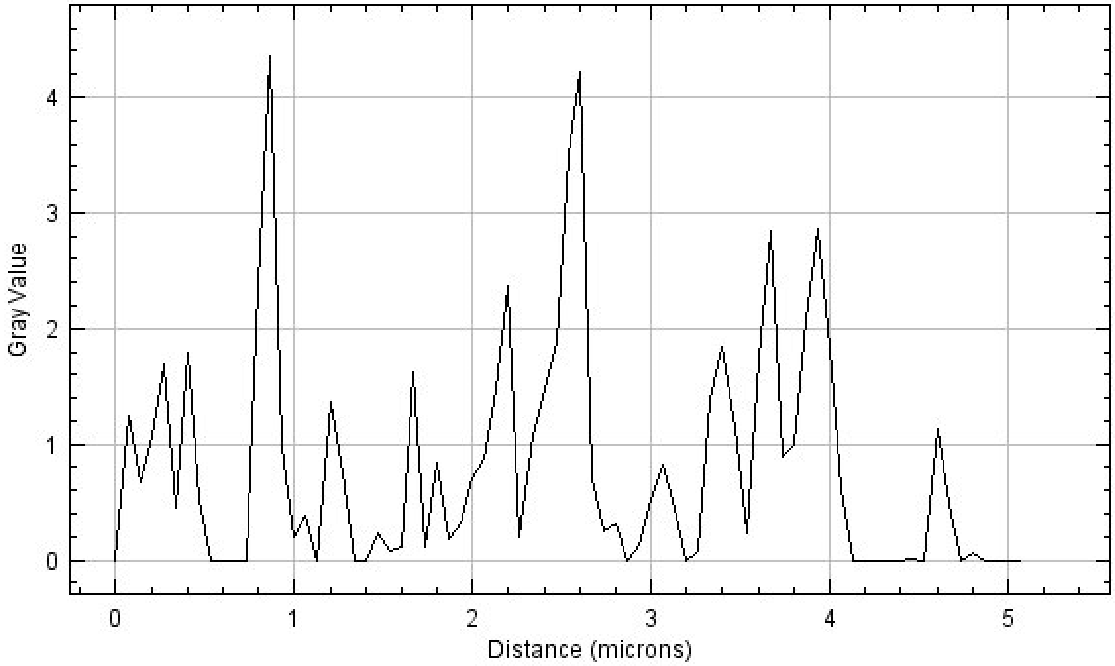

Figure S649 – Intensity profile of line scan generated for cell 5, Figure S644.

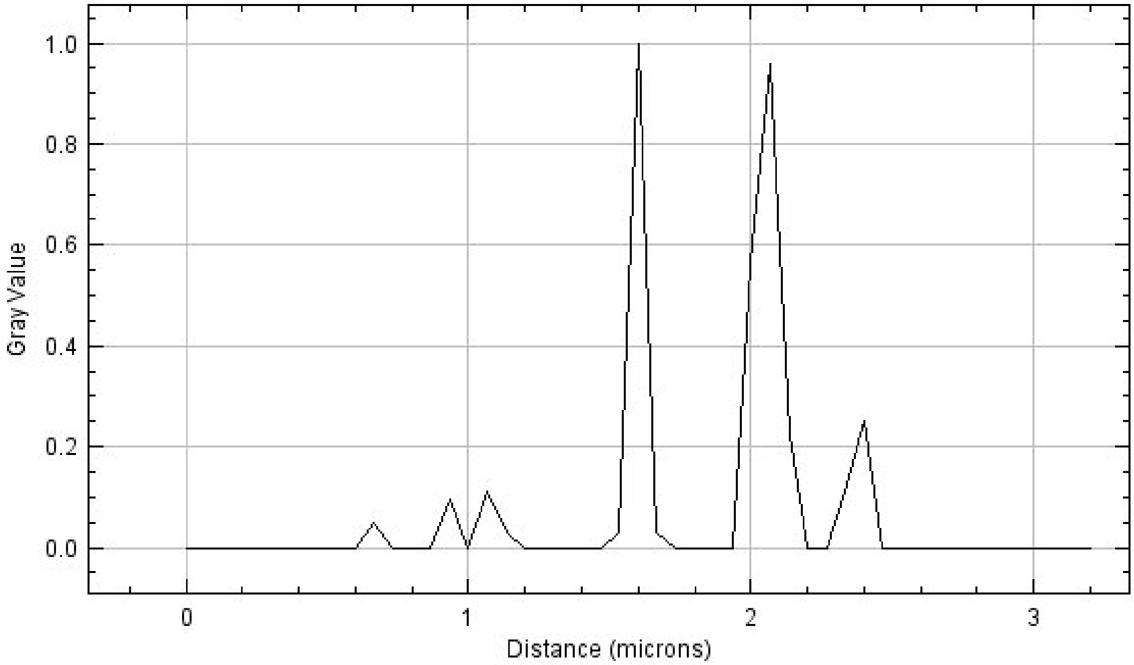

Figure S650 – Intensity profile of line scan generated for cell 6, Figure S644.

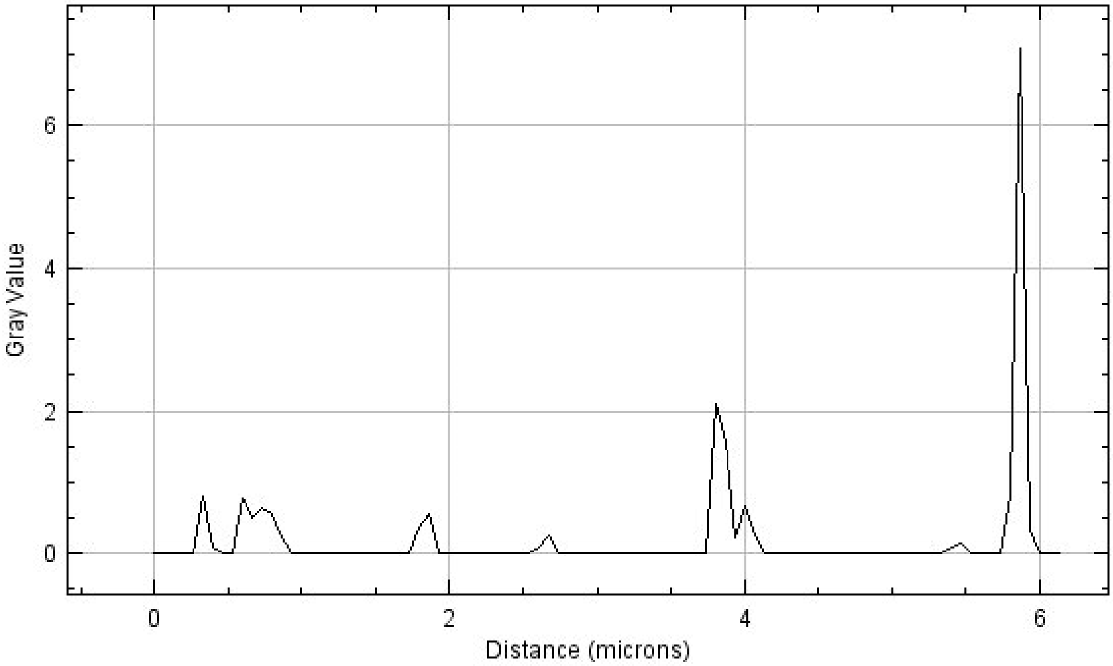

Figure S651 – Intensity profile of line scan generated for cell 7, Figure S644.

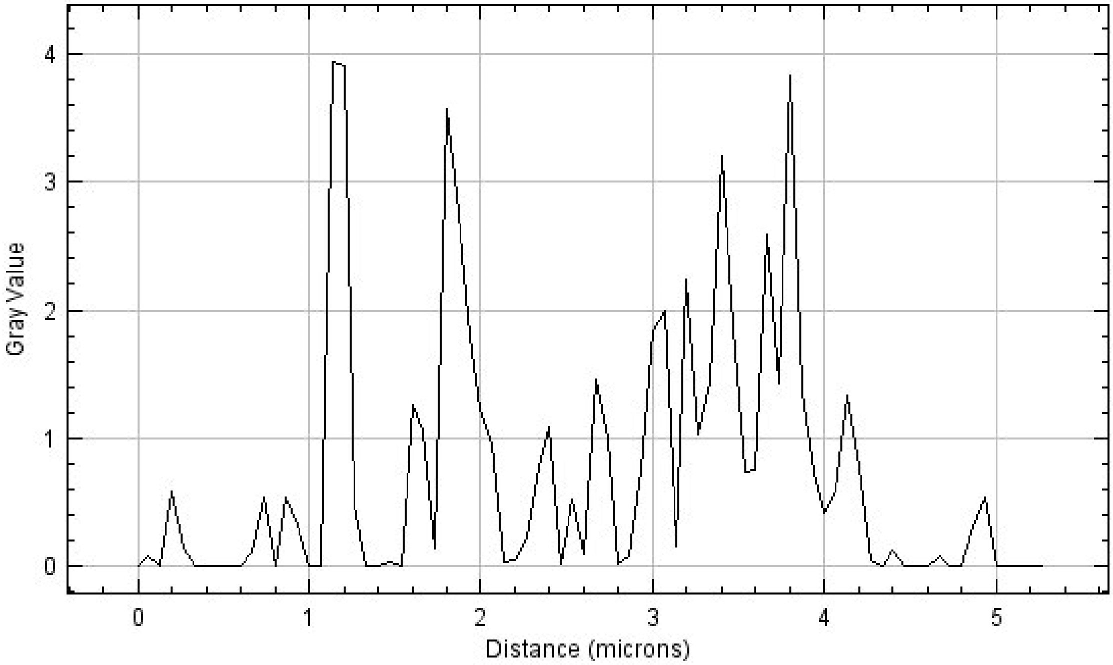

Figure S652 – Intensity profile of line scan generated for cell 8, Figure S644.

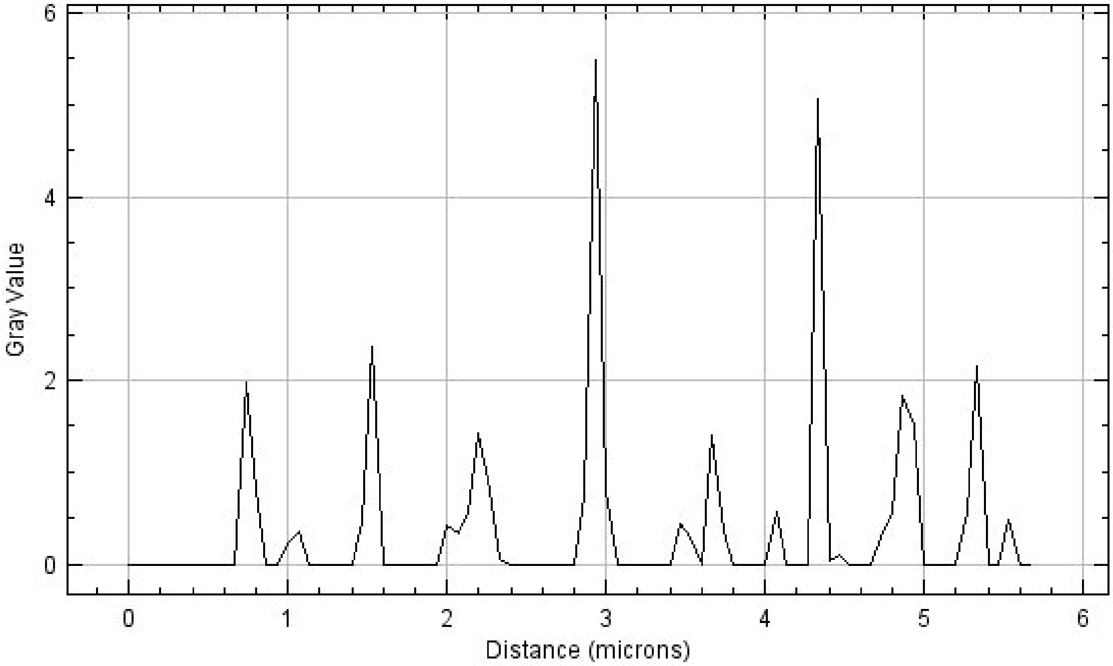

Figure S653 – Intensity profile of line scan generated for cell 9, Figure S644.

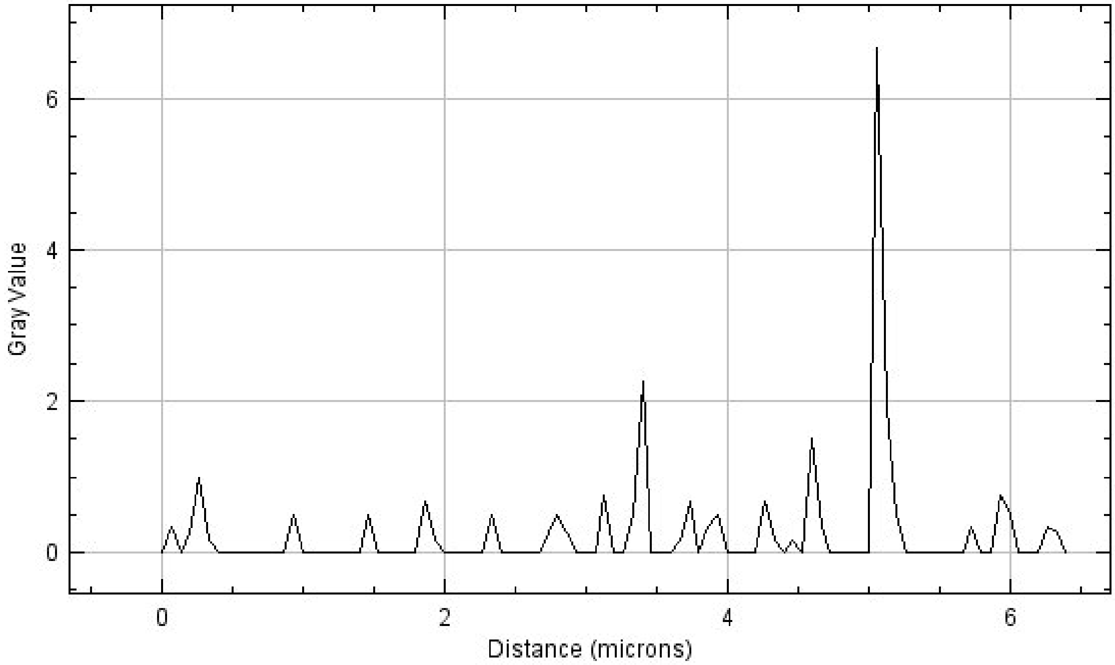

Figure S654 – Intensity profile of line scan generated for cell 10, Figure S644.

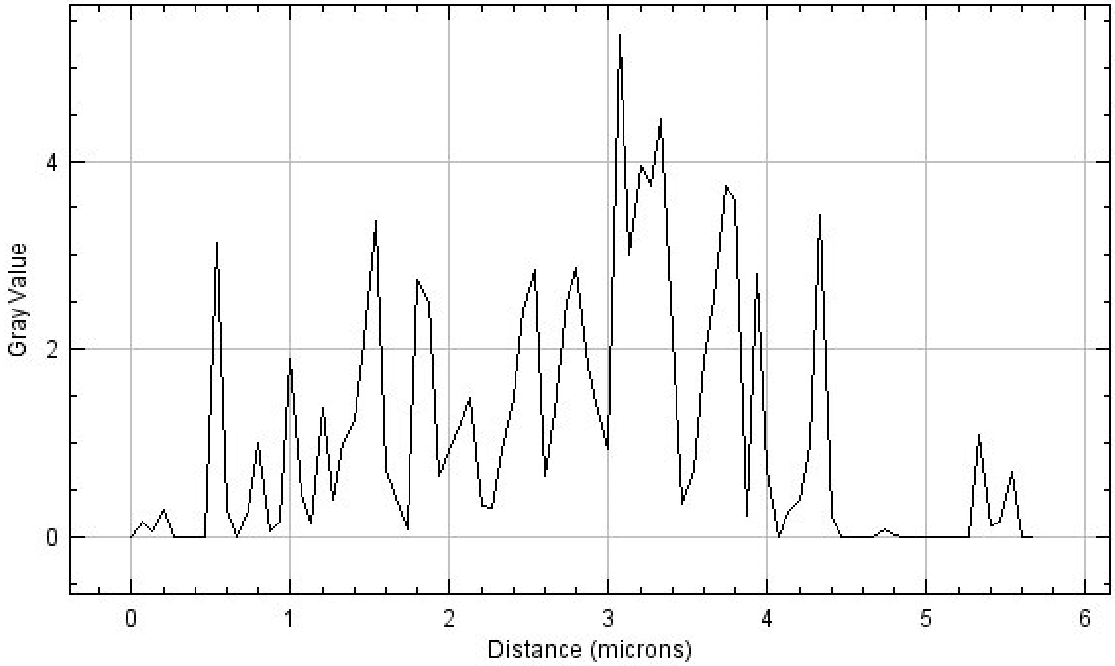

Figure S655 – Intensity profile of line scan generated for cell 11, Figure S644.

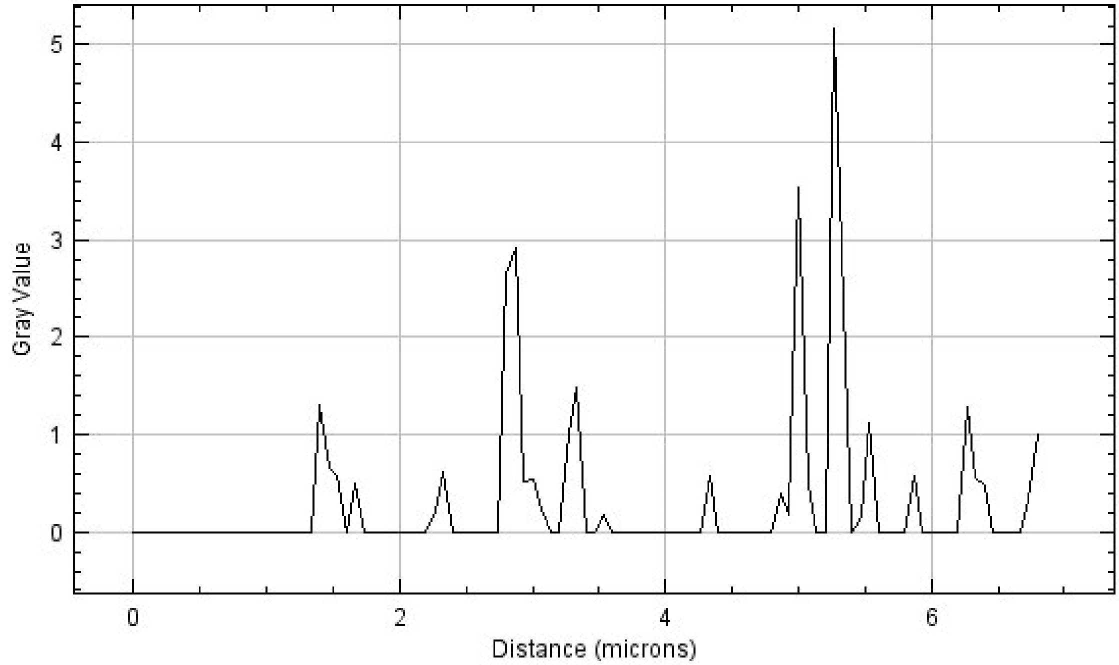

Figure S656 – Intensity profile of line scan generated for cell 12, Figure S644.

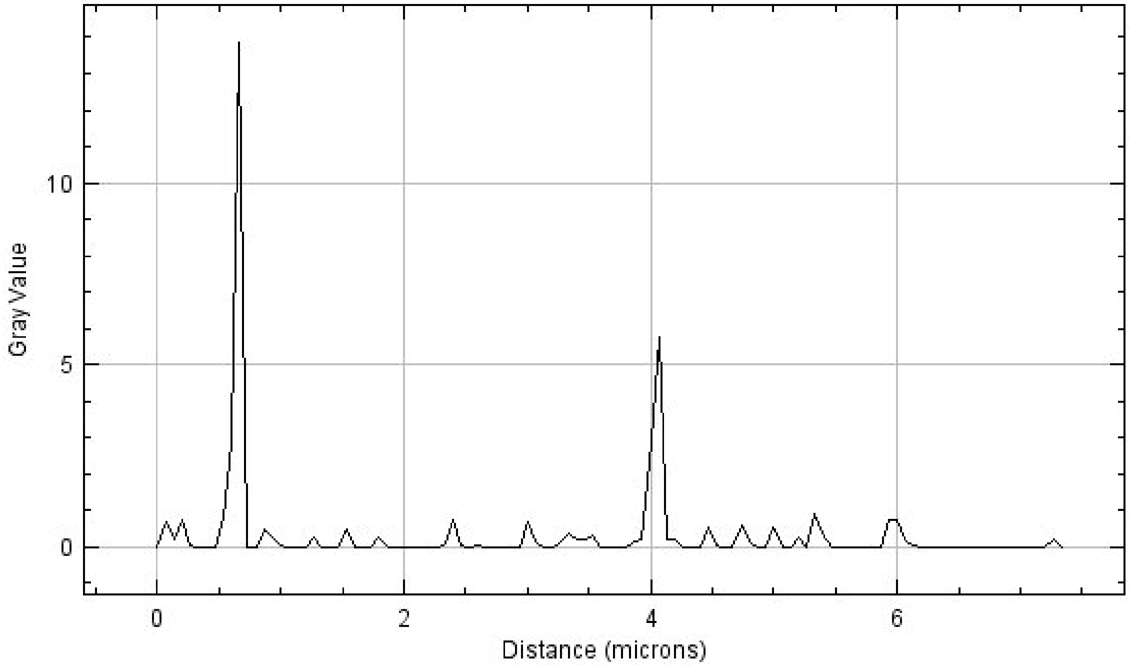

Figure S657 – Intensity profile of line scan generated for cell 13, Figure S644.

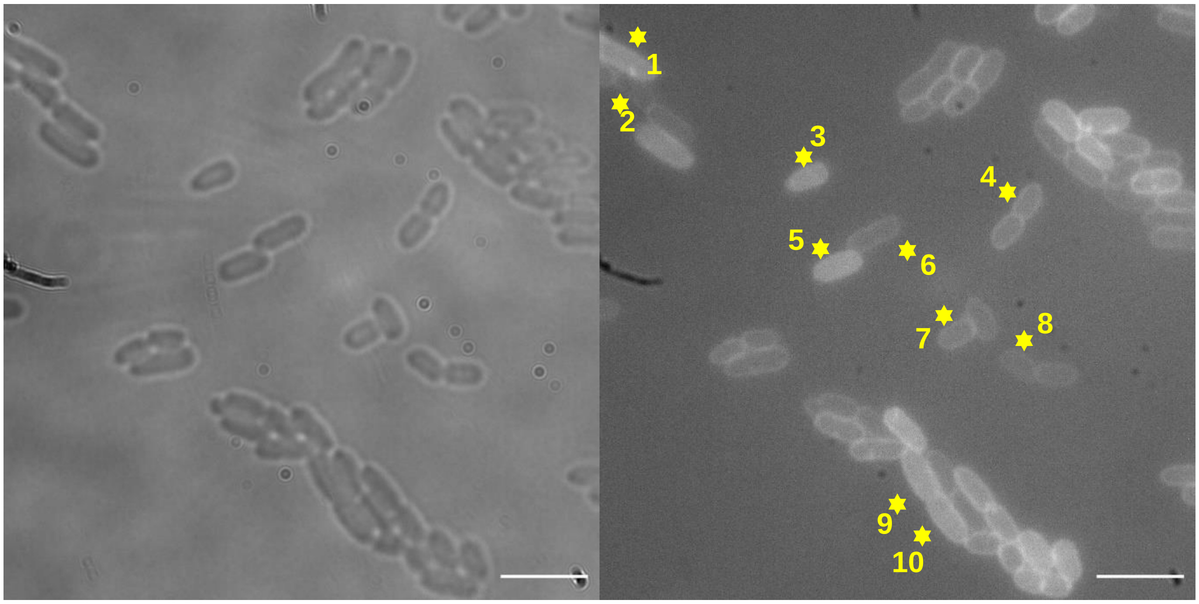

Figure S658 – *E. coli* transmitted and DAPI images used in fluorescence intensity calculations in the presence of compound **39** at T = 30 minutes. Scale bar = 10 µM.

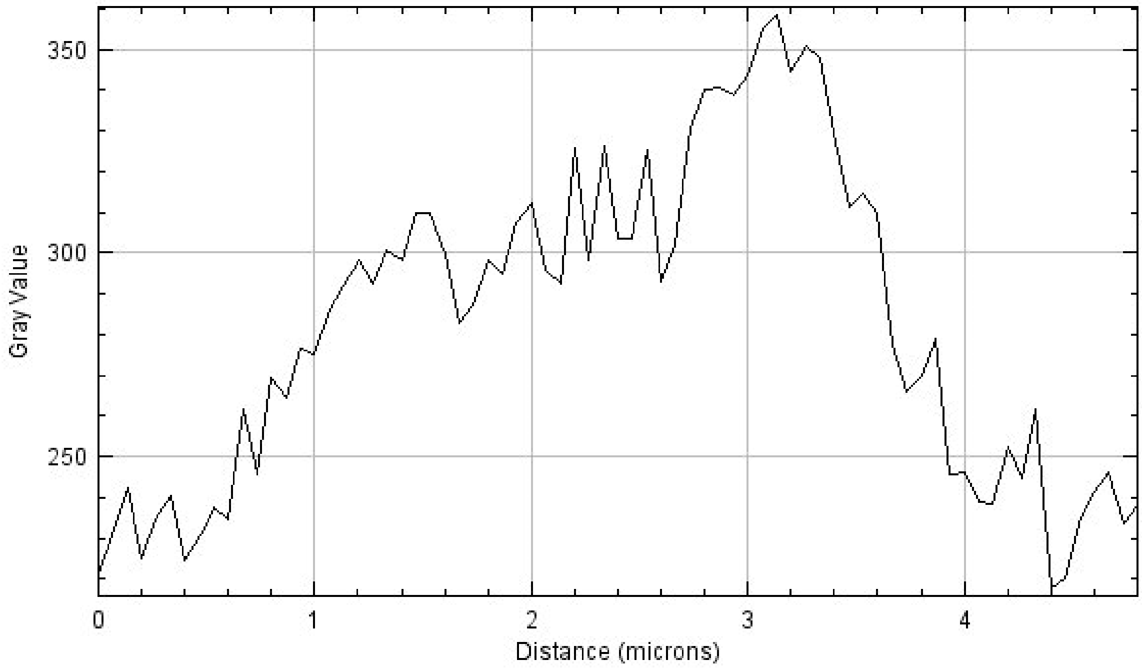

Figure S659 – Intensity profile of line scan generated for cell 1, Figure S658.

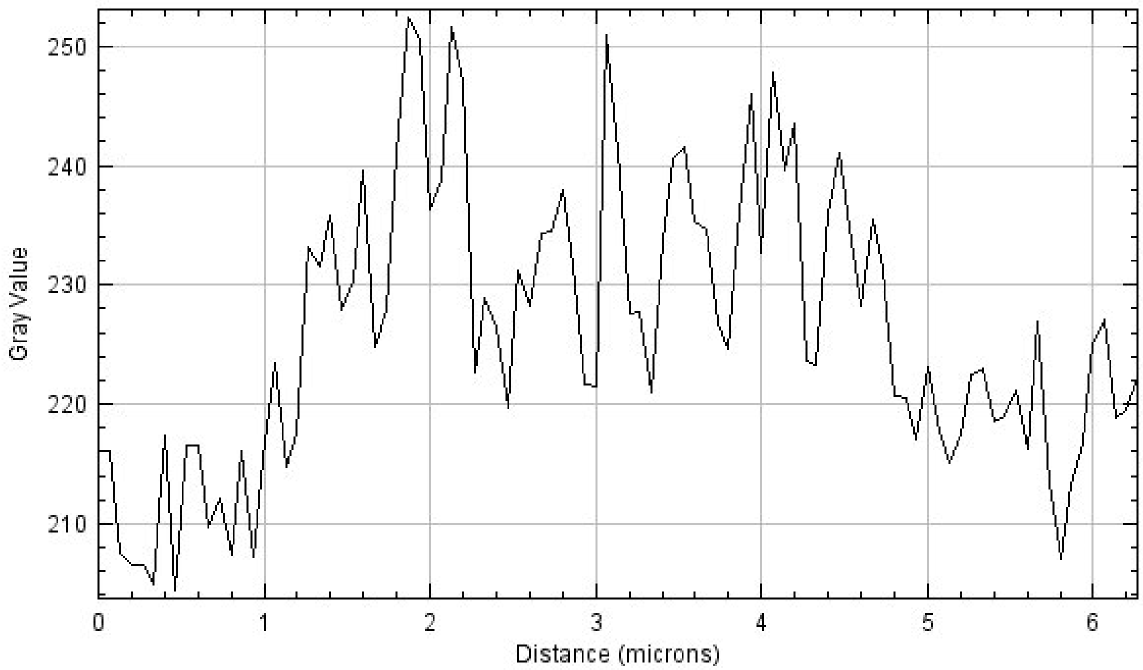

Figure S660 – Intensity profile of line scan generated for cell 2, Figure S658.

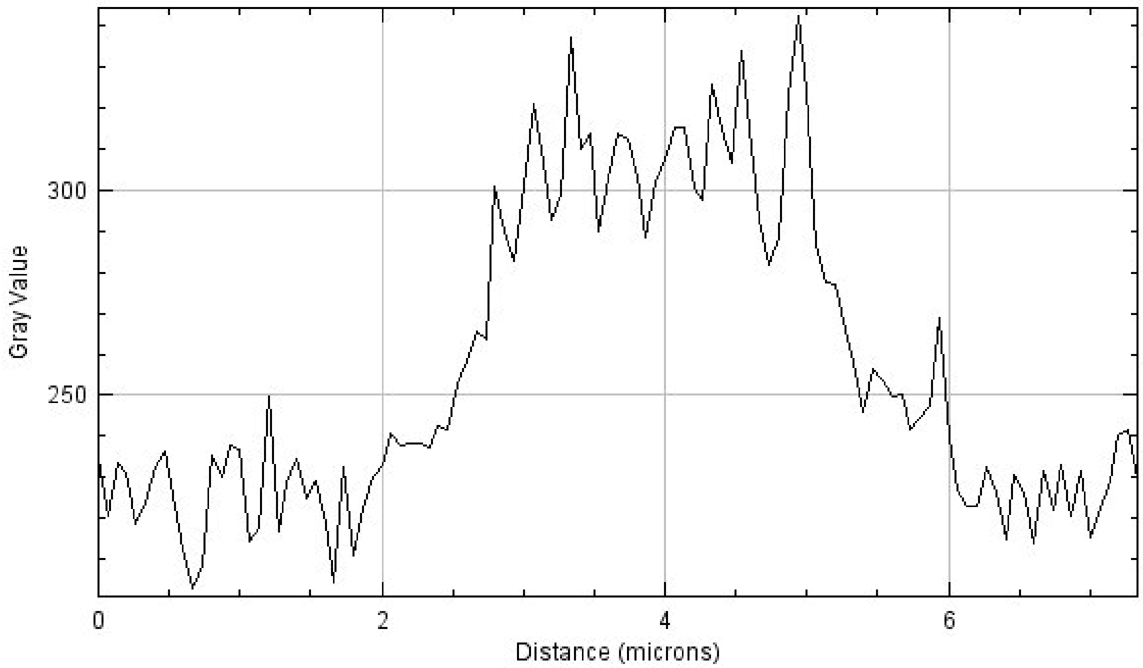

Figure S661 – Intensity profile of line scan generated for cell 3, Figure S658.

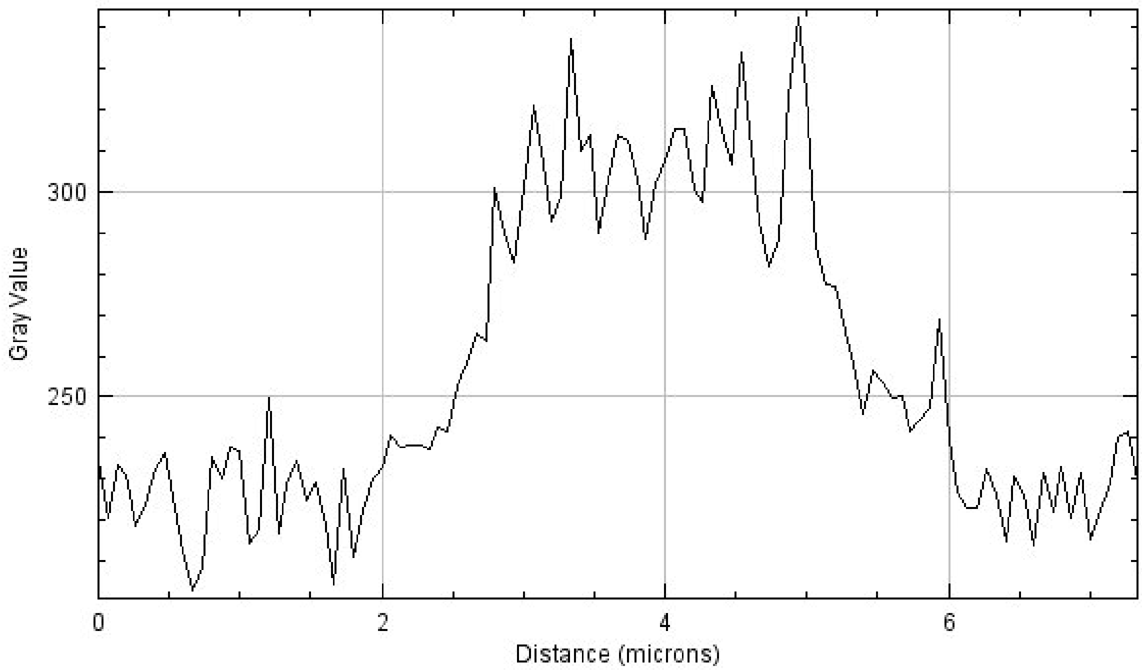

Figure S662 – Intensity profile of line scan generated for cell 4, Figure S658.

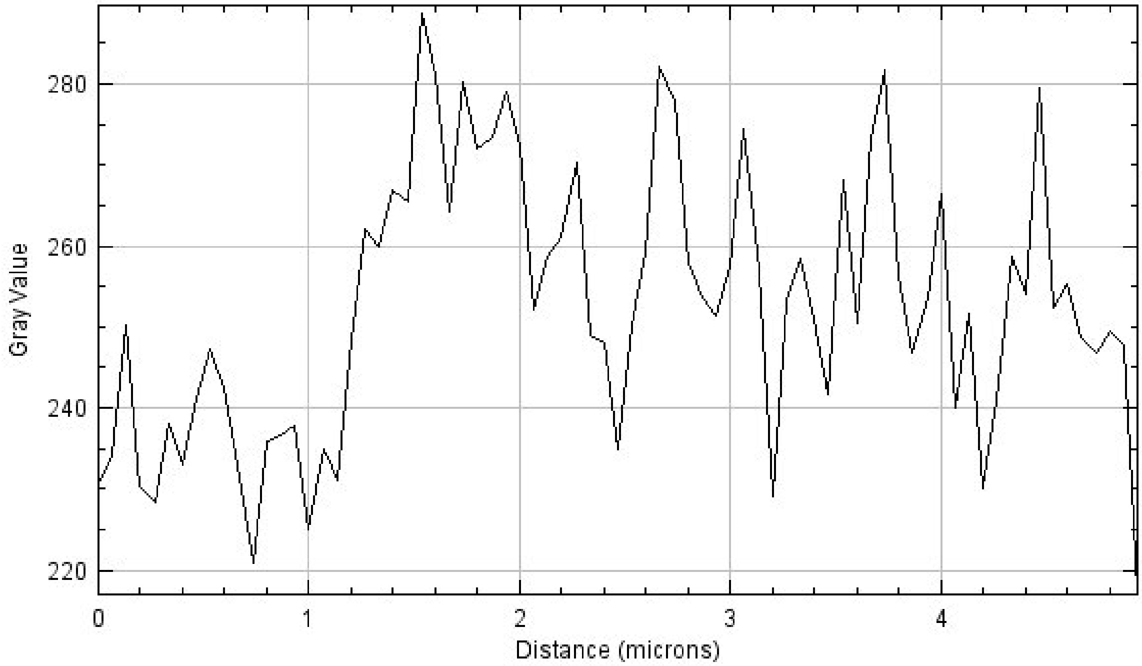

Figure S663 – Intensity profile of line scan generated for cell 5, Figure S658.

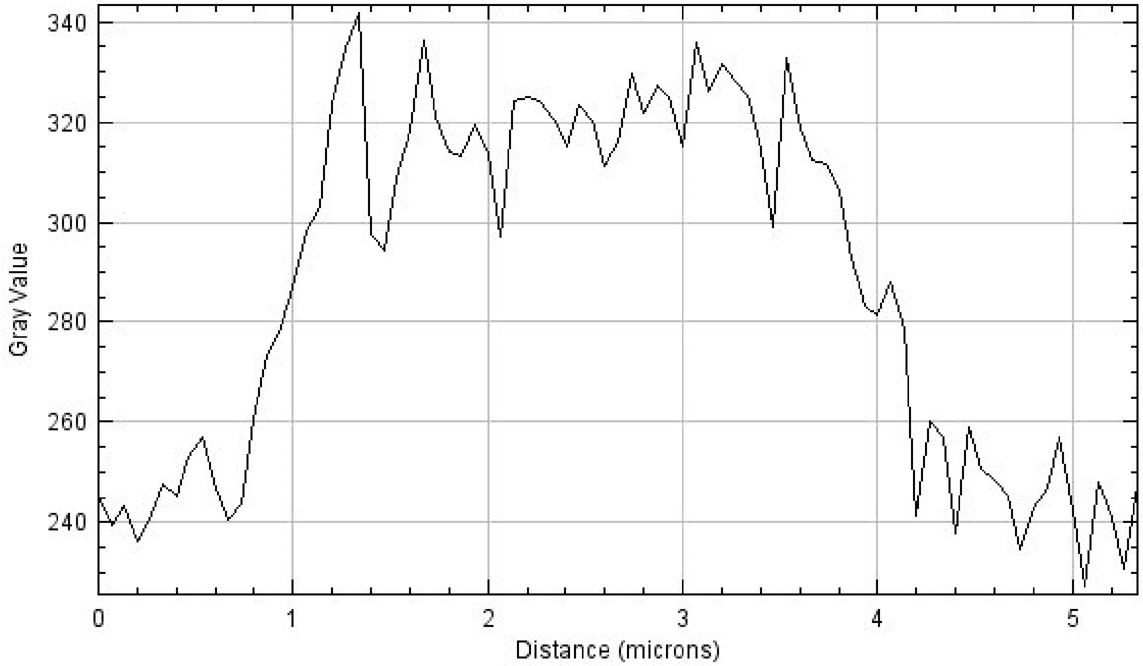

Figure S664 – Intensity profile of line scan generated for cell 6, Figure S658.

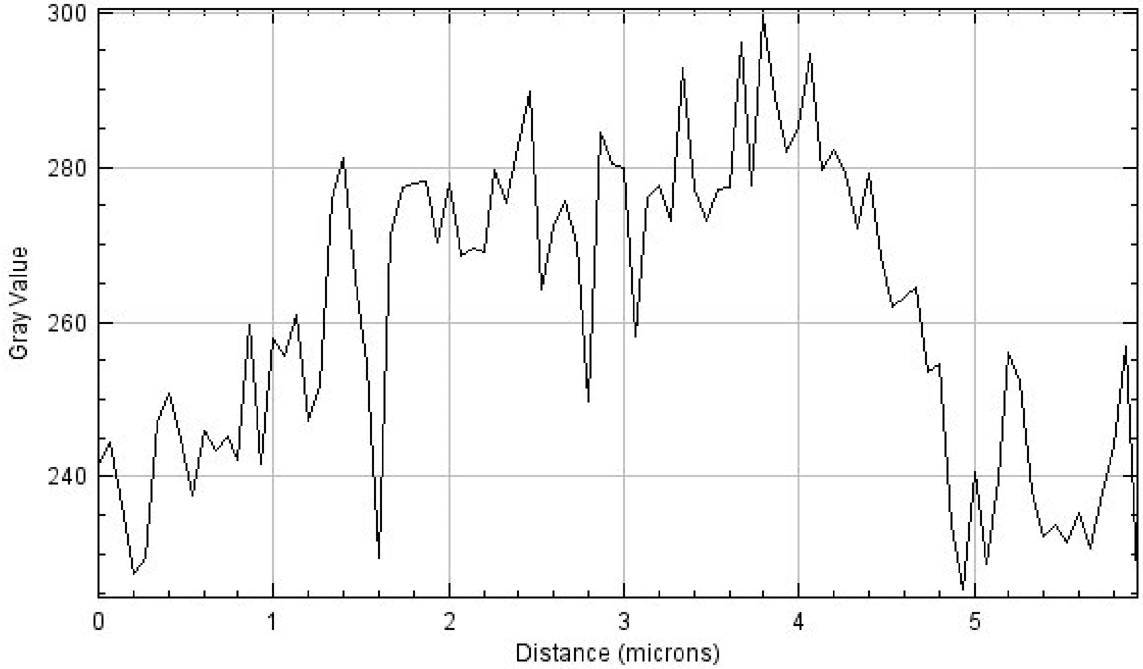

Figure S665 – Intensity profile of line scan generated for cell 7, Figure S658.

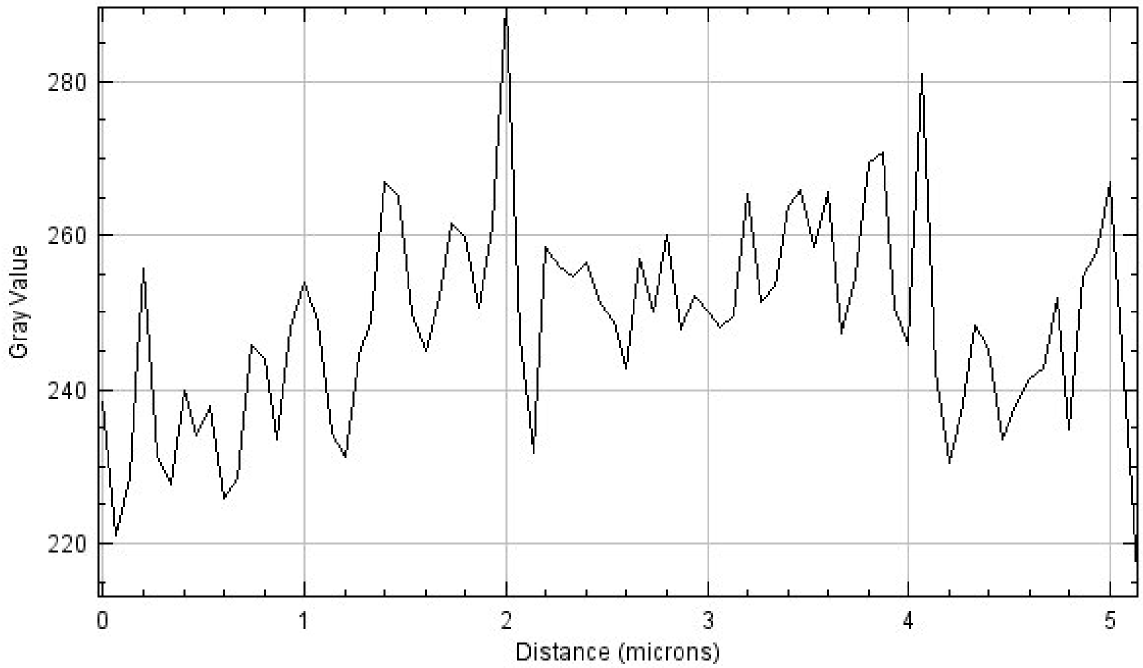

Figure S666 – Intensity profile of line scan generated for cell 8, Figure S658.

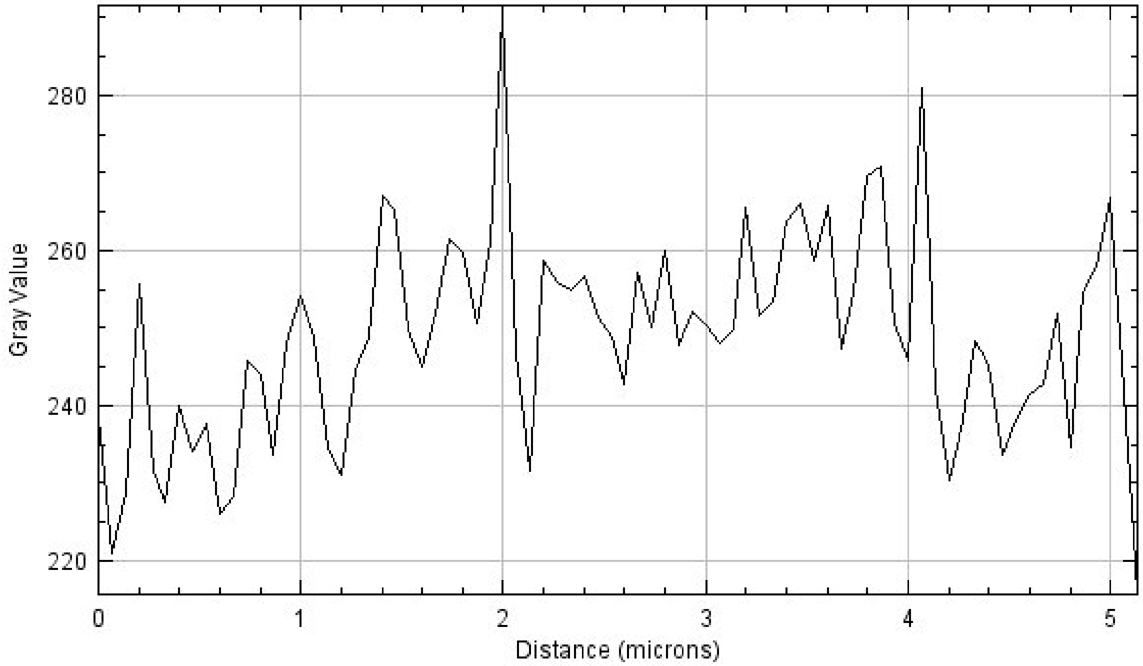

Figure S667 – Intensity profile of line scan generated for cell 9, Figure S658.

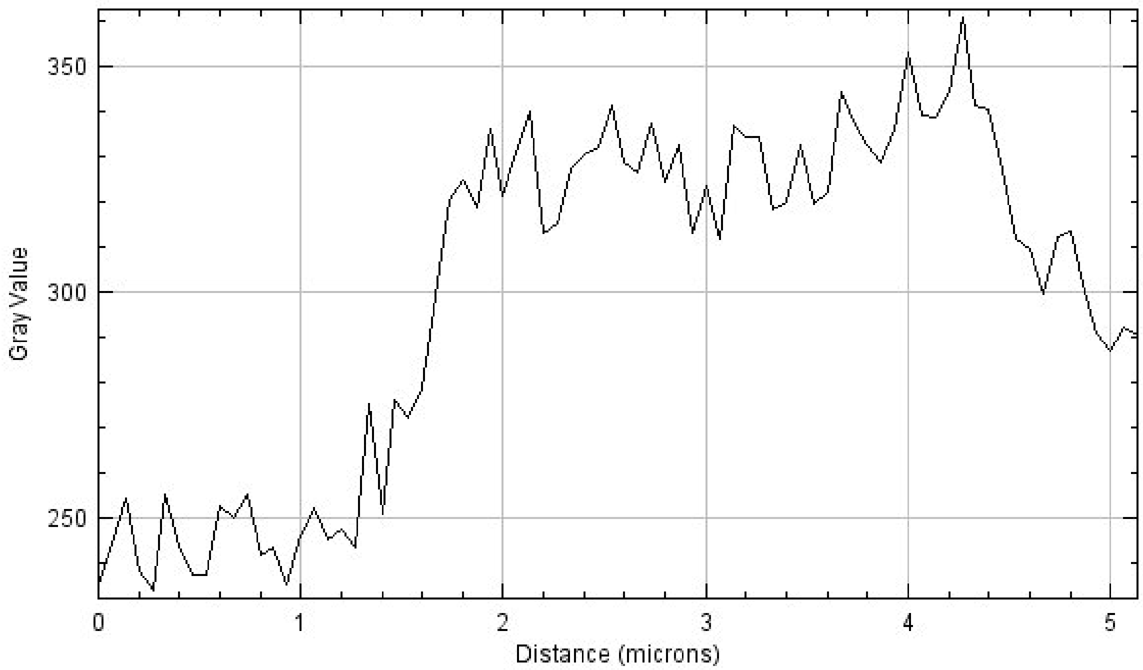

Figure S668 – Intensity profile of line scan generated for cell 10, Figure S658.

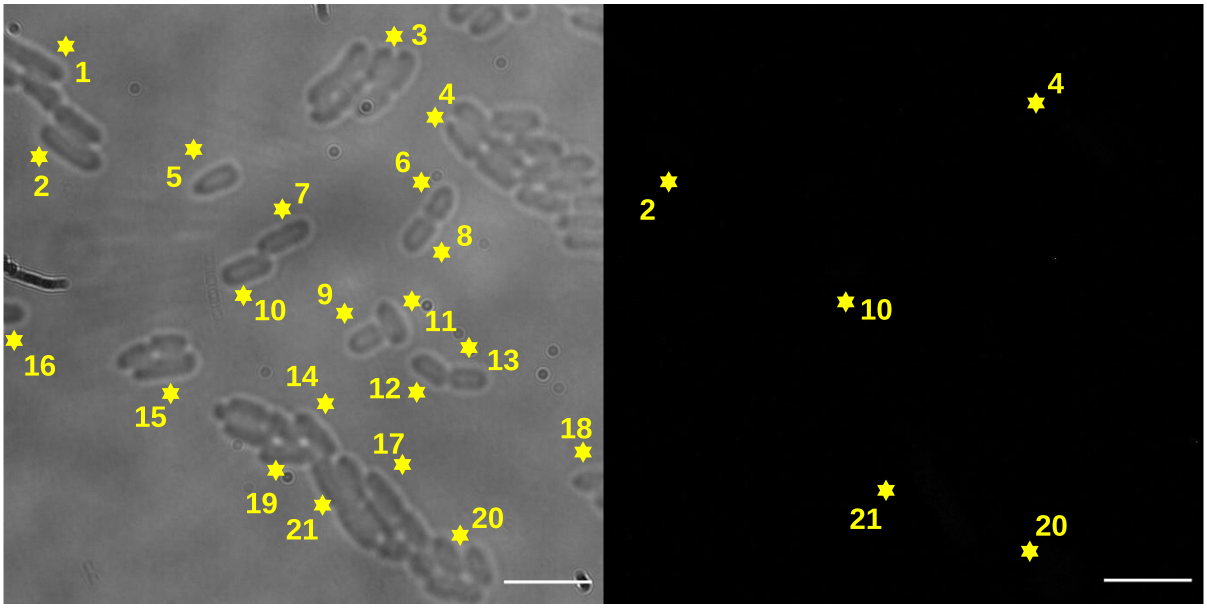

Figure S669 – *E. coli* transmitted and mCherry images used in fluorescence intensity calculations in the presence of compound **39** at T = 30 minutes. Scale bar = 10 µM.

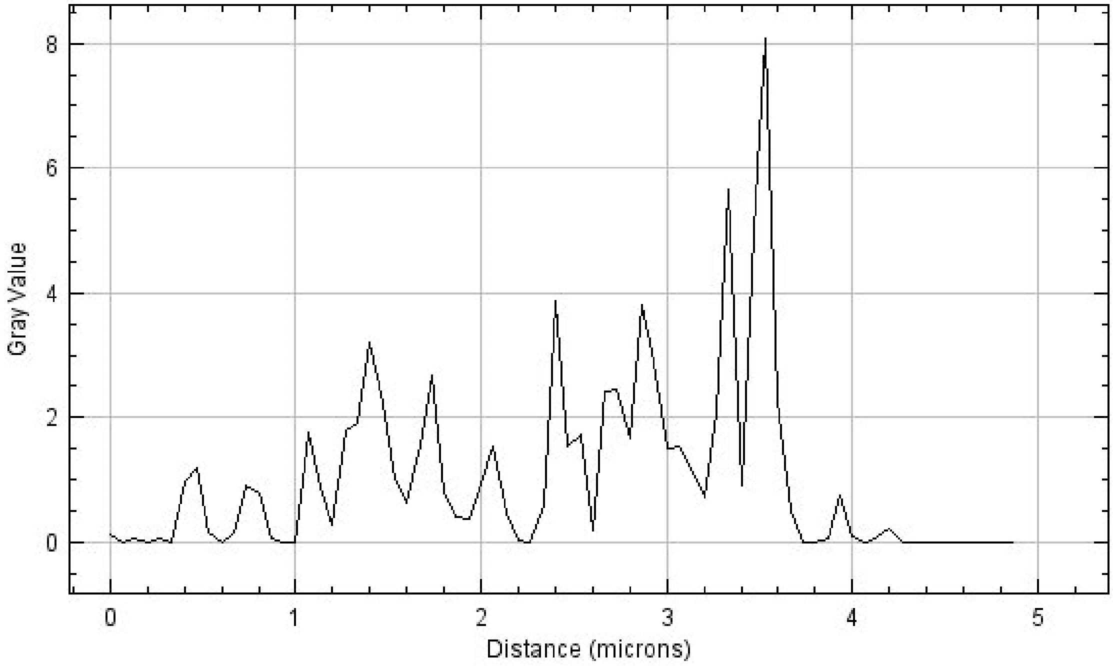

Figure S670 – Intensity profile of line scan generated for cell 1, Figure S669.

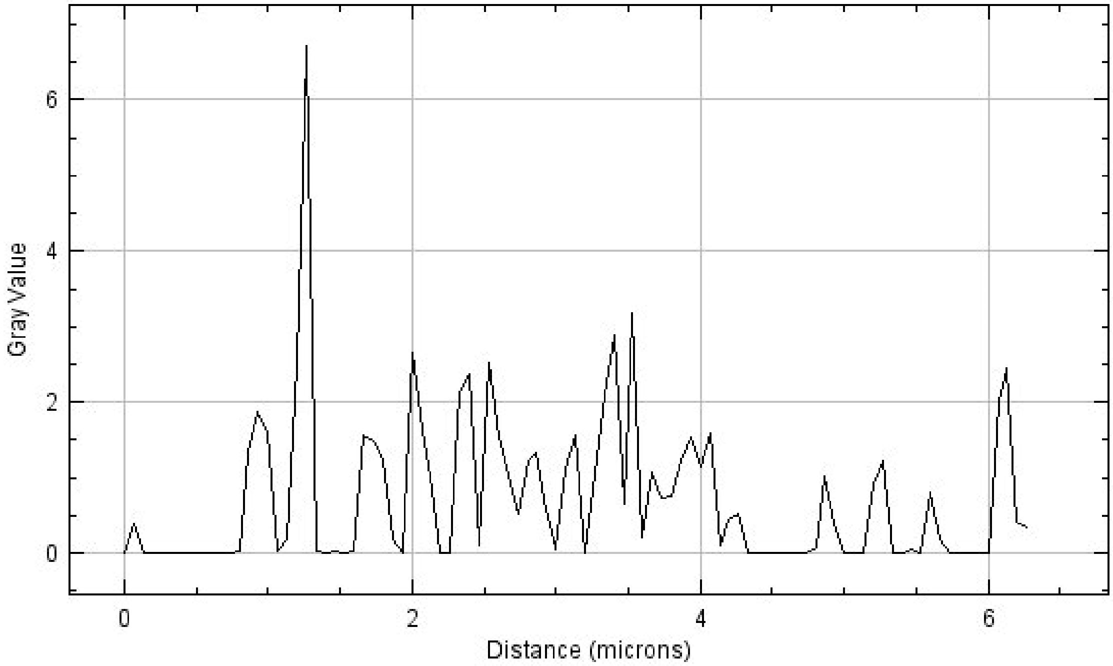

Figure S671 – Intensity profile of line scan generated for cell 2, Figure S669.

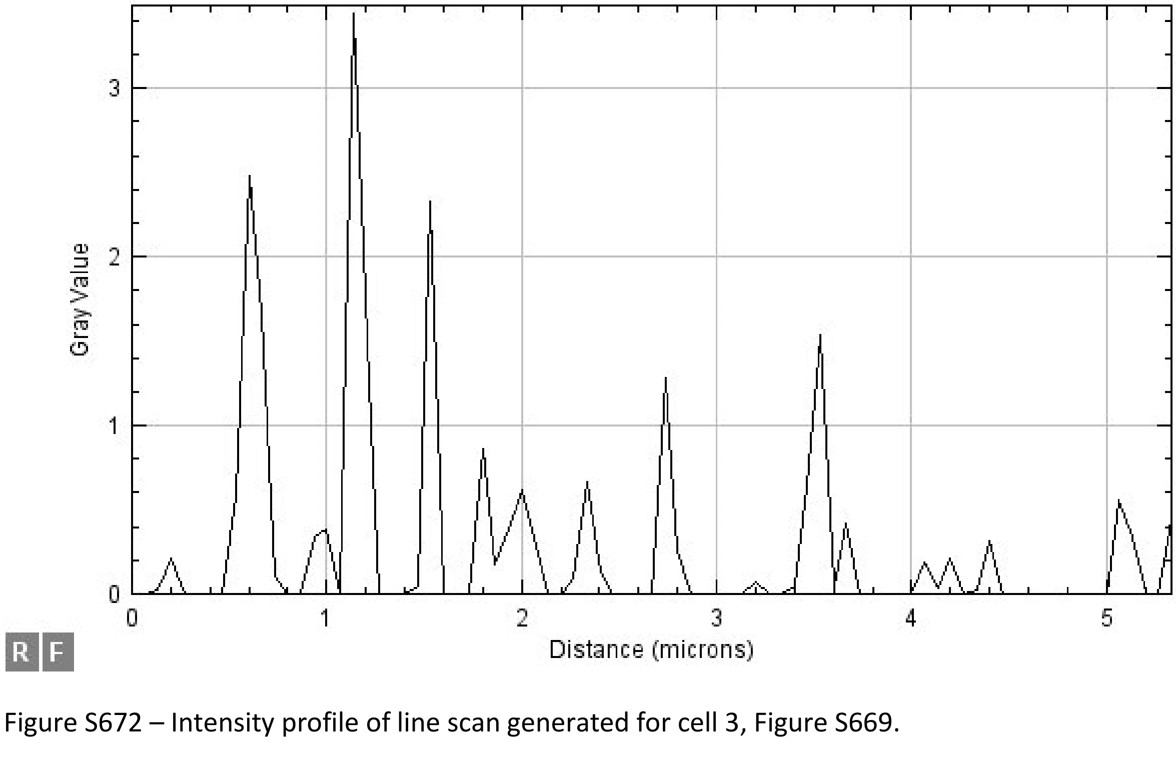

Figure S672 – Intensity profile of line scan generated for cell 3, Figure S669.

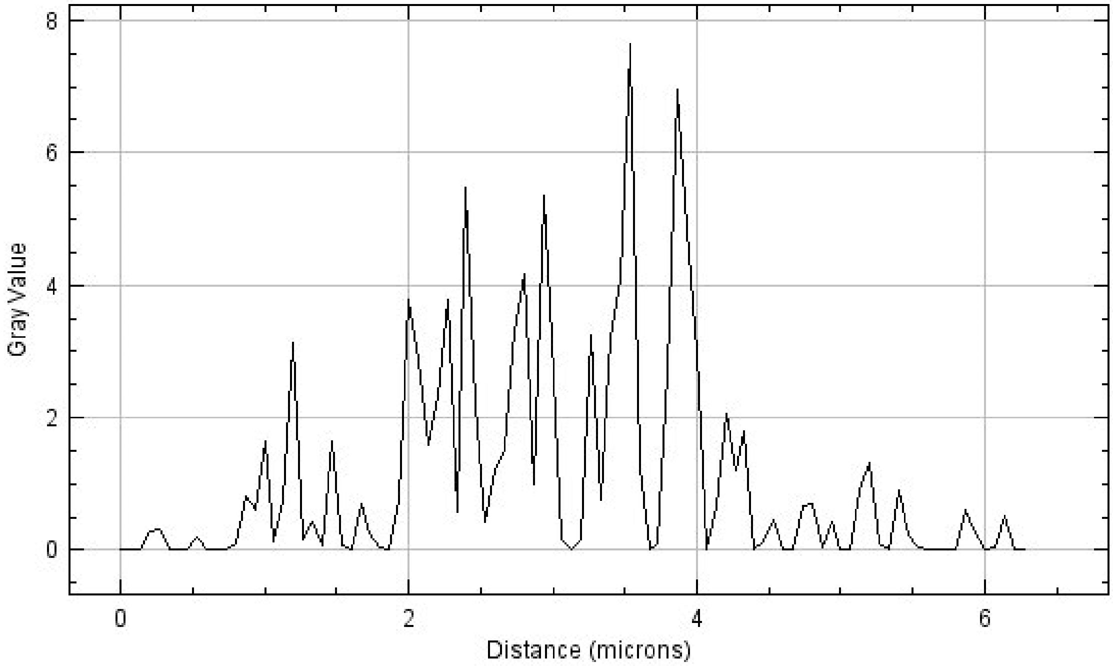

Figure S673 – Intensity profile of line scan generated for cell 4, Figure S669.

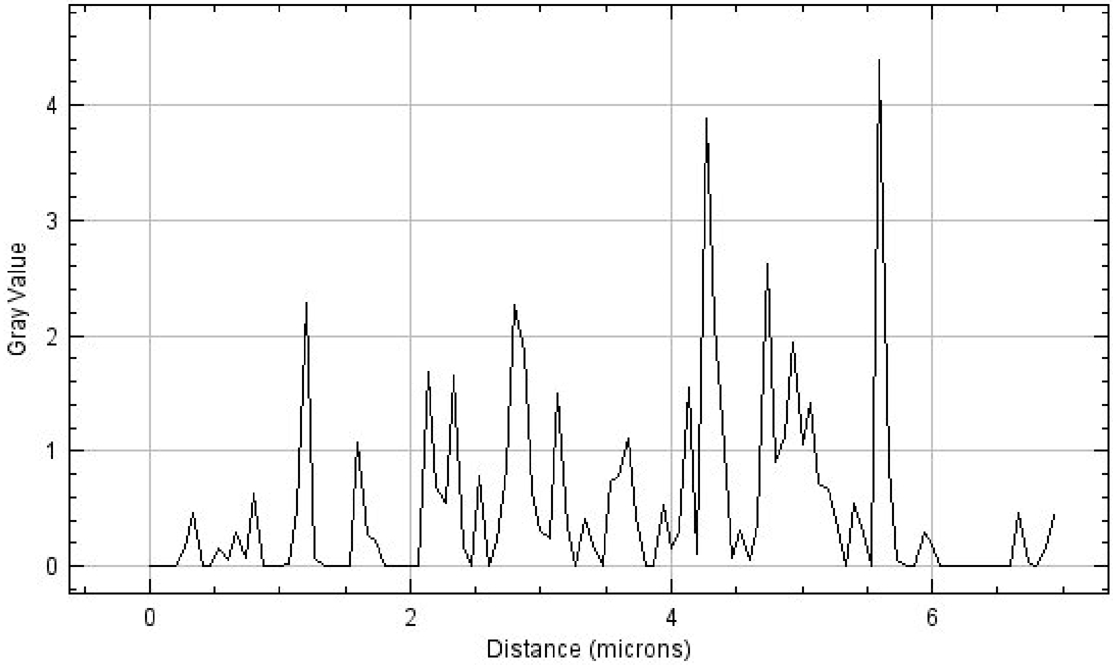

Figure S674 – Intensity profile of line scan generated for cell 5, Figure S669.

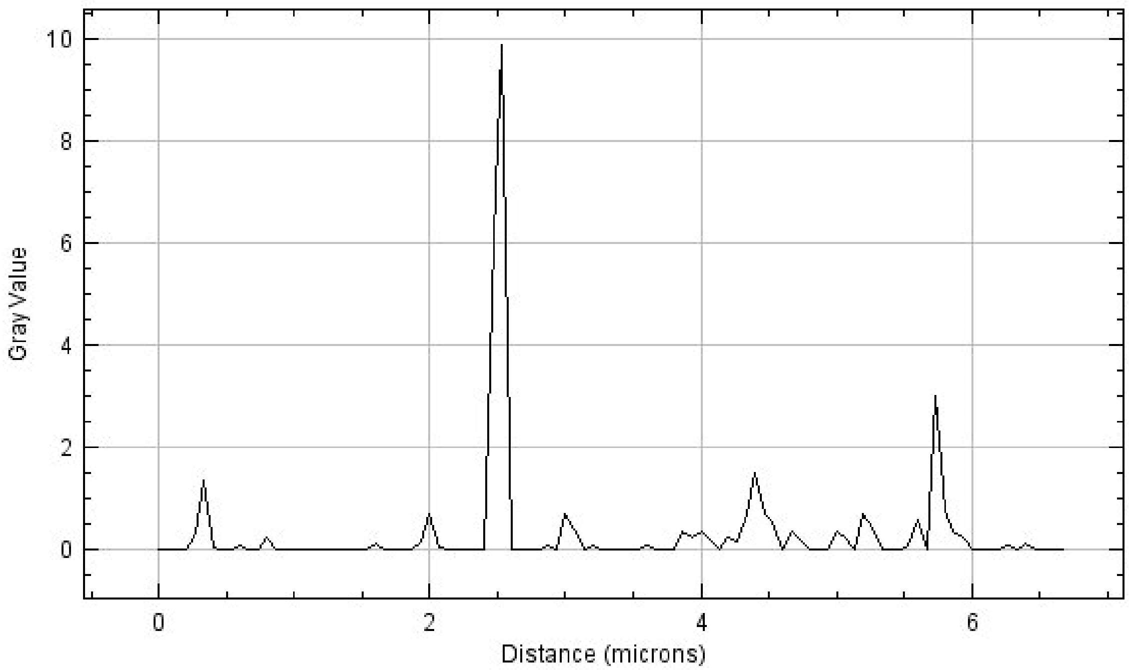

Figure S675 – Intensity profile of line scan generated for cell 6, Figure S669.

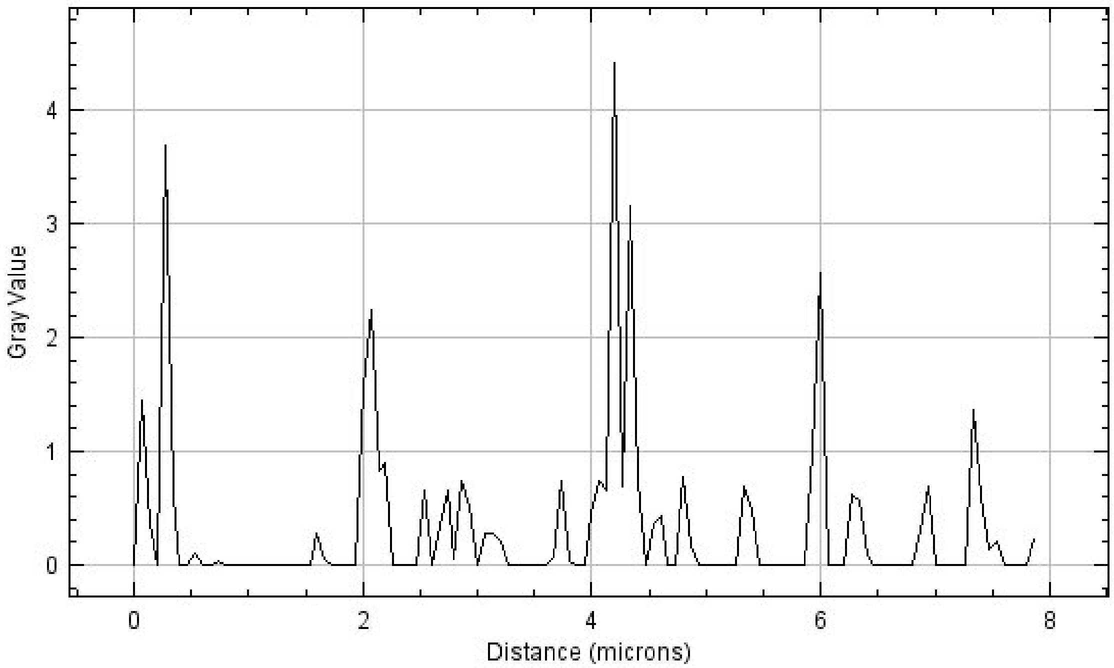

Figure S676 – Intensity profile of line scan generated for cell 7, Figure S669.

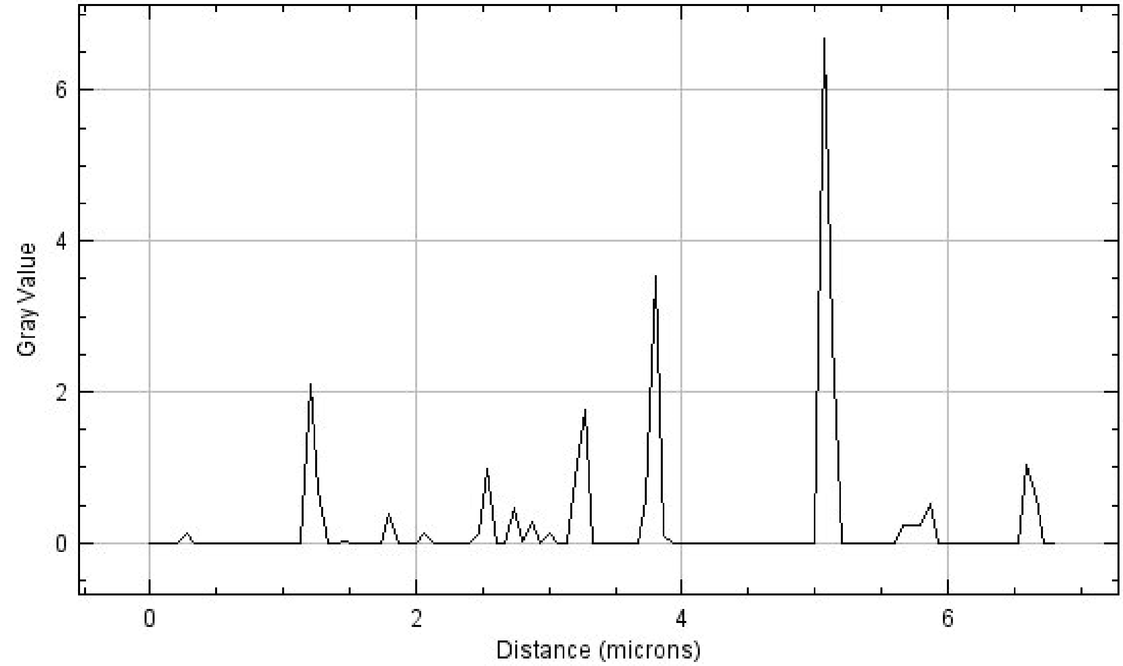

Figure S677 – Intensity profile of line scan generated for cell 8, Figure S669.

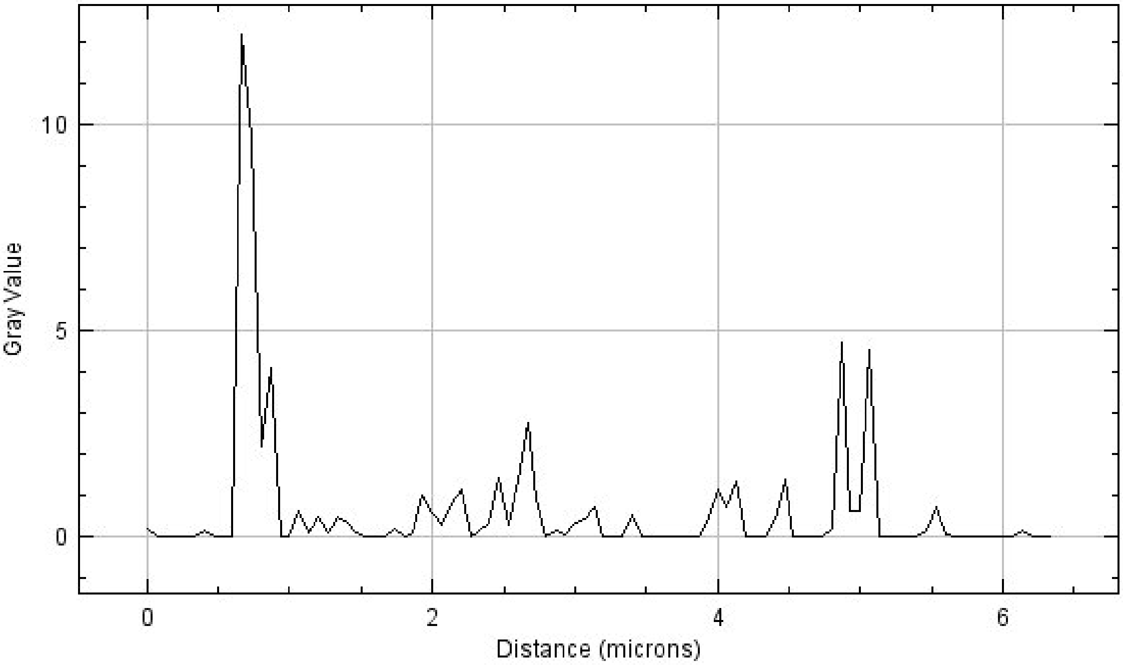

Figure S678 – Intensity profile of line scan generated for cell 9, Figure S669.

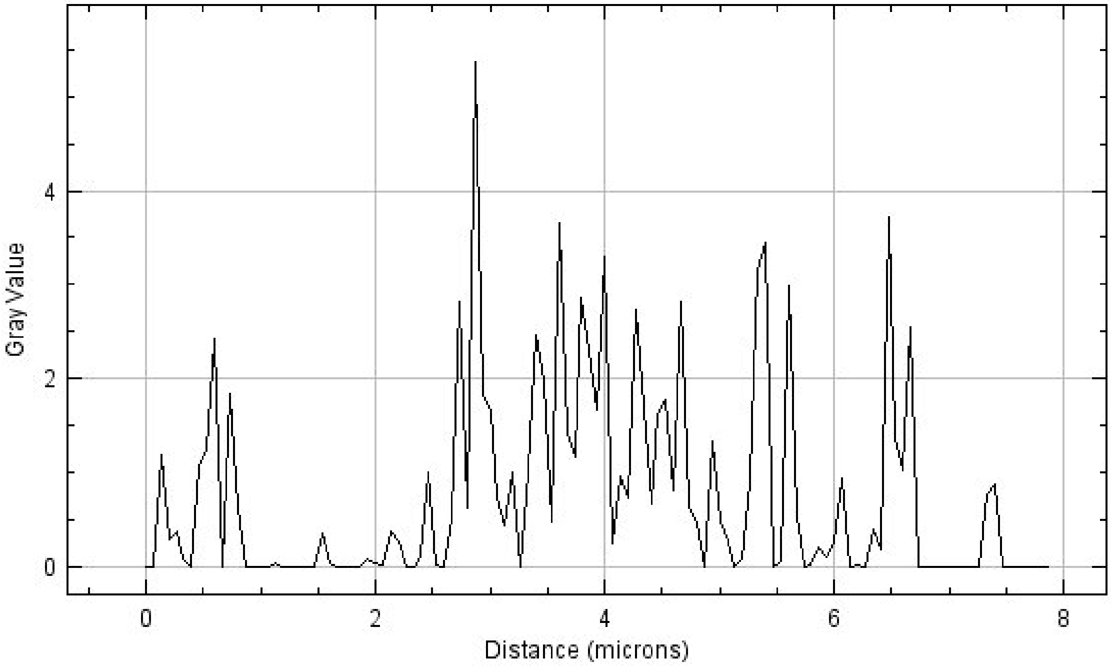

Figure S679 – Intensity profile of line scan generated for cell 10, Figure S669.

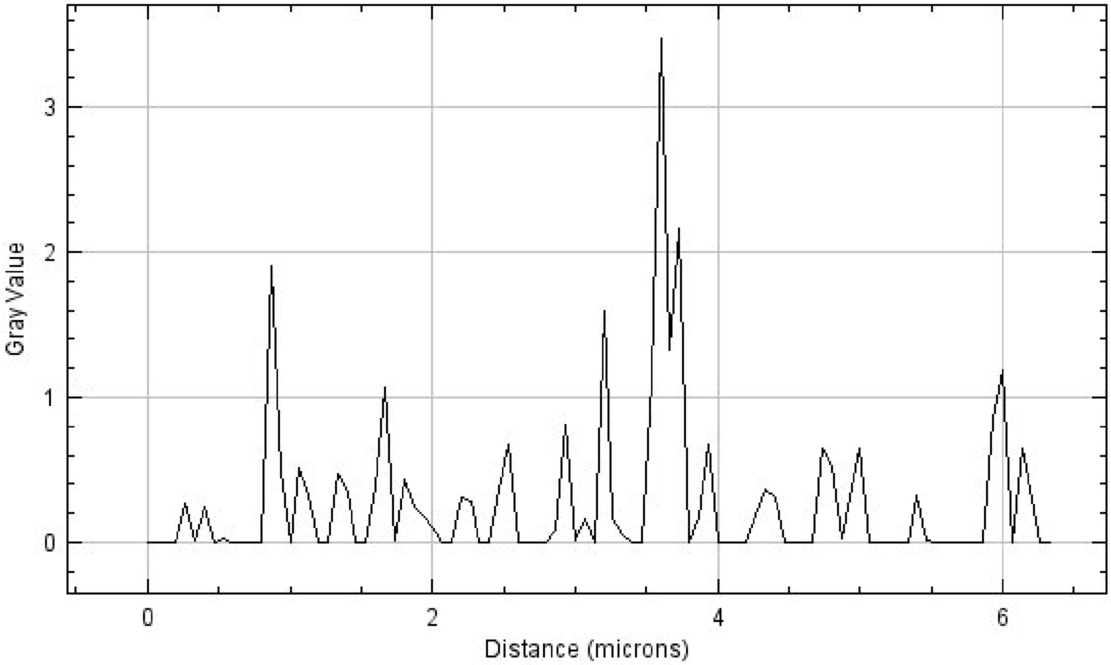

Figure S680 – Intensity profile of line scan generated for cell 11, Figure S669.

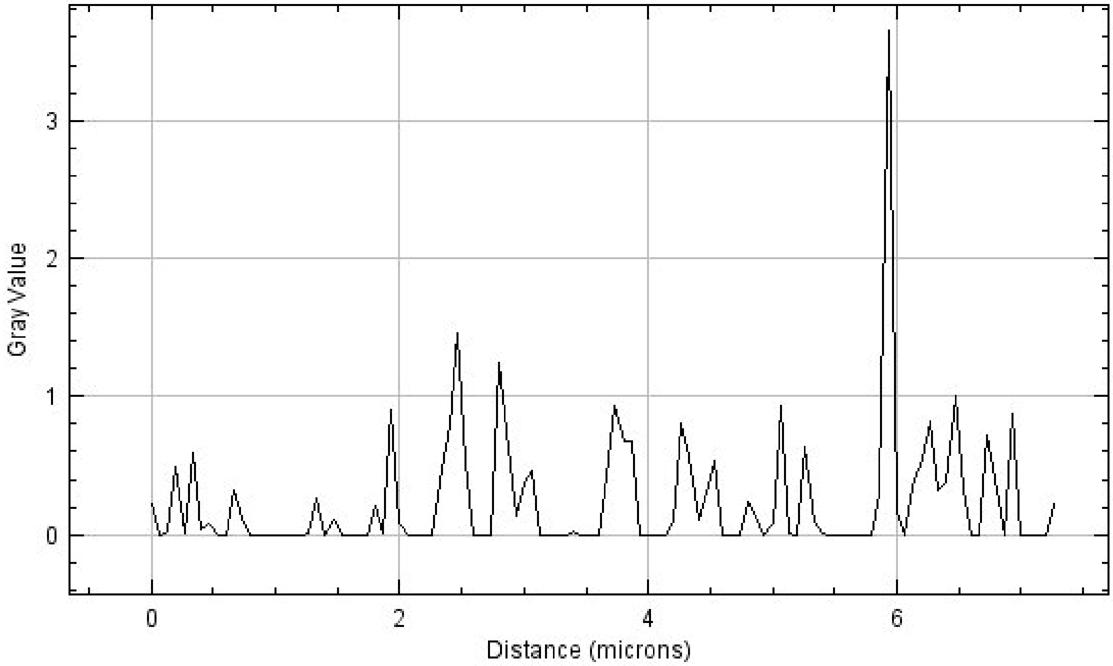

Figure S681 – Intensity profile of line scan generated for cell 12, Figure S669.

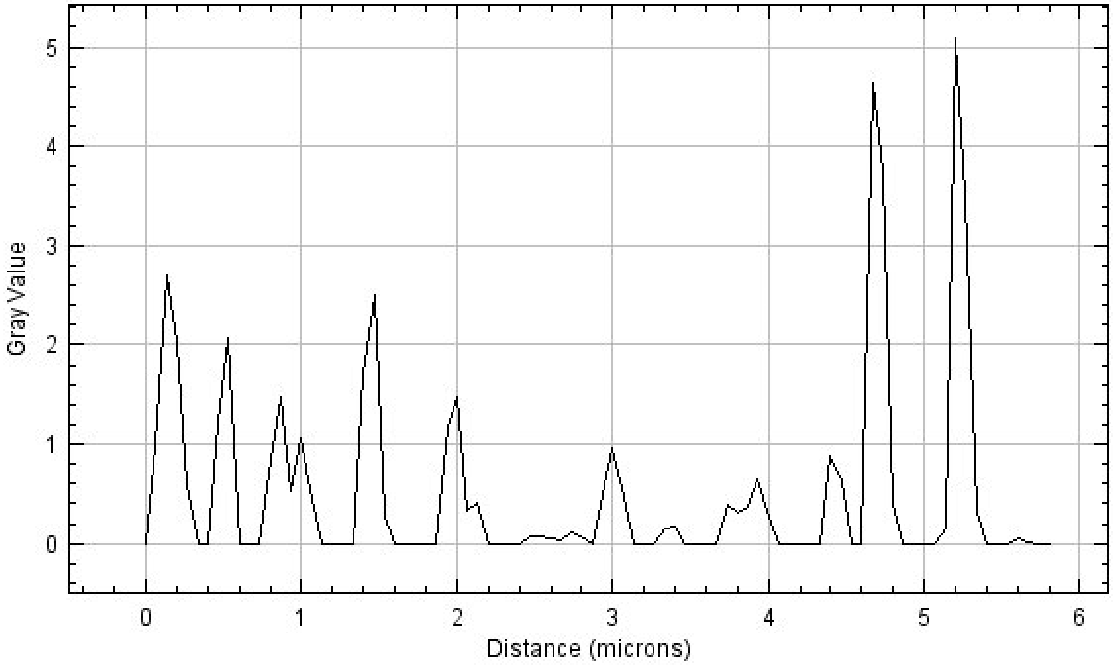

Figure S682 – Intensity profile of line scan generated for cell 13, Figure S669.

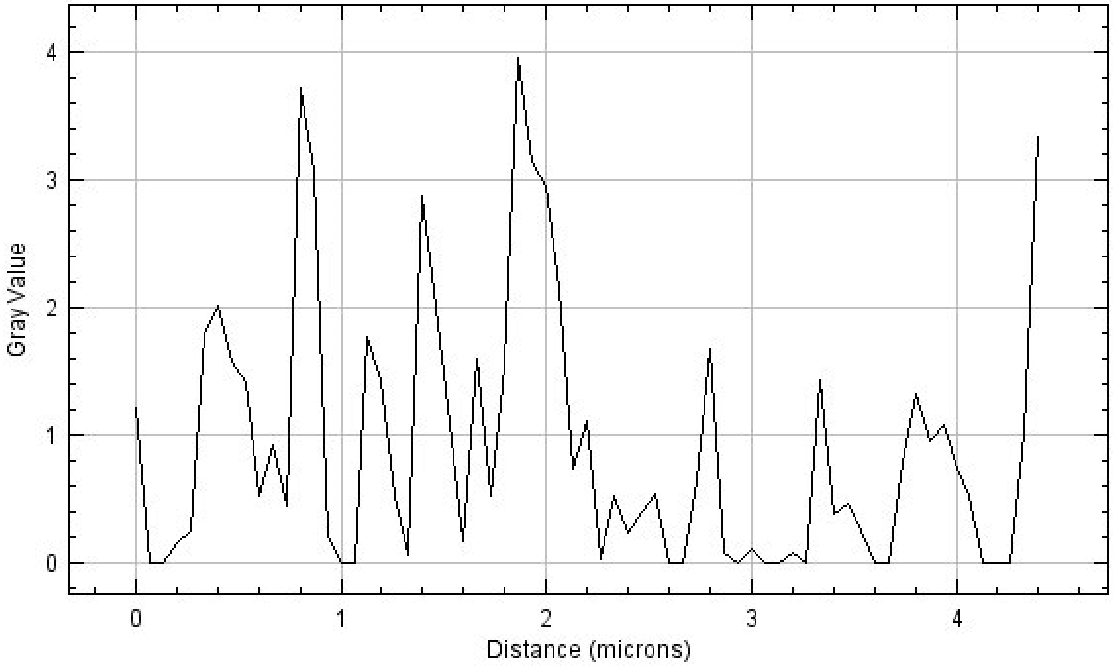

Figure S683 – Intensity profile of line scan generated for cell 14, Figure S669.

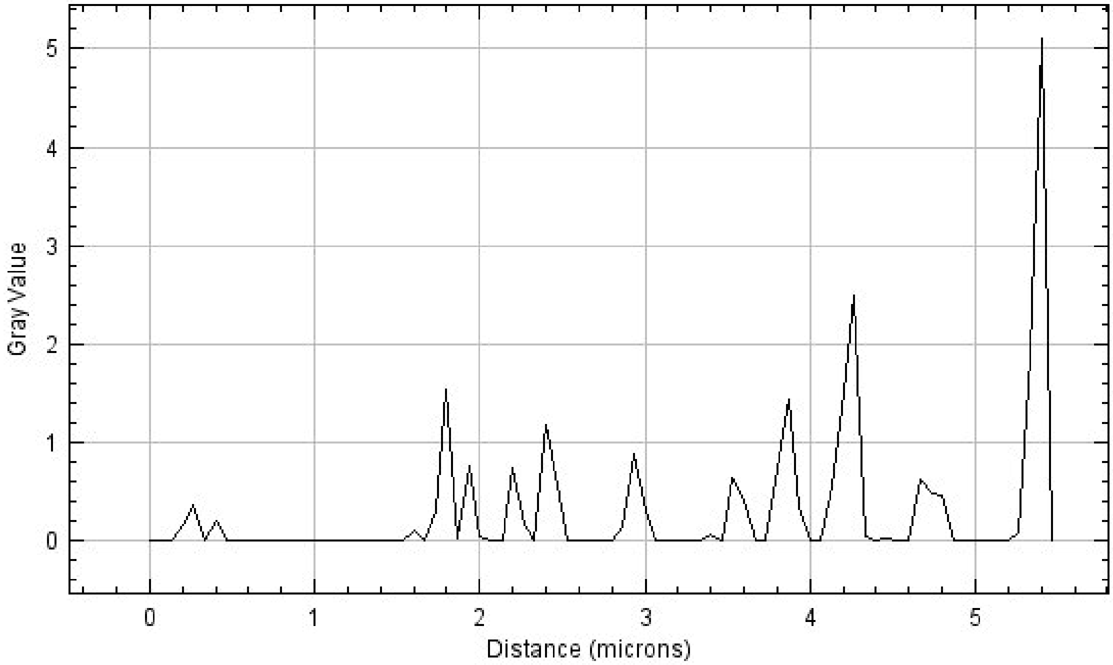

Figure S684 – Intensity profile of line scan generated for cell 15, Figure S669.

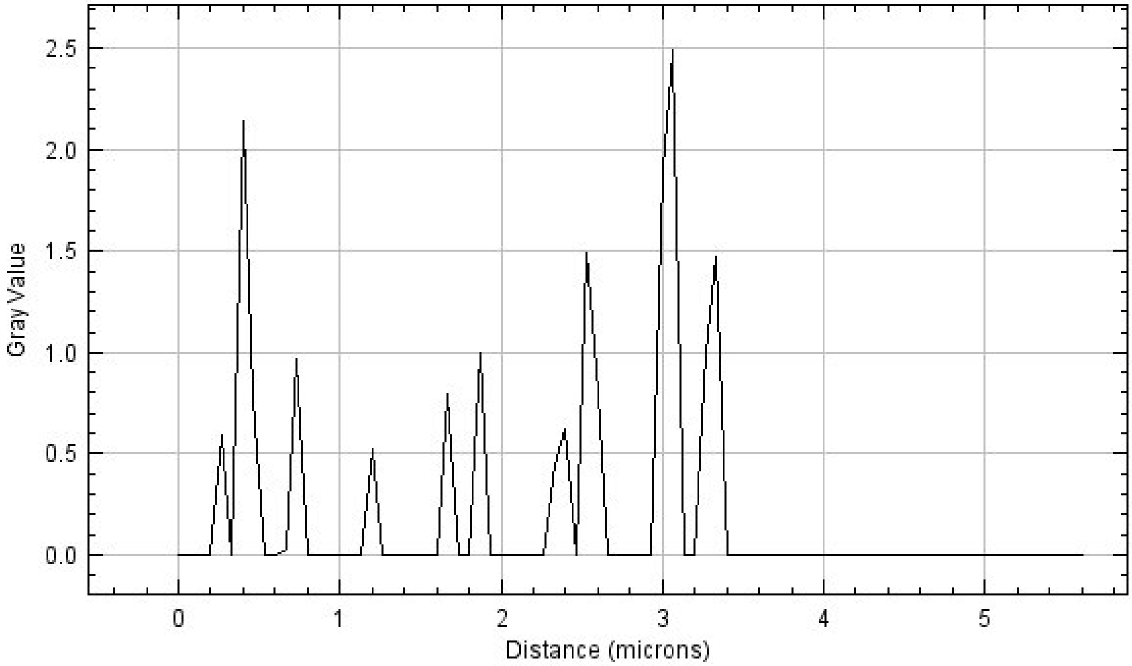

Figure S685 – Intensity profile of line scan generated for cell 16, Figure S669.

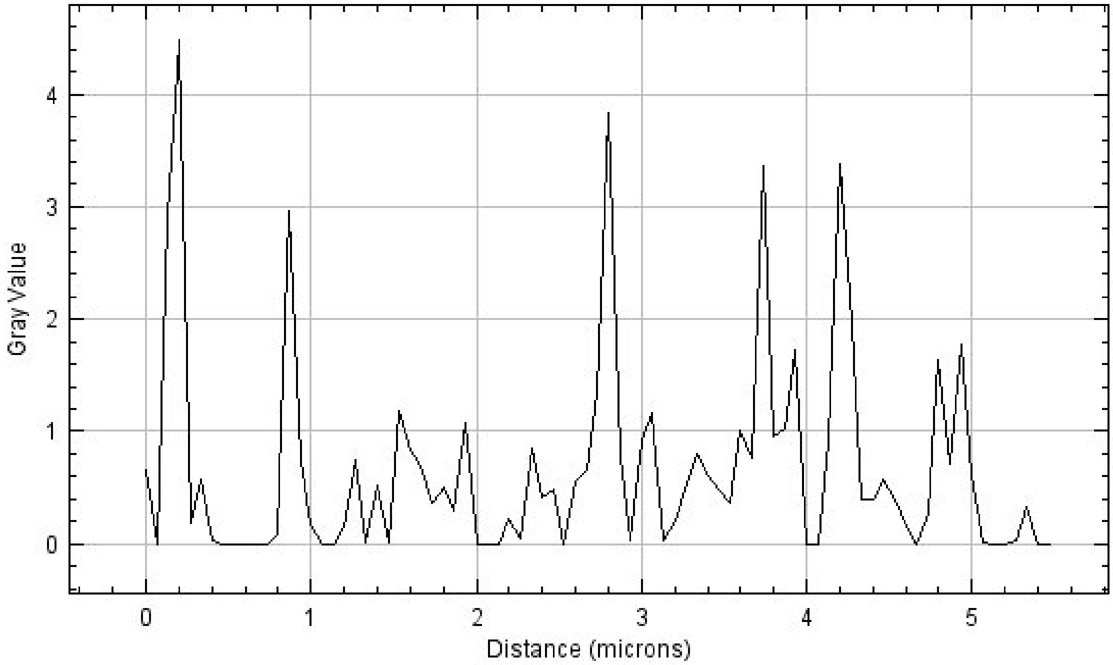

Figure S686 – Intensity profile of line scan generated for cell 17, Figure S669.

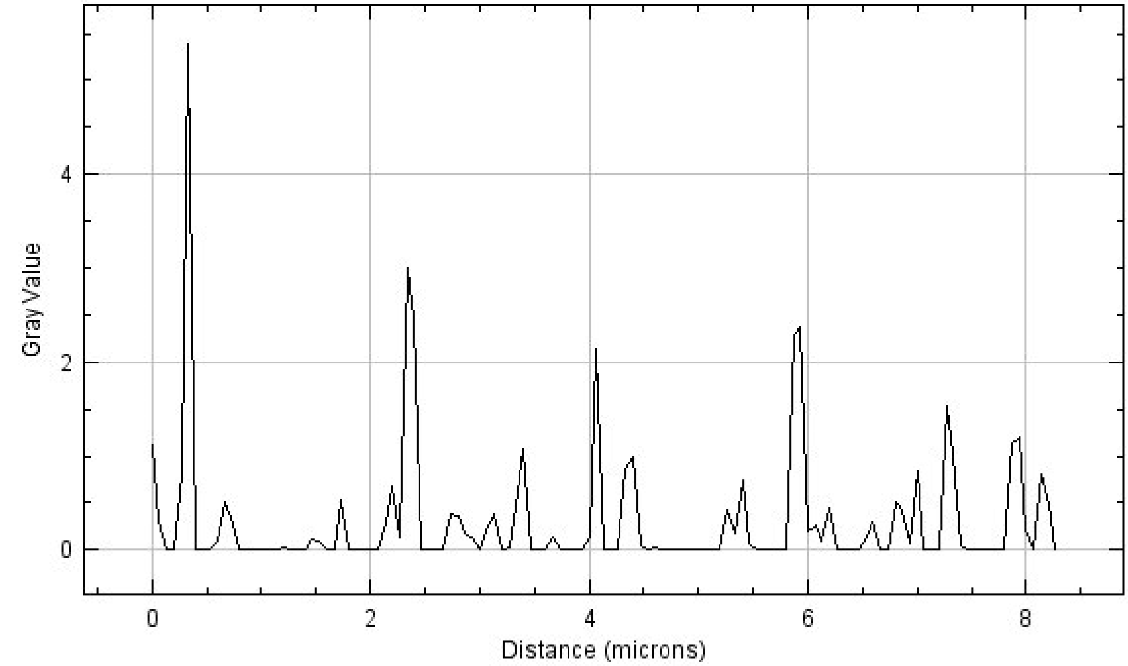

Figure S687 – Intensity profile of line scan generated for cell 18, Figure S669.

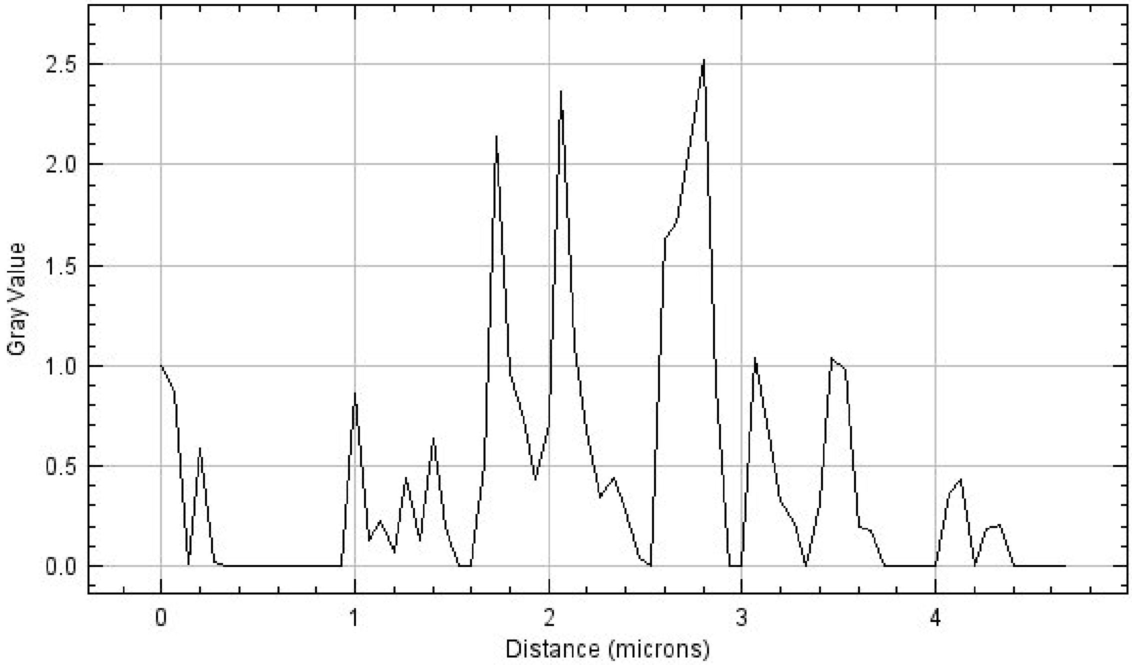

Figure S688 – Intensity profile of line scan generated for cell 19, Figure S669.

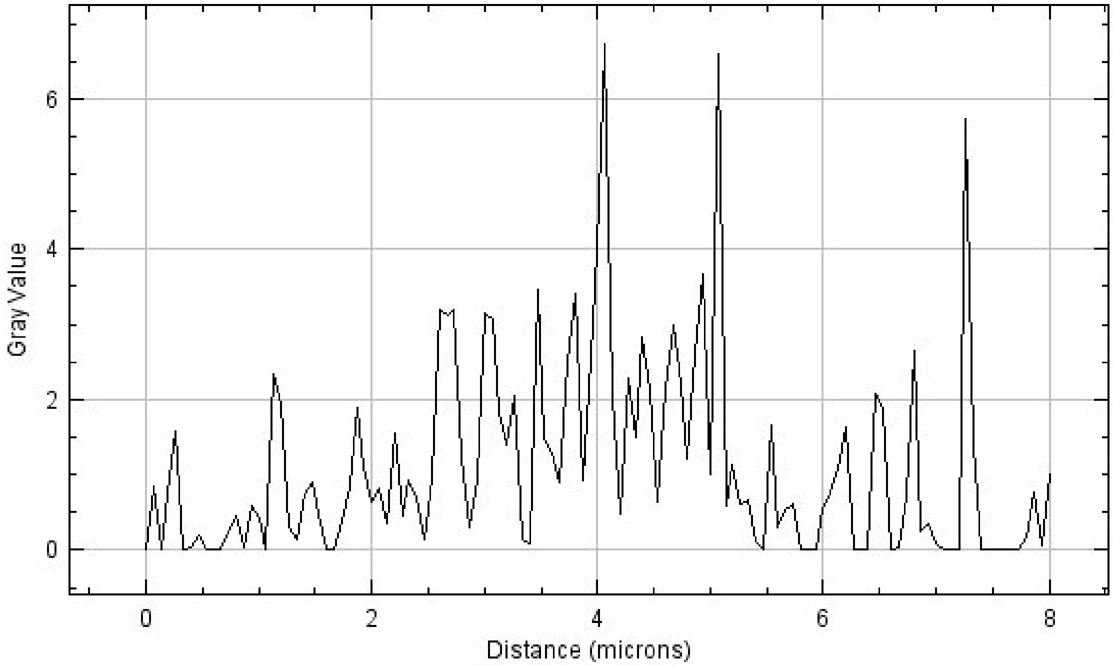

Figure S689 – Intensity profile of line scan generated for cell 20, Figure S669.

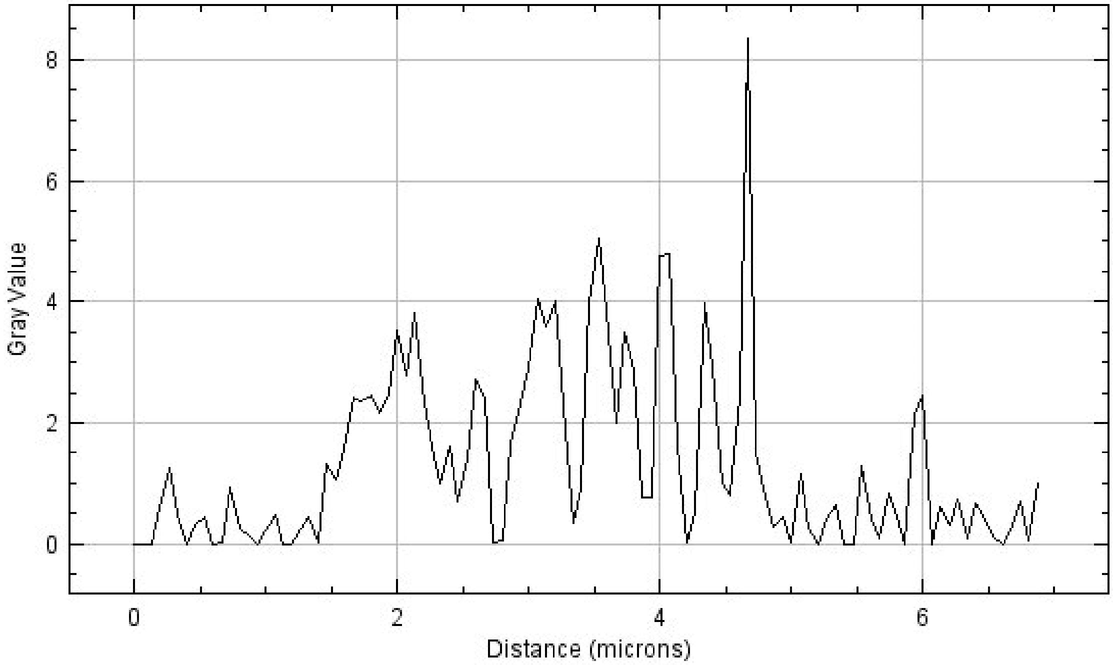

Figure S690 – Intensity profile of line scan generated for cell 21, Figure S669.

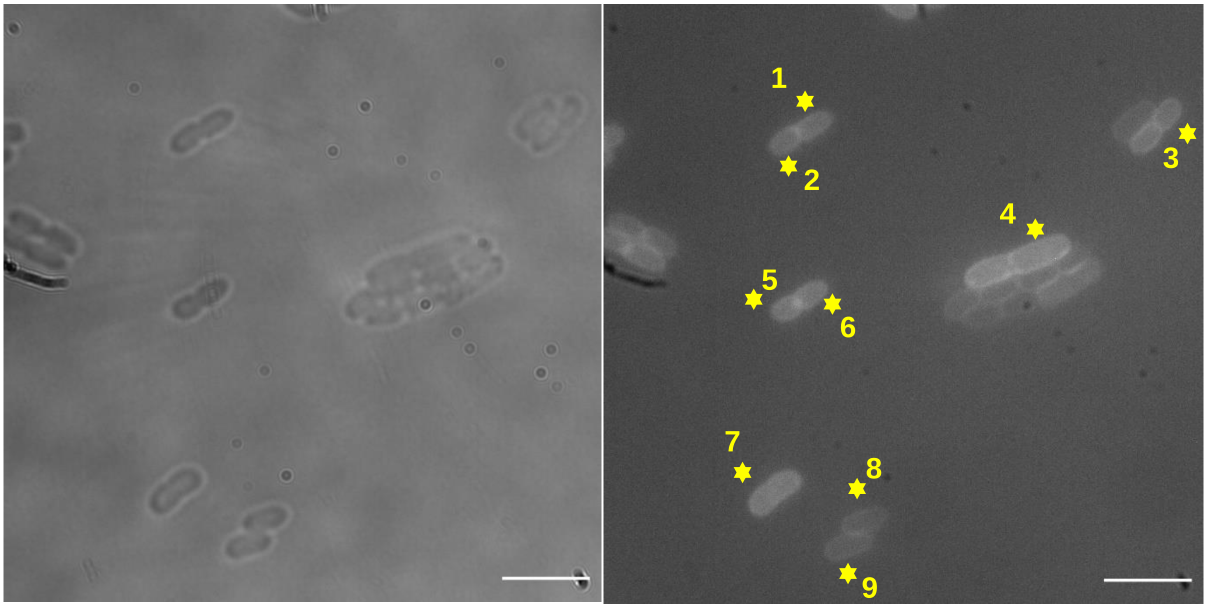

Figure S691 – *E. coli* transmitted and DAPI images used in fluorescence intensity calculations in the presence of compound **39** at T = 30 minutes. Scale bar = 10 µM.

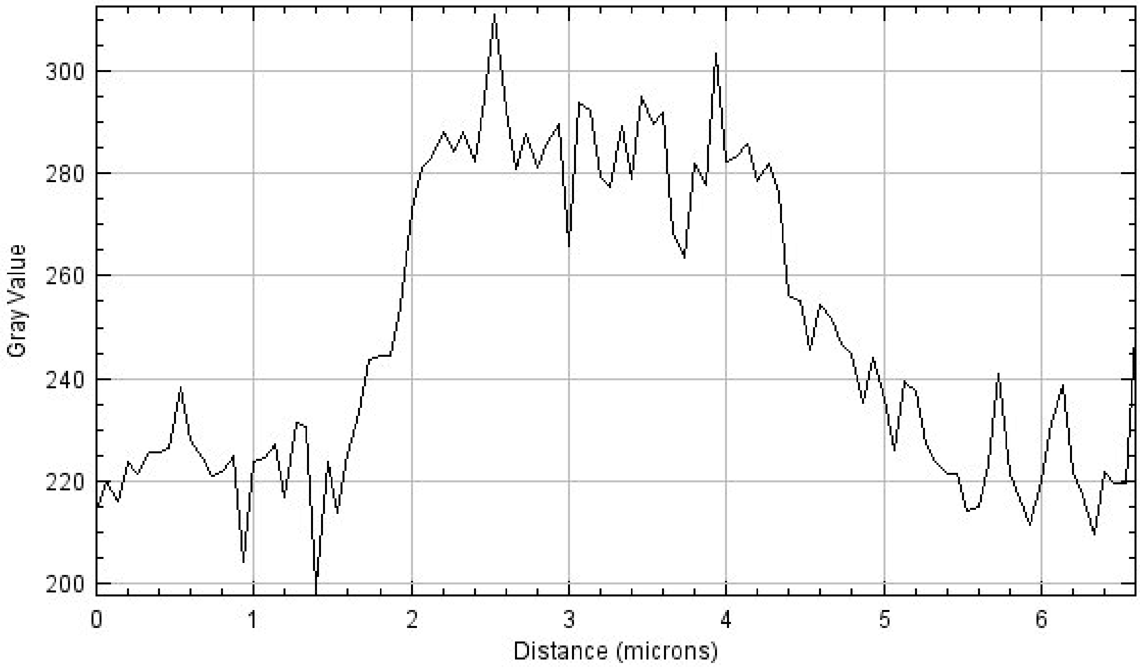

Figure S692 – Intensity profile of line scan generated for cell 1, Figure S691.

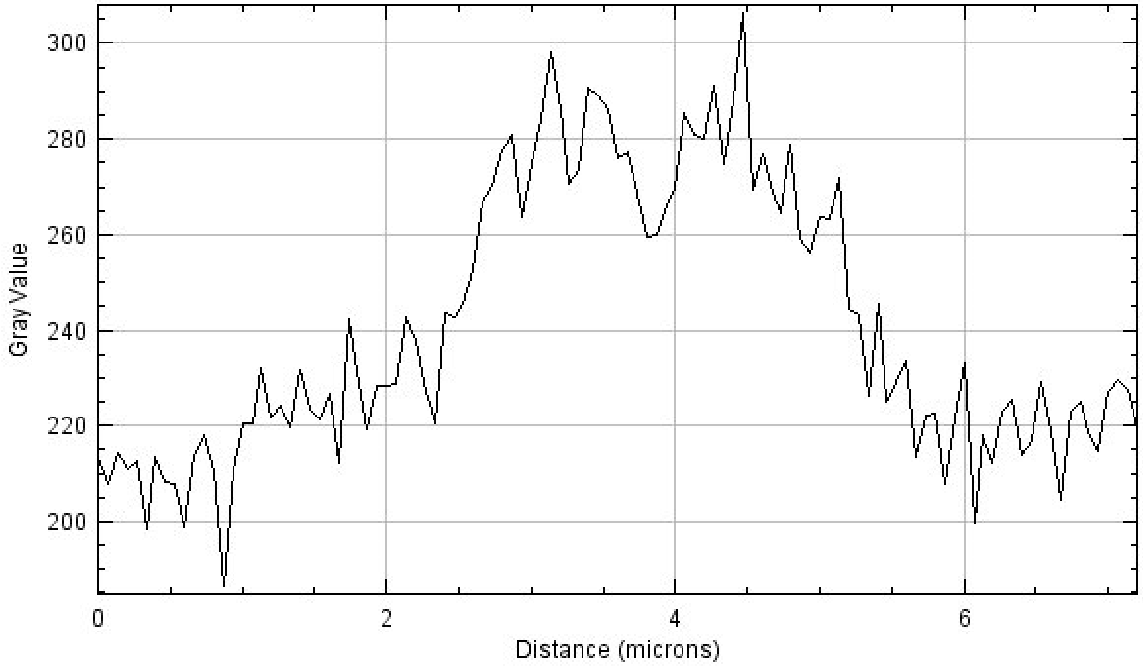

Figure S693 – Intensity profile of line scan generated for cell 2, Figure S691.

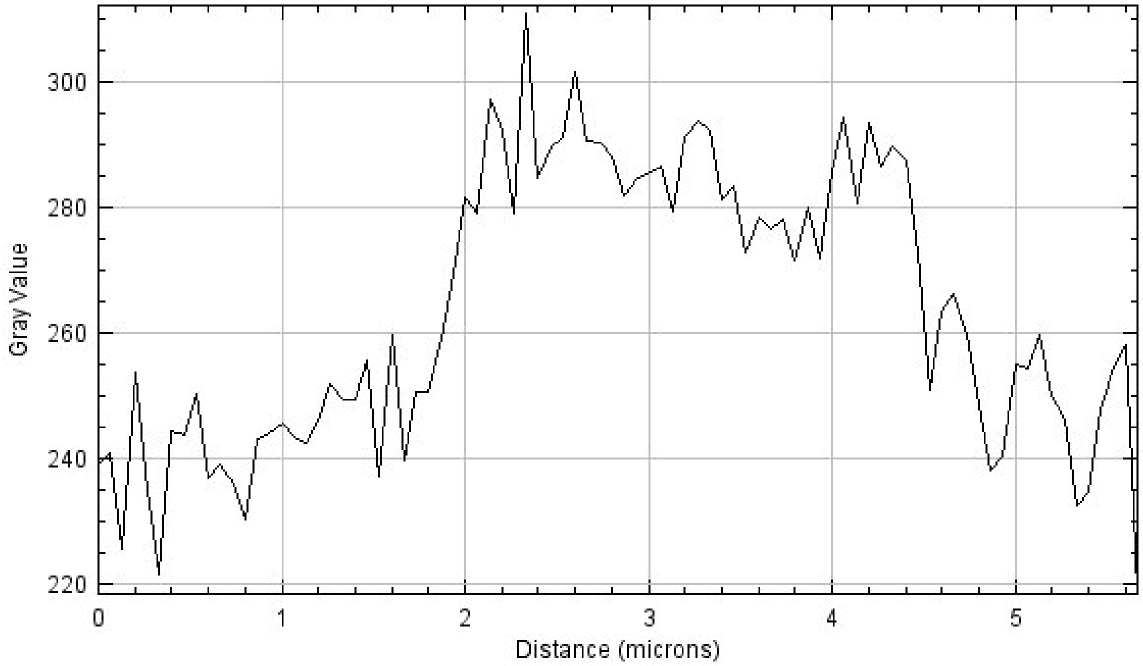

Figure S694 – Intensity profile of line scan generated for cell 3, Figure S691.

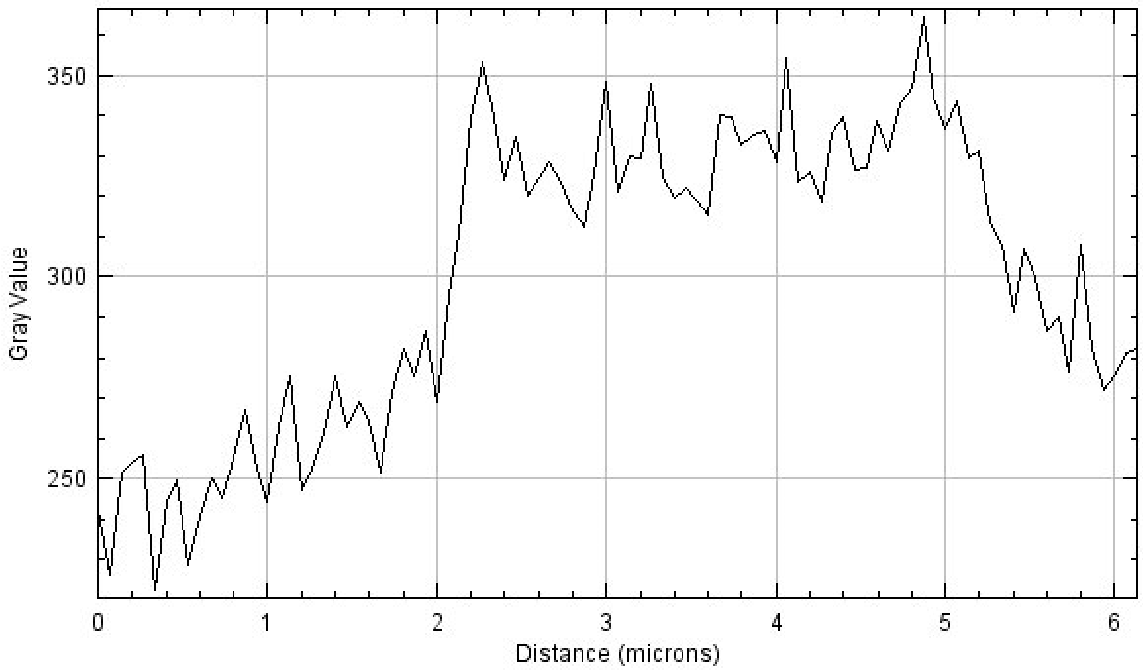

Figure S695 – Intensity profile of line scan generated for cell 4, Figure S691.

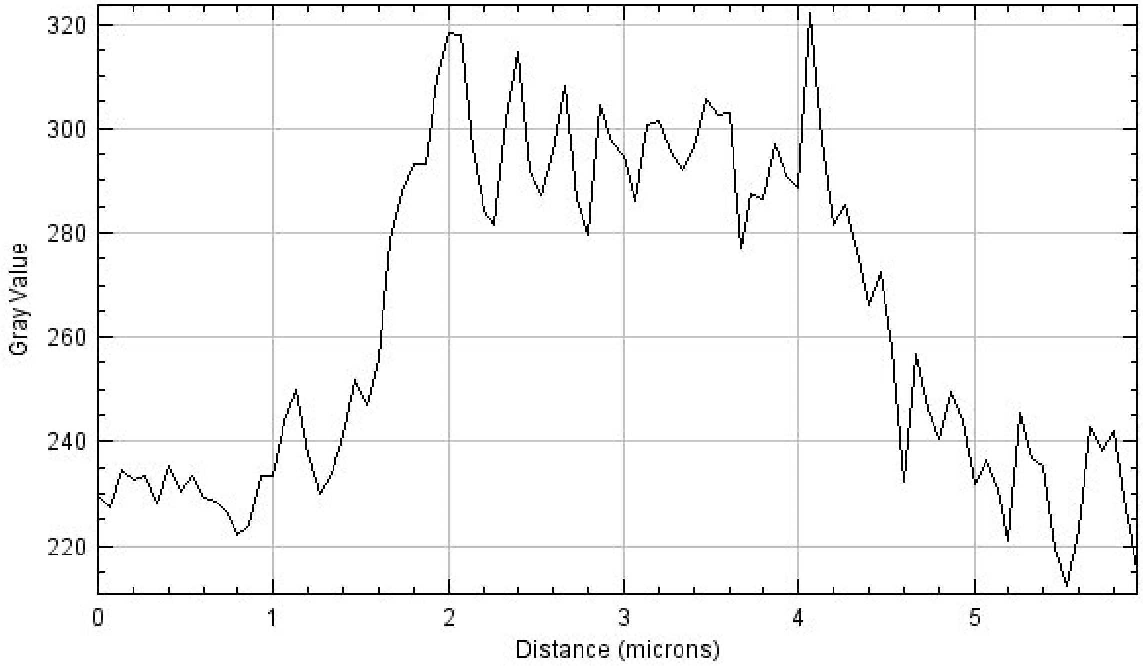

Figure S696 – Intensity profile of line scan generated for cell 5, Figure S691.

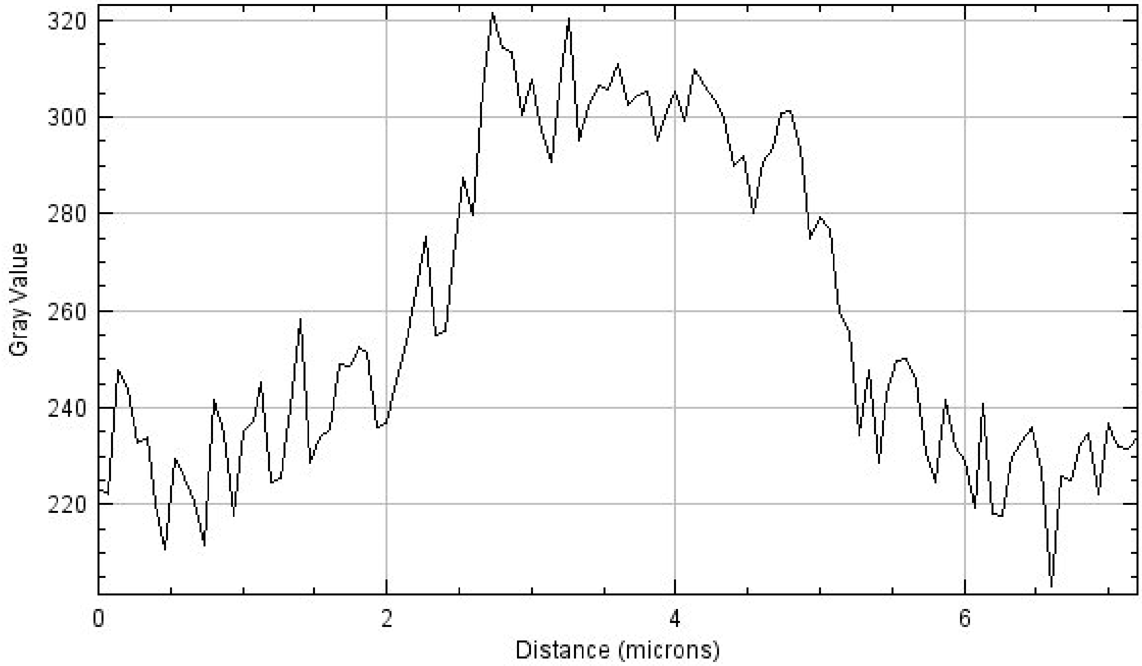

Figure S697 – Intensity profile of line scan generated for cell 6, Figure S691.

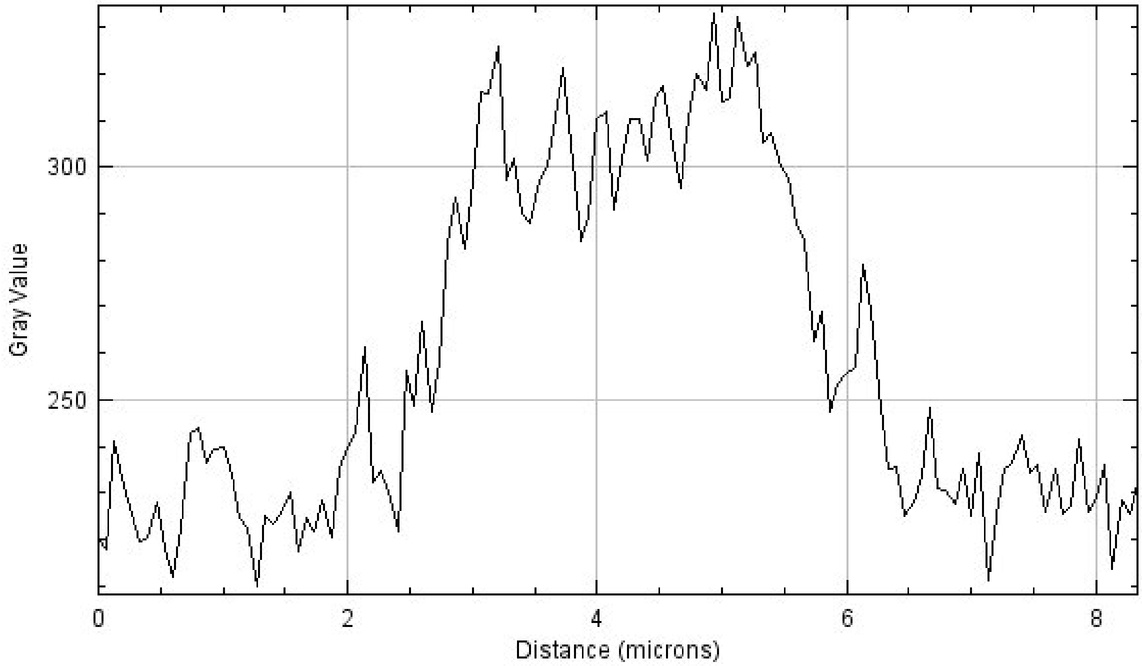

Figure S698 – Intensity profile of line scan generated for cell 7, Figure S691.

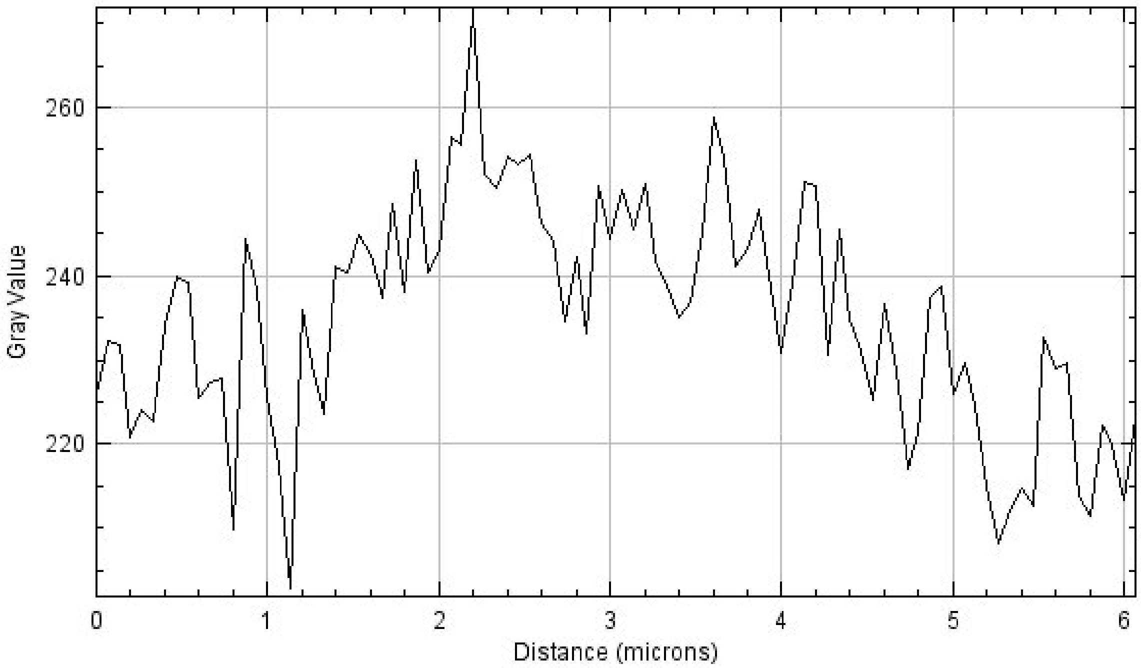

Figure S699 – Intensity profile of line scan generated for cell 8, Figure S691.

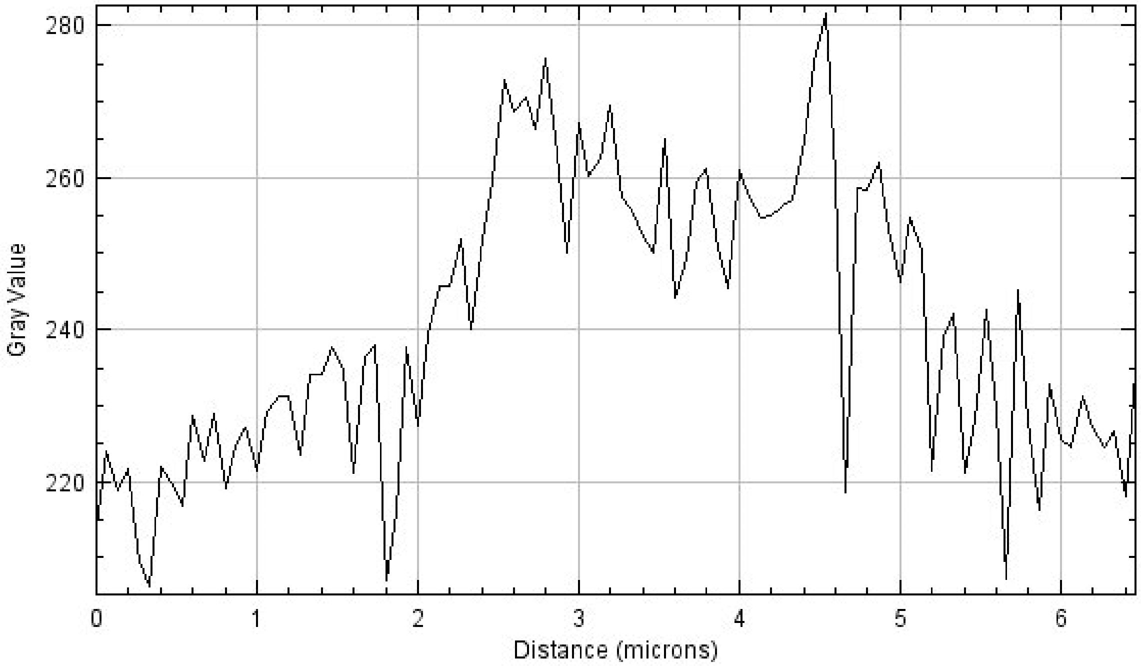

Figure S700 – Intensity profile of line scan generated for cell 9, Figure S691.

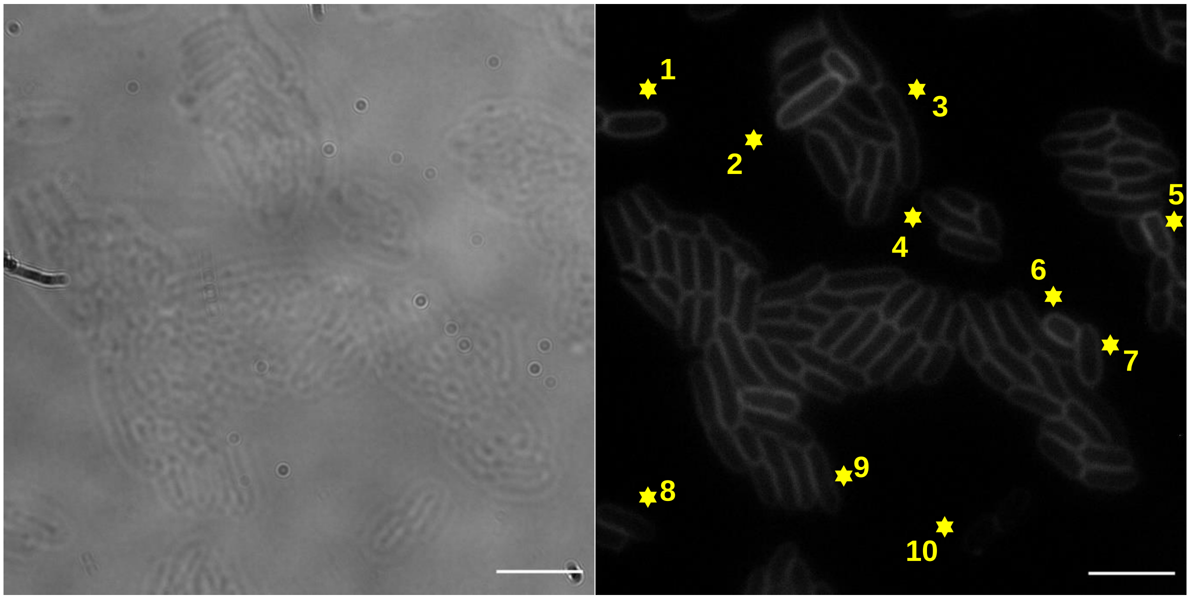

Figure S701 – *E. coli* transmitted and mCherry Images used in fluorescence intensity calculations in the presence of FM4-64 at T = 30 minutes. Scale bar = 10 µM.

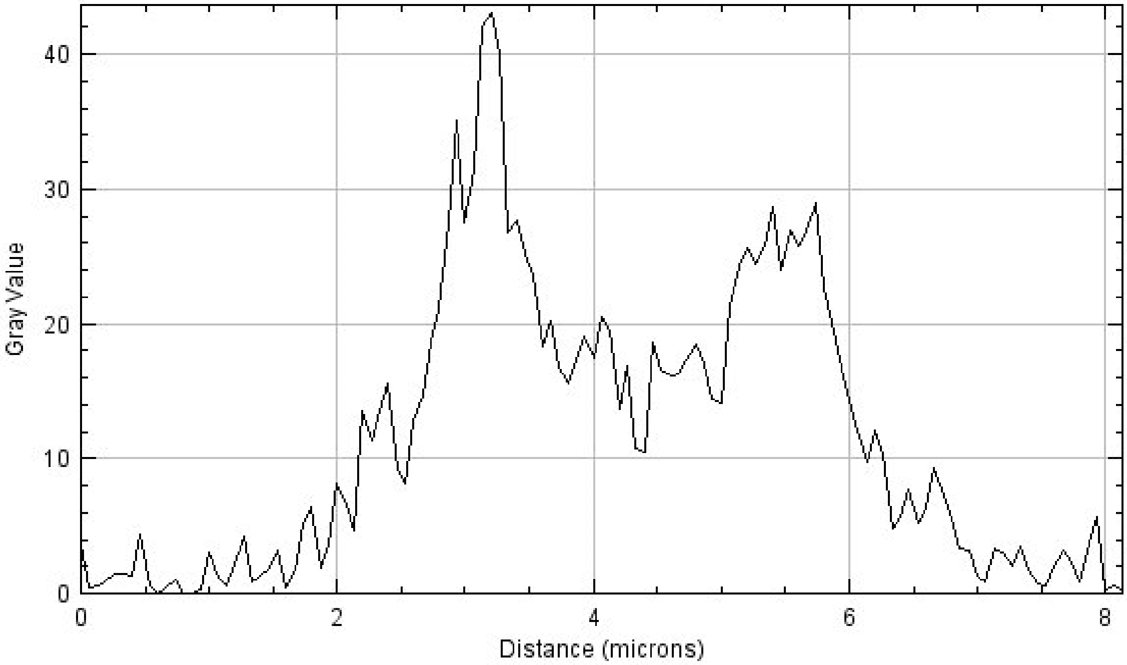

Figure S702 – Intensity profile of line scan generated for cell 1, Figure S701.

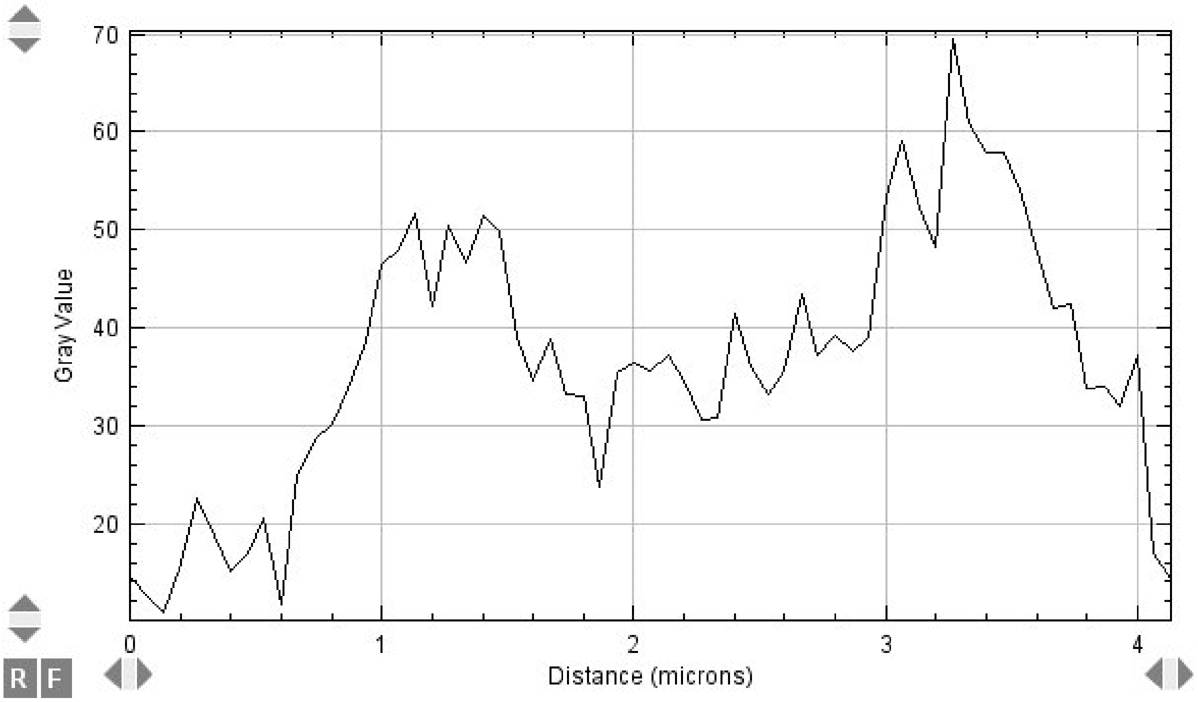

Figure S703 – Intensity profile of line scan generated for cell 2, Figure S701.

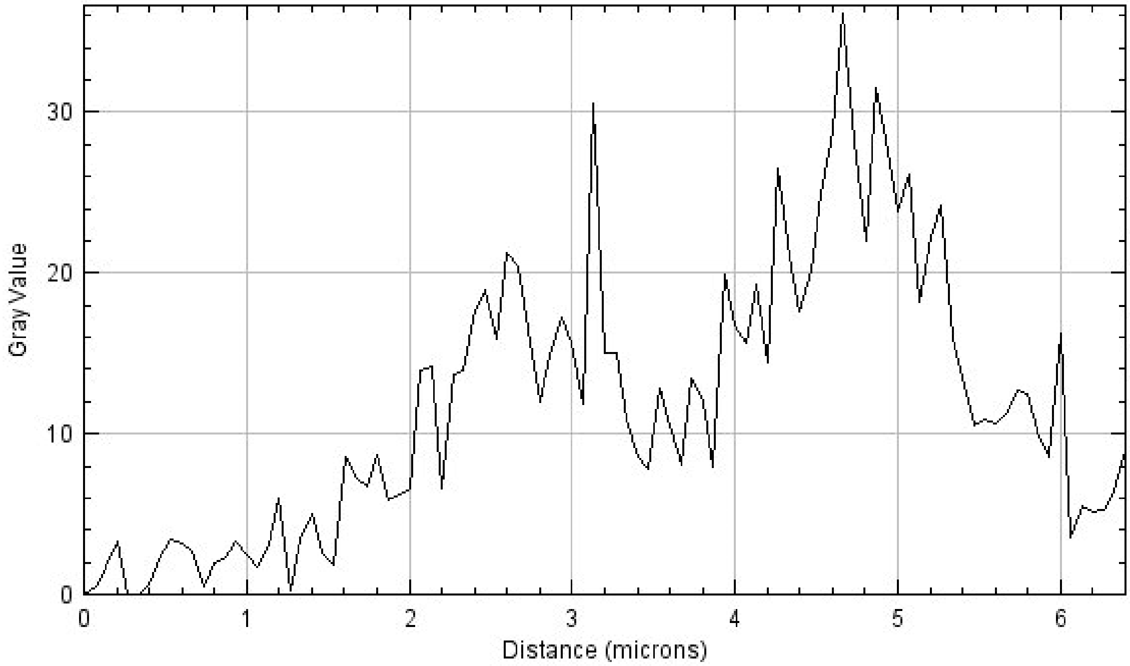

Figure S704 – Intensity profile of line scan generated for cell 3, Figure S701.

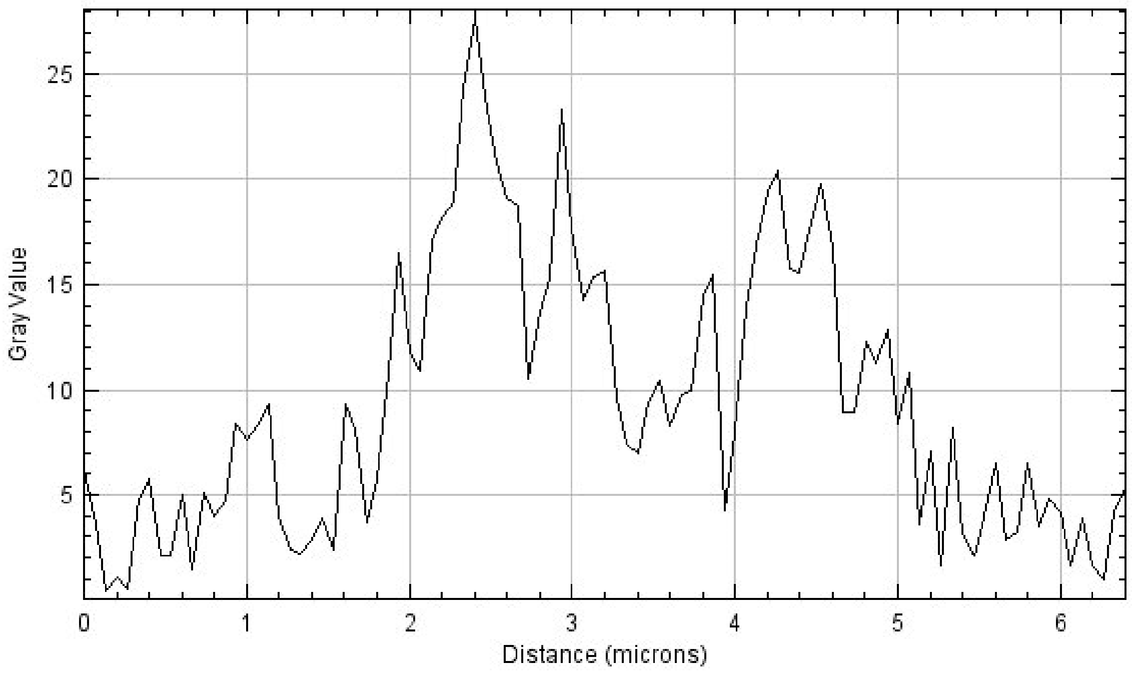

Figure S705 – Intensity profile of line scan generated for cell 4, Figure S701.

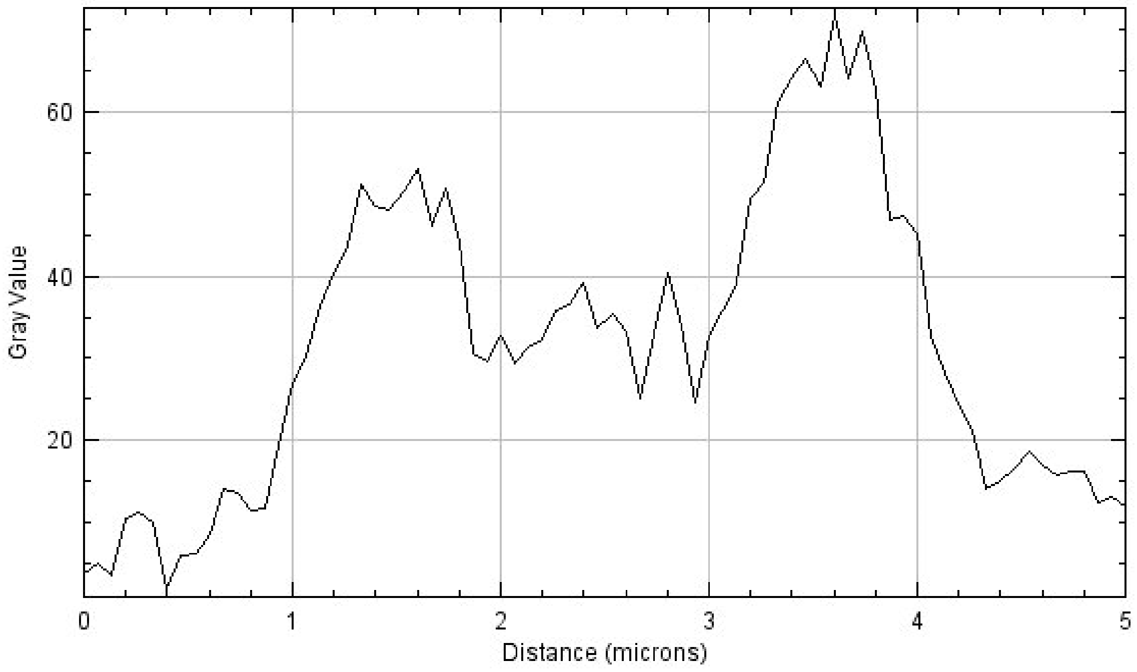

Figure S706 – Intensity profile of line scan generated for cell 5, Figure S701.

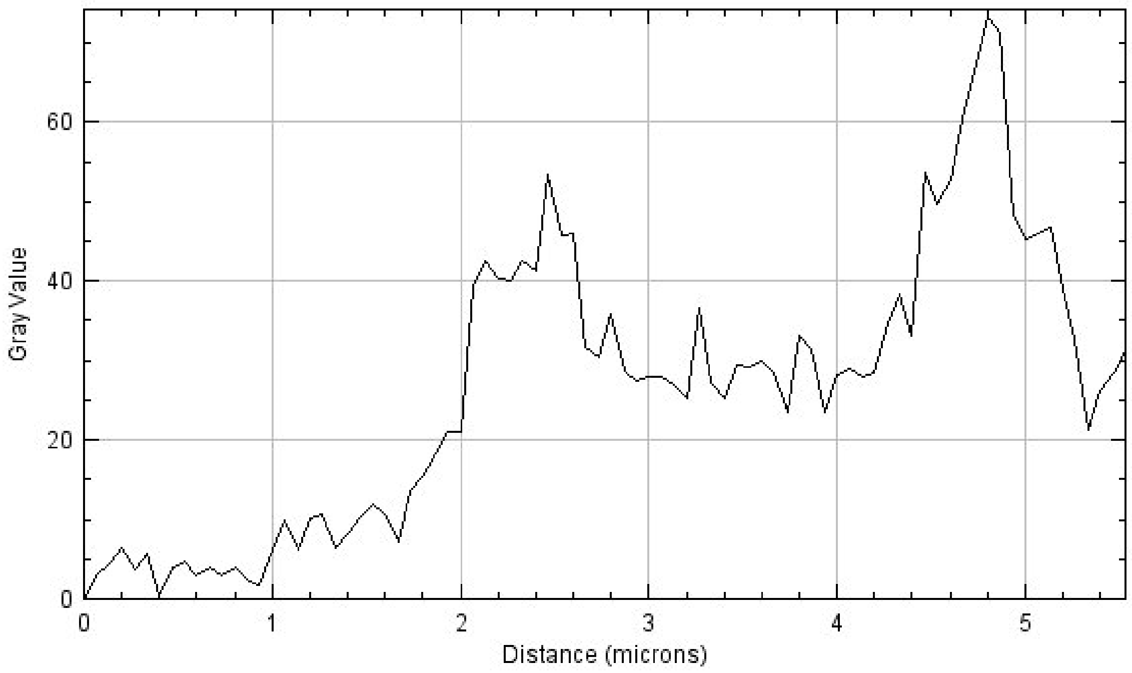

Figure S707 – Intensity profile of line scan generated for cell 6, Figure S701.

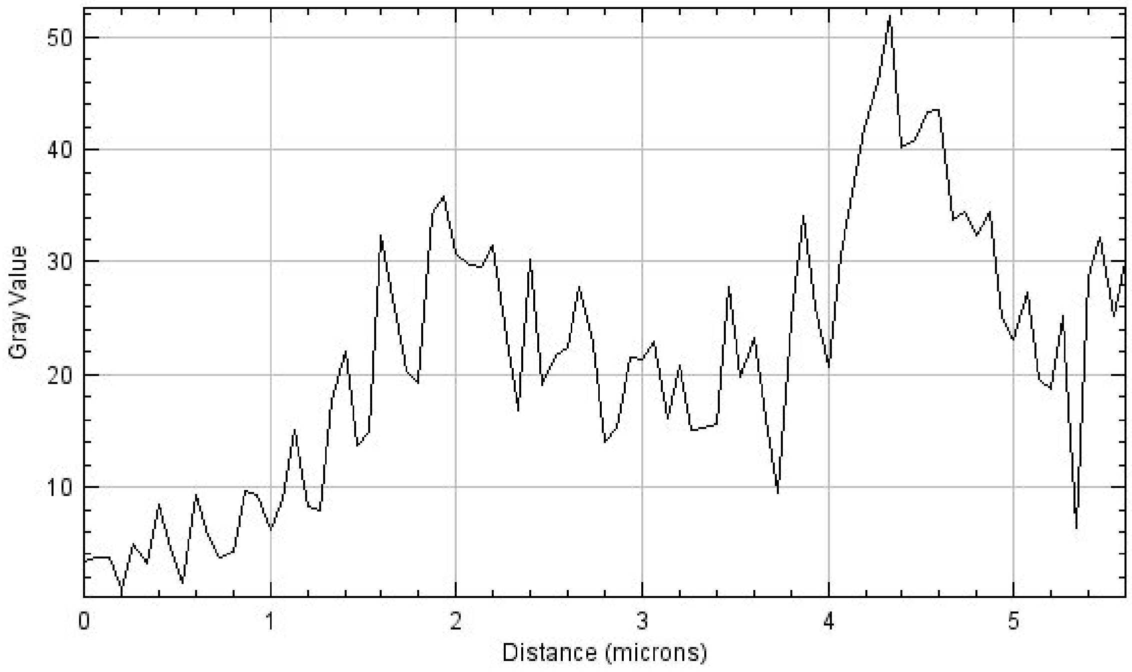

Figure S708 – Intensity profile of line scan generated for cell 7, Figure S701.

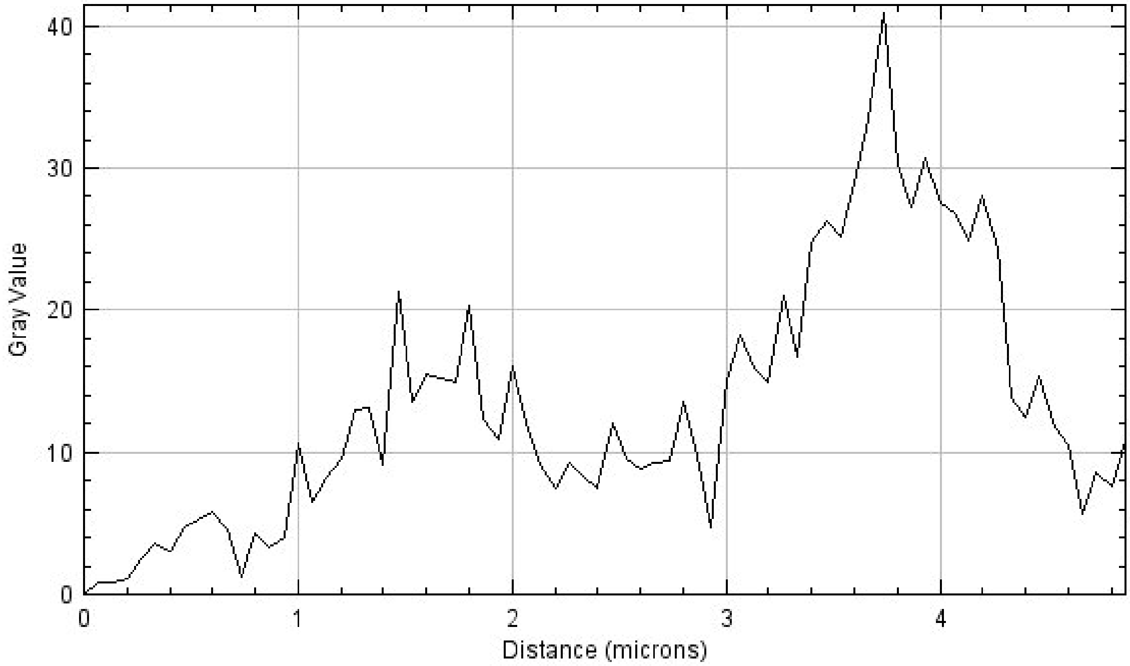

Figure S709 – Intensity profile of line scan generated for cell 8, Figure S701.

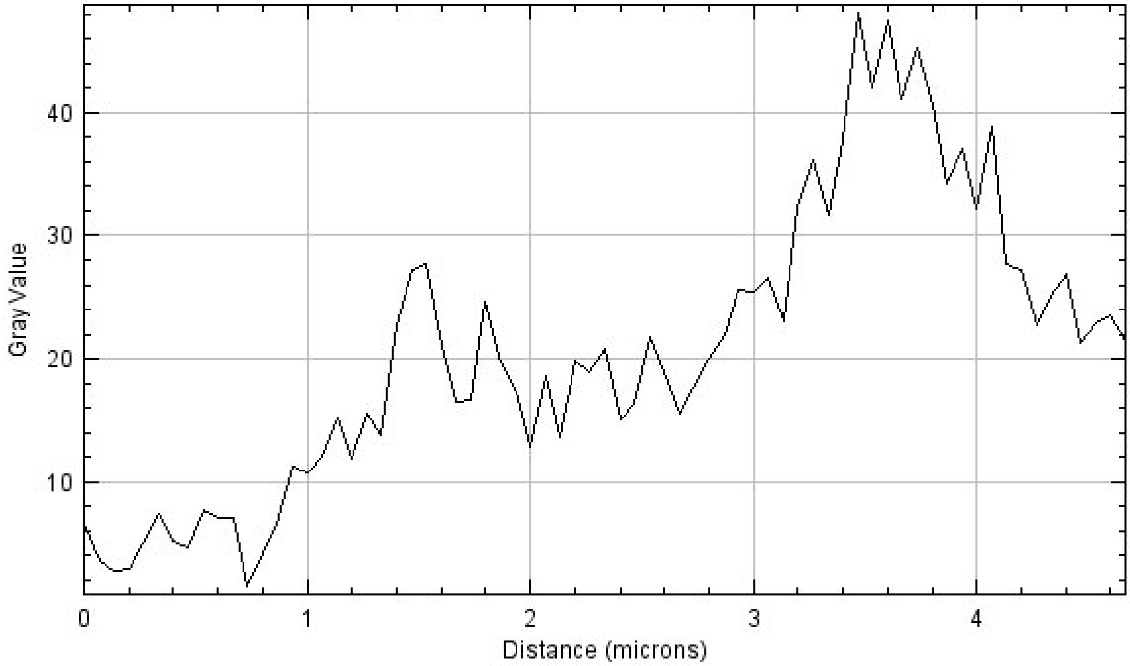

Figure S710 – Intensity profile of line scan generated for cell 9, Figure S701.

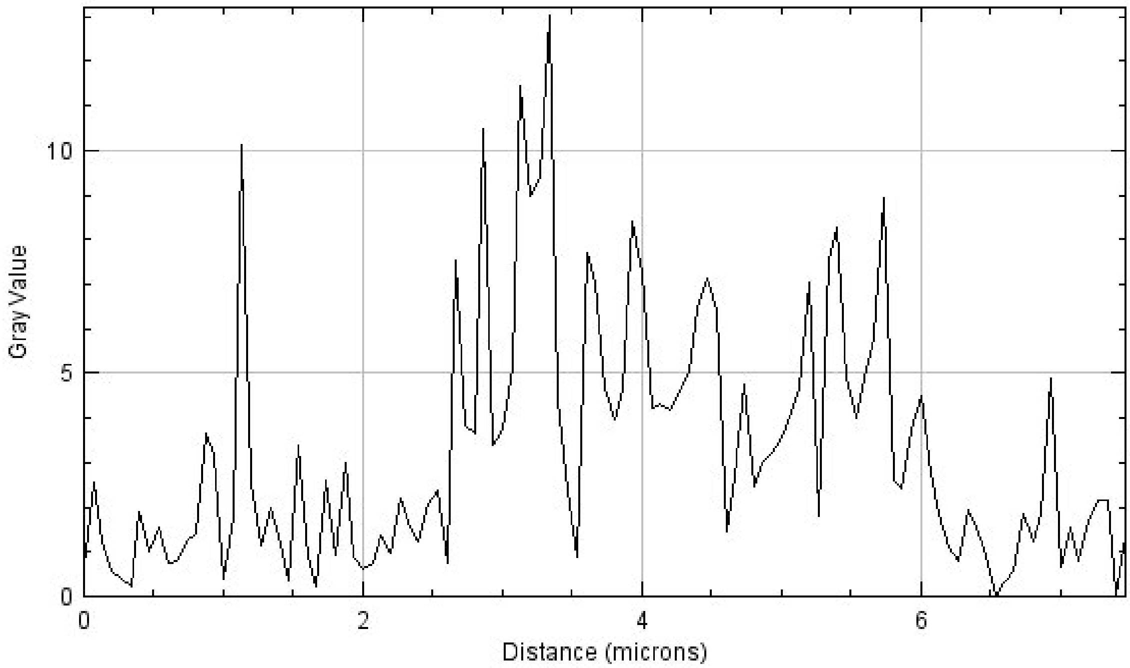

Figure S711 – Intensity profile of line scan generated for cell 10, Figure S701.

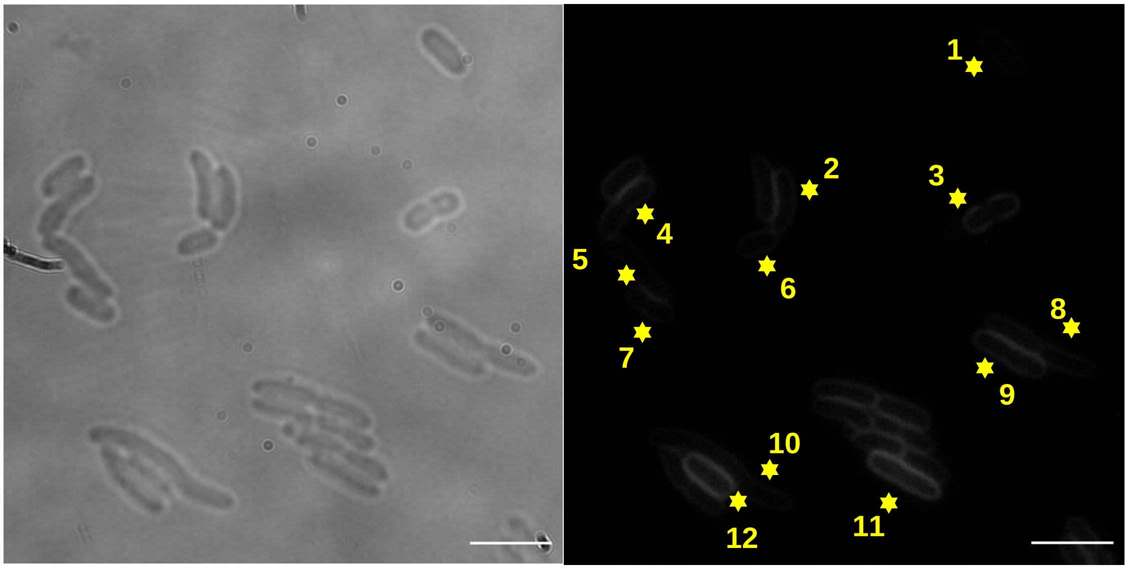

Figure S712 – *E. coli* transmitted and mCherry Images used in fluorescence intensity calculations in the presence of FM4-64 at T = 30 minutes. Scale bar = 10 µM.

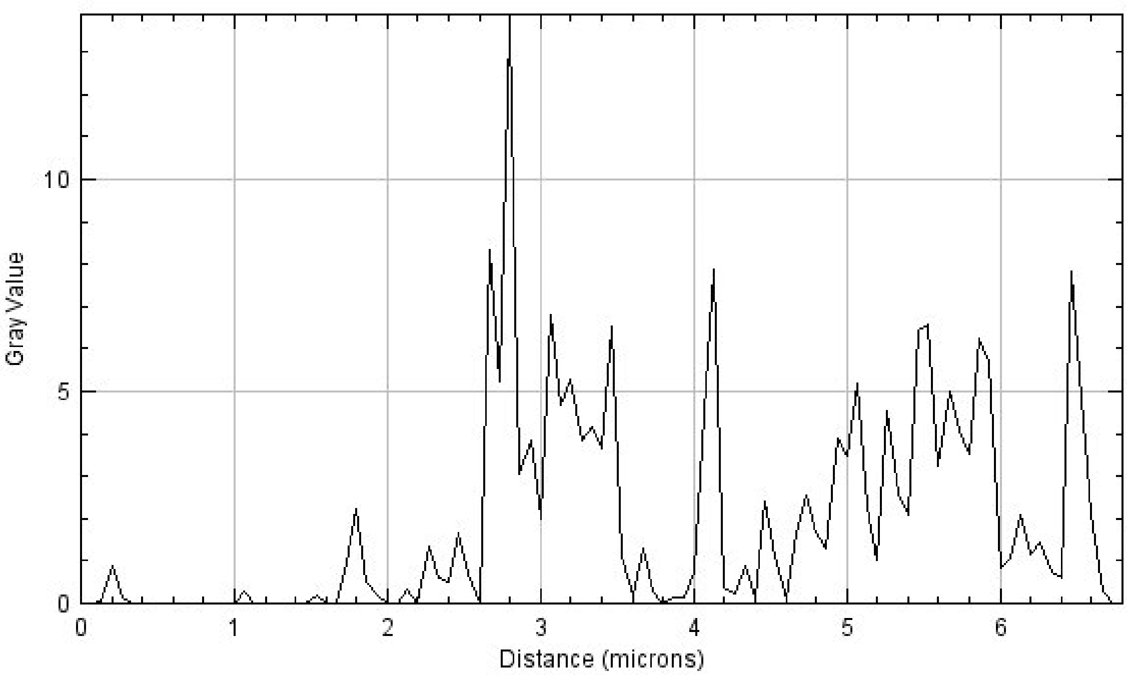

Figure S713 – Intensity profile of line scan generated for cell 1, Figure S712.

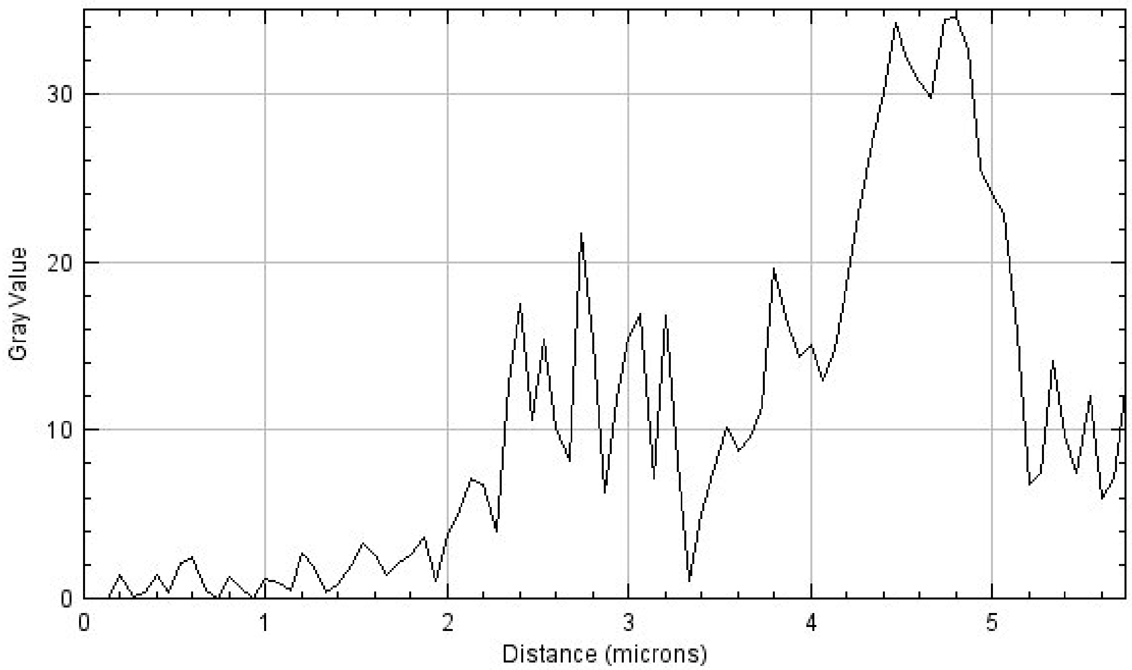

Figure S714 – Intensity profile of line scan generated for cell 2, Figure S712.

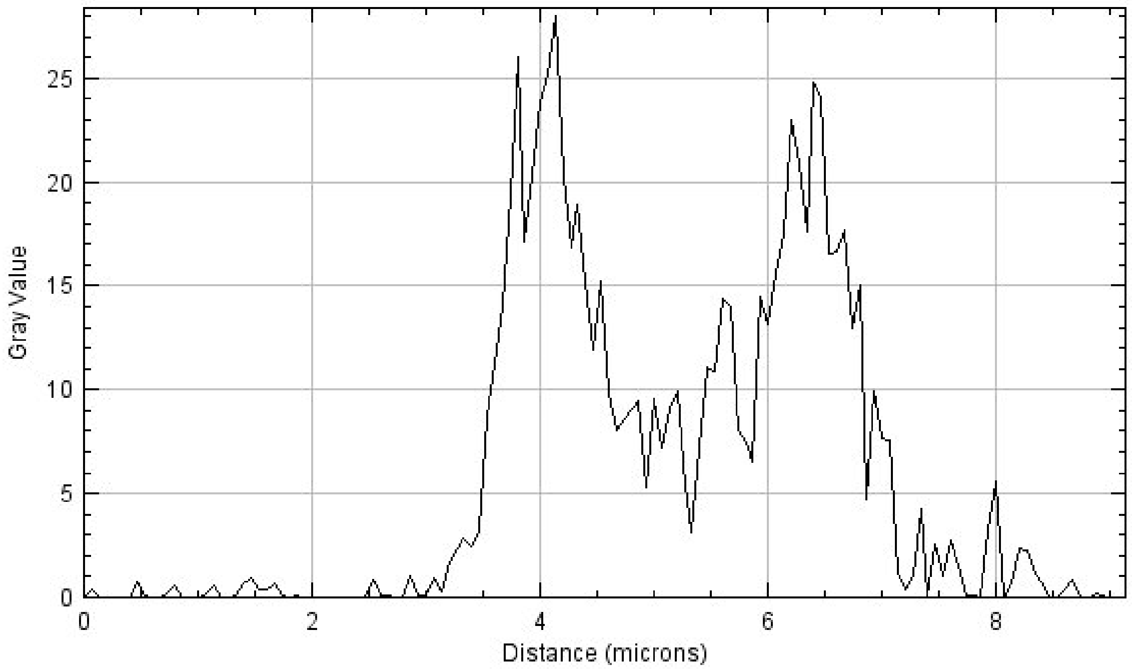

Figure S715 – Intensity profile of line scan generated for cell 3, Figure S712.

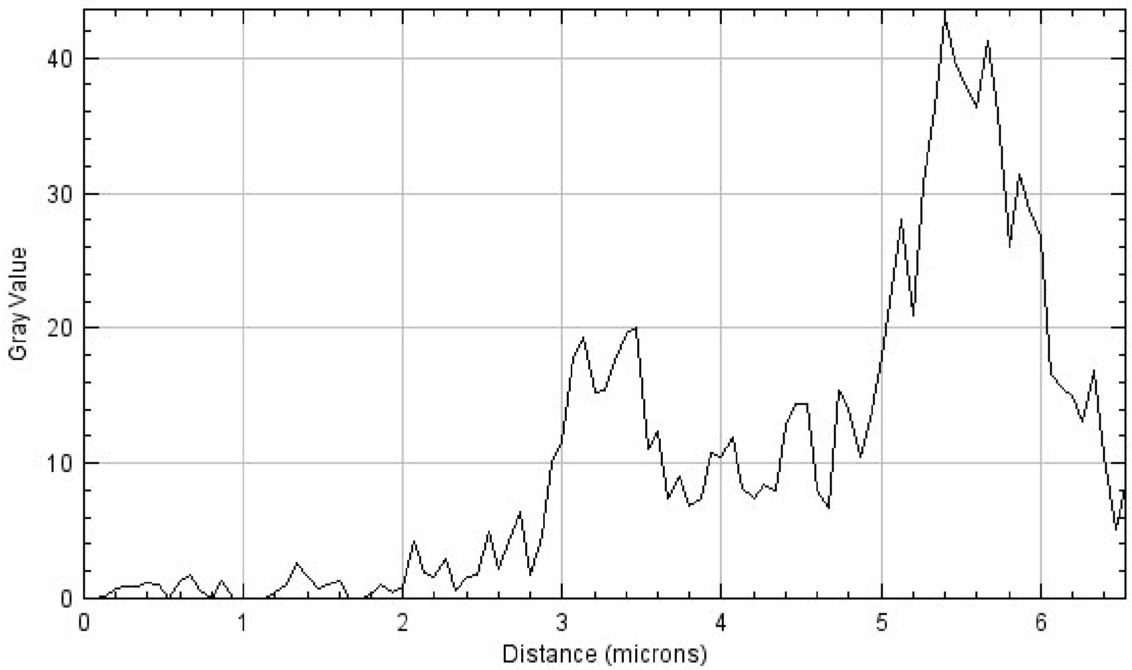

Figure S716 – Intensity profile of line scan generated for cell 4, Figure S712.

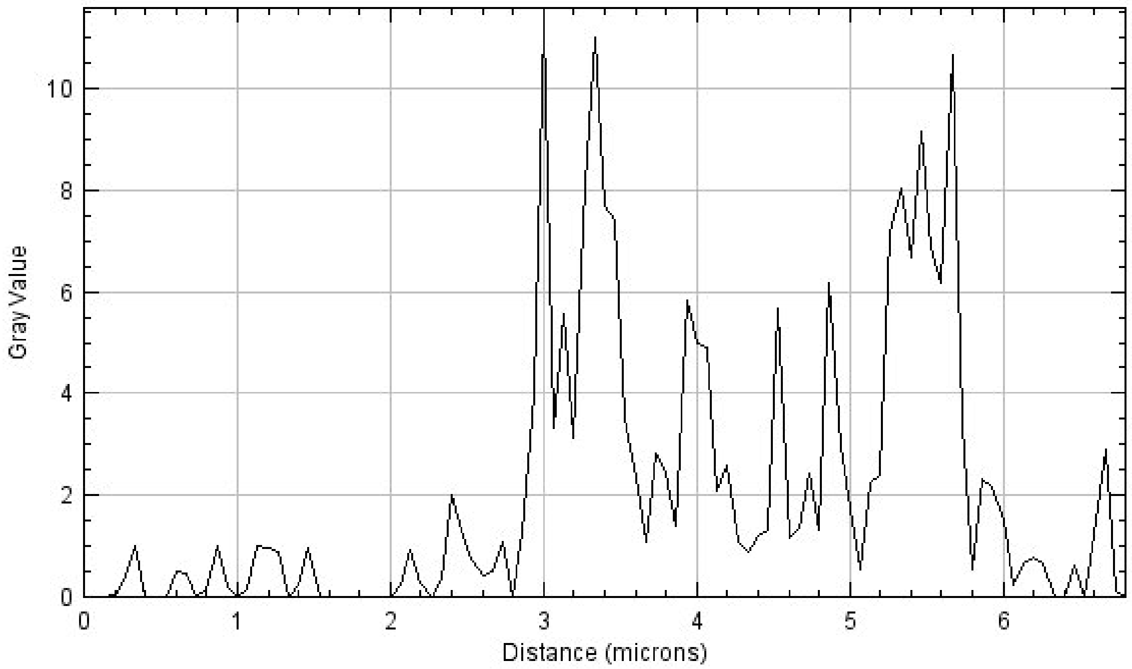

Figure S717 – Intensity profile of line scan generated for cell 5, Figure S712.

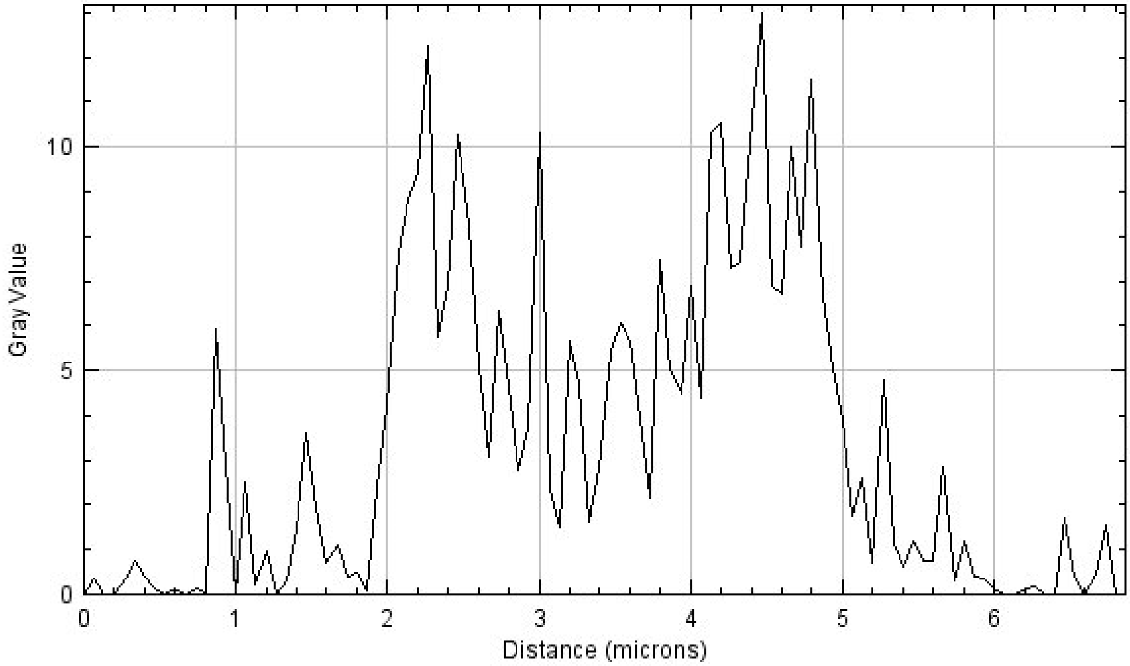

Figure S718 – Intensity profile of line scan generated for cell 6, Figure S712.

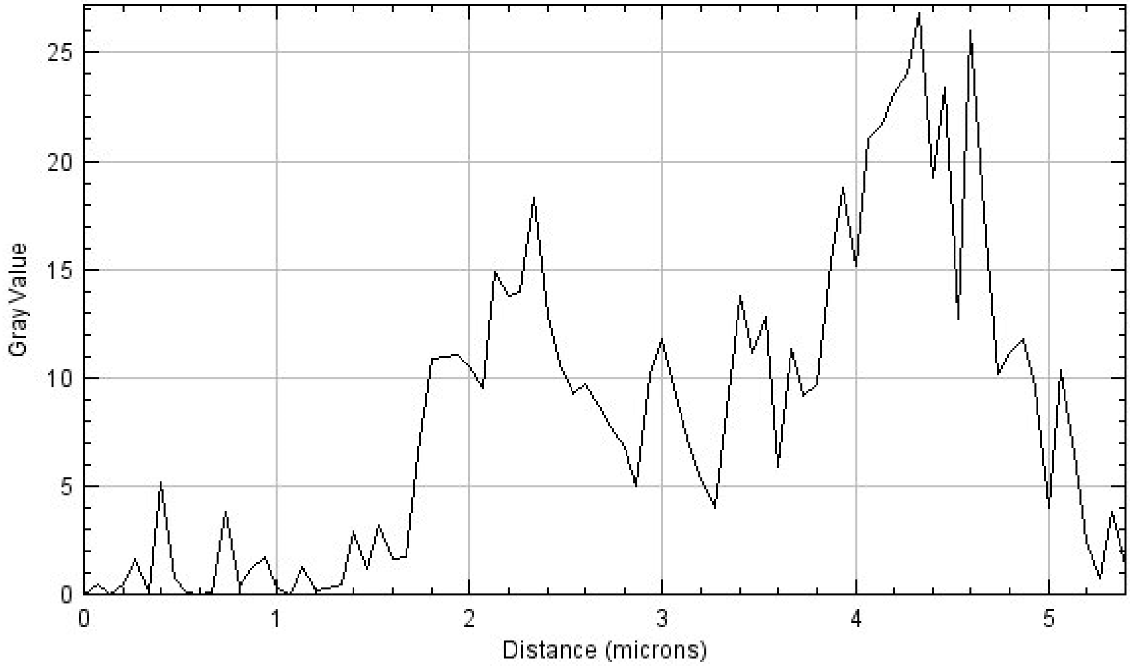

Figure S719 – Intensity profile of line scan generated for cell 7, Figure S712.

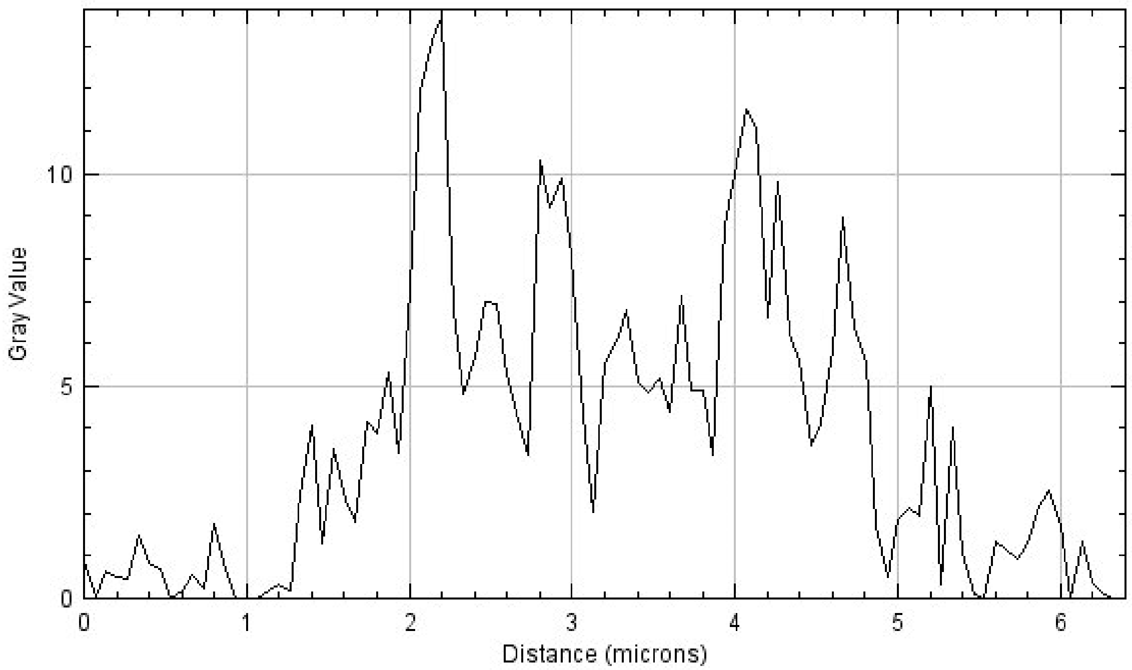

Figure S720 – Intensity profile of line scan generated for cell 8, Figure S712.

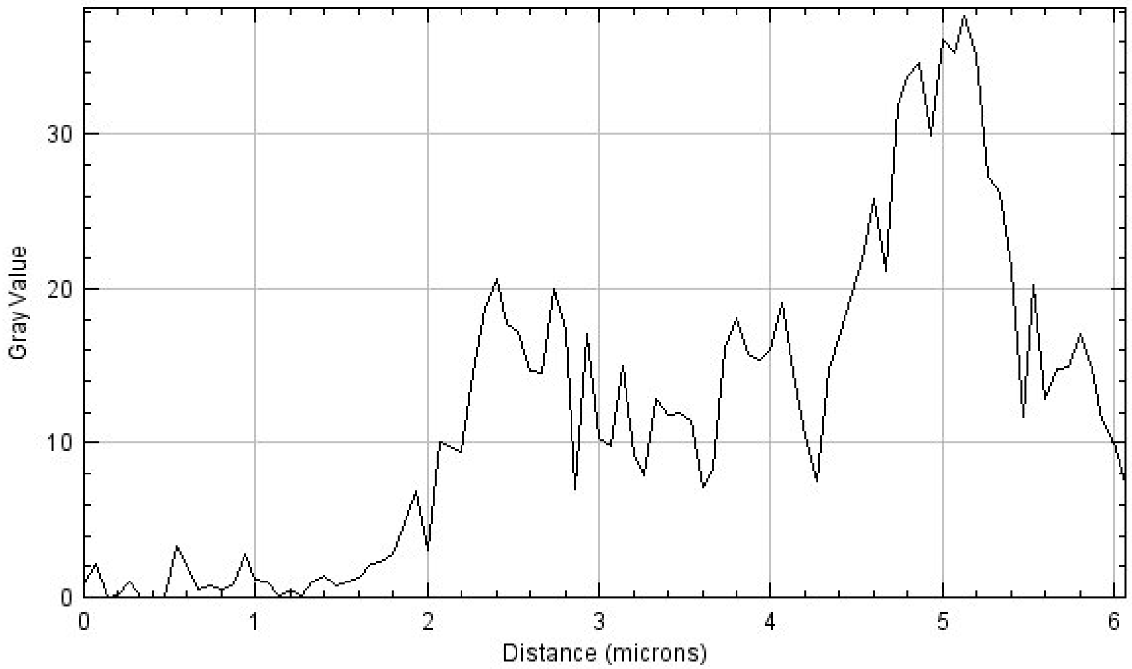

Figure S721 – Intensity profile of line scan generated for cell 9, Figure S712.

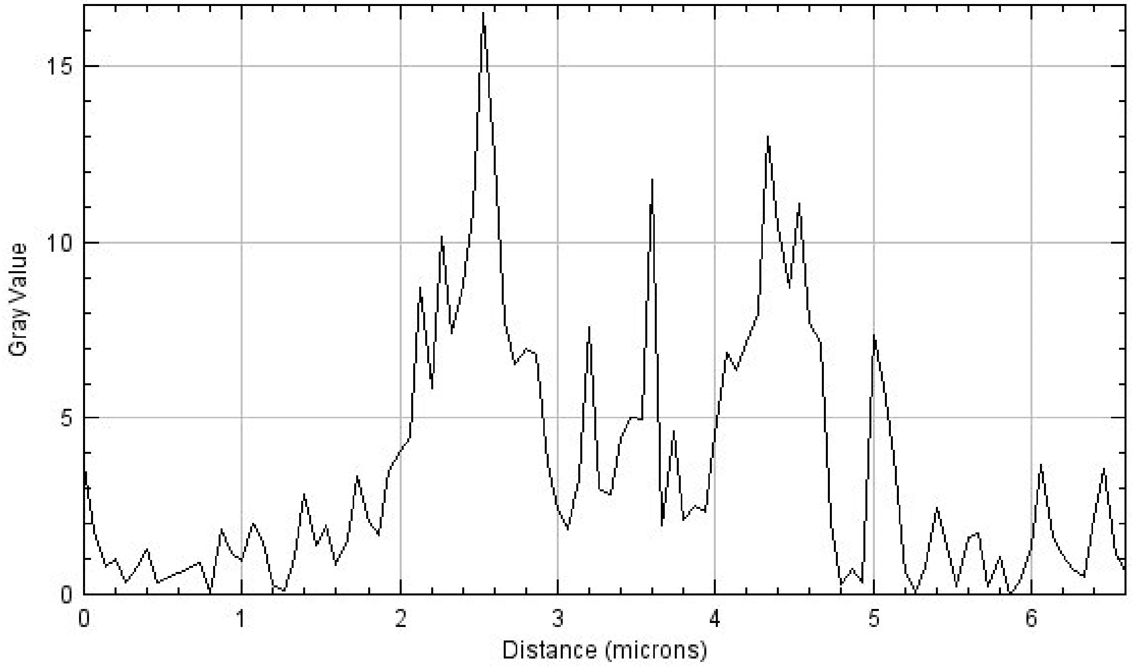

Figure S722 – Intensity profile of line scan generated for cell 10, Figure S712.

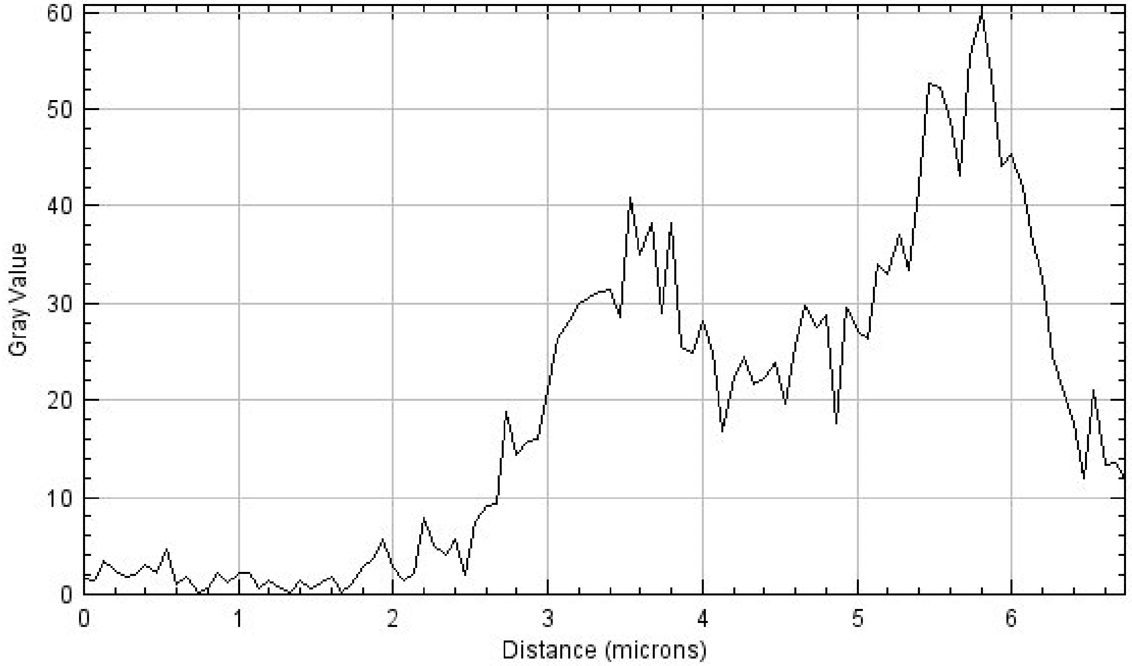

Figure S723 – Intensity profile of line scan generated for cell 11, Figure S712.

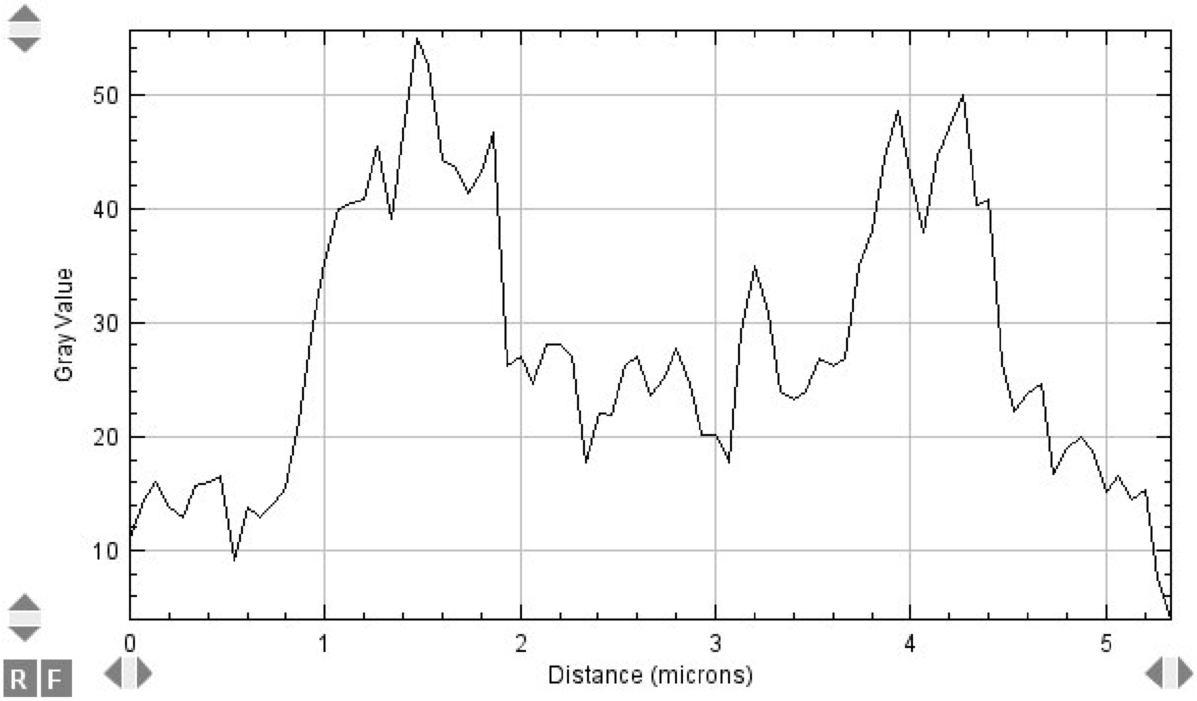

Figure S724 – Intensity profile of line scan generated for cell 12, Figure S712.

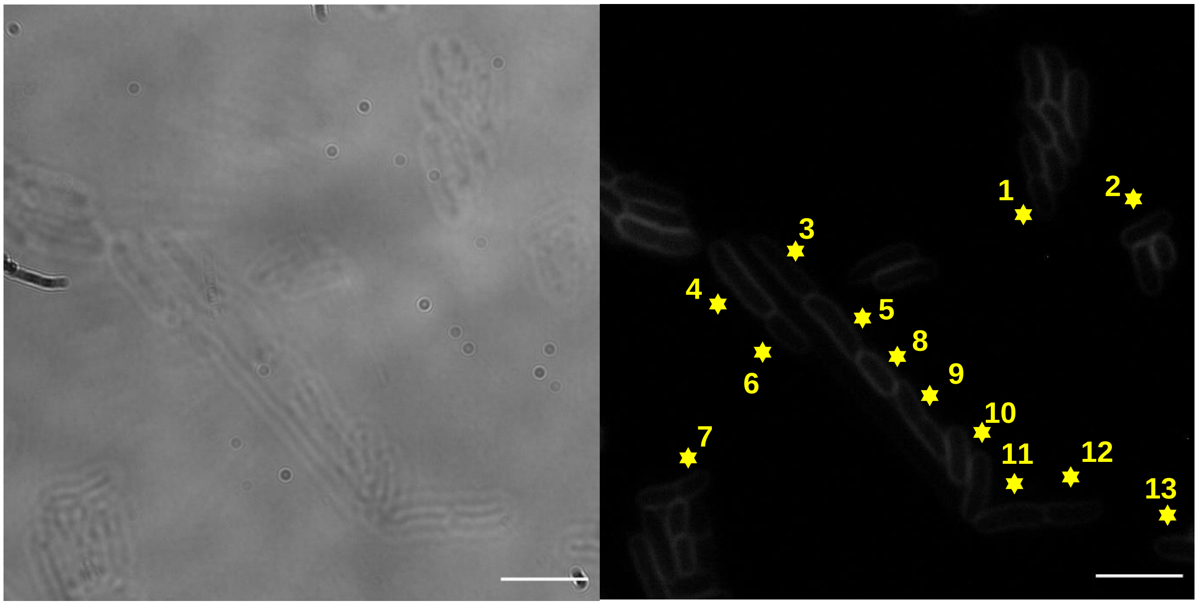

Figure S725 – *E. coli* transmitted and mCherry Images used in fluorescence intensity calculations in the presence of FM4-64 at T = 30 minutes. Scale bar = 10 μM.

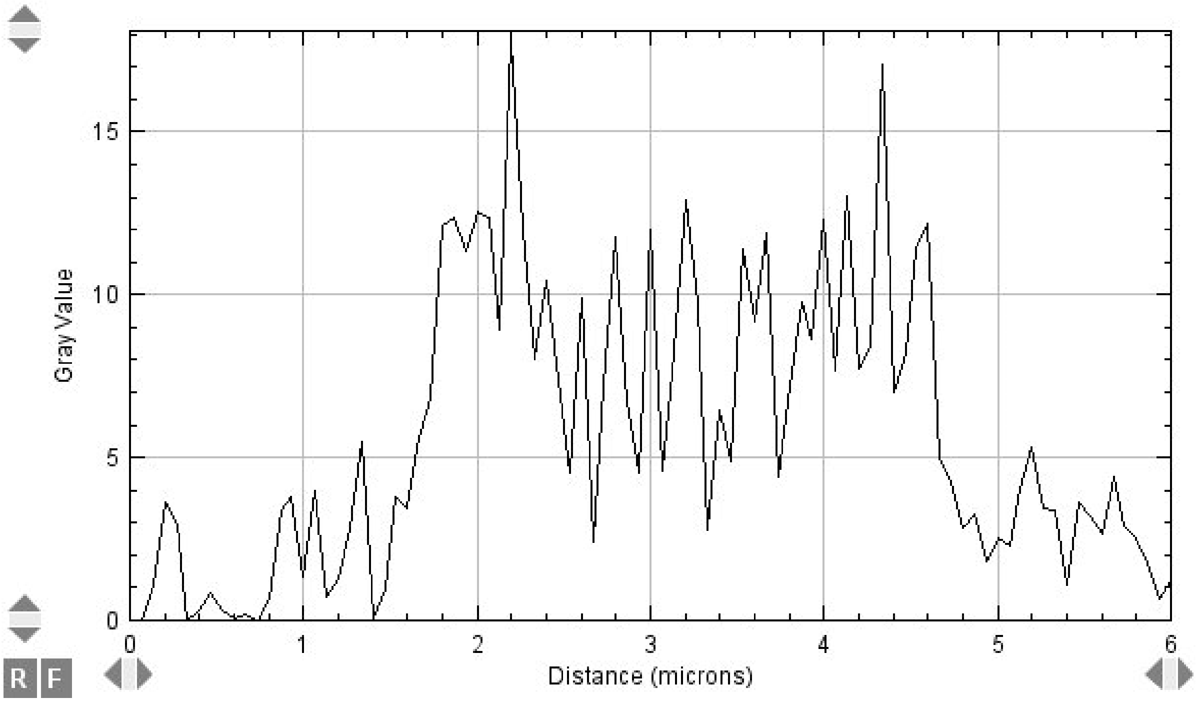

Figure S726 – Intensity profile of line scan generated for cell 1, Figure S725.

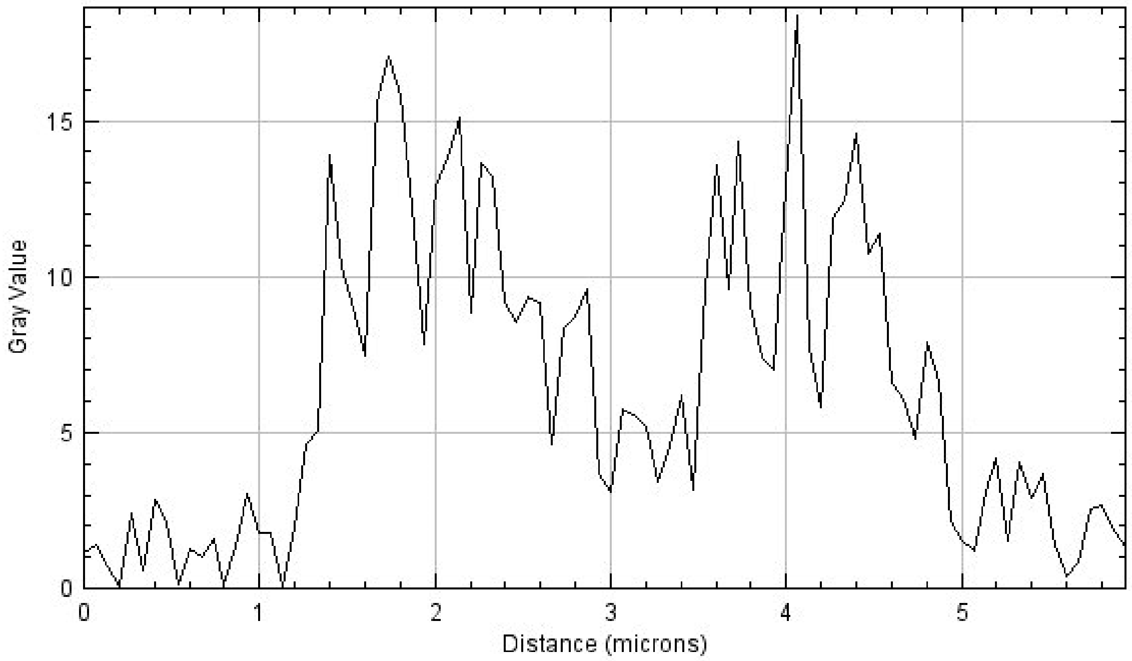

Figure S727 – Intensity profile of line scan generated for cell 2, Figure S725.

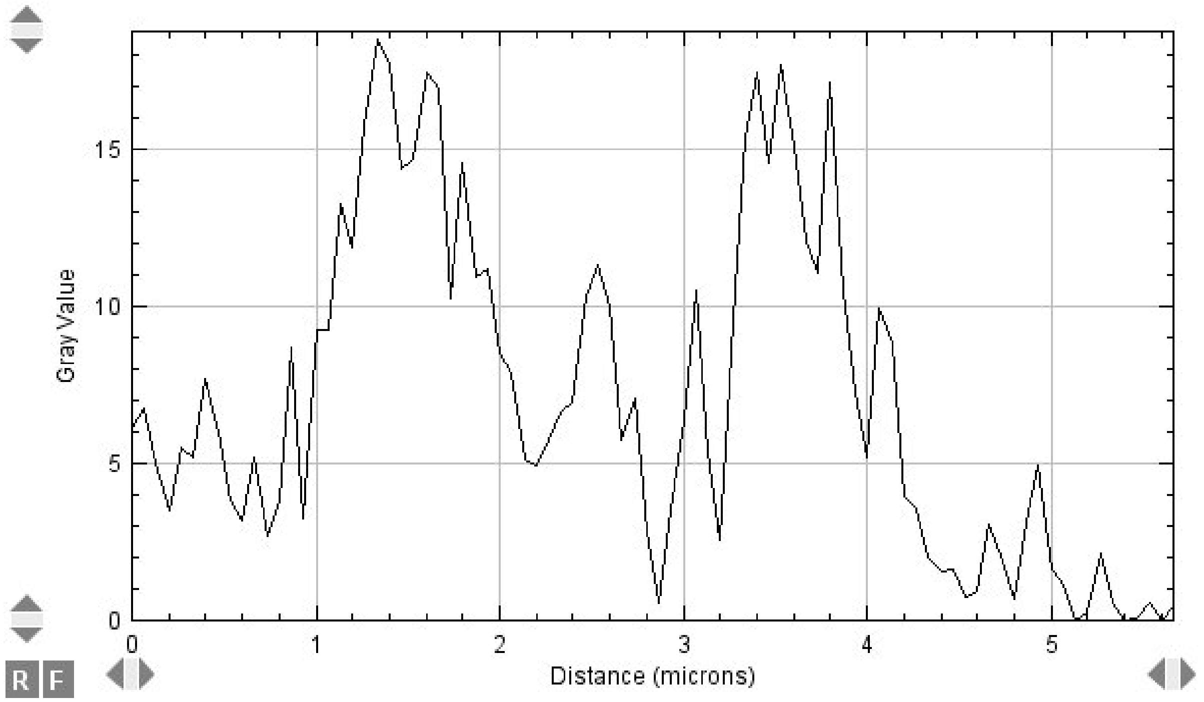

Figure S728 – Intensity profile of line scan generated for cell 3, Figure S725.

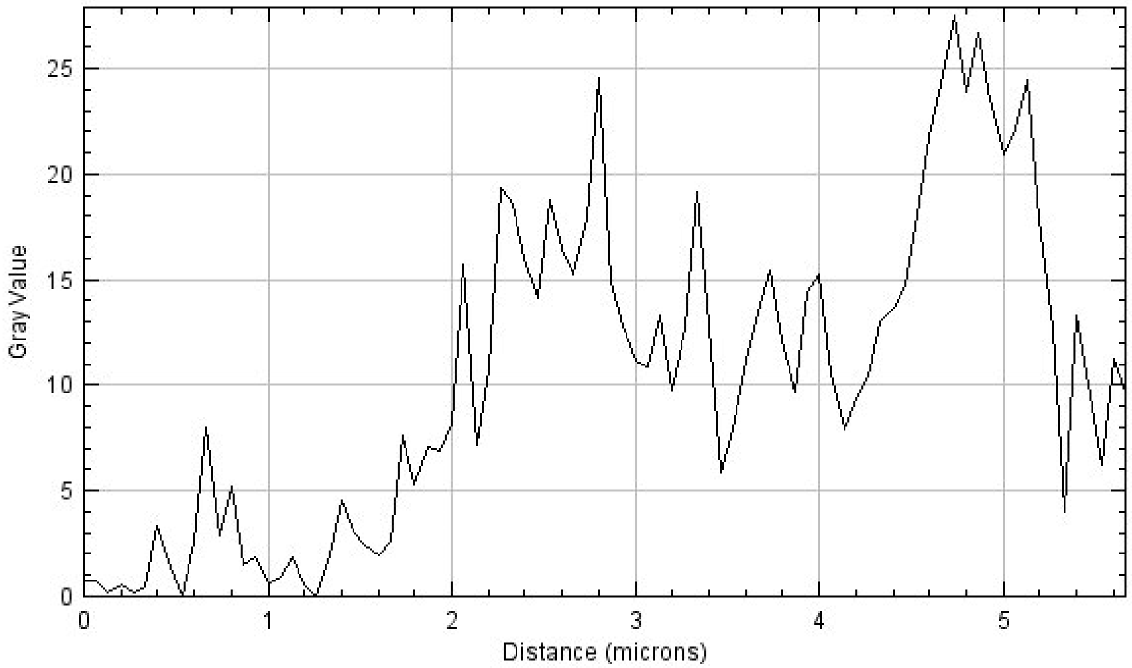

Figure S729 – Intensity profile of line scan generated for cell 4, Figure S725.

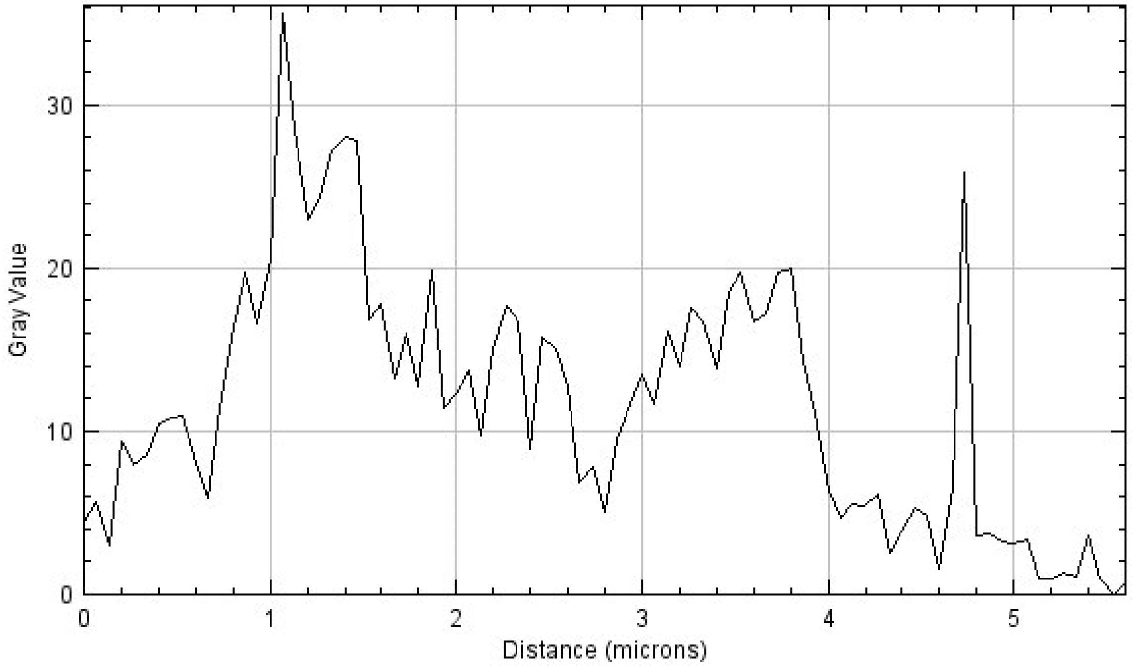

Figure S730 – Intensity profile of line scan generated for cell 5, Figure S725.

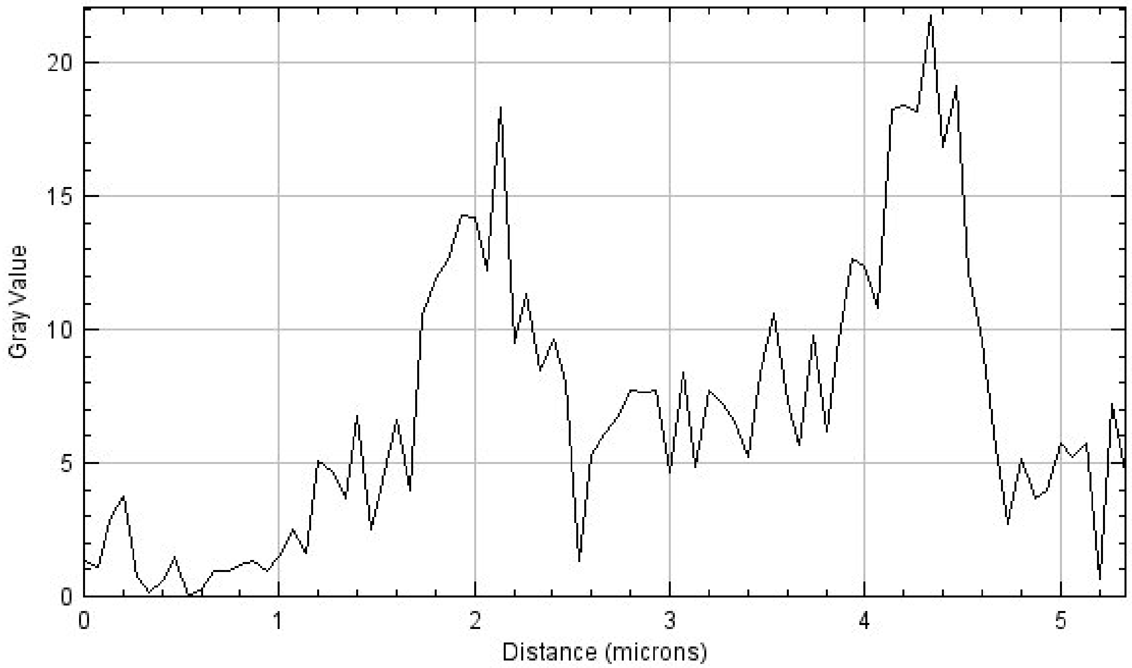

Figure S731 – Intensity profile of line scan generated for cell 6, Figure S725.

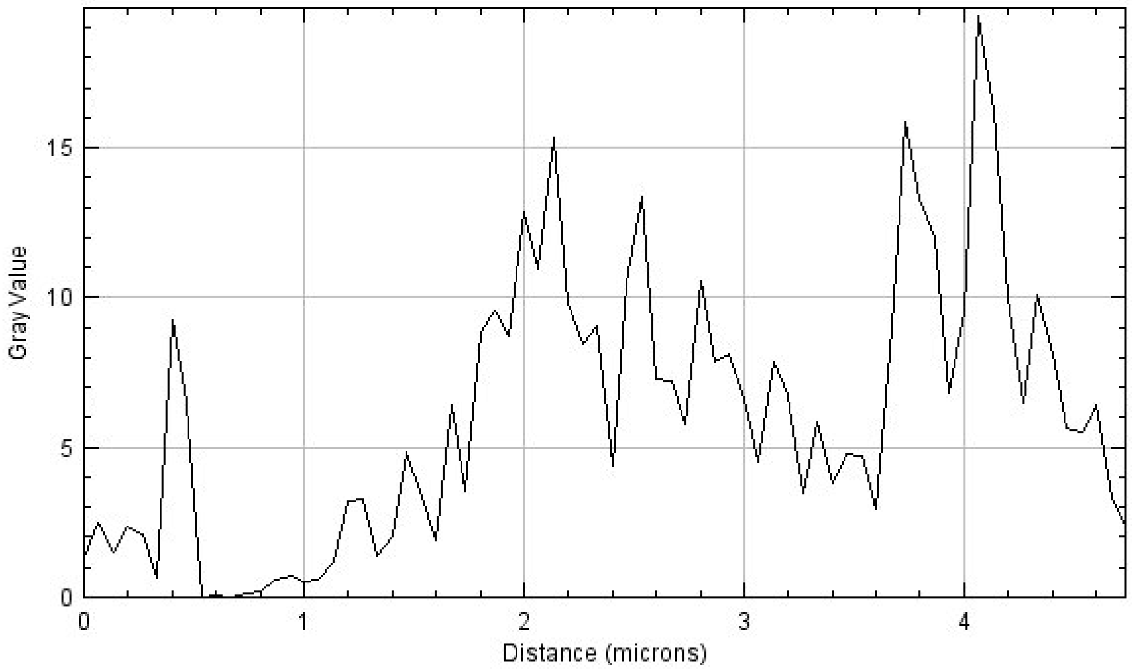

Figure S732 – Intensity profile of line scan generated for cell 7, Figure S725.

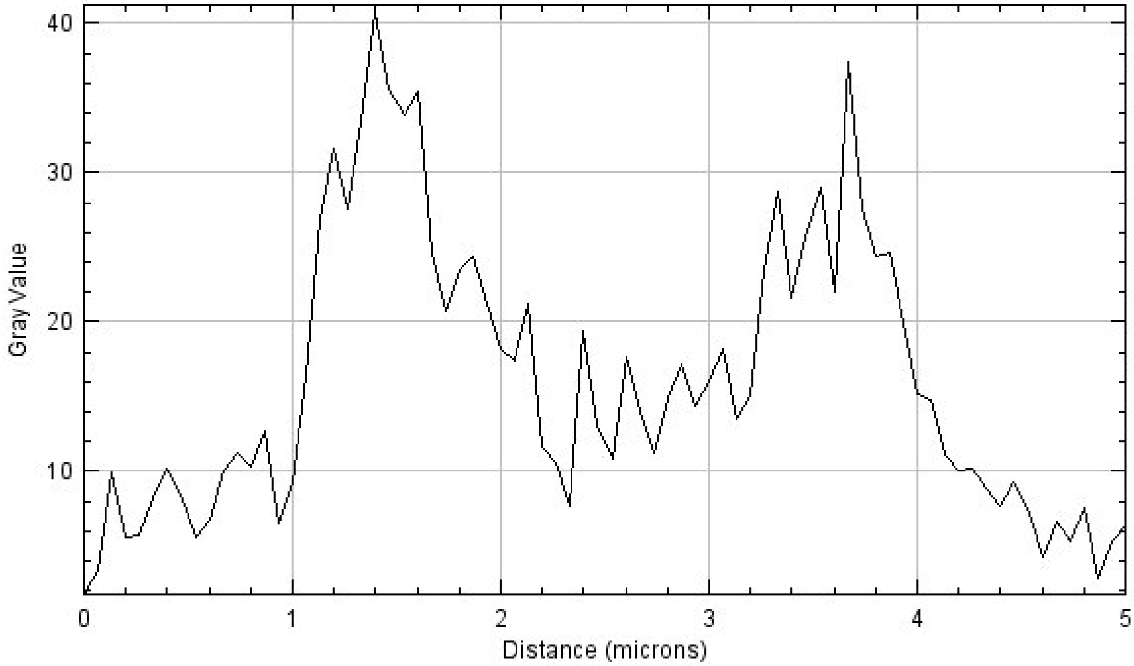

Figure S733 – Intensity profile of line scan generated for cell 8, Figure S725.

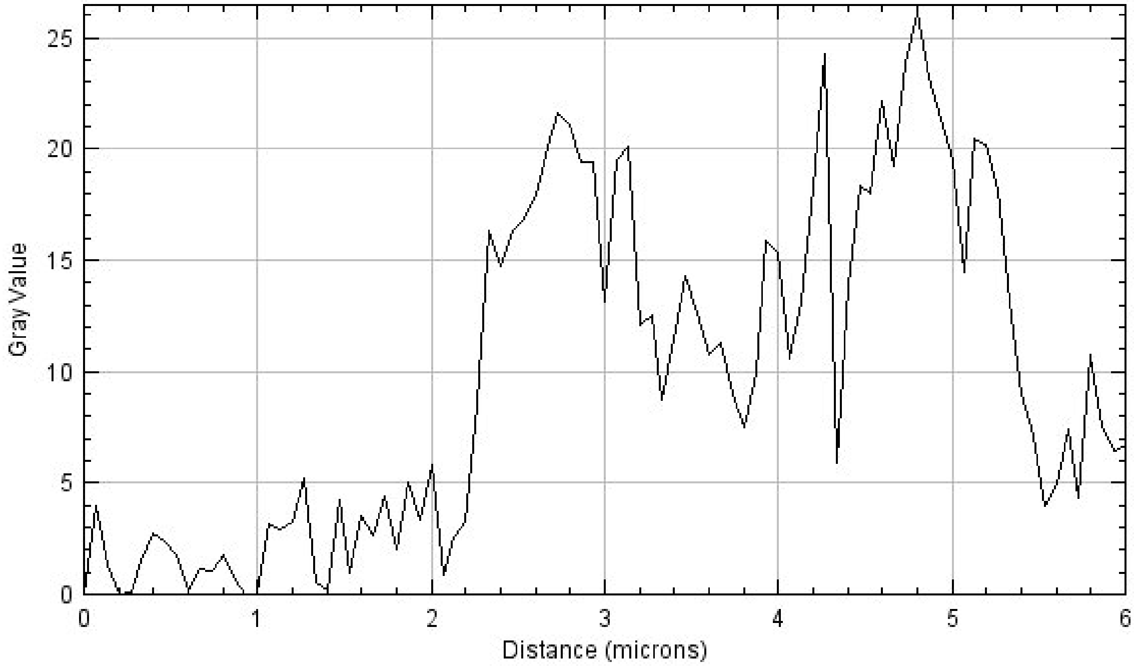

Figure S734 – Intensity profile of line scan generated for cell 9, Figure S725.

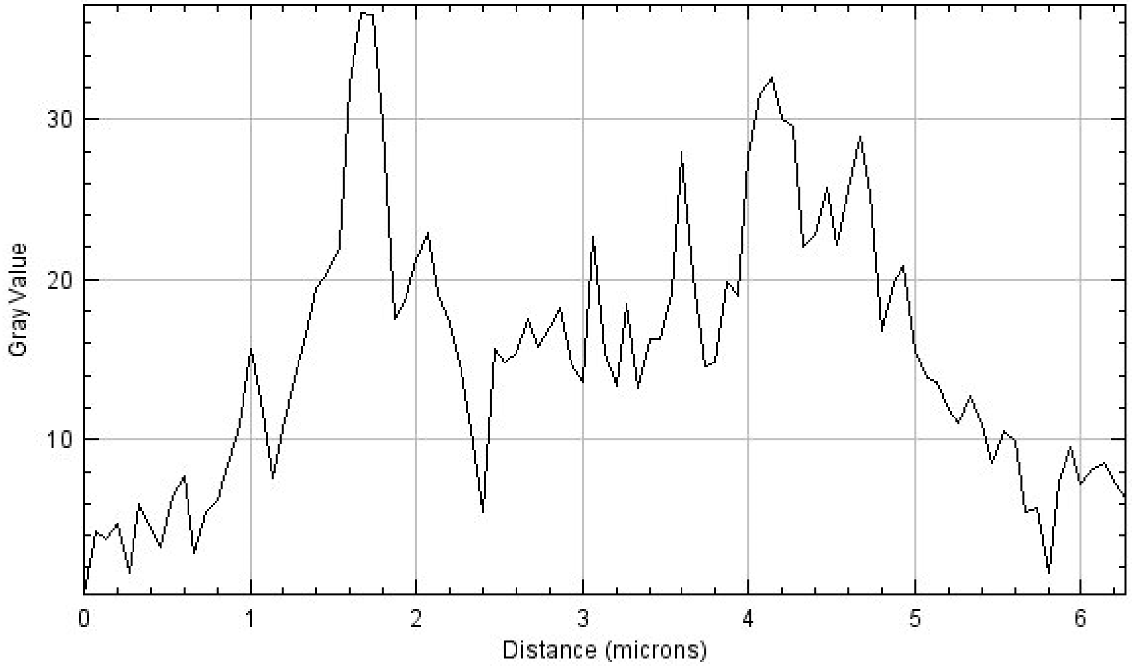

Figure S735 – Intensity profile of line scan generated for cell 10, Figure S725.

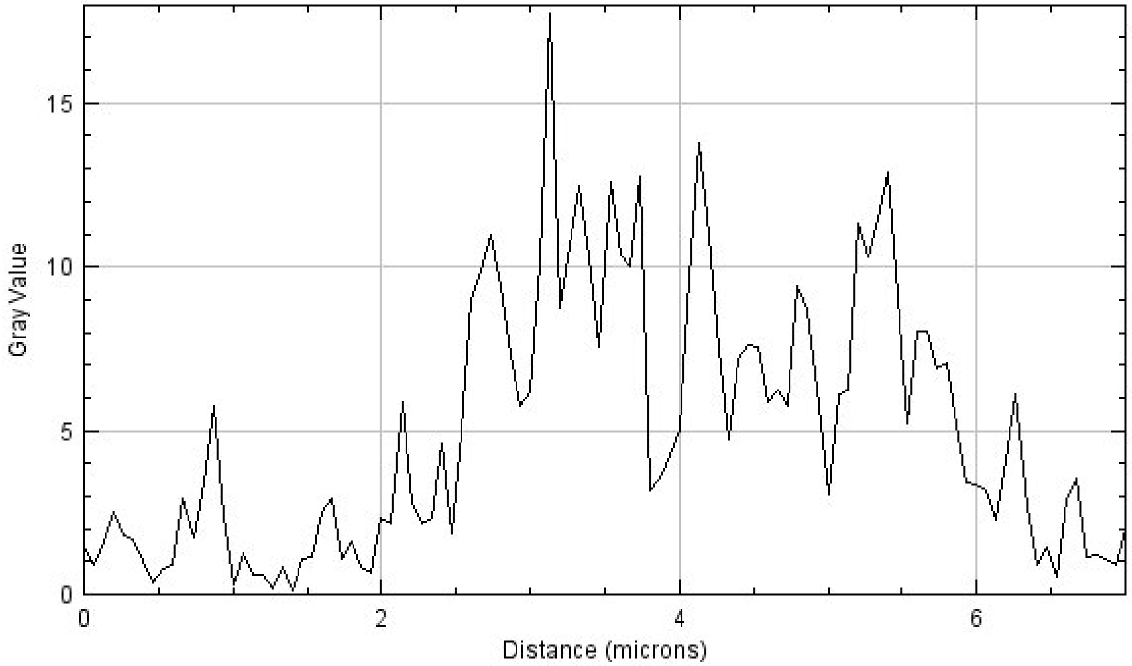

Figure S736 – Intensity profile of line scan generated for cell 11, Figure S725.

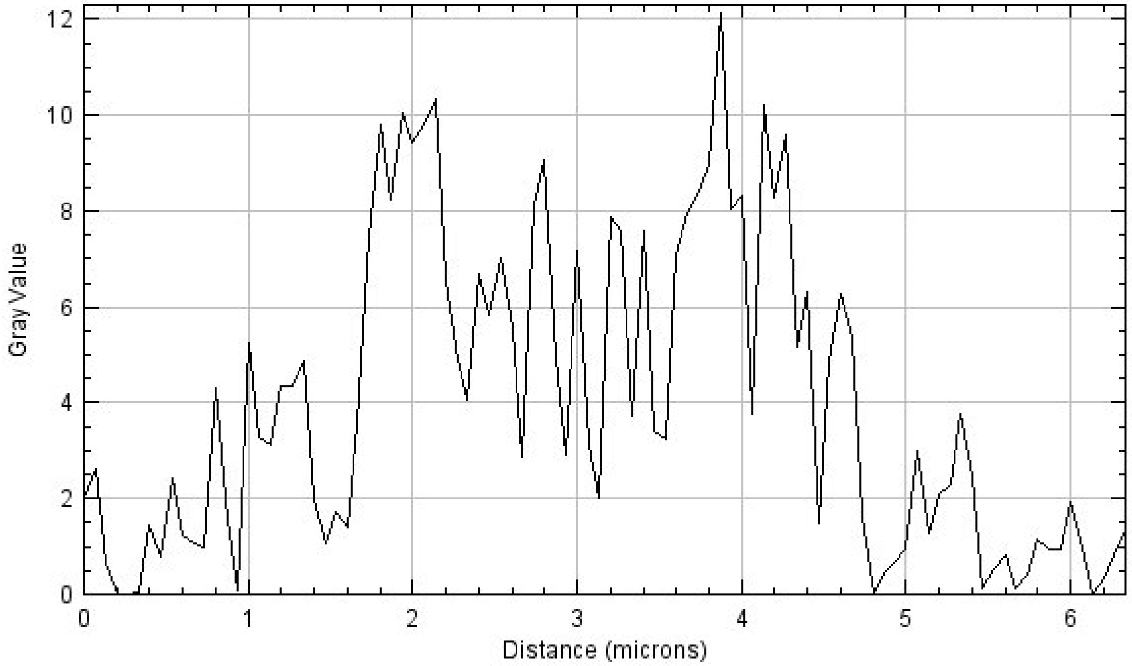

Figure S737 – Intensity profile of line scan generated for cell 12, Figure S725.

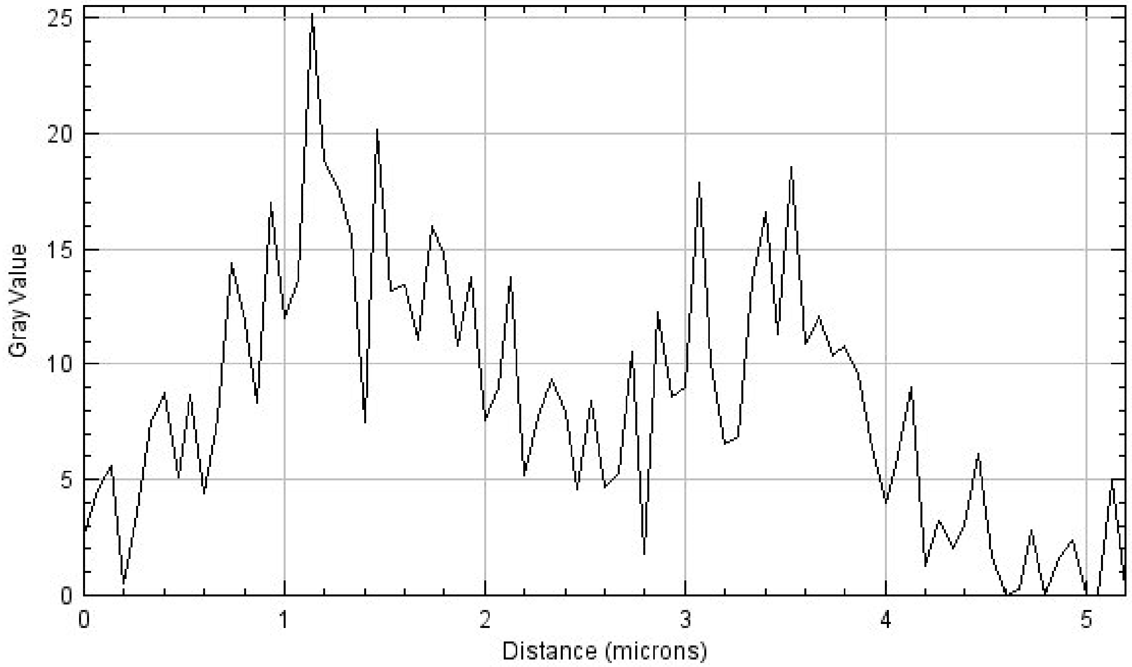

Figure S738 – Intensity profile of line scan generated for cell 13, Figure S725.

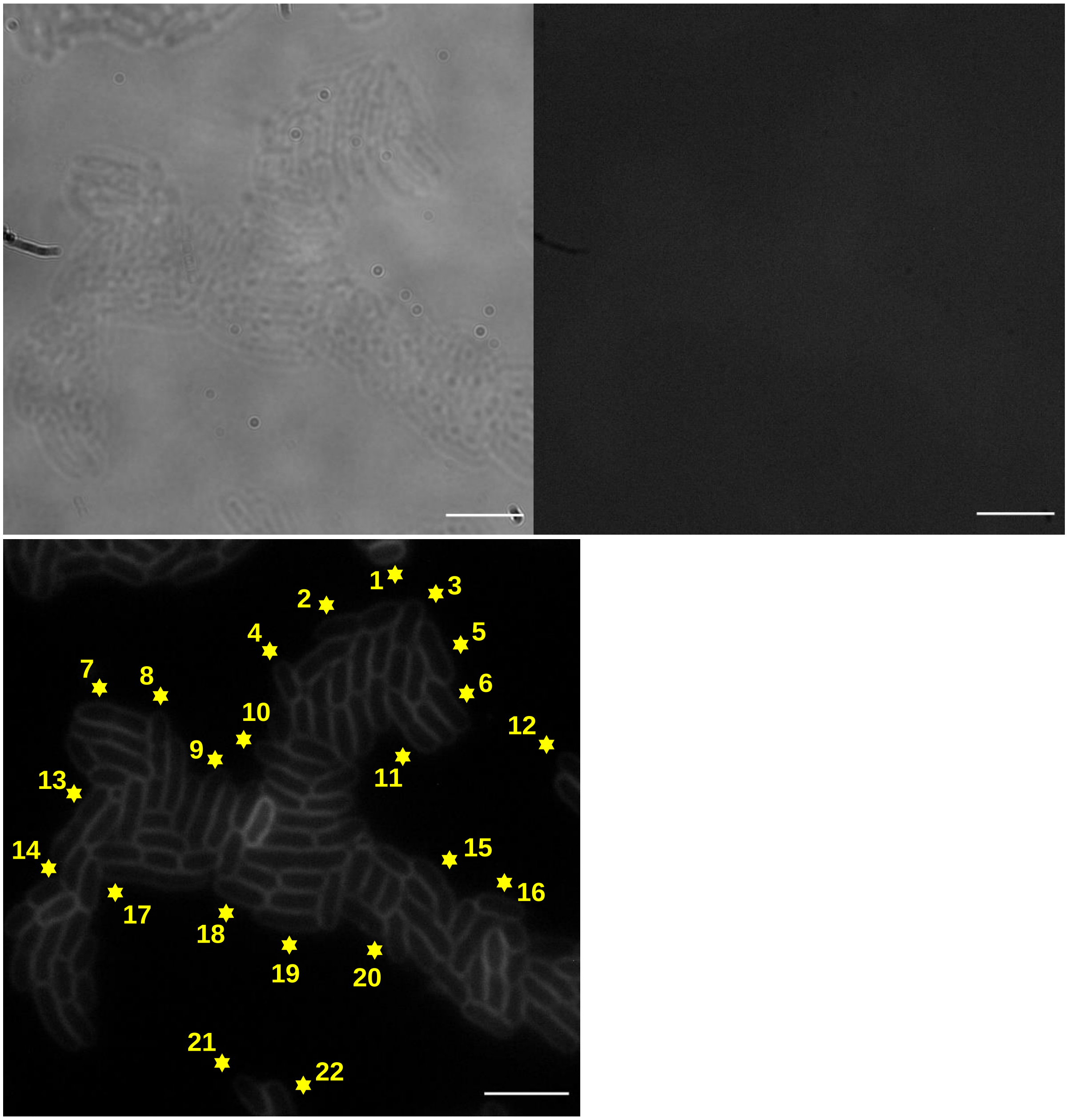

Figure S739 – *E. coli* transmitted and DAPI Images used in fluorescence intensity calculations in the presence of FM4-64 at T = 30 minutes. mCherry image used to locate cells on DAPI image. Scale bar = 10 µM.

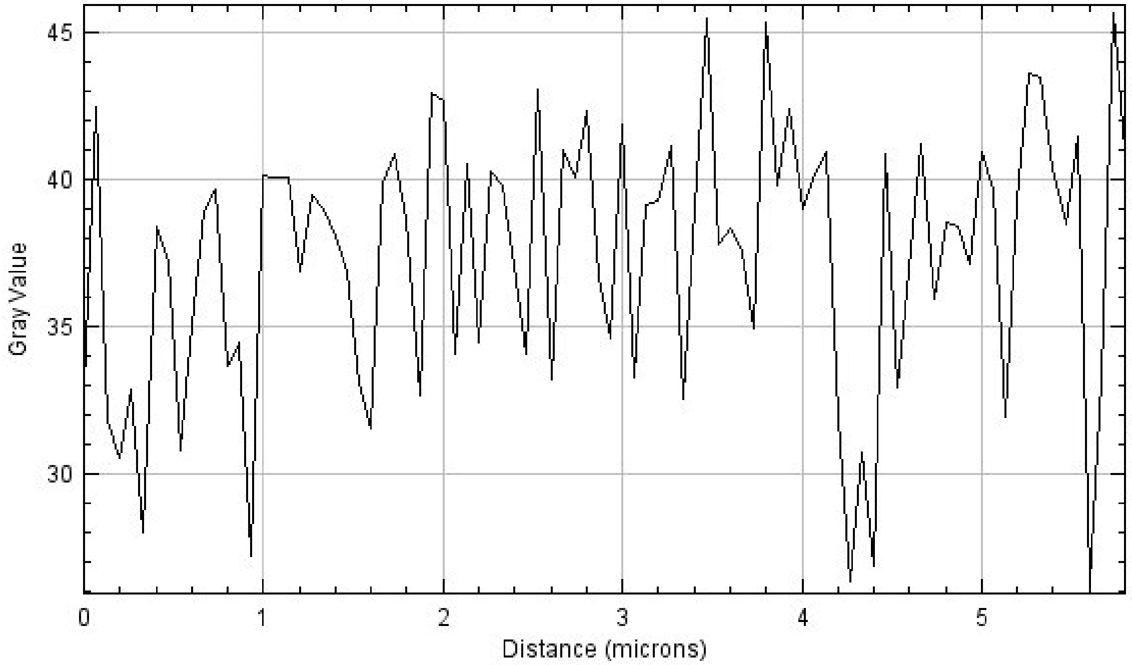

Figure S740 – Intensity profile of line scan generated for cell 1, Figure S739.

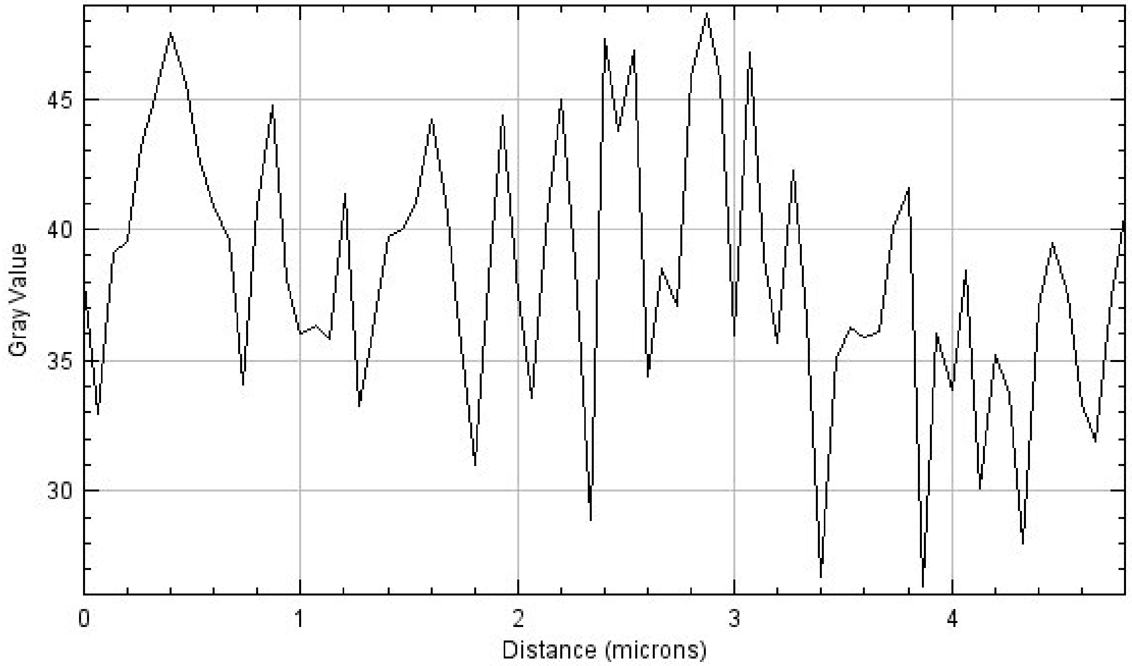

Figure S741 – Intensity profile of line scan generated for cell 2, Figure S739.

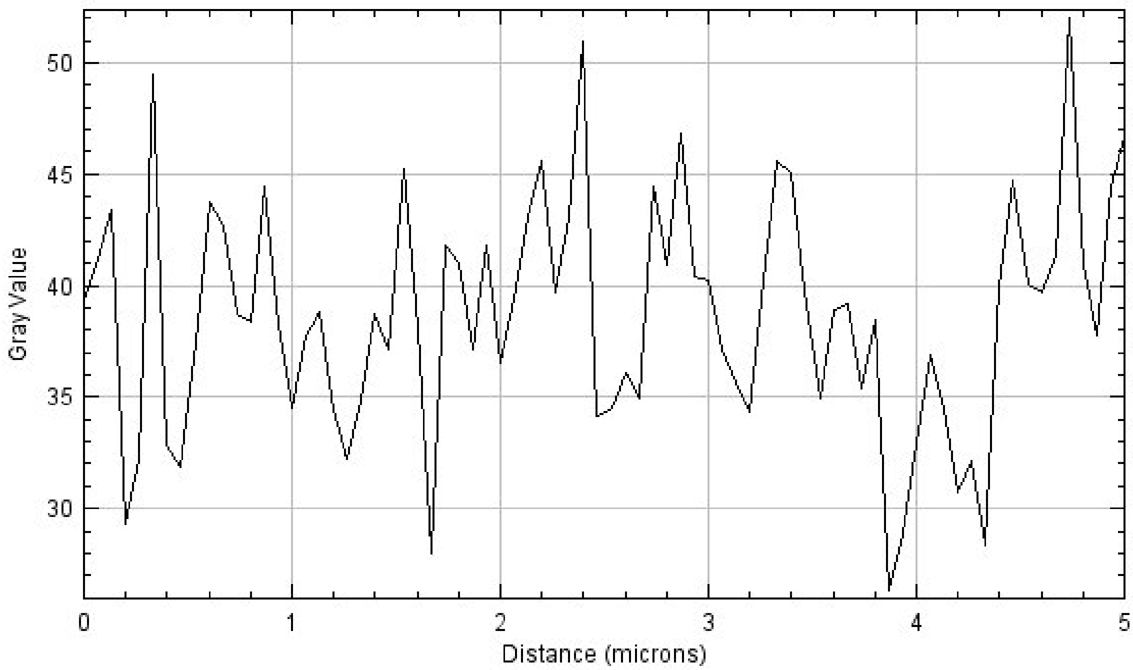

Figure S742 – Intensity profile of line scan generated for cell 3, Figure S739.

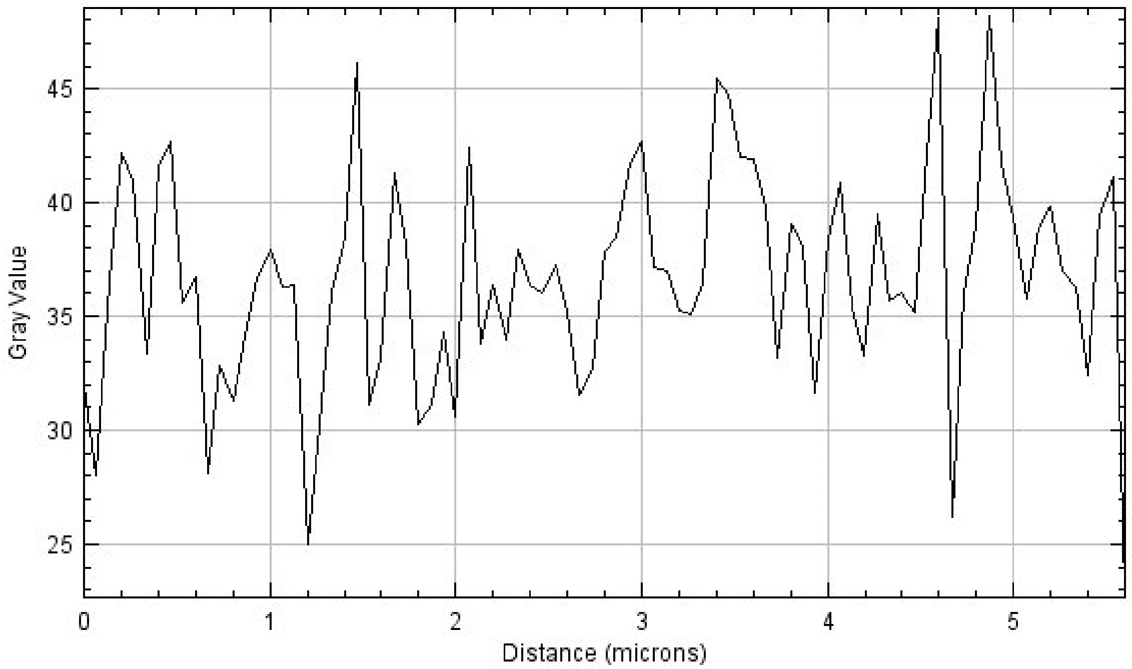

Figure S743 – Intensity profile of line scan generated for cell 4, Figure S739.

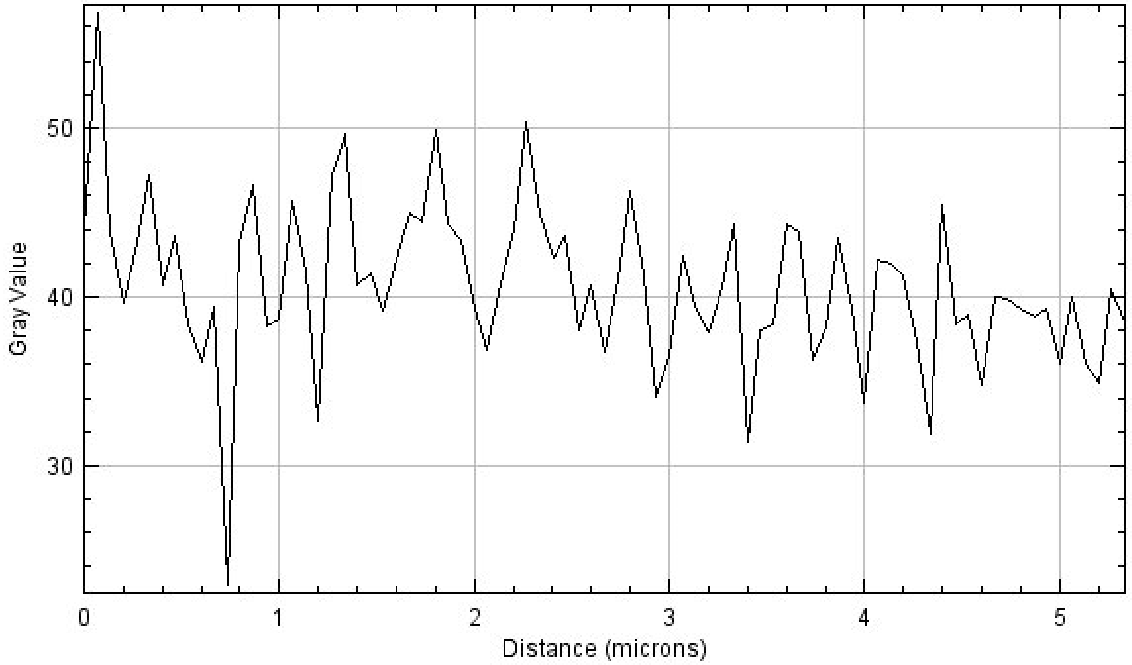

Figure S744 – Intensity profile of line scan generated for cell 5, Figure S739.

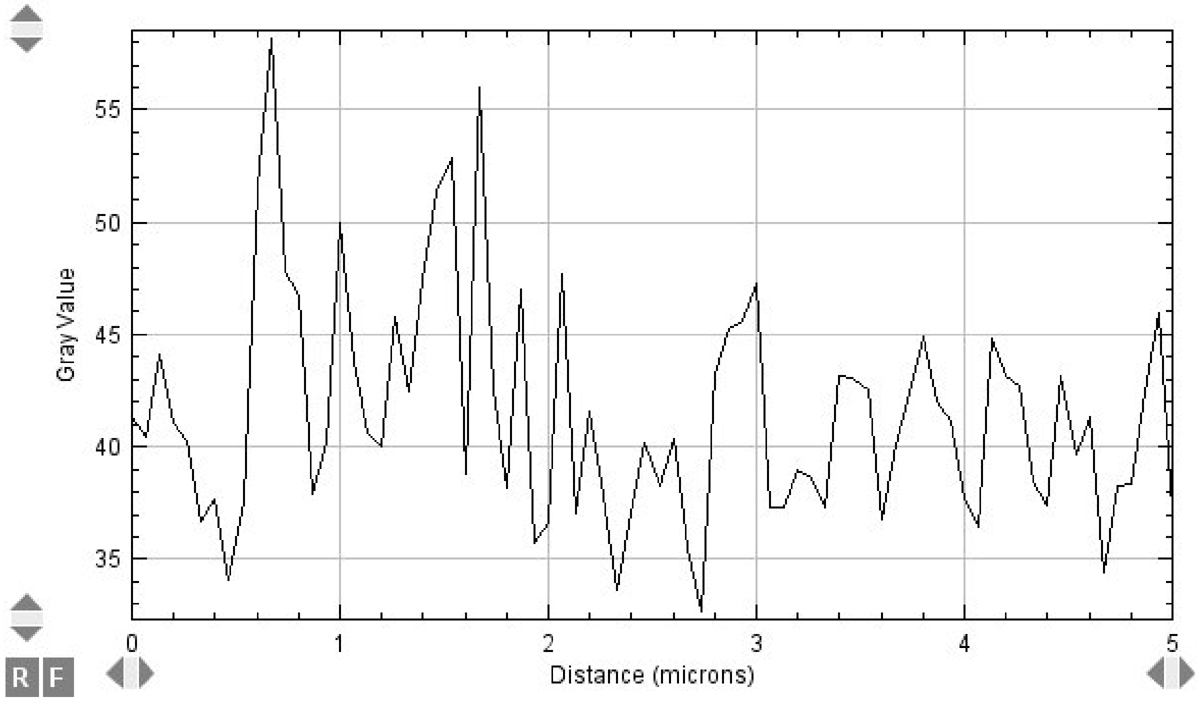

Figure S745 – Intensity profile of line scan generated for cell 6, Figure S739.

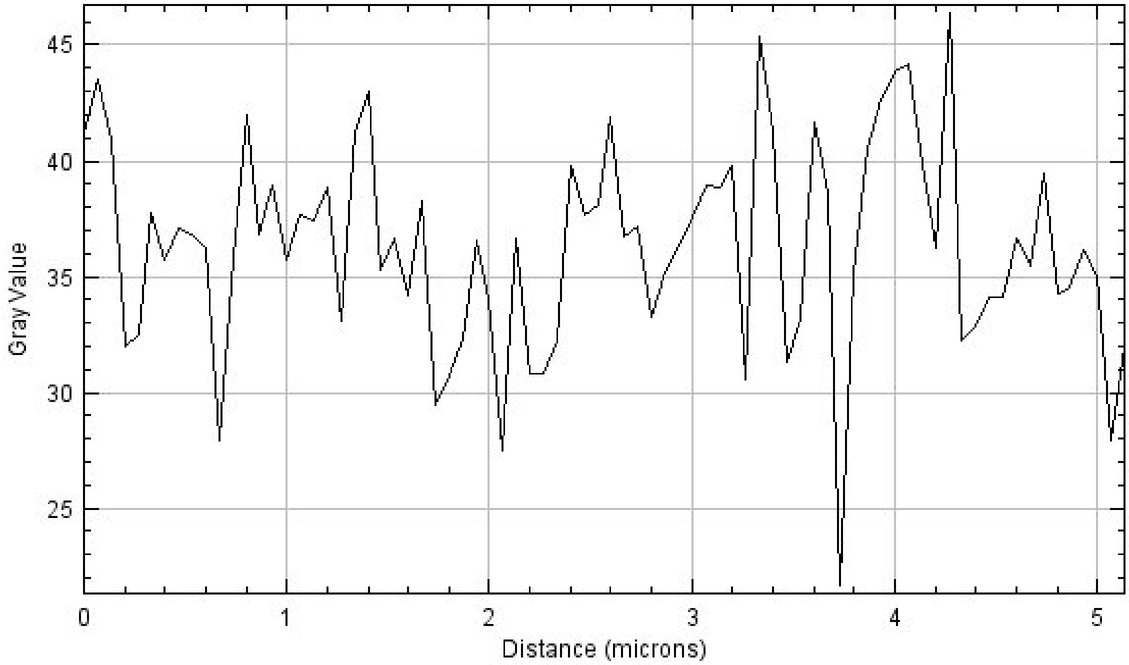

Figure S746 – Intensity profile of line scan generated for cell 7, Figure S739.

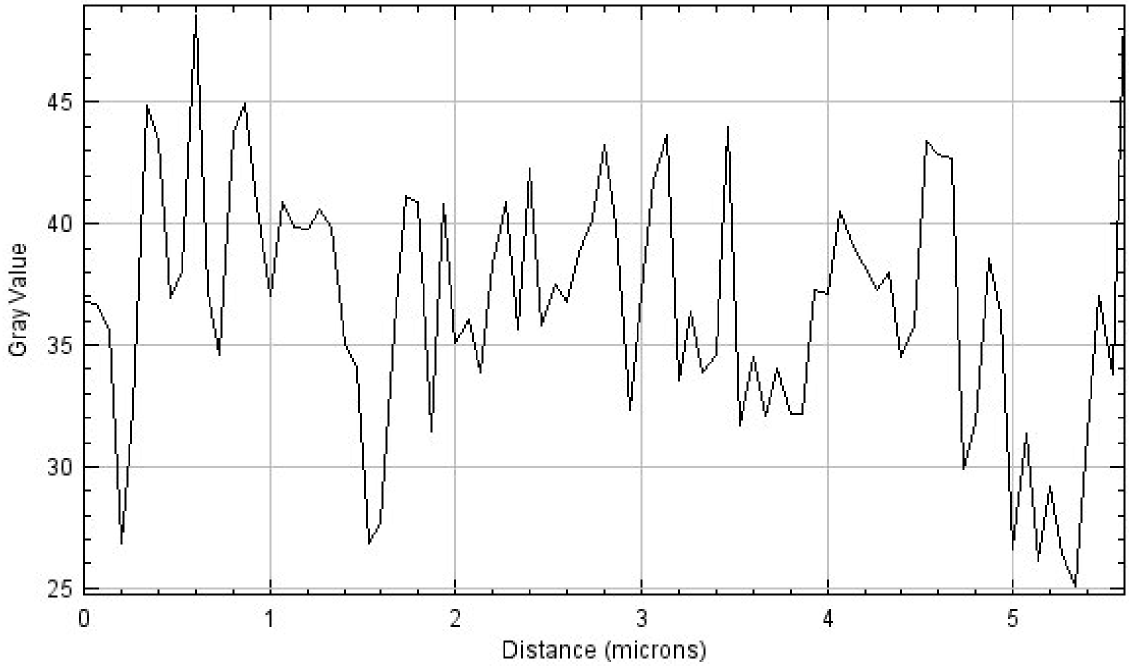

Figure S747 – Intensity profile of line scan generated for cell 8, Figure S739.

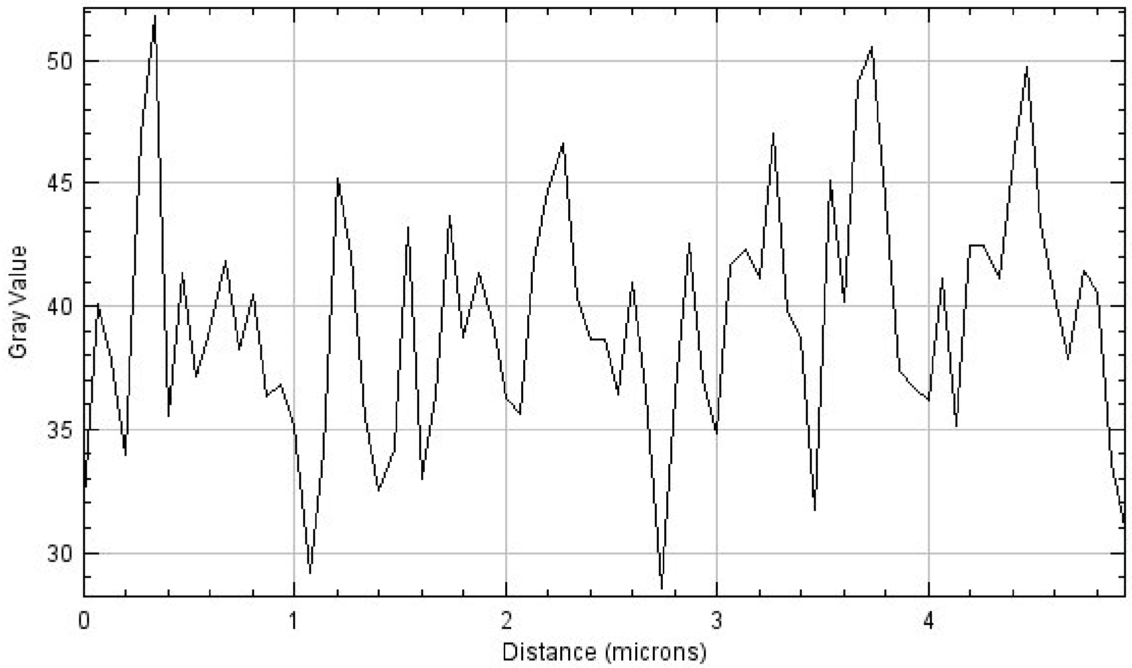

Figure S748 – Intensity profile of line scan generated for cell 9, Figure S739.

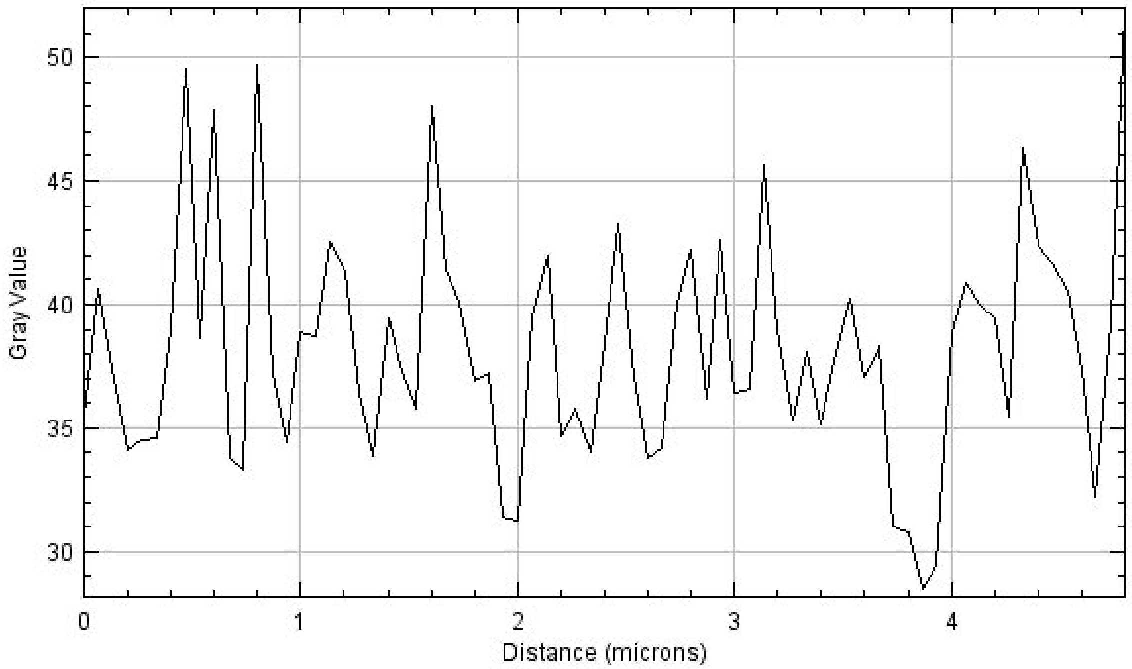

Figure S749 – Intensity profile of line scan generated for cell 10, Figure S739.

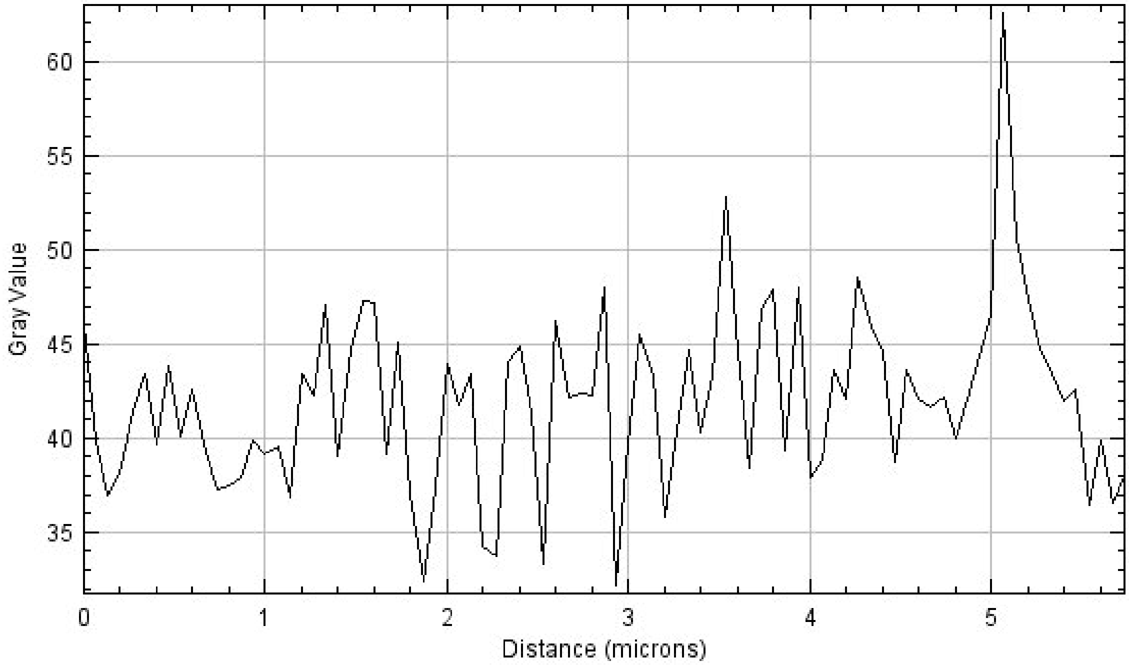

Figure S750 – Intensity profile of line scan generated for cell 11, Figure S739.

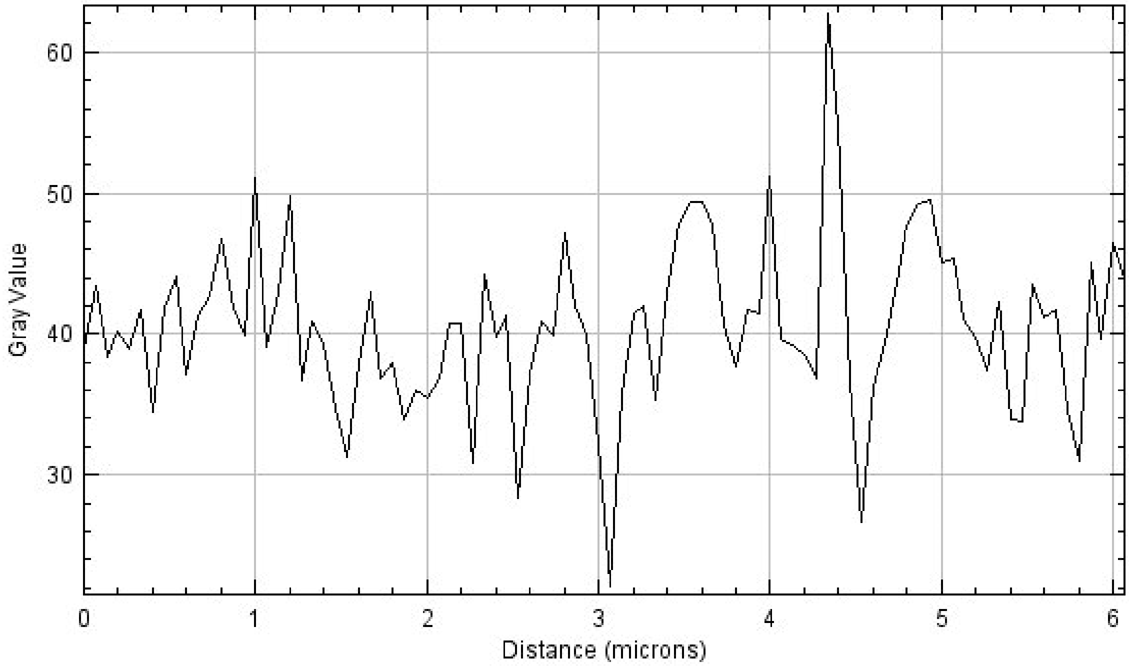

Figure S751 – Intensity profile of line scan generated for cell 12, Figure S739.

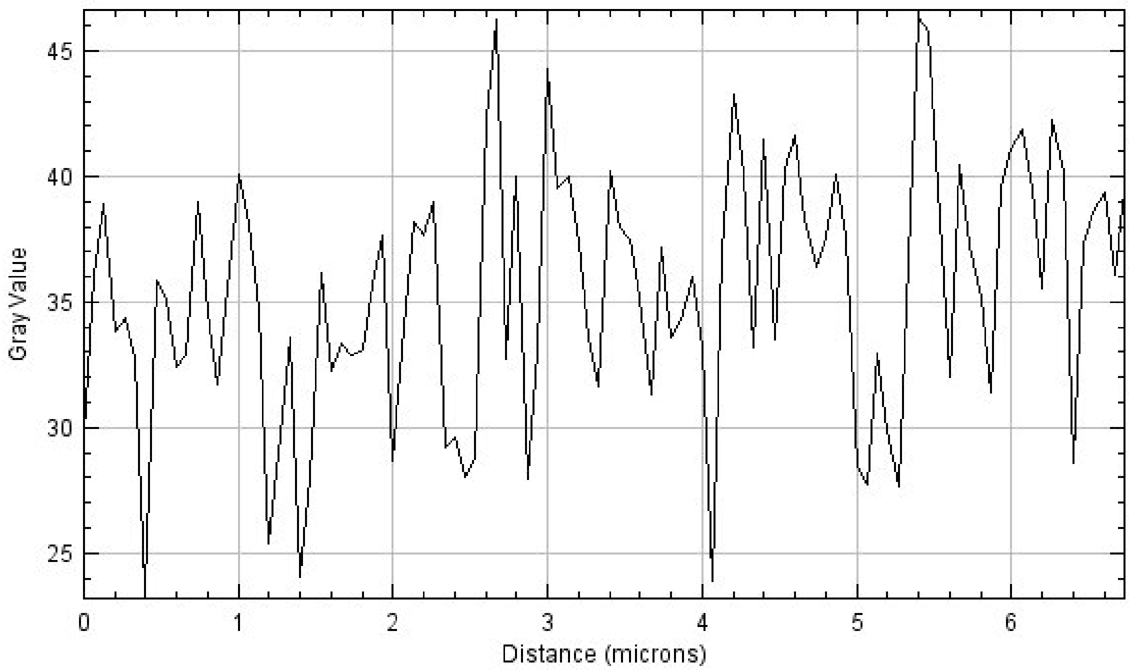

Figure S752 – Intensity profile of line scan generated for cell 13, Figure S739.

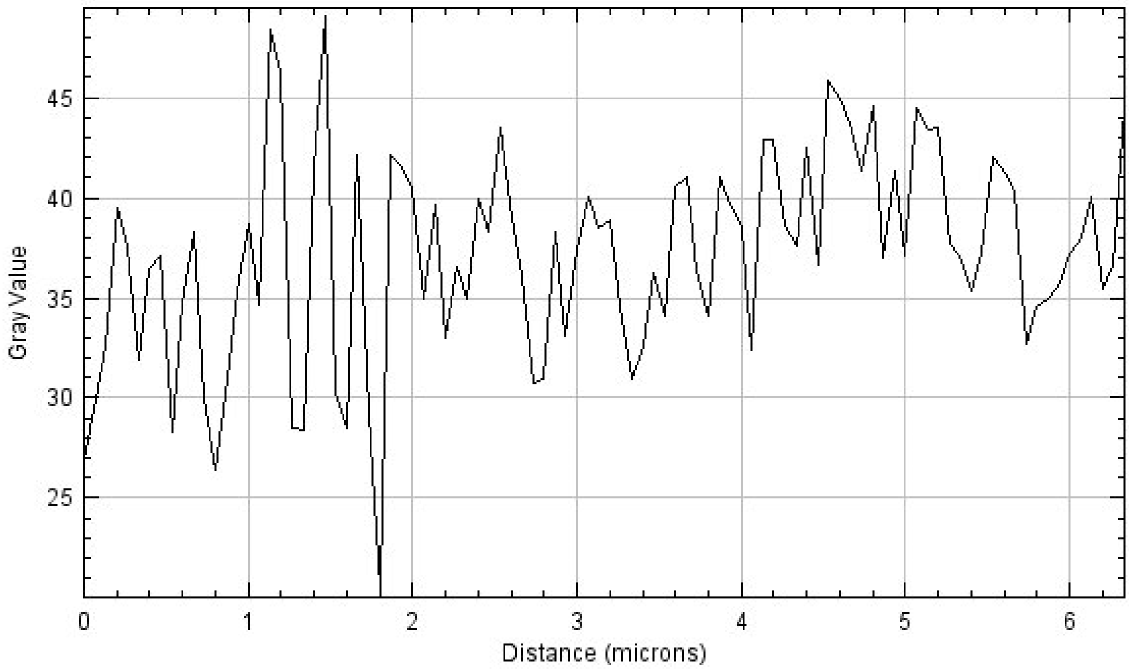

Figure S753 – Intensity profile of line scan generated for cell 14, Figure S739.

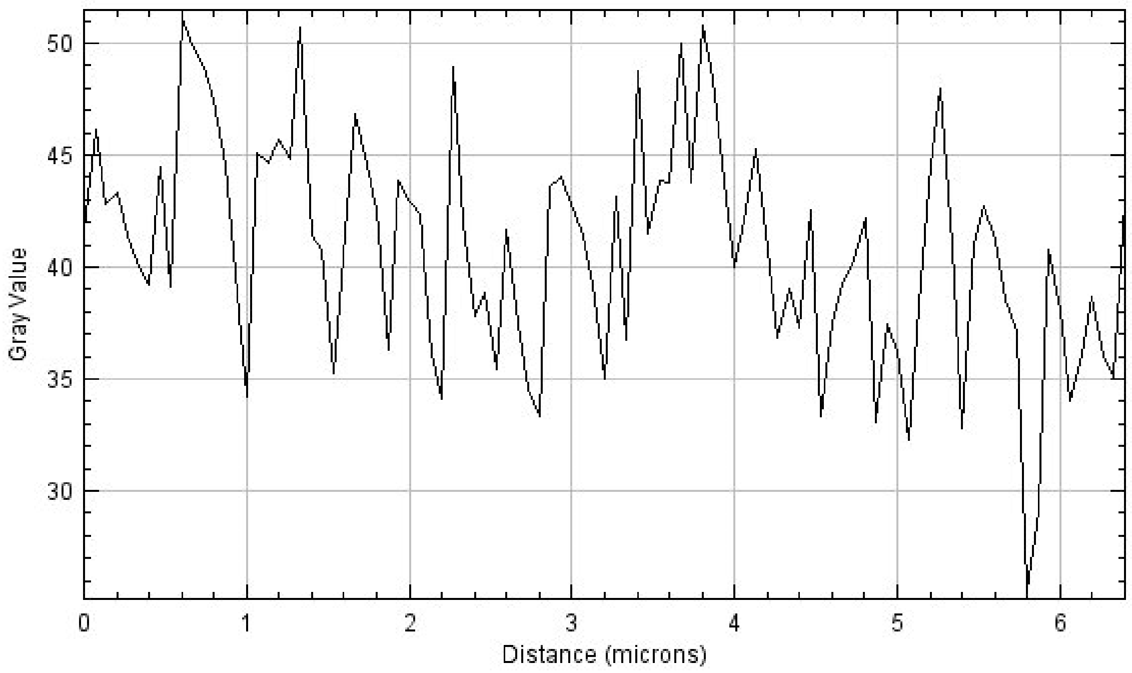

Figure S754 – Intensity profile of line scan generated for cell 15, Figure S739.

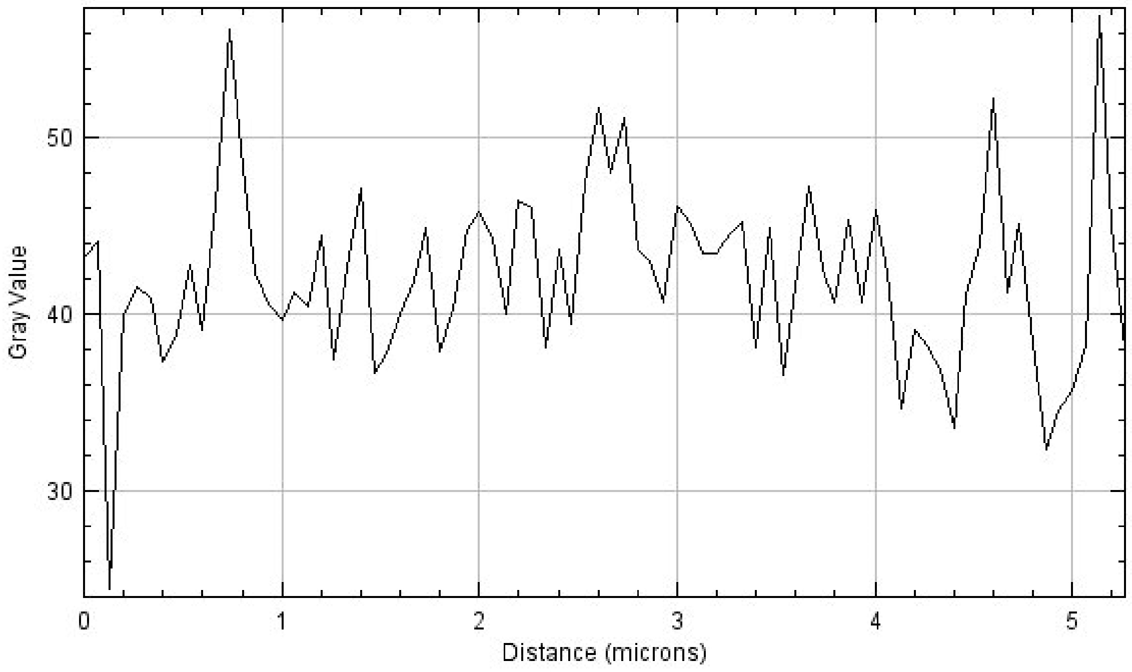

Figure S755 – Intensity profile of line scan generated for cell 16, Figure S739.

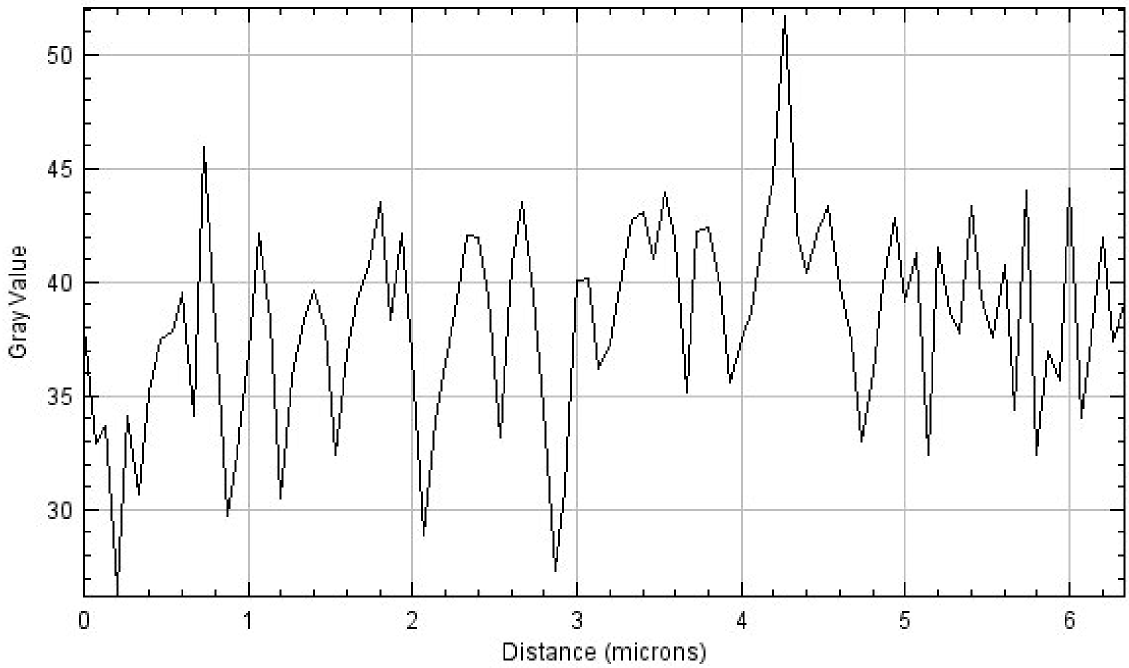

Figure S756 – Intensity profile of line scan generated for cell 17, Figure S739.

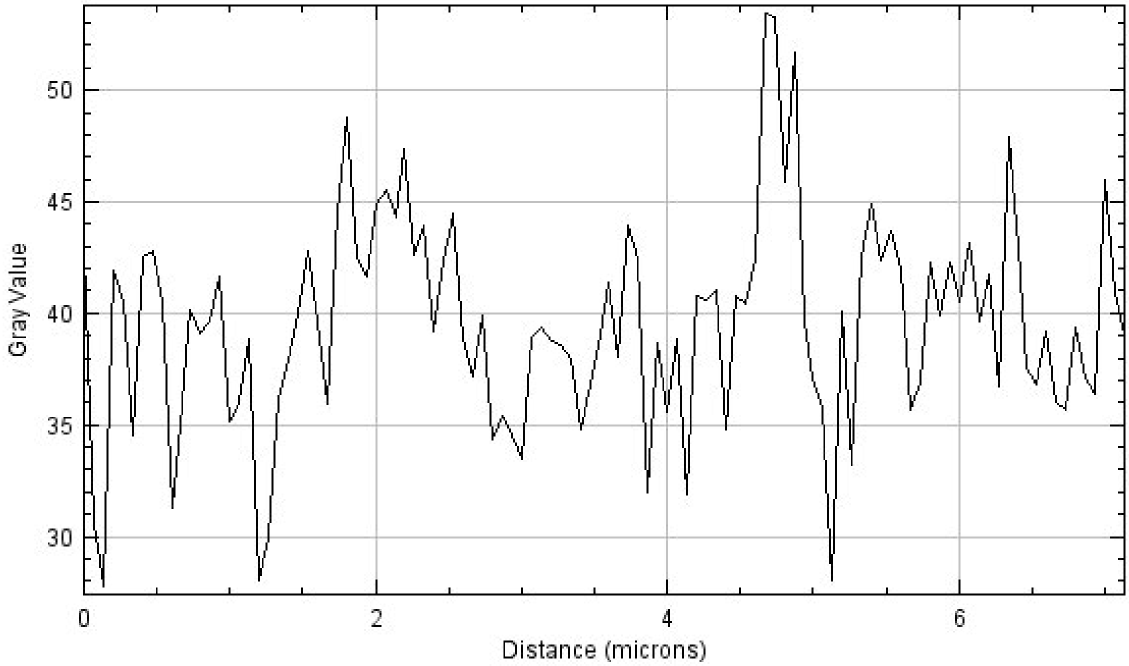

Figure S757 – Intensity profile of line scan generated for cell 18, Figure S739.

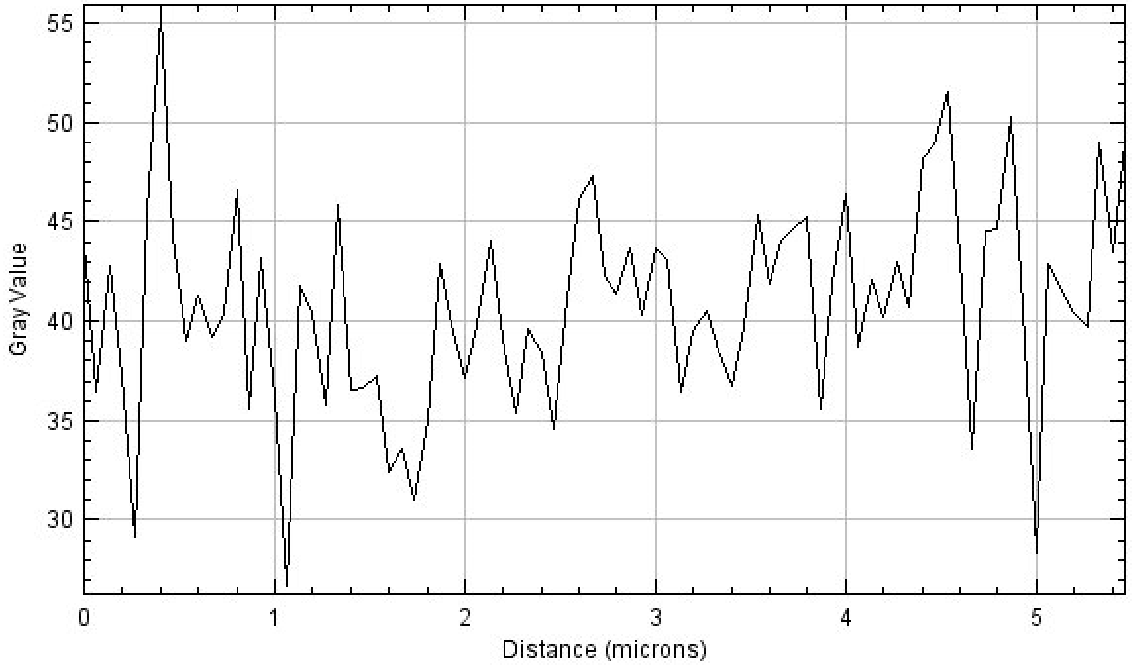

Figure S758 – Intensity profile of line scan generated for cell 19, Figure S739.

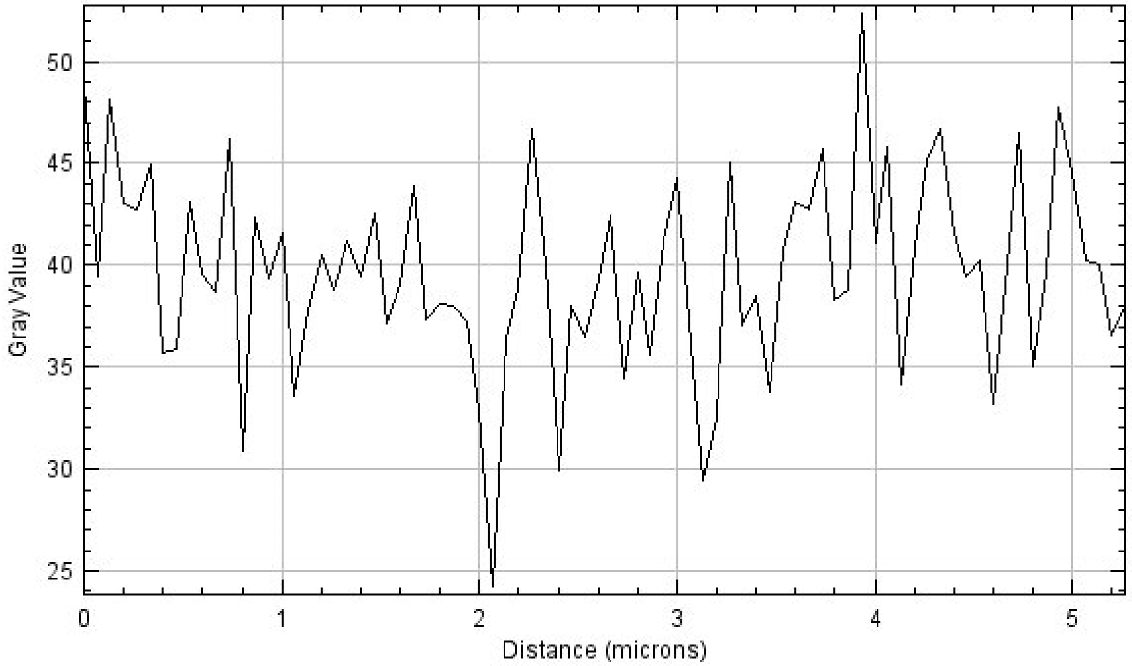

Figure S759 – Intensity profile of line scan generated for cell 20, Figure S739.

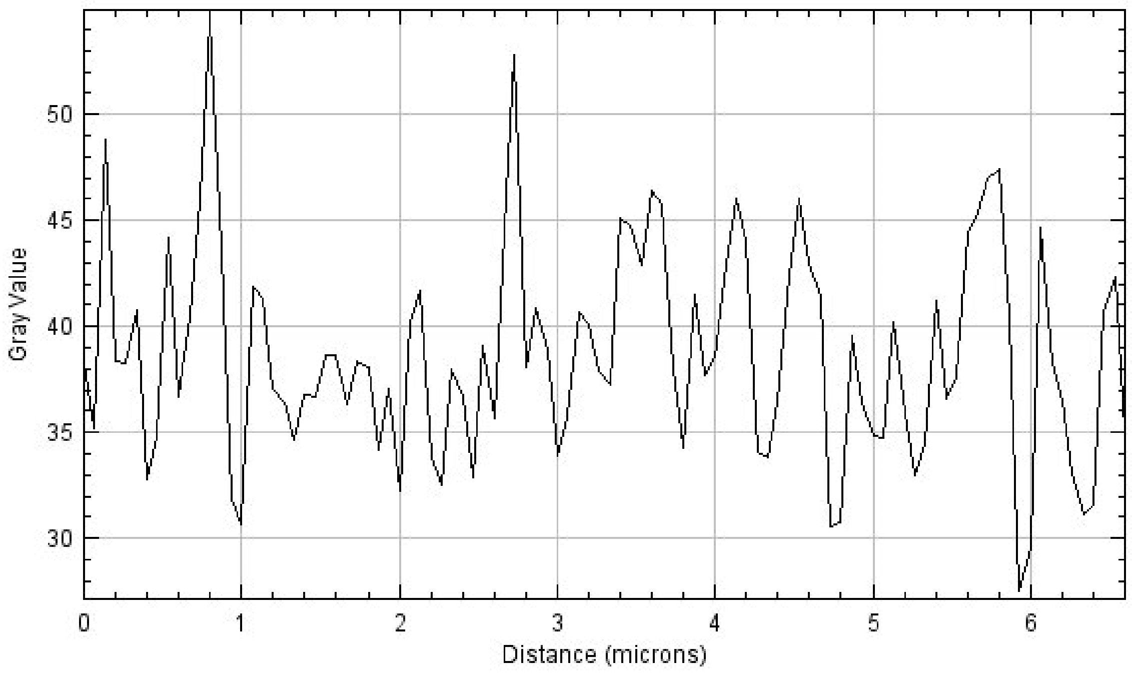

Figure S760 – Intensity profile of line scan generated for cell 21, Figure S739.

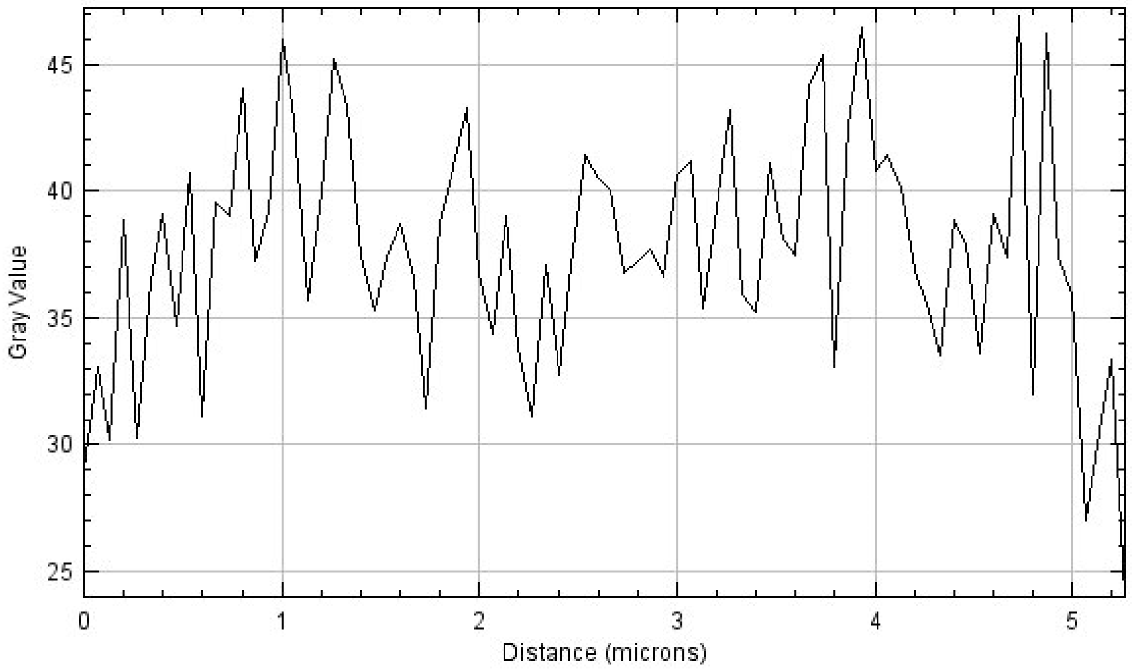

Figure S761 – Intensity profile of line scan generated for cell 22, Figure S739.

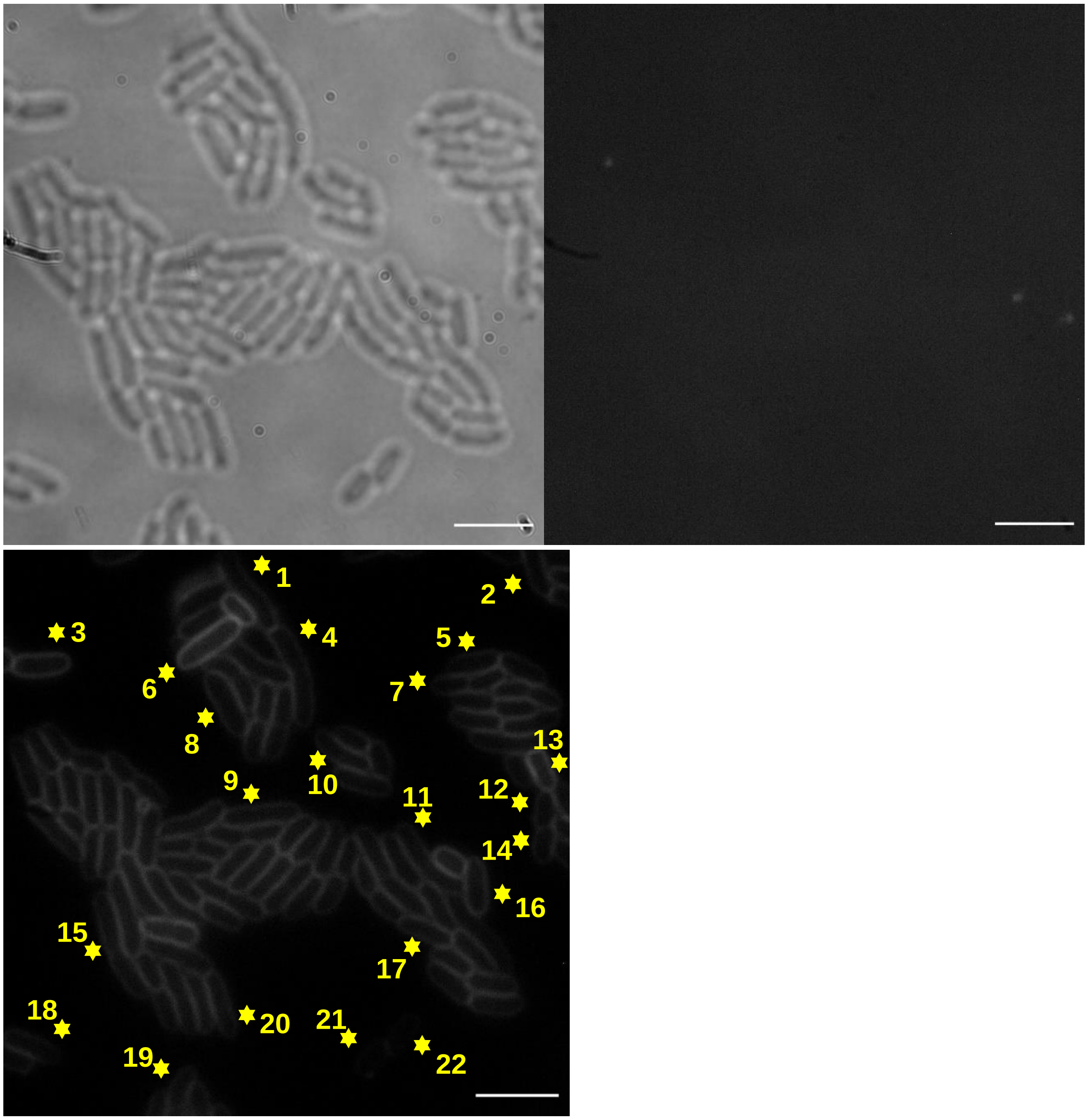

Figure S762 – *E. coli* transmitted and DAPI images used in fluorescence intensity calculations in the presence of FM4-64 at T = 30 minutes. mCherry image used to locate cells on DAPI image. Scale bar = 10 μM.

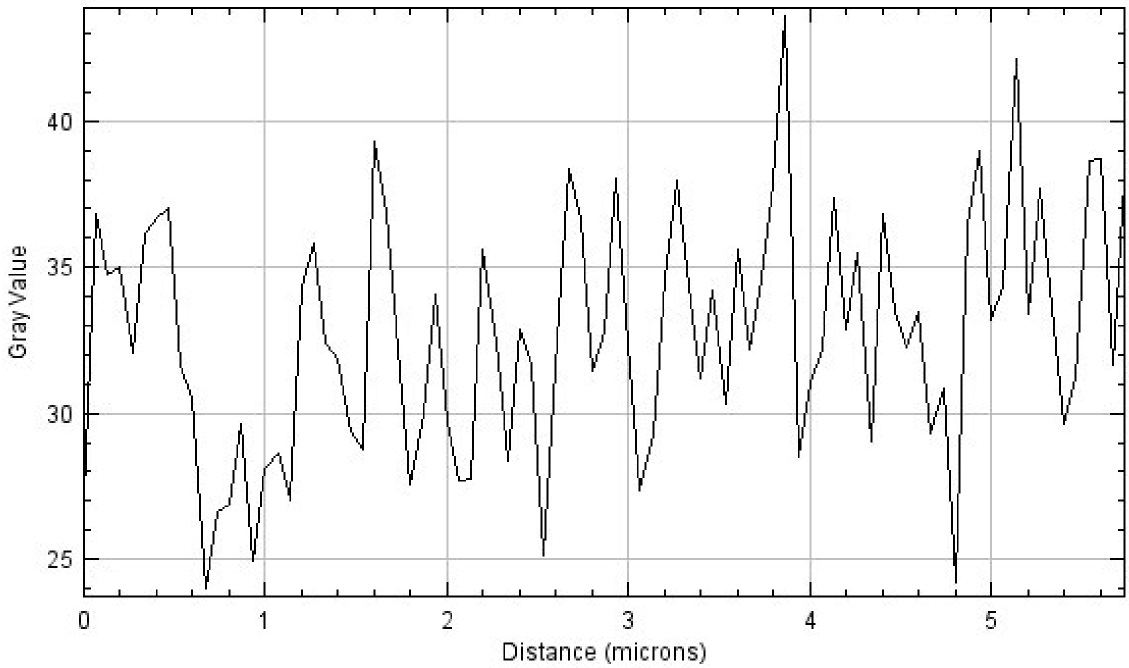

Figure S763 – Intensity profile of line scan generated for cell 1, Figure S762.

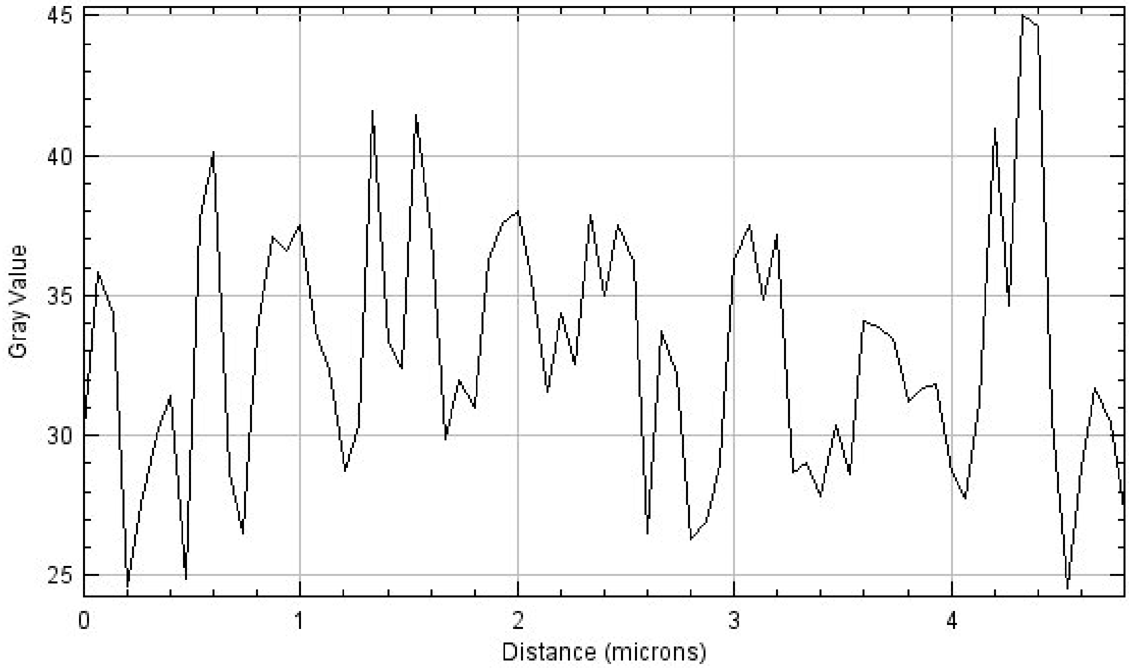

Figure S764 – Intensity profile of line scan generated for cell 2, Figure S762.

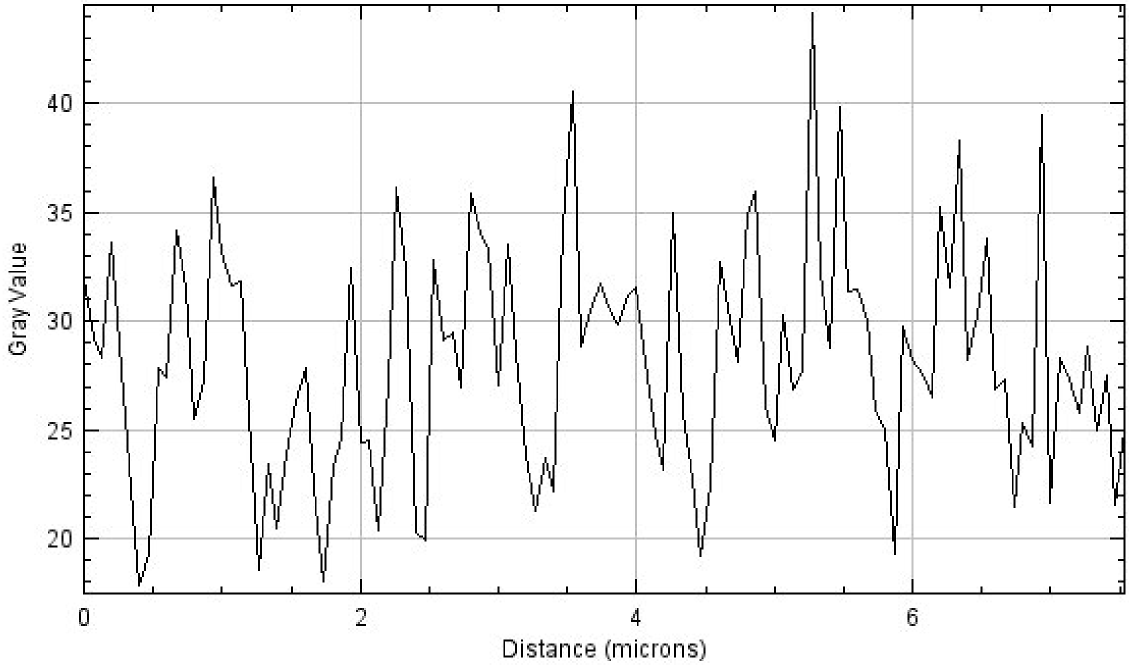

Figure S765 – Intensity profile of line scan generated for cell 3, Figure S762.

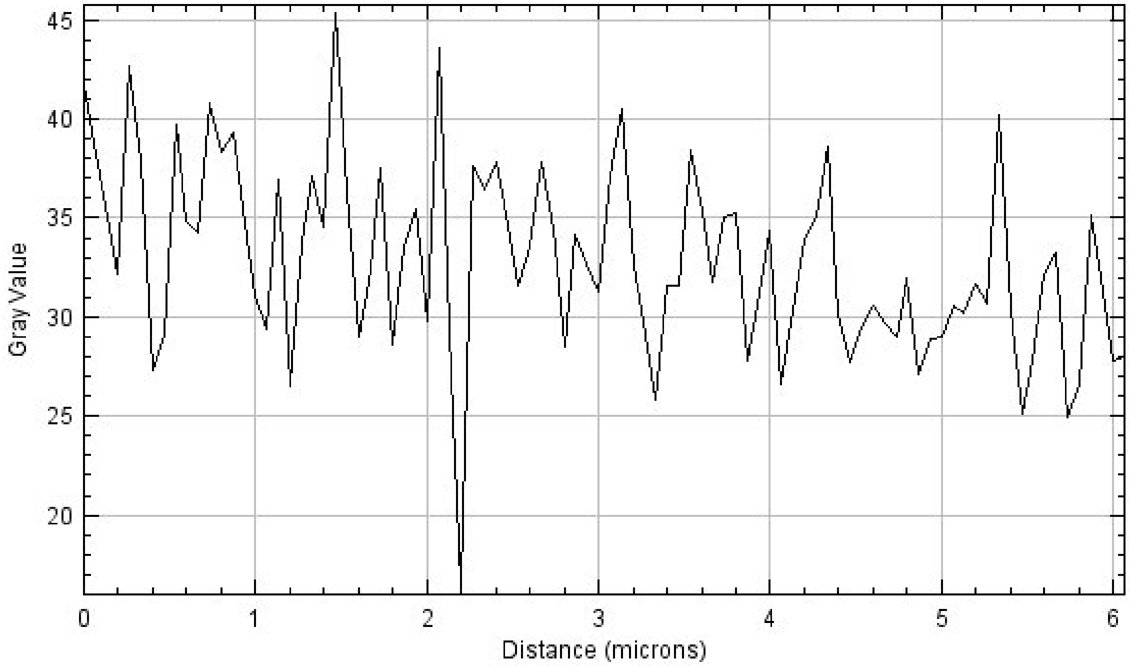

Figure S766 – Intensity profile of line scan generated for cell 4, Figure S762.

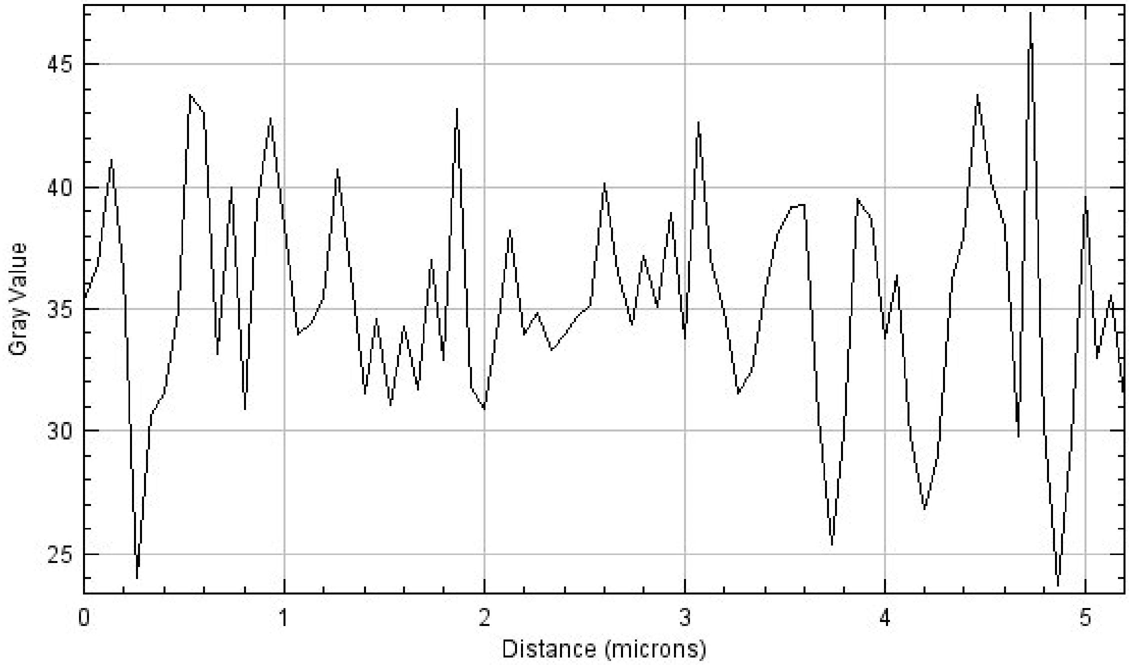

Figure S767 – Intensity profile of line scan generated for cell 5, Figure S762.

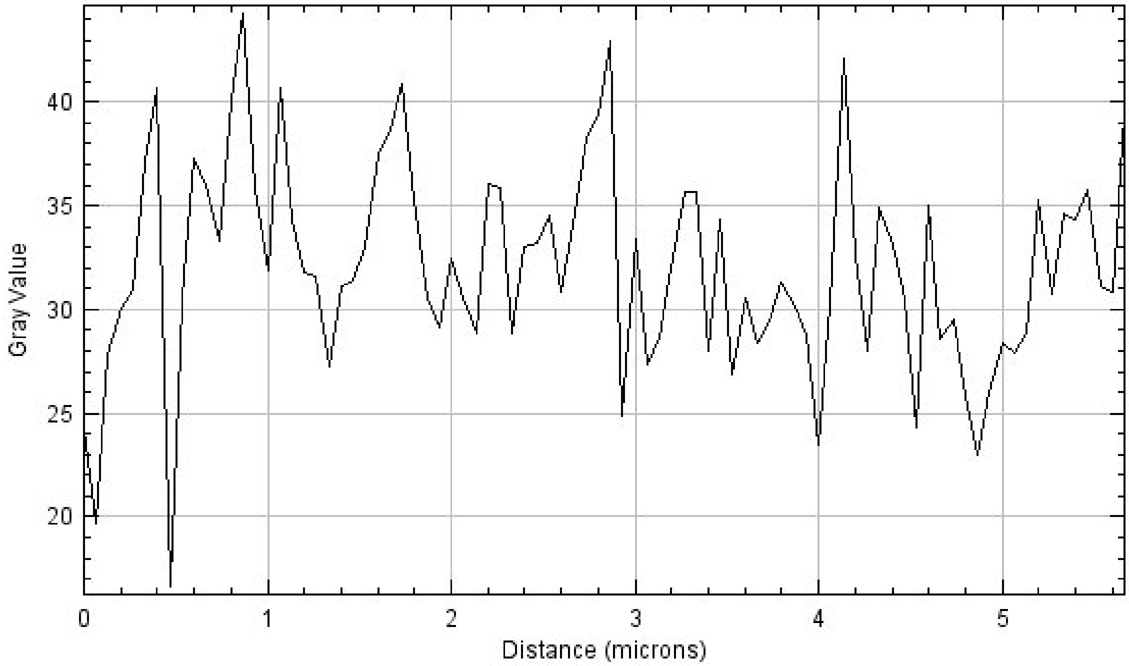

Figure S768 – Intensity profile of line scan generated for cell 6, Figure S762.

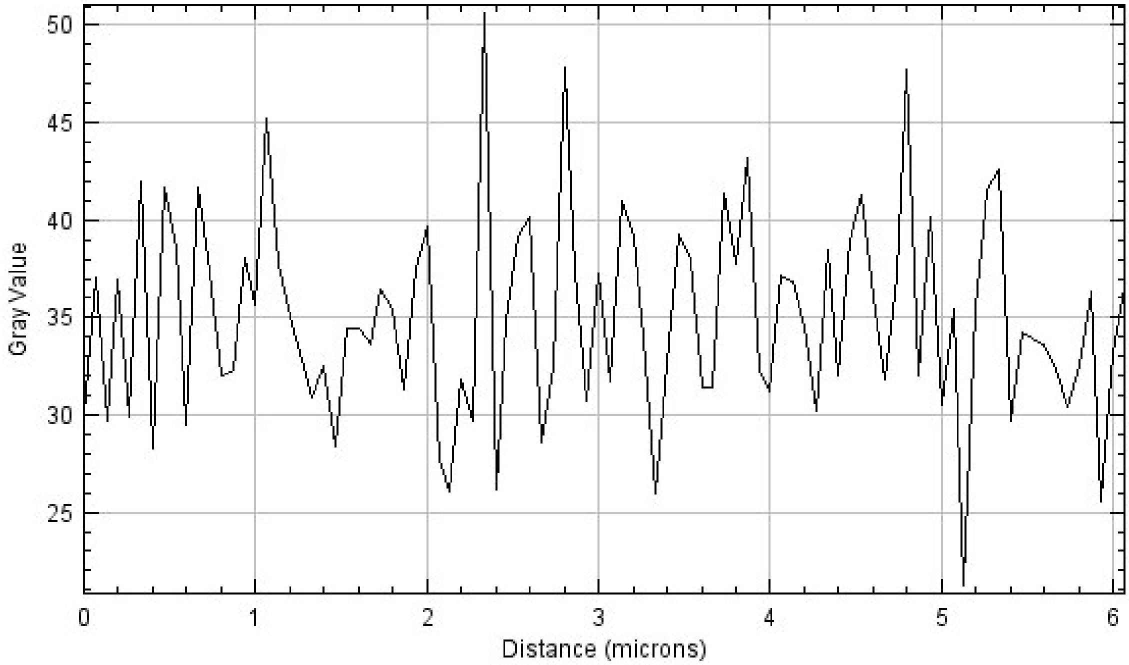

Figure S769 – Intensity profile of line scan generated for cell 7, Figure S762.

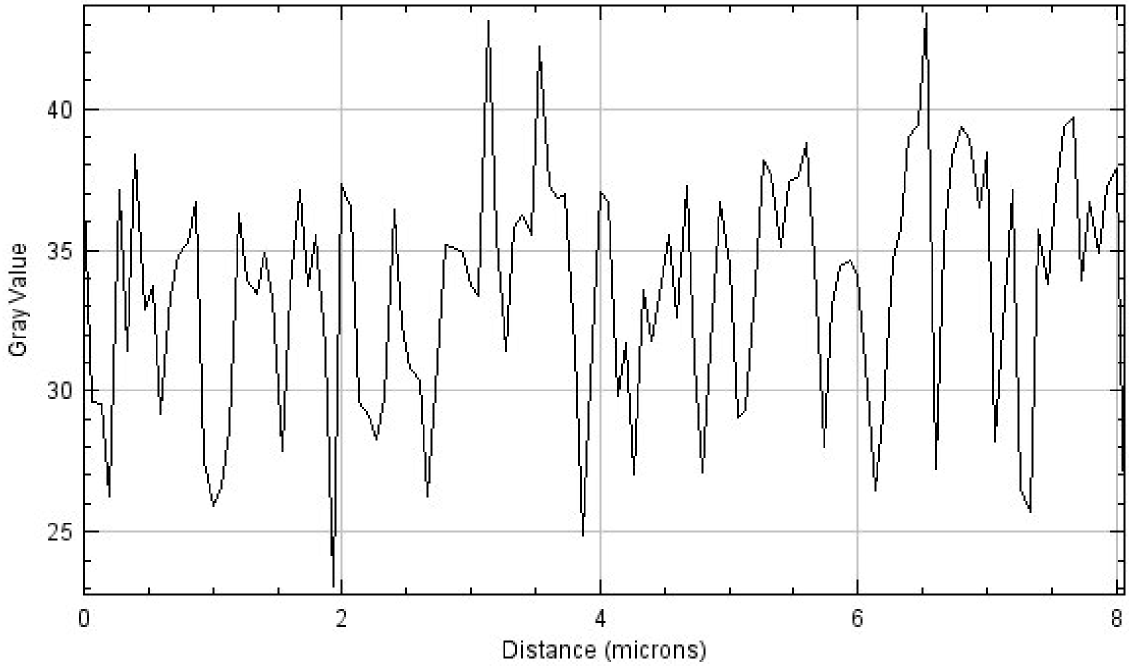

Figure S770 – Intensity profile of line scan generated for cell 8, Figure S762.

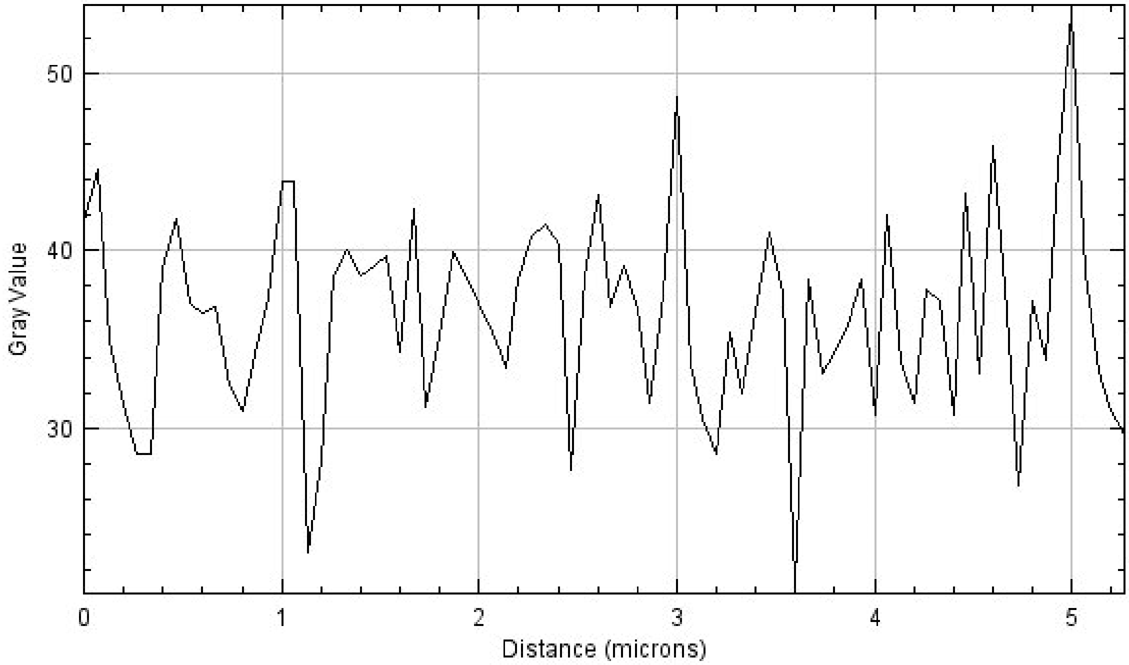

Figure S771 – Intensity profile of line scan generated for cell 9, Figure S762.

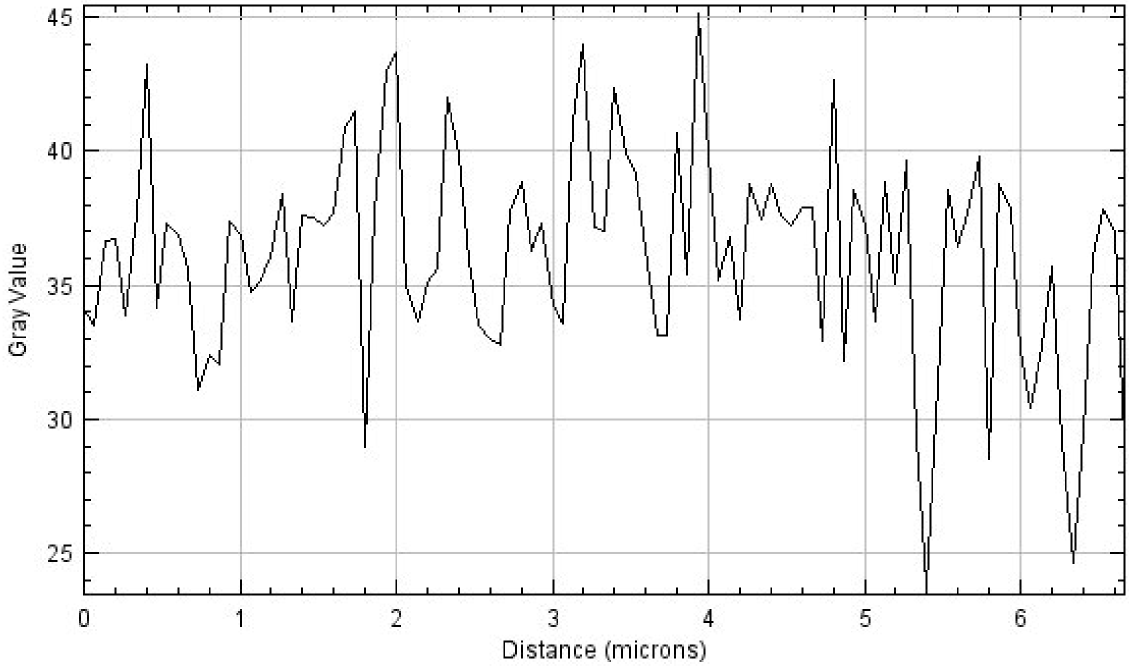

Figure S772 – Intensity profile of line scan generated for cell 10, Figure S762.

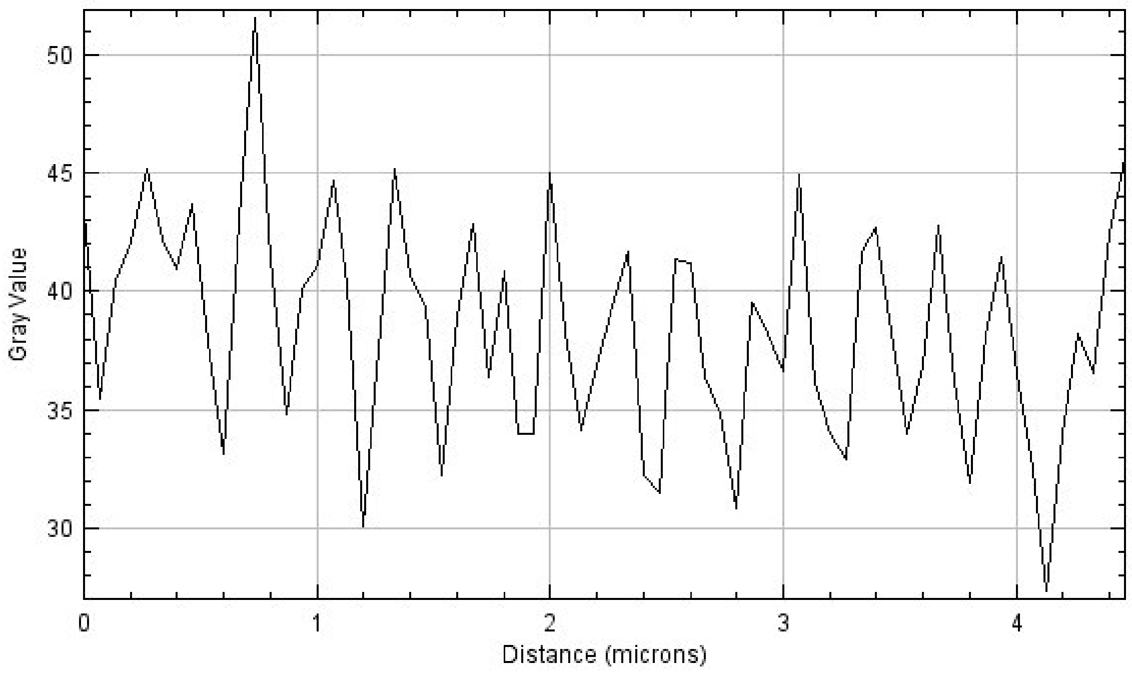

Figure S773 – Intensity profile of line scan generated for cell 11, Figure S762.

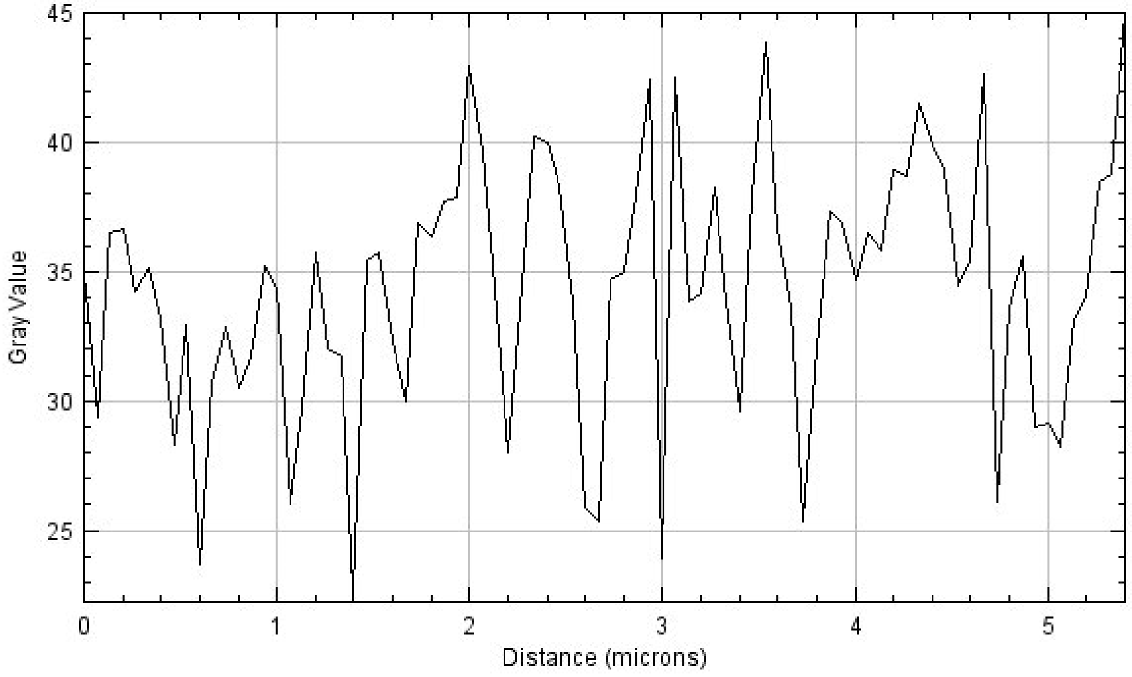

Figure S774 – Intensity profile of line scan generated for cell 12, Figure S762.

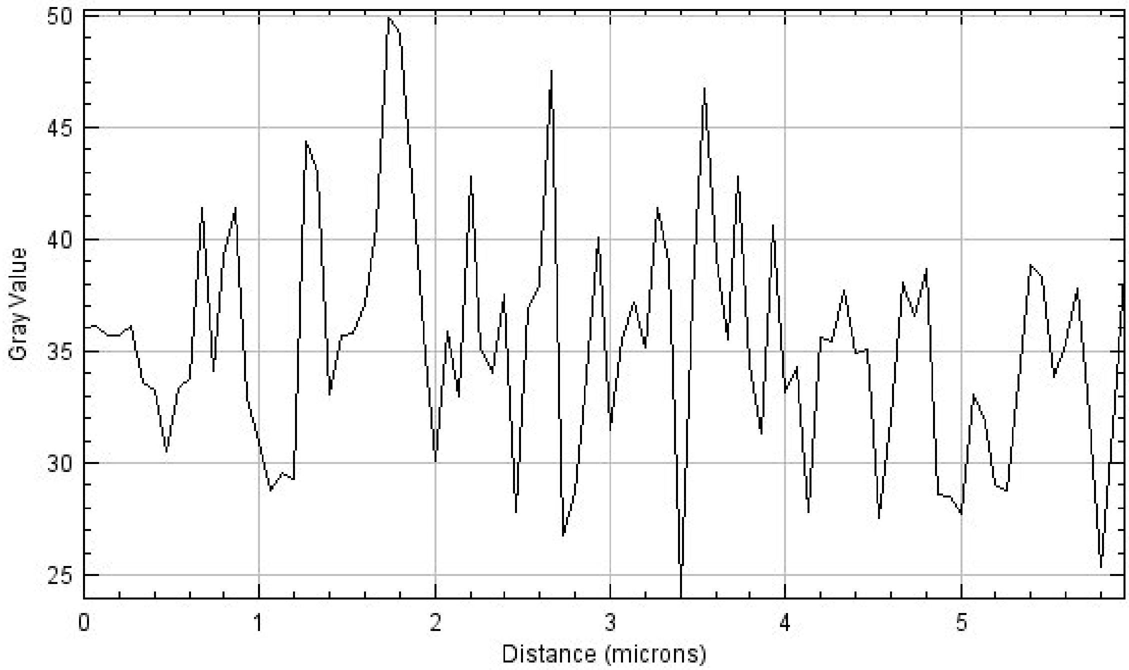

Figure S775 – Intensity profile of line scan generated for cell 13, Figure S762.

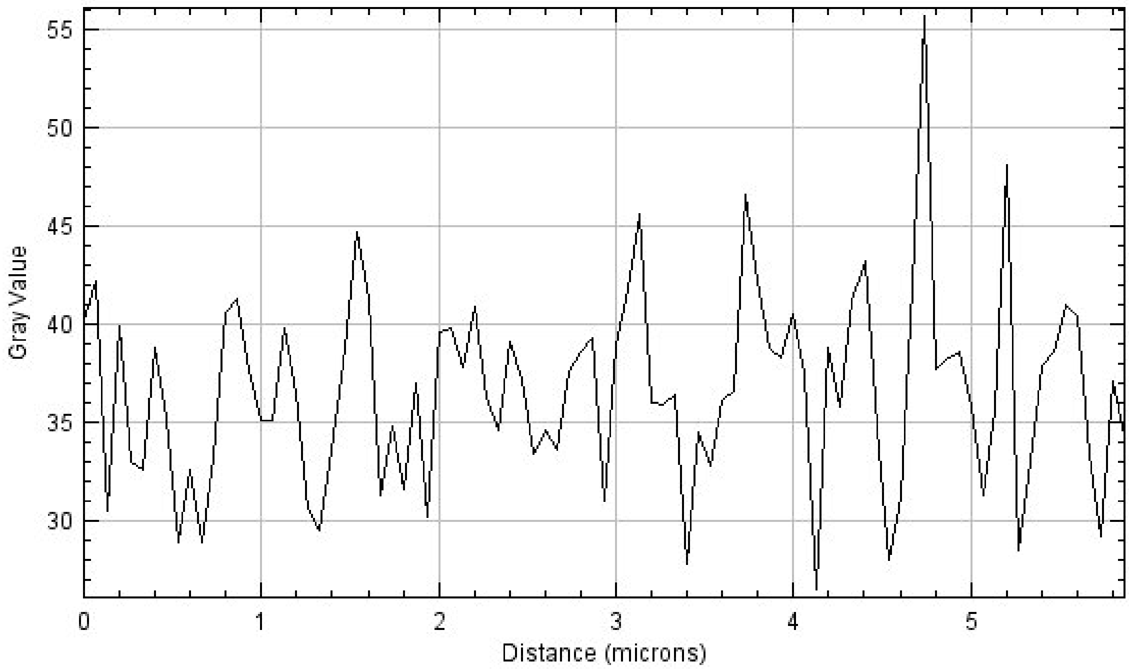

Figure S776 – Intensity profile of line scan generated for cell 14, Figure S762.

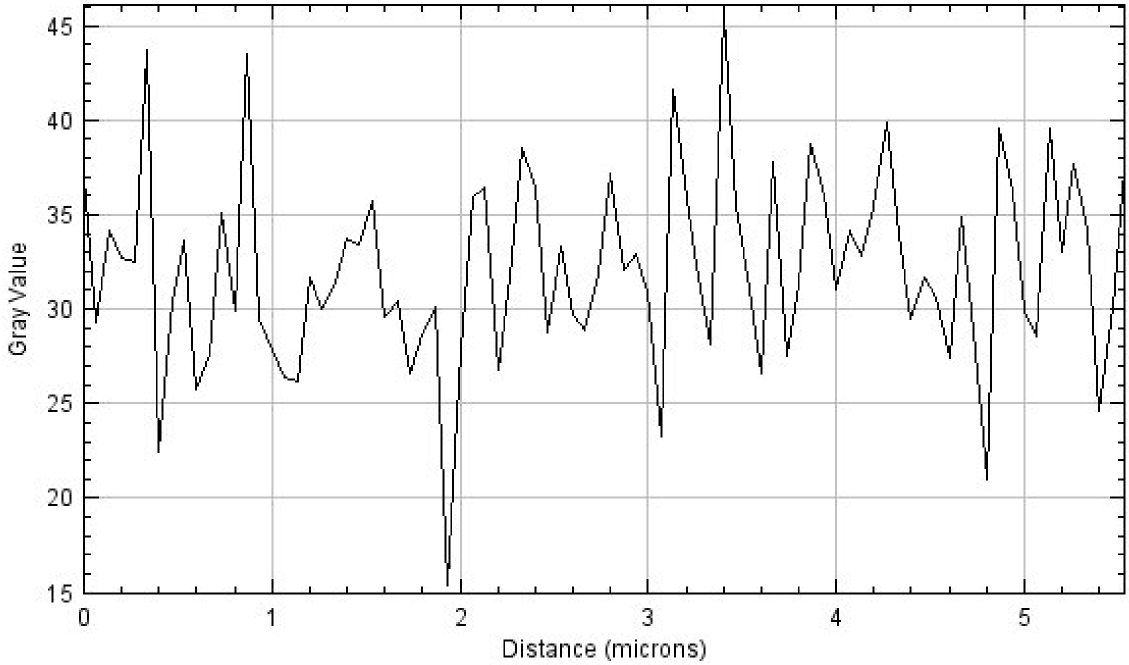

Figure S777 – Intensity profile of line scan generated for cell 15, Figure S762.

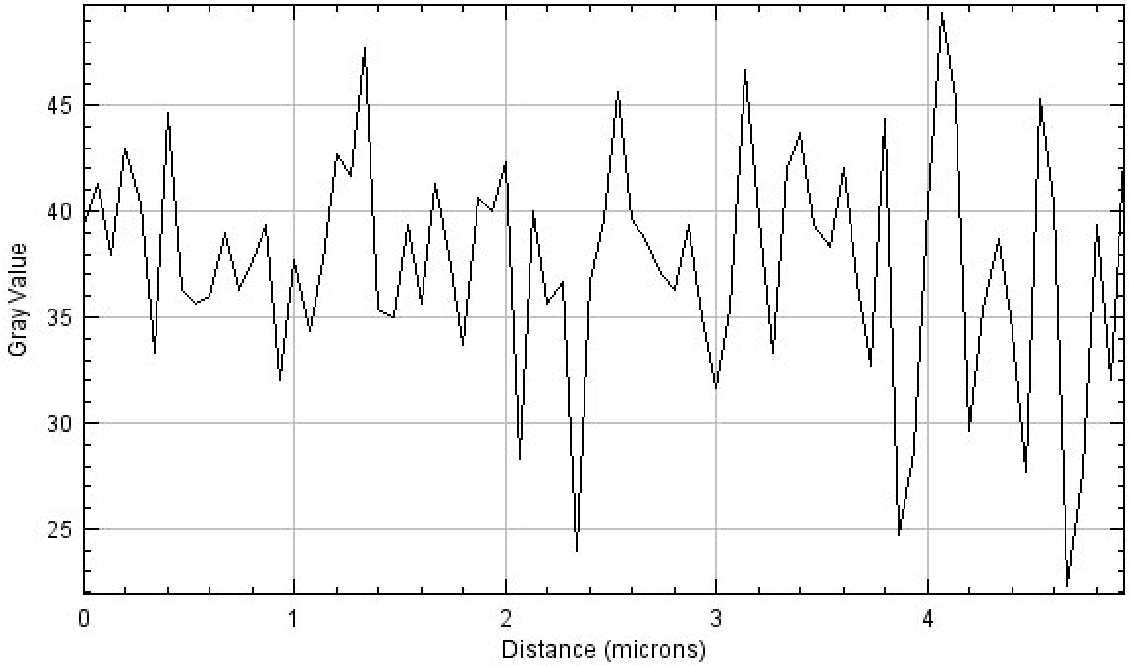

Figure S778 – Intensity profile of line scan generated for cell 16, Figure S762.

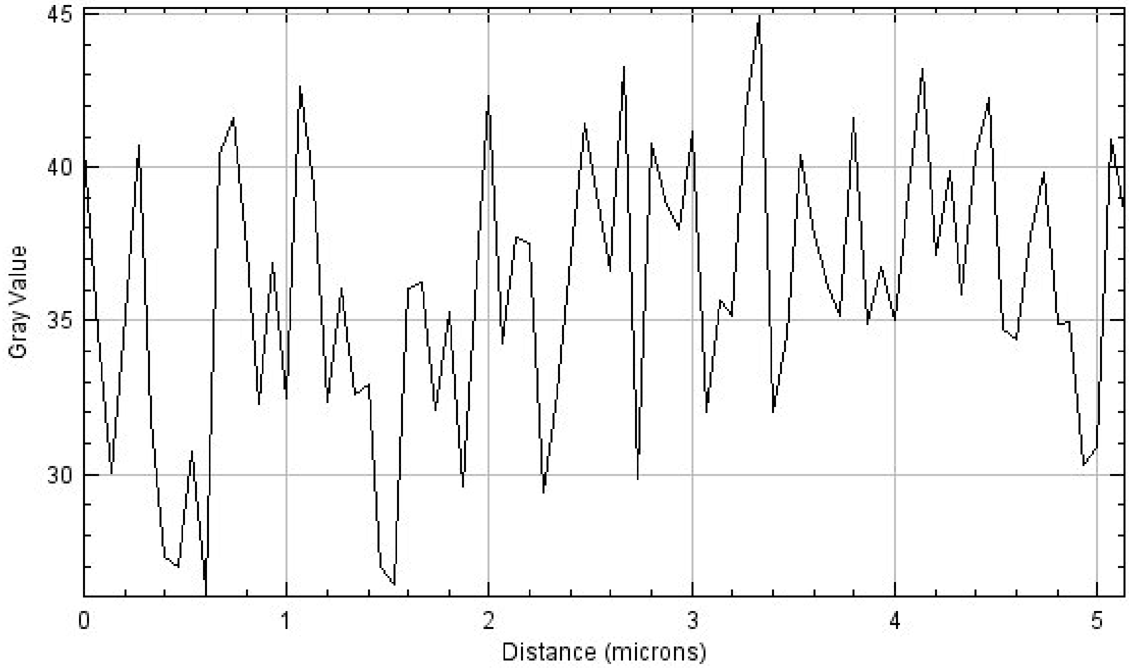

Figure S779 – Intensity profile of line scan generated for cell 17, Figure S762.

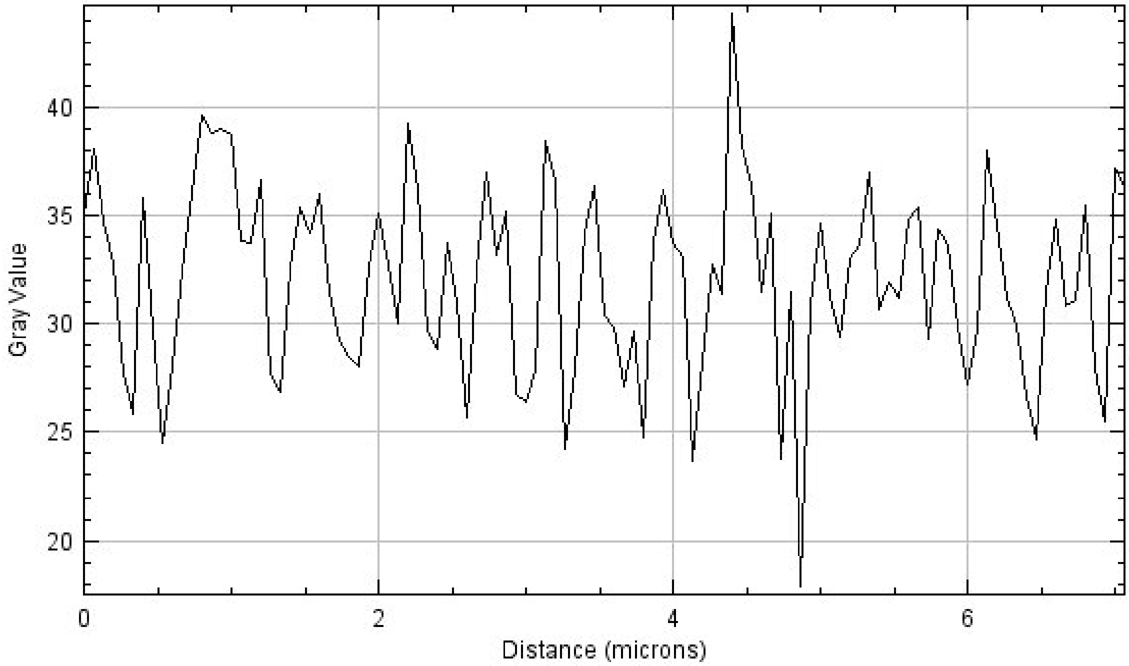

Figure S780 – Intensity profile of line scan generated for cell 18, Figure S762.

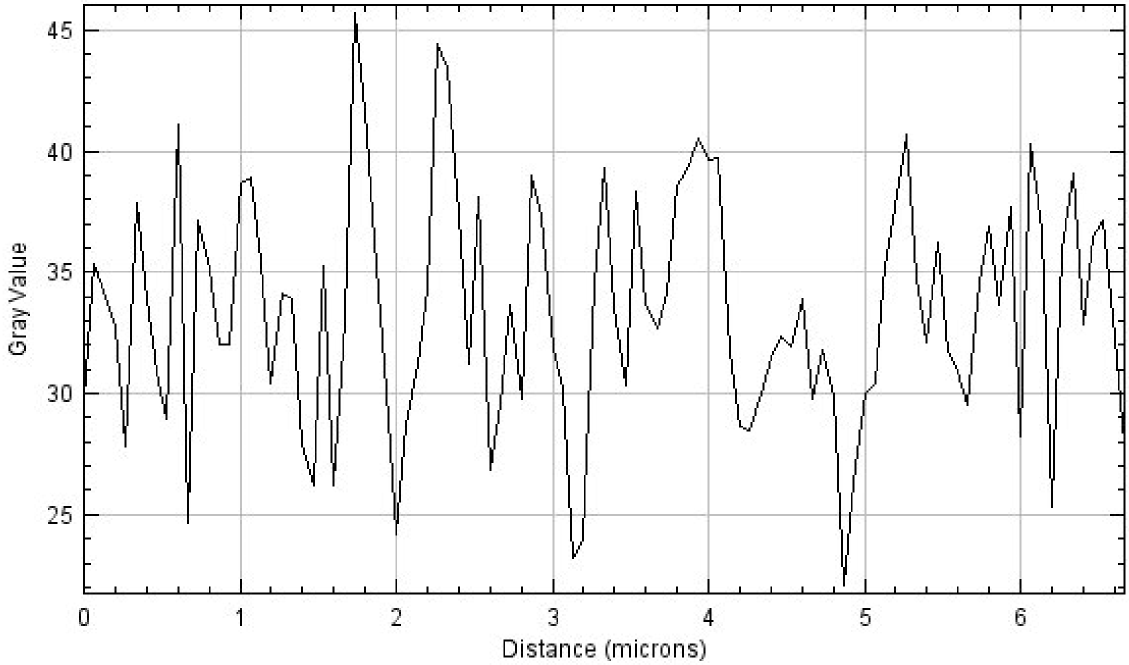

Figure S781 – Intensity profile of line scan generated for cell 19, Figure S762.

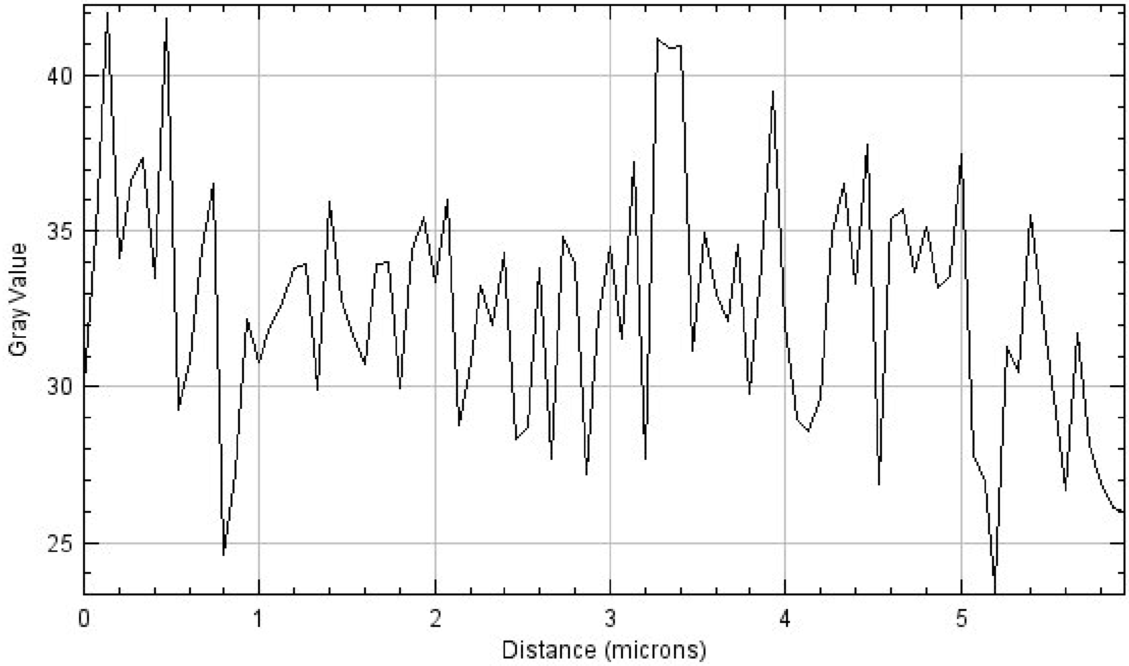

Figure S782 – Intensity profile of line scan generated for cell 20, Figure S762.

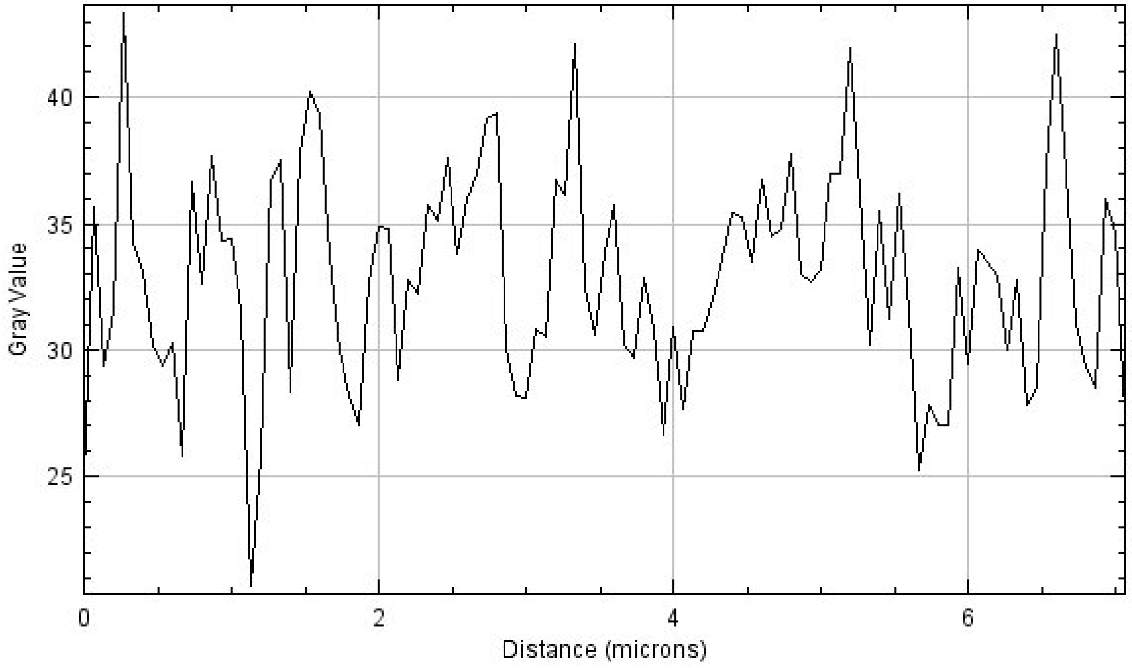

Figure S783 – Intensity profile of line scan generated for cell 21, Figure S762.

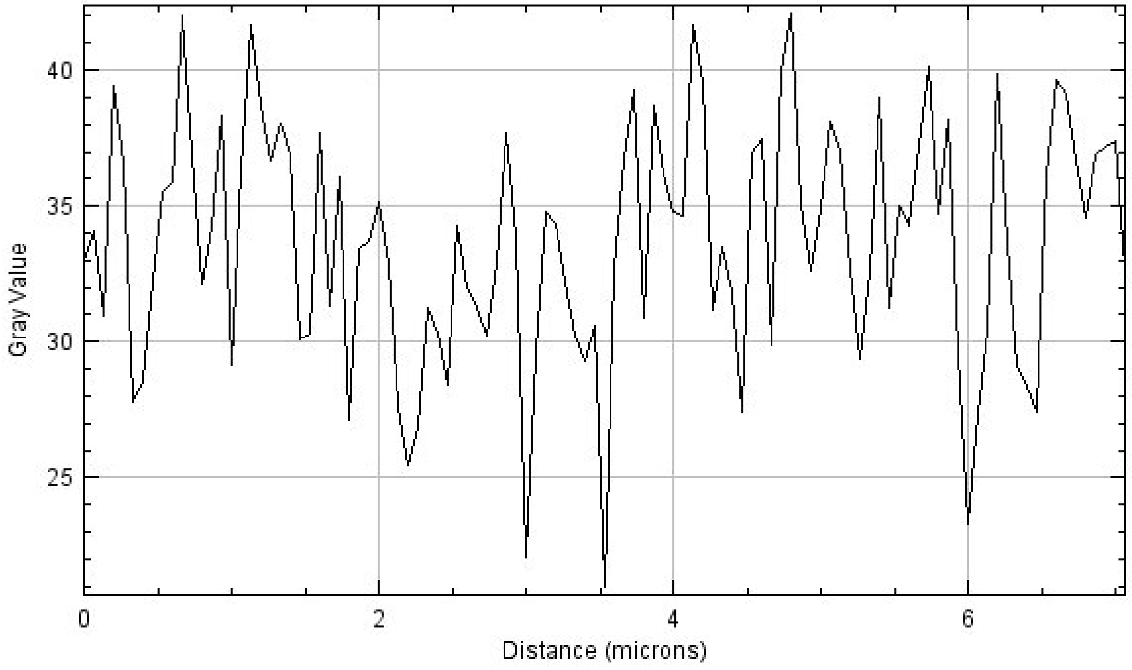

Figure S784 – Intensity profile of line scan generated for cell 22, Figure S762.

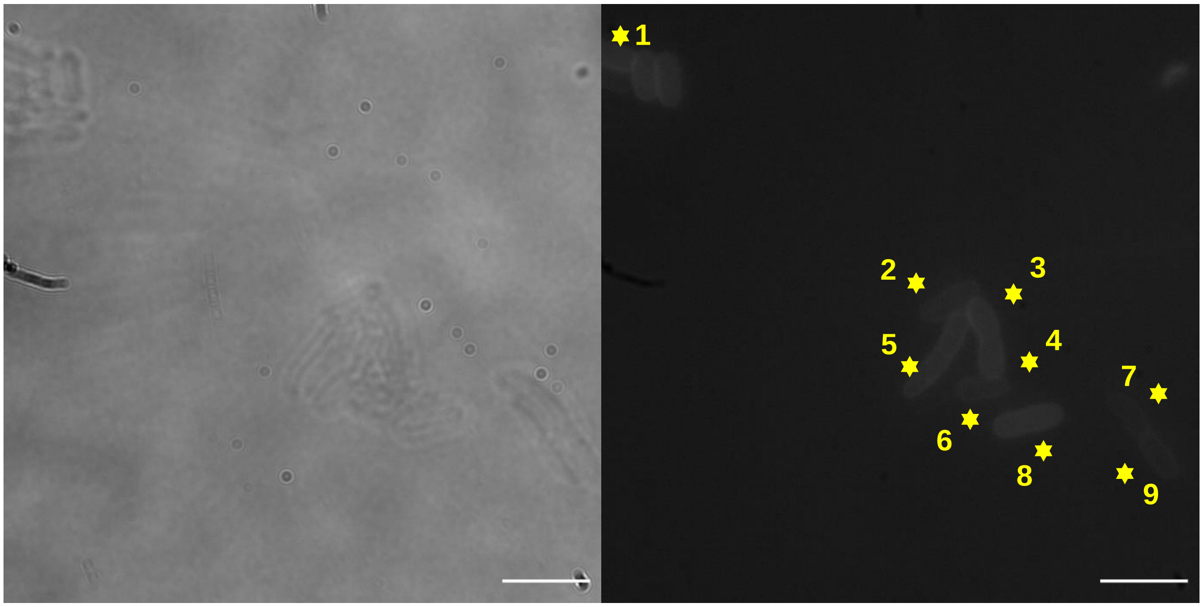

Figure S785 – *E. coli* transmitted and DAPI images used in fluorescence intensity calculations in the presence of both compound **39** and FM4-64 at T = 30 minutes. Scale bar = 10 µM.

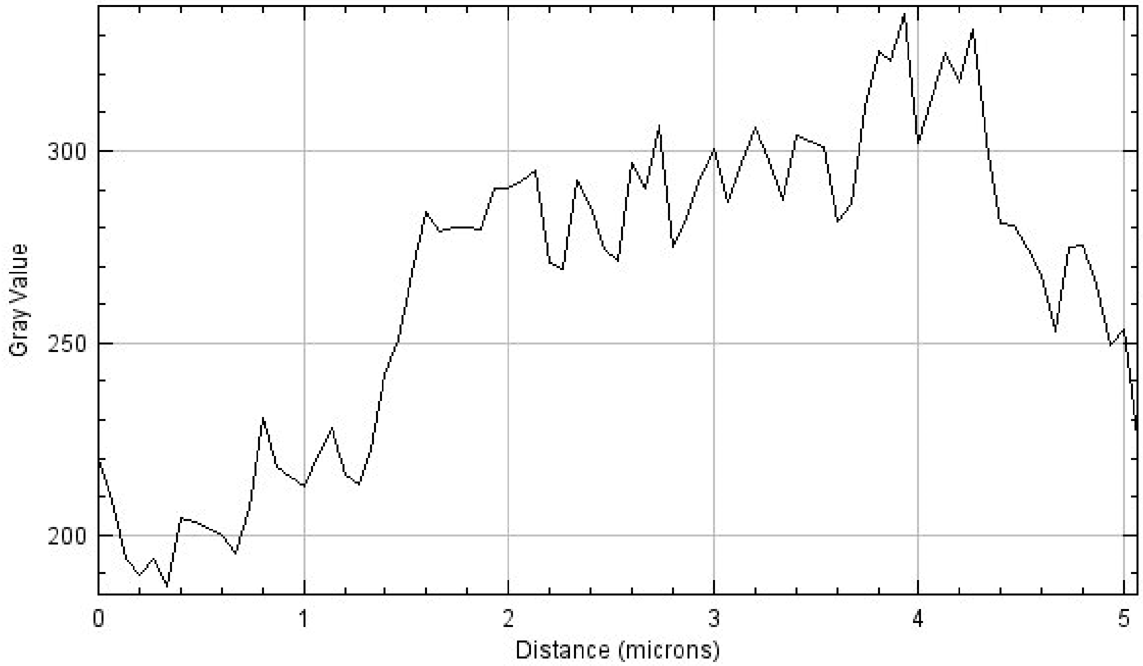

Figure S786 – Intensity profile of line scan generated for cell 1, Figure S785.

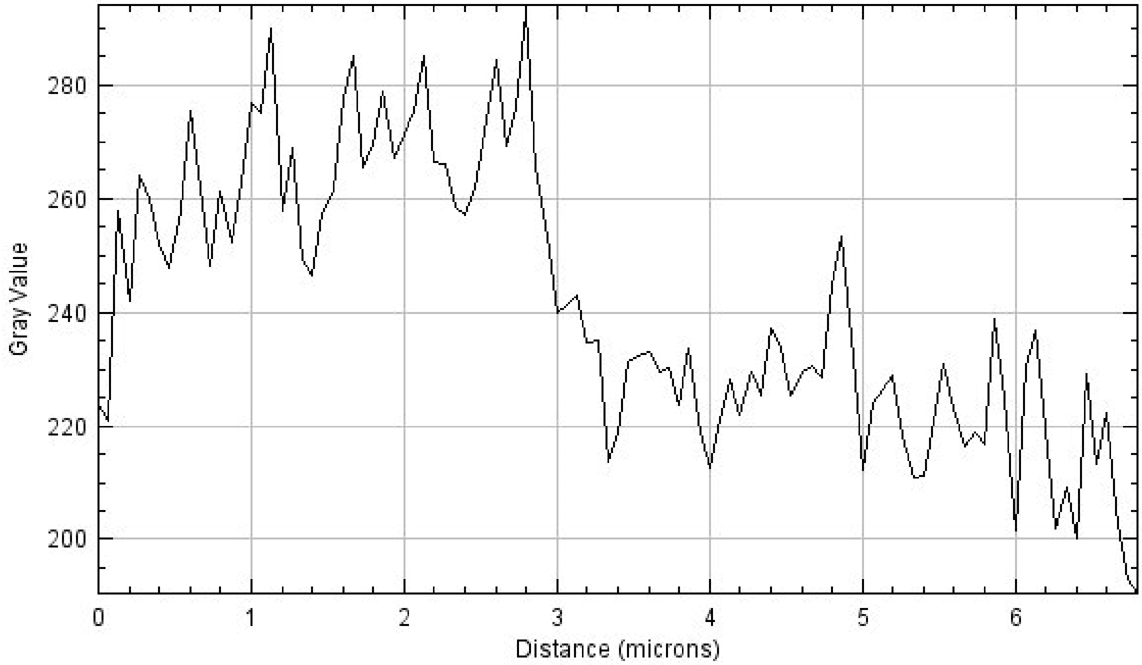

Figure S787 – Intensity profile of line scan generated for cell 2, Figure S785.

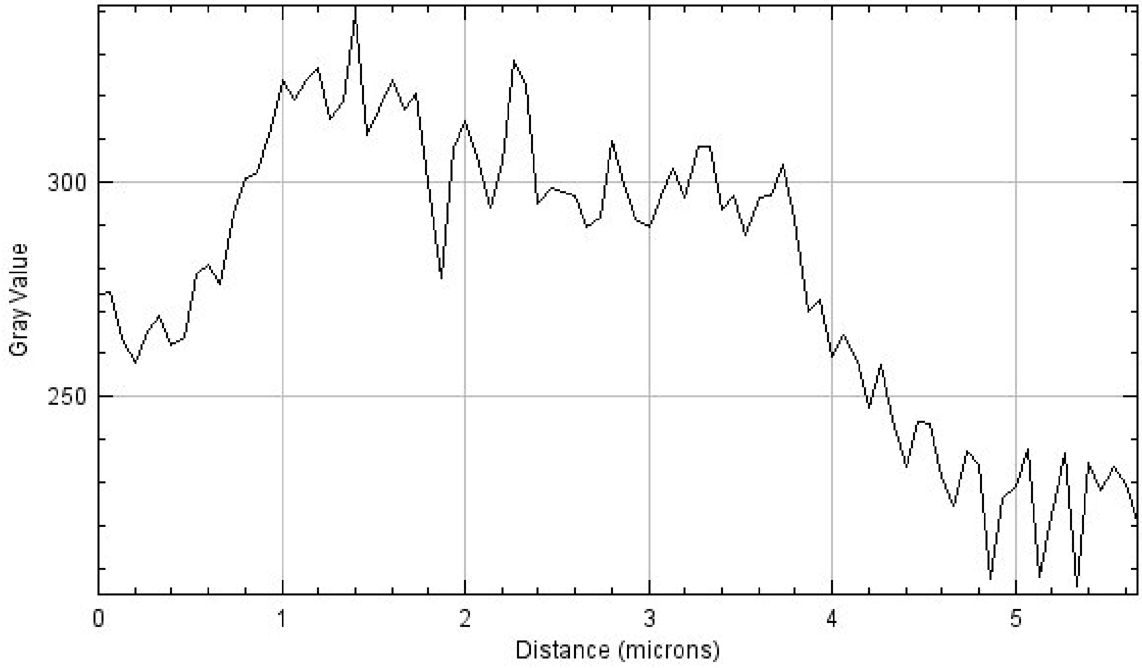

Figure S788 – Intensity profile of line scan generated for cell 3, Figure S785.

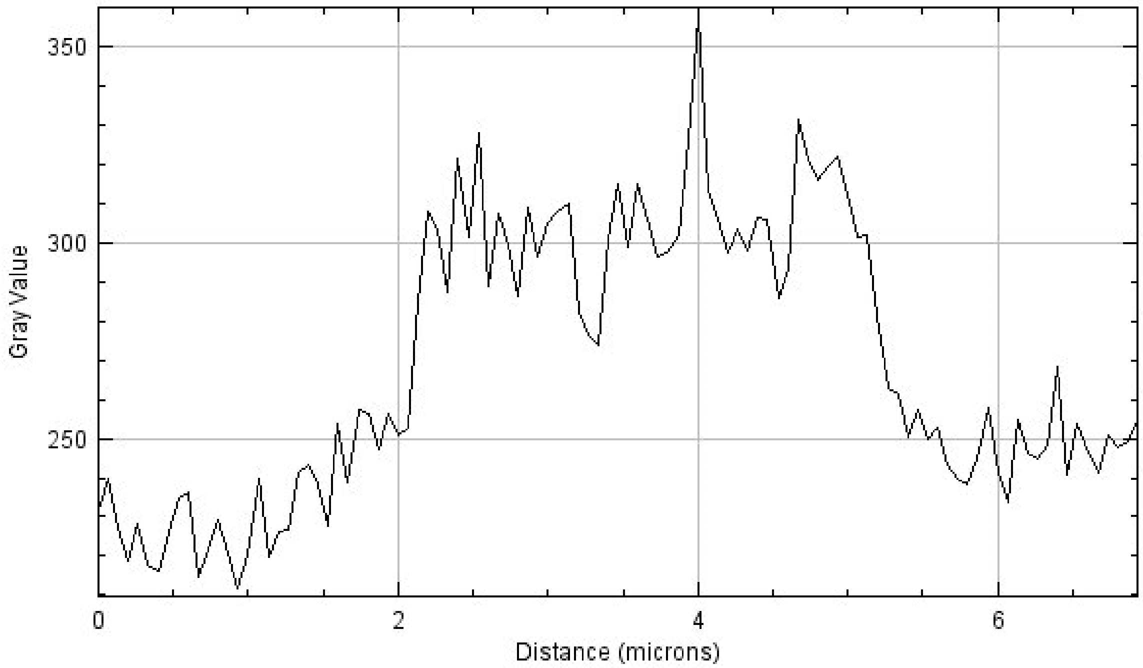

Figure S789 – Intensity profile of line scan generated for cell 4 , Figure S785.

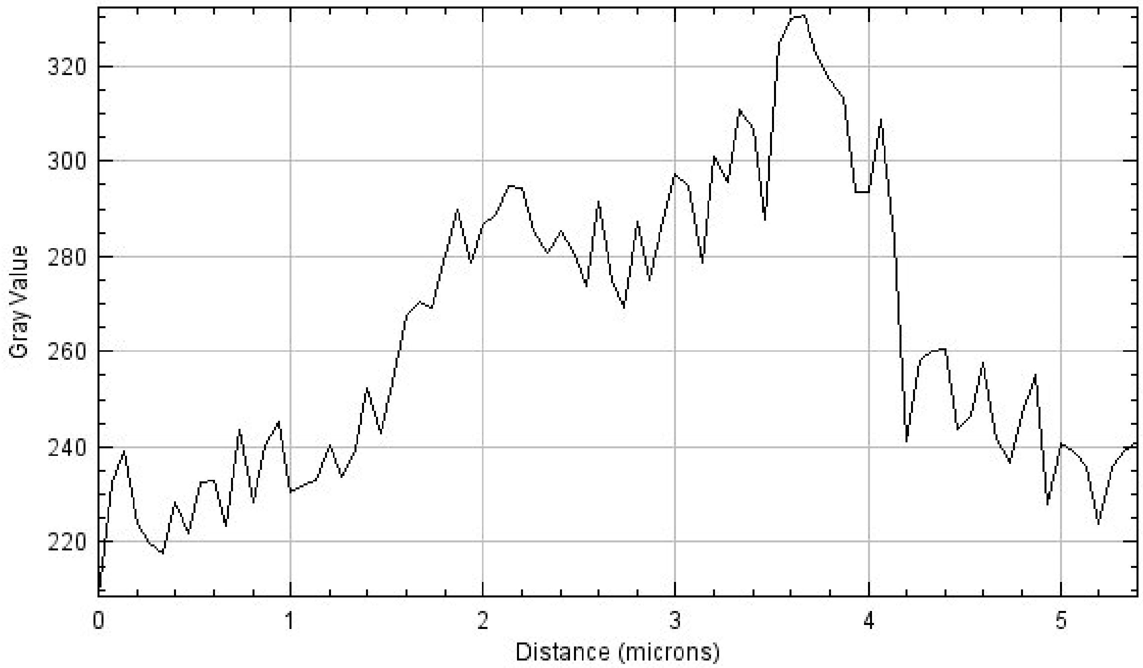

Figure S790 – Intensity profile of line scan generated for cell 5, Figure S785.

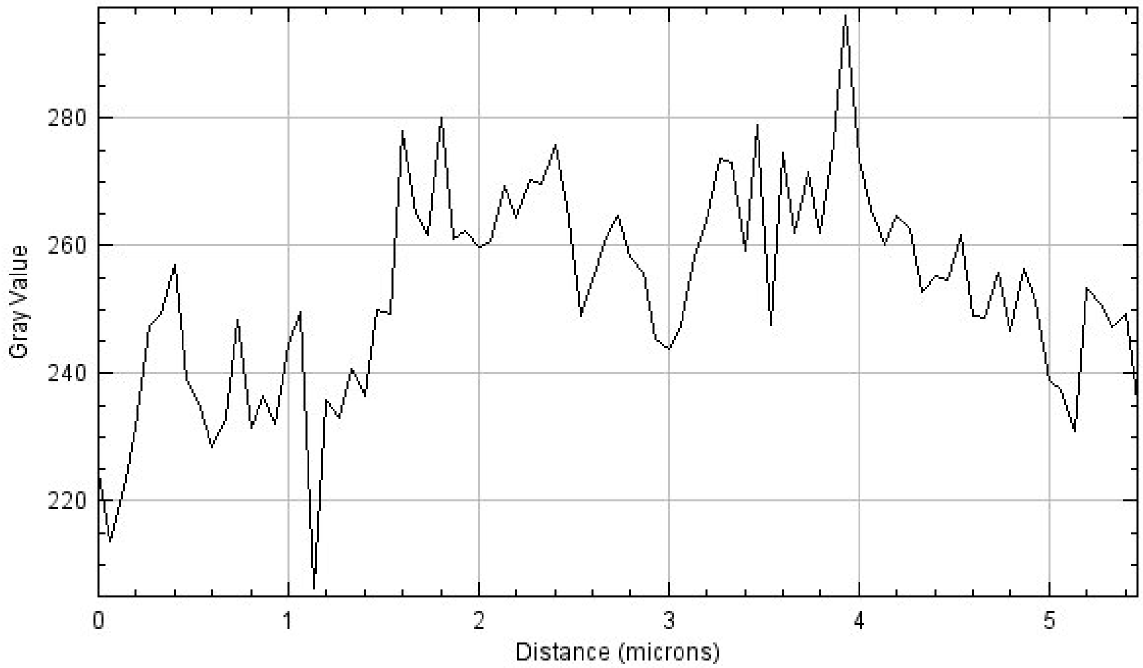

Figure S791 – Intensity profile of line scan generated for cell 6, Figure S785.

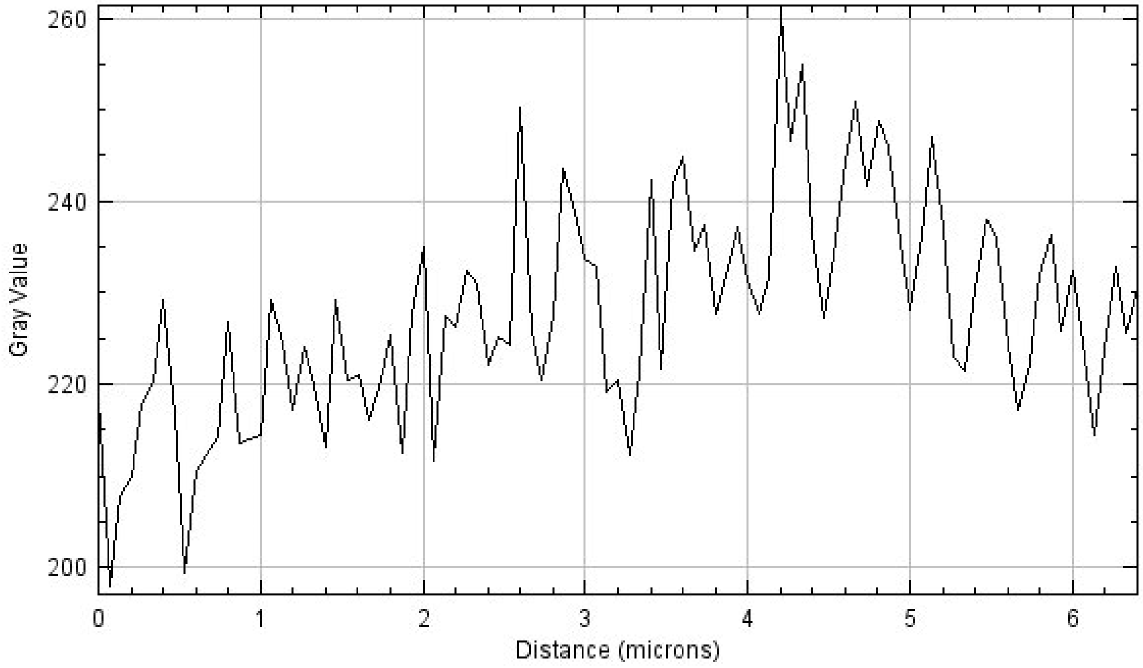

Figure S792 – Intensity profile of line scan generated for cell 7, Figure S785.

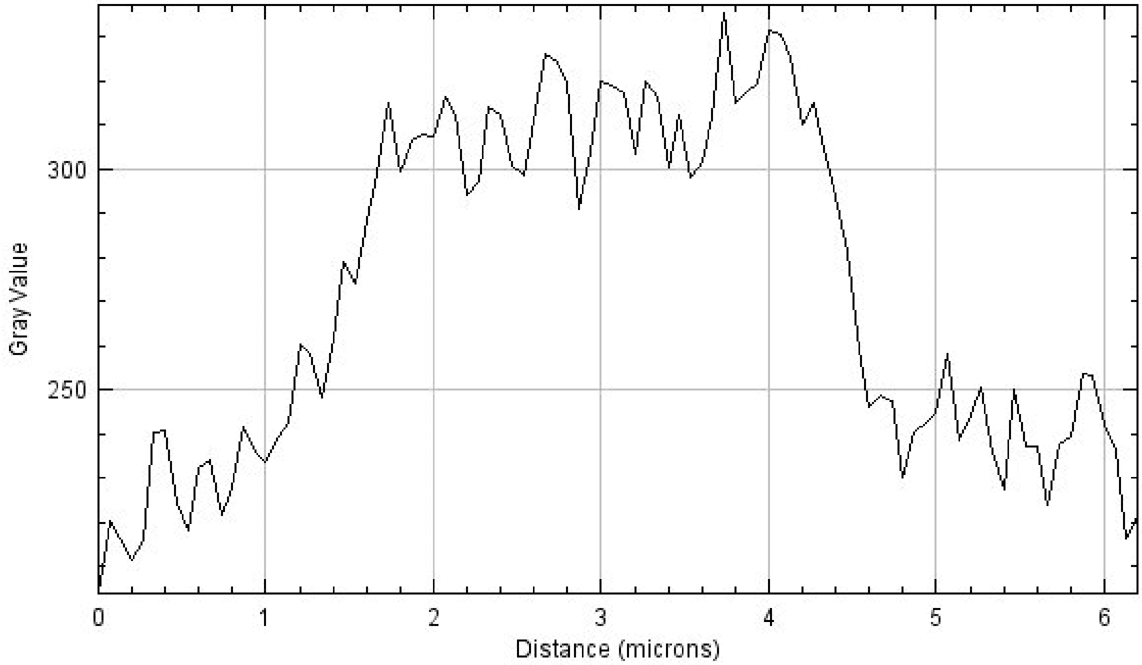

Figure S793 – Intensity profile of line scan generated for cell 8, Figure S785.

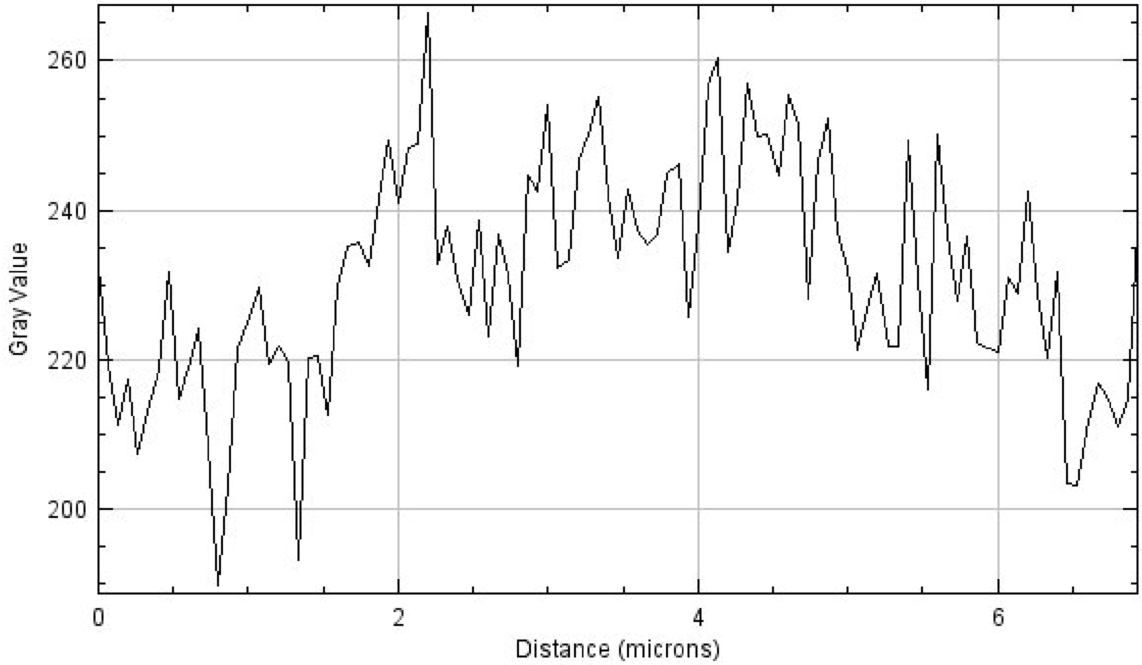

Figure S794 – Intensity profile of line scan generated for cell 9, Figure S785.

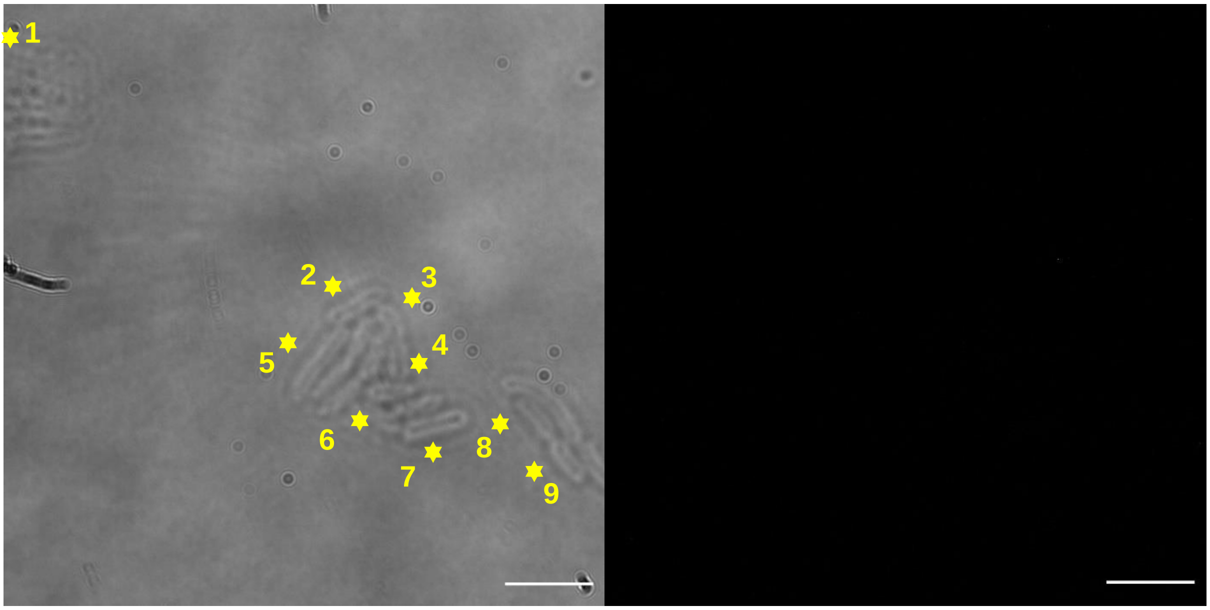

Figure S795 – *E. coli* transmitted and mCherry images used in fluorescence intensity calculations in the presence of both compound **39** and FM4-64 at T = 30 minutes. DAPI and transmitted images used to locate cells on mCherry image. Scale bar = 10 μM.

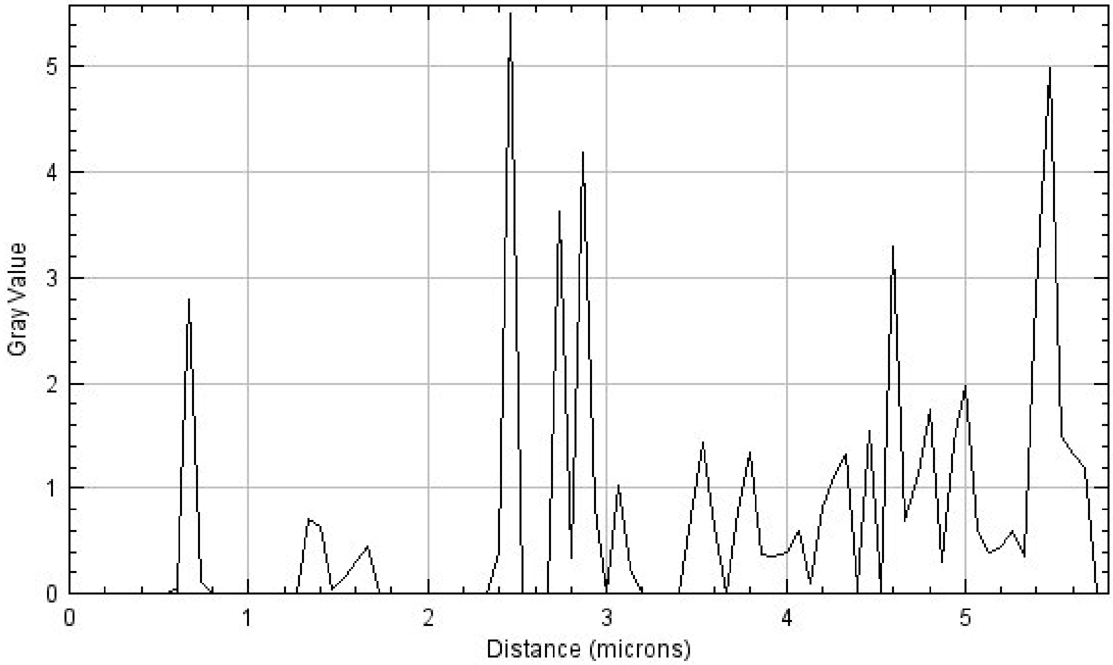

Figure S796 – Intensity profile of line scan generated for cell 1, Figure S795.

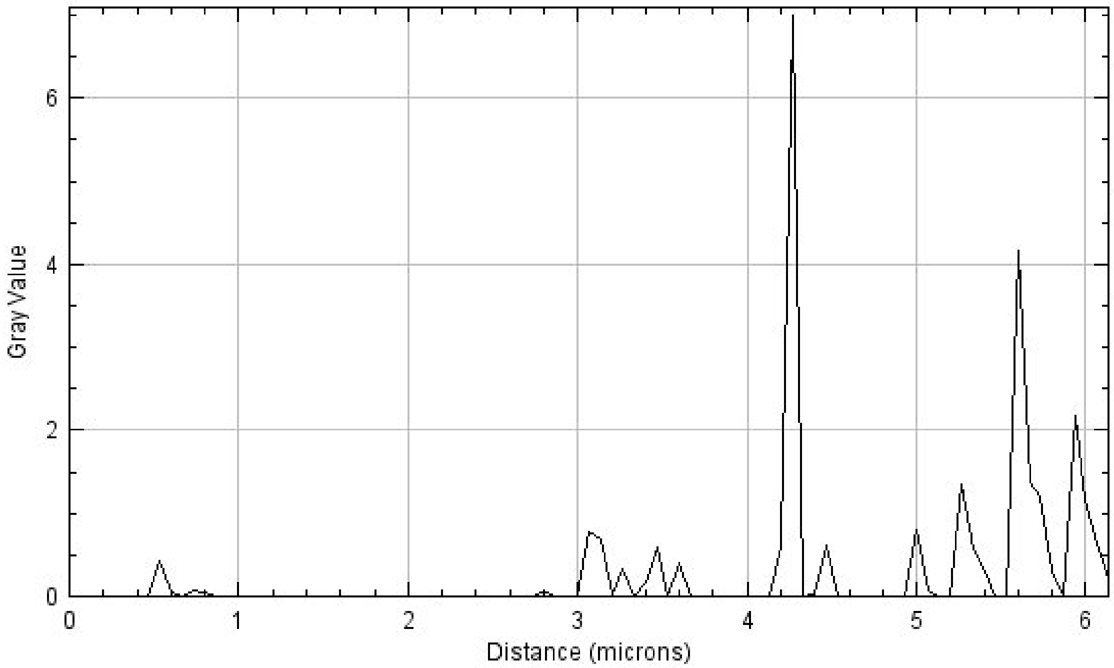

Figure S797 – Intensity profile of line scan generated for cell 2, Figure S795.

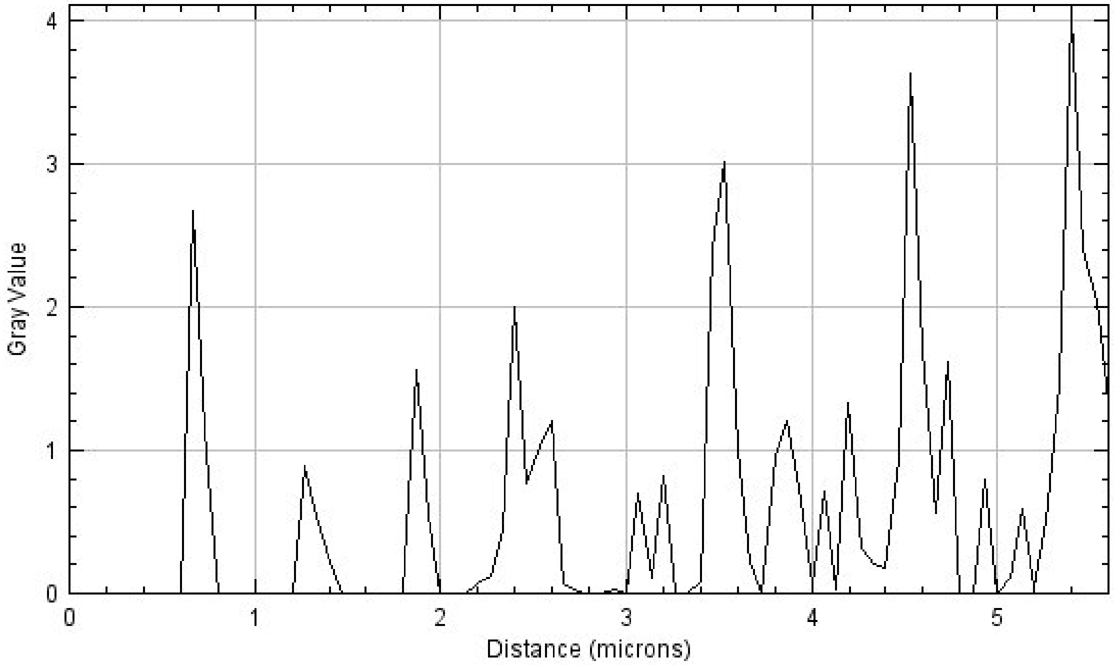

Figure S798 – Intensity profile of line scan generated for cell 3, Figure S795.

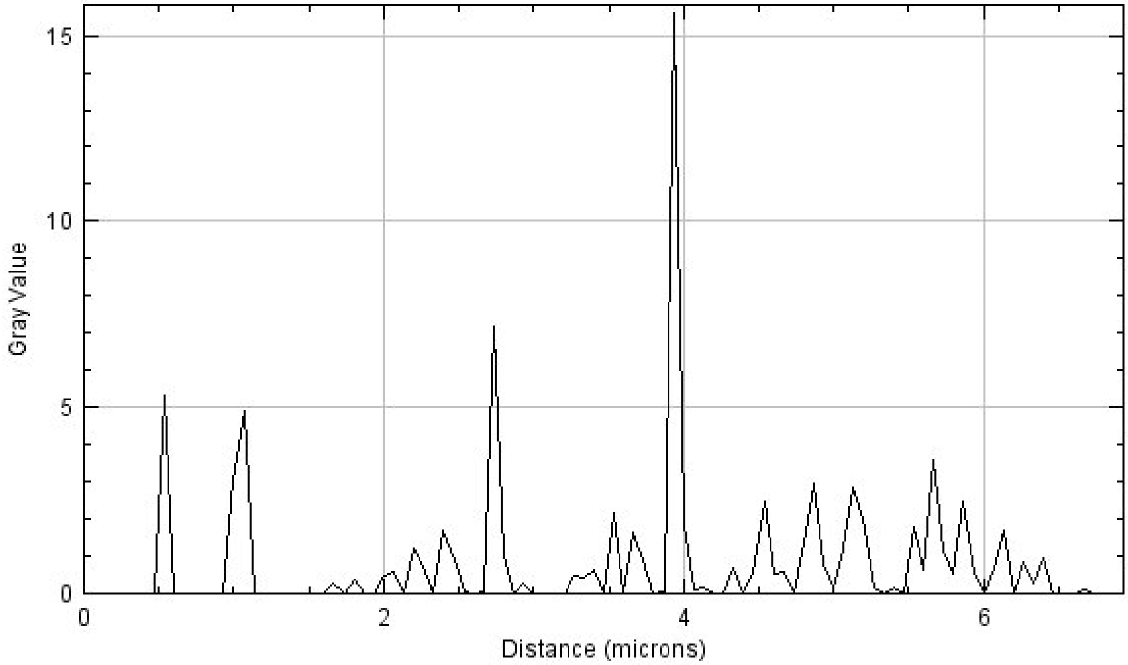

Figure S799 – Intensity profile of line scan generated for cell 4, Figure S795.

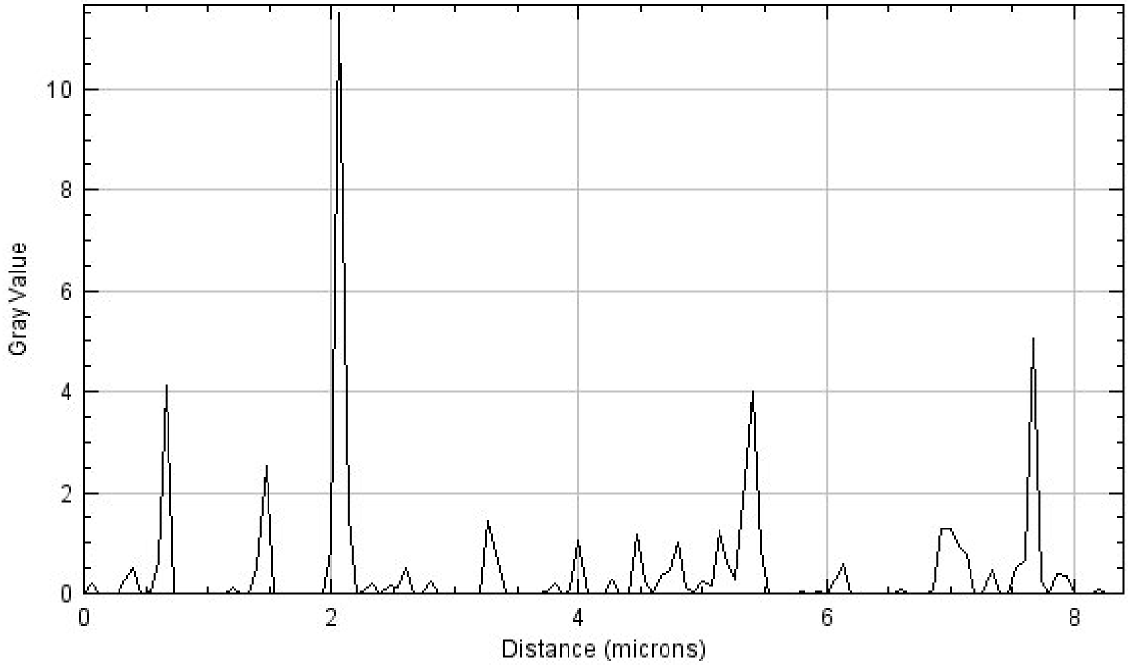

Figure S800 – Intensity profile of line scan generated for cell 5, Figure S795.

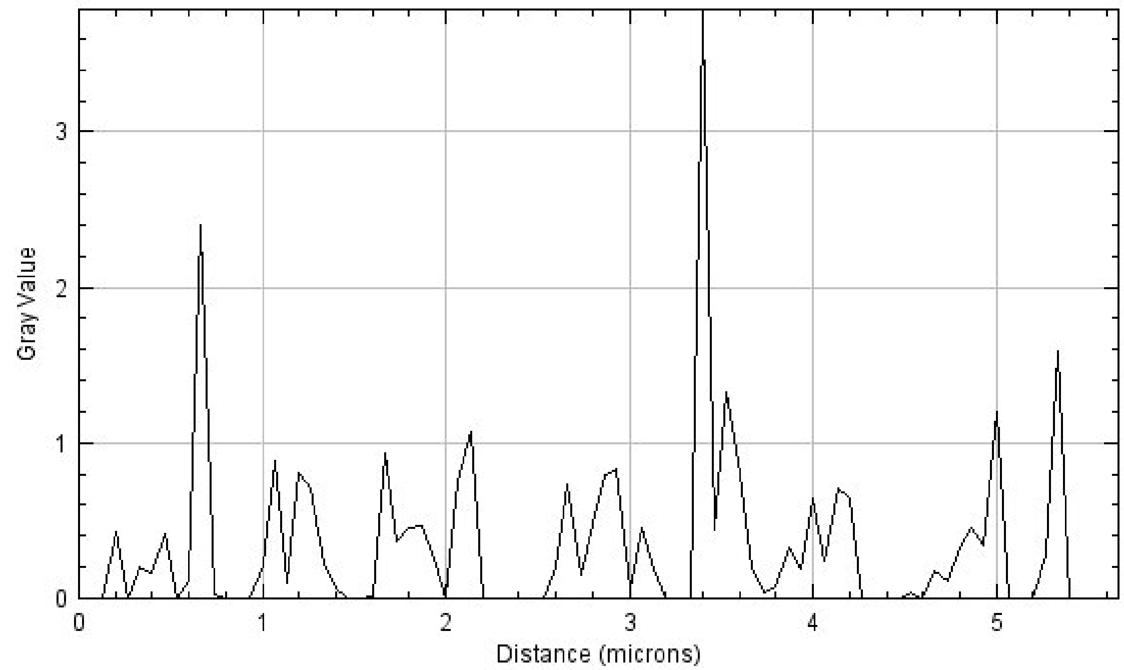

Figure S801 – Intensity profile of line scan generated for cell 6, Figure S795.

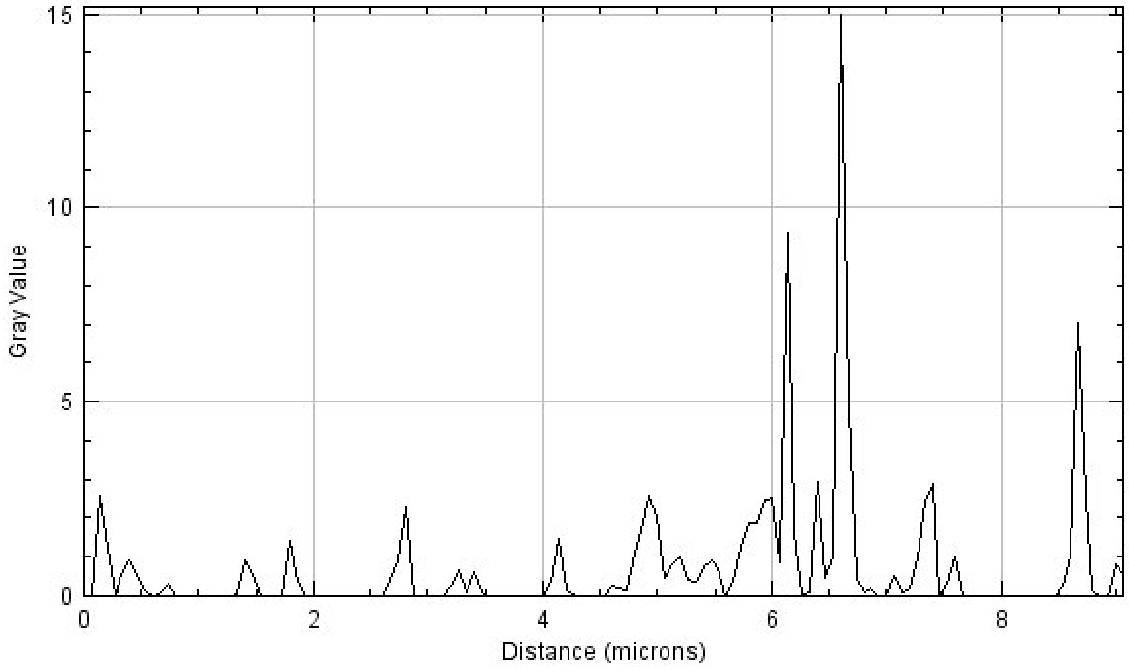

Figure S802 – Intensity profile of line scan generated for cell 7, Figure S795.

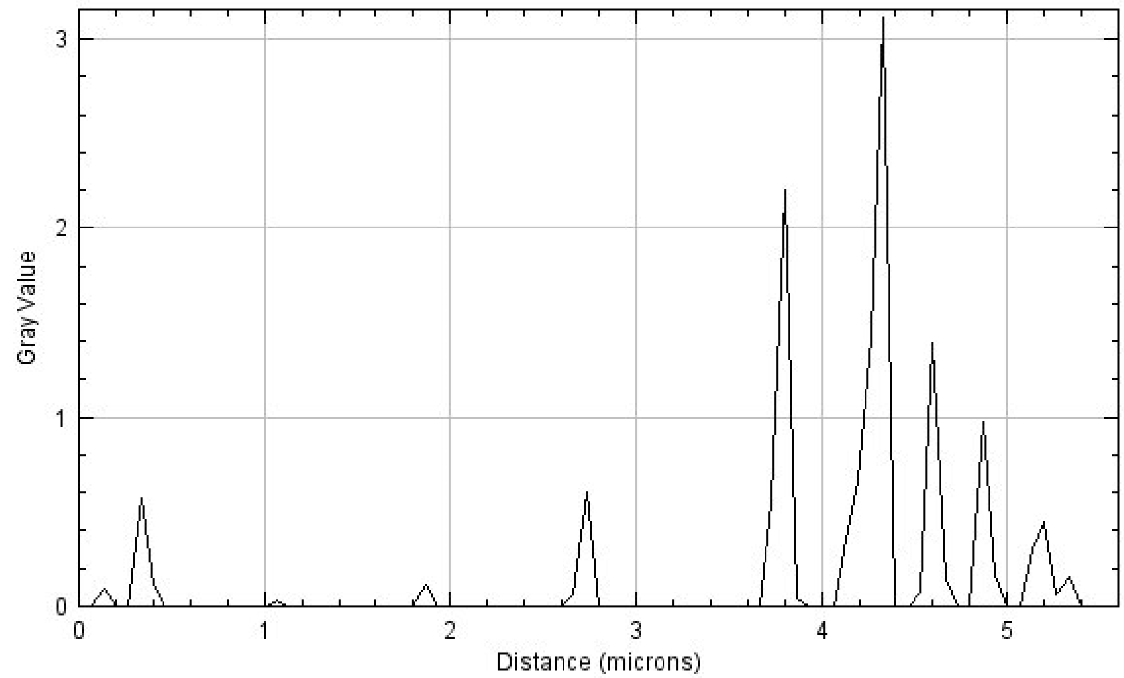

Figure S803 – Intensity profile of line scan generated for cell 8, Figure S795.

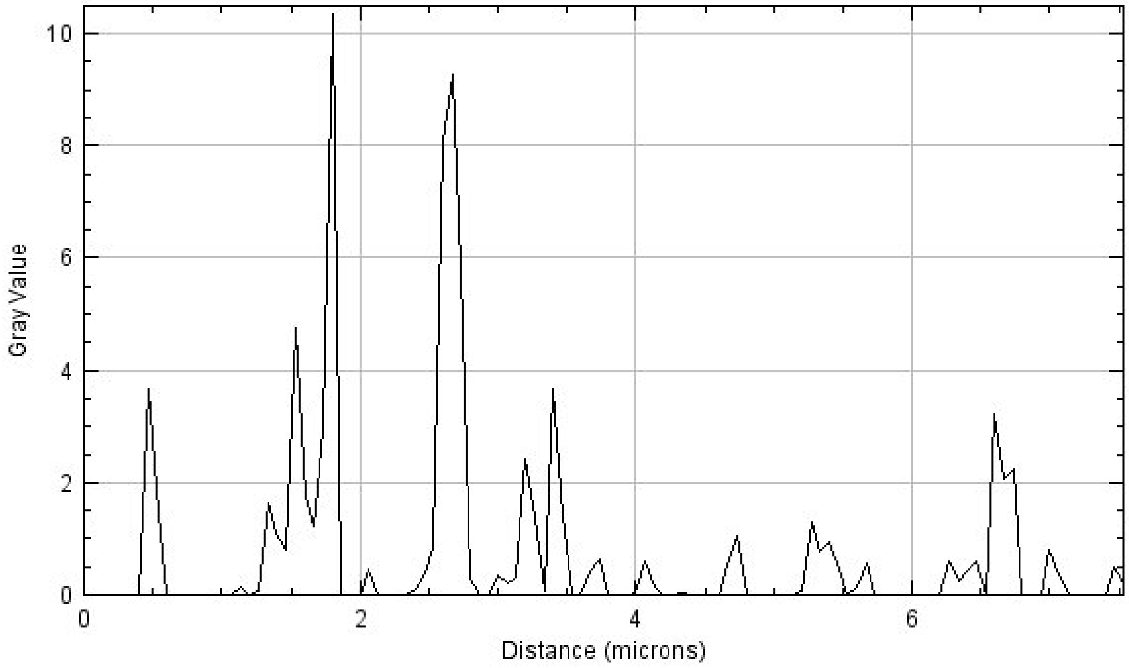

Figure S804 – Intensity profile of line scan generated for cell 9, Figure S795.

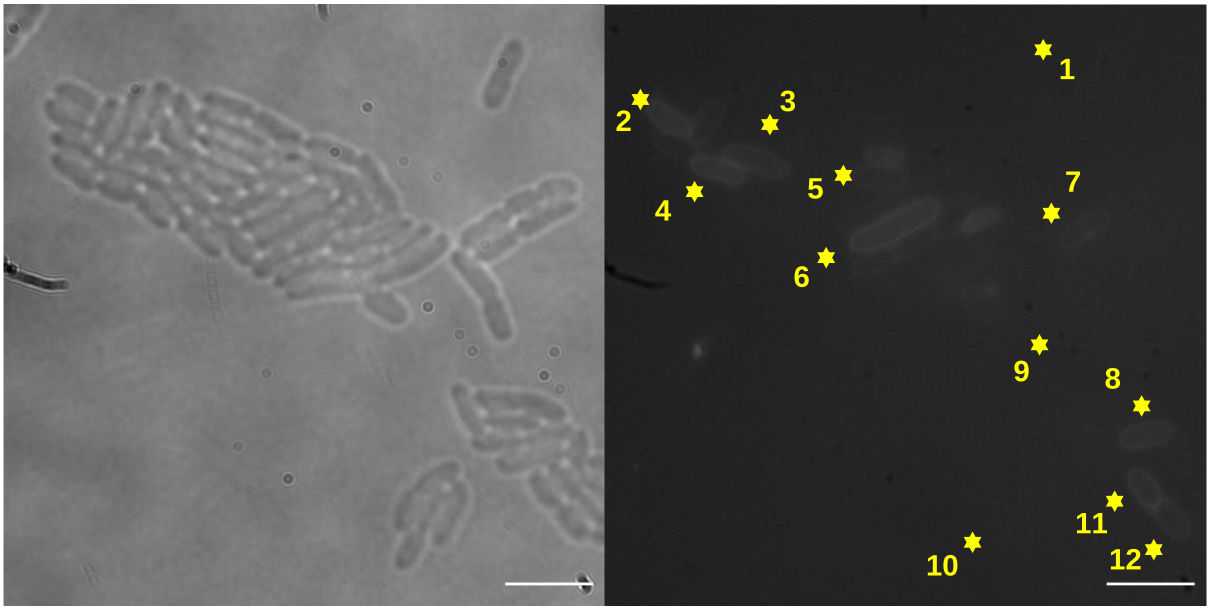

Figure S805 – *E. coli* transmitted and DAPI images used in fluorescence intensity calculations in the presence of both compound **39** and FM4-64 at T = 30 minutes. Scale bar = 10 μM.

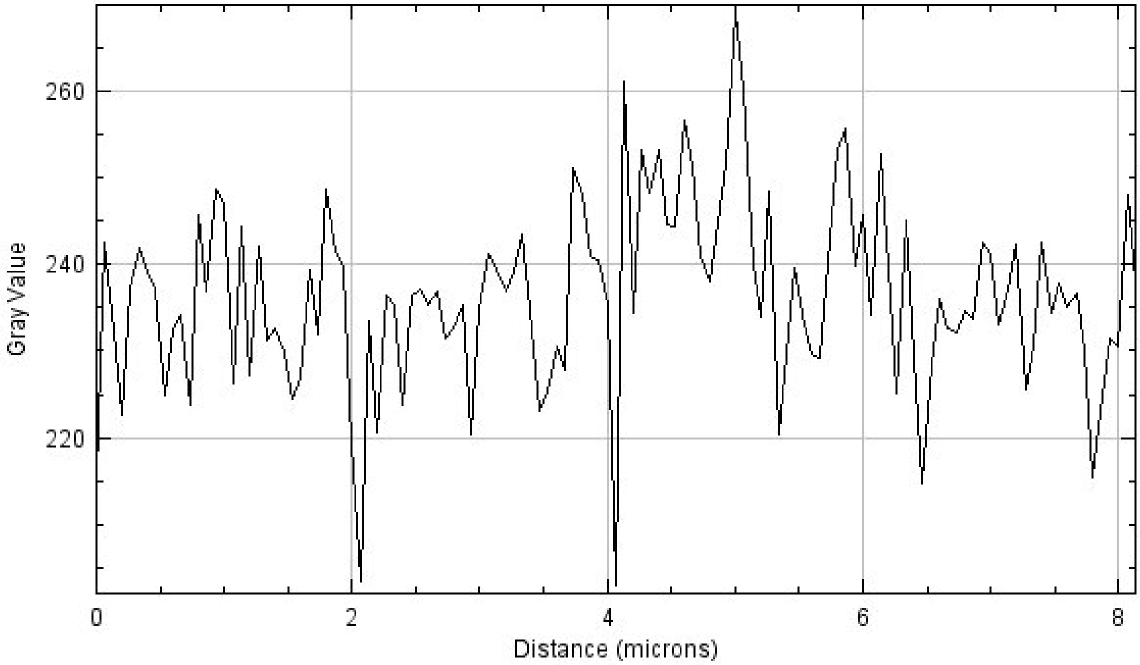

Figure S806 – Intensity profile of line scan generated for cell 1, Figure S805.

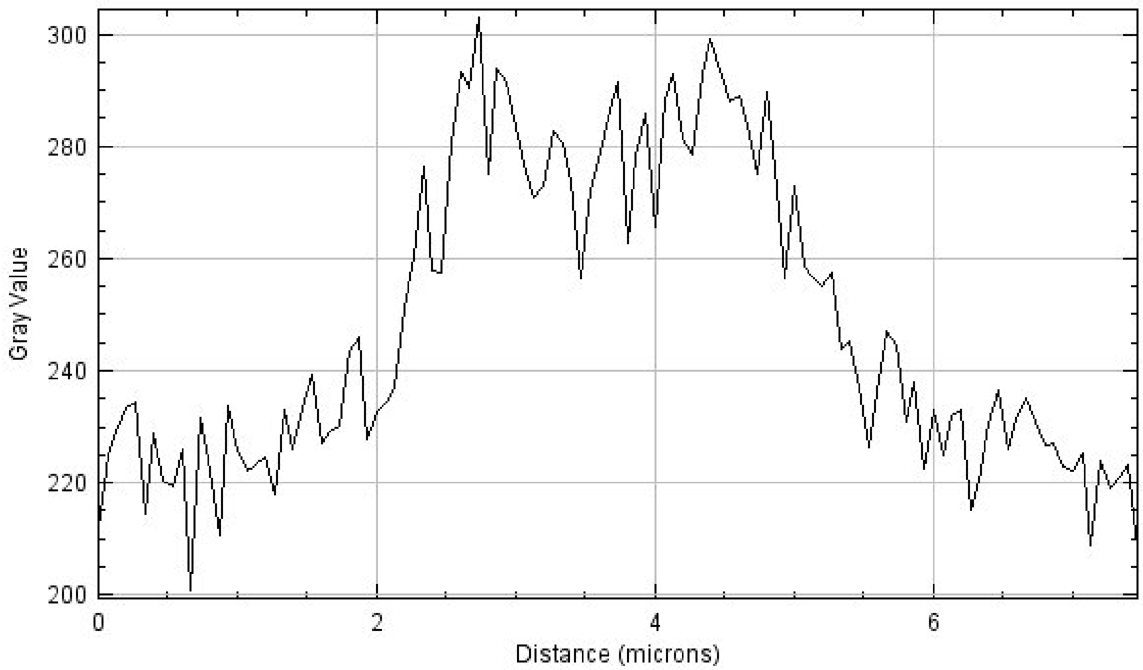

Figure S807 – Intensity profile of line scan generated for cell 2, Figure S805.

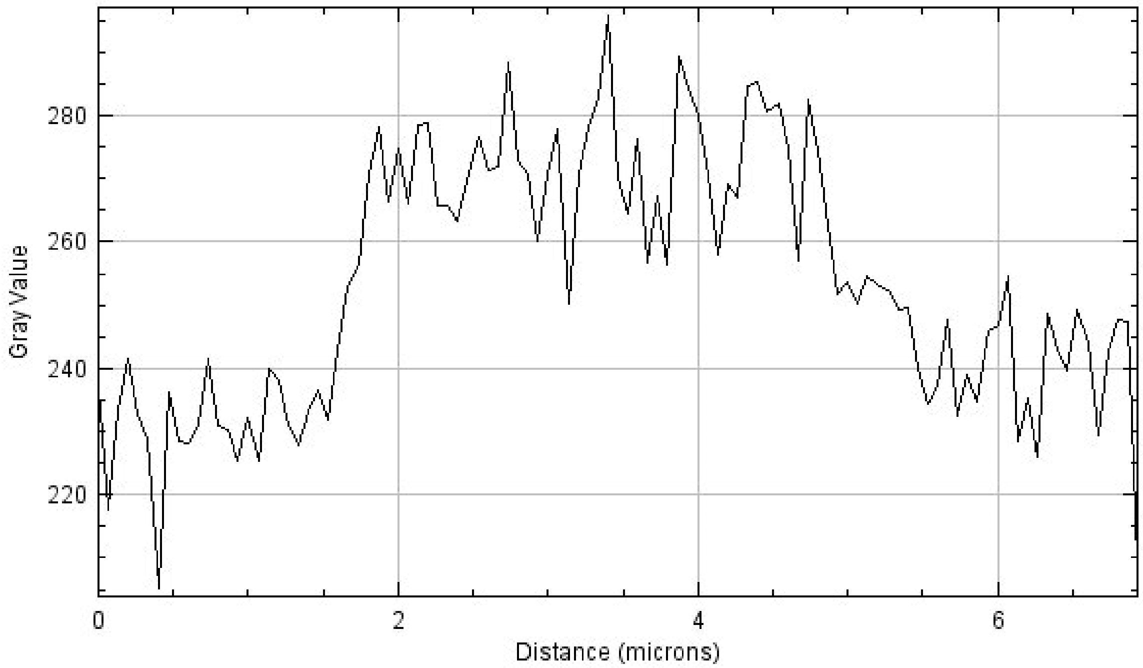

Figure S808 – Intensity profile of line scan generated for cell 3, Figure S805.

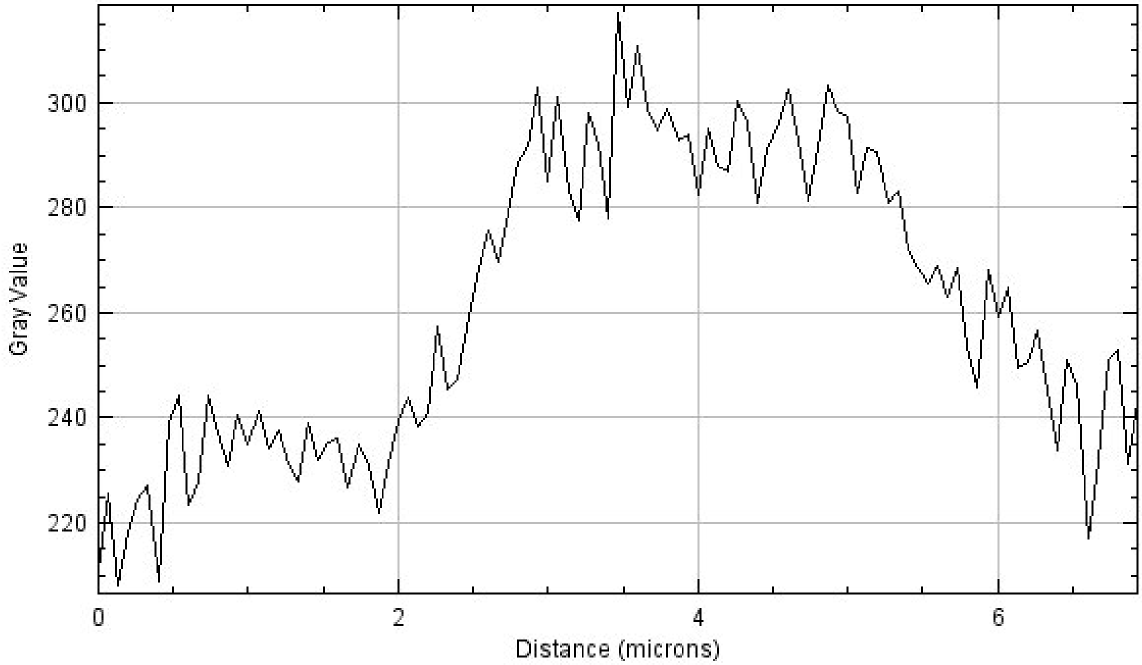

Figure S809 – Intensity profile of line scan generated for cell 4, Figure S805.

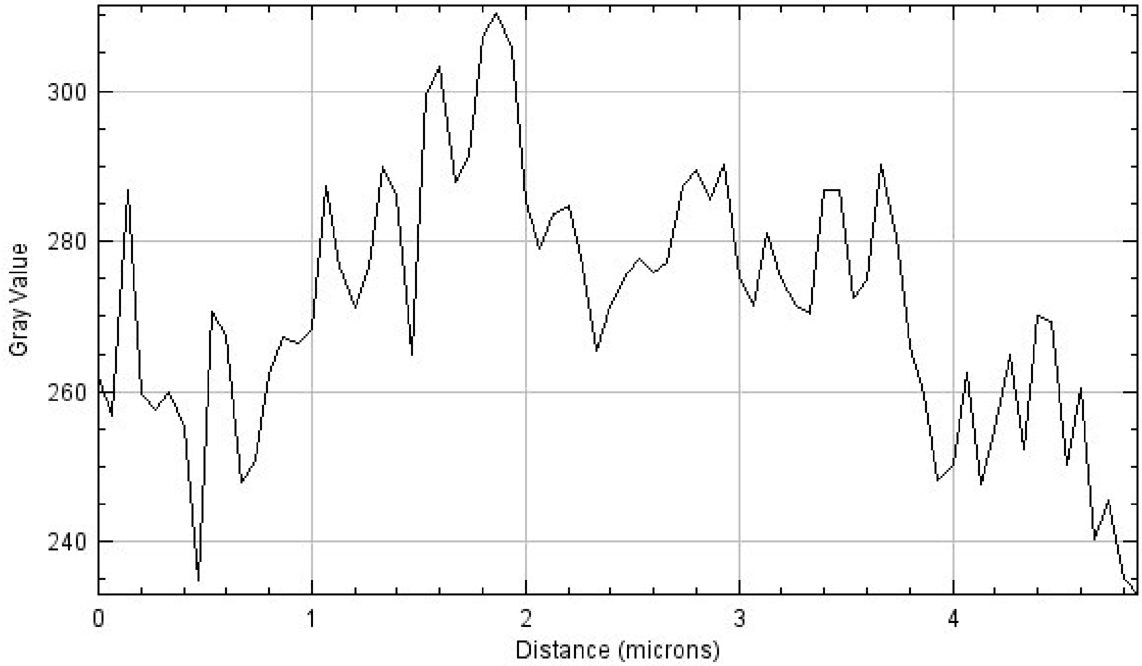

Figure S810 – Intensity profile of line scan generated for cell 5, Figure S805.

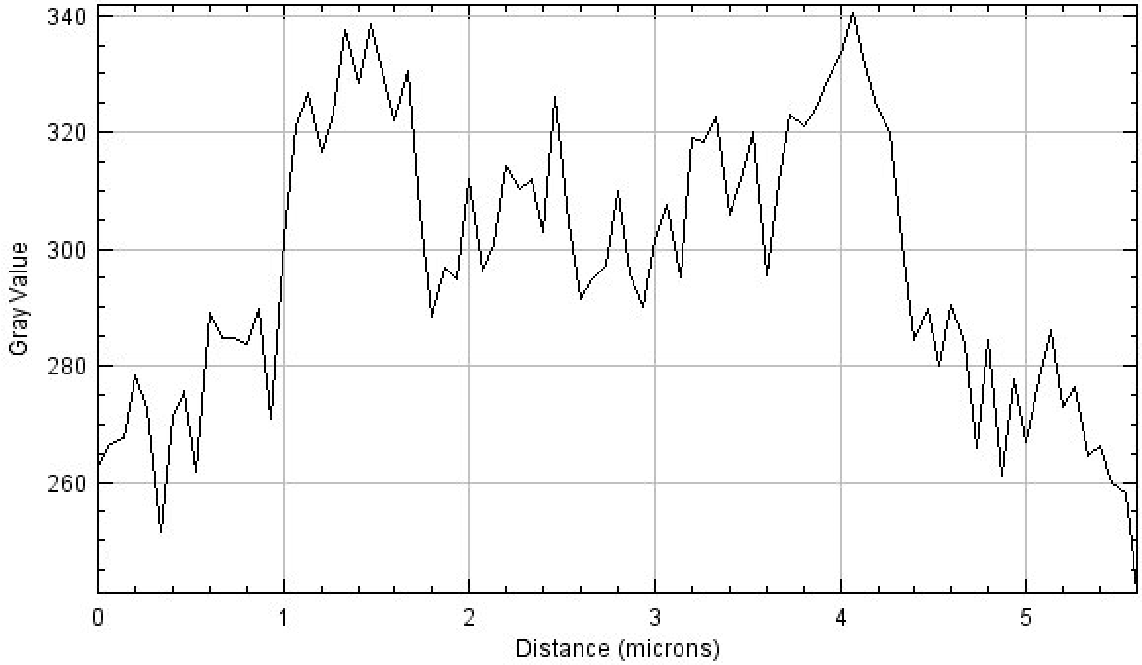

Figure S811 – Intensity profile of line scan generated for cell 6, Figure S805.

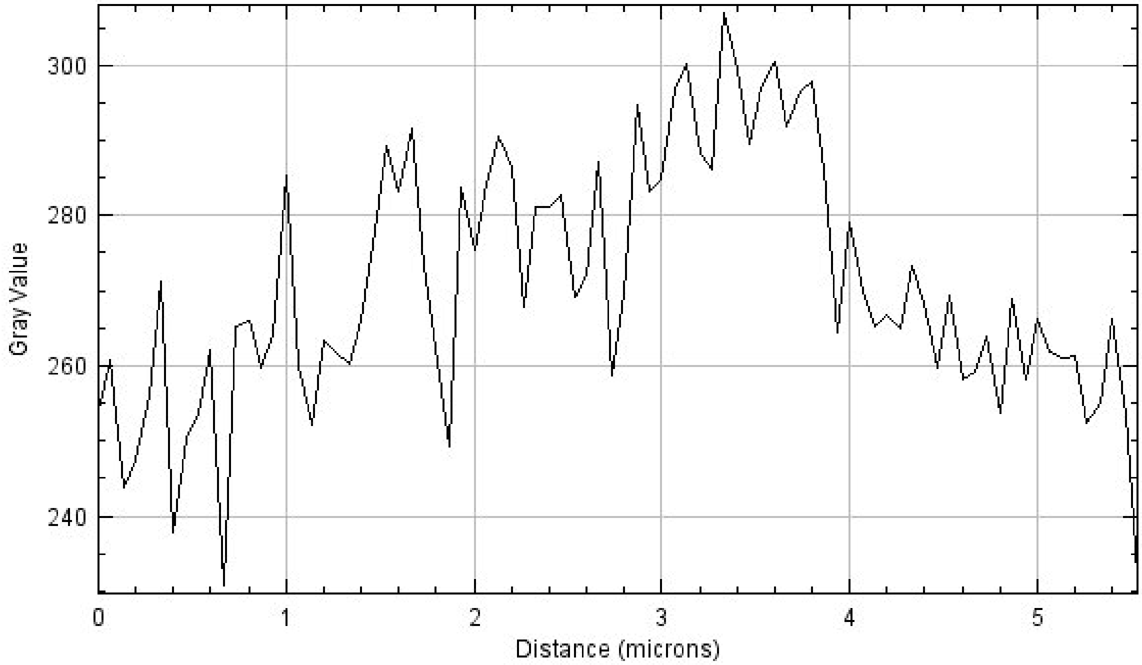

Figure S812 – Intensity profile of line scan generated for cell 7, Figure S805.

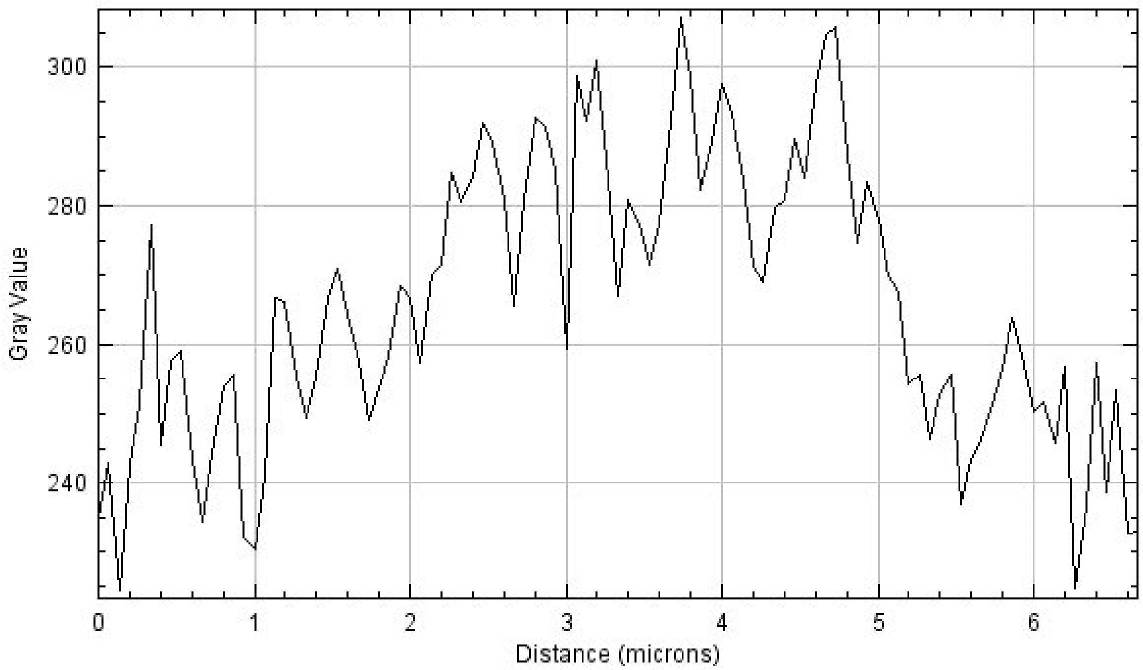

Figure S813 – Intensity profile of line scan generated for cell 8, Figure S805.

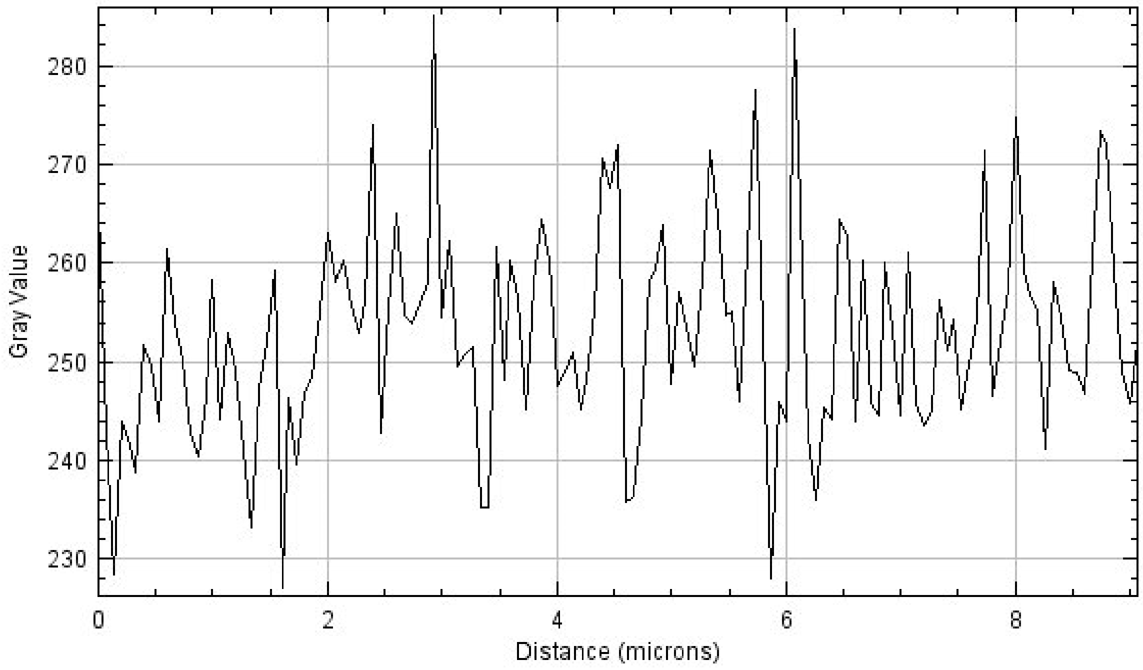

Figure S814 – Intensity profile of line scan generated for cell 9, Figure S805.

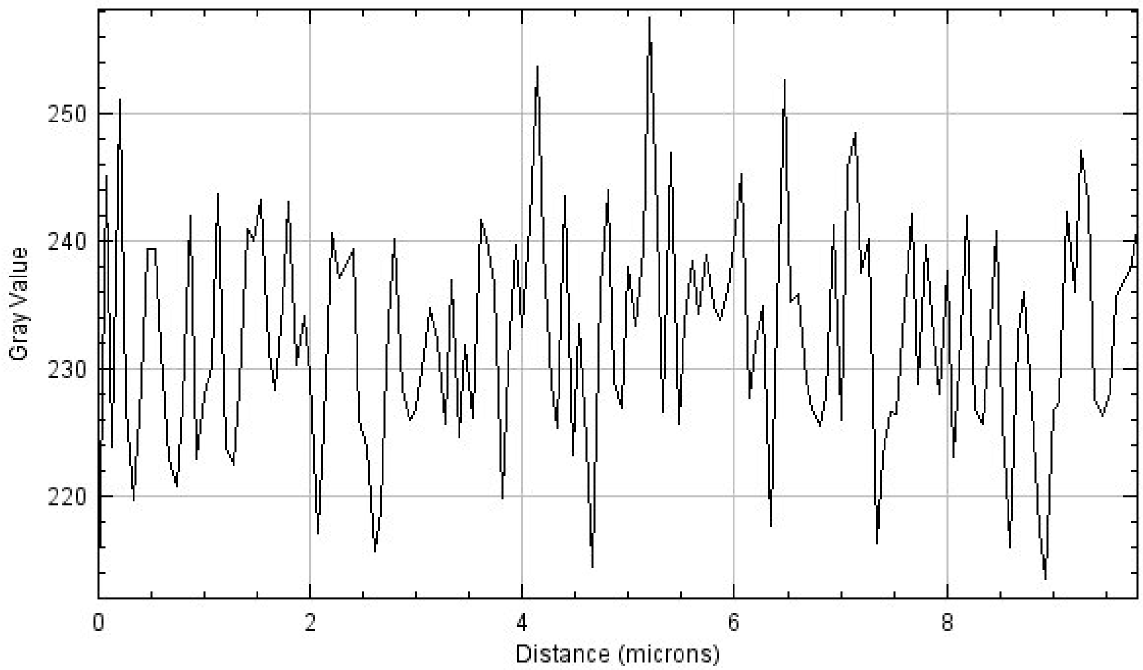

Figure S815 – Intensity profile of line scan generated for cell 10, Figure S805.

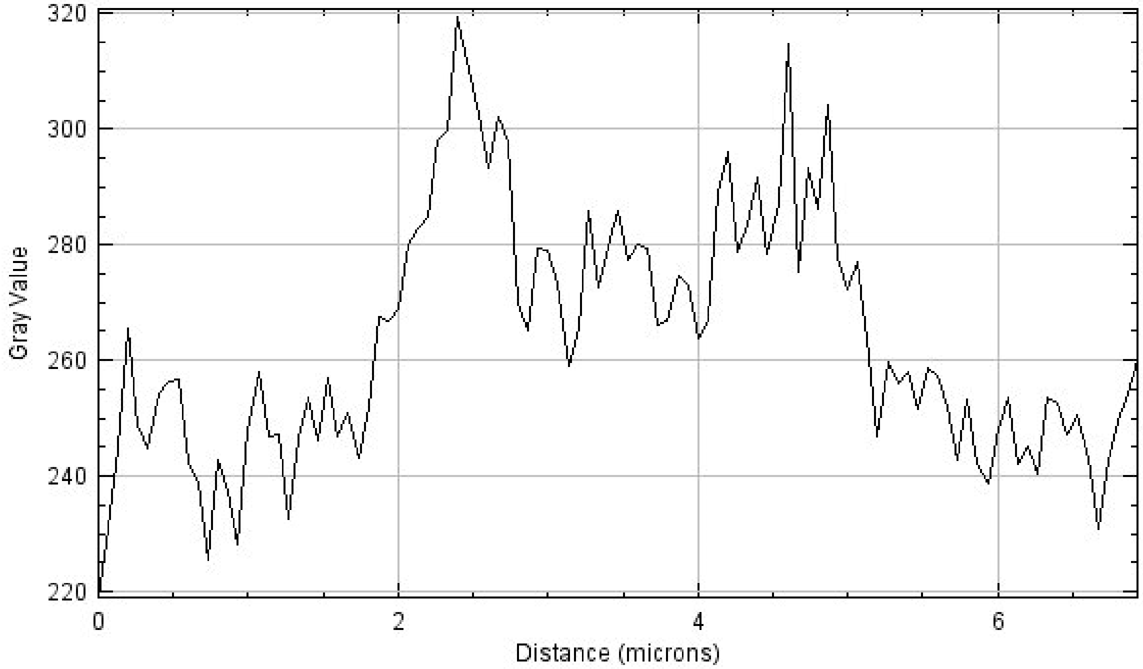

Figure S816 – Intensity profile of line scan generated for cell 11, Figure S805.

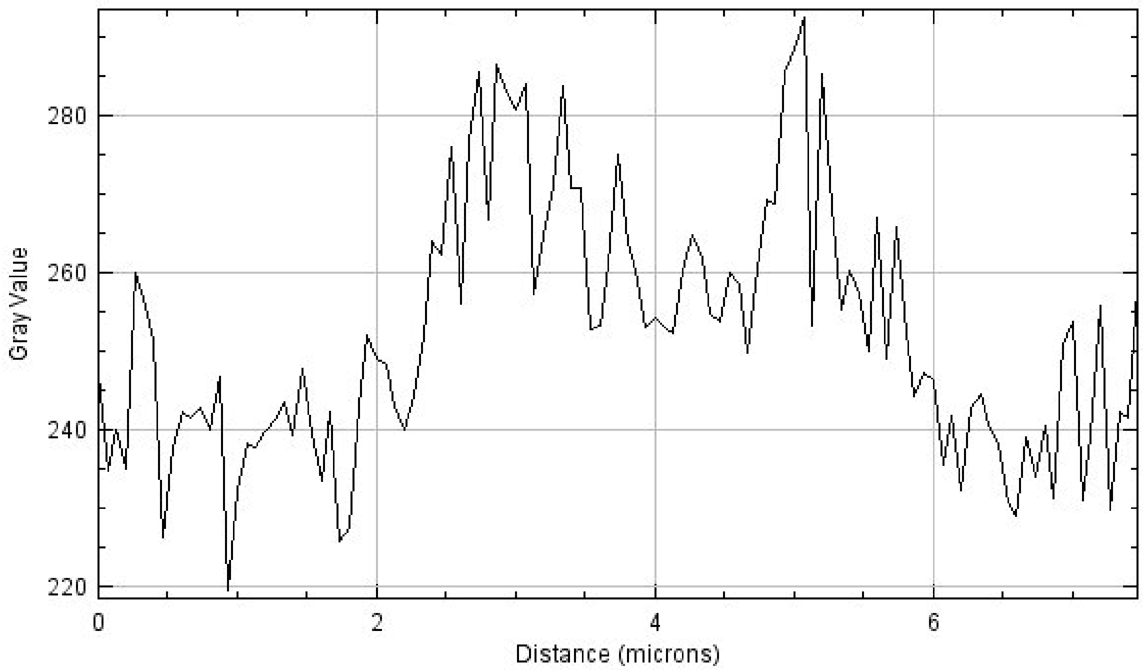

Figure S817 – Intensity profile of line scan generated for cell 12, Figure S805.

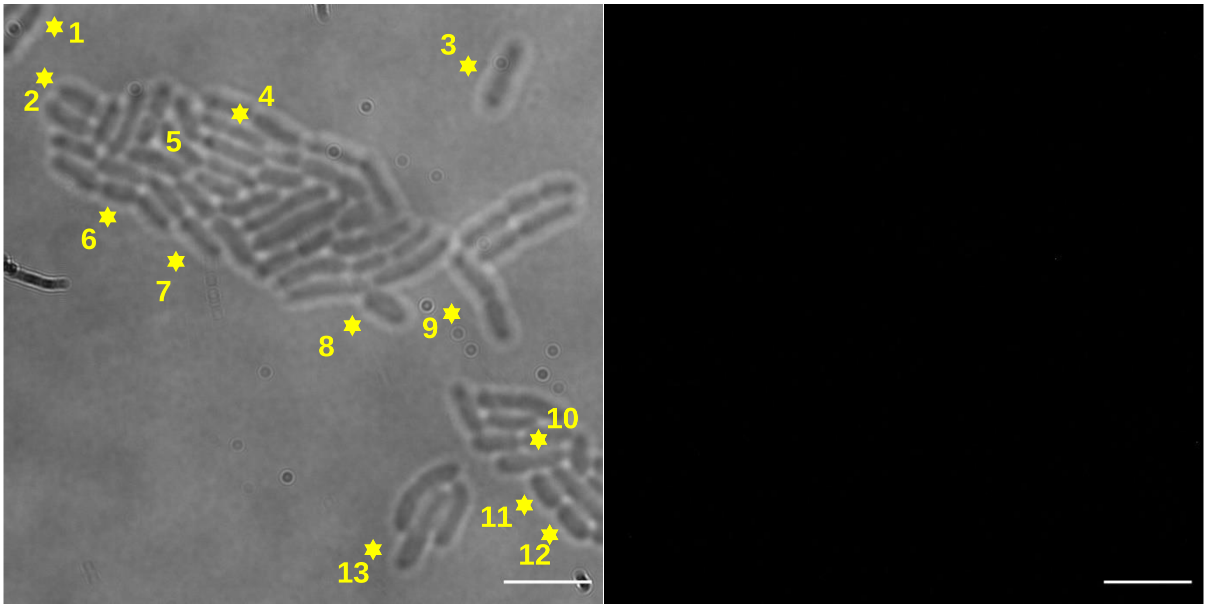

Figure S818 – *E. coli* transmitted and mCherry images used in fluorescence intensity calculations in the presence of both compound **39** and FM4-64 at T = 30 minutes. DAPI and transmitted images used to locate cells on mCherry image. Scale bar = 10 μM.

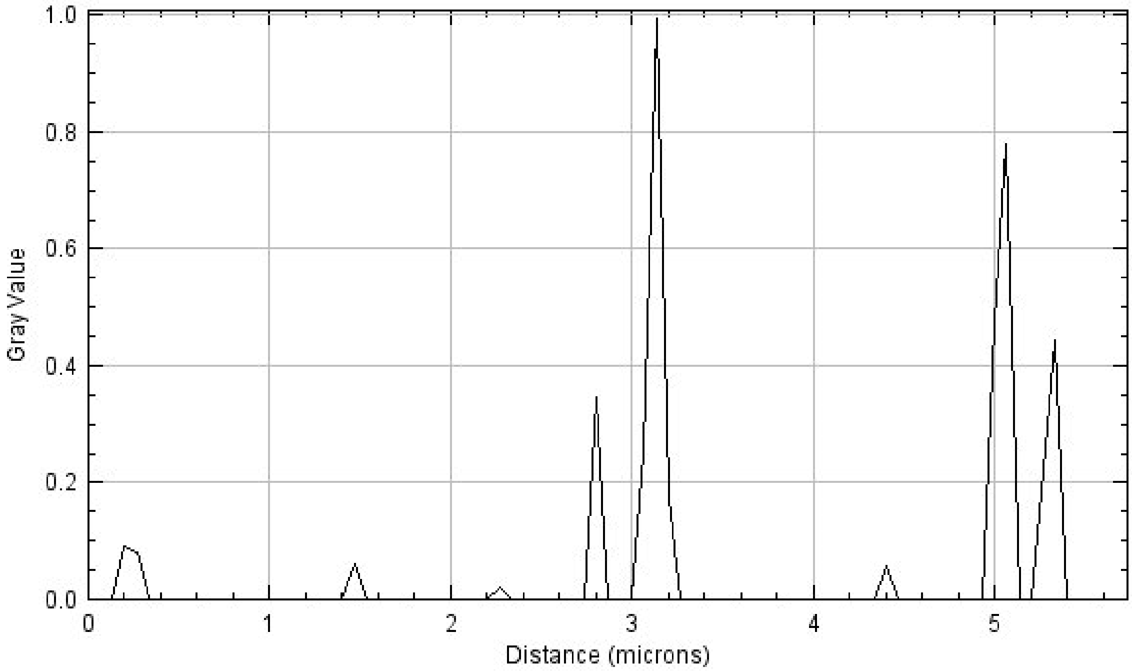

Figure S819 – Intensity profile of line scan generated for cell 1, Figure S818.

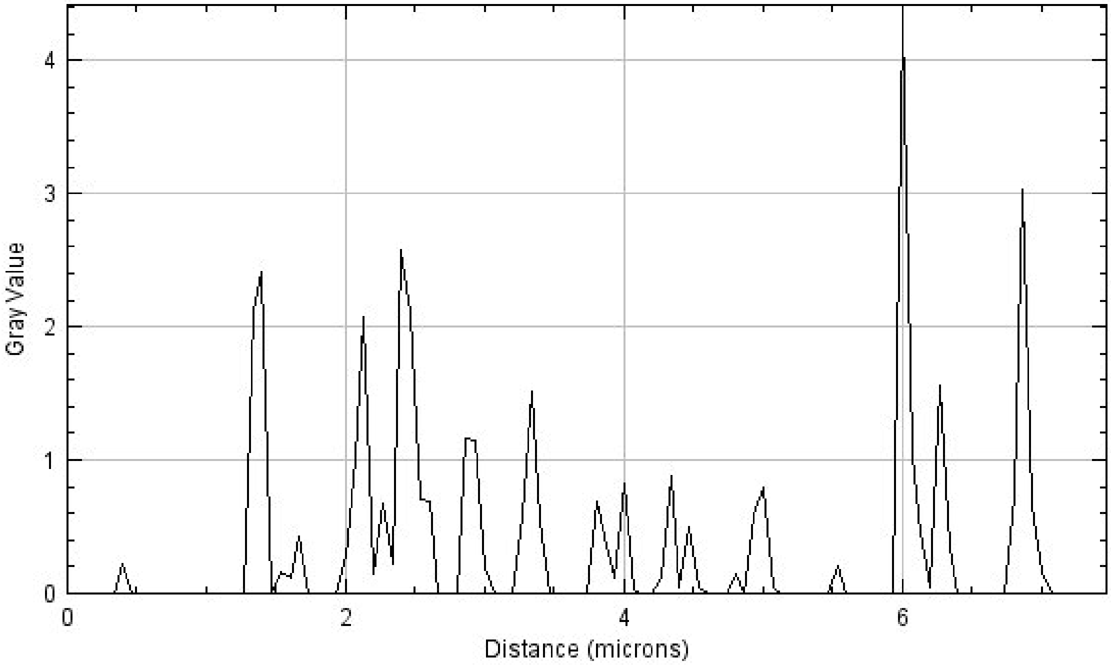

Figure S820 – Intensity profile of line scan generated for cell 2, Figure S818.

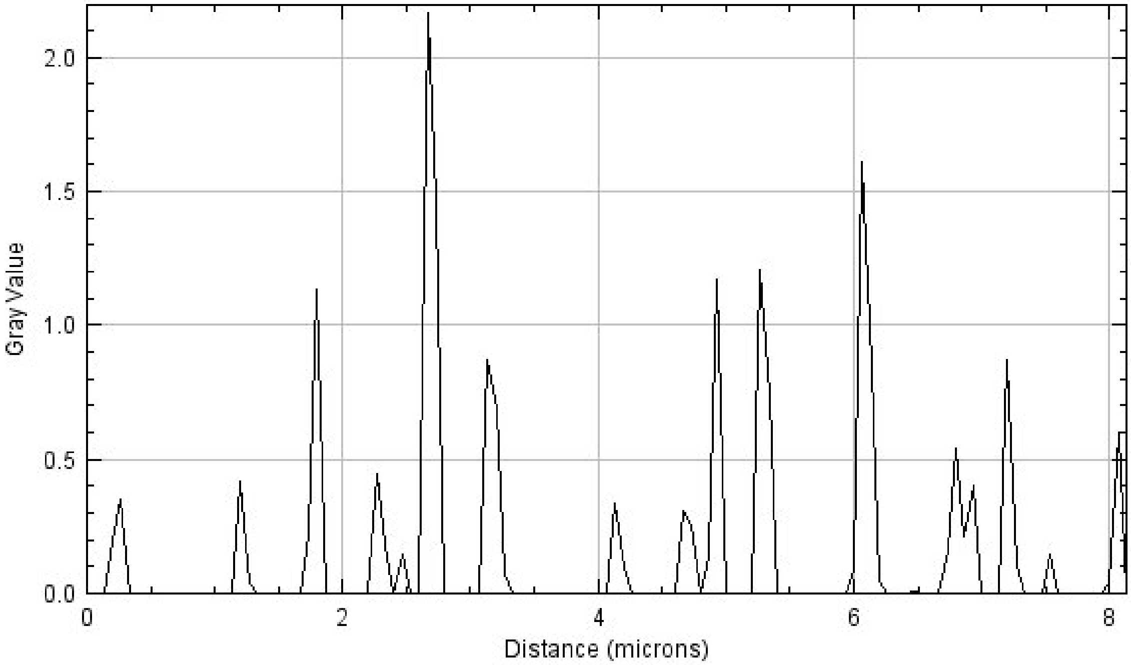

Figure S821 – Intensity profile of line scan generated for cell 3, Figure S818.

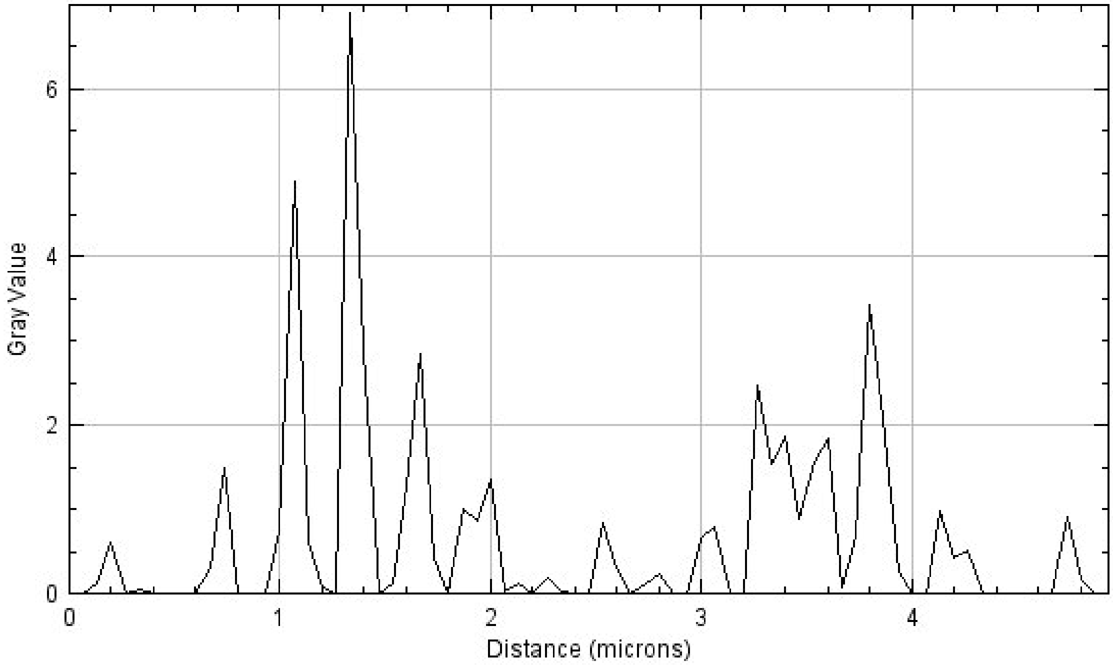

Figure S822 – Intensity profile of line scan generated for cell 4, Figure S818.

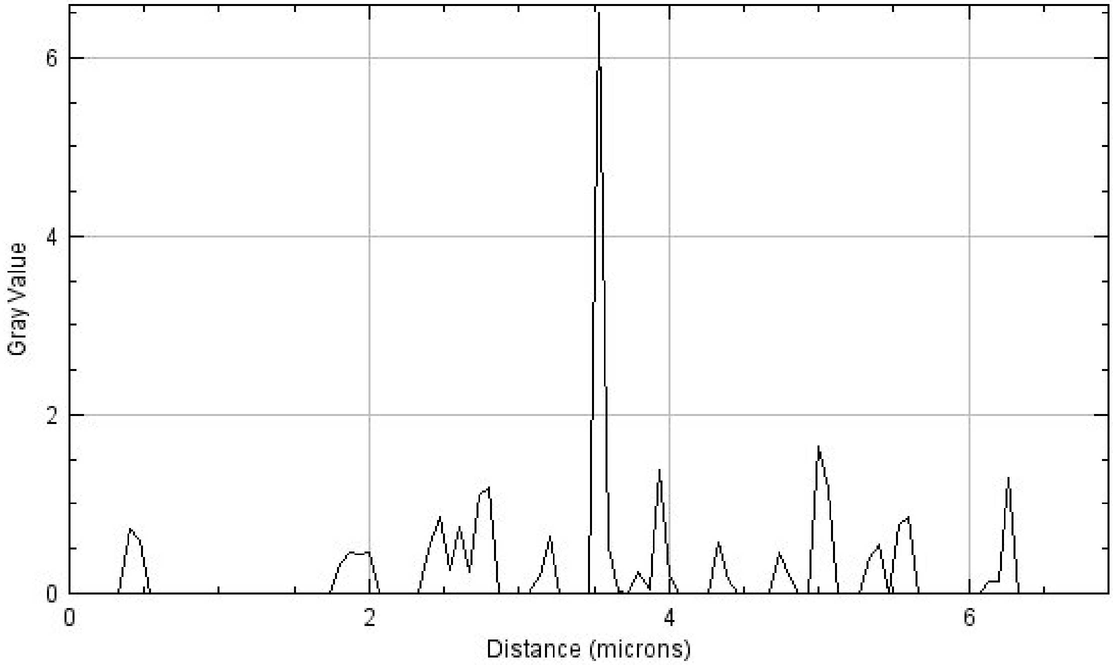

Figure S823 – Intensity profile of line scan generated for cell 5, Figure S818.

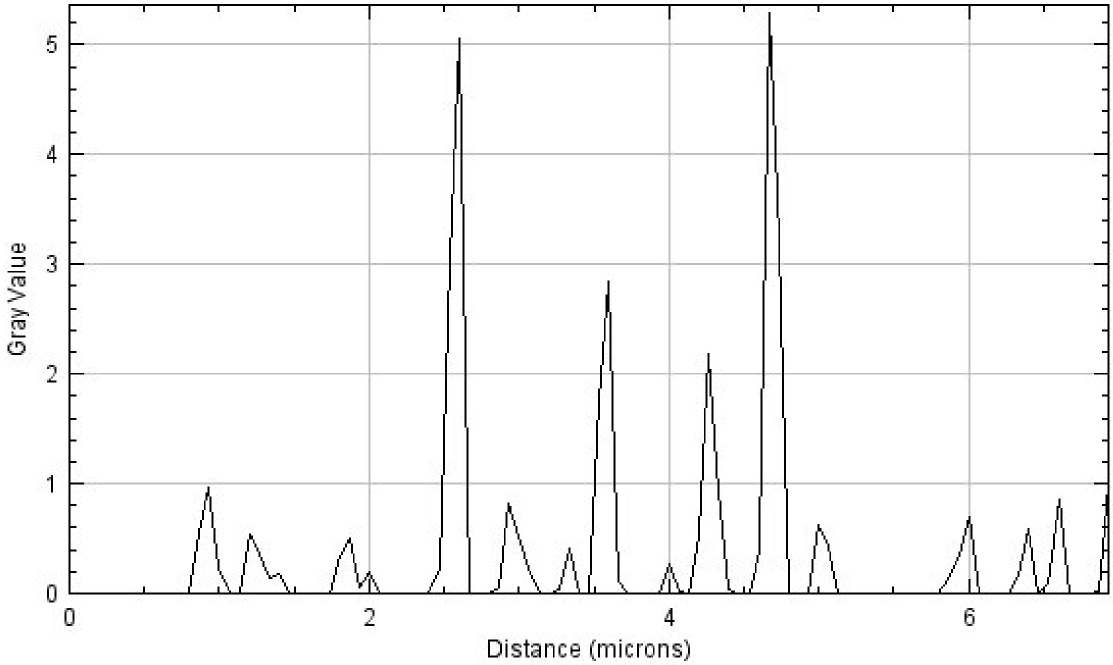

Figure S824 – Intensity profile of line scan generated for cell 6, Figure S818.

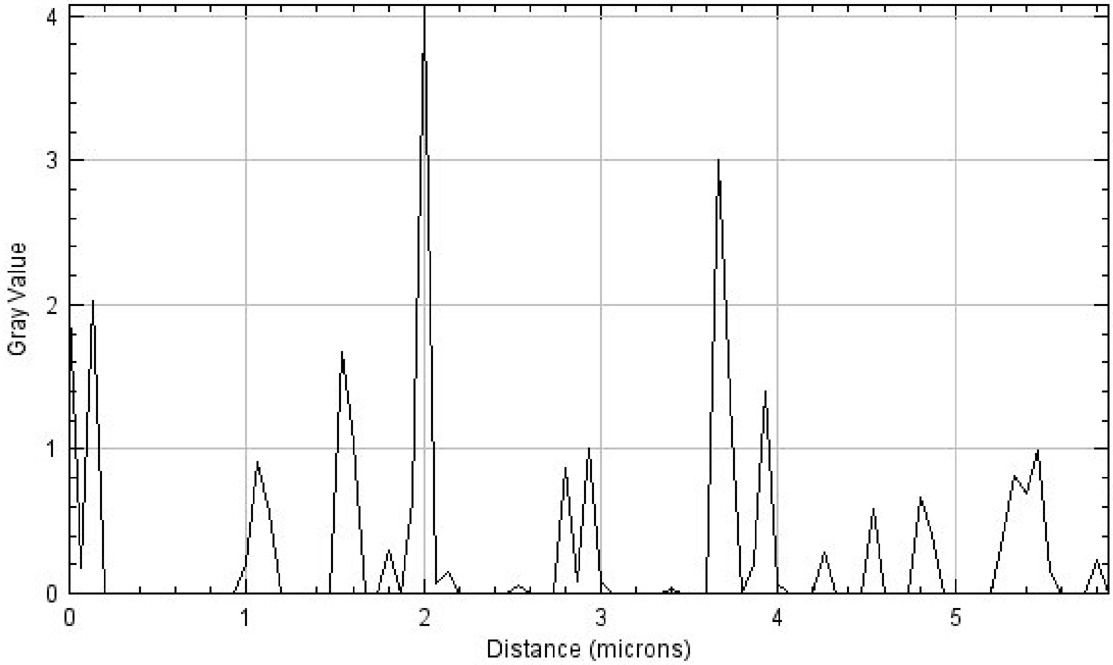

Figure S825 – Intensity profile of line scan generated for cell 7, Figure S818.

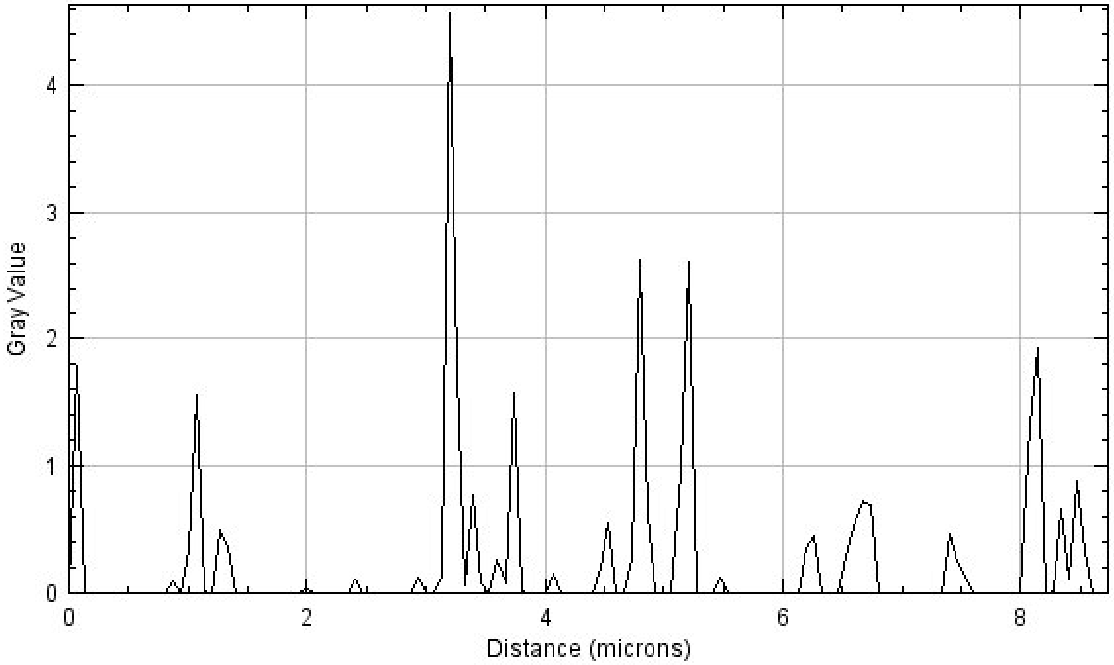

Figure S826 – Intensity profile of line scan generated for cell 8, Figure S818.

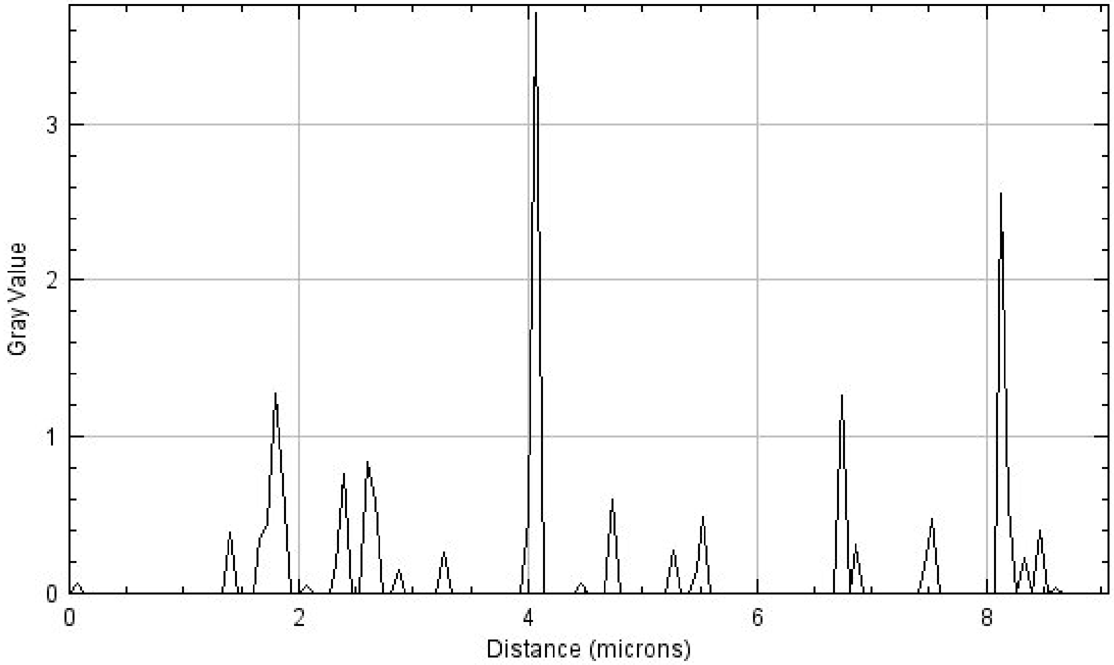

Figure S827 – Intensity profile of line scan generated for cell 9, Figure S818.

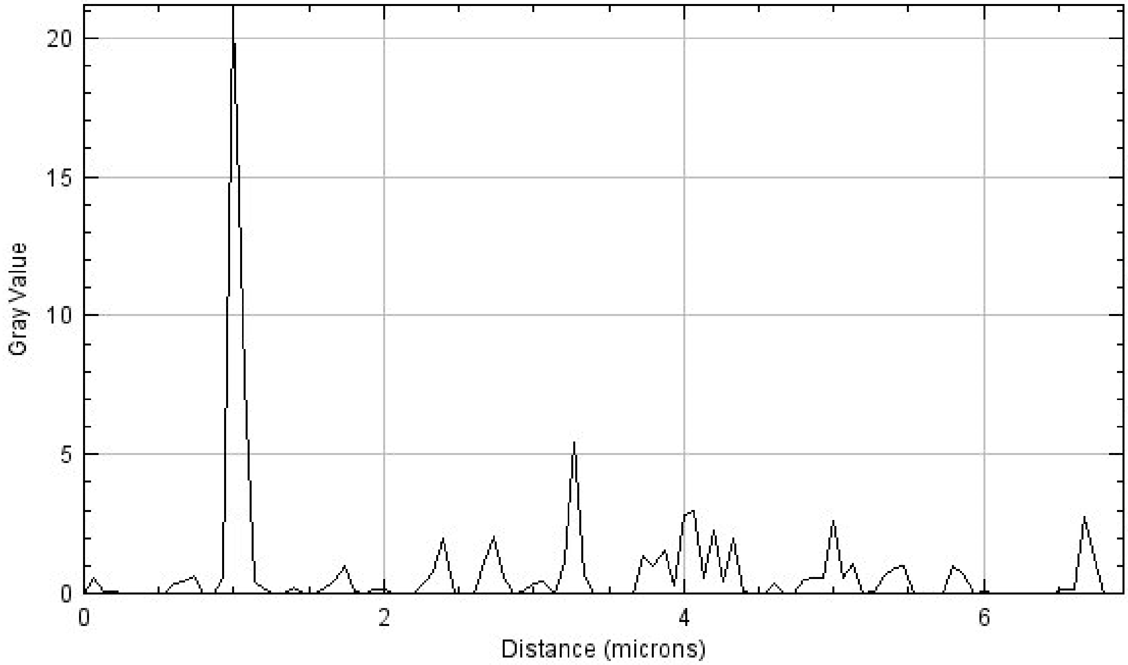

Figure S828 – Intensity profile of line scan generated for cell 10, Figure S818.

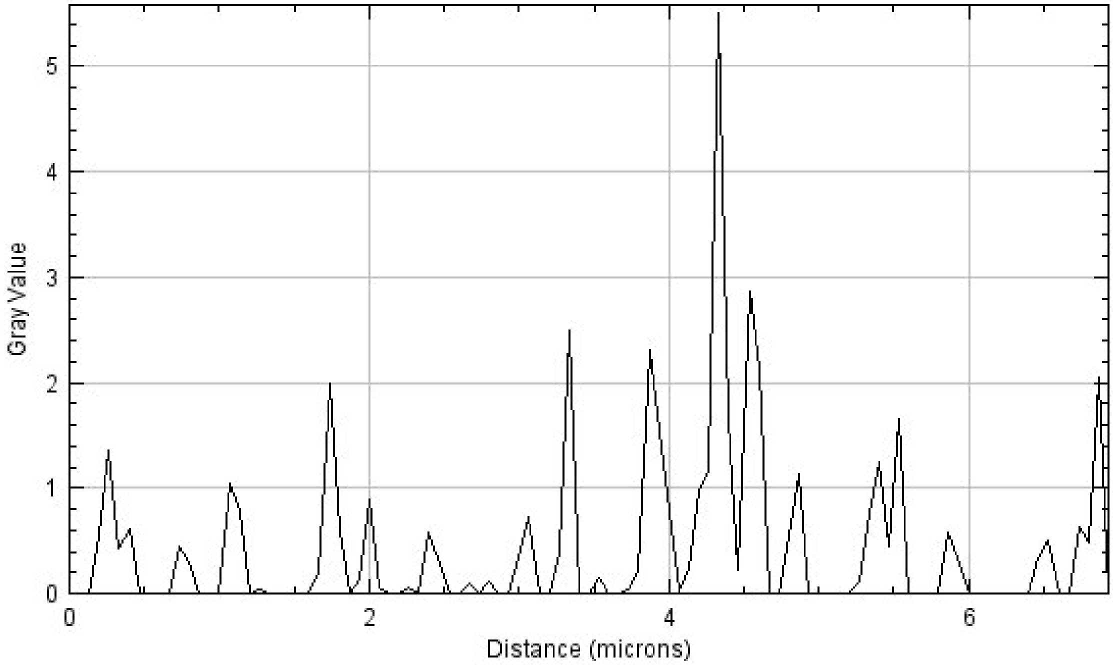

Figure S829 – Intensity profile of line scan generated for cell 11, Figure S818.

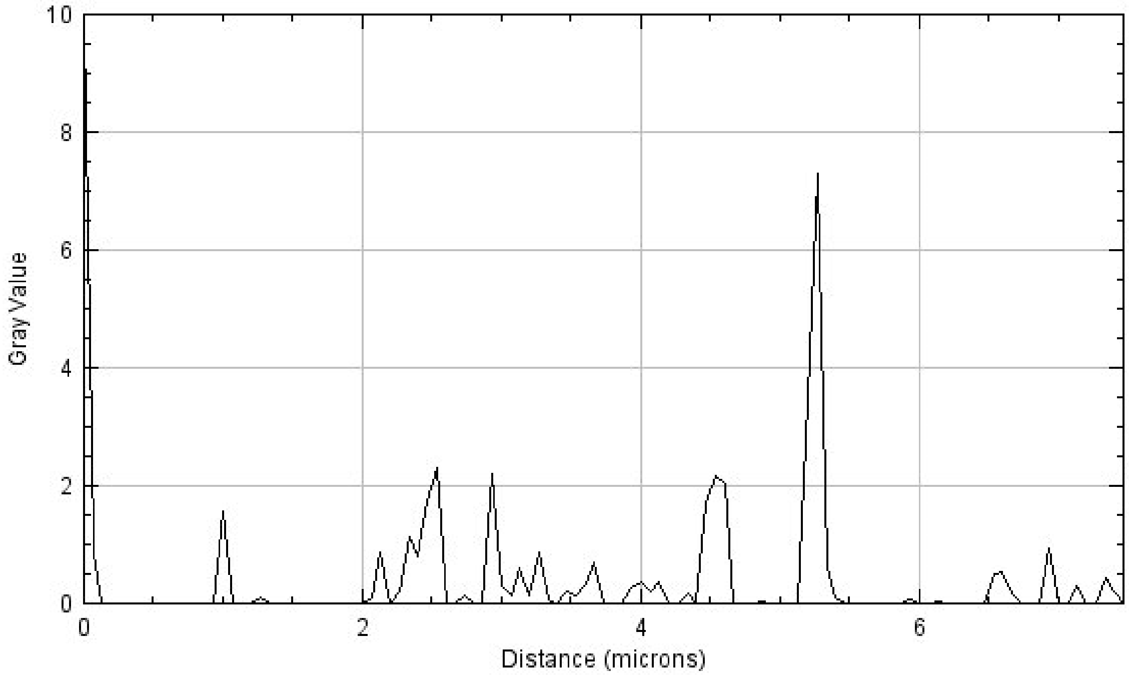

Figure S830 – Intensity profile of line scan generated for cell 12, Figure S818.

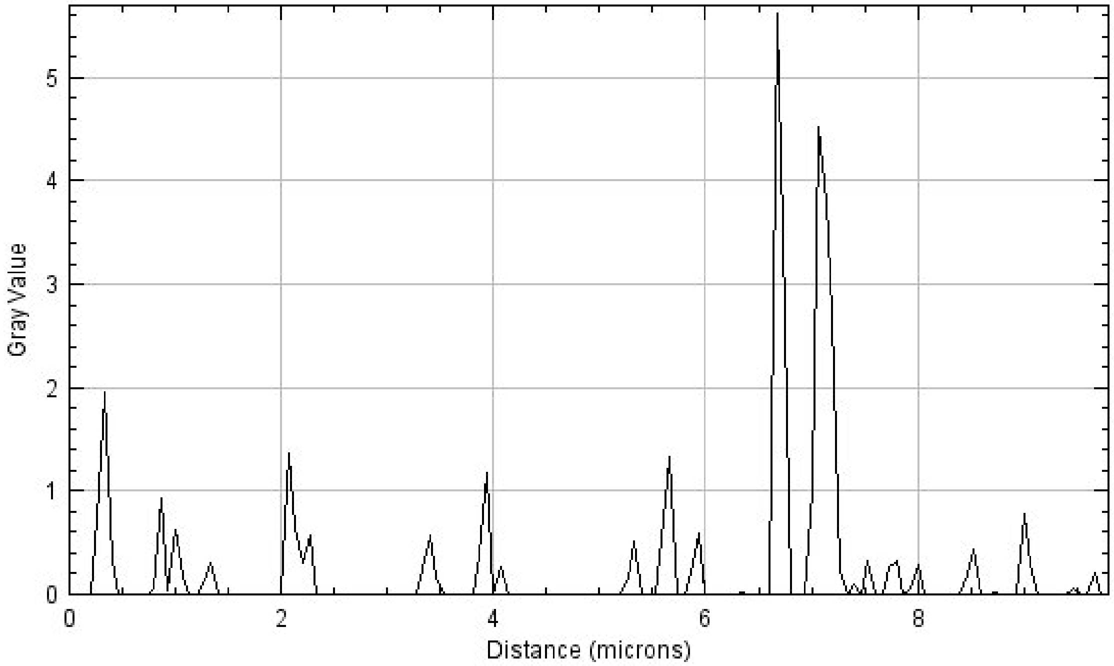

Figure S831 – Intensity profile of line scan generated for cell 13, Figure S818.

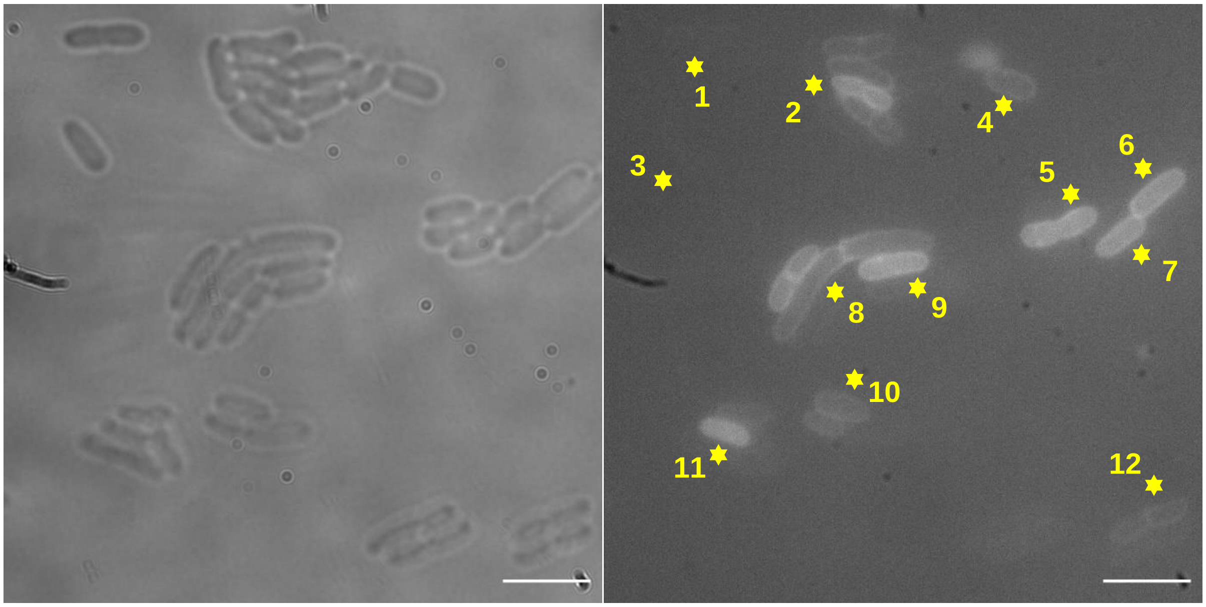

Figure S832 – *E. coli* transmitted and DAPI images used in fluorescence intensity calculations in the presence of both compound **39** and FM4-64 at T = 30 minutes. Scale bar = 10 μM.

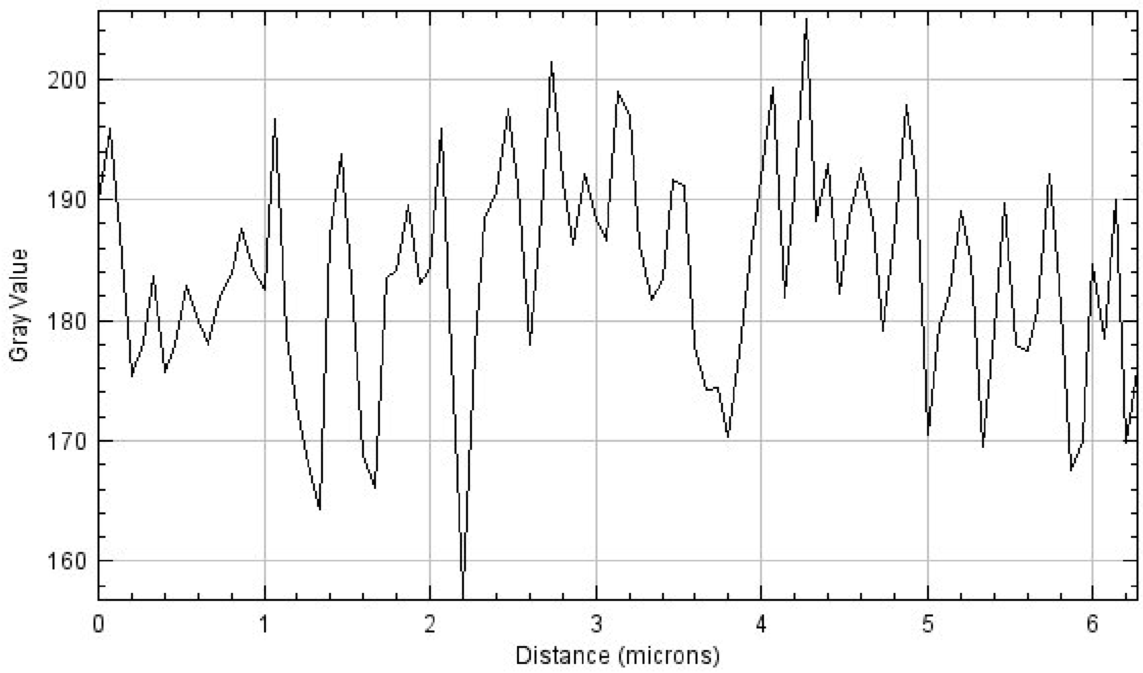

Figure S833 – Intensity profile of line scan generated for cell 1, Figure S832.

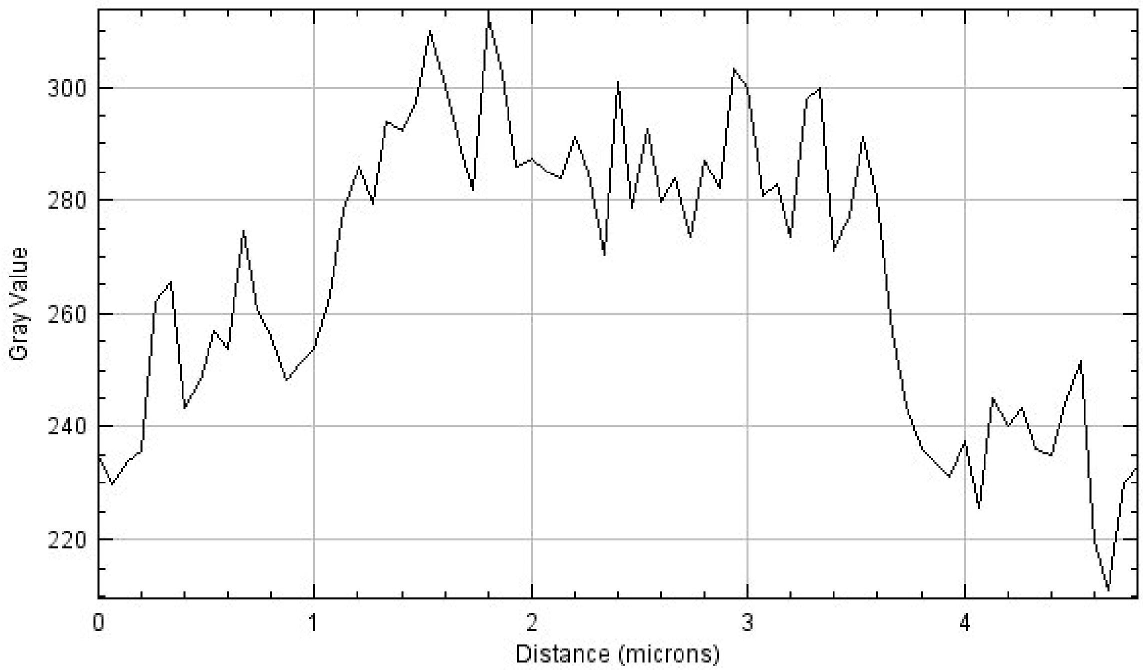

Figure S834 – Intensity profile of line scan generated for cell 2, Figure S832.

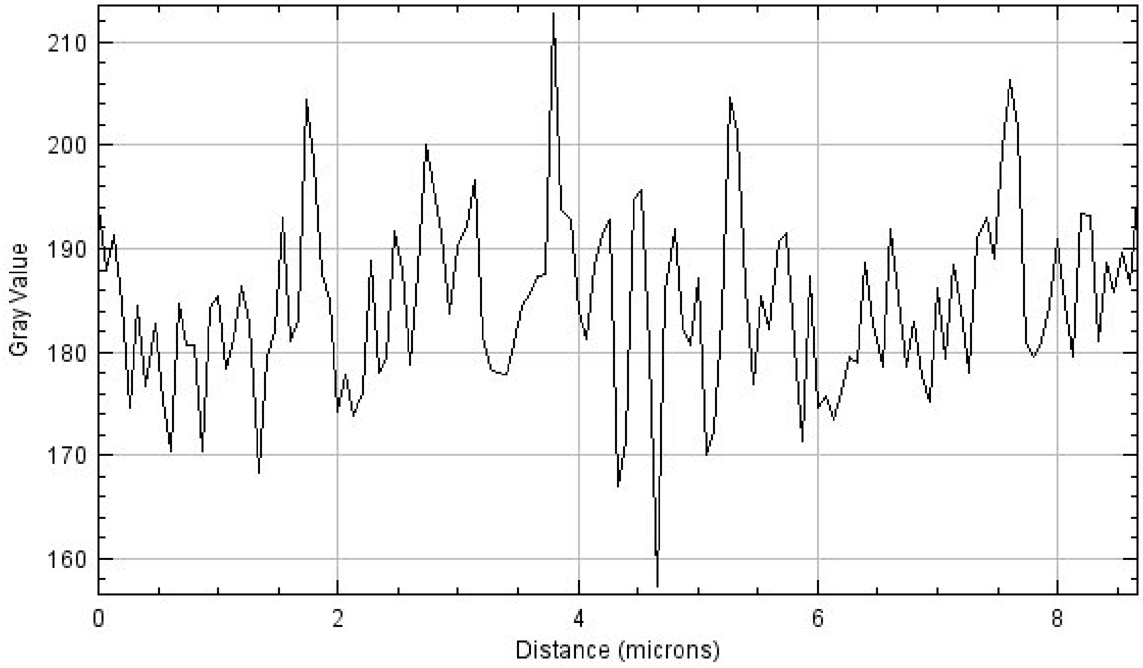

Figure S835 – Intensity profile of line scan generated for cell 3, Figure S832.

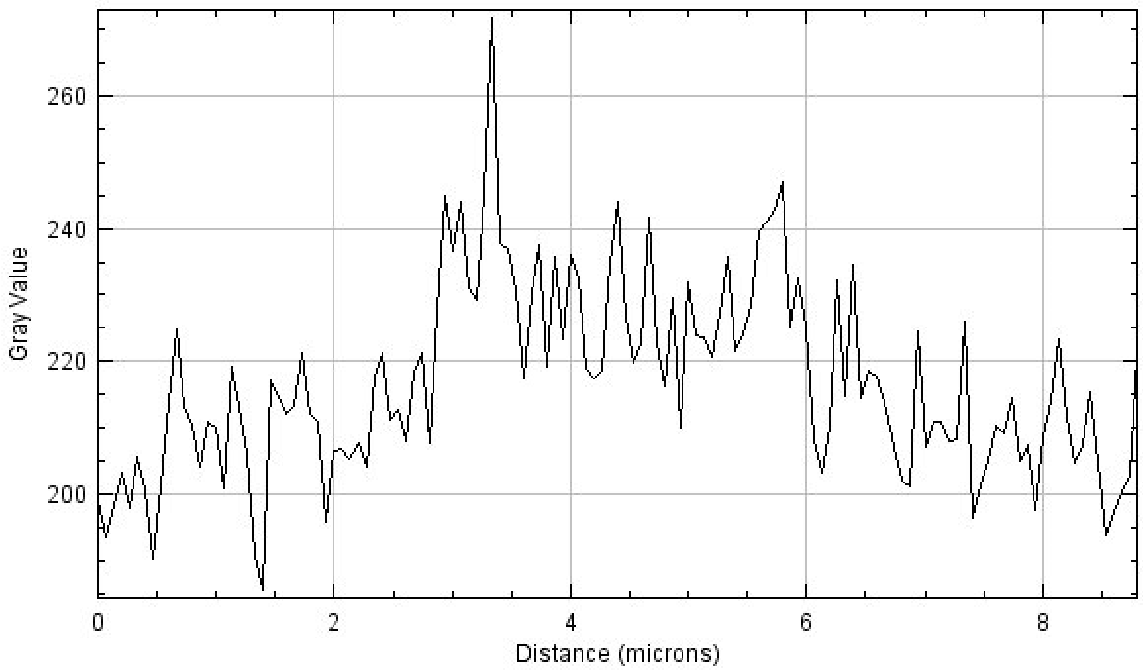

Figure S836 – Intensity profile of line scan generated for cell 4, Figure S832.

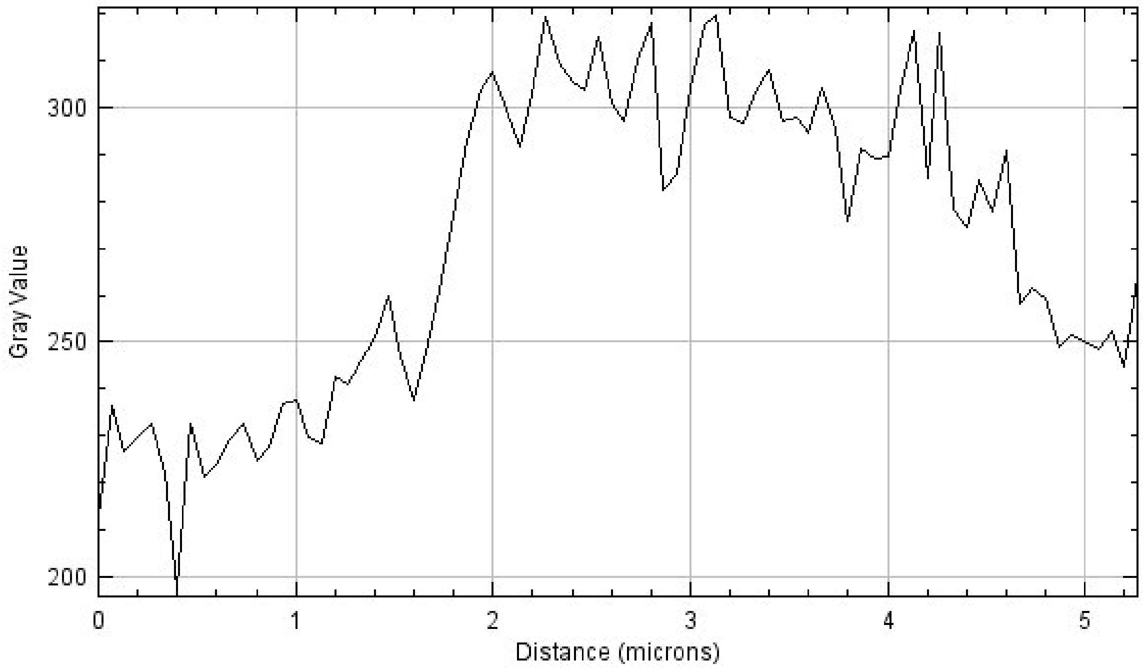

Figure S837 – Intensity profile of line scan generated for cell 5, Figure S832.

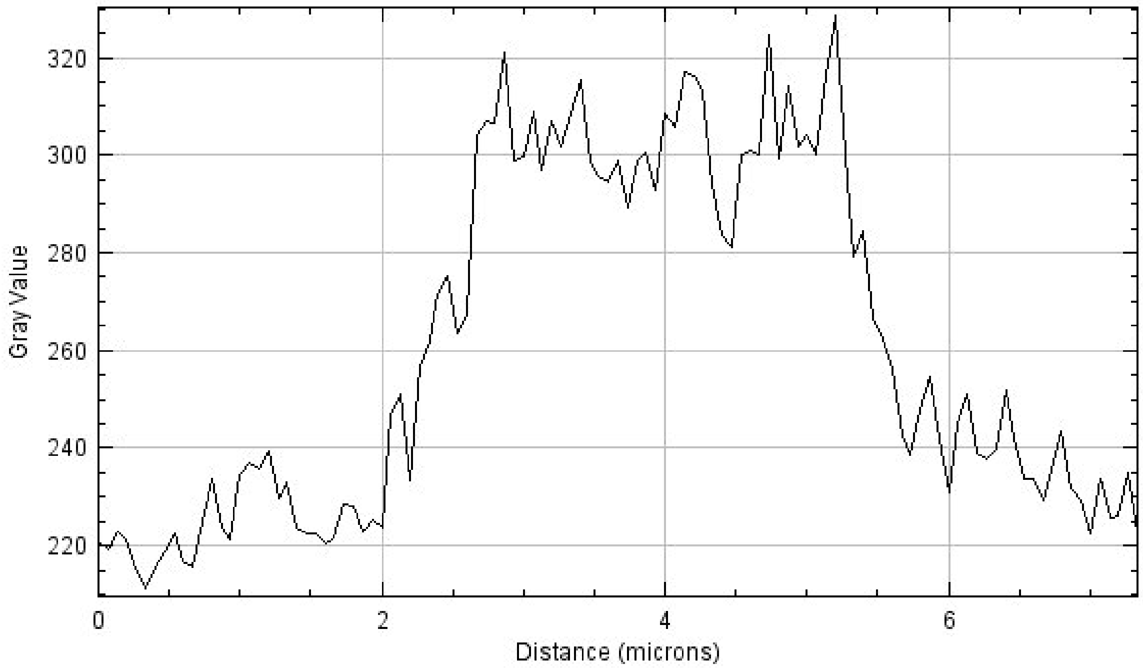

Figure S838 – Intensity profile of line scan generated for cell 6, Figure S832.

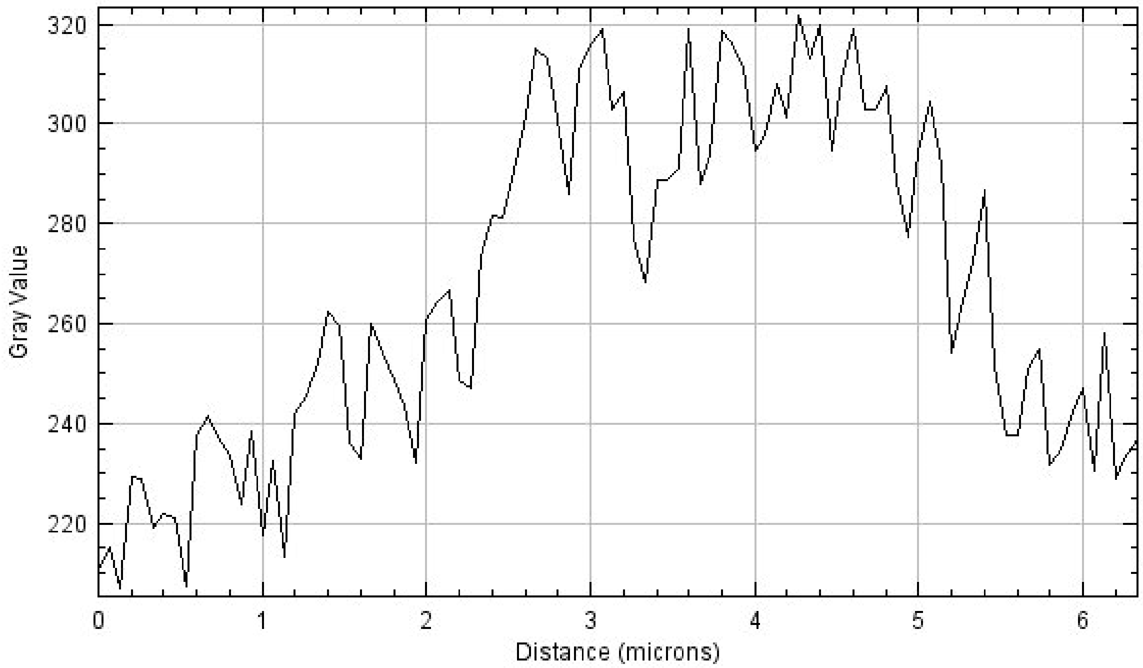

Figure S839 – Intensity profile of line scan generated for cell 7, Figure S832.

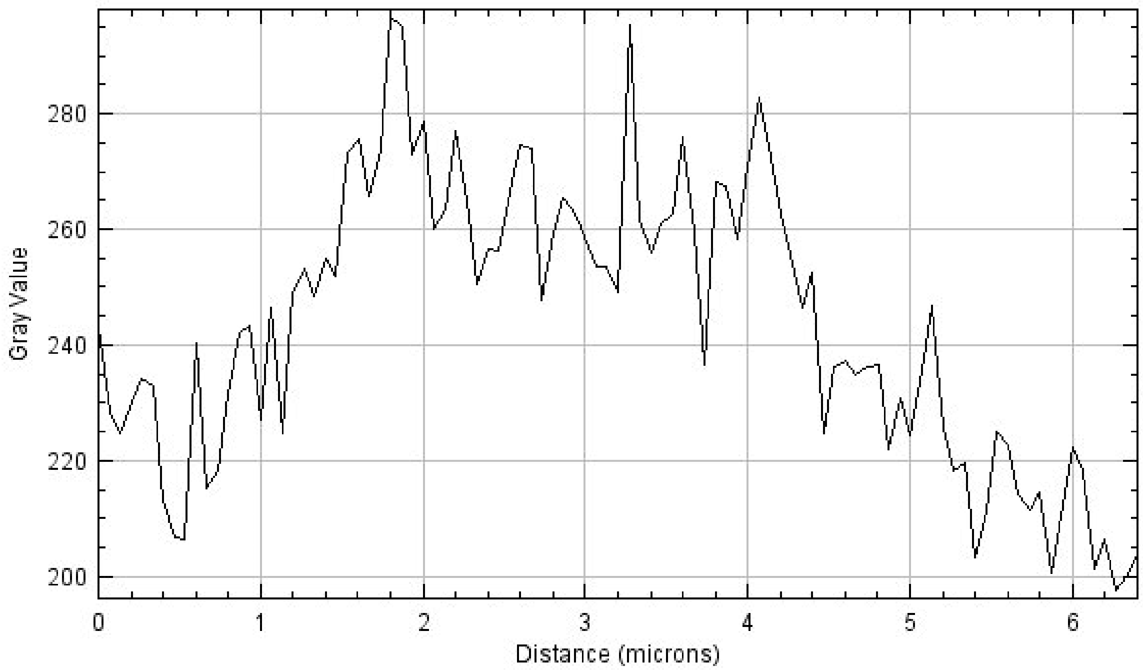

Figure S840 – Intensity profile of line scan generated for cell 8, Figure S832.

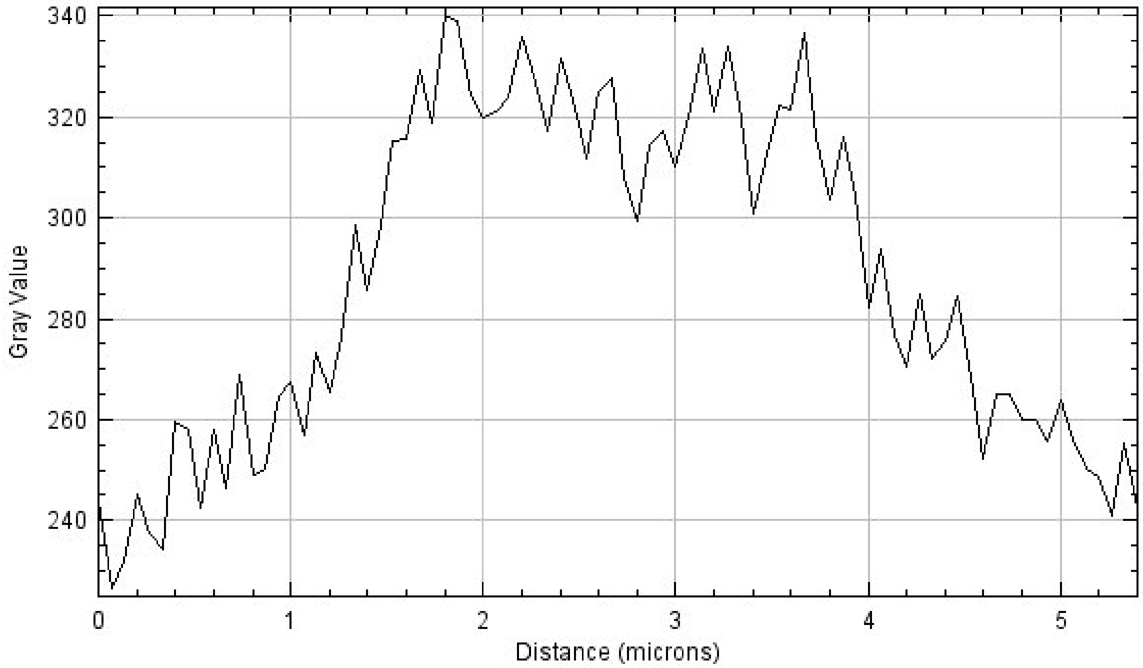

Figure S841 – Intensity profile of line scan generated for cell 9, Figure S832.

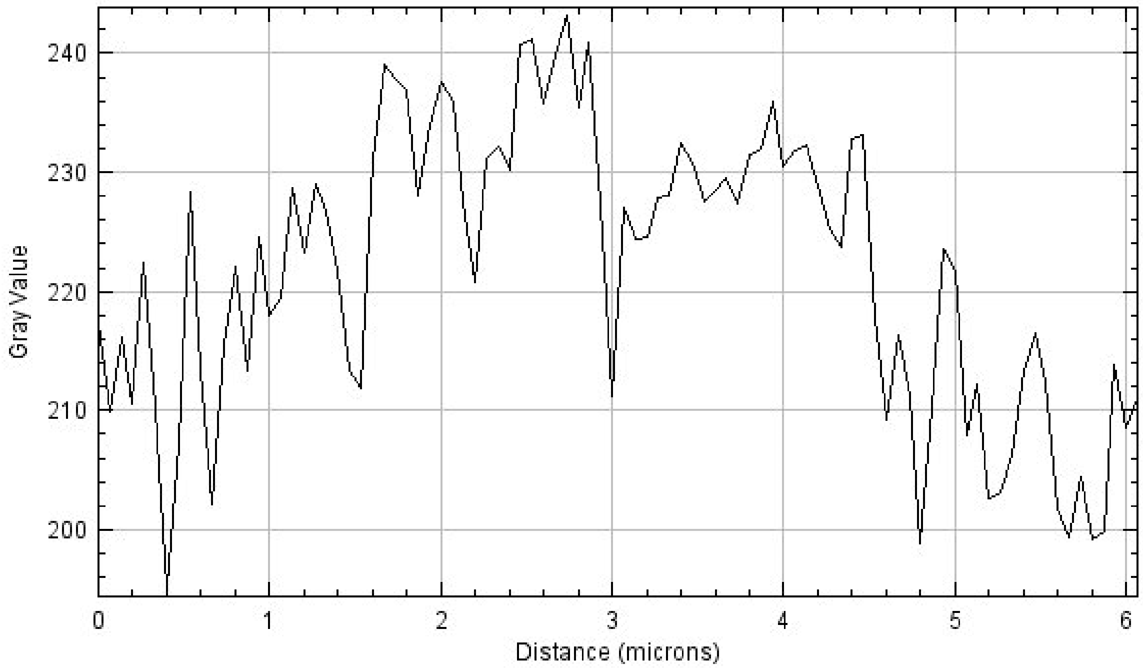

Figure S842 – Intensity profile of line scan generated for cell 10, Figure S832.

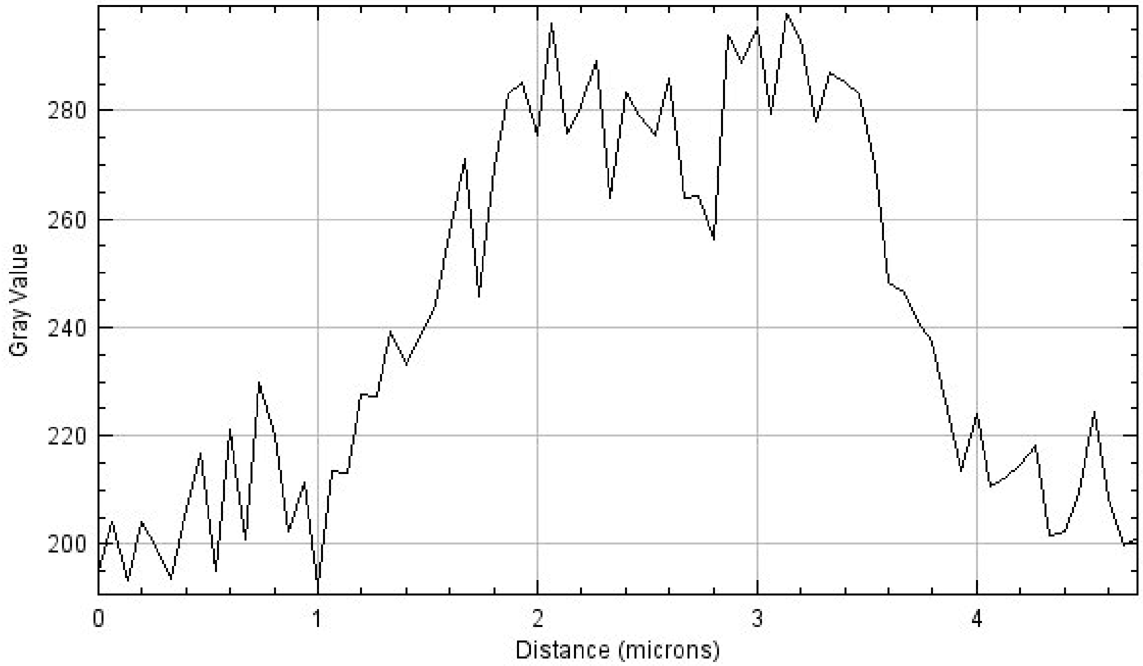

Figure S843 – Intensity profile of line scan generated for cell 11, Figure S832.

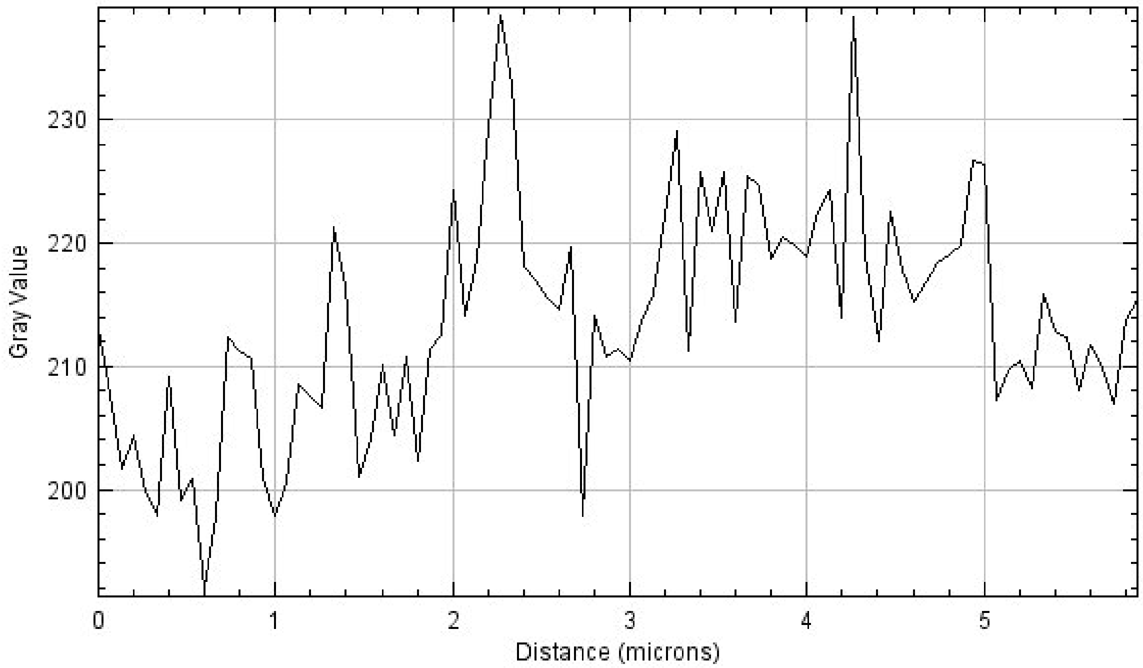

Figure S844 – Intensity profile of line scan generated for cell 12, Figure S832.

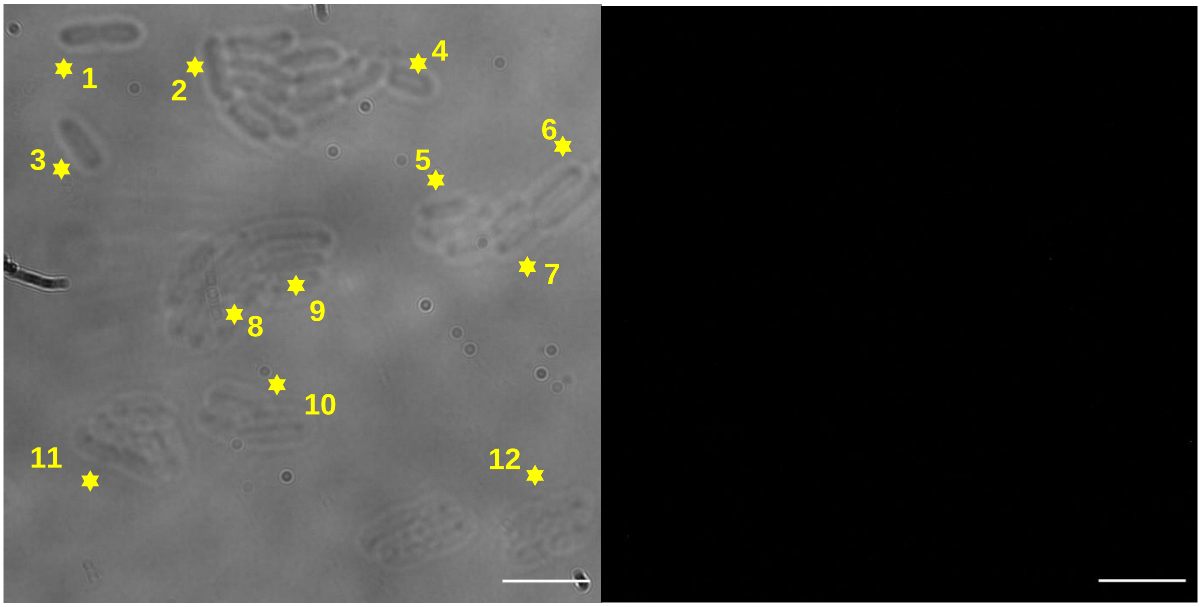

Figure S845 – *E. coli* transmitted and mCherry images used in fluorescence intensity calculations in the presence of both compound **39** and FM4-64 at T = 30 minutes. DAPI and transmitted images used to locate cells on mCherry image. Scale bar = 10 μM.

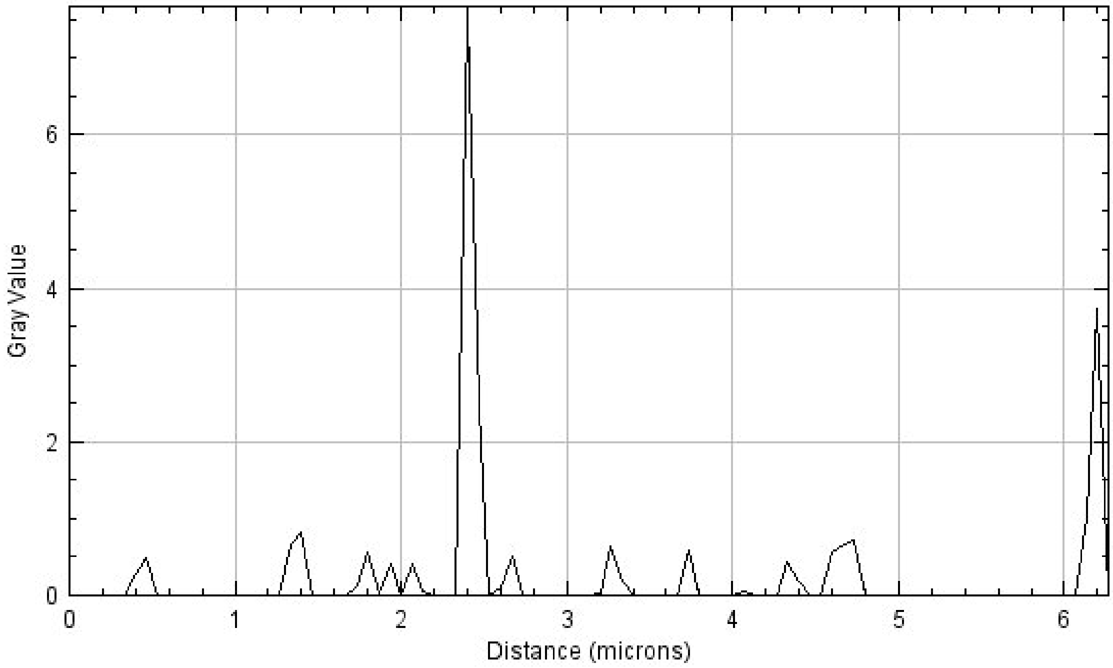

Figure S846 – Intensity profile of line scan generated for cell 1, Figure S845.

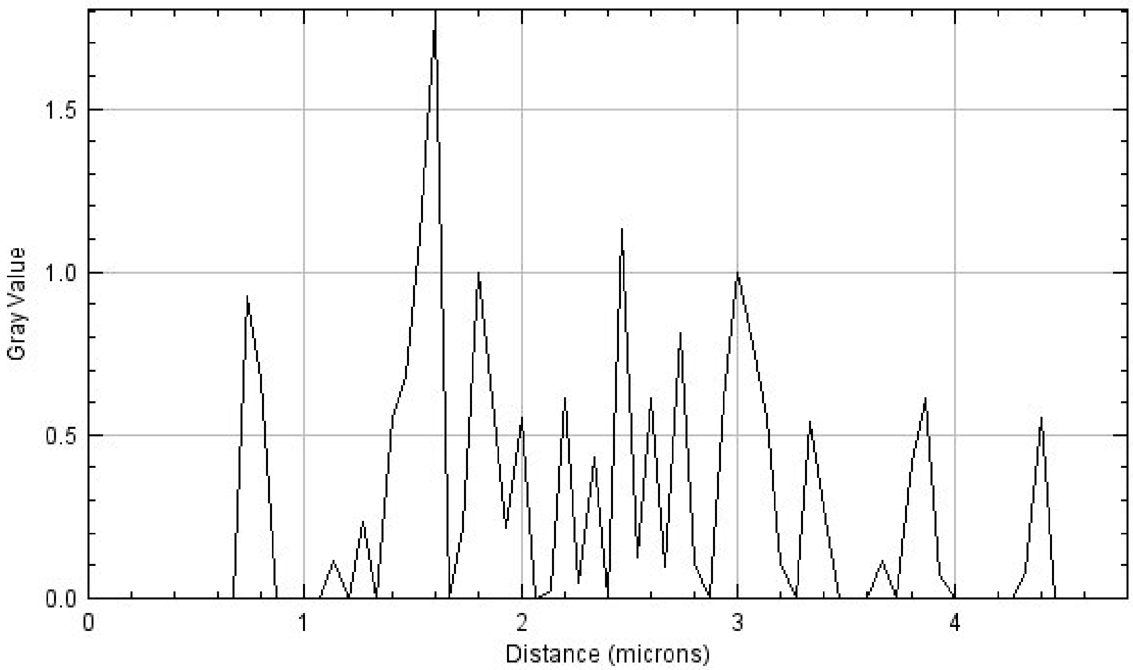

Figure S847 – Intensity profile of line scan generated for cell 2, Figure S845.

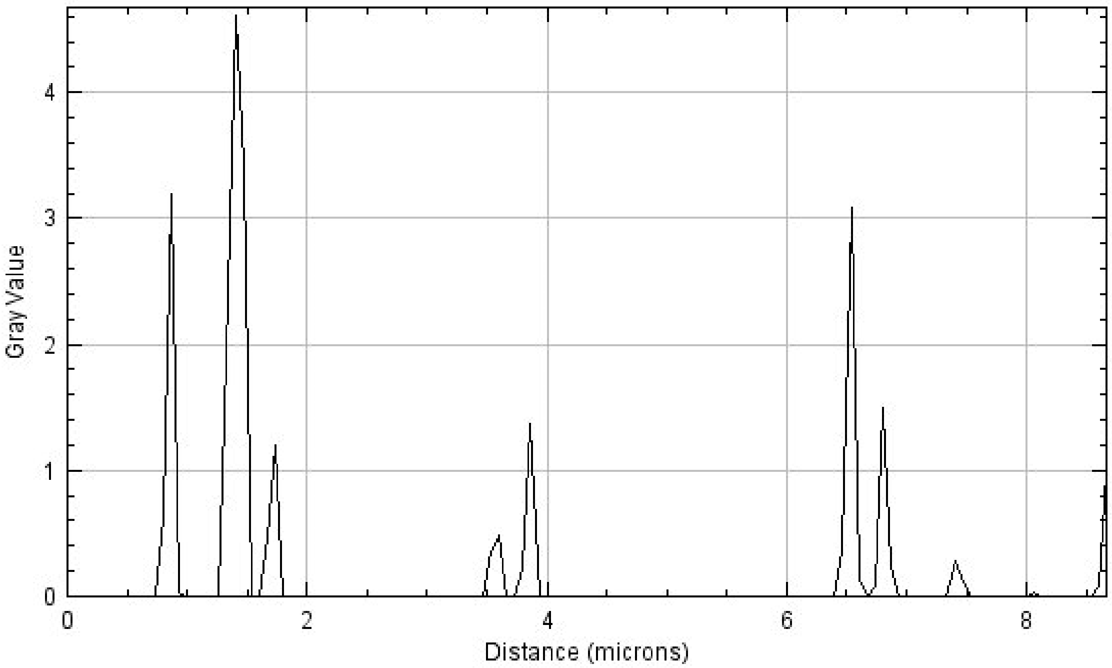

Figure S848 – Intensity profile of line scan generated for cell 3, Figure S845.

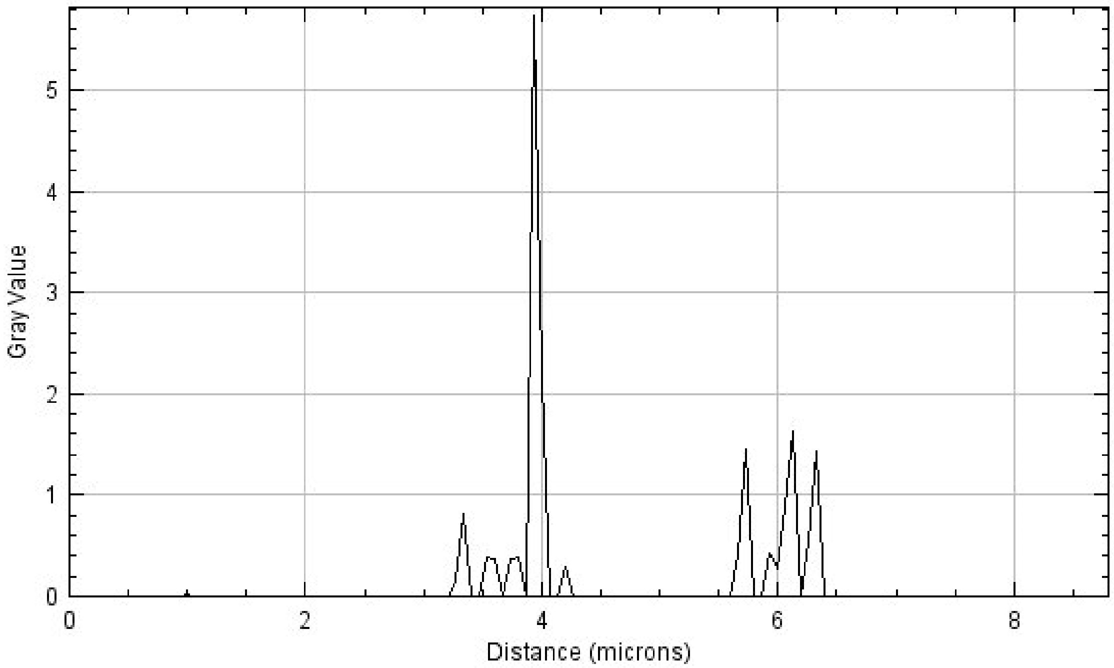

Figure S849 – Intensity profile of line scan generated for cell 4, Figure S845.

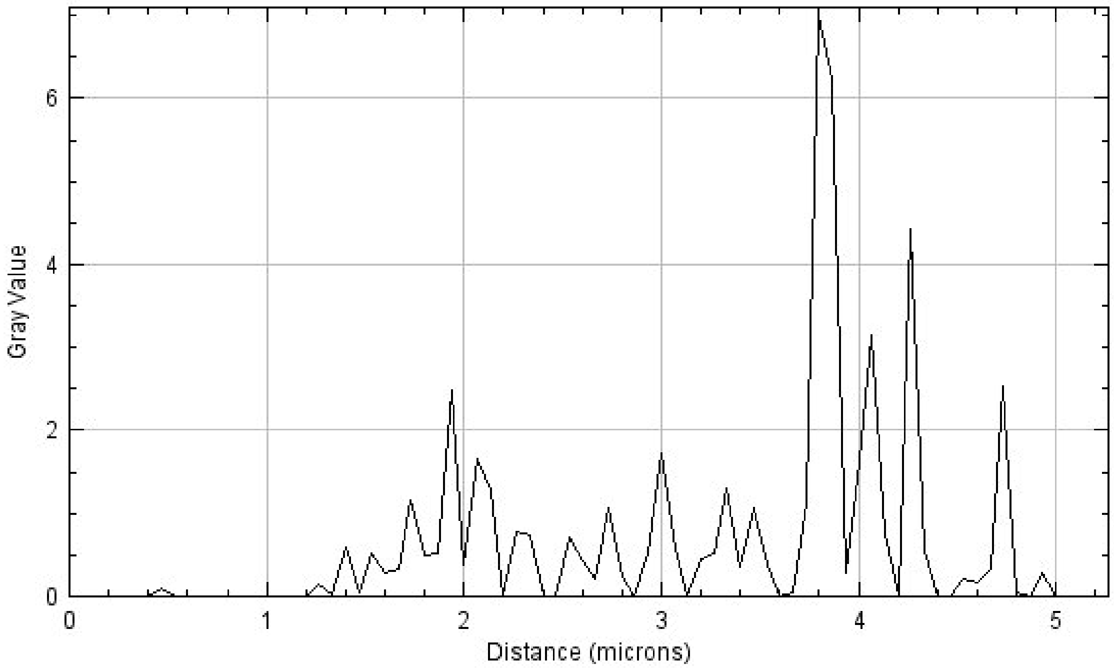

Figure S850 – Intensity profile of line scan generated for cell 5, Figure S845.

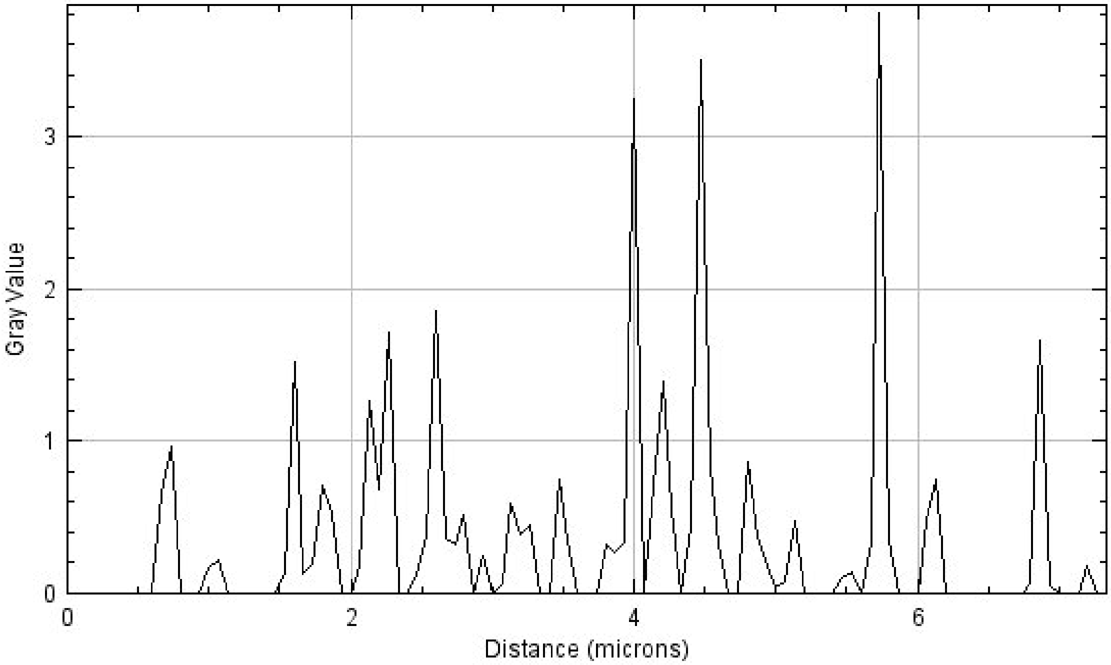

Figure S851 – Intensity profile of line scan generated for cell 6, Figure S845.

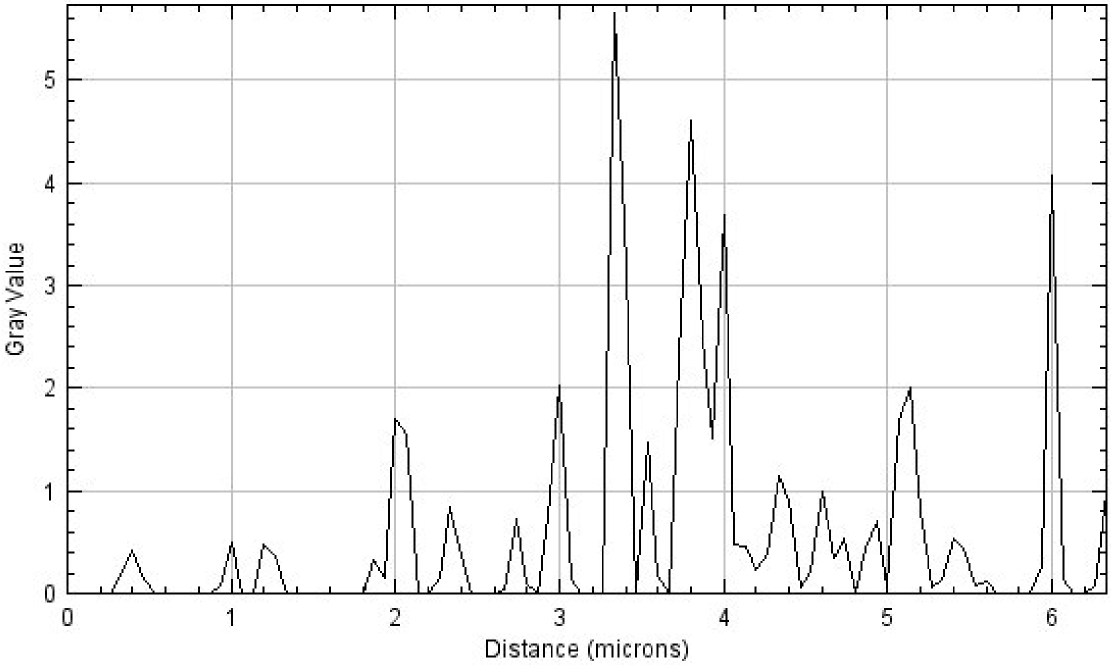

Figure S852 – Intensity profile of line scan generated for cell 7, Figure S845.

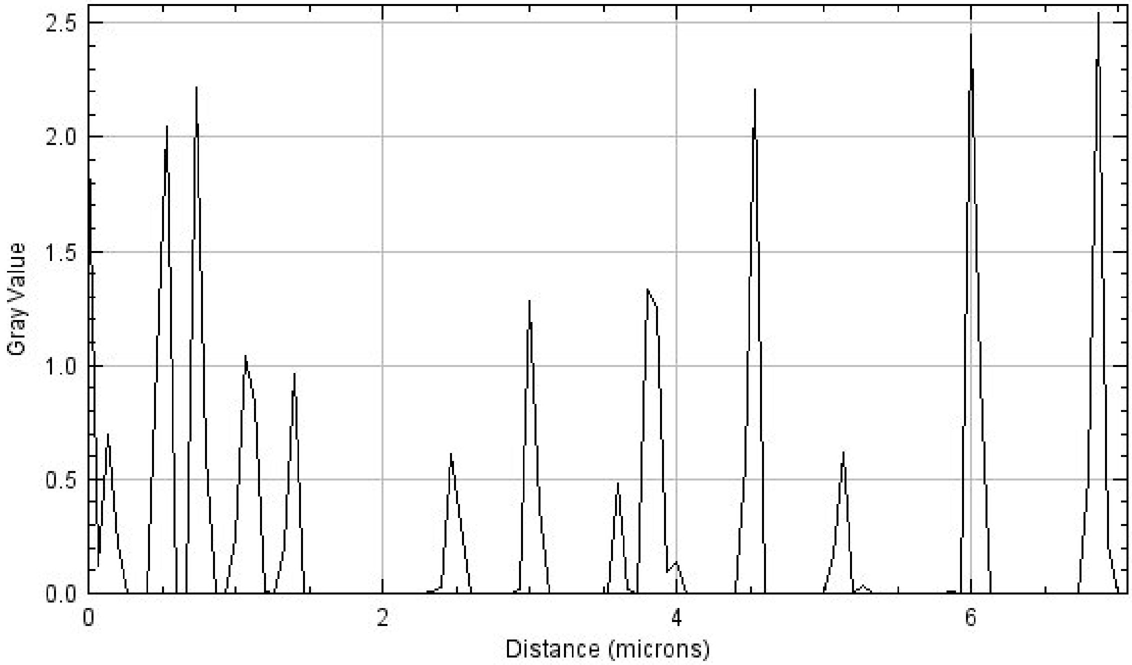

Figure S853 – Intensity profile of line scan generated for cell 8, Figure S845.

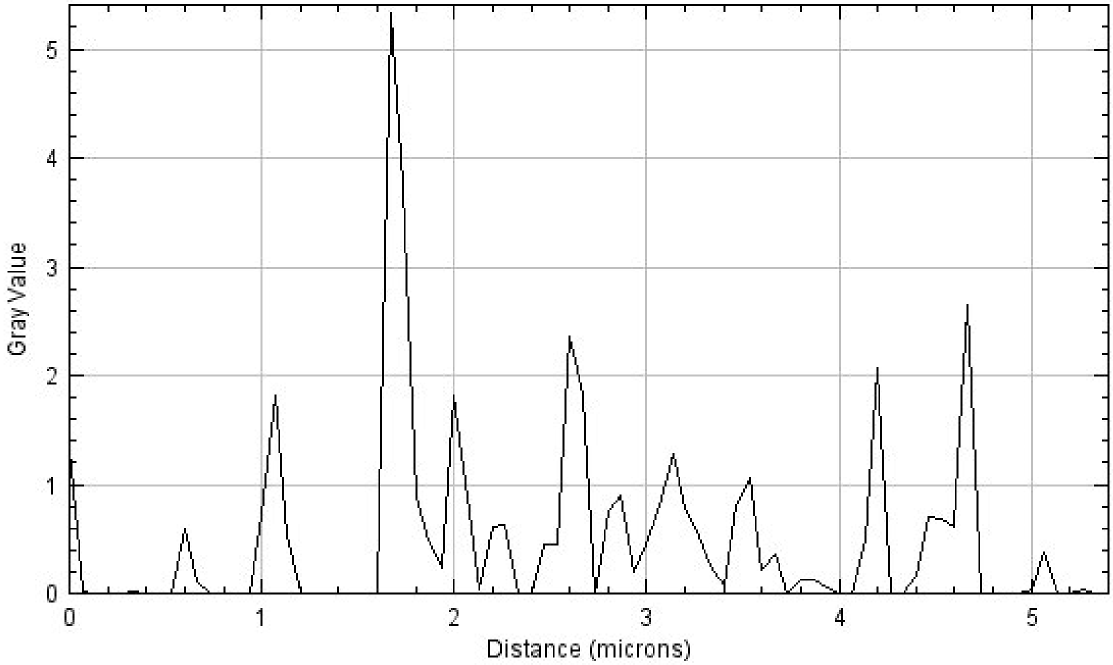

Figure S854 – Intensity profile of line scan generated for cell 9, Figure S845.

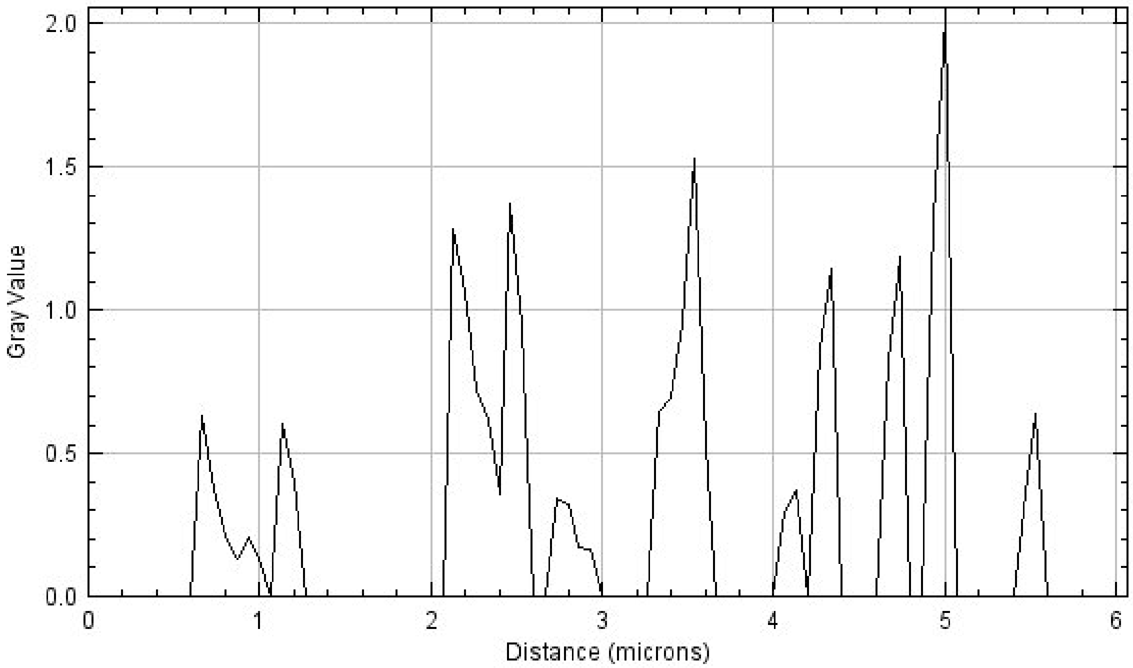

Figure S855 – Intensity profile of line scan generated for cell 10, Figure S845.

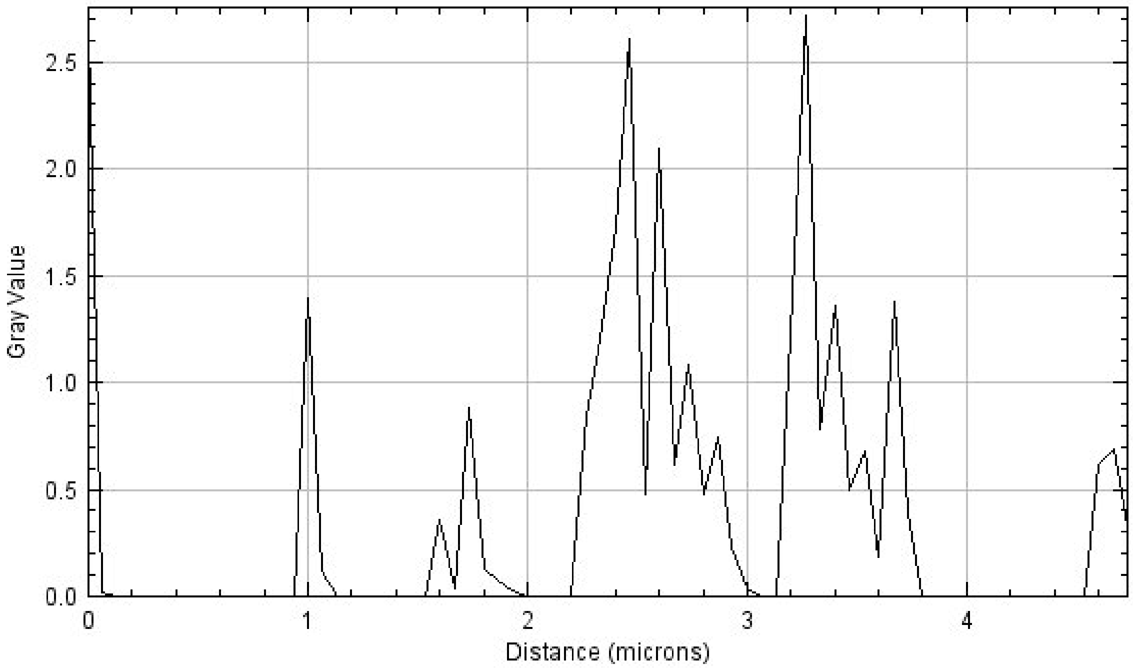

Figure S856 – Intensity profile of line scan generated for cell 11, Figure S845.

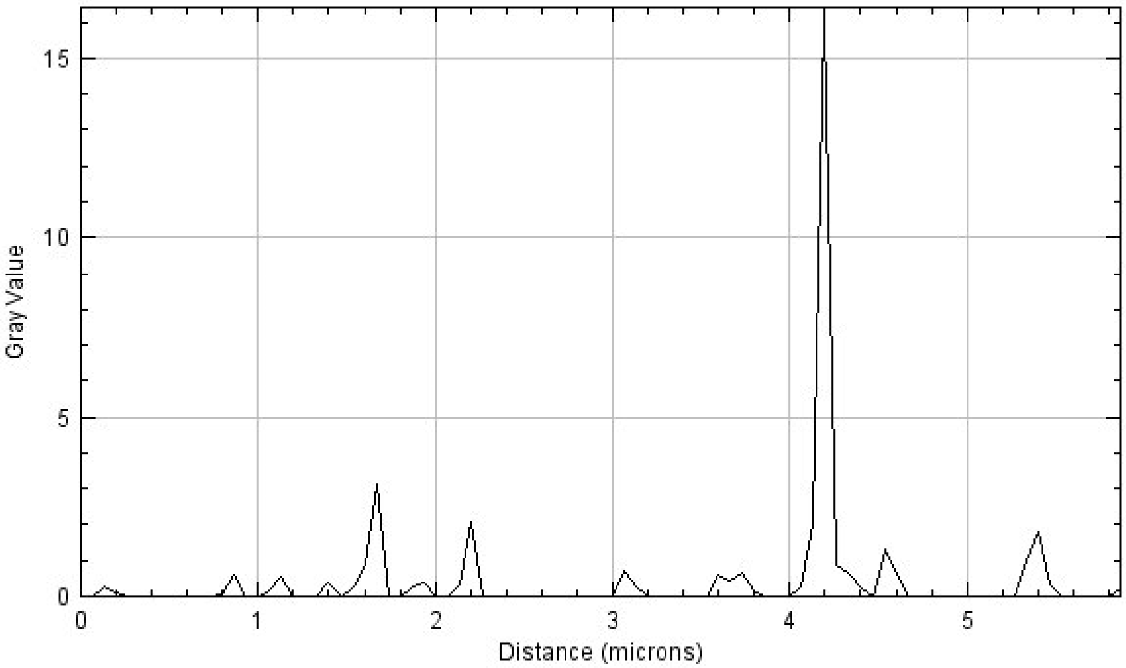

Figure S857 – Intensity profile of line scan generated for cell 12, Figure S845.

Summary Table

Table S15 – Fluorescence intensities (FI) of *E. coli* and MRSA at T = 30 min under various conditions. Cells n_{total} = total number of cells analyzed for that condition. SSA **39** monitored at 450 nm and FM 4-64 monitored at 605nm.

	**Peak Wavelength examined (*E. coli*)**		**Cells n_{total} (*E. coli*)**	**Peak Wavelength examined (MRSA)**		**Cells n_{total} (MRSA)**
	450 nm	605 nm		450 nm	605 nm	
**Solvent alone**	21.15 ± 1.59	4.28 ± 0.74	54	22.46 ± 3.20	6.95 ± 1.87	73
**SSA 39**	104.35 ± 10.86	5.41 ± 0.43	69	133.98 ± 16.90	4.28 ± 1.91	69
**FM 4-64**	22.23 ± 0.91	32.66 ± 11.68	79	-	53.24 ± 8.35	32
**SSA 39 and FM 4-64**	88.30 ± 17.49	6.60 ±1.60	67	187.11 ± 19.30	7.20 ± 3.02	86

References

1. Clinical and Laboratory Standards Institute. 2012. Methods for dilution antimicrobial susceptibility tests for bacteria that grow aerobically; approved standard—9th edition. CLSI document M07-A9. Clinical and Laboratory Standards Institute, Wayne, PA
2. J.M. Andrews, *J. Antimicrob. Chemother.,* 2001, **48**, 5-16.
3. J. R. Hiscock, G. P. Bustone, B. Wilson, K. E. Belsey and L. R. Blackholly, *Soft Matter*, 2016, **12**, 4221-4228.
4. L. J. White, S. N. Tyuleva, B. Wilson, H. J. Shepherd, K. K. L. Ng, S. J. Holder, E. R. Clark and J. R. Hiscock, *Chem. Eur. J.*, 2018, **24**, 7761–7773.
5. L. R. Blackholly, H. J. Shepherd and J. R. Hiscock, *Crystengcomm.*, 2016, **18**, 7021-7028.
6. L. J. White, N. J. Wells, L. R. Blackholly, H. J. Shepherd, B. Wilson, G. P. Bustone, T. J. Runacres and J. R. Hiscock, *Chem. Sci*., 2017, **8**, 7620–7630.
7. S. N. Tyuleva, N. Allen, L. J. White, A. Pepes, H. J. Shepherd, P. J. Saines, R. J. Ellaby, D. P. Mulvihill and J. R. Hiscock, *Chem. Comm*., 2019, **55**, 95-98.
8. D. P. Mulvihill, *Cold Spring Harbor Protocols* **2017**, *2017*, pdb.top090621
9. J. Schindelin, I. Arganda-Carreras, E. Frise, V. Kaynig, M. Longair, T. Pietzsch, S. Preibisch, C. Rueden, S. Saalfeld, B. Schmid, J. Y. Tinevez, D. J. White, V. Hartenstein, K. Eliceiri, P. Tomancak, A. Cardona, Nat. Methods 2012, **9**, 676-682.
10. C. A. Hunter, *Angew. Chem. Int. Ed.*, 2004, **43**, 5310-5324.
11. J. J. P. Stewart, *J. Molec. Mod.*, 2007, **13**, 1173-1213.
